# The Association Between Thymidylate Synthase Gene Polymorphisms and the Risk of Ischemic Stroke in Chinese Han Population

**DOI:** 10.1007/s10528-023-10431-8

**Published:** 2023-06-28

**Authors:** Fuhua Yu, Lei Shi, Qianru Wang, Xiaohui Xing, Zhongchen Li, Lei Hou, Zhengshan Zhou, Zengguang Wang, Yilei Xiao

**Affiliations:** 1 Department of Neurosurgery, Key Laboratory of Post-Trauma Neuro-Repair and Regeneration in Central Nervous System, Ministry of Education & Key Laboratory of Injuries, Variations and Regeneration of Nervous System, Tianjin Medical University General Hospital, Neurological Institute, 154 Anshan Road, Tianjin, 300052 China; 2 Department of Neurosurgery, Dongchangfu District, Liaocheng People’s Hospital, No.67 Dongchang West Road, Liaocheng, 252000 Shandong People’s Republic of China; 3 Department of Neurology, Dongchangfu District, Liaocheng People’s Hospital, No.67 Dongchang West Road, Liaocheng, 252000 Shandong People’s Republic of China; 4 Department of Pharmacy, Liaocheng Fourth People’s Hospital. No, 47 Huayuan North Road, Dongchangfu District, Liaocheng, 252000 Shandong People’s Republic of China; 5 Department of Neurosurgery, People’s Hospital of Chiping District, No.1057 Wenhua Road, Chiping District, Liaocheng, 252100 Shandong People’s Republic of China

**Keywords:** Thymidylate Synthase gene, Ischemic stroke, Single nucleotide polymorphism (SNP), Diagnostic markers, Folic acid metabolism

## Abstract

Family history of hypertension, smoking, diabetes and alcohol consumption and atherosclerotic plaque were identified as common risk factors in IS. We aimed at investigating the relationship between Thymidylate Synthase (TS) gene polymorphisms and ischemic stroke (IS).This case–control research selected and genotyped three single nucleotide polymorphisms (SNPs)of TS( rs699517, rs2790, and rs151264360) with Sanger sequencing in Chinese Han population. We also adopted logistic regression analysis in genetic models for calculating odds ratios and 95% confidence intervals. Genotype-Tissue Expression(GTEx) database analyzed the tissue-specific expression and TS polymorphisms. The ischemic stroke patients showed higher low-density lipoprotein cholesterol and total homocysteine (tHcy). It was found that patients with the TT genotype of rs699517 and GG genotype of rs2790 had larger degrees of tHcy than those with CC + CT genotypes and AA + AG genotypes, respectively. The genotype distribution of the three SNPs did not deviate from Hardy–Weinberg equilibrium (HWE). Haplotype analysis showed that T-G-del was the major haplotype in IS, and C-A-ins was the major haplotype in controls. GTEx database indicated that the rs699517 and rs2790 increased the expression of TS in healthy human and associated with TS expression level in a single tissue. In conclusion**:** This study has shown that TS rs699517 and rs2790 were significantly related to ischemic stroke patients.

**Supplementary Information:**

The online version contains supplementary material available at 10.1007/s10528-023-10431-8.

## Introduction

Cerebral stroke, also known as cerebrovascular accident, is a complex neurological disorder and the second leading cause of death worldwide (Katan and Luft 2018), with the features such as high level of morbidity, disability rate, mortality rate, recurrence rate, and a lot of complications, bringing serious harm to human health (Gorelick 2019). Stroke is an acute cerebrovascular disease including hemorrhagic stroke and ischemic stroke (Khaku and Tadi 2023), which result from the sudden vascular rupture in the brain or the vascular occlusion preventing blood flow to the brain, causing damage to brain tissue (Paul and Candelario-Jalil 2021). In China, stroke has become the first cause of death and the leading cause of adult disability (Xu et al. 2021). Ischemic stroke(IS) accounts for about 70% of stroke cases, and its incidence varies widely geographically with higher incidence in the northern China than in southern China (Wu et al. 2013).

There are a lot of risk factors for stroke, such as age, race, diabetes, gender, hypertension, obesity, atherosclerosis, and dyslipidemia (Bhat et al. 2008; Khoury et al. 2013; Kleindorfer et al. 2010; Tirschwell et al. 2004). In addition, genetic factors are considered to be significant for stroke (Boehme et al. 2017). It has been identified that polymorphisms of some genes are related to the growth of stroke, such as DIAPH1 (Ren et al. 2020), ABO (Ling et al. 2016), MTHFR (Chauhan and Debette 2016), Kalirin (Li et al. 2017), and Lp-PLA2 G994T (Ni et al. 2017).

Ischemic stroke is a disease caused by multiple genes and factors, such as environmental, dietary habits and genetic factors (Liu et al. 2021). Stroke prevention follows the strategy of tertiary prevention (Manosalva et al. 2018). Identification of genetic variants which related to ischemic stroke risk could clearly illustrate the pathogenesis of stroke and provide a approach to prevent and treat this complicated disease. Folic acid metabolism plays an indirect or direct role in the function, division, and differentiation of cells (Hiraoka and Kagawa 2017). Insufficient or excessive folic acid will impair hematopoiesis, impact cell cycle progression and accelerate DNA damage (Henry et al. 2017). The reductases controlling the metabolism of folic acid and homocysteine are MTHFR, MTR, CBS, TS and so on (Hiraoka and Kagawa 2017; Moulik et al. 2017). The normal action of various enzymes is directly related to the normal operation of folic acid and homocysteine metabolism (Hiraoka and Kagawa 2017).

Thymidyate Synthase (TS) is an enzyme which involved in folic acid metabolism (Liu et al. 2016). To be specific, TS, the key enzyme in de novo synthesis of deoxythymine monophosphates (dTMP), catalyzes the conversion of deoxyuridine monophosphate (dUMP) to dTMP, a nucleotide necessary for DNA synthesis and recovery as a substrate (Hiraoka and Kagawa 2017; Kawakami et al. 1999; Choi and Mason 2000; Zhou et al. 2012). Hence, abnormality in the genes that encode these components can result in various issues such as MTHFR reduction and TS protein increase, even lead to vascular disease by accumulating tHcy, folate deficiency or both (Ahn et al. 2018; Choi et al. 2016). The MTHFR (677) C > T gene polymorphism also affects circulating tHcy levels and plays an important role in predicting the risk of ischemic stroke (Huang et al. 2022).TS genetic variants have been reported in cancer therapeutics because of the participation of TS in DNA repair mechanisms (Ahn et al. 2018; Jeon et al. 2021; Silva et al. 2020; Han et al. 2018; Donner et al. 2019), then we supposed that the single-nucleotide polymorphisms (SNPs) of TS is associated with the attack of stroke closely. Jung Oh Kim’s study displayed that TS was associated with Hypertensive, Diabetic, tHcy and Folate susceptibility to stroke, and TS polymorphism are related to ischemic stroke in the Korean population Kim et al. 2021.

In Chinese Han population, the relationship between TS genetic variants and ischemic stroke susceptibility is still unclear. This study aimed to confirm the relationships between ischemic stroke and TS gene polymorphisms in the northern Han Chinese population, providing available data for preventing and managing ischemic stroke.

## Materials and Methods

### Study Subjects

All patients were from the Liaocheng People’s Hospital, including 259 ischemic stroke patients and 240 control members. All patients were informed of the purpose of sample collection and signed the written informed consent. The inclusive criteria: the patients who suffering from ischemic stroke from firstly diagnosed depending on clinical features and neuroimaging criteria. Anterior circulation stroke is the inclusion standards of patients. Besides, the exclusive criteria: brain injuries, cerebral tumors, not stroke for the first diagnosis, autoimmune diseases, osteoarthrosis and psychological illness. Control subjects without history of stroke or cerebrovascular diseases were selected from health check-up center of our hospital and received a physical examination. Several healthy controls suffered from vascular risk factors including diabetes, hypertension, drinking, smoking, and hypercholesterolemia. The color Doppler ultrasonography (Model: Philips EPIQ7C) was made to investigate the carotid artery (Abreu et al. 2015). The ethics was approved from Committee of Liaocheng People’s Hospital (ethical code: 2,020,016). All tests were conducted under the guidance of the Declaration of Helsinki (Snaedal 2014).

### Single-Nucleotide Polymorphism Selection and Genotyping

Detailed data for TS SNPs (rs699517, rs2790, and rs151264360) were acquired from the dbSNP database (http://www.ncbi.nlm.nih.gov/SNP/). The minor allele frequency of these SNPs was 5% greater than in Chinese Han population (Dong et al. 2021). The blood genomic DNA purification kit (GoldMag) was used to extract the genomic DNA of peripheral blood samples in all subjects according to the manufacturer’s protocol. Detected by NanoDrop 2000C spectrophotometer (Thermo Scientific), the purity and concentration of genomic DNA were kept at − 20 °C before further analysis. The Agena Bioscience Assay Design Suite V2.0 software was adopted to design primers for PCR amplification. Primer information was listed in Table 1. Sanger sequencing was adopted to identify SNPs genotyping.

**Table 1:** Primer information for genotyping assay of the TS gene

SNP	Primer sequences (5′-3′)	Product size (bp)
rs699517		
Forward	ATAATGGCCTTATTTTGTTTTTAGCTTCA	267
Reverse	TTTTGACCTAGTTCCTTTTTCTTTTAGAGC	
rs2790		
Forward	CCAACTATTAAAATGGAAATGGCTGTTTAG	182
Reverse	AGTGGCAACATCCTTAAAAATTAATAACTG	
rs151264360		
Forward	AAGTAGCATCCAAACCAGAATACA	212
Reverse	GAGCTGAGTAACACCATCGATCA	

### Bioinformatics Data Analysis

We selected GTEx database (Version 8) (https://gtexportal.org/home/)to analyze the tissue-specific expression and TS polymorphisms (Expression and (GTEx), 2013; Stanfill and Cao 2021).

### Statistical Analysis

Student’s t-test was adopted to analyze the diversities of demographic features in case and control individuals for constant variables. Pearson’s χ2 tests were used for classified variables. A chi-square goodness-of-fit test was used to test Hardy–Weinberg Equilibrium (HWE) for each genotype. The relationships between TS SNPs and ischemic stroke risk were assessed to calculate odds ratios (ORs) and 95% confidence intervals (CIs) applying logistic regression analysis. The SHEsis analysis platform was adopted to make the linkage disequilibrium index (D-prime and r^2^) with the deductive haplotypes of these three SNPs. SPSS v24.0 (SPSS) was adopted to calculate all statistical analyses, and a two-tailed *P* < 0.05 indicated a statistically significant difference.

## Results

### Characteristics of Individuals

The relationship of Thymidyate Synthase single-nucleotide polymorphisms with ischemic stroke risk was determined by enrolling 259 ischemic stroke patients and 240 control members. The general clinical data were displayed in Table 2. No obvious significance was found in body mass index, mean age, high-density lipoprotein cholesterol, serum total cholesterol or overall triglyceride degrees between the patients and controls. According to the risk factor profile, hypertension, smoking, diabetes, alcohol consumption, family history and atherosclerotic plaque were ordinary risk factors among the patients. The ischemic stroke patients showed higher low-density lipoprotein cholesterol and tHcy.

**Table 2:** Clinical characteristics of the study participants

Variables	Controls (*n* = 240)	Ischemic stroke (*n* = 259)	*P*-value
Age (mean ± SD, year)	63.53 ± 10.48	64.66 ± 8.68	0.189
Male/Female	114/126	127/132	0.732
BMI (mean ± SD, kg/m^2^)	24.26 ± 2.42	24.09 ± 2.57	0.365
Hypertension [n (%)]	66(27.5)	93(35.9) *	0.044
Diabetes [n (%)]	18(7.5)	34(13.1) *	0.039
Smoking [n (%)]	47(19.6)	72(36.3) *	0.031
Alcohol consumption [n (%)]	42(17.5)	65(25.1) *	0.004
Family history [n (%)]	19(7.9)	35(13.5) *	0.044
Atherosclerotic plaque [n (%)]	160(66.7)	195(75.3) *	0.034
Total cholesterol (mean ± SD, mmol/L)	4.93 ± 0.69	5.02 ± 0.86	0.2
Triglycerides (mean ± SD, mmol/L)	1.73 ± 1.03	1.83 ± 0.78	0.22
HDL-C (mean ± SD, mmol/L)	1.34 ± 0.32	1.29 ± 0.37	0.108
LDL-C (mean ± SD, mmol/L)	2.68 ± 0.37	3.02 ± 0.81*	< 0.001
tHcy (mean ± SD, µmol/L)	10.05 ± 3.86	18.92 ± 5.87*	< 0.001

### TS SNPs and Ischemic Stroke Risk Assessment

The genotype distribution of the three TS SNPs (rs699517, rs2790, and rs151264360) did not deviate from HWE P value. The correlation results of TS SNPs genotype and allele frequency in patients with ischemic stroke and control group are shown in Table 3. No great diversities were shown in the TS rs151264360 genotypes or allele allocation between ischemic stroke patients and controls (*P* all > 0.05). On the contrary, the distributions of rs699517 genotypes (TT vs CC AOR = 3.128, 95% CI = 1.724–5.677; CT vs CC AOR = 1.933,95% CI = 1.080–3.462; CT + TT vs CC AOR = 2.404, 95% CI = 1.378–4.195; allele T vs C AOR = 1.684, 95% CI = 1.298–2.185) and rs2790 genotypes (GG vs AA AOR = 3.997, 95% CI = 2.009–7.953; AG vs AA AOR = 1.831, 95% CI = 1.259–2.664; AG + GG vs AA AOR = 2.076, 95% CI = 1.448–2.977; allele G vs A: AOR = 1.424, 95% CI = 1.091–1.859) were greatly different between ischemic patients and control members.

**Table 3:** Genotype frequencies of TS gene polymorphisms in control subjects and ischemic stroke patients

Genotypes	Controls *n* = 240 (%)	IS *n* = 259 (%)	AOR (95% CI) ^a^	*P*-value
rs699517				
CC	42(17.5)	21(8.1)	1.000(reference)	
CT	120(50)	116(44.8)	1.933(1.080–3.462)	0.025
TT	78(32.5)	122(47.1)	3.128(1.724–5.677)	< 0.001
CT + TT vs CC			2.404(1.378–4.195)	0.002
TT vs CC + CT			1.850(1.285–2.662)	0.001
C	204(42.5)	158(30.5)	1.000(reference)	
T	276(57.5)	360(69.5)	1.684 (1.298–2.185)	< 0.001
HWE* P*	0.721	0.364		
rs2790				
AA	125(52.1)	89(34.4)	1.000(reference)	
AG	102(42.5)	133(51.4)	1.831(1.259–2.664)	0.001
GG	13(5.4)	37(14.2)	3.997(2.009–7.953)	< 0.001
AG + GG vs AA			2.076(1.448–2.977)	< 0.001
GG vs AA + AG			2.910(1.507–5.622)	0.001
A	342(71.3)	329(63.5)	1.000(reference)	
G	138(28.7)	189(36.5)	1.424(1.091–1.859)	0.009
HWE *P*	0.179	0.259		
rs151264360				
ins/ins	45	45	1.000(reference)	
ins/del	130	142	1.092(0.678–1.760)	0.717
del/del	65	72	1.108(0.651–1.886)	0.706
ins/del + del/del vs ins/ins			1.097(0.695–1.732)	0.690
del/del vs ins/ins + ins/del			1.037(0.699–1.537)	0.858
ins	220	232	1.000(reference)	
del	260	286	1.043(0.813–1.339)	0.740
HWE *P*	0.159	0.801		

*AOR* adjusted odds ratio; *CI* confidence interval; *HWE* hardy–weinberg equilibrium

^a^Adjusted by Hypertension, Diabetes, Smoking, Alcohol consumption, Family history, Atherosclerotic plaque, LDL-C and tHcy

The ‘reference’ means that it is the standard for analysis by genotype in the table

### Haplotype Analysis

**Fig. 1:**
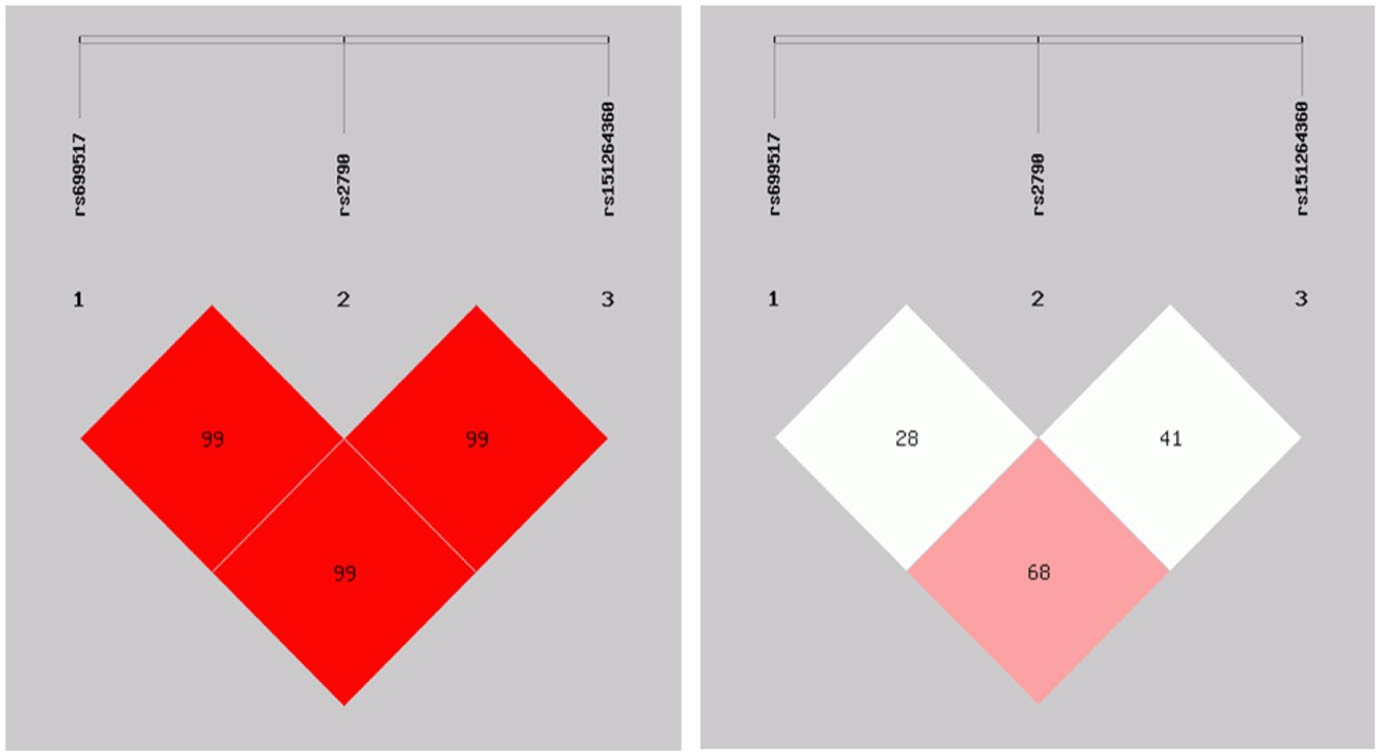
D’of the 3 SNPs: it showed that they were in linkage disequilibrium. r^2^ of the 3 SNPs: it showed that they were in linkage disequilibrium

LD measurement and haplotype analysis were made by SHEsis. These three SNPs were in linkage disequilibrium (Fig. 1). Of 8 possible haplotypes, only 4 had frequencies of > 0.03 were included in our haplotype analysis (Table 4). It was found that T-G-del was the major haplotype in IS, and C-A-ins was the major haplotype in controls (40.0% and 42.5%). Besides, it was found that the T-G-del haplotype may be related to an increasing risk of IS (OR = 1.832, 95% CI = 1.401–2.395), while the C-A-ins haplotype may be associated with a decreasing risk of IS.

**Table 4:** Haplotype analysis in the patients with IS and the controls

Haplotypes	IS (%)	Controls (%)	OR (95% CI)	*P*-value
C-A-ins	158(30.5)	204(42.5)	0.594(0.458–0.770)	< 0.001
T-A-del	79(15.3)	132(27.5)	0.474(0.347–0.647)	< 0.001
T-A-ins	74(14.3)	16(3.3)	4.868(2.788–8.500)	< 0.001
T-G-del	207(40.0)	128(26.6)	1.832(1.401–2.395)	< 0.001

All haplotypes with a frequency of < 0.03 were ignored for this analysis

### Multiple Logistic Regression Analysis

Logistic regression analysis was adopted to analyze IS risk factors in Table 5. The risk factors contained hypertension (OR = 3.982; 95% CI = 1.915–8.282), diabetes (OR = 4.250; 95% CI = 1.595–11.324), smoking (OR = 2.462; 95% CI = 1.240–4.885), alcohol consumption (OR = 3.057; 95% CI = 1.487–6.284), family history (OR = 3.855; 95% CI = 1.446–10.274), atherosclerotic plaque (OR = 1.946; 95% CI = 1.013–3.741), LDL-C (OR = 3.129; 95% CI = 1.802–5.435) and tHcy (OR = 1.441; 95% CI = 1.347–1.543). Nevertheless, after being corrected through comparison, TG and HDL-C showed no statistical significance (Table 2). Therefore, further studies need to determine our outcomes in larger sample size.

**Table 5:** Logistic regression analysis for identifying risk factors of IS

Variables	B	OR (95% CI)	*P-*value
Hypertension	1.382	3.982(1.915–8.282)	< 0.001
Diabetes	1.447	4.250(1.595–11.324)	0.004
Smoking	0.901	2.462(1.240–4.885)	0.010
Alcohol consumption	1.118	3.057(1.487–6.284)	0.002
Family history	1.349	3.855(1.446–10.274)	0.007
Atherosclerotic plaque	0.666	1.946(1.013–3.741)	0.046
LDL-C	1.141	3.129(1.802–5.435)	< 0.001
tHcy	0.366	1.441(1.347–1.543)	< 0.001

### Association Between TS rs699517, rs2790 and Serum tHcy Levels

**Fig. 2:**
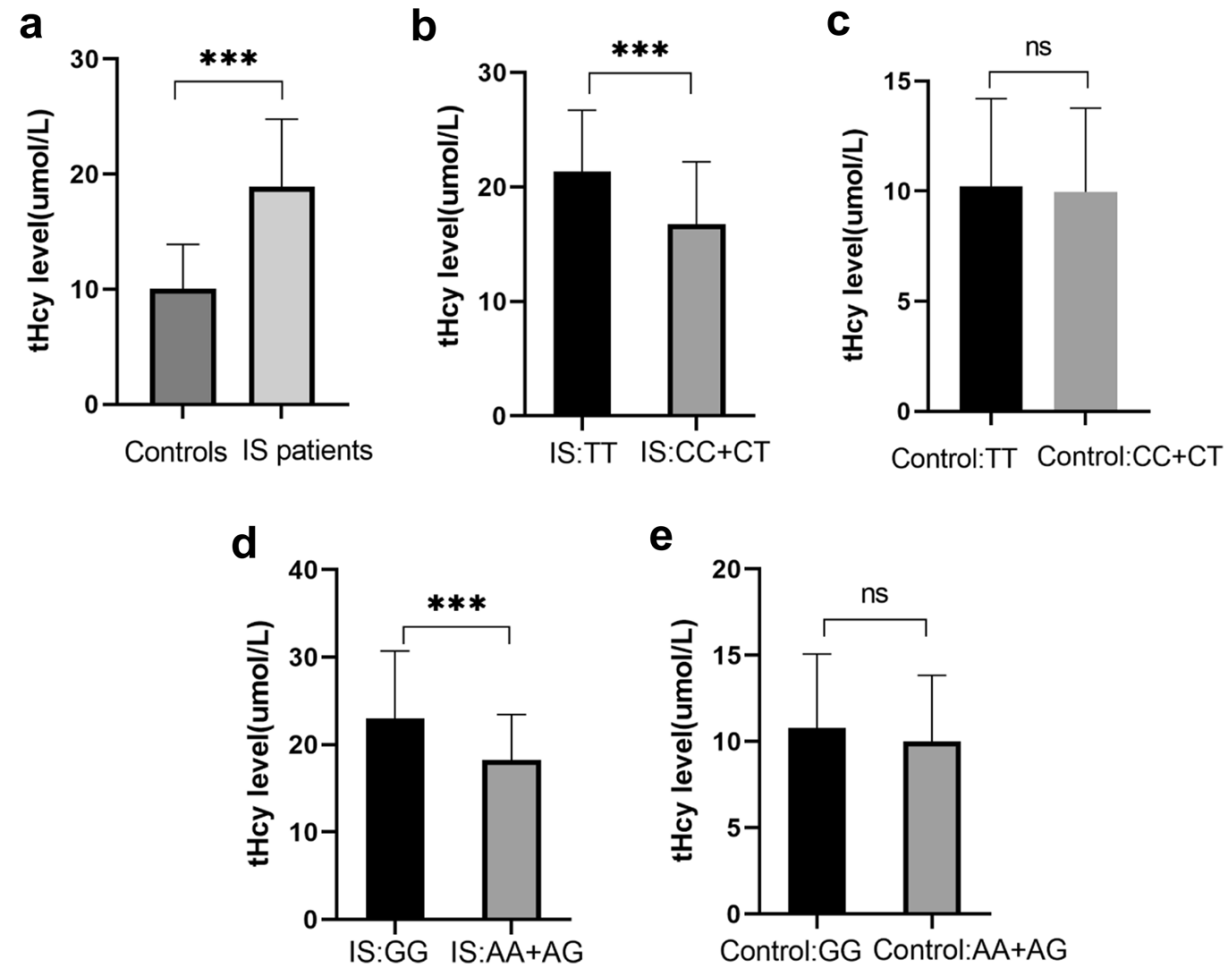
Association between rs699517, rs2790 polymorphisms and levels of tHcy

The relationship between serum degrees of tHcy and IS was explored. The tHcy degrees among IS patients were greatly increased compared with the controls (Fig. 2A). Even logistic regression was adopted for ordinary risks, including the hypertension, diabetes, smoking, alcohol consumption, family history and atherosclerotic plaque, tHcy levels were still associated with a growing risk of IS (Table 5). It was found that patients with the TT genotype of rs699517 and GG genotype of rs2790 had larger degrees of tHcy than those with CC + CT genotypes and AA + AG genotypes, respectively (Fig. 2B, D). However, individuals with the TT genotype of rs699517 and GG genotype of rs2790 in the control group showed no great diversity in tHcy levels compared with the CC + CT and AG + GG of control group, respectively (Fig. 2C, E).

An increased level of tHcy in IS patients compared with controls (A). ***, *P* < 0.001. In IS patients, rs699517 TT genotype and rs2790 GG genotype had higher levels of tHcy than those with CC + CT genotypes and AA + AG genotypes, respectively (B, D). ***, *P* < 0.001. The levels of tHcy showed no significant differences among different genotype in controls (C, E).

### Bioinformatics Data Analysis

**Fig. 3:**
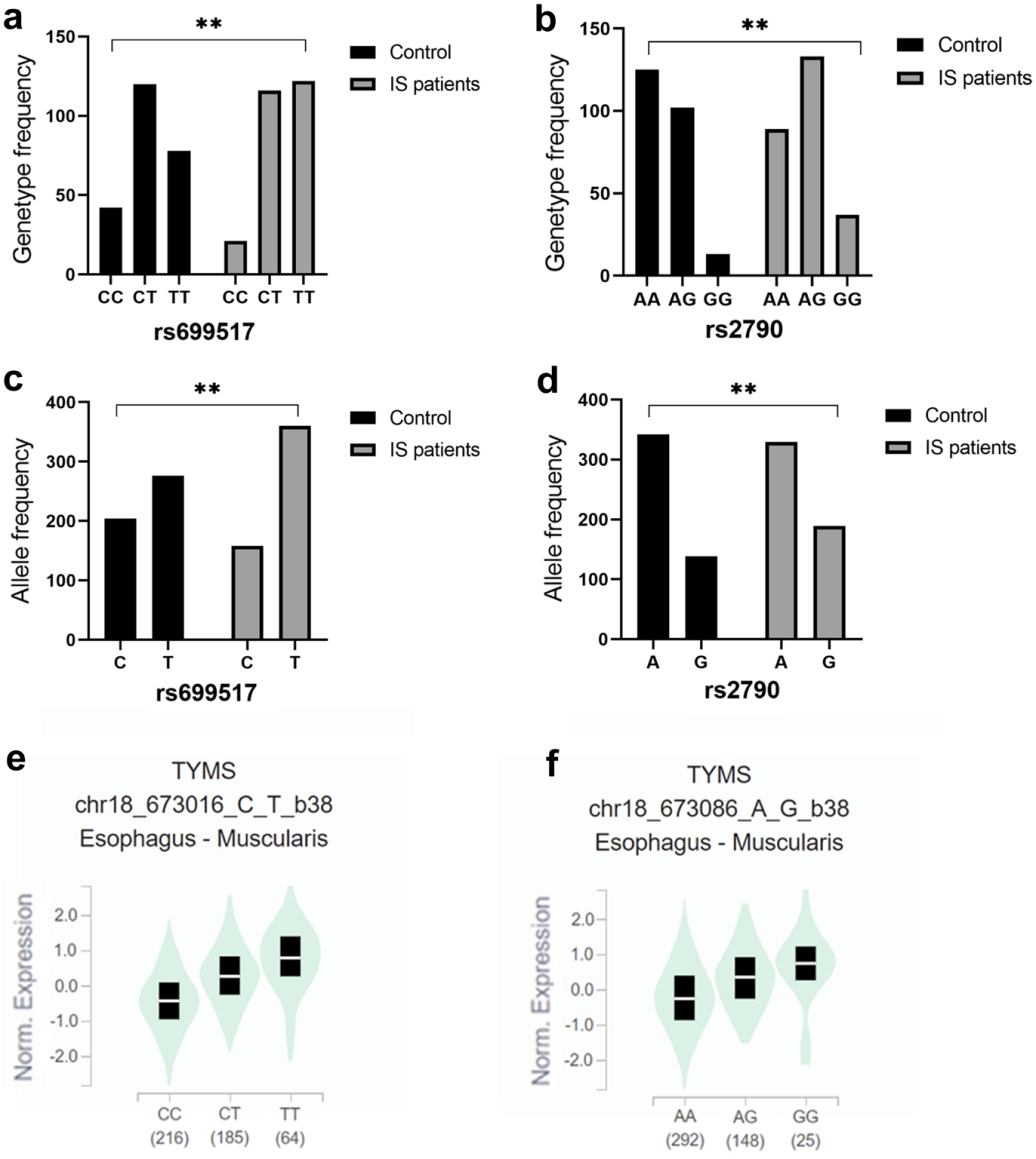
Genotype and allele frequencies distribution of rs699517 and rs2790 in control and IS patients (**A**–**D**). **, *P* < 0.01. There were significant differences in genotype and allele frequencies of rs699517 and rs2790 between two groups. The rs699517 TT genotype and rs2790 GG genotype were associated with increased levels of TS compared with CC (E) and AA (F) respectively. **, *P* < 0.01

**Fig. 4:**
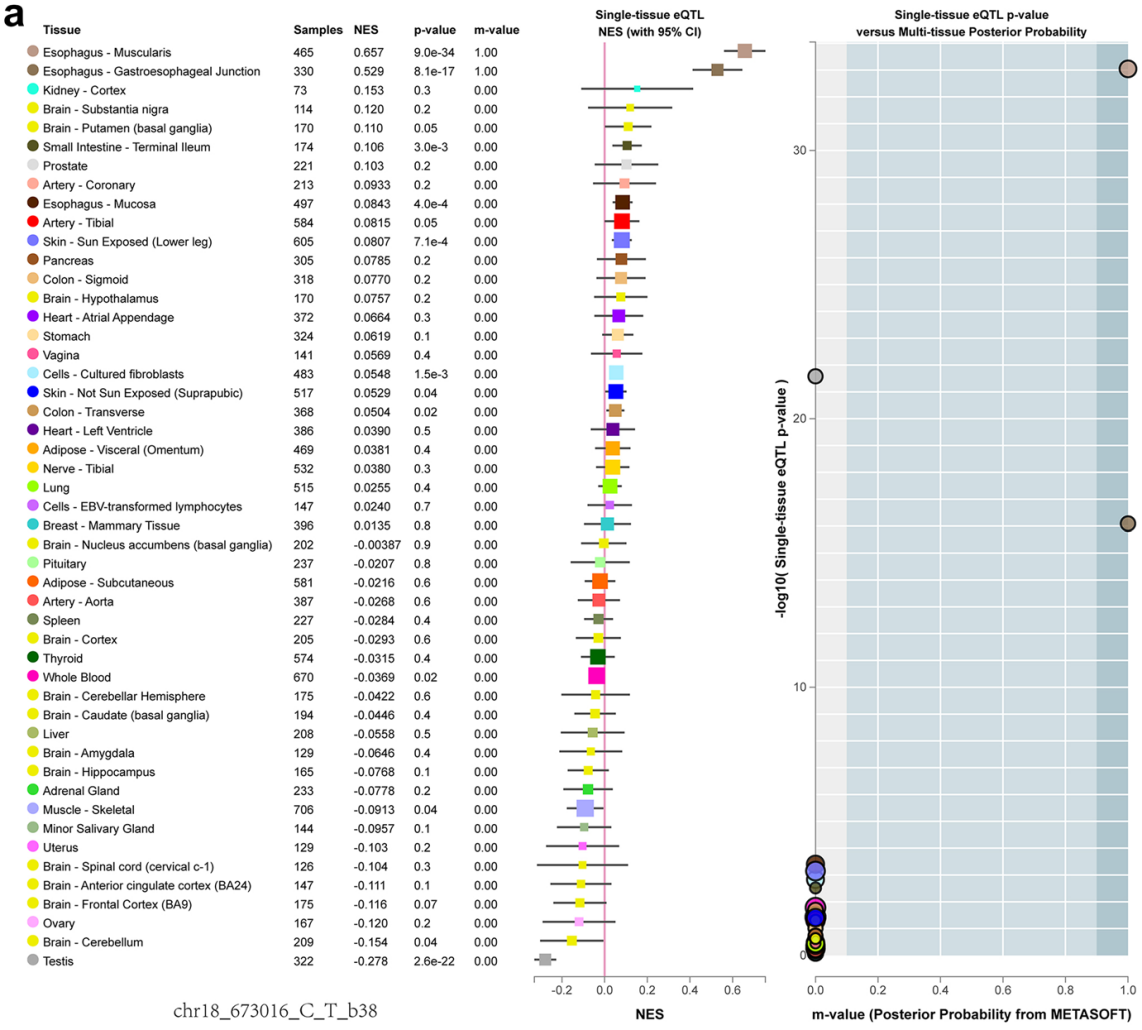

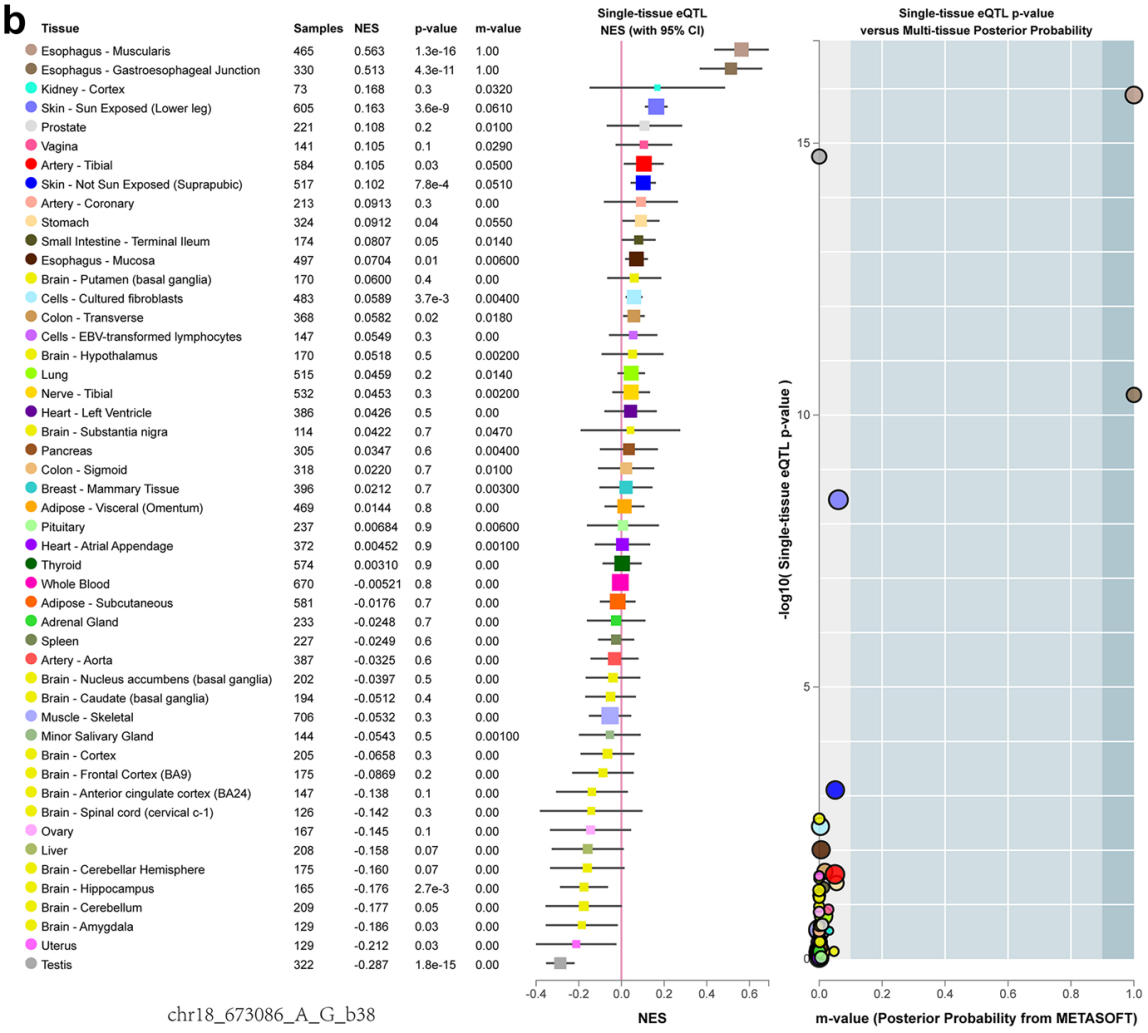
Expression quantitative trait loci (eQTL) analysis of rs699517 and rs2790 with TS expression in single tissue. The rs699517 and rs2790 polymorphisms had differences in TS expression

GTEx researched autopsy samples from healthy human donors. Genotype and allele frequencies distribution of rs699517 and rs2790 in control and IS patients (Fig. 3A-D). The TT genotype of rs699517 and GG genotype of rs2790 increased the expression of TS in healthy human (Fig. 3E, F). The expressed quantitative trait locus (eQTL) indicated that the TS rs699517 and rs2790 were associated with TS expression level in a single tissue (Fig. 4).

## Discussion

This research examined potential relationships between three SNPs of the TS and ischemic stroke in Chinese Han population. It was observed that rs699517 and rs2790 were related to IS. The T allele of rs699517 and G allele of rs2790 were risk factors for IS. However, the rs151264360 genotypes or allele distributions have no great diversities between ischemic stroke patients and controls. This is the first research to show that these SNPs were related to the attack of ischemic stroke in Chinese population.

Relationships between the occurrence of IS and folate-associated genes have been recognized in numerous research (Fekih-Mrissa et al. 2013; Holmes et al. 2011). Most previous research indicated that MTHFR (677) C > T was related to an increasing risk of stroke (Qin et al. 2020; Chang et al. 2019; Kim et al. 2013). TS was located on chromosome 18p11.32 and was mutated in various types of diseases (Kim et al. 2021; Gusella and Padrini 2007). TS is the most important protein taking part in tHcy and folate metabolism, and its polymorphism may exert a significant effect on the disease susceptibility of everybody (Ho et al. 2013). One of the most commonly researched polymorphisms is in the the 3′-untranslated region (3′-UTR) of TS mRNA, associated with a lessening of mRNA stability and translation, which lead to the low expression of protein (Ulrich et al. 2000). The TS 3′-UTR polymorphic allele (del) doubled the risk of cytological abnormalities, possibly due to TS reduction catalyzed by TS enzyme. The activity of this enzyme is reduced by TS 3′-UTR polymorphism, which is critical for cancer development (Ulrich et al. 2000; Mandola et al. 2004). Hyper homocysteine is one of the risk factors for IS, and insufficient MTHFR activity can lead to elevated plasma tHcy (Kim et al. 2017).

Alteration in folate metabolism can occur because of modified activity/availability of folate pathway enzymes, which relies on the polymorphisms in their coding genes in turn (Hiraoka and Kagawa 2017). These polymorphisms lead to reducing folate availability in the place of reaction, resulting in hyperhomocysteinaemia through epigenetic impacts including DNA methylation, uracil misincorporation and modified purine synthesis (Moulik et al. 2017). The toxicity of antifolic acid drugs used in cancer therapy is also affected by the modification of genes encoding folic acid pathway protein, that is, the reduction of protein vector, folic acid vector, and the alteration of enzyme activities such as TS and MTHFR (Petrone et al. 2021; Song et al. 2021). Nevertheless, the mechanisms regulating TS expression are not clear, and polymorphisms within the TS are seemingly significant determinants of the level of TS expression (Donner et al. 2019). TS binds to RNA and inhibits the mRNA translation, thereby regulating cell cycle progression (Choi and Mason 2000). Bioinformatics data analysis indicated that the rs699517 TT genotype and rs2790 GG genotype increased the expression of TS. Our results suggested that the rs699517 TT genotype and rs2790 GG genotype were related to increasing levels of TS comparing with CC and AA, respectively. Meanwhile, rs699517 TT genotype and rs2790 GG genotype increased the risk of IS. The GTEx resource has provided insights into the regulatory impact of genetic variation on gene expression across human tissues. The GTEx database showed that the rs699517 and rs2790 had differences in TS expression (Fig. 4). We observed that the rs699517 TT genotype frequency and rs2790 GG genotype frequency in IS were clearly higher than that in control (Fig. 3A, B). The GTEx database also showed that subjects carrying the rs699517 TT genotype frequency and rs2790 GG genotype frequency had higher levels of TS expression (Fig. 3E, F). Further mechanism experiments are needed to verify the connection between these 2 SNPs and TS expression. In a word, the important role of rs699517 and rs2790 in TS may be considered as a novel target for predicting the risk of IS.

Luca Zanoli et al. found that aortic stiffness was increased in patients with inflammation and dependent on disease duration and white blood cell count (Zanoli et al. 2017). Emerging evidence suggests that perturbations of folate/homocysteine metabolism can directly modify production of inflammatory mediators (Hammons et al. 2012). This research also examined the association between tHcy and IS risk. The tHcy serum levels of IS patients were higher significantly than the controls. The high expression of TS can upregulate tHcy levels and reduce folate degrees, causing stroke occurrence (Ho et al. 2013). There is an inverse correlation between plasma folate concentrations and tHcy levels (Brevik et al. 2005). So rs699517 and rs2790 may accelerate the inflammatory response of the body and increase the susceptibility to ischemic stroke by affecting the metabolism of folic acid. Past research illustrated vascular disease among patients who suffering from greatly increasing plasma tHcy levels (Park et al. 2013). It is believed that tHcy increases thrombotic risk by stimulating endothelial damage in blood vascular system (Joachim et al. 2013).

Nevertheless, this research is preliminary because of small sample size, shortage of measuring TS mRNA and protein expression and simultaneously only evaluated three SNPs in TS. Therefore, it is necessary to perform a replicative study with a larger sample size to confirm our findings, including genotyping, expression, and interpretation. Moreover, the functional mechanisms should be clearly illuminated for better understanding of the etiology of ischemic stroke.

## Conclusion

Our research suggested two polymorphisms in TS, rs699517 C > T and rs2790 A > G, increasing the susceptibility to ischemic stroke in the north of Chinese Han population. Once the correlation between TS and ischemic stroke is confirmed by larger cohort of patients, TS SNPs could be potential markers of ischemic stroke, which would assist to prevent ischemic stroke in Chinese Han individuals.

### Supplementary Information

Below is the link to the electronic supplementary material.

Supplementary file1 (PDF 337 KB)

Supplementary file2 (PDF 4883 KB)

Supplementary file3 (ZIP 25359 KB)

Supplementary file4 (ZIP 25697 KB)

Supplementary file5 (ZIP 24834 KB)

Supplementary file6 (ZIP 29080 KB)

Supplementary file7 (ZIP 27016 KB)

Supplementary file8 (ZIP 23226 KB)

Supplementary file9 (ZIP 24580 KB)

Supplementary file10 (ZIP 21741 KB)

Supplementary file11 (ZIP 25089 KB)

Supplementary file12 (ZIP 2998 KB)

Supplementary file13 (ZIP 28344 KB)

Supplementary file14 (ZIP 27750 KB)

Supplementary file15 (ZIP 22488 KB)

## Data Availability

The author will provide the raw data supporting the conclusions without reservation.
