# Supplementary material for: The Association Between Thymidylate Synthase Gene Polymorphisms and the Risk of Ischemic Stroke in Chinese Han Population
Source: Biochem Genet. 2023 Jun 28;62(1):468–84. doi: 10.1007/s10528-023-10431-8 (PMC10901929; doi:10.1007/s10528-023-10431-8)
Supplement: Supplementary file 1 — Supplementary file1 (PDF 337 KB) [file 10528_2023_10431_MOESM1_ESM.pdf]

聊城市人民医院医学伦理委员会

涉及人的生物医学研究项目伦理审批件

伦研批第（2020016）号

项目名称	脑卒中易感基因筛查研究				
项目类别	基础 √　　　临床 　　　药物				
项目来源					
申办单位	聊城市人民医院				
研究部门	聊城市人民医院	项目负责人	肖以磊	职称	主任医师

伦理审查意见	
△同意	√
△修改后同意	
△不同意（项目终止或暂停）	

审批意见

接受肖以磊主任医师的申请，本伦理委员会对其申报项目“脑卒中易感基因筛查研究”进行了伦理审查，审查认为：本研究以脑卒中易感人群及脑卒中患者血清为研究对象，旨在采用新型的CRISPR技术快速检测患者血清中脑卒中易感基因MTHFR多态性位点的突变情况。该研究项目基本符合国际性医学伦理文件以及中国的有关法律法规和伦理学要求。

该研究人员保证：遵守世界医学协会（WMA）所阐述原则，尊重伦理委员会对本项目所提出的伦理建议，做好保密工作，所有原始数据、文件材料作为机要档案保管，至少在研究结束后保管三年以上。研究过程中保存精确记录，以备检查总结。

经聊城市人民医院医学伦理委员会评议，从伦理方面同意该项目进行研究。该项目研究过程中的伦理问题，仍需接受本委员会的监督与审查。

聊城市人民医院医学伦理委员会（盖章）

聊城市人民医院医学伦理委员会

2020年10月11日
