# Supplementary material for: The Association Between Thymidylate Synthase Gene Polymorphisms and the Risk of Ischemic Stroke in Chinese Han Population
Source: Biochem Genet. 2023 Jun 28;62(1):468–84. doi: 10.1007/s10528-023-10431-8 (PMC10901929; doi:10.1007/s10528-023-10431-8)
Supplement: Supplementary file 2 — Supplementary file2 (PDF 4883 KB) [file 10528_2023_10431_MOESM2_ESM.pdf]

项目批准号	81701159
申请代码	H0906
归口管理部门	
依托单位代码	25010008A0746-1394

817011591006639

国家自然科学基金委员会

资助项目计划书

资助类别：青年科学基金项目

亚类说明：

附注说明：

项目名称：外泌体miRNA-182在BMSCs治疗脑出血神经损伤中的作用及机制研究

直接费用：20万元 执行年限：2018.01-2020.12

负 责 人：肖以磊

通讯地址：山东省聊城市东昌西路67号

邮政编码：252000 电 话：0635-8505626

电子邮件：15906354846@163.com

依托单位：山东大学

联 系 人：牛娟玲 电 话：0531-88369277

填表日期：2017年08月18日

国家自然科学基金委员会制

Version：1.006.639

山东省自然科学基金面上项目

资 助 项 目 立 项 任 务 书

<table>
<tr><td rowspan="4">项目基本信息</td><td>项目名称</td><td colspan="5">lncRNA H19 结合膜转运蛋白 Rab27a 调控外泌体分泌在 BMSCs 治疗脑出血神经损伤中的作用机制研究</td></tr>
<tr><td>立项编号</td><td colspan="3">ZR2021MH303</td><td>项目类别</td><td>面上项目</td></tr>
<tr><td>执行期限</td><td colspan="3">2022.1-2024.12</td><td>资助经费(万元)</td><td>10</td></tr>
<tr><td>学科分类</td><td colspan="3">脑血管结构、功能异常及相关疾病</td><td>学科代码</td><td>H0906</td></tr>
<tr><td rowspan="5">项目承担人信息</td><td>姓名</td><td>肖以磊</td><td>性别</td><td>男</td><td>学位</td><td>博士</td></tr>
<tr><td>电子邮箱</td><td colspan="3">15906354846@163.com</td><td>手机</td><td>15906354846</td></tr>
<tr><td>依托单位</td><td colspan="3">聊城市人民医院</td><td>专业技术职务</td><td>主任医师</td></tr>
<tr><td>所在单位(院系)</td><td colspan="3">神经外科</td><td>主管部门</td><td>聊城市科技局</td></tr>
<tr><td colspan="2">所在省级以上重点实验室</td><td colspan="4"></td></tr>
</table>

项目组成员（与申请书一致，不包含主持人）

姓名	性别	出生年月	学位	职称	工作单位	任务分工	每年工作时间（月）	签名
张丽娜	女	1979-12-09	博士	副主任医师	聊城市人民医院	课题设计、数据整理	6	
李中辰	男	1988-01-25	硕士	在读博士	聊城市人民医院	分子生物学实验	8	
纪振刚	男	1983-11-26	硕士	主治医师	聊城市人民医院	分子生物学实验	6	
刘超	男	1984-05-05	博士	在读博士后	聊城市人民医院	动物模型制备、动物实验	8	
韩洪峰	男	1995-02-10	硕士	住院医师	聊城市人民医院	动物模型制备、动物实验	8	
王继跃	男	1963-01-19	学士	主任医师	聊城市人民医院	课题设计实施指导	4	

山 东 省 重 点 研 发 计 划

项 目 任 务 书

项目编号：2018GSF118046

项目名称：外泌体miRNAs在脑出血神经损伤中的作用机制研究

项目主管部门（甲方）：聊城市科技局

项目承担单位（乙方）：聊城市人民医院

项目协作单位：聊城职业技术学院

项目负责人：肖以磊

联系电话：06358505626

起止时间：2018 年01 月至 2019 年12 月

山东省科学技术厅

二○一七年制

中国博士后科学基金资助证书

Certificate of China Postdoctoral Science Foundation Grant

上海交通大学 博士后研究人员 肖以磊

（全国博管办编号为 189081 ），获得第 63 批中国博士后科学基金面上资助 二 等资助，资助编号为 2018M632123 。

特颁此证。

中国博士后科学基金会

2018 年 05 月 04 日

证书查验请登录中国博士后科学基金会网站

泰山学者

Taishan Scholars

青年专家　　肖以磊

山东省人民政府

二〇二一年二月

NO.tsqn202103200
