# Supplementary figures and images for: The Association Between Thymidylate Synthase Gene Polymorphisms and the Risk of Ischemic Stroke in Chinese Han Population (part 1 of 6)
Source: Biochem Genet. 2023 Jun 28;62(1):468–84. doi: 10.1007/s10528-023-10431-8 (PMC10901929; doi:10.1007/s10528-023-10431-8)

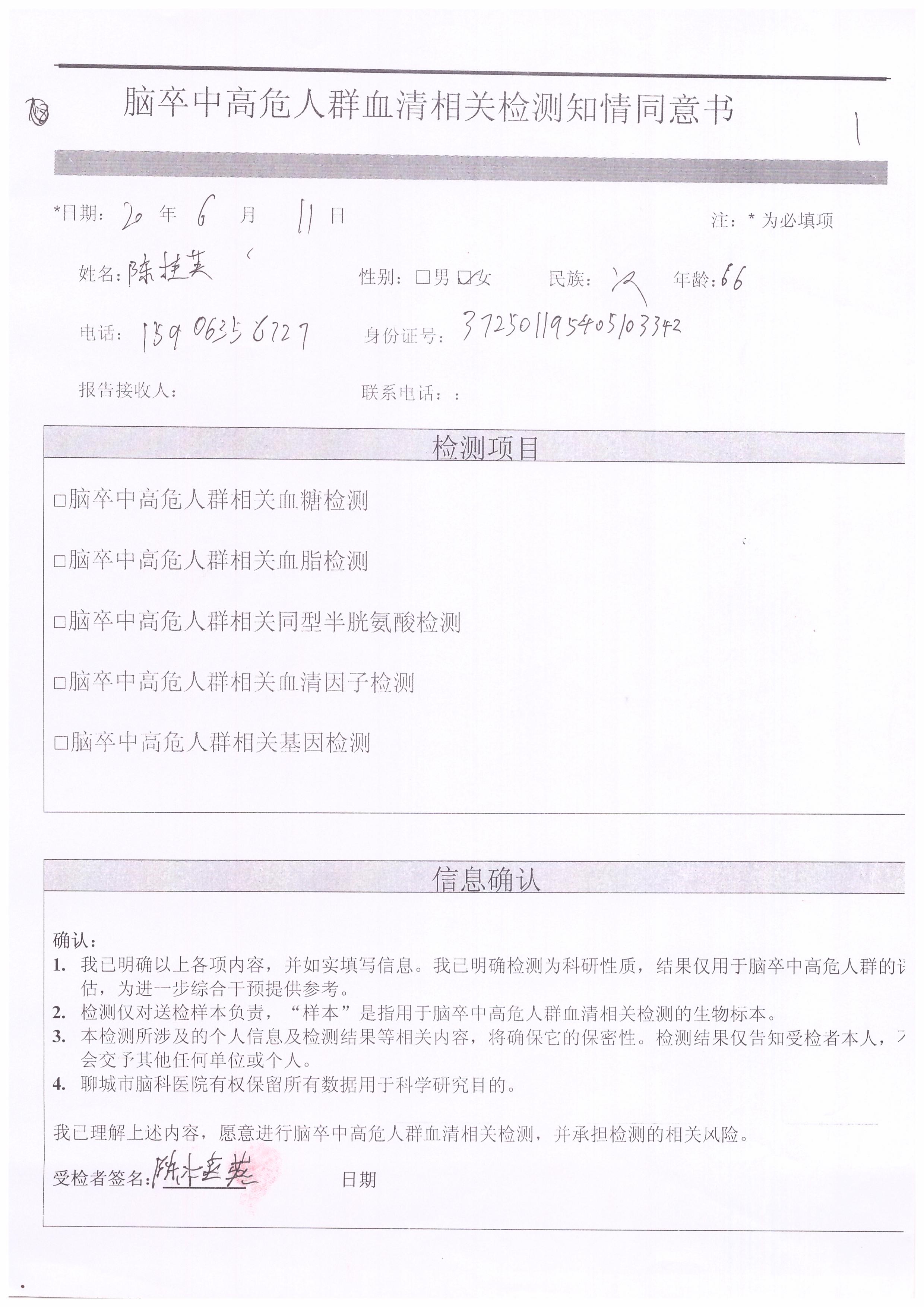

Supplement: Supplementary file 3 — Supplementary file3 (ZIP 25359 KB) [file 10528_2023_10431_MOESM3_ESM.zip › ╓¬╟Θ═1⁄4╥Γ╩Θ1/001 (2).jpg]

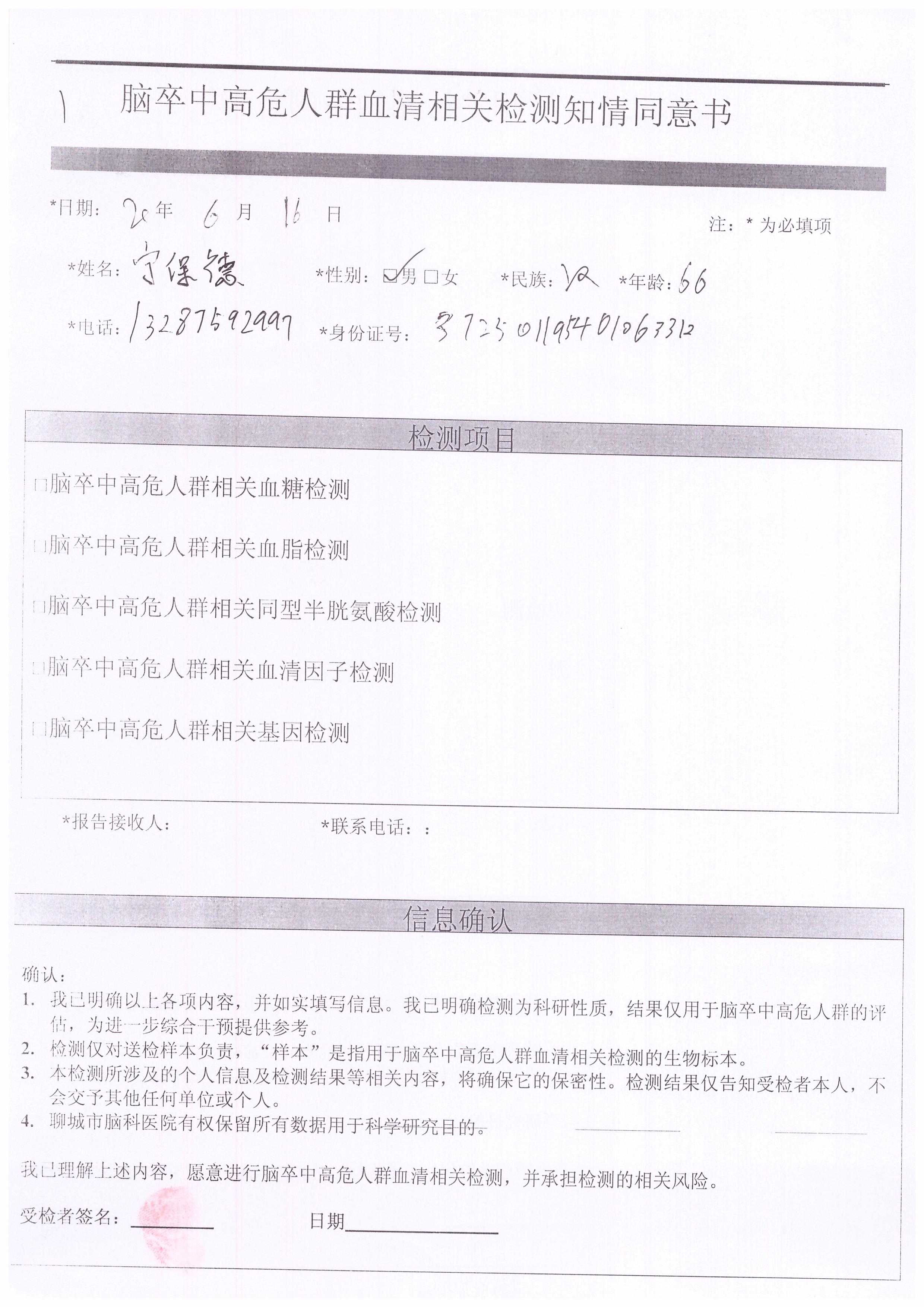

Supplement: Supplementary file 3 — Supplementary file3 (ZIP 25359 KB) [file 10528_2023_10431_MOESM3_ESM.zip › ╓¬╟Θ═1⁄4╥Γ╩Θ1/001.jpg]

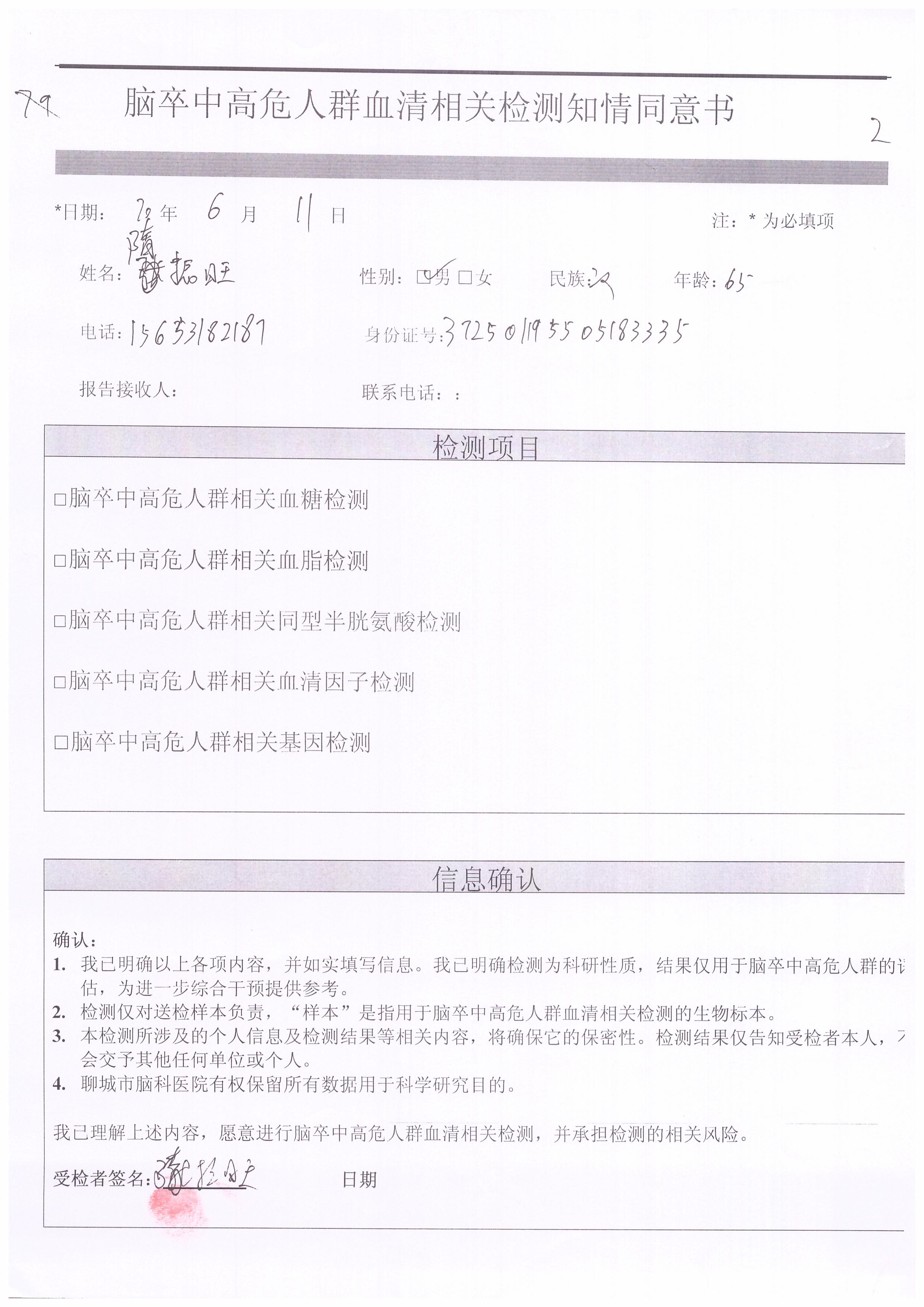

Supplement: Supplementary file 3 — Supplementary file3 (ZIP 25359 KB) [file 10528_2023_10431_MOESM3_ESM.zip › ╓¬╟Θ═1⁄4╥Γ╩Θ1/002 (2).jpg]

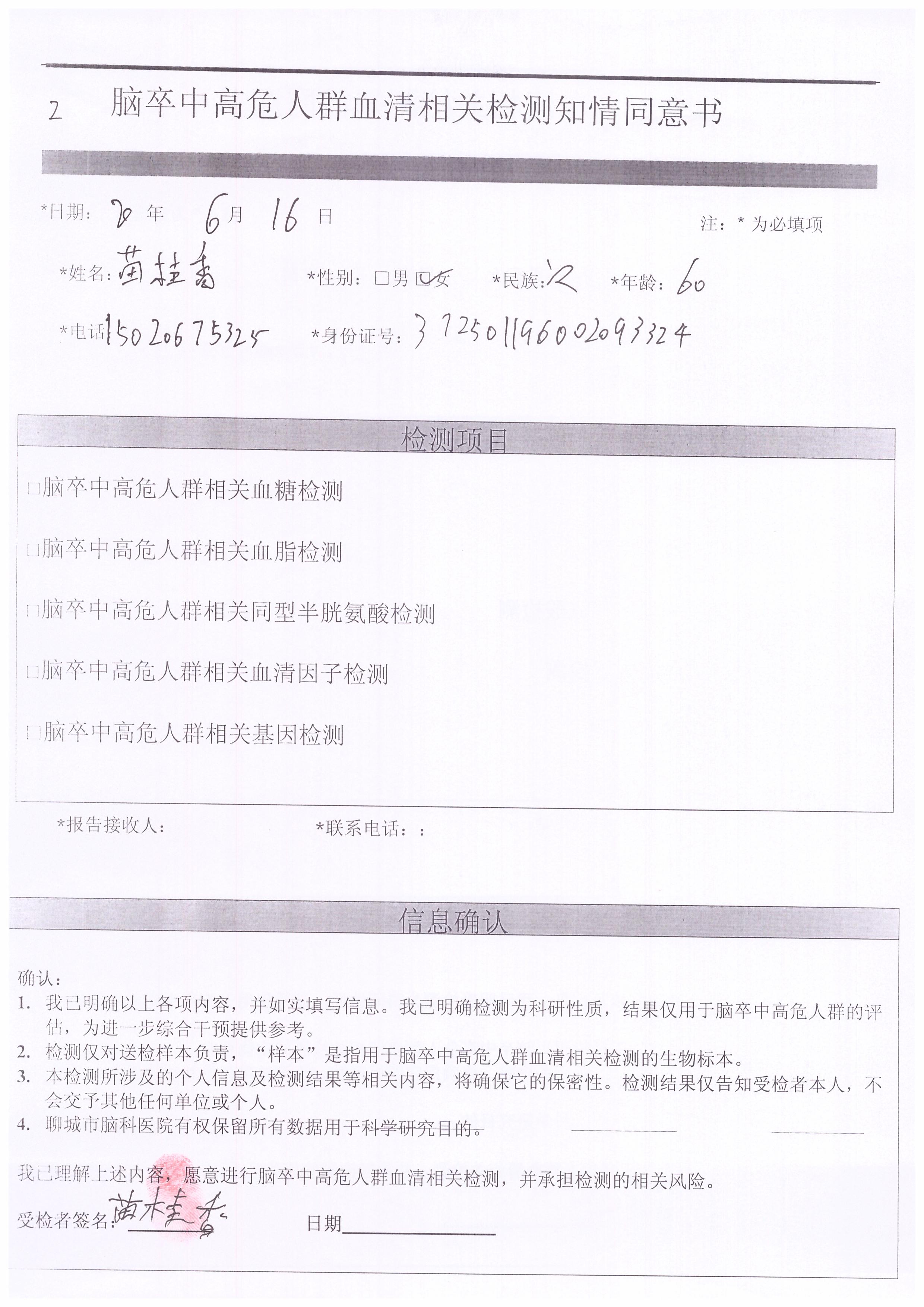

Supplement: Supplementary file 3 — Supplementary file3 (ZIP 25359 KB) [file 10528_2023_10431_MOESM3_ESM.zip › ╓¬╟Θ═1⁄4╥Γ╩Θ1/002.jpg]

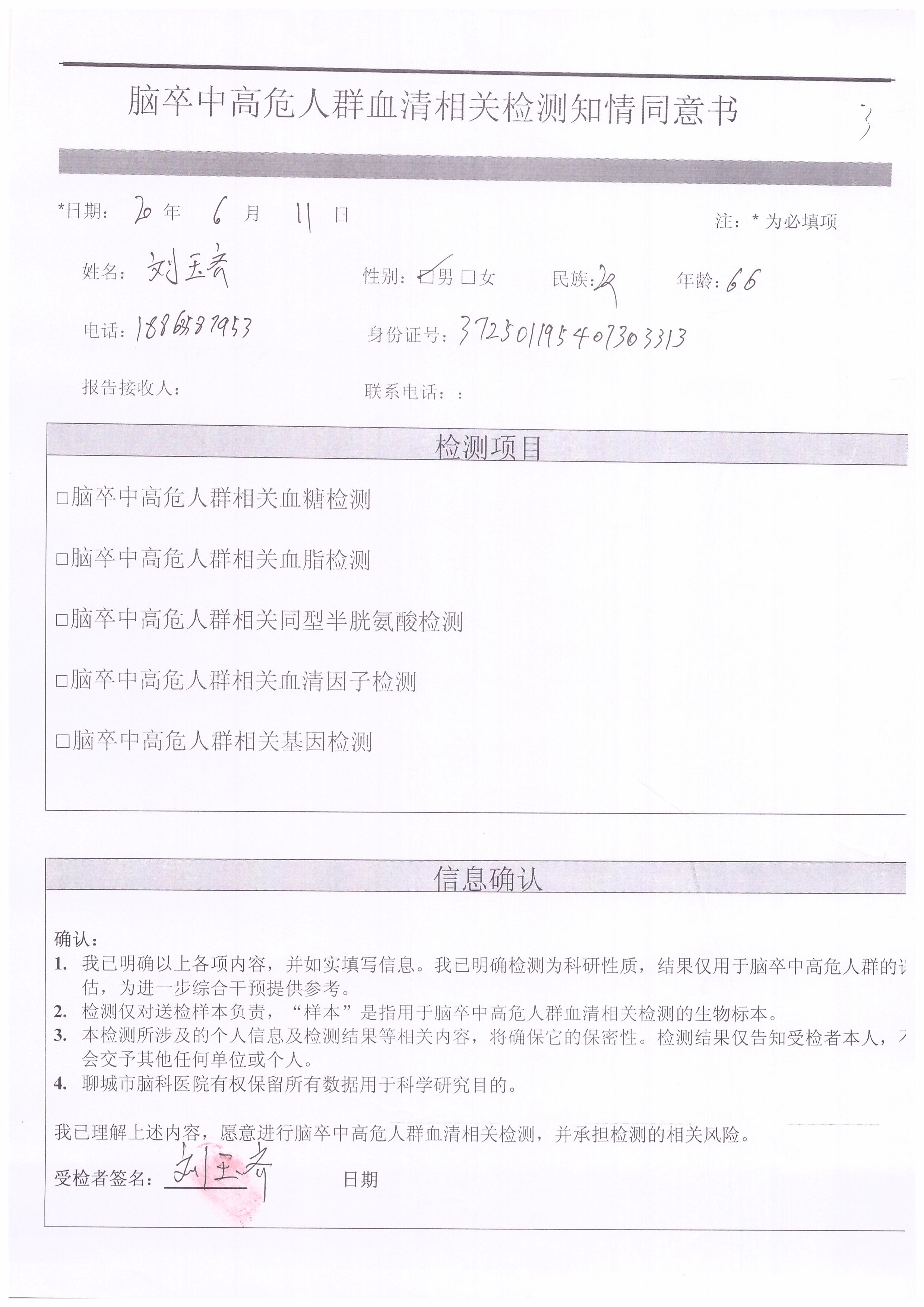

Supplement: Supplementary file 3 — Supplementary file3 (ZIP 25359 KB) [file 10528_2023_10431_MOESM3_ESM.zip › ╓¬╟Θ═1⁄4╥Γ╩Θ1/003 (2).jpg]

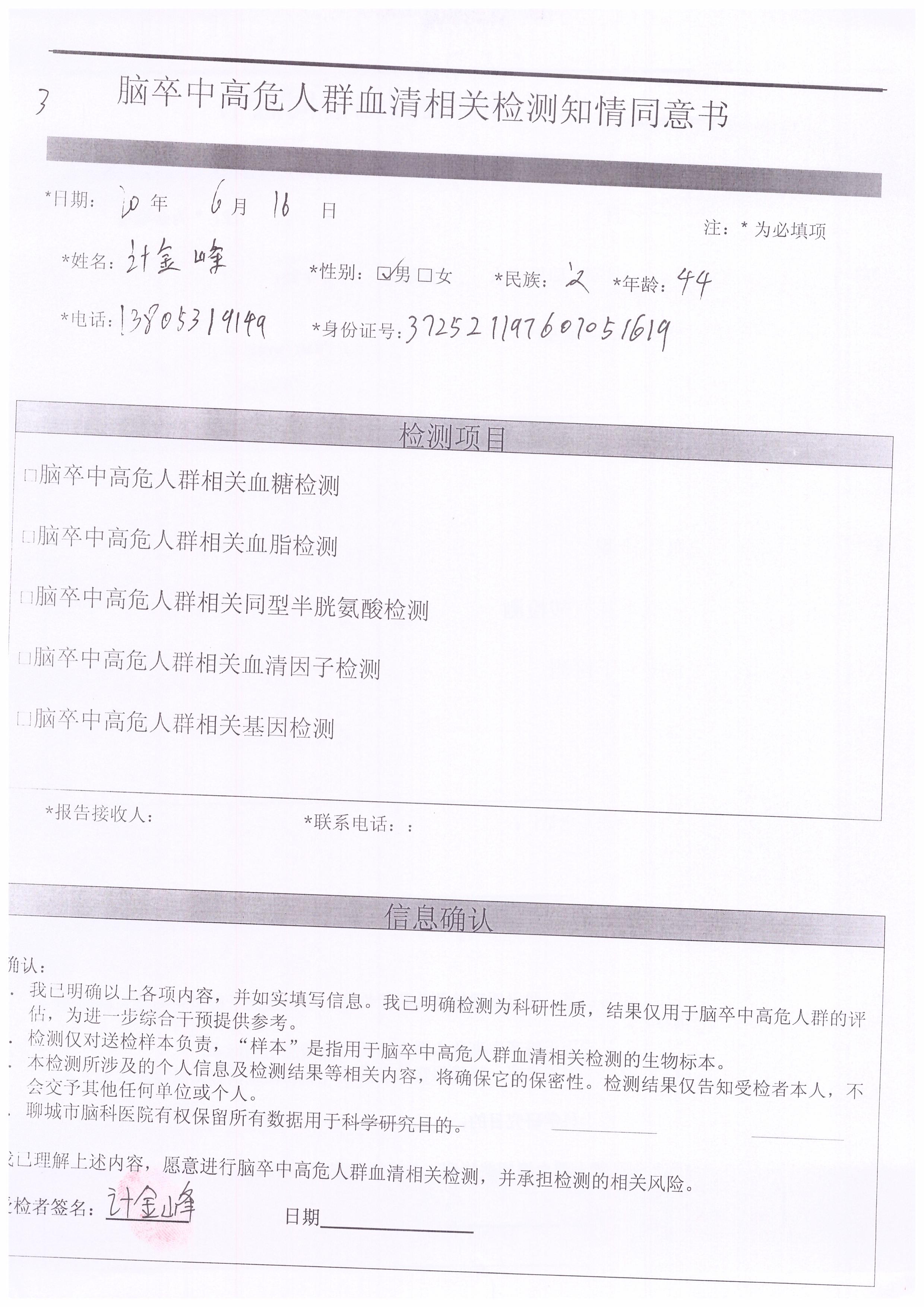

Supplement: Supplementary file 3 — Supplementary file3 (ZIP 25359 KB) [file 10528_2023_10431_MOESM3_ESM.zip › ╓¬╟Θ═1⁄4╥Γ╩Θ1/003.jpg]

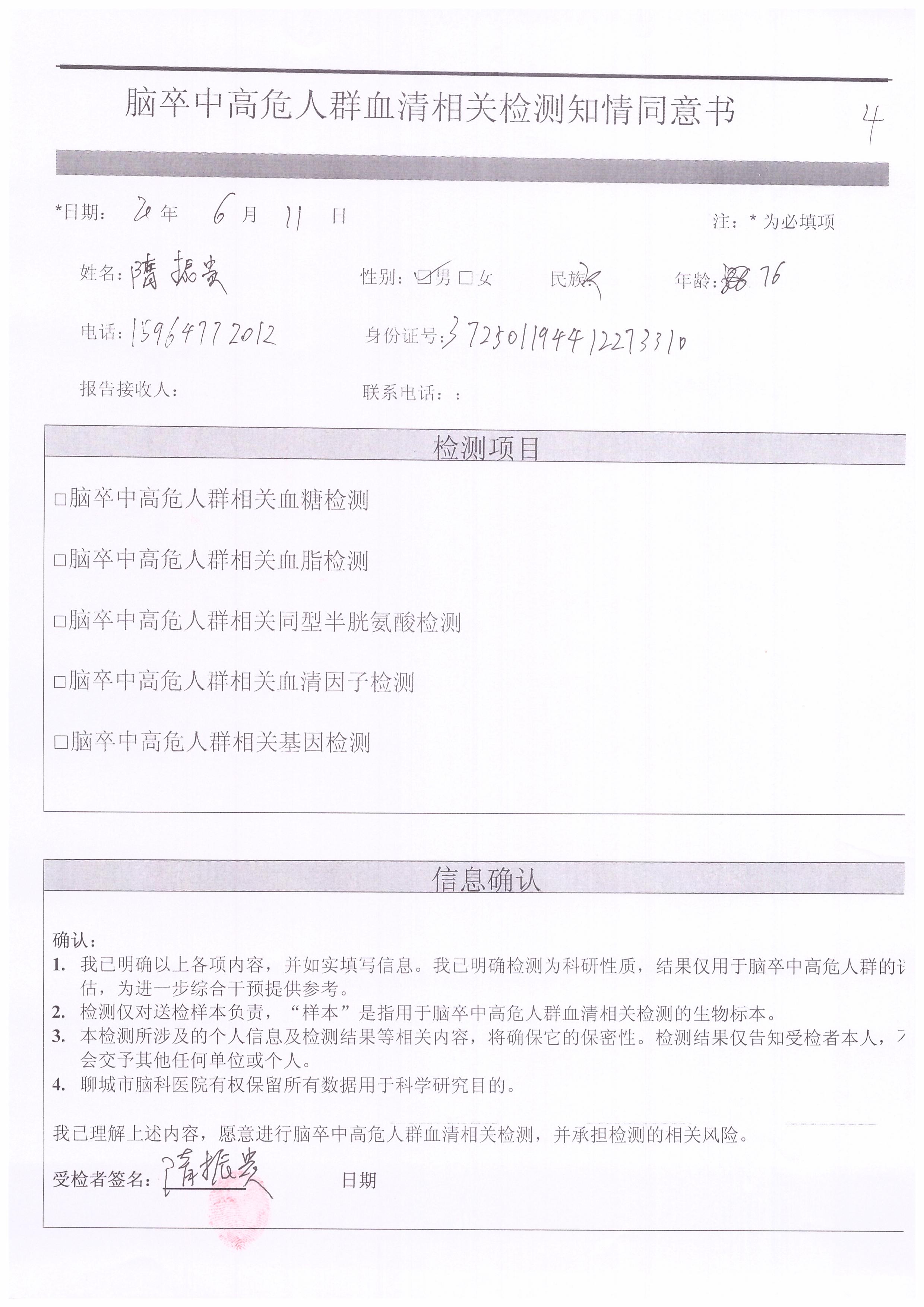

Supplement: Supplementary file 3 — Supplementary file3 (ZIP 25359 KB) [file 10528_2023_10431_MOESM3_ESM.zip › ╓¬╟Θ═1⁄4╥Γ╩Θ1/004 (2).jpg]

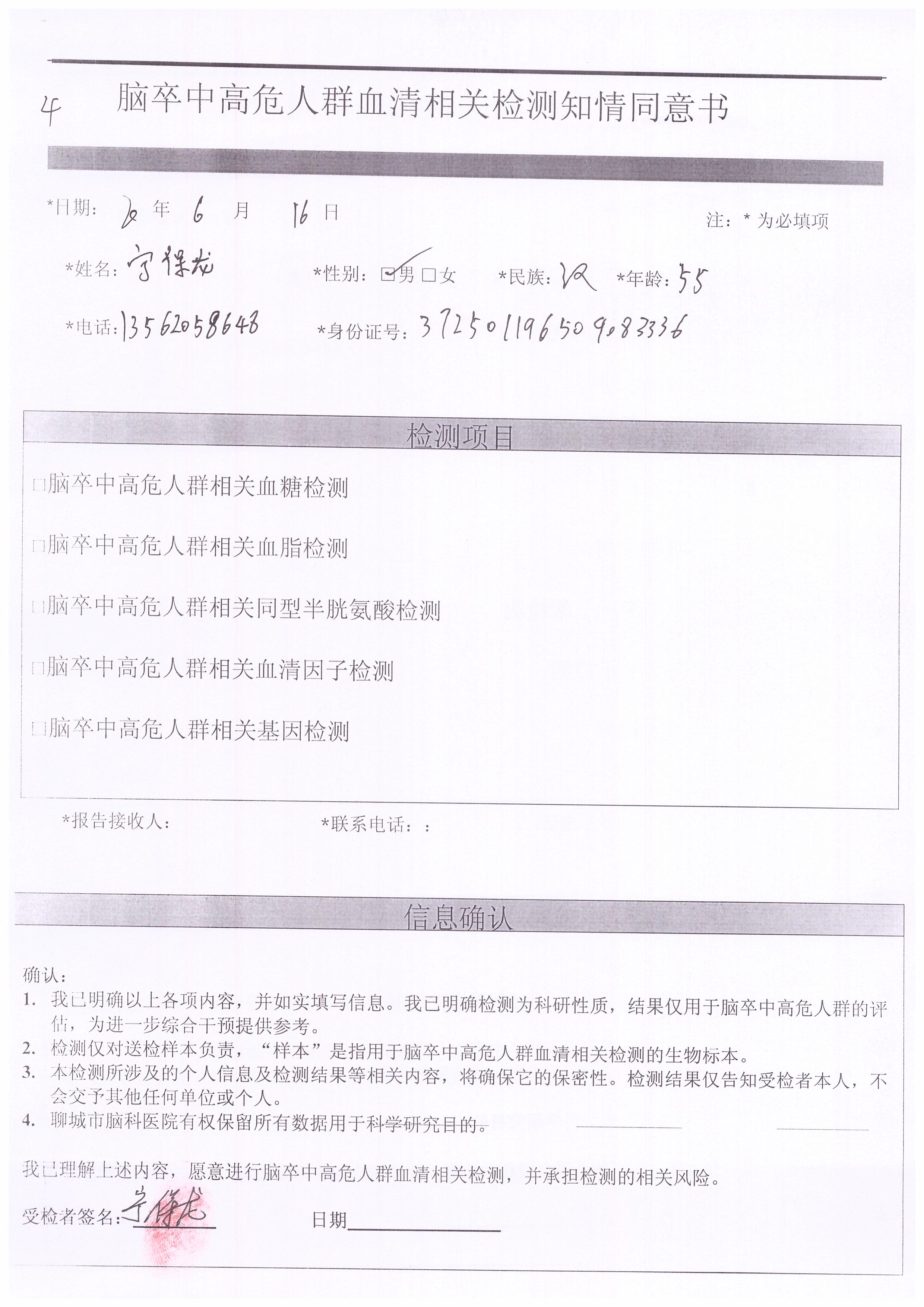

Supplement: Supplementary file 3 — Supplementary file3 (ZIP 25359 KB) [file 10528_2023_10431_MOESM3_ESM.zip › ╓¬╟Θ═1⁄4╥Γ╩Θ1/004.jpg]

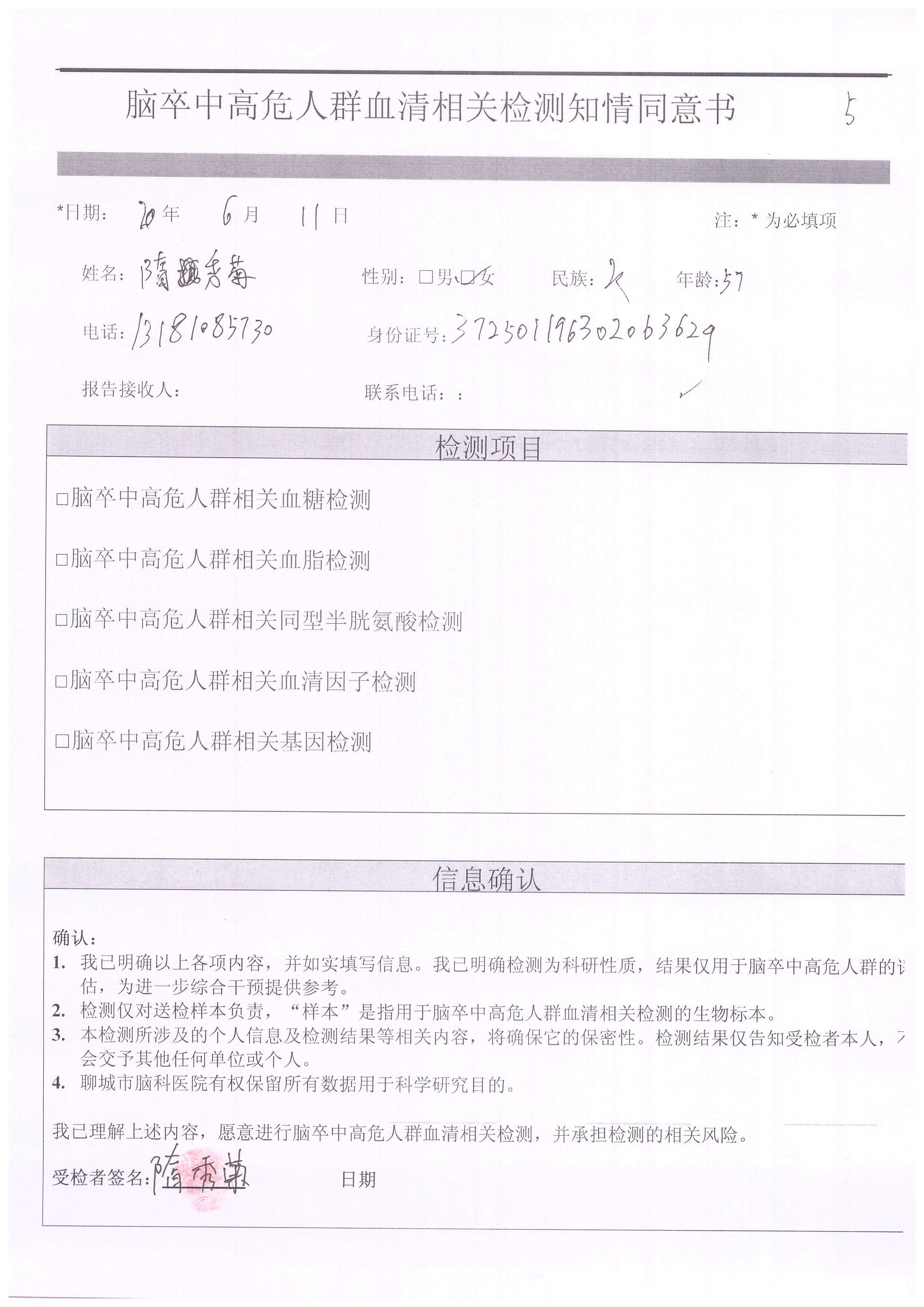

Supplement: Supplementary file 3 — Supplementary file3 (ZIP 25359 KB) [file 10528_2023_10431_MOESM3_ESM.zip › ╓¬╟Θ═1⁄4╥Γ╩Θ1/005 (2).jpg]

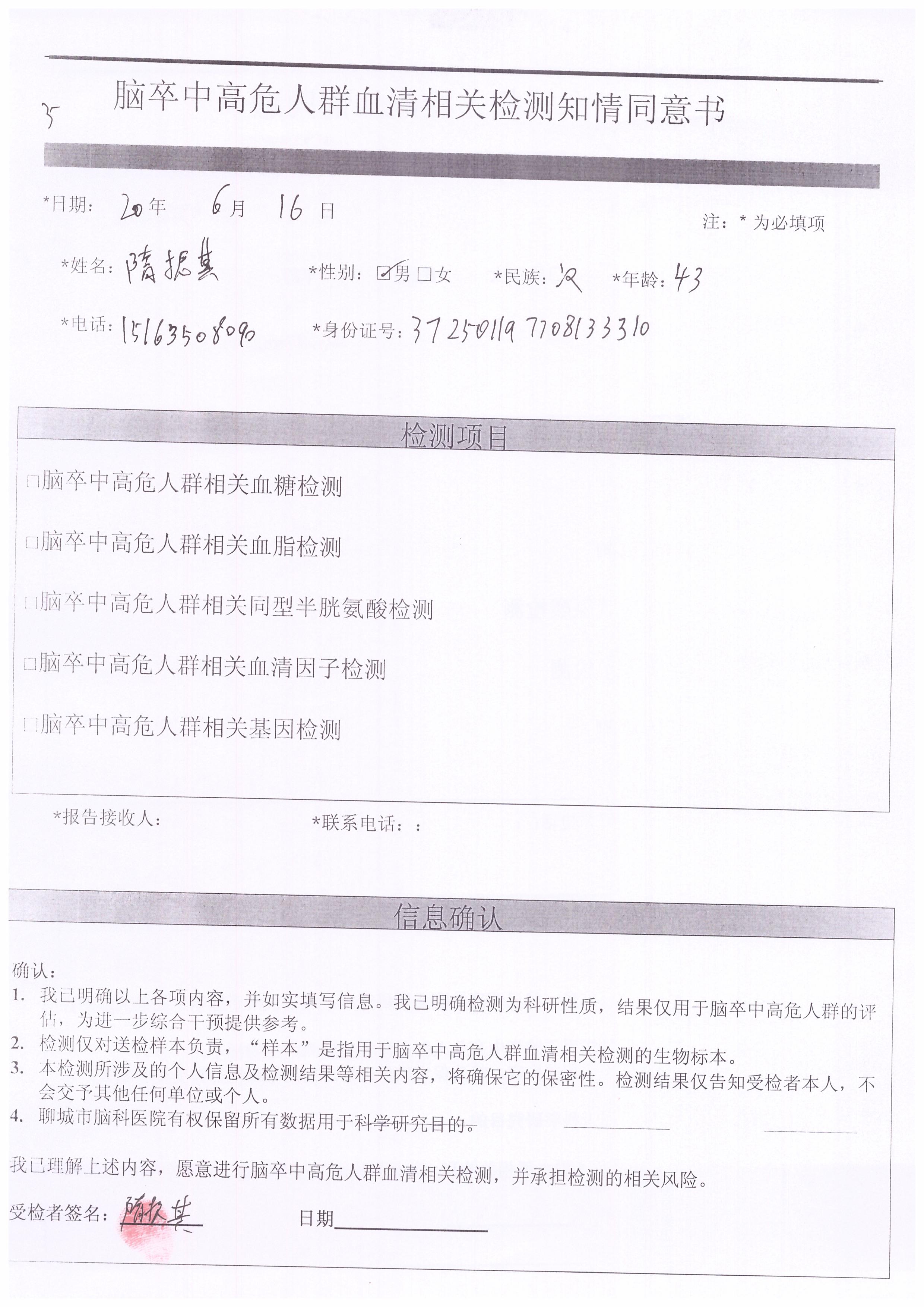

Supplement: Supplementary file 3 — Supplementary file3 (ZIP 25359 KB) [file 10528_2023_10431_MOESM3_ESM.zip › ╓¬╟Θ═1⁄4╥Γ╩Θ1/005.jpg]

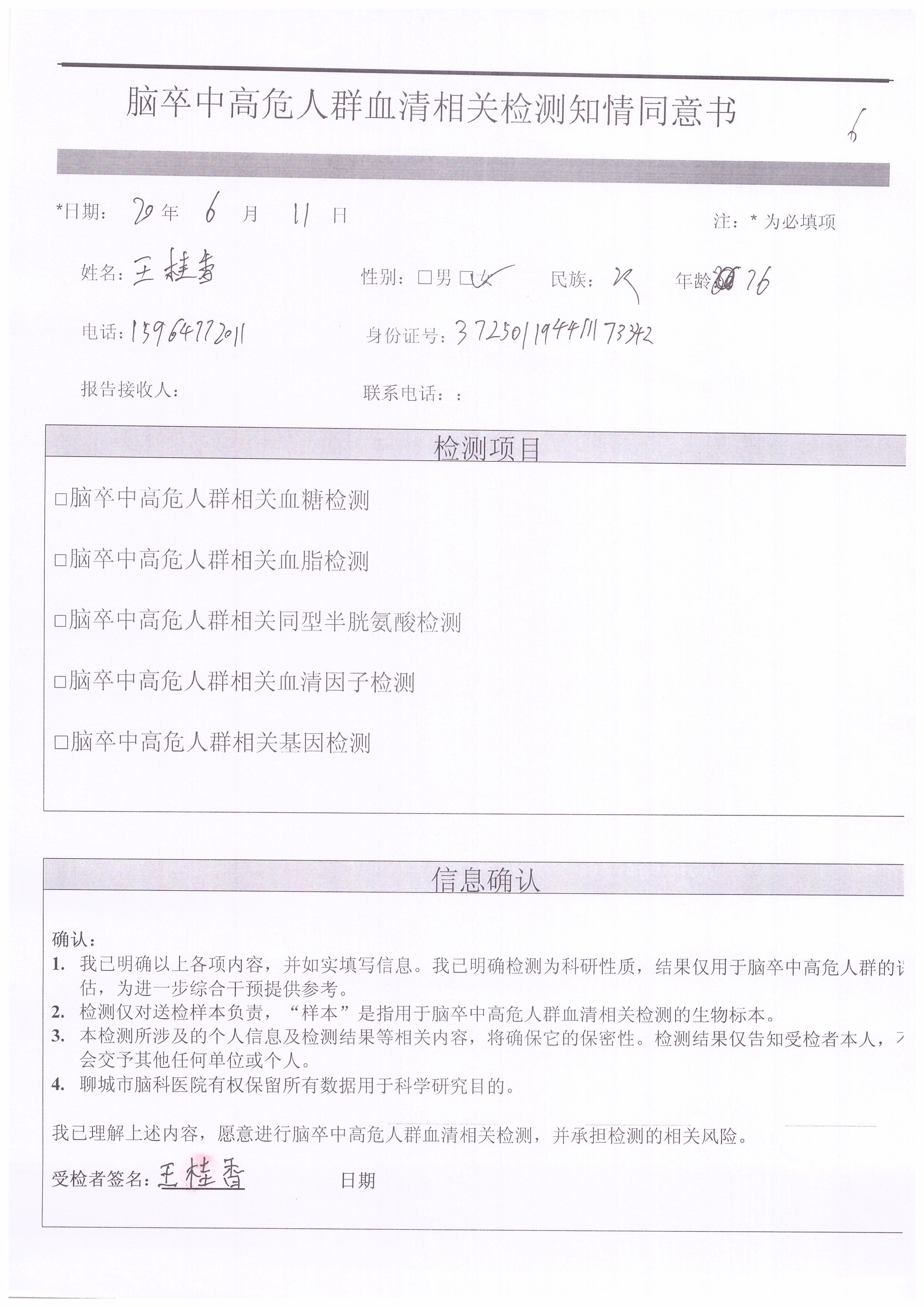

Supplement: Supplementary file 3 — Supplementary file3 (ZIP 25359 KB) [file 10528_2023_10431_MOESM3_ESM.zip › ╓¬╟Θ═1⁄4╥Γ╩Θ1/006 (2).jpg]

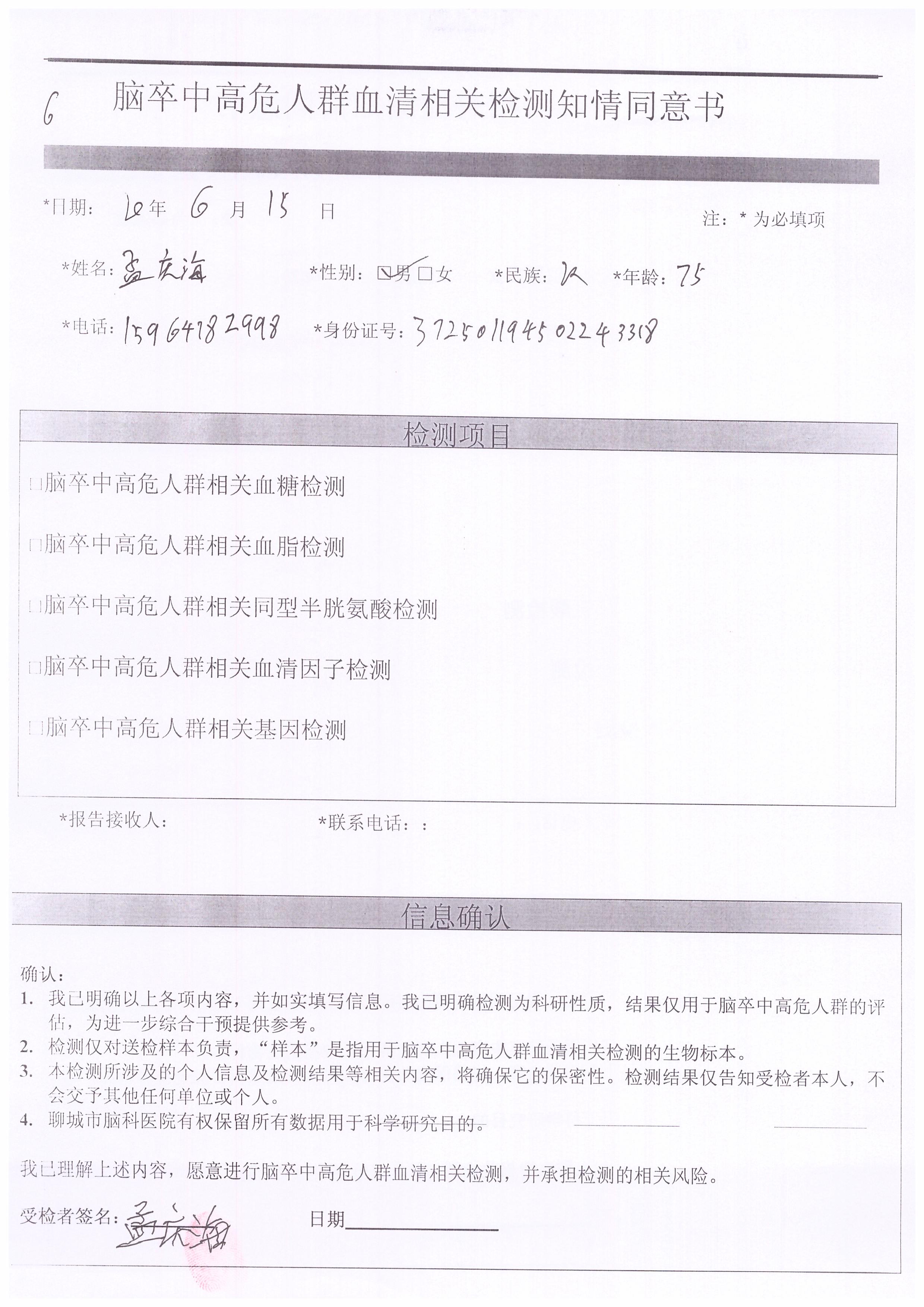

Supplement: Supplementary file 3 — Supplementary file3 (ZIP 25359 KB) [file 10528_2023_10431_MOESM3_ESM.zip › ╓¬╟Θ═1⁄4╥Γ╩Θ1/006.jpg]

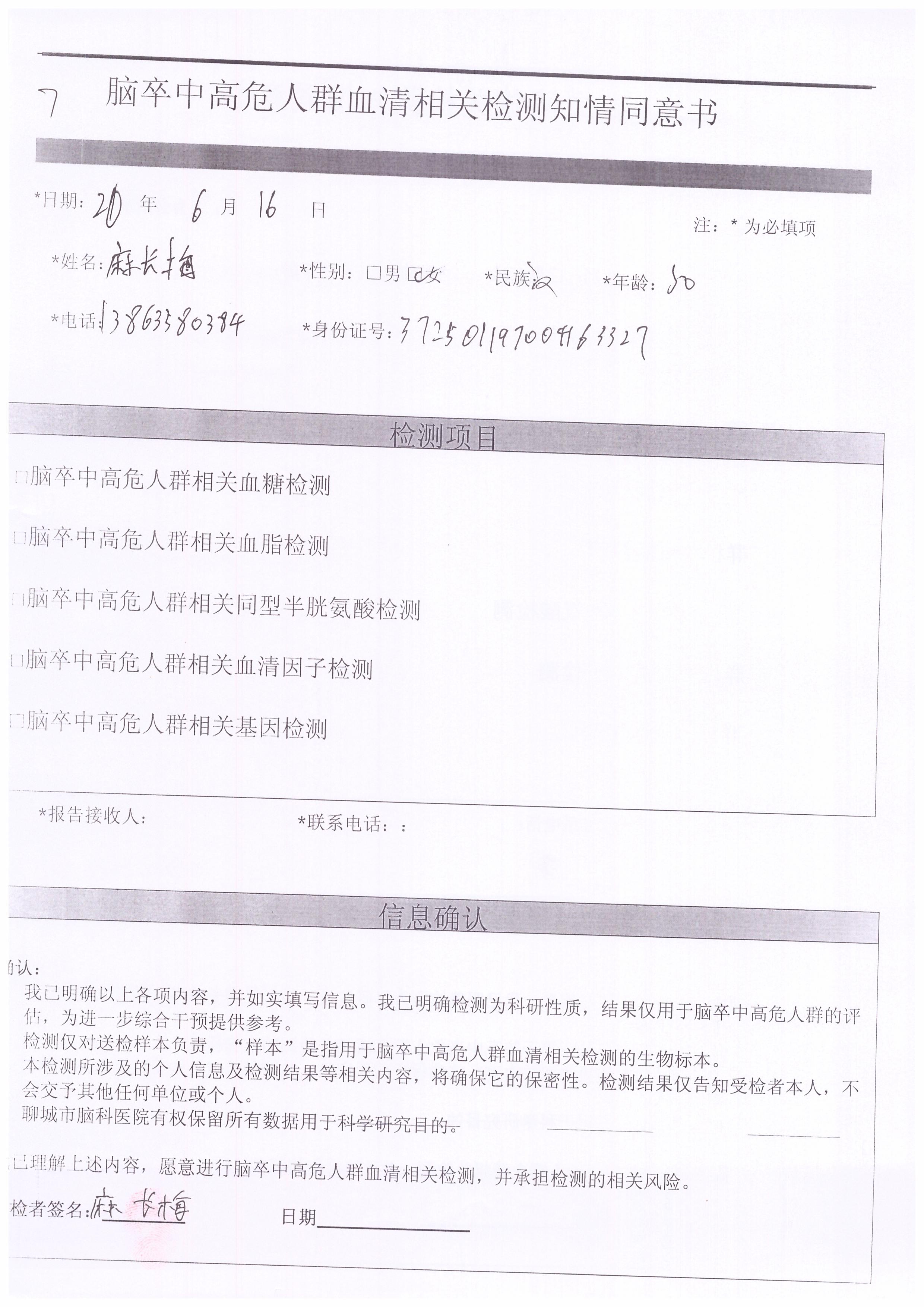

Supplement: Supplementary file 3 — Supplementary file3 (ZIP 25359 KB) [file 10528_2023_10431_MOESM3_ESM.zip › ╓¬╟Θ═1⁄4╥Γ╩Θ1/007.jpg]

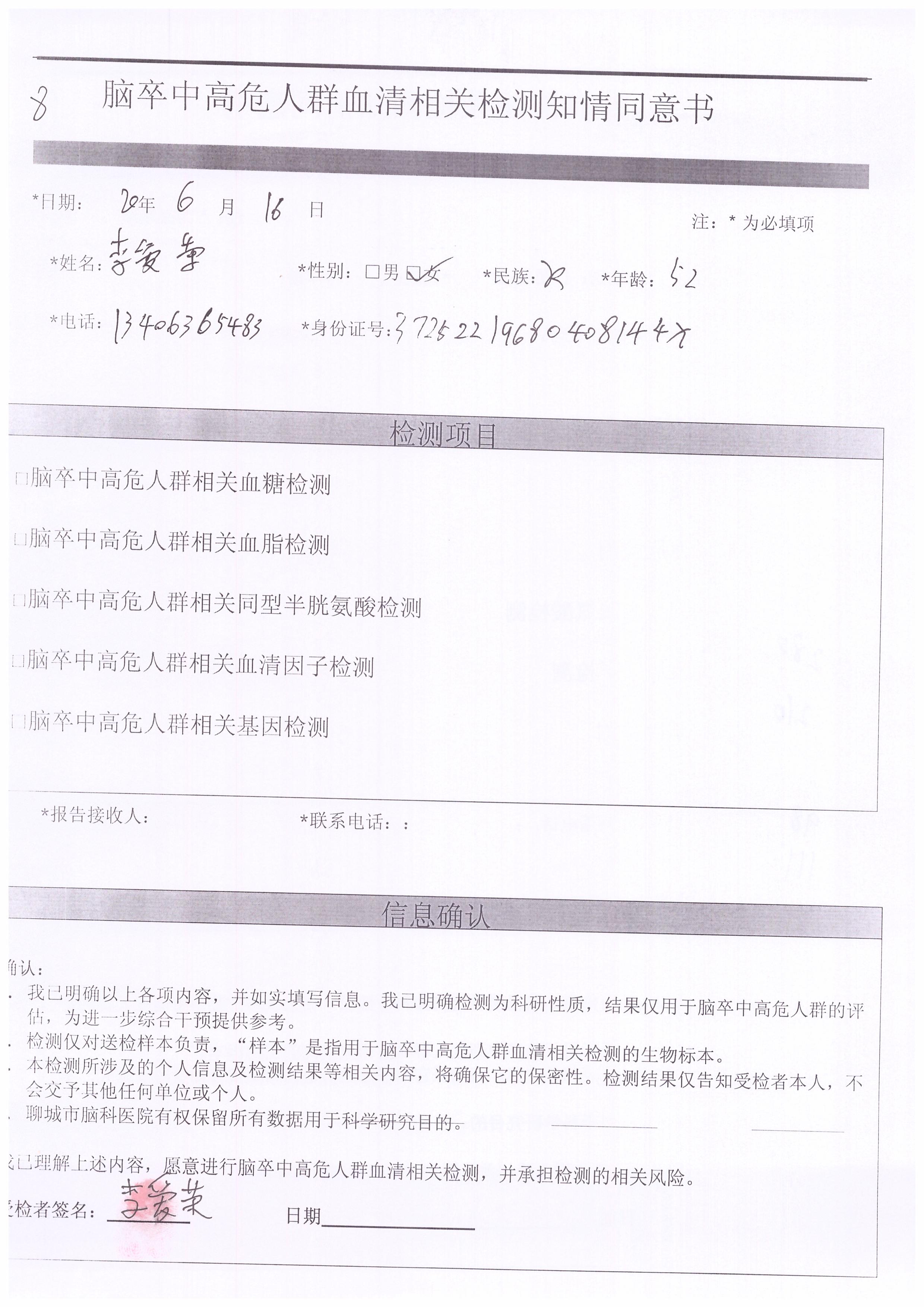

Supplement: Supplementary file 3 — Supplementary file3 (ZIP 25359 KB) [file 10528_2023_10431_MOESM3_ESM.zip › ╓¬╟Θ═1⁄4╥Γ╩Θ1/008.jpg]

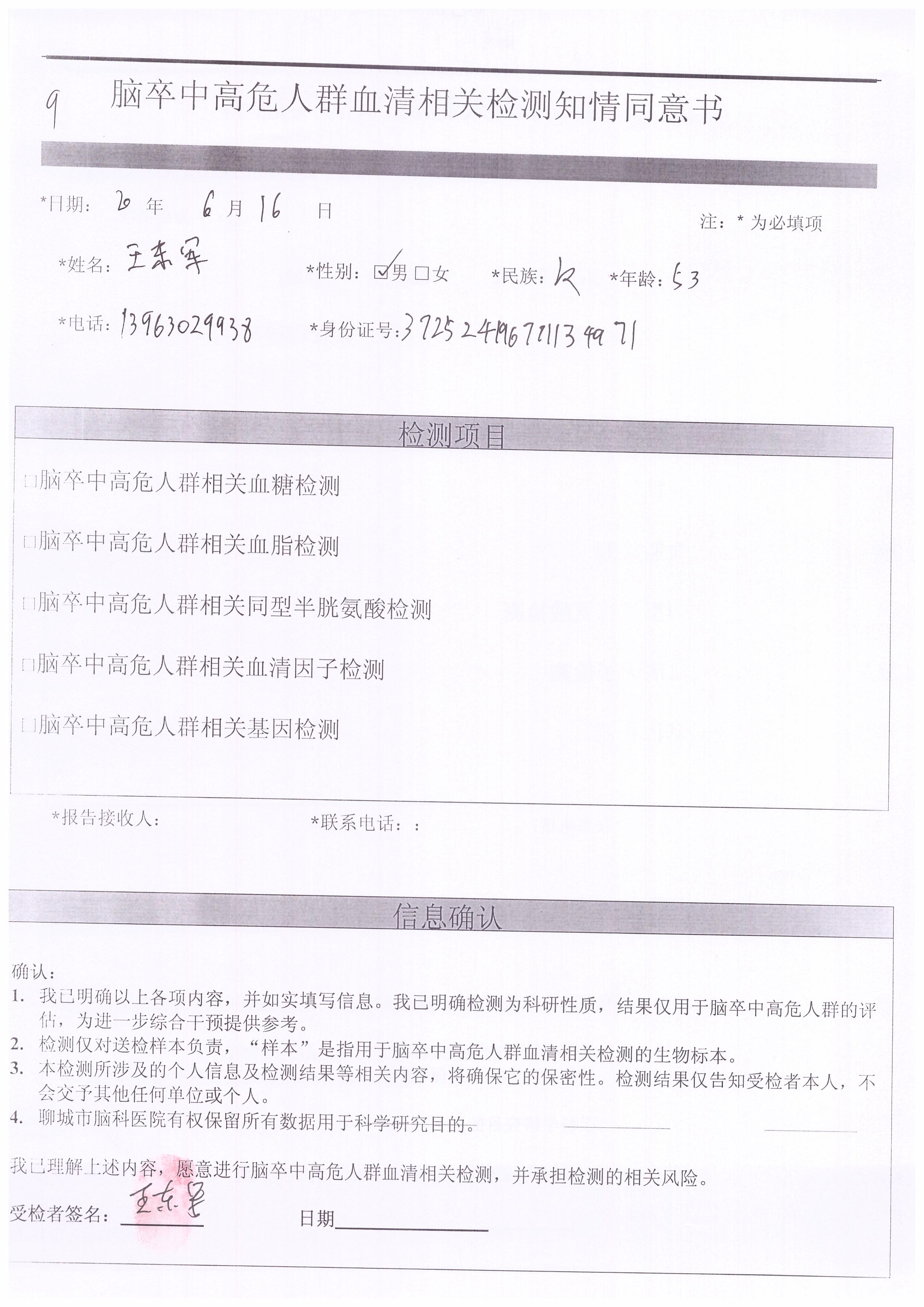

Supplement: Supplementary file 3 — Supplementary file3 (ZIP 25359 KB) [file 10528_2023_10431_MOESM3_ESM.zip › ╓¬╟Θ═1⁄4╥Γ╩Θ1/009.jpg]

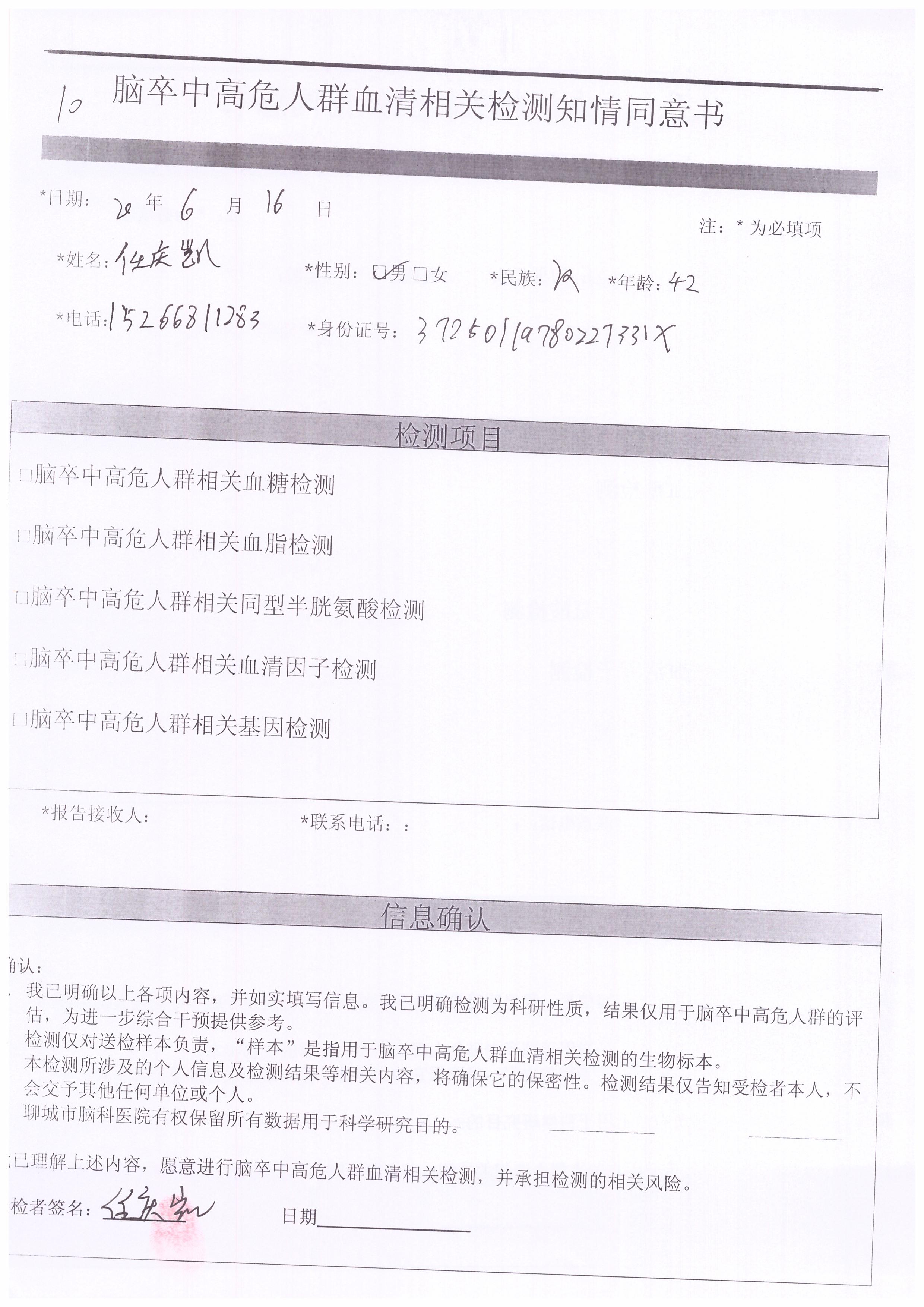

Supplement: Supplementary file 3 — Supplementary file3 (ZIP 25359 KB) [file 10528_2023_10431_MOESM3_ESM.zip › ╓¬╟Θ═1⁄4╥Γ╩Θ1/010.jpg]

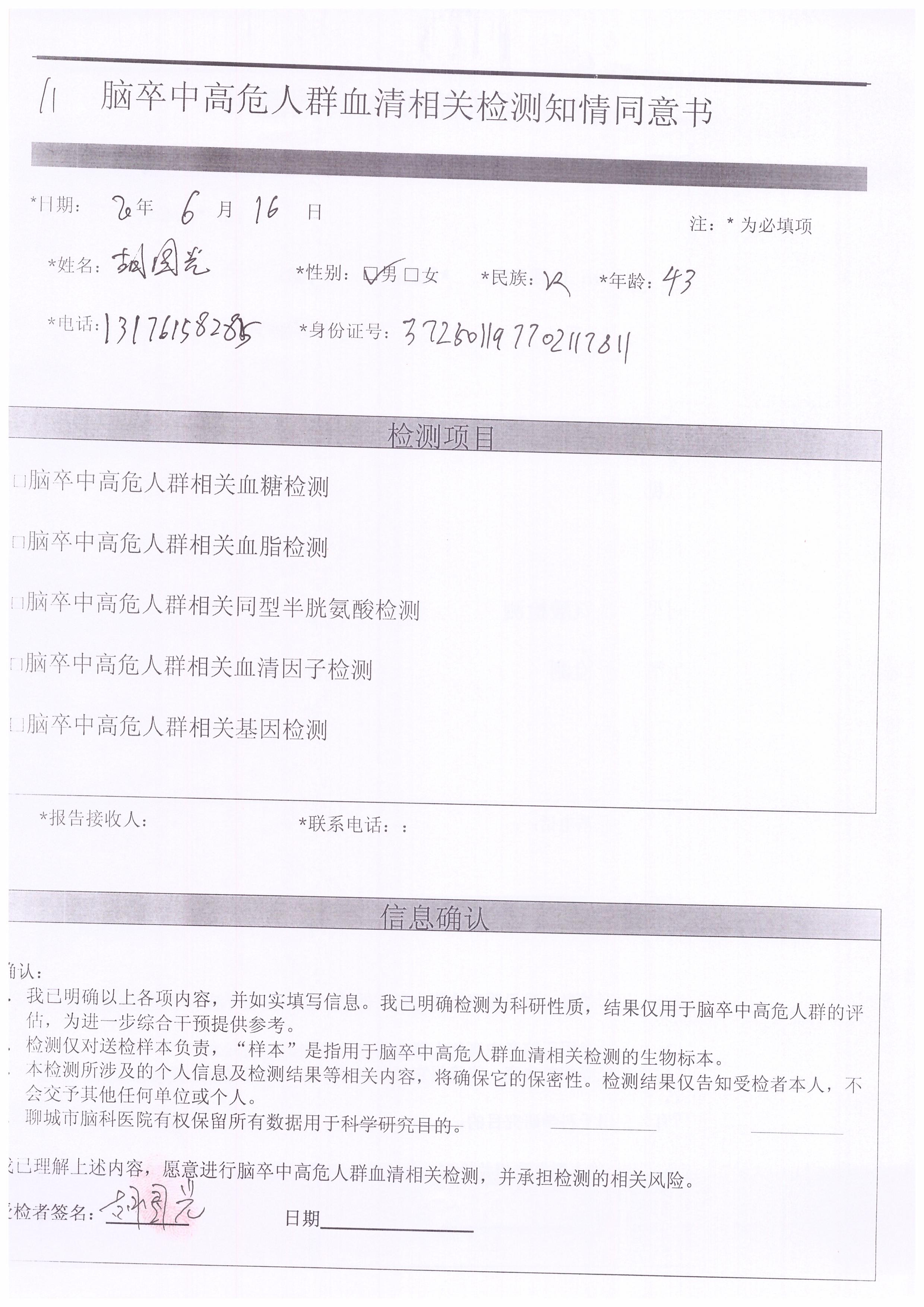

Supplement: Supplementary file 3 — Supplementary file3 (ZIP 25359 KB) [file 10528_2023_10431_MOESM3_ESM.zip › ╓¬╟Θ═1⁄4╥Γ╩Θ1/011.jpg]

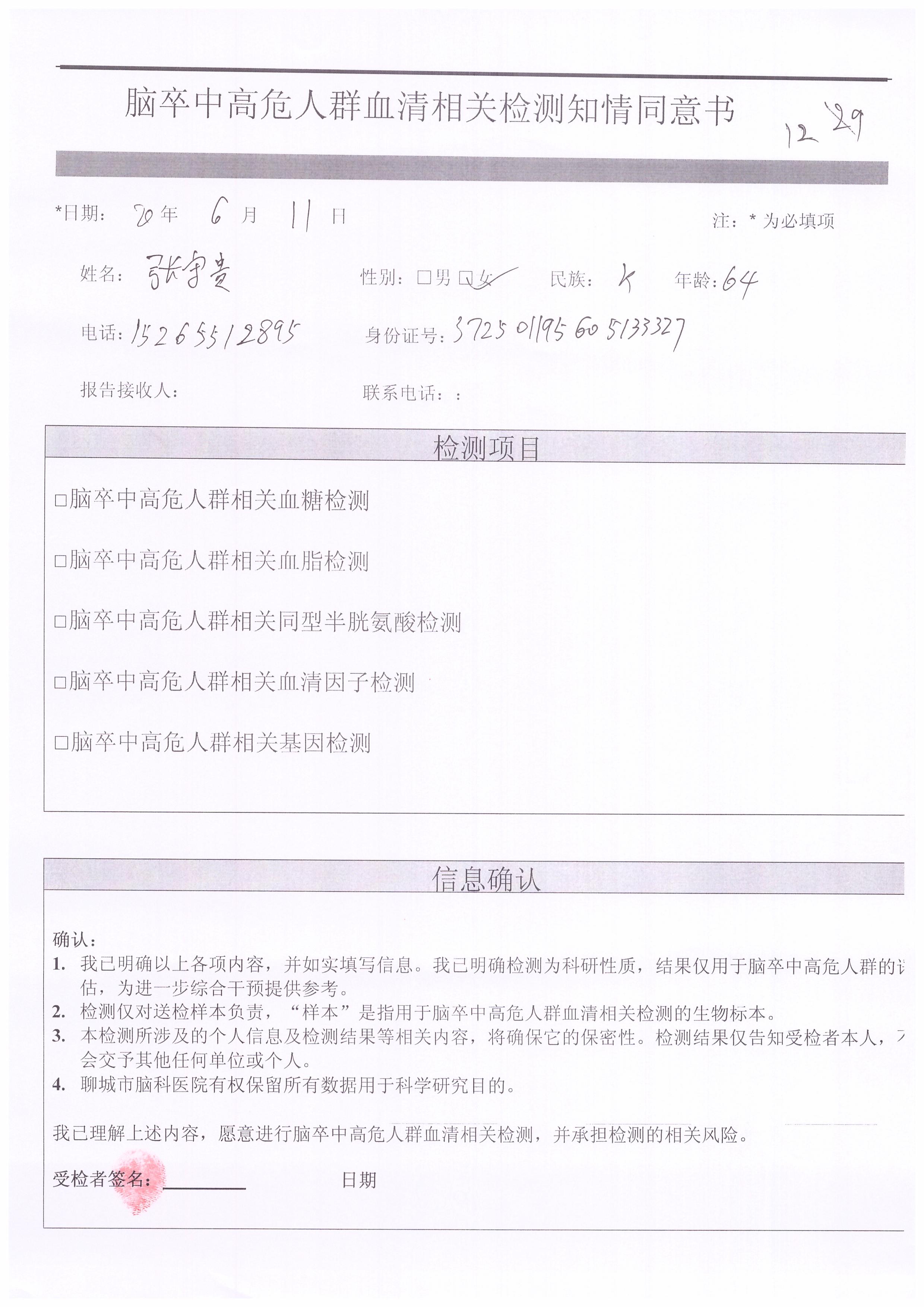

Supplement: Supplementary file 3 — Supplementary file3 (ZIP 25359 KB) [file 10528_2023_10431_MOESM3_ESM.zip › ╓¬╟Θ═1⁄4╥Γ╩Θ1/012 (2).jpg]

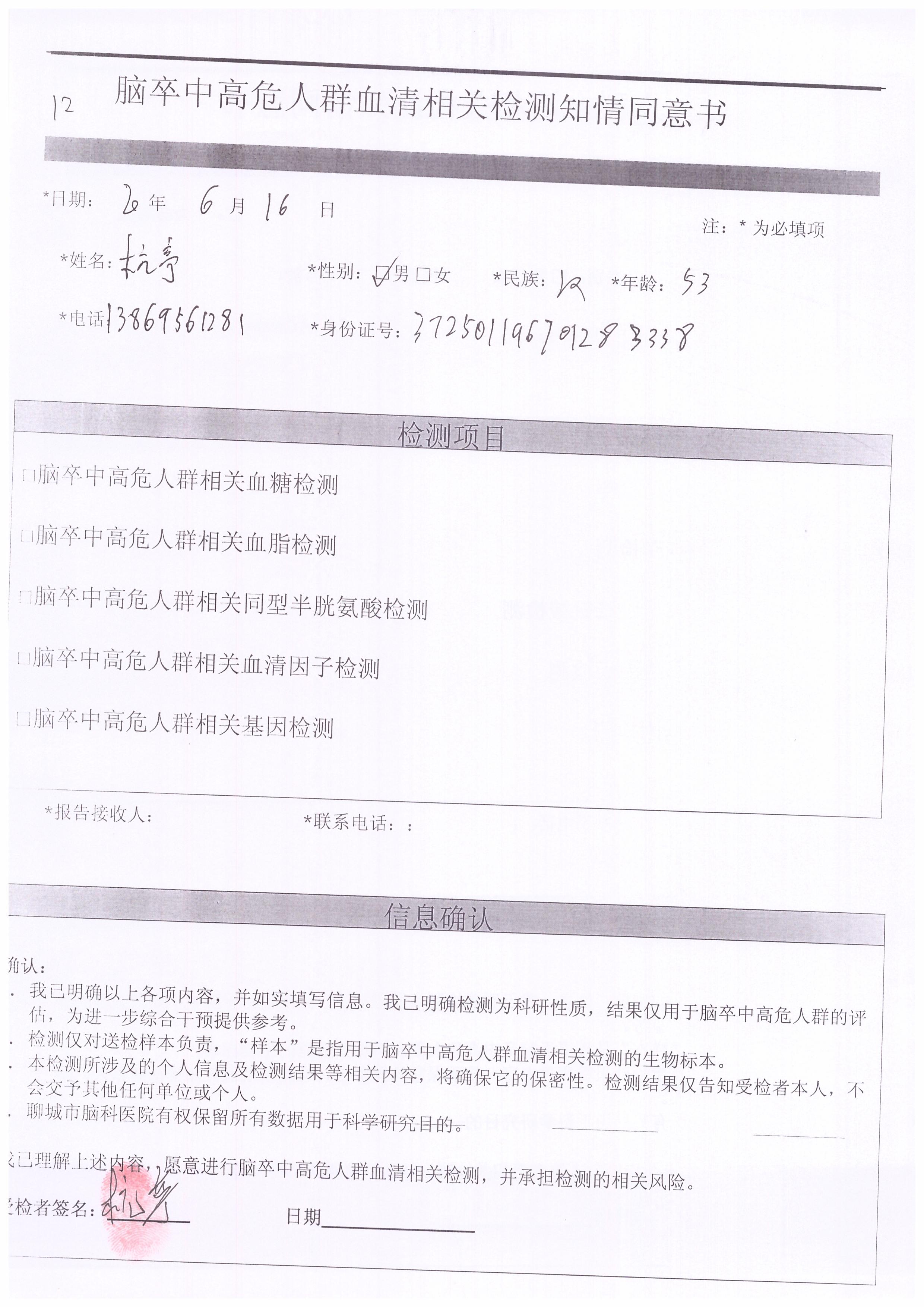

Supplement: Supplementary file 3 — Supplementary file3 (ZIP 25359 KB) [file 10528_2023_10431_MOESM3_ESM.zip › ╓¬╟Θ═1⁄4╥Γ╩Θ1/012.jpg]

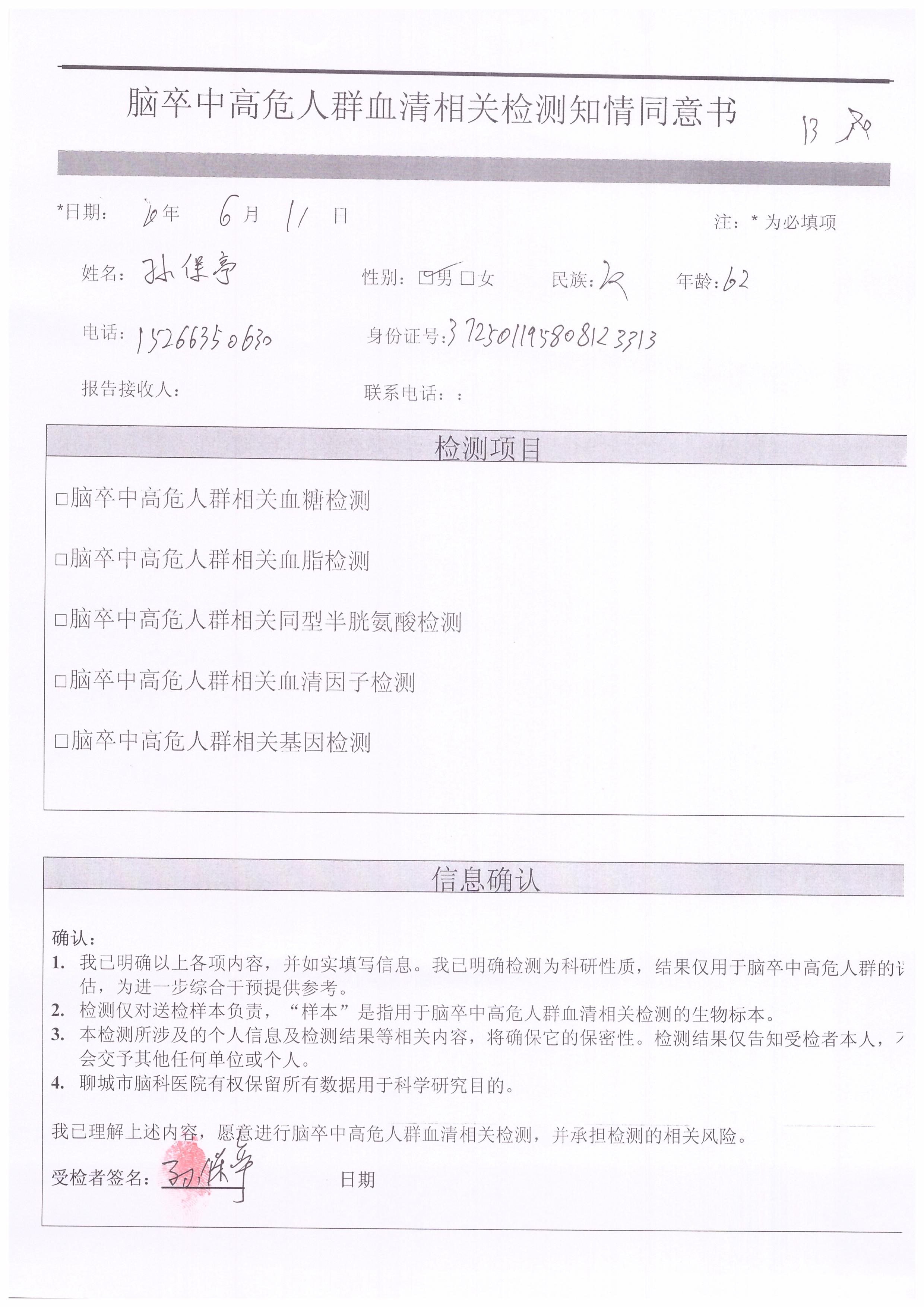

Supplement: Supplementary file 3 — Supplementary file3 (ZIP 25359 KB) [file 10528_2023_10431_MOESM3_ESM.zip › ╓¬╟Θ═1⁄4╥Γ╩Θ1/013 (2).jpg]

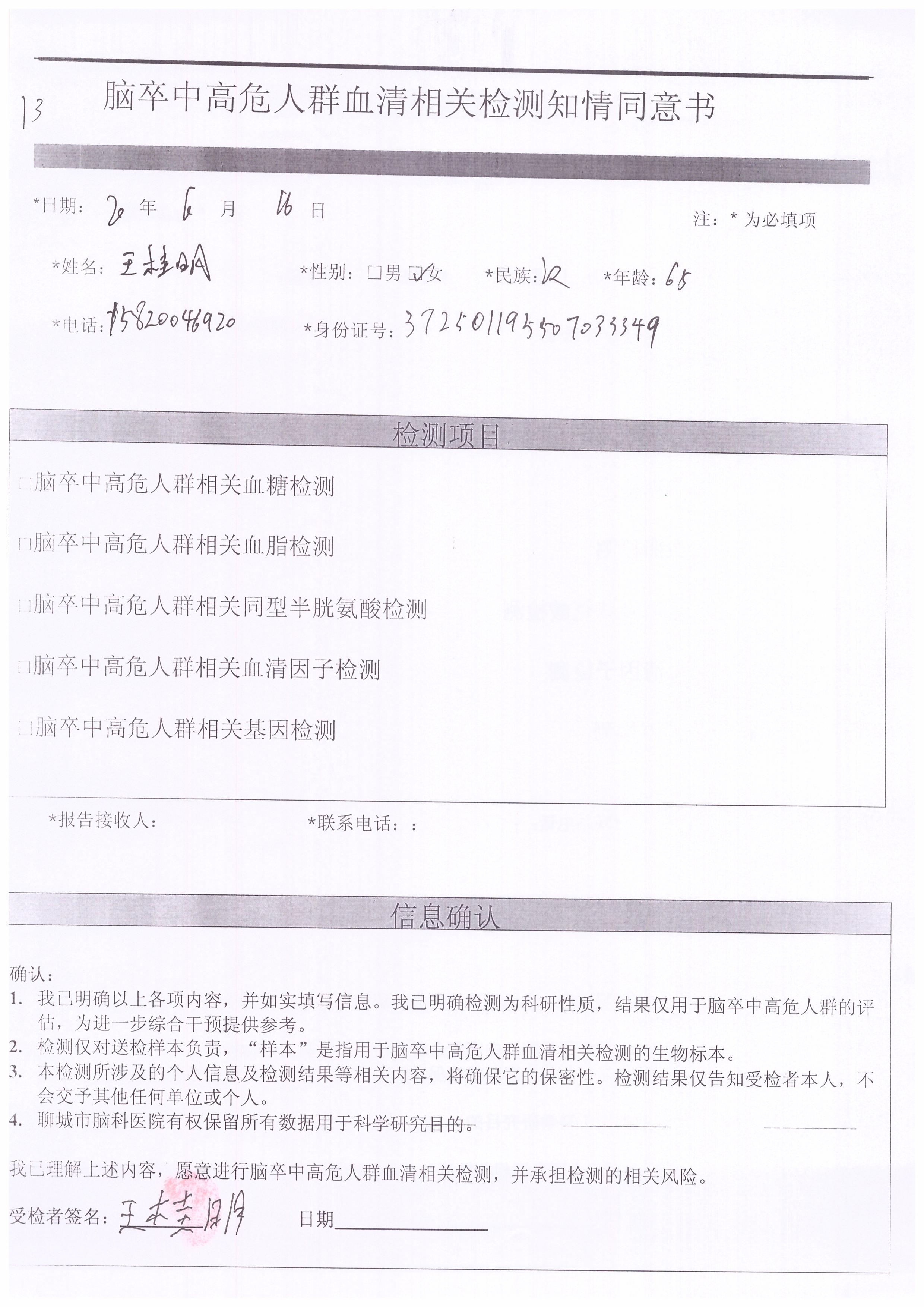

Supplement: Supplementary file 3 — Supplementary file3 (ZIP 25359 KB) [file 10528_2023_10431_MOESM3_ESM.zip › ╓¬╟Θ═1⁄4╥Γ╩Θ1/013.jpg]

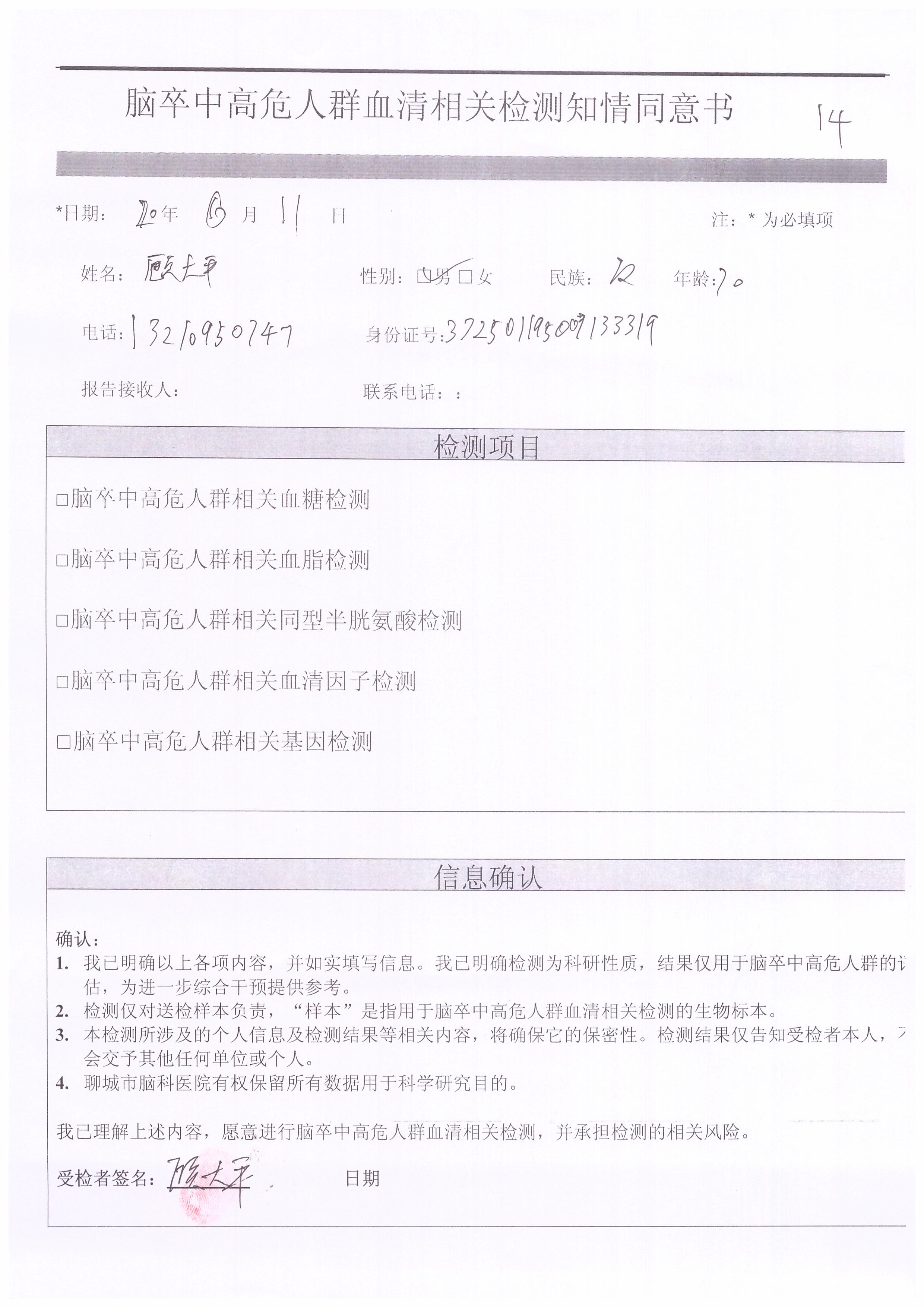

Supplement: Supplementary file 3 — Supplementary file3 (ZIP 25359 KB) [file 10528_2023_10431_MOESM3_ESM.zip › ╓¬╟Θ═1⁄4╥Γ╩Θ1/014 (2).jpg]

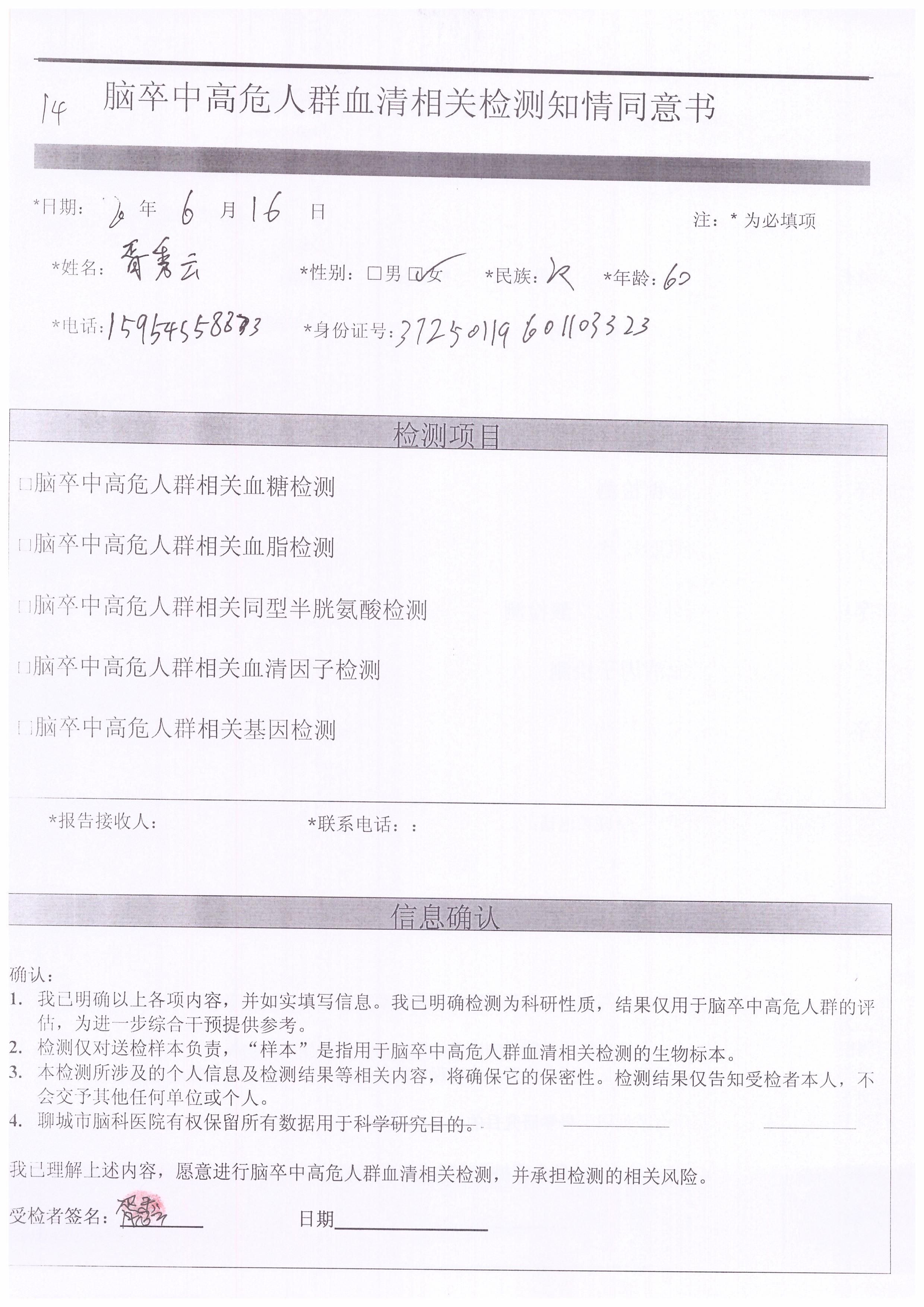

Supplement: Supplementary file 3 — Supplementary file3 (ZIP 25359 KB) [file 10528_2023_10431_MOESM3_ESM.zip › ╓¬╟Θ═1⁄4╥Γ╩Θ1/014.jpg]

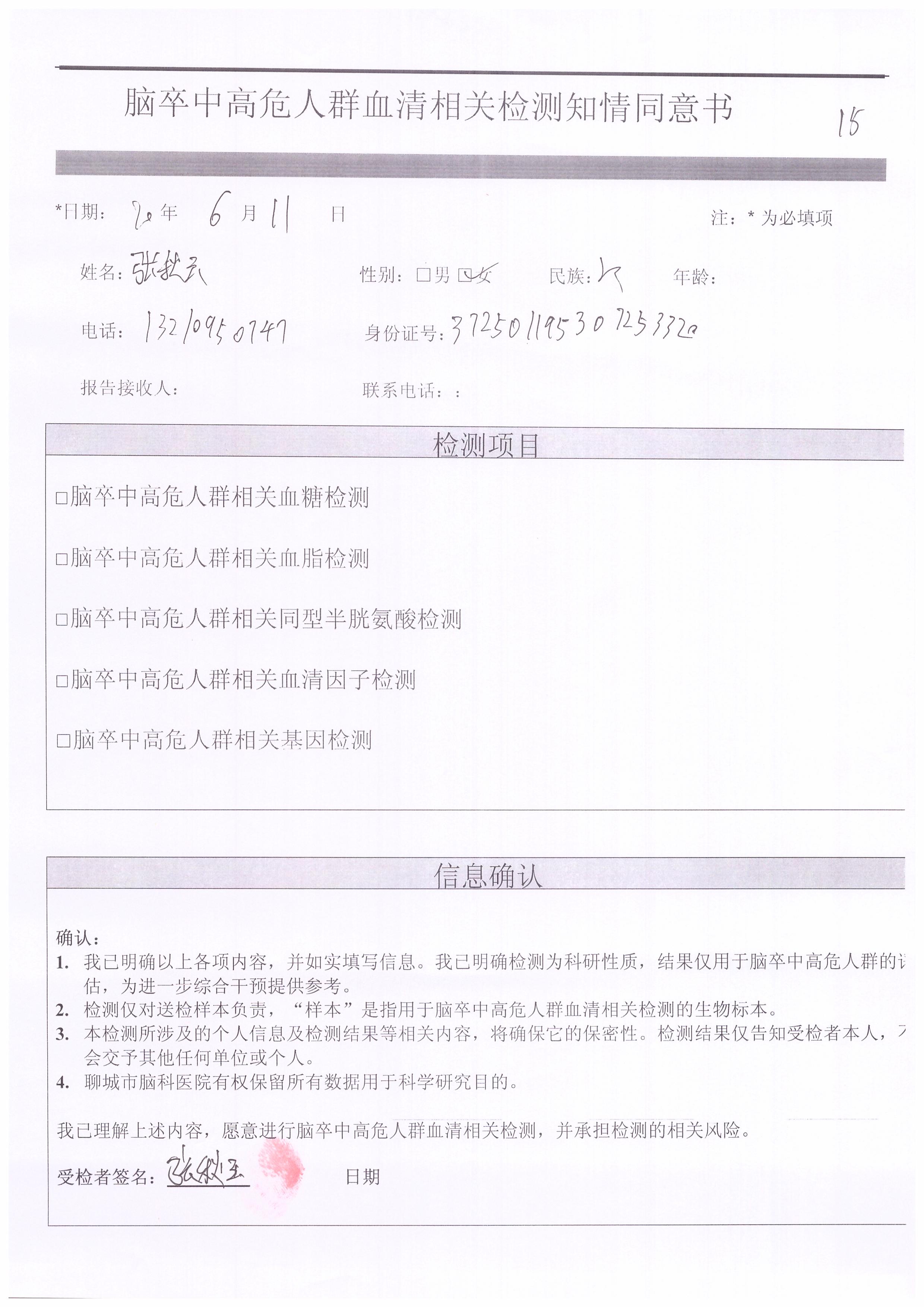

Supplement: Supplementary file 3 — Supplementary file3 (ZIP 25359 KB) [file 10528_2023_10431_MOESM3_ESM.zip › ╓¬╟Θ═1⁄4╥Γ╩Θ1/015 (2).jpg]

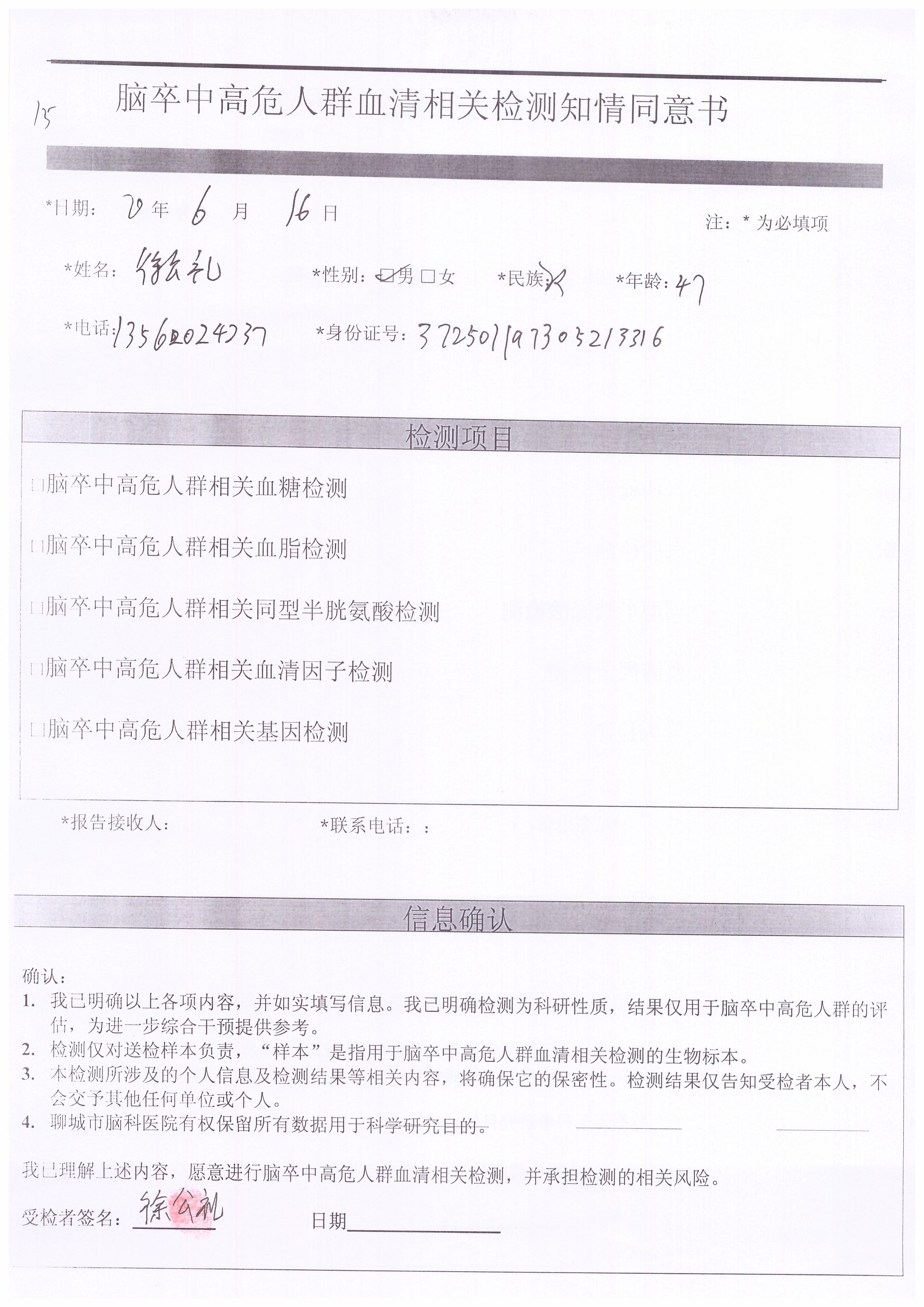

Supplement: Supplementary file 3 — Supplementary file3 (ZIP 25359 KB) [file 10528_2023_10431_MOESM3_ESM.zip › ╓¬╟Θ═1⁄4╥Γ╩Θ1/015.jpg]

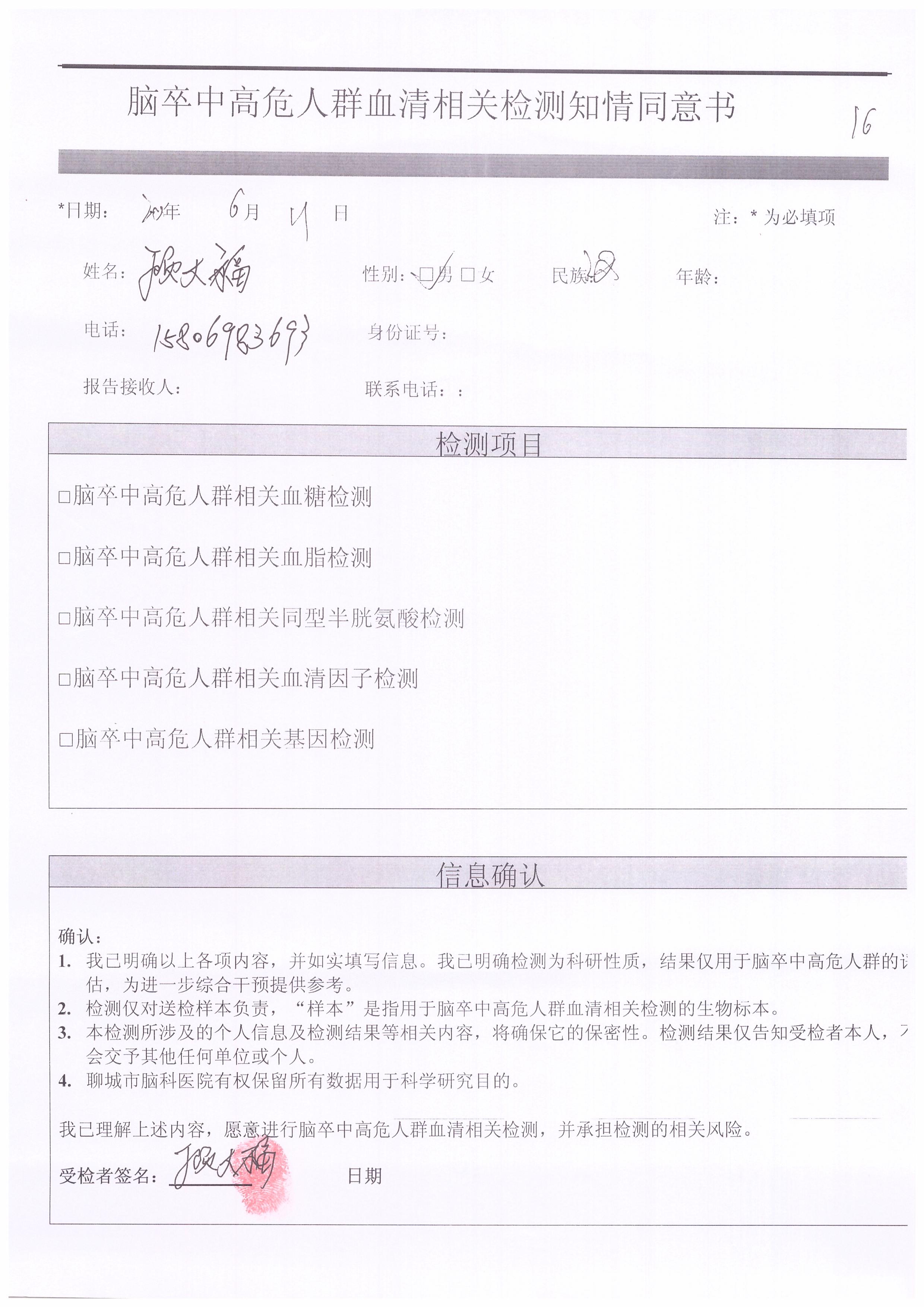

Supplement: Supplementary file 3 — Supplementary file3 (ZIP 25359 KB) [file 10528_2023_10431_MOESM3_ESM.zip › ╓¬╟Θ═1⁄4╥Γ╩Θ1/016 (2).jpg]

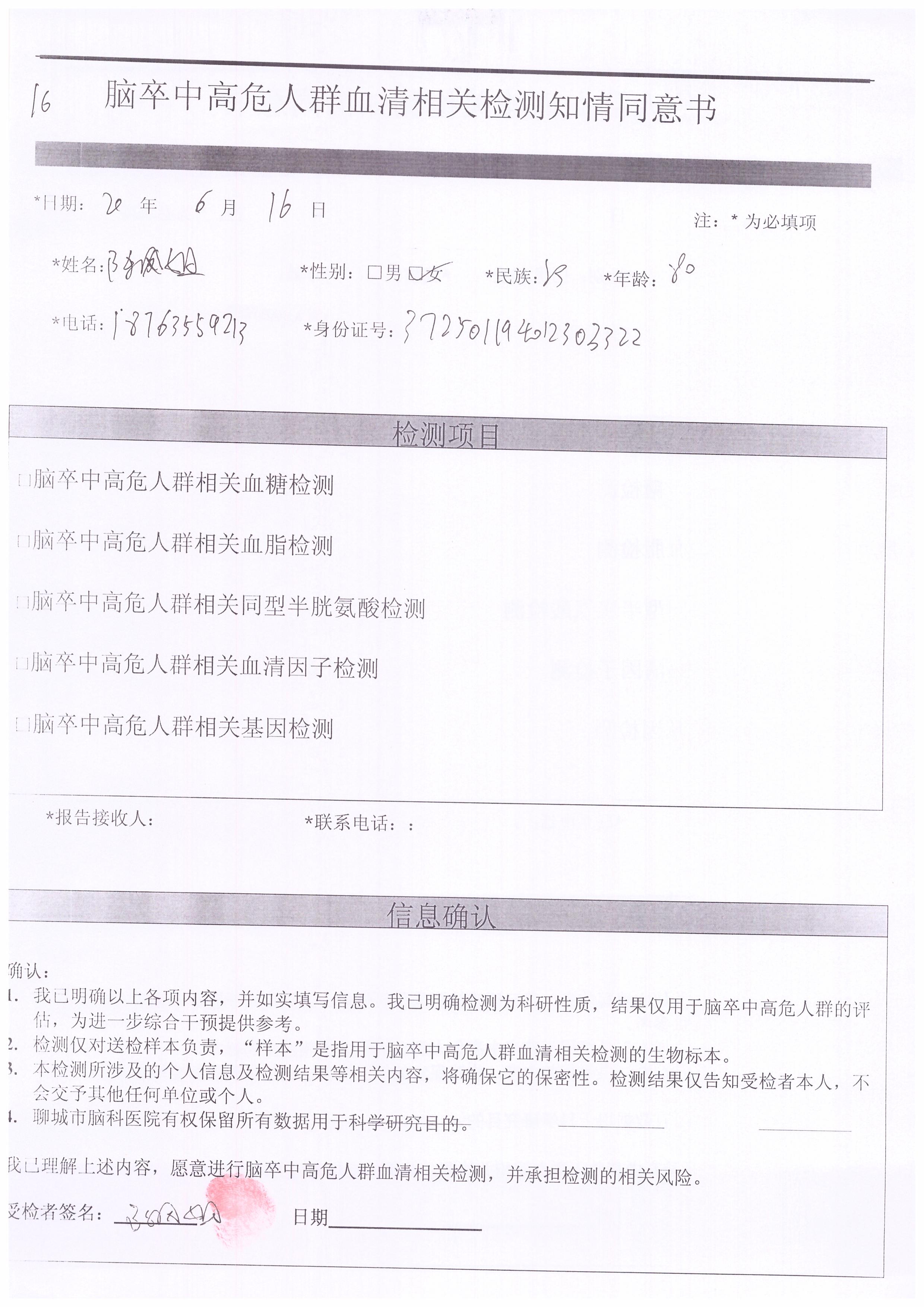

Supplement: Supplementary file 3 — Supplementary file3 (ZIP 25359 KB) [file 10528_2023_10431_MOESM3_ESM.zip › ╓¬╟Θ═1⁄4╥Γ╩Θ1/016.jpg]

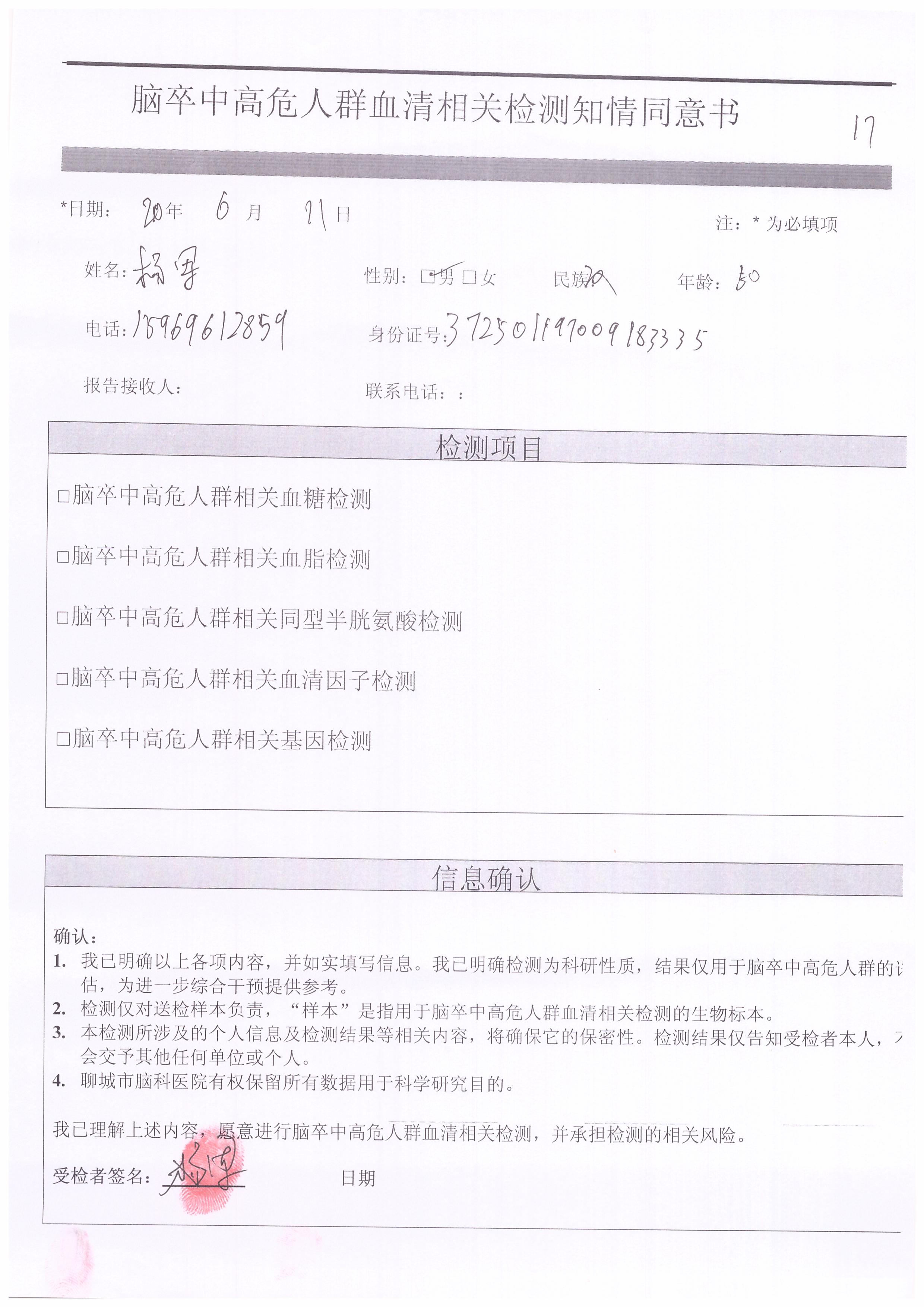

Supplement: Supplementary file 3 — Supplementary file3 (ZIP 25359 KB) [file 10528_2023_10431_MOESM3_ESM.zip › ╓¬╟Θ═1⁄4╥Γ╩Θ1/017 (2).jpg]

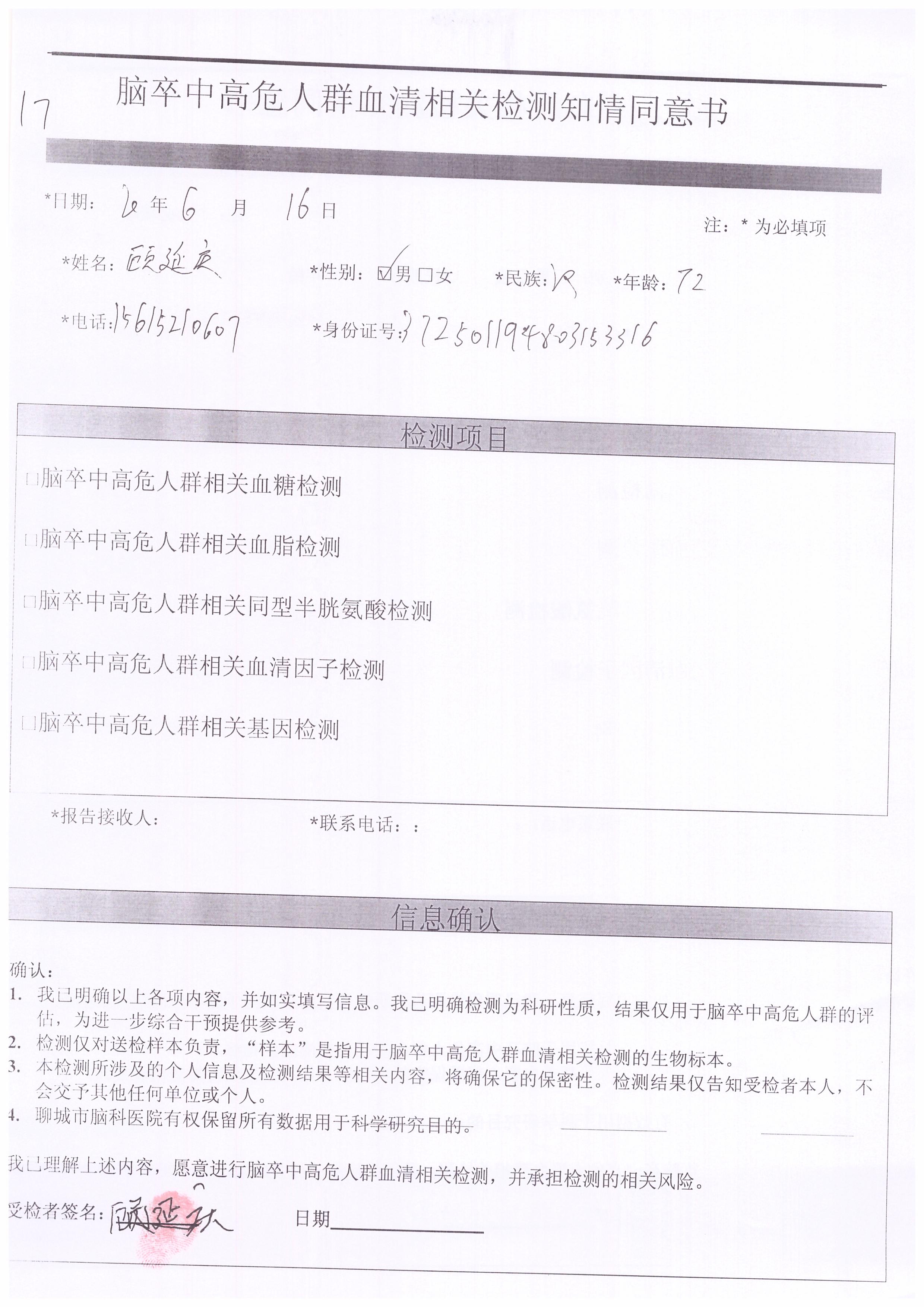

Supplement: Supplementary file 3 — Supplementary file3 (ZIP 25359 KB) [file 10528_2023_10431_MOESM3_ESM.zip › ╓¬╟Θ═1⁄4╥Γ╩Θ1/017.jpg]

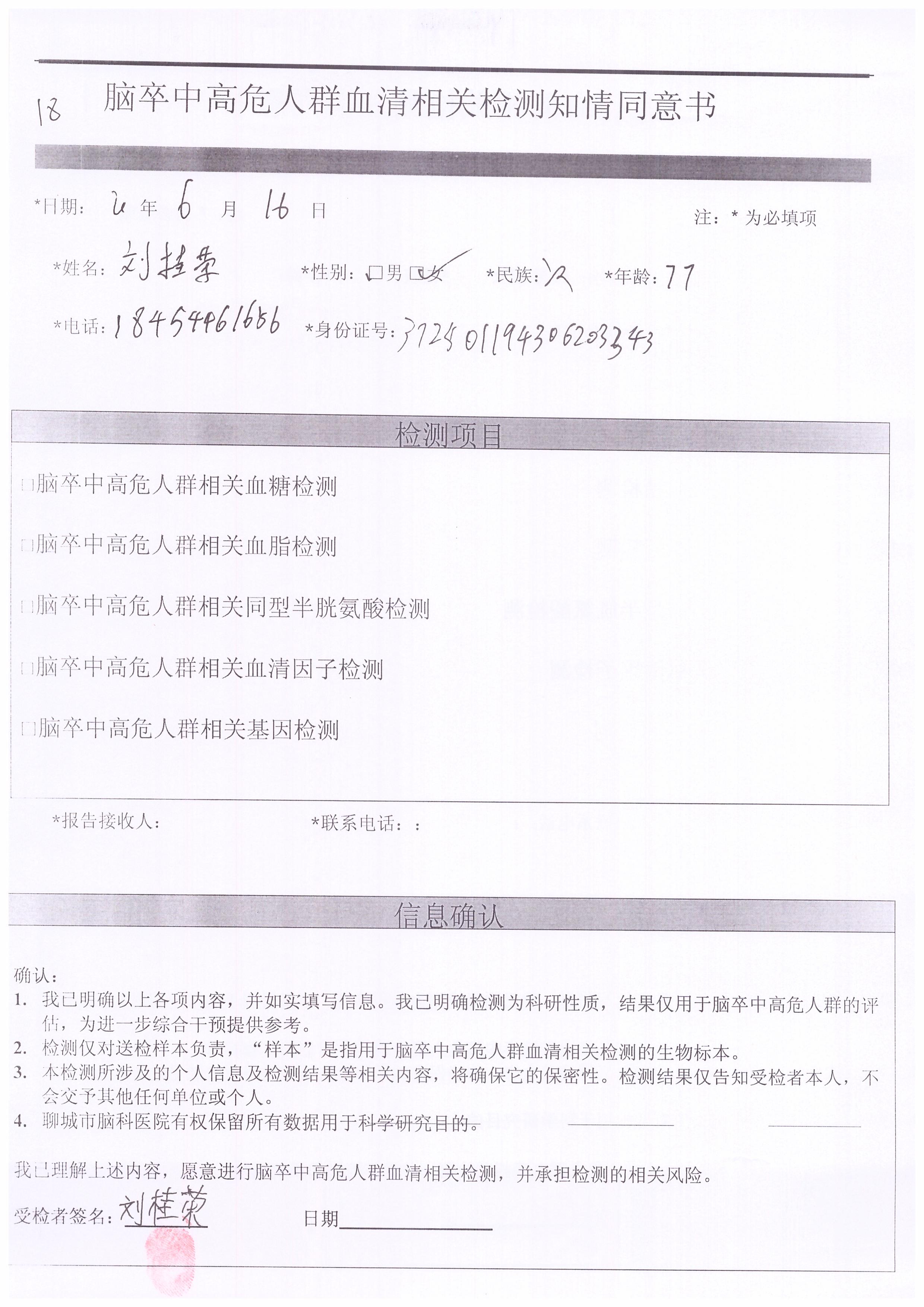

Supplement: Supplementary file 3 — Supplementary file3 (ZIP 25359 KB) [file 10528_2023_10431_MOESM3_ESM.zip › ╓¬╟Θ═1⁄4╥Γ╩Θ1/018.jpg]

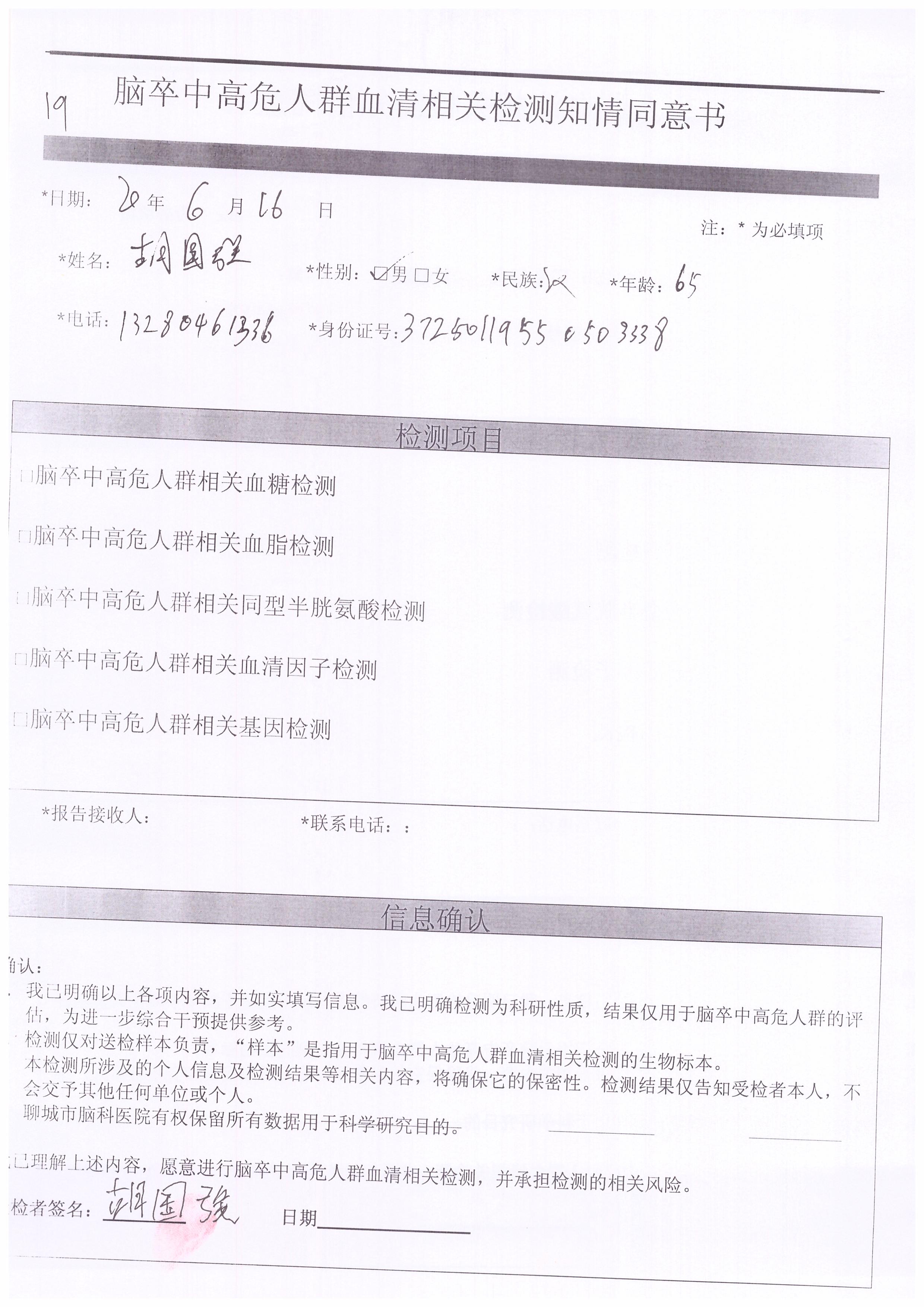

Supplement: Supplementary file 3 — Supplementary file3 (ZIP 25359 KB) [file 10528_2023_10431_MOESM3_ESM.zip › ╓¬╟Θ═1⁄4╥Γ╩Θ1/019.jpg]

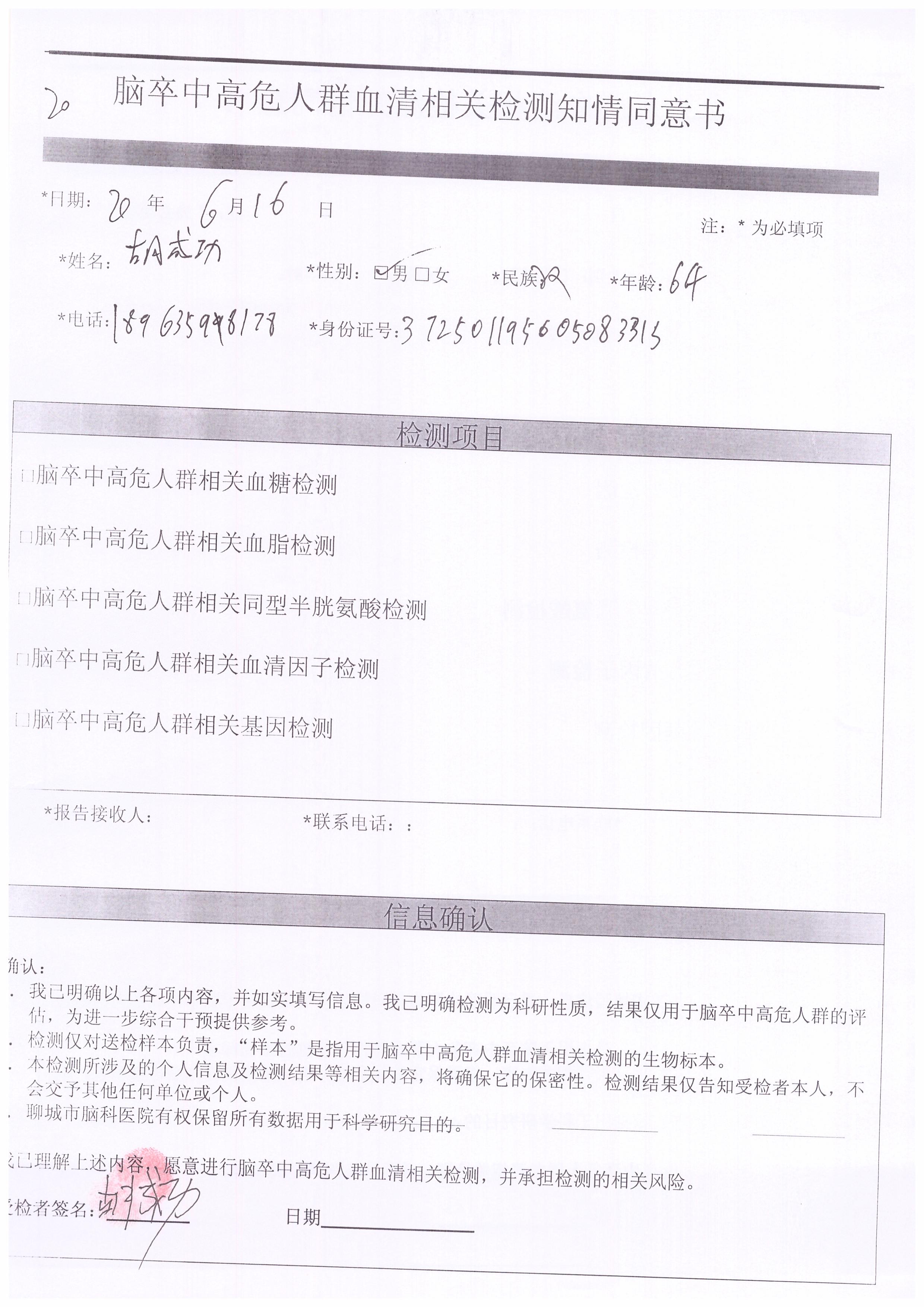

Supplement: Supplementary file 3 — Supplementary file3 (ZIP 25359 KB) [file 10528_2023_10431_MOESM3_ESM.zip › ╓¬╟Θ═1⁄4╥Γ╩Θ1/020.jpg]

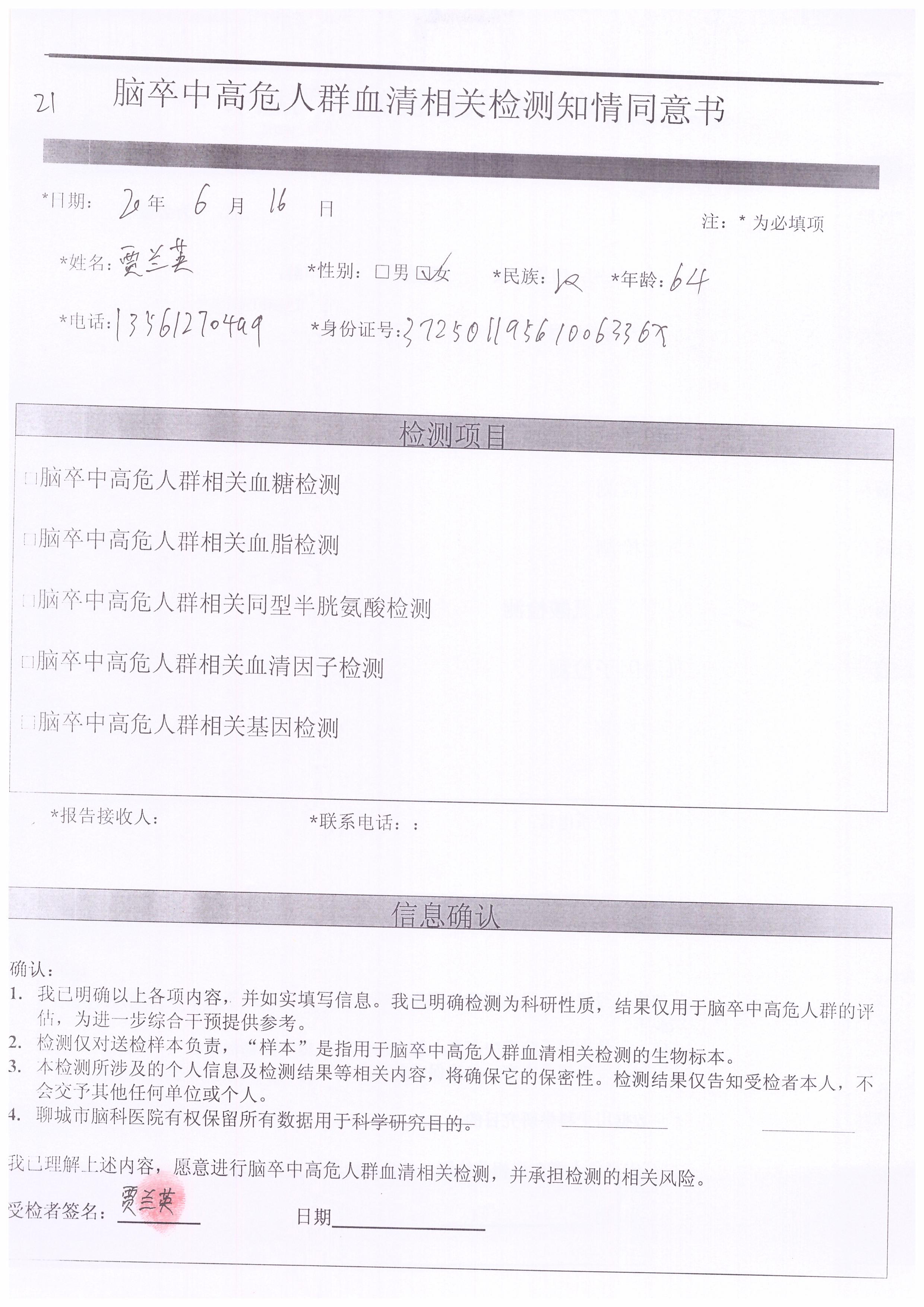

Supplement: Supplementary file 3 — Supplementary file3 (ZIP 25359 KB) [file 10528_2023_10431_MOESM3_ESM.zip › ╓¬╟Θ═1⁄4╥Γ╩Θ1/021.jpg]

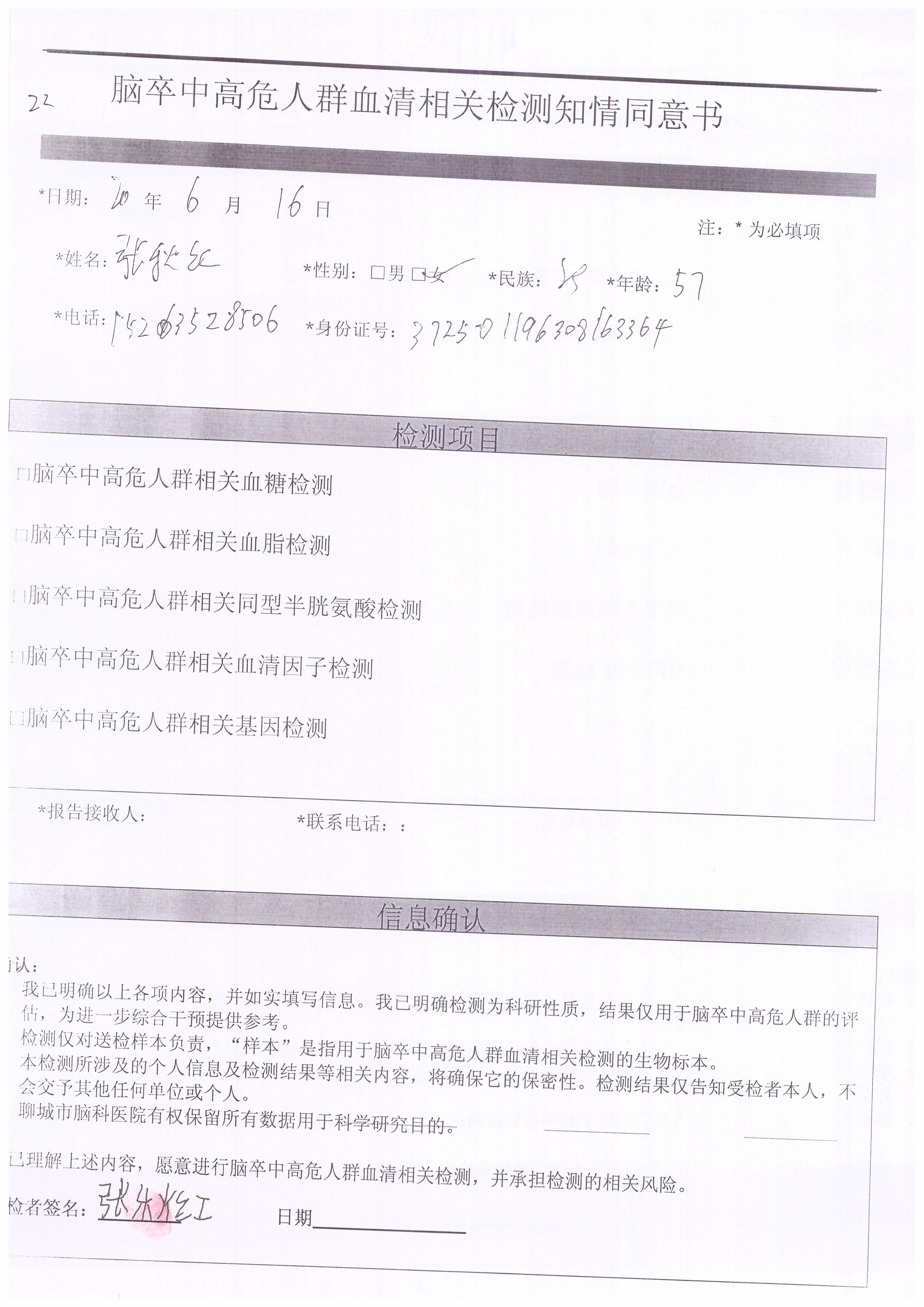

Supplement: Supplementary file 3 — Supplementary file3 (ZIP 25359 KB) [file 10528_2023_10431_MOESM3_ESM.zip › ╓¬╟Θ═1⁄4╥Γ╩Θ1/022.jpg]

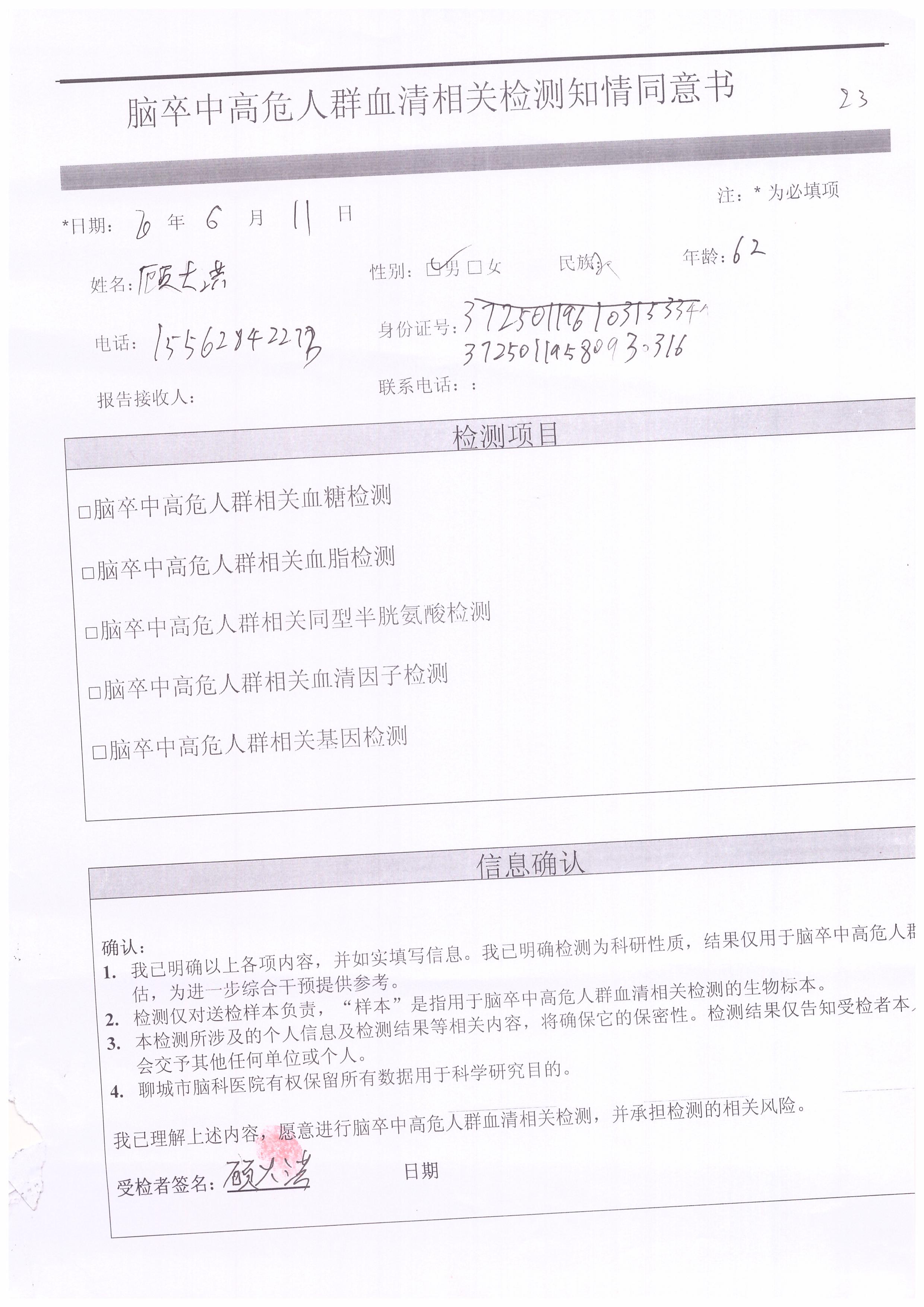

Supplement: Supplementary file 3 — Supplementary file3 (ZIP 25359 KB) [file 10528_2023_10431_MOESM3_ESM.zip › ╓¬╟Θ═1⁄4╥Γ╩Θ1/023 (2).jpg]

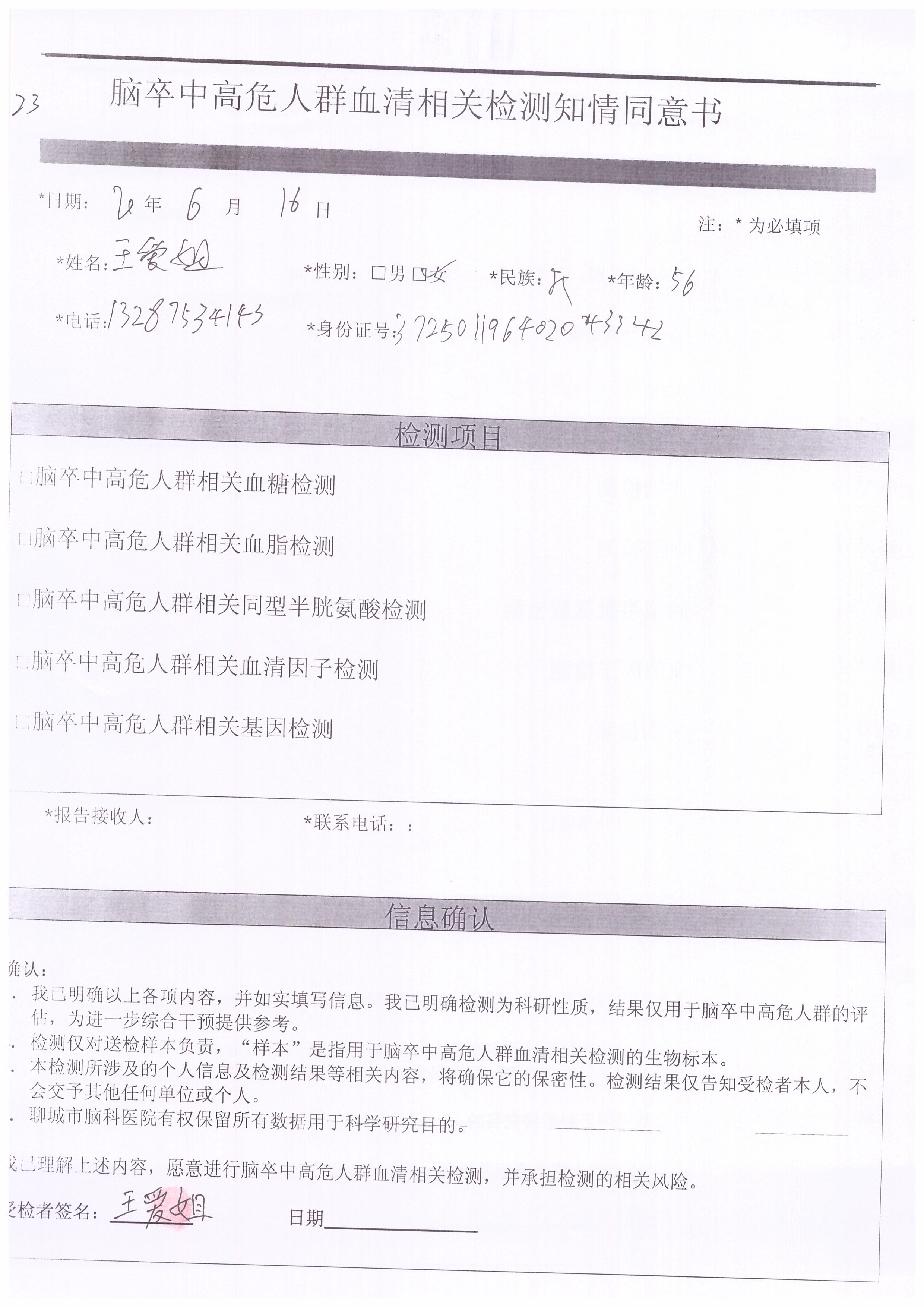

Supplement: Supplementary file 3 — Supplementary file3 (ZIP 25359 KB) [file 10528_2023_10431_MOESM3_ESM.zip › ╓¬╟Θ═1⁄4╥Γ╩Θ1/023.jpg]

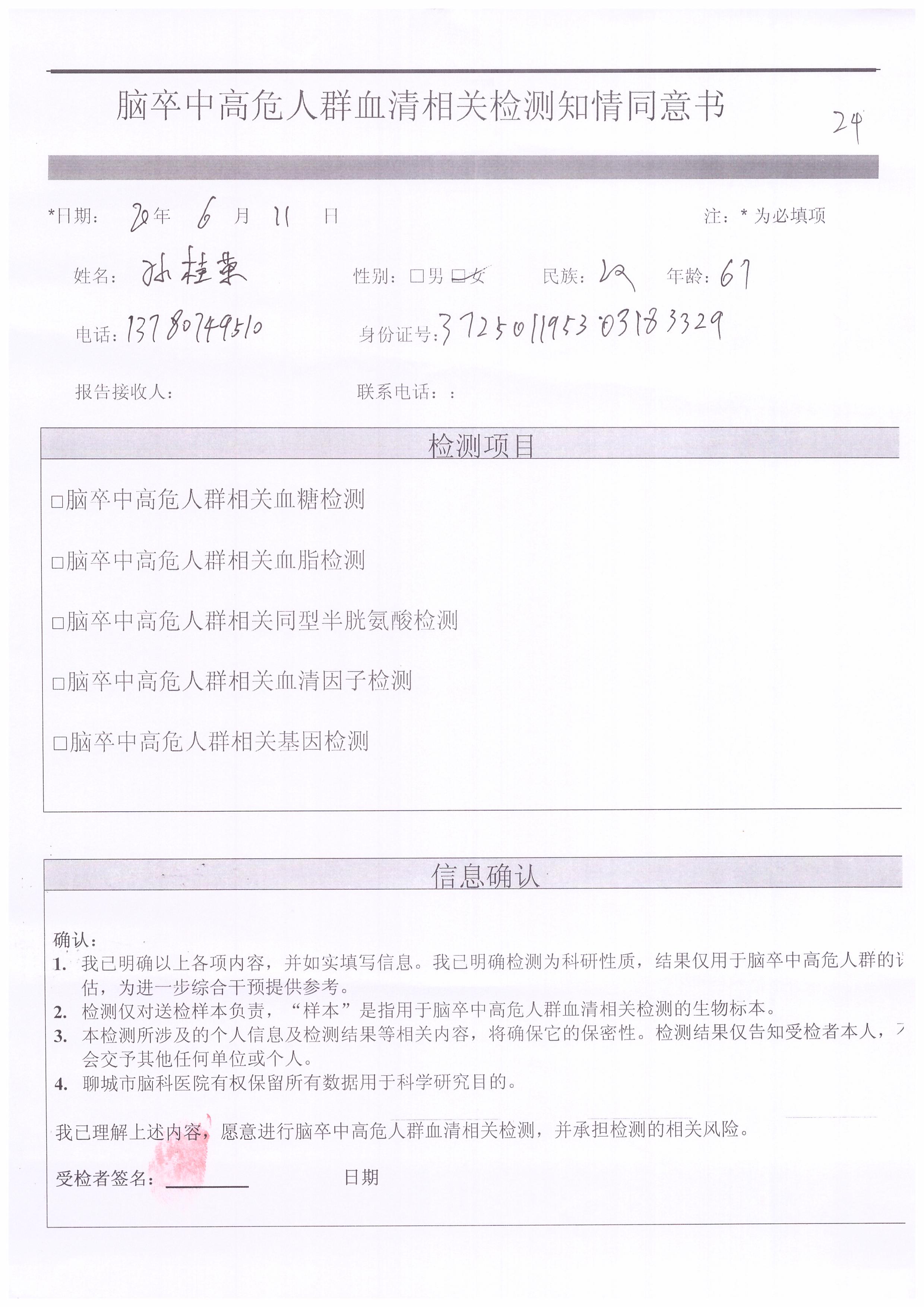

Supplement: Supplementary file 3 — Supplementary file3 (ZIP 25359 KB) [file 10528_2023_10431_MOESM3_ESM.zip › ╓¬╟Θ═1⁄4╥Γ╩Θ1/024 (2).jpg]

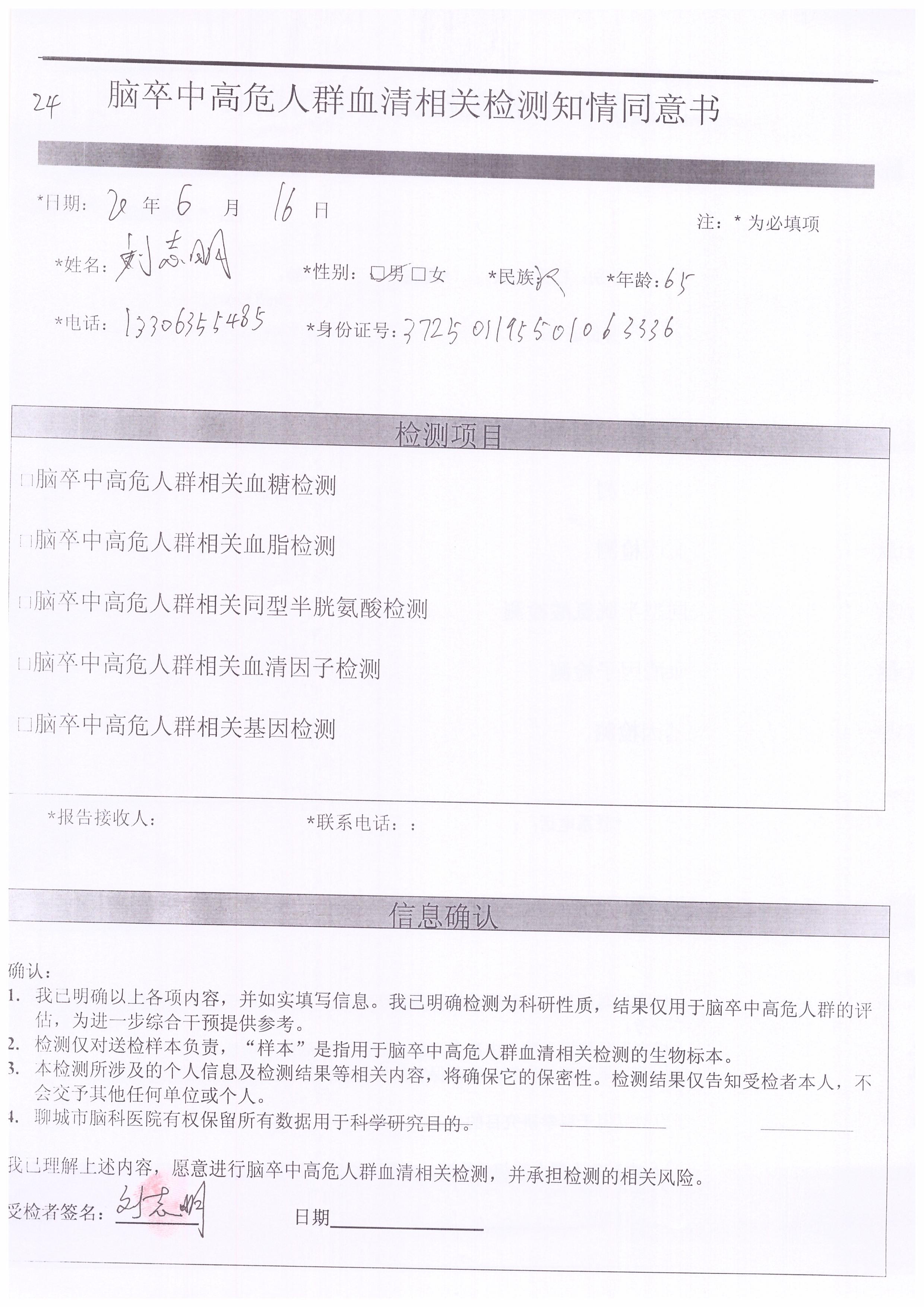

Supplement: Supplementary file 3 — Supplementary file3 (ZIP 25359 KB) [file 10528_2023_10431_MOESM3_ESM.zip › ╓¬╟Θ═1⁄4╥Γ╩Θ1/024.jpg]

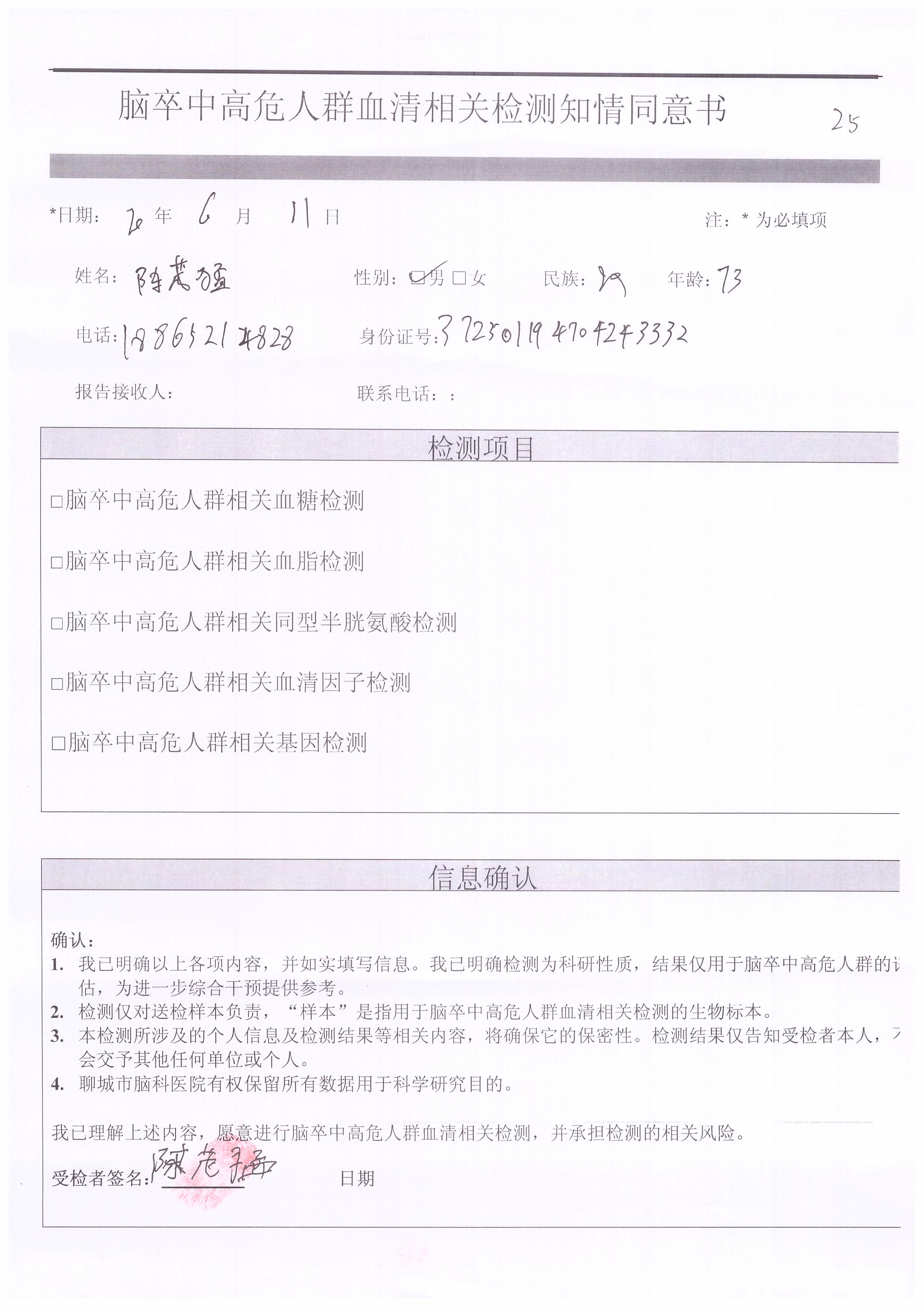

Supplement: Supplementary file 3 — Supplementary file3 (ZIP 25359 KB) [file 10528_2023_10431_MOESM3_ESM.zip › ╓¬╟Θ═1⁄4╥Γ╩Θ1/025 (2).jpg]

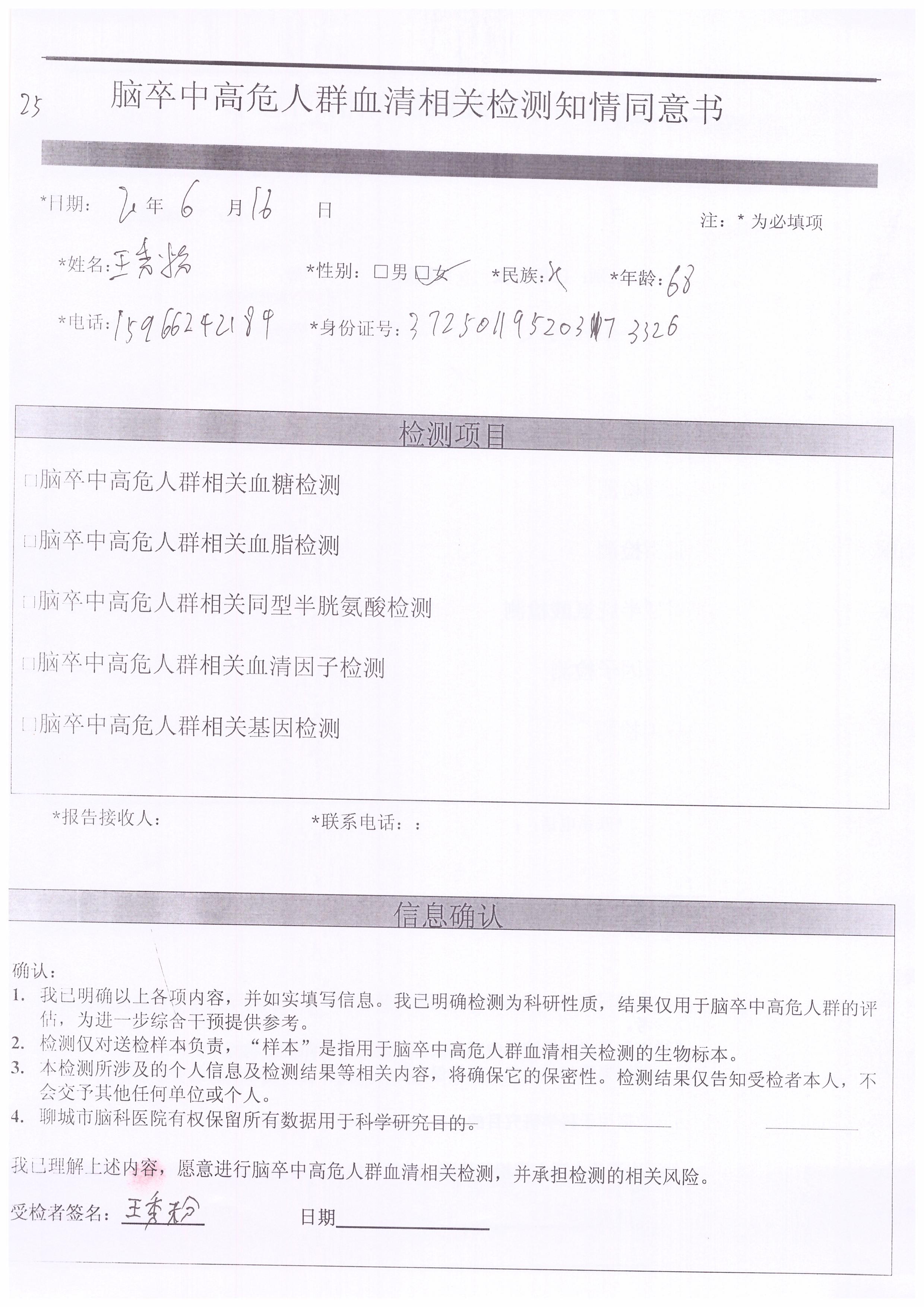

Supplement: Supplementary file 3 — Supplementary file3 (ZIP 25359 KB) [file 10528_2023_10431_MOESM3_ESM.zip › ╓¬╟Θ═1⁄4╥Γ╩Θ1/025.jpg]

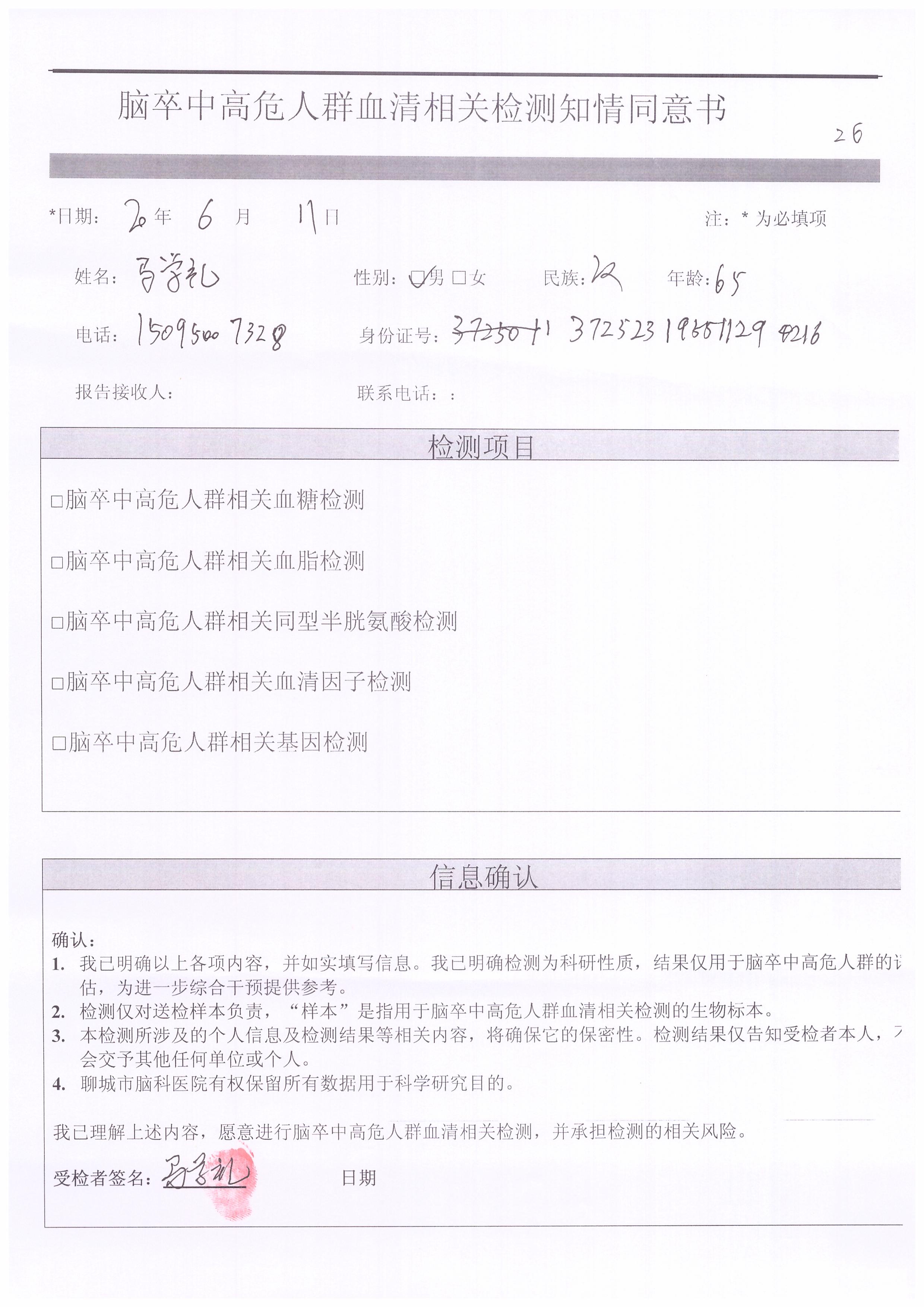

Supplement: Supplementary file 3 — Supplementary file3 (ZIP 25359 KB) [file 10528_2023_10431_MOESM3_ESM.zip › ╓¬╟Θ═1⁄4╥Γ╩Θ1/026 (2).jpg]

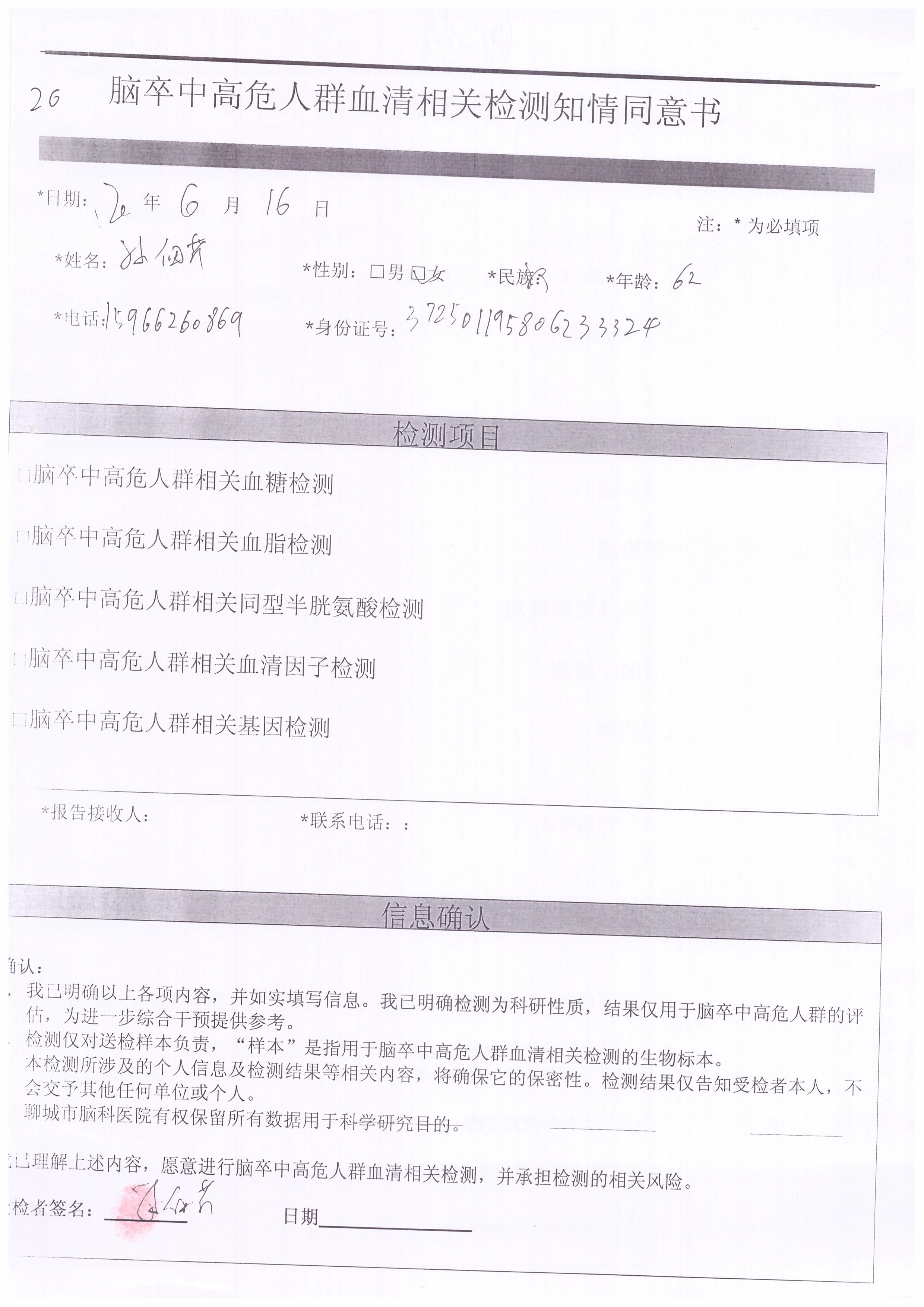

Supplement: Supplementary file 3 — Supplementary file3 (ZIP 25359 KB) [file 10528_2023_10431_MOESM3_ESM.zip › ╓¬╟Θ═1⁄4╥Γ╩Θ1/026.jpg]

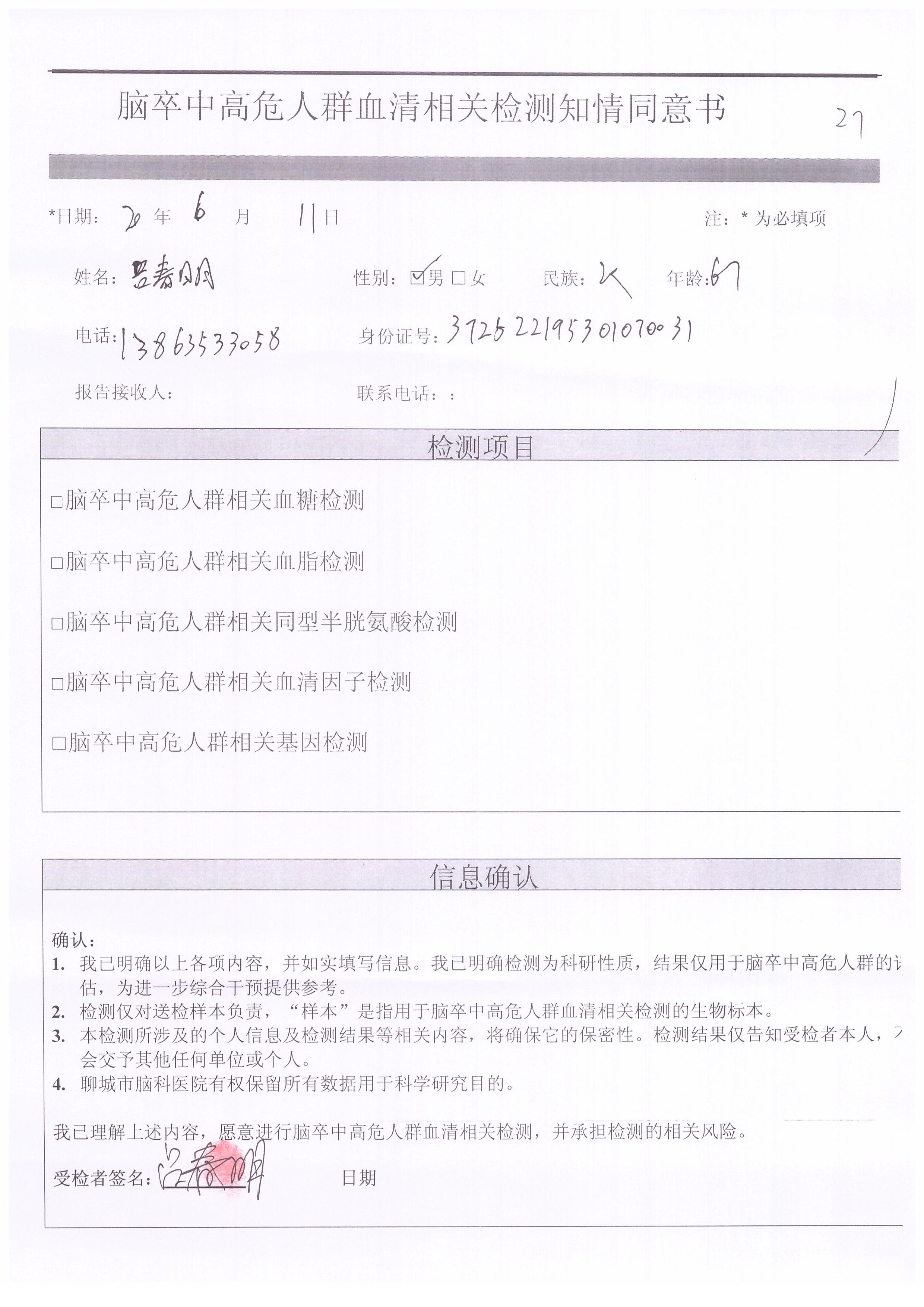

Supplement: Supplementary file 3 — Supplementary file3 (ZIP 25359 KB) [file 10528_2023_10431_MOESM3_ESM.zip › ╓¬╟Θ═1⁄4╥Γ╩Θ1/027 (2).jpg]

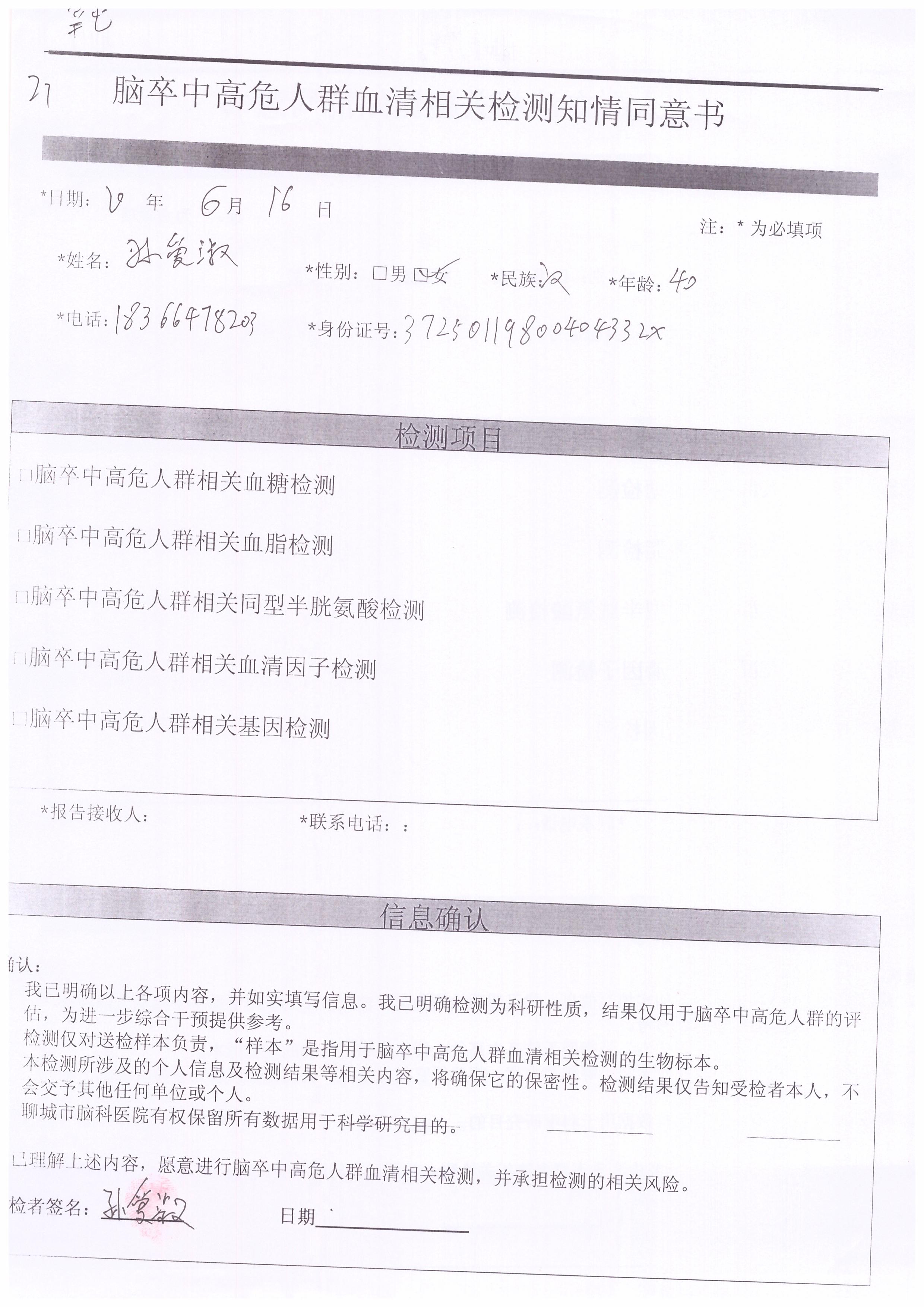

Supplement: Supplementary file 3 — Supplementary file3 (ZIP 25359 KB) [file 10528_2023_10431_MOESM3_ESM.zip › ╓¬╟Θ═1⁄4╥Γ╩Θ1/027.jpg]

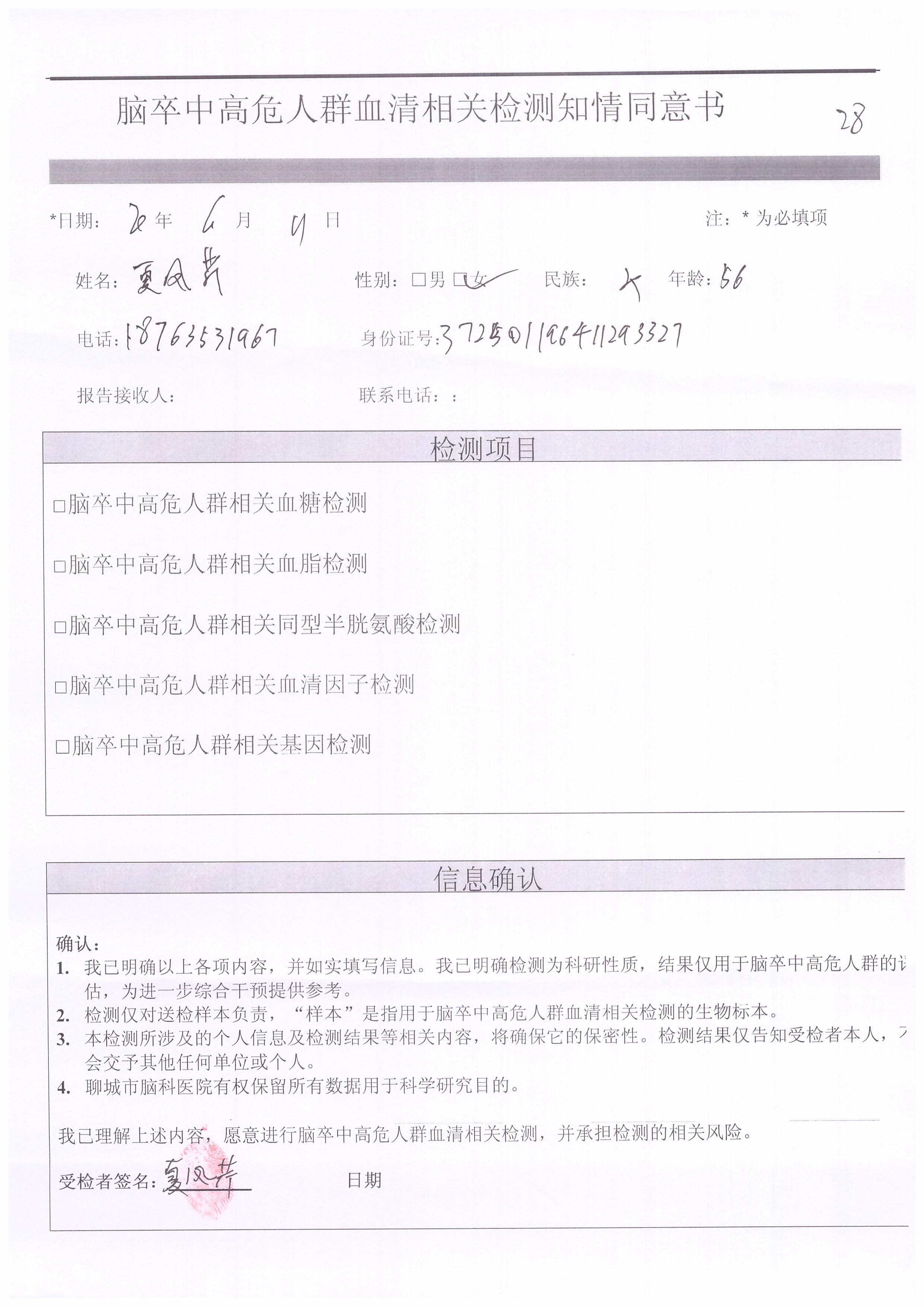

Supplement: Supplementary file 3 — Supplementary file3 (ZIP 25359 KB) [file 10528_2023_10431_MOESM3_ESM.zip › ╓¬╟Θ═1⁄4╥Γ╩Θ1/028.jpg]

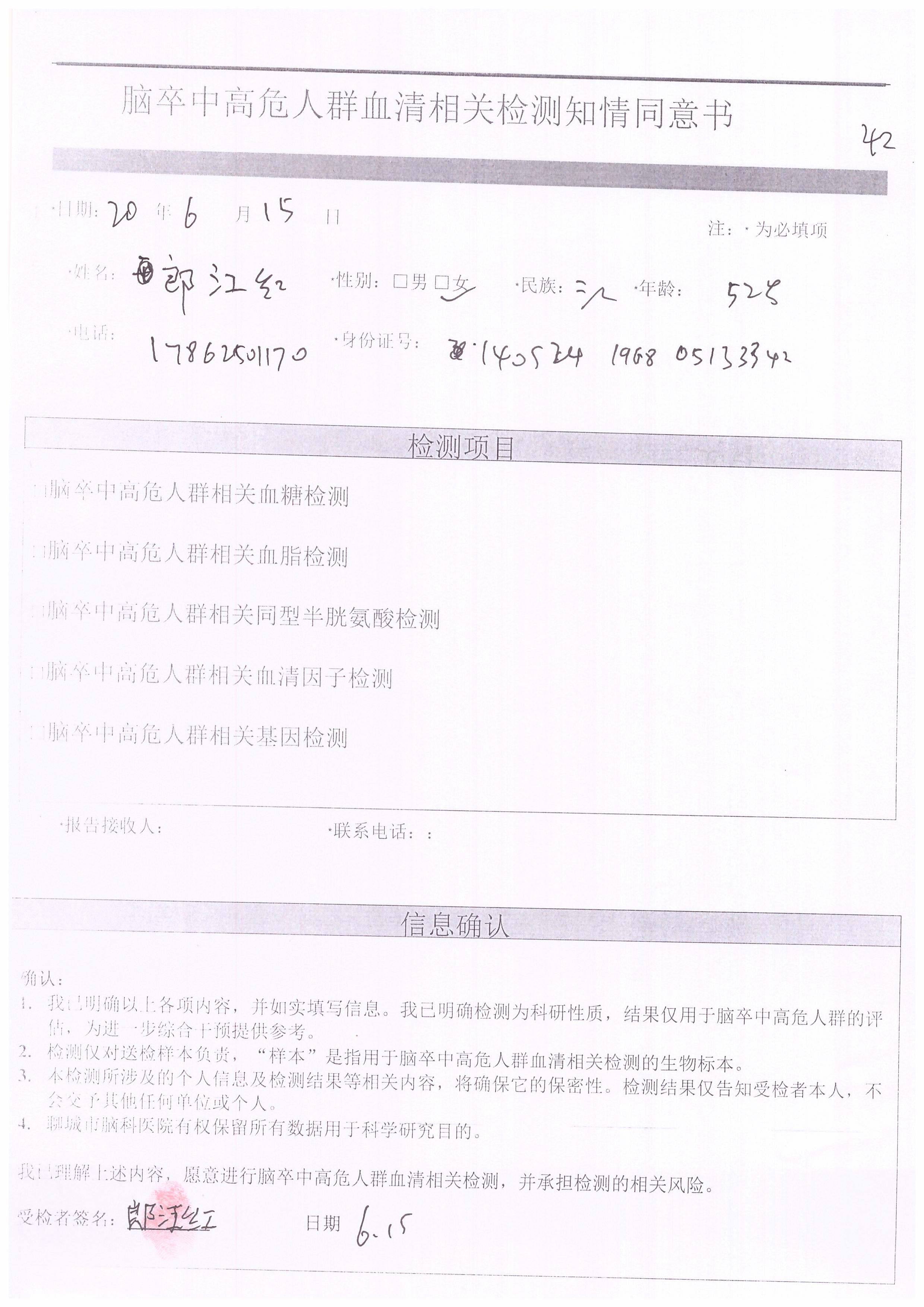

Supplement: Supplementary file 4 — Supplementary file4 (ZIP 25697 KB) [file 10528_2023_10431_MOESM4_ESM.zip › ╓¬╟Θ═1⁄4╥Γ╩Θ2/001 (2).jpg]

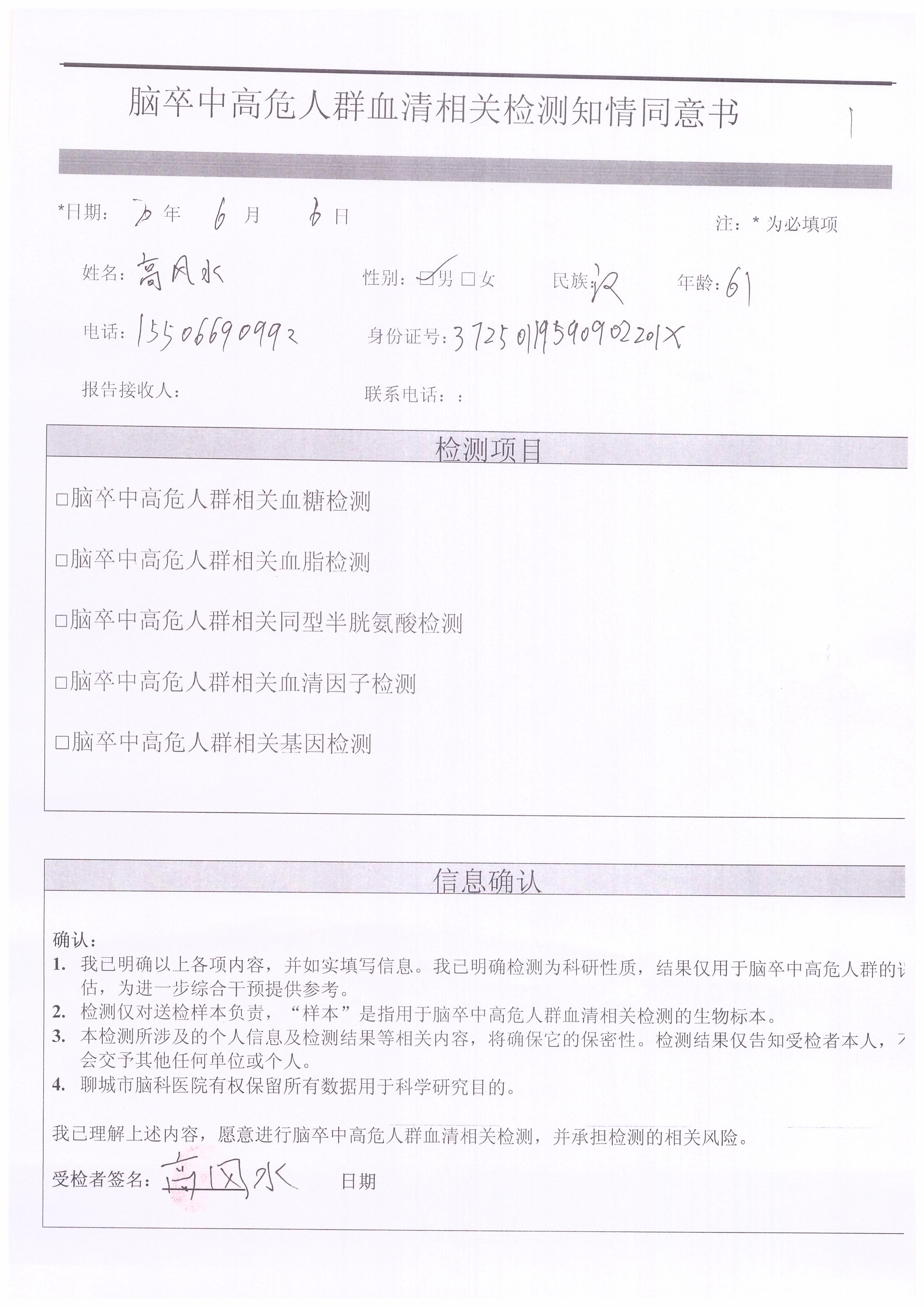

Supplement: Supplementary file 4 — Supplementary file4 (ZIP 25697 KB) [file 10528_2023_10431_MOESM4_ESM.zip › ╓¬╟Θ═1⁄4╥Γ╩Θ2/001.jpg]

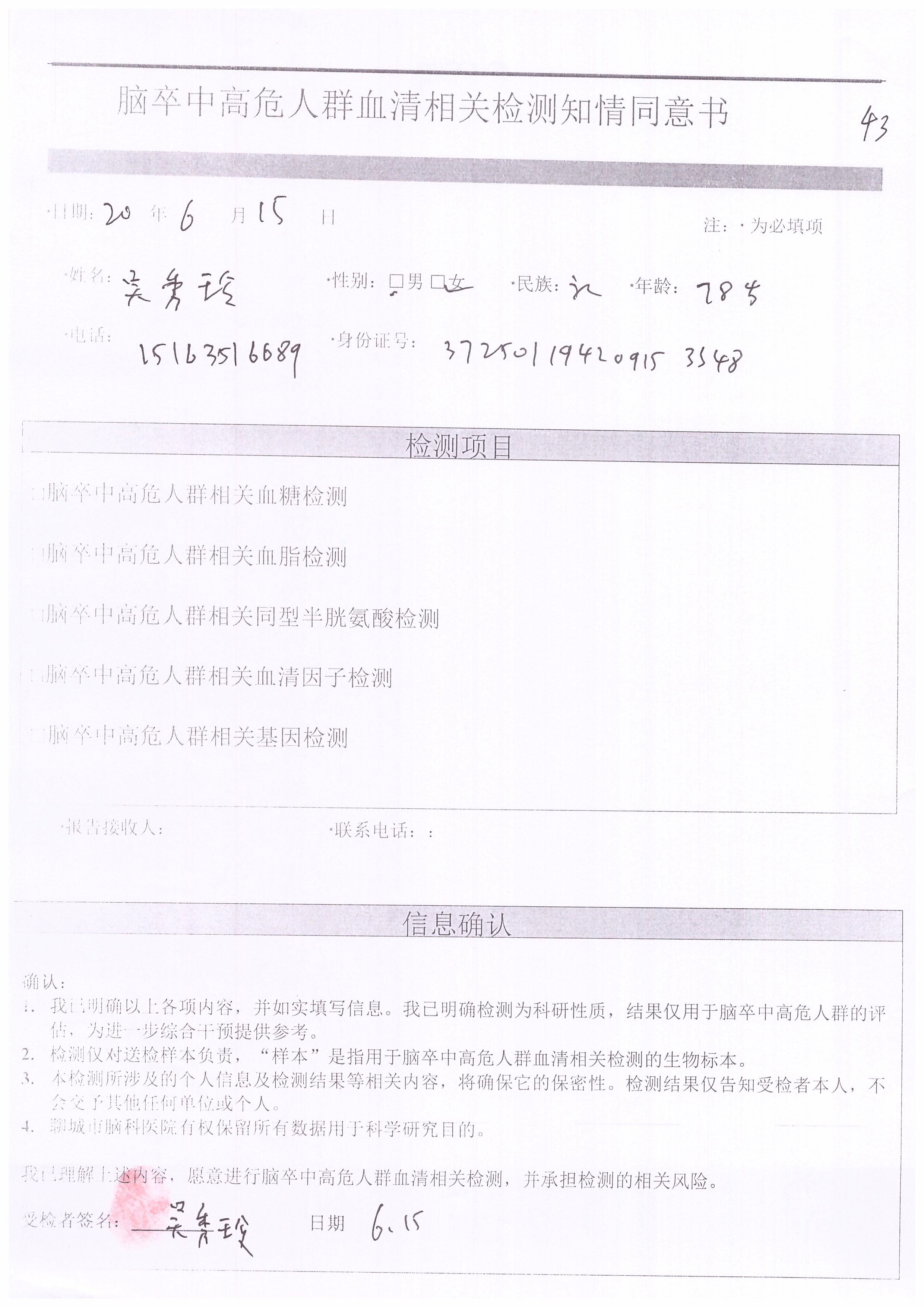

Supplement: Supplementary file 4 — Supplementary file4 (ZIP 25697 KB) [file 10528_2023_10431_MOESM4_ESM.zip › ╓¬╟Θ═1⁄4╥Γ╩Θ2/002 (2).jpg]

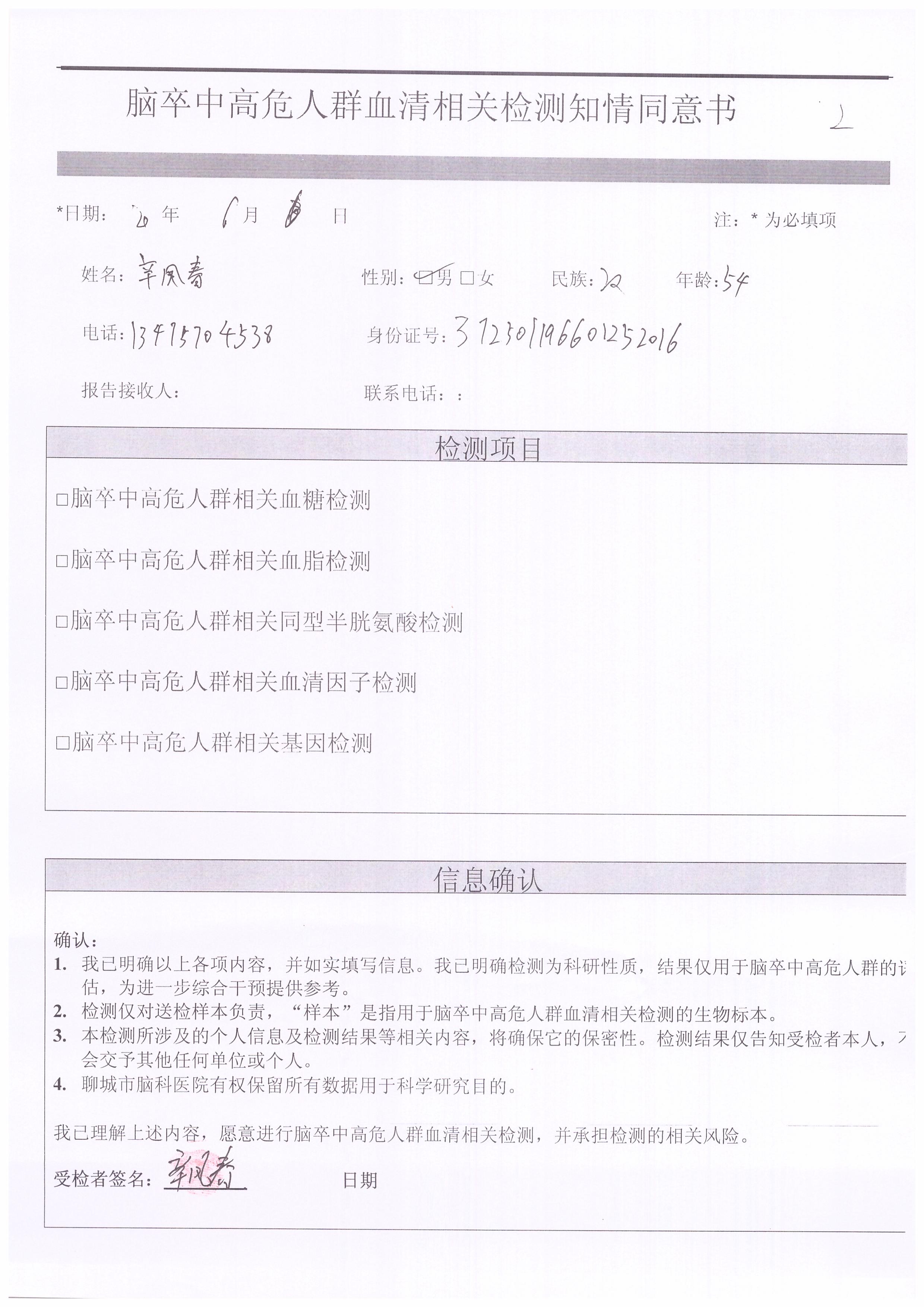

Supplement: Supplementary file 4 — Supplementary file4 (ZIP 25697 KB) [file 10528_2023_10431_MOESM4_ESM.zip › ╓¬╟Θ═1⁄4╥Γ╩Θ2/002.jpg]

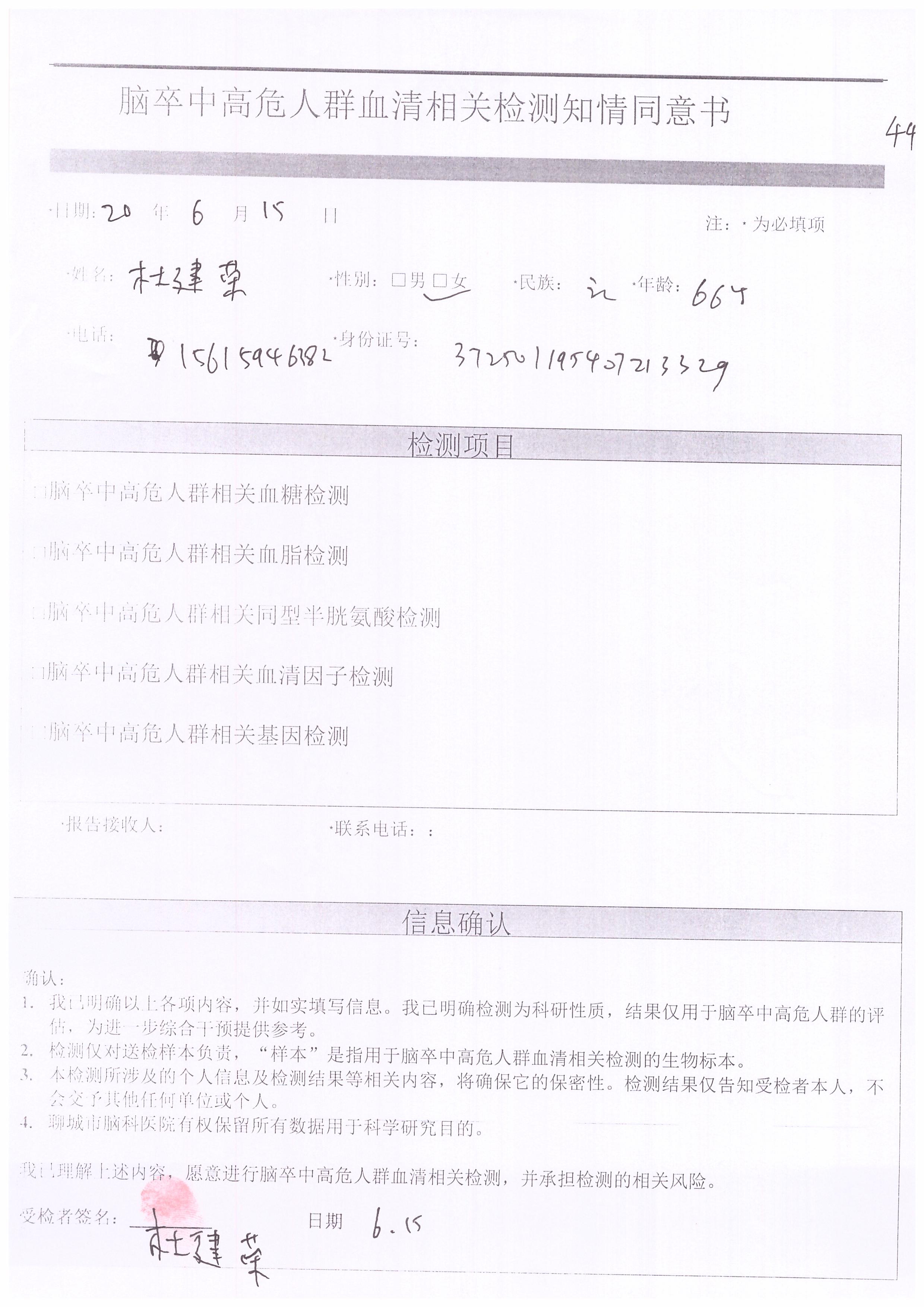

Supplement: Supplementary file 4 — Supplementary file4 (ZIP 25697 KB) [file 10528_2023_10431_MOESM4_ESM.zip › ╓¬╟Θ═1⁄4╥Γ╩Θ2/003 (2).jpg]

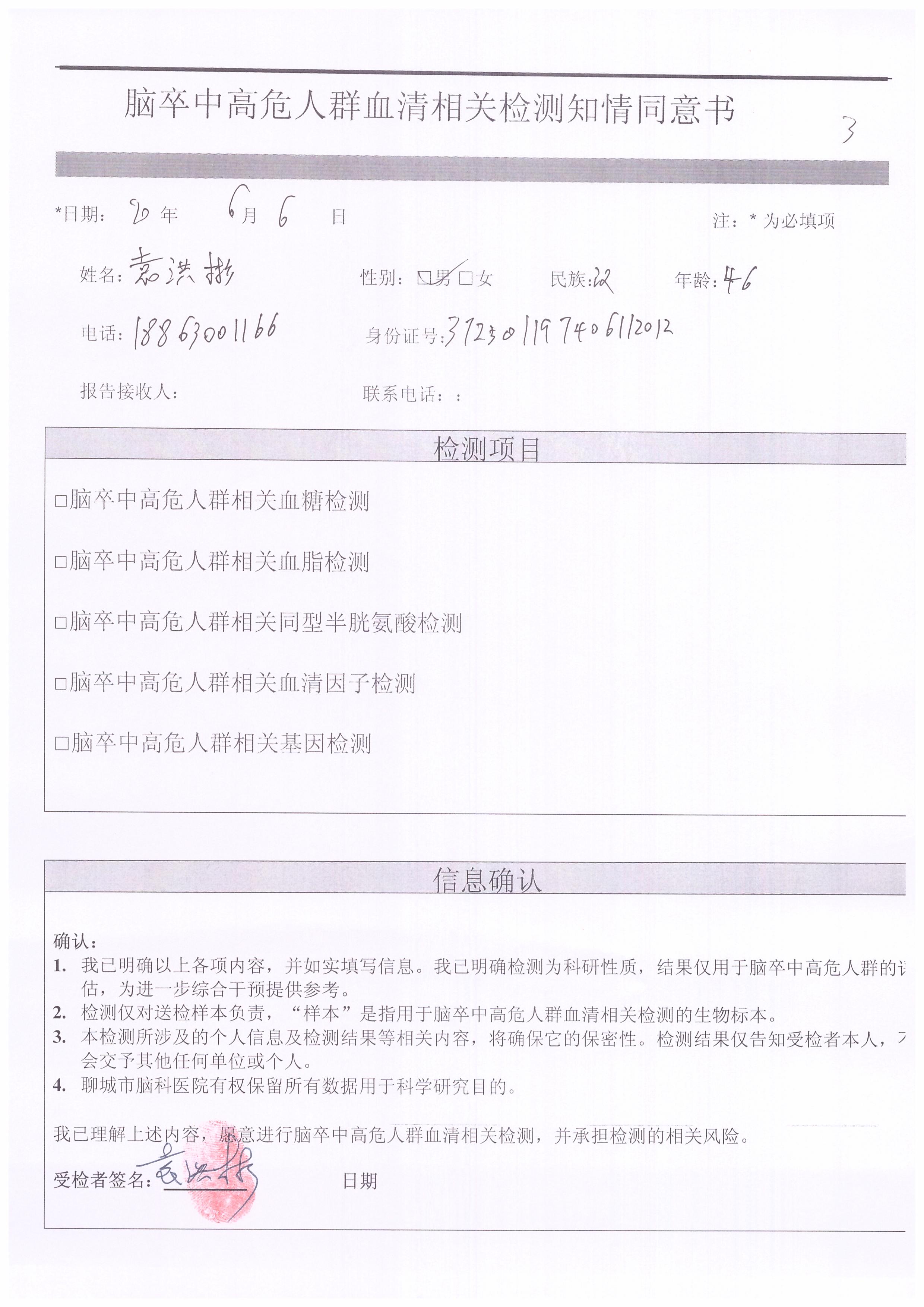

Supplement: Supplementary file 4 — Supplementary file4 (ZIP 25697 KB) [file 10528_2023_10431_MOESM4_ESM.zip › ╓¬╟Θ═1⁄4╥Γ╩Θ2/003.jpg]

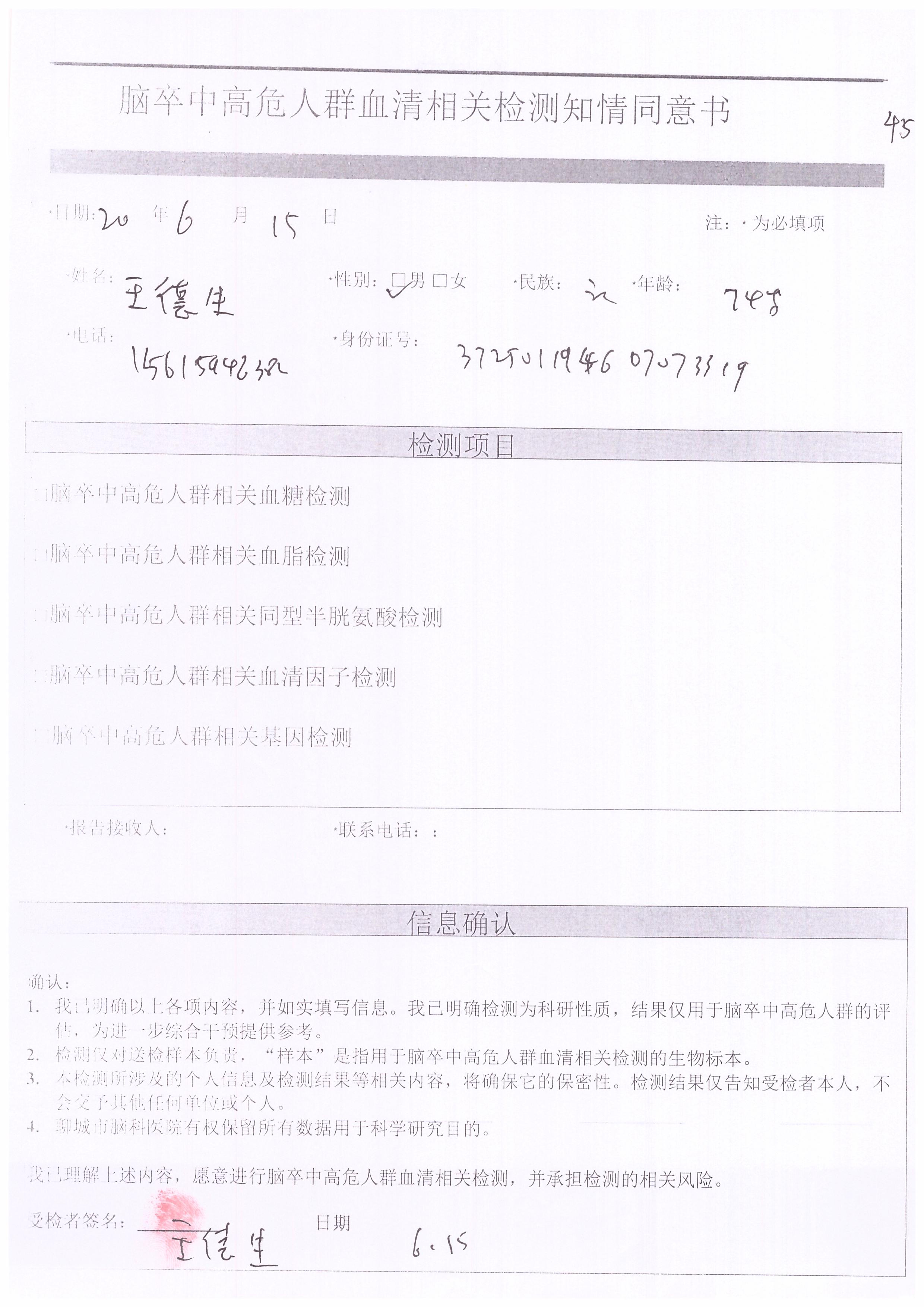

Supplement: Supplementary file 4 — Supplementary file4 (ZIP 25697 KB) [file 10528_2023_10431_MOESM4_ESM.zip › ╓¬╟Θ═1⁄4╥Γ╩Θ2/004 (2).jpg]

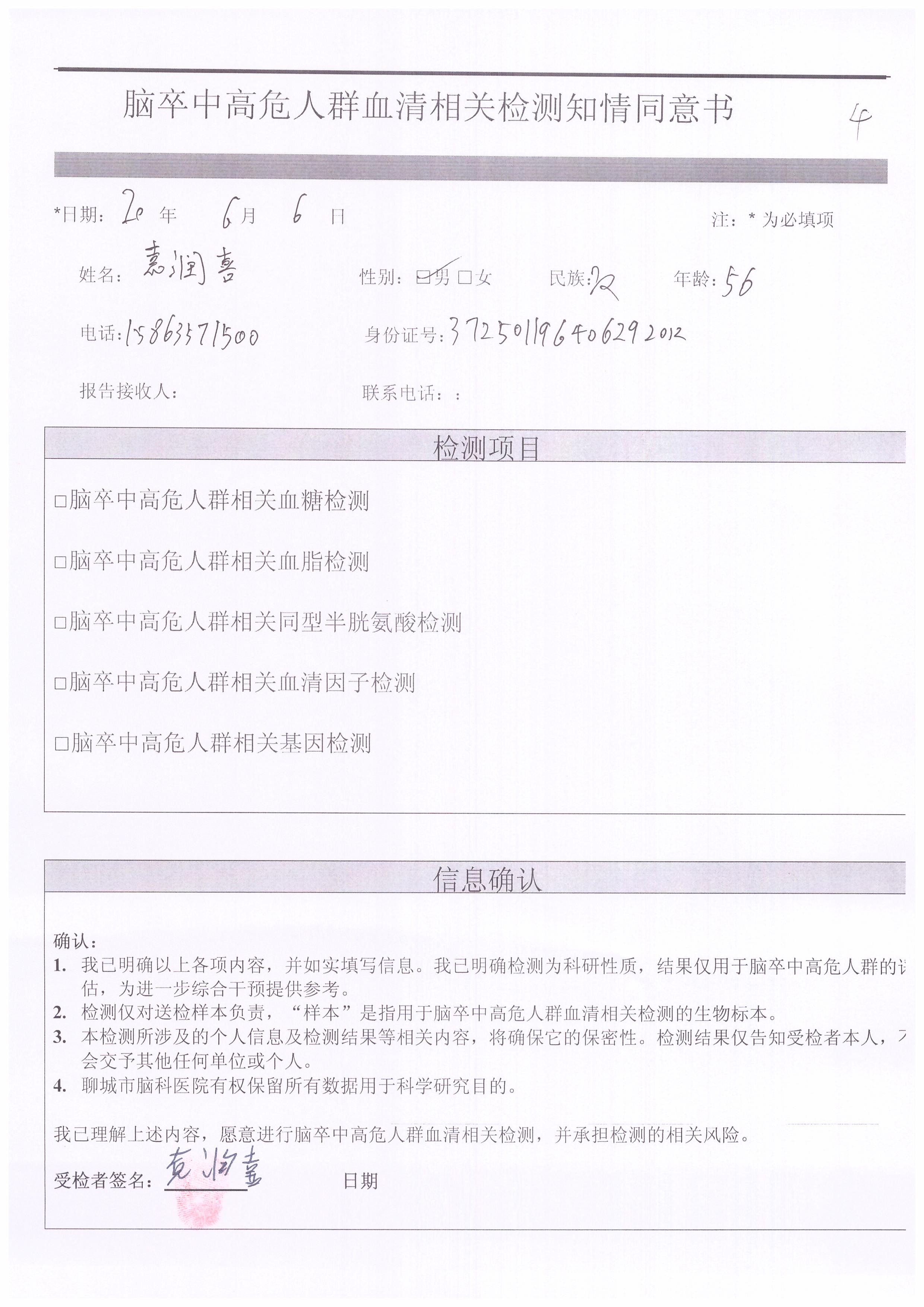

Supplement: Supplementary file 4 — Supplementary file4 (ZIP 25697 KB) [file 10528_2023_10431_MOESM4_ESM.zip › ╓¬╟Θ═1⁄4╥Γ╩Θ2/004.jpg]

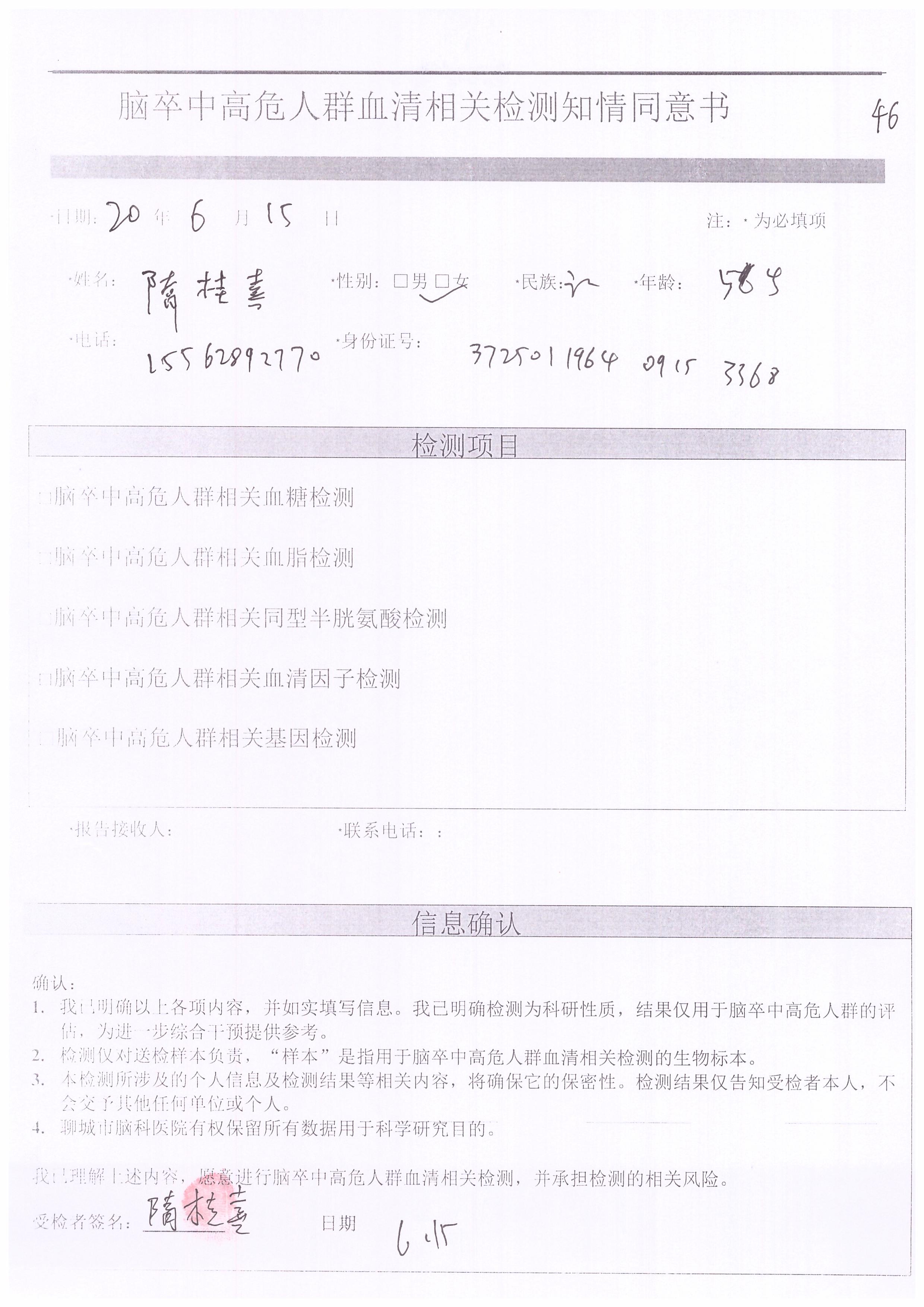

Supplement: Supplementary file 4 — Supplementary file4 (ZIP 25697 KB) [file 10528_2023_10431_MOESM4_ESM.zip › ╓¬╟Θ═1⁄4╥Γ╩Θ2/005 (2).jpg]

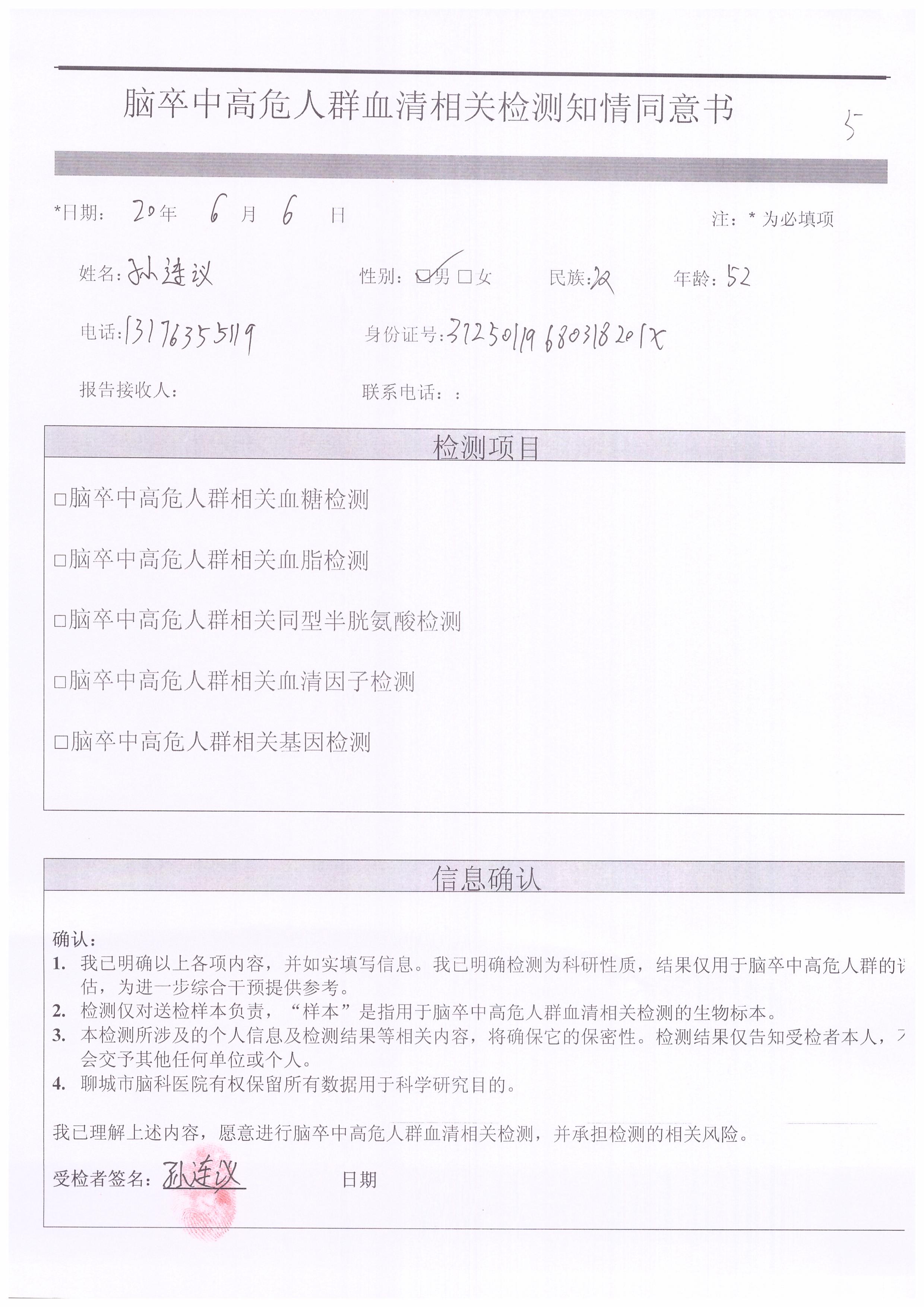

Supplement: Supplementary file 4 — Supplementary file4 (ZIP 25697 KB) [file 10528_2023_10431_MOESM4_ESM.zip › ╓¬╟Θ═1⁄4╥Γ╩Θ2/005.jpg]

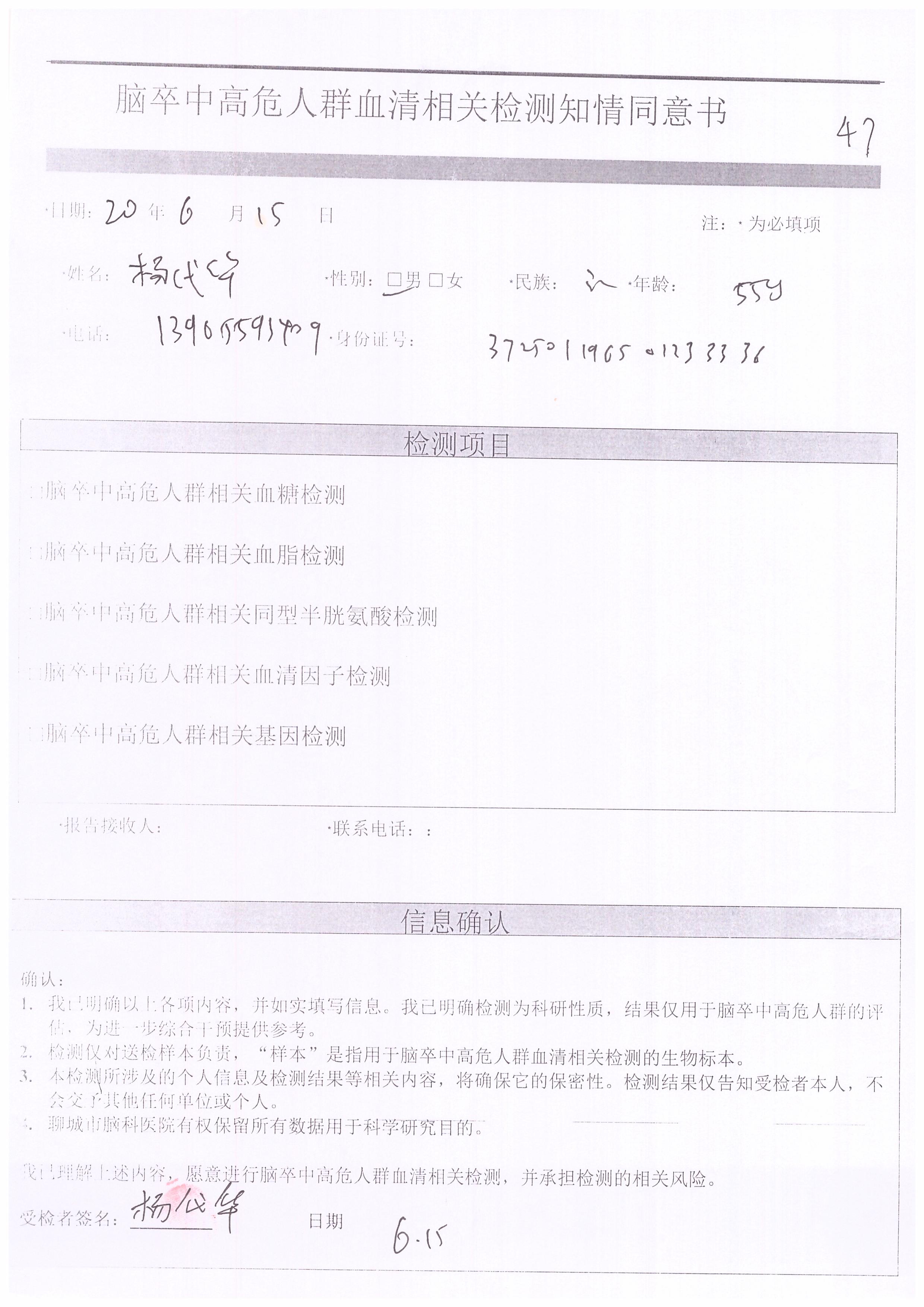

Supplement: Supplementary file 4 — Supplementary file4 (ZIP 25697 KB) [file 10528_2023_10431_MOESM4_ESM.zip › ╓¬╟Θ═1⁄4╥Γ╩Θ2/006 (2).jpg]

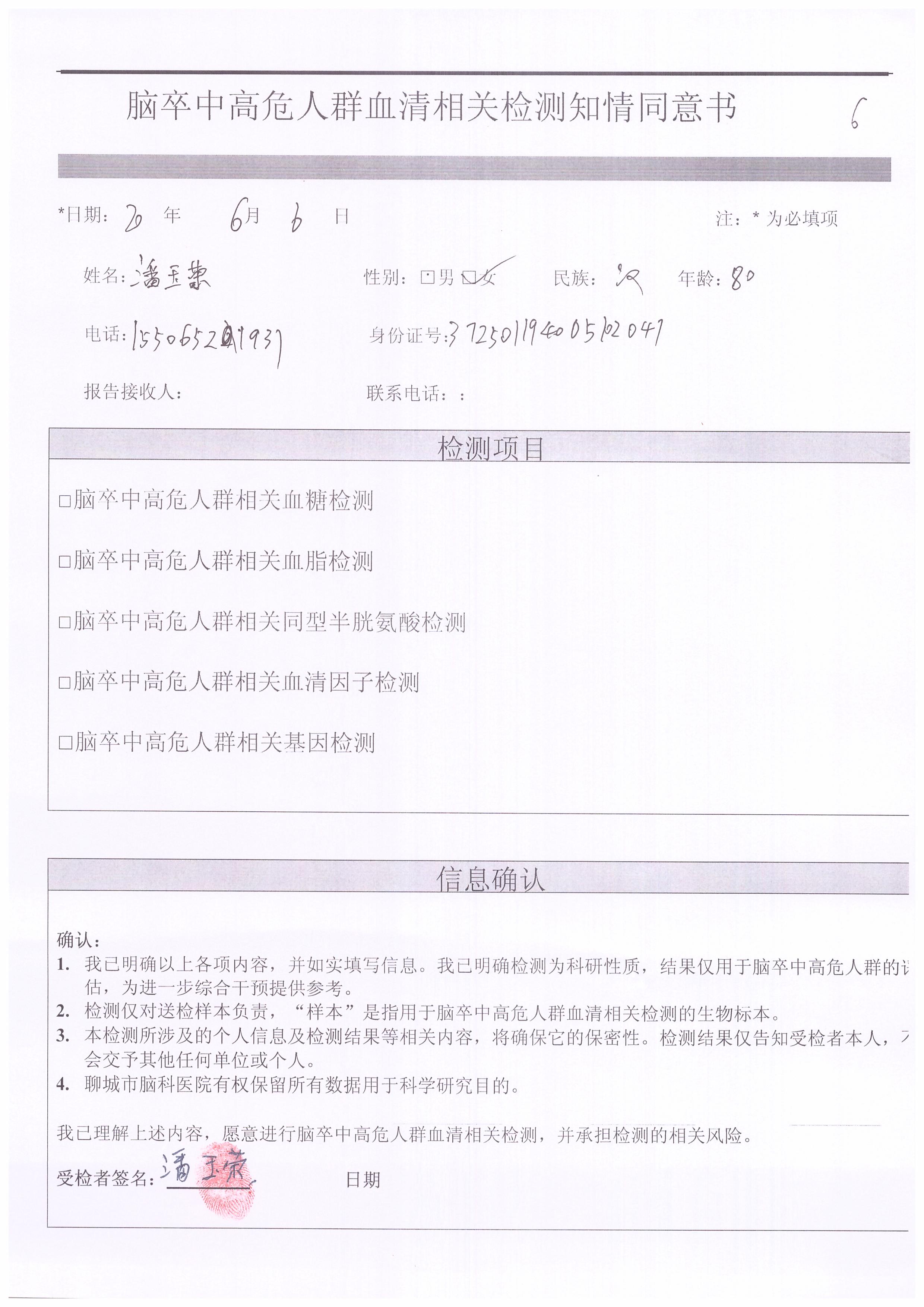

Supplement: Supplementary file 4 — Supplementary file4 (ZIP 25697 KB) [file 10528_2023_10431_MOESM4_ESM.zip › ╓¬╟Θ═1⁄4╥Γ╩Θ2/006.jpg]

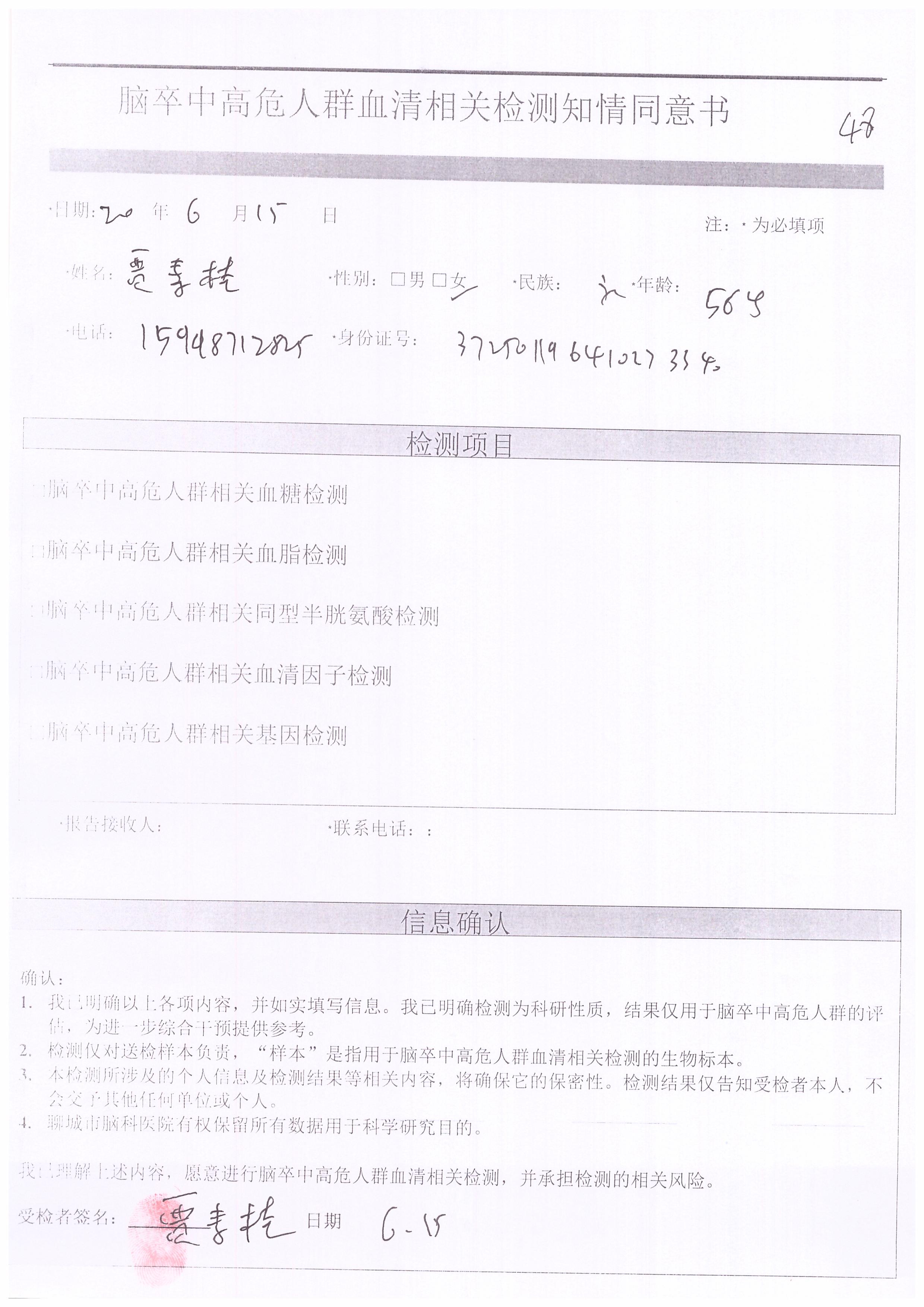

Supplement: Supplementary file 4 — Supplementary file4 (ZIP 25697 KB) [file 10528_2023_10431_MOESM4_ESM.zip › ╓¬╟Θ═1⁄4╥Γ╩Θ2/007 (2).jpg]

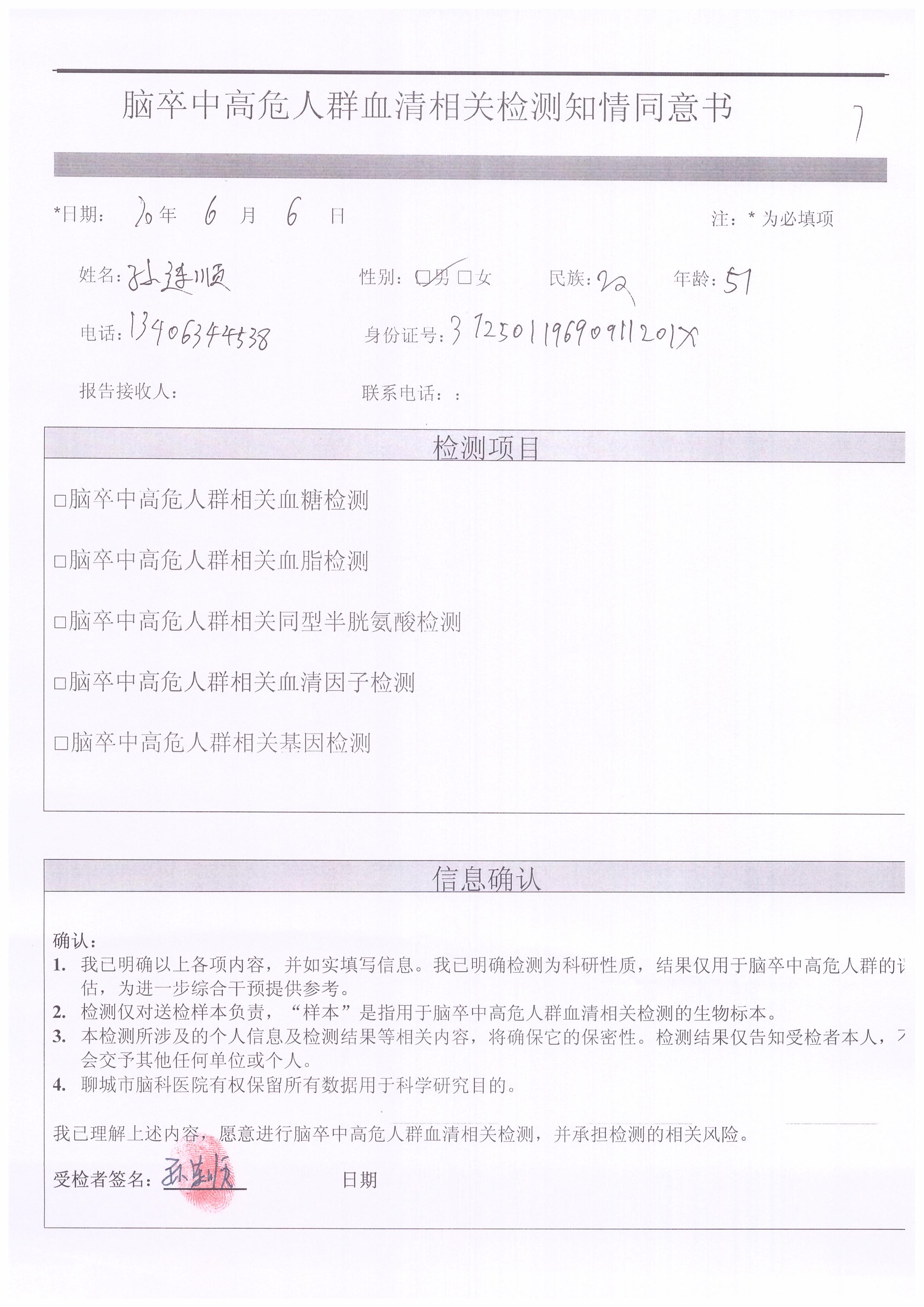

Supplement: Supplementary file 4 — Supplementary file4 (ZIP 25697 KB) [file 10528_2023_10431_MOESM4_ESM.zip › ╓¬╟Θ═1⁄4╥Γ╩Θ2/007.jpg]

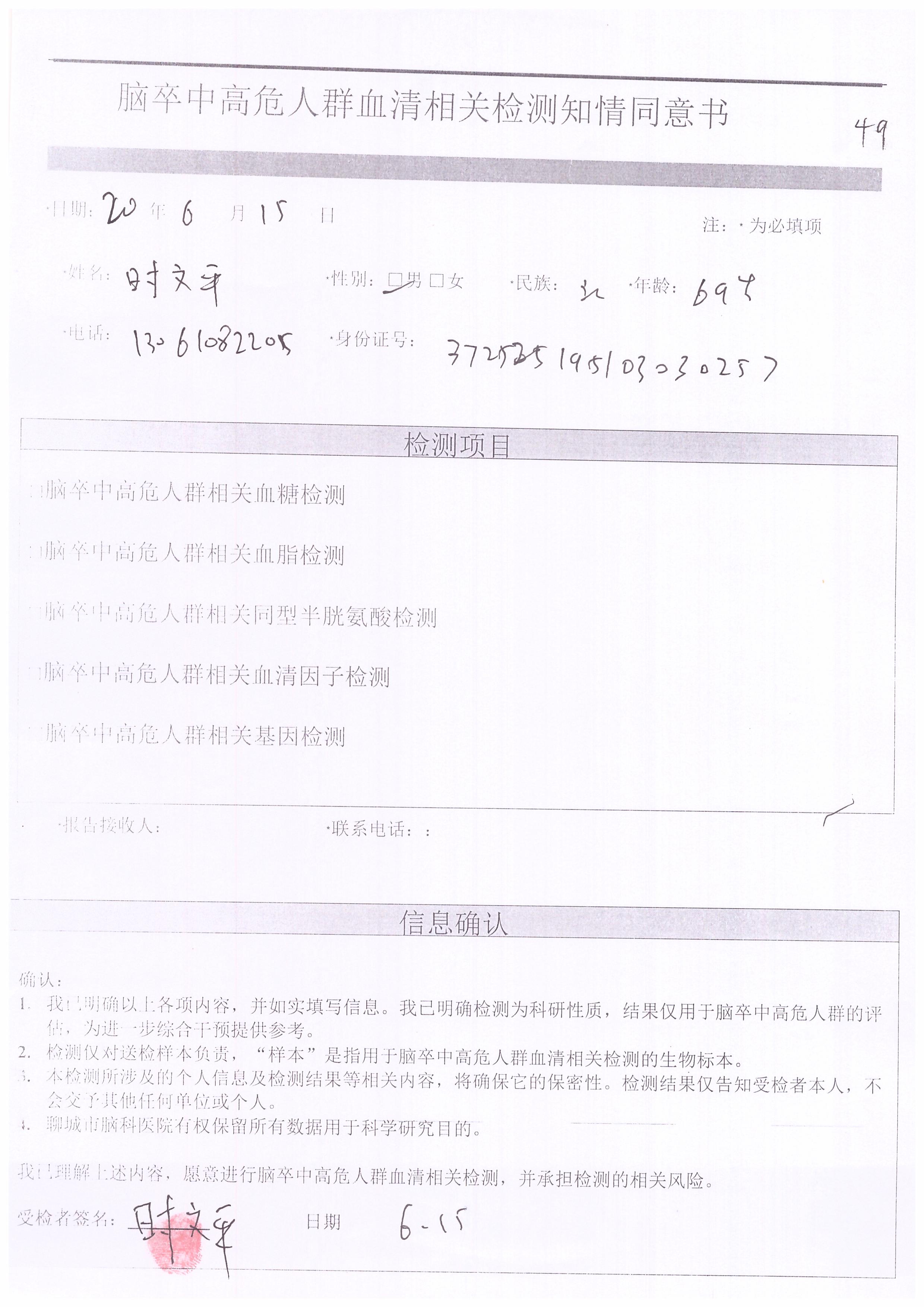

Supplement: Supplementary file 4 — Supplementary file4 (ZIP 25697 KB) [file 10528_2023_10431_MOESM4_ESM.zip › ╓¬╟Θ═1⁄4╥Γ╩Θ2/008 (2).jpg]

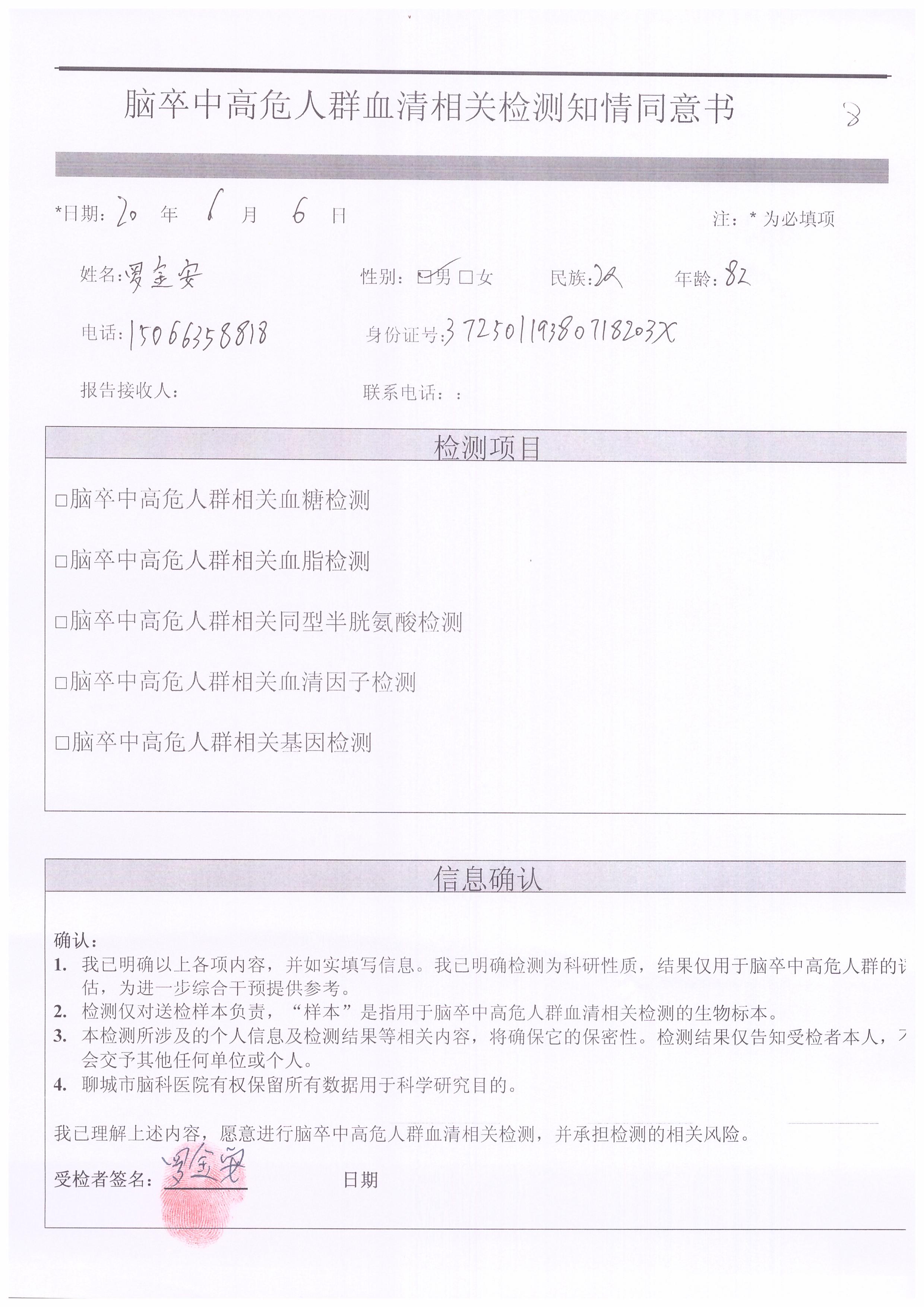

Supplement: Supplementary file 4 — Supplementary file4 (ZIP 25697 KB) [file 10528_2023_10431_MOESM4_ESM.zip › ╓¬╟Θ═1⁄4╥Γ╩Θ2/008.jpg]

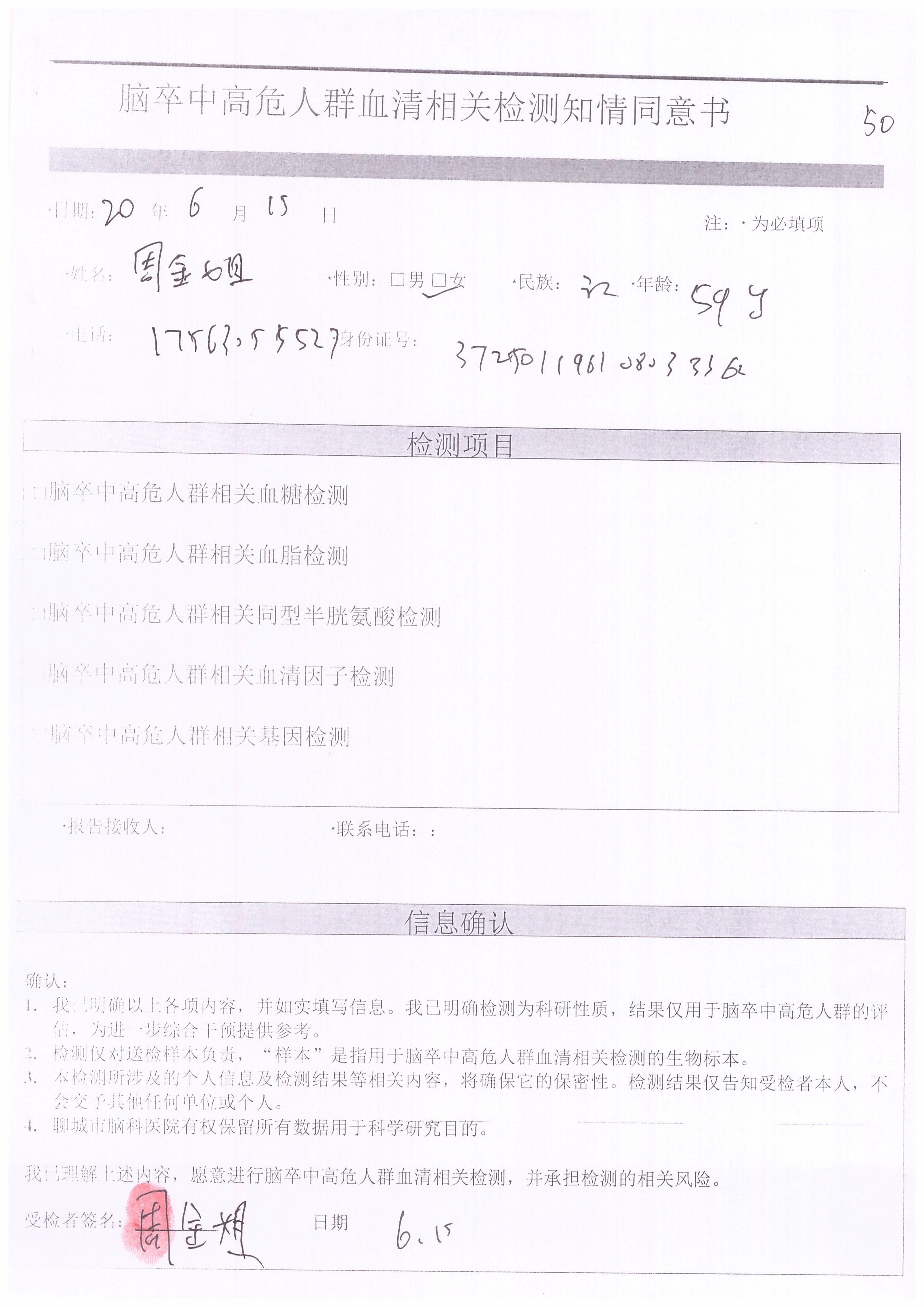

Supplement: Supplementary file 4 — Supplementary file4 (ZIP 25697 KB) [file 10528_2023_10431_MOESM4_ESM.zip › ╓¬╟Θ═1⁄4╥Γ╩Θ2/009 (2).jpg]

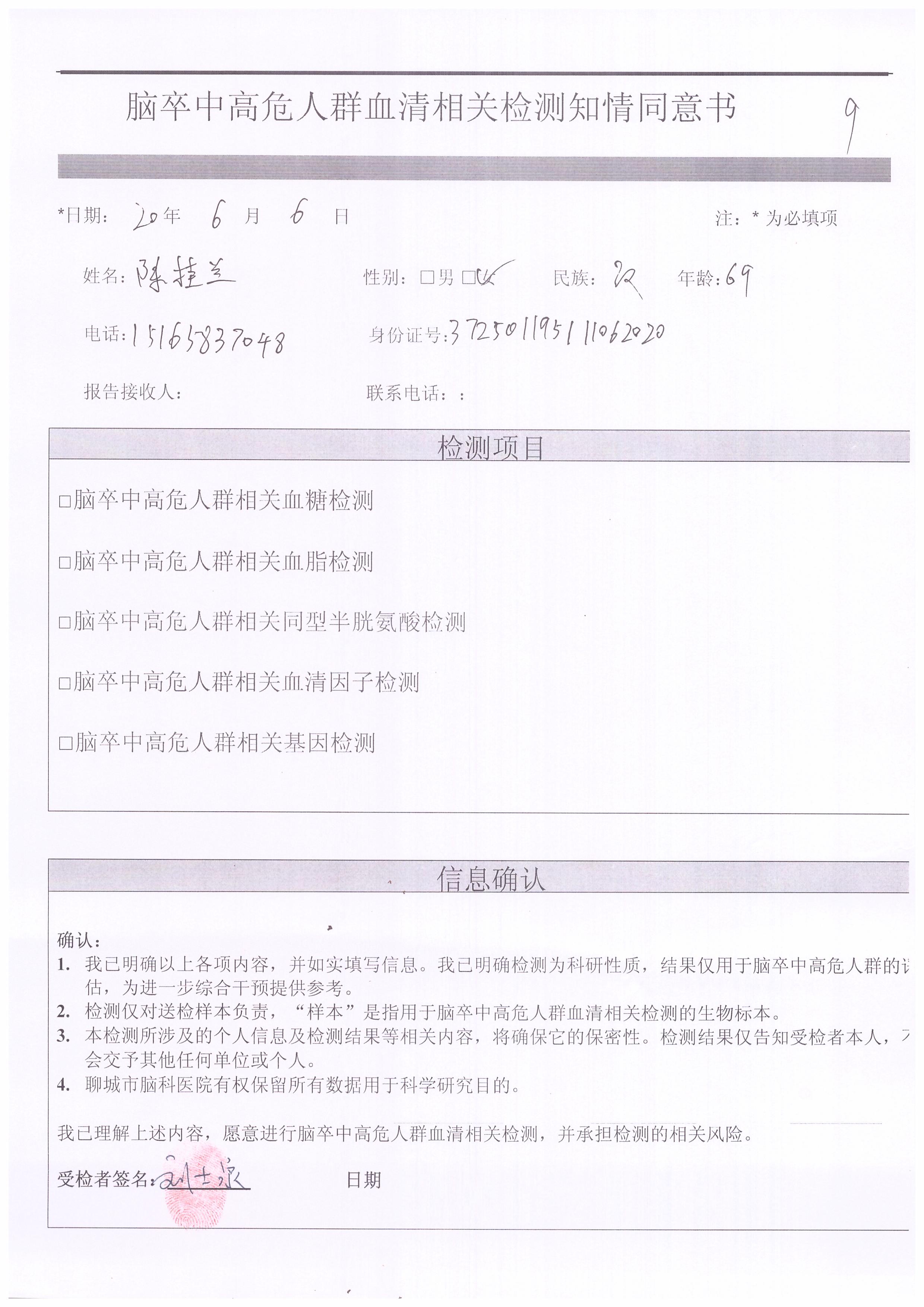

Supplement: Supplementary file 4 — Supplementary file4 (ZIP 25697 KB) [file 10528_2023_10431_MOESM4_ESM.zip › ╓¬╟Θ═1⁄4╥Γ╩Θ2/009.jpg]

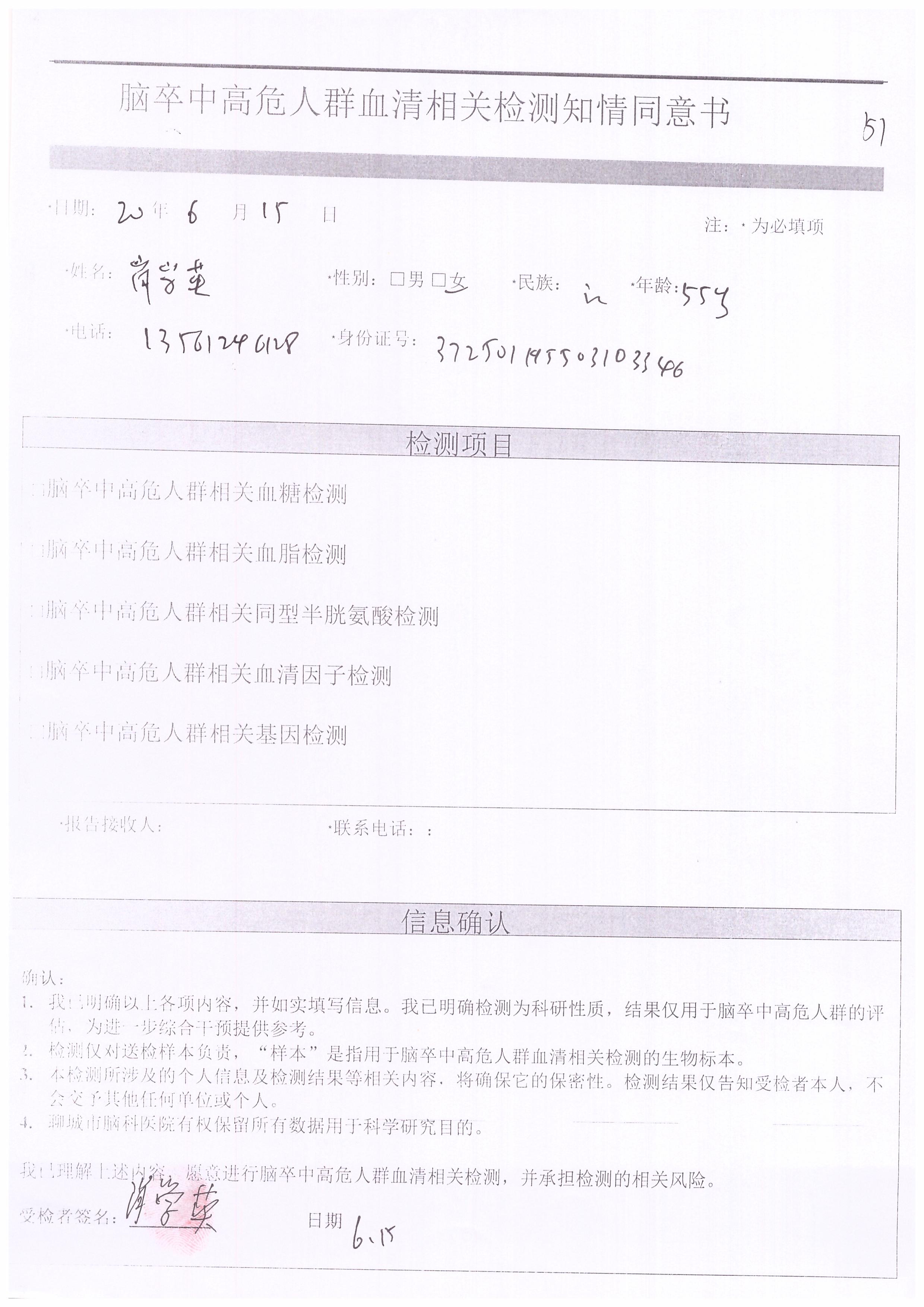

Supplement: Supplementary file 4 — Supplementary file4 (ZIP 25697 KB) [file 10528_2023_10431_MOESM4_ESM.zip › ╓¬╟Θ═1⁄4╥Γ╩Θ2/010 (2).jpg]

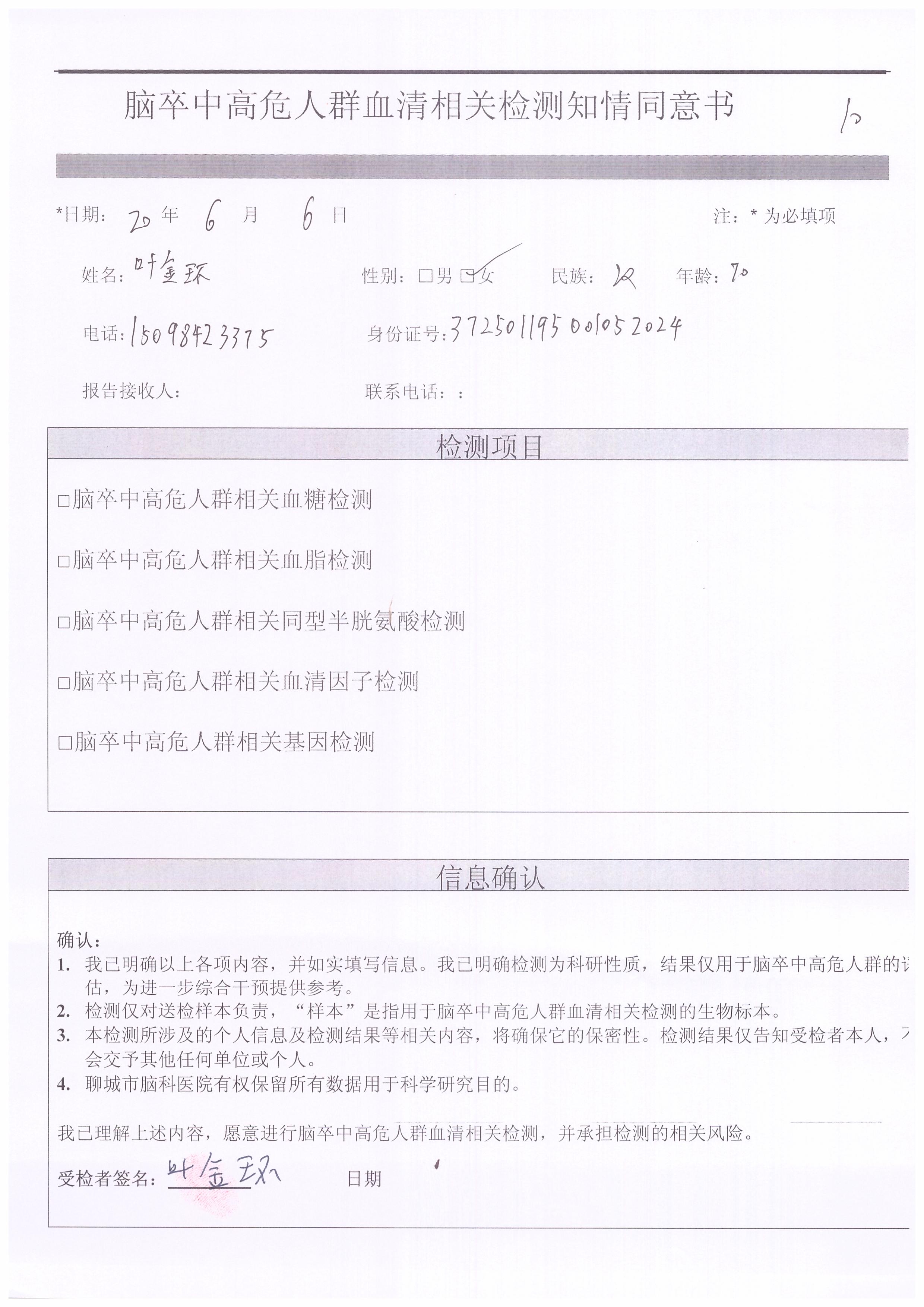

Supplement: Supplementary file 4 — Supplementary file4 (ZIP 25697 KB) [file 10528_2023_10431_MOESM4_ESM.zip › ╓¬╟Θ═1⁄4╥Γ╩Θ2/010.jpg]

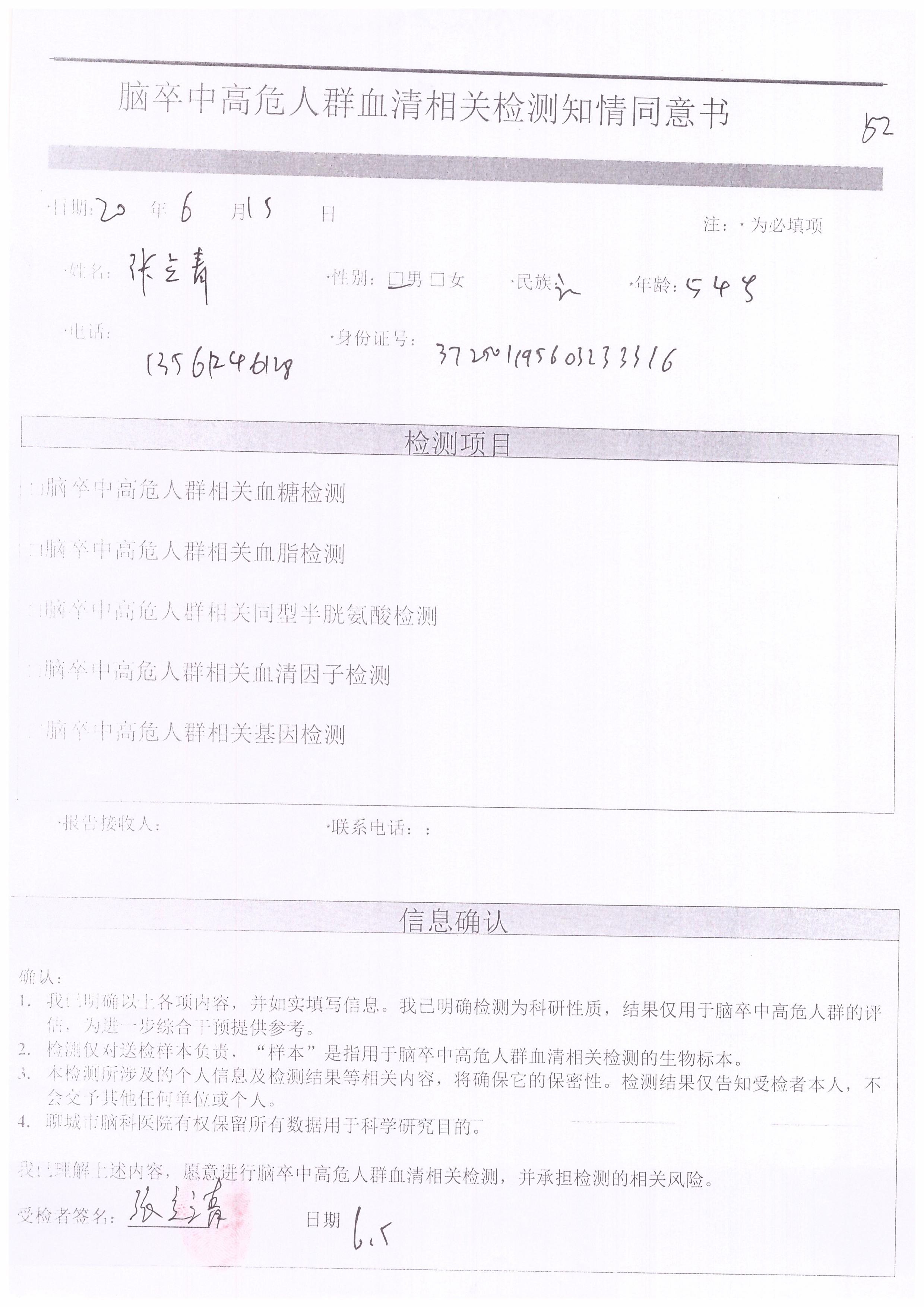

Supplement: Supplementary file 4 — Supplementary file4 (ZIP 25697 KB) [file 10528_2023_10431_MOESM4_ESM.zip › ╓¬╟Θ═1⁄4╥Γ╩Θ2/011 (2).jpg]

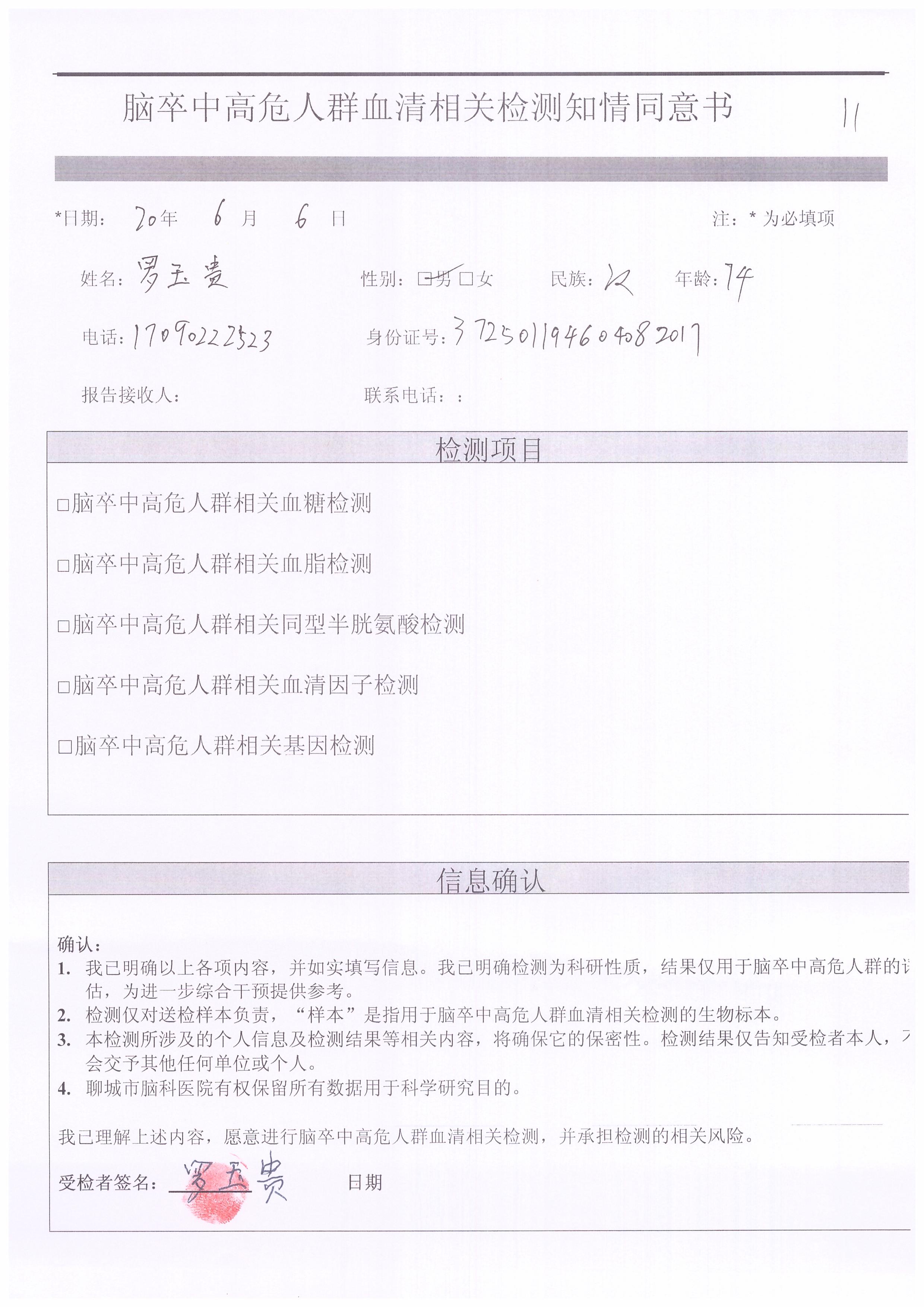

Supplement: Supplementary file 4 — Supplementary file4 (ZIP 25697 KB) [file 10528_2023_10431_MOESM4_ESM.zip › ╓¬╟Θ═1⁄4╥Γ╩Θ2/011.jpg]

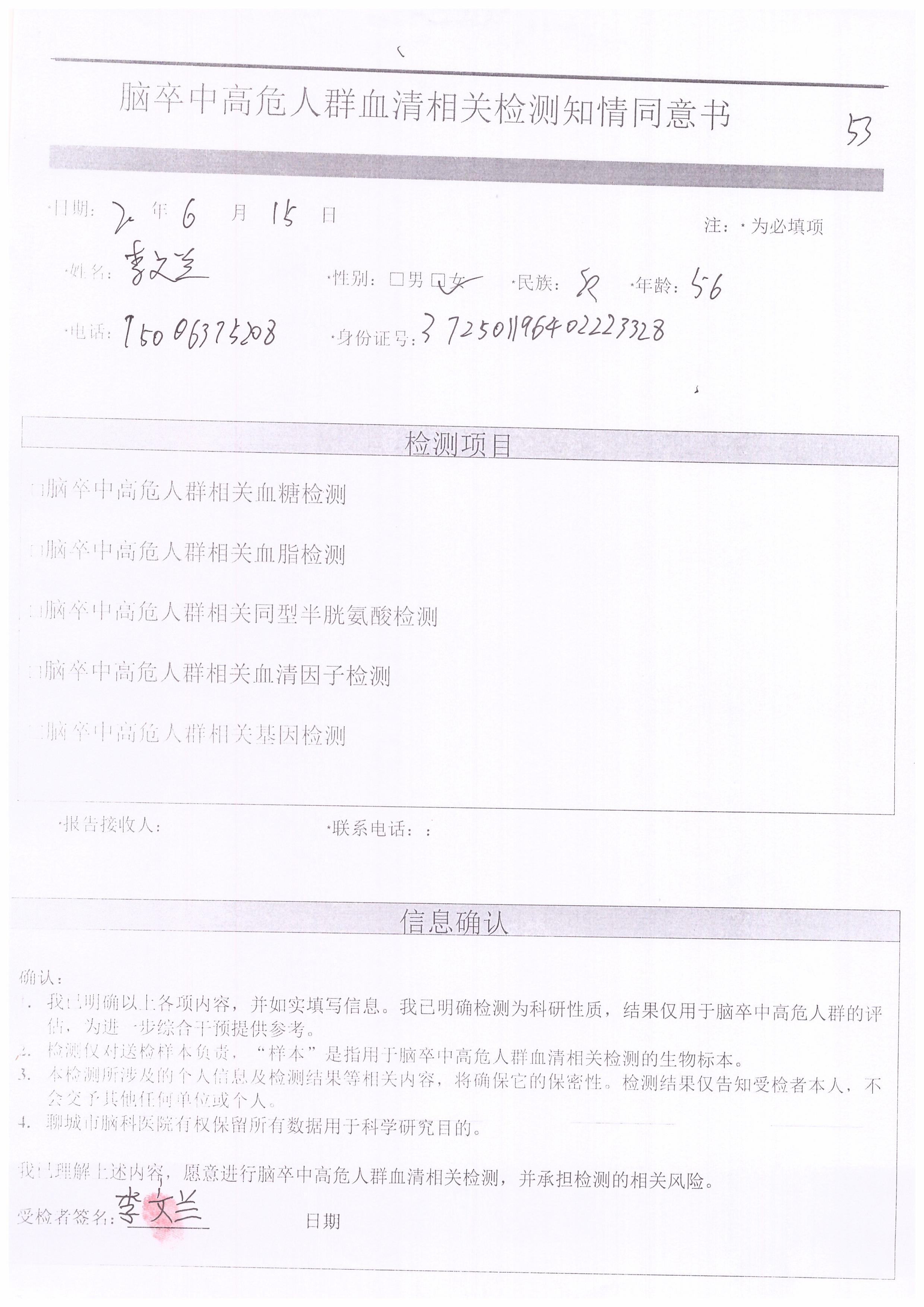

Supplement: Supplementary file 4 — Supplementary file4 (ZIP 25697 KB) [file 10528_2023_10431_MOESM4_ESM.zip › ╓¬╟Θ═1⁄4╥Γ╩Θ2/012 (2).jpg]

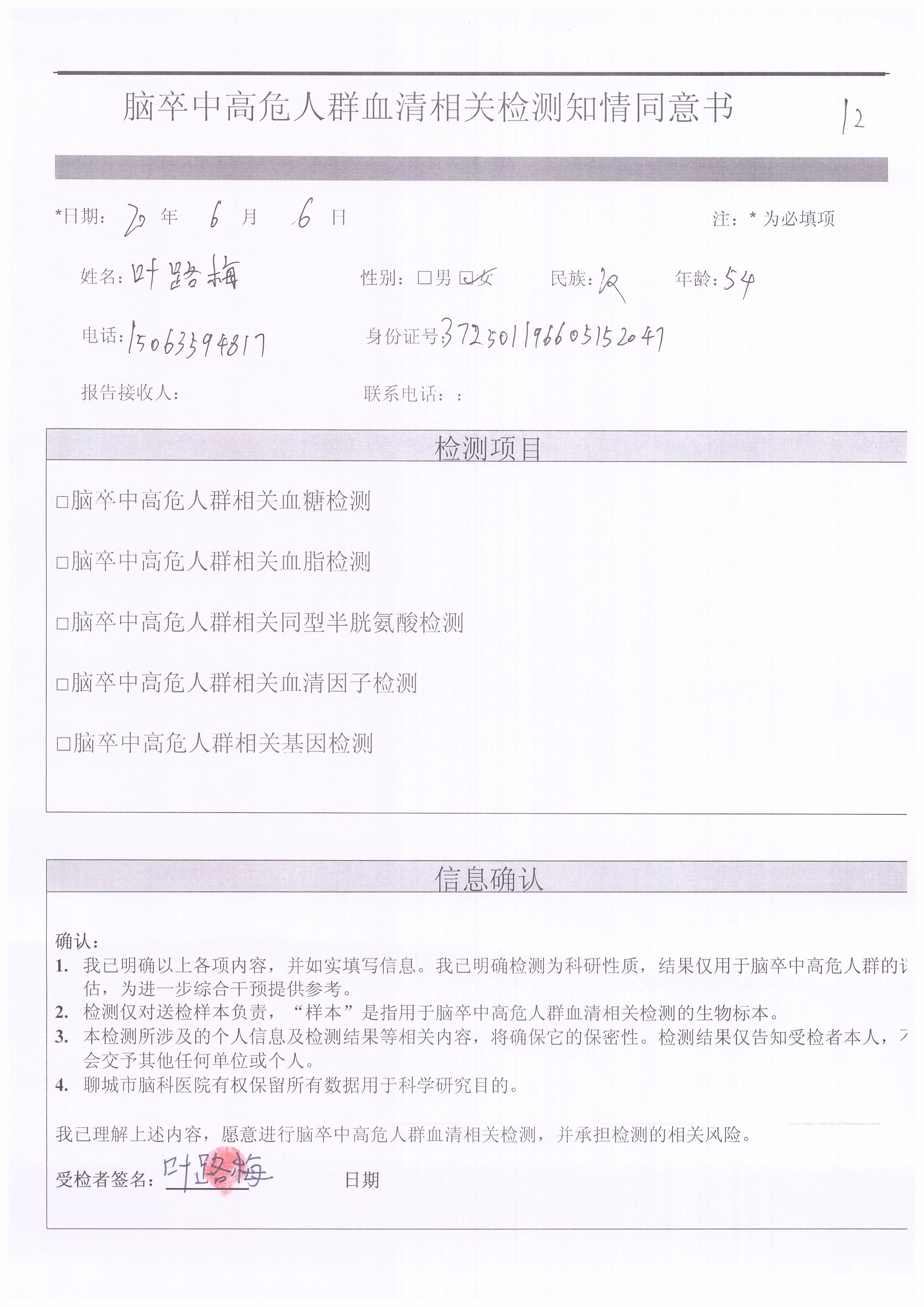

Supplement: Supplementary file 4 — Supplementary file4 (ZIP 25697 KB) [file 10528_2023_10431_MOESM4_ESM.zip › ╓¬╟Θ═1⁄4╥Γ╩Θ2/012.jpg]

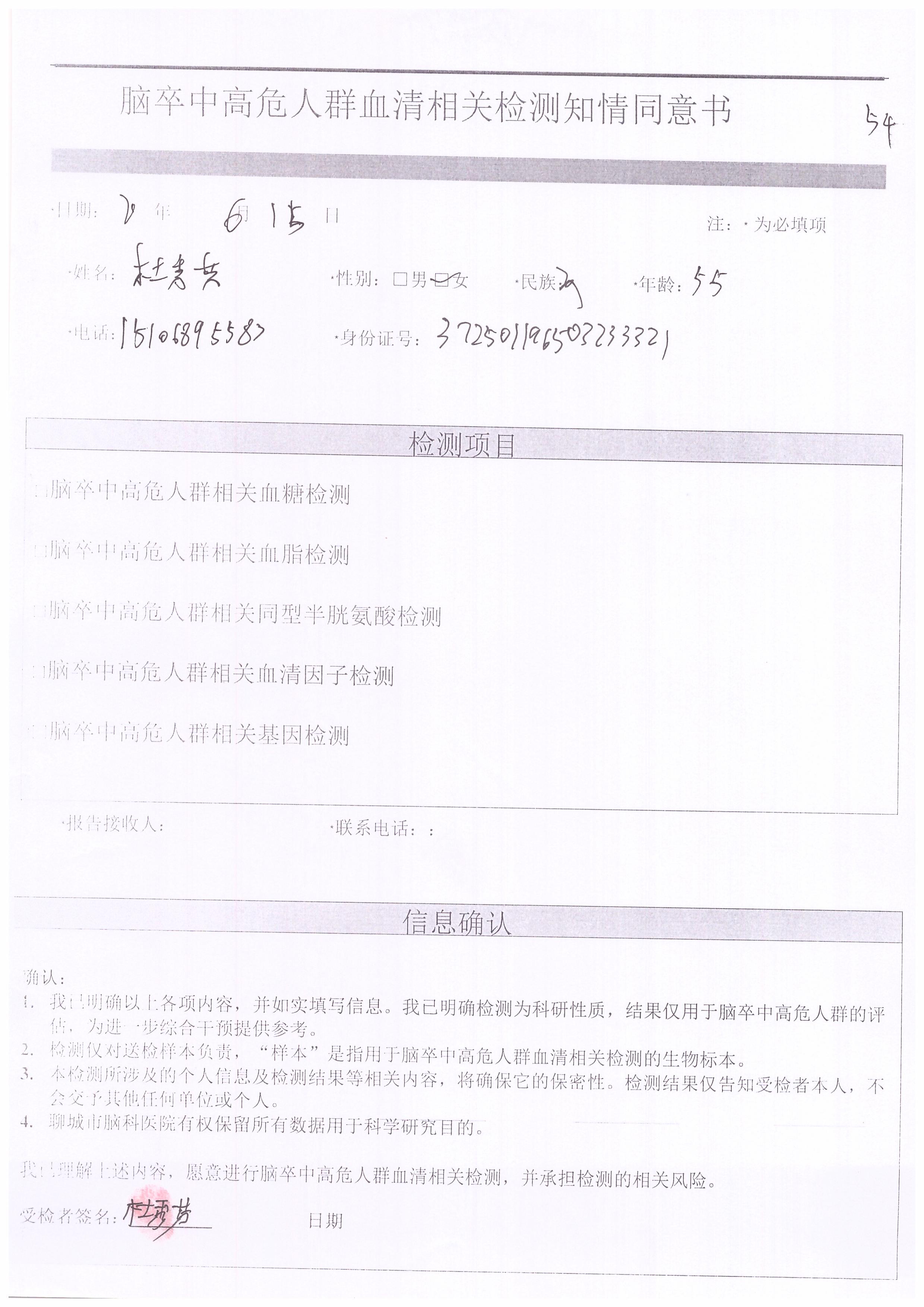

Supplement: Supplementary file 4 — Supplementary file4 (ZIP 25697 KB) [file 10528_2023_10431_MOESM4_ESM.zip › ╓¬╟Θ═1⁄4╥Γ╩Θ2/013 (2).jpg]

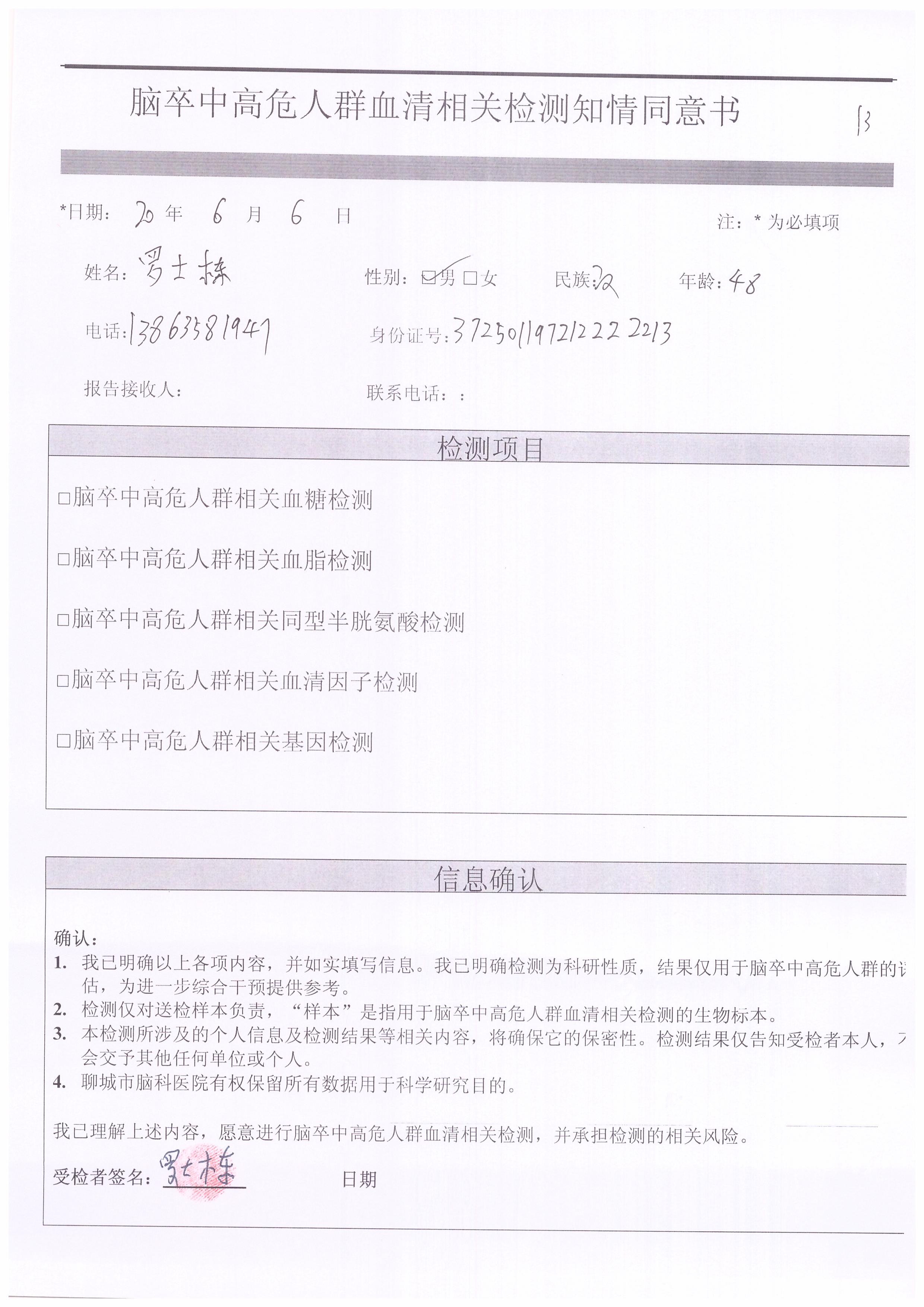

Supplement: Supplementary file 4 — Supplementary file4 (ZIP 25697 KB) [file 10528_2023_10431_MOESM4_ESM.zip › ╓¬╟Θ═1⁄4╥Γ╩Θ2/013.jpg]

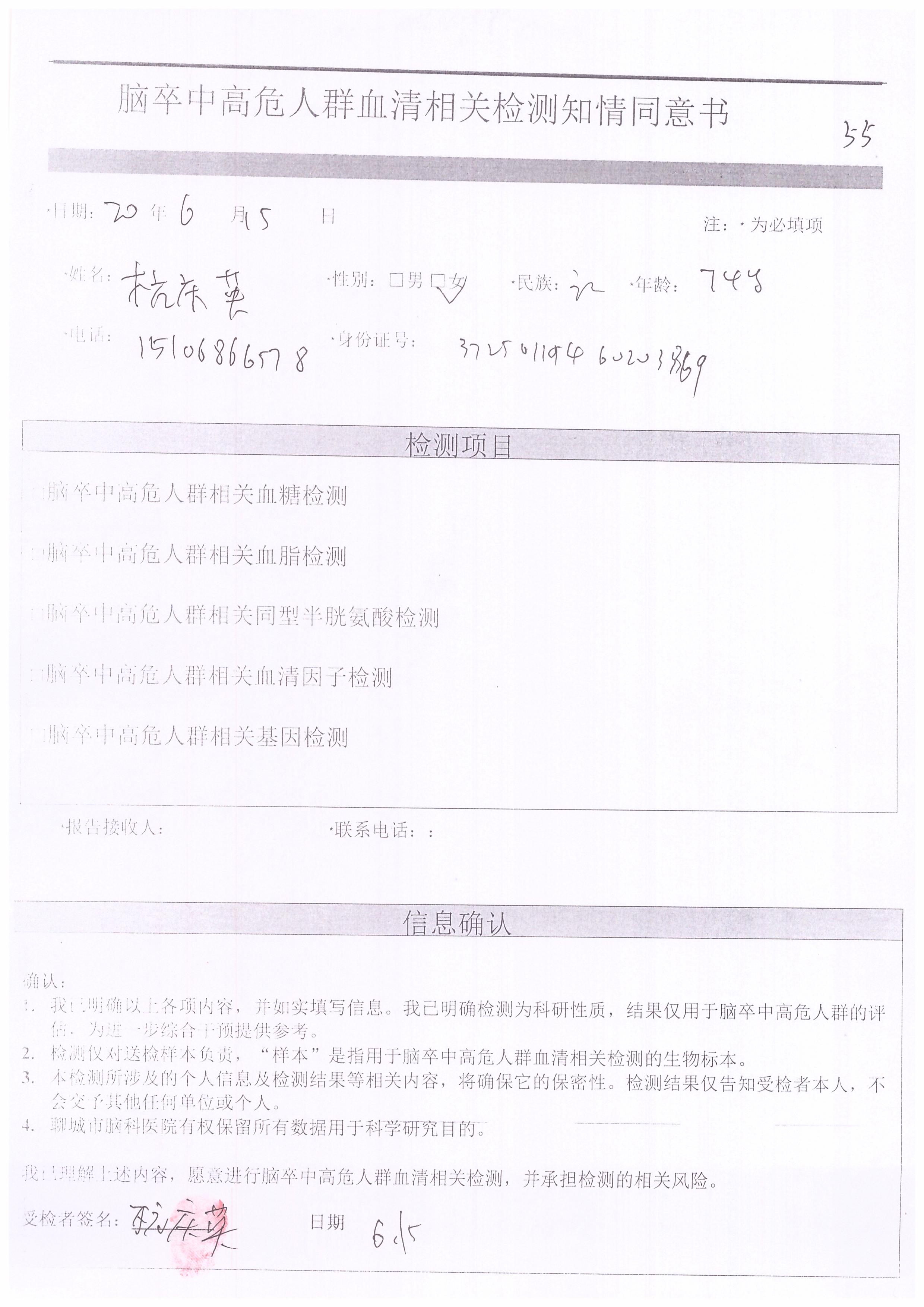

Supplement: Supplementary file 4 — Supplementary file4 (ZIP 25697 KB) [file 10528_2023_10431_MOESM4_ESM.zip › ╓¬╟Θ═1⁄4╥Γ╩Θ2/014 (2).jpg]

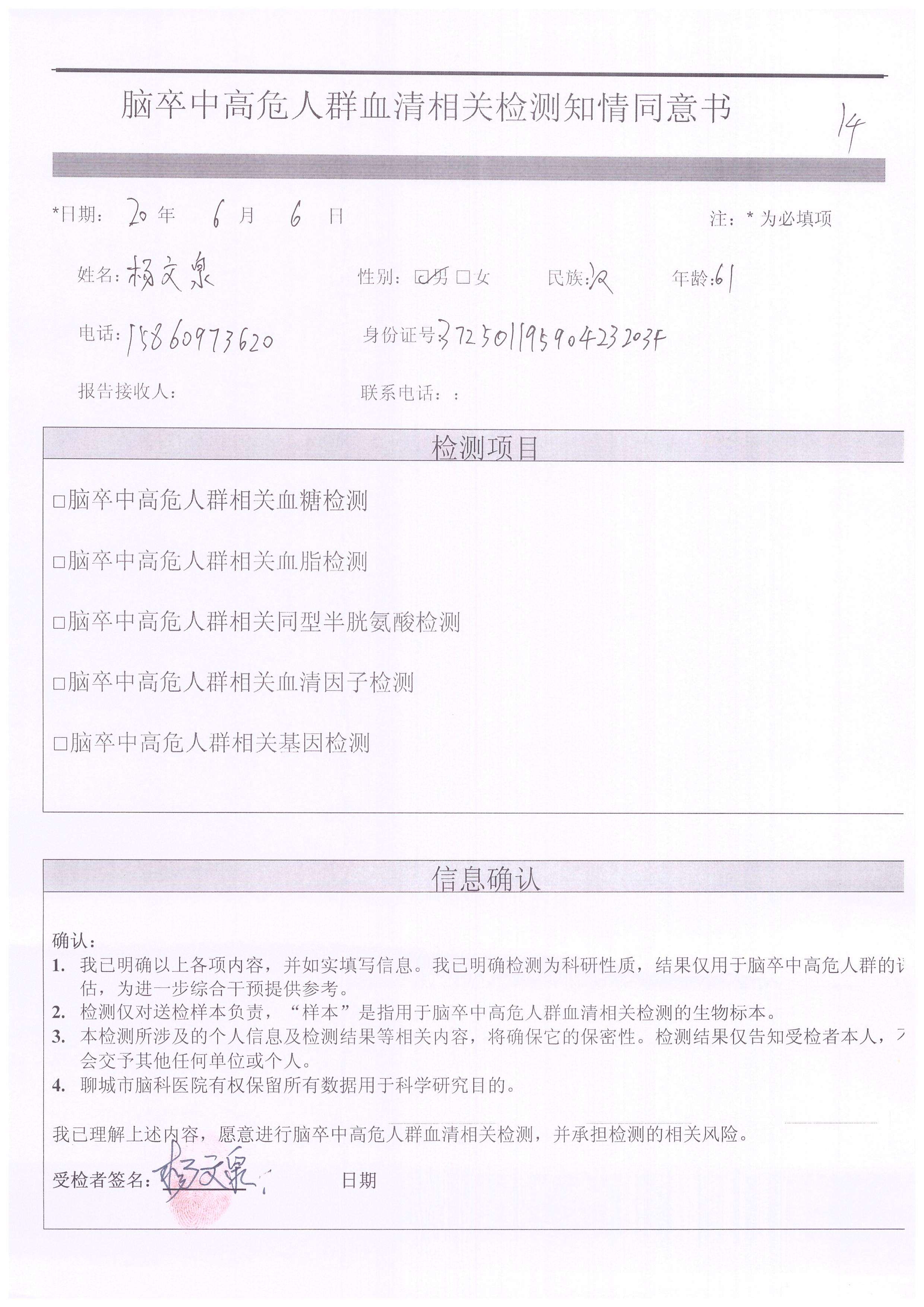

Supplement: Supplementary file 4 — Supplementary file4 (ZIP 25697 KB) [file 10528_2023_10431_MOESM4_ESM.zip › ╓¬╟Θ═1⁄4╥Γ╩Θ2/014.jpg]

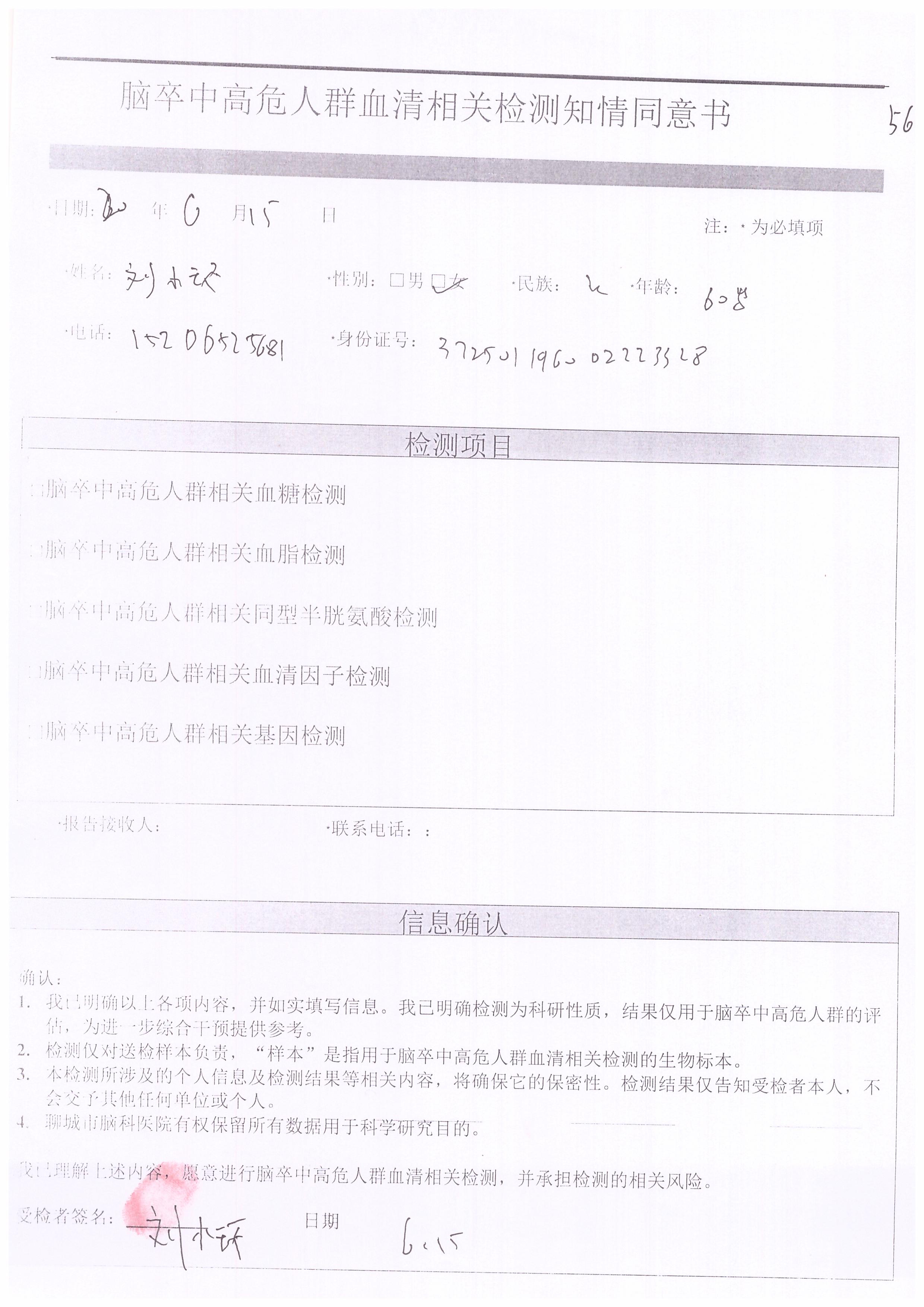

Supplement: Supplementary file 4 — Supplementary file4 (ZIP 25697 KB) [file 10528_2023_10431_MOESM4_ESM.zip › ╓¬╟Θ═1⁄4╥Γ╩Θ2/015 (2).jpg]

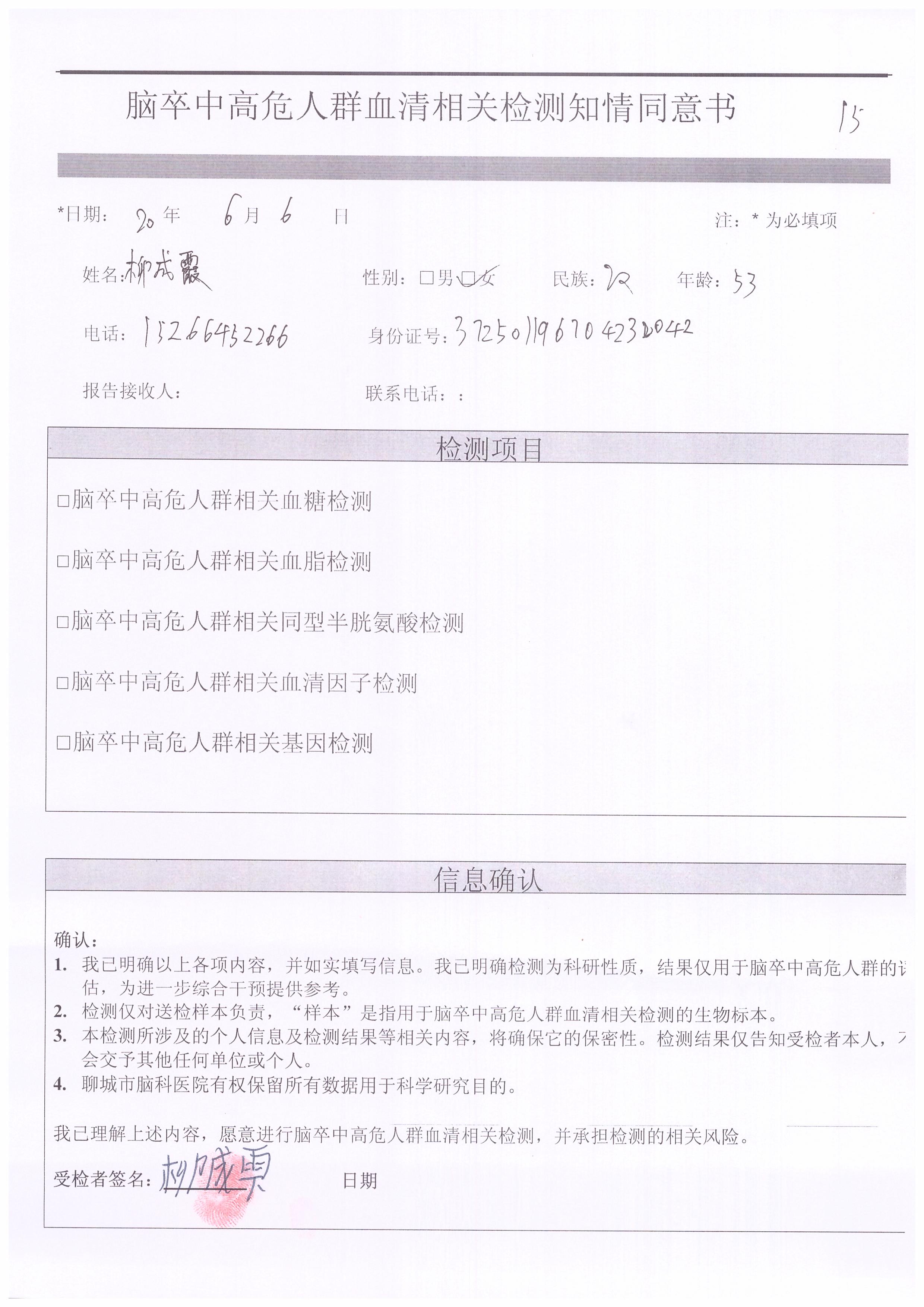

Supplement: Supplementary file 4 — Supplementary file4 (ZIP 25697 KB) [file 10528_2023_10431_MOESM4_ESM.zip › ╓¬╟Θ═1⁄4╥Γ╩Θ2/015.jpg]

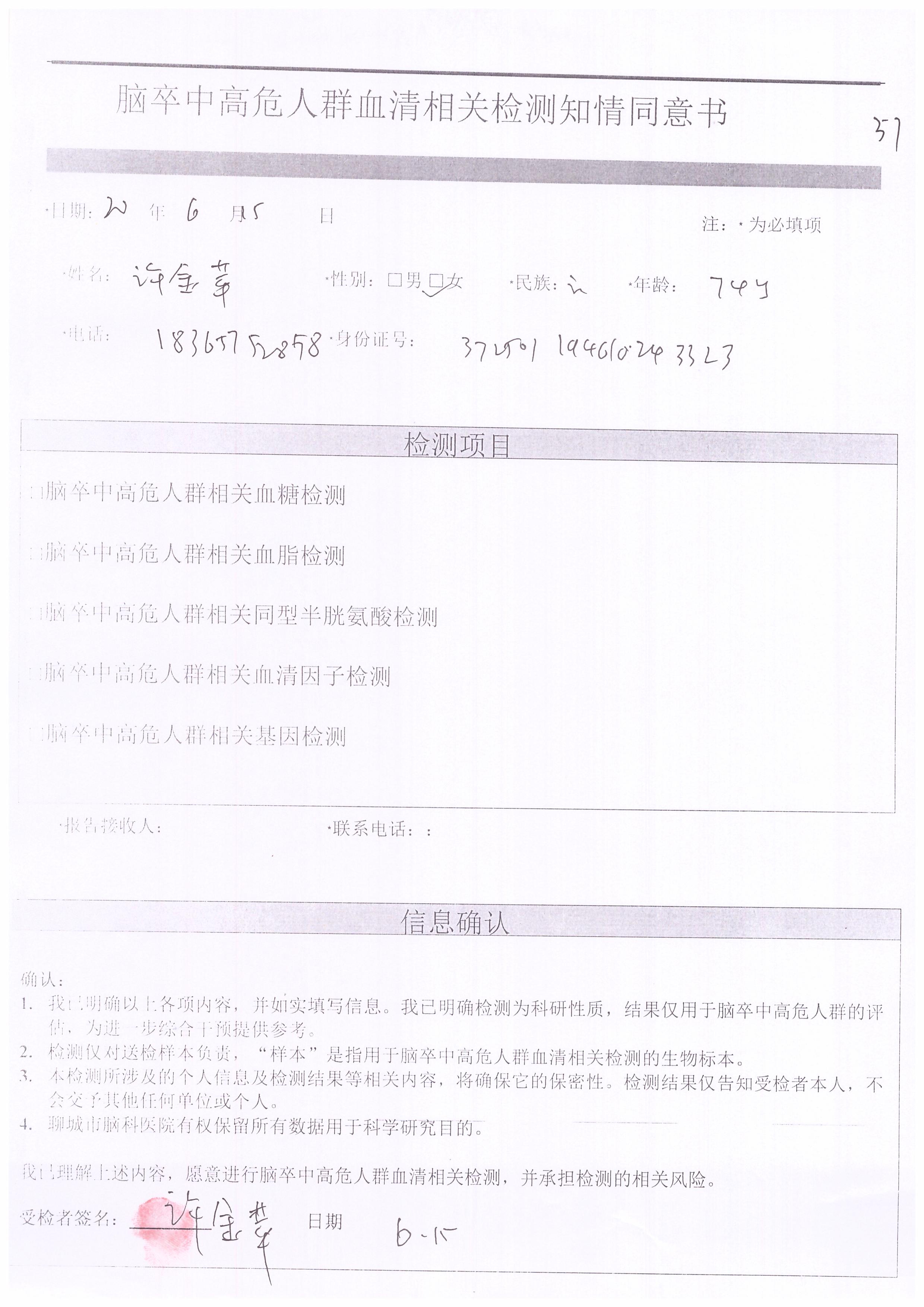

Supplement: Supplementary file 4 — Supplementary file4 (ZIP 25697 KB) [file 10528_2023_10431_MOESM4_ESM.zip › ╓¬╟Θ═1⁄4╥Γ╩Θ2/016 (2).jpg]

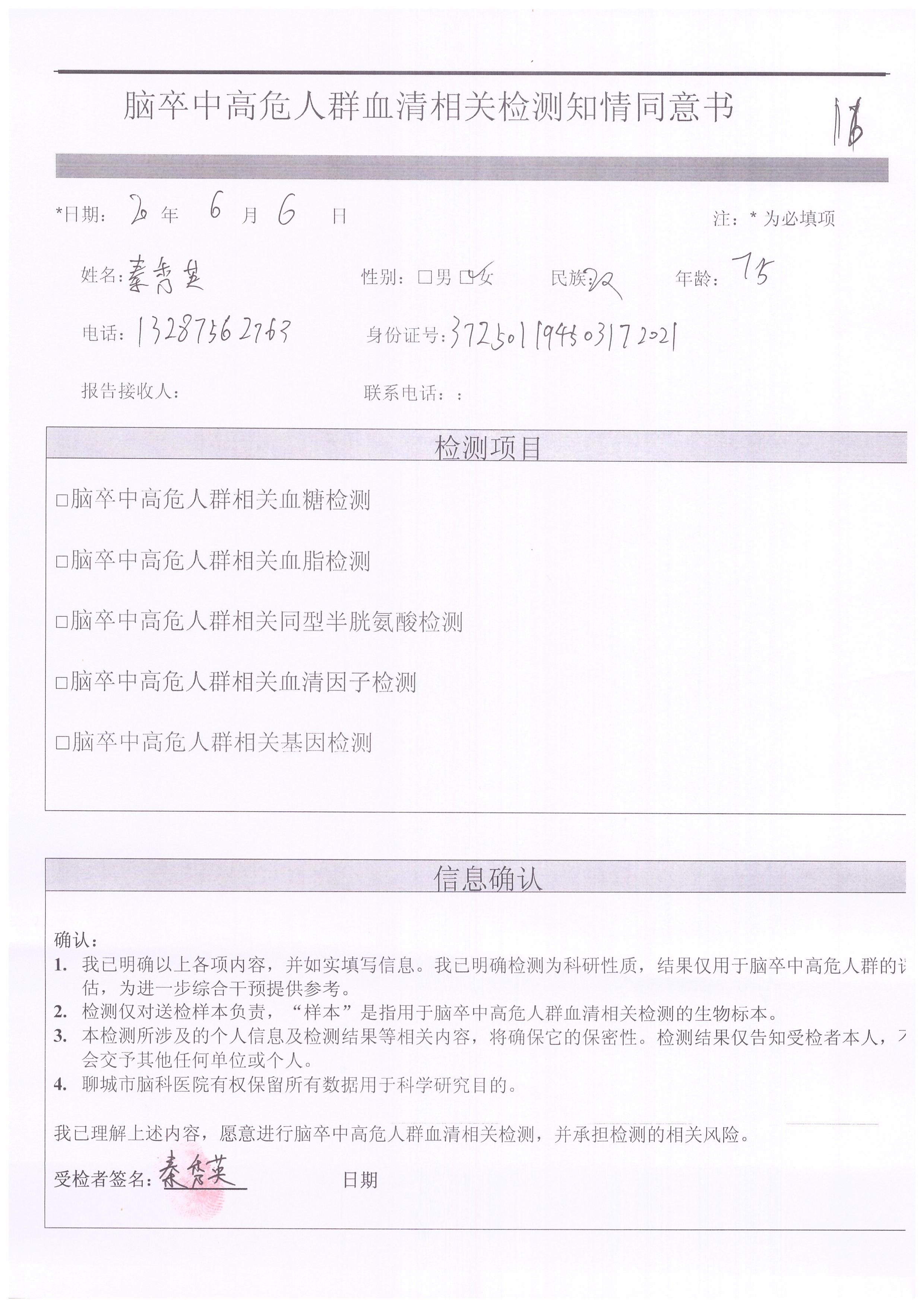

Supplement: Supplementary file 4 — Supplementary file4 (ZIP 25697 KB) [file 10528_2023_10431_MOESM4_ESM.zip › ╓¬╟Θ═1⁄4╥Γ╩Θ2/016.jpg]

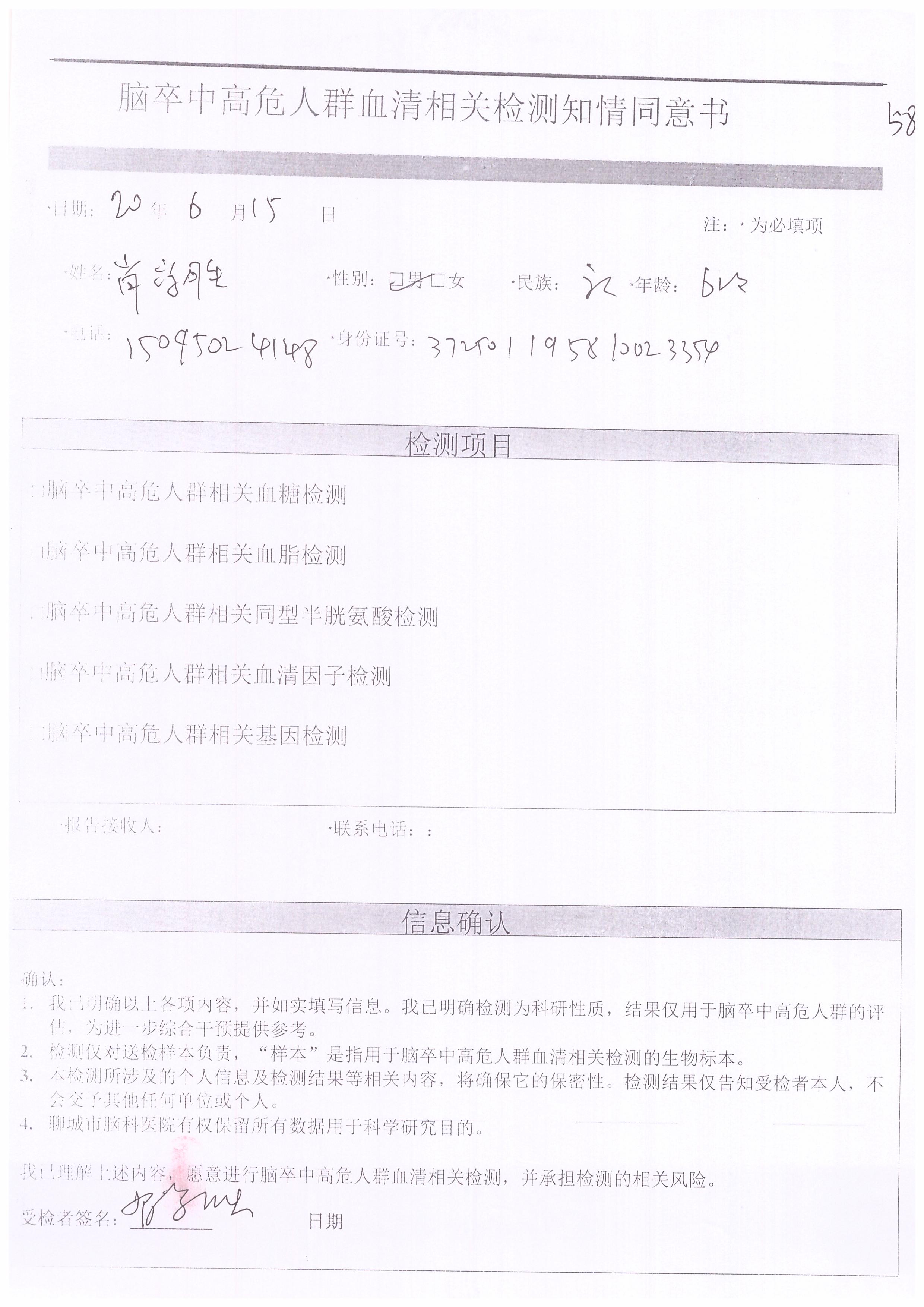

Supplement: Supplementary file 4 — Supplementary file4 (ZIP 25697 KB) [file 10528_2023_10431_MOESM4_ESM.zip › ╓¬╟Θ═1⁄4╥Γ╩Θ2/017 (2).jpg]

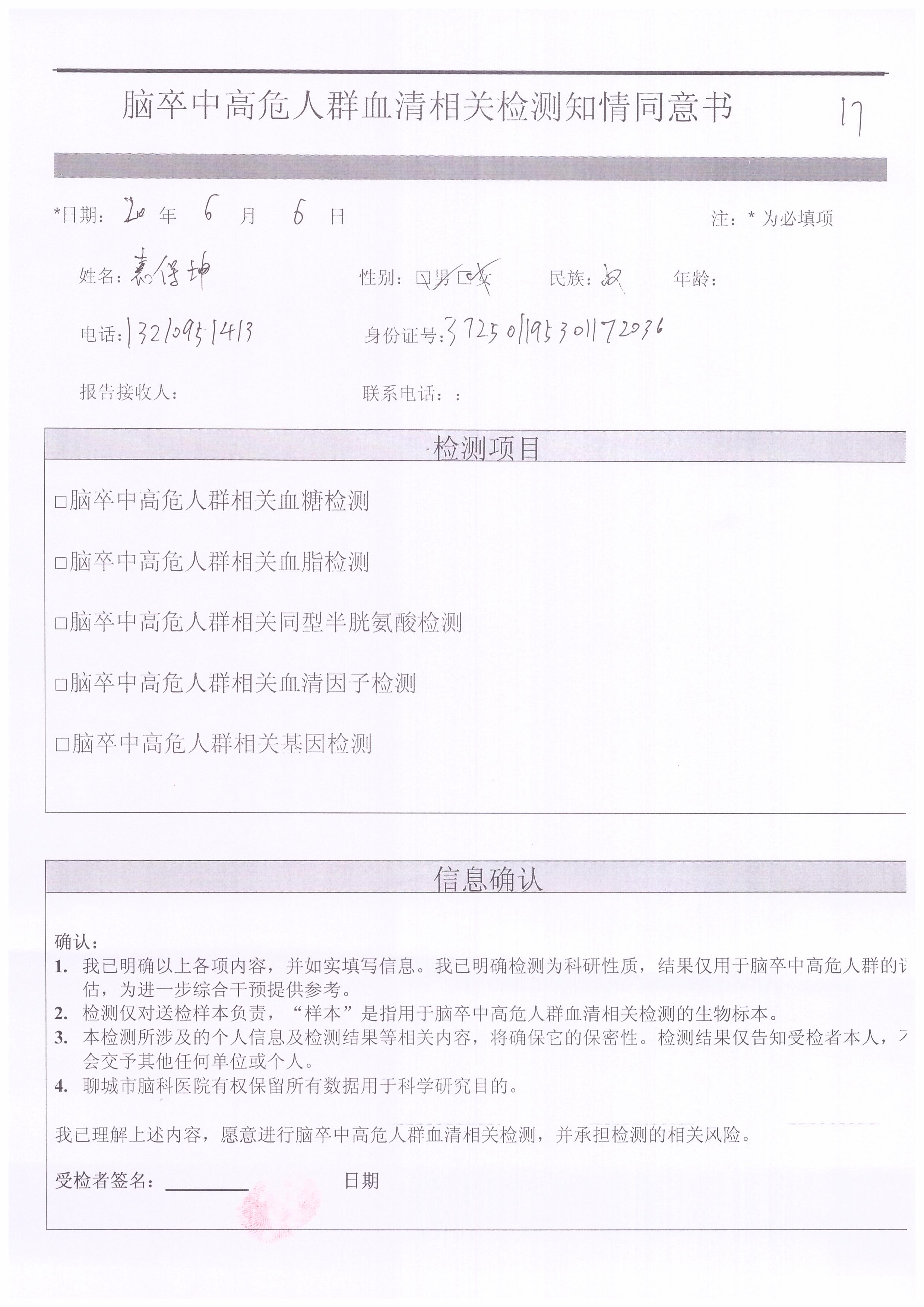

Supplement: Supplementary file 4 — Supplementary file4 (ZIP 25697 KB) [file 10528_2023_10431_MOESM4_ESM.zip › ╓¬╟Θ═1⁄4╥Γ╩Θ2/017.jpg]

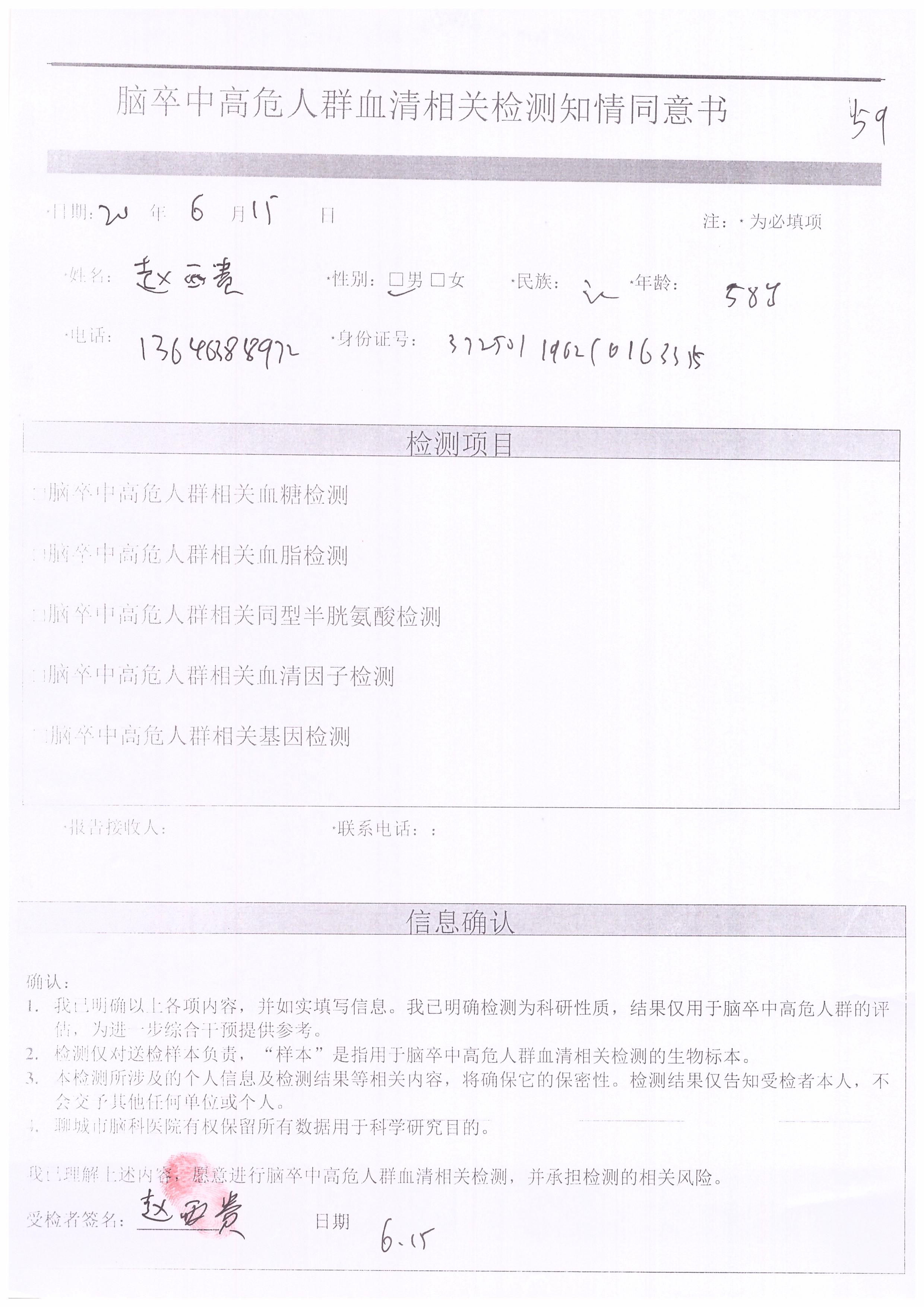

Supplement: Supplementary file 4 — Supplementary file4 (ZIP 25697 KB) [file 10528_2023_10431_MOESM4_ESM.zip › ╓¬╟Θ═1⁄4╥Γ╩Θ2/018 (2).jpg]

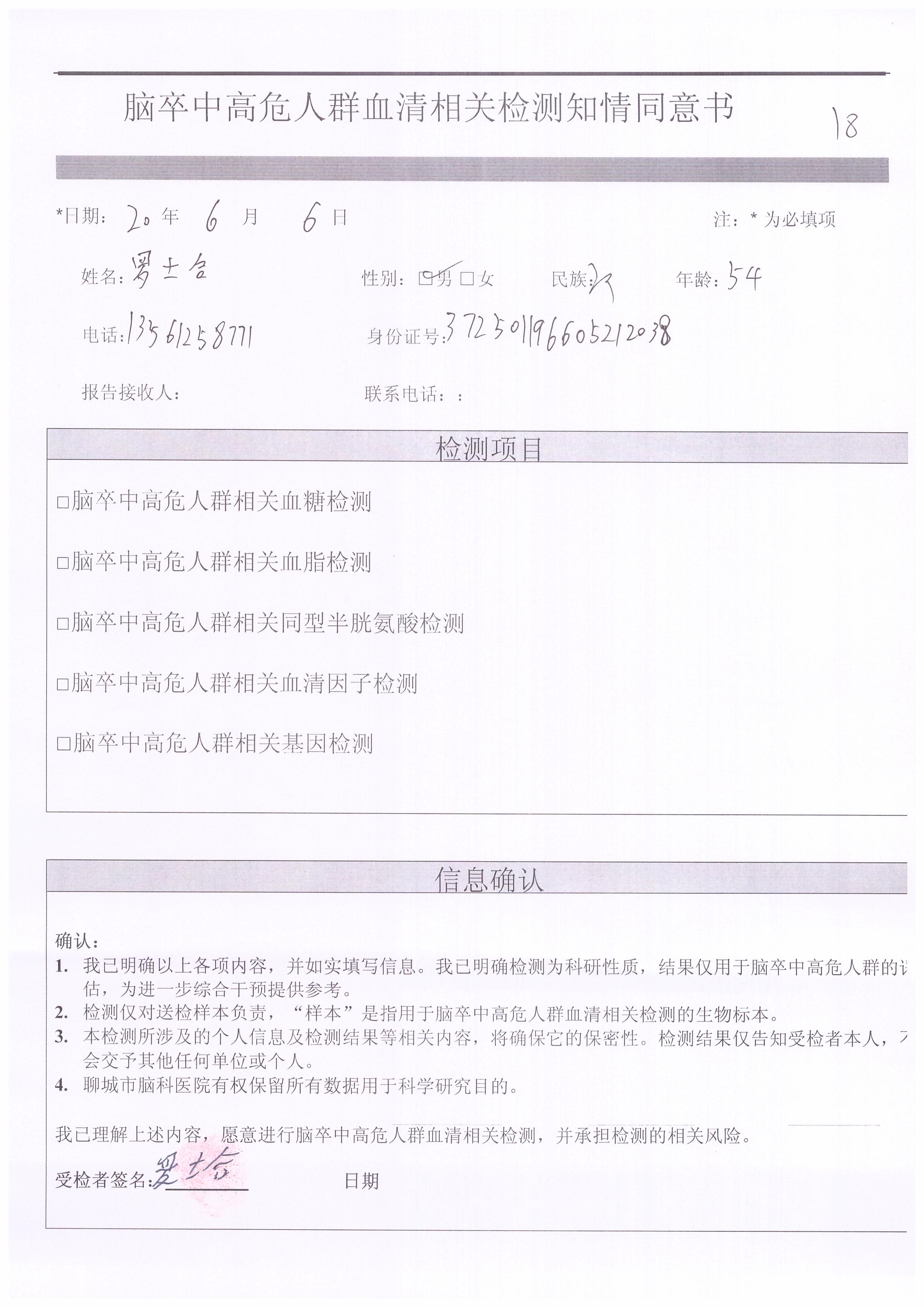

Supplement: Supplementary file 4 — Supplementary file4 (ZIP 25697 KB) [file 10528_2023_10431_MOESM4_ESM.zip › ╓¬╟Θ═1⁄4╥Γ╩Θ2/018.jpg]

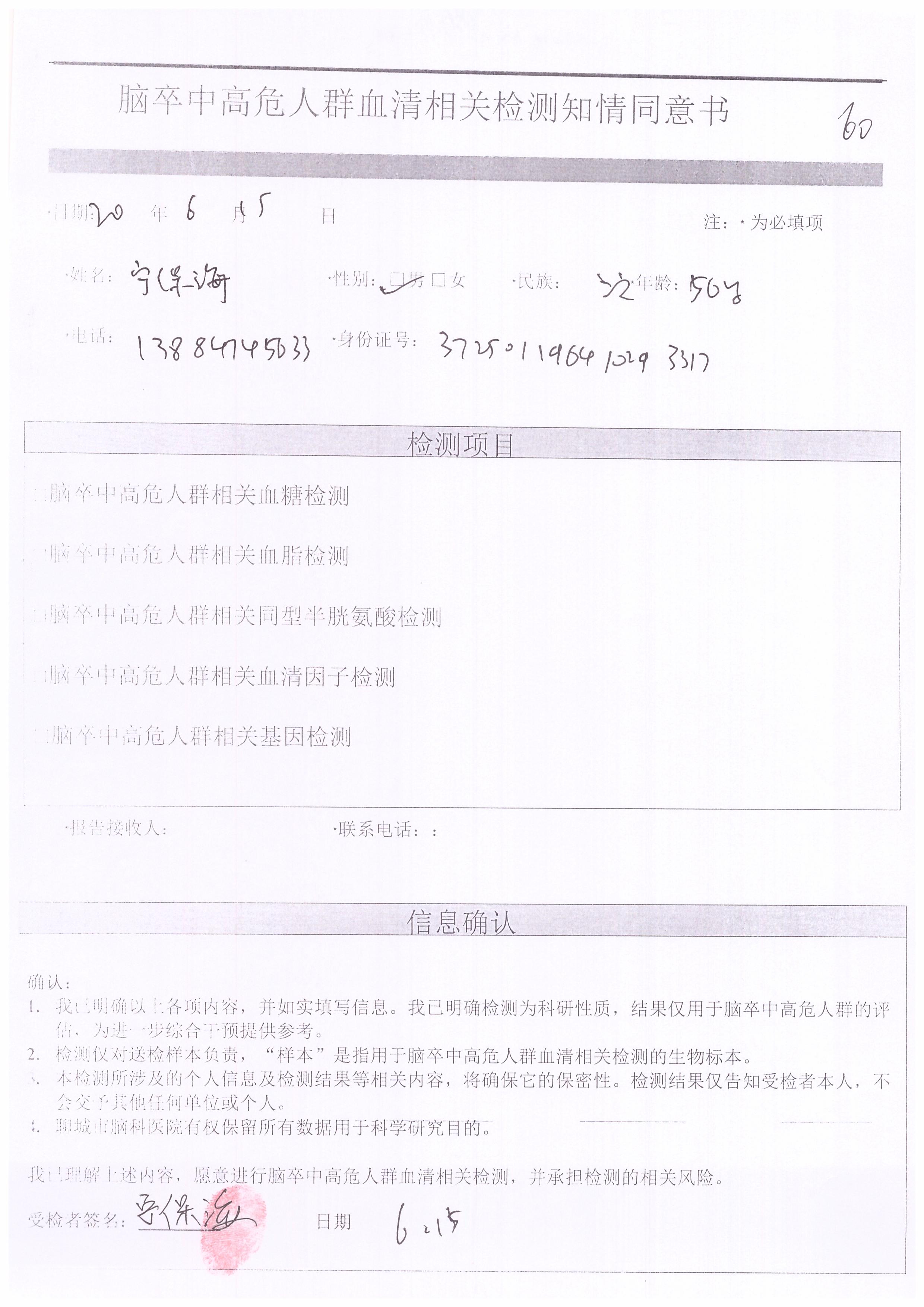

Supplement: Supplementary file 4 — Supplementary file4 (ZIP 25697 KB) [file 10528_2023_10431_MOESM4_ESM.zip › ╓¬╟Θ═1⁄4╥Γ╩Θ2/019 (2).jpg]

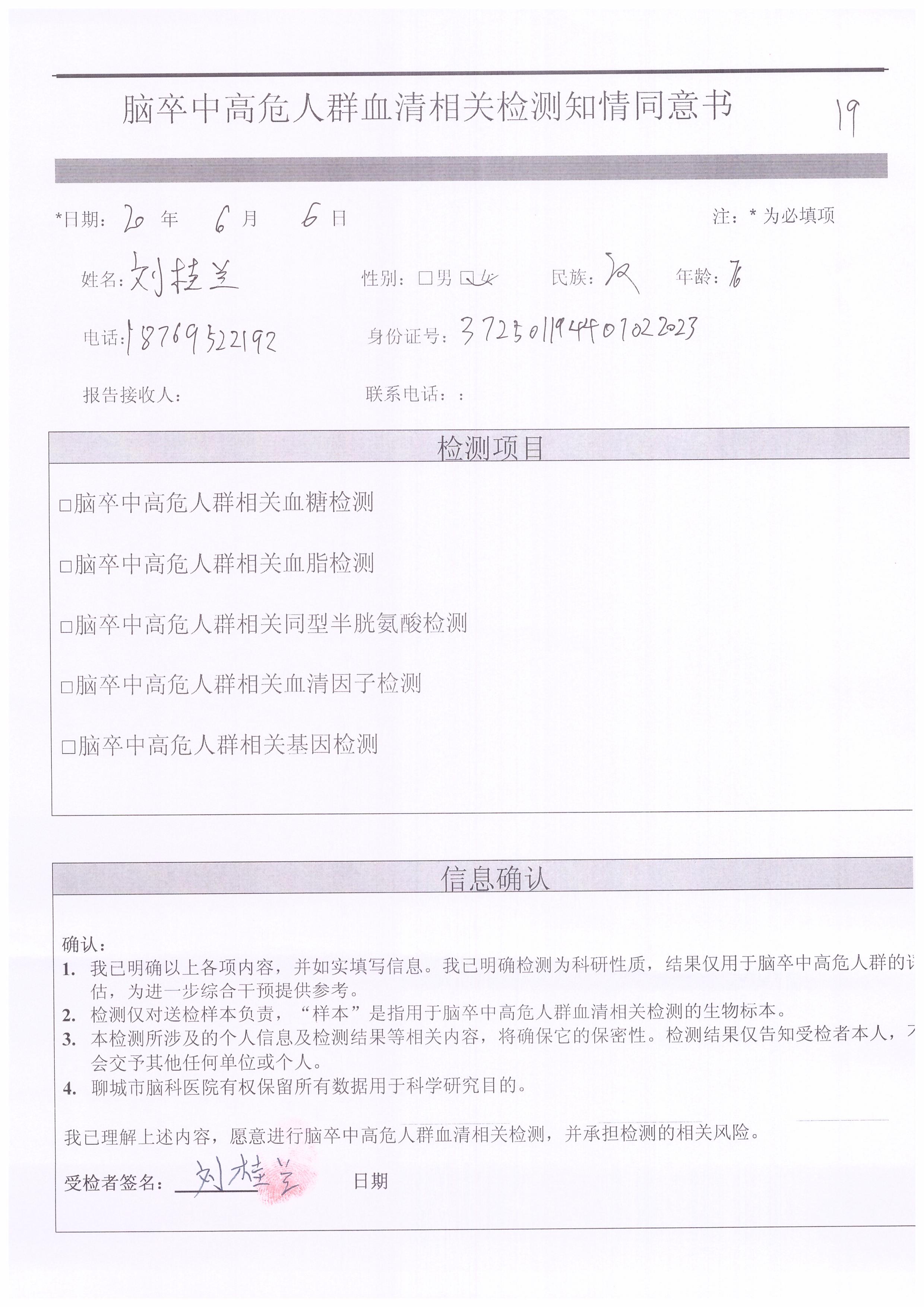

Supplement: Supplementary file 4 — Supplementary file4 (ZIP 25697 KB) [file 10528_2023_10431_MOESM4_ESM.zip › ╓¬╟Θ═1⁄4╥Γ╩Θ2/019.jpg]

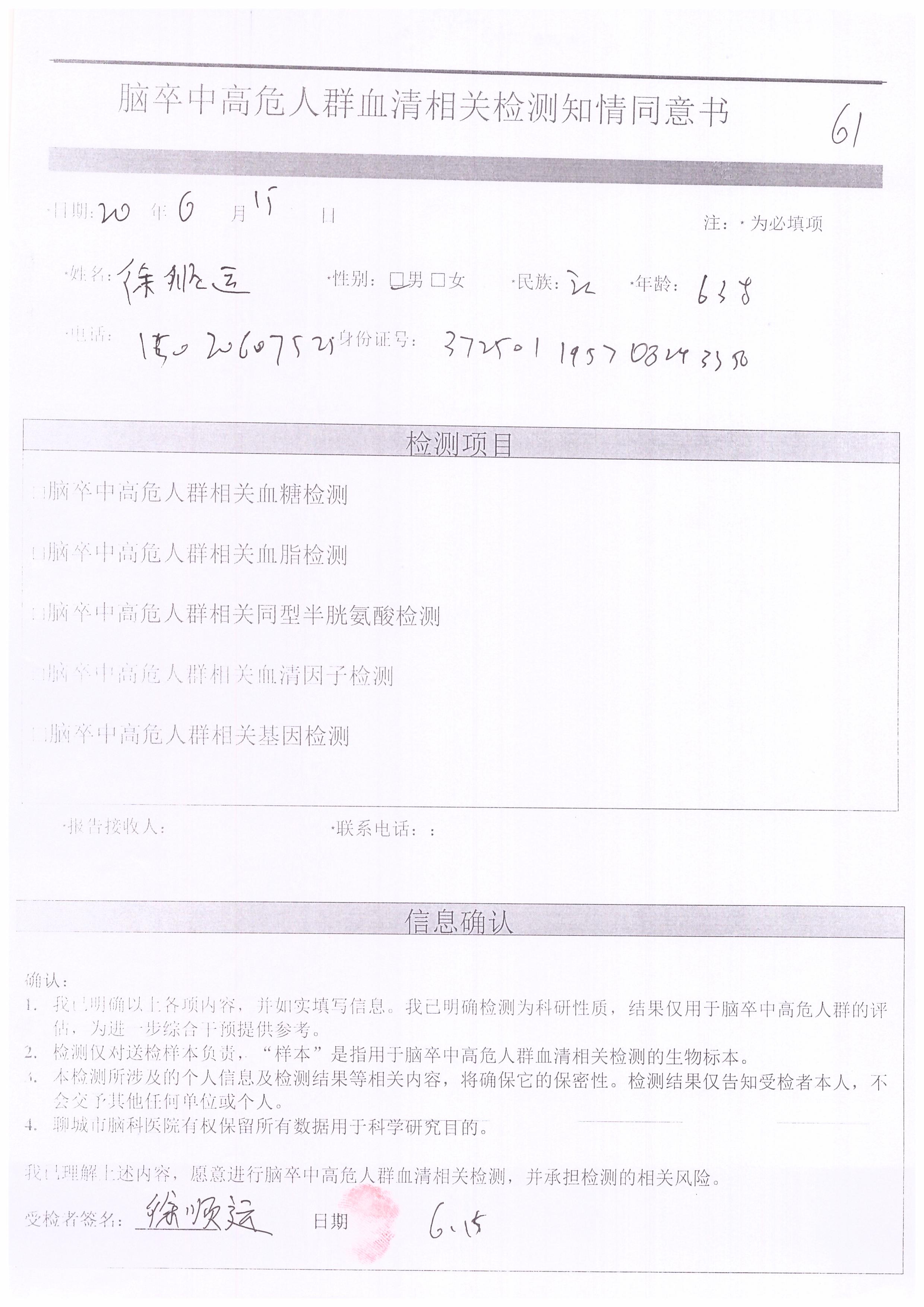

Supplement: Supplementary file 4 — Supplementary file4 (ZIP 25697 KB) [file 10528_2023_10431_MOESM4_ESM.zip › ╓¬╟Θ═1⁄4╥Γ╩Θ2/020 (2).jpg]

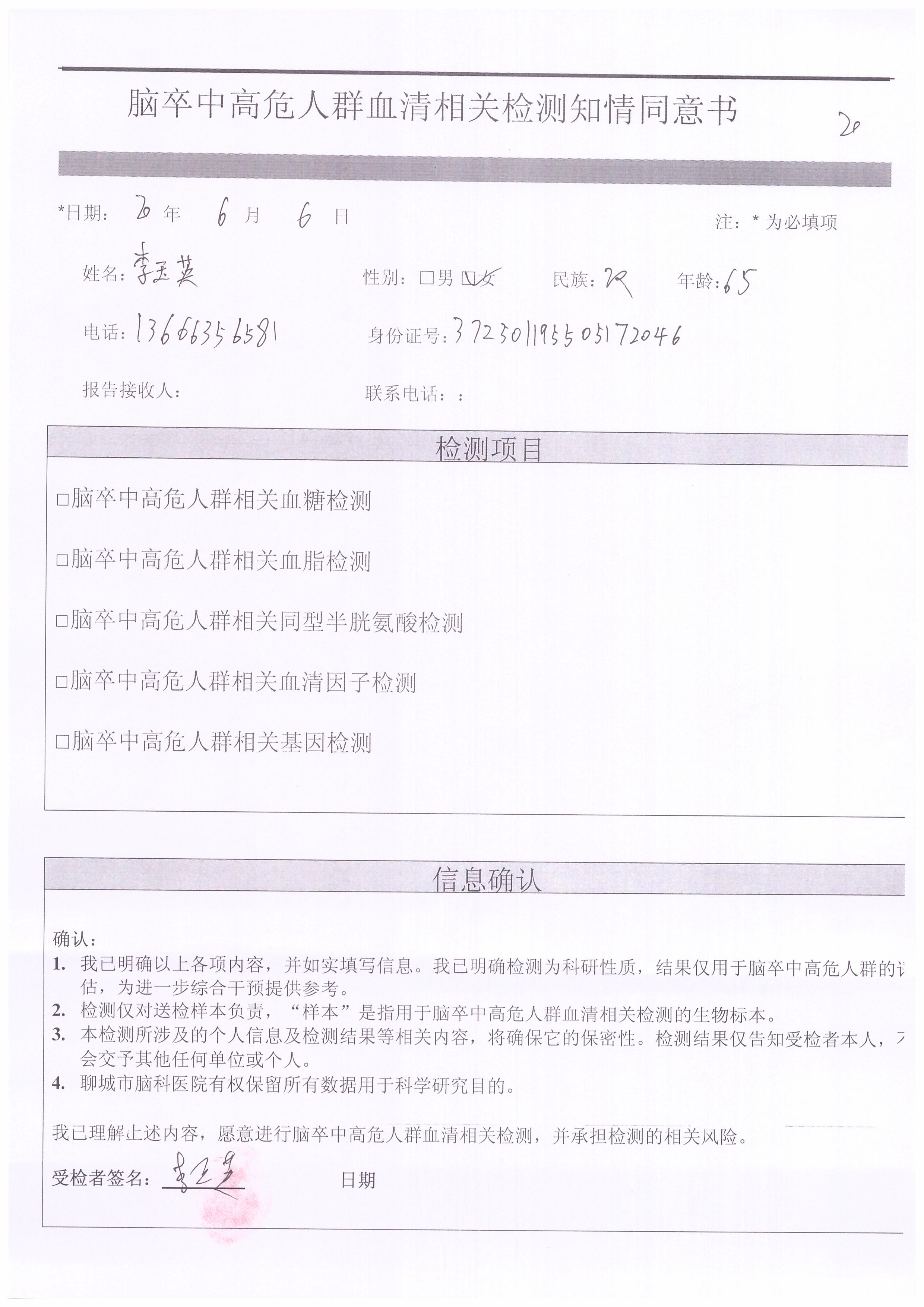

Supplement: Supplementary file 4 — Supplementary file4 (ZIP 25697 KB) [file 10528_2023_10431_MOESM4_ESM.zip › ╓¬╟Θ═1⁄4╥Γ╩Θ2/020.jpg]

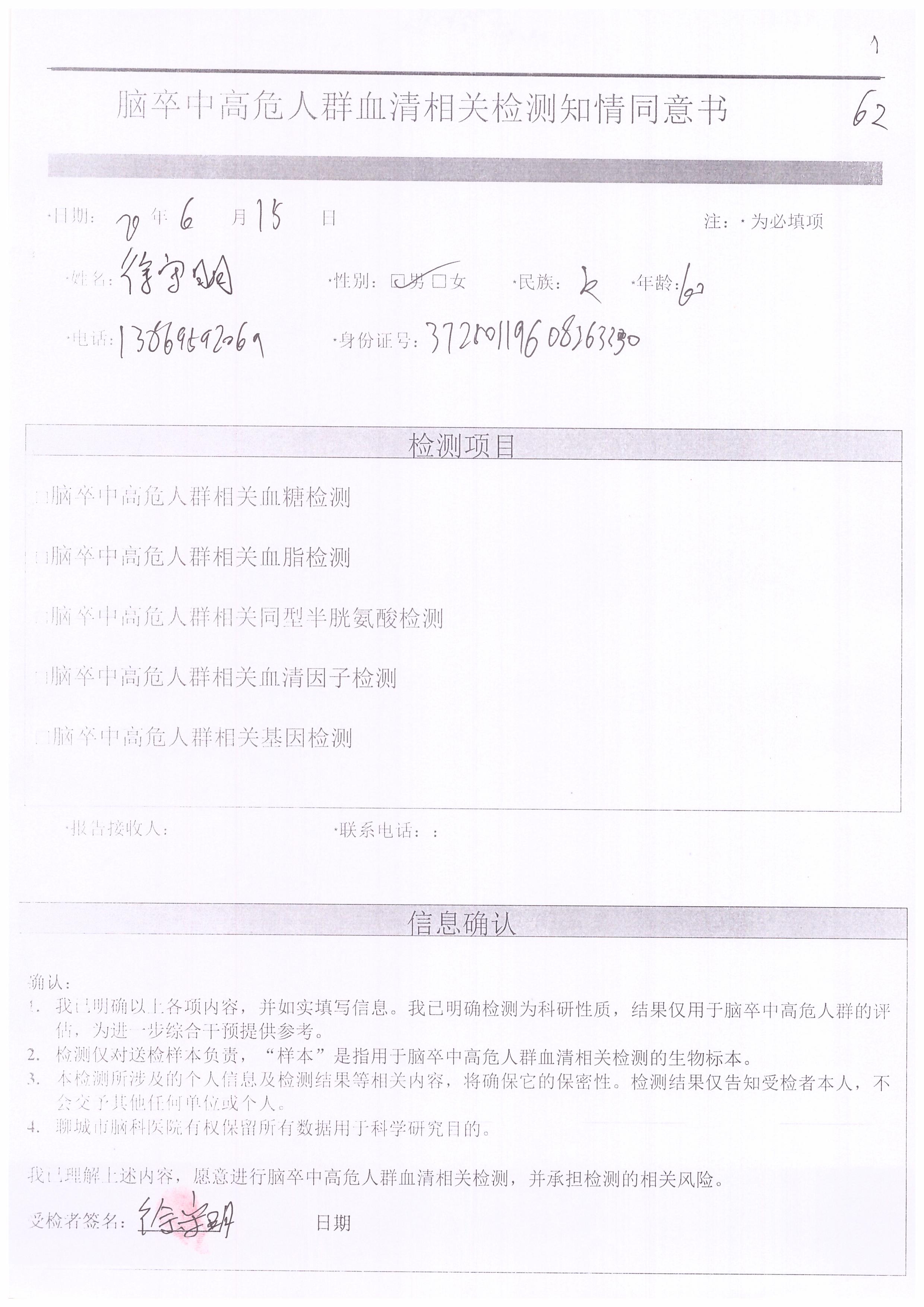

Supplement: Supplementary file 4 — Supplementary file4 (ZIP 25697 KB) [file 10528_2023_10431_MOESM4_ESM.zip › ╓¬╟Θ═1⁄4╥Γ╩Θ2/021 (2).jpg]

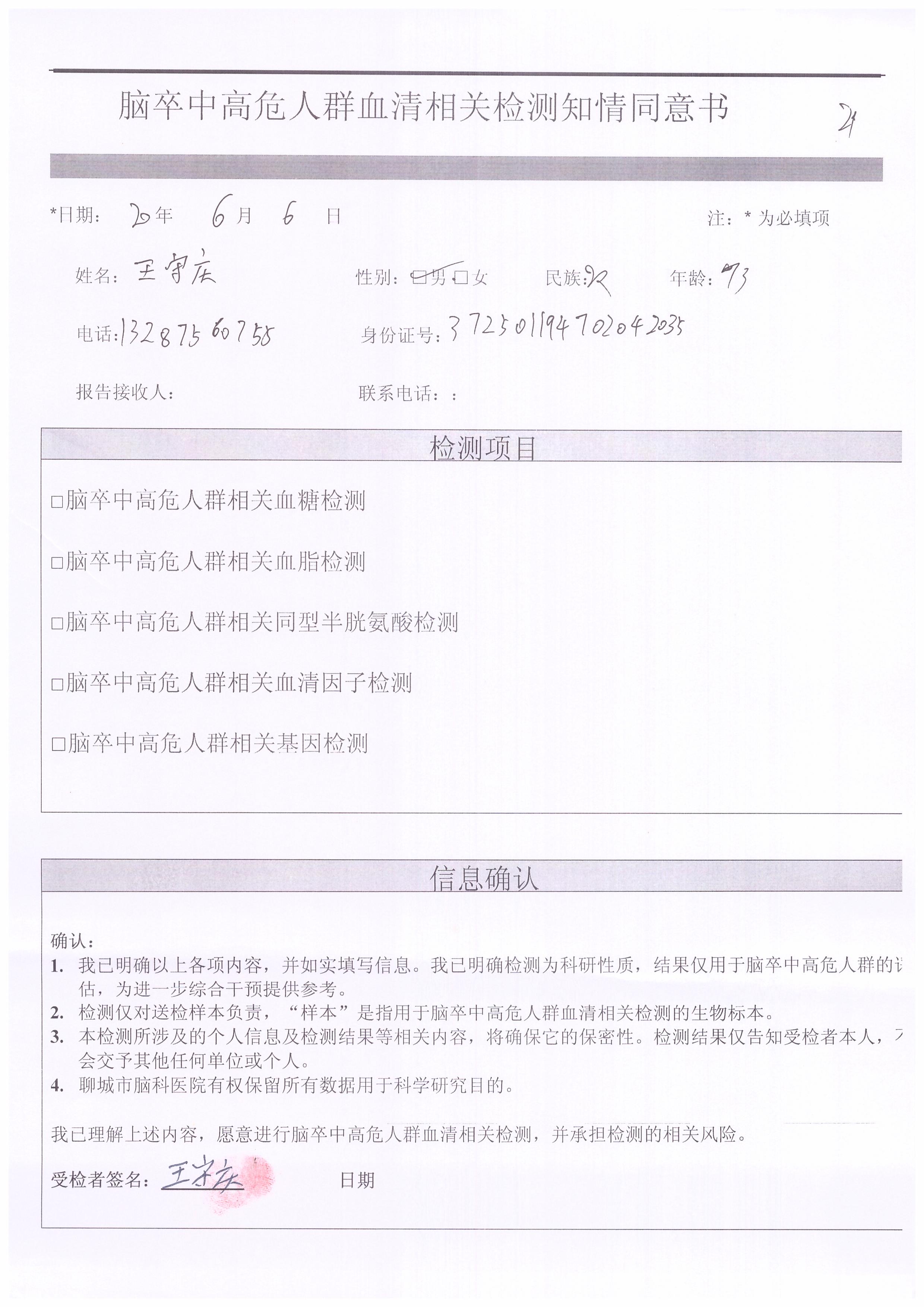

Supplement: Supplementary file 4 — Supplementary file4 (ZIP 25697 KB) [file 10528_2023_10431_MOESM4_ESM.zip › ╓¬╟Θ═1⁄4╥Γ╩Θ2/021.jpg]

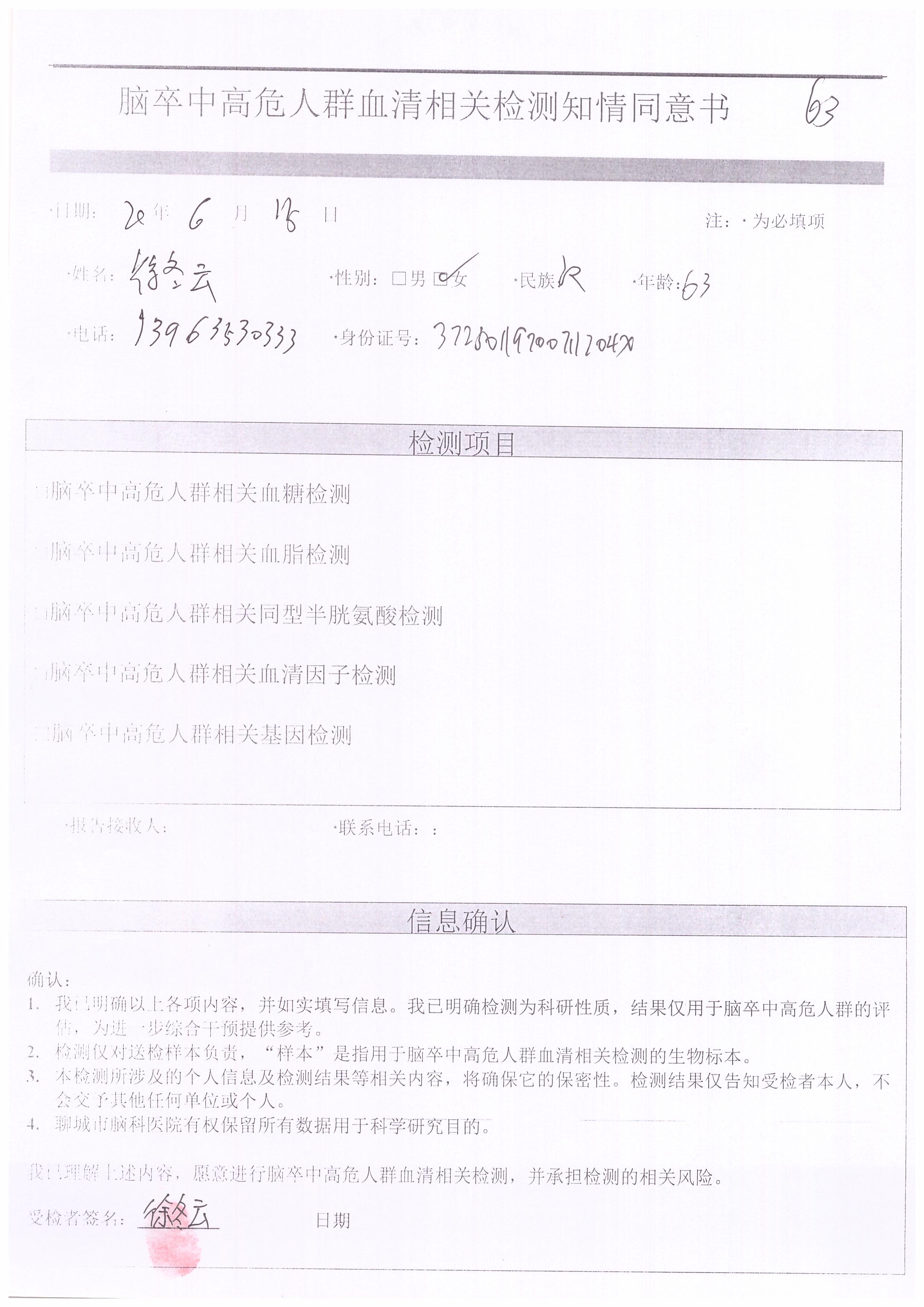

Supplement: Supplementary file 4 — Supplementary file4 (ZIP 25697 KB) [file 10528_2023_10431_MOESM4_ESM.zip › ╓¬╟Θ═1⁄4╥Γ╩Θ2/022 (2).jpg]

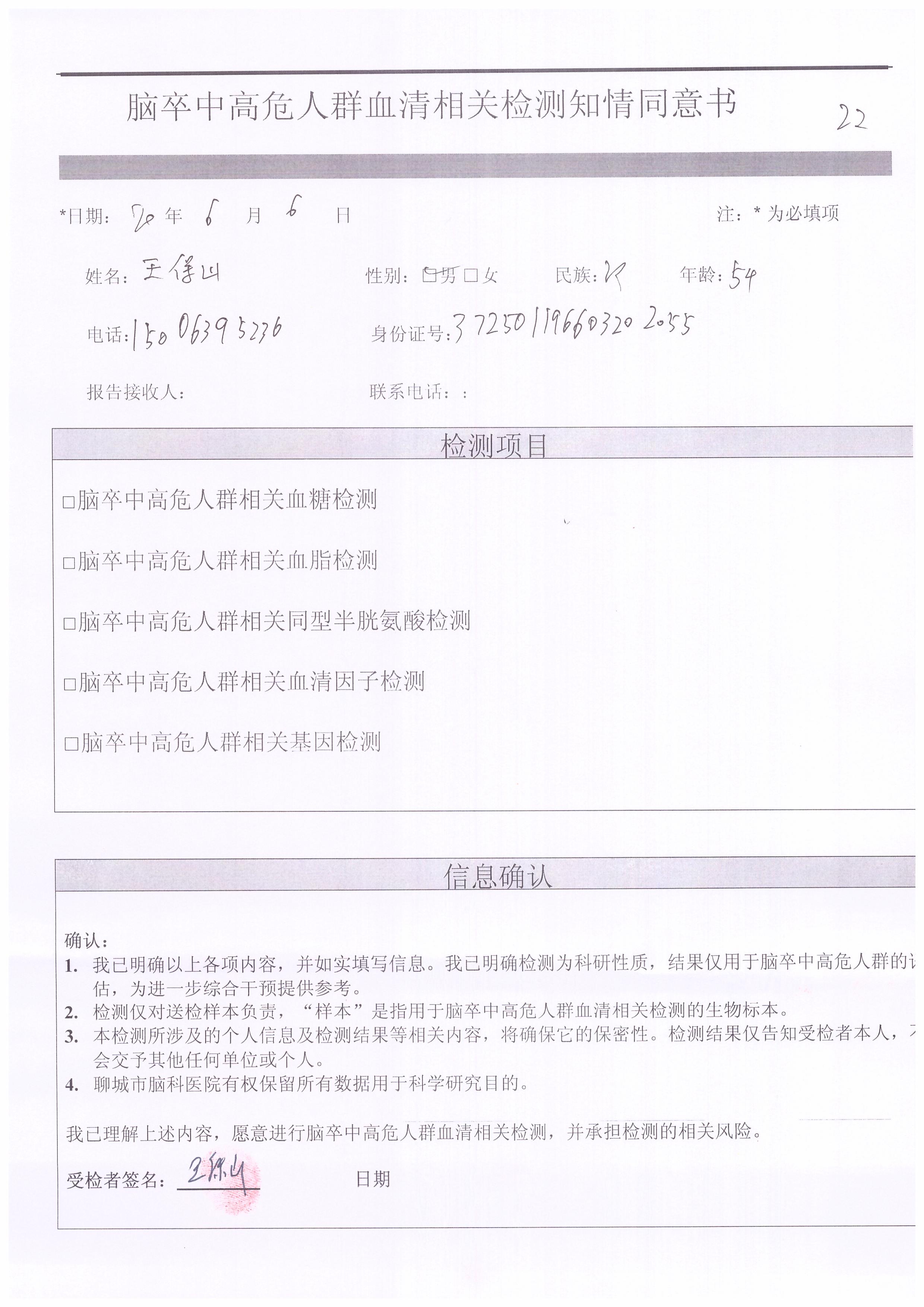

Supplement: Supplementary file 4 — Supplementary file4 (ZIP 25697 KB) [file 10528_2023_10431_MOESM4_ESM.zip › ╓¬╟Θ═1⁄4╥Γ╩Θ2/022.jpg]

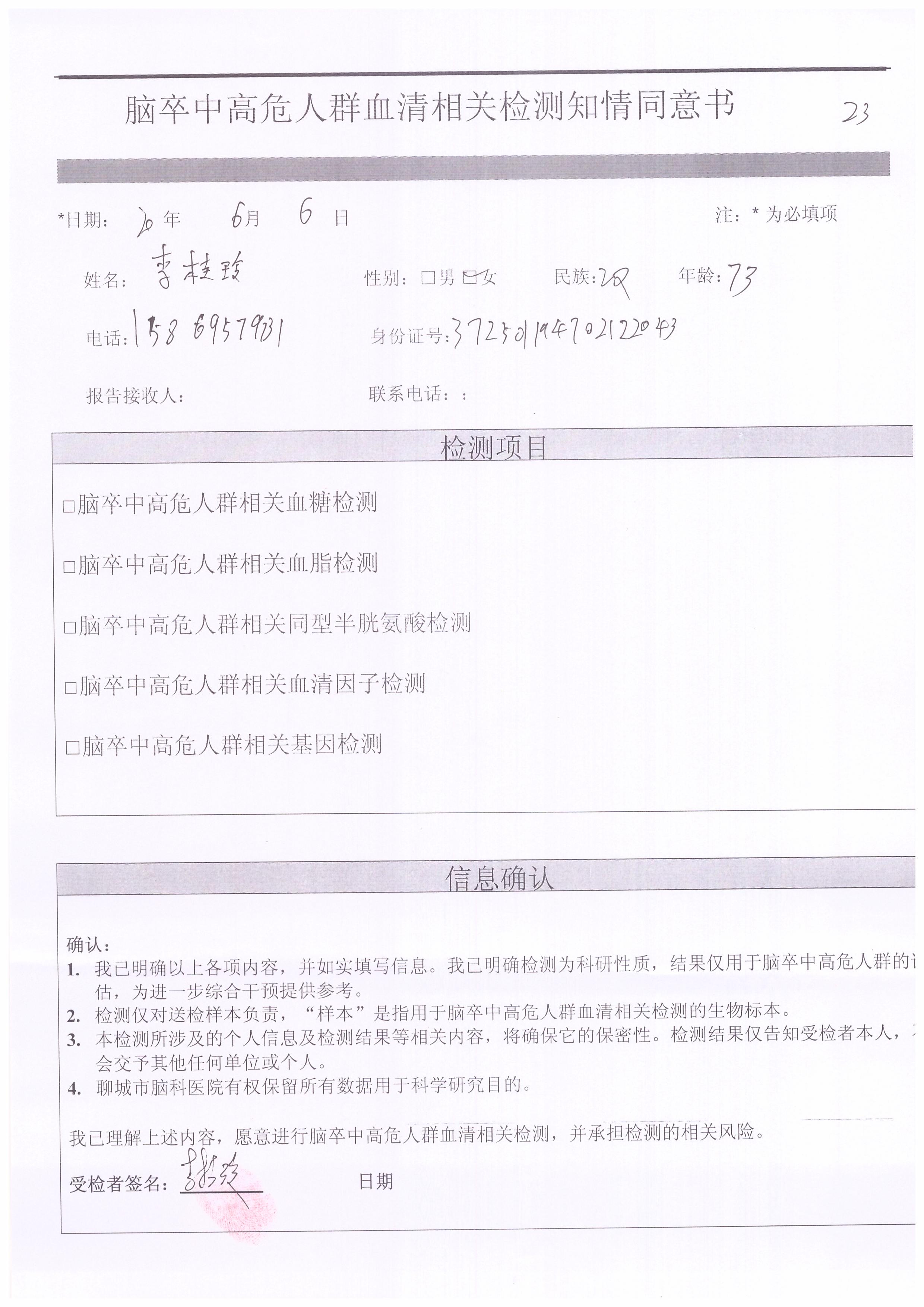

Supplement: Supplementary file 4 — Supplementary file4 (ZIP 25697 KB) [file 10528_2023_10431_MOESM4_ESM.zip › ╓¬╟Θ═1⁄4╥Γ╩Θ2/023.jpg]

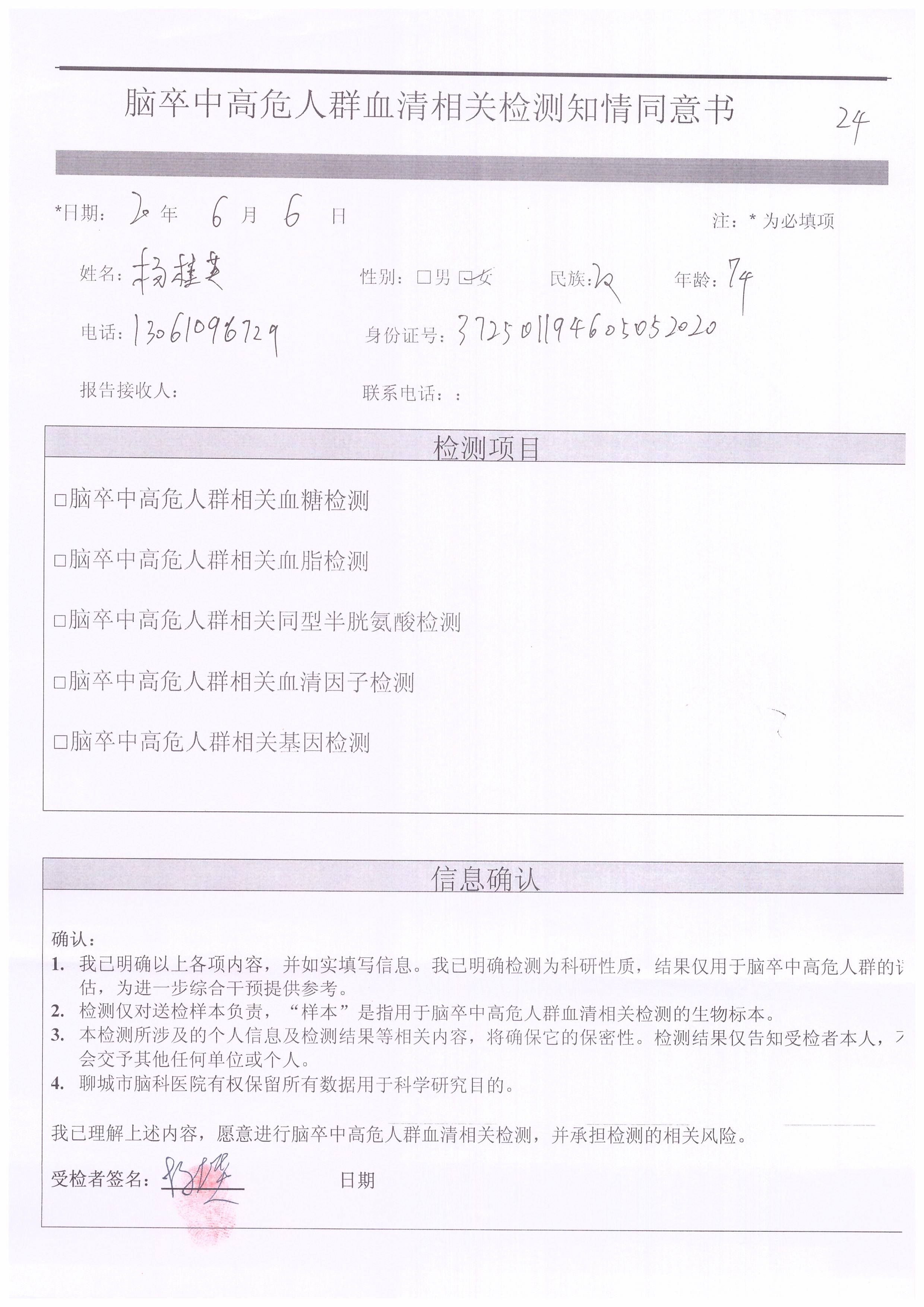

Supplement: Supplementary file 4 — Supplementary file4 (ZIP 25697 KB) [file 10528_2023_10431_MOESM4_ESM.zip › ╓¬╟Θ═1⁄4╥Γ╩Θ2/024.jpg]

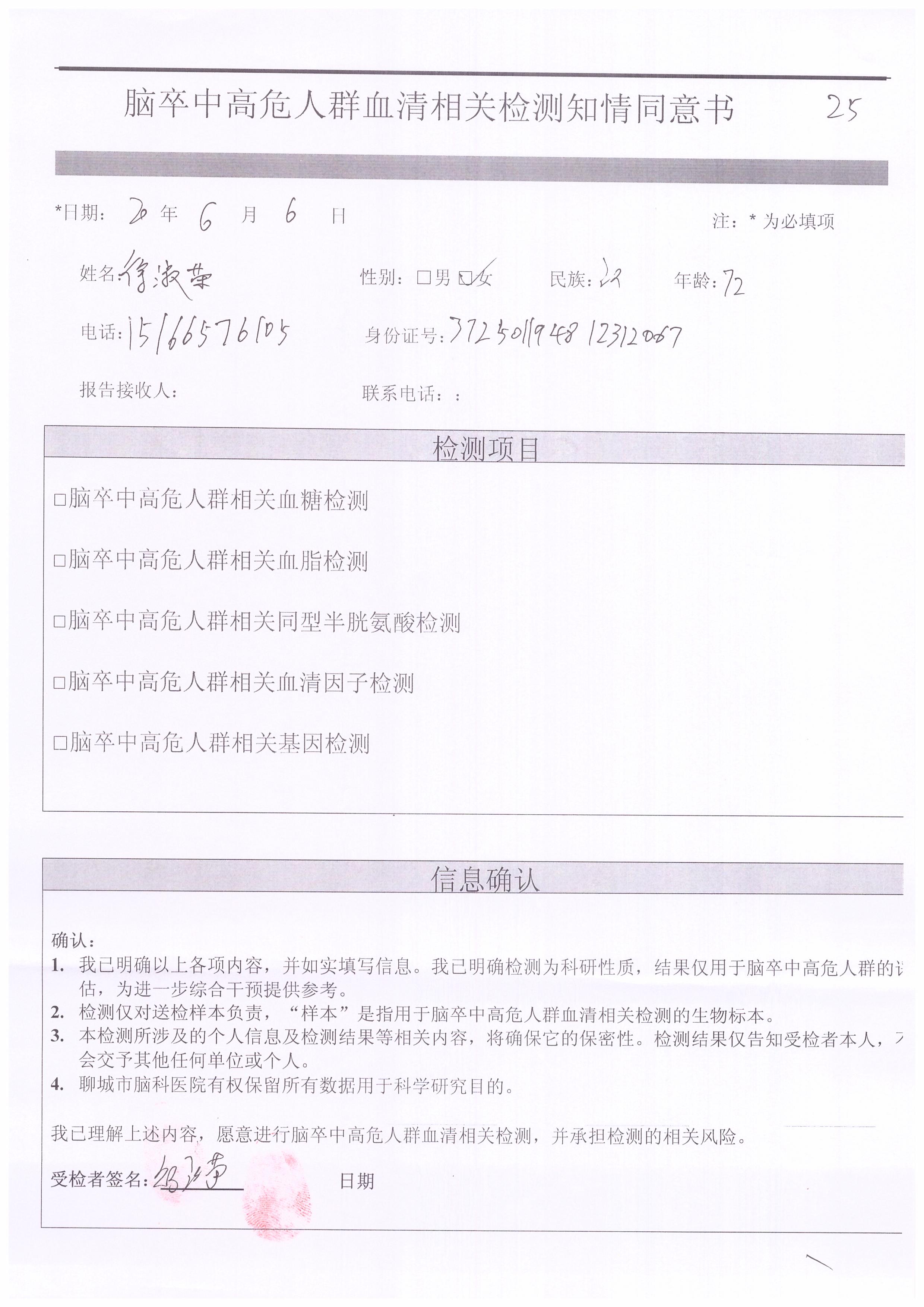

Supplement: Supplementary file 4 — Supplementary file4 (ZIP 25697 KB) [file 10528_2023_10431_MOESM4_ESM.zip › ╓¬╟Θ═1⁄4╥Γ╩Θ2/025.jpg]

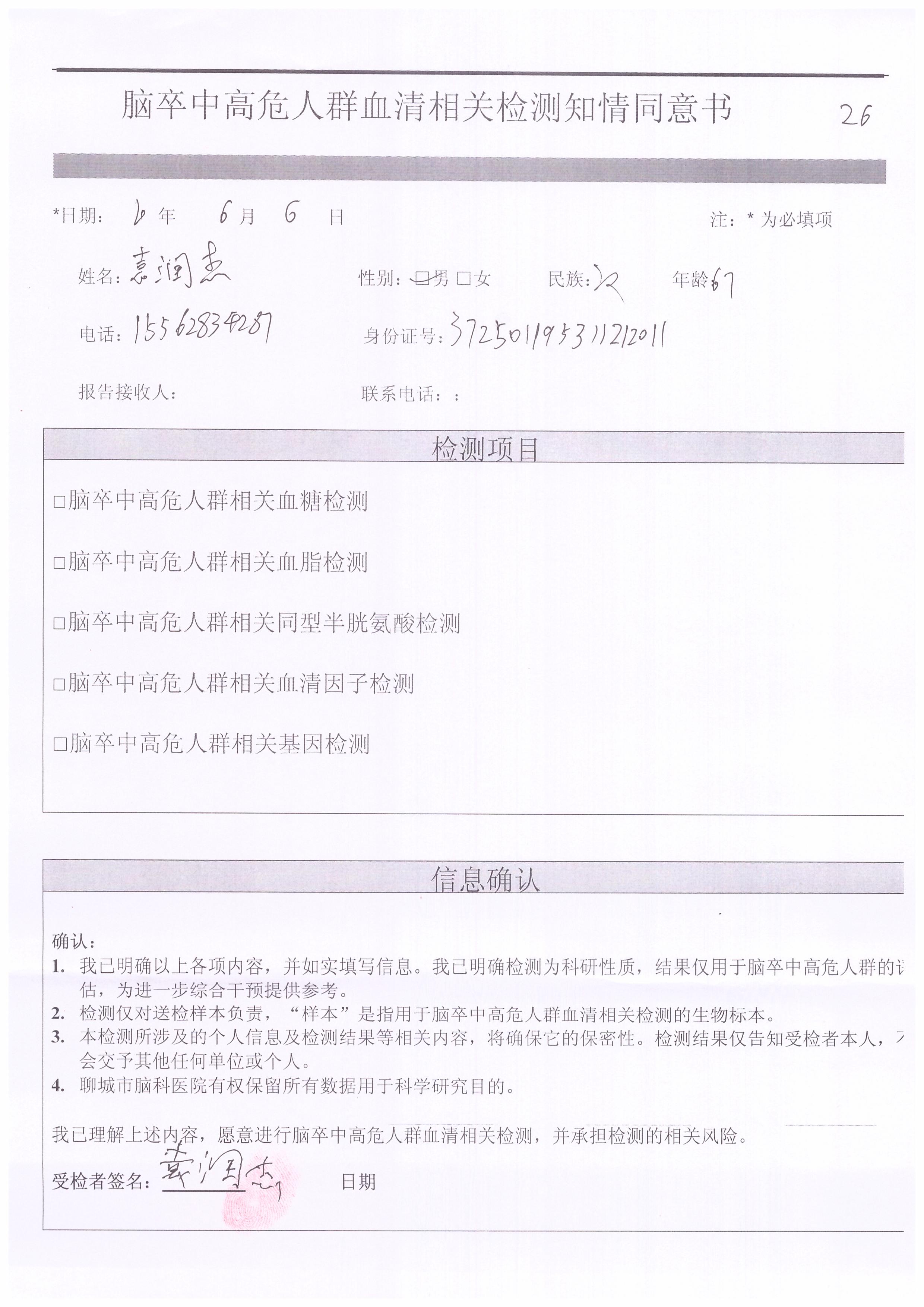

Supplement: Supplementary file 4 — Supplementary file4 (ZIP 25697 KB) [file 10528_2023_10431_MOESM4_ESM.zip › ╓¬╟Θ═1⁄4╥Γ╩Θ2/026.jpg]

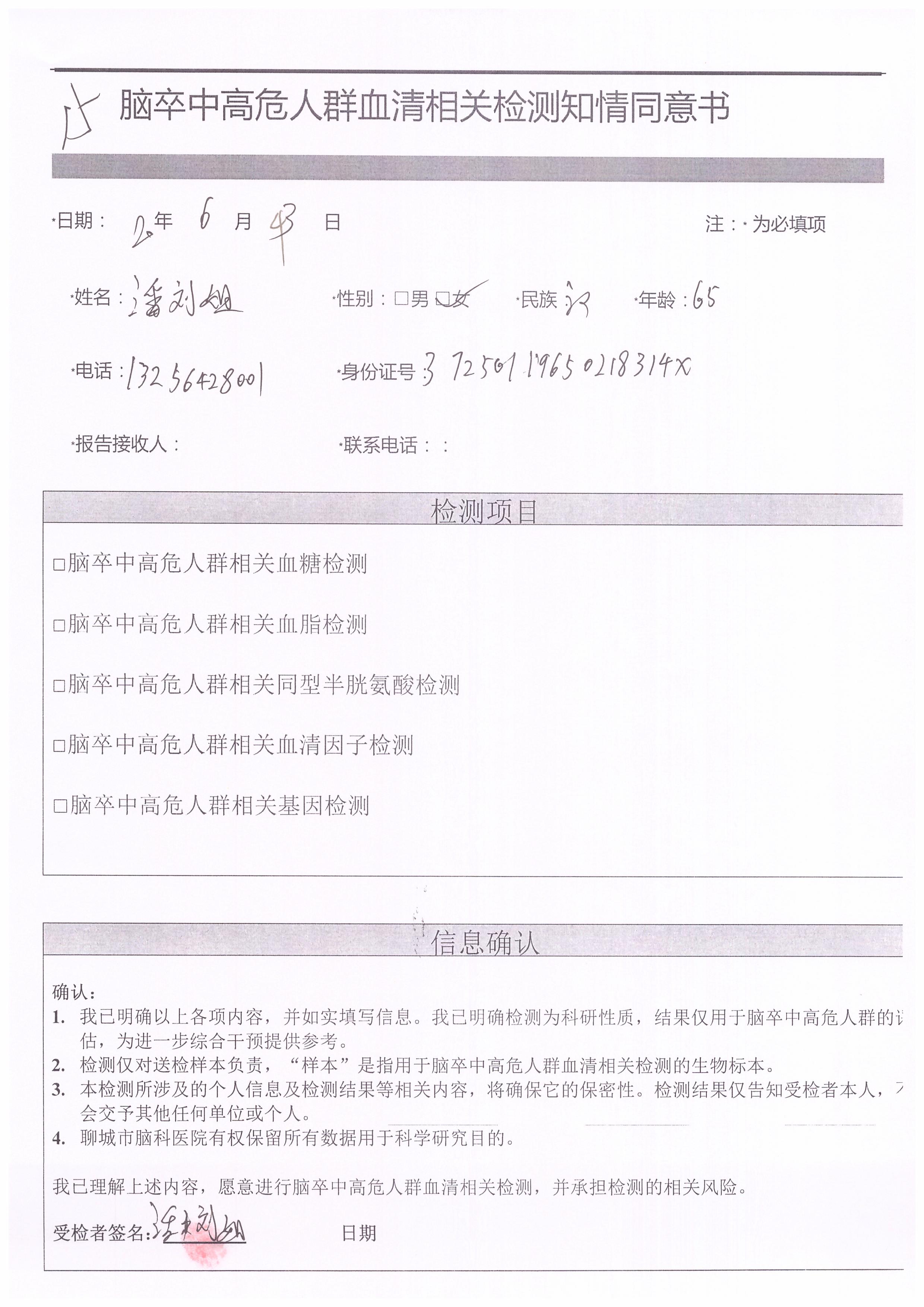

Supplement: Supplementary file 5 — Supplementary file5 (ZIP 24834 KB) [file 10528_2023_10431_MOESM5_ESM.zip › ╓¬╟Θ═1⁄4╥Γ╩Θ3/015.jpg]

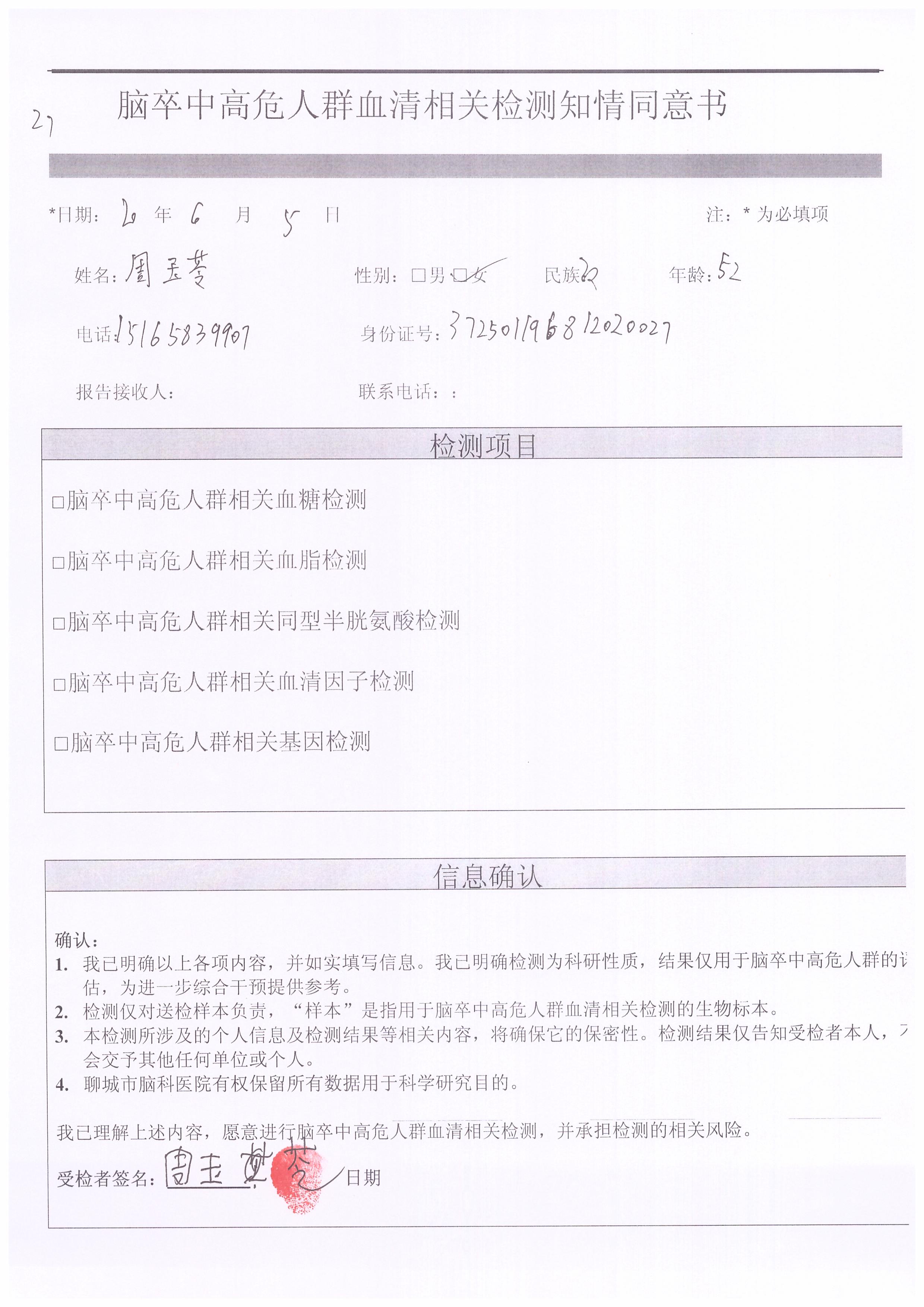

Supplement: Supplementary file 5 — Supplementary file5 (ZIP 24834 KB) [file 10528_2023_10431_MOESM5_ESM.zip › ╓¬╟Θ═1⁄4╥Γ╩Θ3/027.jpg]

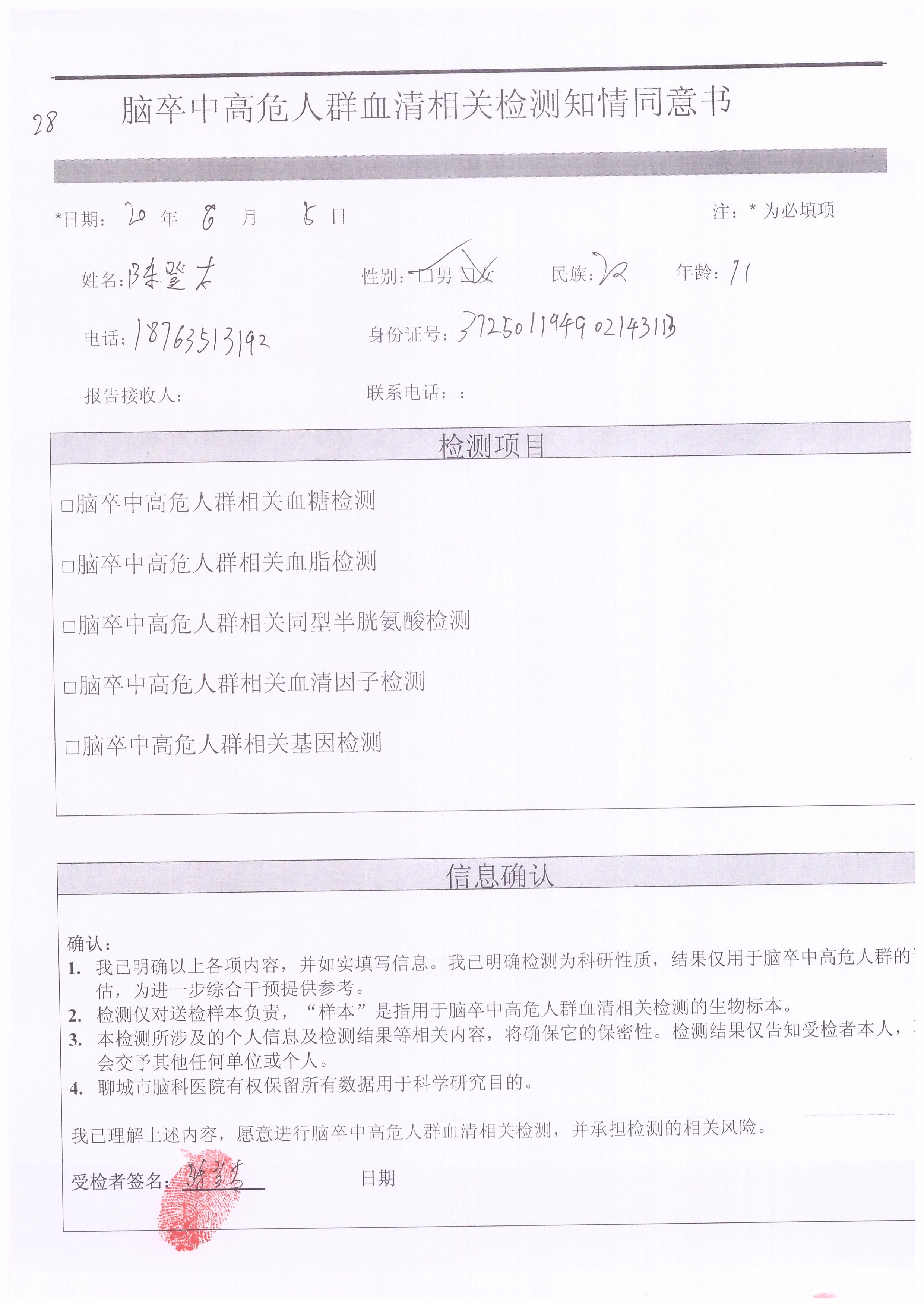

Supplement: Supplementary file 5 — Supplementary file5 (ZIP 24834 KB) [file 10528_2023_10431_MOESM5_ESM.zip › ╓¬╟Θ═1⁄4╥Γ╩Θ3/028.jpg]

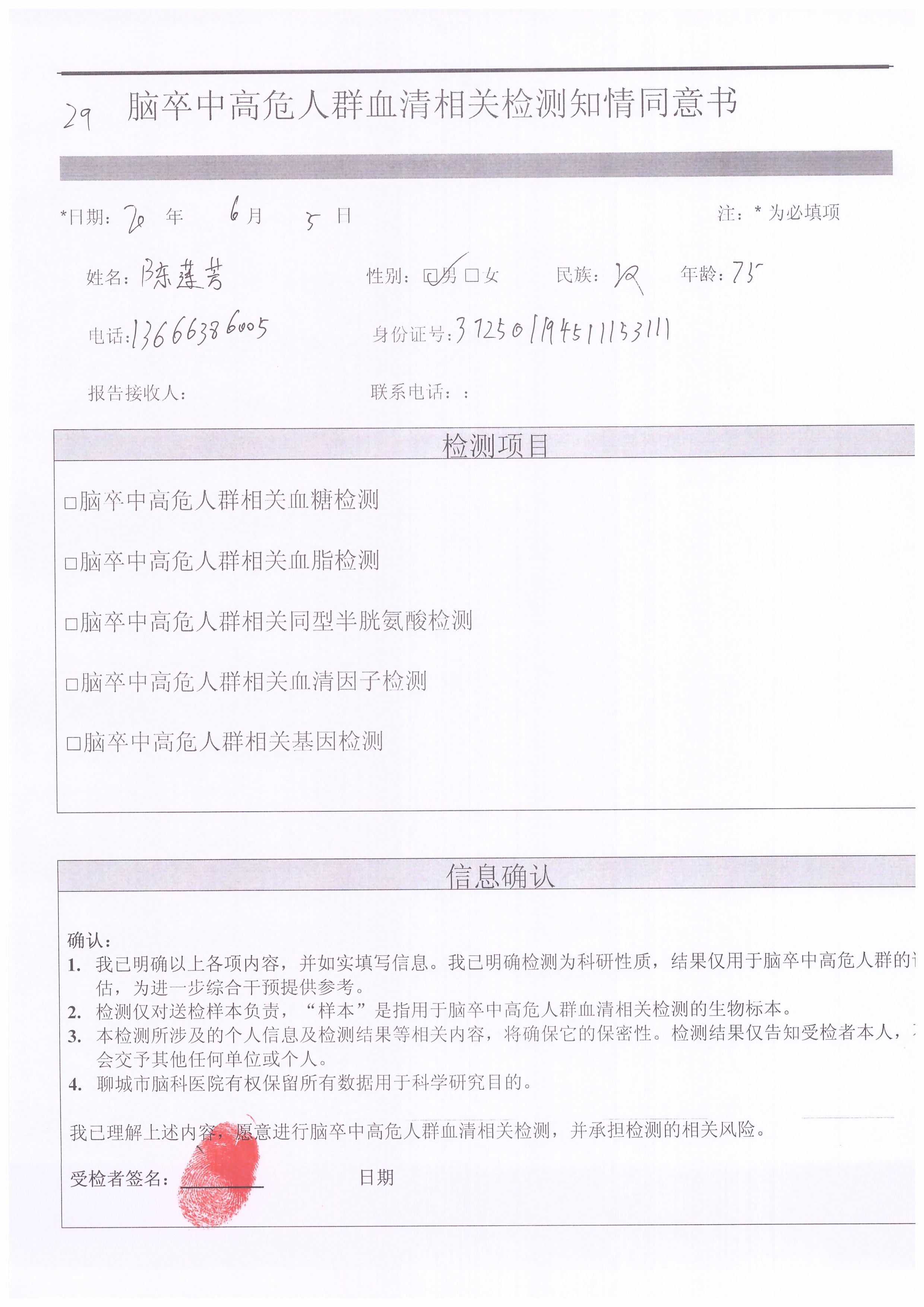

Supplement: Supplementary file 5 — Supplementary file5 (ZIP 24834 KB) [file 10528_2023_10431_MOESM5_ESM.zip › ╓¬╟Θ═1⁄4╥Γ╩Θ3/029.jpg]

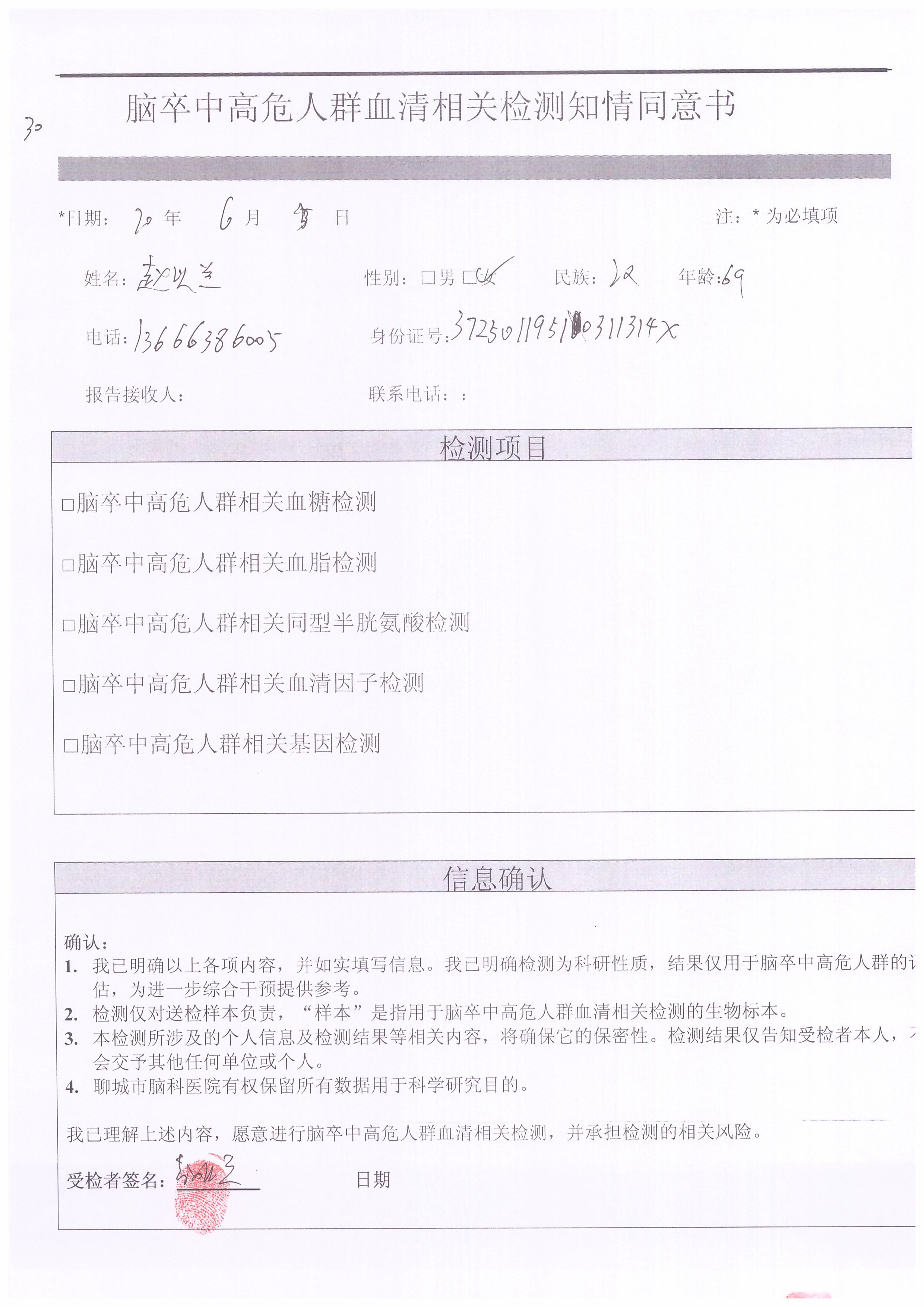

Supplement: Supplementary file 5 — Supplementary file5 (ZIP 24834 KB) [file 10528_2023_10431_MOESM5_ESM.zip › ╓¬╟Θ═1⁄4╥Γ╩Θ3/030 (2).jpg]

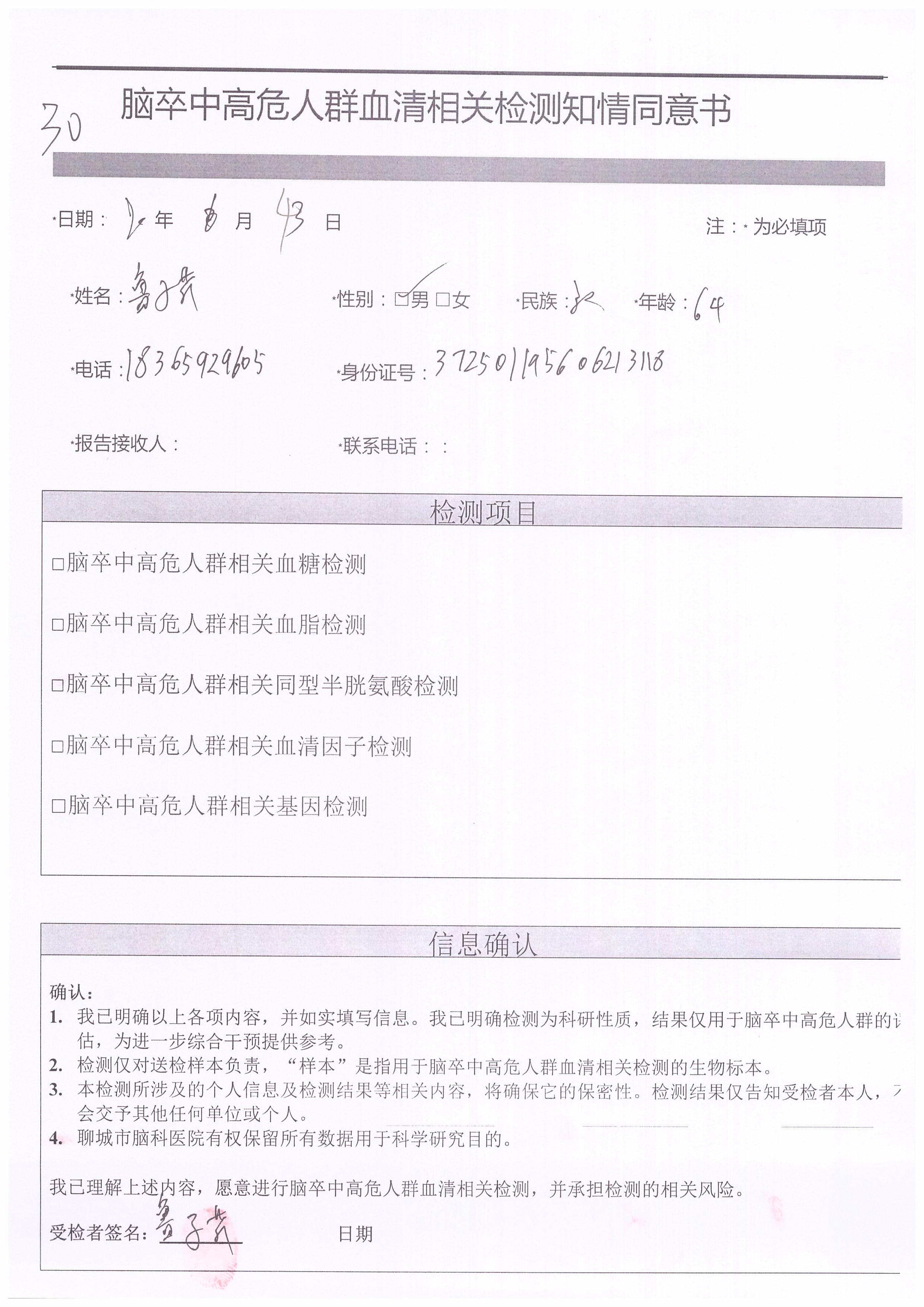

Supplement: Supplementary file 5 — Supplementary file5 (ZIP 24834 KB) [file 10528_2023_10431_MOESM5_ESM.zip › ╓¬╟Θ═1⁄4╥Γ╩Θ3/030.jpg]

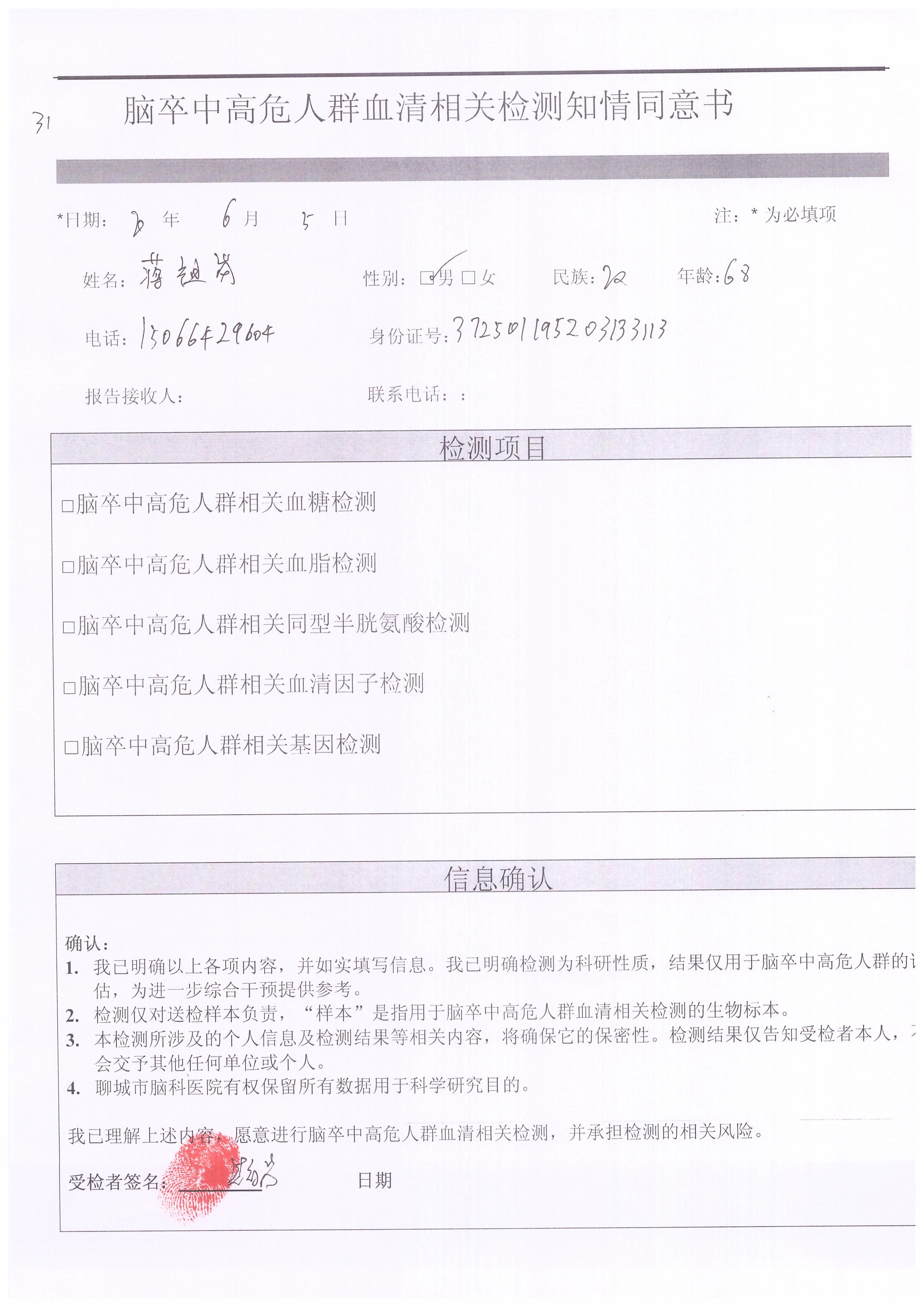

Supplement: Supplementary file 5 — Supplementary file5 (ZIP 24834 KB) [file 10528_2023_10431_MOESM5_ESM.zip › ╓¬╟Θ═1⁄4╥Γ╩Θ3/031.jpg]
