# Supplementary figures and images for: The Association Between Thymidylate Synthase Gene Polymorphisms and the Risk of Ischemic Stroke in Chinese Han Population (part 2 of 6)
Source: Biochem Genet. 2023 Jun 28;62(1):468–84. doi: 10.1007/s10528-023-10431-8 (PMC10901929; doi:10.1007/s10528-023-10431-8)

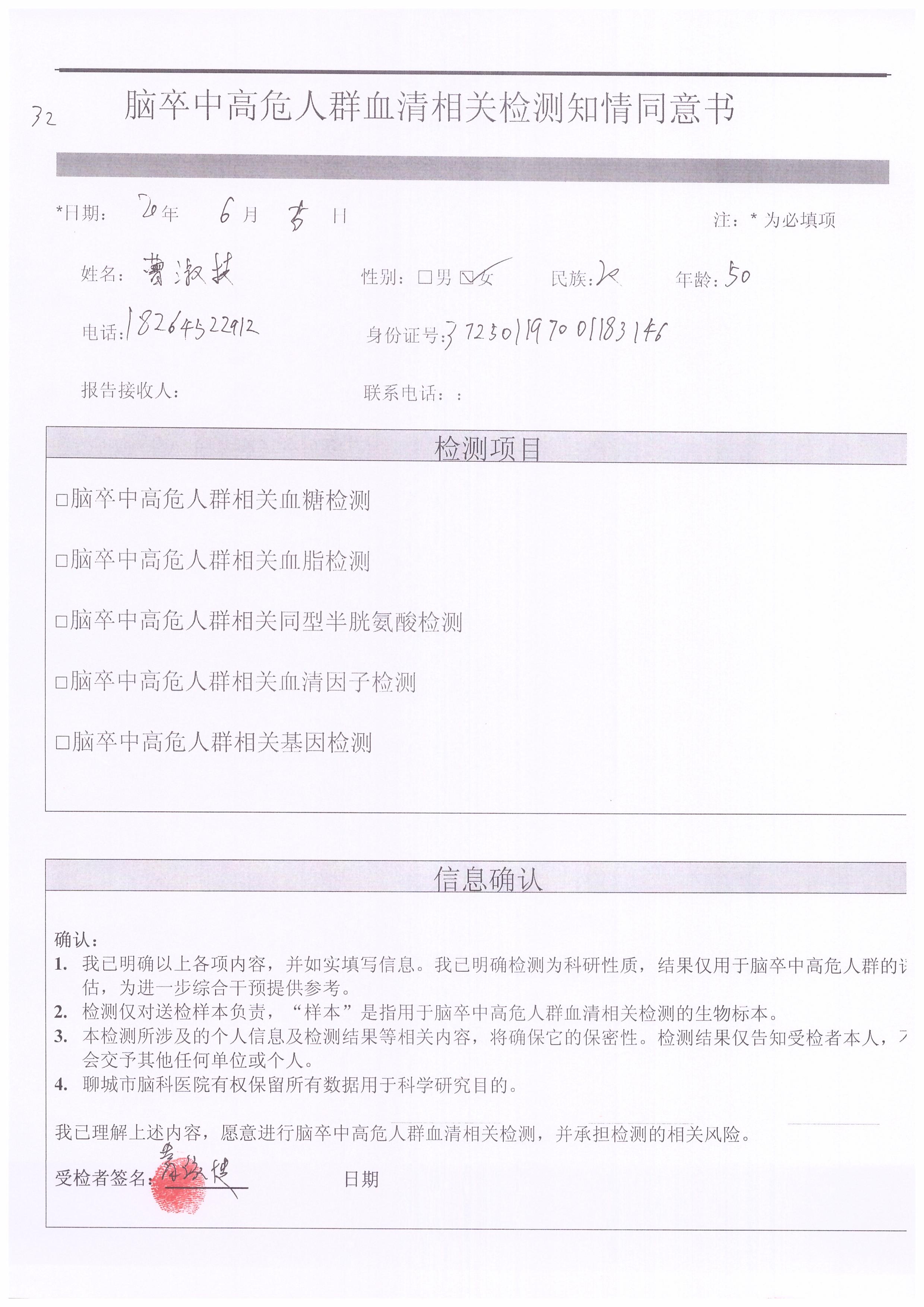

Supplement: Supplementary file 5 — Supplementary file5 (ZIP 24834 KB) [file 10528_2023_10431_MOESM5_ESM.zip › ╓¬╟Θ═1⁄4╥Γ╩Θ3/032.jpg]

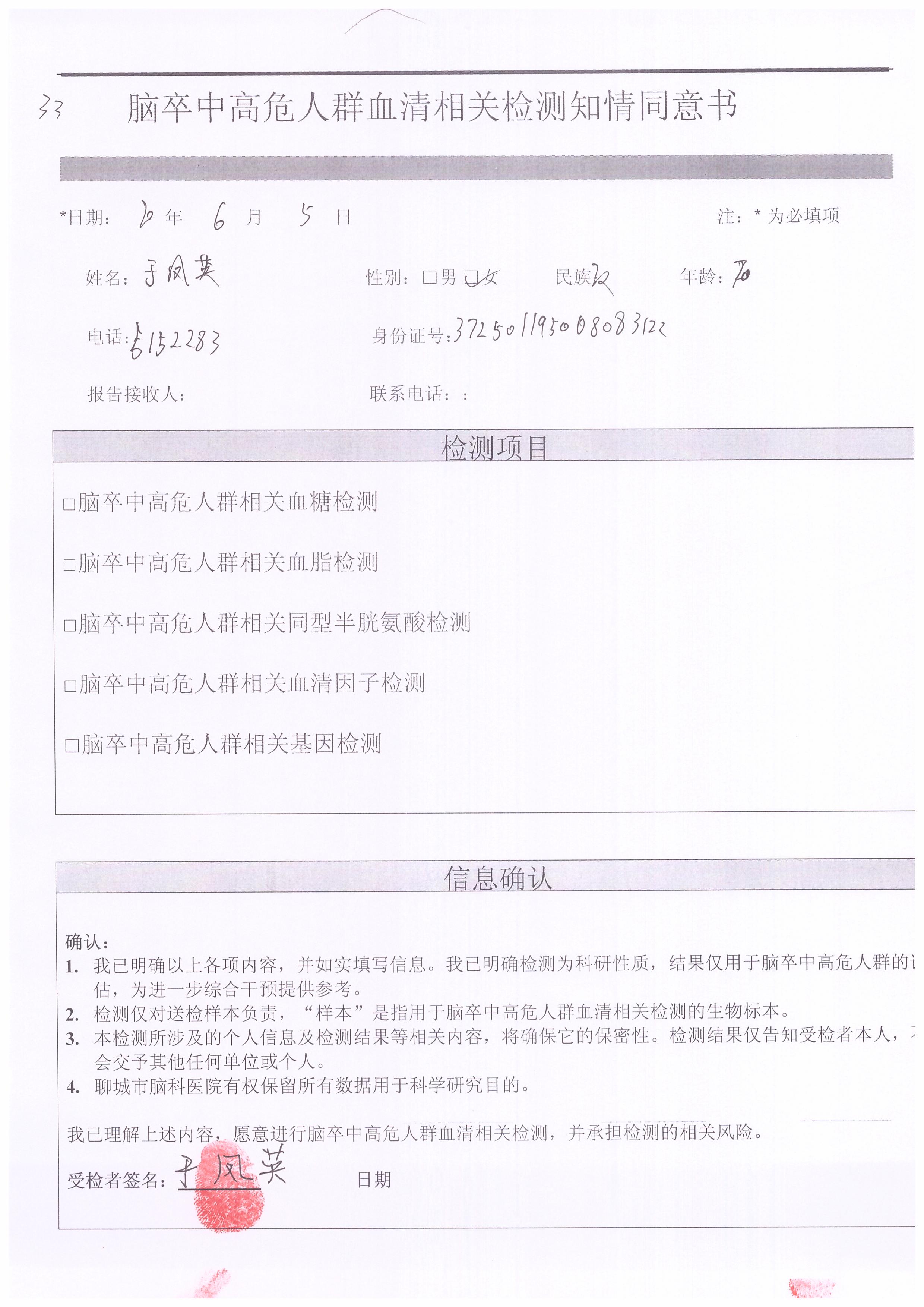

Supplement: Supplementary file 5 — Supplementary file5 (ZIP 24834 KB) [file 10528_2023_10431_MOESM5_ESM.zip › ╓¬╟Θ═1⁄4╥Γ╩Θ3/033.jpg]

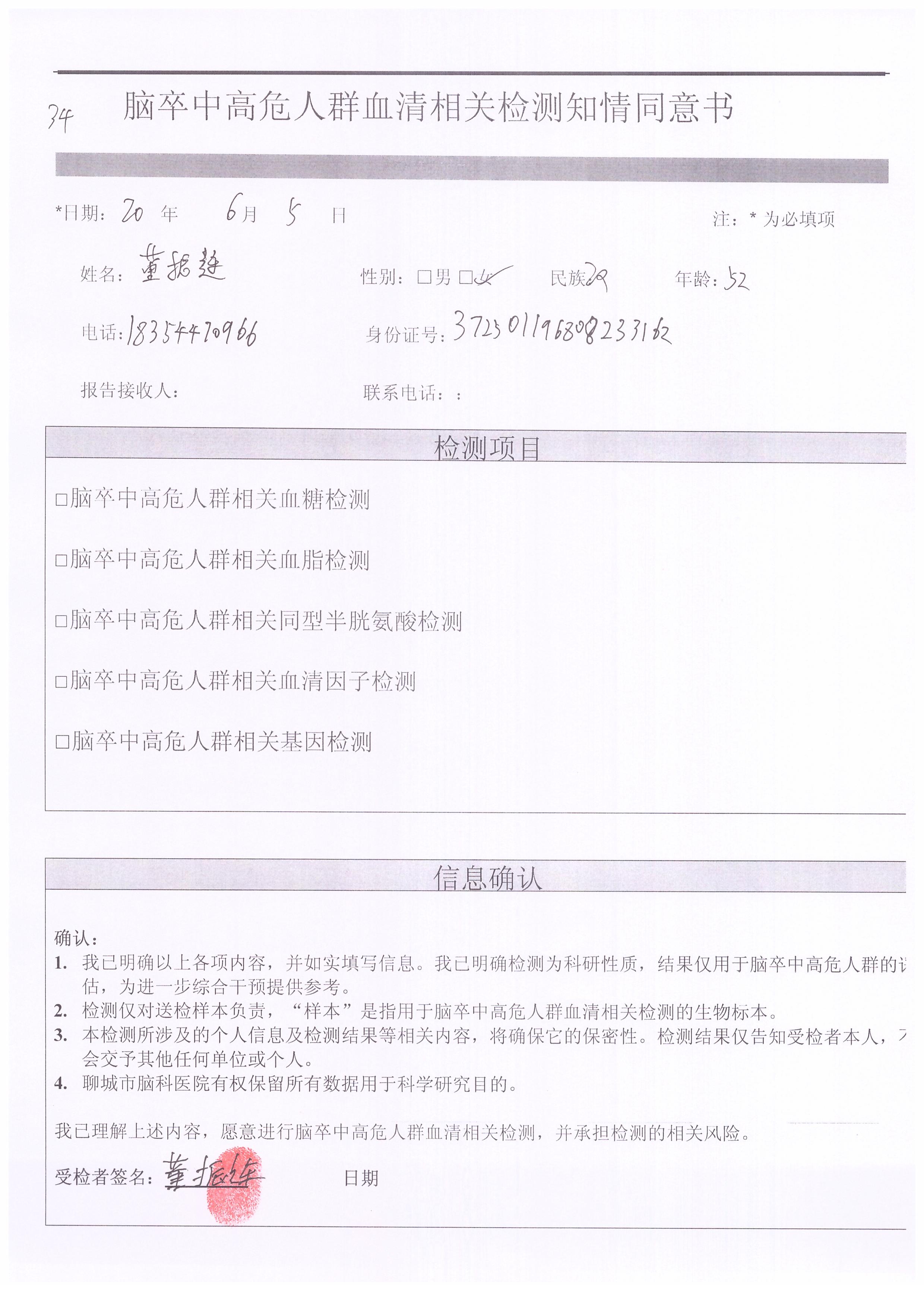

Supplement: Supplementary file 5 — Supplementary file5 (ZIP 24834 KB) [file 10528_2023_10431_MOESM5_ESM.zip › ╓¬╟Θ═1⁄4╥Γ╩Θ3/034.jpg]

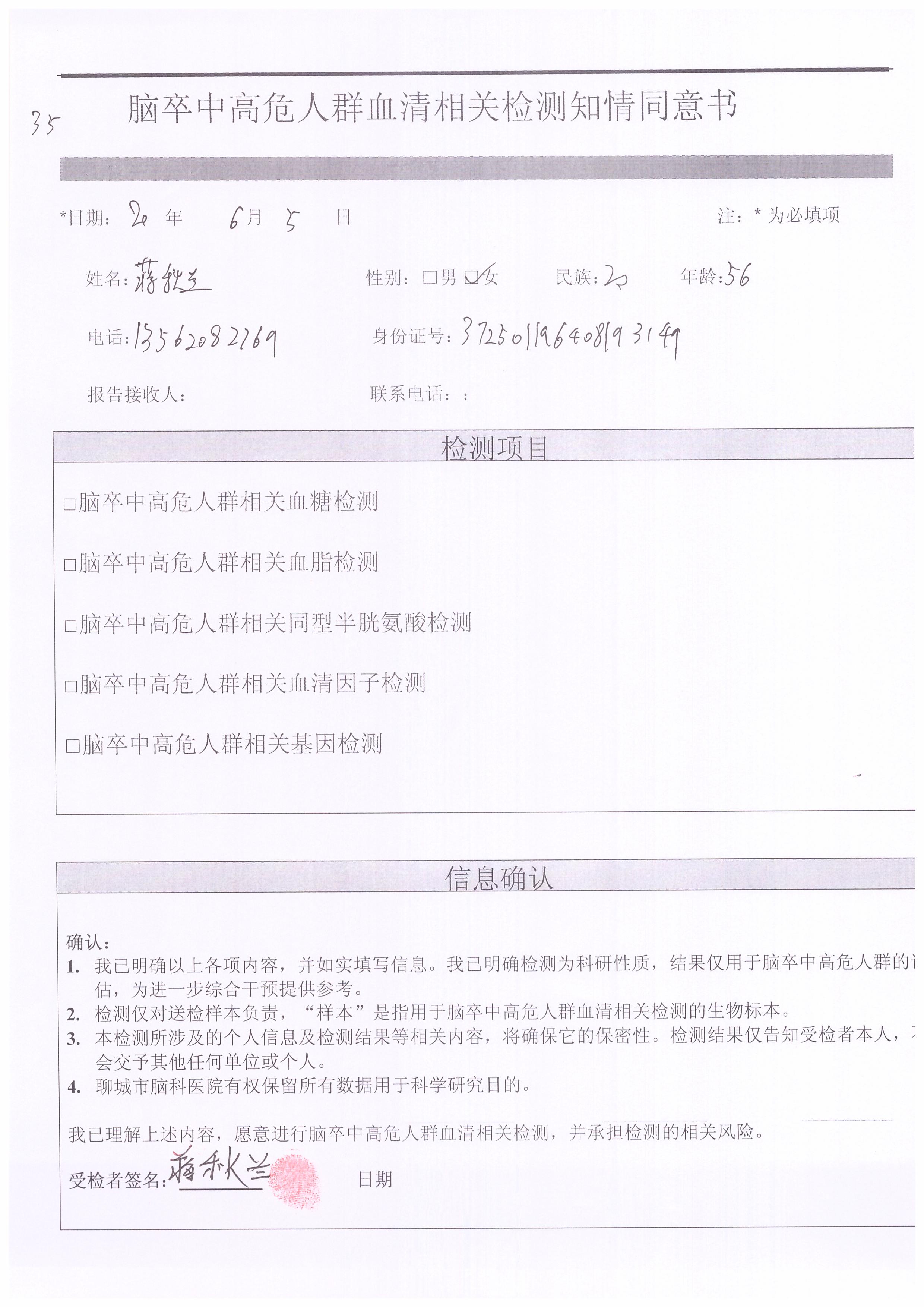

Supplement: Supplementary file 5 — Supplementary file5 (ZIP 24834 KB) [file 10528_2023_10431_MOESM5_ESM.zip › ╓¬╟Θ═1⁄4╥Γ╩Θ3/035.jpg]

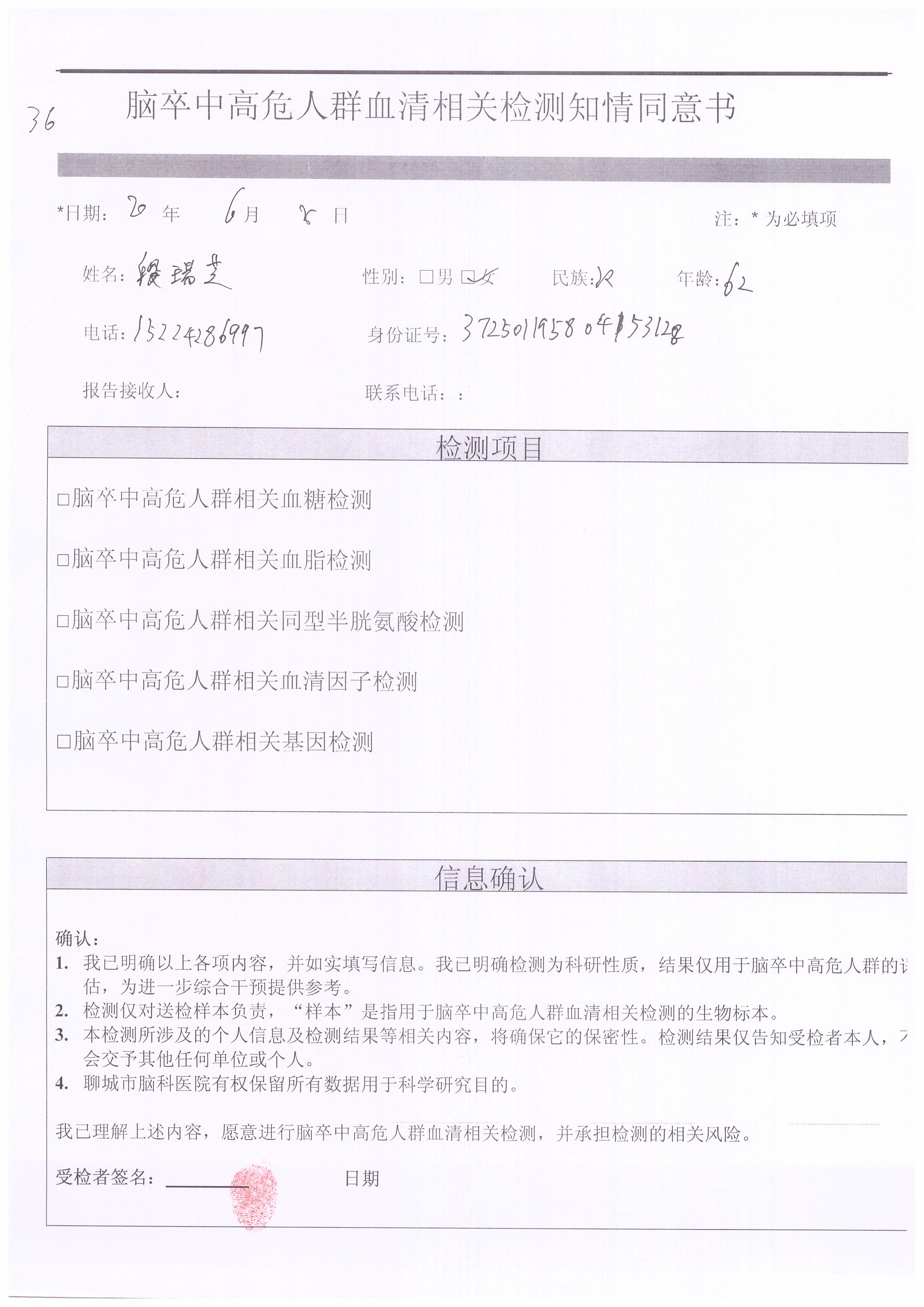

Supplement: Supplementary file 5 — Supplementary file5 (ZIP 24834 KB) [file 10528_2023_10431_MOESM5_ESM.zip › ╓¬╟Θ═1⁄4╥Γ╩Θ3/036.jpg]

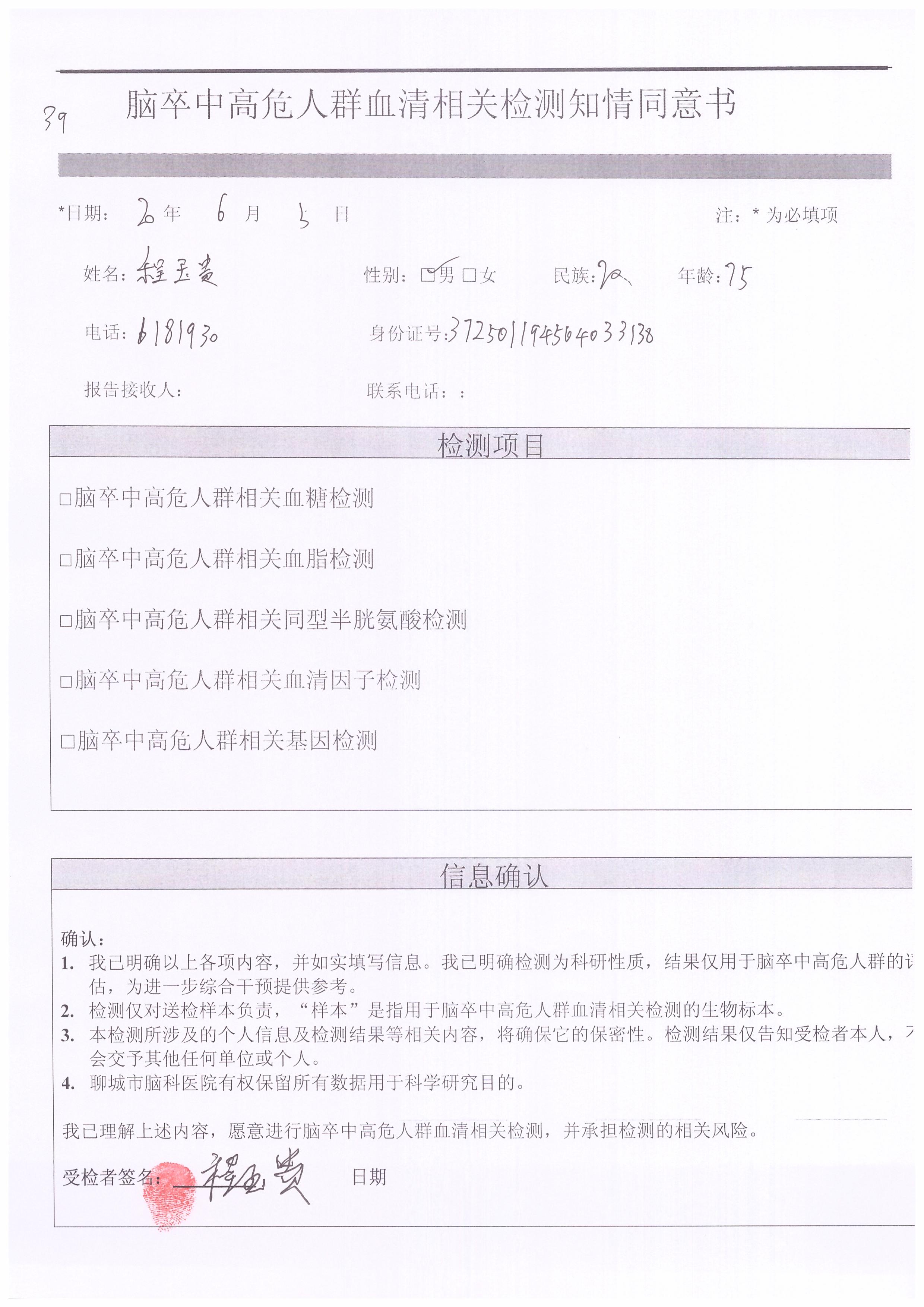

Supplement: Supplementary file 5 — Supplementary file5 (ZIP 24834 KB) [file 10528_2023_10431_MOESM5_ESM.zip › ╓¬╟Θ═1⁄4╥Γ╩Θ3/039.jpg]

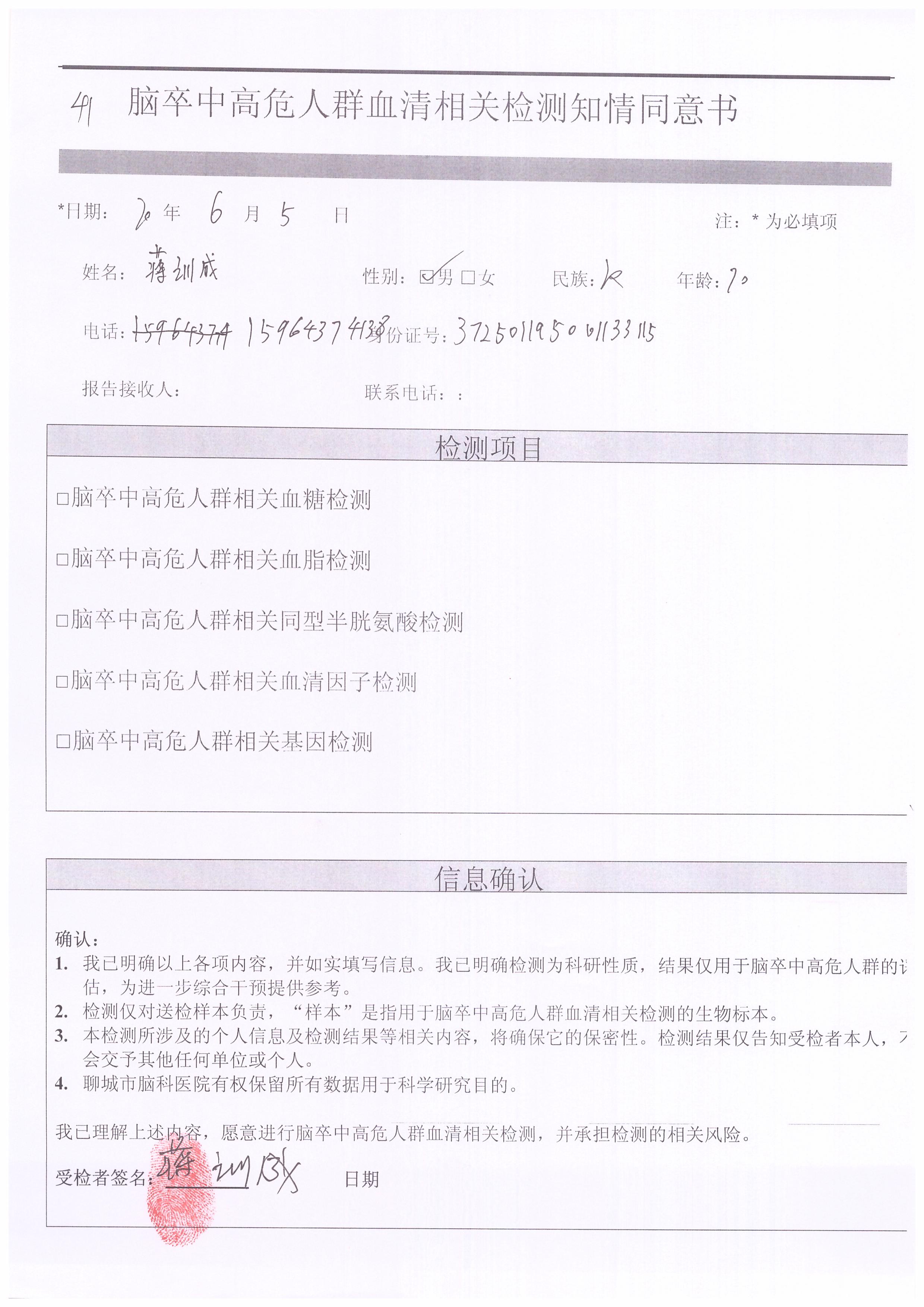

Supplement: Supplementary file 5 — Supplementary file5 (ZIP 24834 KB) [file 10528_2023_10431_MOESM5_ESM.zip › ╓¬╟Θ═1⁄4╥Γ╩Θ3/041 (2).jpg]

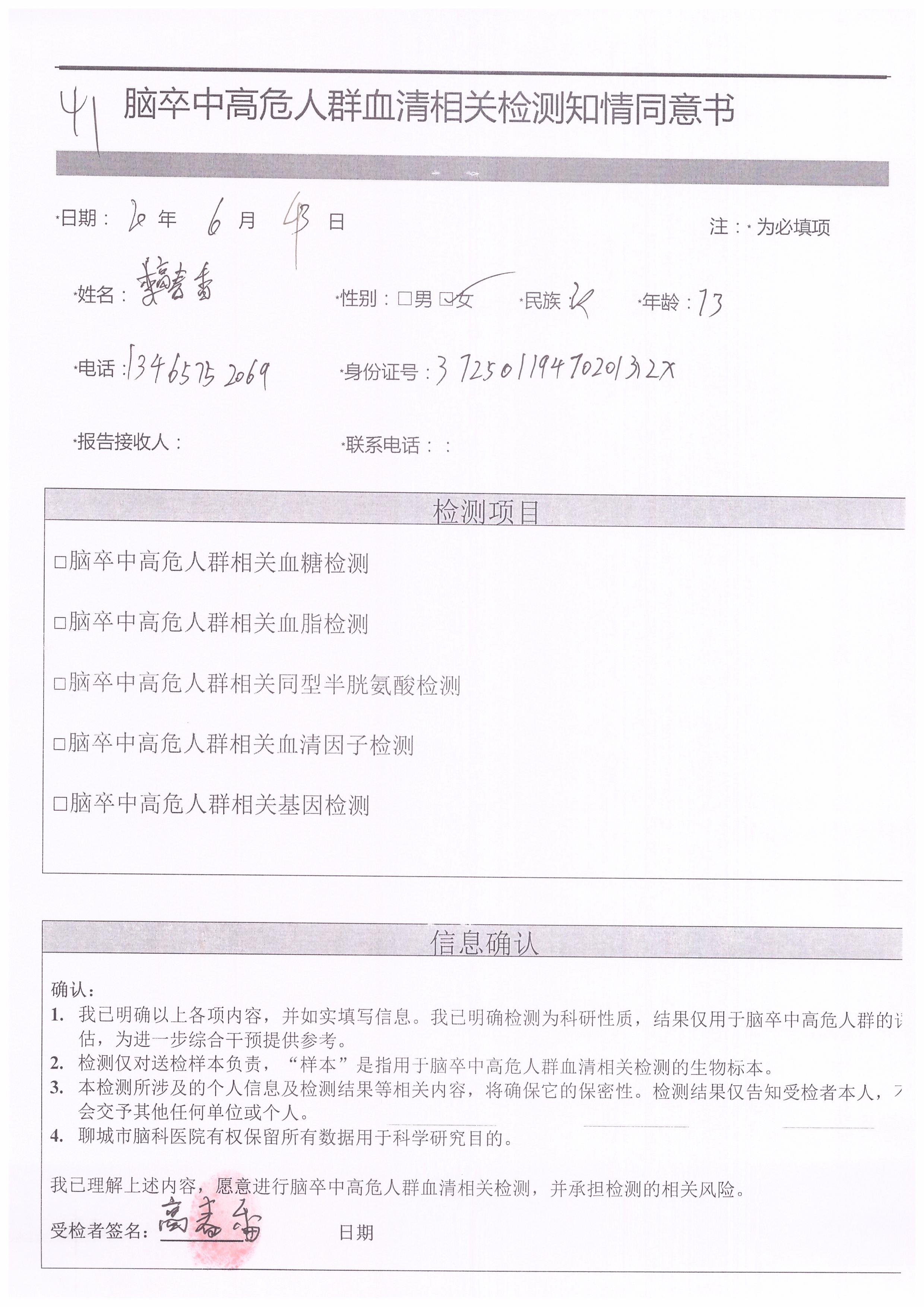

Supplement: Supplementary file 5 — Supplementary file5 (ZIP 24834 KB) [file 10528_2023_10431_MOESM5_ESM.zip › ╓¬╟Θ═1⁄4╥Γ╩Θ3/041.jpg]

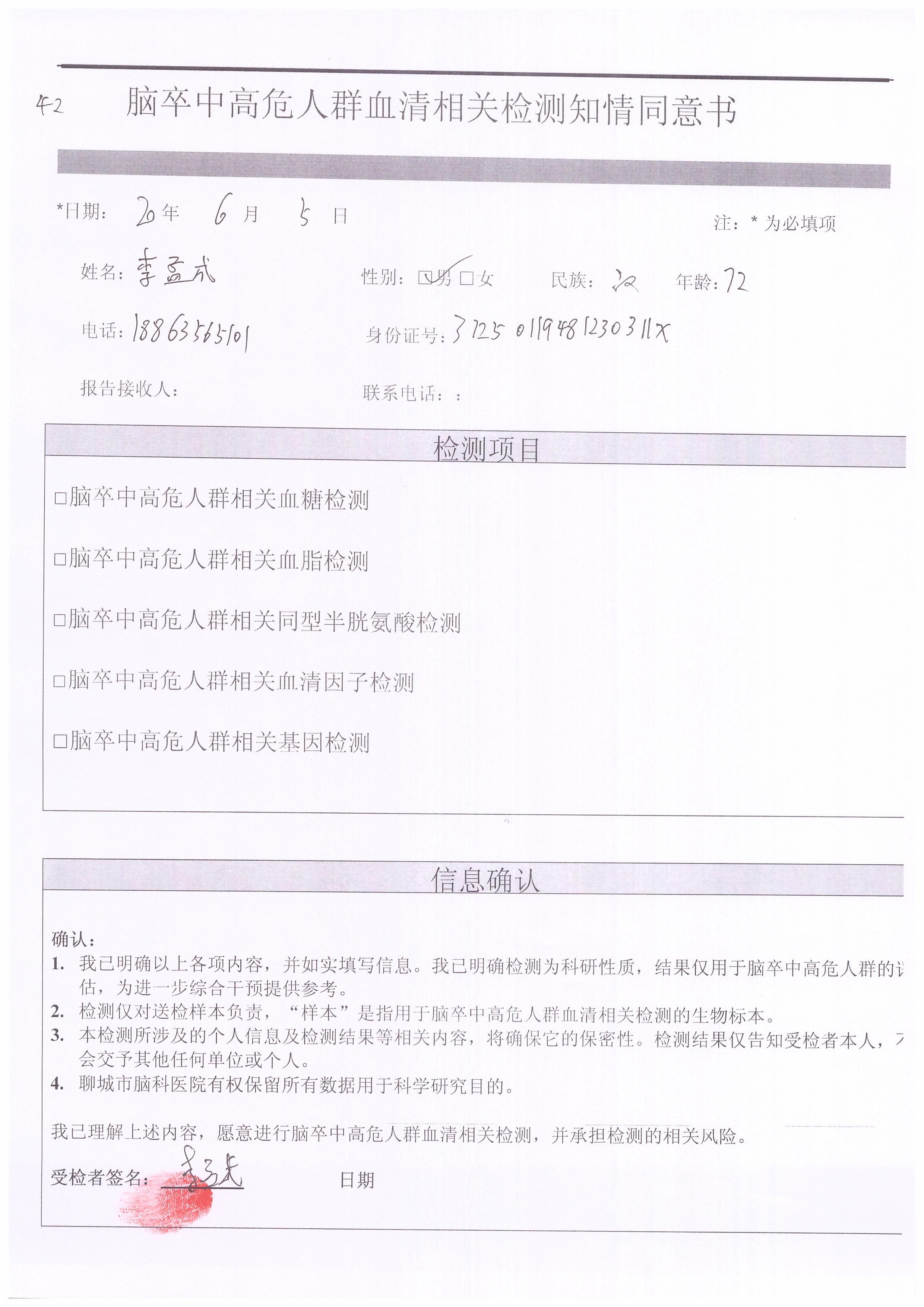

Supplement: Supplementary file 5 — Supplementary file5 (ZIP 24834 KB) [file 10528_2023_10431_MOESM5_ESM.zip › ╓¬╟Θ═1⁄4╥Γ╩Θ3/042.jpg]

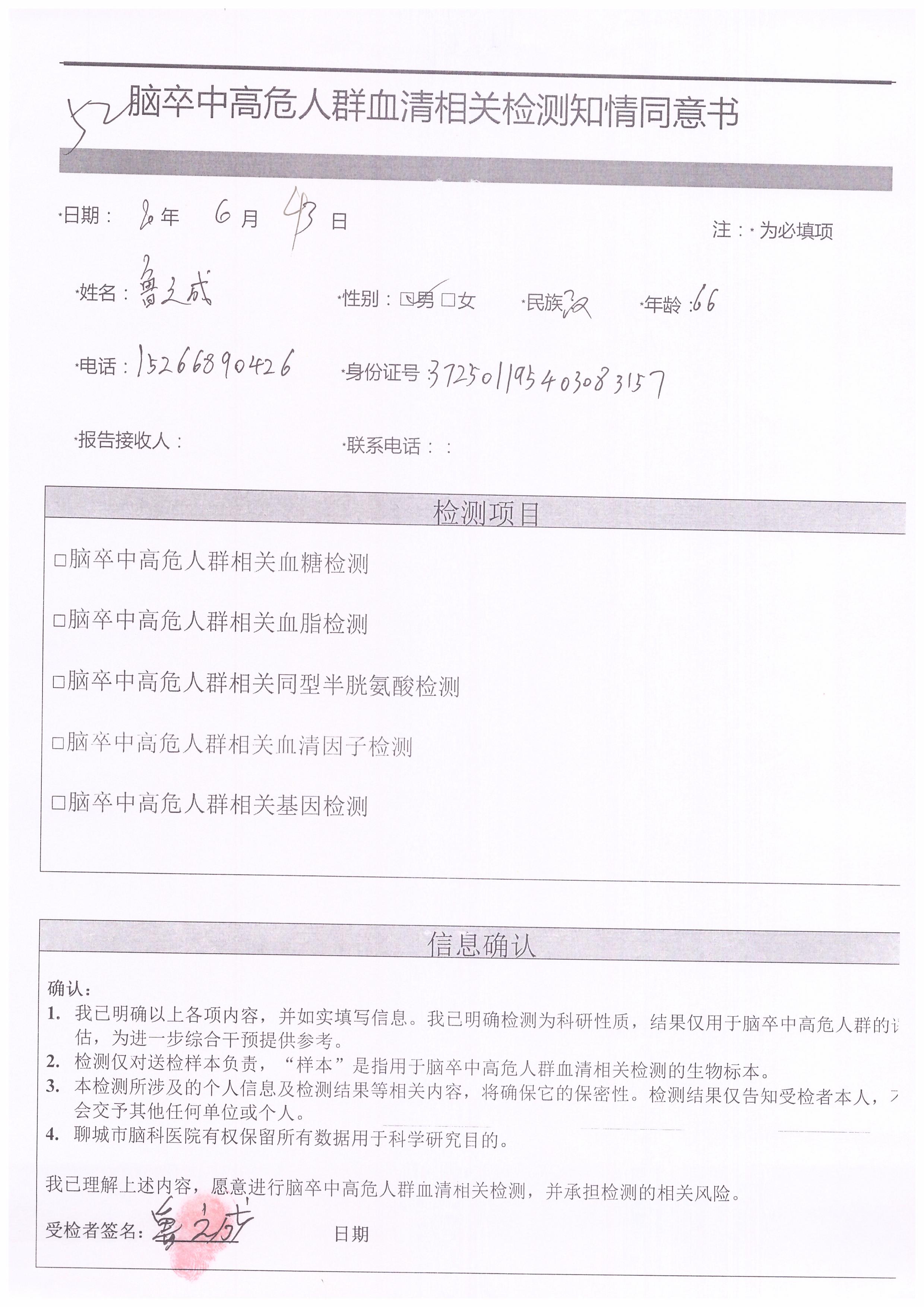

Supplement: Supplementary file 5 — Supplementary file5 (ZIP 24834 KB) [file 10528_2023_10431_MOESM5_ESM.zip › ╓¬╟Θ═1⁄4╥Γ╩Θ3/052.jpg]

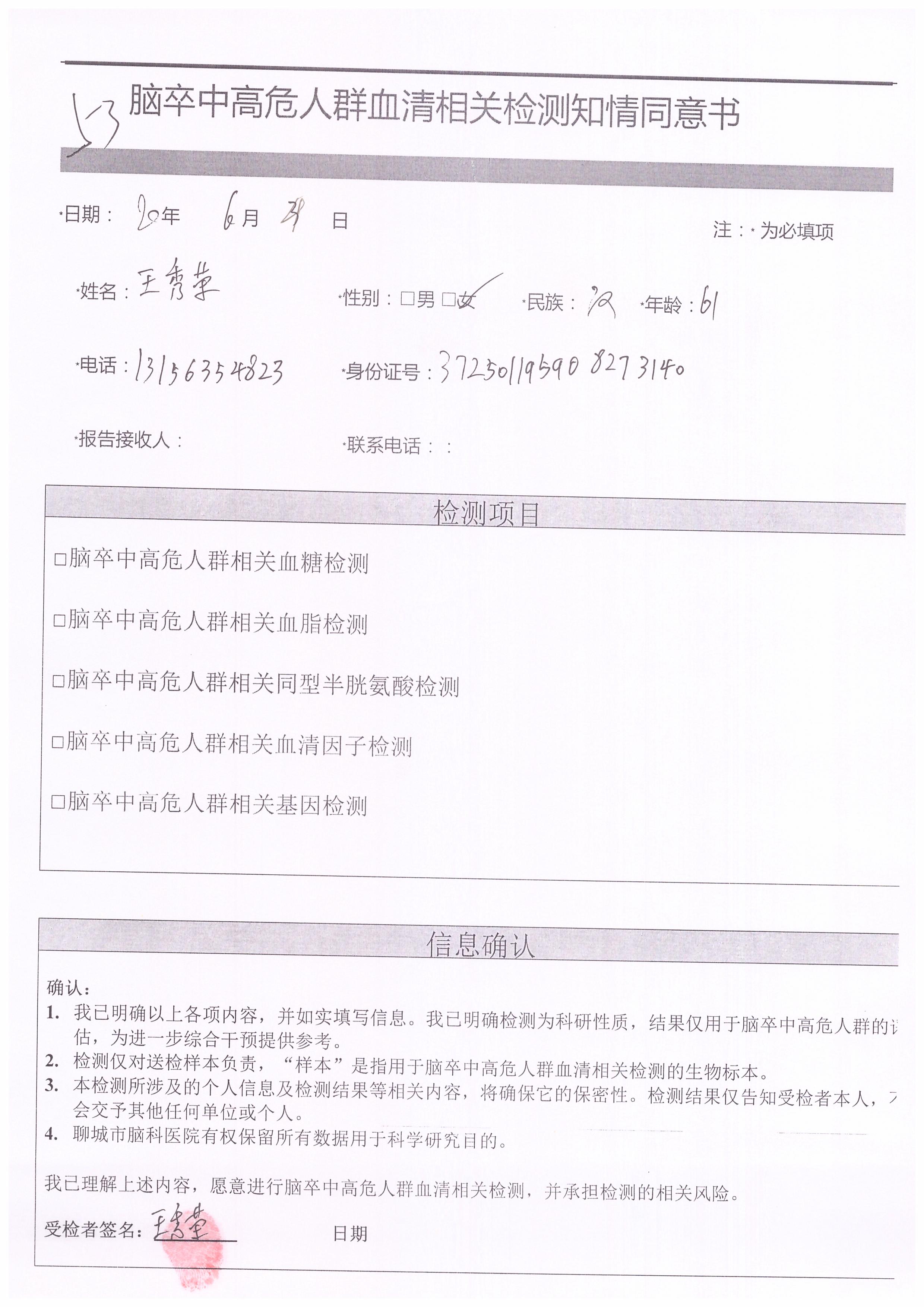

Supplement: Supplementary file 5 — Supplementary file5 (ZIP 24834 KB) [file 10528_2023_10431_MOESM5_ESM.zip › ╓¬╟Θ═1⁄4╥Γ╩Θ3/053.jpg]

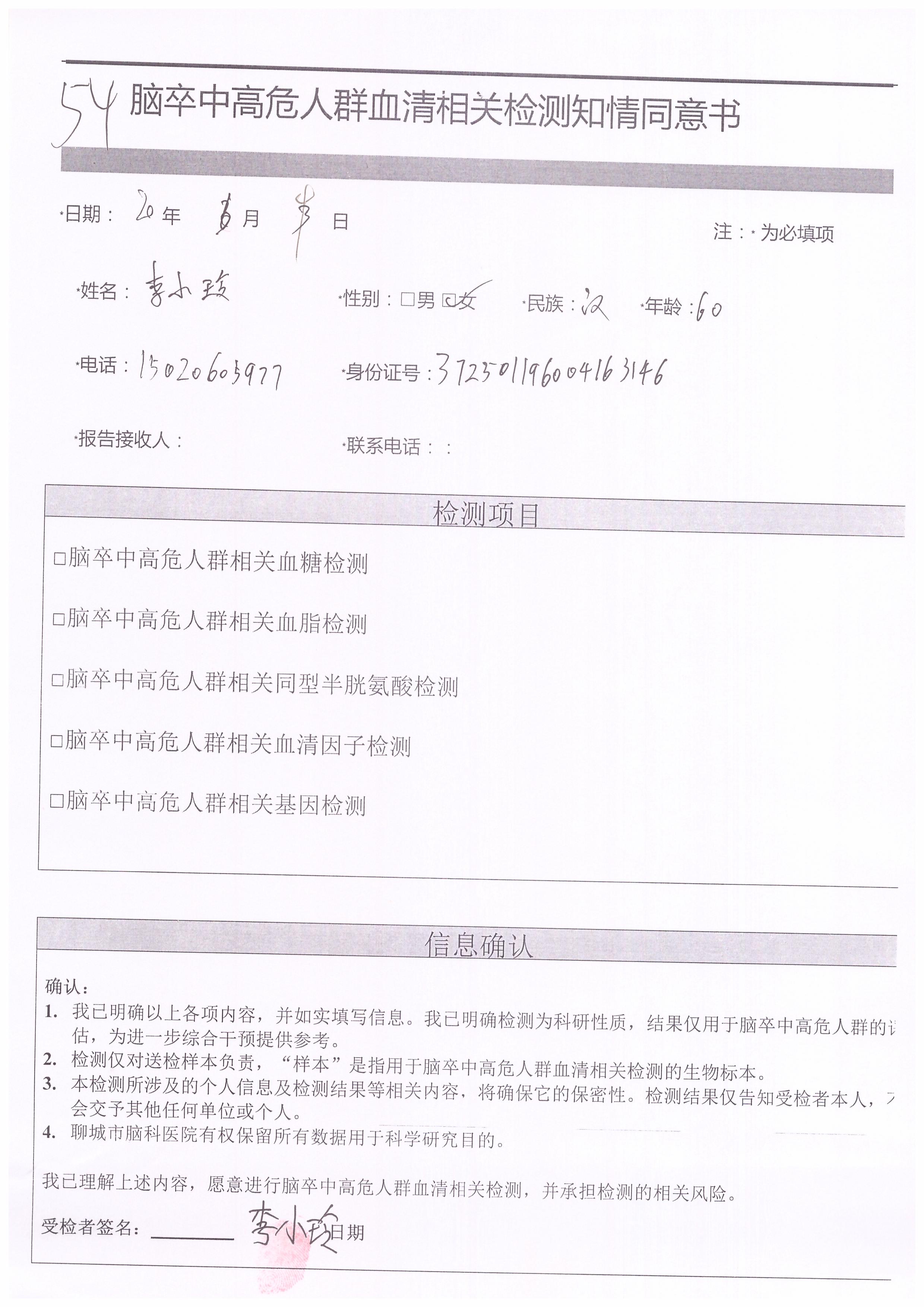

Supplement: Supplementary file 5 — Supplementary file5 (ZIP 24834 KB) [file 10528_2023_10431_MOESM5_ESM.zip › ╓¬╟Θ═1⁄4╥Γ╩Θ3/054.jpg]

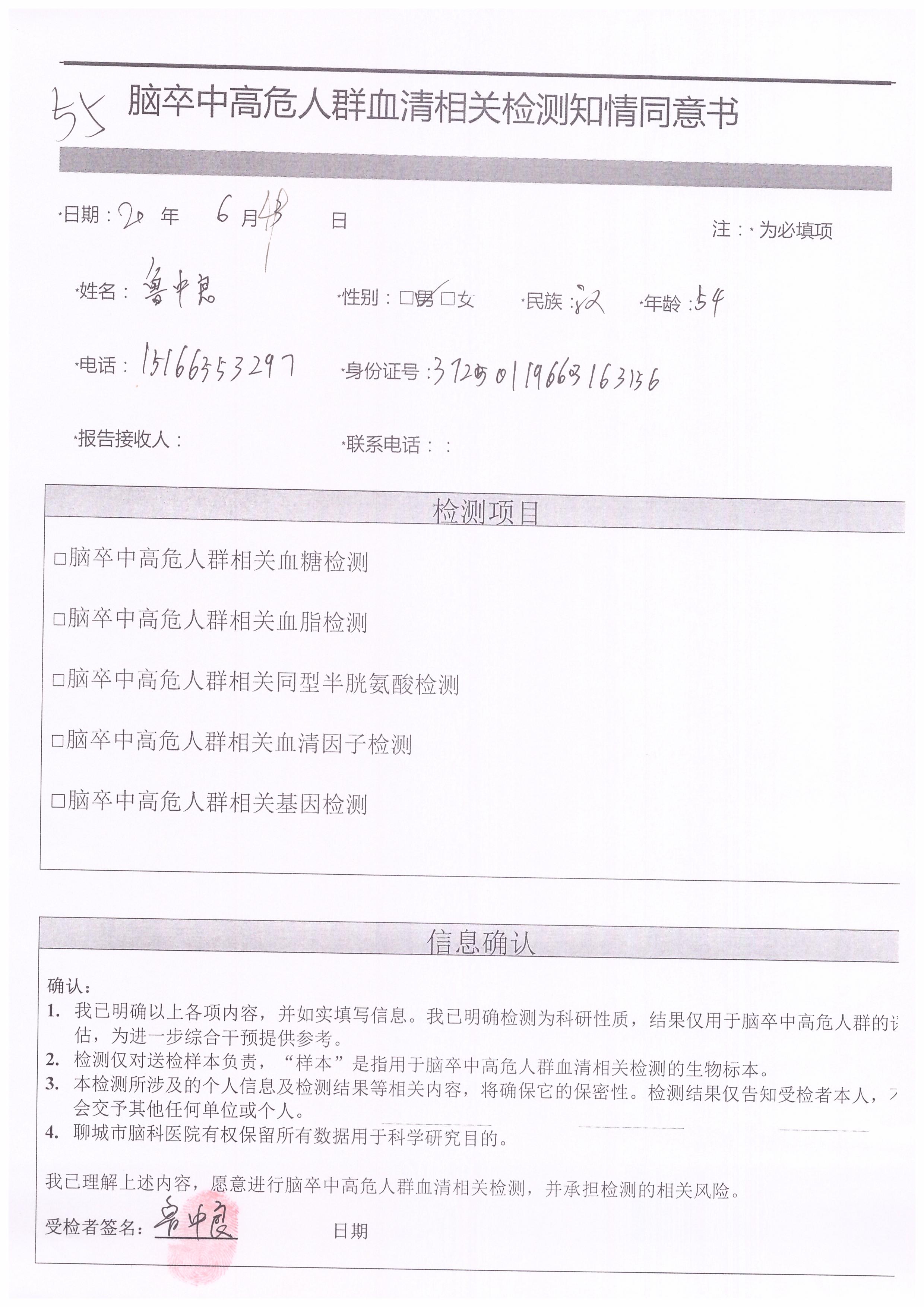

Supplement: Supplementary file 5 — Supplementary file5 (ZIP 24834 KB) [file 10528_2023_10431_MOESM5_ESM.zip › ╓¬╟Θ═1⁄4╥Γ╩Θ3/055.jpg]

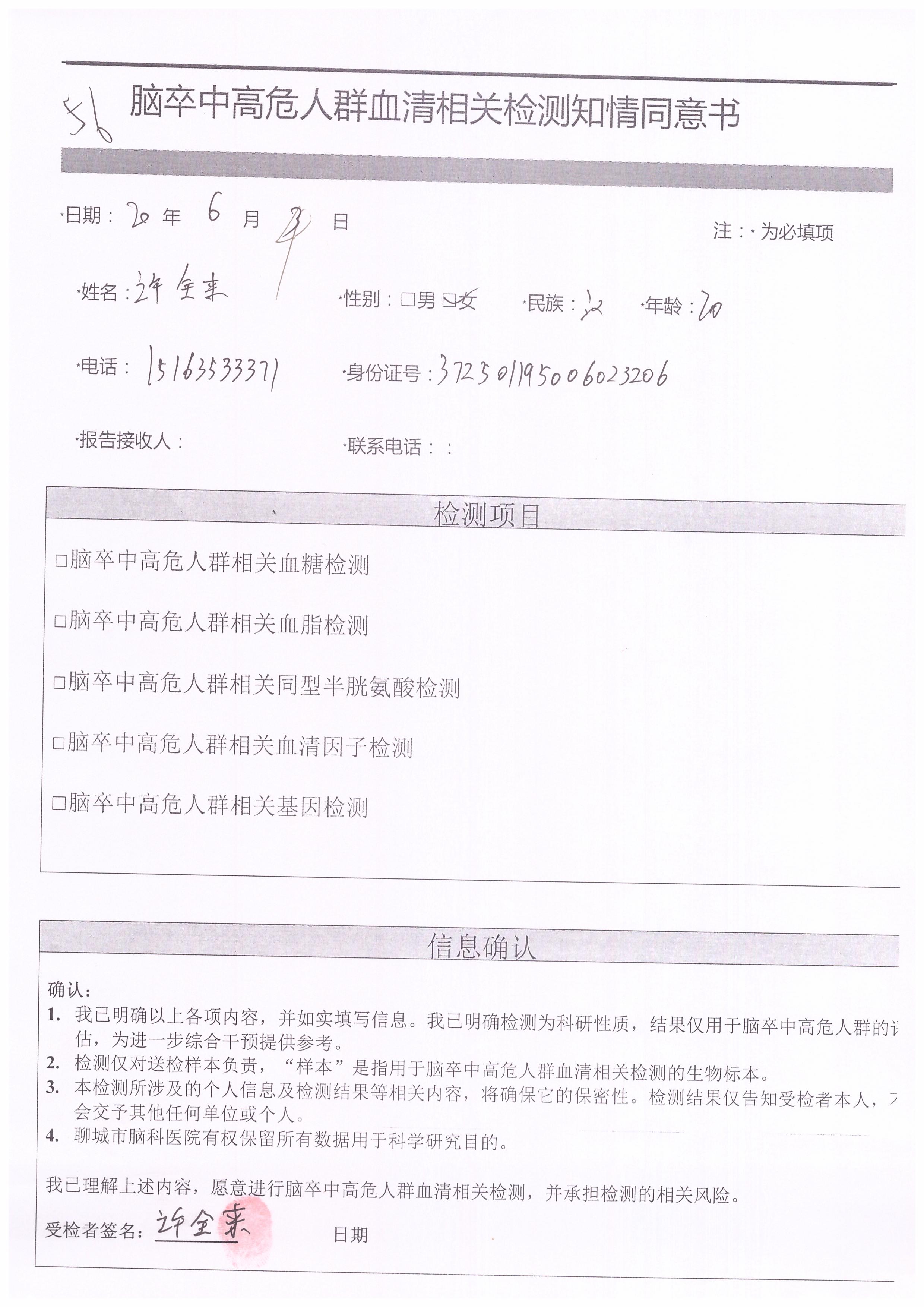

Supplement: Supplementary file 5 — Supplementary file5 (ZIP 24834 KB) [file 10528_2023_10431_MOESM5_ESM.zip › ╓¬╟Θ═1⁄4╥Γ╩Θ3/056.jpg]

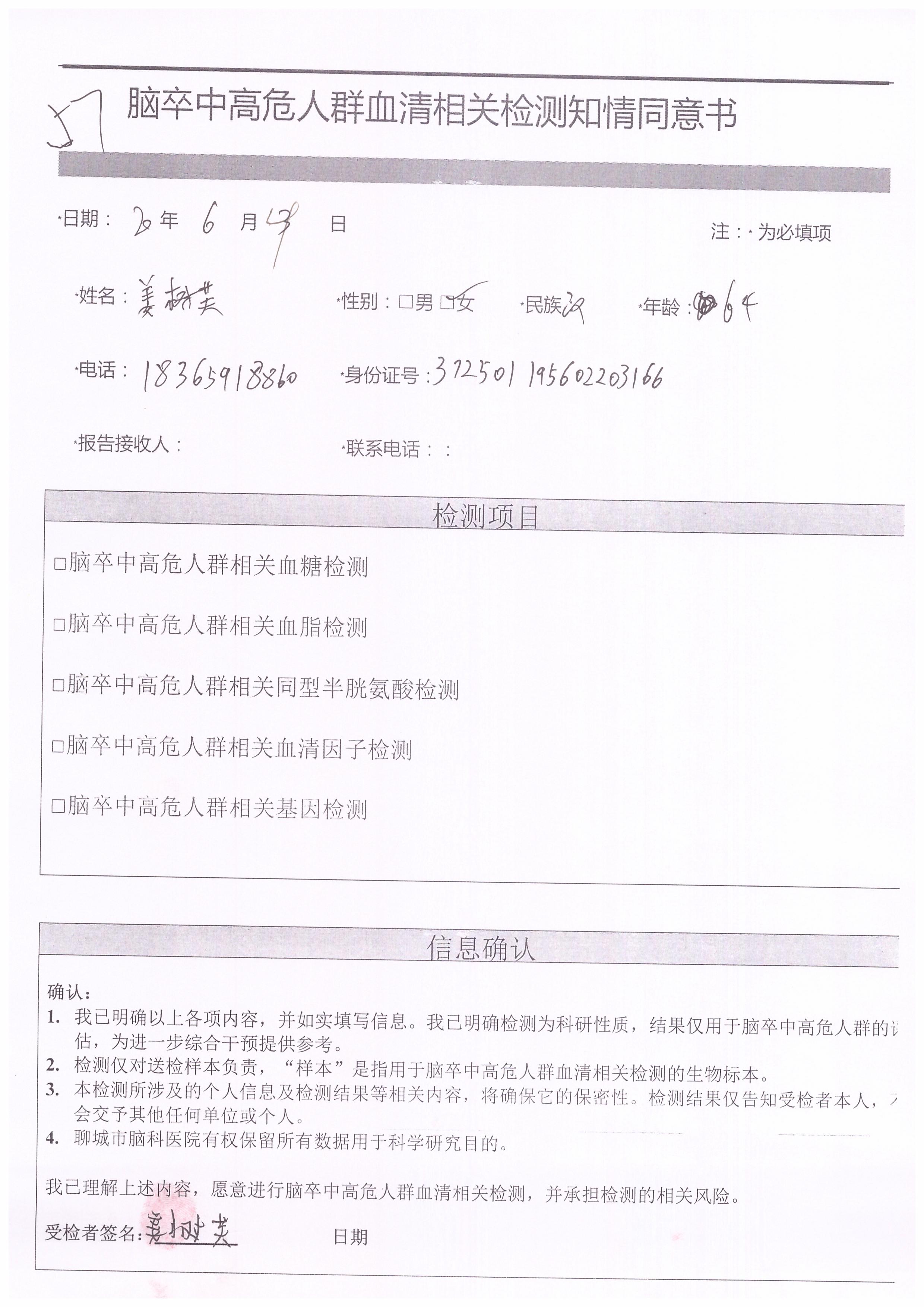

Supplement: Supplementary file 5 — Supplementary file5 (ZIP 24834 KB) [file 10528_2023_10431_MOESM5_ESM.zip › ╓¬╟Θ═1⁄4╥Γ╩Θ3/057.jpg]

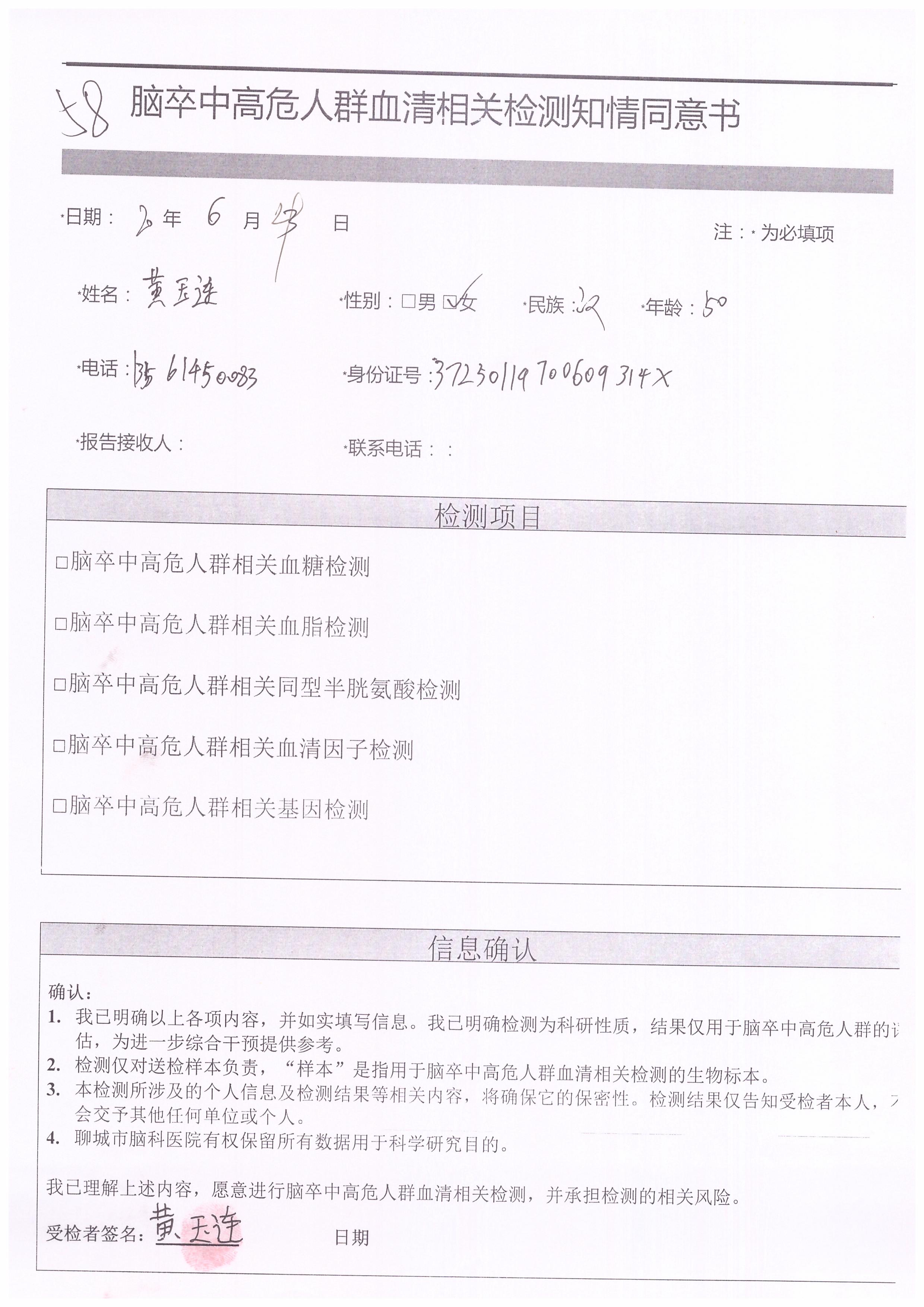

Supplement: Supplementary file 5 — Supplementary file5 (ZIP 24834 KB) [file 10528_2023_10431_MOESM5_ESM.zip › ╓¬╟Θ═1⁄4╥Γ╩Θ3/058.jpg]

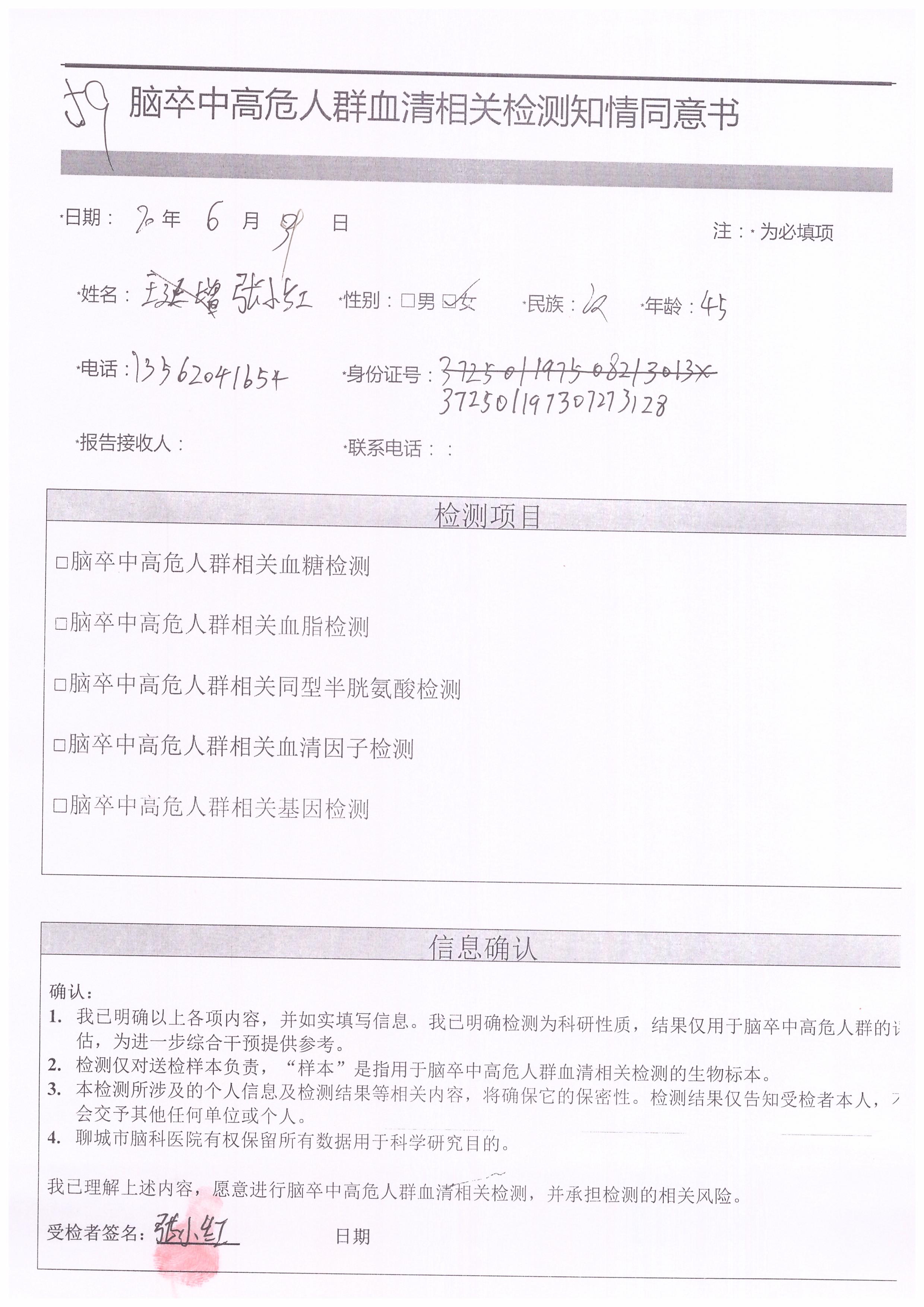

Supplement: Supplementary file 5 — Supplementary file5 (ZIP 24834 KB) [file 10528_2023_10431_MOESM5_ESM.zip › ╓¬╟Θ═1⁄4╥Γ╩Θ3/059.jpg]

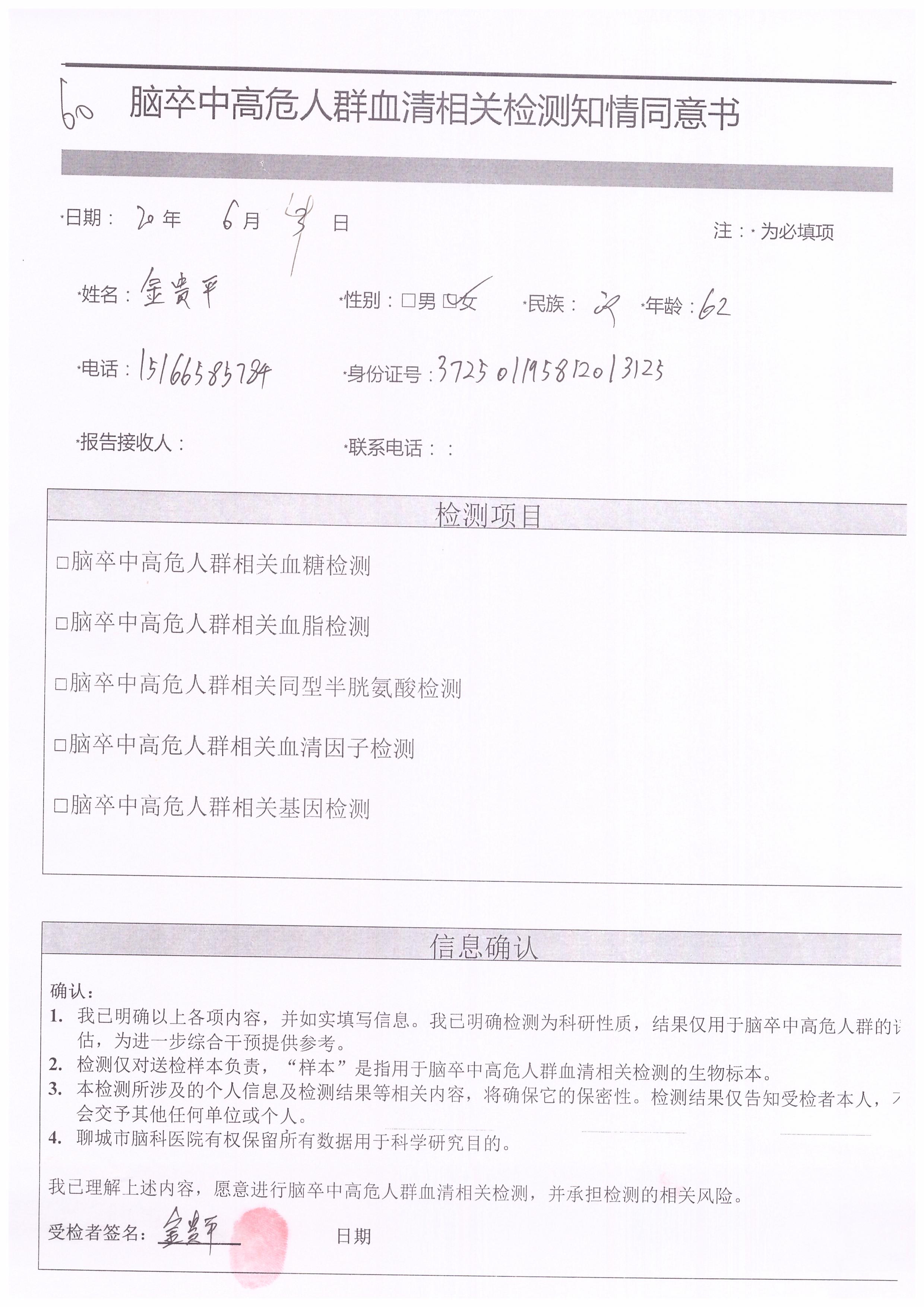

Supplement: Supplementary file 5 — Supplementary file5 (ZIP 24834 KB) [file 10528_2023_10431_MOESM5_ESM.zip › ╓¬╟Θ═1⁄4╥Γ╩Θ3/060.jpg]

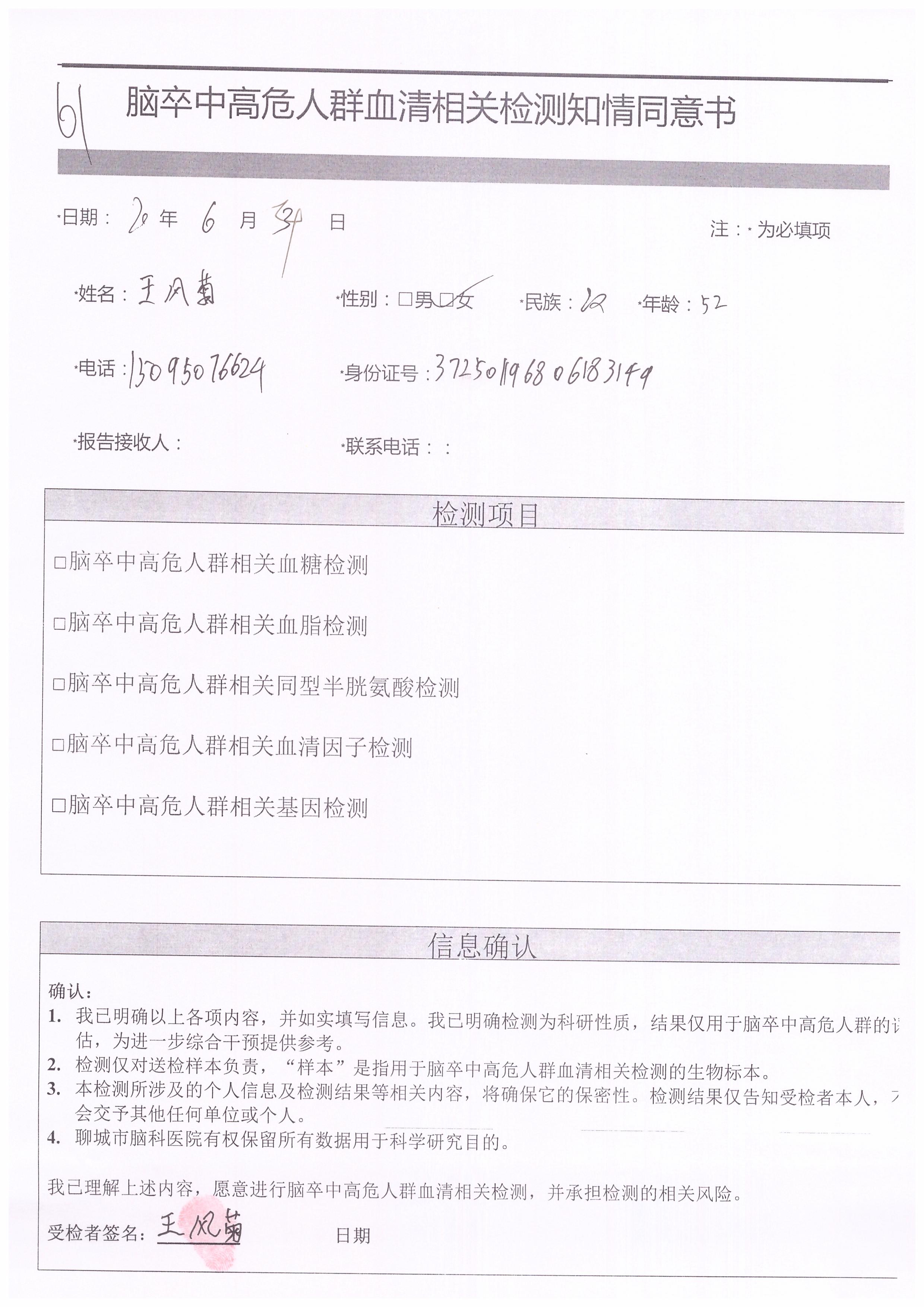

Supplement: Supplementary file 5 — Supplementary file5 (ZIP 24834 KB) [file 10528_2023_10431_MOESM5_ESM.zip › ╓¬╟Θ═1⁄4╥Γ╩Θ3/061.jpg]

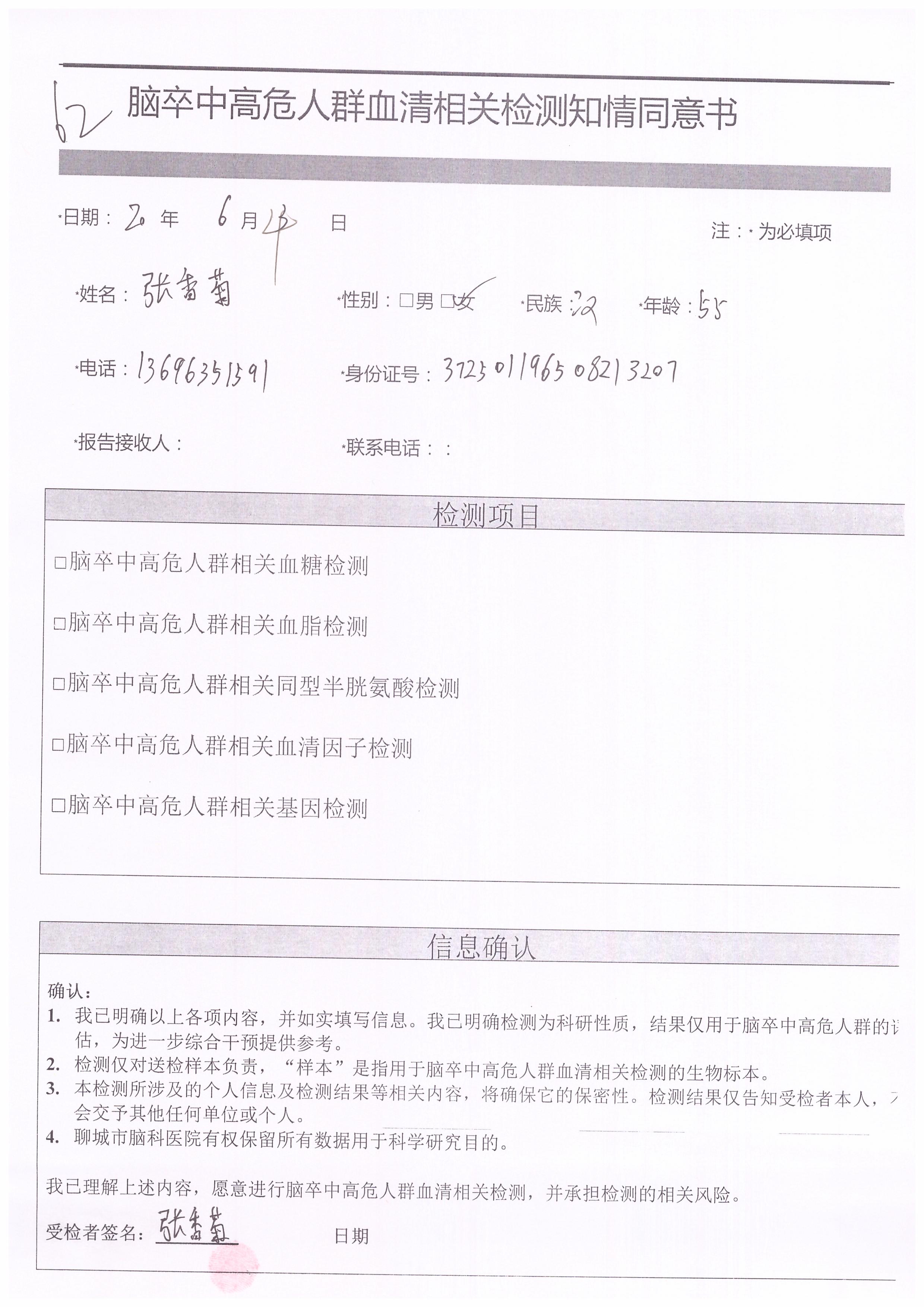

Supplement: Supplementary file 5 — Supplementary file5 (ZIP 24834 KB) [file 10528_2023_10431_MOESM5_ESM.zip › ╓¬╟Θ═1⁄4╥Γ╩Θ3/062.jpg]

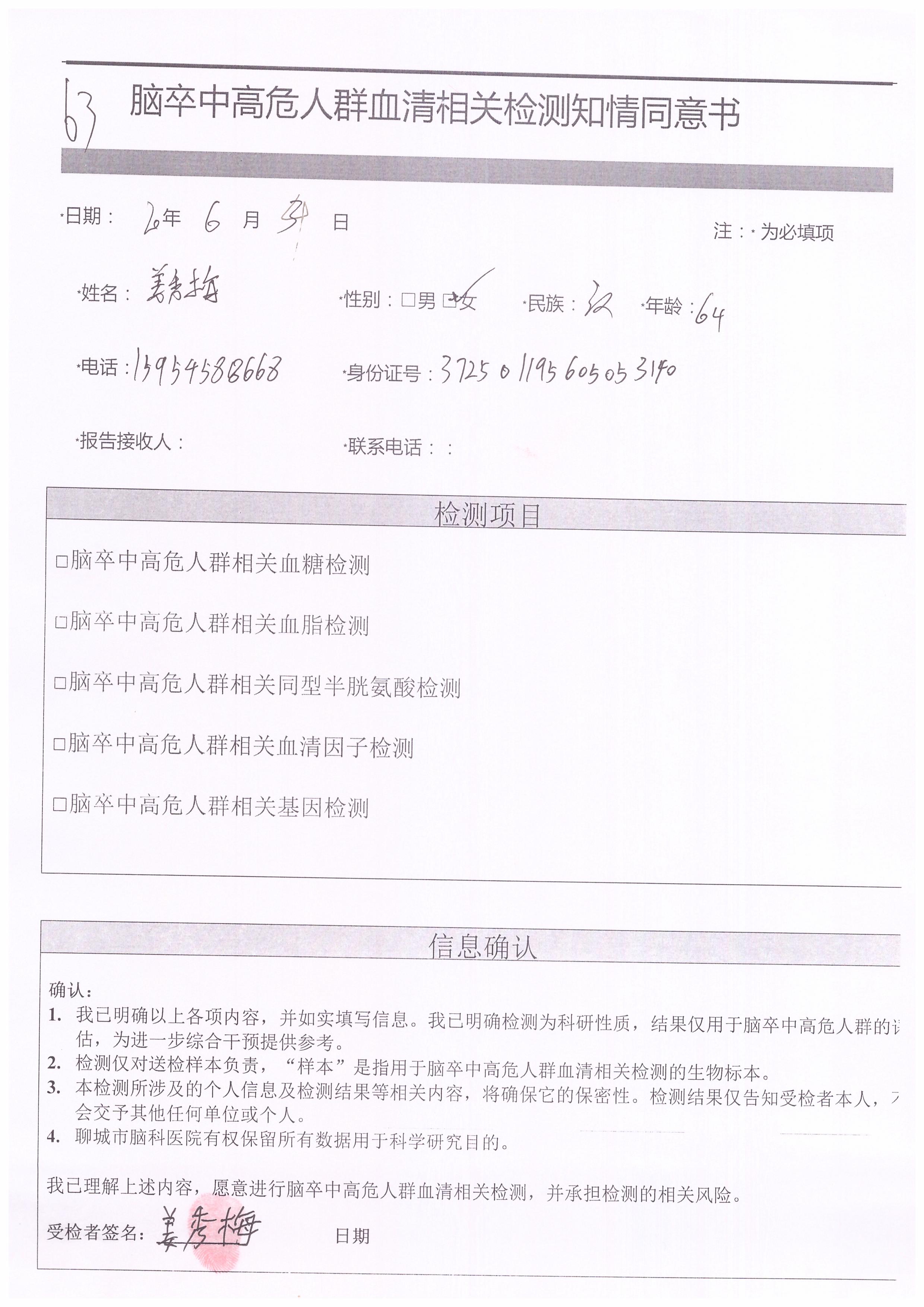

Supplement: Supplementary file 5 — Supplementary file5 (ZIP 24834 KB) [file 10528_2023_10431_MOESM5_ESM.zip › ╓¬╟Θ═1⁄4╥Γ╩Θ3/063.jpg]

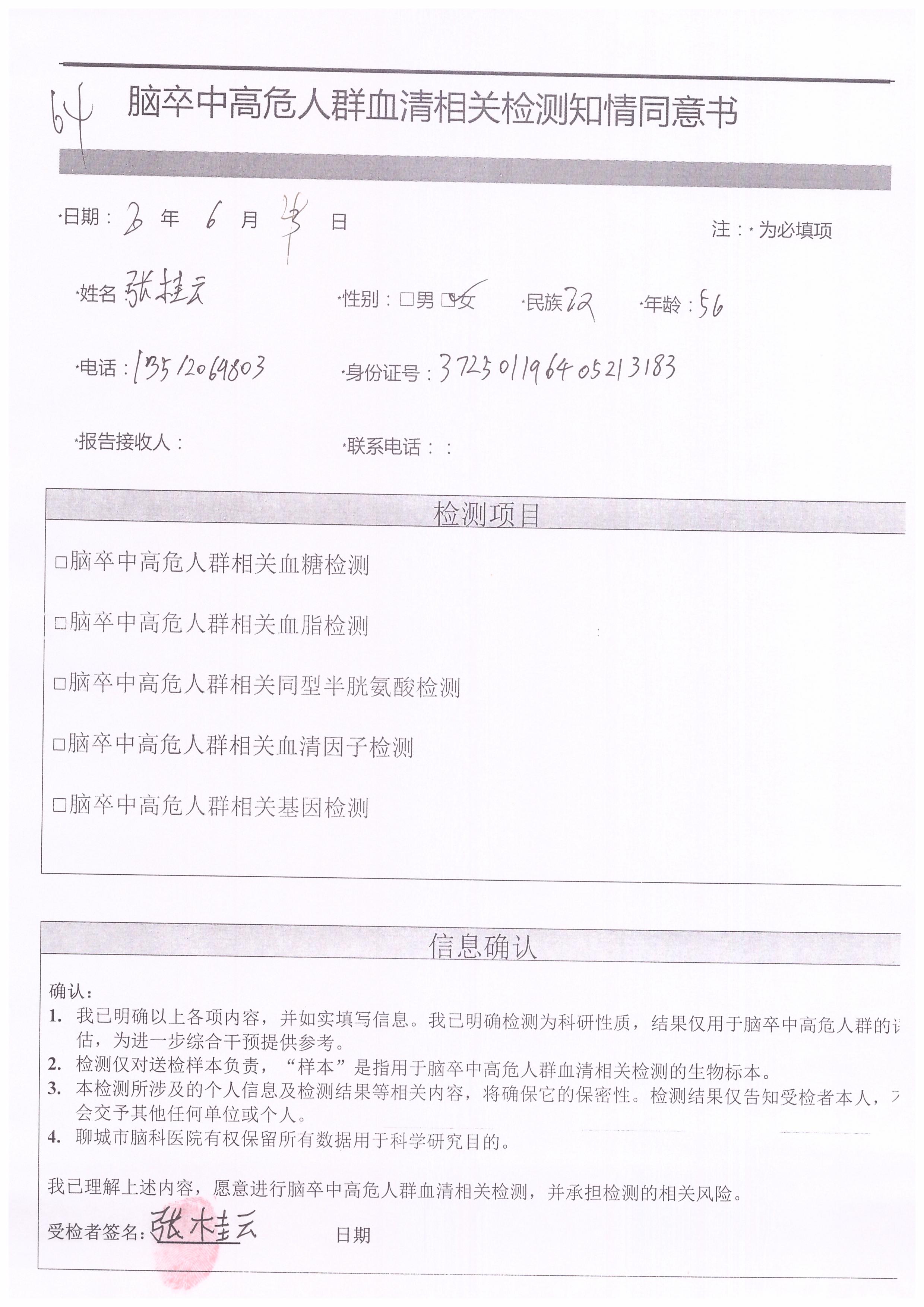

Supplement: Supplementary file 5 — Supplementary file5 (ZIP 24834 KB) [file 10528_2023_10431_MOESM5_ESM.zip › ╓¬╟Θ═1⁄4╥Γ╩Θ3/064.jpg]

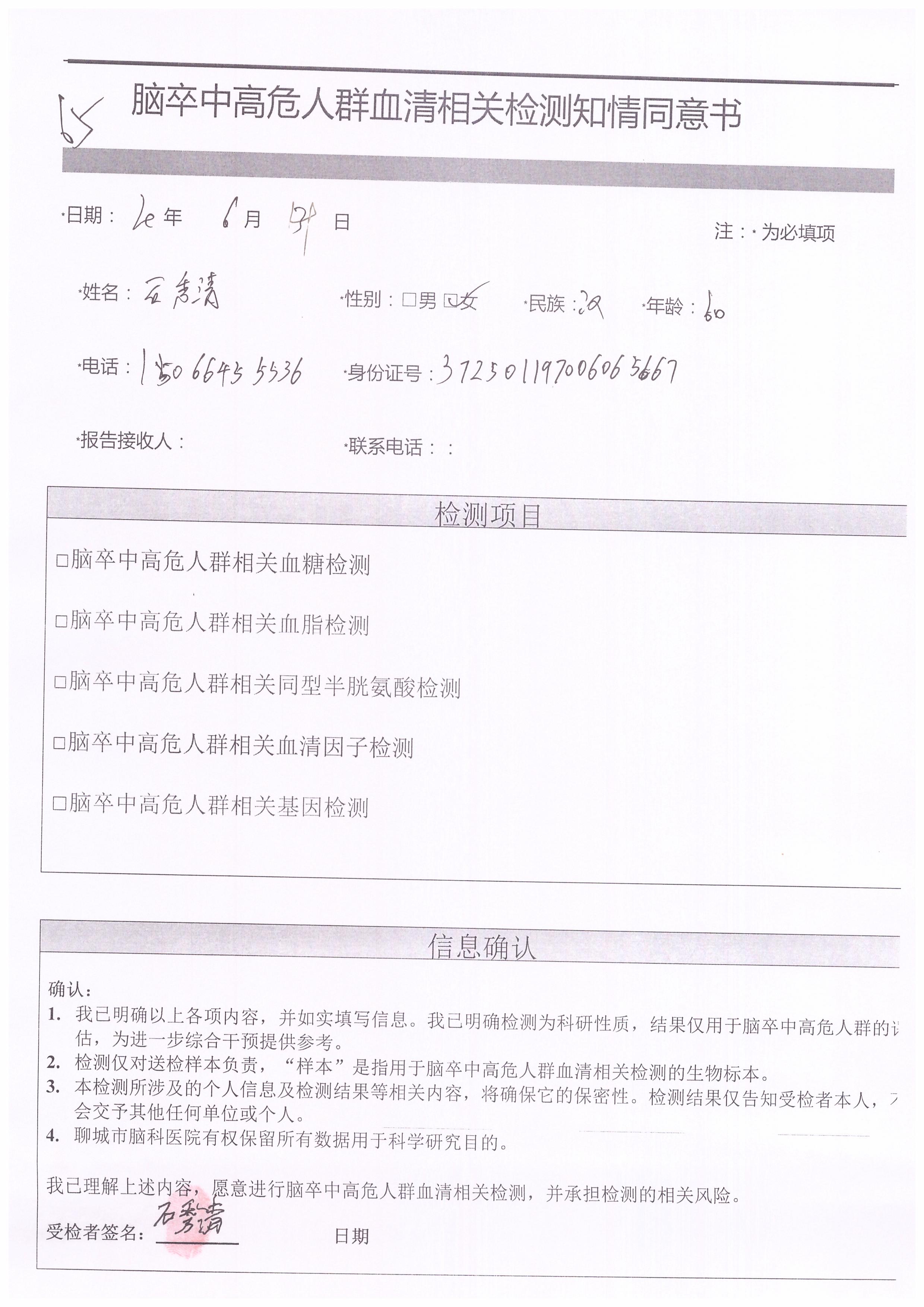

Supplement: Supplementary file 5 — Supplementary file5 (ZIP 24834 KB) [file 10528_2023_10431_MOESM5_ESM.zip › ╓¬╟Θ═1⁄4╥Γ╩Θ3/065.jpg]

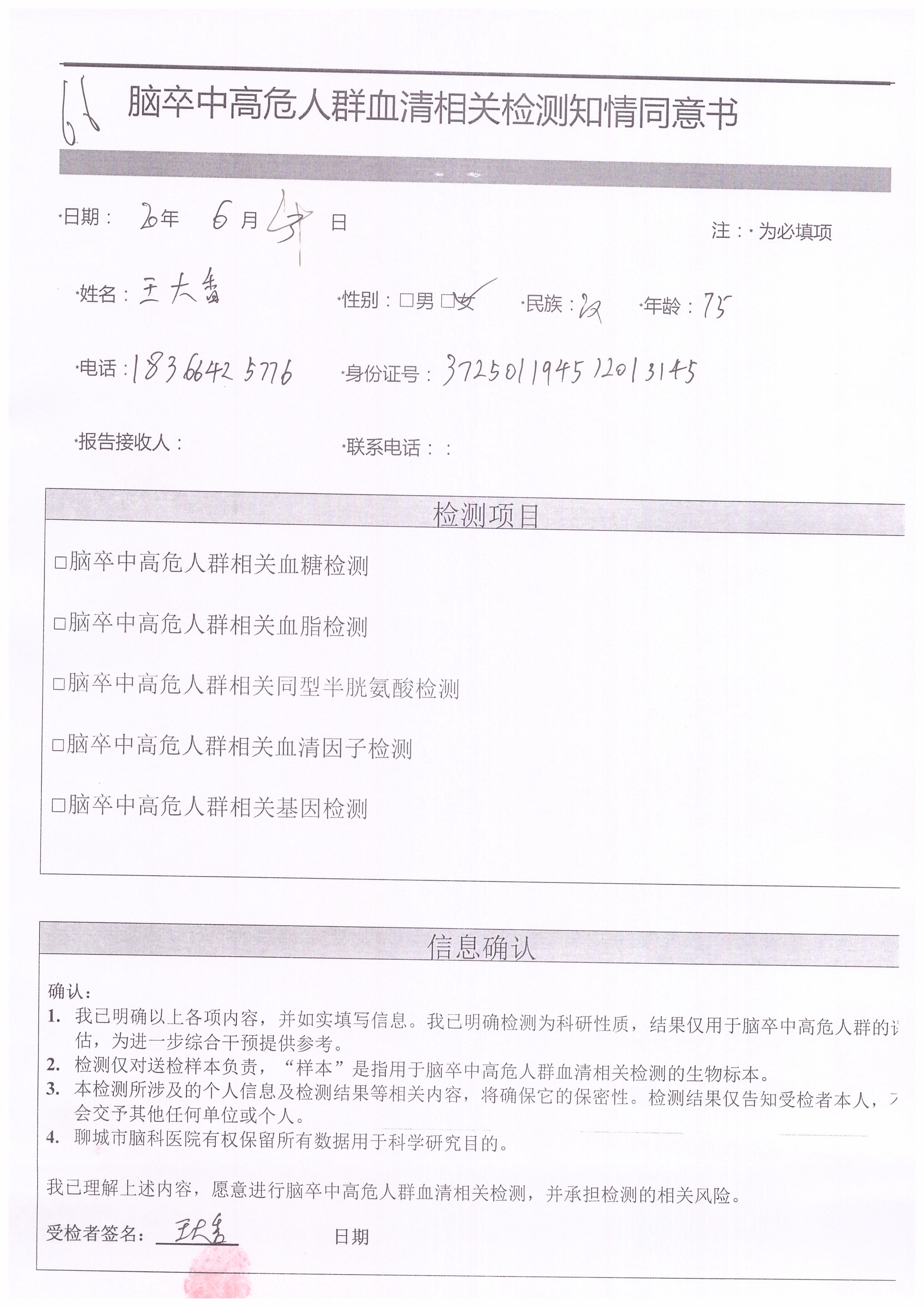

Supplement: Supplementary file 5 — Supplementary file5 (ZIP 24834 KB) [file 10528_2023_10431_MOESM5_ESM.zip › ╓¬╟Θ═1⁄4╥Γ╩Θ3/066.jpg]

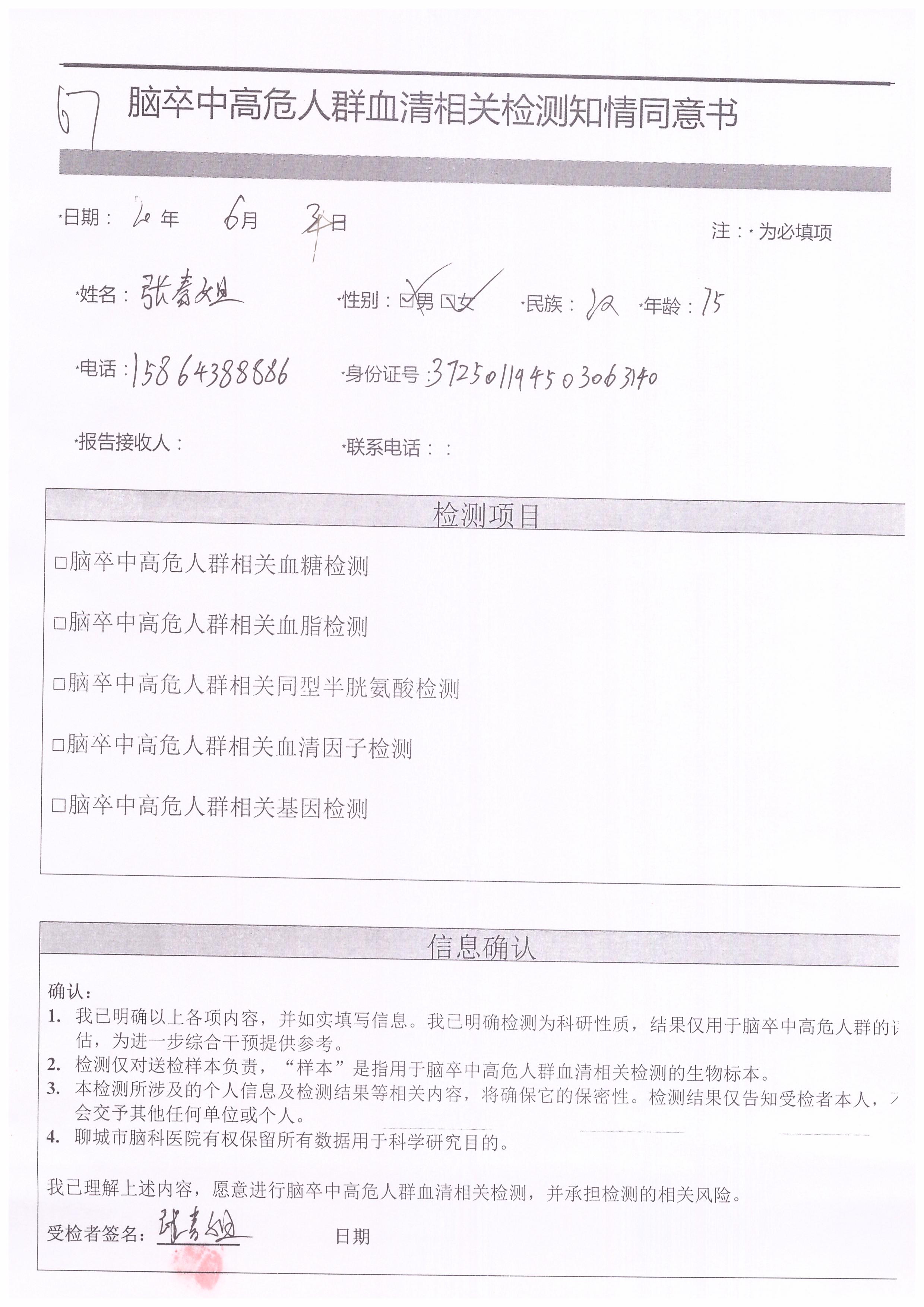

Supplement: Supplementary file 5 — Supplementary file5 (ZIP 24834 KB) [file 10528_2023_10431_MOESM5_ESM.zip › ╓¬╟Θ═1⁄4╥Γ╩Θ3/067.jpg]

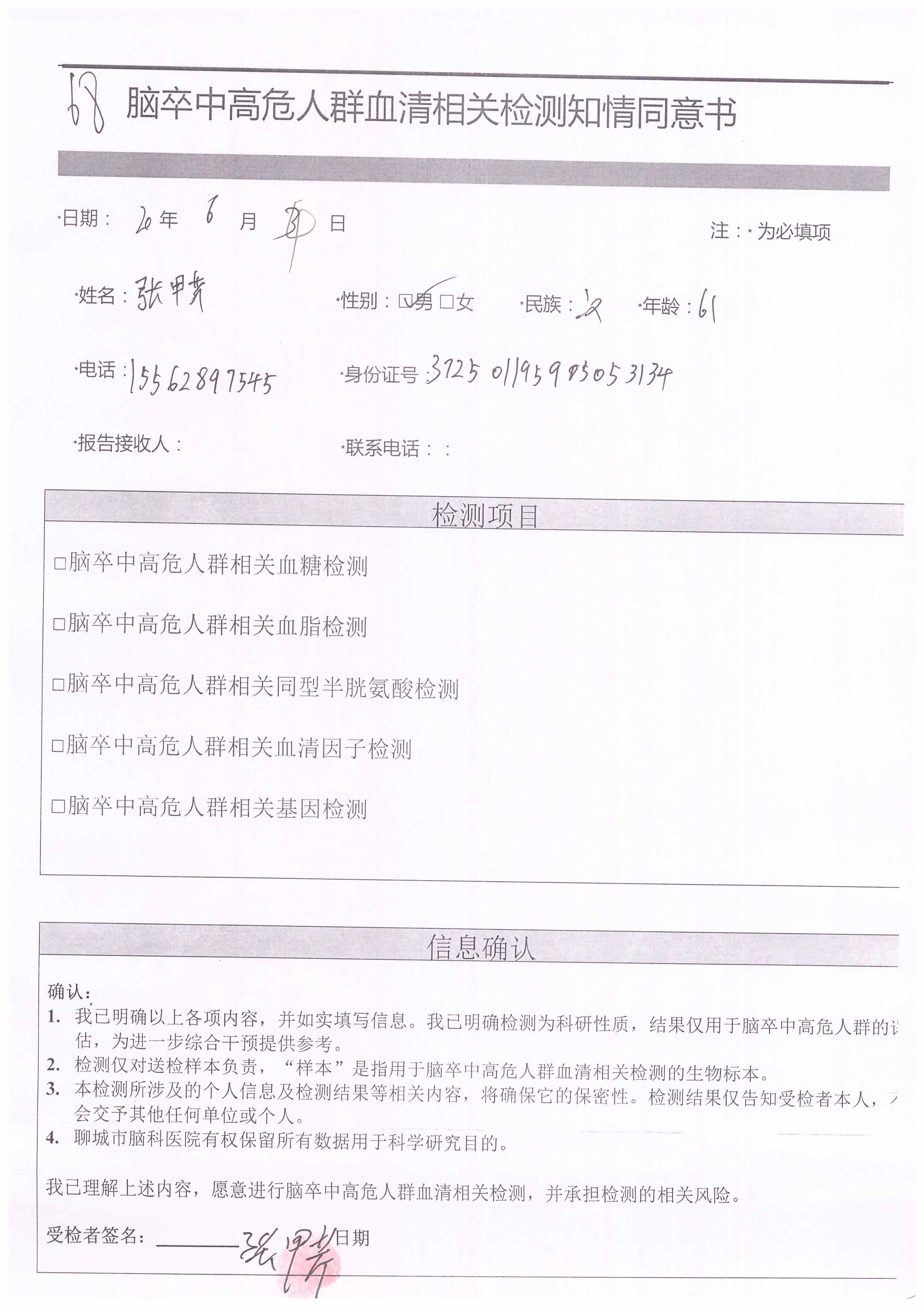

Supplement: Supplementary file 5 — Supplementary file5 (ZIP 24834 KB) [file 10528_2023_10431_MOESM5_ESM.zip › ╓¬╟Θ═1⁄4╥Γ╩Θ3/068.jpg]

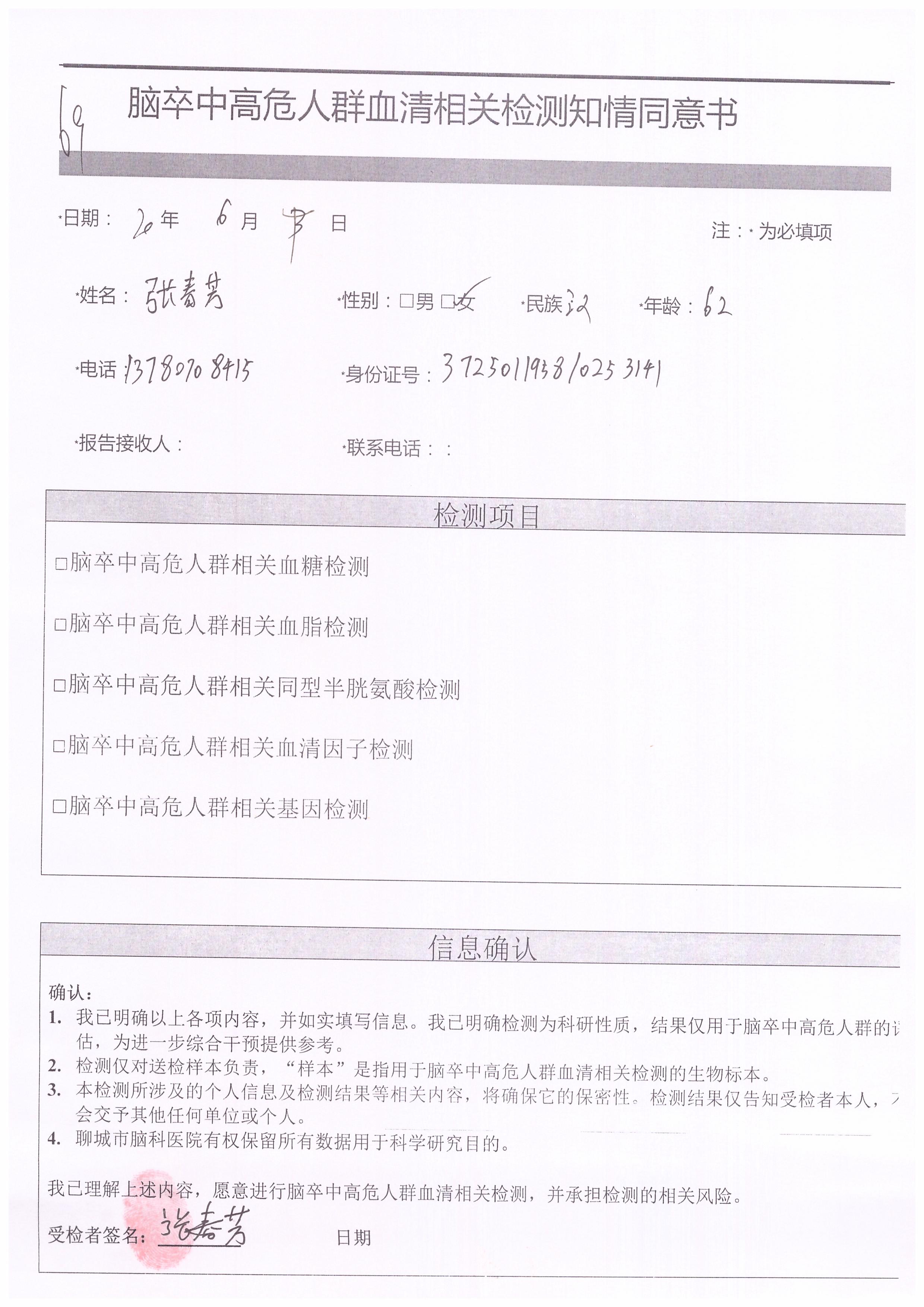

Supplement: Supplementary file 5 — Supplementary file5 (ZIP 24834 KB) [file 10528_2023_10431_MOESM5_ESM.zip › ╓¬╟Θ═1⁄4╥Γ╩Θ3/069.jpg]

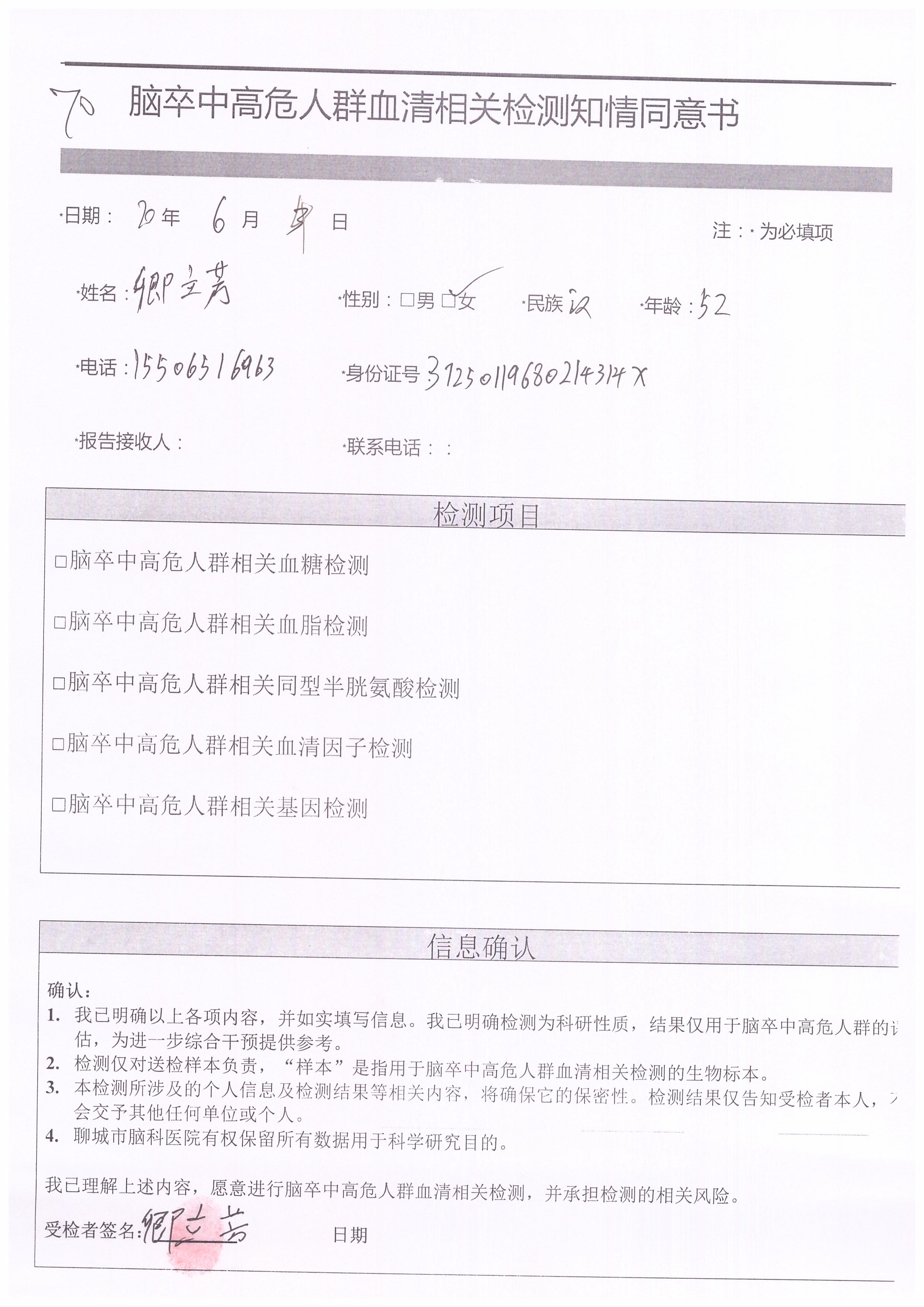

Supplement: Supplementary file 5 — Supplementary file5 (ZIP 24834 KB) [file 10528_2023_10431_MOESM5_ESM.zip › ╓¬╟Θ═1⁄4╥Γ╩Θ3/070.jpg]

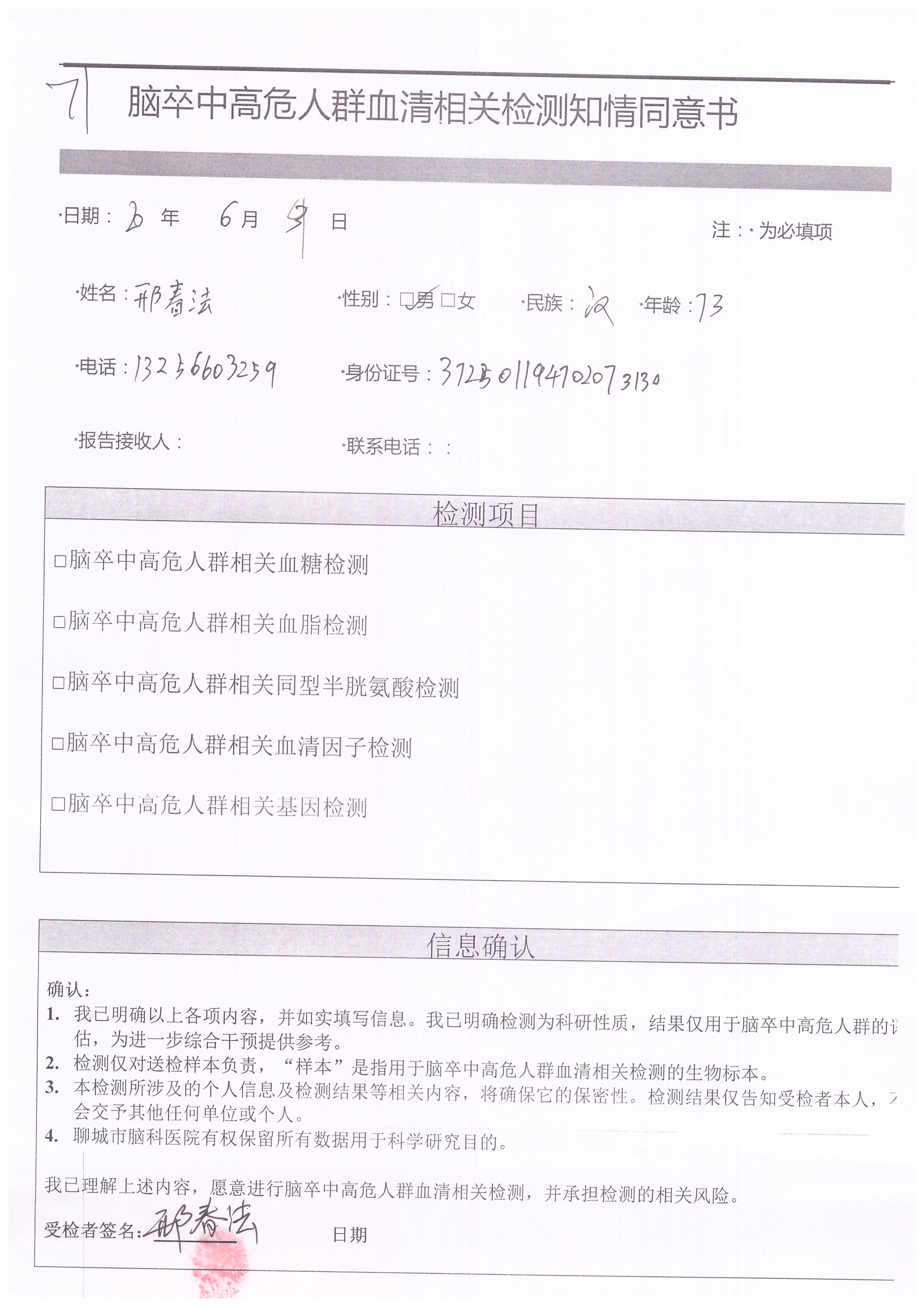

Supplement: Supplementary file 5 — Supplementary file5 (ZIP 24834 KB) [file 10528_2023_10431_MOESM5_ESM.zip › ╓¬╟Θ═1⁄4╥Γ╩Θ3/071.jpg]

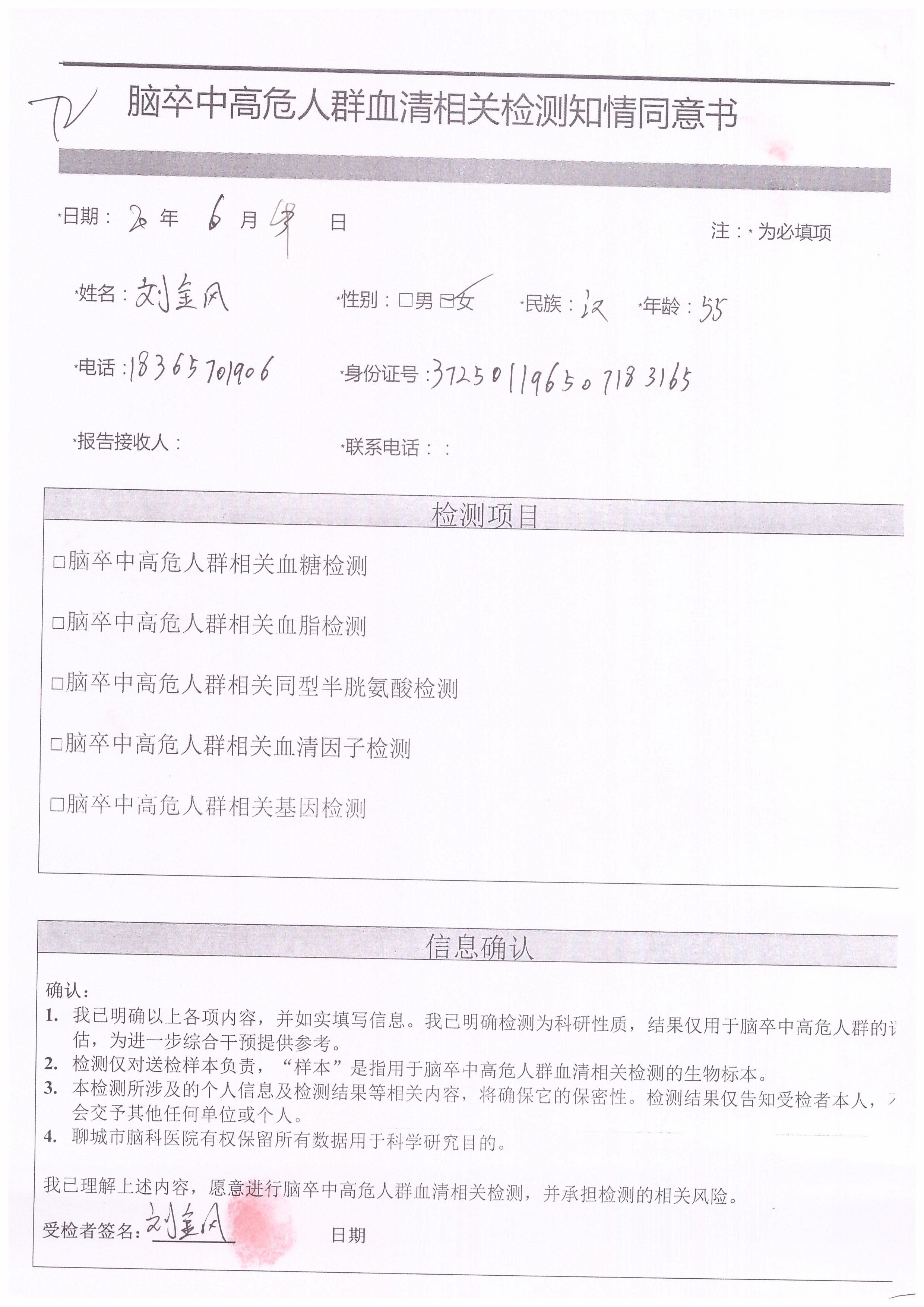

Supplement: Supplementary file 5 — Supplementary file5 (ZIP 24834 KB) [file 10528_2023_10431_MOESM5_ESM.zip › ╓¬╟Θ═1⁄4╥Γ╩Θ3/072.jpg]

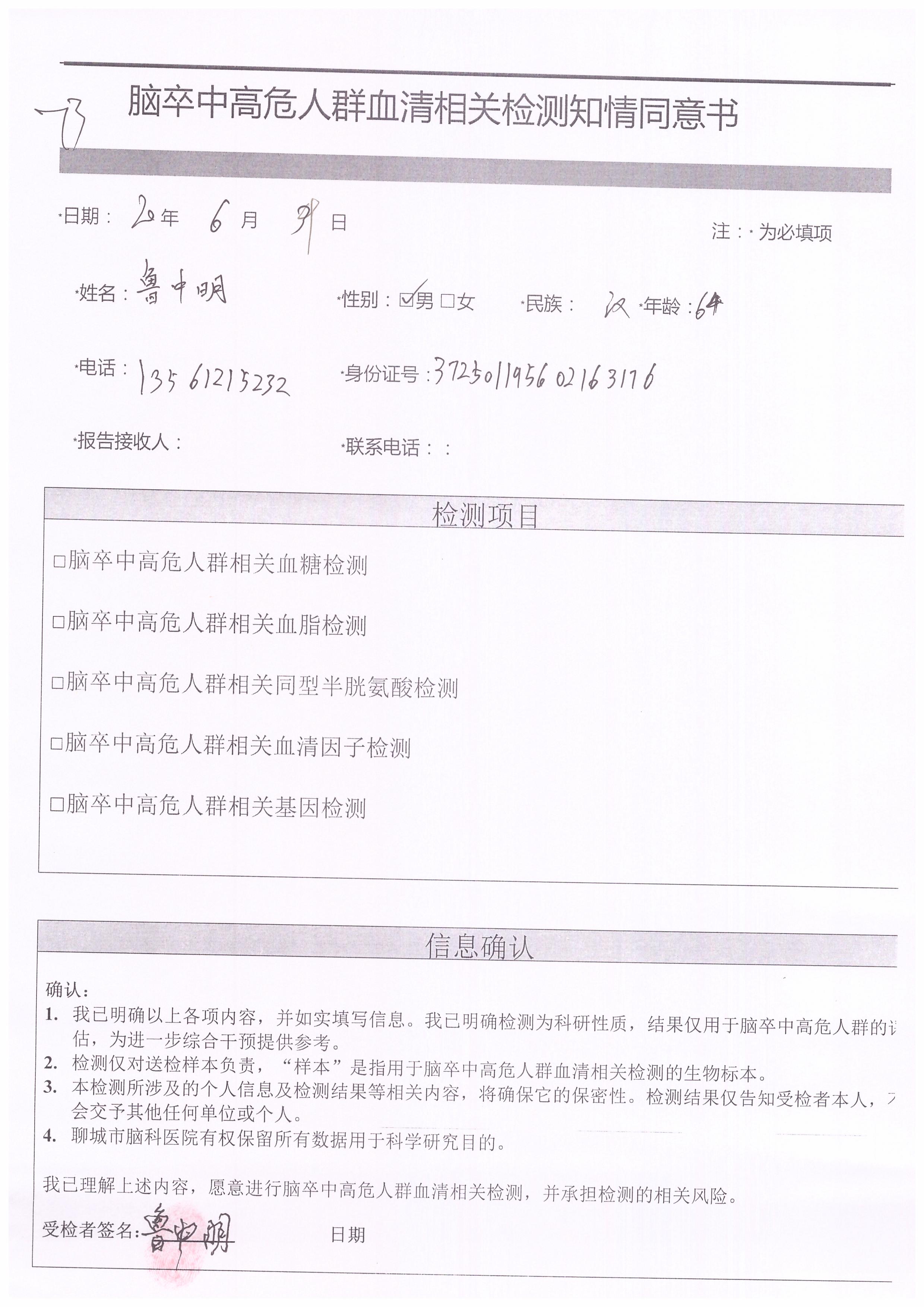

Supplement: Supplementary file 5 — Supplementary file5 (ZIP 24834 KB) [file 10528_2023_10431_MOESM5_ESM.zip › ╓¬╟Θ═1⁄4╥Γ╩Θ3/073.jpg]

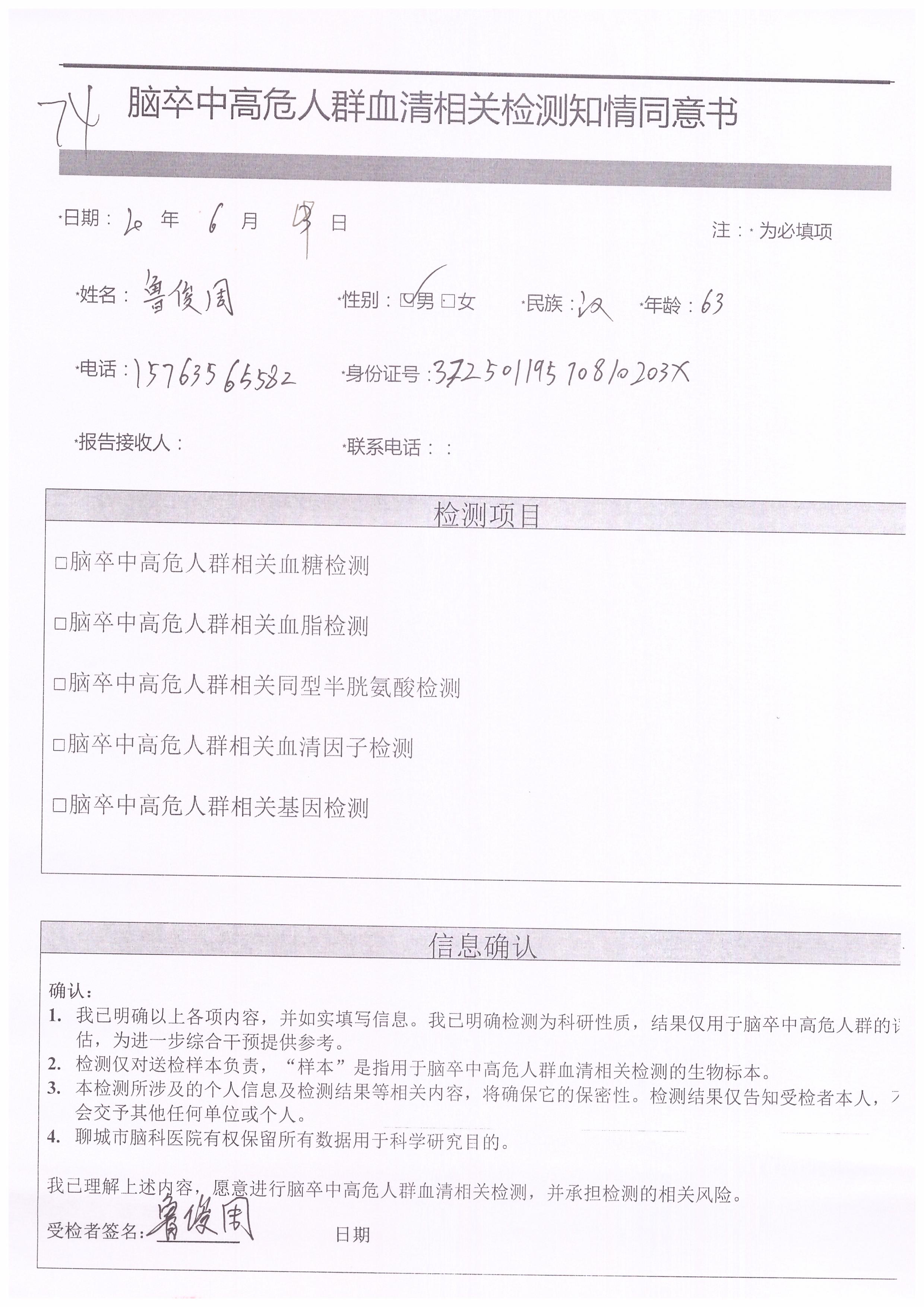

Supplement: Supplementary file 5 — Supplementary file5 (ZIP 24834 KB) [file 10528_2023_10431_MOESM5_ESM.zip › ╓¬╟Θ═1⁄4╥Γ╩Θ3/074.jpg]

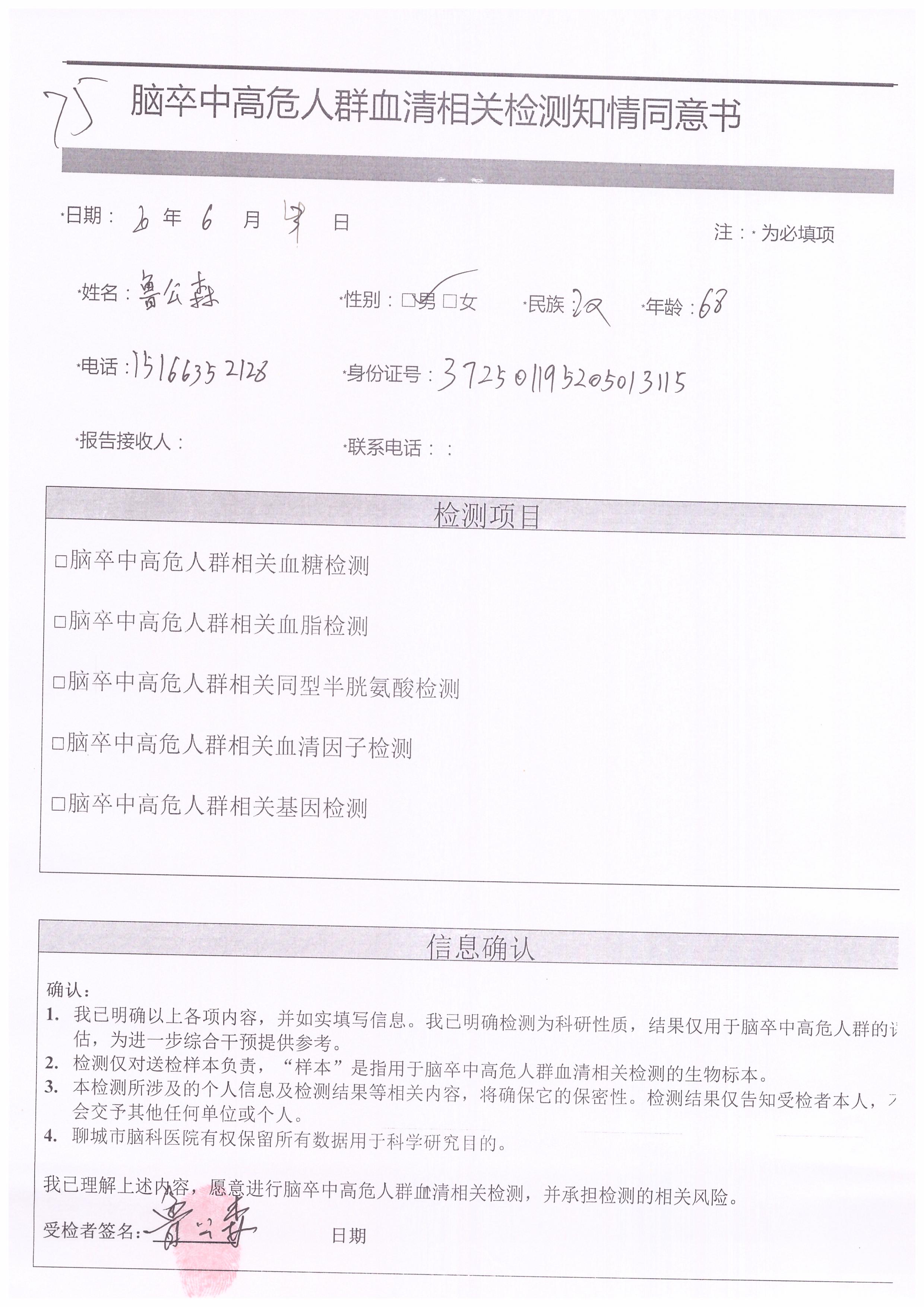

Supplement: Supplementary file 5 — Supplementary file5 (ZIP 24834 KB) [file 10528_2023_10431_MOESM5_ESM.zip › ╓¬╟Θ═1⁄4╥Γ╩Θ3/075.jpg]

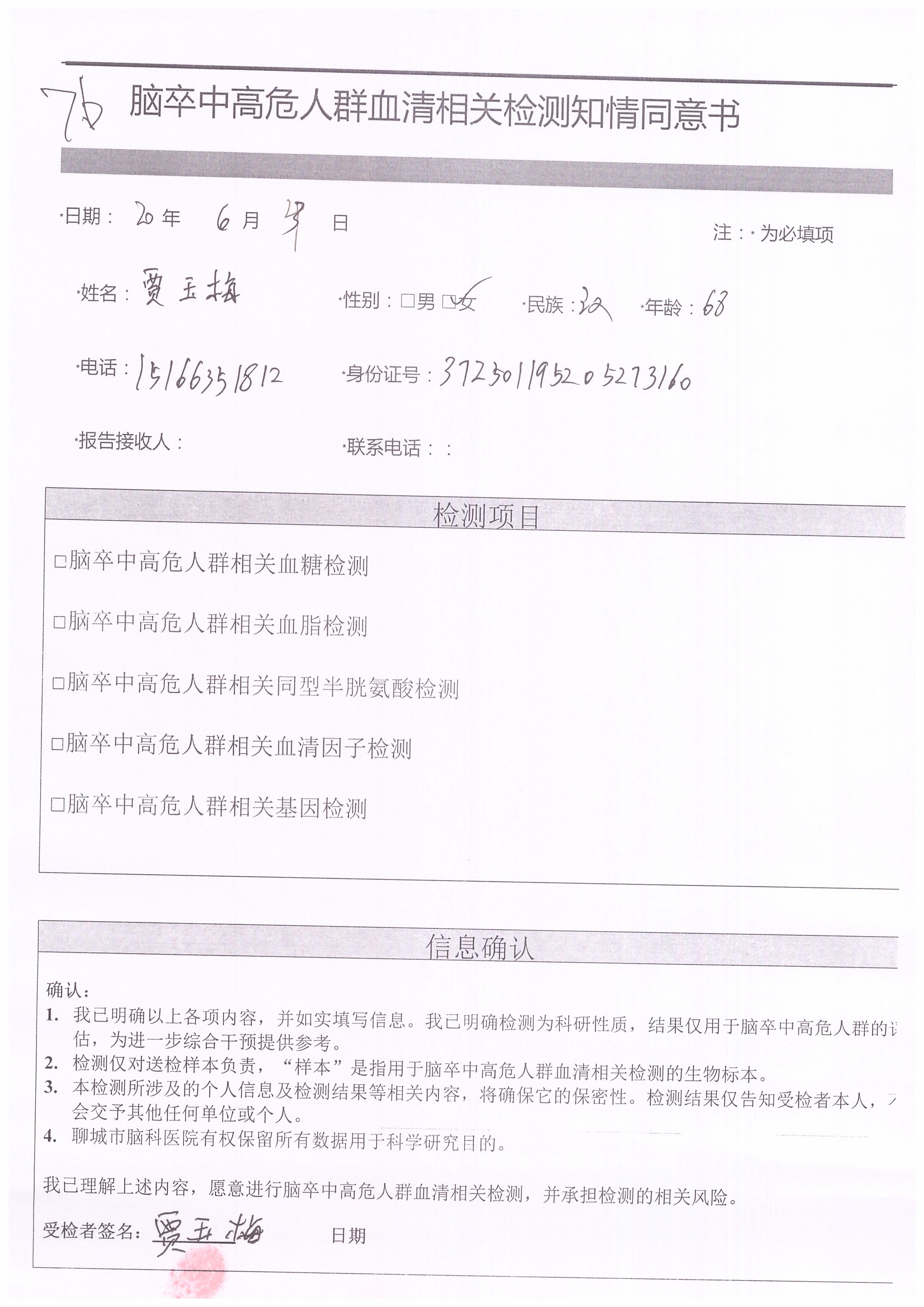

Supplement: Supplementary file 5 — Supplementary file5 (ZIP 24834 KB) [file 10528_2023_10431_MOESM5_ESM.zip › ╓¬╟Θ═1⁄4╥Γ╩Θ3/076.jpg]

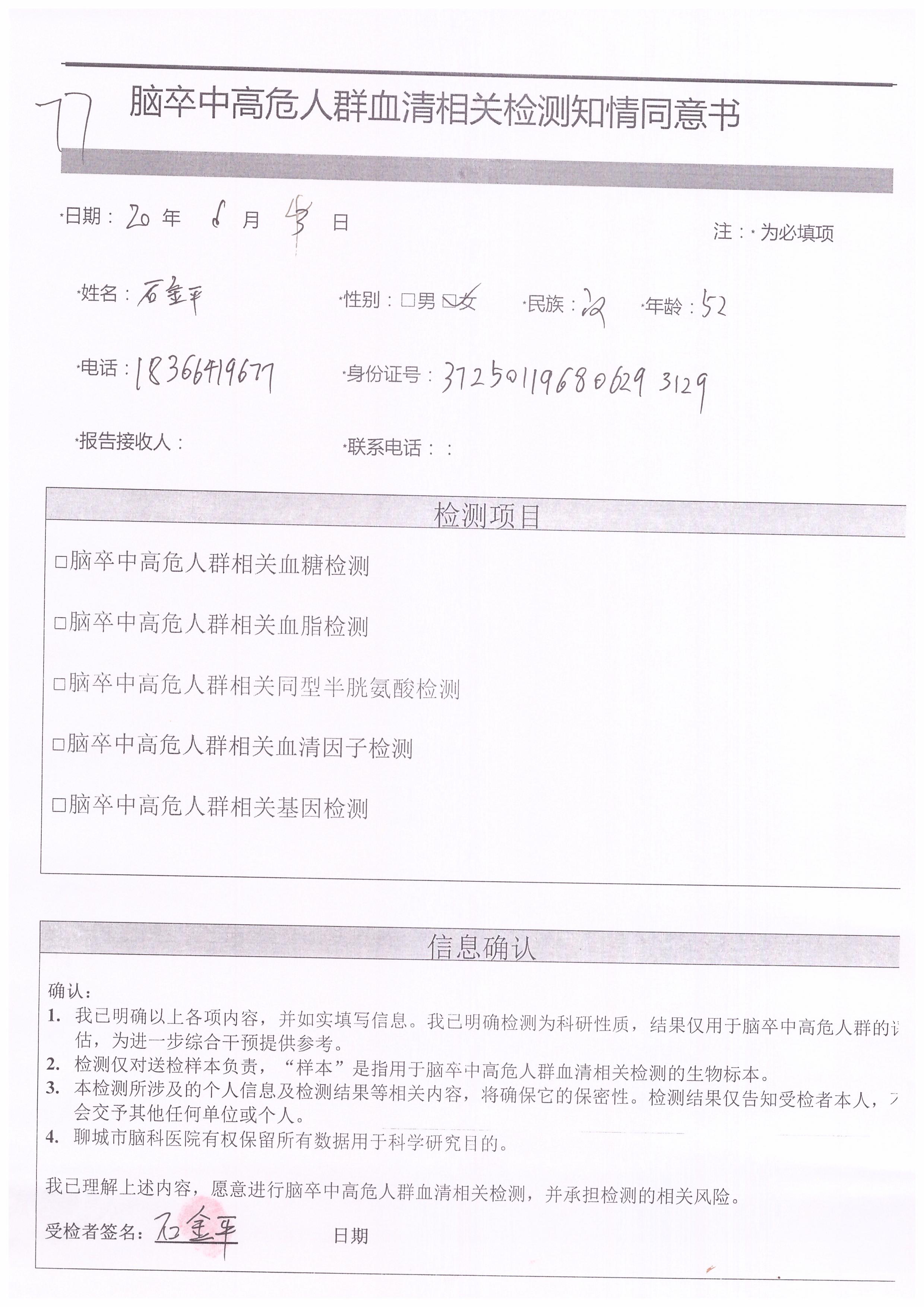

Supplement: Supplementary file 5 — Supplementary file5 (ZIP 24834 KB) [file 10528_2023_10431_MOESM5_ESM.zip › ╓¬╟Θ═1⁄4╥Γ╩Θ3/077.jpg]

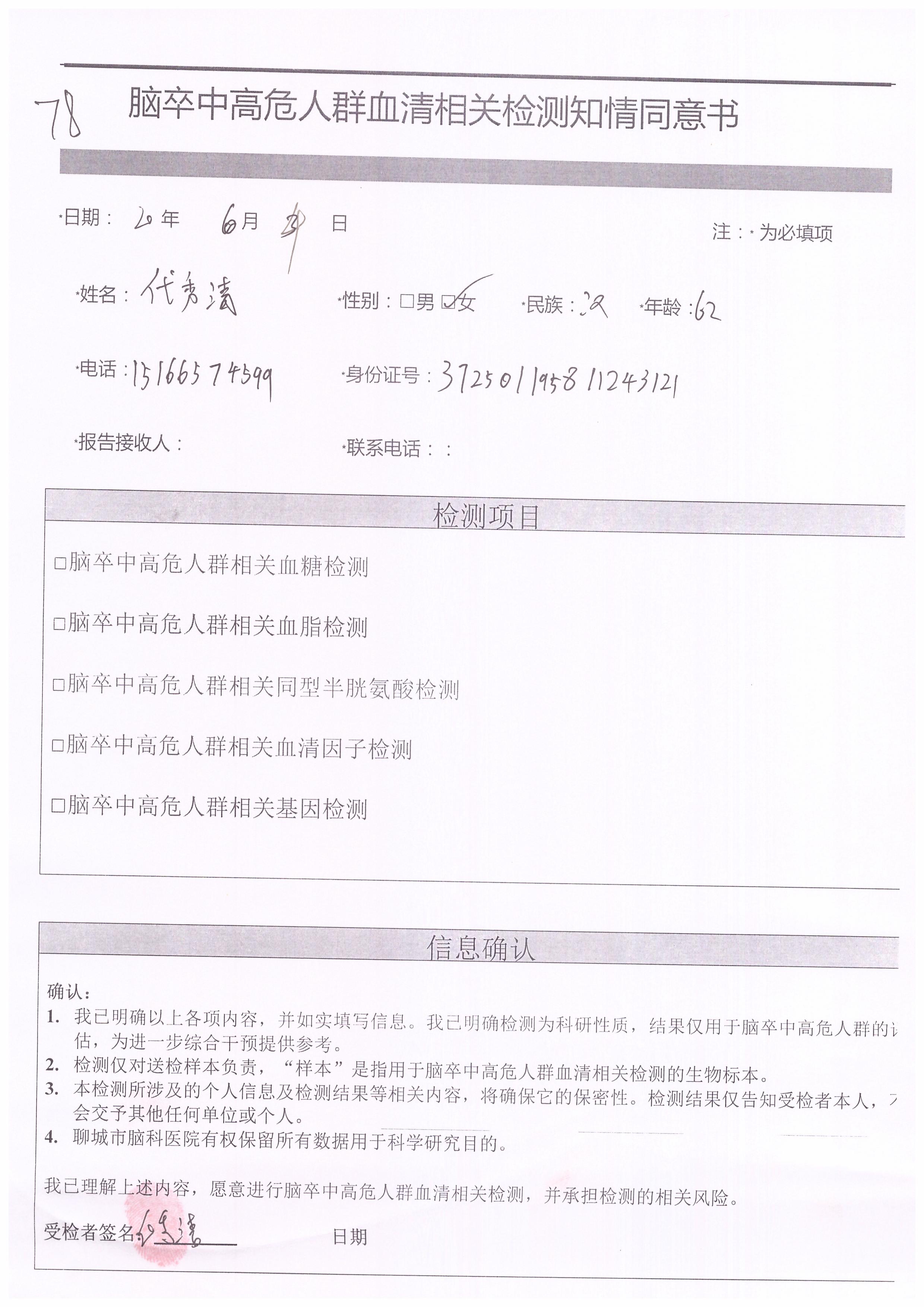

Supplement: Supplementary file 5 — Supplementary file5 (ZIP 24834 KB) [file 10528_2023_10431_MOESM5_ESM.zip › ╓¬╟Θ═1⁄4╥Γ╩Θ3/078.jpg]

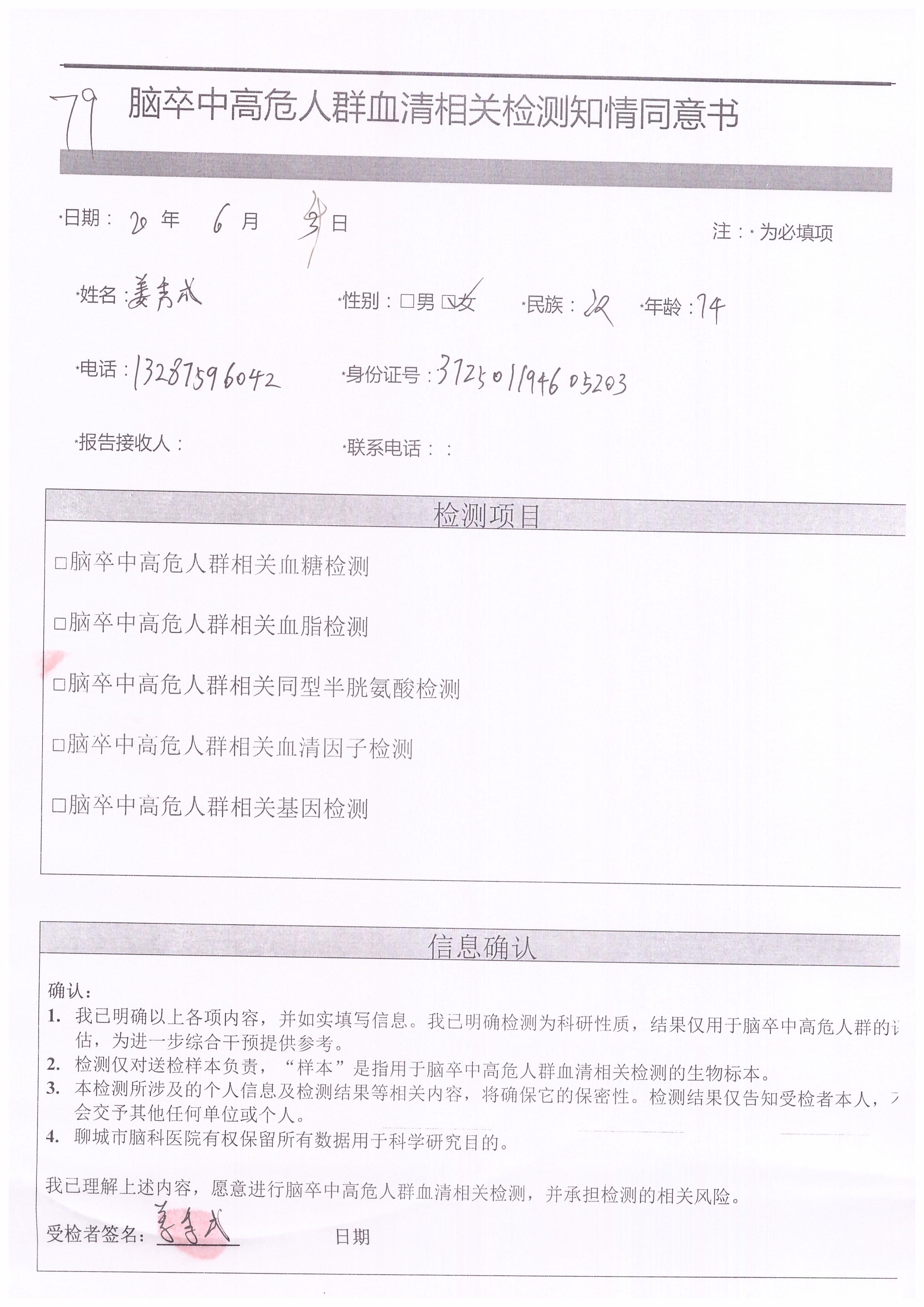

Supplement: Supplementary file 5 — Supplementary file5 (ZIP 24834 KB) [file 10528_2023_10431_MOESM5_ESM.zip › ╓¬╟Θ═1⁄4╥Γ╩Θ3/079.jpg]

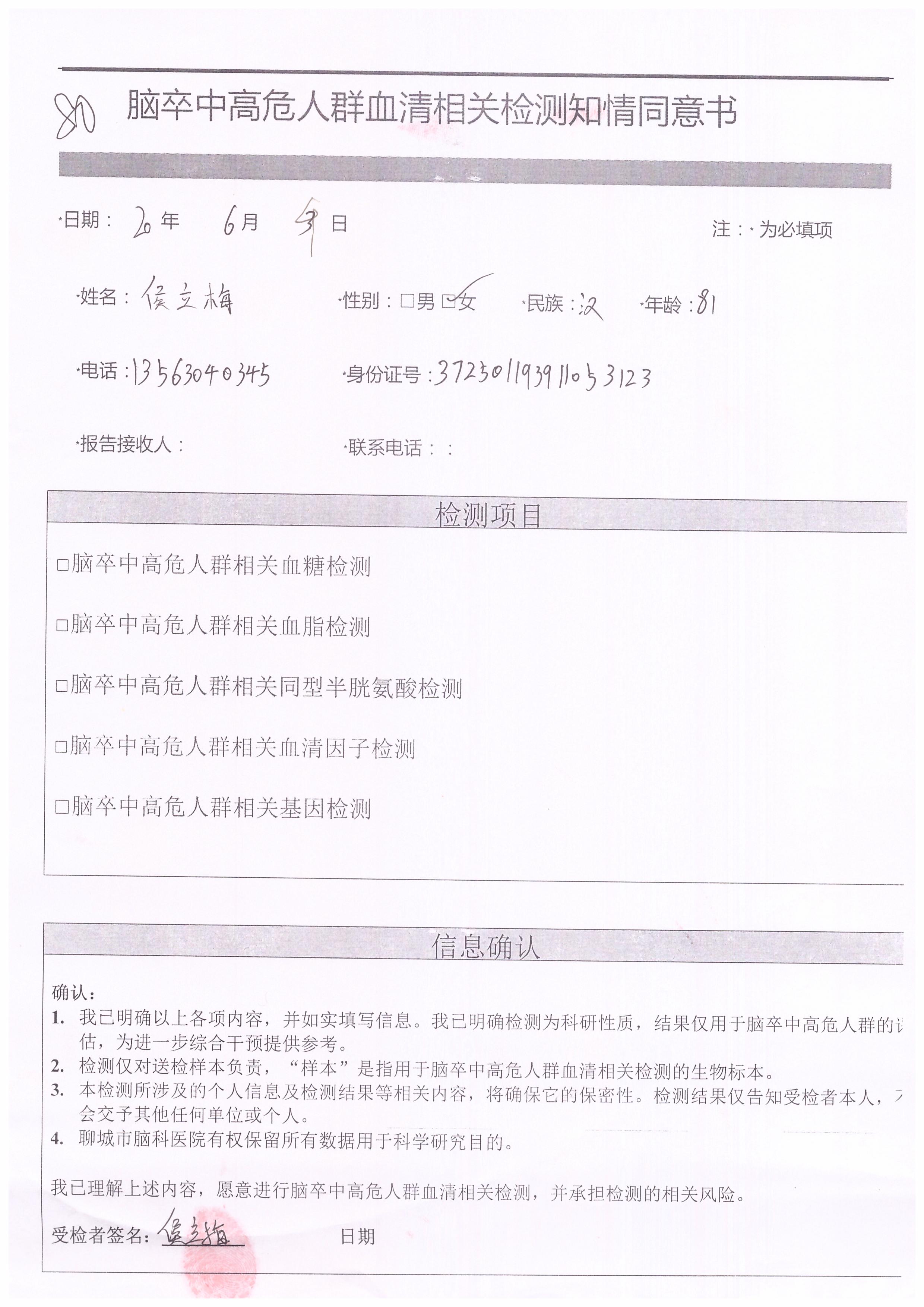

Supplement: Supplementary file 5 — Supplementary file5 (ZIP 24834 KB) [file 10528_2023_10431_MOESM5_ESM.zip › ╓¬╟Θ═1⁄4╥Γ╩Θ3/080.jpg]

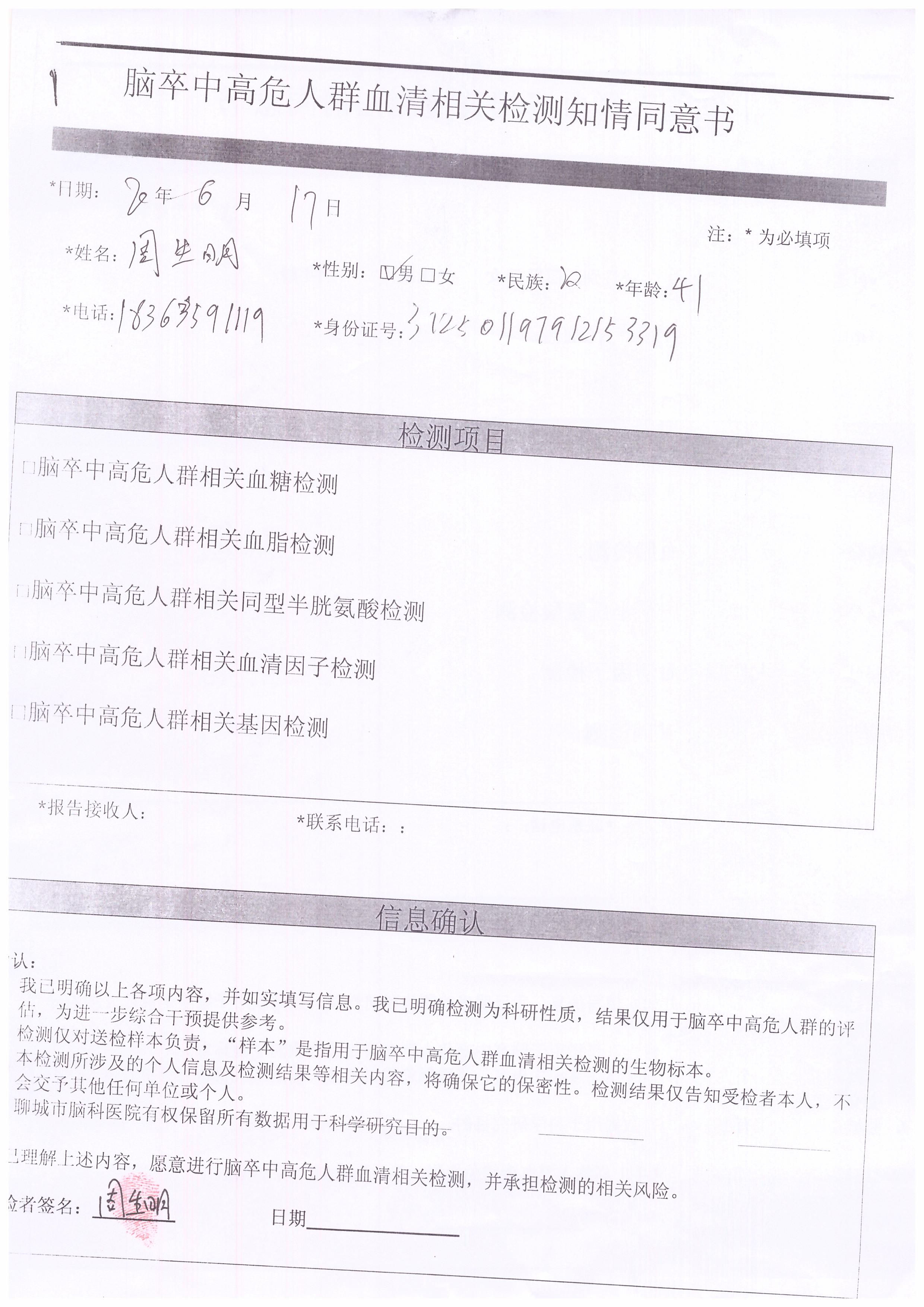

Supplement: Supplementary file 6 — Supplementary file6 (ZIP 29080 KB) [file 10528_2023_10431_MOESM6_ESM.zip › ╓¬╟Θ═1⁄4╥Γ╩Θ4/001.jpg]

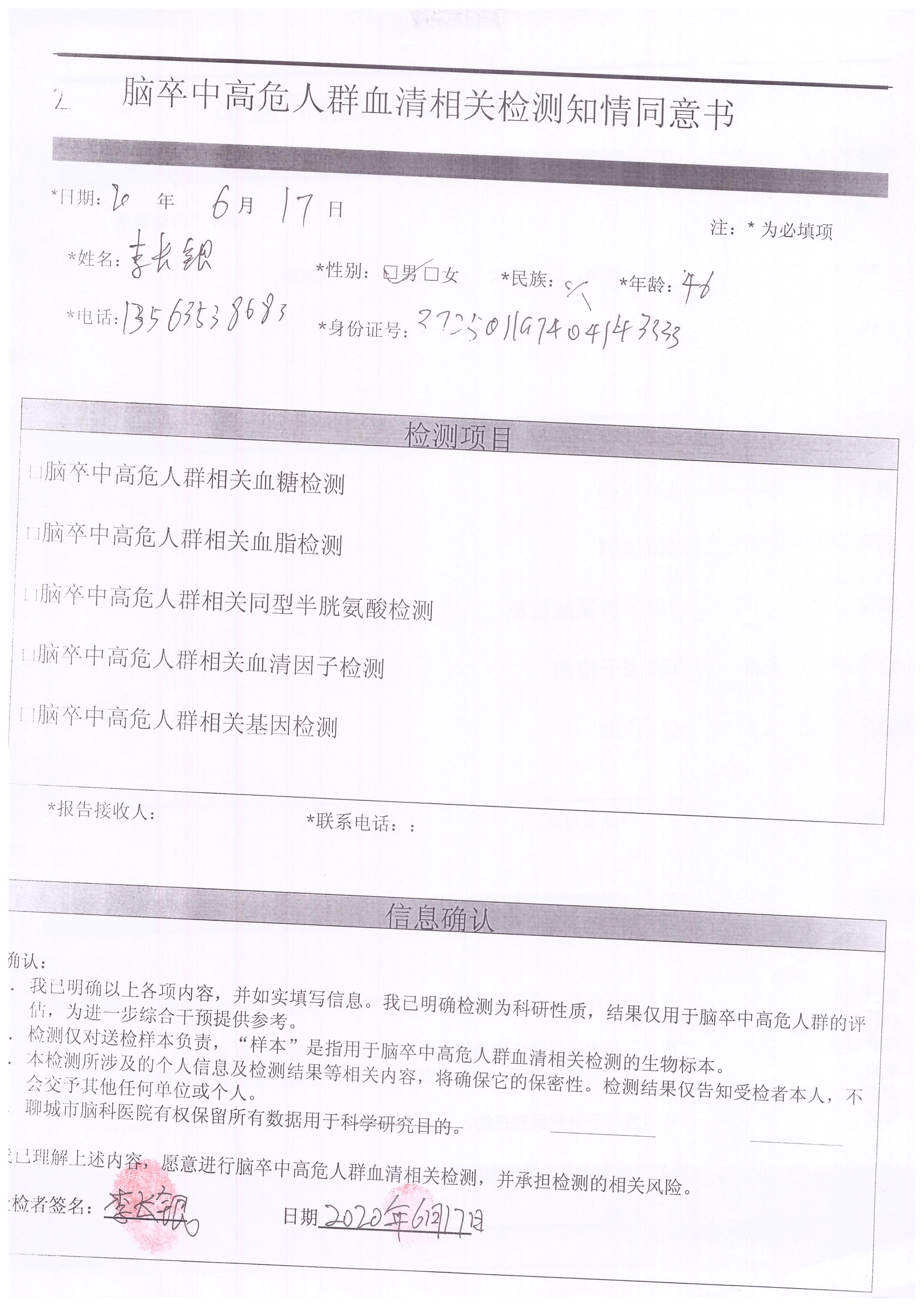

Supplement: Supplementary file 6 — Supplementary file6 (ZIP 29080 KB) [file 10528_2023_10431_MOESM6_ESM.zip › ╓¬╟Θ═1⁄4╥Γ╩Θ4/002.jpg]

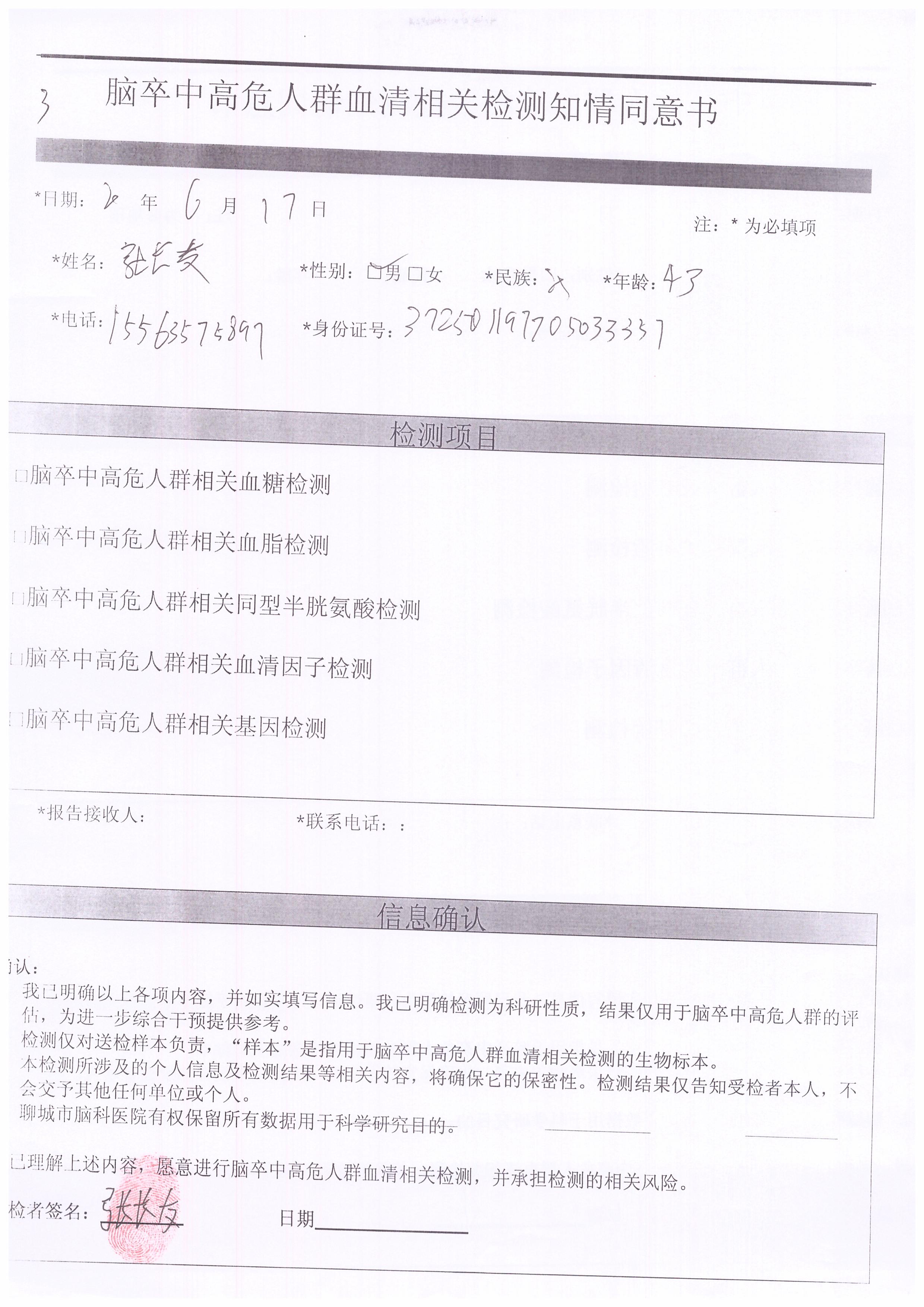

Supplement: Supplementary file 6 — Supplementary file6 (ZIP 29080 KB) [file 10528_2023_10431_MOESM6_ESM.zip › ╓¬╟Θ═1⁄4╥Γ╩Θ4/003.jpg]

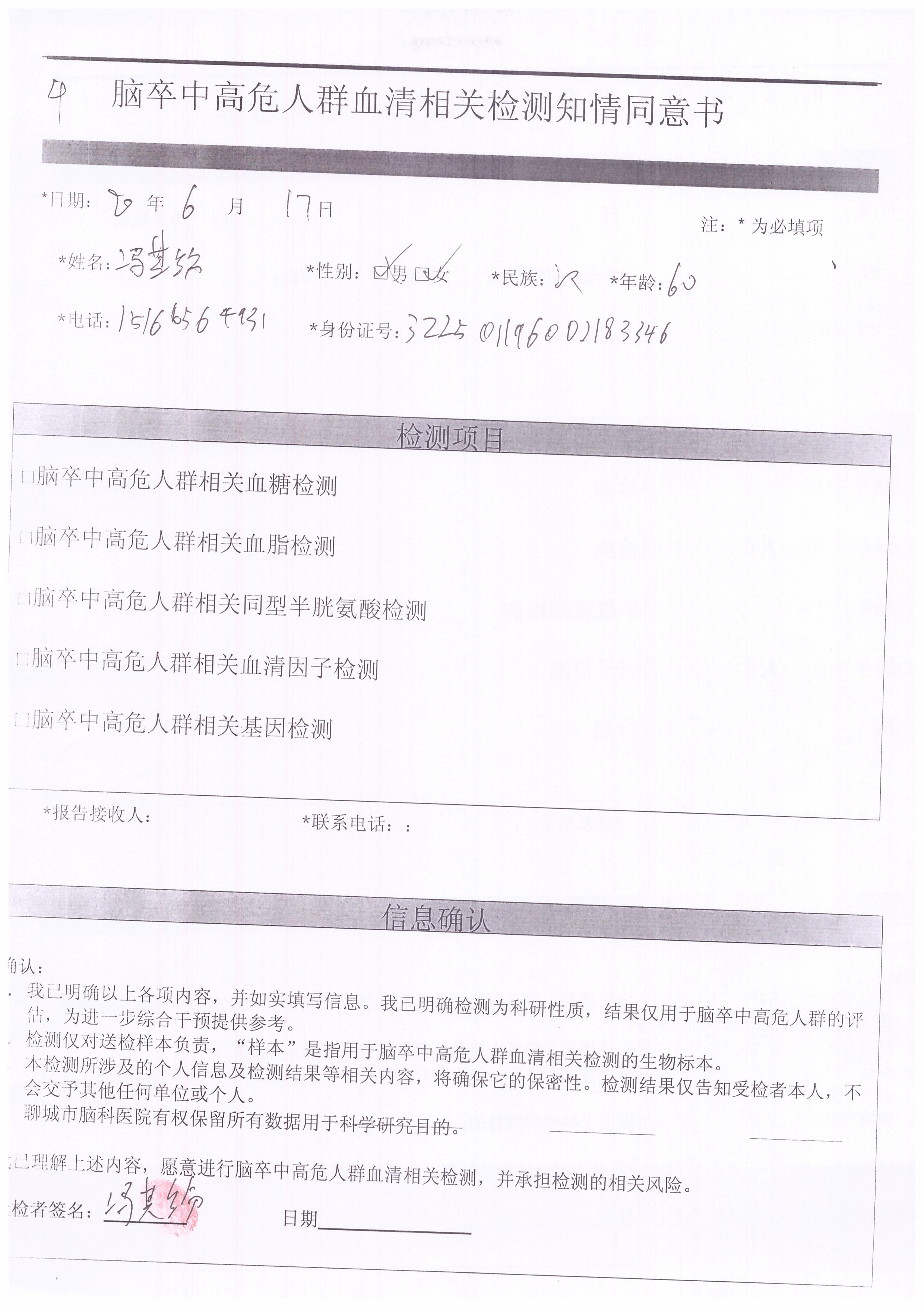

Supplement: Supplementary file 6 — Supplementary file6 (ZIP 29080 KB) [file 10528_2023_10431_MOESM6_ESM.zip › ╓¬╟Θ═1⁄4╥Γ╩Θ4/004.jpg]

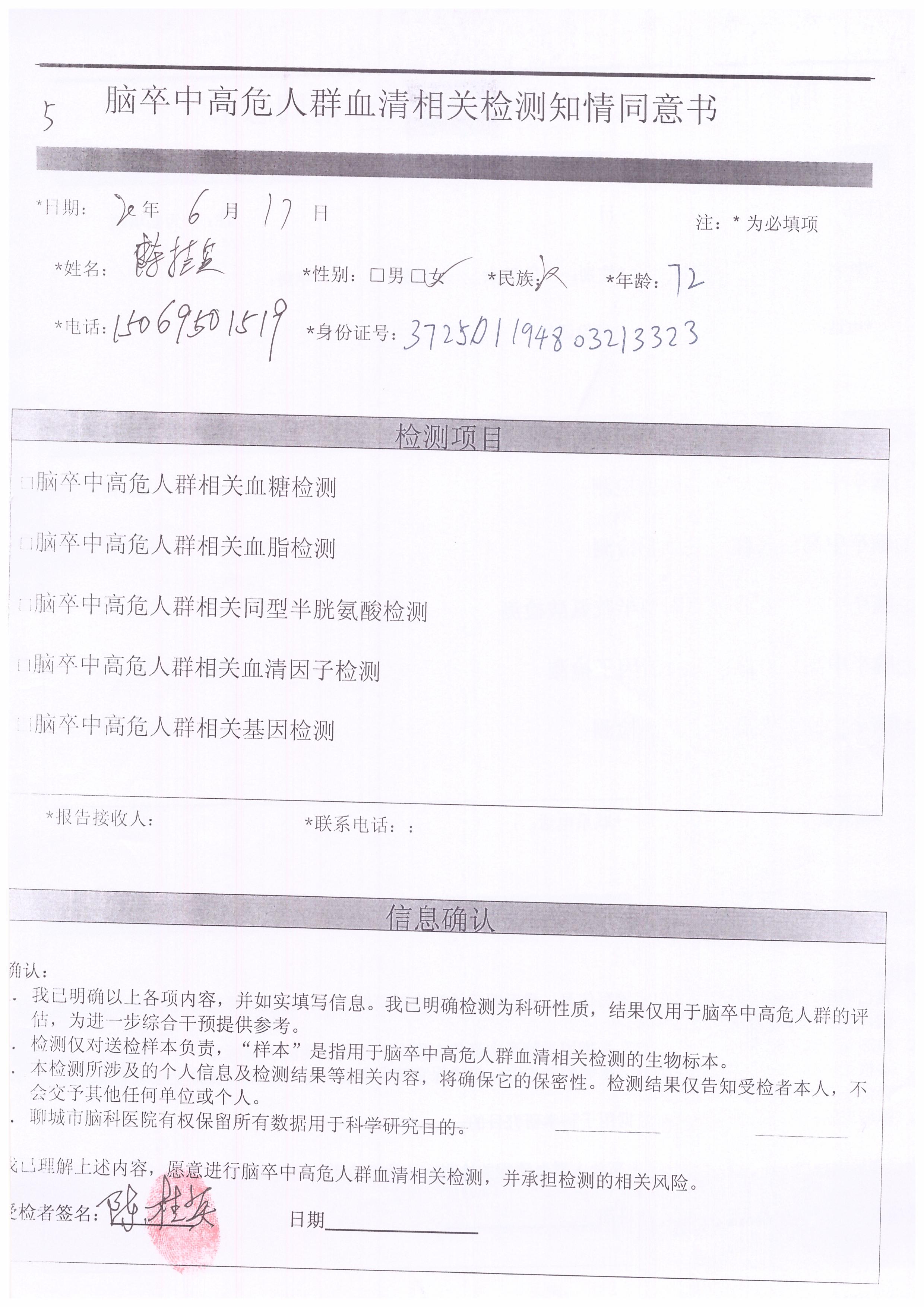

Supplement: Supplementary file 6 — Supplementary file6 (ZIP 29080 KB) [file 10528_2023_10431_MOESM6_ESM.zip › ╓¬╟Θ═1⁄4╥Γ╩Θ4/005.jpg]

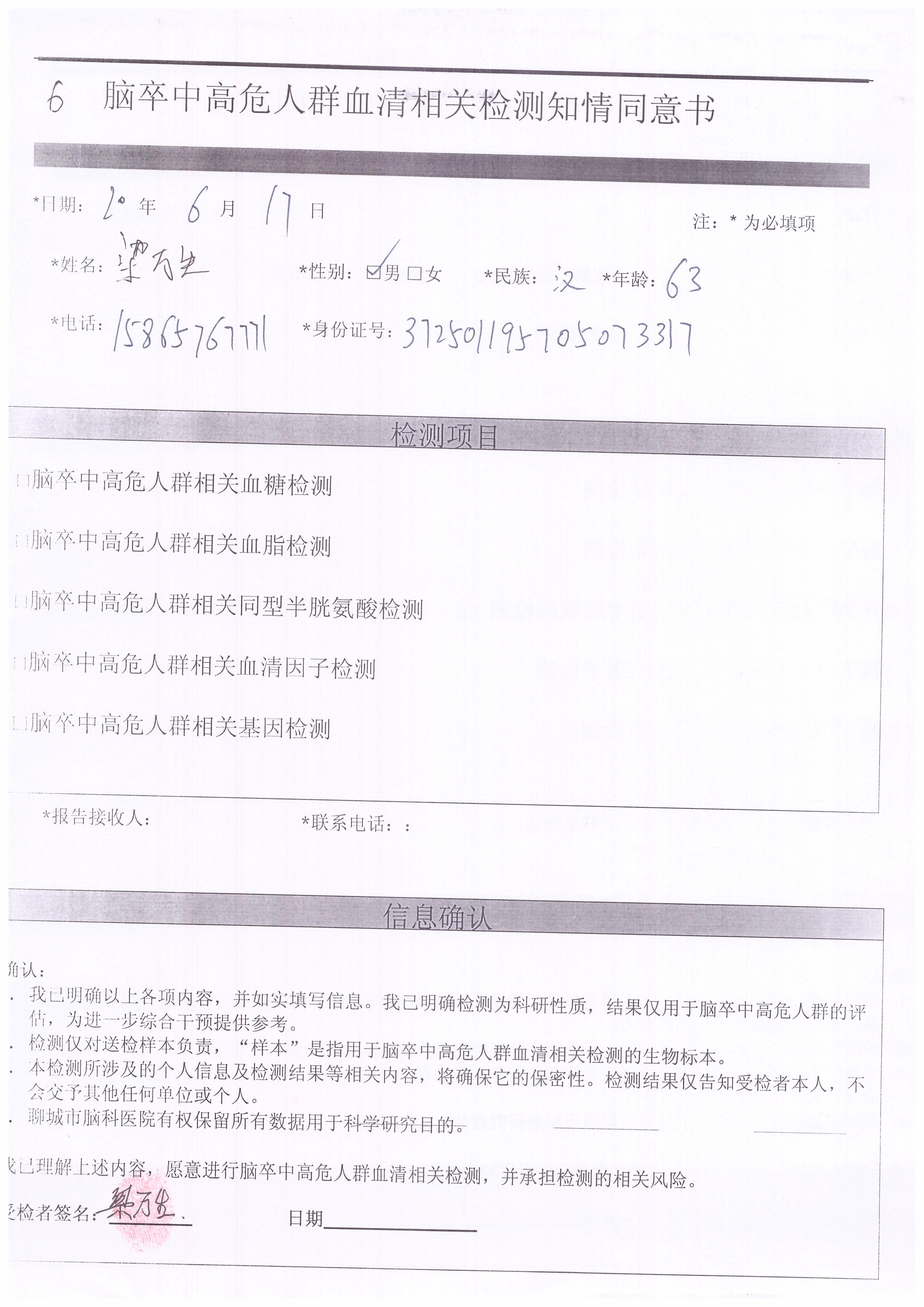

Supplement: Supplementary file 6 — Supplementary file6 (ZIP 29080 KB) [file 10528_2023_10431_MOESM6_ESM.zip › ╓¬╟Θ═1⁄4╥Γ╩Θ4/006.jpg]

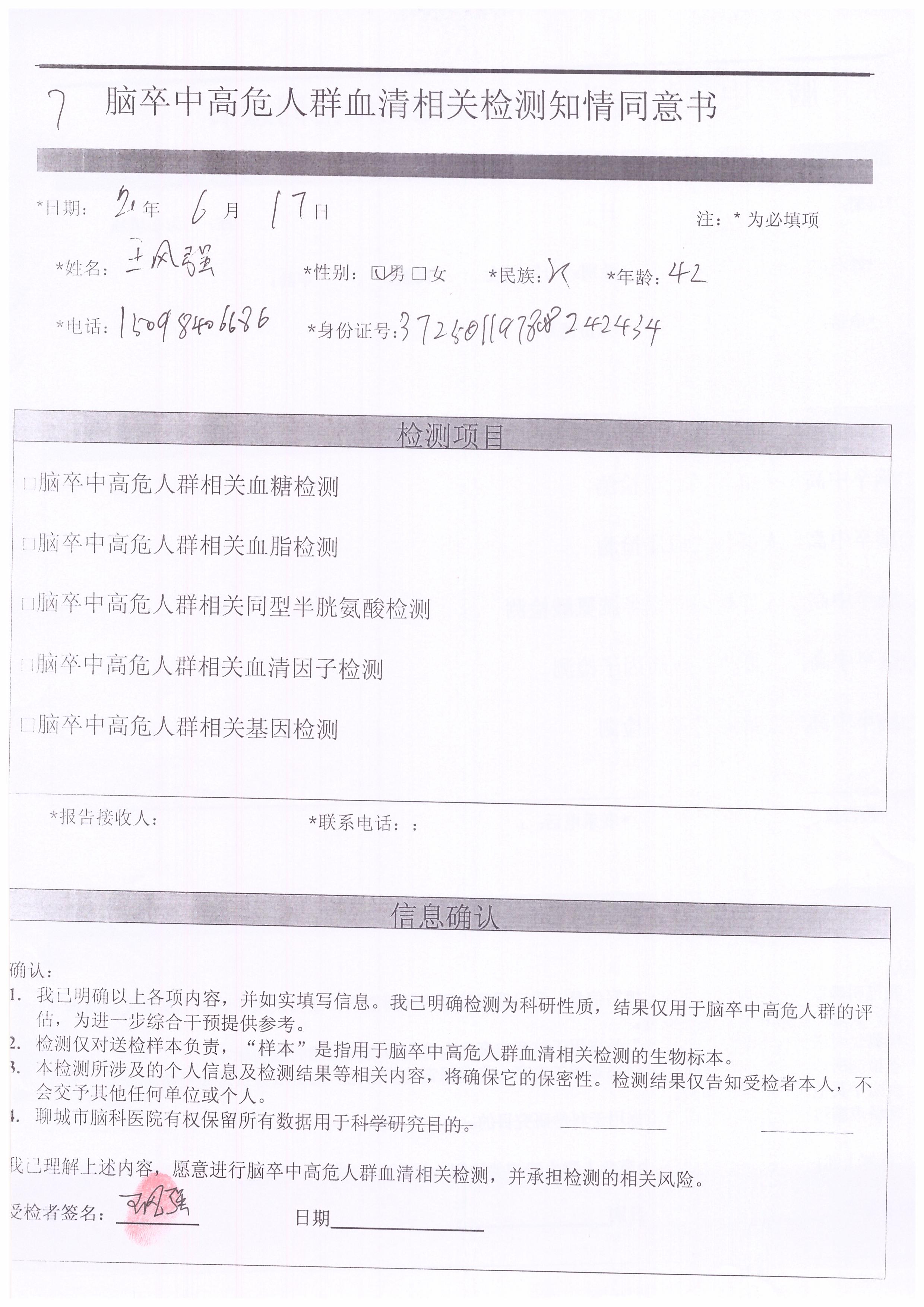

Supplement: Supplementary file 6 — Supplementary file6 (ZIP 29080 KB) [file 10528_2023_10431_MOESM6_ESM.zip › ╓¬╟Θ═1⁄4╥Γ╩Θ4/007.jpg]

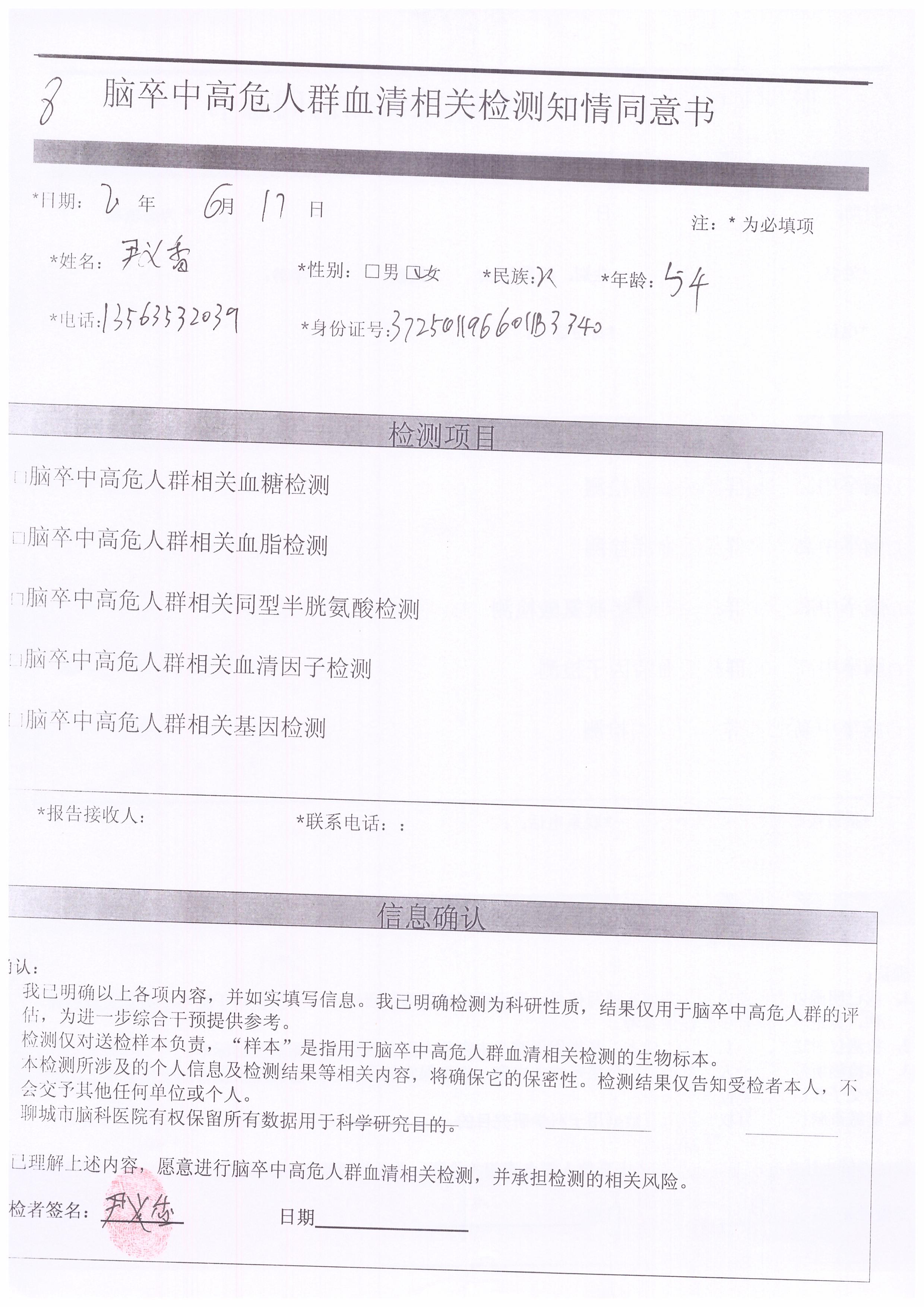

Supplement: Supplementary file 6 — Supplementary file6 (ZIP 29080 KB) [file 10528_2023_10431_MOESM6_ESM.zip › ╓¬╟Θ═1⁄4╥Γ╩Θ4/008.jpg]

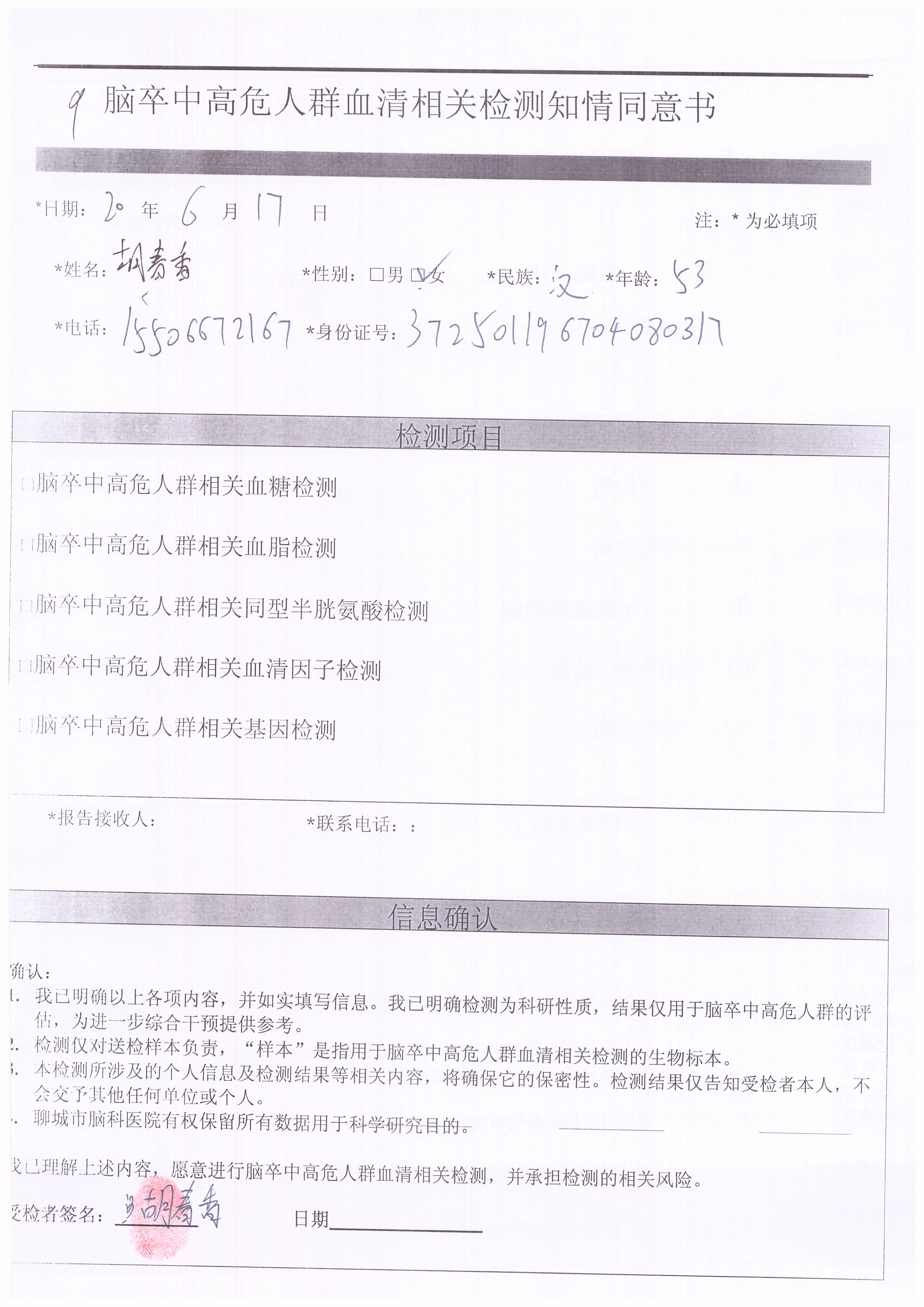

Supplement: Supplementary file 6 — Supplementary file6 (ZIP 29080 KB) [file 10528_2023_10431_MOESM6_ESM.zip › ╓¬╟Θ═1⁄4╥Γ╩Θ4/009.jpg]

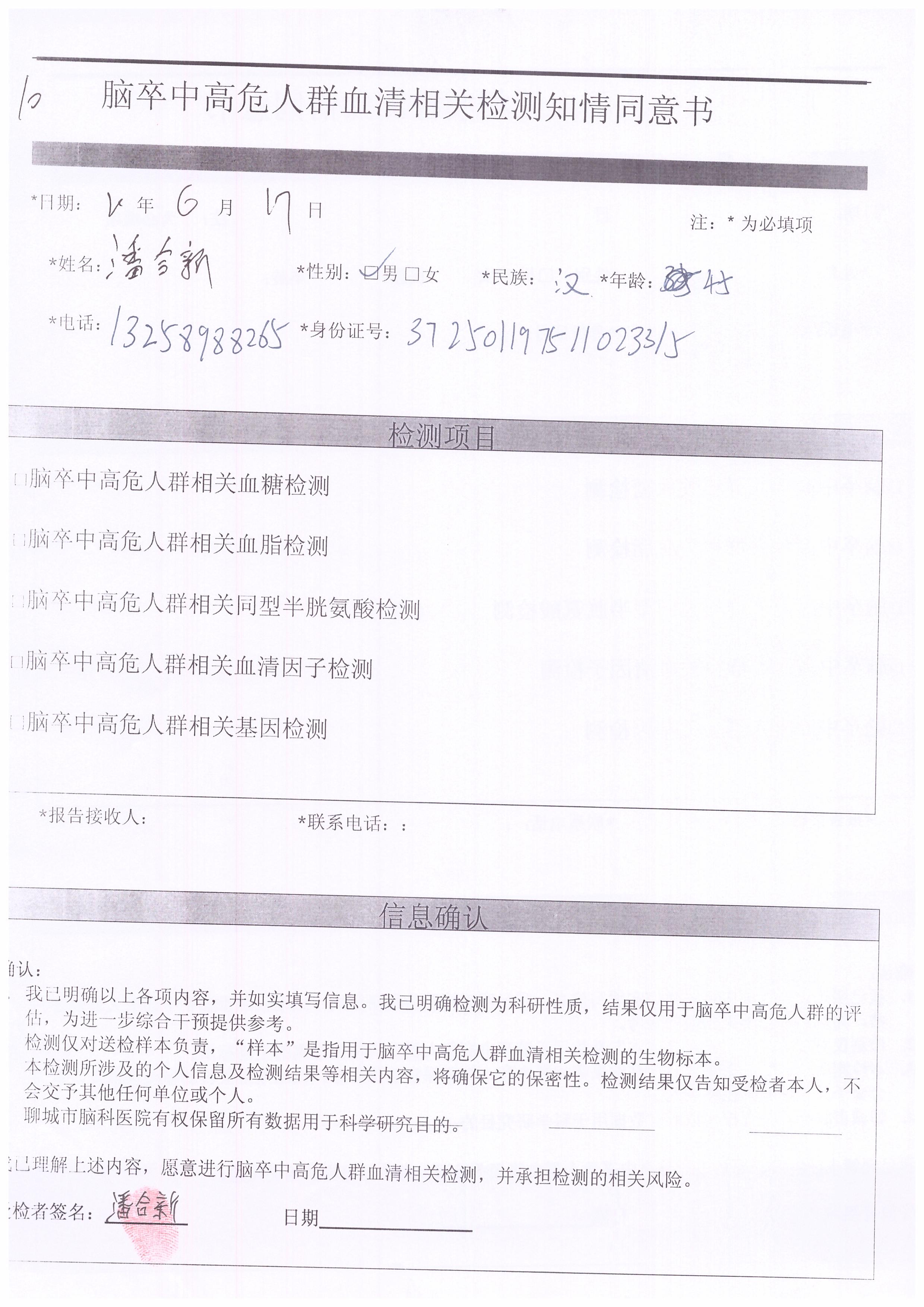

Supplement: Supplementary file 6 — Supplementary file6 (ZIP 29080 KB) [file 10528_2023_10431_MOESM6_ESM.zip › ╓¬╟Θ═1⁄4╥Γ╩Θ4/010.jpg]

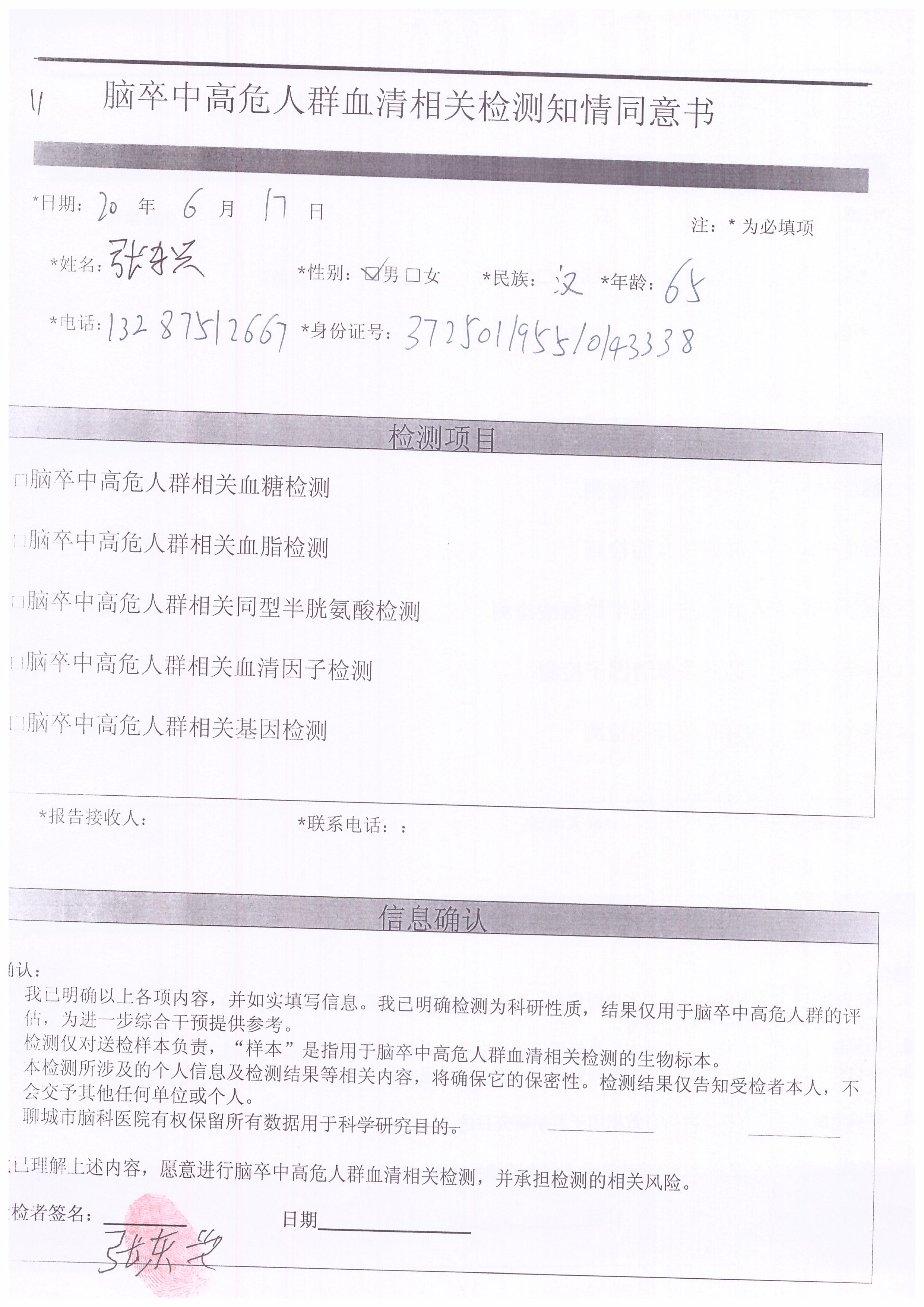

Supplement: Supplementary file 6 — Supplementary file6 (ZIP 29080 KB) [file 10528_2023_10431_MOESM6_ESM.zip › ╓¬╟Θ═1⁄4╥Γ╩Θ4/011.jpg]

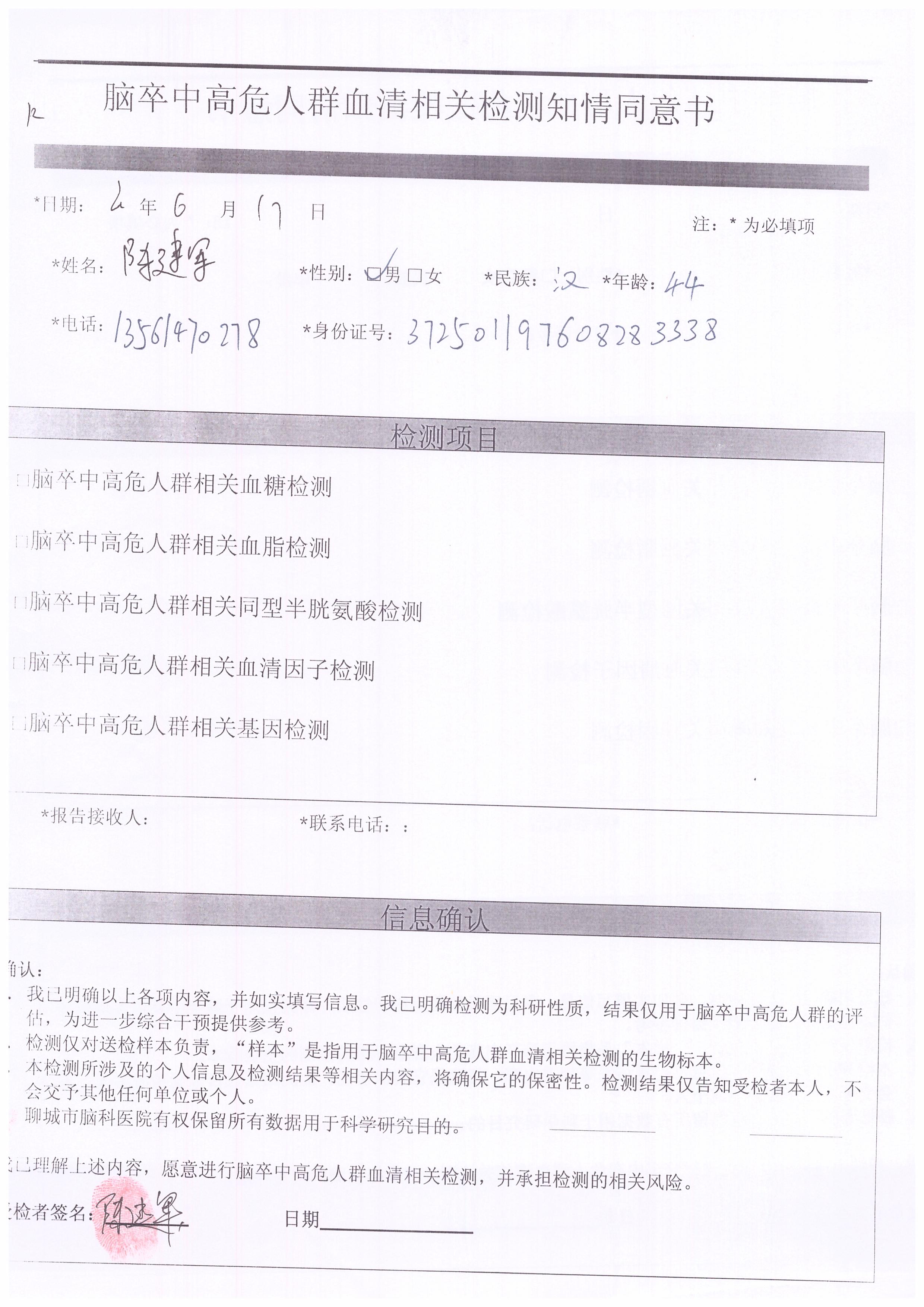

Supplement: Supplementary file 6 — Supplementary file6 (ZIP 29080 KB) [file 10528_2023_10431_MOESM6_ESM.zip › ╓¬╟Θ═1⁄4╥Γ╩Θ4/012.jpg]

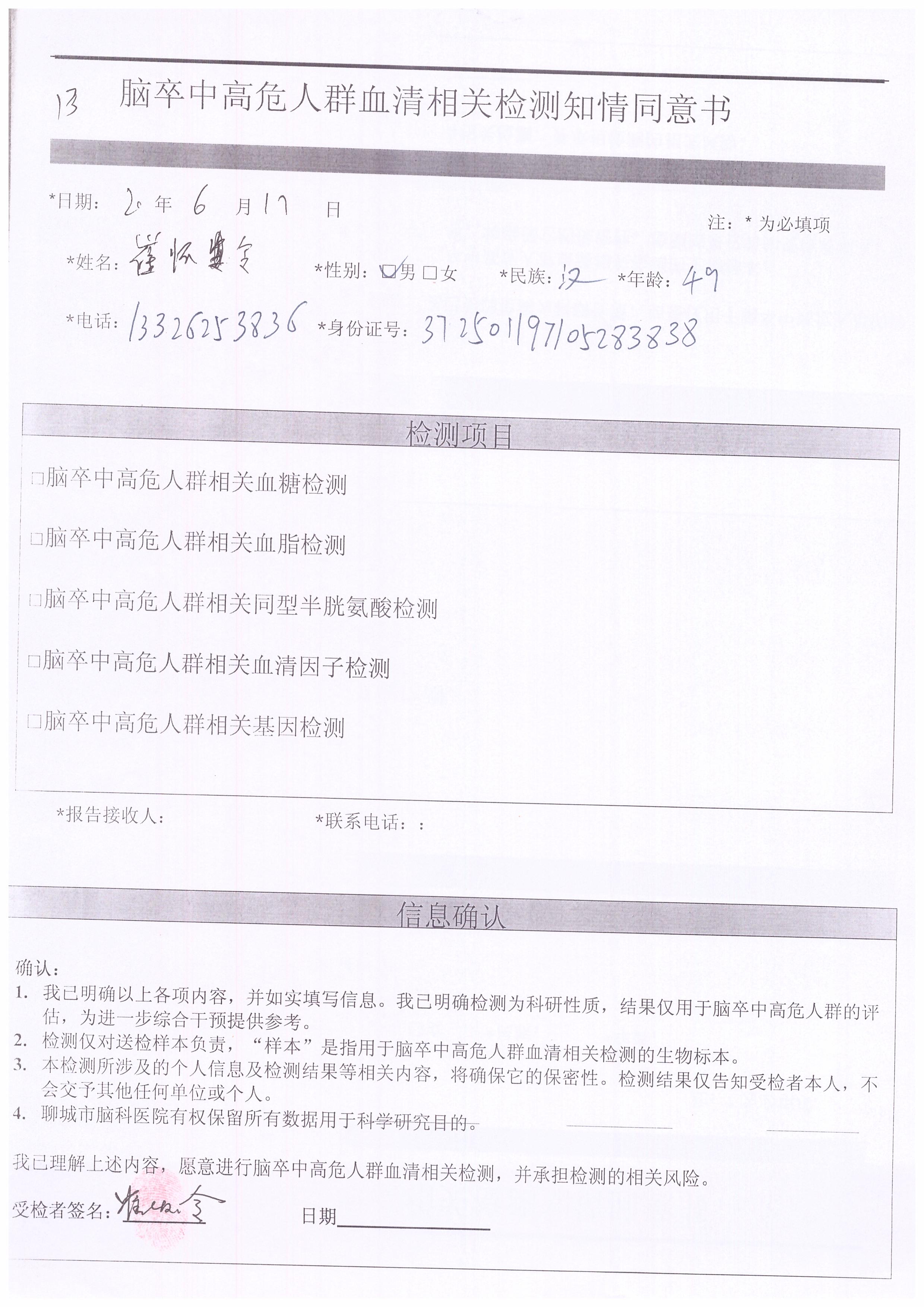

Supplement: Supplementary file 6 — Supplementary file6 (ZIP 29080 KB) [file 10528_2023_10431_MOESM6_ESM.zip › ╓¬╟Θ═1⁄4╥Γ╩Θ4/013.jpg]

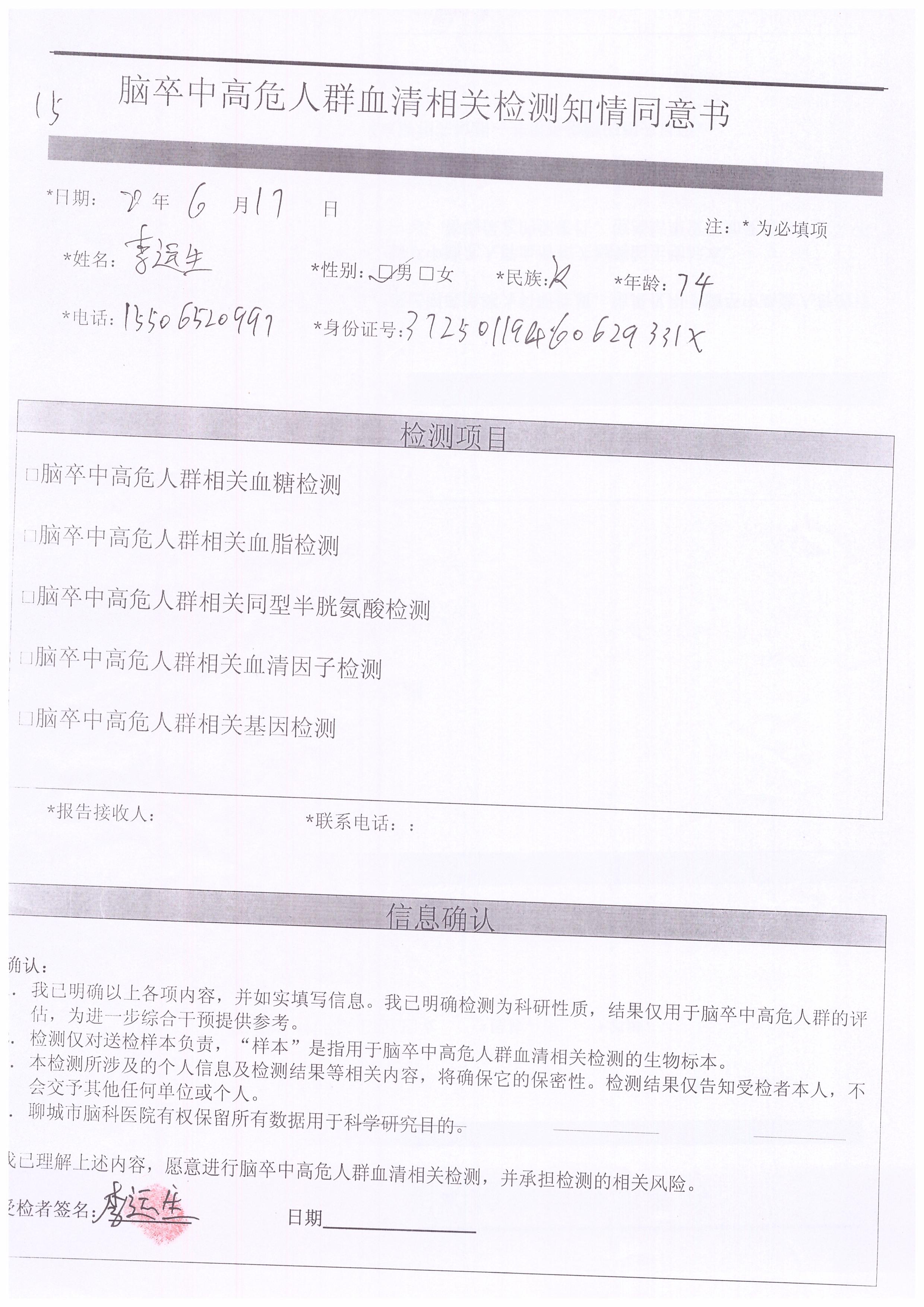

Supplement: Supplementary file 6 — Supplementary file6 (ZIP 29080 KB) [file 10528_2023_10431_MOESM6_ESM.zip › ╓¬╟Θ═1⁄4╥Γ╩Θ4/015.jpg]

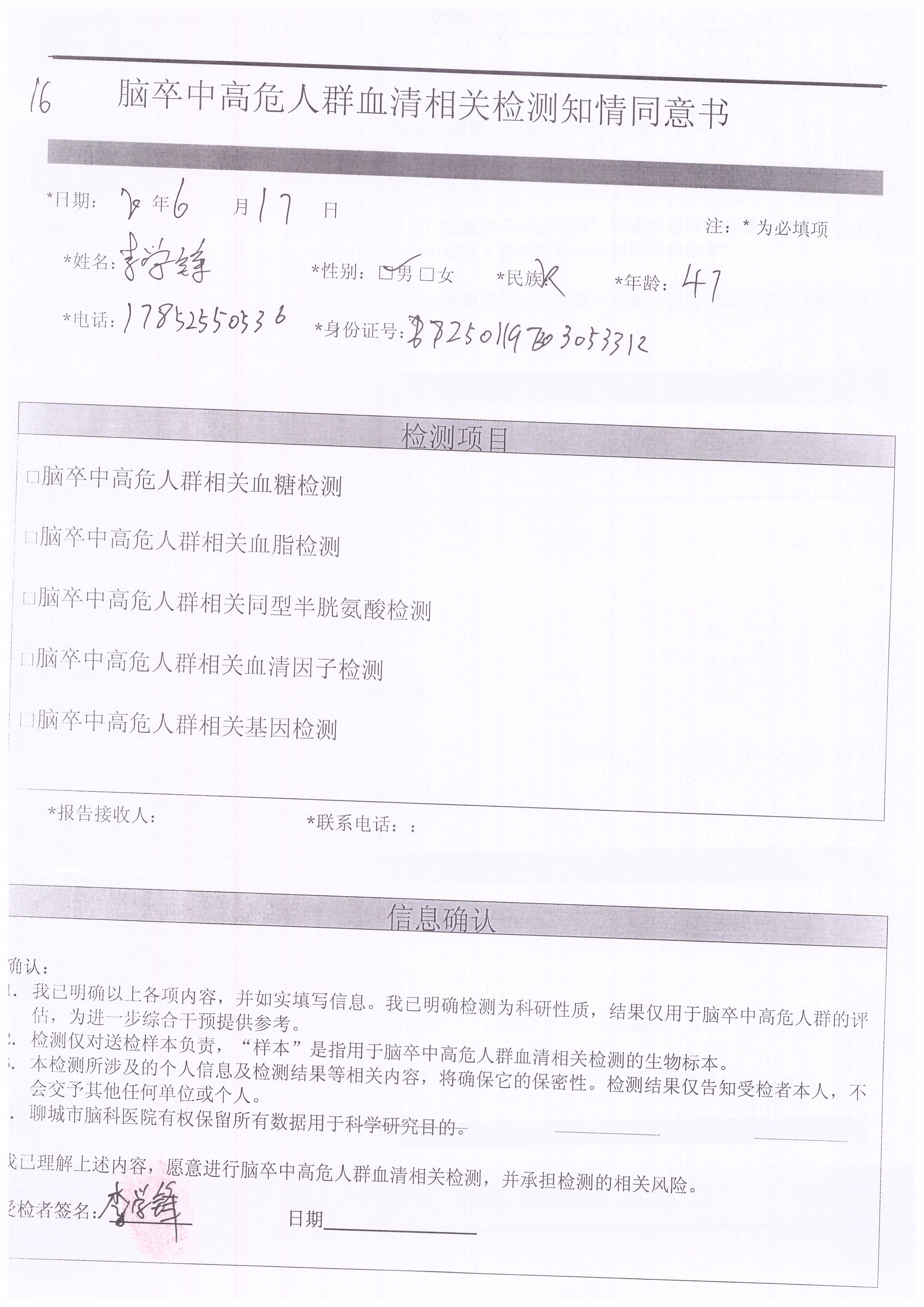

Supplement: Supplementary file 6 — Supplementary file6 (ZIP 29080 KB) [file 10528_2023_10431_MOESM6_ESM.zip › ╓¬╟Θ═1⁄4╥Γ╩Θ4/016.jpg]

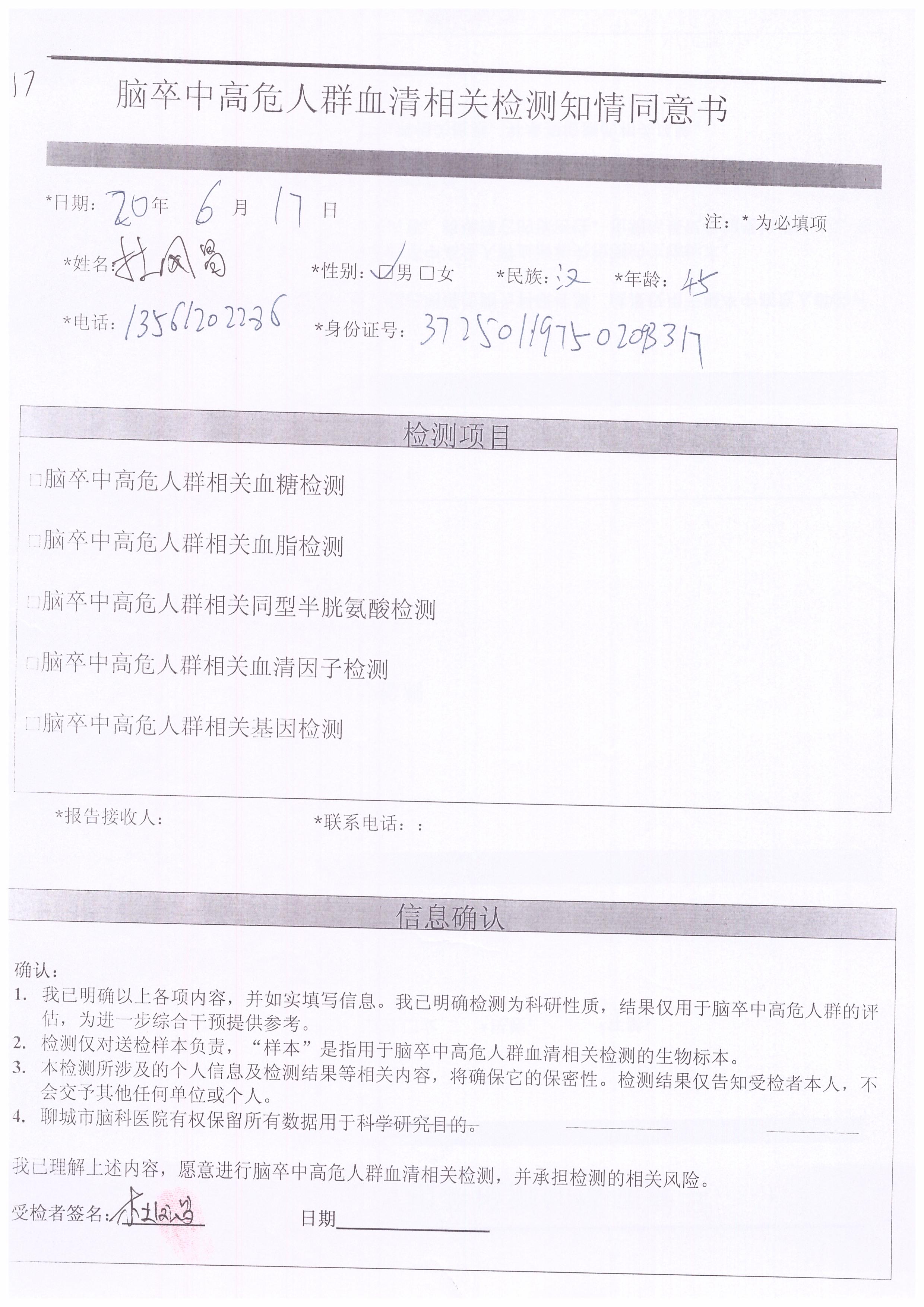

Supplement: Supplementary file 6 — Supplementary file6 (ZIP 29080 KB) [file 10528_2023_10431_MOESM6_ESM.zip › ╓¬╟Θ═1⁄4╥Γ╩Θ4/017.jpg]

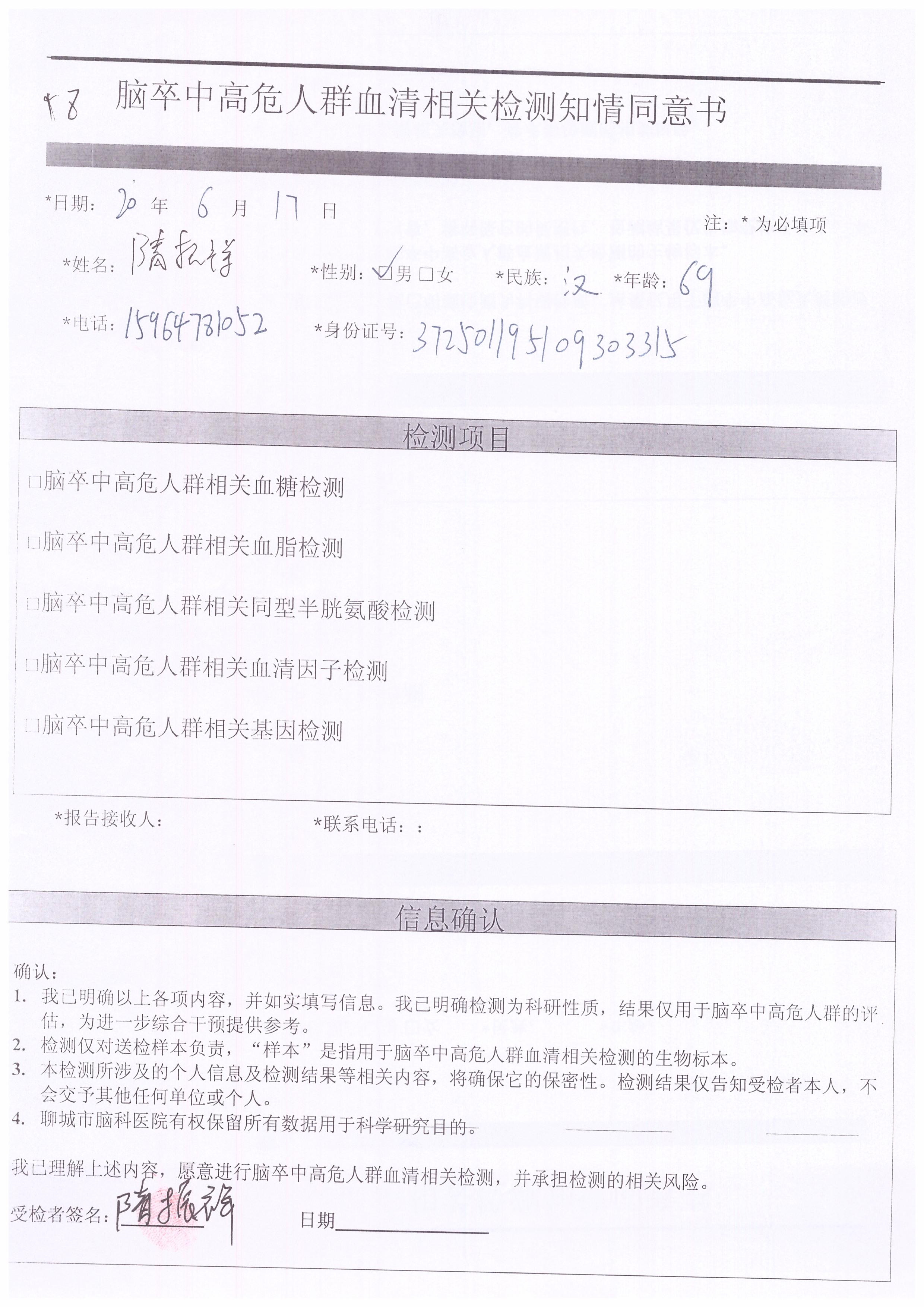

Supplement: Supplementary file 6 — Supplementary file6 (ZIP 29080 KB) [file 10528_2023_10431_MOESM6_ESM.zip › ╓¬╟Θ═1⁄4╥Γ╩Θ4/018.jpg]

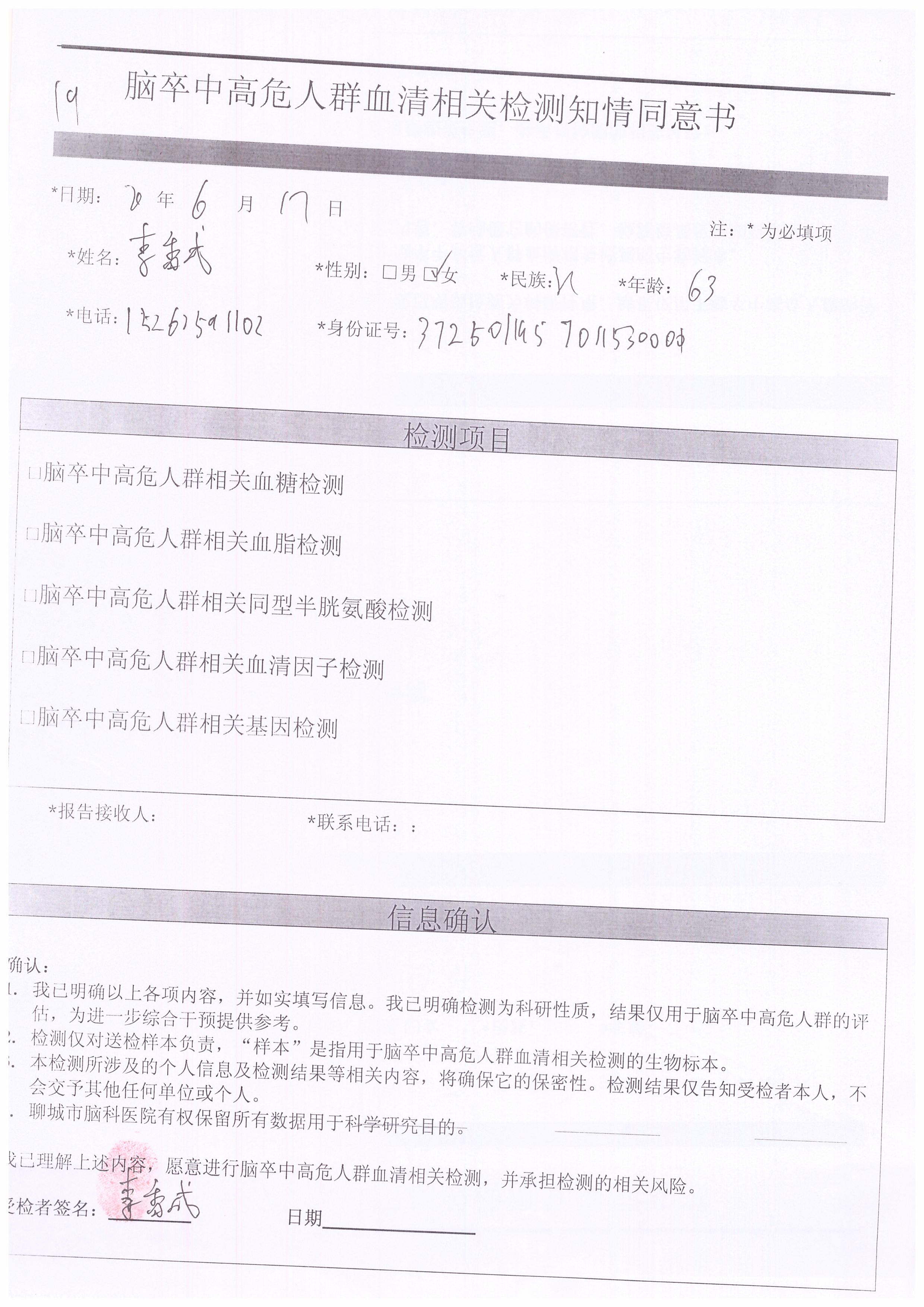

Supplement: Supplementary file 6 — Supplementary file6 (ZIP 29080 KB) [file 10528_2023_10431_MOESM6_ESM.zip › ╓¬╟Θ═1⁄4╥Γ╩Θ4/019.jpg]

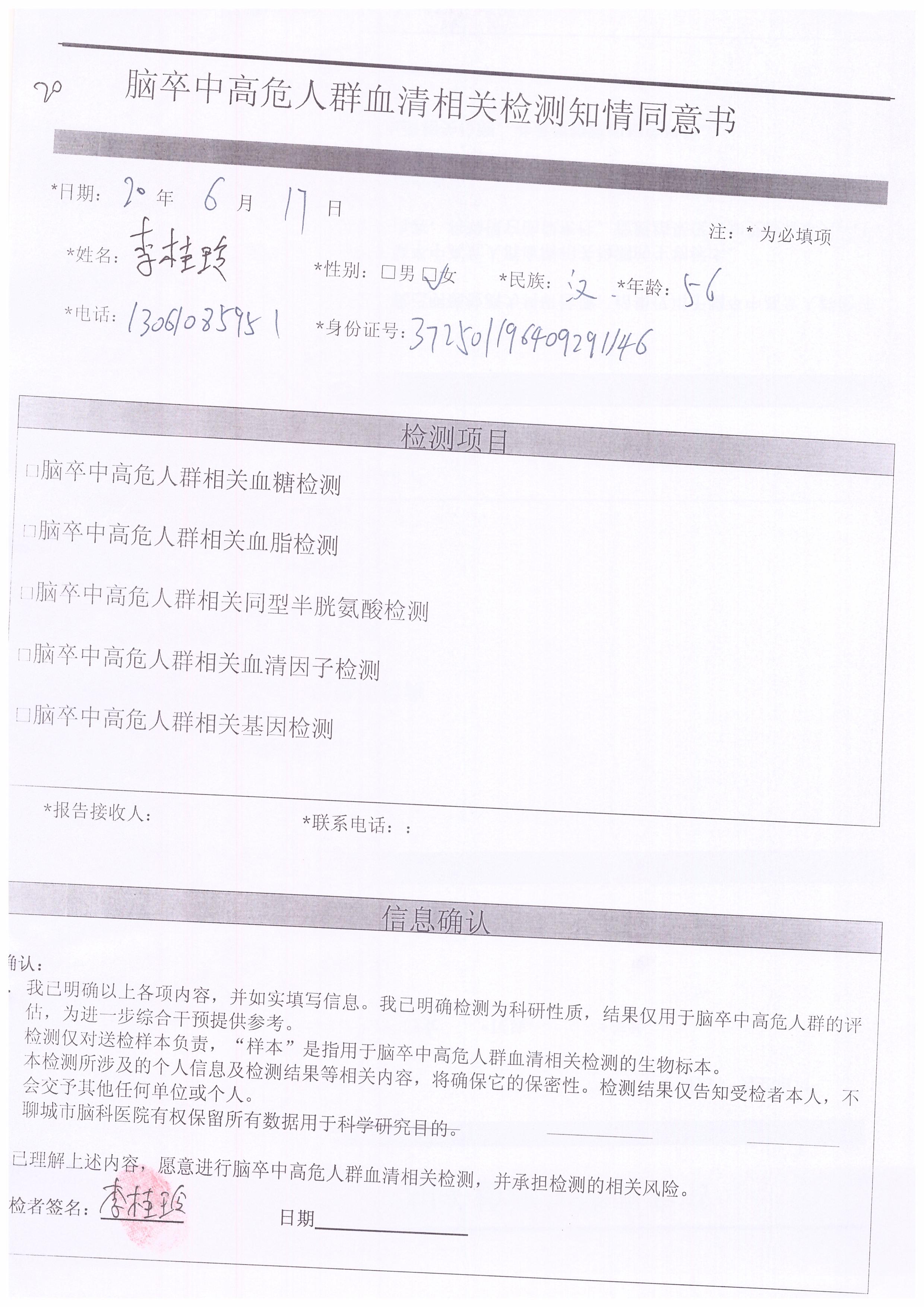

Supplement: Supplementary file 6 — Supplementary file6 (ZIP 29080 KB) [file 10528_2023_10431_MOESM6_ESM.zip › ╓¬╟Θ═1⁄4╥Γ╩Θ4/020.jpg]

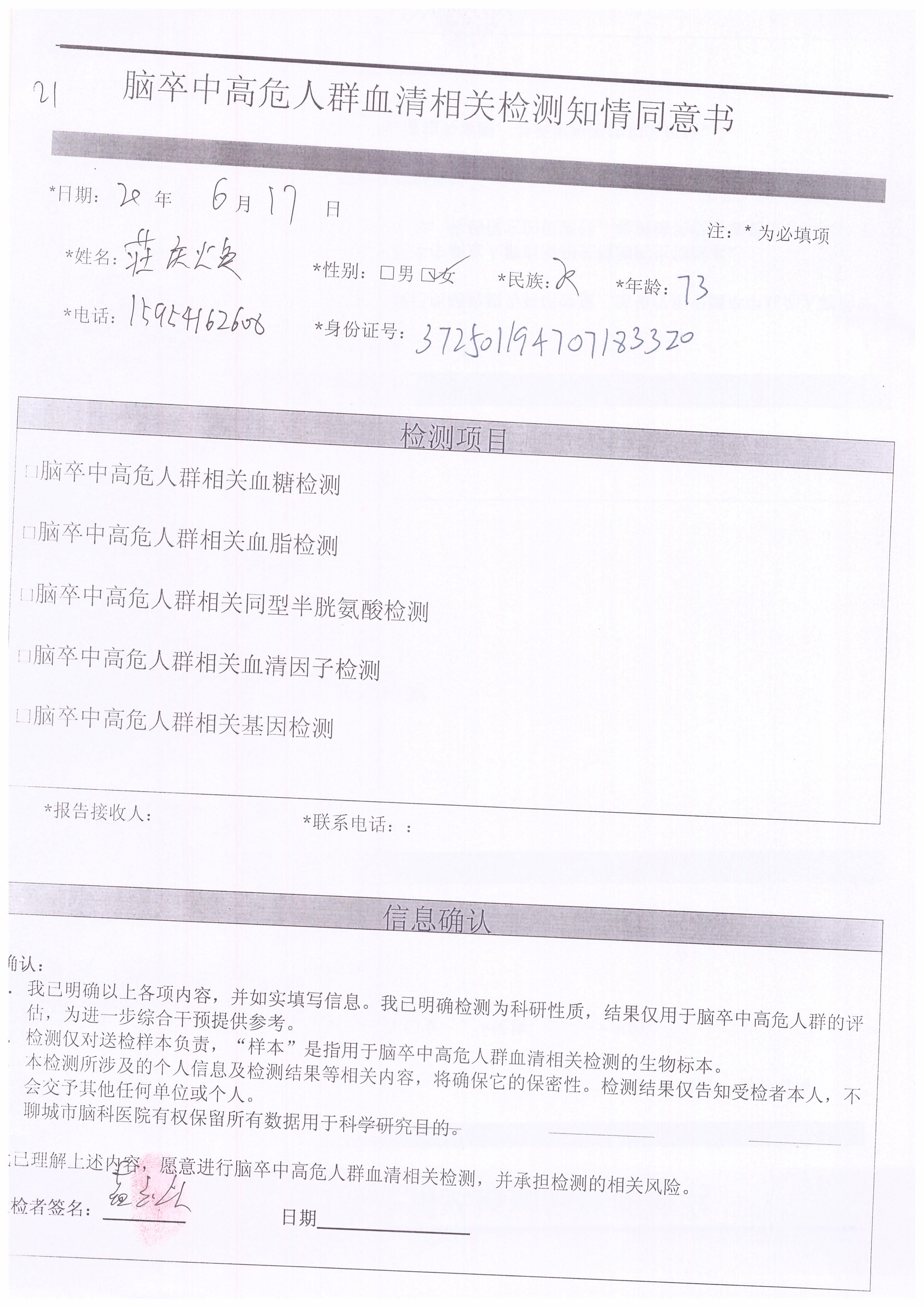

Supplement: Supplementary file 6 — Supplementary file6 (ZIP 29080 KB) [file 10528_2023_10431_MOESM6_ESM.zip › ╓¬╟Θ═1⁄4╥Γ╩Θ4/021.jpg]

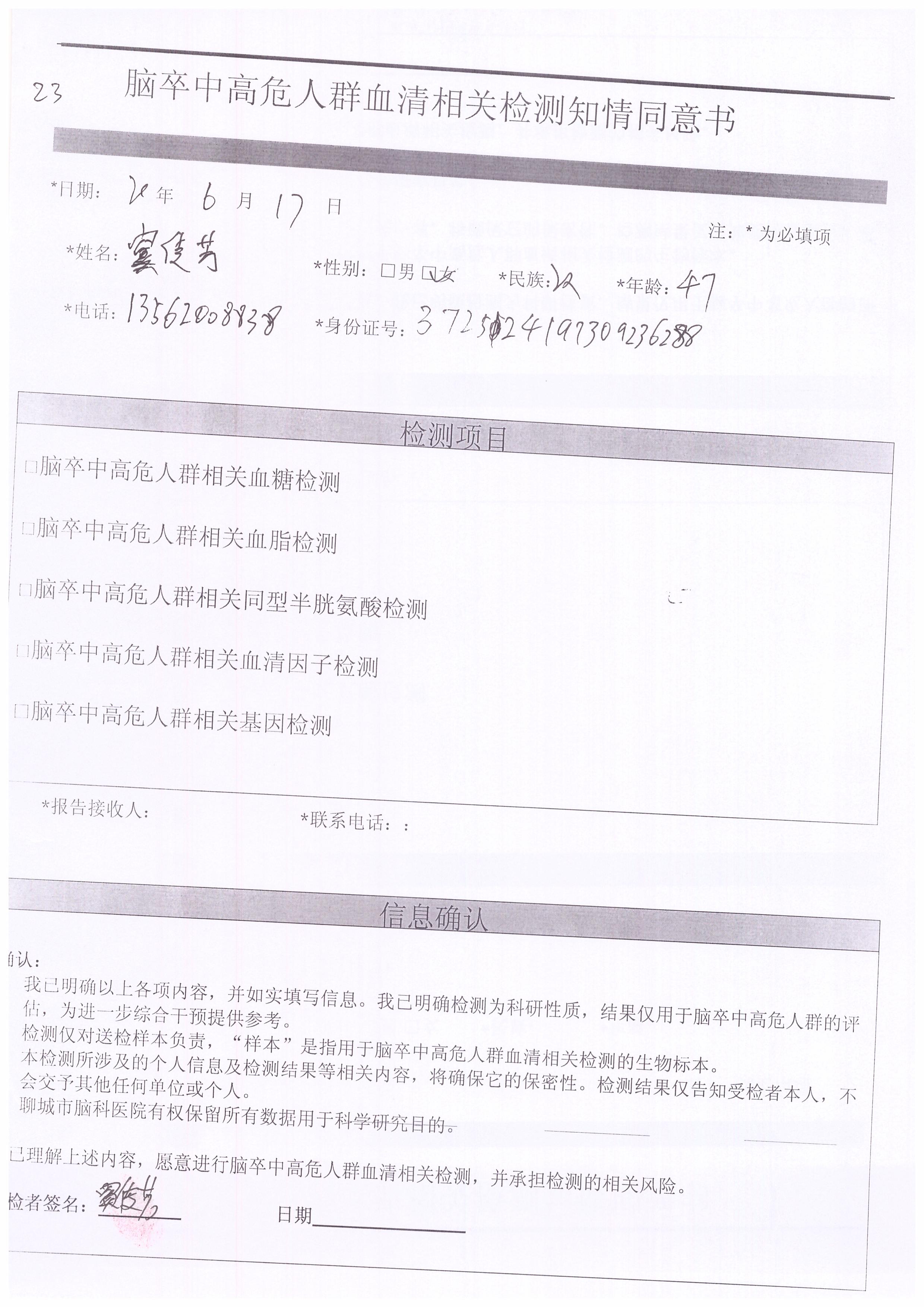

Supplement: Supplementary file 6 — Supplementary file6 (ZIP 29080 KB) [file 10528_2023_10431_MOESM6_ESM.zip › ╓¬╟Θ═1⁄4╥Γ╩Θ4/023.jpg]

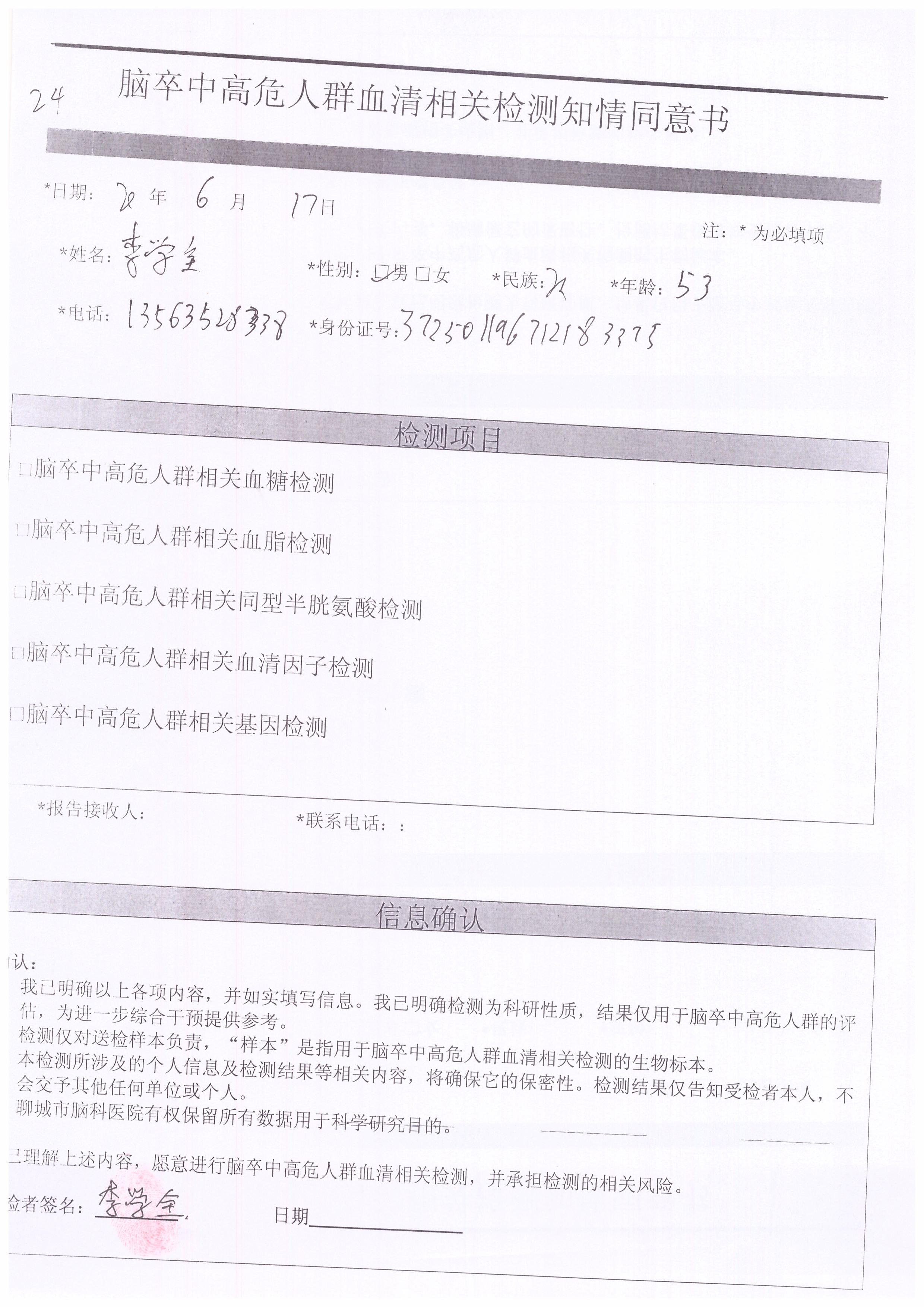

Supplement: Supplementary file 6 — Supplementary file6 (ZIP 29080 KB) [file 10528_2023_10431_MOESM6_ESM.zip › ╓¬╟Θ═1⁄4╥Γ╩Θ4/024.jpg]

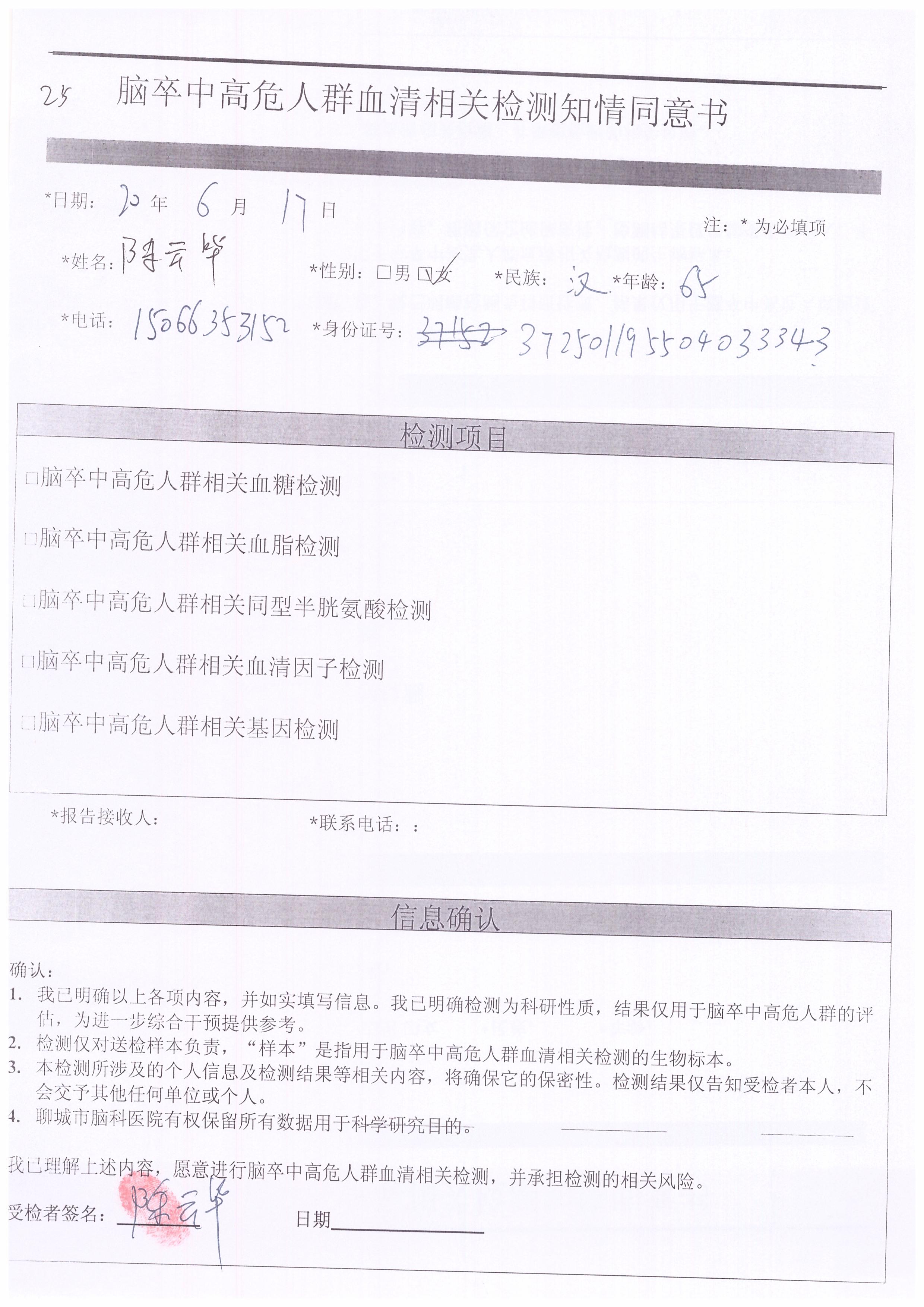

Supplement: Supplementary file 6 — Supplementary file6 (ZIP 29080 KB) [file 10528_2023_10431_MOESM6_ESM.zip › ╓¬╟Θ═1⁄4╥Γ╩Θ4/025.jpg]

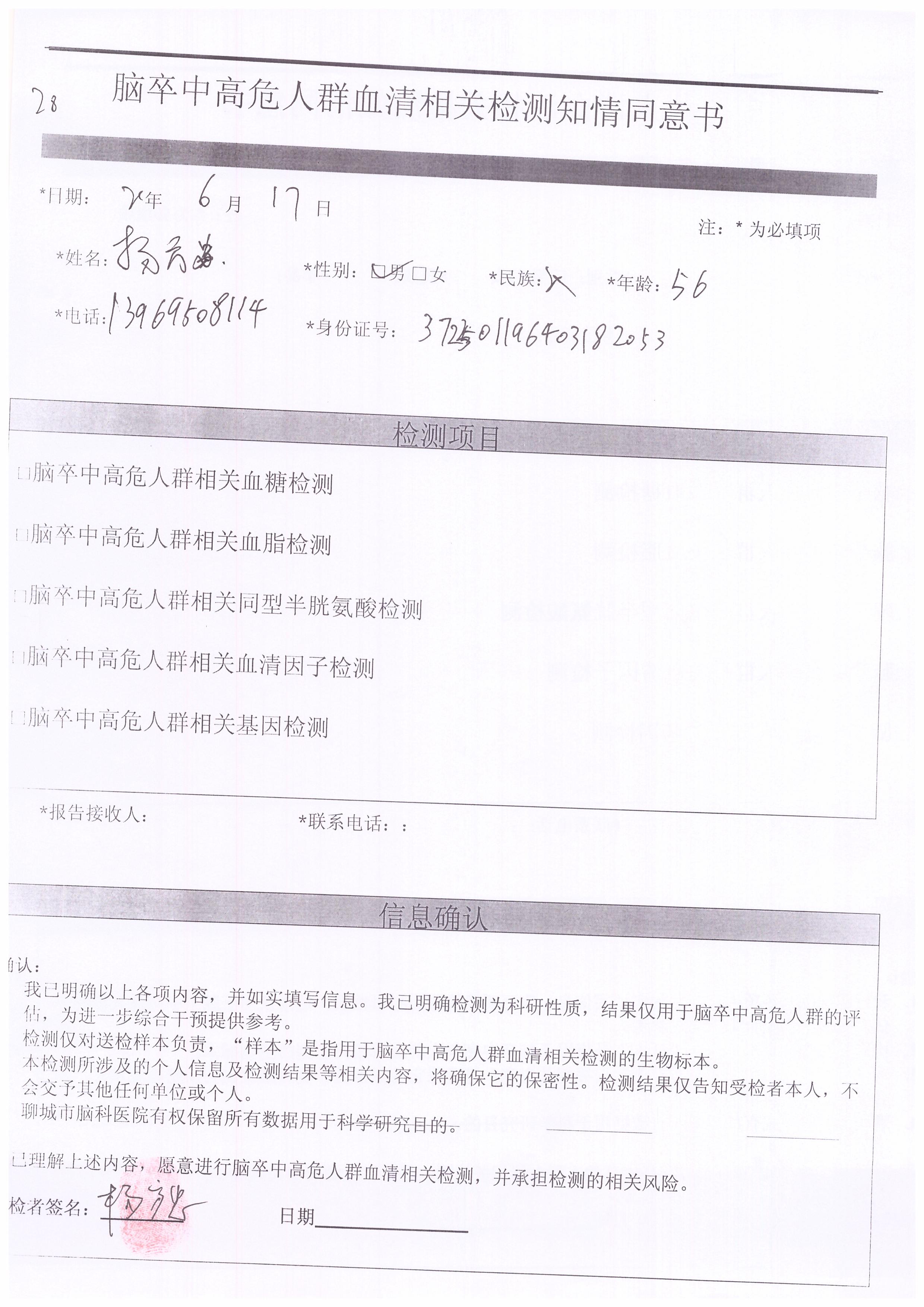

Supplement: Supplementary file 6 — Supplementary file6 (ZIP 29080 KB) [file 10528_2023_10431_MOESM6_ESM.zip › ╓¬╟Θ═1⁄4╥Γ╩Θ4/028.jpg]

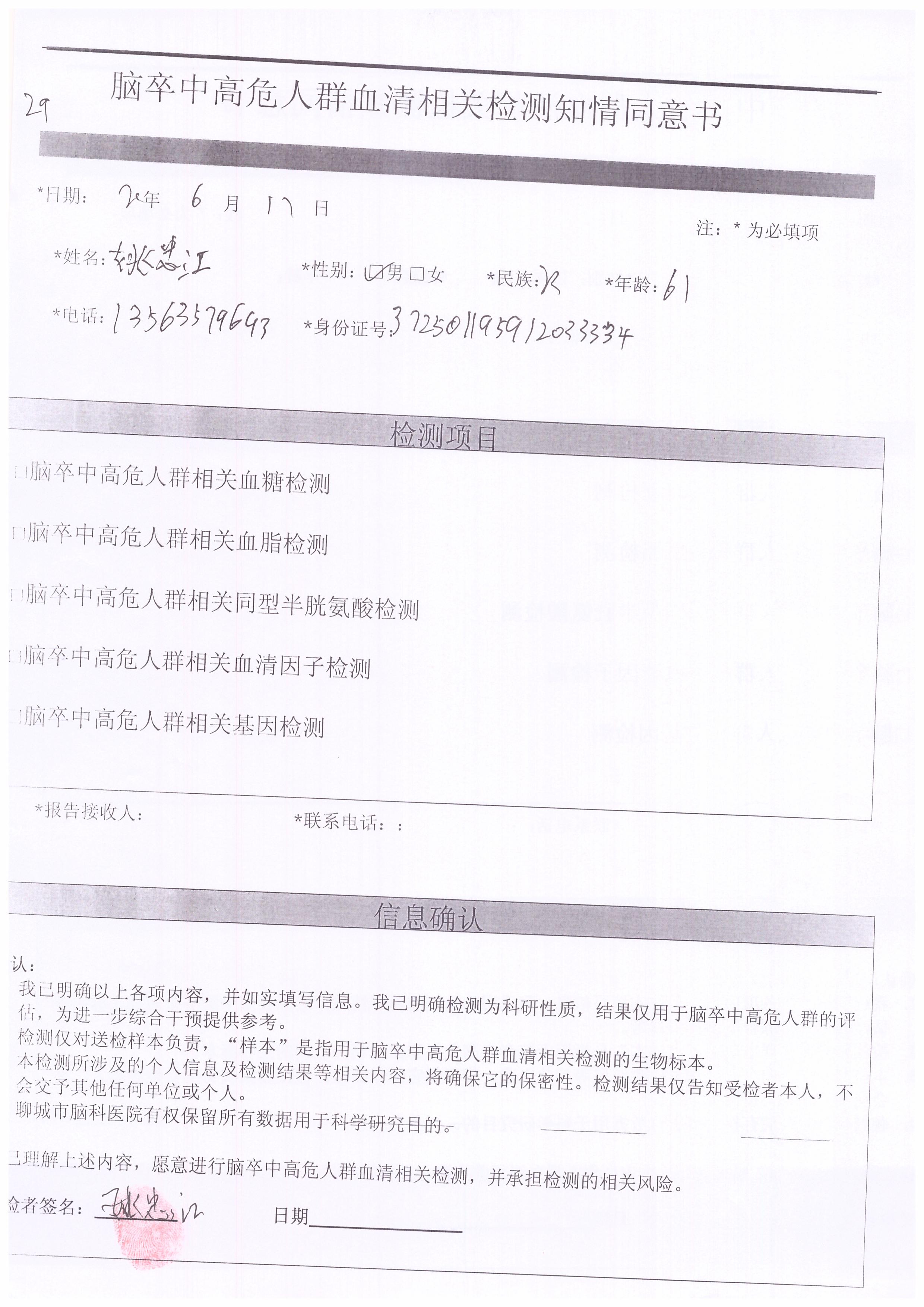

Supplement: Supplementary file 6 — Supplementary file6 (ZIP 29080 KB) [file 10528_2023_10431_MOESM6_ESM.zip › ╓¬╟Θ═1⁄4╥Γ╩Θ4/029.jpg]

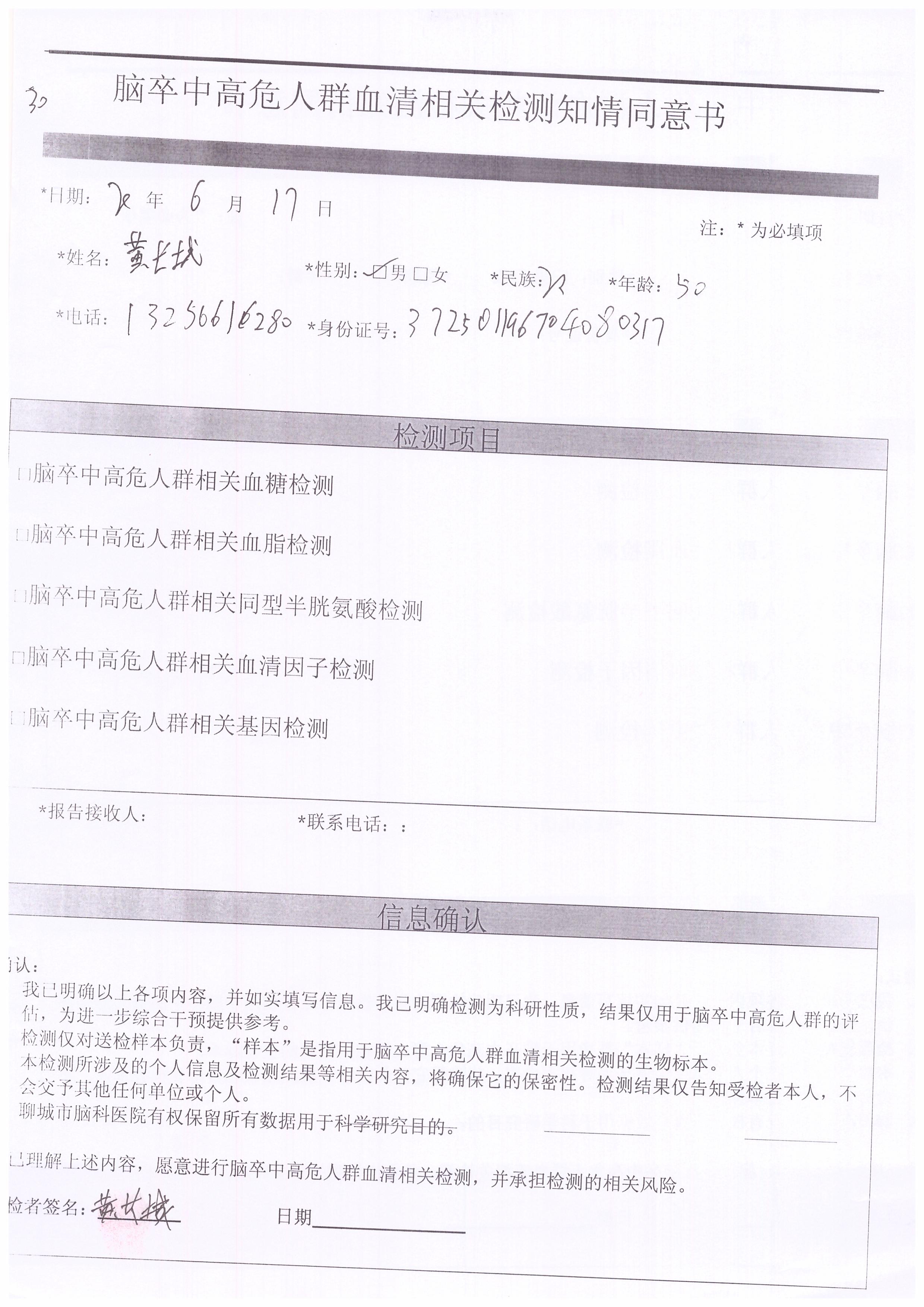

Supplement: Supplementary file 6 — Supplementary file6 (ZIP 29080 KB) [file 10528_2023_10431_MOESM6_ESM.zip › ╓¬╟Θ═1⁄4╥Γ╩Θ4/030.jpg]

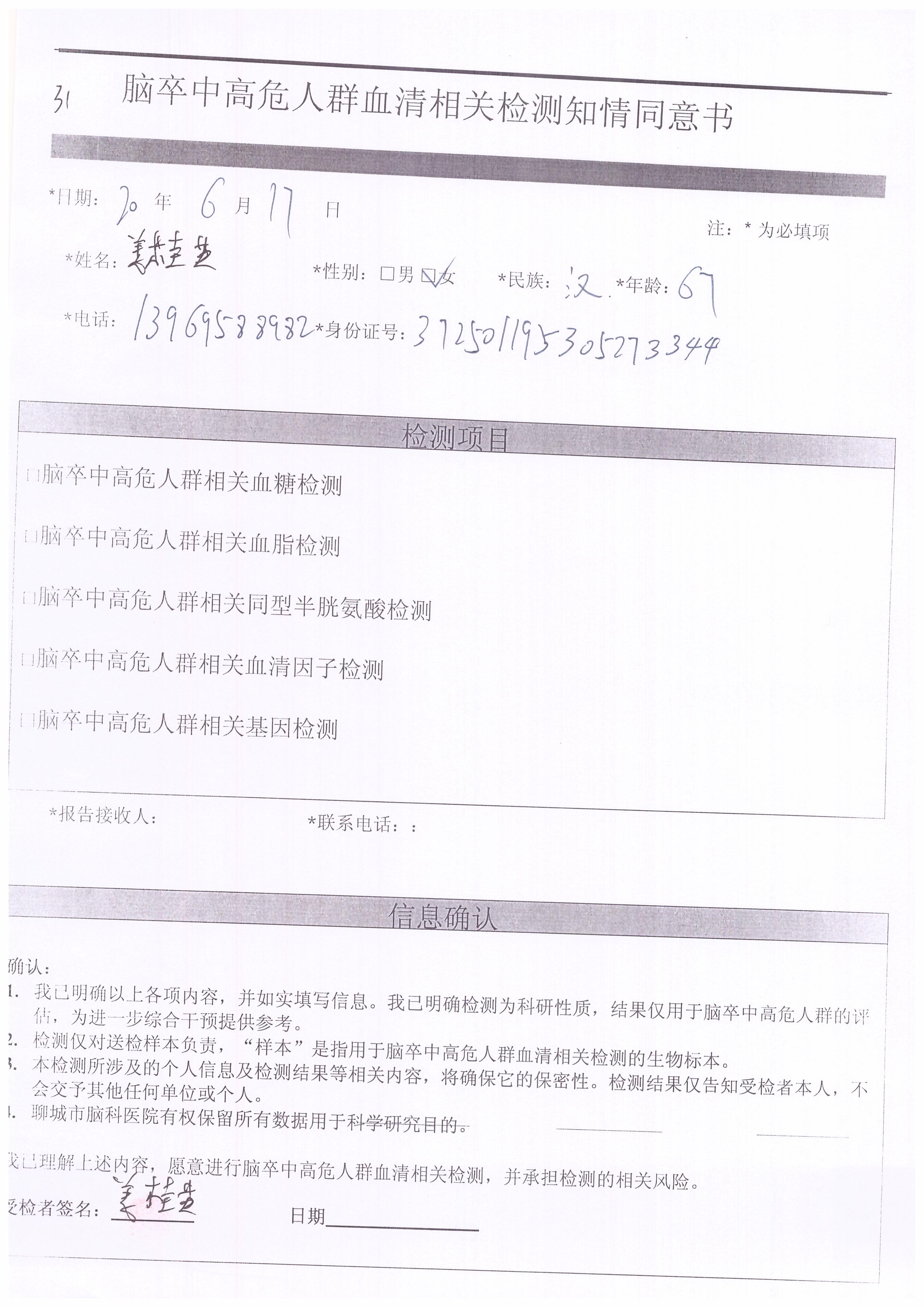

Supplement: Supplementary file 6 — Supplementary file6 (ZIP 29080 KB) [file 10528_2023_10431_MOESM6_ESM.zip › ╓¬╟Θ═1⁄4╥Γ╩Θ4/031.jpg]

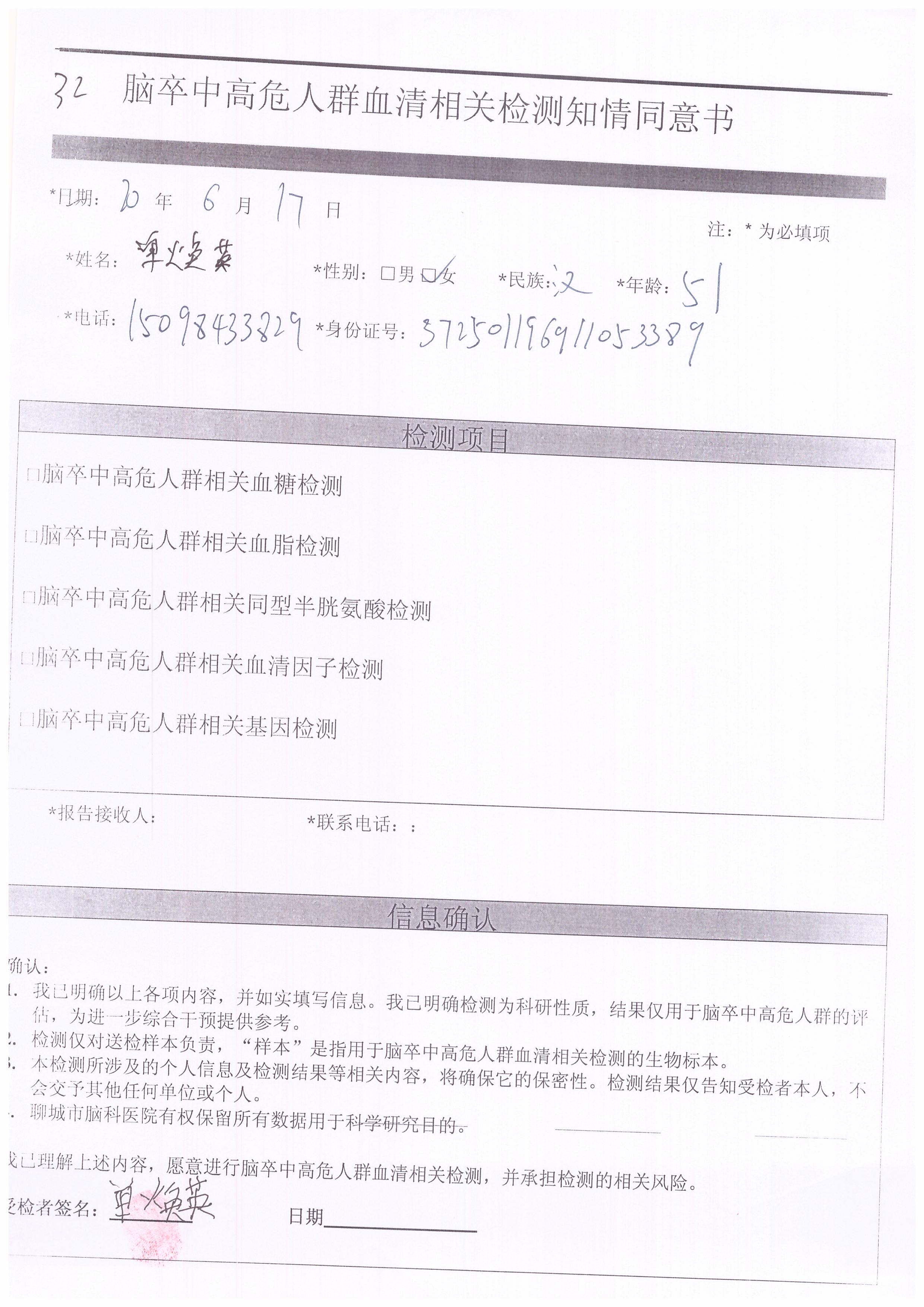

Supplement: Supplementary file 6 — Supplementary file6 (ZIP 29080 KB) [file 10528_2023_10431_MOESM6_ESM.zip › ╓¬╟Θ═1⁄4╥Γ╩Θ4/032.jpg]

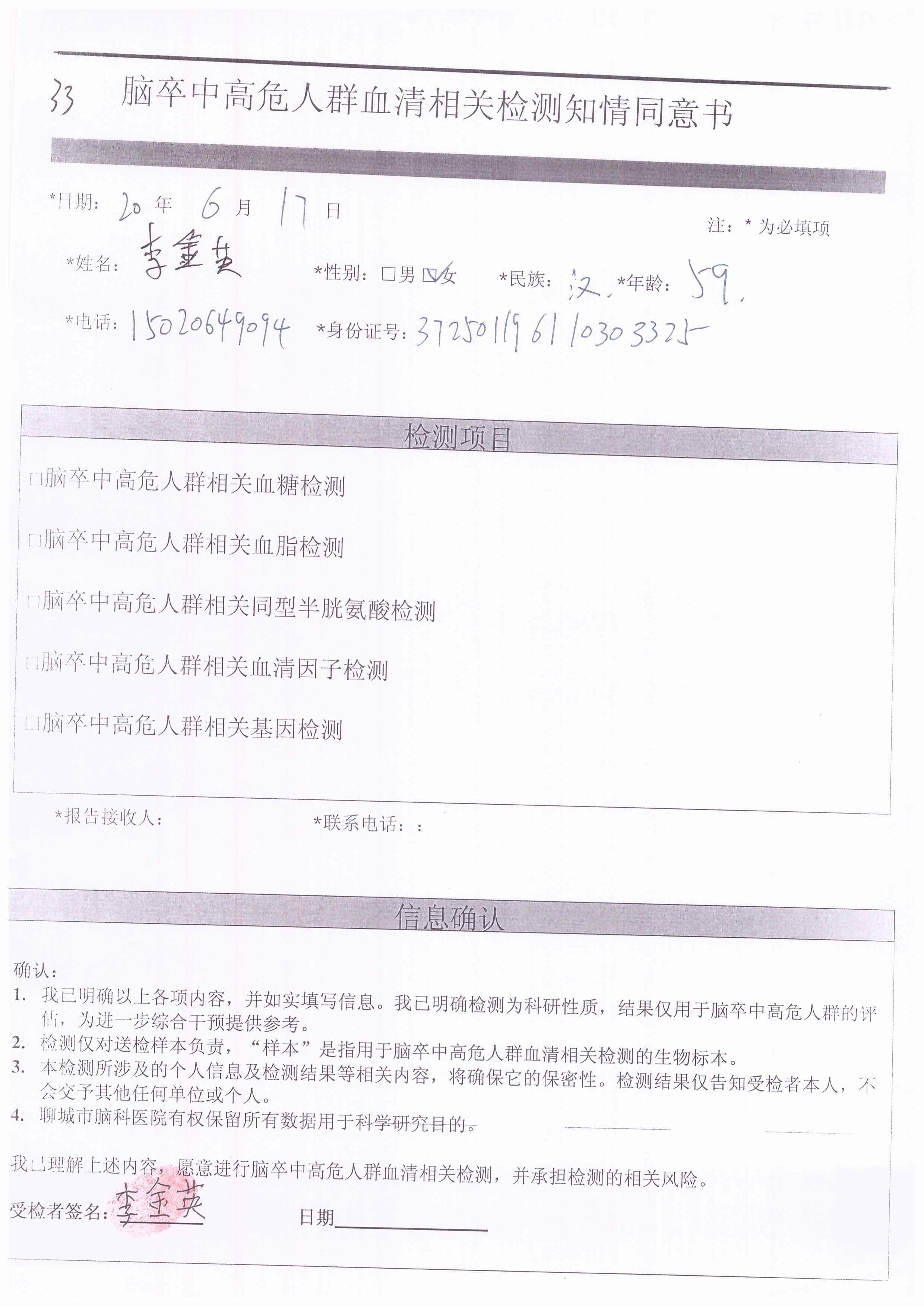

Supplement: Supplementary file 6 — Supplementary file6 (ZIP 29080 KB) [file 10528_2023_10431_MOESM6_ESM.zip › ╓¬╟Θ═1⁄4╥Γ╩Θ4/033.jpg]

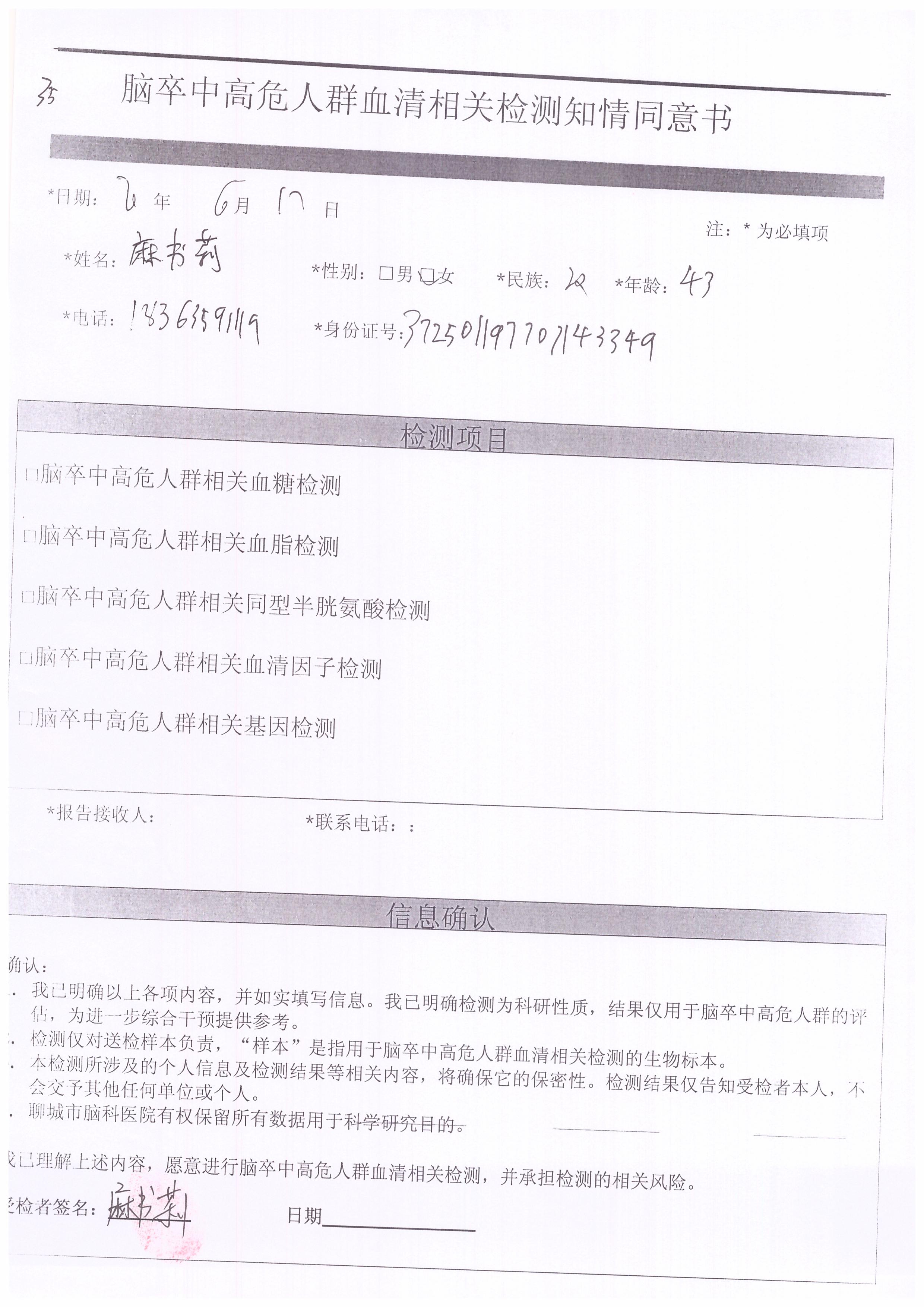

Supplement: Supplementary file 6 — Supplementary file6 (ZIP 29080 KB) [file 10528_2023_10431_MOESM6_ESM.zip › ╓¬╟Θ═1⁄4╥Γ╩Θ4/035.jpg]

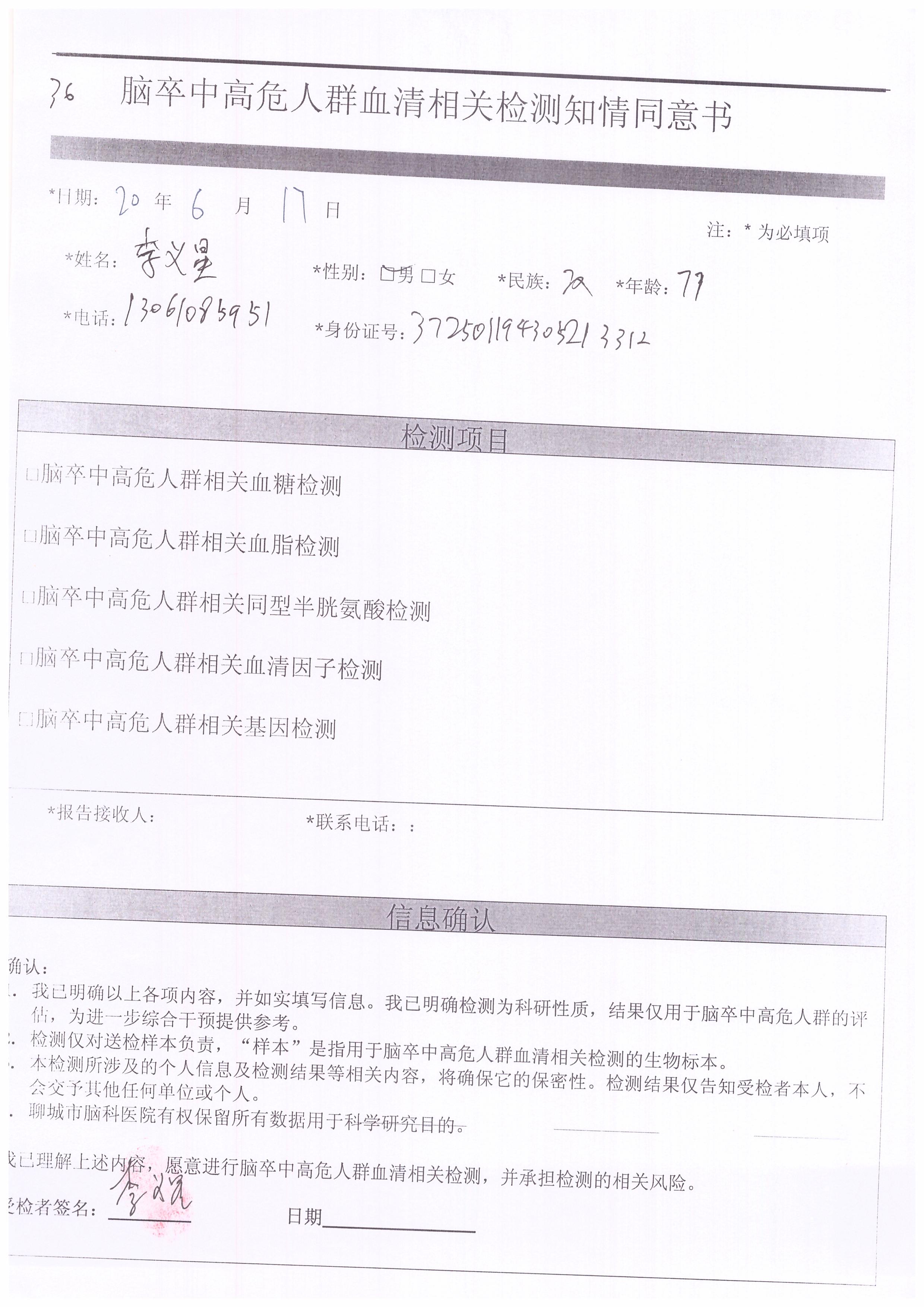

Supplement: Supplementary file 6 — Supplementary file6 (ZIP 29080 KB) [file 10528_2023_10431_MOESM6_ESM.zip › ╓¬╟Θ═1⁄4╥Γ╩Θ4/036.jpg]

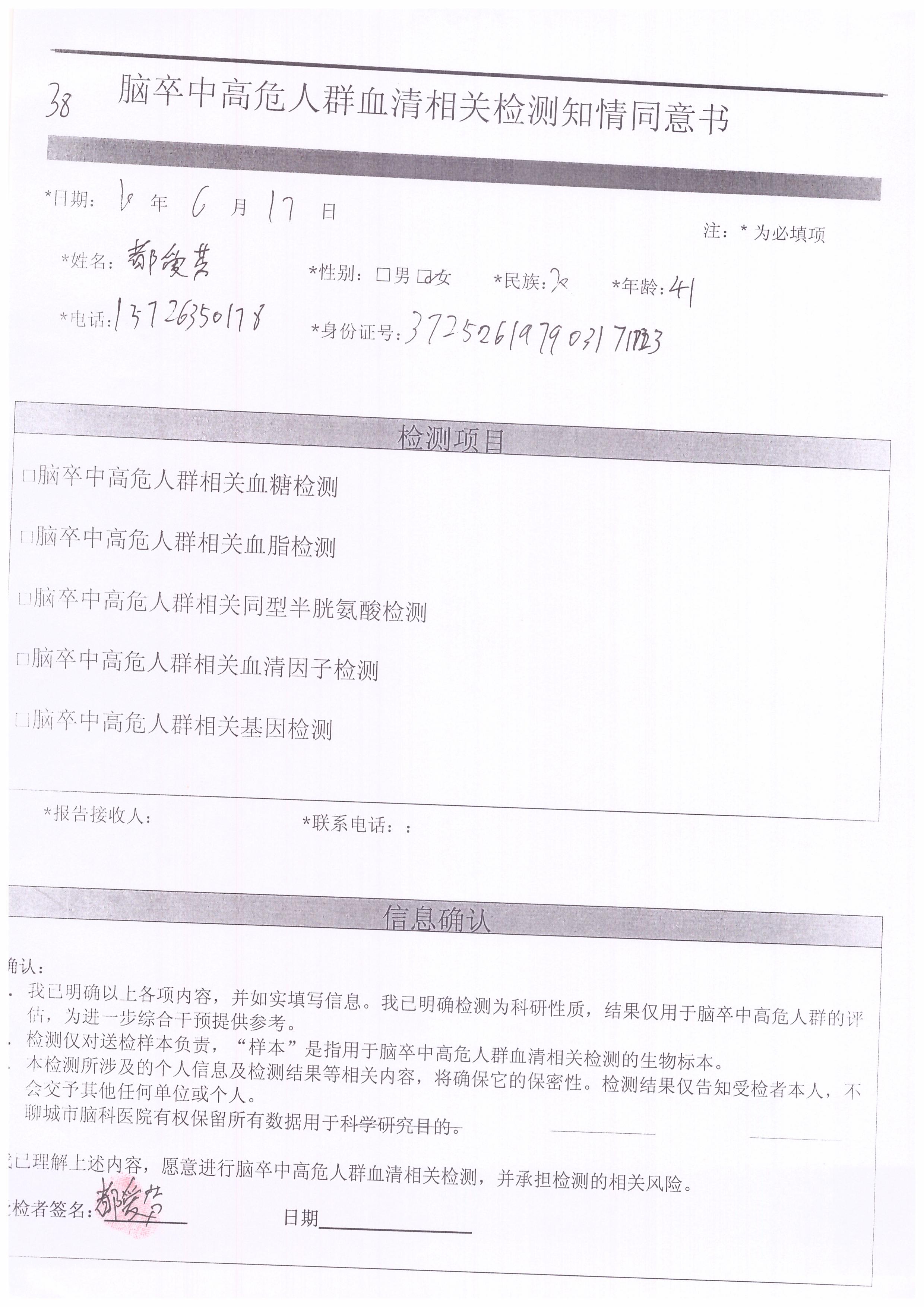

Supplement: Supplementary file 6 — Supplementary file6 (ZIP 29080 KB) [file 10528_2023_10431_MOESM6_ESM.zip › ╓¬╟Θ═1⁄4╥Γ╩Θ4/038.jpg]

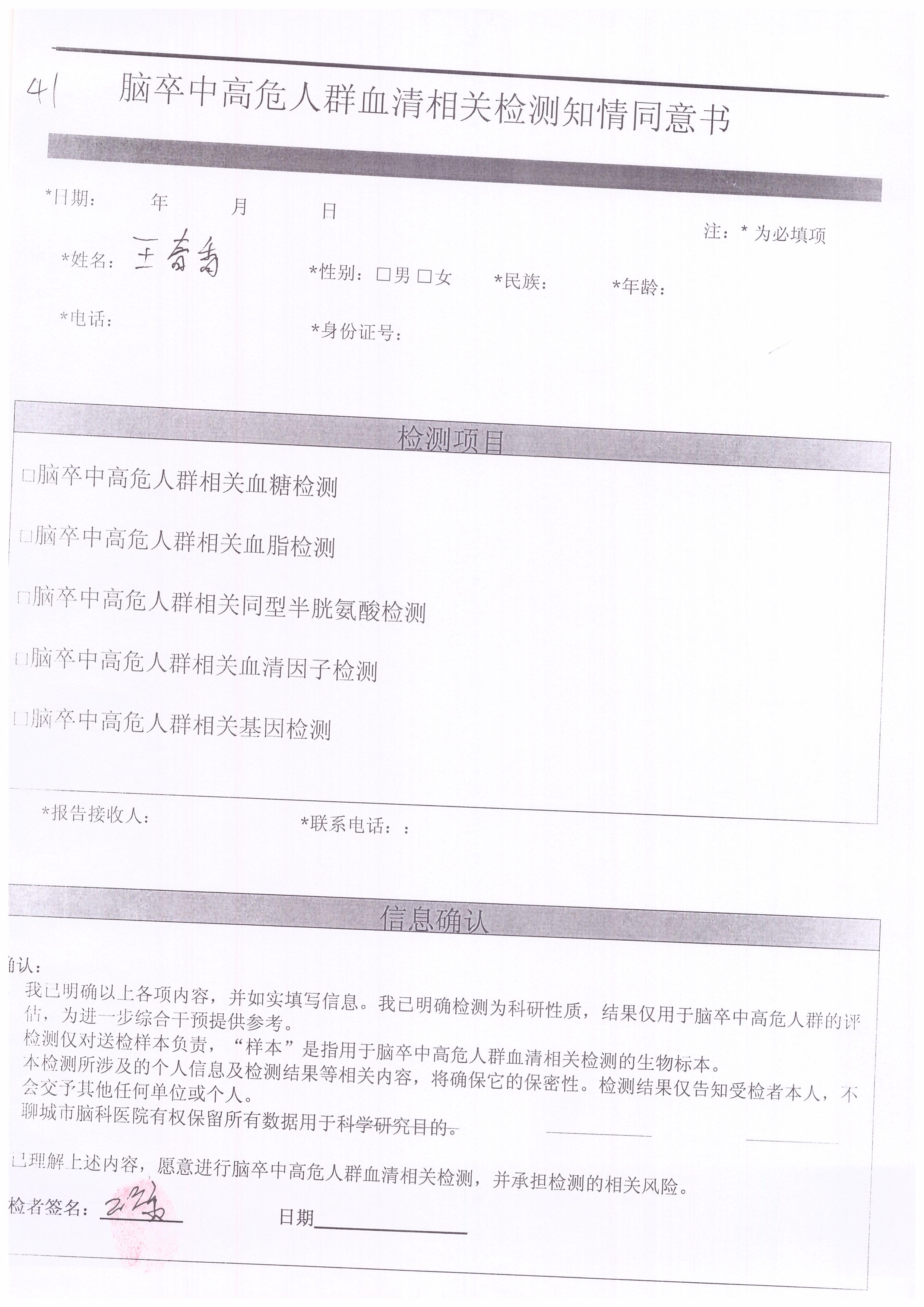

Supplement: Supplementary file 6 — Supplementary file6 (ZIP 29080 KB) [file 10528_2023_10431_MOESM6_ESM.zip › ╓¬╟Θ═1⁄4╥Γ╩Θ4/041.jpg]

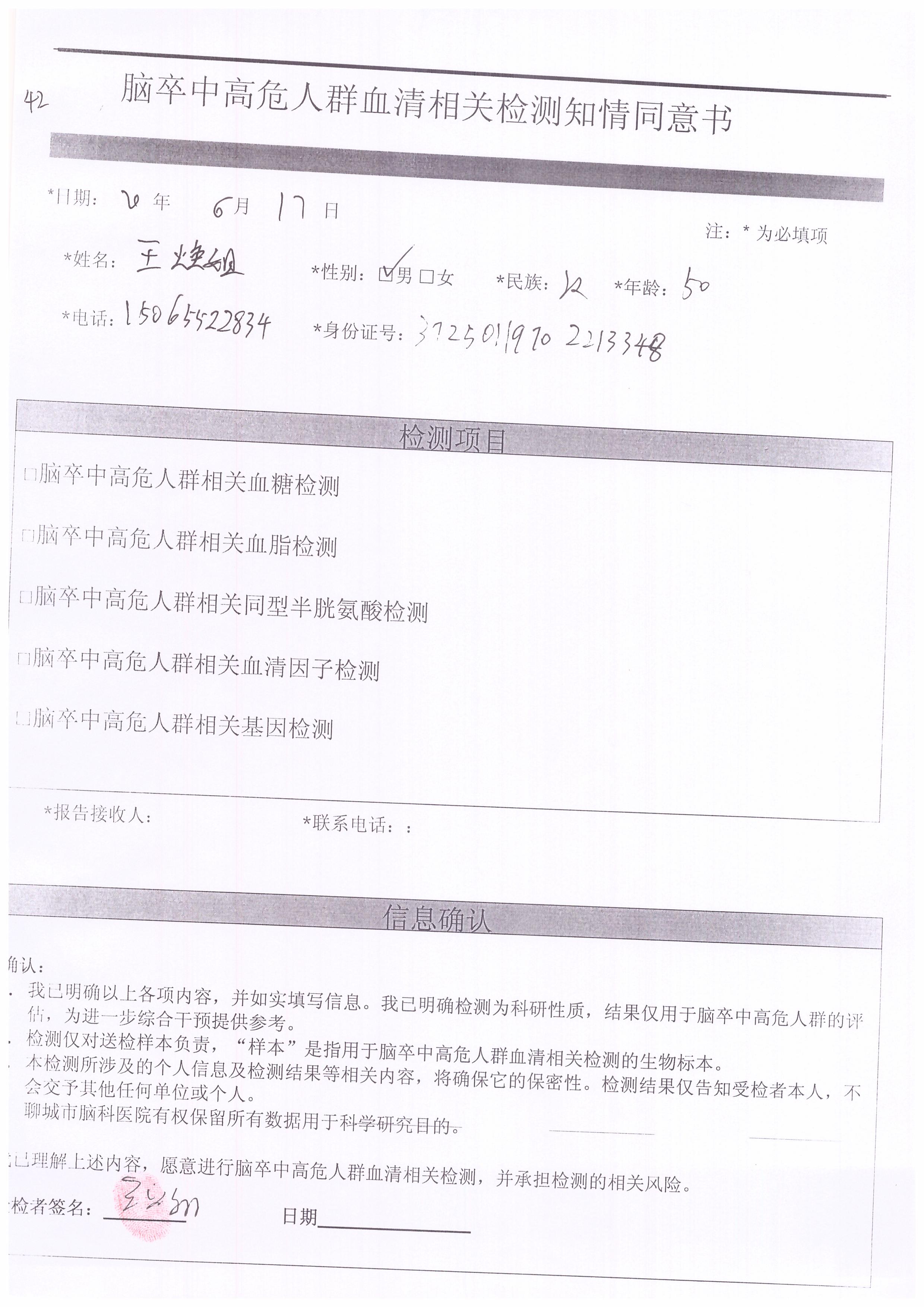

Supplement: Supplementary file 6 — Supplementary file6 (ZIP 29080 KB) [file 10528_2023_10431_MOESM6_ESM.zip › ╓¬╟Θ═1⁄4╥Γ╩Θ4/042.jpg]

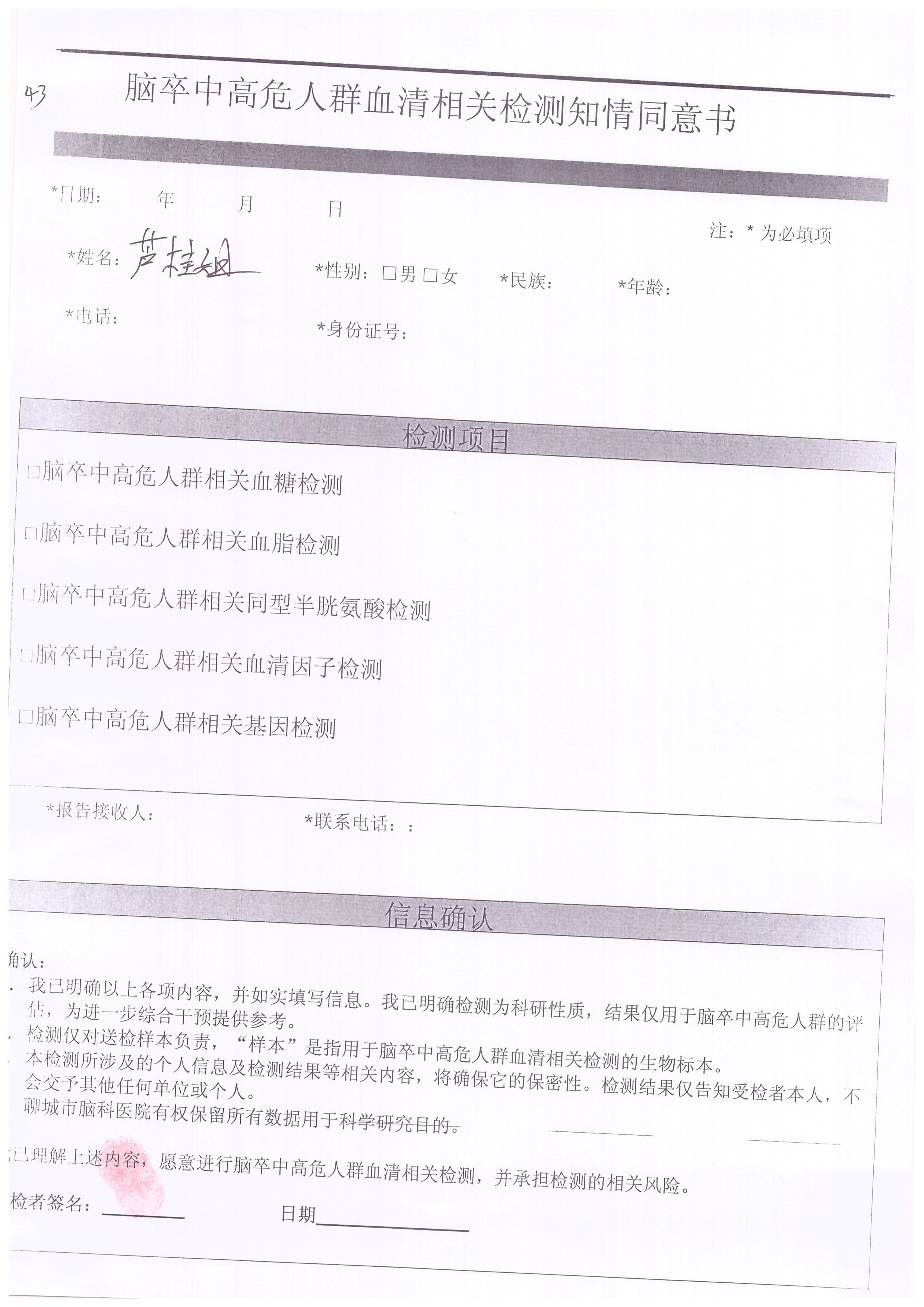

Supplement: Supplementary file 6 — Supplementary file6 (ZIP 29080 KB) [file 10528_2023_10431_MOESM6_ESM.zip › ╓¬╟Θ═1⁄4╥Γ╩Θ4/043.jpg]

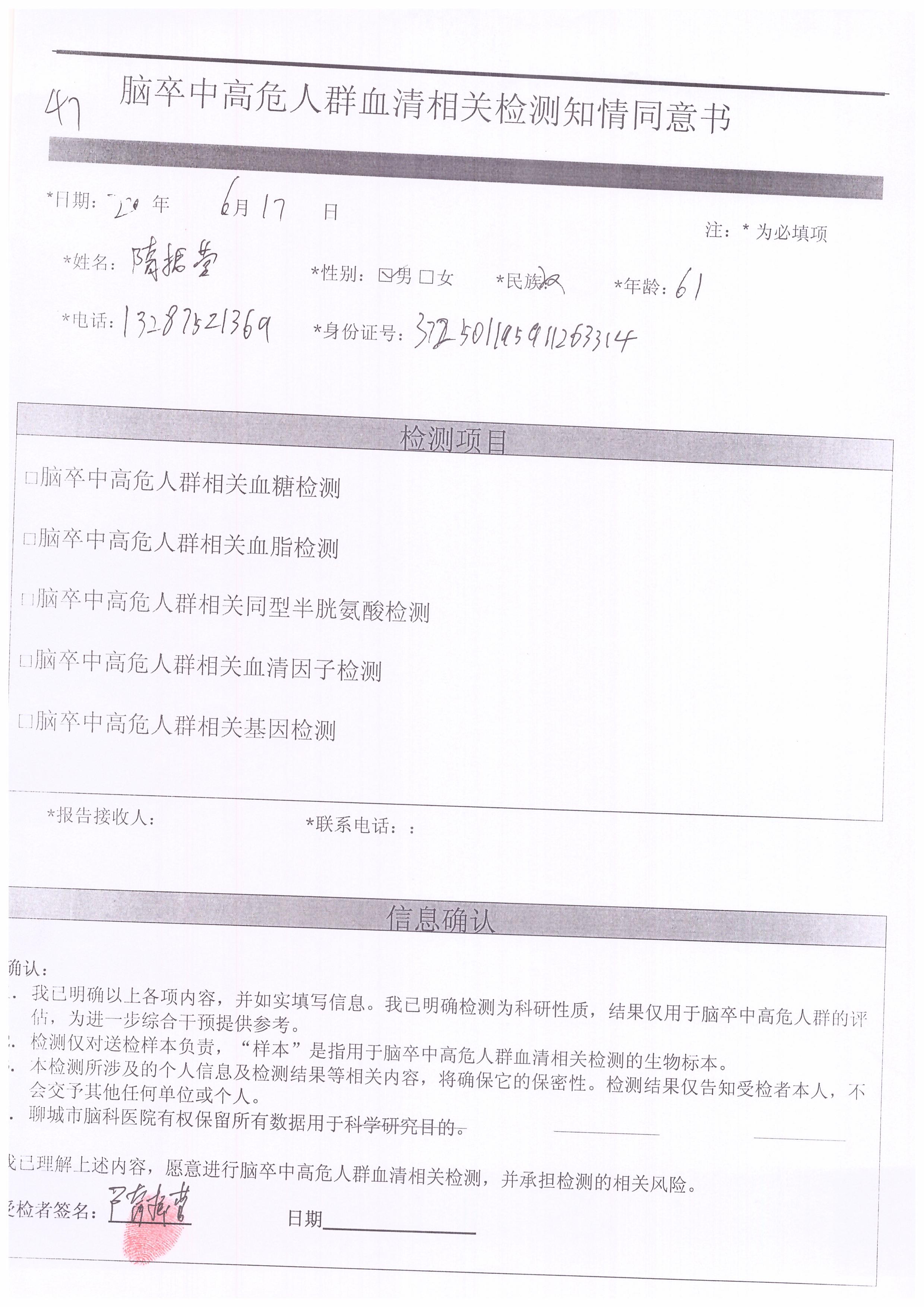

Supplement: Supplementary file 6 — Supplementary file6 (ZIP 29080 KB) [file 10528_2023_10431_MOESM6_ESM.zip › ╓¬╟Θ═1⁄4╥Γ╩Θ4/047.jpg]

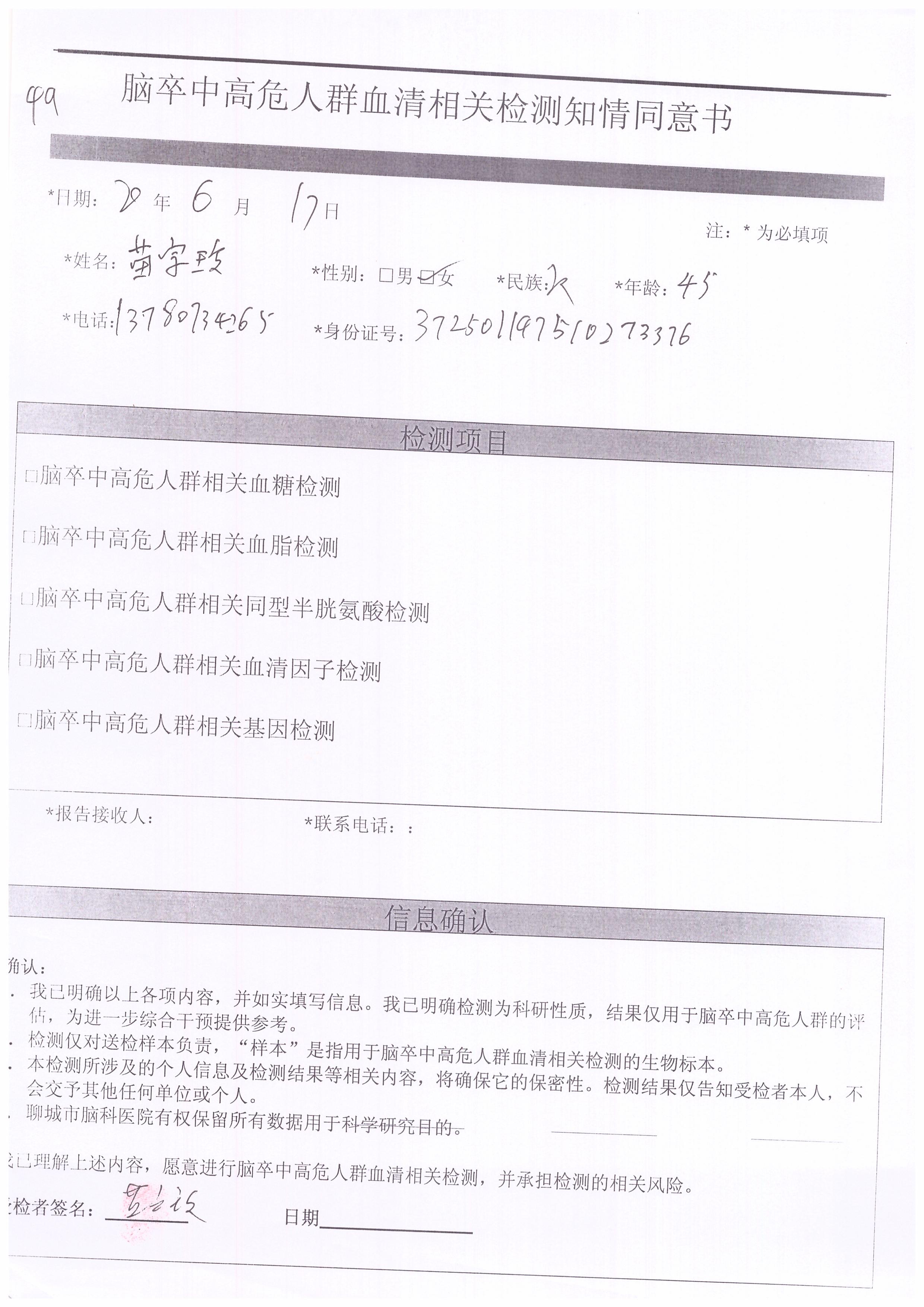

Supplement: Supplementary file 6 — Supplementary file6 (ZIP 29080 KB) [file 10528_2023_10431_MOESM6_ESM.zip › ╓¬╟Θ═1⁄4╥Γ╩Θ4/049.jpg]

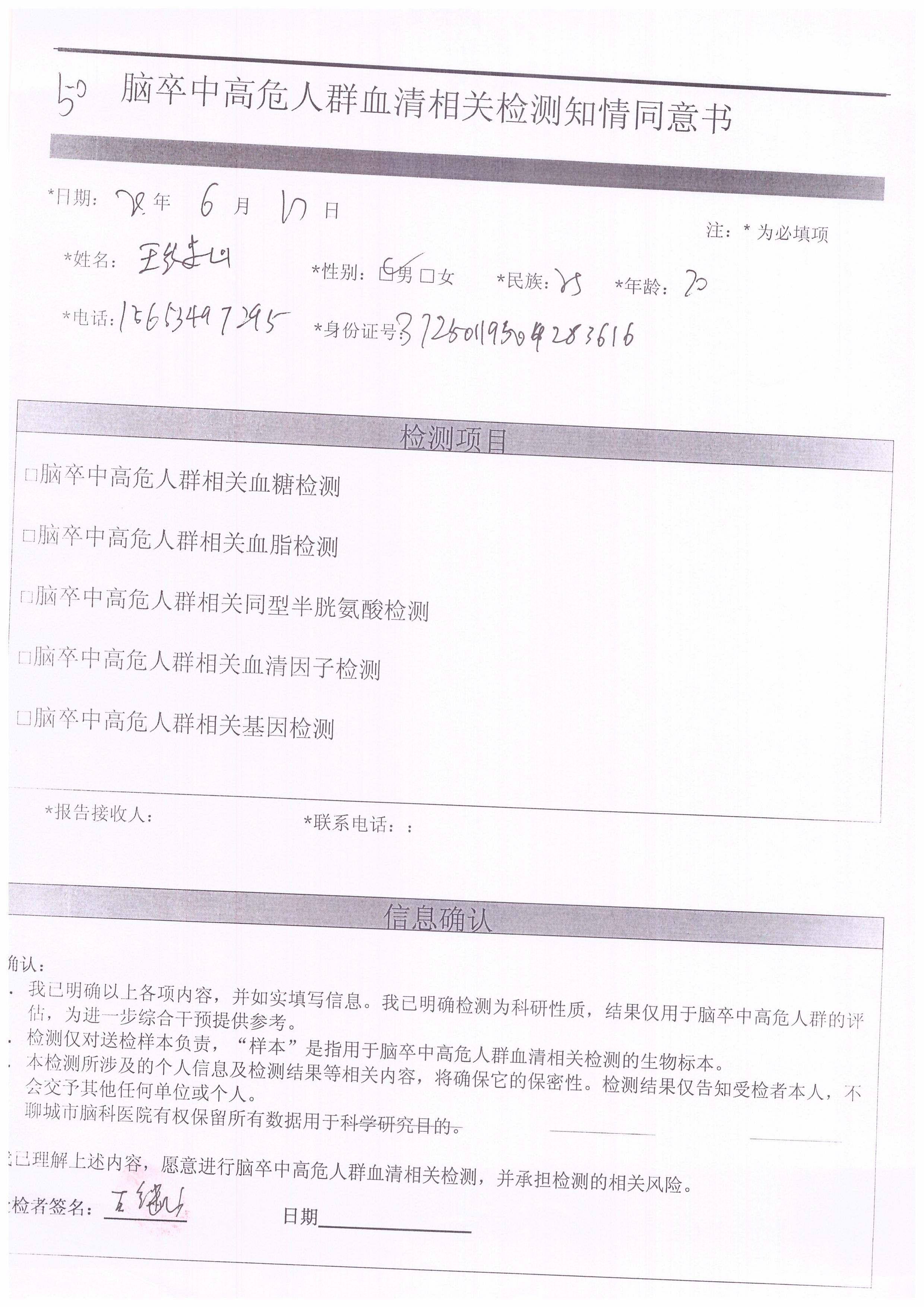

Supplement: Supplementary file 6 — Supplementary file6 (ZIP 29080 KB) [file 10528_2023_10431_MOESM6_ESM.zip › ╓¬╟Θ═1⁄4╥Γ╩Θ4/050.jpg]

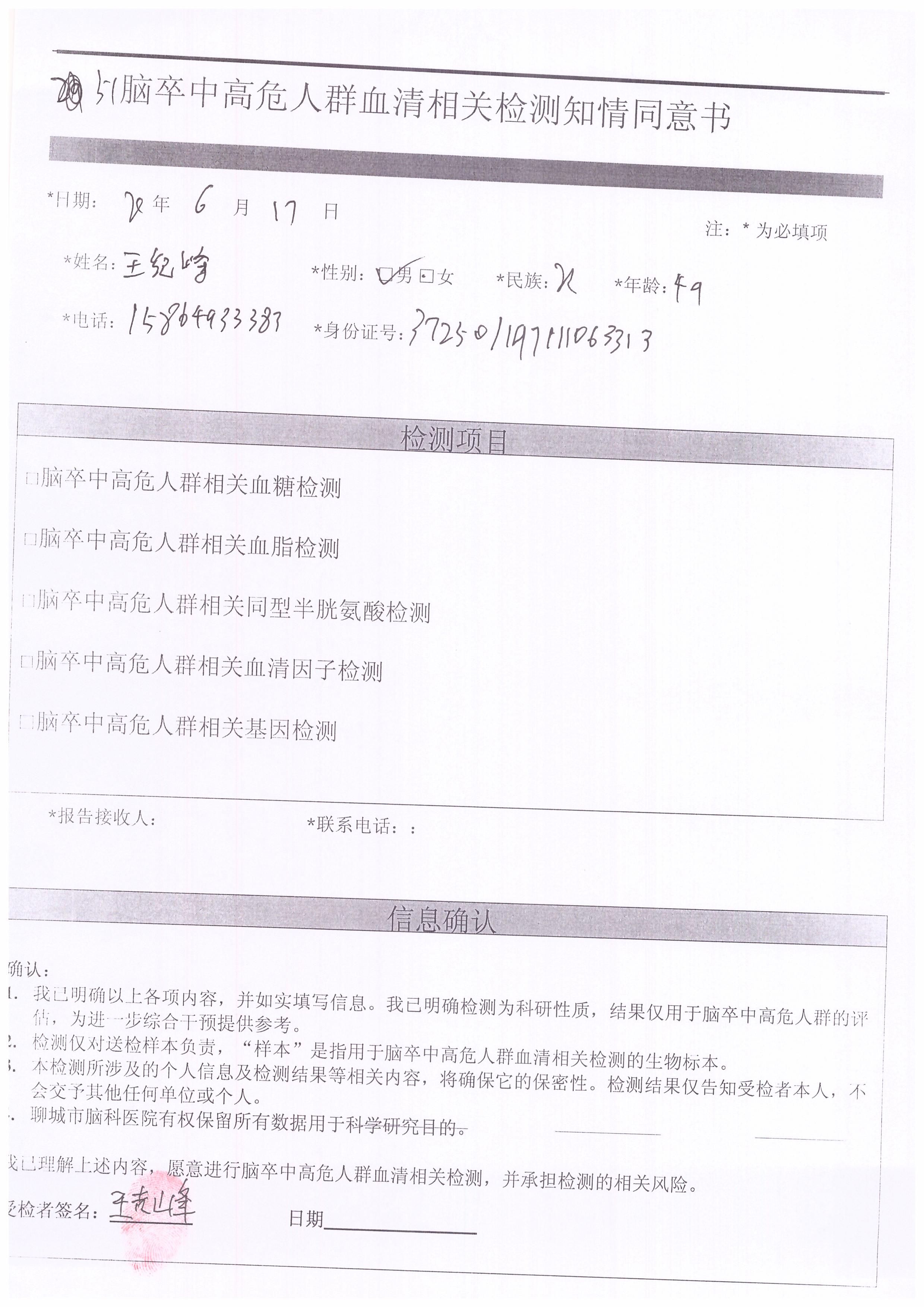

Supplement: Supplementary file 6 — Supplementary file6 (ZIP 29080 KB) [file 10528_2023_10431_MOESM6_ESM.zip › ╓¬╟Θ═1⁄4╥Γ╩Θ4/051.jpg]

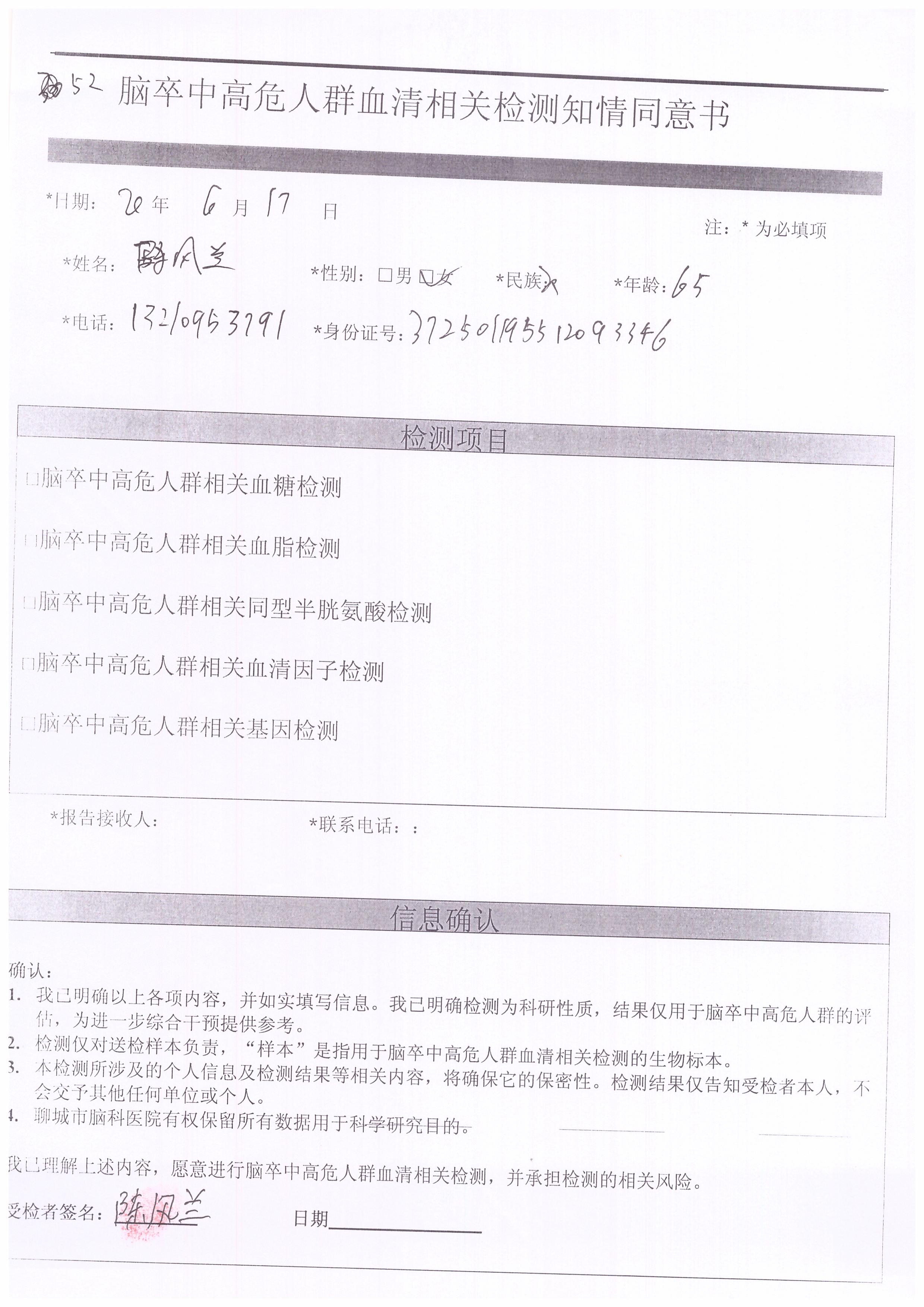

Supplement: Supplementary file 6 — Supplementary file6 (ZIP 29080 KB) [file 10528_2023_10431_MOESM6_ESM.zip › ╓¬╟Θ═1⁄4╥Γ╩Θ4/052.jpg]

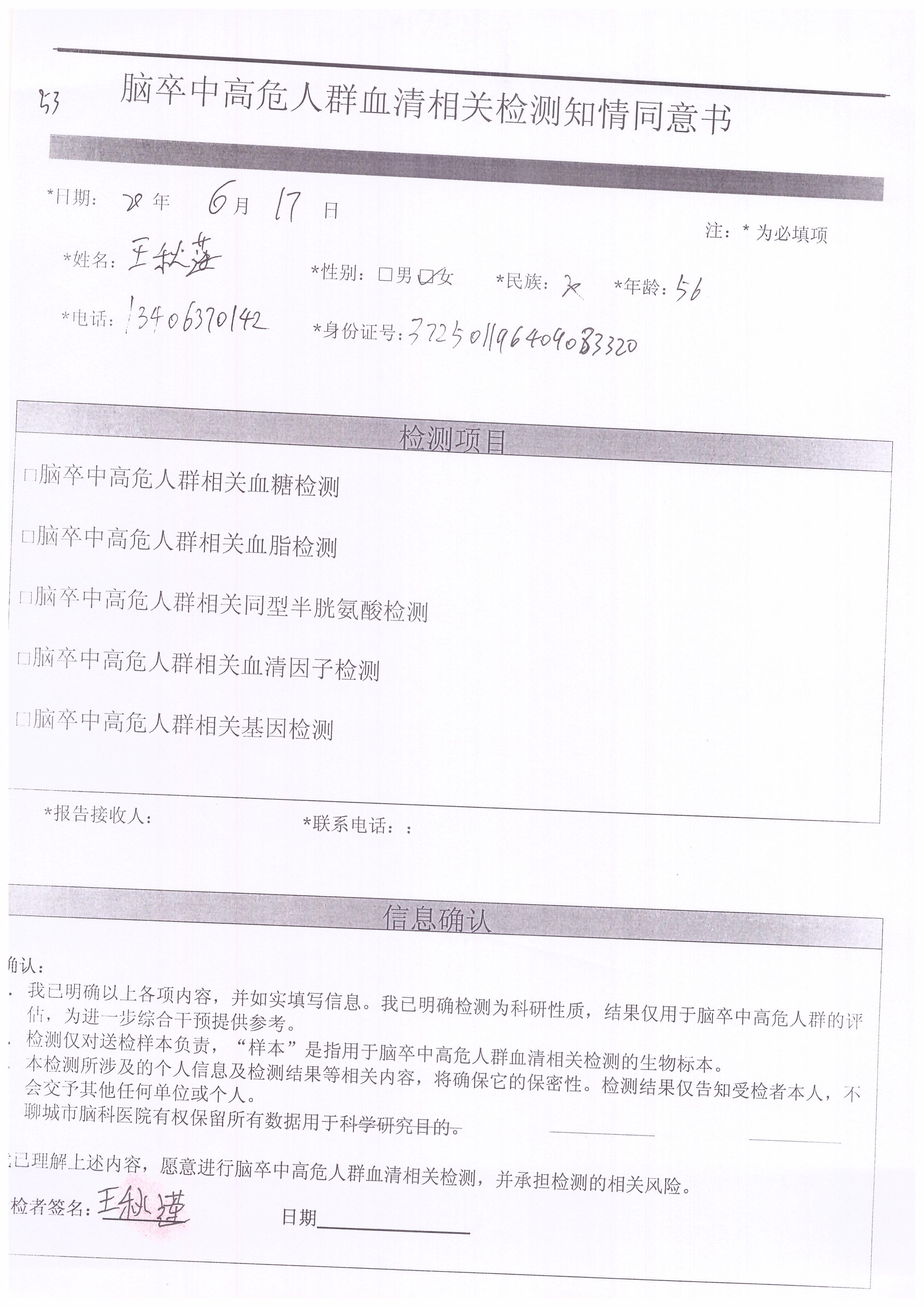

Supplement: Supplementary file 6 — Supplementary file6 (ZIP 29080 KB) [file 10528_2023_10431_MOESM6_ESM.zip › ╓¬╟Θ═1⁄4╥Γ╩Θ4/053.jpg]

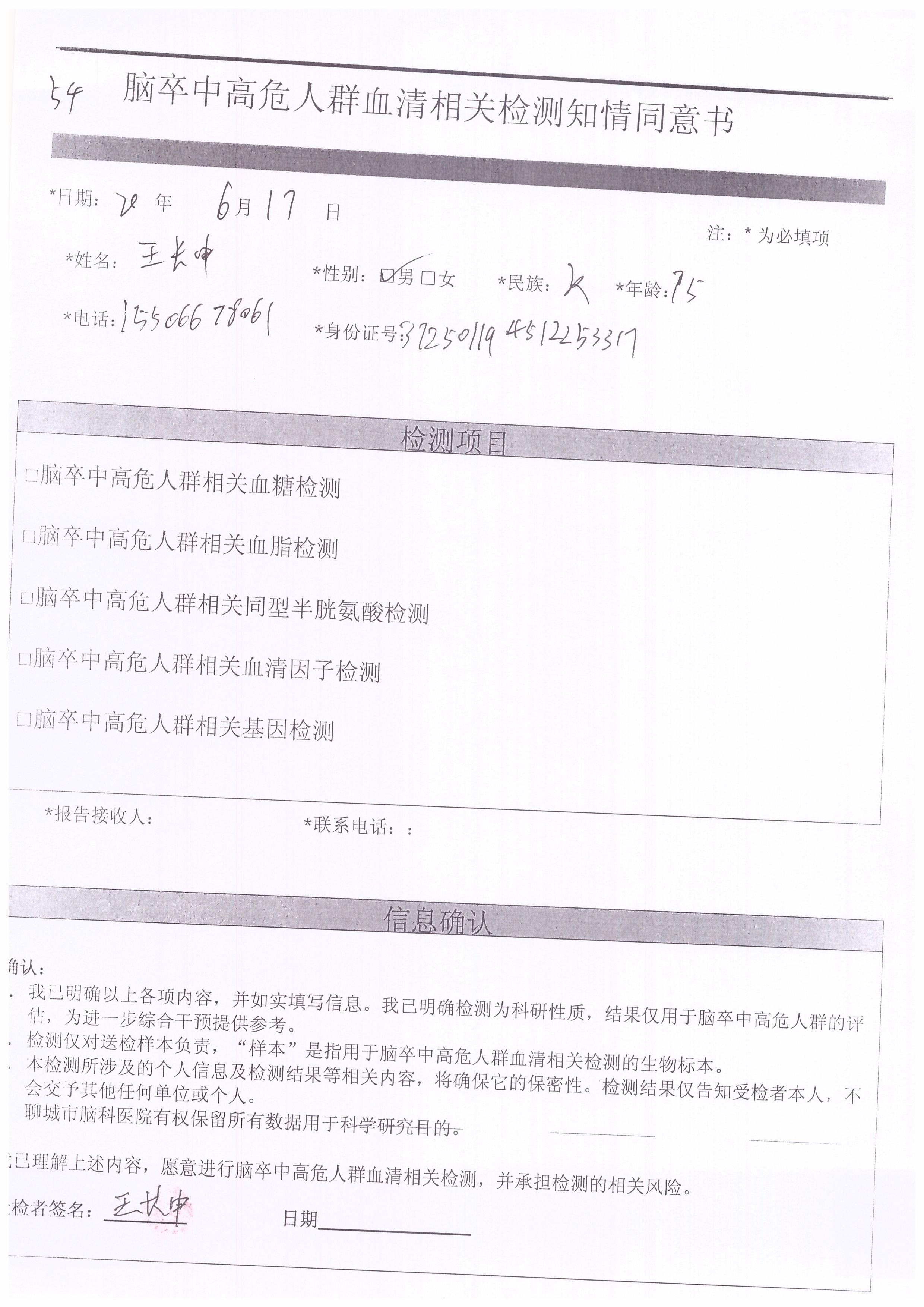

Supplement: Supplementary file 6 — Supplementary file6 (ZIP 29080 KB) [file 10528_2023_10431_MOESM6_ESM.zip › ╓¬╟Θ═1⁄4╥Γ╩Θ4/054.jpg]

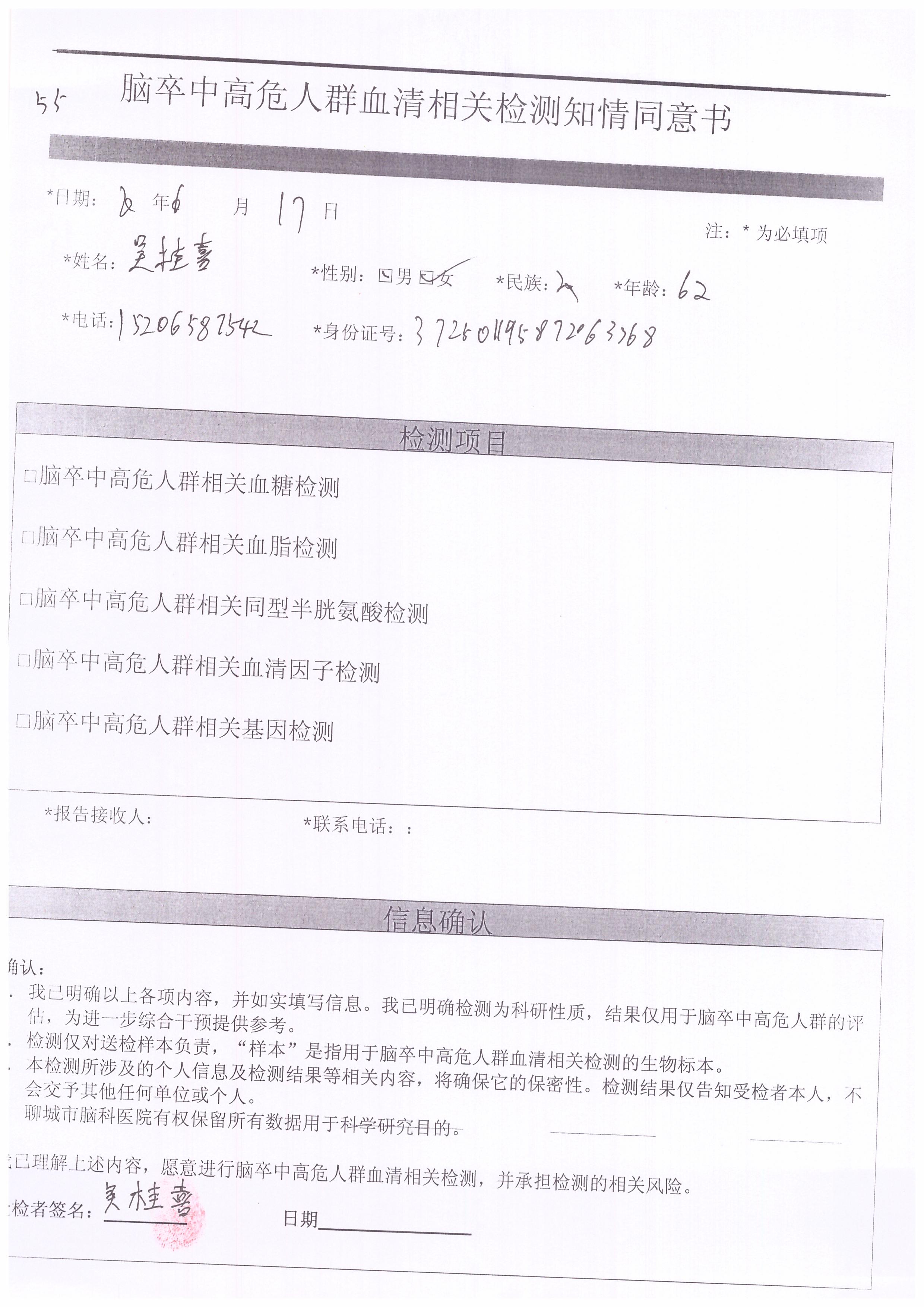

Supplement: Supplementary file 6 — Supplementary file6 (ZIP 29080 KB) [file 10528_2023_10431_MOESM6_ESM.zip › ╓¬╟Θ═1⁄4╥Γ╩Θ4/055.jpg]

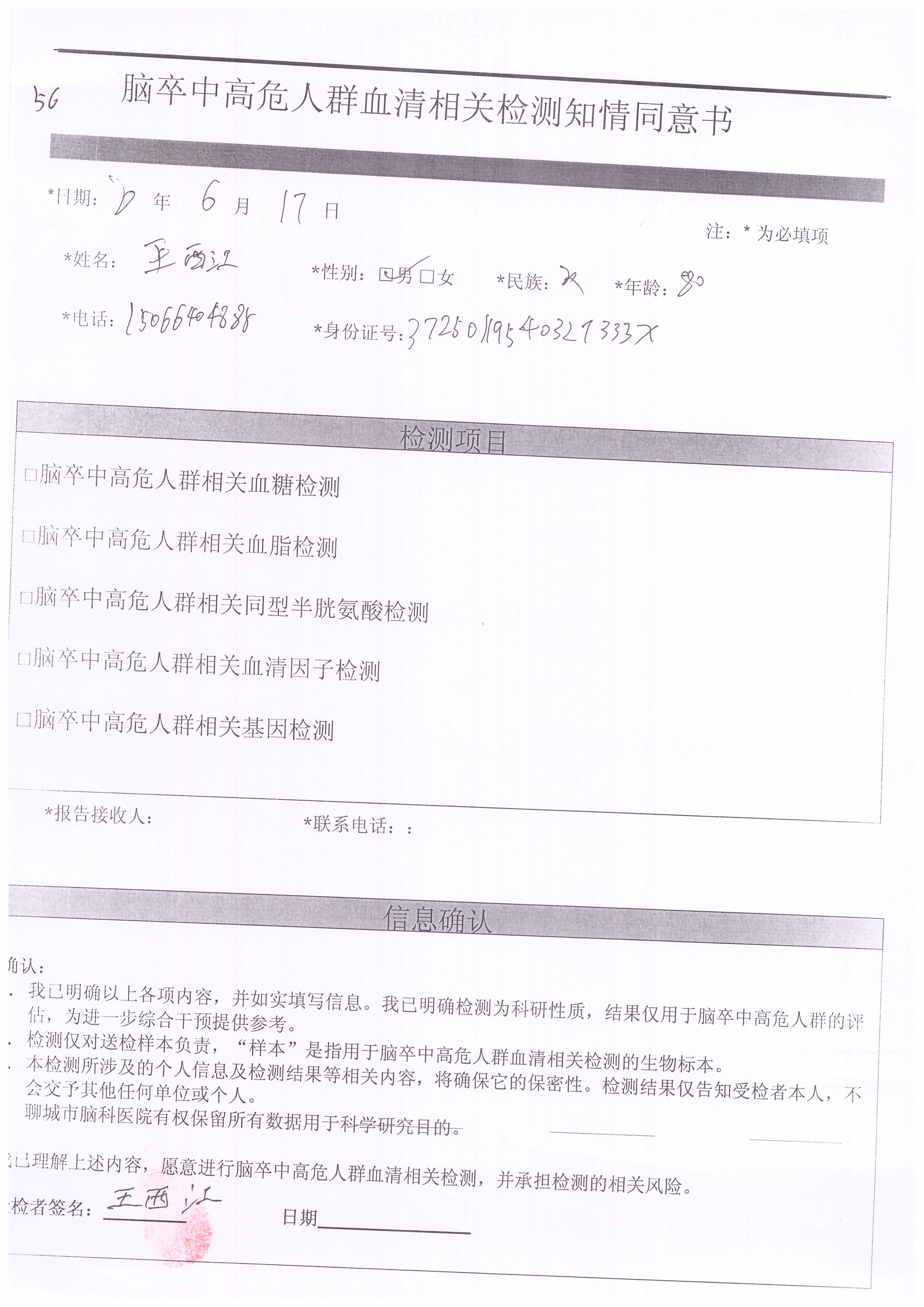

Supplement: Supplementary file 6 — Supplementary file6 (ZIP 29080 KB) [file 10528_2023_10431_MOESM6_ESM.zip › ╓¬╟Θ═1⁄4╥Γ╩Θ4/056.jpg]

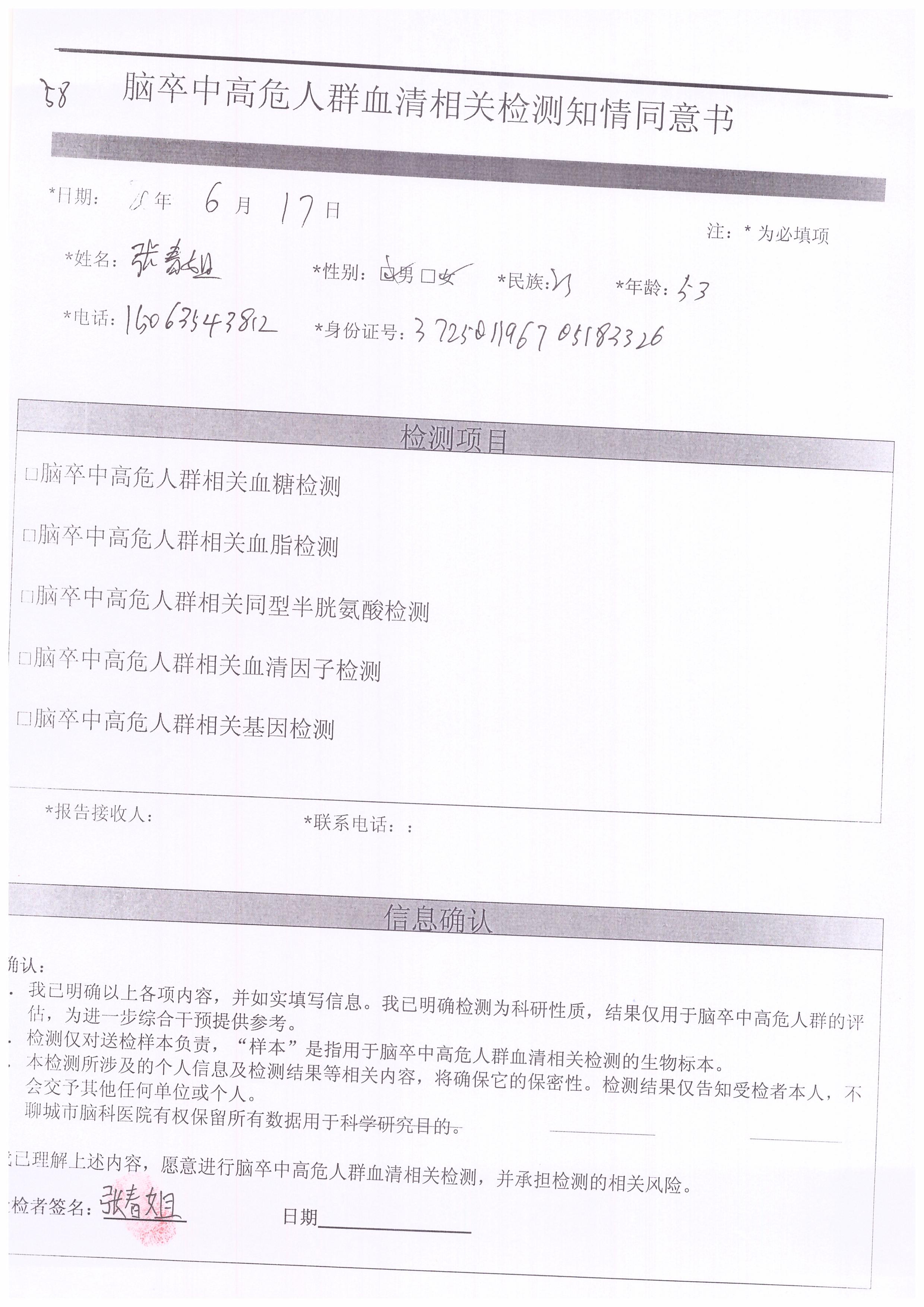

Supplement: Supplementary file 6 — Supplementary file6 (ZIP 29080 KB) [file 10528_2023_10431_MOESM6_ESM.zip › ╓¬╟Θ═1⁄4╥Γ╩Θ4/057.jpg]

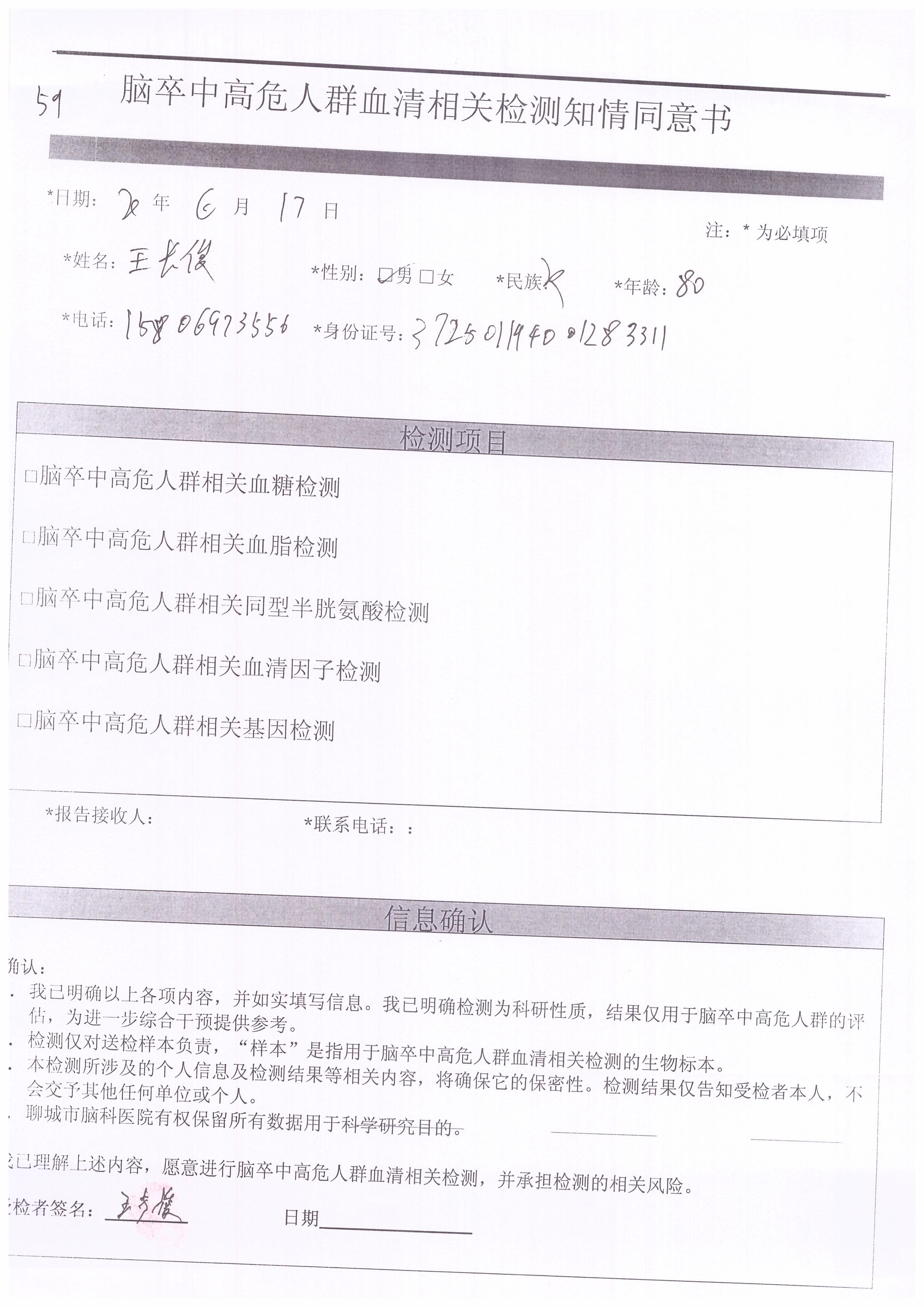

Supplement: Supplementary file 6 — Supplementary file6 (ZIP 29080 KB) [file 10528_2023_10431_MOESM6_ESM.zip › ╓¬╟Θ═1⁄4╥Γ╩Θ4/058.jpg]

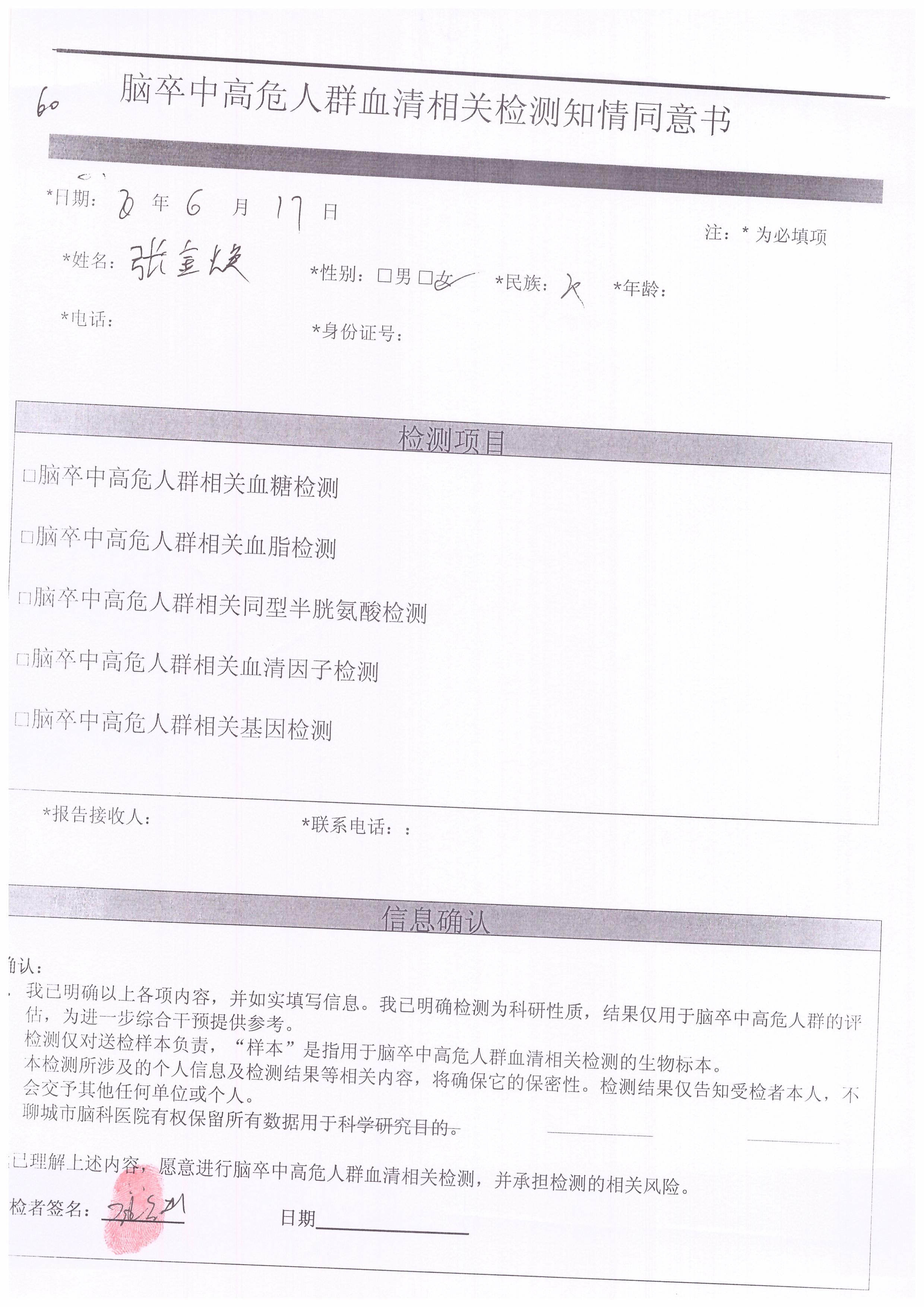

Supplement: Supplementary file 6 — Supplementary file6 (ZIP 29080 KB) [file 10528_2023_10431_MOESM6_ESM.zip › ╓¬╟Θ═1⁄4╥Γ╩Θ4/059.jpg]

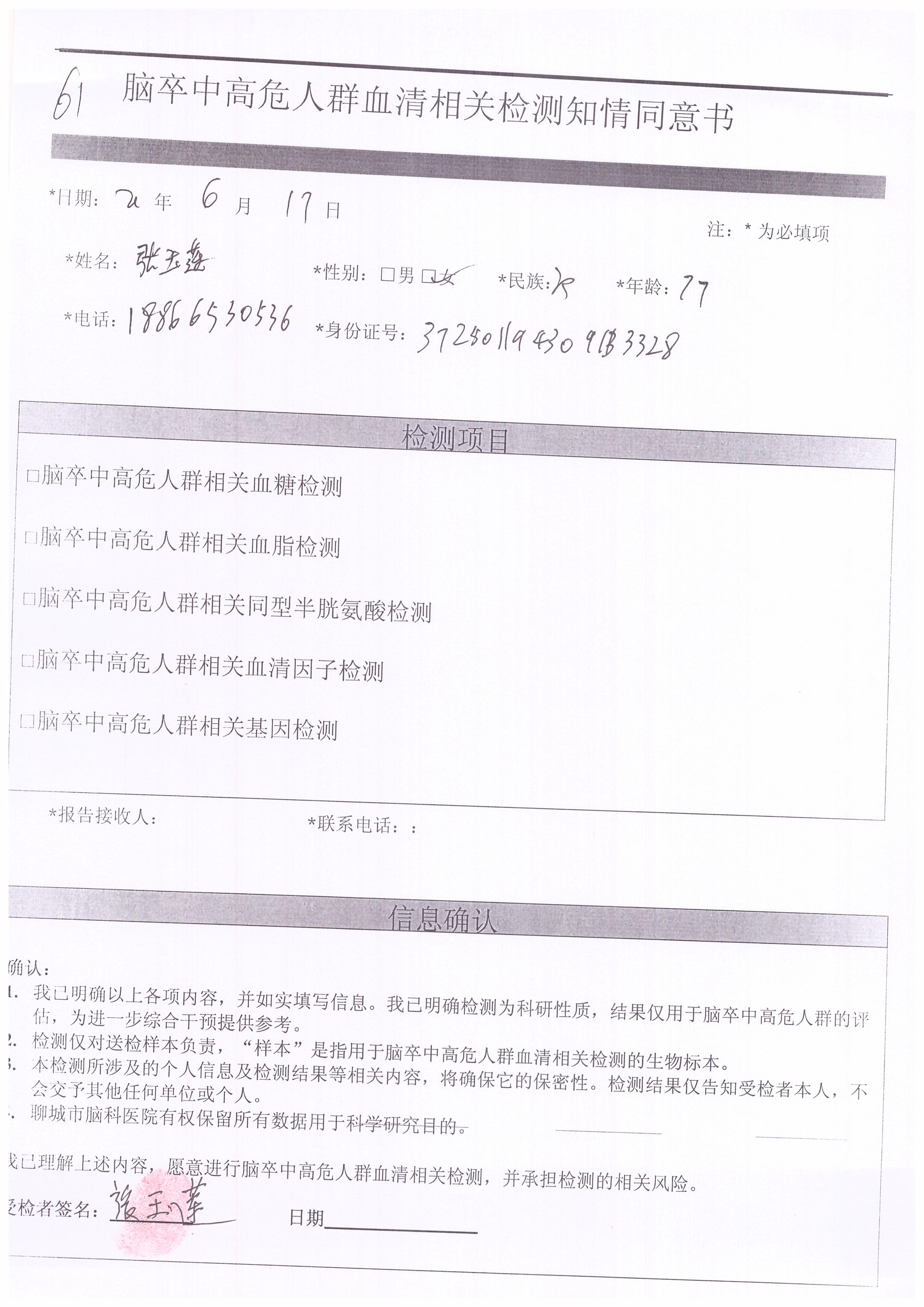

Supplement: Supplementary file 6 — Supplementary file6 (ZIP 29080 KB) [file 10528_2023_10431_MOESM6_ESM.zip › ╓¬╟Θ═1⁄4╥Γ╩Θ4/060.jpg]

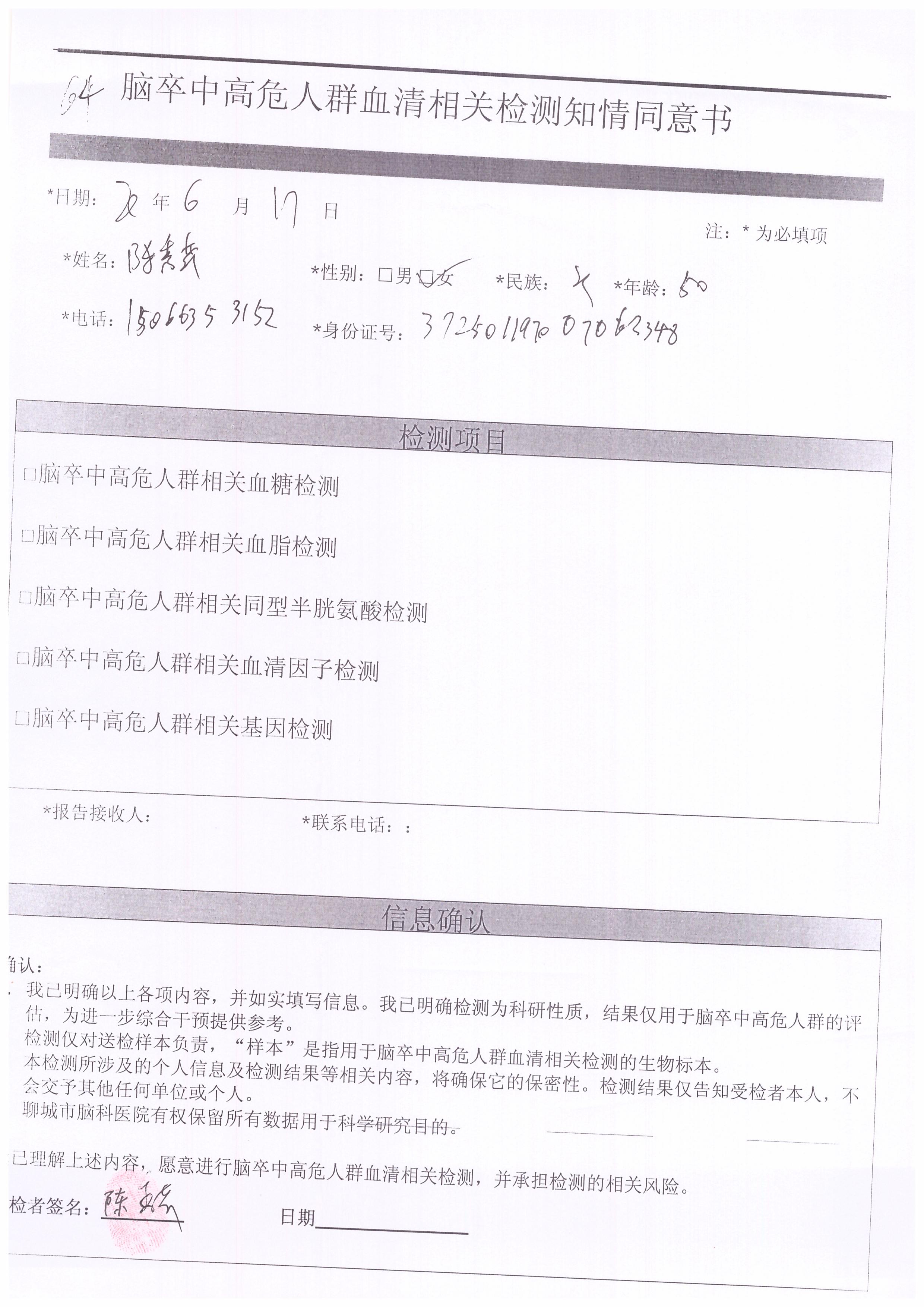

Supplement: Supplementary file 6 — Supplementary file6 (ZIP 29080 KB) [file 10528_2023_10431_MOESM6_ESM.zip › ╓¬╟Θ═1⁄4╥Γ╩Θ4/063.jpg]

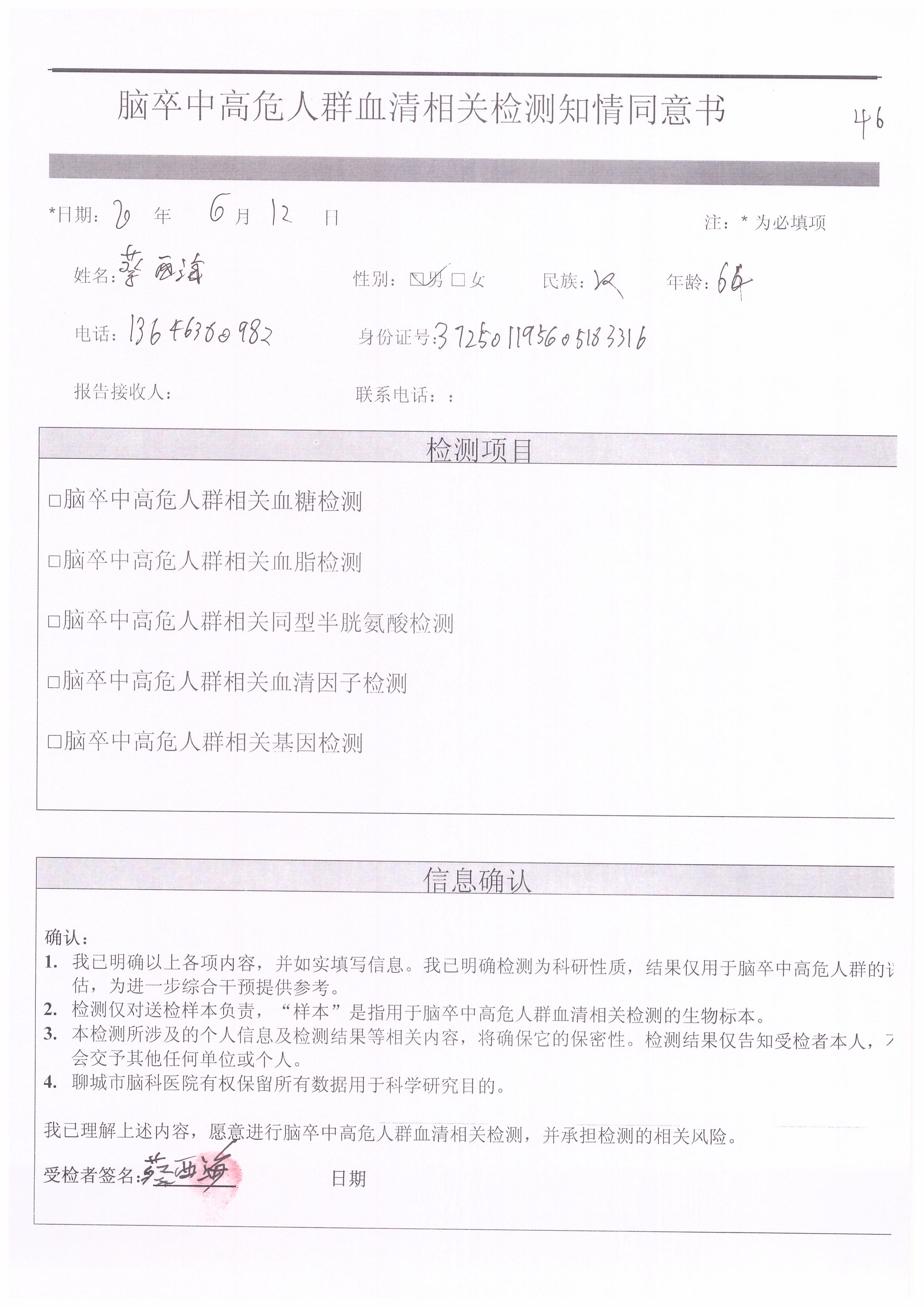

Supplement: Supplementary file 7 — Supplementary file7 (ZIP 27016 KB) [file 10528_2023_10431_MOESM7_ESM.zip › ╓¬╟Θ═1⁄4╥Γ╩Θ5/001.jpg]

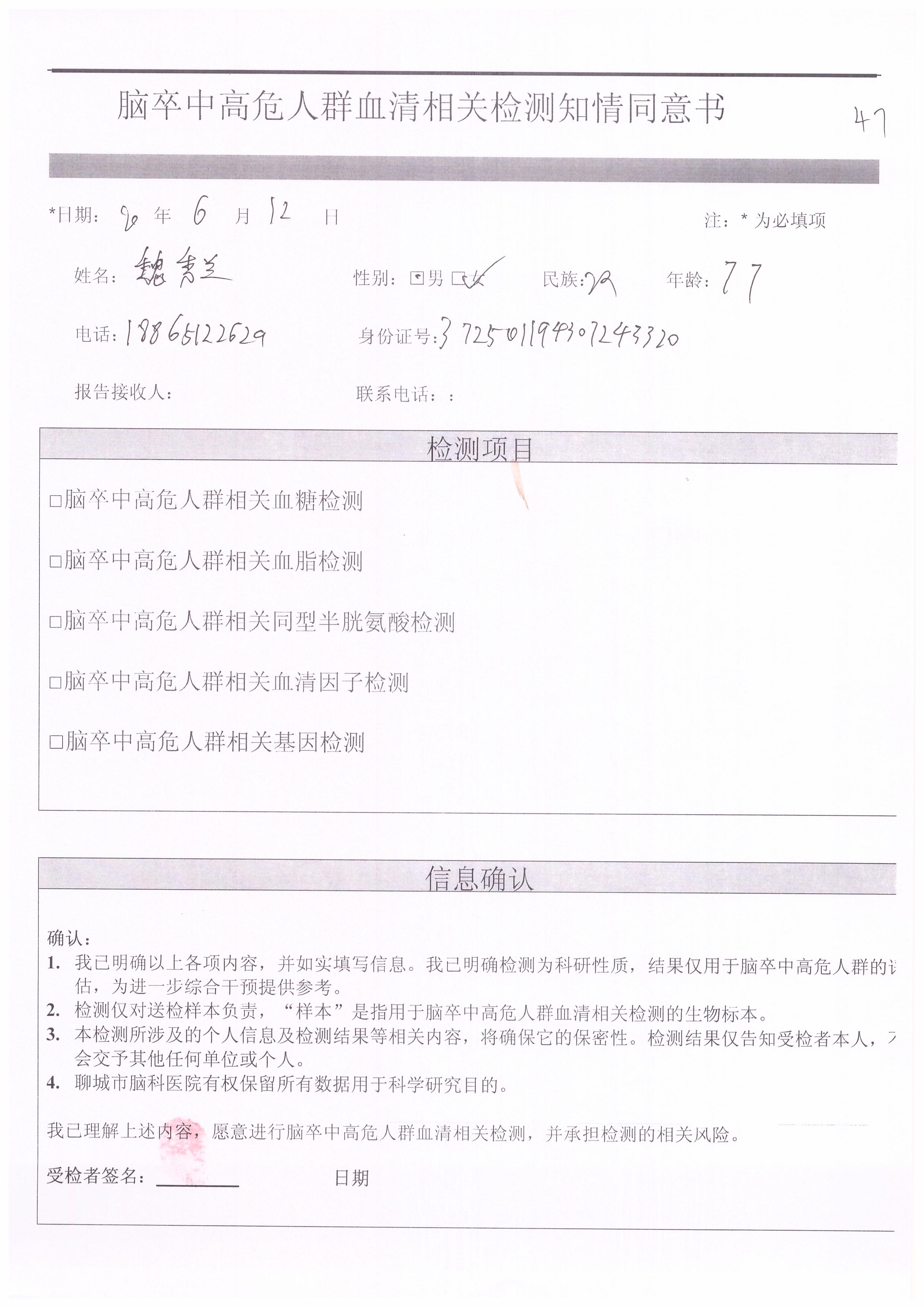

Supplement: Supplementary file 7 — Supplementary file7 (ZIP 27016 KB) [file 10528_2023_10431_MOESM7_ESM.zip › ╓¬╟Θ═1⁄4╥Γ╩Θ5/002.jpg]

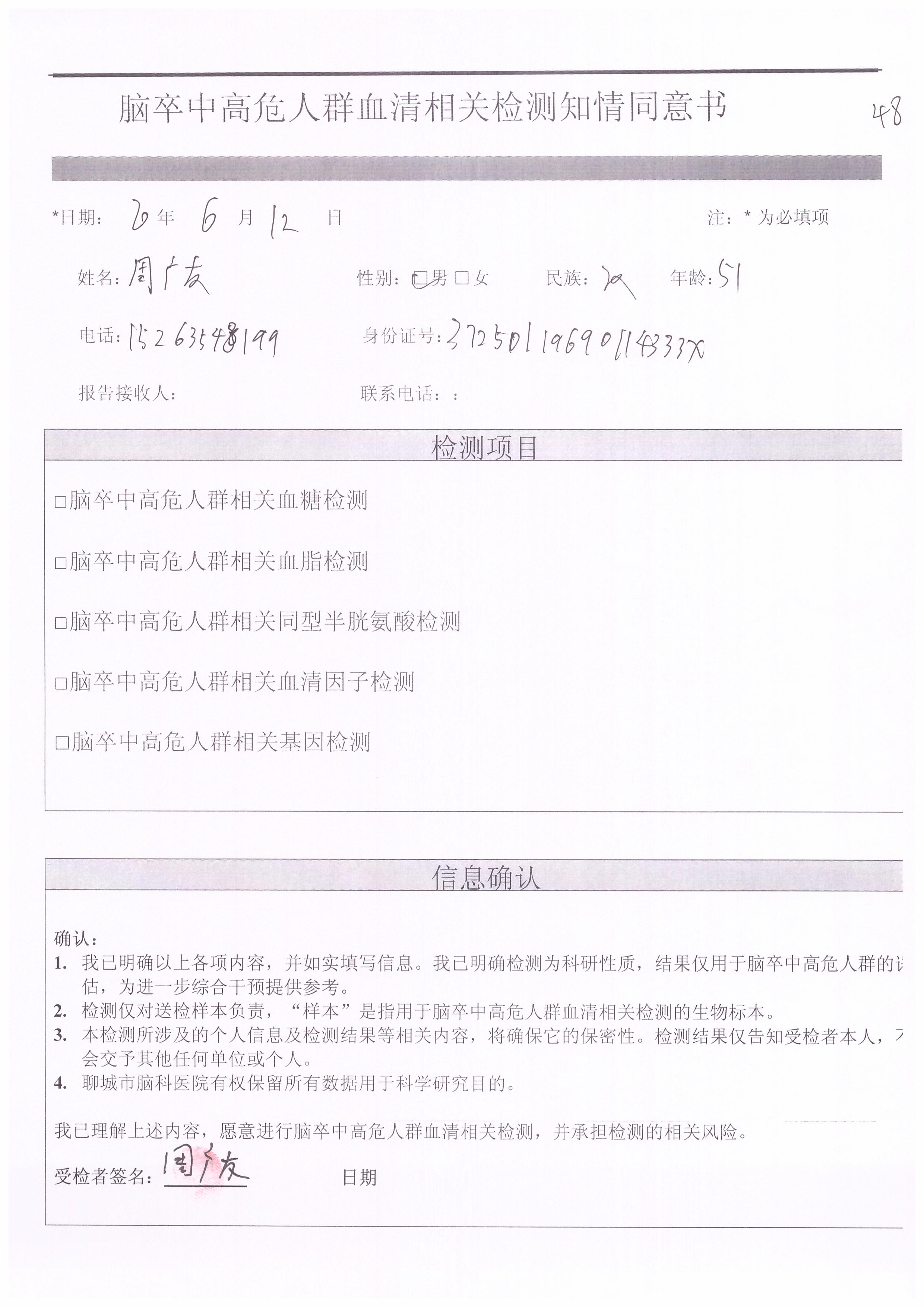

Supplement: Supplementary file 7 — Supplementary file7 (ZIP 27016 KB) [file 10528_2023_10431_MOESM7_ESM.zip › ╓¬╟Θ═1⁄4╥Γ╩Θ5/003.jpg]

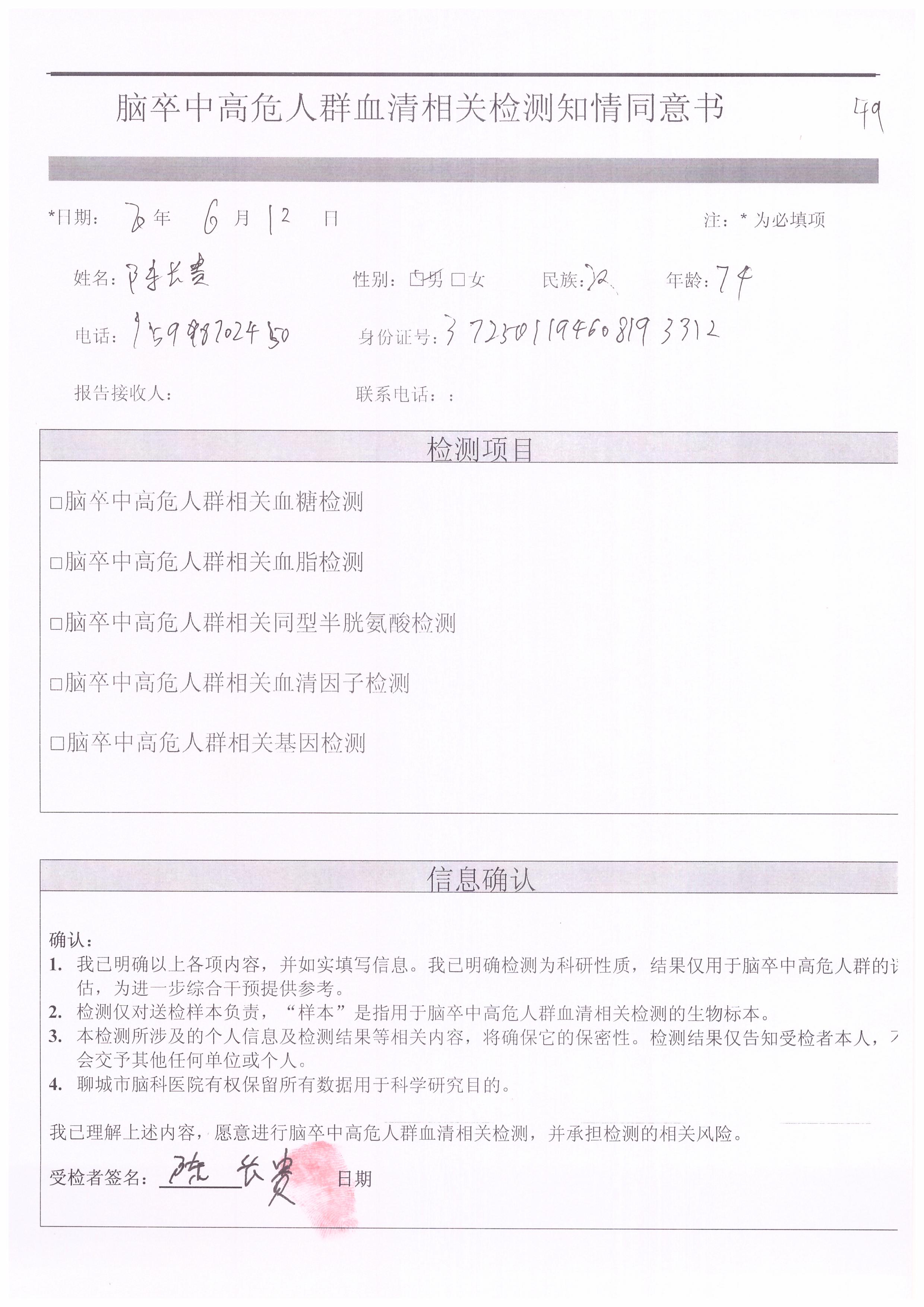

Supplement: Supplementary file 7 — Supplementary file7 (ZIP 27016 KB) [file 10528_2023_10431_MOESM7_ESM.zip › ╓¬╟Θ═1⁄4╥Γ╩Θ5/004.jpg]

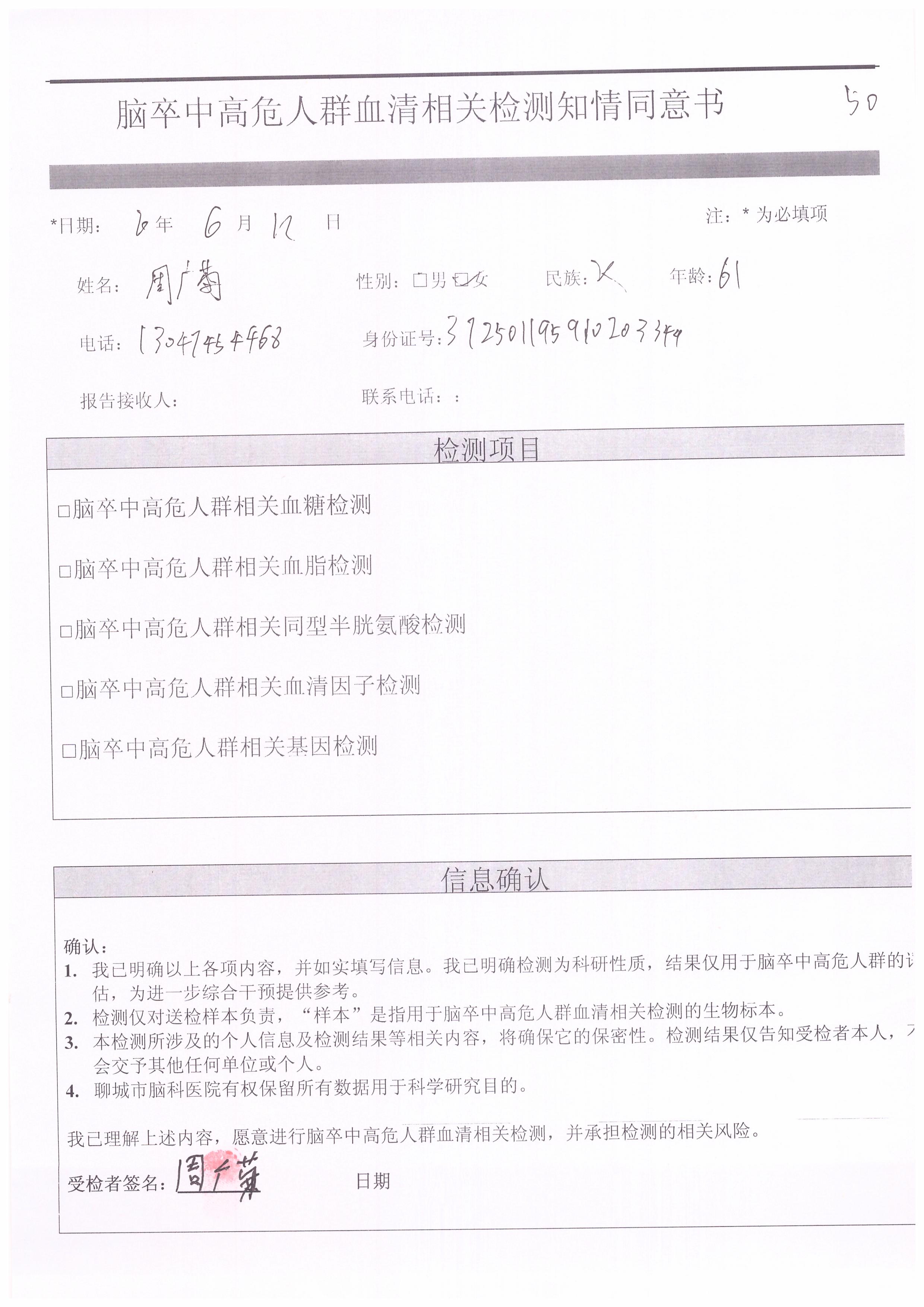

Supplement: Supplementary file 7 — Supplementary file7 (ZIP 27016 KB) [file 10528_2023_10431_MOESM7_ESM.zip › ╓¬╟Θ═1⁄4╥Γ╩Θ5/005.jpg]

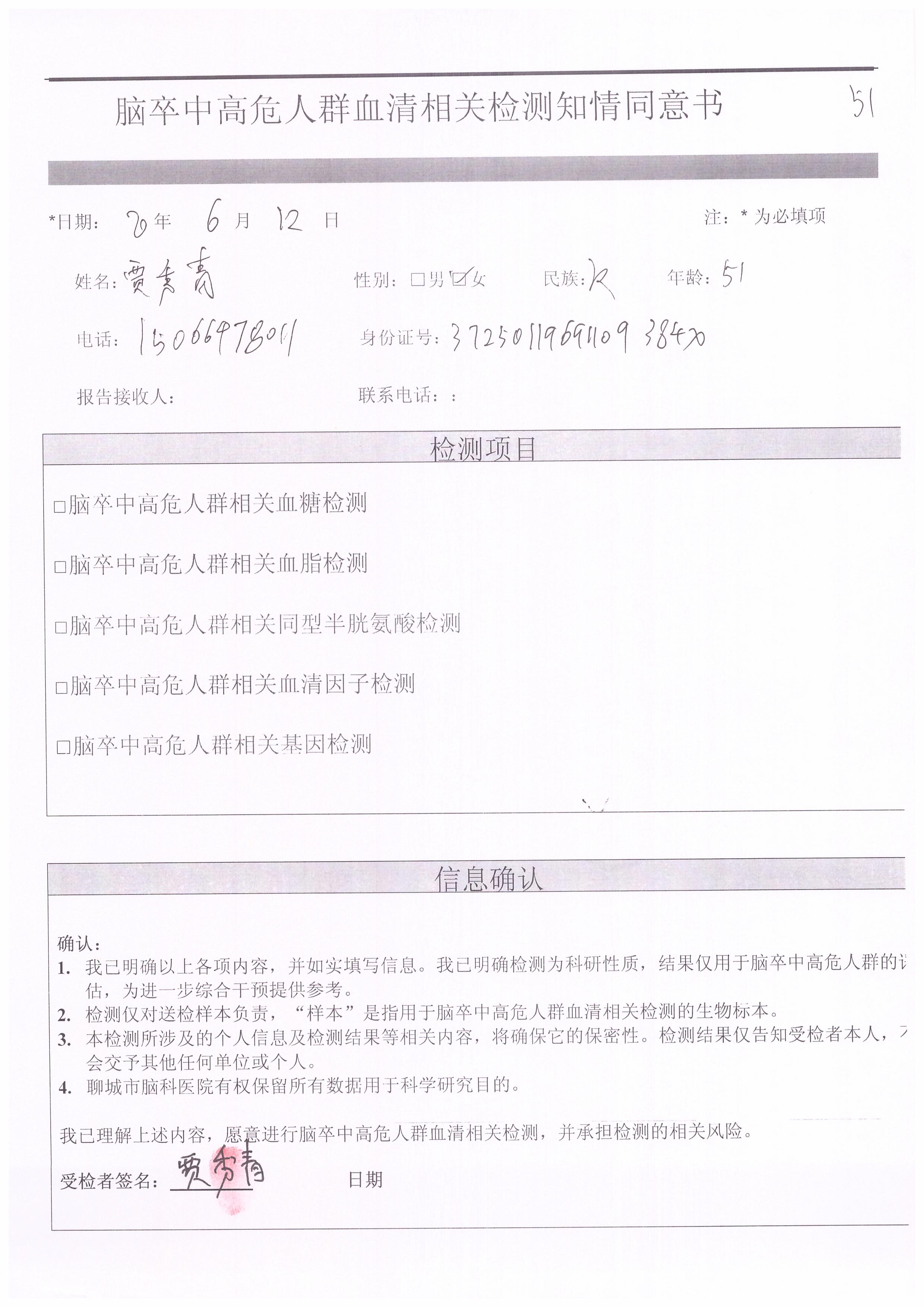

Supplement: Supplementary file 7 — Supplementary file7 (ZIP 27016 KB) [file 10528_2023_10431_MOESM7_ESM.zip › ╓¬╟Θ═1⁄4╥Γ╩Θ5/006.jpg]

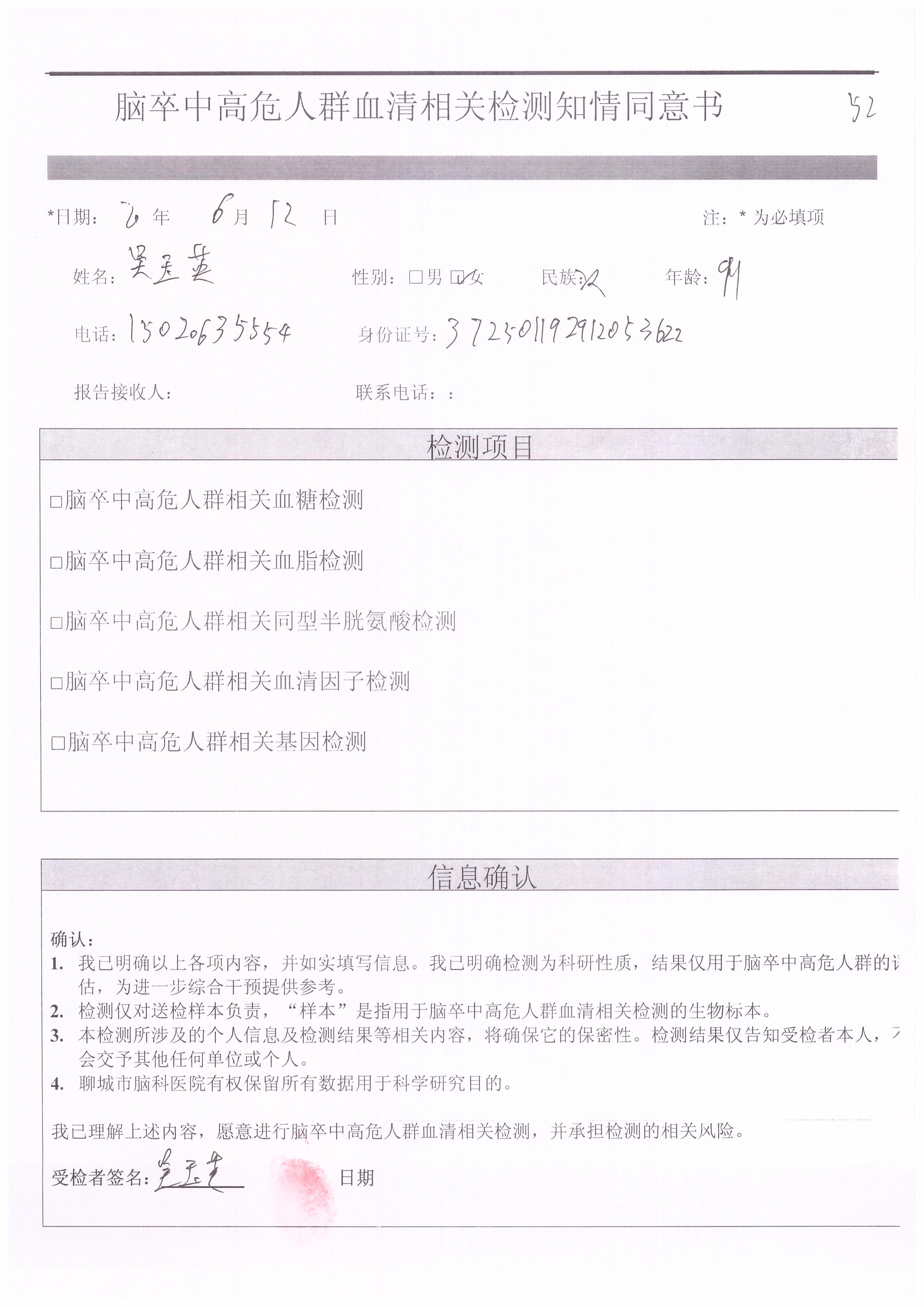

Supplement: Supplementary file 7 — Supplementary file7 (ZIP 27016 KB) [file 10528_2023_10431_MOESM7_ESM.zip › ╓¬╟Θ═1⁄4╥Γ╩Θ5/007.jpg]

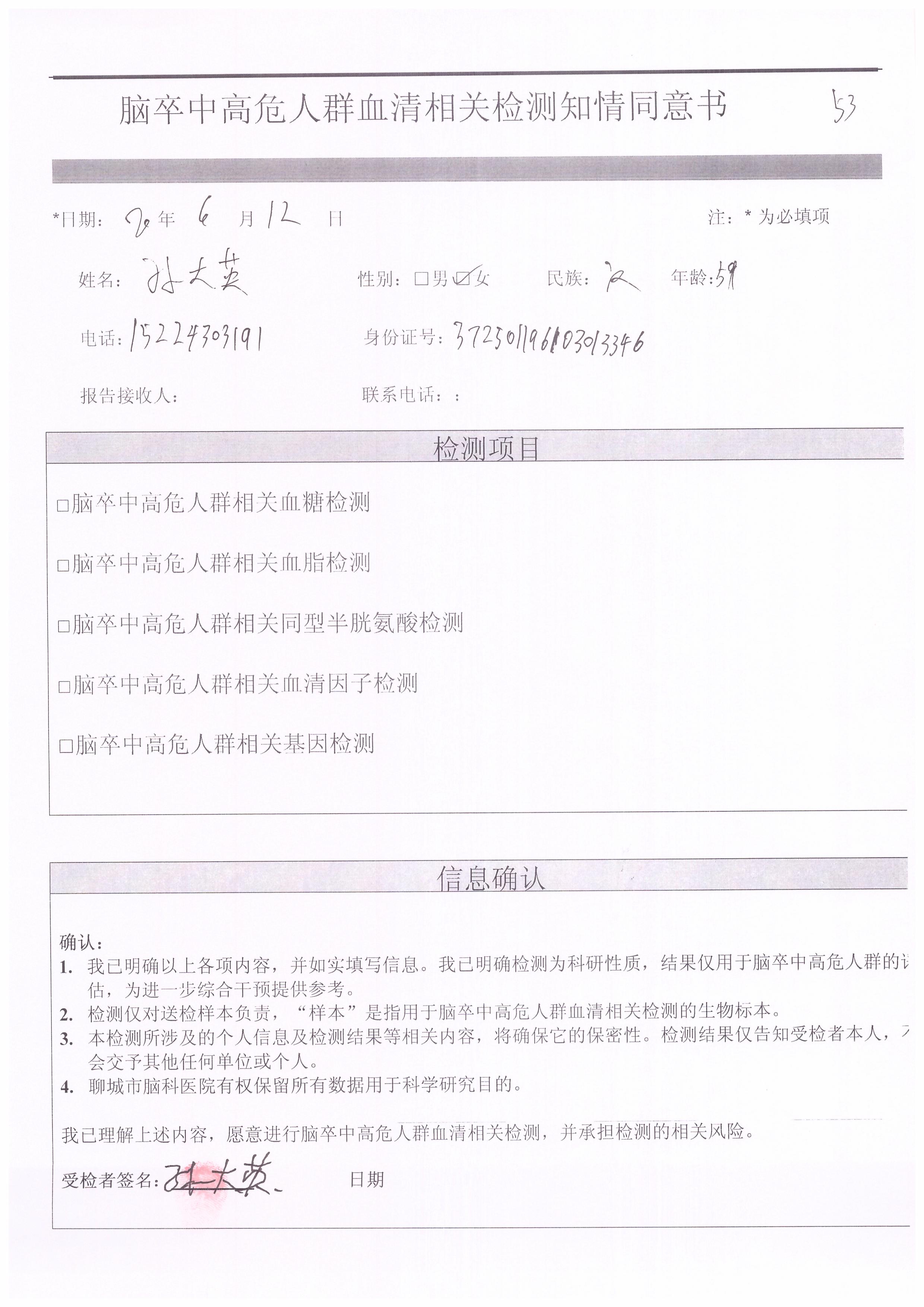

Supplement: Supplementary file 7 — Supplementary file7 (ZIP 27016 KB) [file 10528_2023_10431_MOESM7_ESM.zip › ╓¬╟Θ═1⁄4╥Γ╩Θ5/008.jpg]

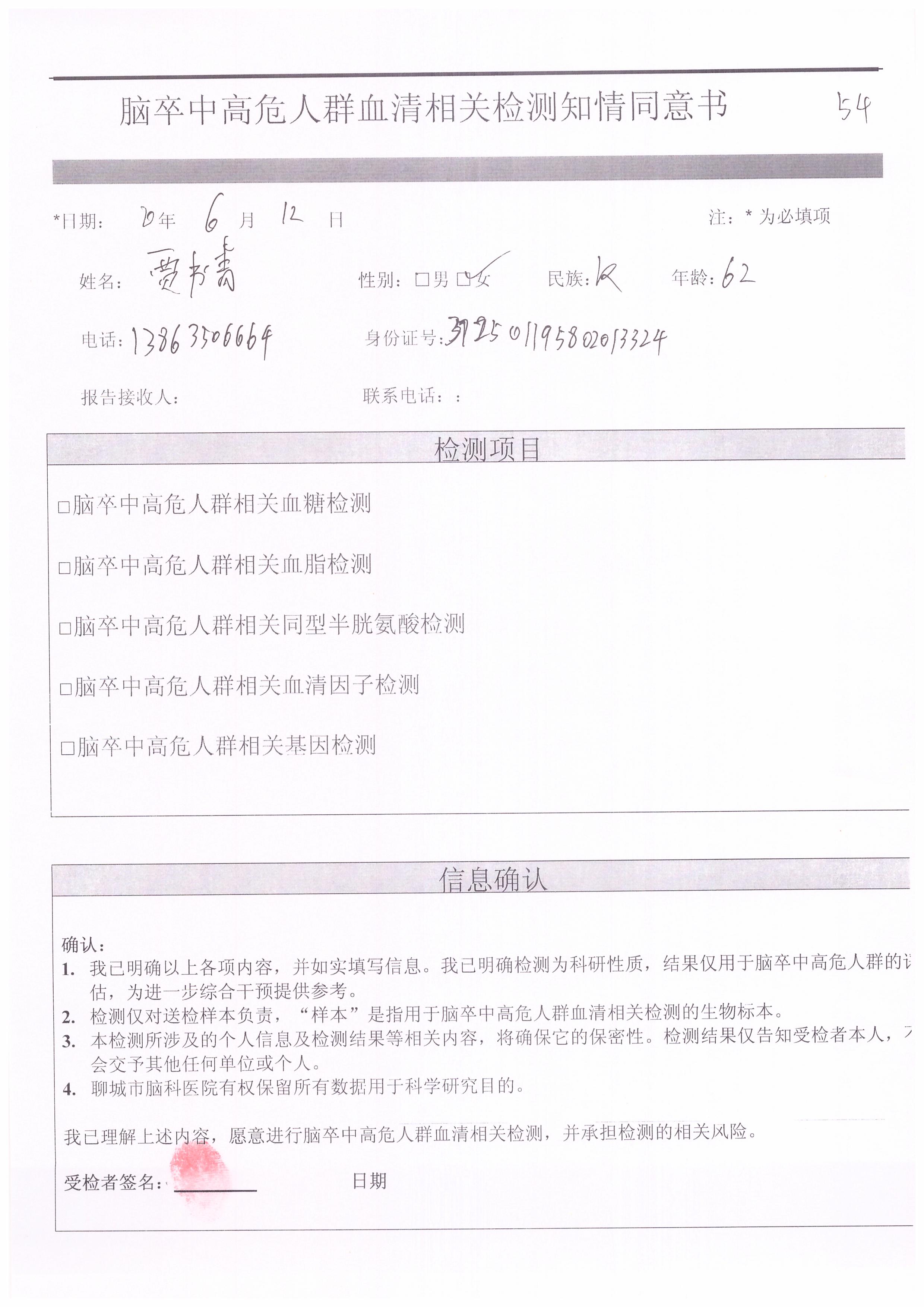

Supplement: Supplementary file 7 — Supplementary file7 (ZIP 27016 KB) [file 10528_2023_10431_MOESM7_ESM.zip › ╓¬╟Θ═1⁄4╥Γ╩Θ5/009.jpg]

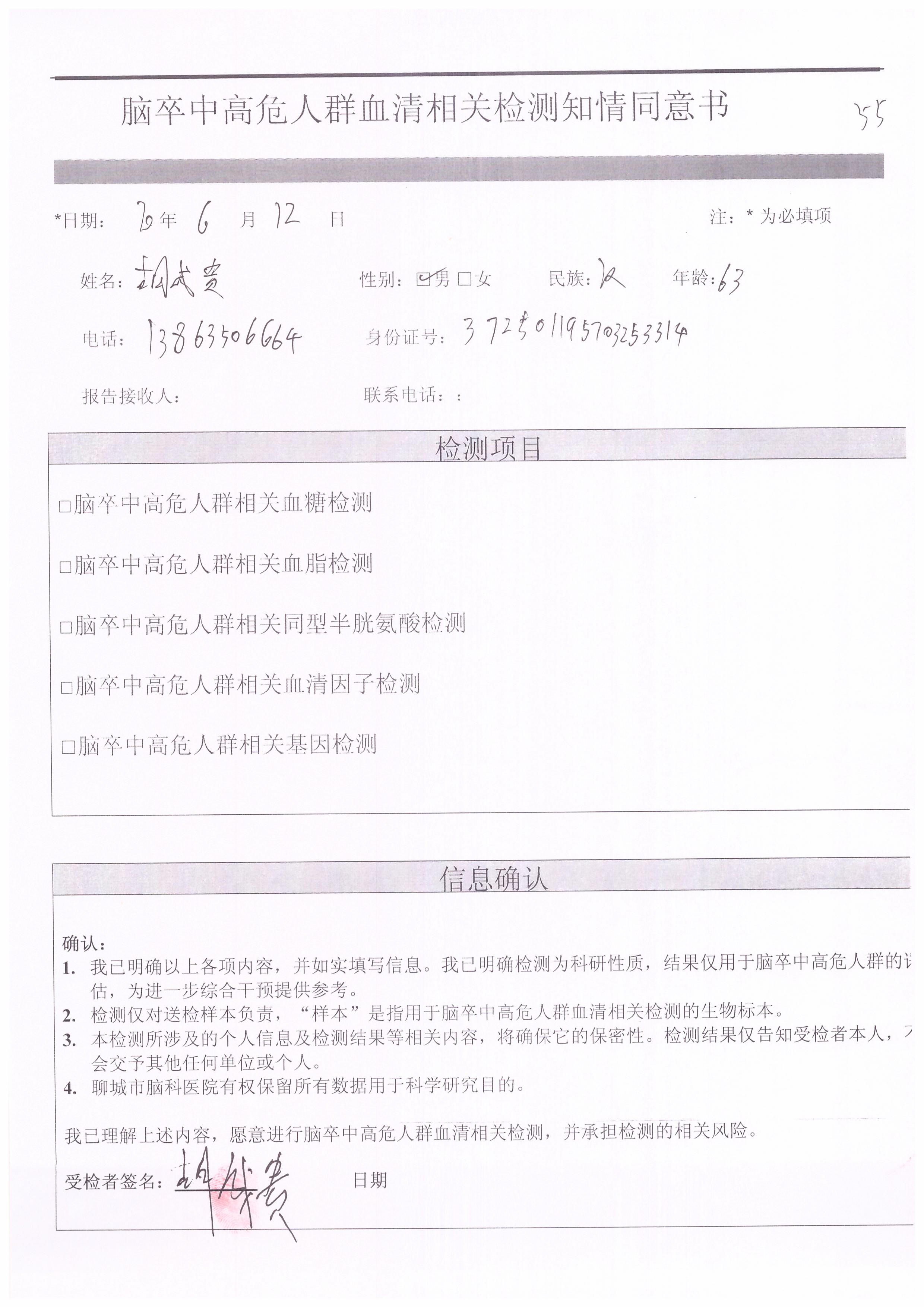

Supplement: Supplementary file 7 — Supplementary file7 (ZIP 27016 KB) [file 10528_2023_10431_MOESM7_ESM.zip › ╓¬╟Θ═1⁄4╥Γ╩Θ5/010.jpg]

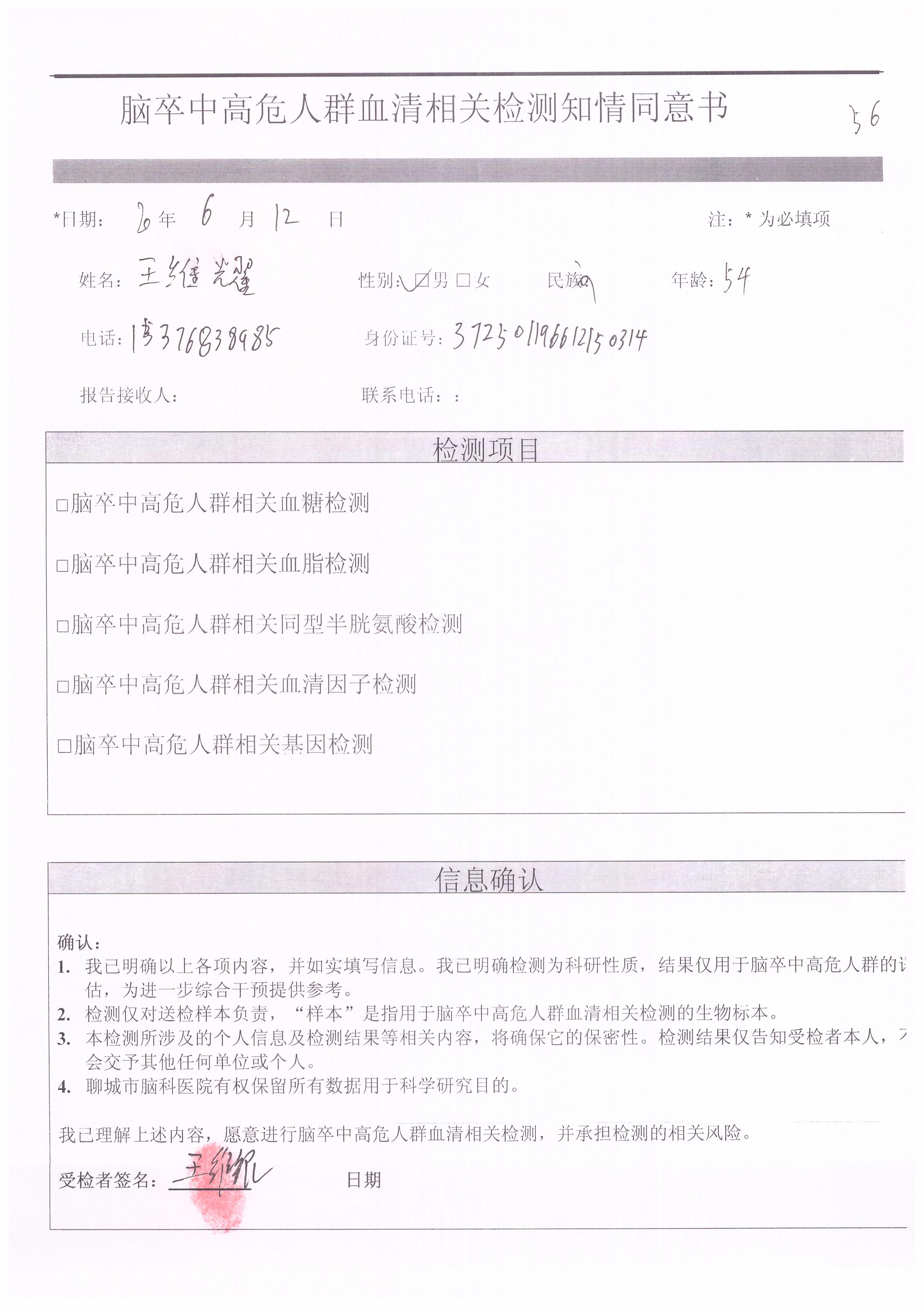

Supplement: Supplementary file 7 — Supplementary file7 (ZIP 27016 KB) [file 10528_2023_10431_MOESM7_ESM.zip › ╓¬╟Θ═1⁄4╥Γ╩Θ5/011.jpg]

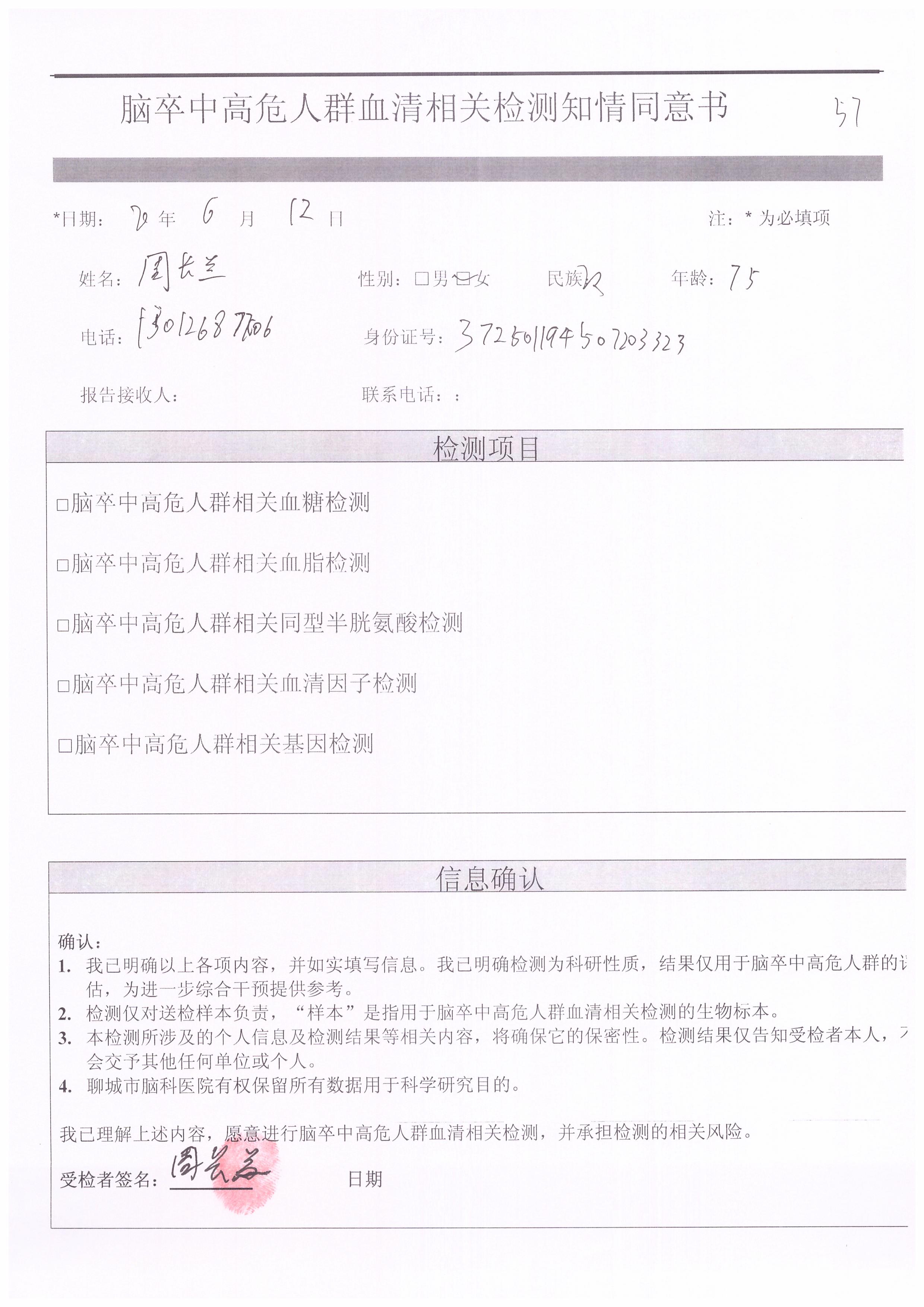

Supplement: Supplementary file 7 — Supplementary file7 (ZIP 27016 KB) [file 10528_2023_10431_MOESM7_ESM.zip › ╓¬╟Θ═1⁄4╥Γ╩Θ5/012.jpg]

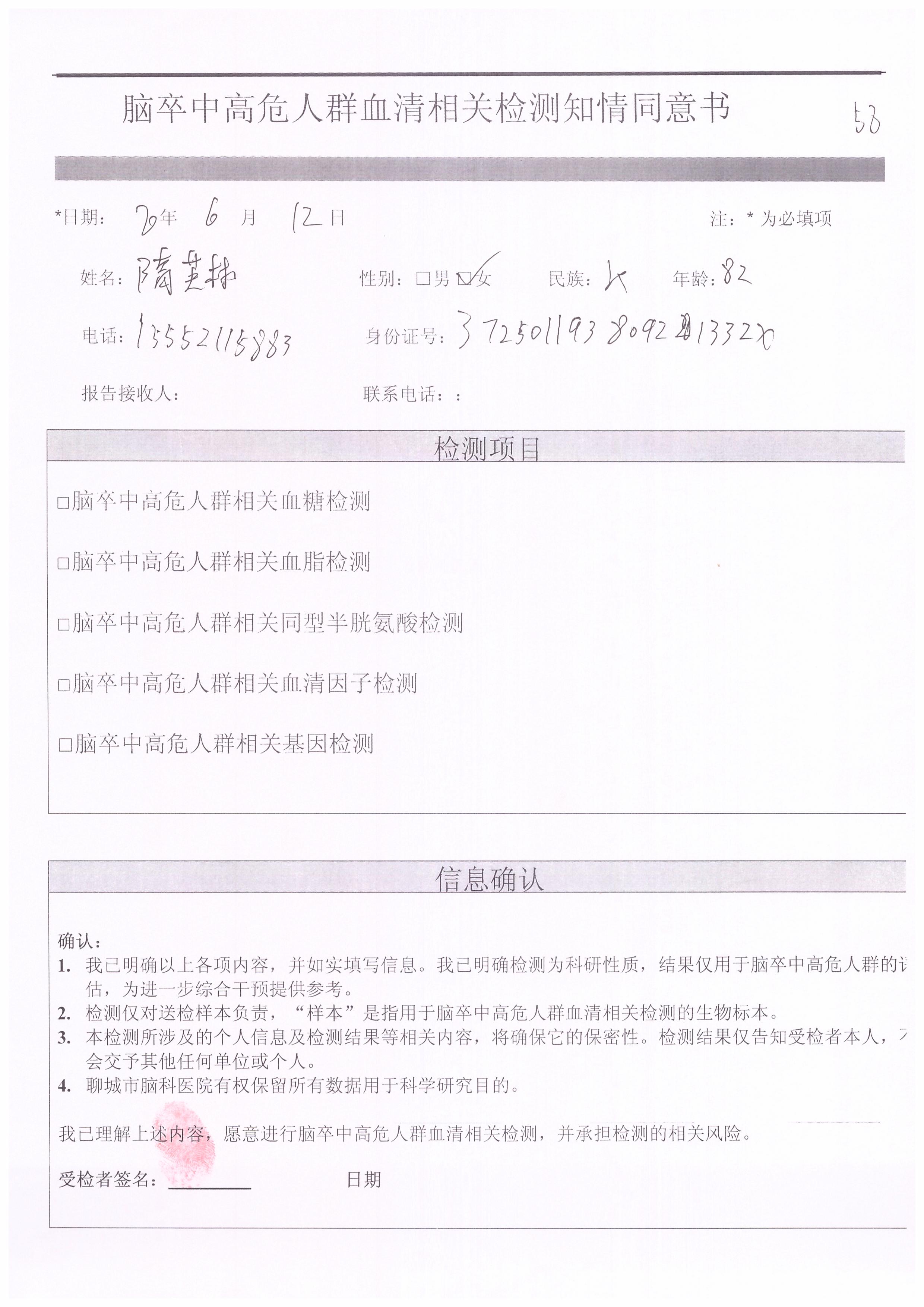

Supplement: Supplementary file 7 — Supplementary file7 (ZIP 27016 KB) [file 10528_2023_10431_MOESM7_ESM.zip › ╓¬╟Θ═1⁄4╥Γ╩Θ5/013.jpg]
