# Supplementary figures and images for: The Association Between Thymidylate Synthase Gene Polymorphisms and the Risk of Ischemic Stroke in Chinese Han Population (part 3 of 6)
Source: Biochem Genet. 2023 Jun 28;62(1):468–84. doi: 10.1007/s10528-023-10431-8 (PMC10901929; doi:10.1007/s10528-023-10431-8)

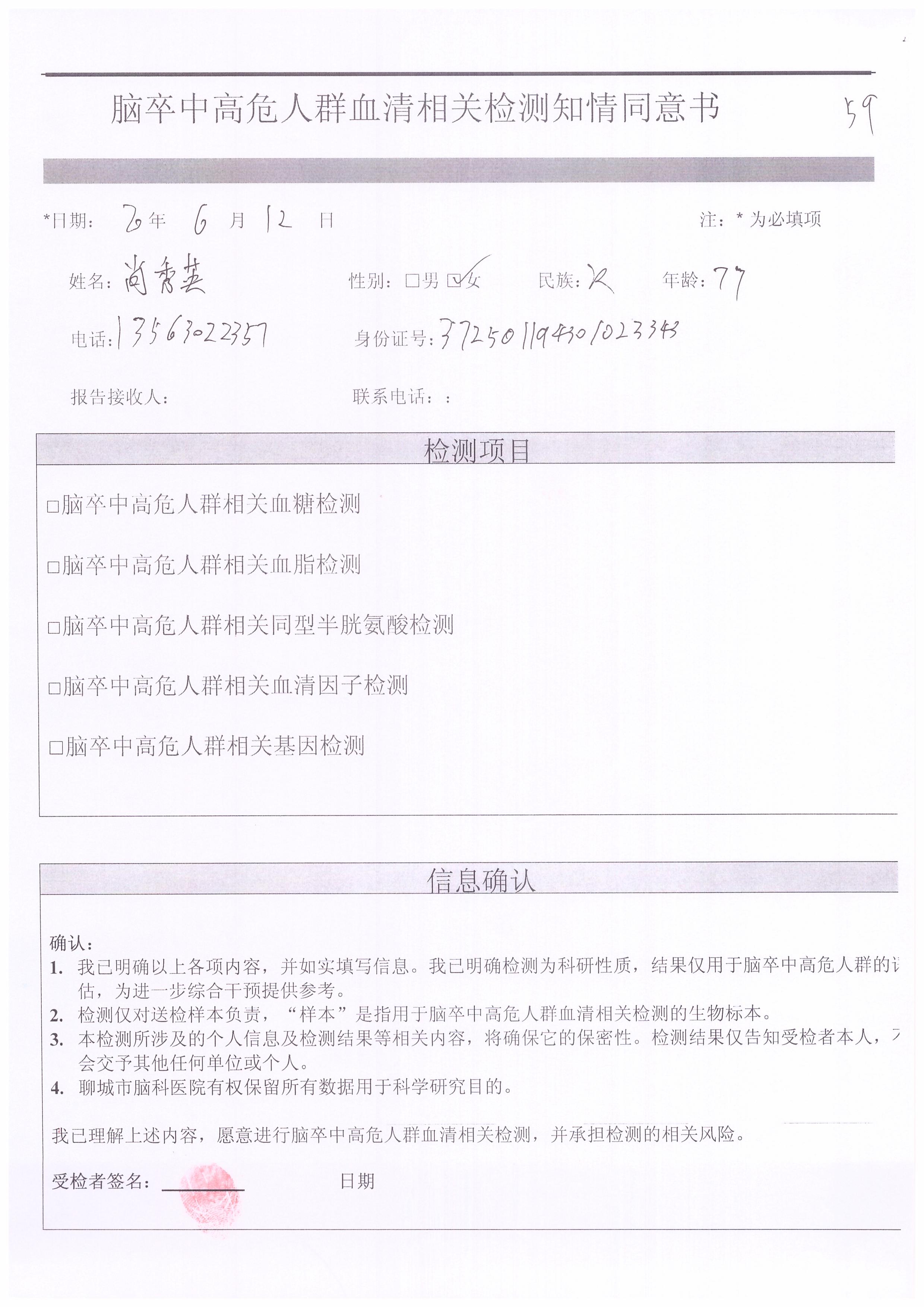

Supplement: Supplementary file 7 — Supplementary file7 (ZIP 27016 KB) [file 10528_2023_10431_MOESM7_ESM.zip › ╓¬╟Θ═1⁄4╥Γ╩Θ5/014.jpg]

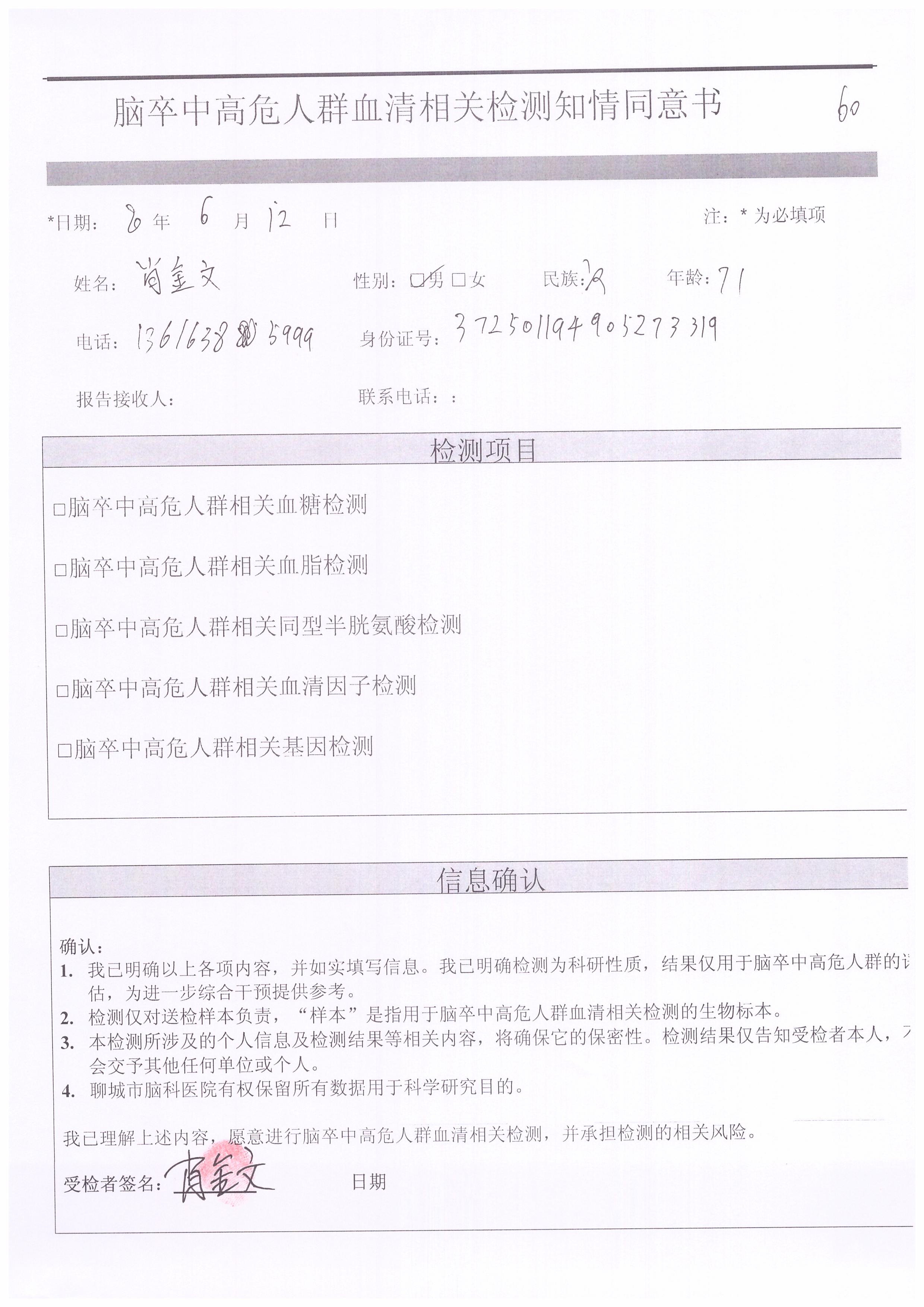

Supplement: Supplementary file 7 — Supplementary file7 (ZIP 27016 KB) [file 10528_2023_10431_MOESM7_ESM.zip › ╓¬╟Θ═1⁄4╥Γ╩Θ5/015.jpg]

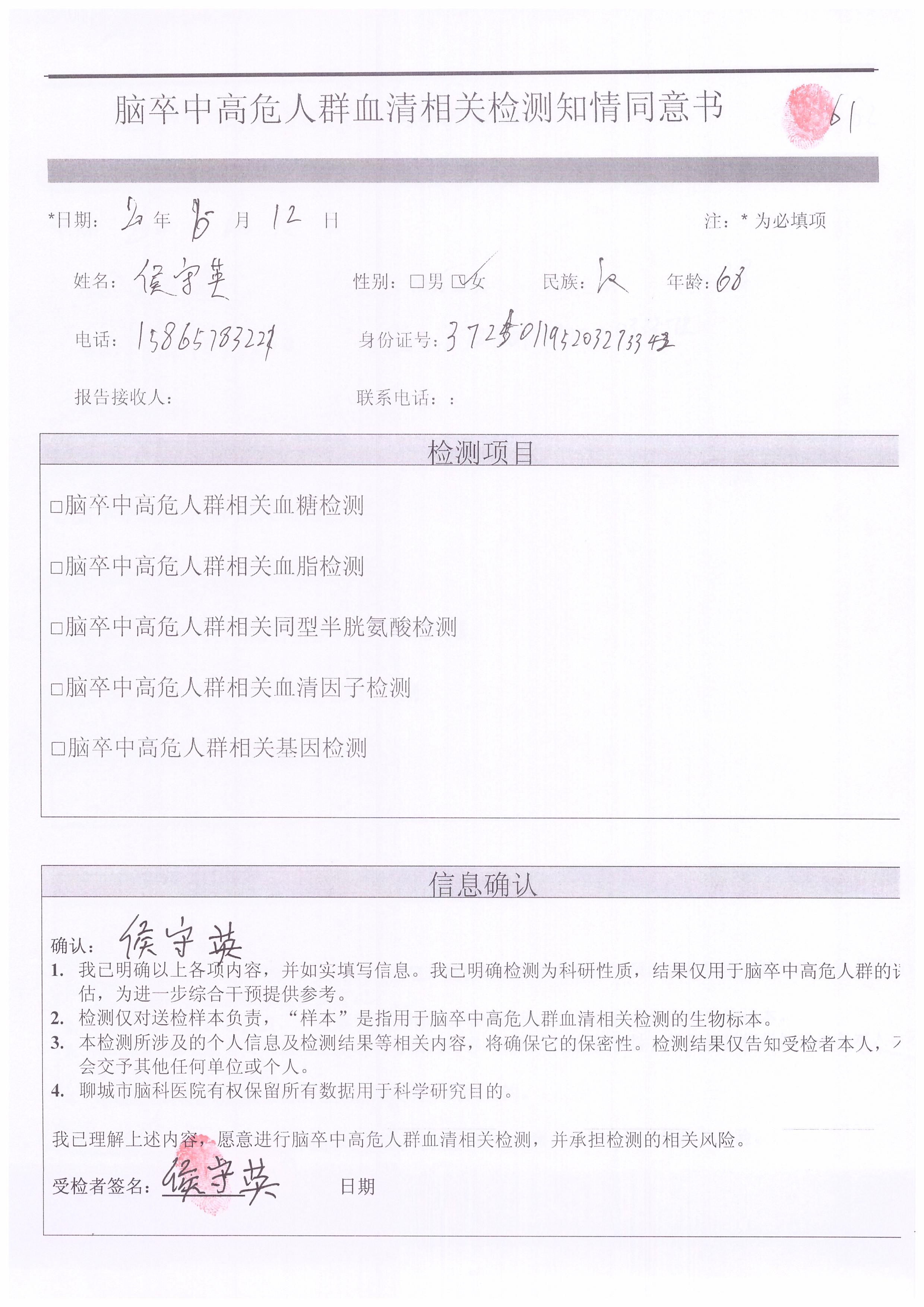

Supplement: Supplementary file 7 — Supplementary file7 (ZIP 27016 KB) [file 10528_2023_10431_MOESM7_ESM.zip › ╓¬╟Θ═1⁄4╥Γ╩Θ5/016.jpg]

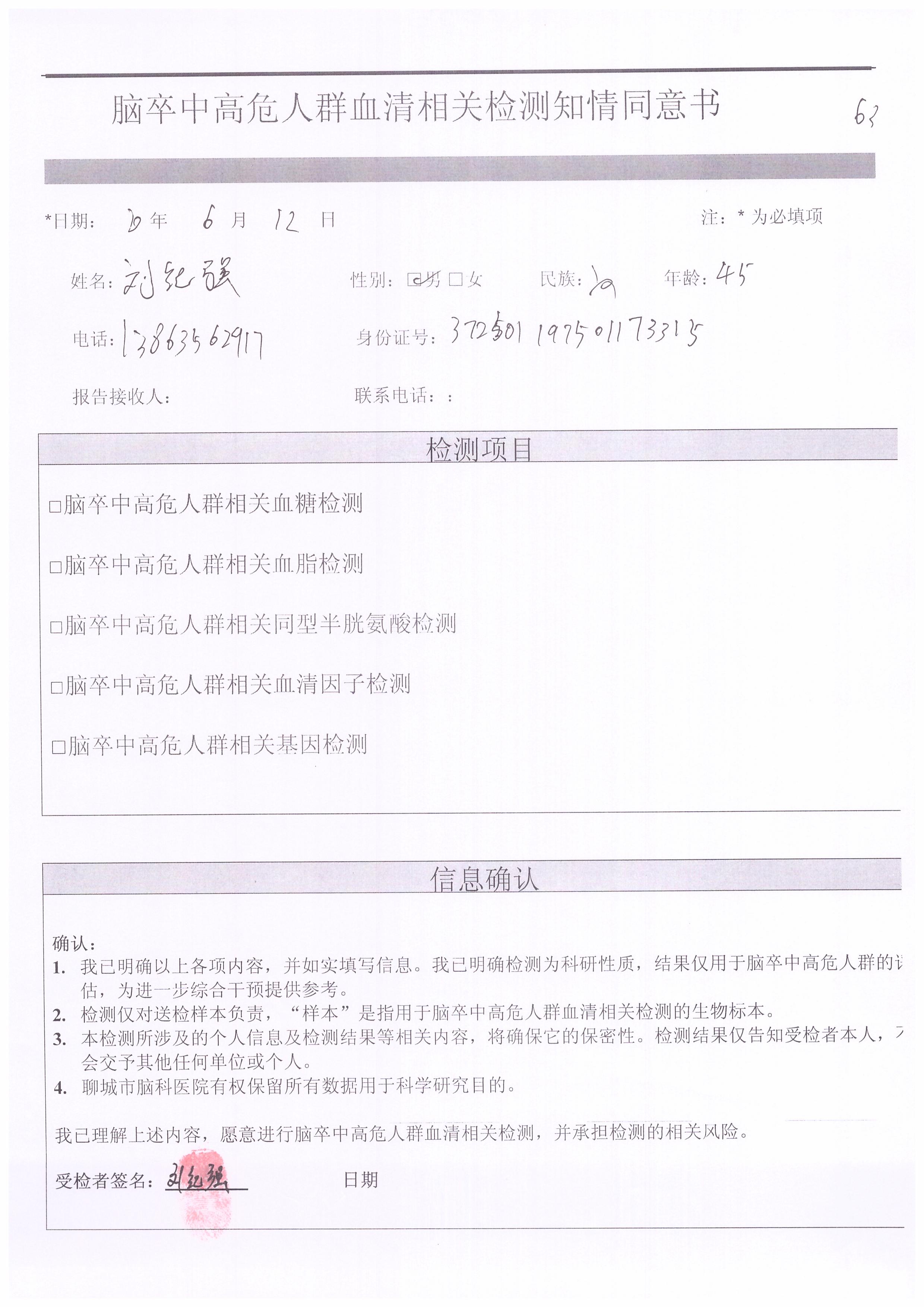

Supplement: Supplementary file 7 — Supplementary file7 (ZIP 27016 KB) [file 10528_2023_10431_MOESM7_ESM.zip › ╓¬╟Θ═1⁄4╥Γ╩Θ5/017.jpg]

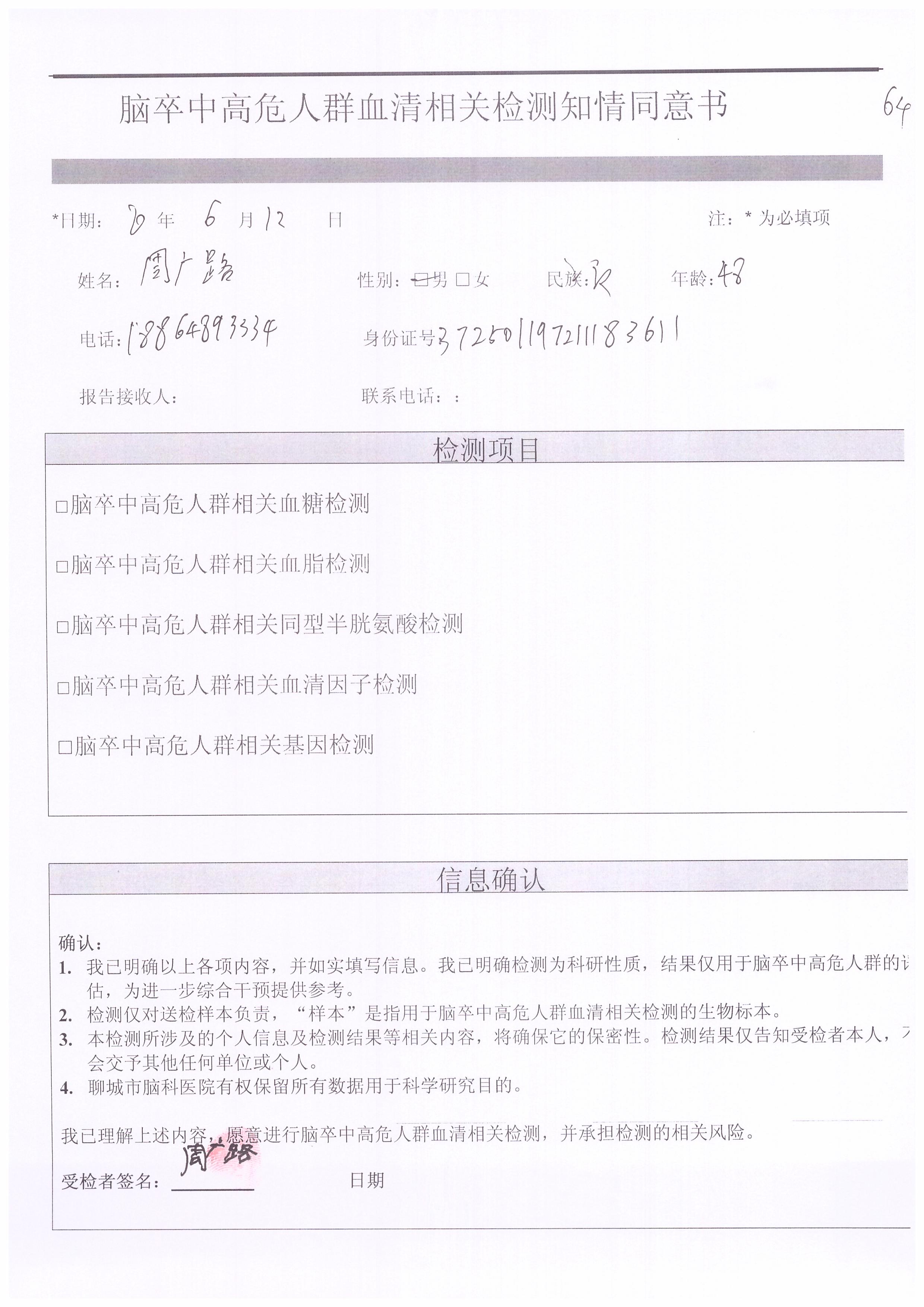

Supplement: Supplementary file 7 — Supplementary file7 (ZIP 27016 KB) [file 10528_2023_10431_MOESM7_ESM.zip › ╓¬╟Θ═1⁄4╥Γ╩Θ5/018.jpg]

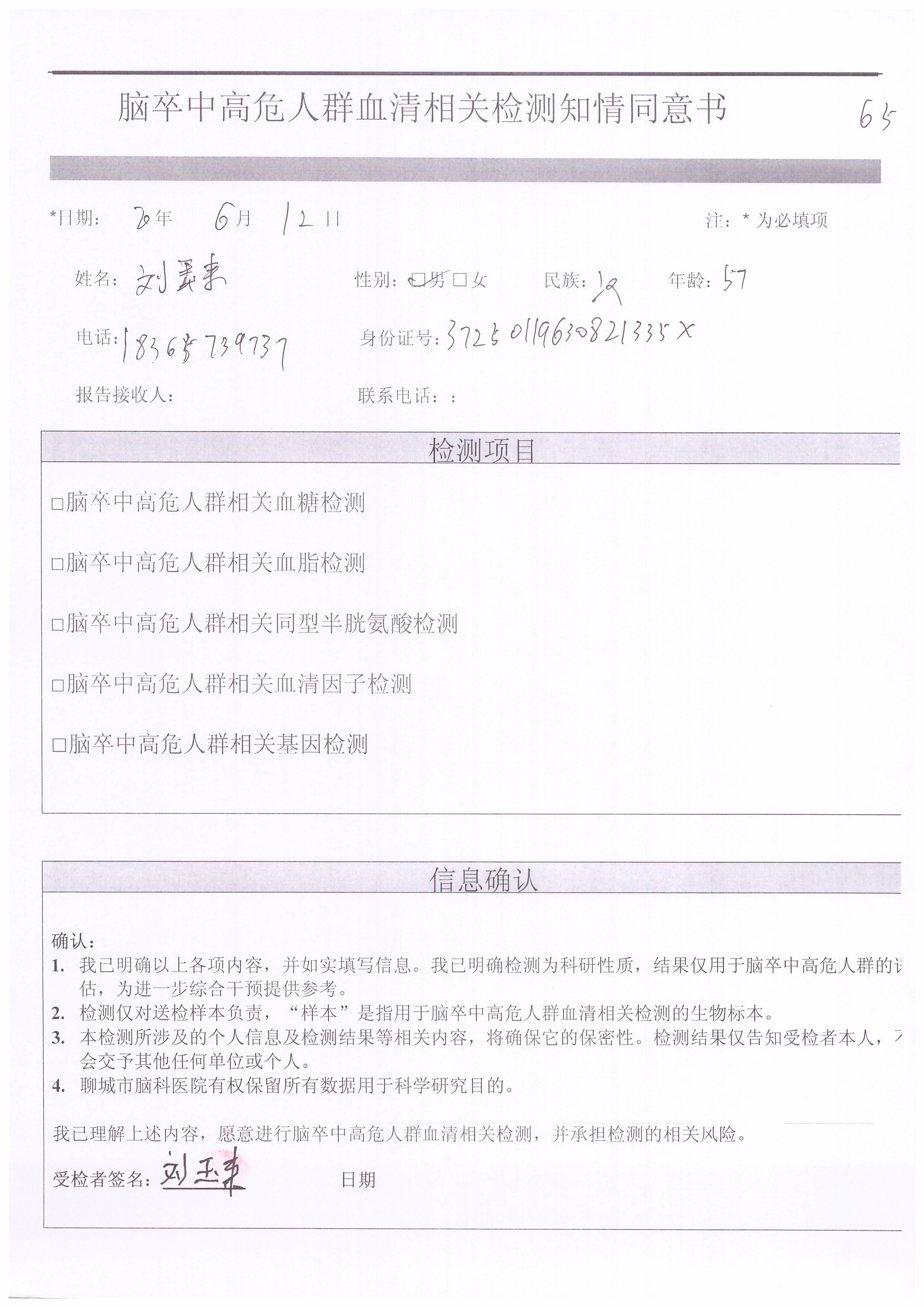

Supplement: Supplementary file 7 — Supplementary file7 (ZIP 27016 KB) [file 10528_2023_10431_MOESM7_ESM.zip › ╓¬╟Θ═1⁄4╥Γ╩Θ5/019.jpg]

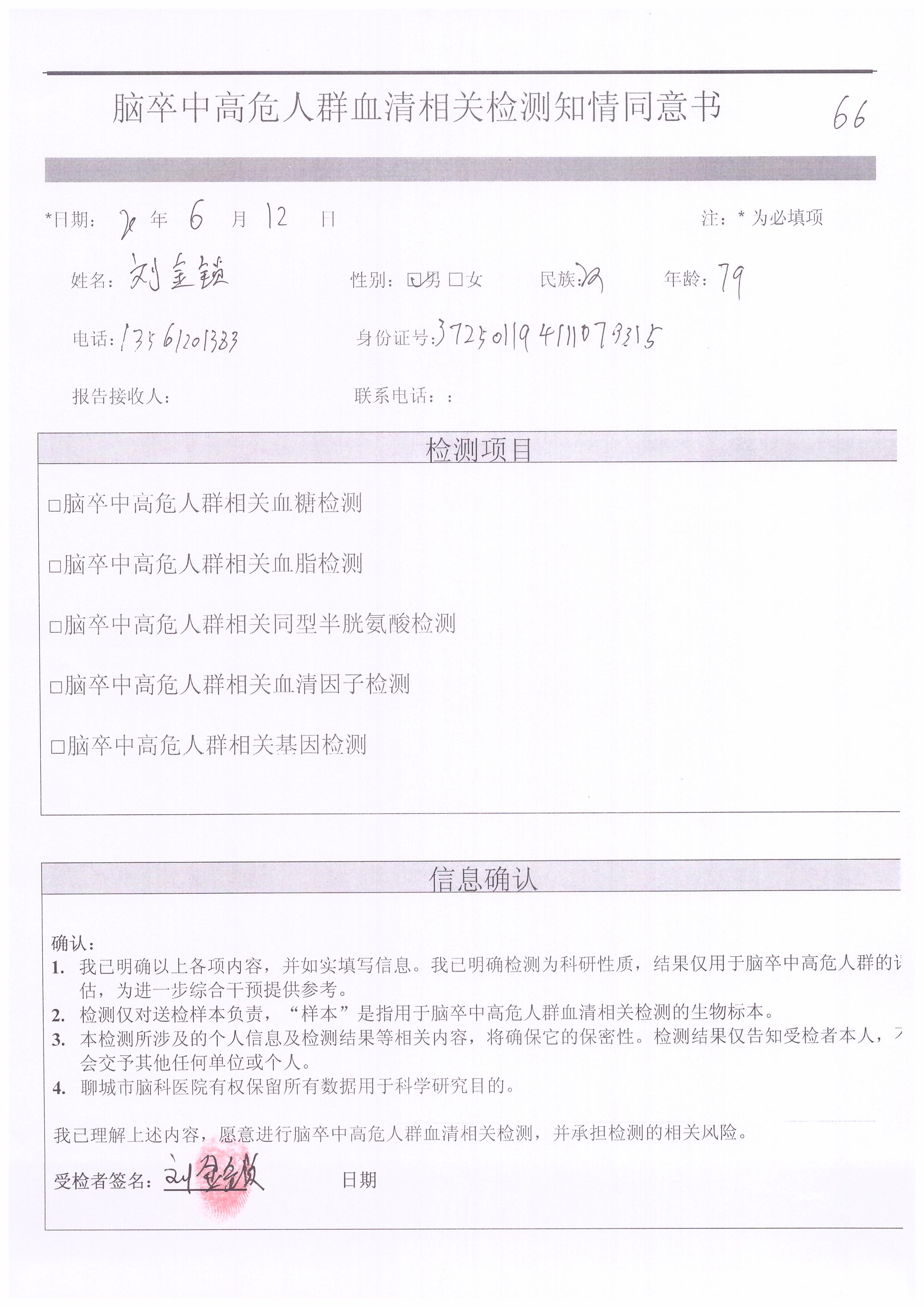

Supplement: Supplementary file 7 — Supplementary file7 (ZIP 27016 KB) [file 10528_2023_10431_MOESM7_ESM.zip › ╓¬╟Θ═1⁄4╥Γ╩Θ5/020.jpg]

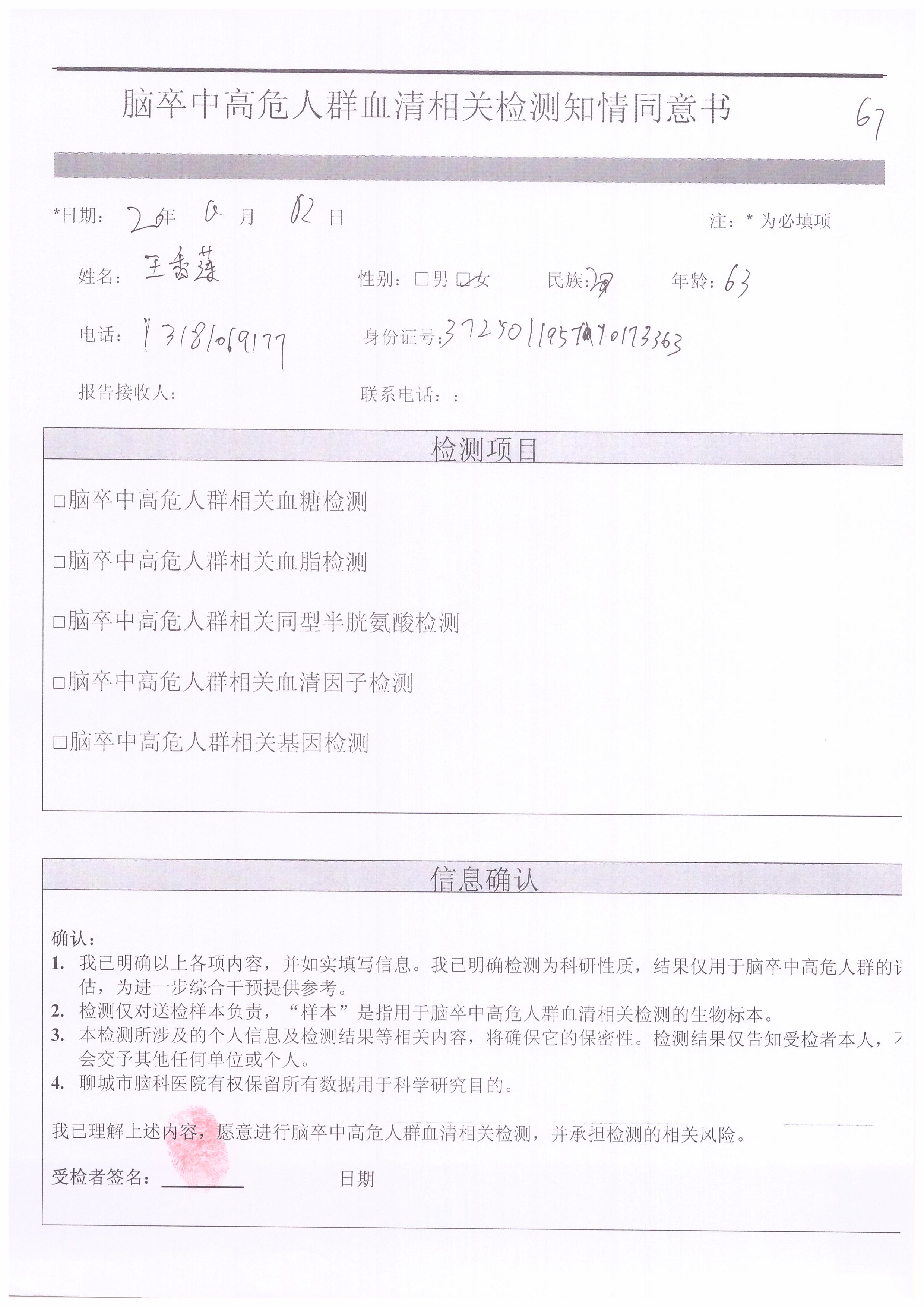

Supplement: Supplementary file 7 — Supplementary file7 (ZIP 27016 KB) [file 10528_2023_10431_MOESM7_ESM.zip › ╓¬╟Θ═1⁄4╥Γ╩Θ5/021.jpg]

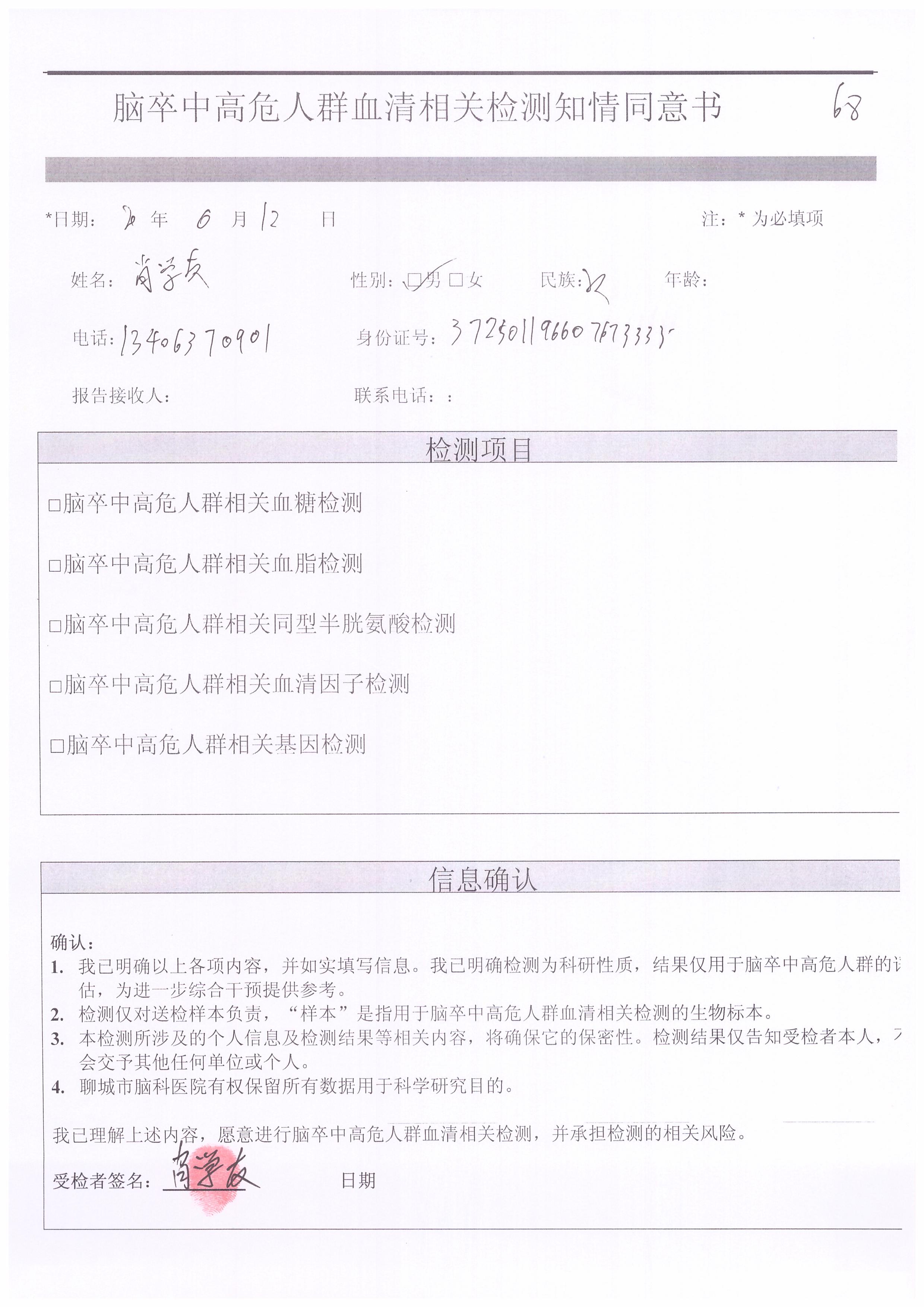

Supplement: Supplementary file 7 — Supplementary file7 (ZIP 27016 KB) [file 10528_2023_10431_MOESM7_ESM.zip › ╓¬╟Θ═1⁄4╥Γ╩Θ5/022.jpg]

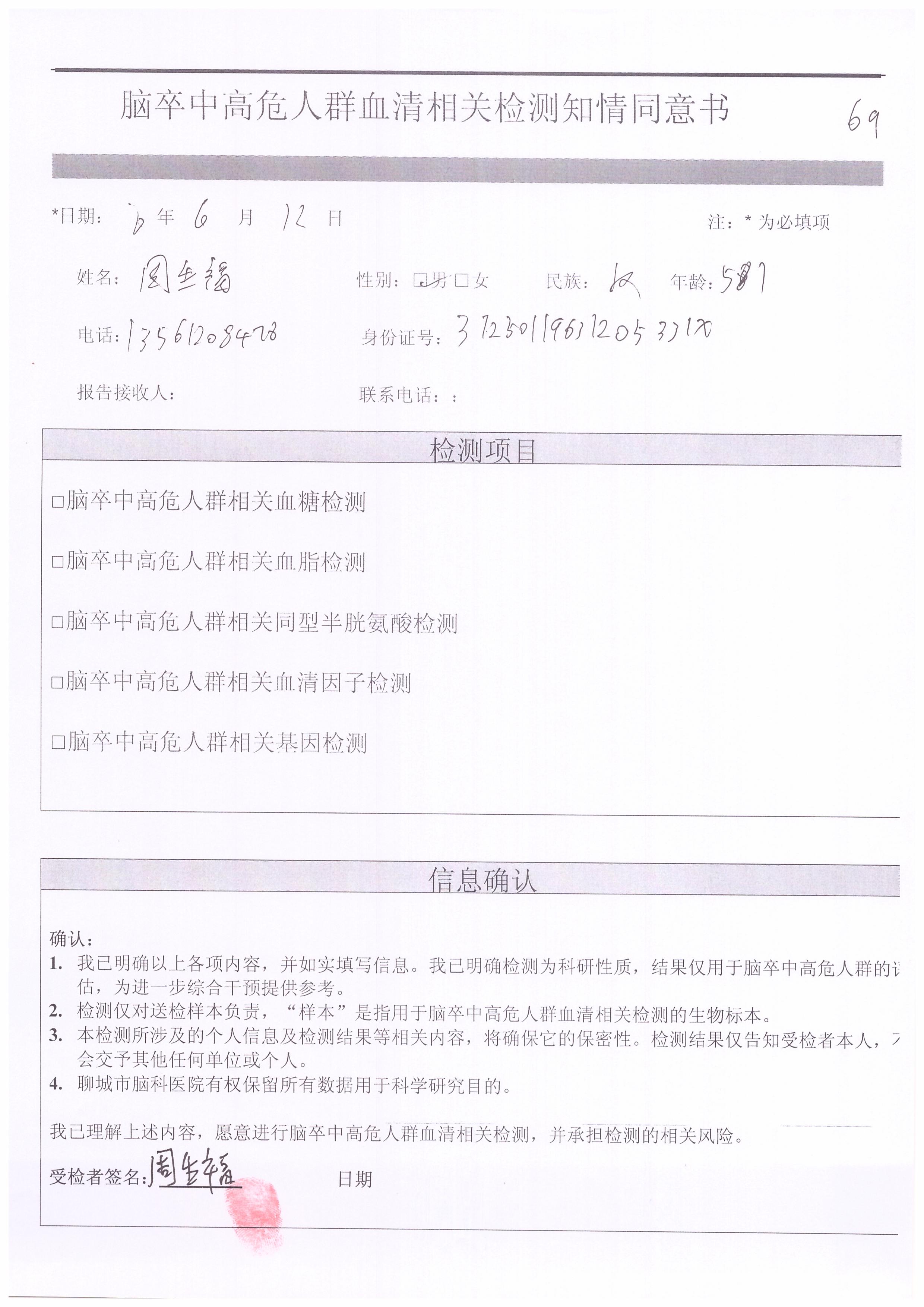

Supplement: Supplementary file 7 — Supplementary file7 (ZIP 27016 KB) [file 10528_2023_10431_MOESM7_ESM.zip › ╓¬╟Θ═1⁄4╥Γ╩Θ5/023.jpg]

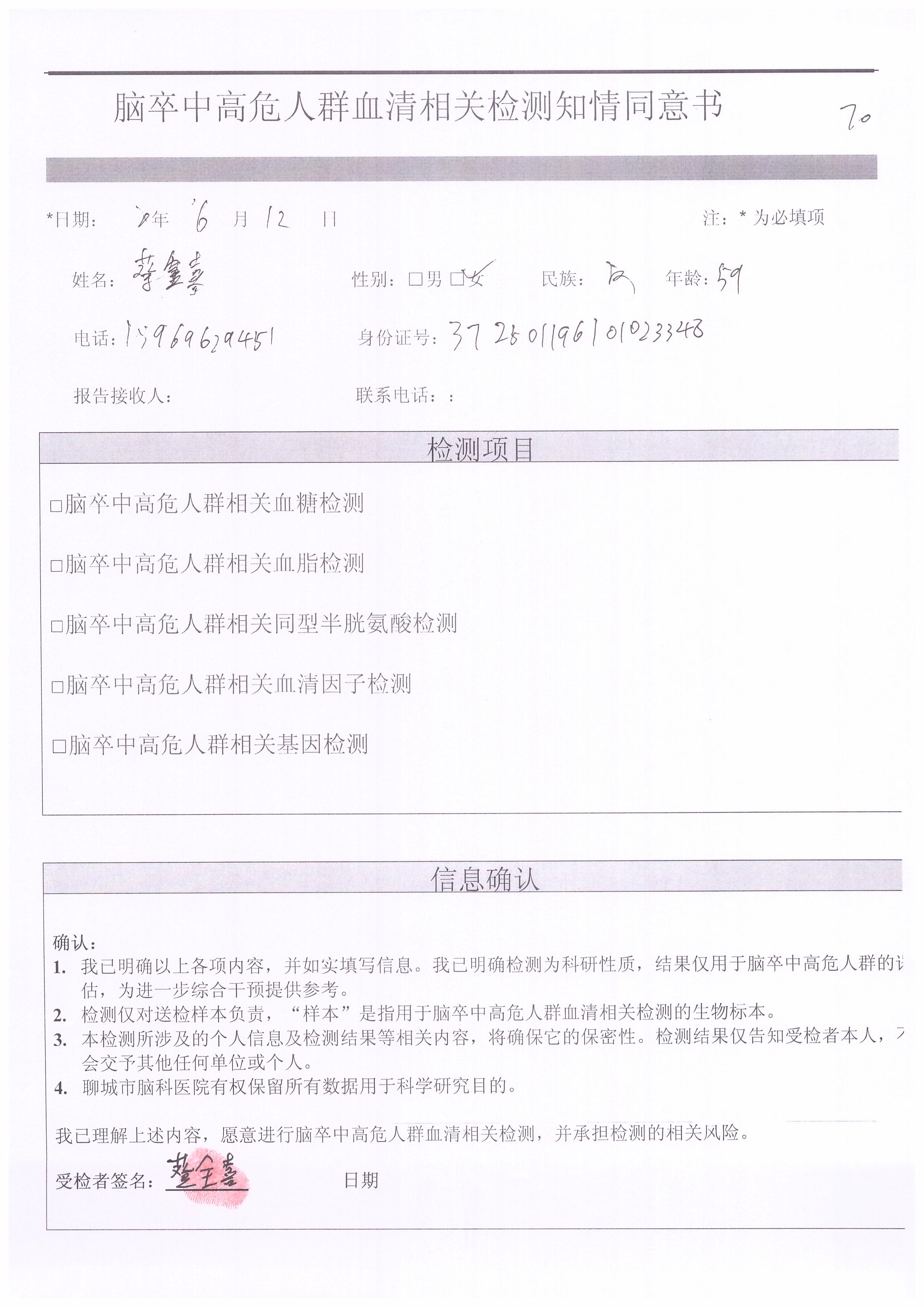

Supplement: Supplementary file 7 — Supplementary file7 (ZIP 27016 KB) [file 10528_2023_10431_MOESM7_ESM.zip › ╓¬╟Θ═1⁄4╥Γ╩Θ5/024.jpg]

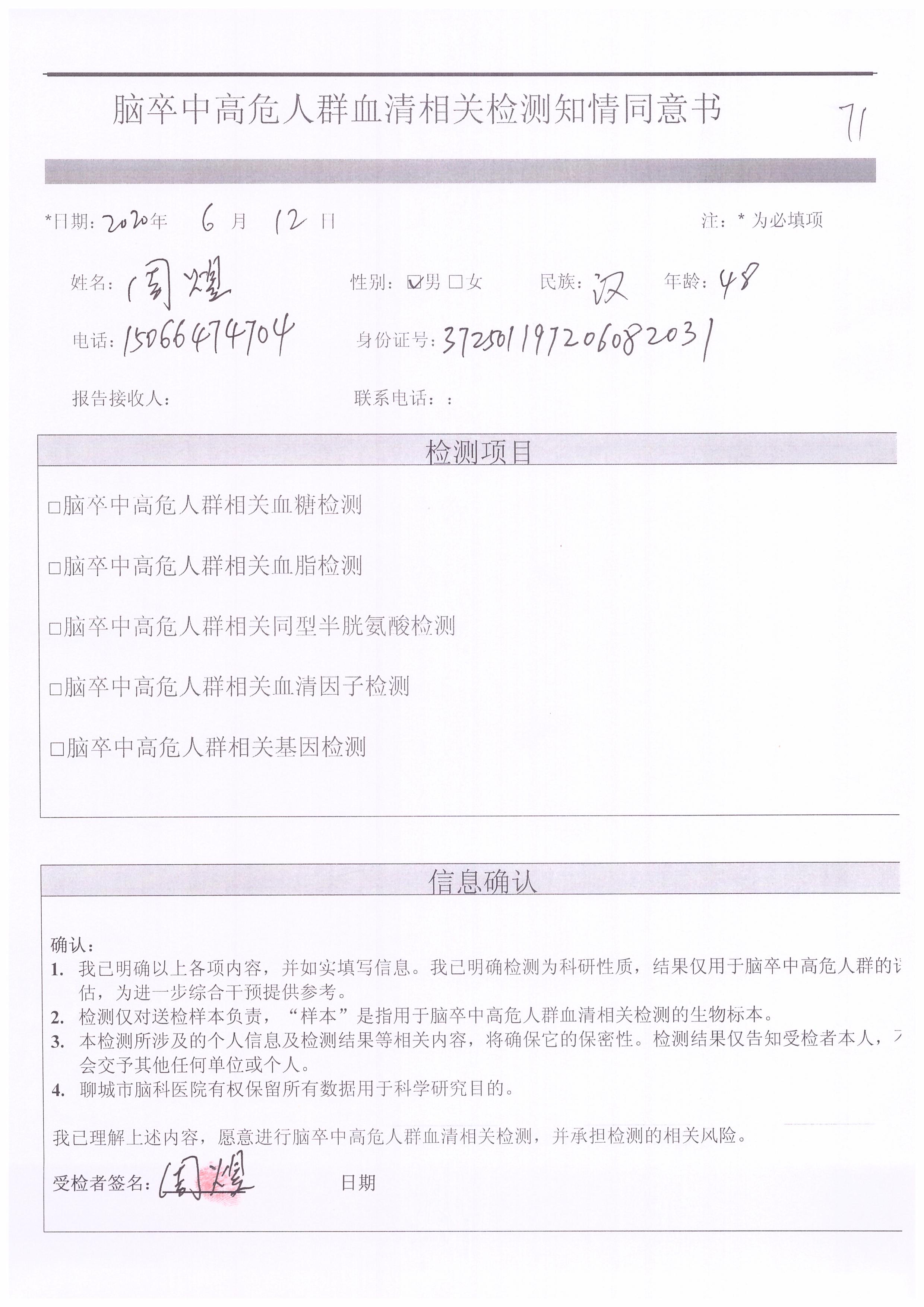

Supplement: Supplementary file 7 — Supplementary file7 (ZIP 27016 KB) [file 10528_2023_10431_MOESM7_ESM.zip › ╓¬╟Θ═1⁄4╥Γ╩Θ5/025.jpg]

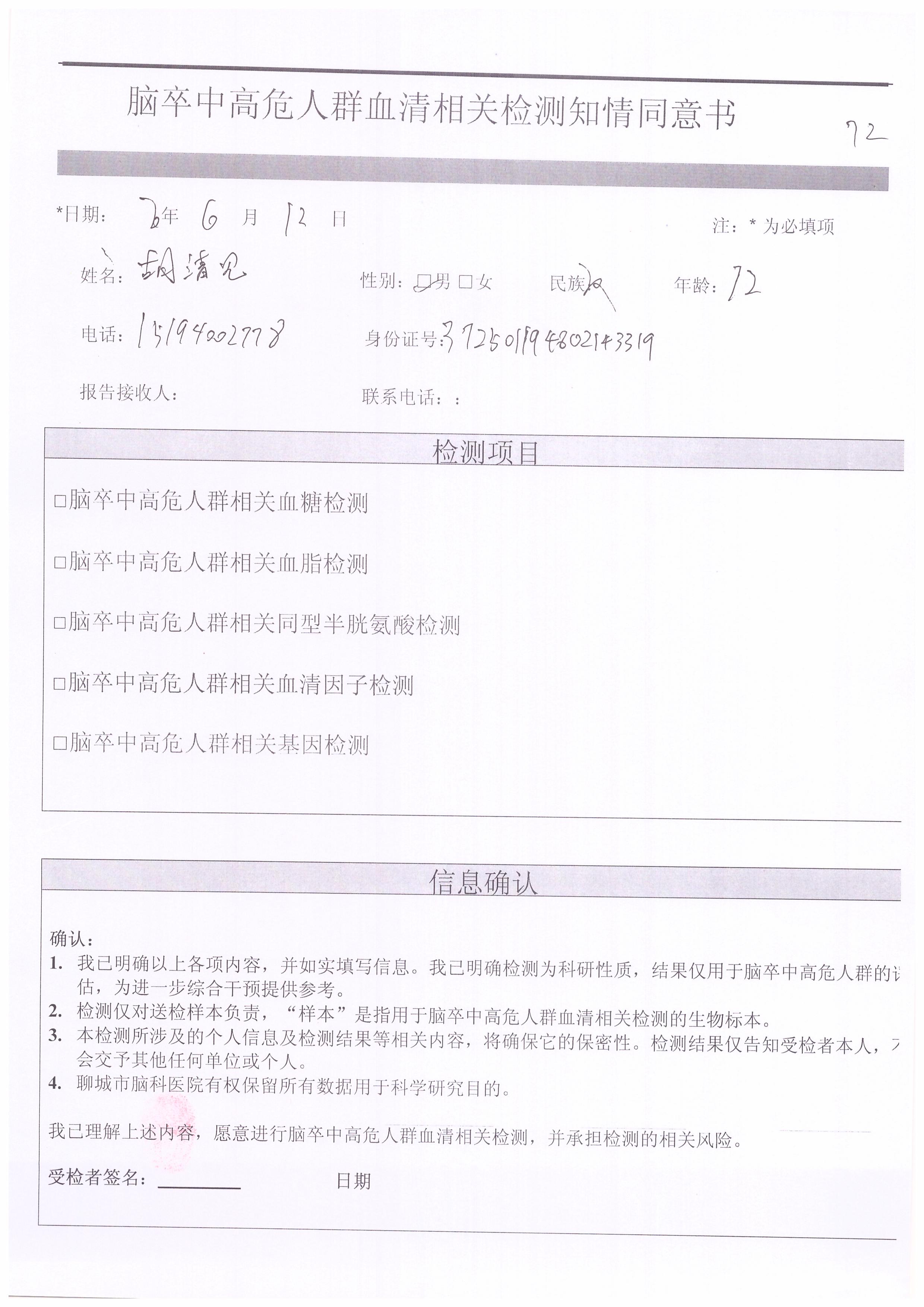

Supplement: Supplementary file 7 — Supplementary file7 (ZIP 27016 KB) [file 10528_2023_10431_MOESM7_ESM.zip › ╓¬╟Θ═1⁄4╥Γ╩Θ5/026.jpg]

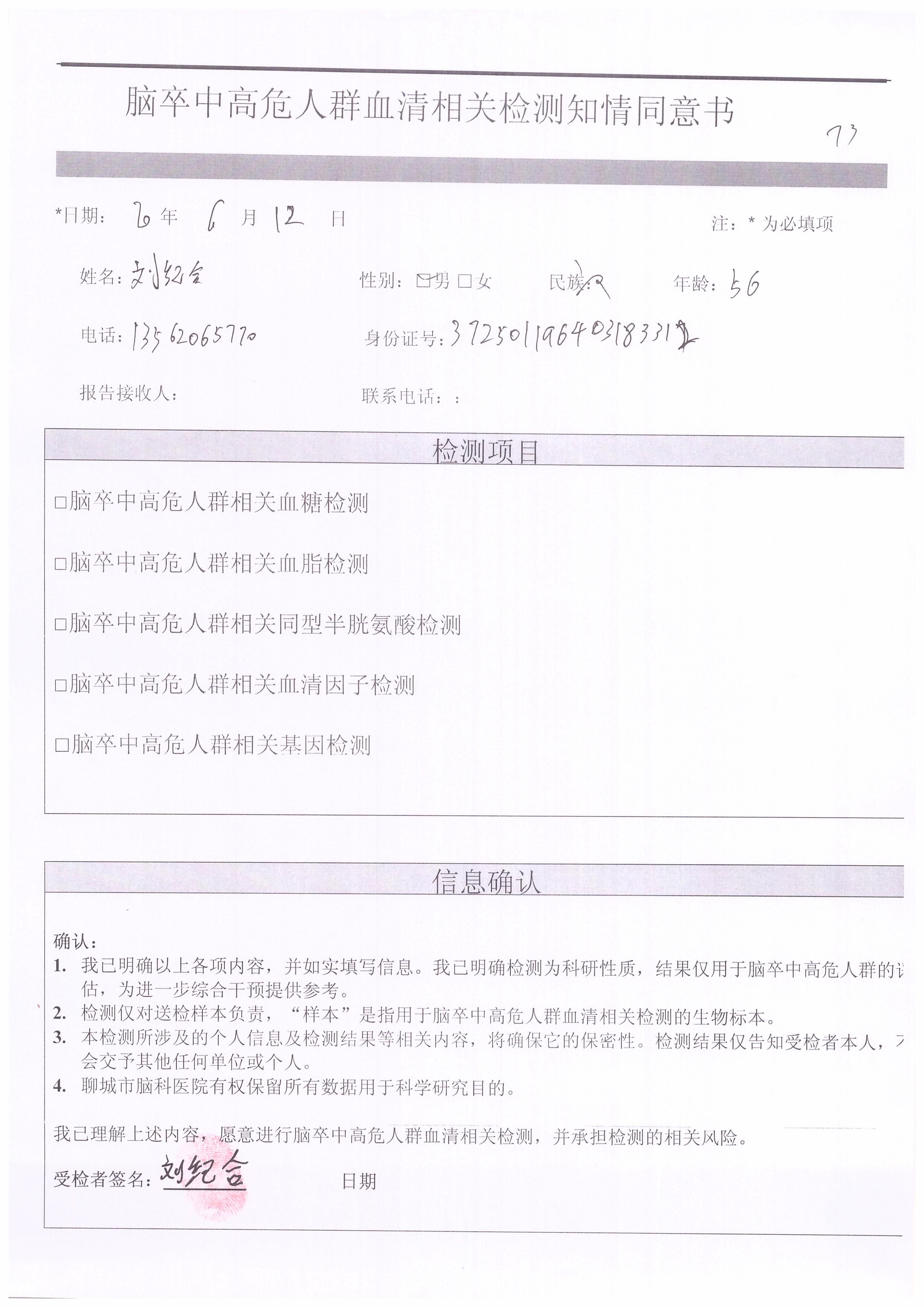

Supplement: Supplementary file 7 — Supplementary file7 (ZIP 27016 KB) [file 10528_2023_10431_MOESM7_ESM.zip › ╓¬╟Θ═1⁄4╥Γ╩Θ5/027.jpg]

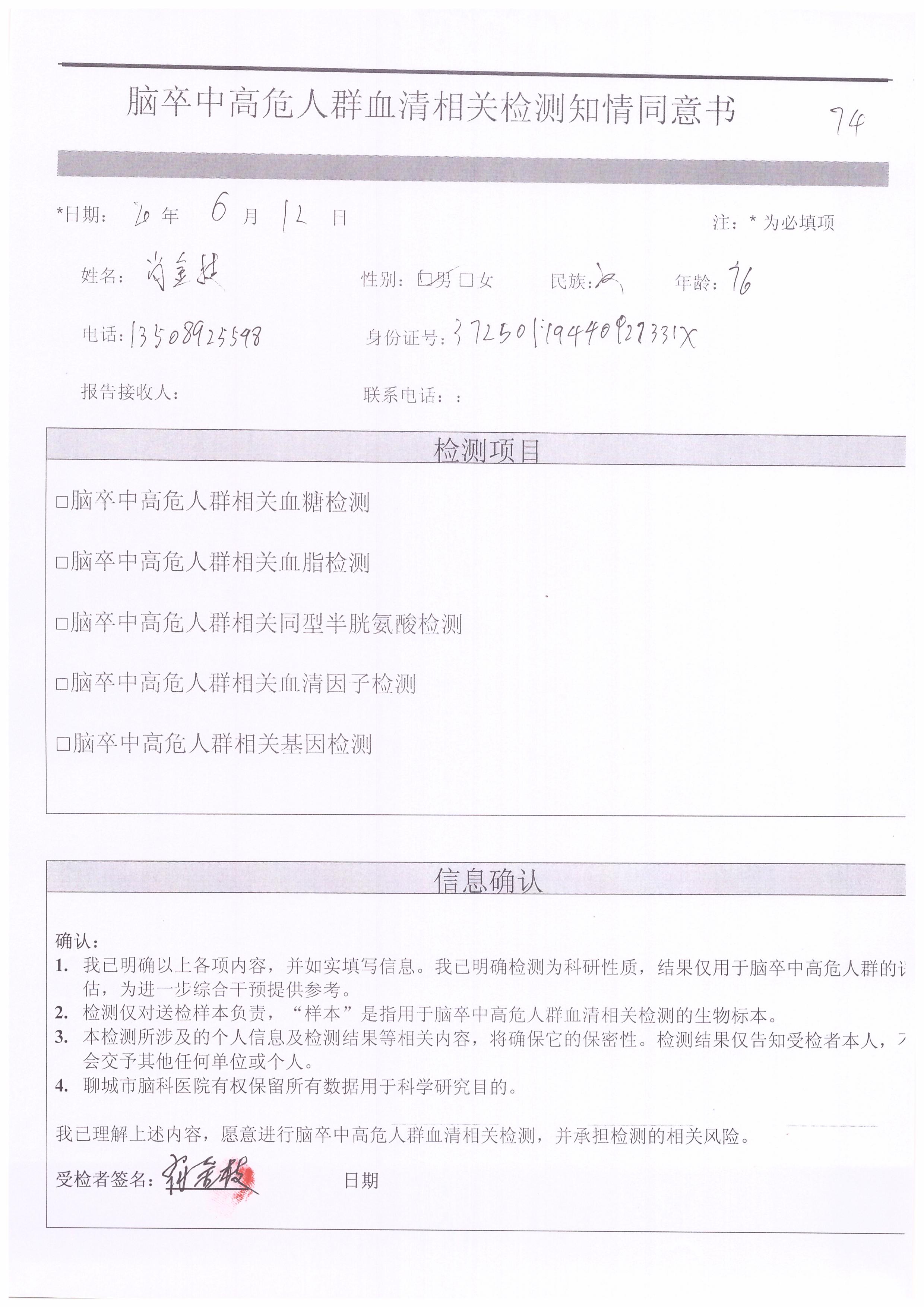

Supplement: Supplementary file 7 — Supplementary file7 (ZIP 27016 KB) [file 10528_2023_10431_MOESM7_ESM.zip › ╓¬╟Θ═1⁄4╥Γ╩Θ5/028.jpg]

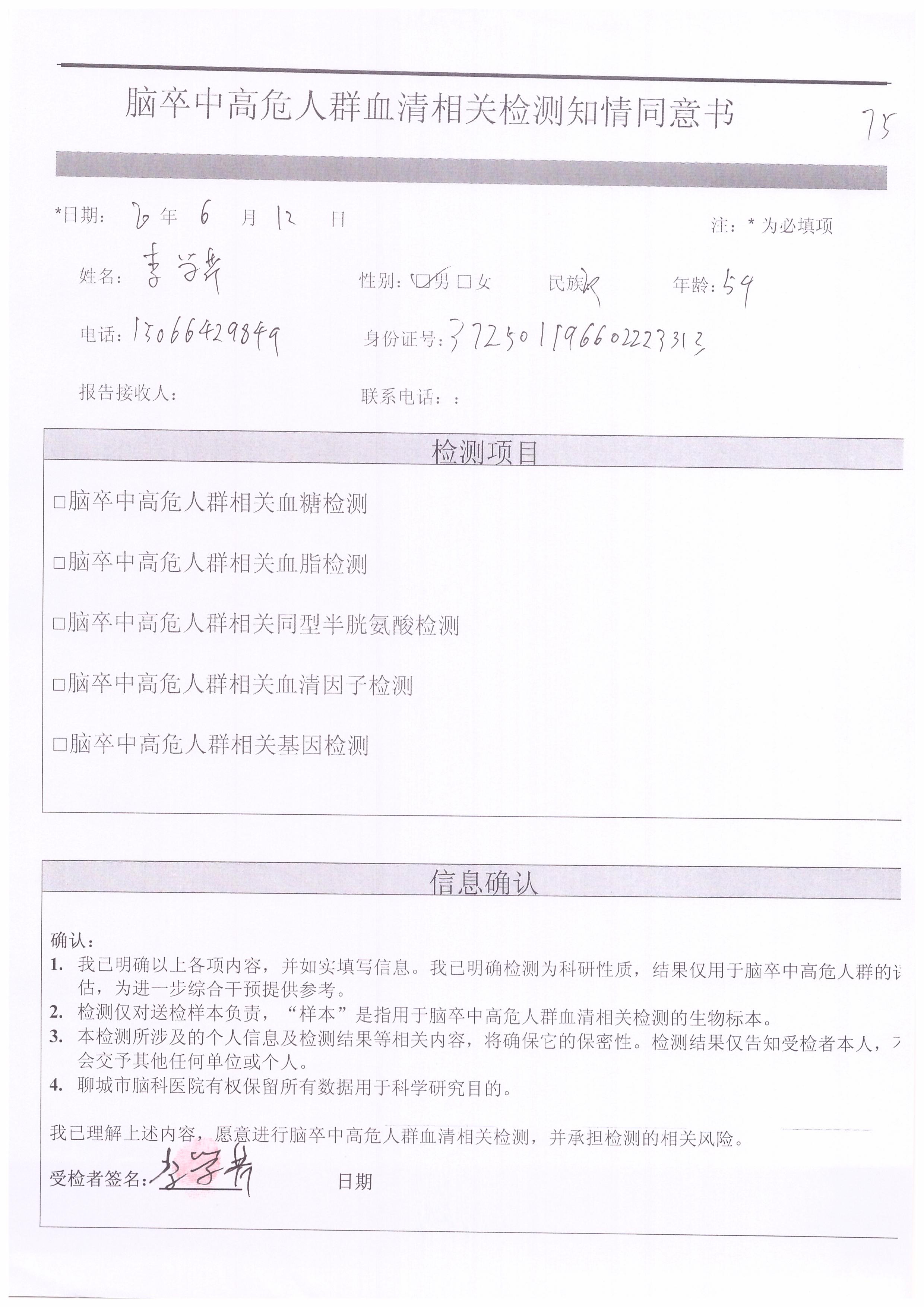

Supplement: Supplementary file 7 — Supplementary file7 (ZIP 27016 KB) [file 10528_2023_10431_MOESM7_ESM.zip › ╓¬╟Θ═1⁄4╥Γ╩Θ5/029.jpg]

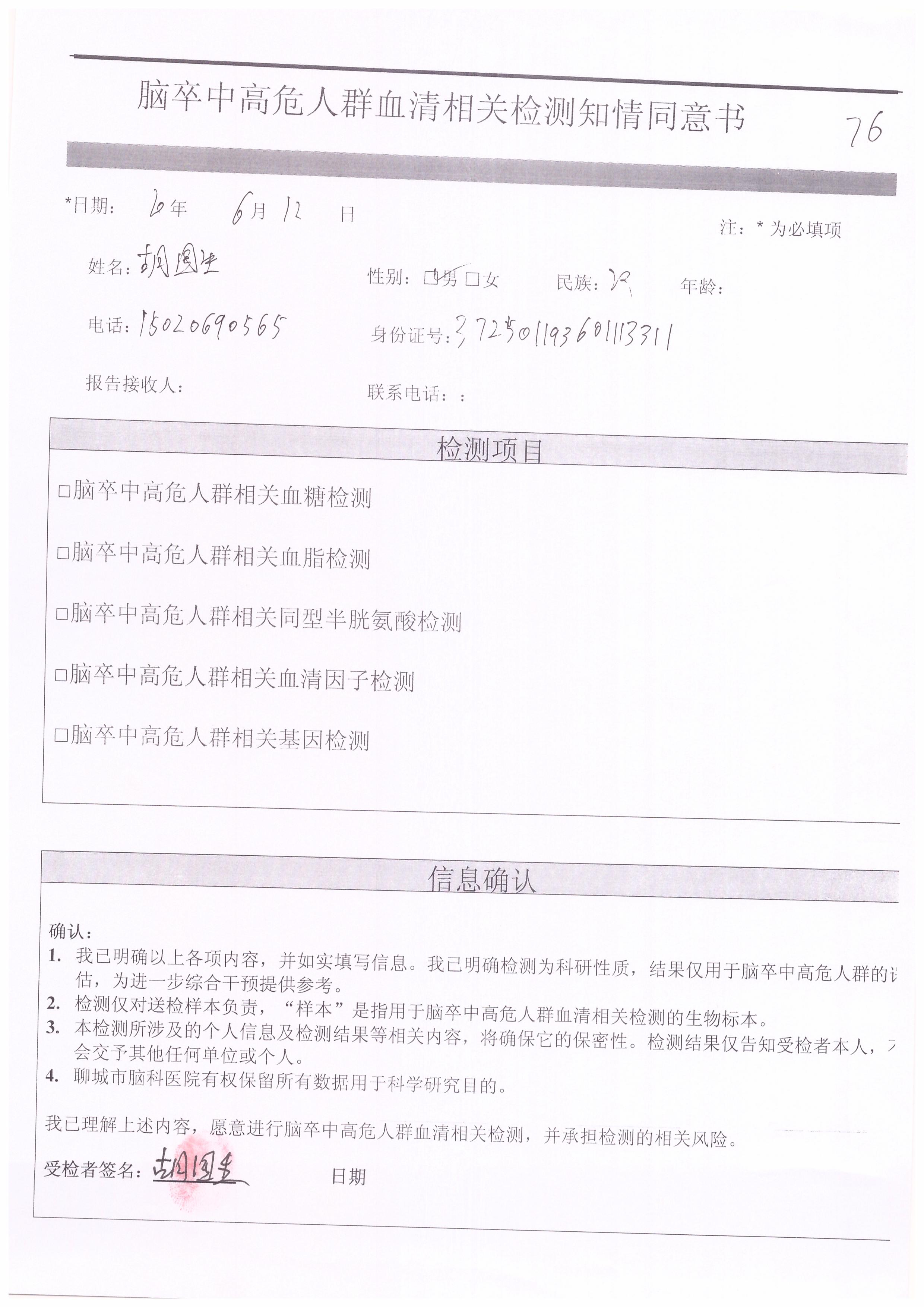

Supplement: Supplementary file 7 — Supplementary file7 (ZIP 27016 KB) [file 10528_2023_10431_MOESM7_ESM.zip › ╓¬╟Θ═1⁄4╥Γ╩Θ5/030.jpg]

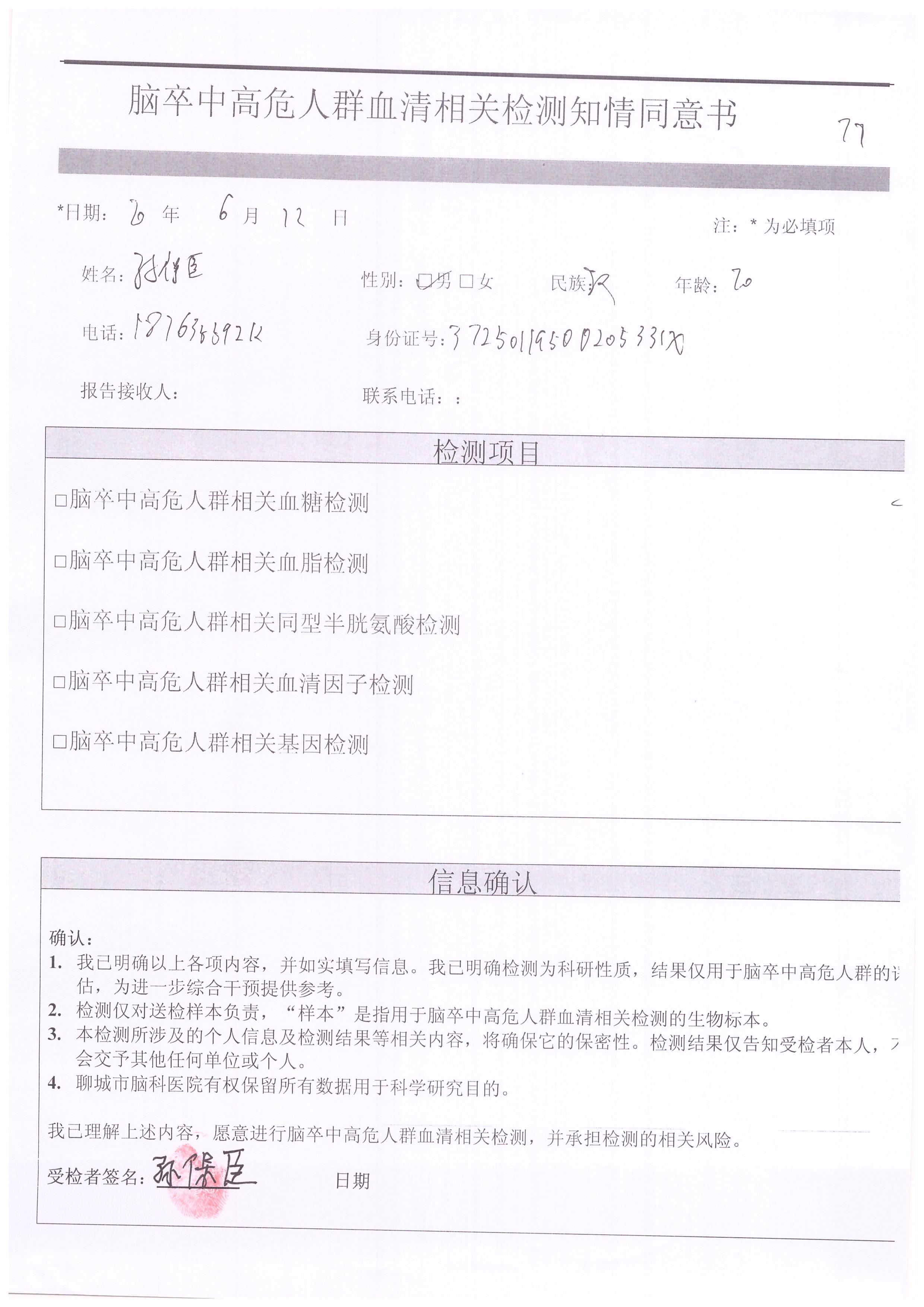

Supplement: Supplementary file 7 — Supplementary file7 (ZIP 27016 KB) [file 10528_2023_10431_MOESM7_ESM.zip › ╓¬╟Θ═1⁄4╥Γ╩Θ5/031.jpg]

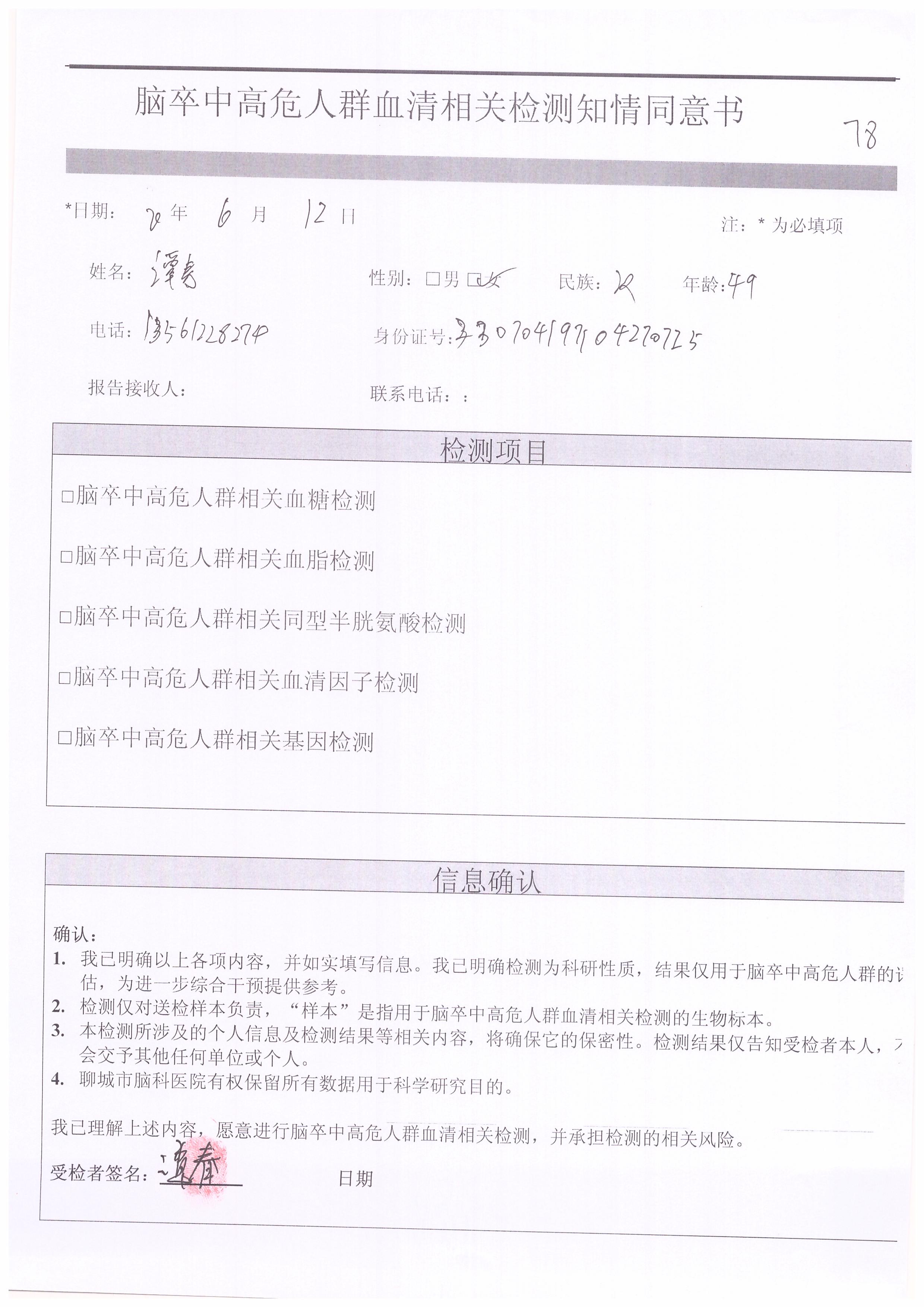

Supplement: Supplementary file 7 — Supplementary file7 (ZIP 27016 KB) [file 10528_2023_10431_MOESM7_ESM.zip › ╓¬╟Θ═1⁄4╥Γ╩Θ5/032.jpg]

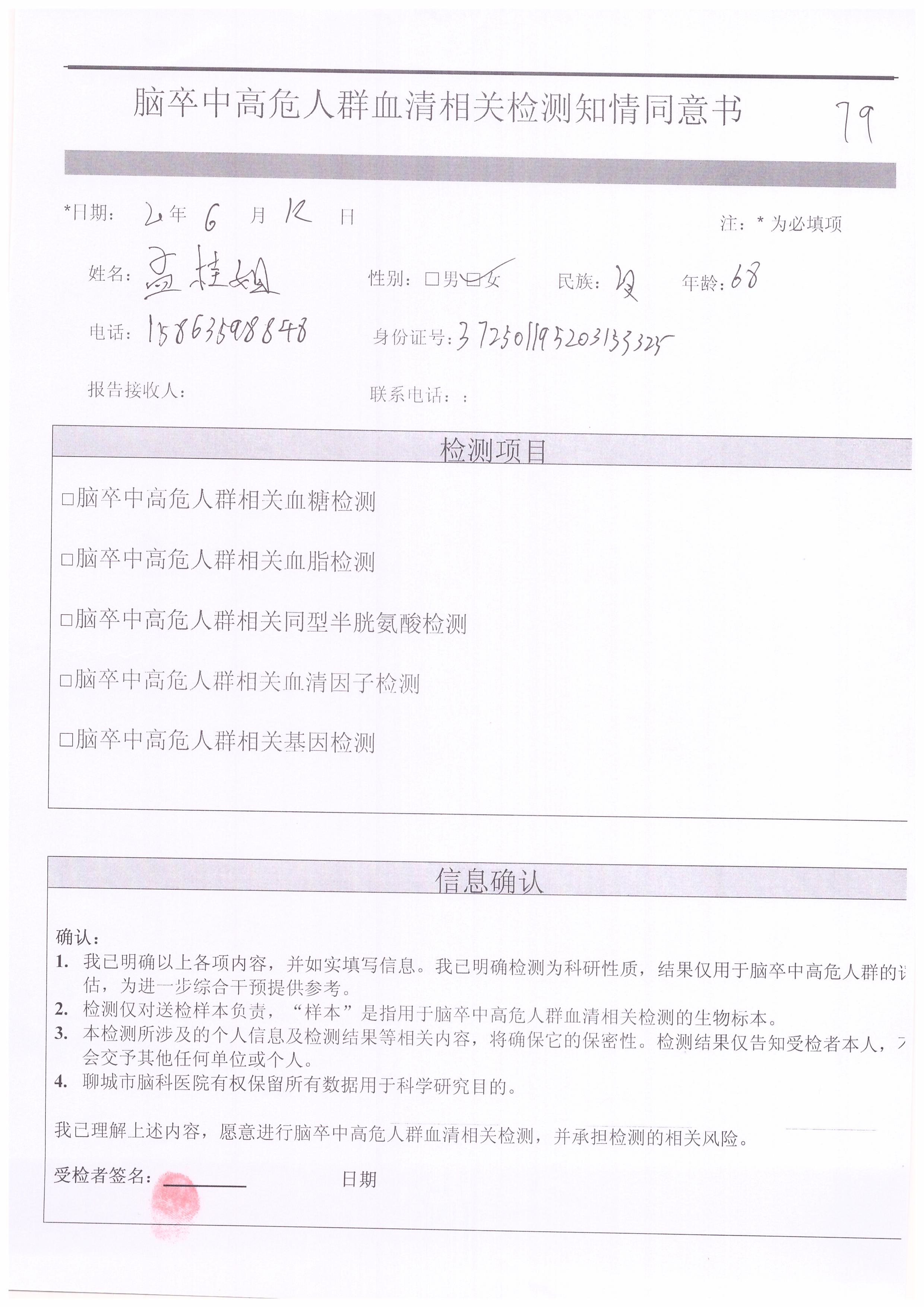

Supplement: Supplementary file 7 — Supplementary file7 (ZIP 27016 KB) [file 10528_2023_10431_MOESM7_ESM.zip › ╓¬╟Θ═1⁄4╥Γ╩Θ5/033.jpg]

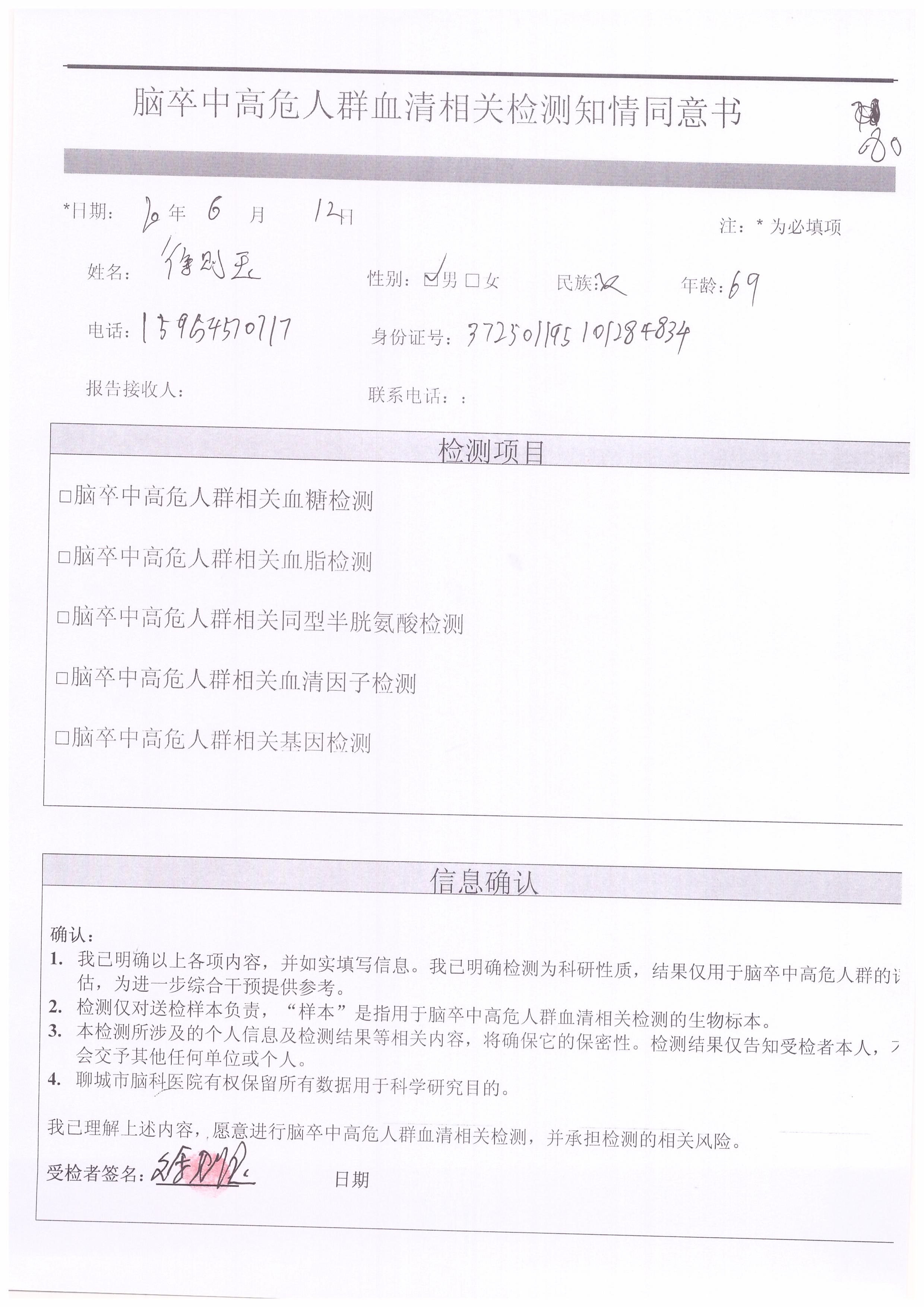

Supplement: Supplementary file 7 — Supplementary file7 (ZIP 27016 KB) [file 10528_2023_10431_MOESM7_ESM.zip › ╓¬╟Θ═1⁄4╥Γ╩Θ5/034.jpg]

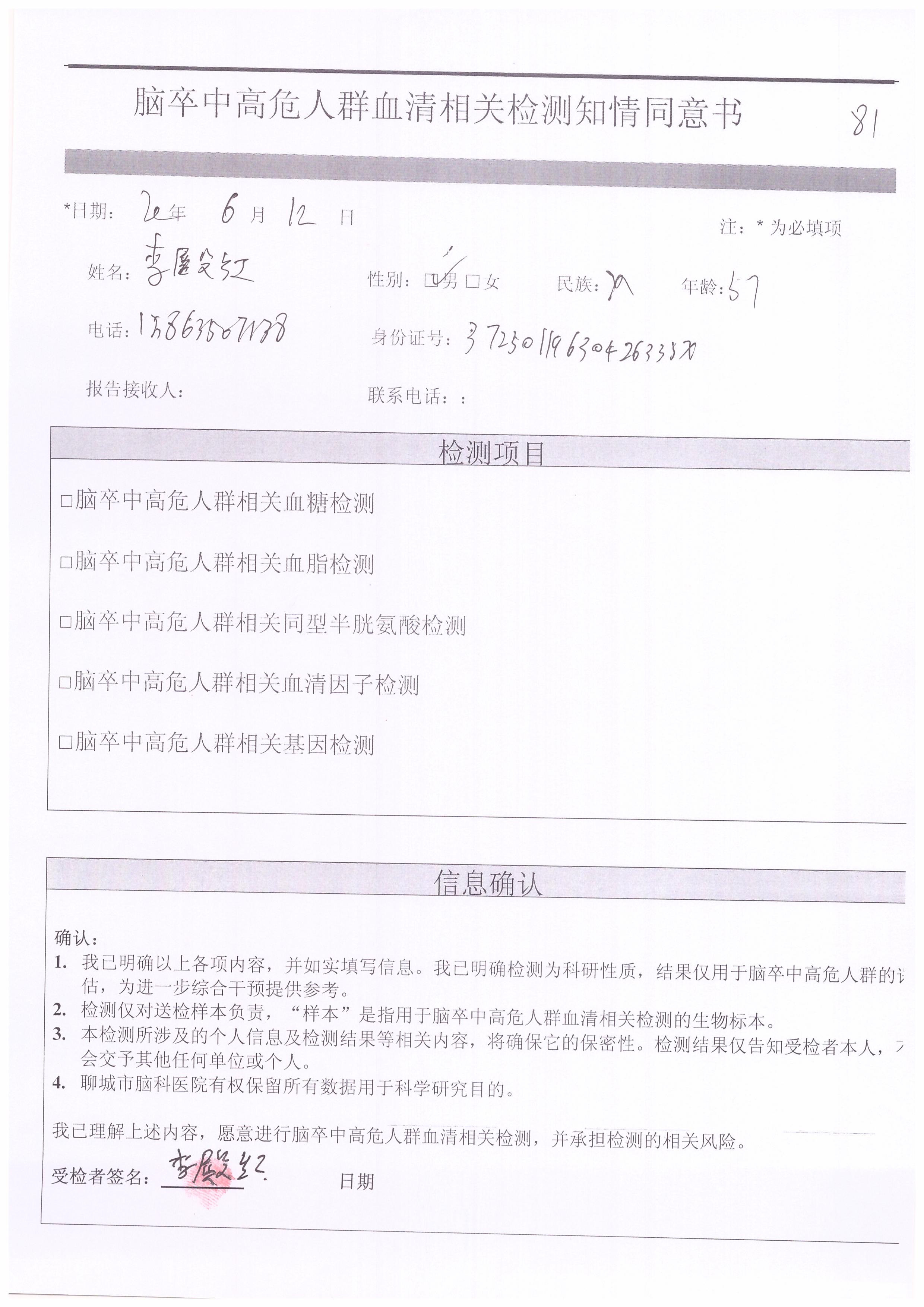

Supplement: Supplementary file 7 — Supplementary file7 (ZIP 27016 KB) [file 10528_2023_10431_MOESM7_ESM.zip › ╓¬╟Θ═1⁄4╥Γ╩Θ5/035.jpg]

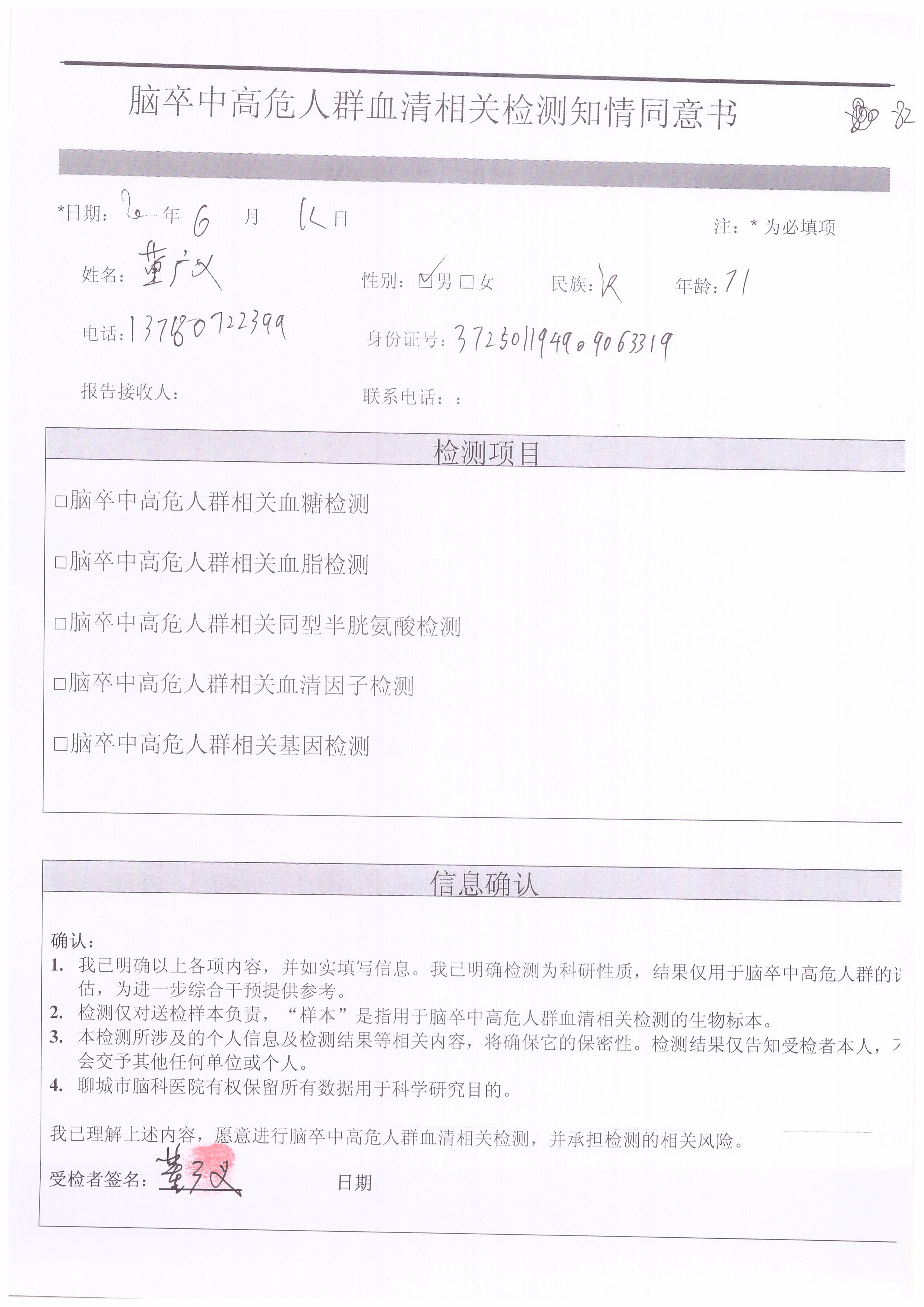

Supplement: Supplementary file 7 — Supplementary file7 (ZIP 27016 KB) [file 10528_2023_10431_MOESM7_ESM.zip › ╓¬╟Θ═1⁄4╥Γ╩Θ5/036.jpg]

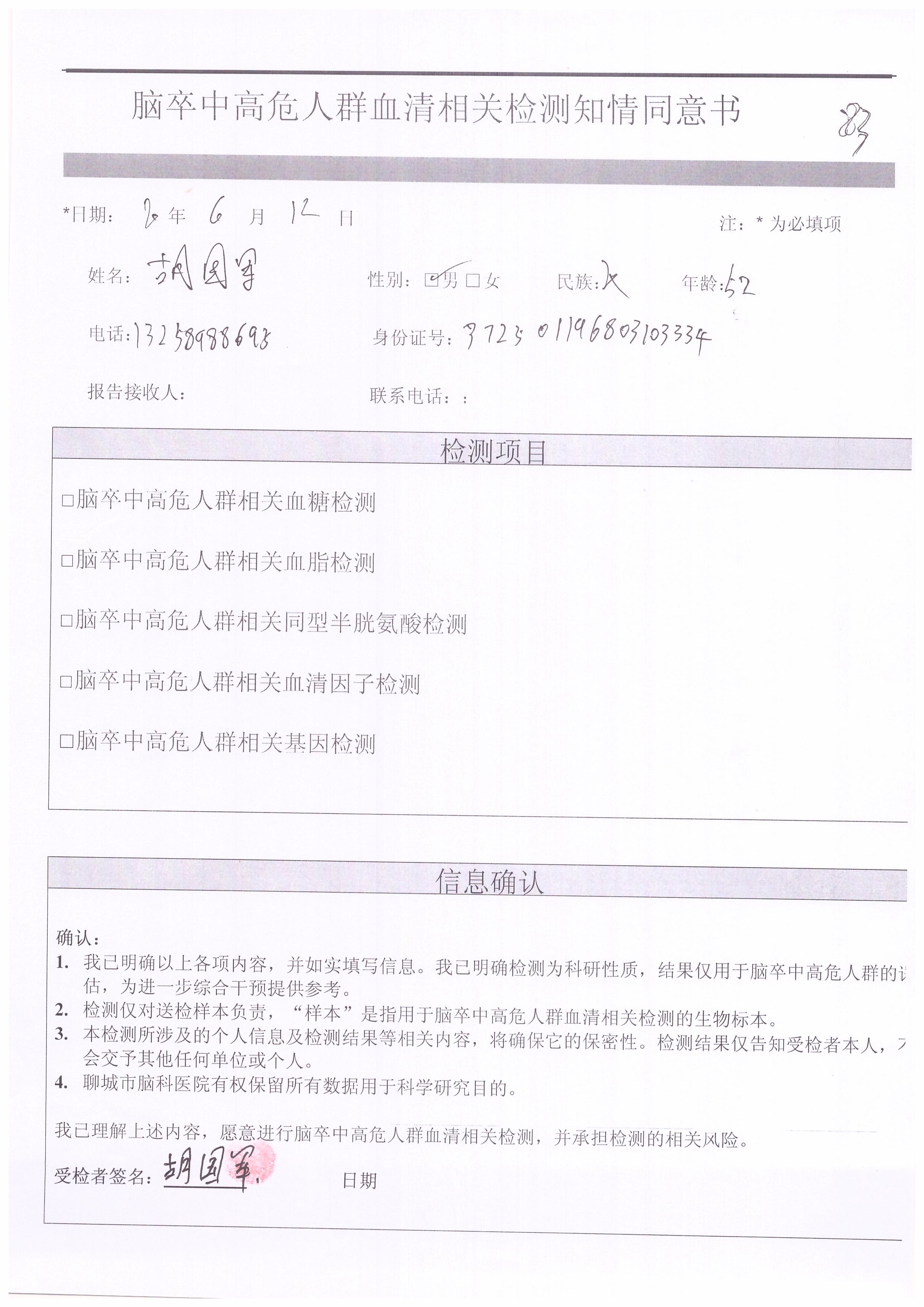

Supplement: Supplementary file 7 — Supplementary file7 (ZIP 27016 KB) [file 10528_2023_10431_MOESM7_ESM.zip › ╓¬╟Θ═1⁄4╥Γ╩Θ5/037.jpg]

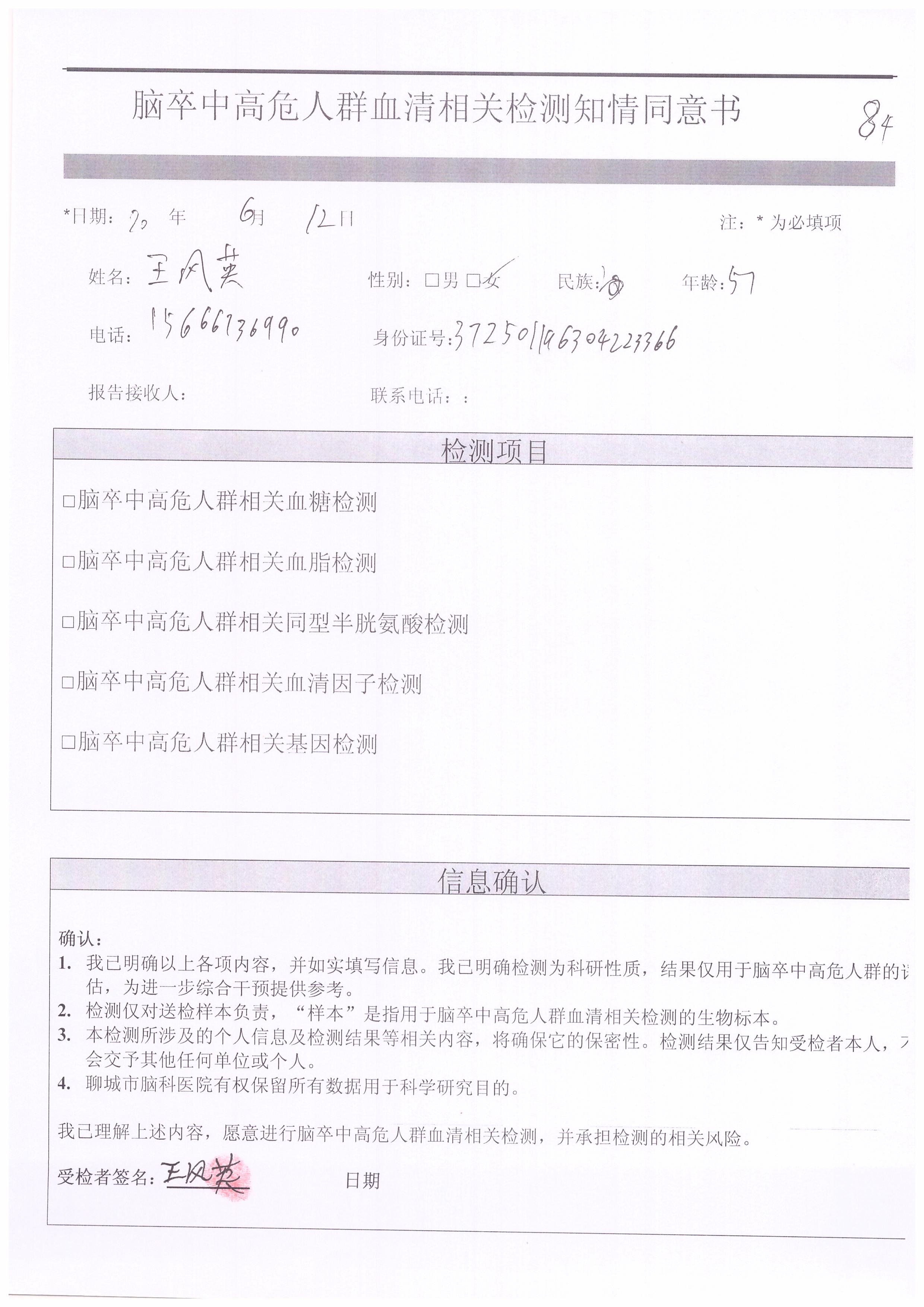

Supplement: Supplementary file 7 — Supplementary file7 (ZIP 27016 KB) [file 10528_2023_10431_MOESM7_ESM.zip › ╓¬╟Θ═1⁄4╥Γ╩Θ5/038.jpg]

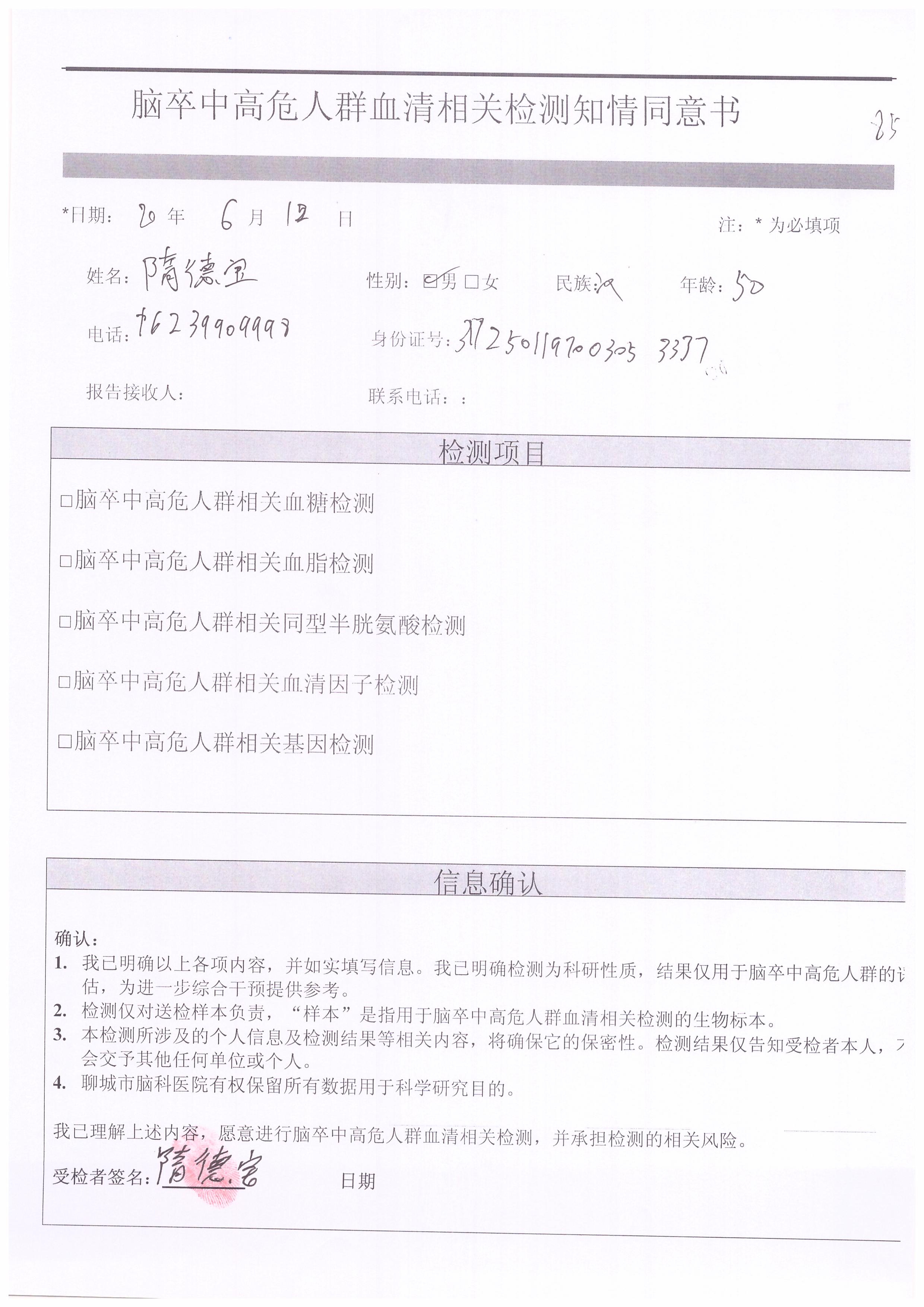

Supplement: Supplementary file 7 — Supplementary file7 (ZIP 27016 KB) [file 10528_2023_10431_MOESM7_ESM.zip › ╓¬╟Θ═1⁄4╥Γ╩Θ5/039.jpg]

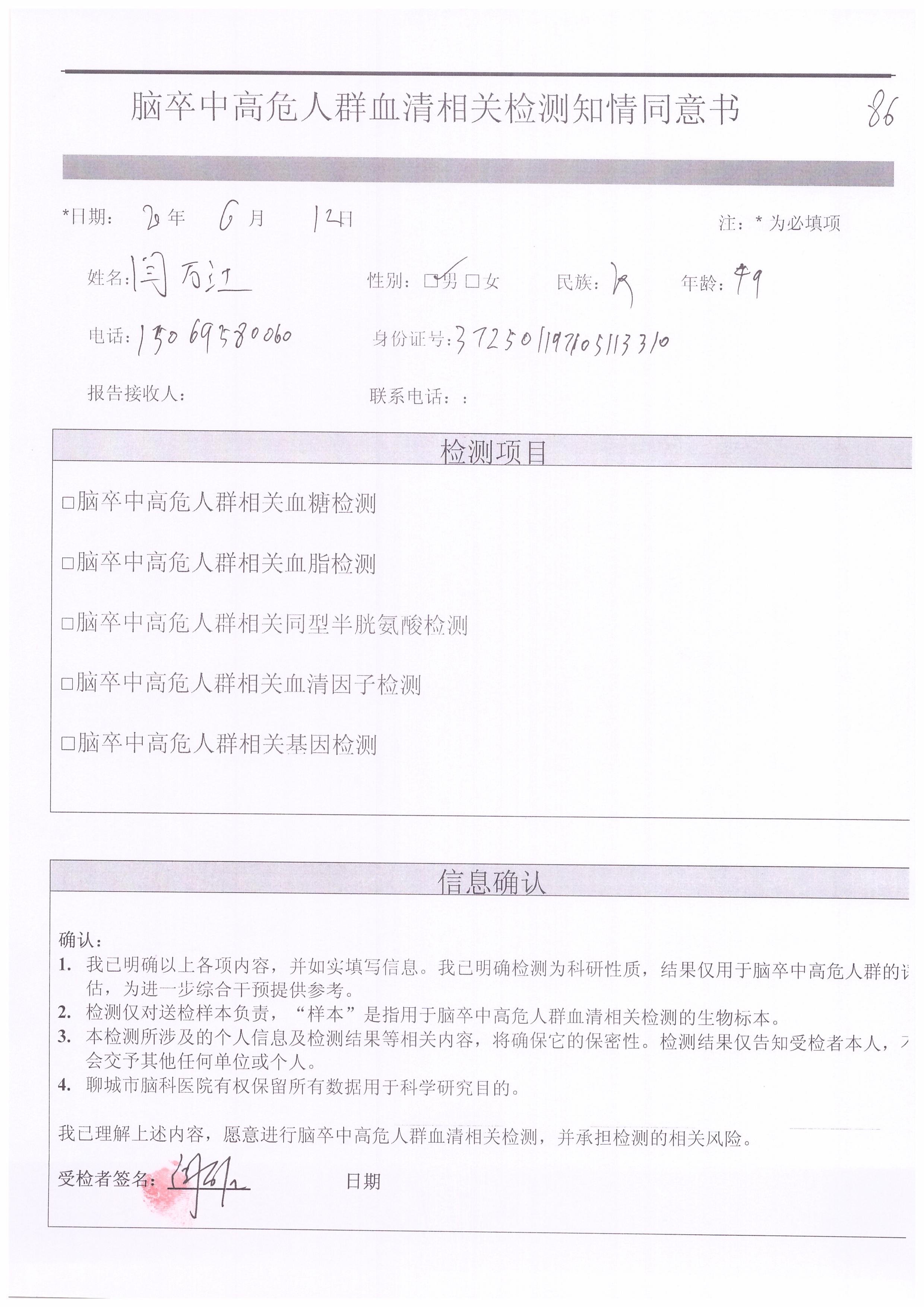

Supplement: Supplementary file 7 — Supplementary file7 (ZIP 27016 KB) [file 10528_2023_10431_MOESM7_ESM.zip › ╓¬╟Θ═1⁄4╥Γ╩Θ5/040.jpg]

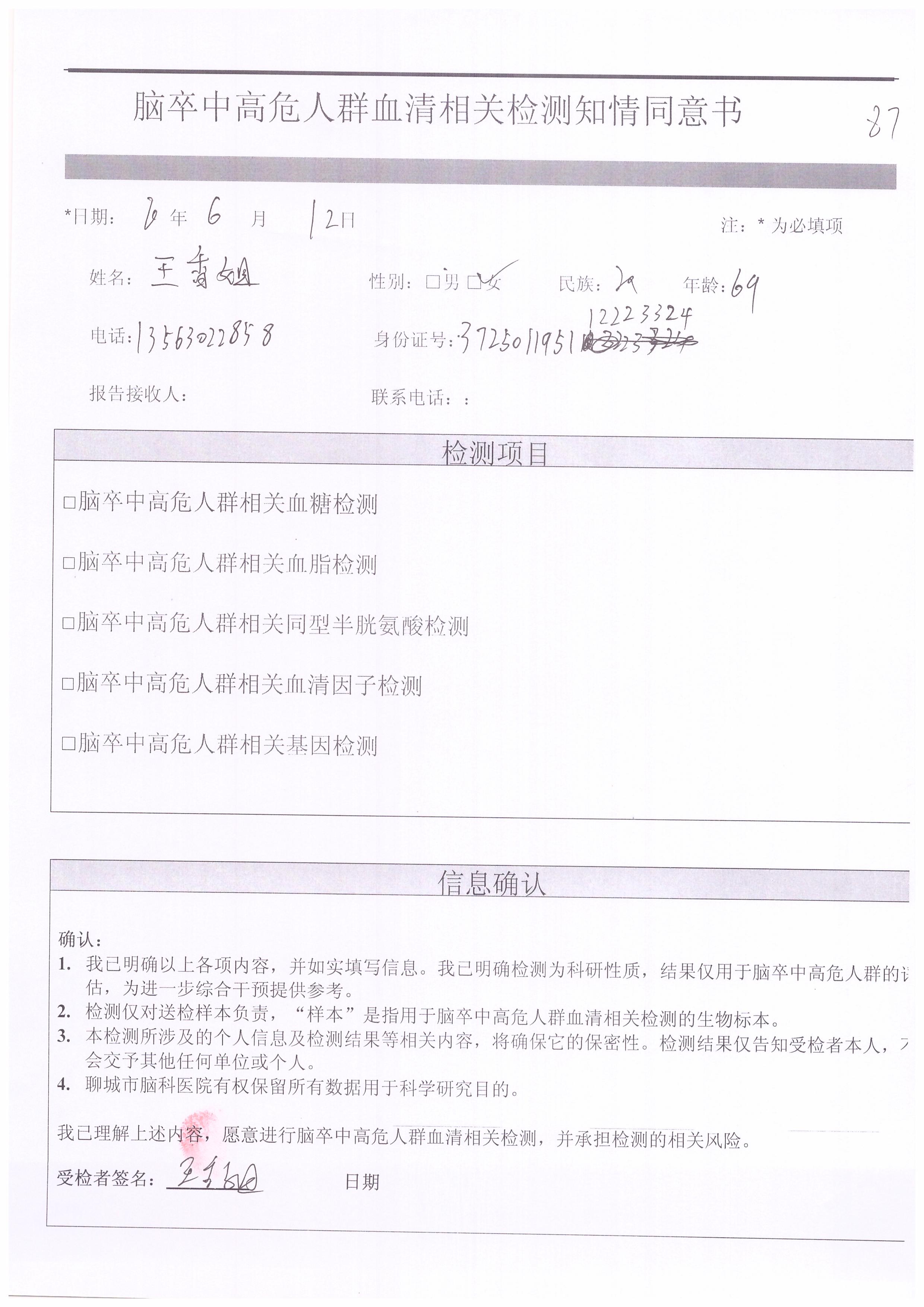

Supplement: Supplementary file 7 — Supplementary file7 (ZIP 27016 KB) [file 10528_2023_10431_MOESM7_ESM.zip › ╓¬╟Θ═1⁄4╥Γ╩Θ5/041.jpg]

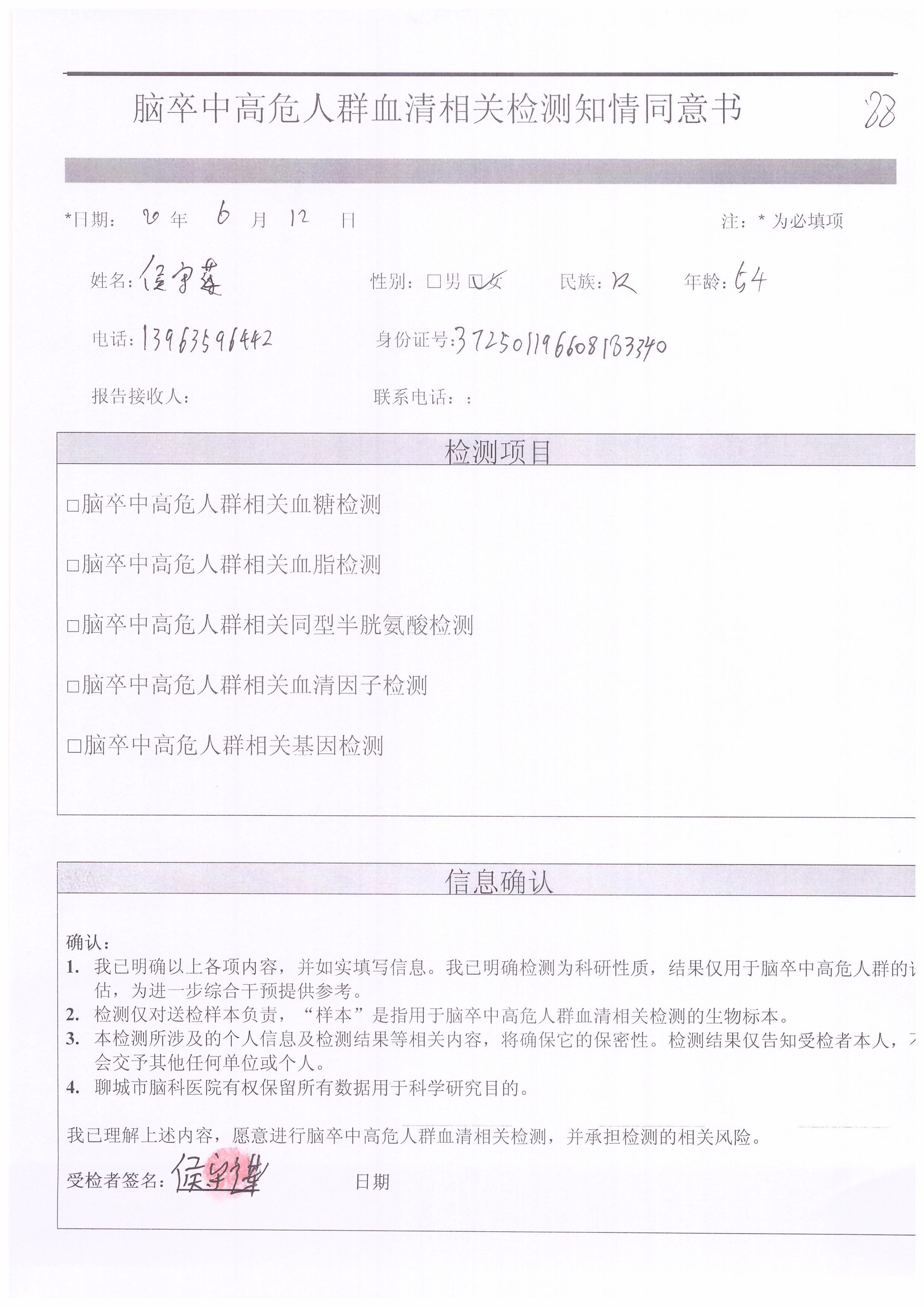

Supplement: Supplementary file 7 — Supplementary file7 (ZIP 27016 KB) [file 10528_2023_10431_MOESM7_ESM.zip › ╓¬╟Θ═1⁄4╥Γ╩Θ5/042.jpg]

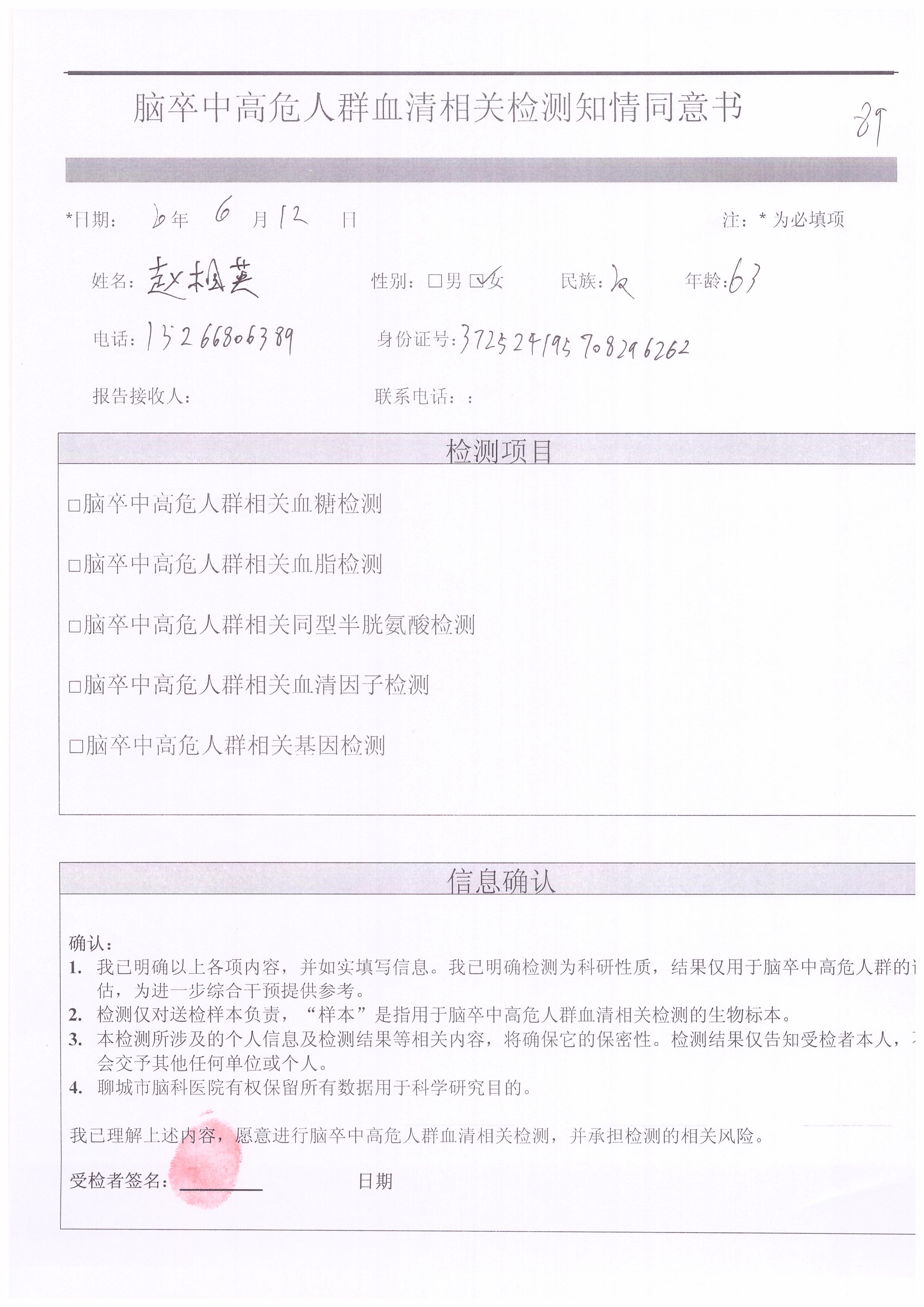

Supplement: Supplementary file 7 — Supplementary file7 (ZIP 27016 KB) [file 10528_2023_10431_MOESM7_ESM.zip › ╓¬╟Θ═1⁄4╥Γ╩Θ5/043.jpg]

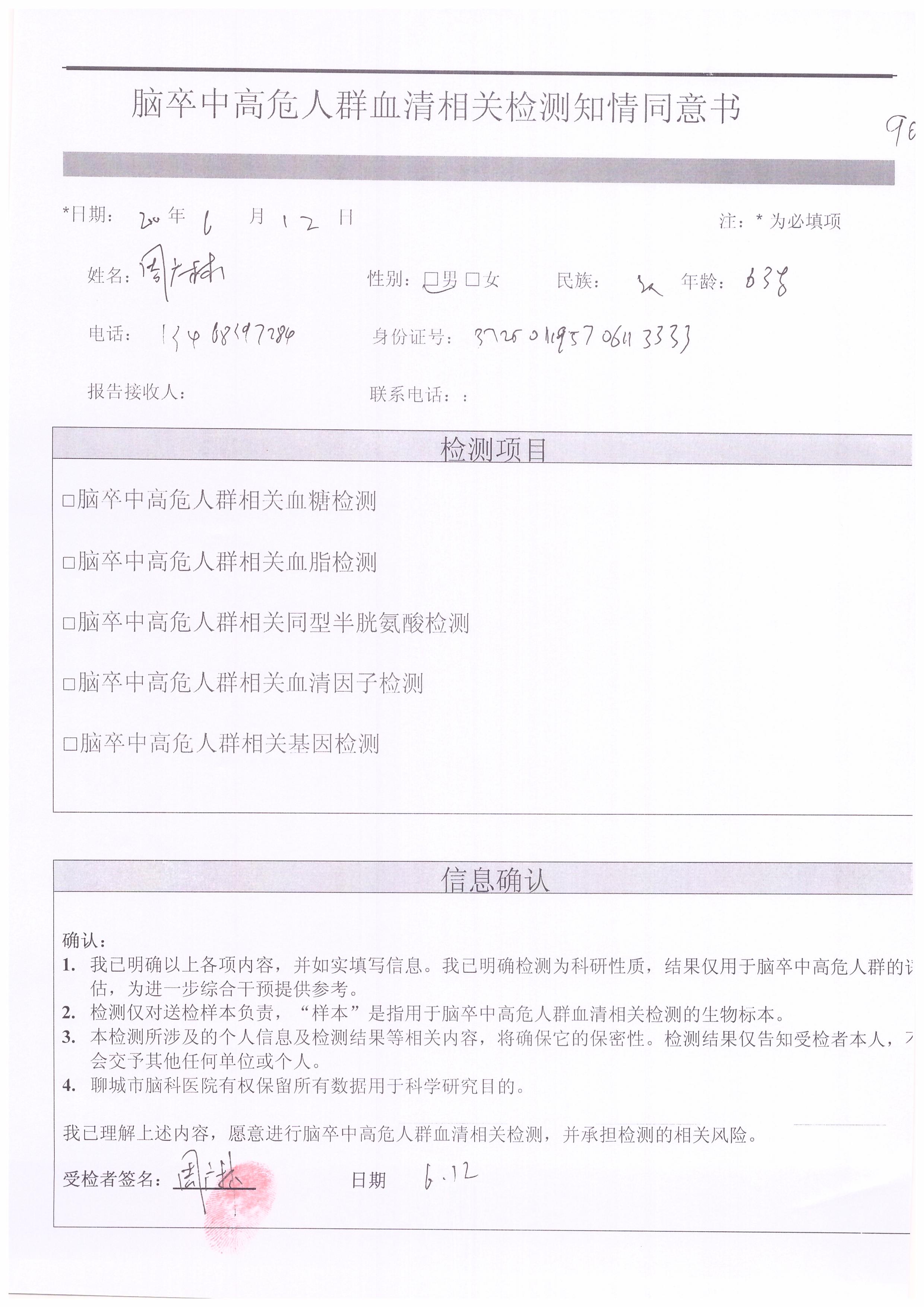

Supplement: Supplementary file 7 — Supplementary file7 (ZIP 27016 KB) [file 10528_2023_10431_MOESM7_ESM.zip › ╓¬╟Θ═1⁄4╥Γ╩Θ5/044.jpg]

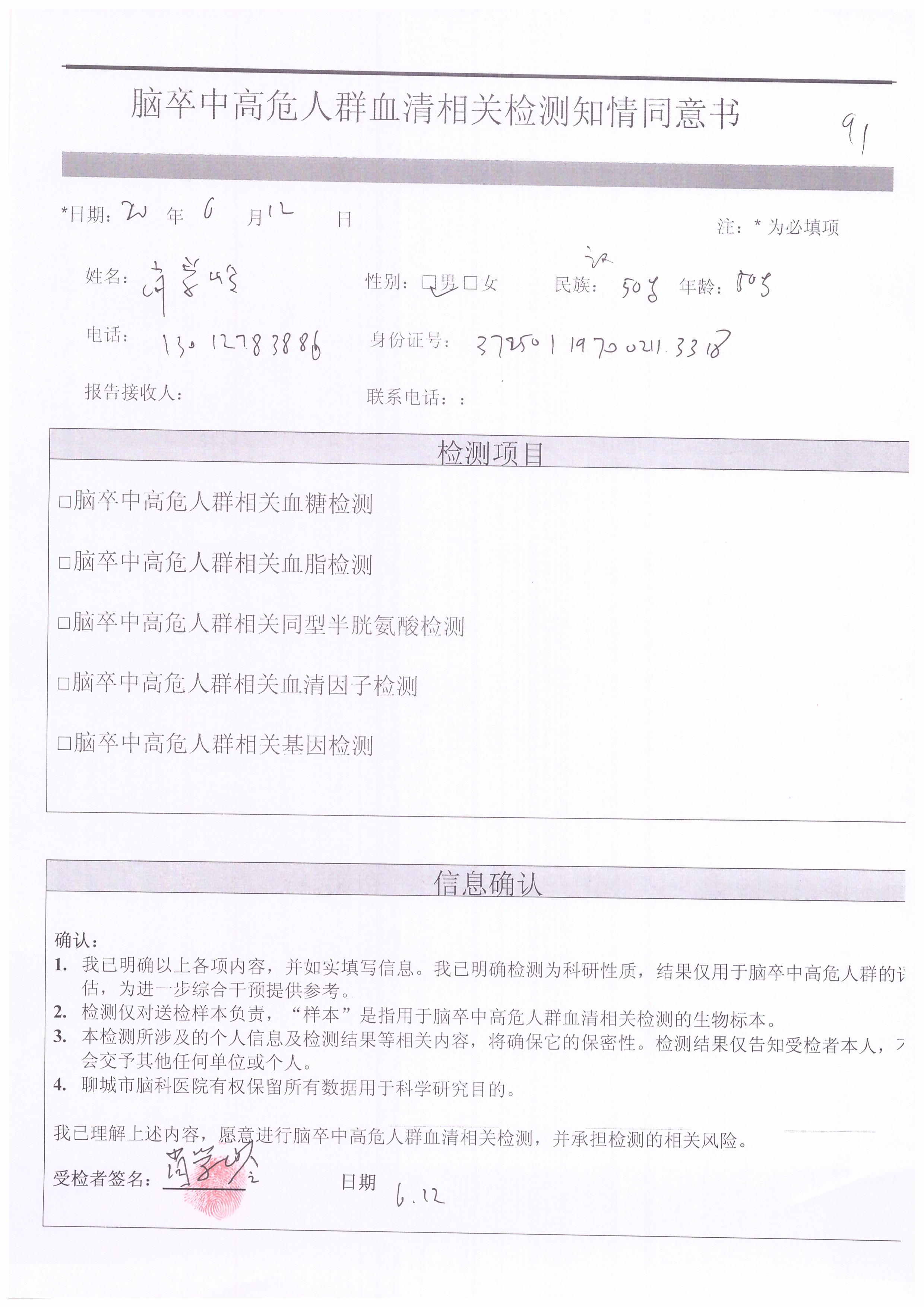

Supplement: Supplementary file 7 — Supplementary file7 (ZIP 27016 KB) [file 10528_2023_10431_MOESM7_ESM.zip › ╓¬╟Θ═1⁄4╥Γ╩Θ5/045.jpg]

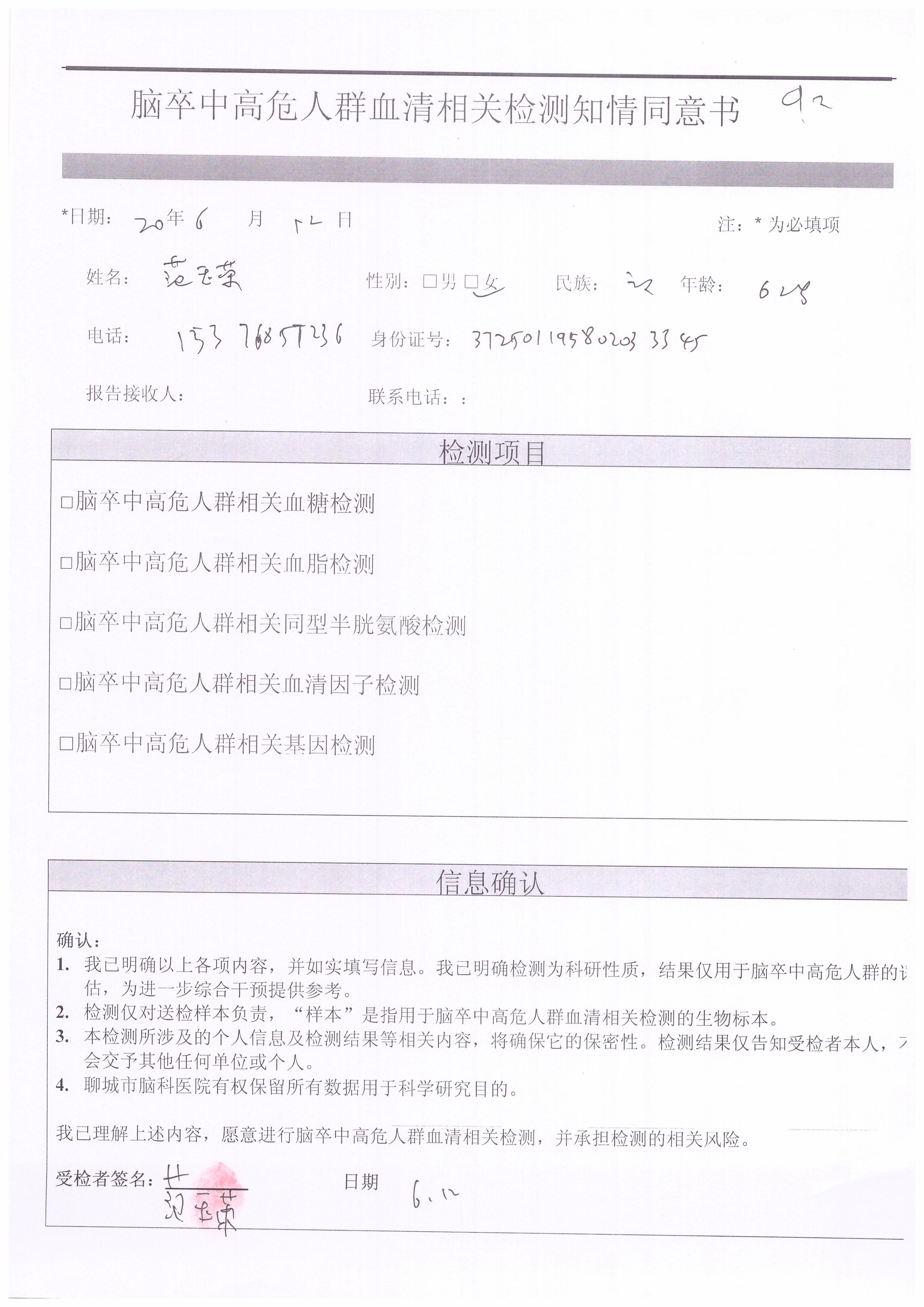

Supplement: Supplementary file 7 — Supplementary file7 (ZIP 27016 KB) [file 10528_2023_10431_MOESM7_ESM.zip › ╓¬╟Θ═1⁄4╥Γ╩Θ5/046.jpg]

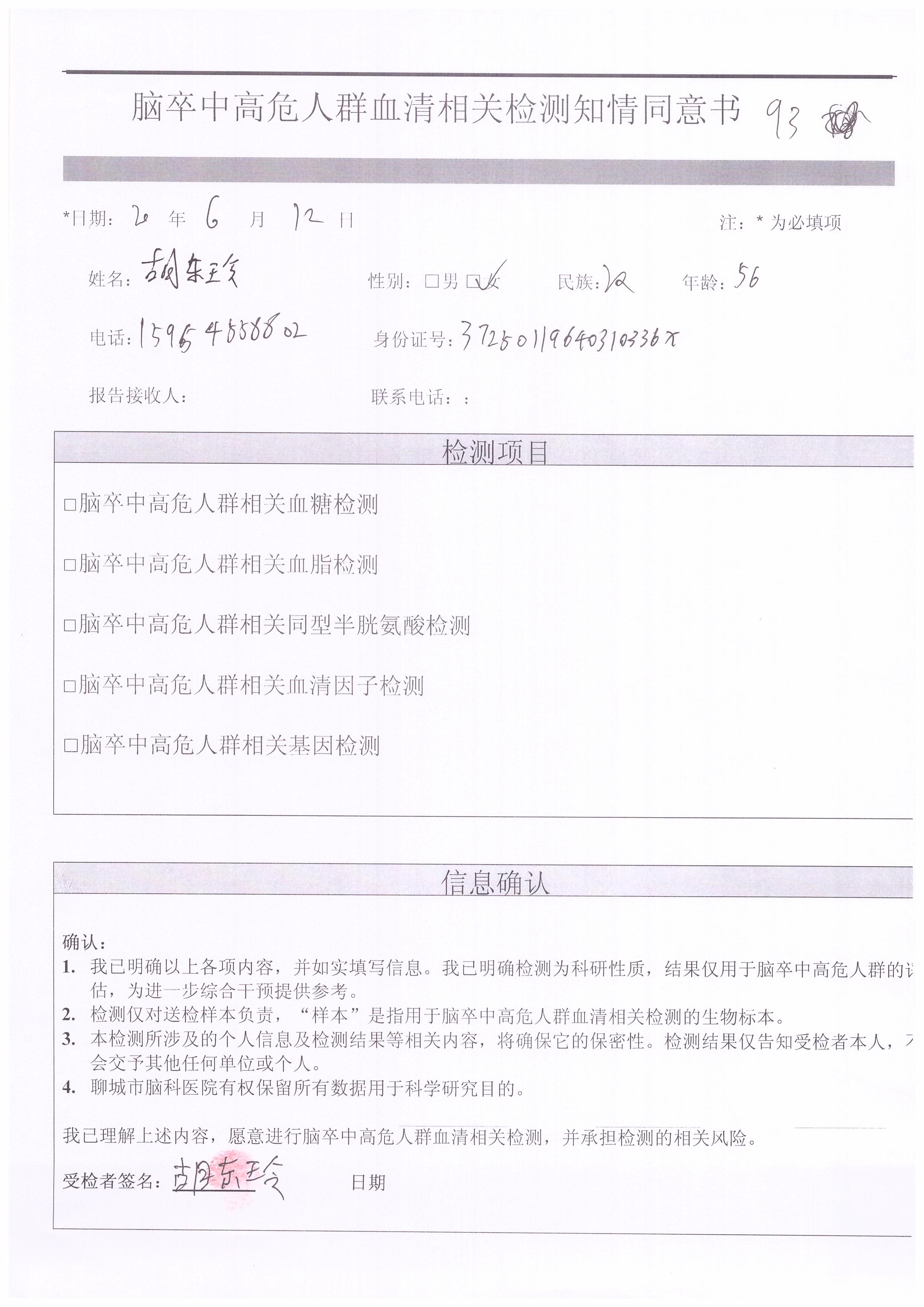

Supplement: Supplementary file 7 — Supplementary file7 (ZIP 27016 KB) [file 10528_2023_10431_MOESM7_ESM.zip › ╓¬╟Θ═1⁄4╥Γ╩Θ5/047.jpg]

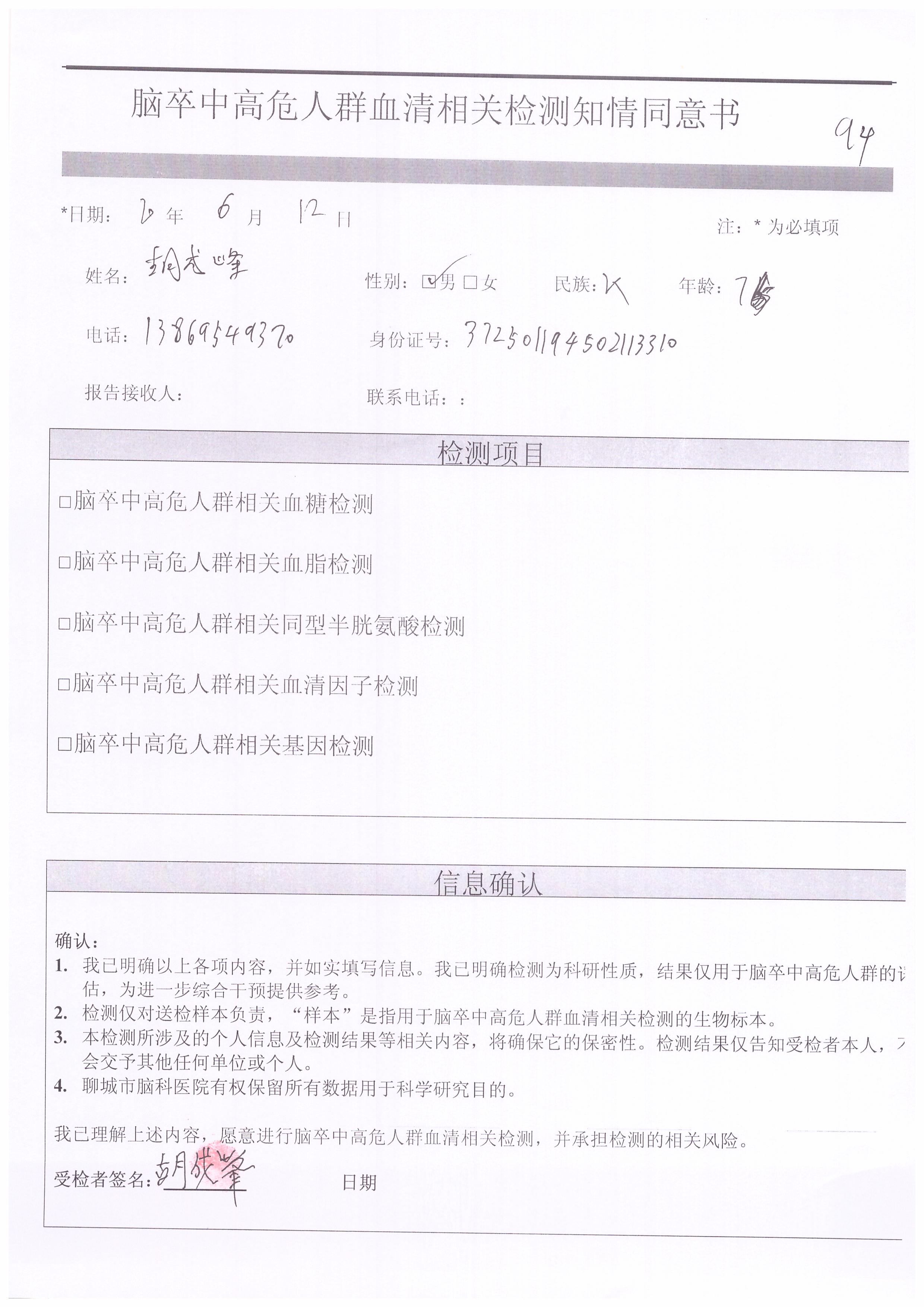

Supplement: Supplementary file 7 — Supplementary file7 (ZIP 27016 KB) [file 10528_2023_10431_MOESM7_ESM.zip › ╓¬╟Θ═1⁄4╥Γ╩Θ5/048.jpg]

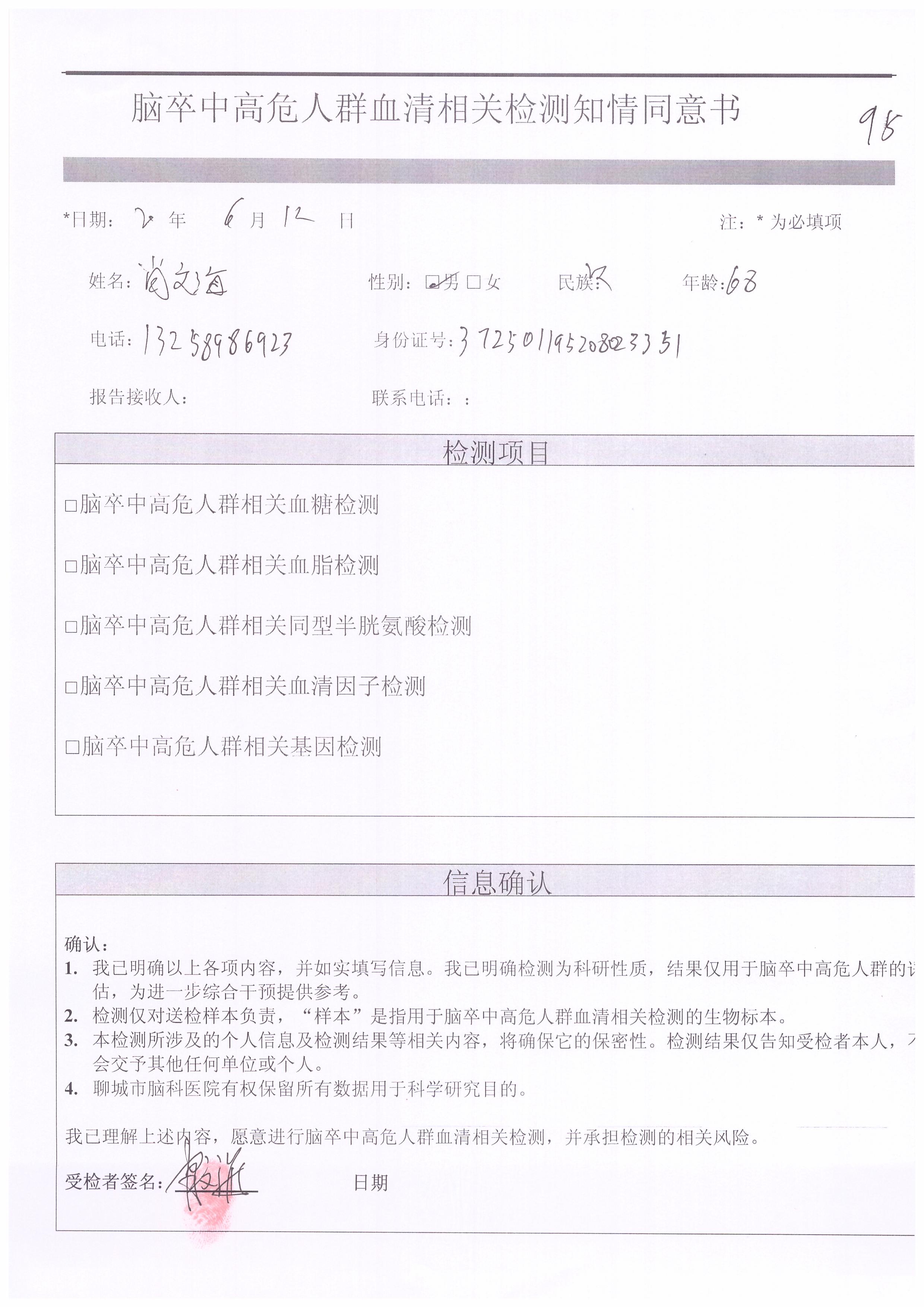

Supplement: Supplementary file 7 — Supplementary file7 (ZIP 27016 KB) [file 10528_2023_10431_MOESM7_ESM.zip › ╓¬╟Θ═1⁄4╥Γ╩Θ5/049.jpg]

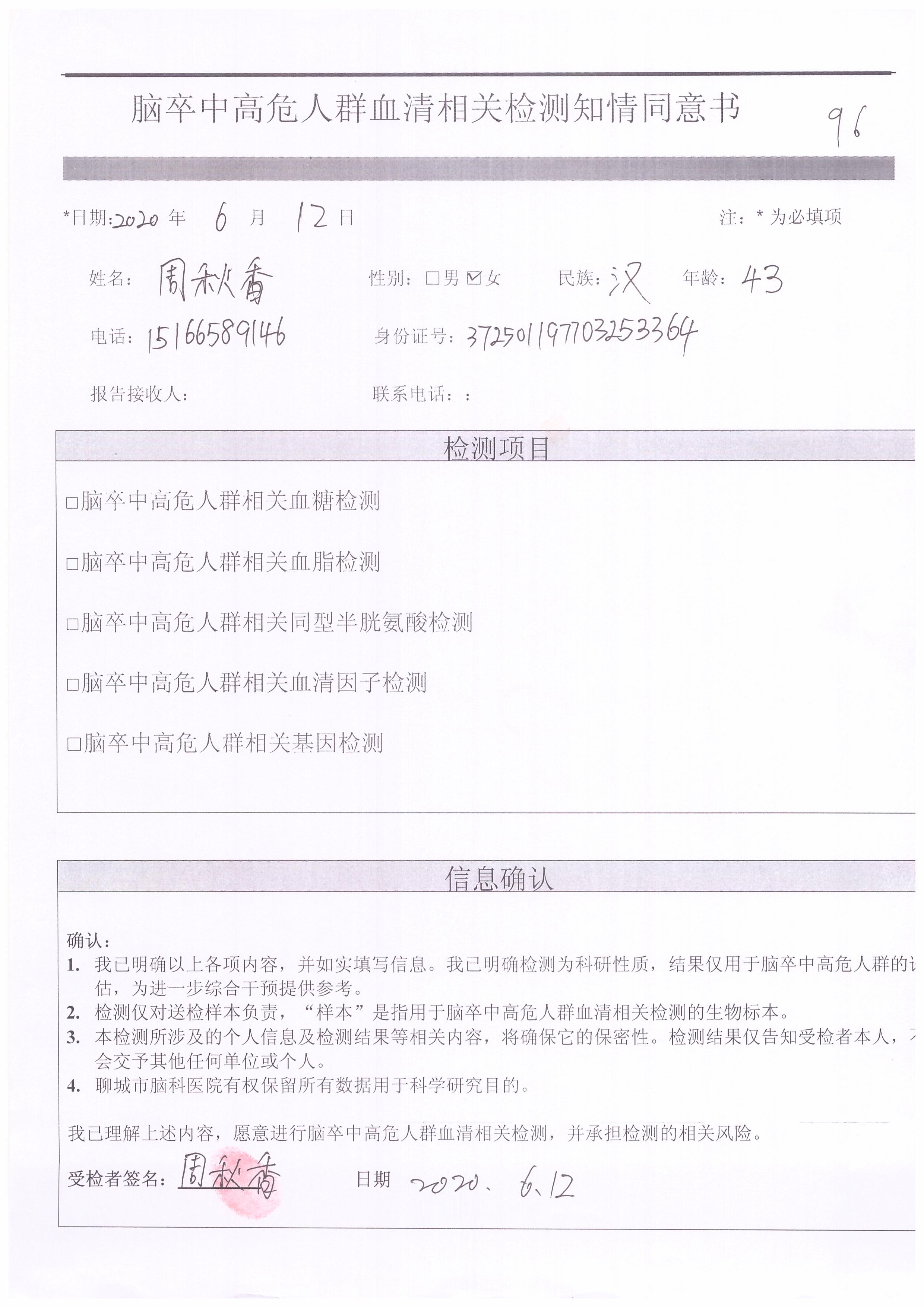

Supplement: Supplementary file 7 — Supplementary file7 (ZIP 27016 KB) [file 10528_2023_10431_MOESM7_ESM.zip › ╓¬╟Θ═1⁄4╥Γ╩Θ5/050.jpg]

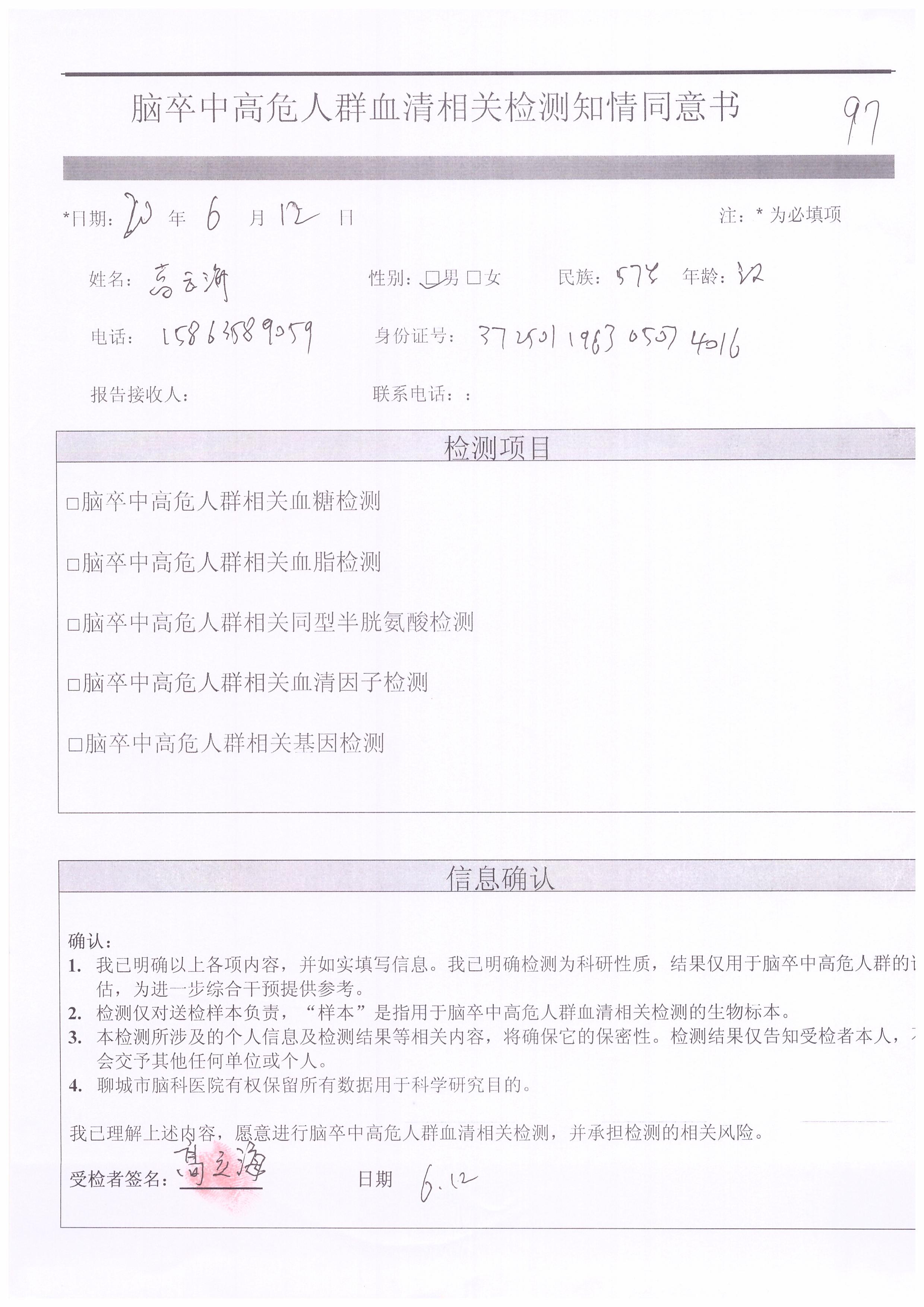

Supplement: Supplementary file 7 — Supplementary file7 (ZIP 27016 KB) [file 10528_2023_10431_MOESM7_ESM.zip › ╓¬╟Θ═1⁄4╥Γ╩Θ5/051.jpg]

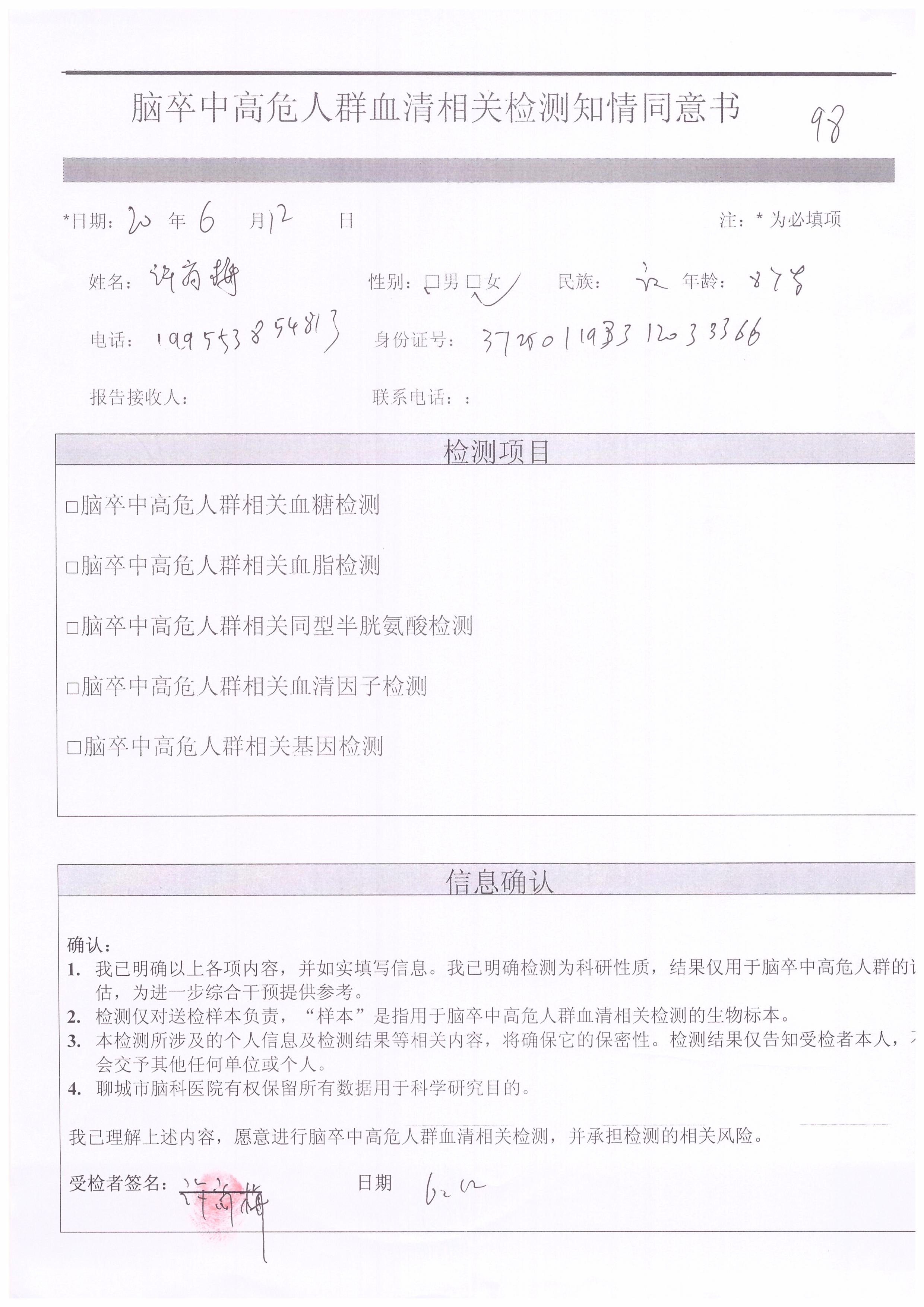

Supplement: Supplementary file 7 — Supplementary file7 (ZIP 27016 KB) [file 10528_2023_10431_MOESM7_ESM.zip › ╓¬╟Θ═1⁄4╥Γ╩Θ5/052.jpg]

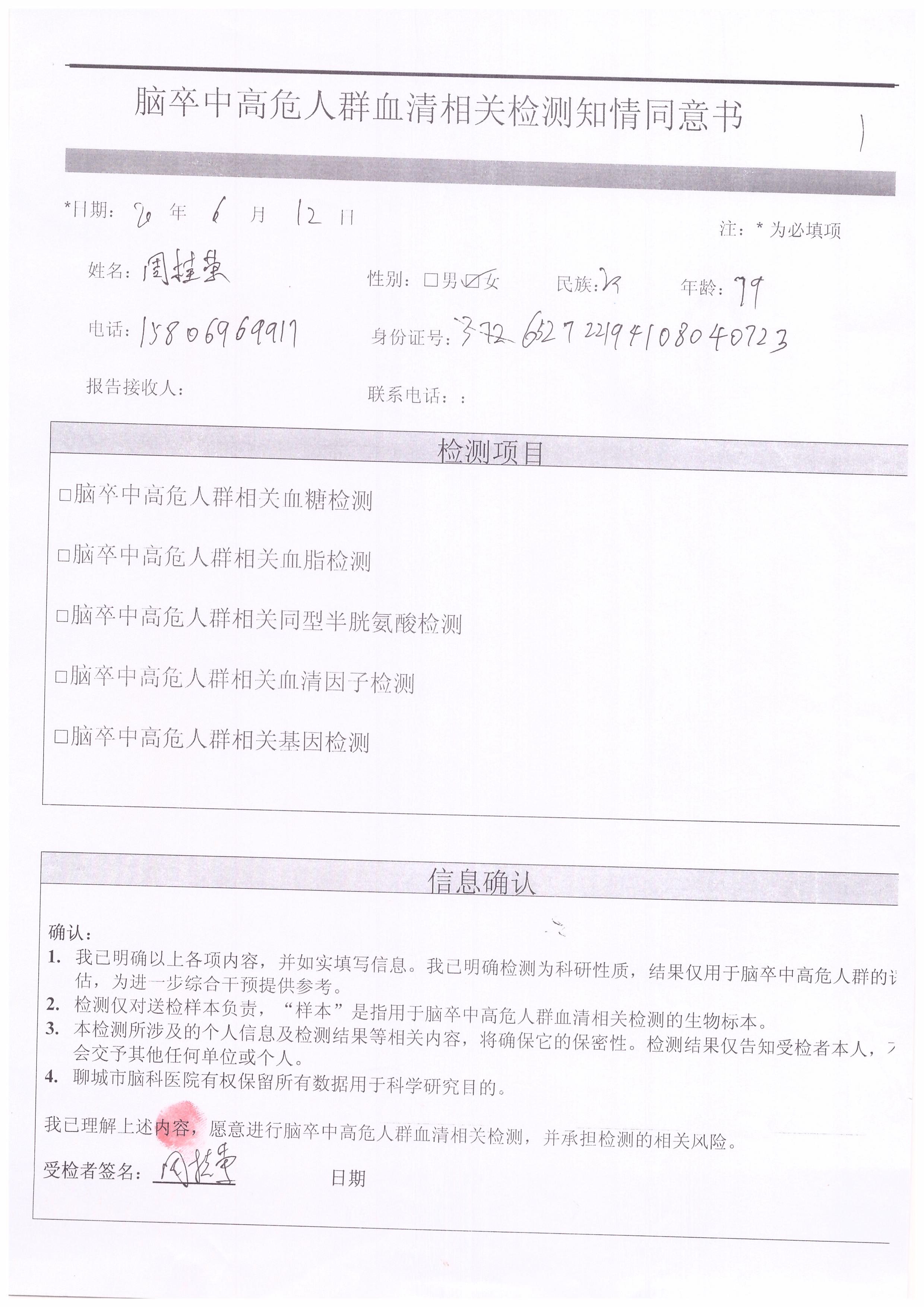

Supplement: Supplementary file 8 — Supplementary file8 (ZIP 23226 KB) [file 10528_2023_10431_MOESM8_ESM.zip › ╓¬╟Θ═1⁄4╥Γ╩Θ6/001.jpg]

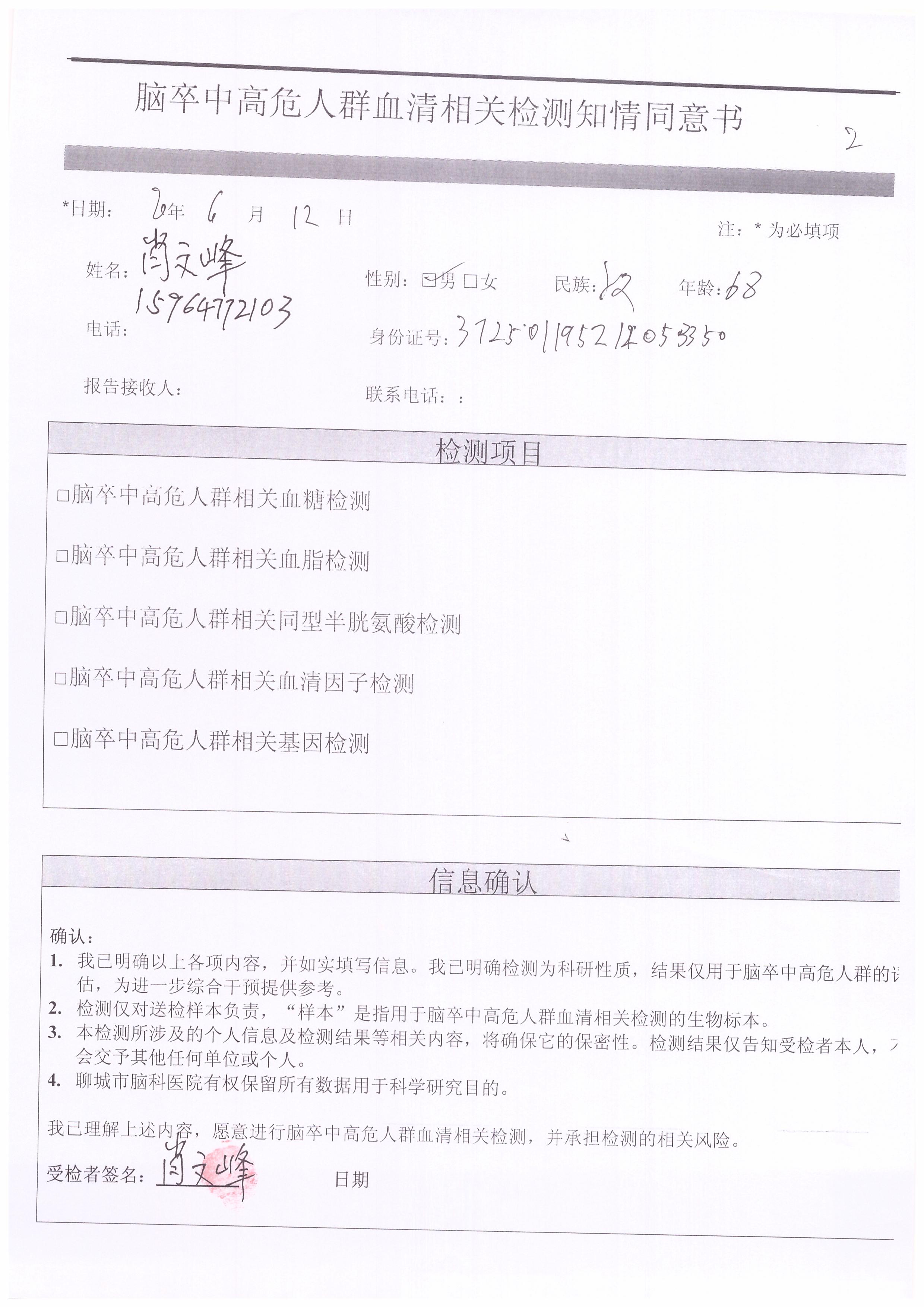

Supplement: Supplementary file 8 — Supplementary file8 (ZIP 23226 KB) [file 10528_2023_10431_MOESM8_ESM.zip › ╓¬╟Θ═1⁄4╥Γ╩Θ6/002.jpg]

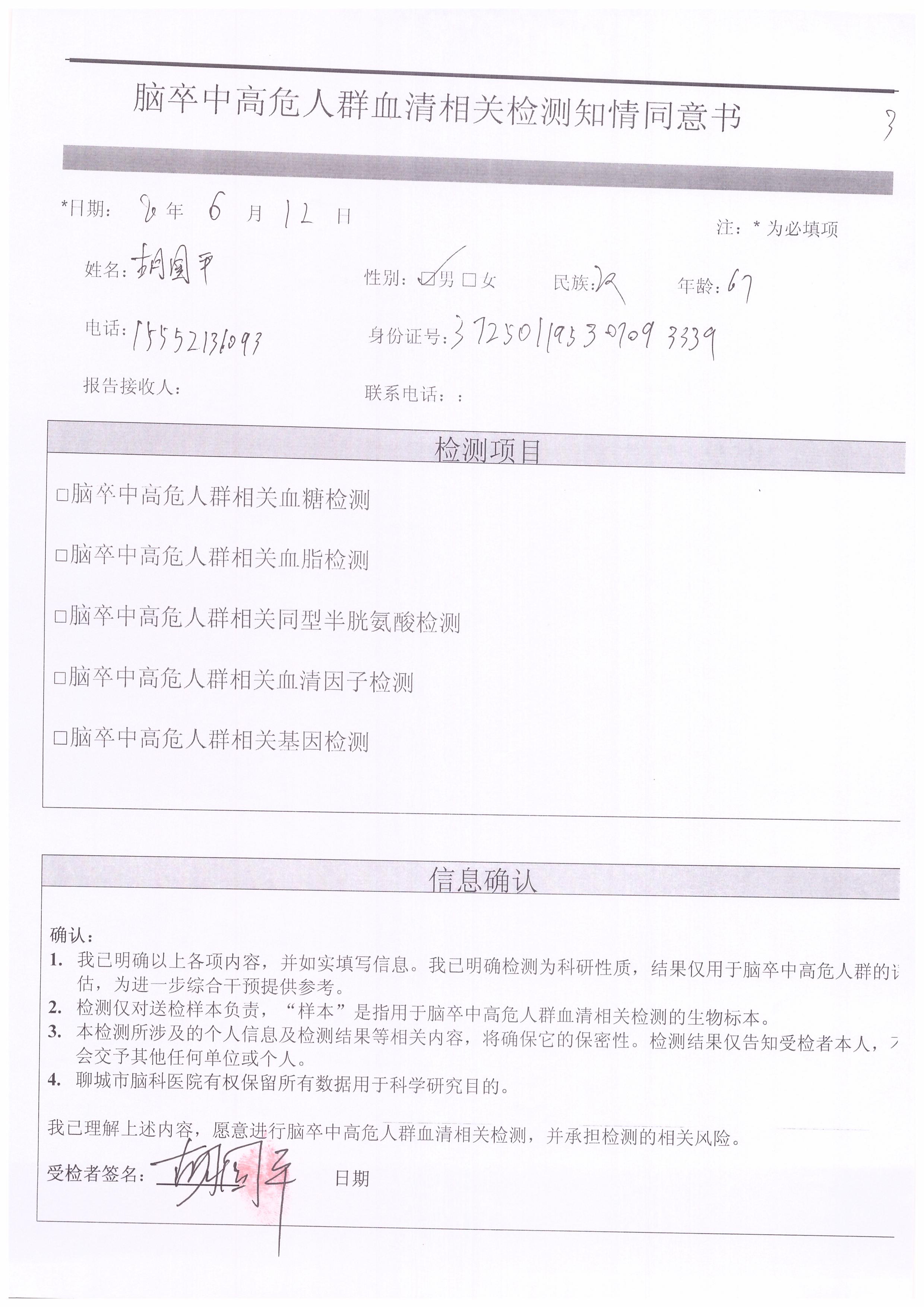

Supplement: Supplementary file 8 — Supplementary file8 (ZIP 23226 KB) [file 10528_2023_10431_MOESM8_ESM.zip › ╓¬╟Θ═1⁄4╥Γ╩Θ6/003.jpg]

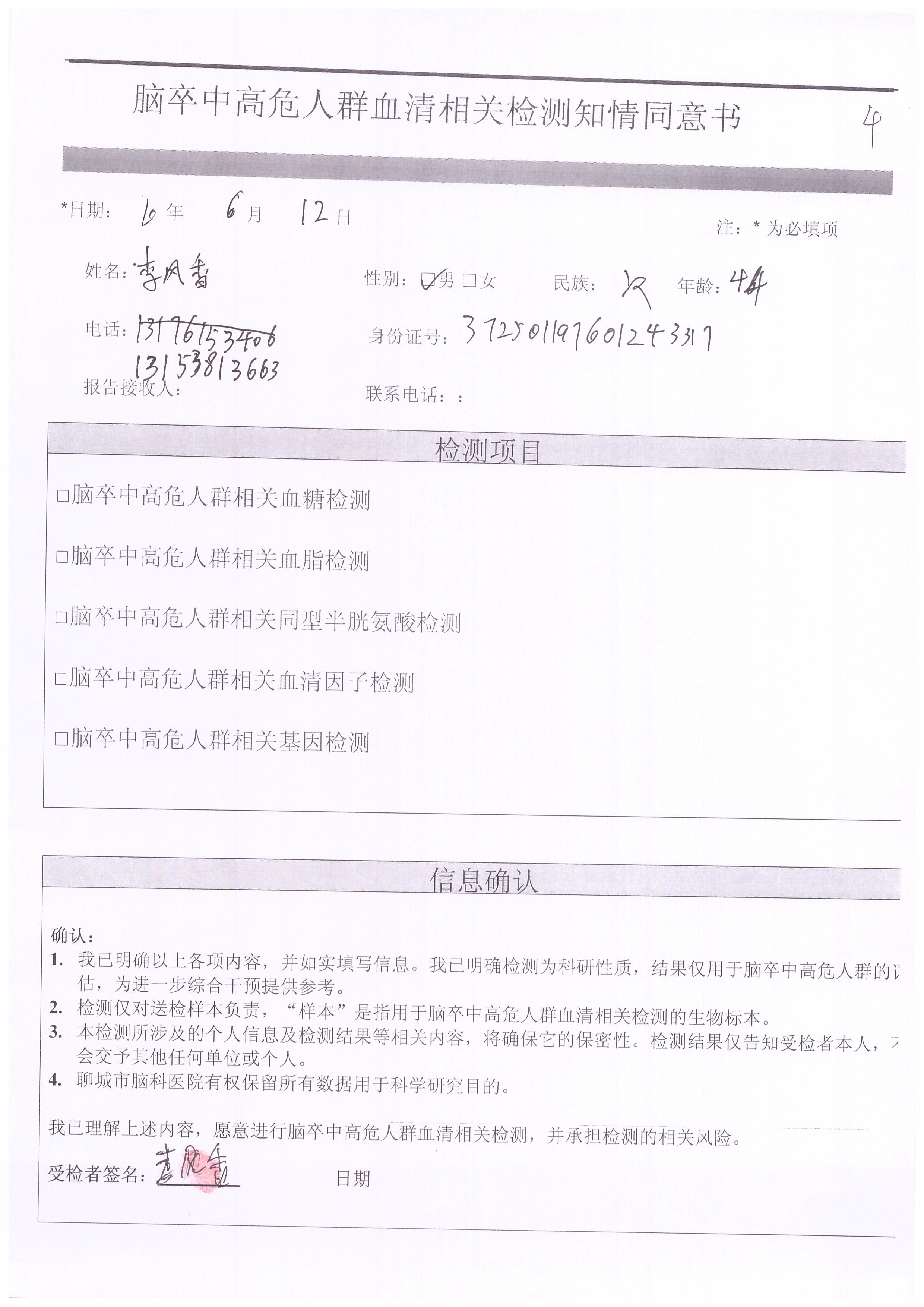

Supplement: Supplementary file 8 — Supplementary file8 (ZIP 23226 KB) [file 10528_2023_10431_MOESM8_ESM.zip › ╓¬╟Θ═1⁄4╥Γ╩Θ6/004.jpg]

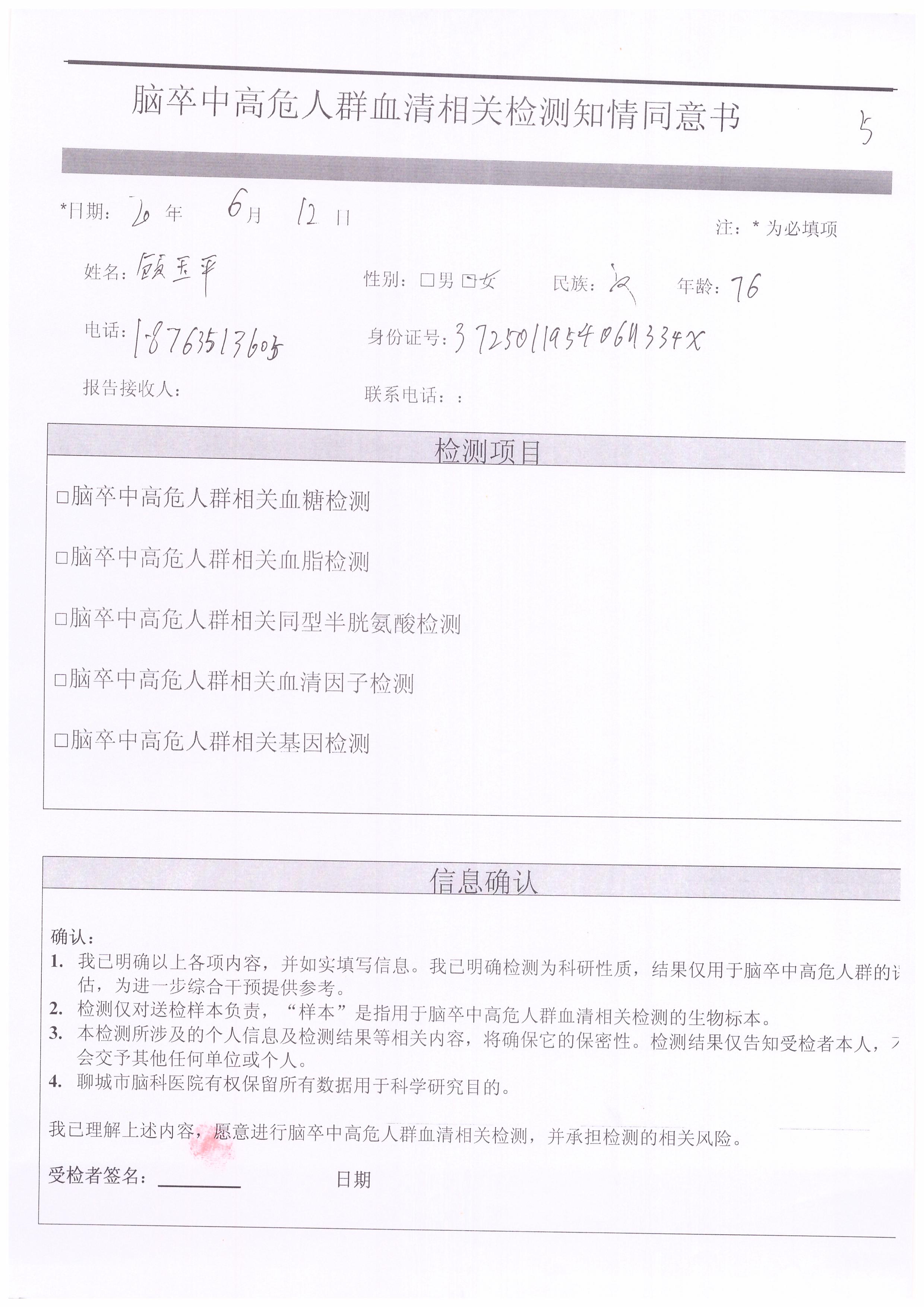

Supplement: Supplementary file 8 — Supplementary file8 (ZIP 23226 KB) [file 10528_2023_10431_MOESM8_ESM.zip › ╓¬╟Θ═1⁄4╥Γ╩Θ6/005.jpg]

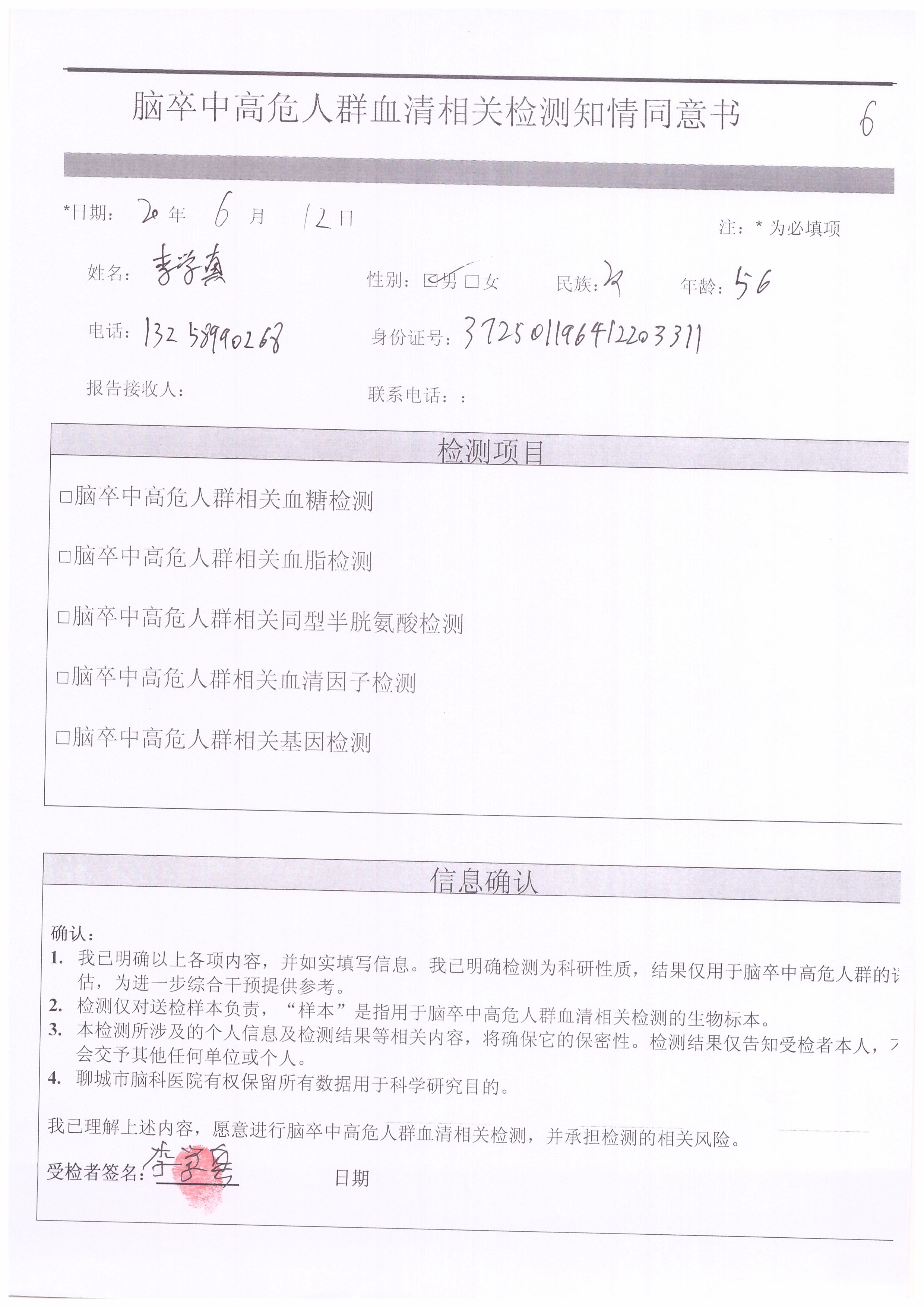

Supplement: Supplementary file 8 — Supplementary file8 (ZIP 23226 KB) [file 10528_2023_10431_MOESM8_ESM.zip › ╓¬╟Θ═1⁄4╥Γ╩Θ6/006.jpg]

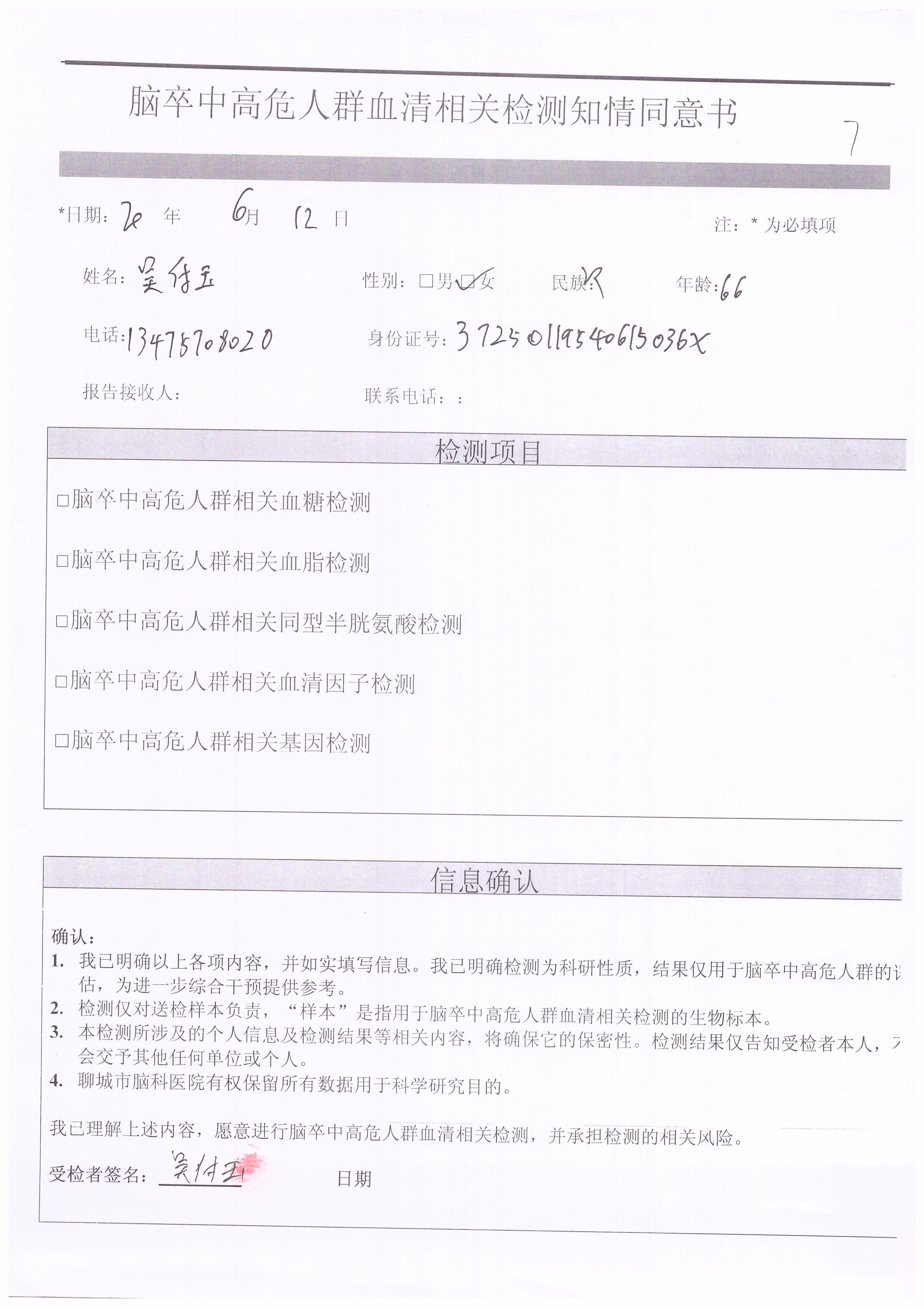

Supplement: Supplementary file 8 — Supplementary file8 (ZIP 23226 KB) [file 10528_2023_10431_MOESM8_ESM.zip › ╓¬╟Θ═1⁄4╥Γ╩Θ6/007.jpg]

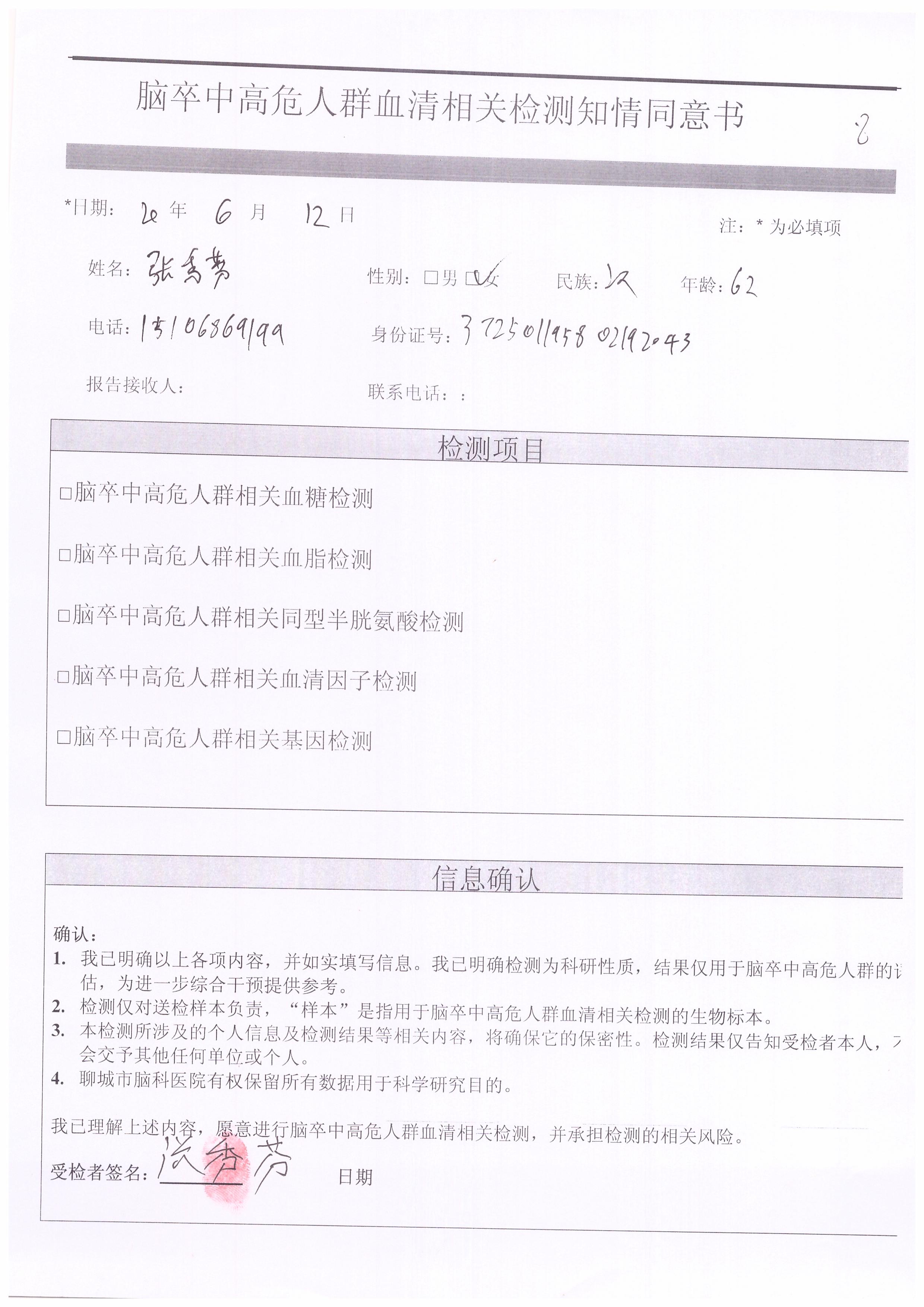

Supplement: Supplementary file 8 — Supplementary file8 (ZIP 23226 KB) [file 10528_2023_10431_MOESM8_ESM.zip › ╓¬╟Θ═1⁄4╥Γ╩Θ6/008.jpg]

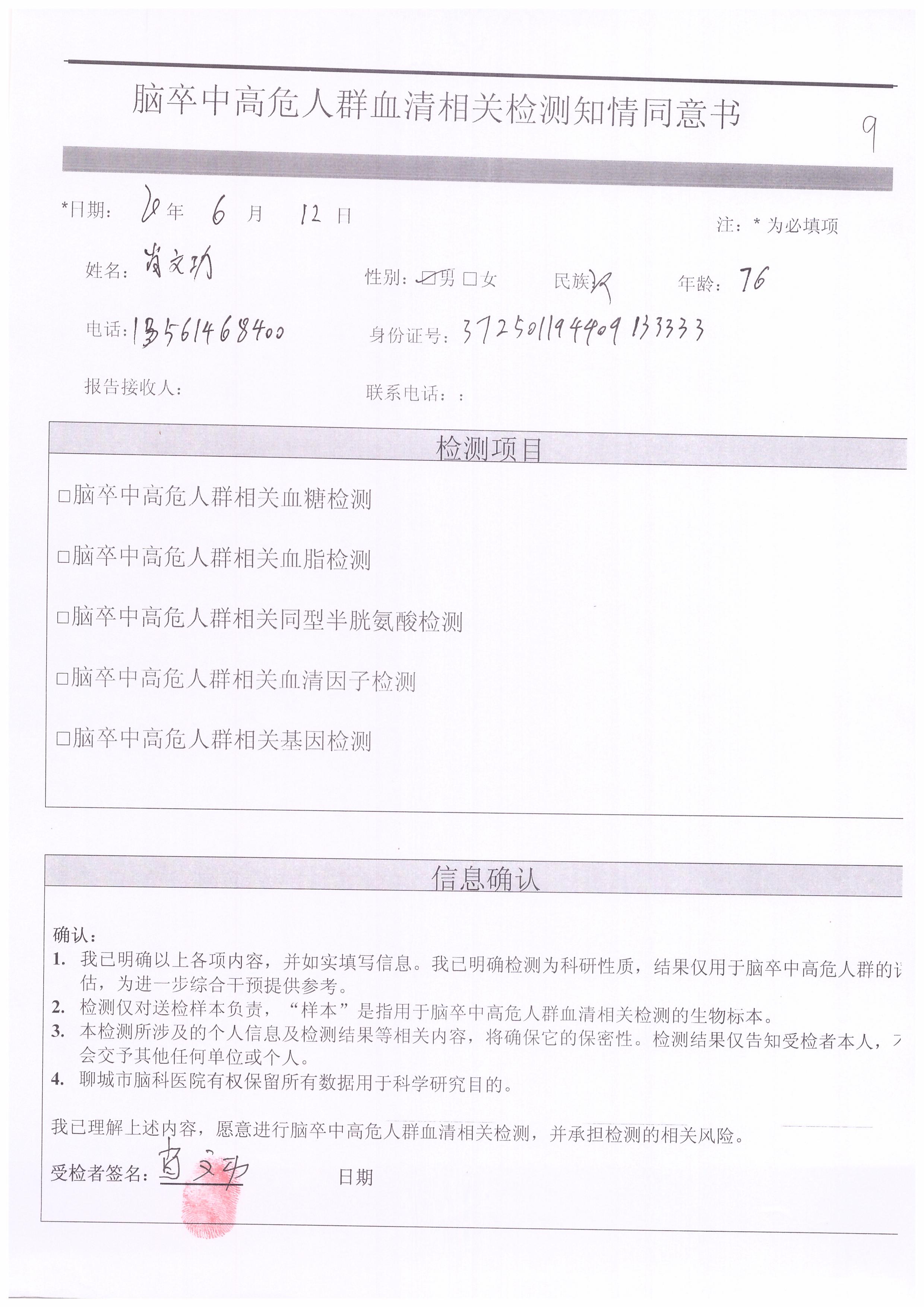

Supplement: Supplementary file 8 — Supplementary file8 (ZIP 23226 KB) [file 10528_2023_10431_MOESM8_ESM.zip › ╓¬╟Θ═1⁄4╥Γ╩Θ6/009.jpg]

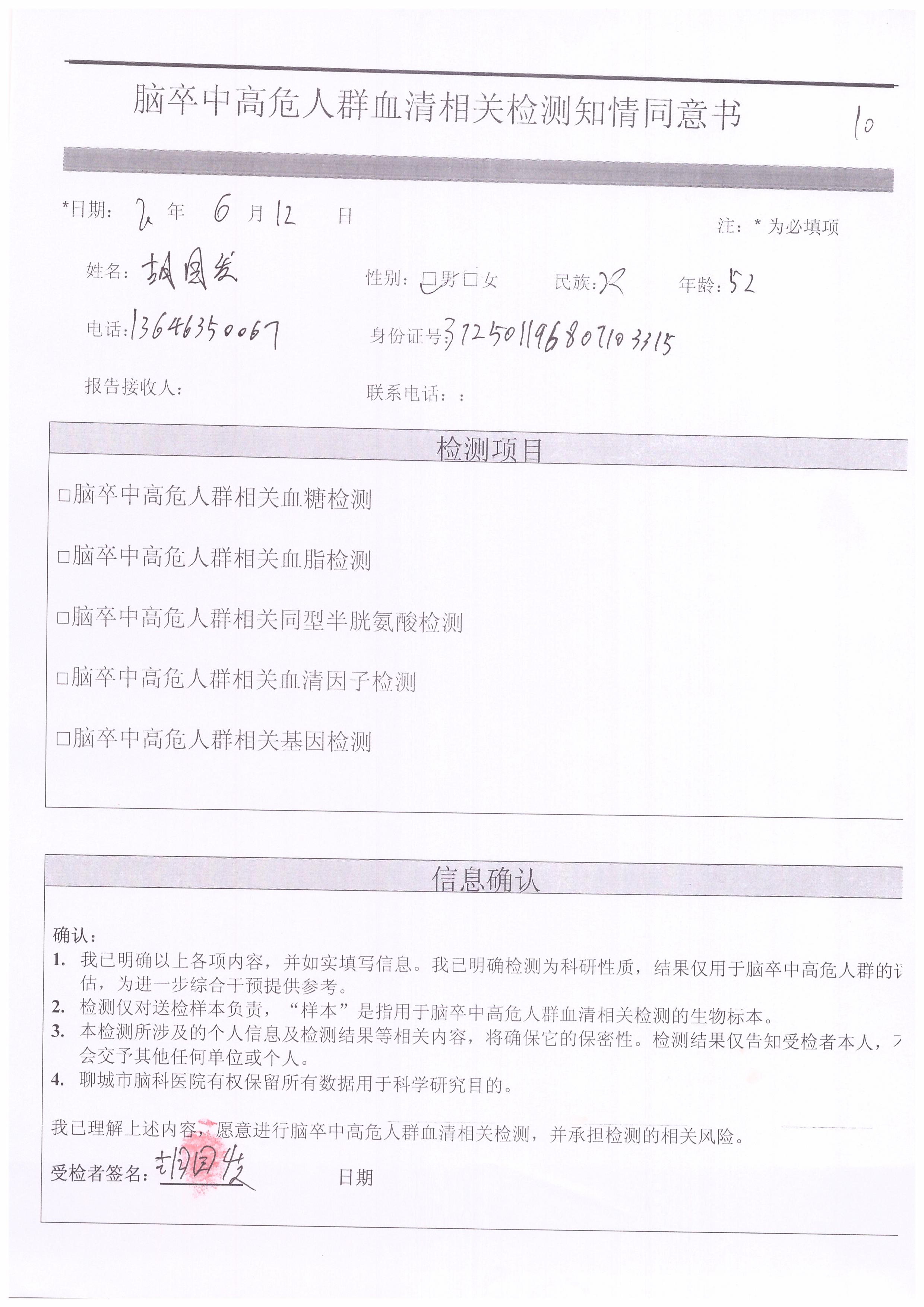

Supplement: Supplementary file 8 — Supplementary file8 (ZIP 23226 KB) [file 10528_2023_10431_MOESM8_ESM.zip › ╓¬╟Θ═1⁄4╥Γ╩Θ6/010.jpg]

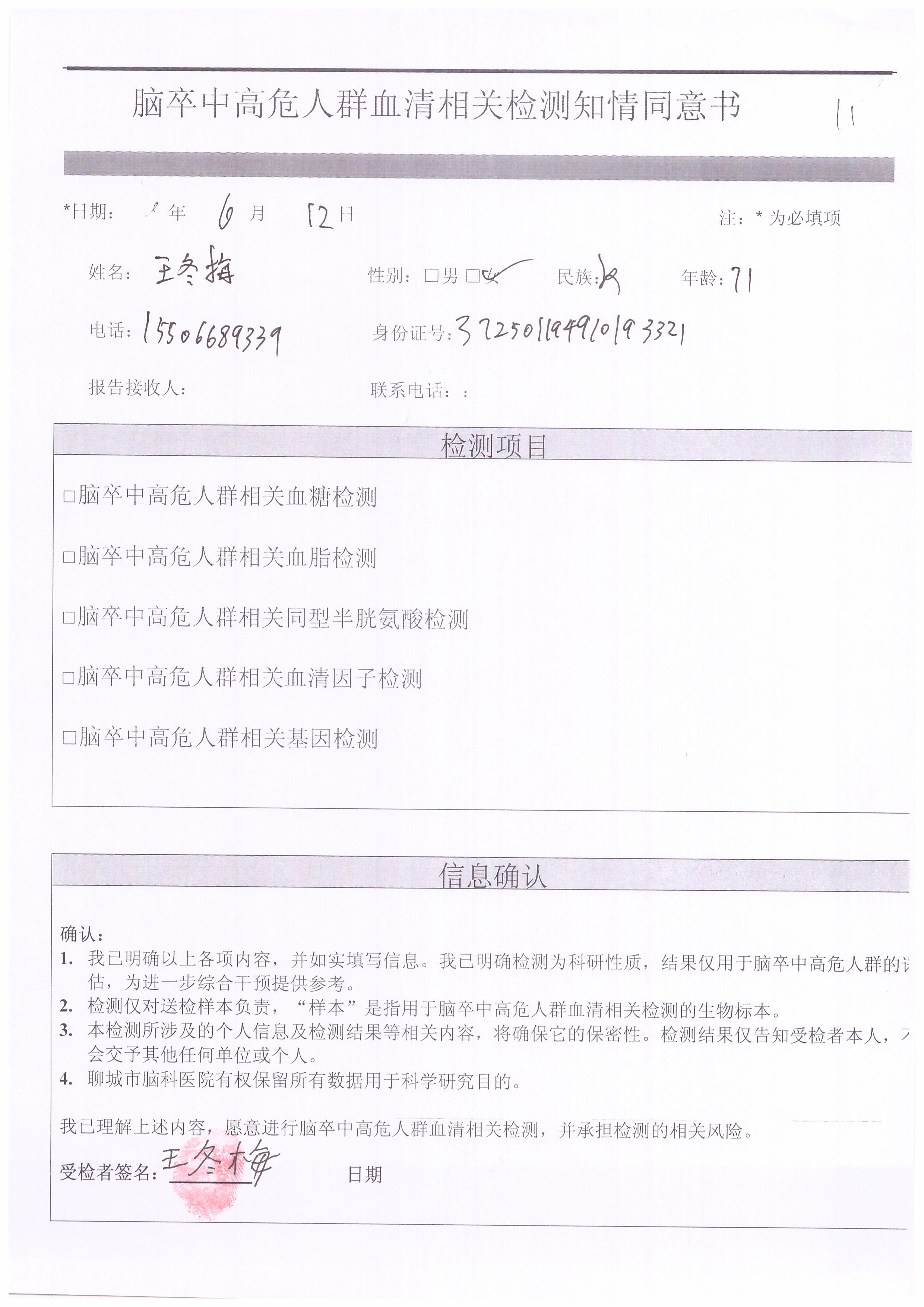

Supplement: Supplementary file 8 — Supplementary file8 (ZIP 23226 KB) [file 10528_2023_10431_MOESM8_ESM.zip › ╓¬╟Θ═1⁄4╥Γ╩Θ6/011.jpg]

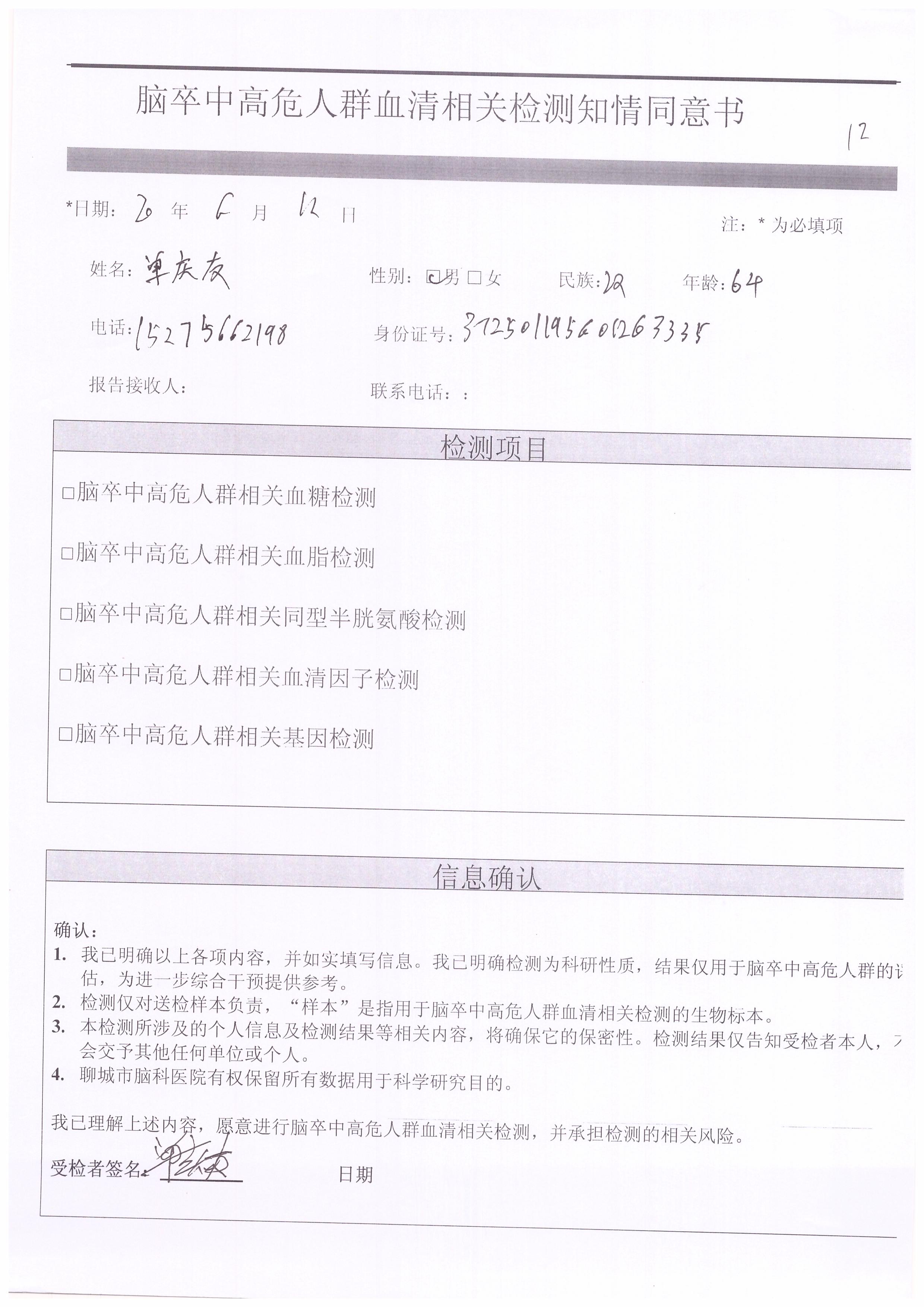

Supplement: Supplementary file 8 — Supplementary file8 (ZIP 23226 KB) [file 10528_2023_10431_MOESM8_ESM.zip › ╓¬╟Θ═1⁄4╥Γ╩Θ6/012.jpg]

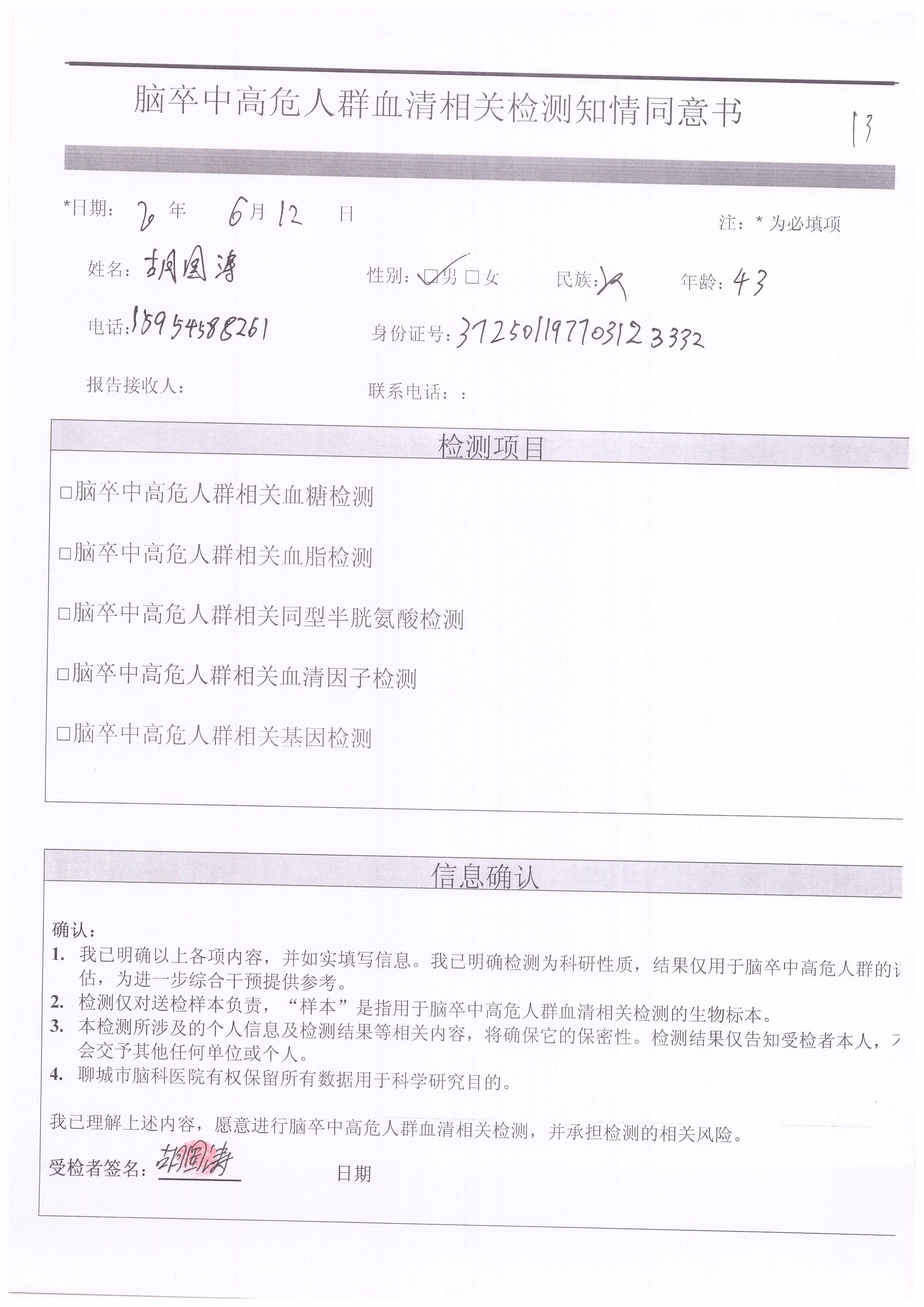

Supplement: Supplementary file 8 — Supplementary file8 (ZIP 23226 KB) [file 10528_2023_10431_MOESM8_ESM.zip › ╓¬╟Θ═1⁄4╥Γ╩Θ6/013.jpg]

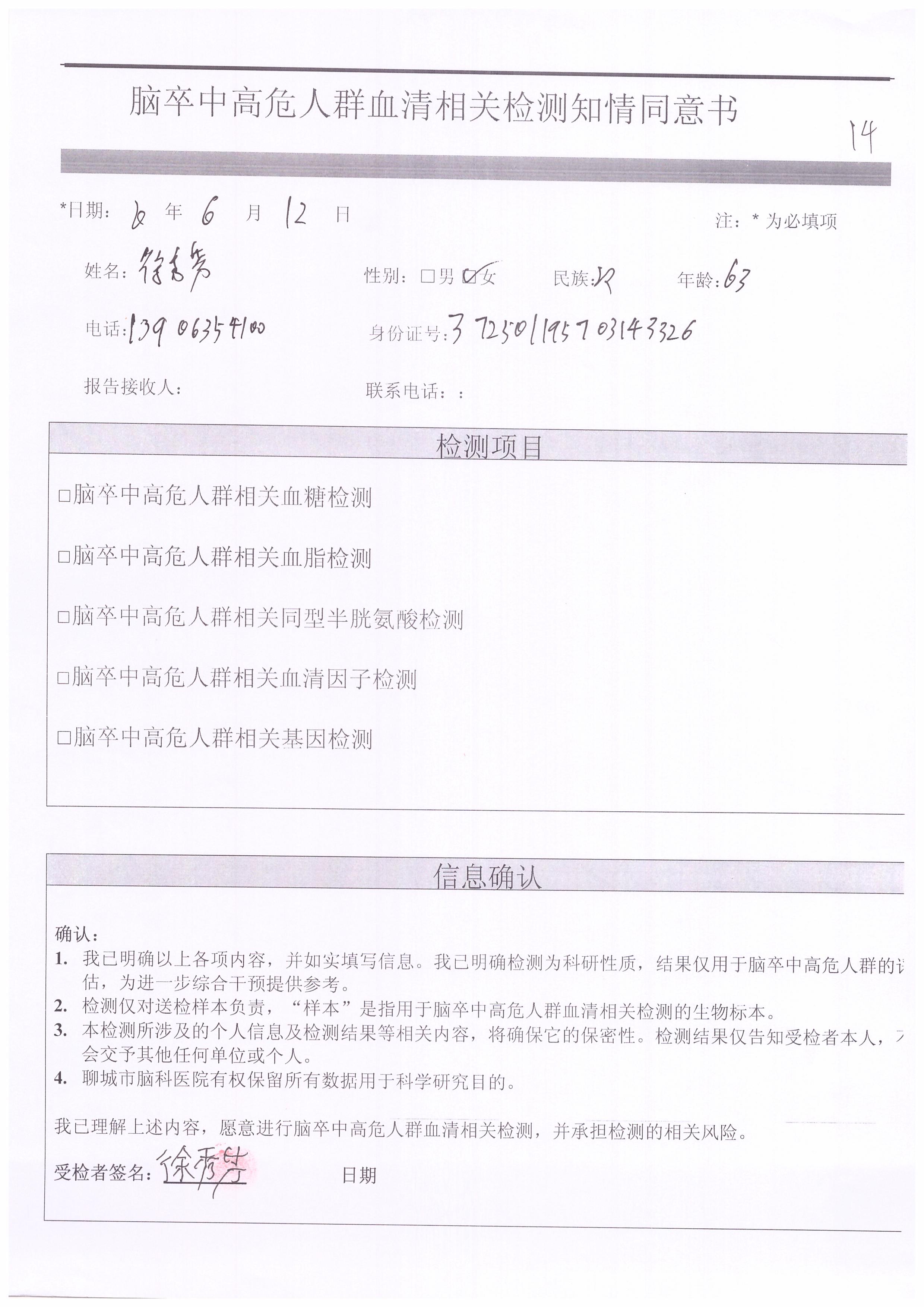

Supplement: Supplementary file 8 — Supplementary file8 (ZIP 23226 KB) [file 10528_2023_10431_MOESM8_ESM.zip › ╓¬╟Θ═1⁄4╥Γ╩Θ6/014.jpg]

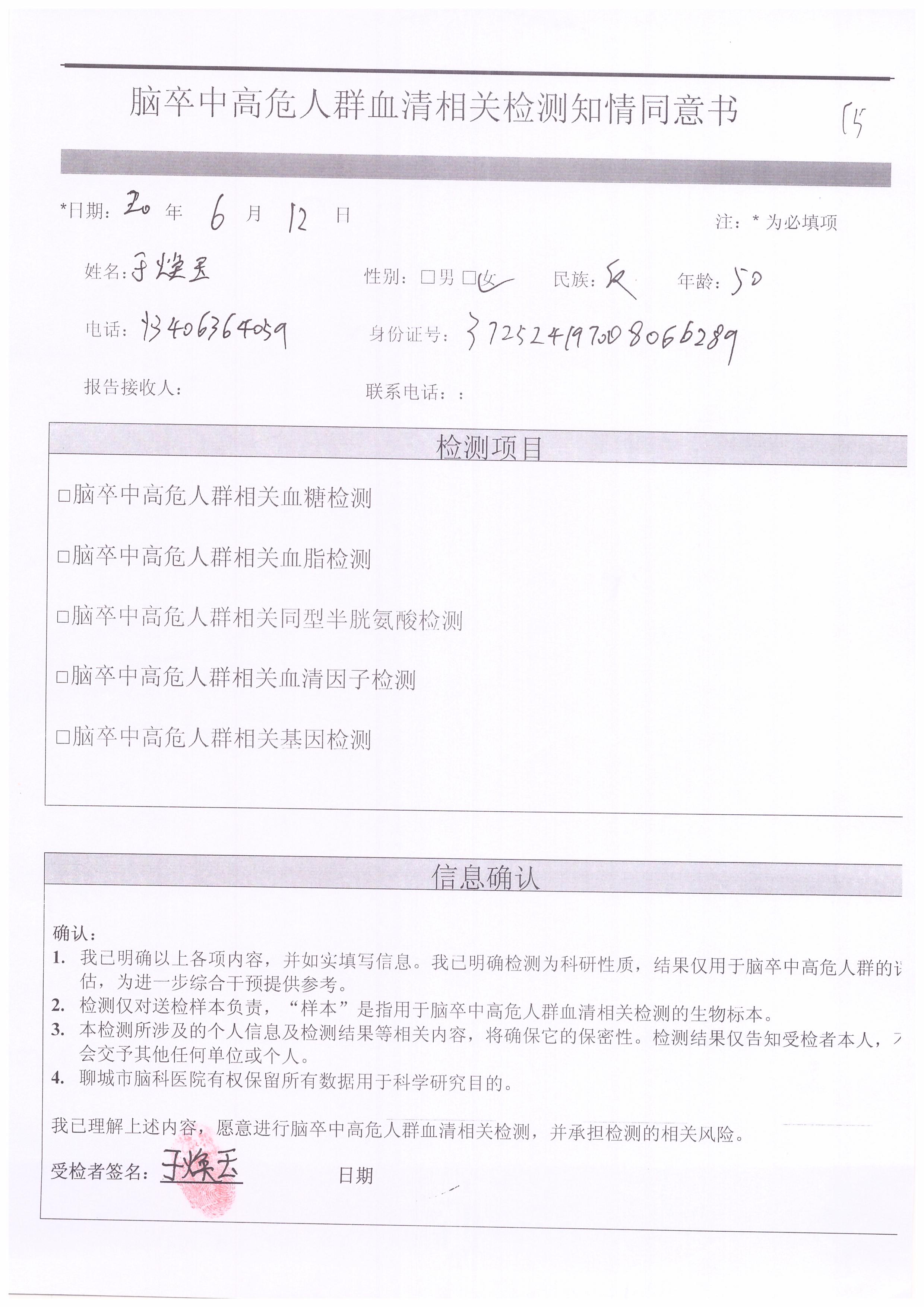

Supplement: Supplementary file 8 — Supplementary file8 (ZIP 23226 KB) [file 10528_2023_10431_MOESM8_ESM.zip › ╓¬╟Θ═1⁄4╥Γ╩Θ6/015.jpg]

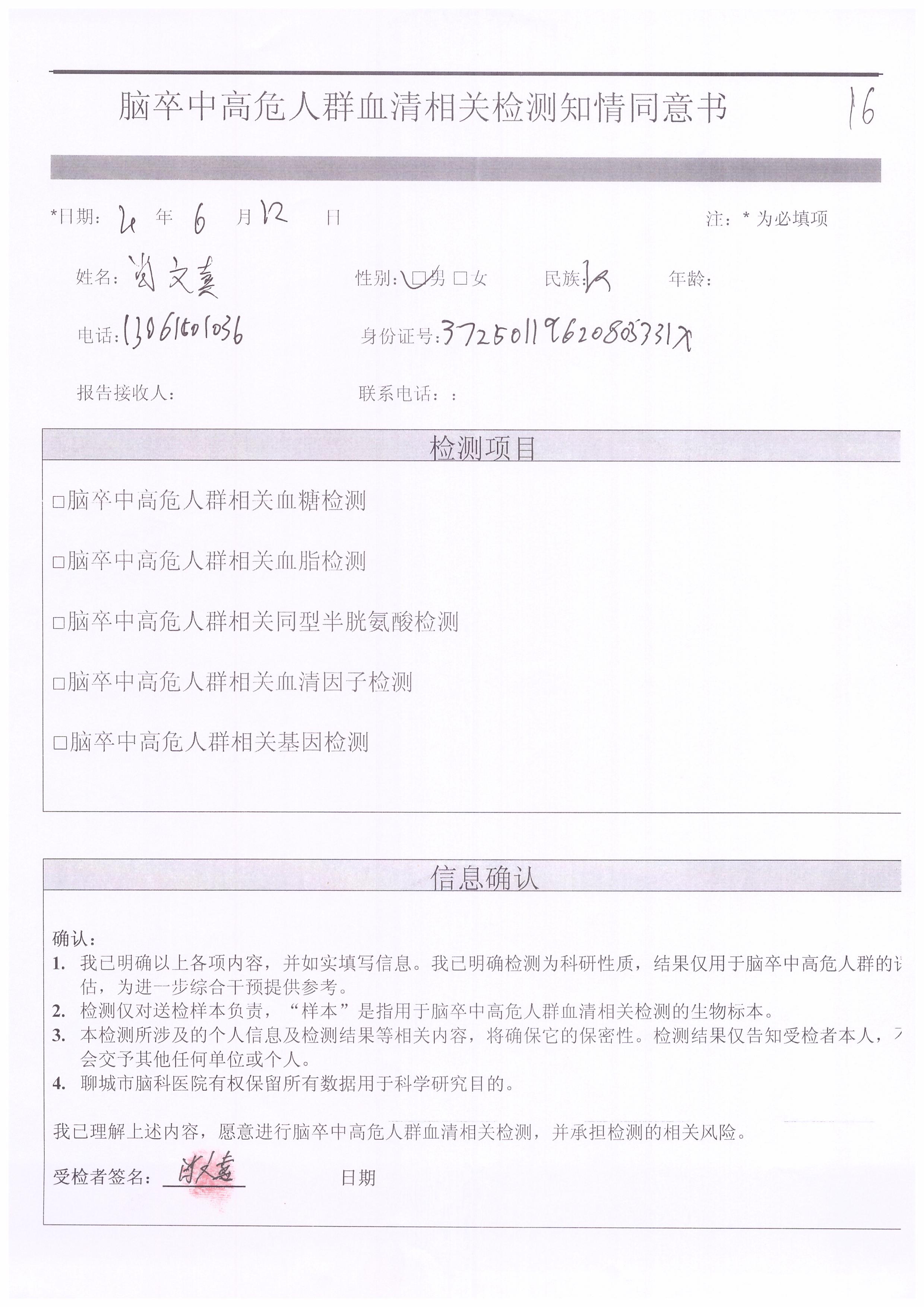

Supplement: Supplementary file 8 — Supplementary file8 (ZIP 23226 KB) [file 10528_2023_10431_MOESM8_ESM.zip › ╓¬╟Θ═1⁄4╥Γ╩Θ6/016.jpg]

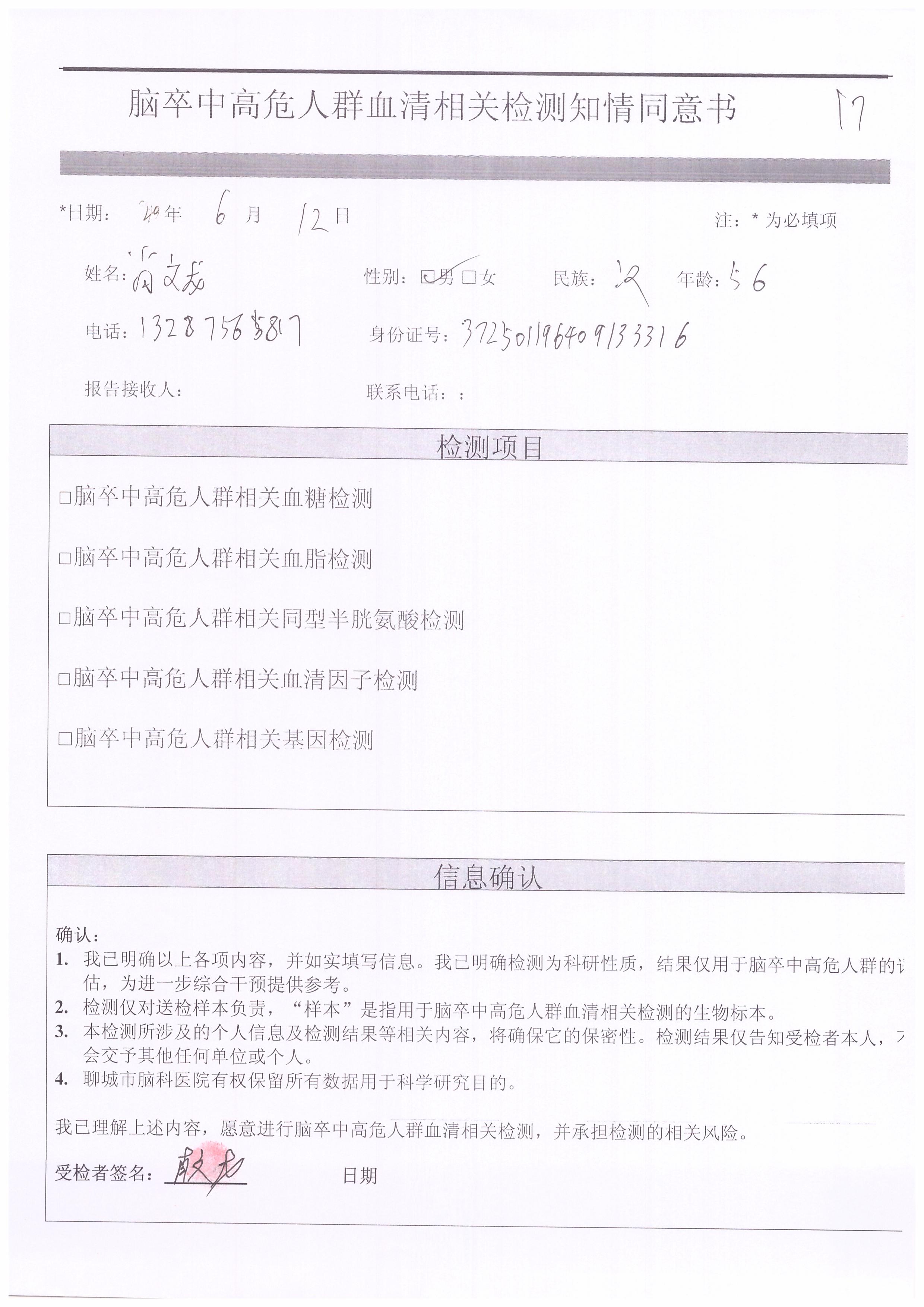

Supplement: Supplementary file 8 — Supplementary file8 (ZIP 23226 KB) [file 10528_2023_10431_MOESM8_ESM.zip › ╓¬╟Θ═1⁄4╥Γ╩Θ6/017.jpg]

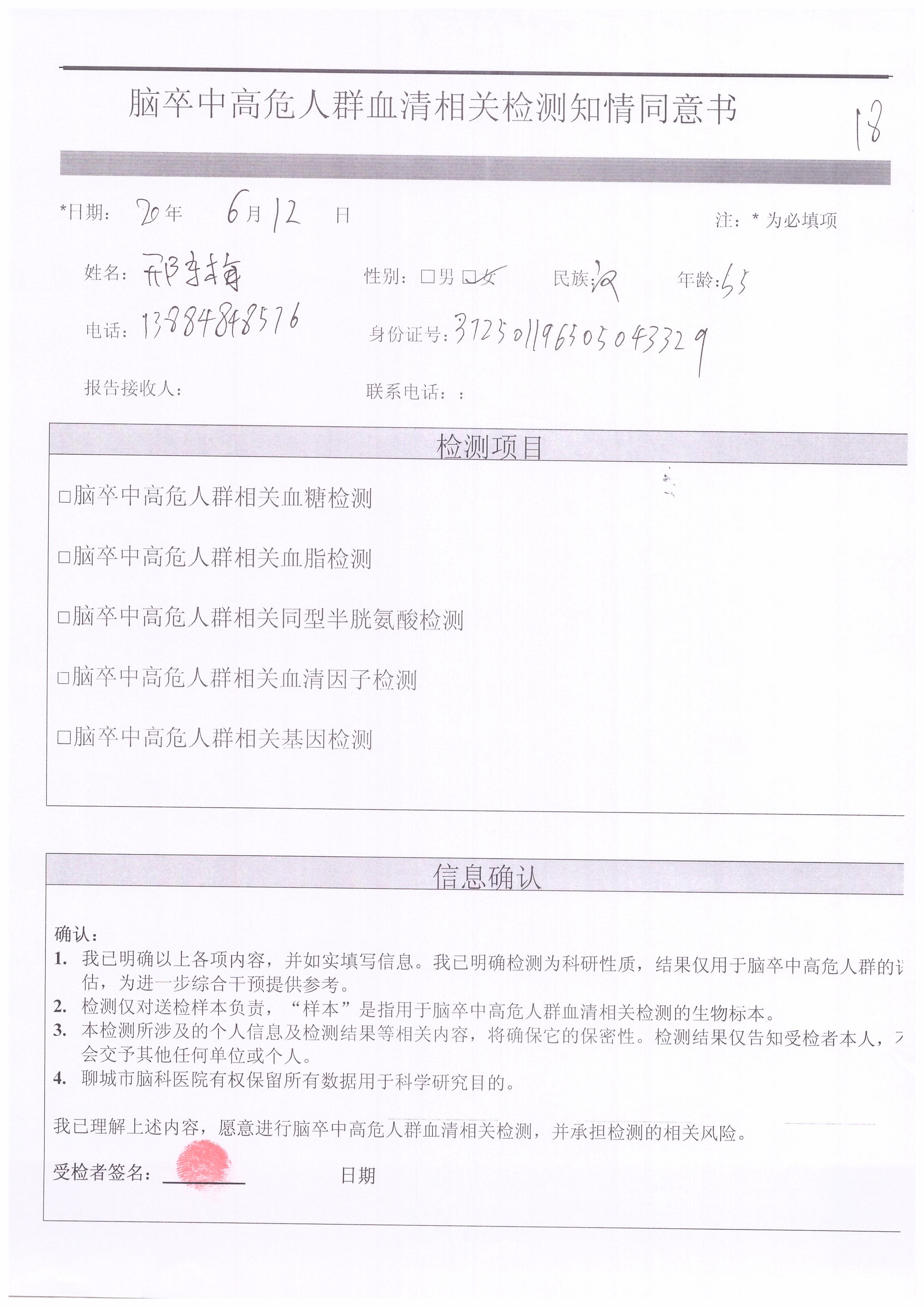

Supplement: Supplementary file 8 — Supplementary file8 (ZIP 23226 KB) [file 10528_2023_10431_MOESM8_ESM.zip › ╓¬╟Θ═1⁄4╥Γ╩Θ6/018.jpg]

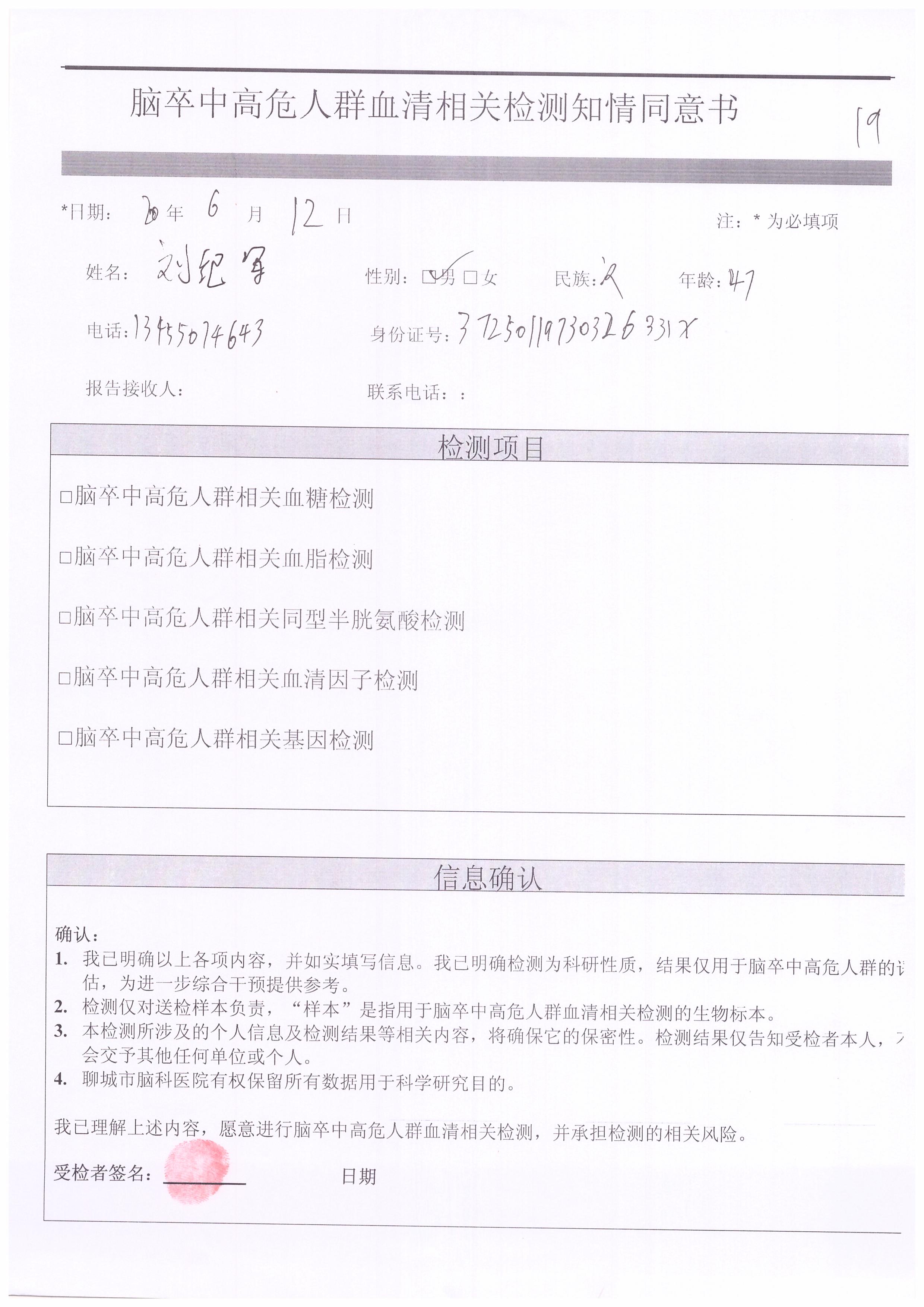

Supplement: Supplementary file 8 — Supplementary file8 (ZIP 23226 KB) [file 10528_2023_10431_MOESM8_ESM.zip › ╓¬╟Θ═1⁄4╥Γ╩Θ6/019.jpg]

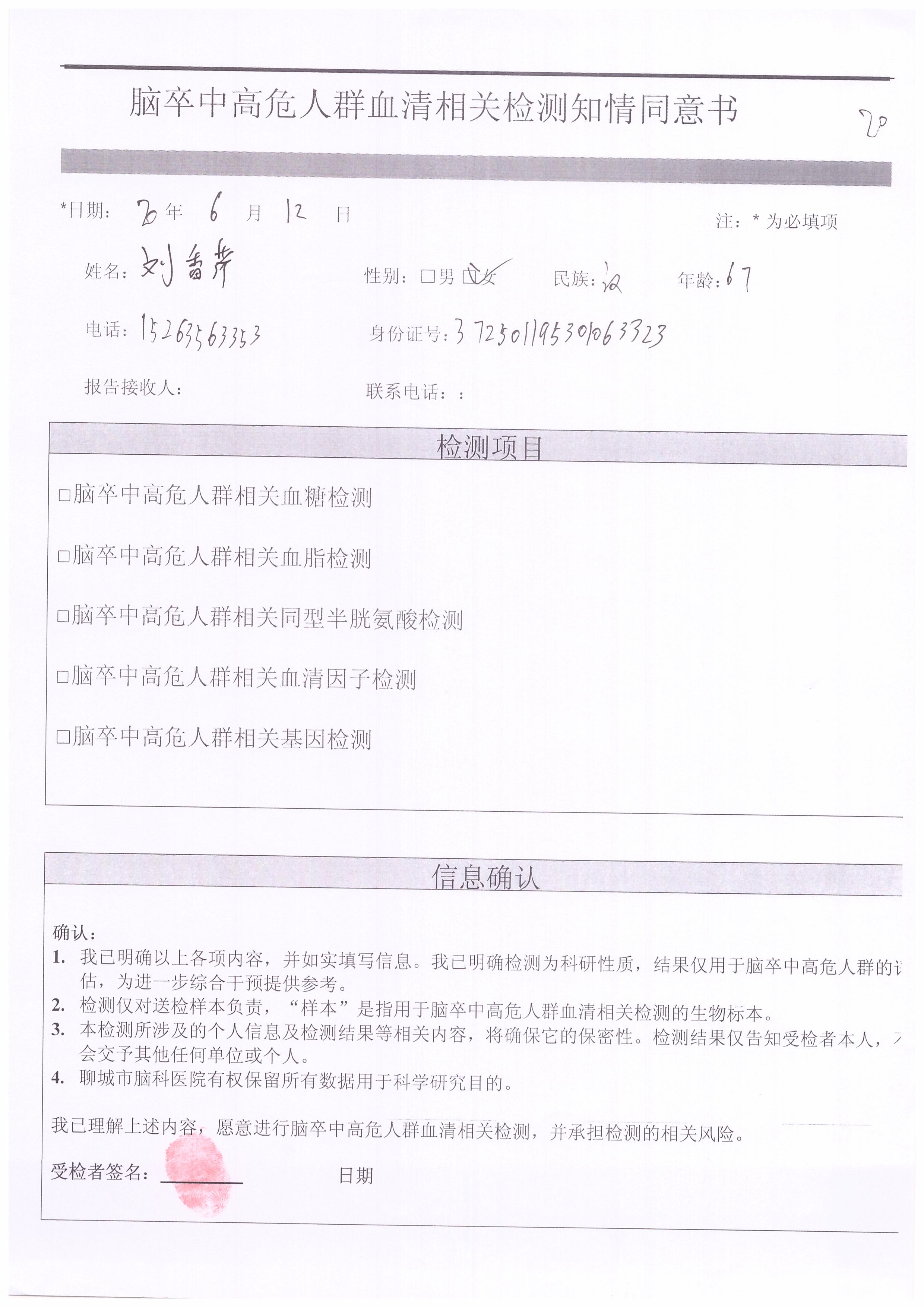

Supplement: Supplementary file 8 — Supplementary file8 (ZIP 23226 KB) [file 10528_2023_10431_MOESM8_ESM.zip › ╓¬╟Θ═1⁄4╥Γ╩Θ6/020.jpg]

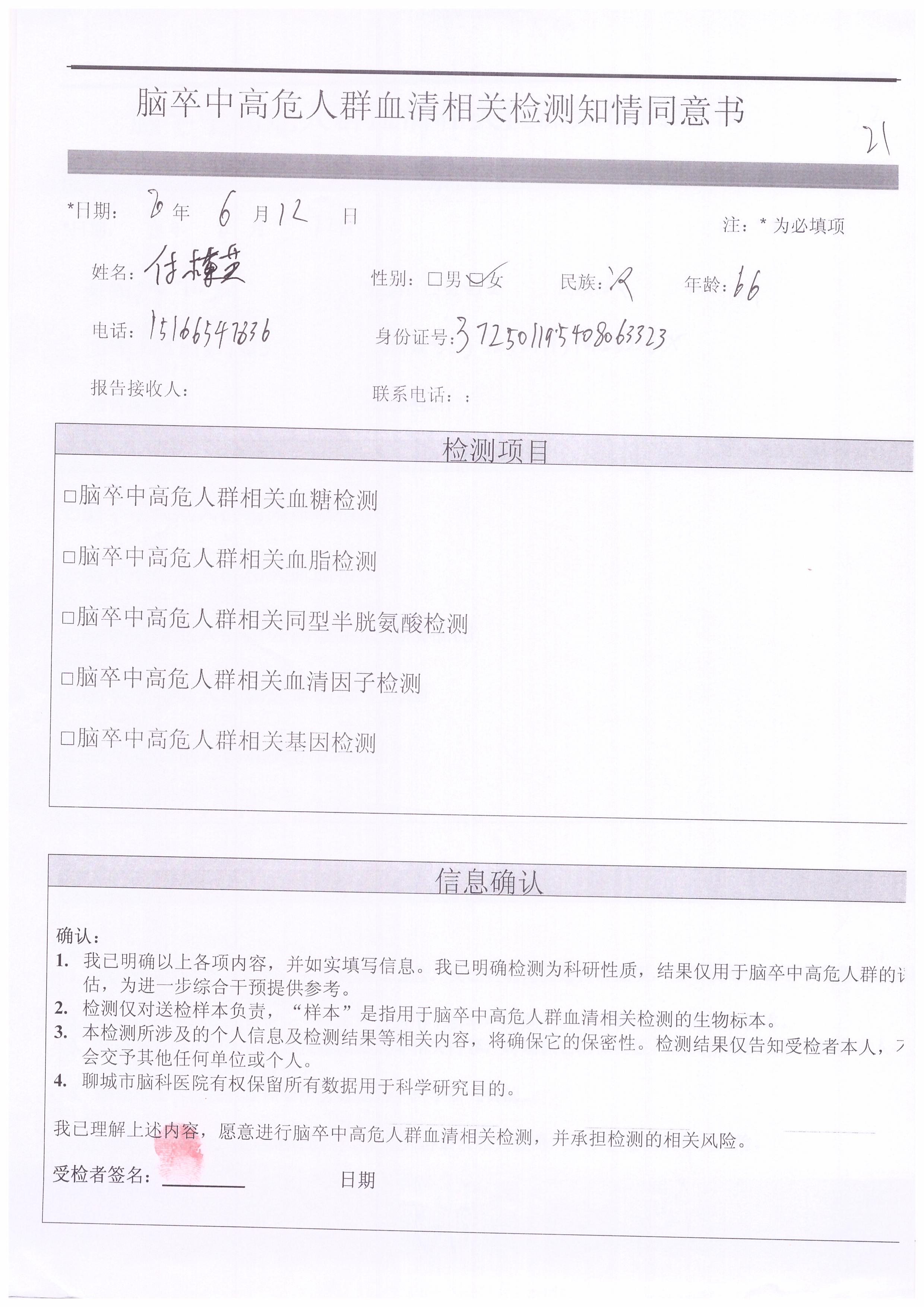

Supplement: Supplementary file 8 — Supplementary file8 (ZIP 23226 KB) [file 10528_2023_10431_MOESM8_ESM.zip › ╓¬╟Θ═1⁄4╥Γ╩Θ6/021.jpg]

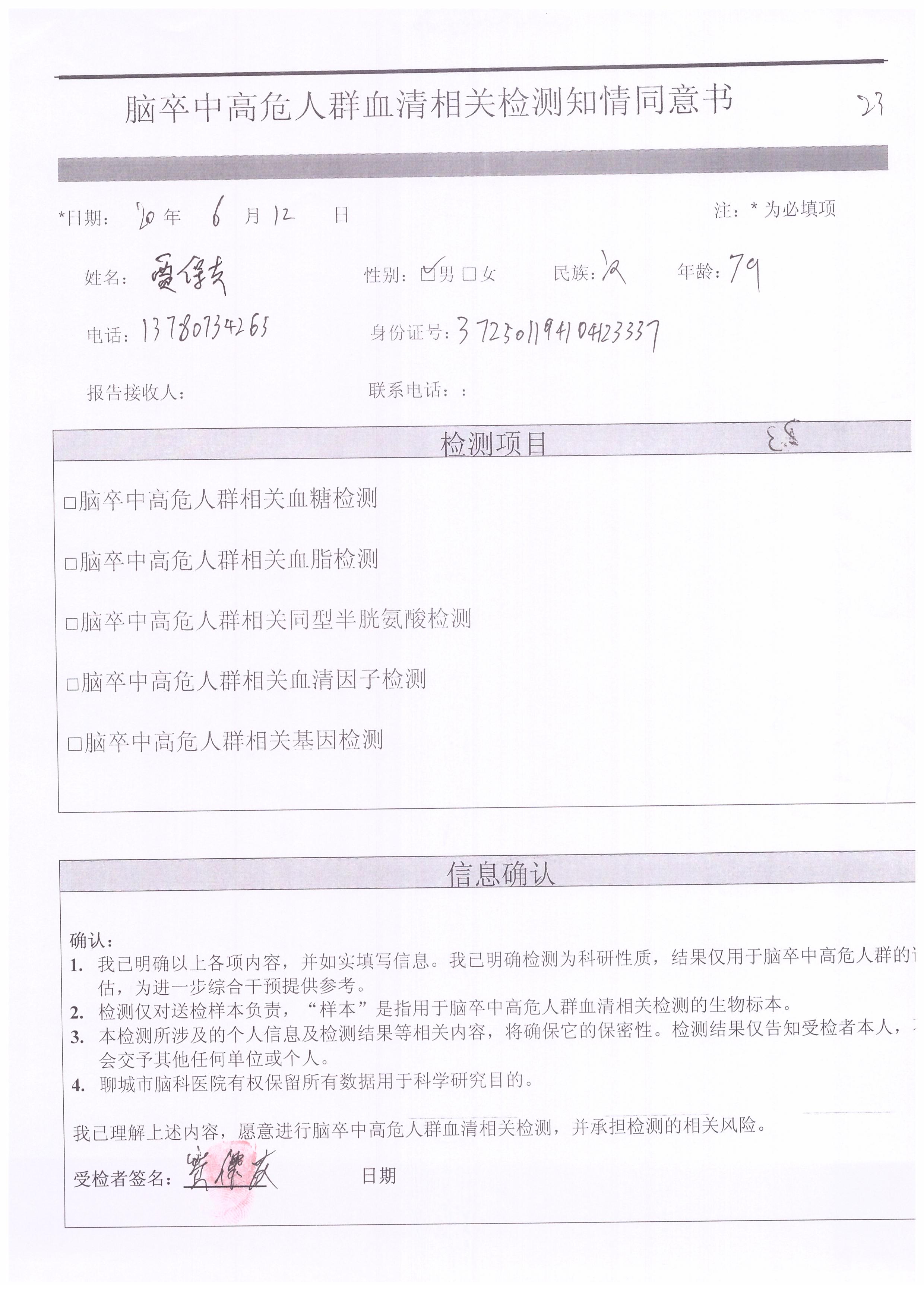

Supplement: Supplementary file 8 — Supplementary file8 (ZIP 23226 KB) [file 10528_2023_10431_MOESM8_ESM.zip › ╓¬╟Θ═1⁄4╥Γ╩Θ6/022.jpg]

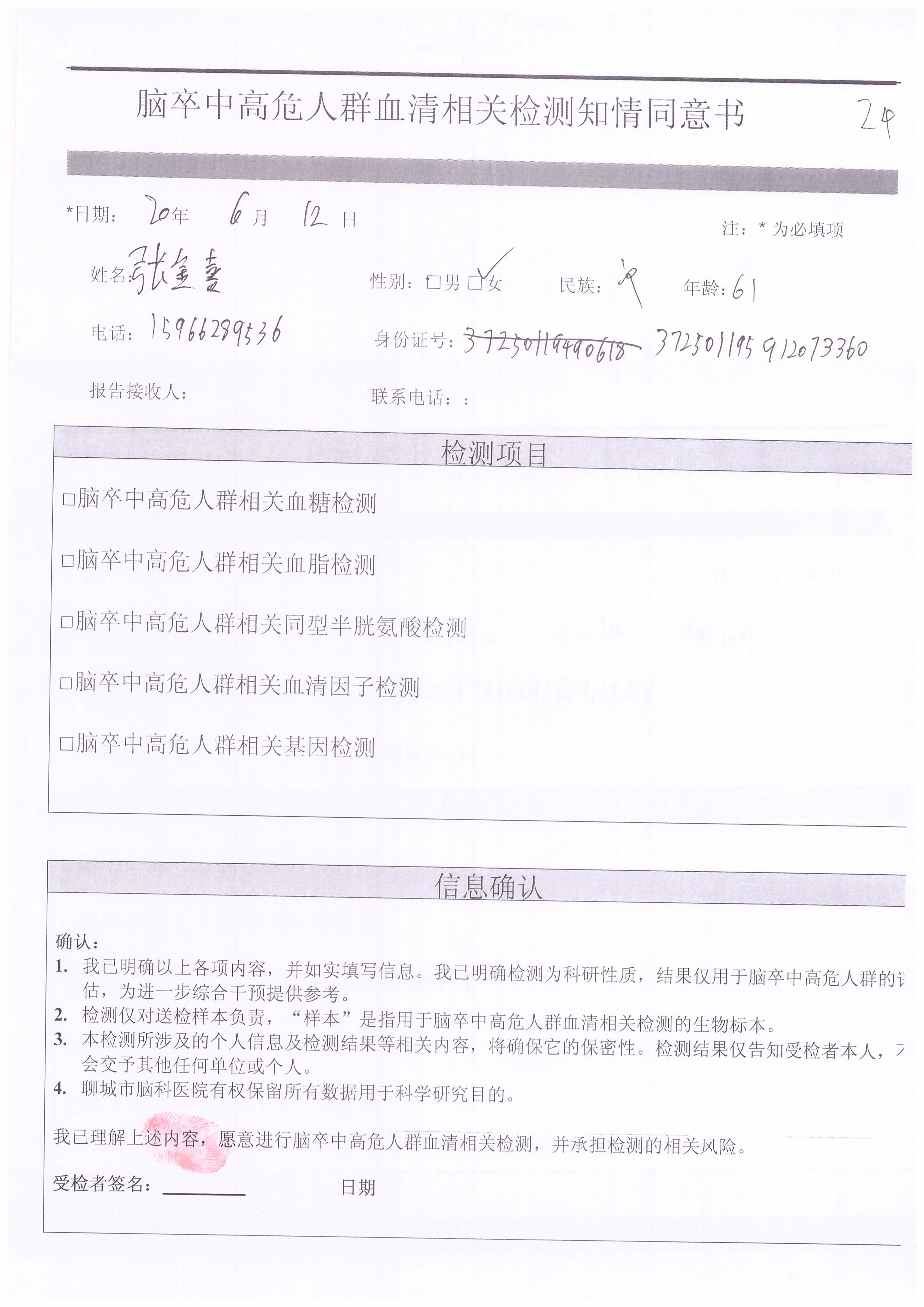

Supplement: Supplementary file 8 — Supplementary file8 (ZIP 23226 KB) [file 10528_2023_10431_MOESM8_ESM.zip › ╓¬╟Θ═1⁄4╥Γ╩Θ6/023.jpg]

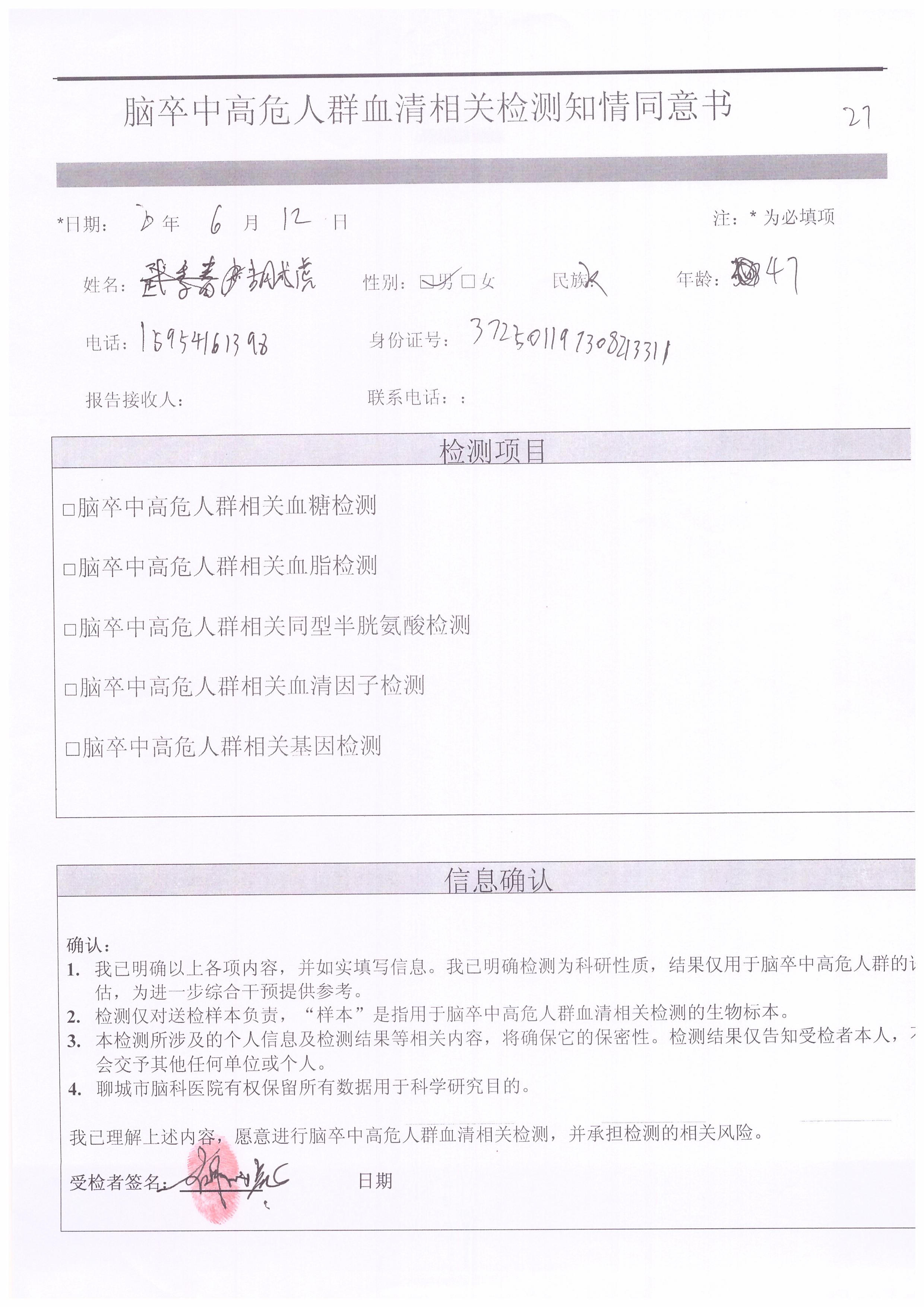

Supplement: Supplementary file 8 — Supplementary file8 (ZIP 23226 KB) [file 10528_2023_10431_MOESM8_ESM.zip › ╓¬╟Θ═1⁄4╥Γ╩Θ6/024.jpg]

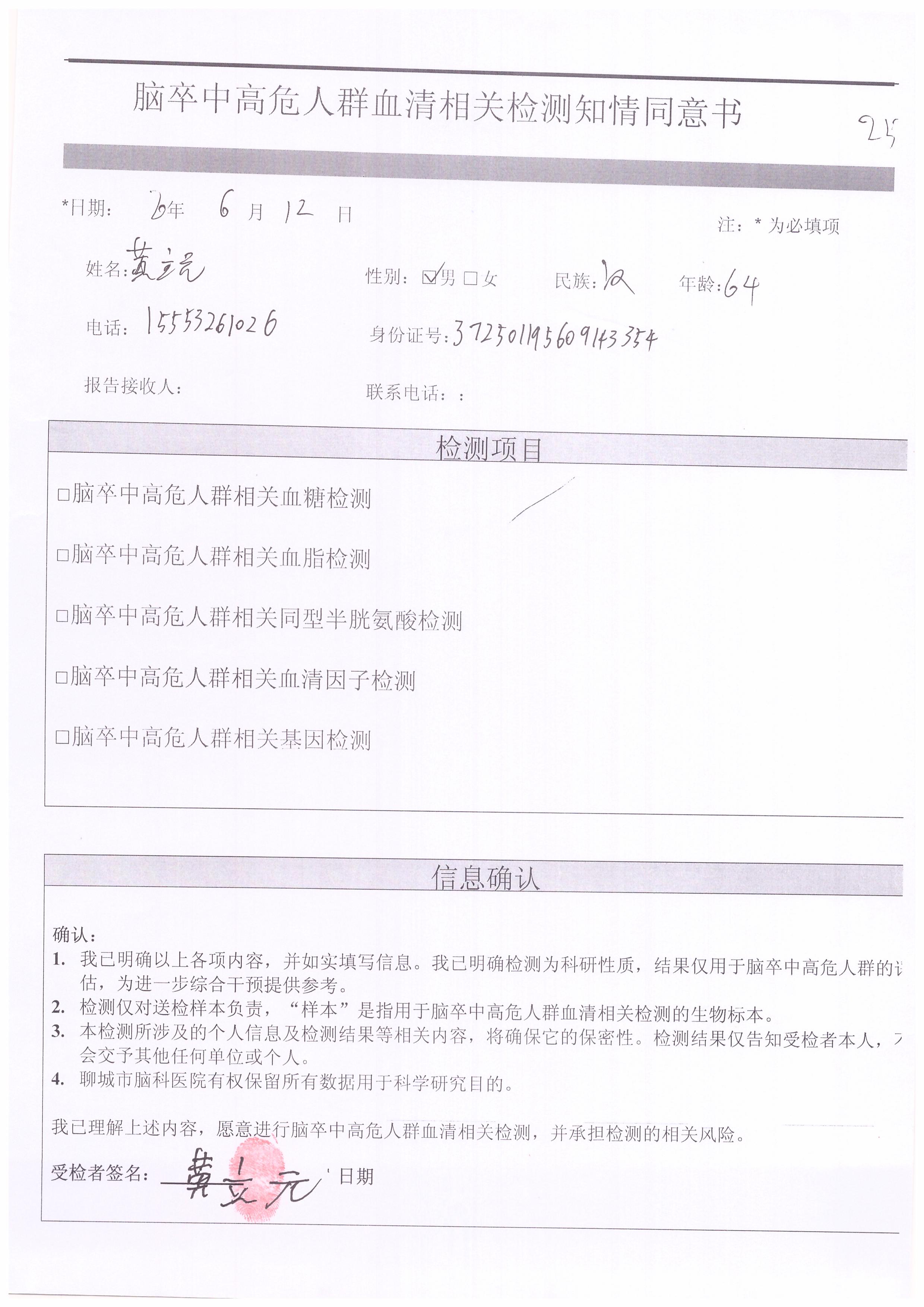

Supplement: Supplementary file 8 — Supplementary file8 (ZIP 23226 KB) [file 10528_2023_10431_MOESM8_ESM.zip › ╓¬╟Θ═1⁄4╥Γ╩Θ6/025.jpg]

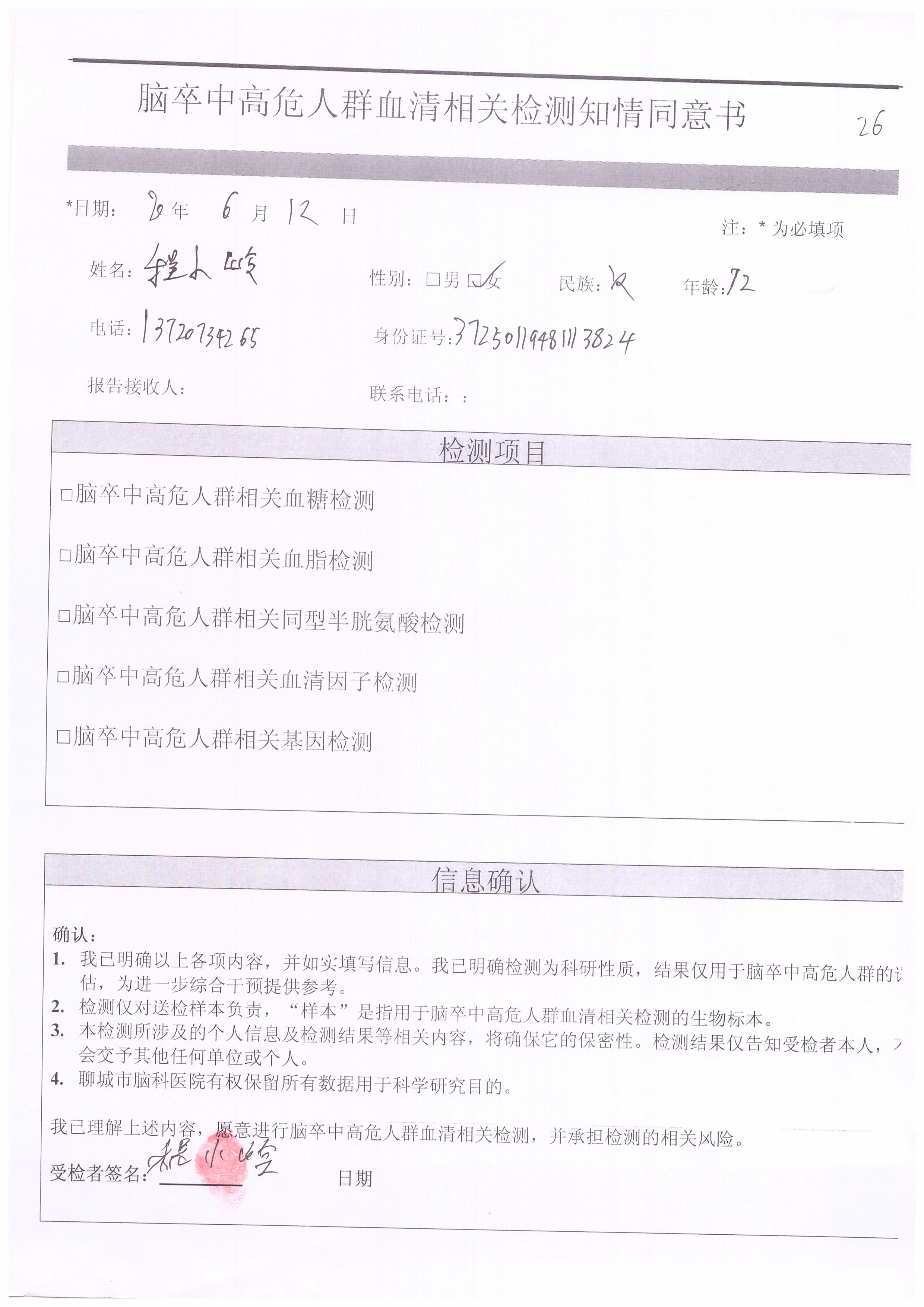

Supplement: Supplementary file 8 — Supplementary file8 (ZIP 23226 KB) [file 10528_2023_10431_MOESM8_ESM.zip › ╓¬╟Θ═1⁄4╥Γ╩Θ6/026.jpg]

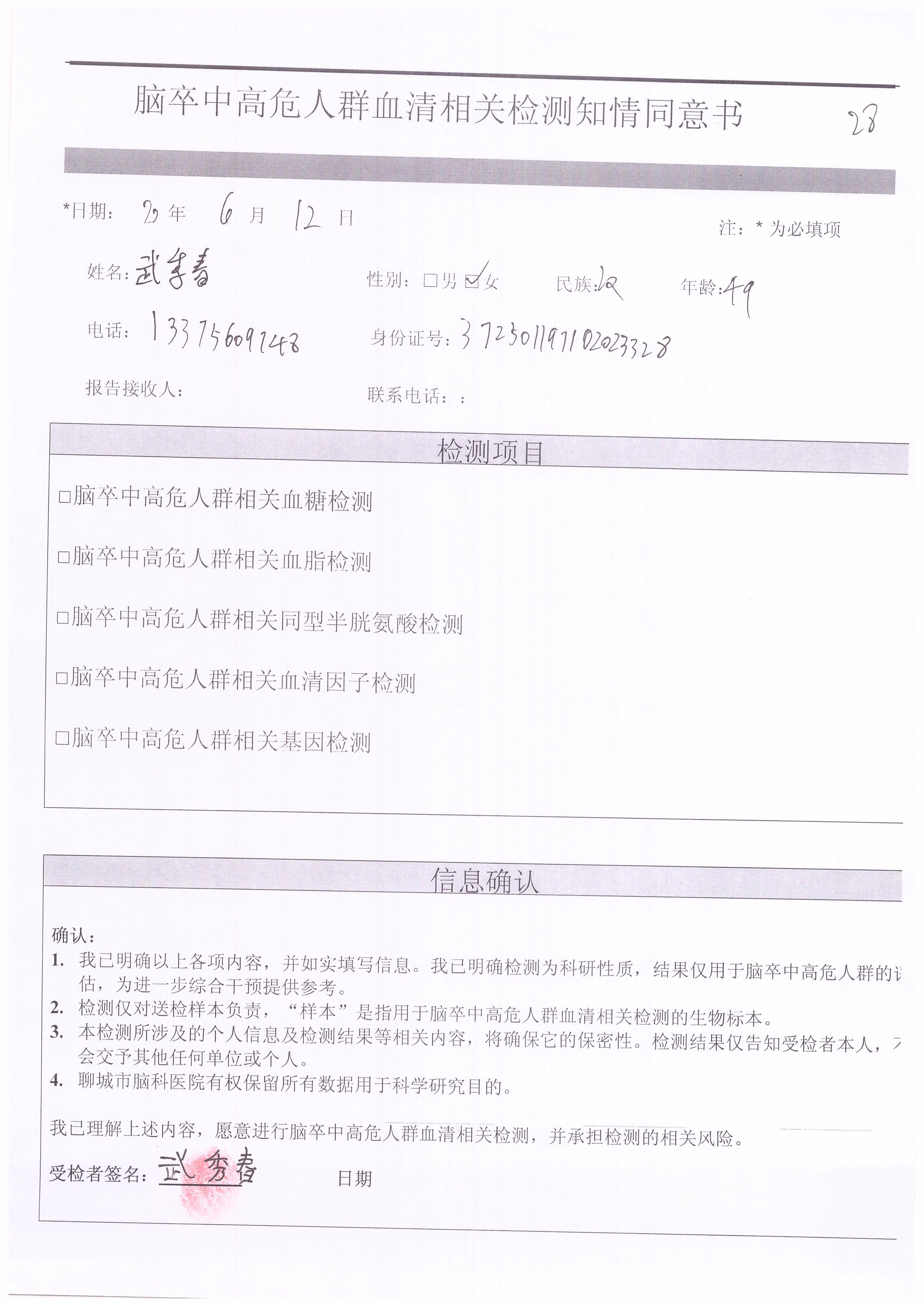

Supplement: Supplementary file 8 — Supplementary file8 (ZIP 23226 KB) [file 10528_2023_10431_MOESM8_ESM.zip › ╓¬╟Θ═1⁄4╥Γ╩Θ6/027.jpg]

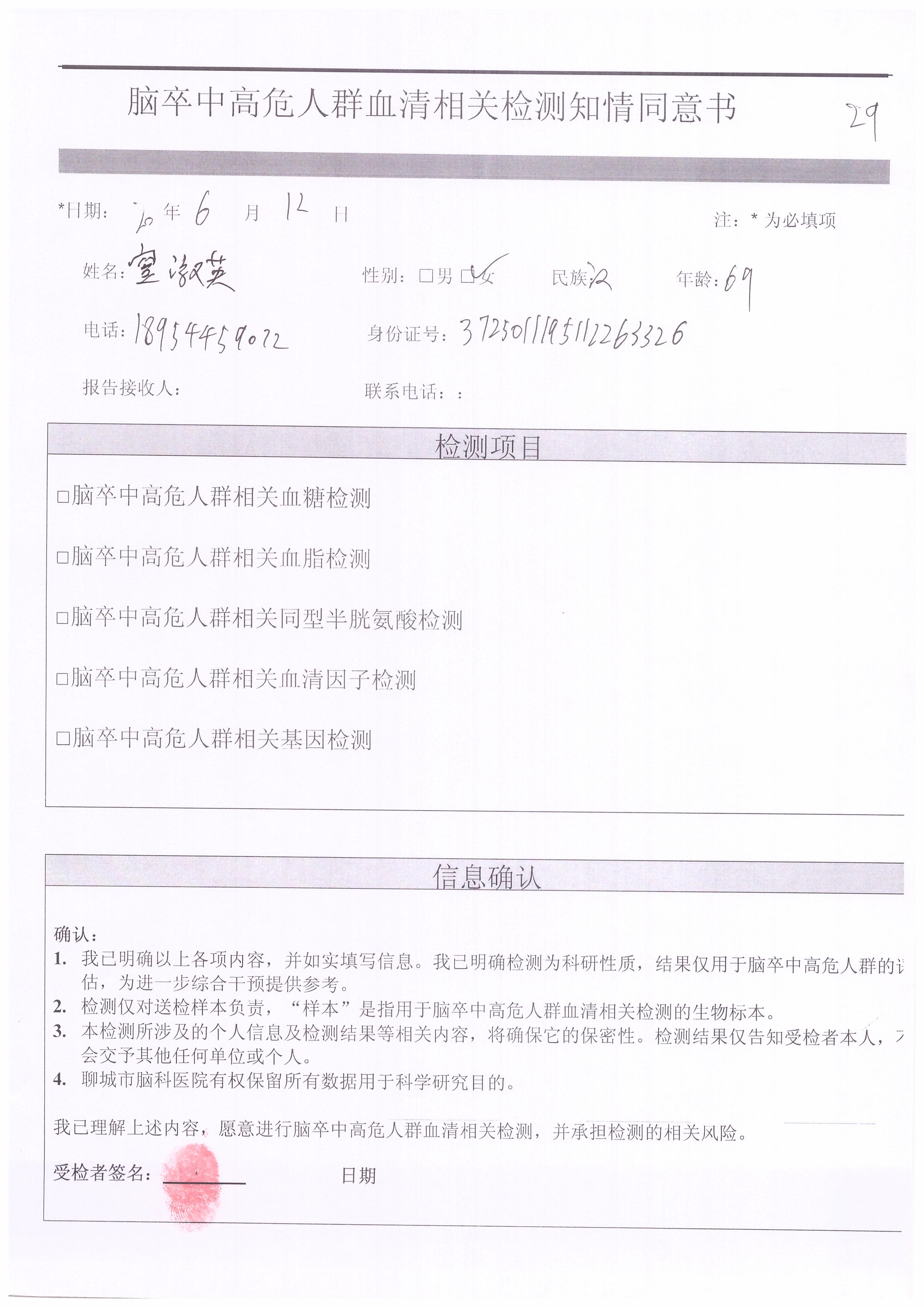

Supplement: Supplementary file 8 — Supplementary file8 (ZIP 23226 KB) [file 10528_2023_10431_MOESM8_ESM.zip › ╓¬╟Θ═1⁄4╥Γ╩Θ6/028.jpg]

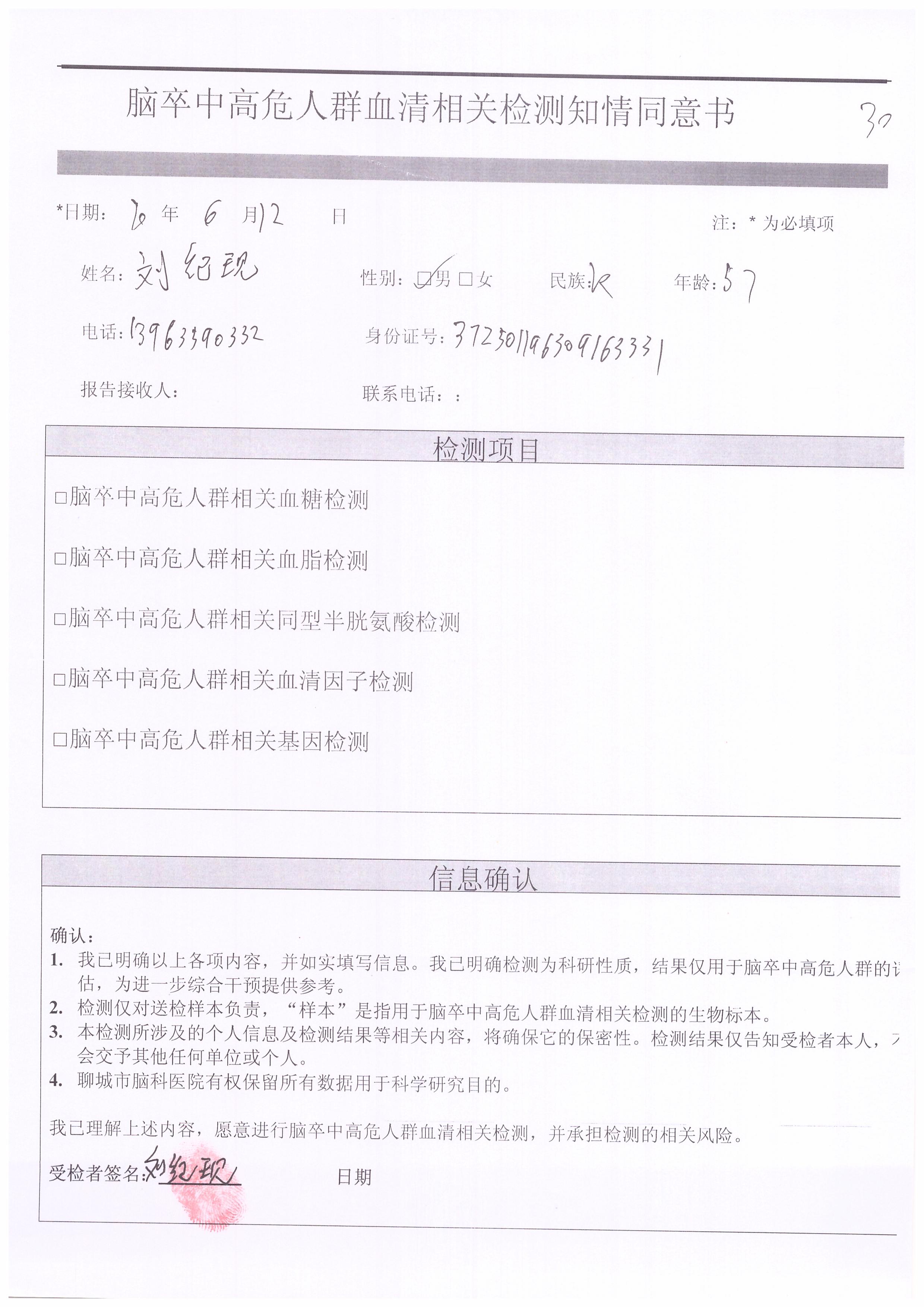

Supplement: Supplementary file 8 — Supplementary file8 (ZIP 23226 KB) [file 10528_2023_10431_MOESM8_ESM.zip › ╓¬╟Θ═1⁄4╥Γ╩Θ6/029.jpg]

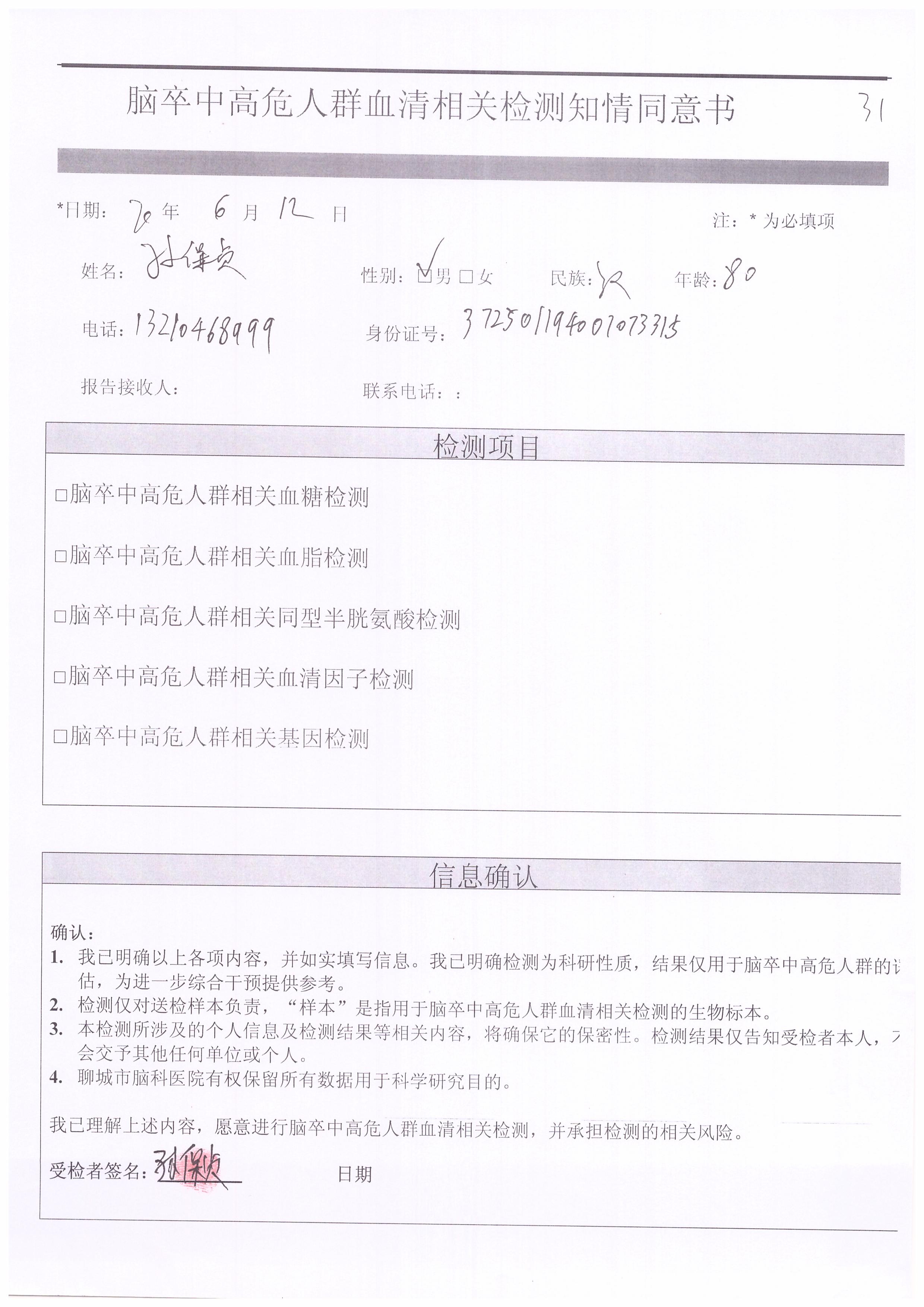

Supplement: Supplementary file 8 — Supplementary file8 (ZIP 23226 KB) [file 10528_2023_10431_MOESM8_ESM.zip › ╓¬╟Θ═1⁄4╥Γ╩Θ6/030.jpg]

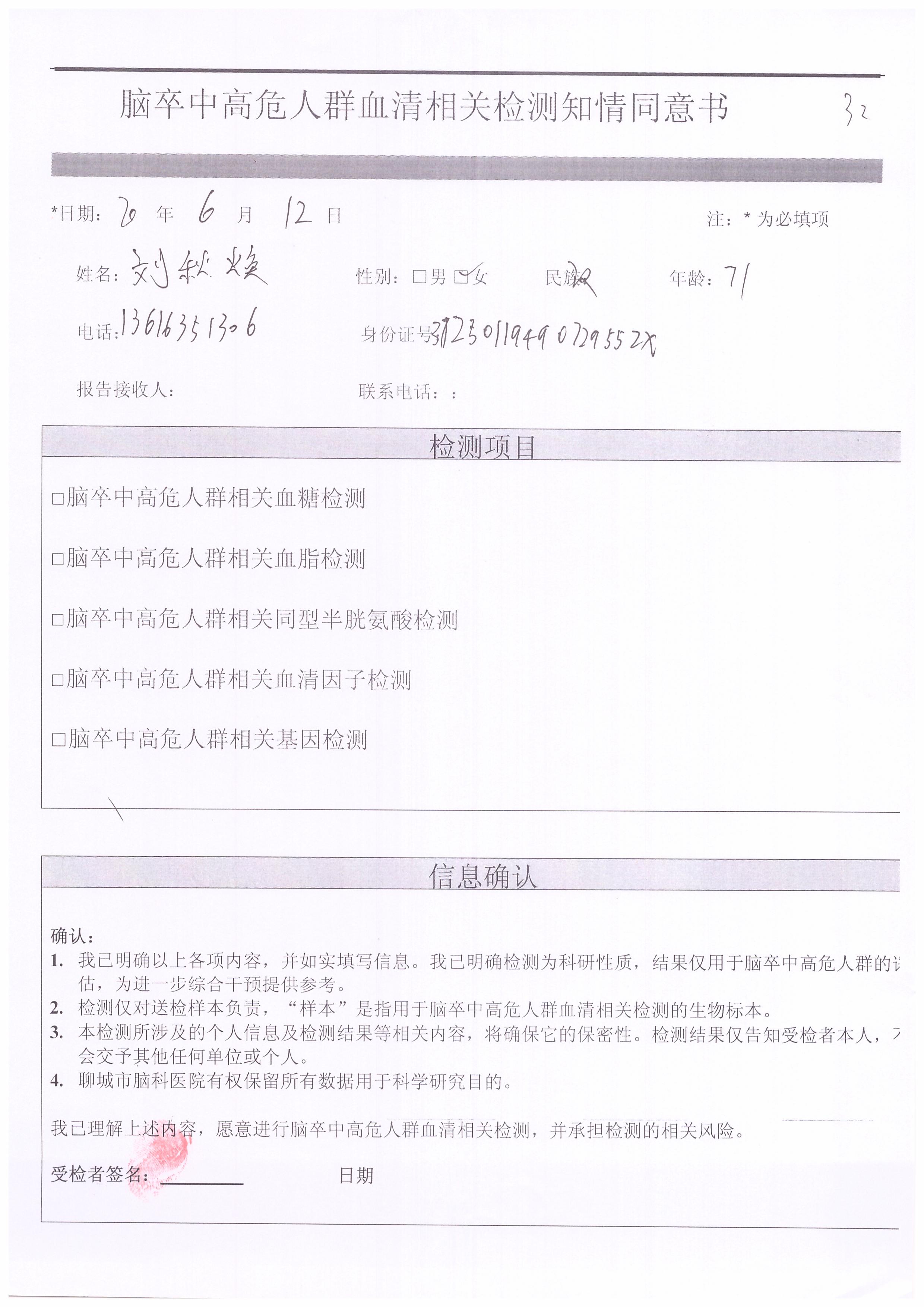

Supplement: Supplementary file 8 — Supplementary file8 (ZIP 23226 KB) [file 10528_2023_10431_MOESM8_ESM.zip › ╓¬╟Θ═1⁄4╥Γ╩Θ6/031.jpg]

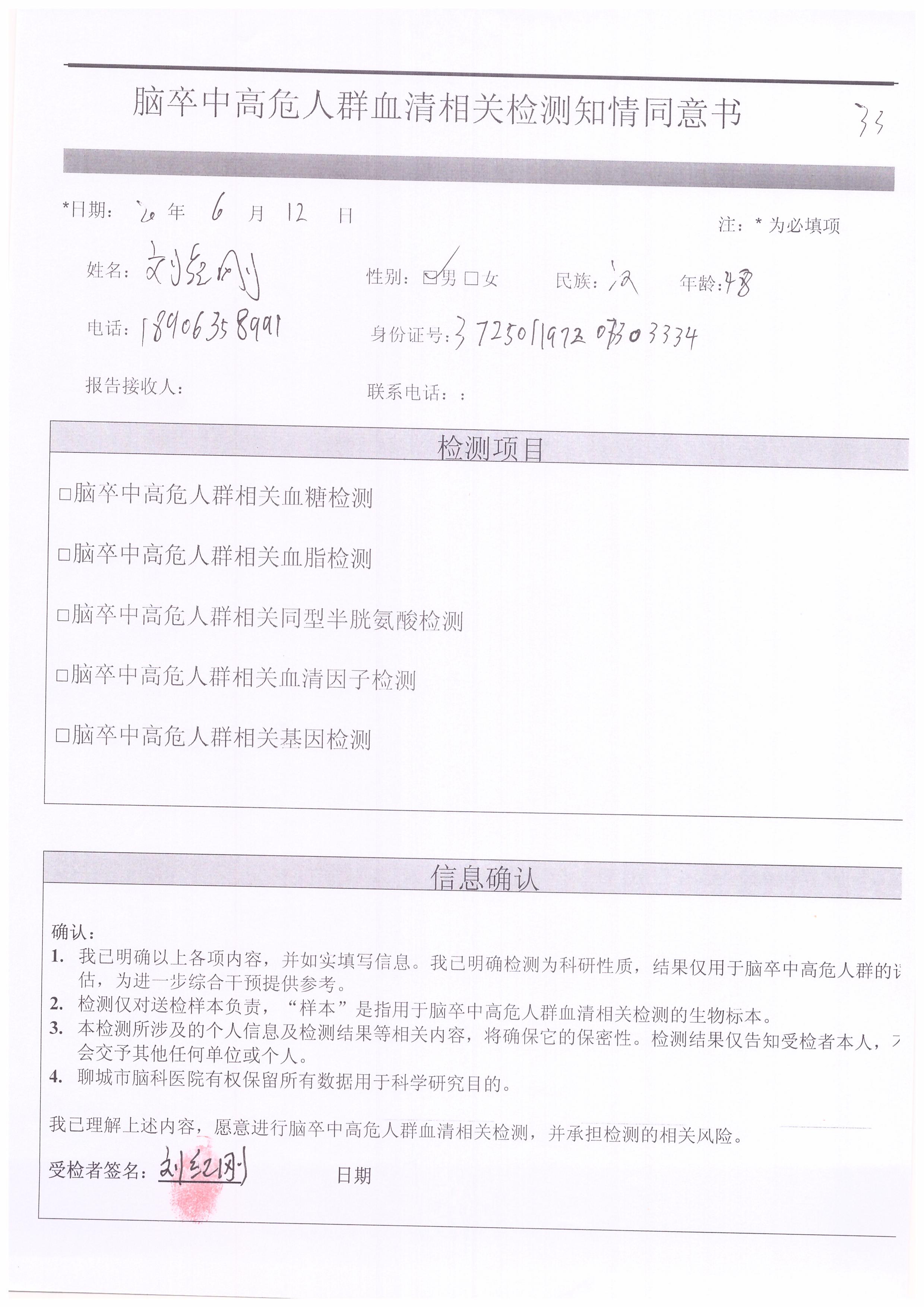

Supplement: Supplementary file 8 — Supplementary file8 (ZIP 23226 KB) [file 10528_2023_10431_MOESM8_ESM.zip › ╓¬╟Θ═1⁄4╥Γ╩Θ6/032.jpg]

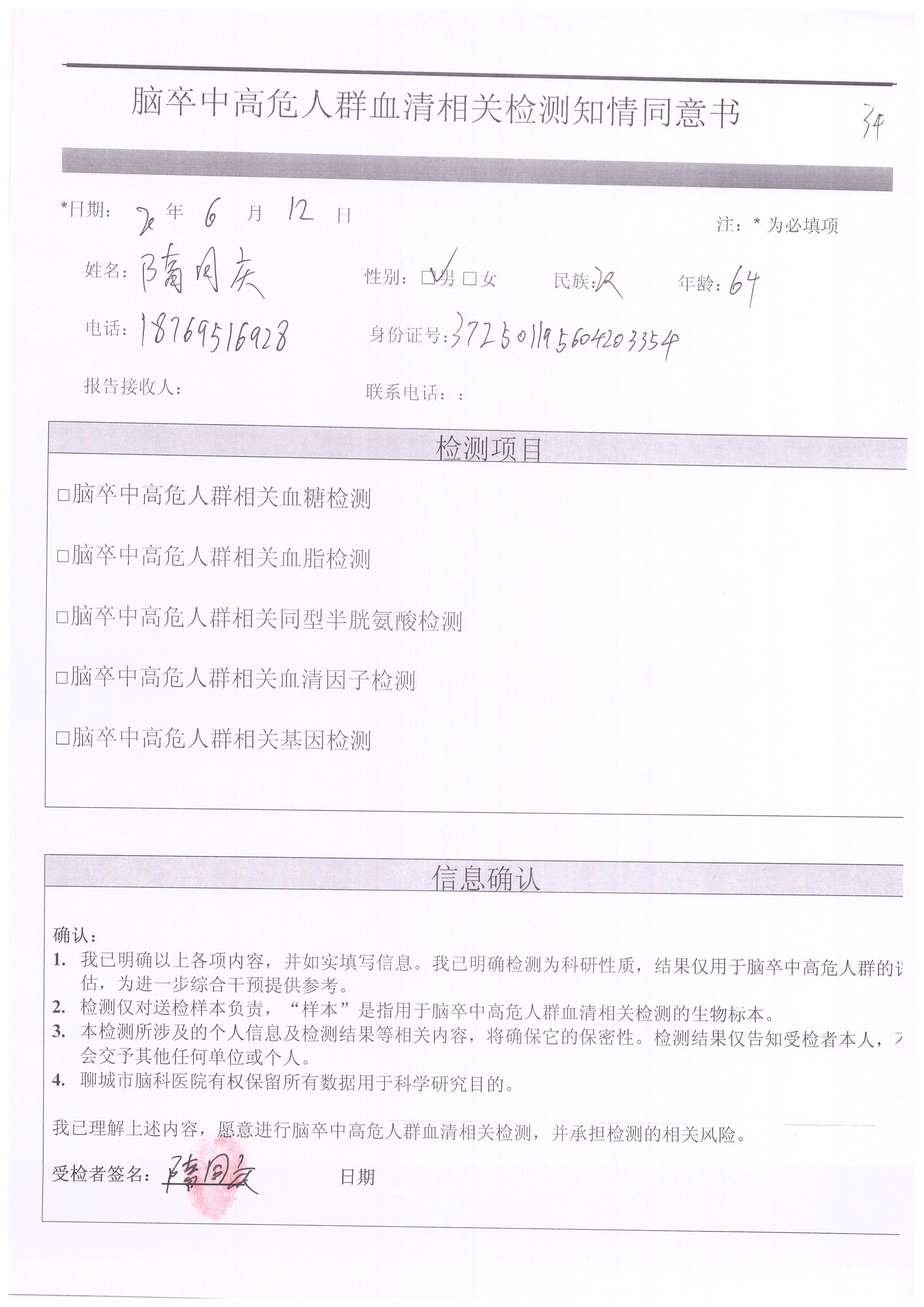

Supplement: Supplementary file 8 — Supplementary file8 (ZIP 23226 KB) [file 10528_2023_10431_MOESM8_ESM.zip › ╓¬╟Θ═1⁄4╥Γ╩Θ6/033.jpg]

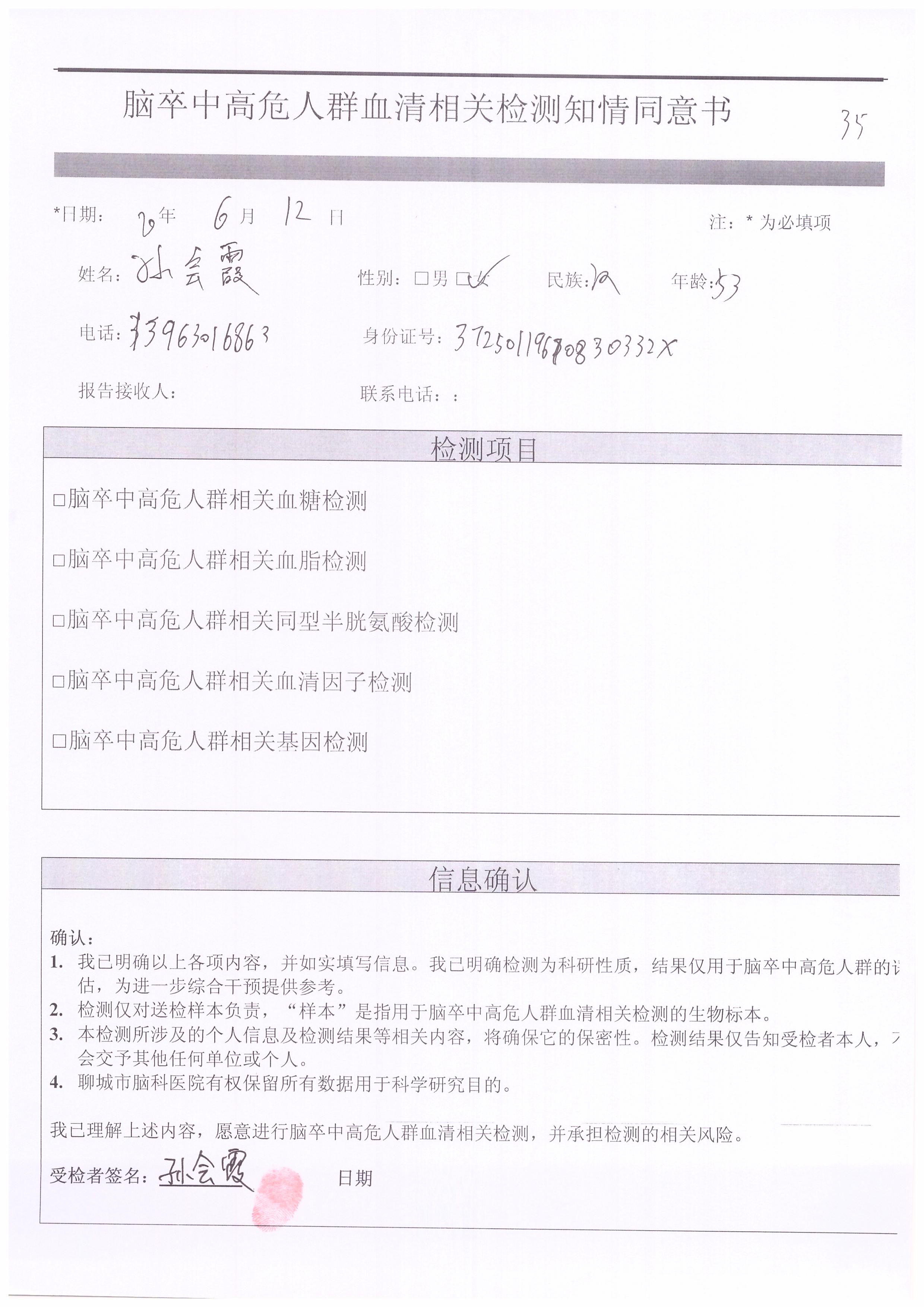

Supplement: Supplementary file 8 — Supplementary file8 (ZIP 23226 KB) [file 10528_2023_10431_MOESM8_ESM.zip › ╓¬╟Θ═1⁄4╥Γ╩Θ6/034.jpg]

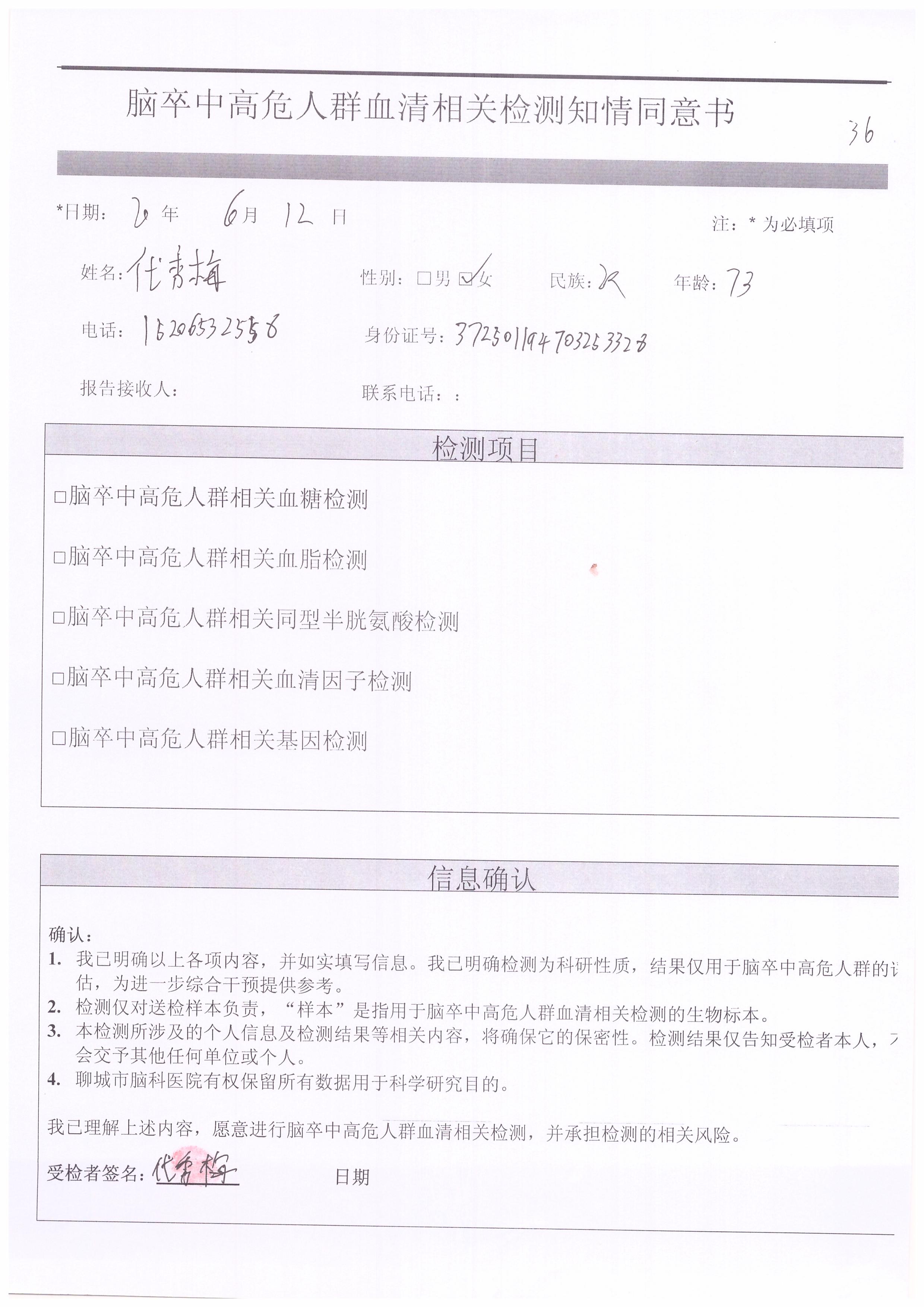

Supplement: Supplementary file 8 — Supplementary file8 (ZIP 23226 KB) [file 10528_2023_10431_MOESM8_ESM.zip › ╓¬╟Θ═1⁄4╥Γ╩Θ6/035.jpg]

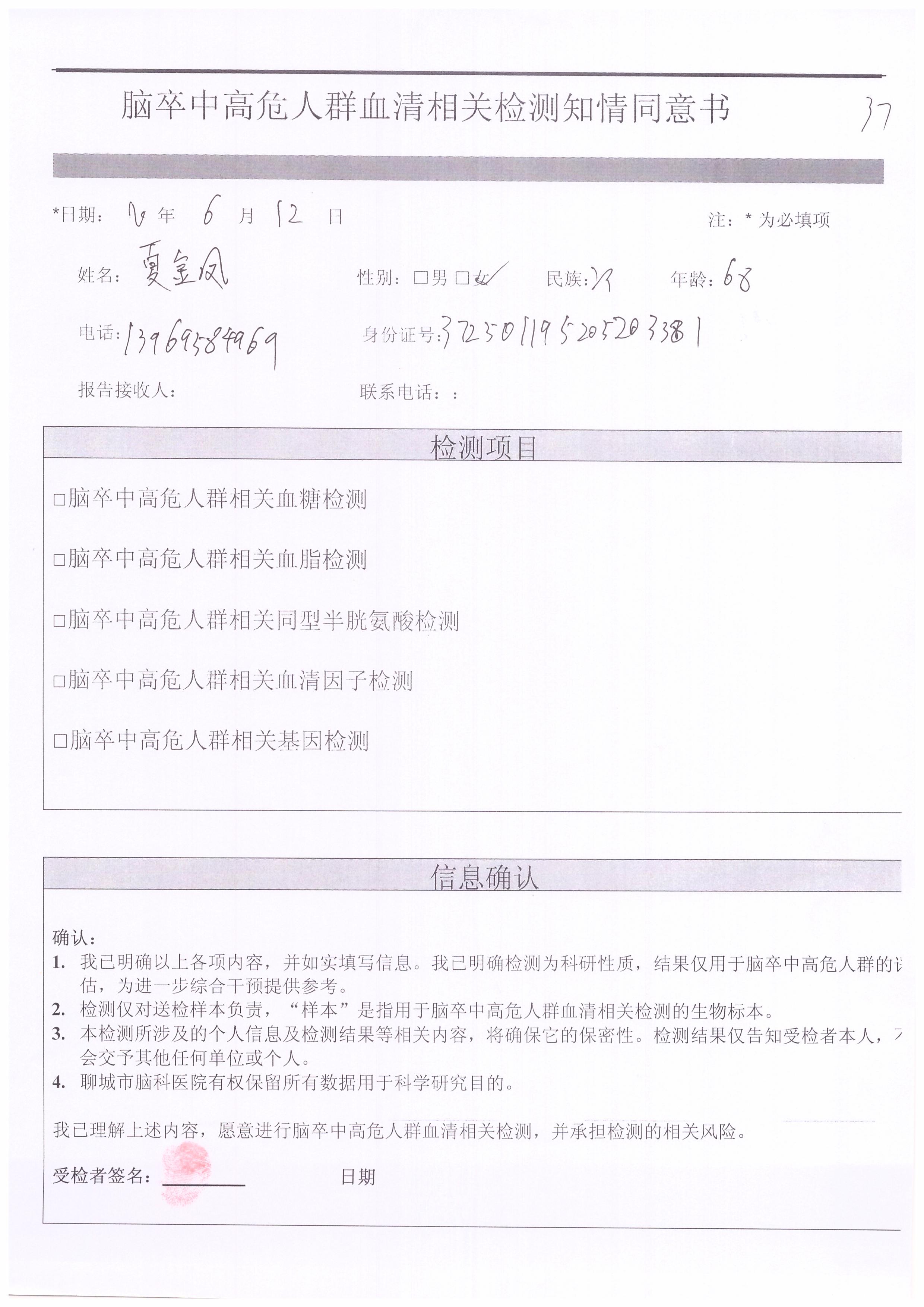

Supplement: Supplementary file 8 — Supplementary file8 (ZIP 23226 KB) [file 10528_2023_10431_MOESM8_ESM.zip › ╓¬╟Θ═1⁄4╥Γ╩Θ6/036.jpg]

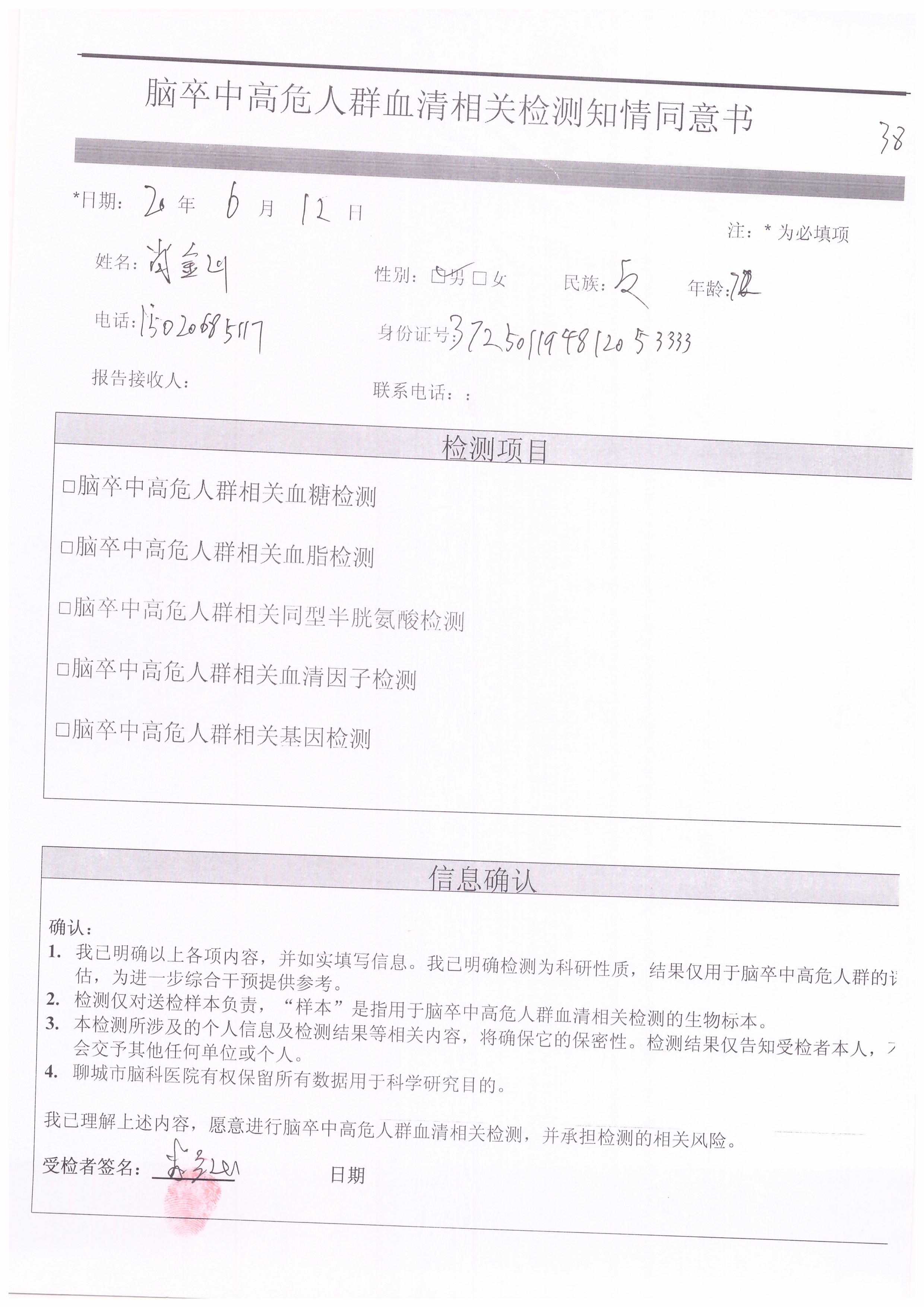

Supplement: Supplementary file 8 — Supplementary file8 (ZIP 23226 KB) [file 10528_2023_10431_MOESM8_ESM.zip › ╓¬╟Θ═1⁄4╥Γ╩Θ6/037.jpg]

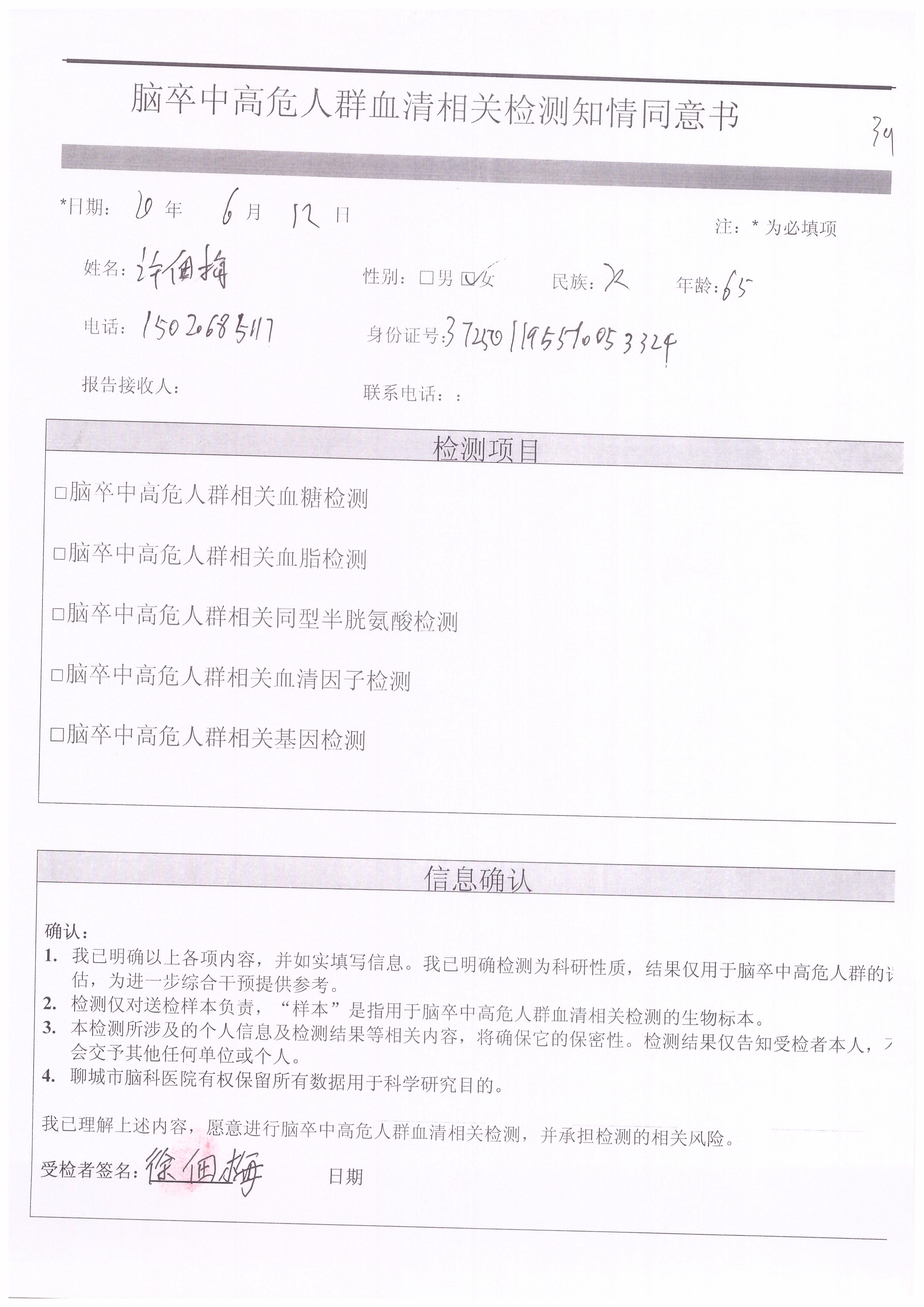

Supplement: Supplementary file 8 — Supplementary file8 (ZIP 23226 KB) [file 10528_2023_10431_MOESM8_ESM.zip › ╓¬╟Θ═1⁄4╥Γ╩Θ6/038.jpg]

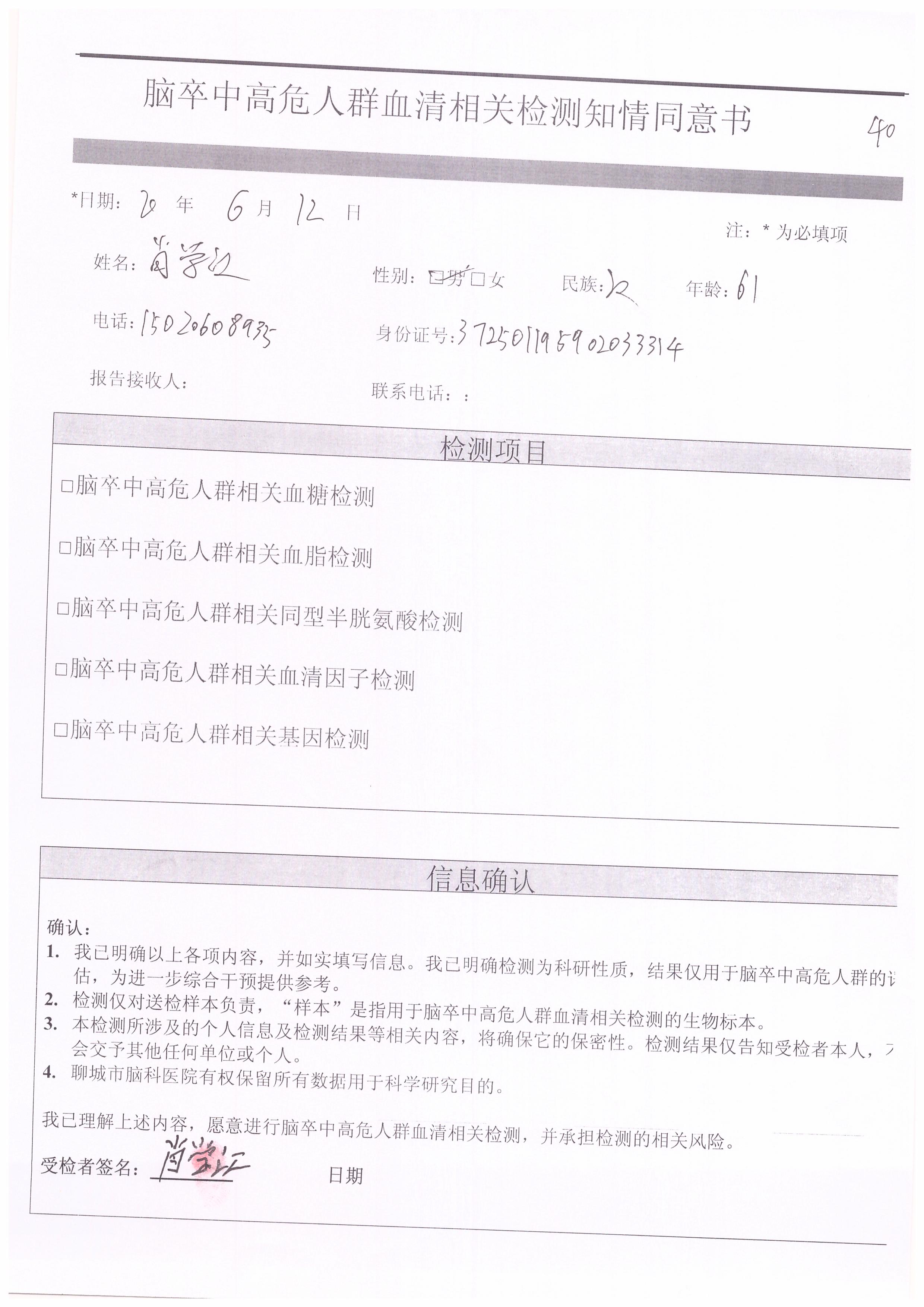

Supplement: Supplementary file 8 — Supplementary file8 (ZIP 23226 KB) [file 10528_2023_10431_MOESM8_ESM.zip › ╓¬╟Θ═1⁄4╥Γ╩Θ6/039.jpg]

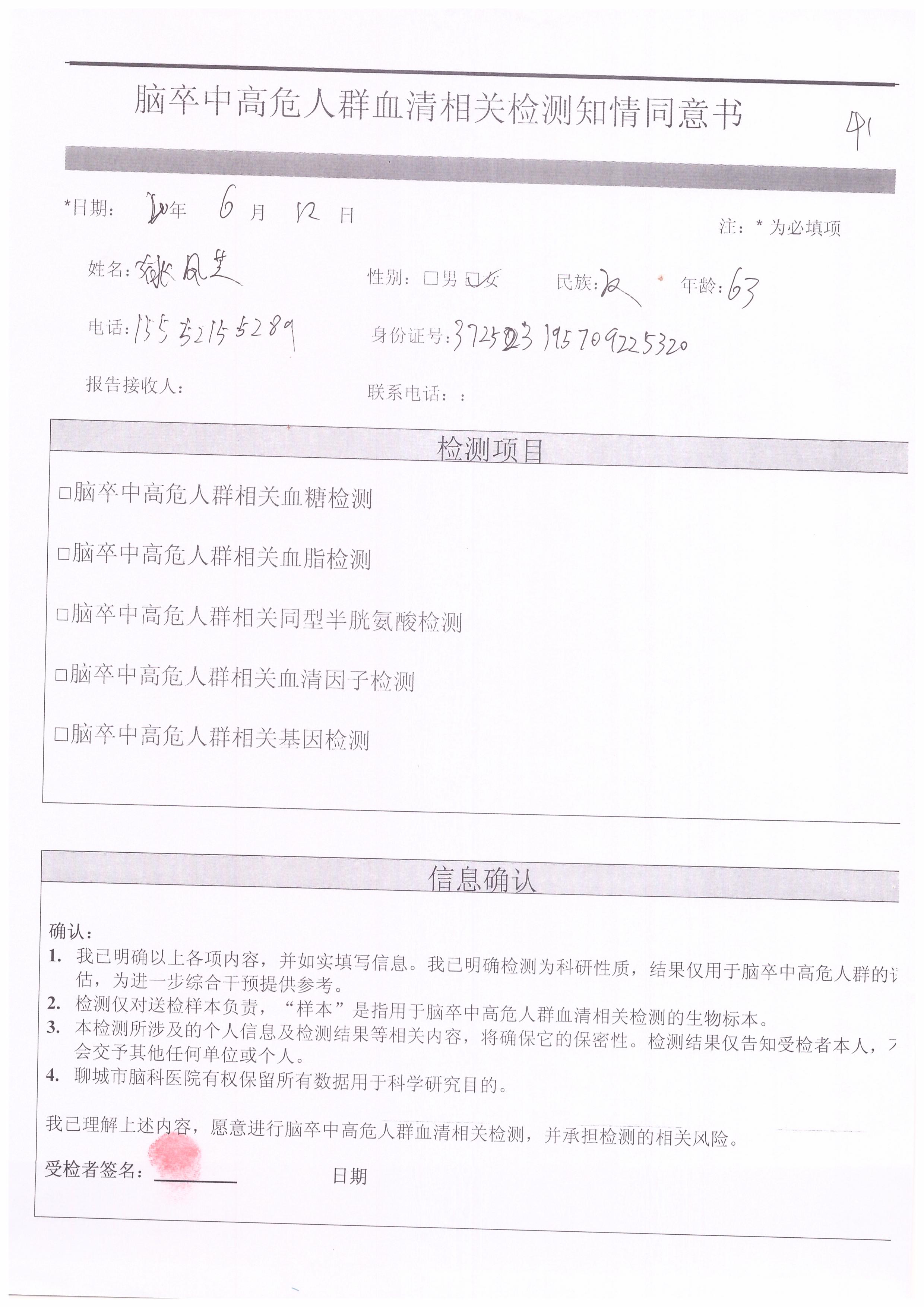

Supplement: Supplementary file 8 — Supplementary file8 (ZIP 23226 KB) [file 10528_2023_10431_MOESM8_ESM.zip › ╓¬╟Θ═1⁄4╥Γ╩Θ6/040.jpg]

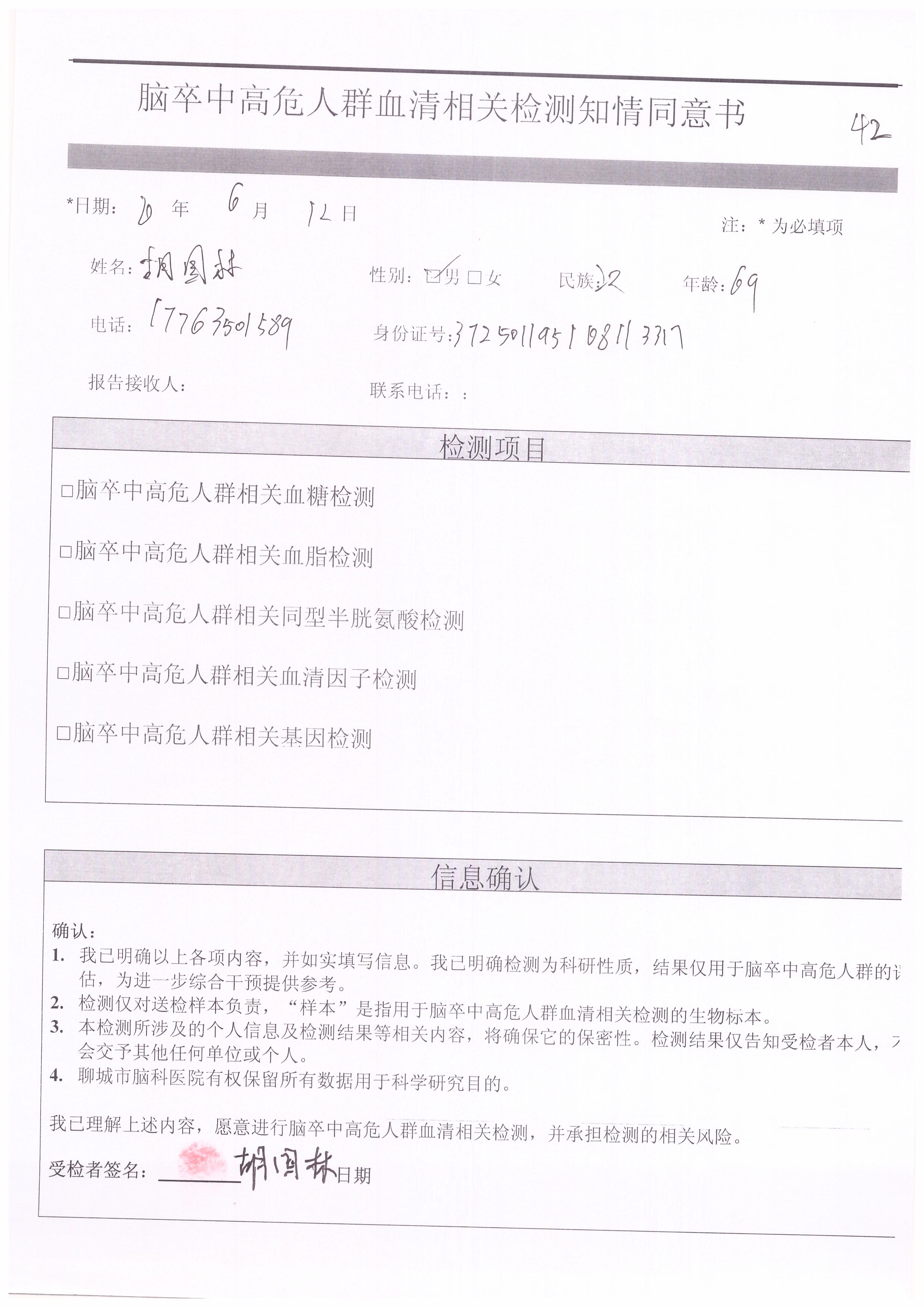

Supplement: Supplementary file 8 — Supplementary file8 (ZIP 23226 KB) [file 10528_2023_10431_MOESM8_ESM.zip › ╓¬╟Θ═1⁄4╥Γ╩Θ6/041.jpg]

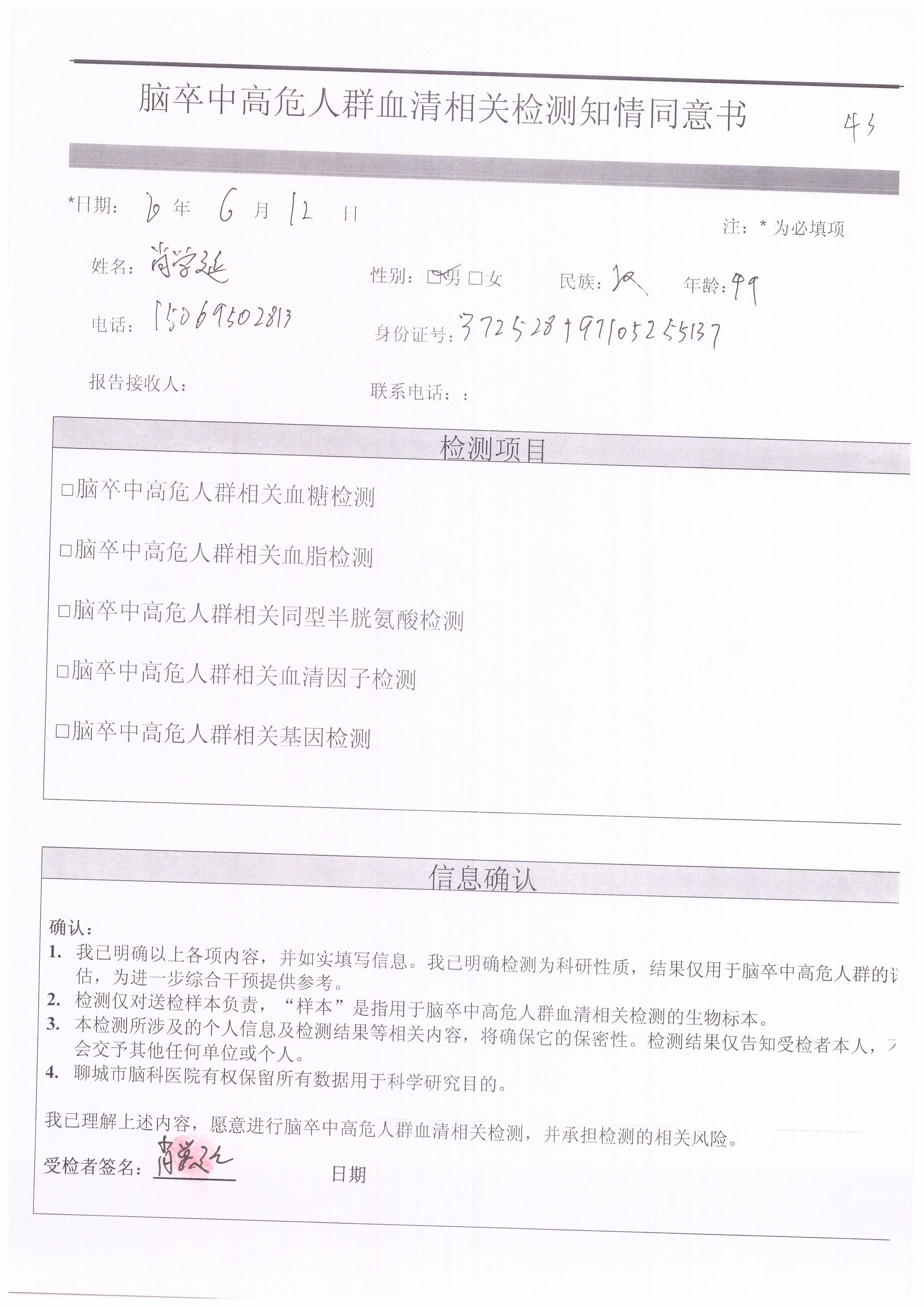

Supplement: Supplementary file 8 — Supplementary file8 (ZIP 23226 KB) [file 10528_2023_10431_MOESM8_ESM.zip › ╓¬╟Θ═1⁄4╥Γ╩Θ6/042.jpg]

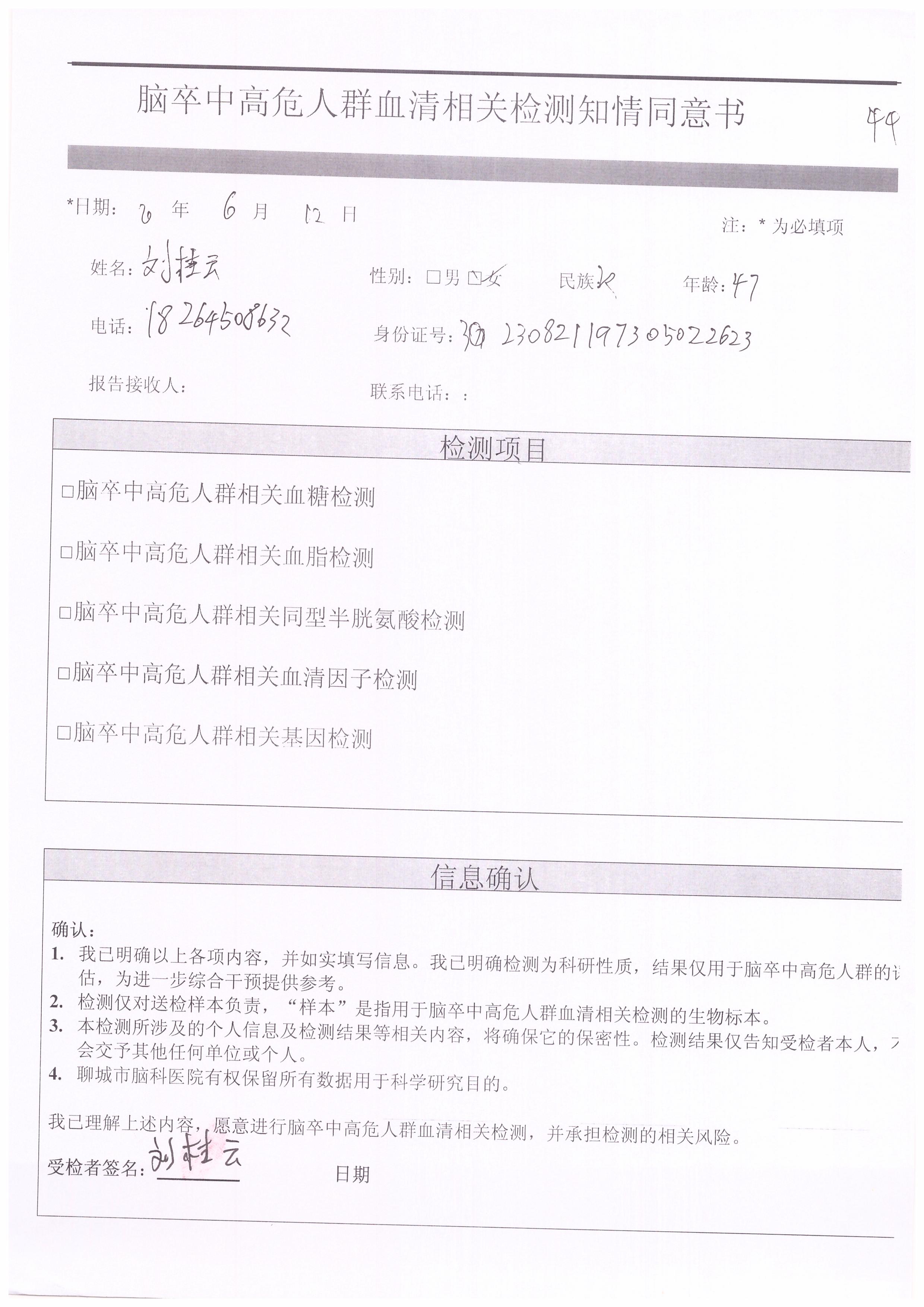

Supplement: Supplementary file 8 — Supplementary file8 (ZIP 23226 KB) [file 10528_2023_10431_MOESM8_ESM.zip › ╓¬╟Θ═1⁄4╥Γ╩Θ6/043.jpg]

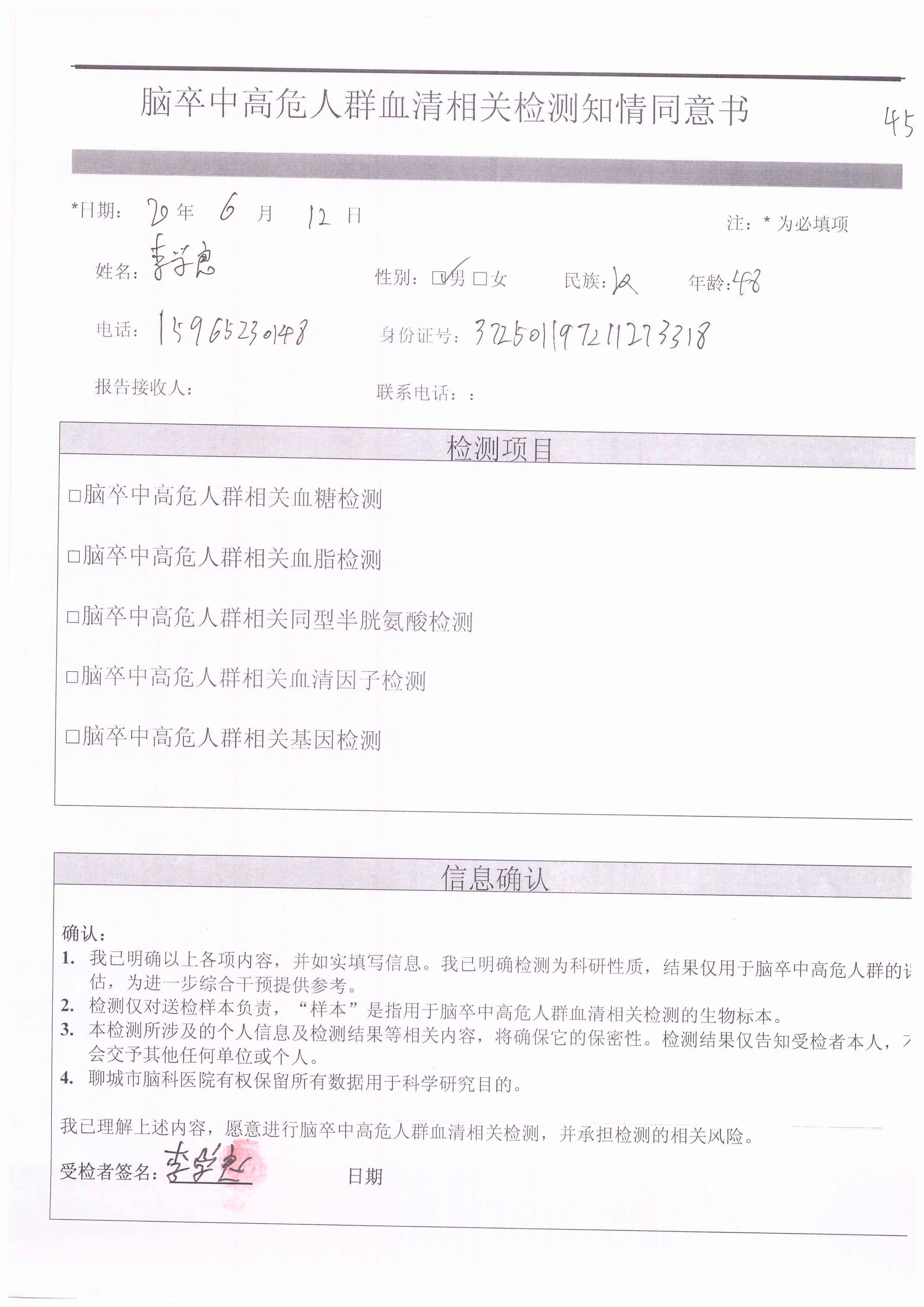

Supplement: Supplementary file 8 — Supplementary file8 (ZIP 23226 KB) [file 10528_2023_10431_MOESM8_ESM.zip › ╓¬╟Θ═1⁄4╥Γ╩Θ6/044.jpg]

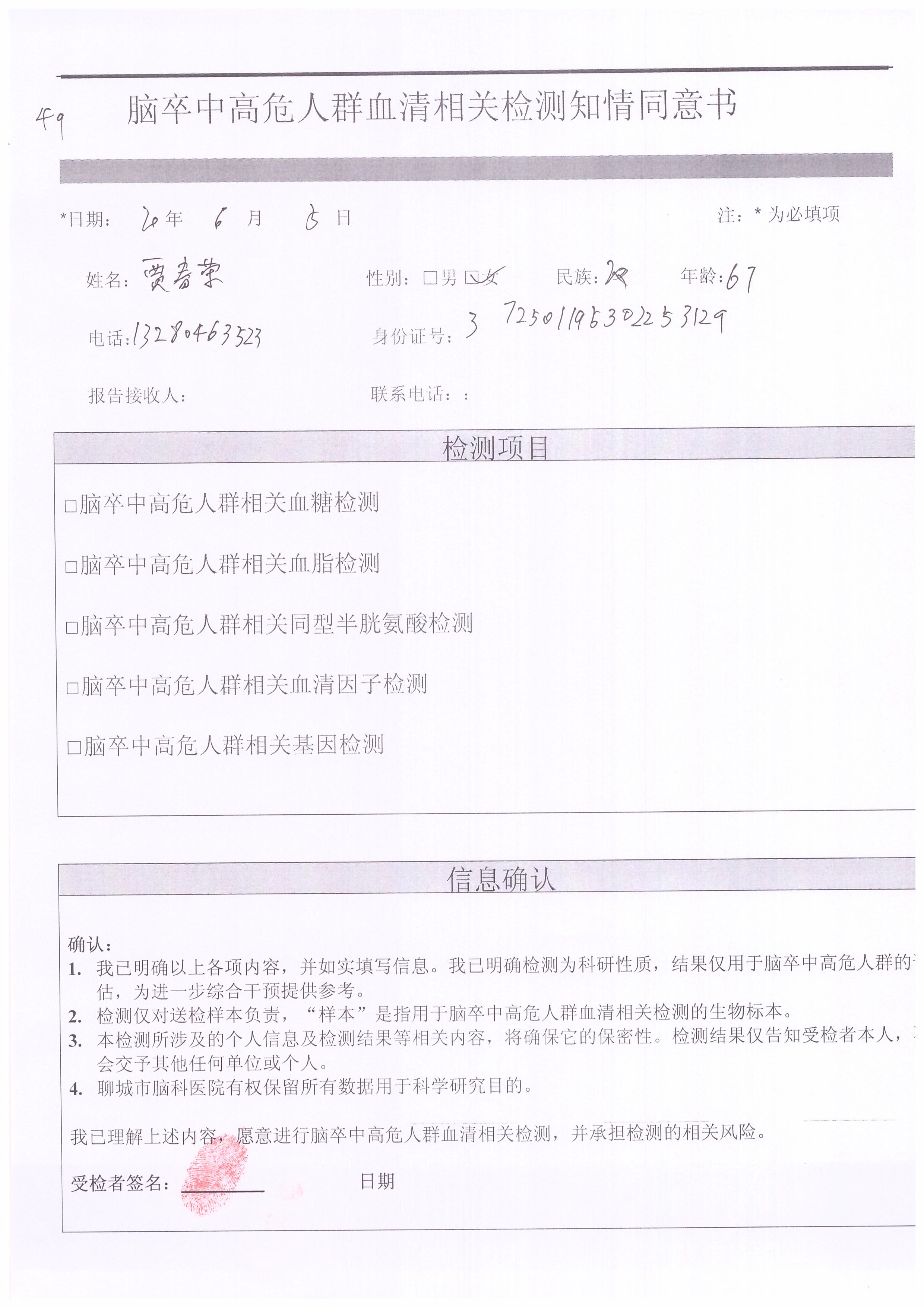

Supplement: Supplementary file 9 — Supplementary file9 (ZIP 24580 KB) [file 10528_2023_10431_MOESM9_ESM.zip › ╓¬╟Θ═1⁄4╥Γ╩Θ7/╡┌2▓┐╖╓/004.jpg]

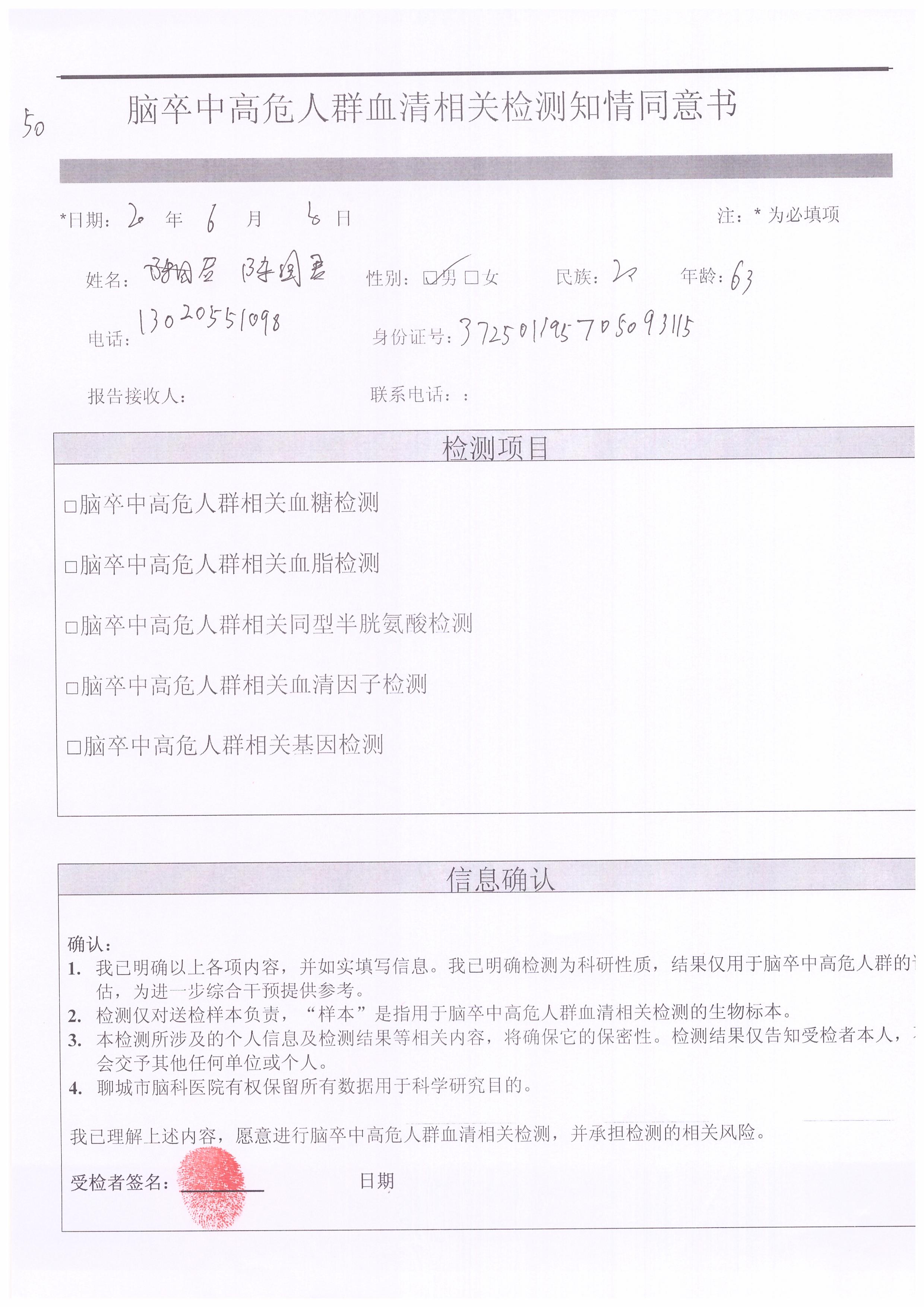

Supplement: Supplementary file 9 — Supplementary file9 (ZIP 24580 KB) [file 10528_2023_10431_MOESM9_ESM.zip › ╓¬╟Θ═1⁄4╥Γ╩Θ7/╡┌2▓┐╖╓/005.jpg]

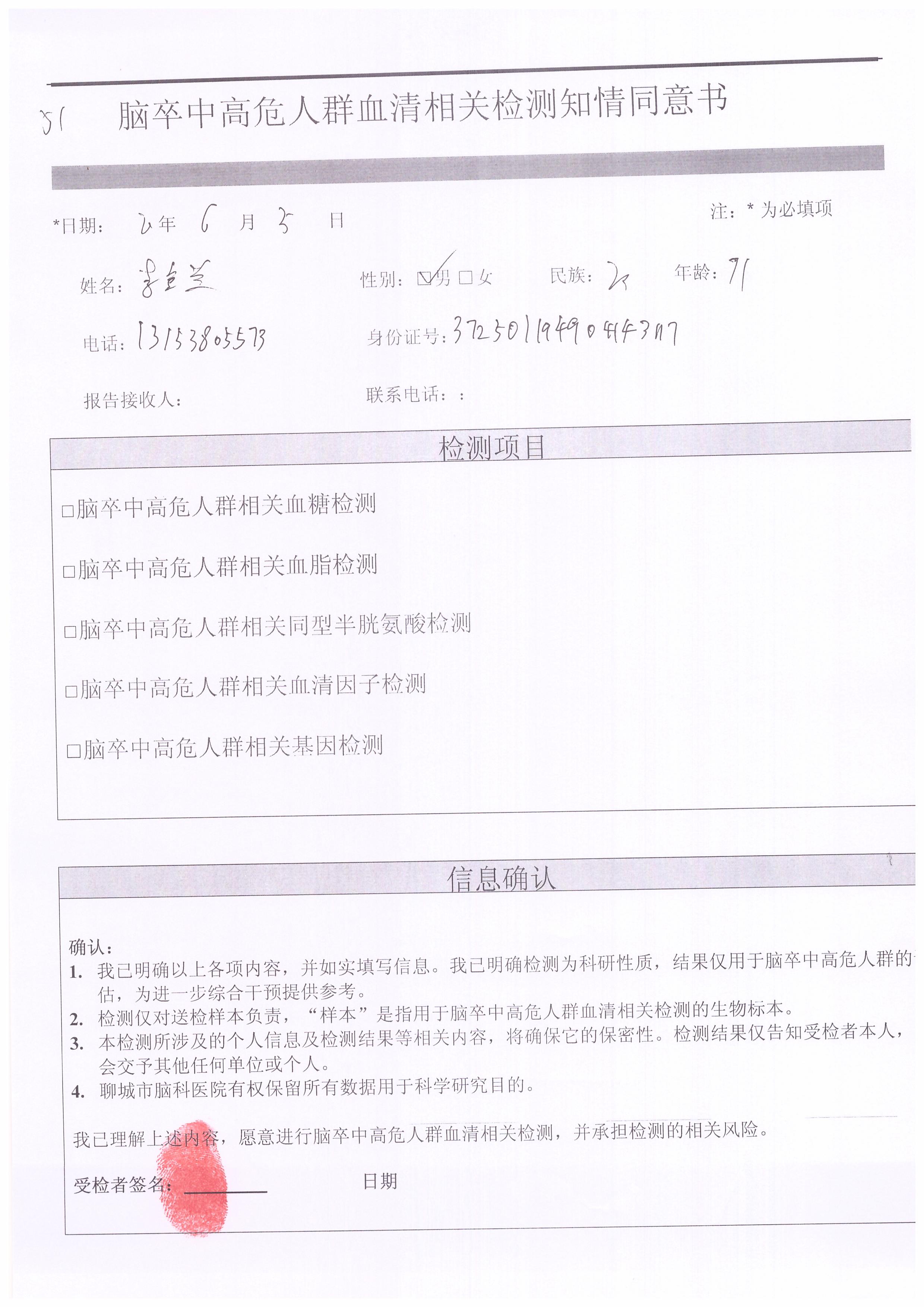

Supplement: Supplementary file 9 — Supplementary file9 (ZIP 24580 KB) [file 10528_2023_10431_MOESM9_ESM.zip › ╓¬╟Θ═1⁄4╥Γ╩Θ7/╡┌2▓┐╖╓/006.jpg]

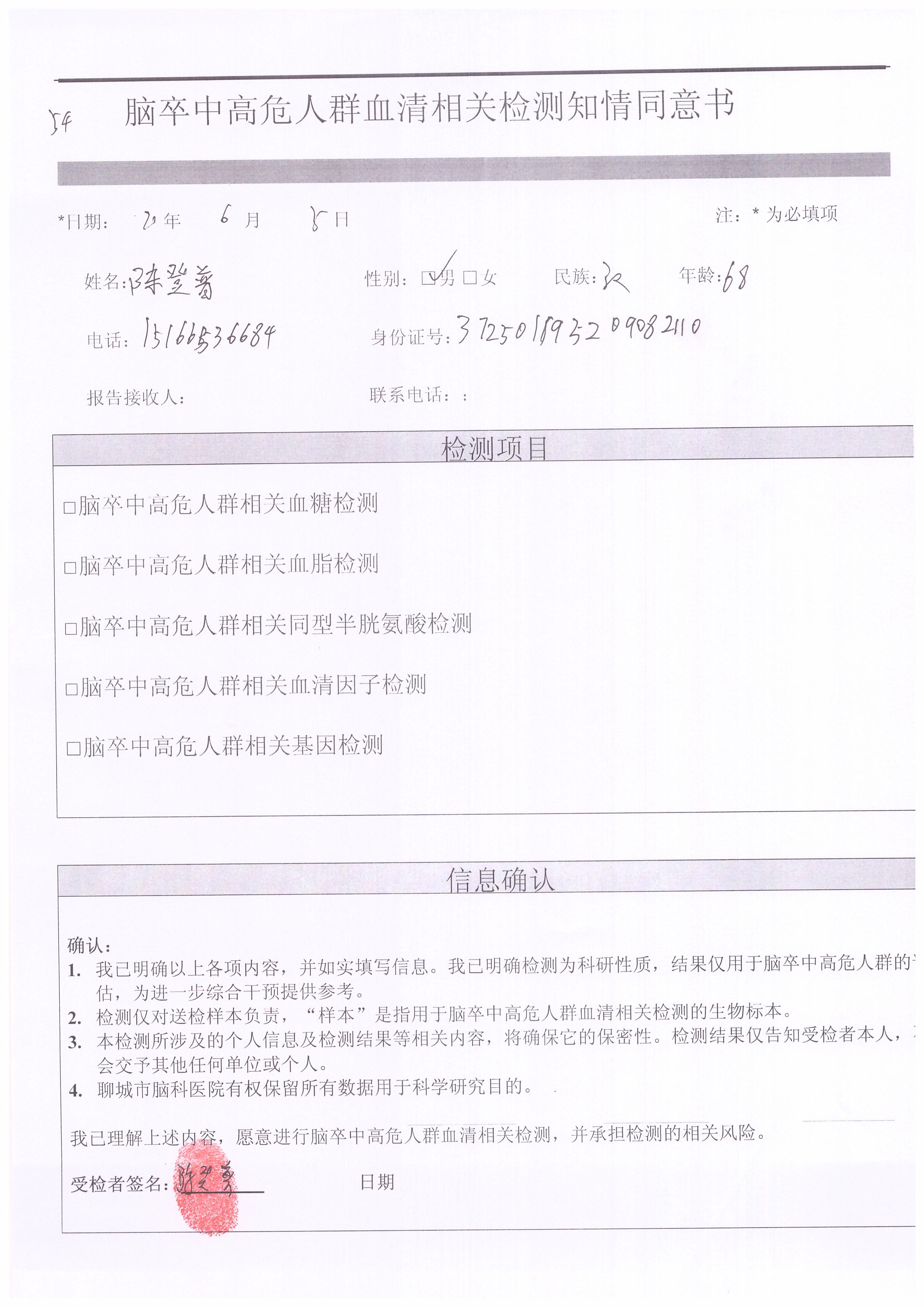

Supplement: Supplementary file 9 — Supplementary file9 (ZIP 24580 KB) [file 10528_2023_10431_MOESM9_ESM.zip › ╓¬╟Θ═1⁄4╥Γ╩Θ7/╡┌2▓┐╖╓/009.jpg]

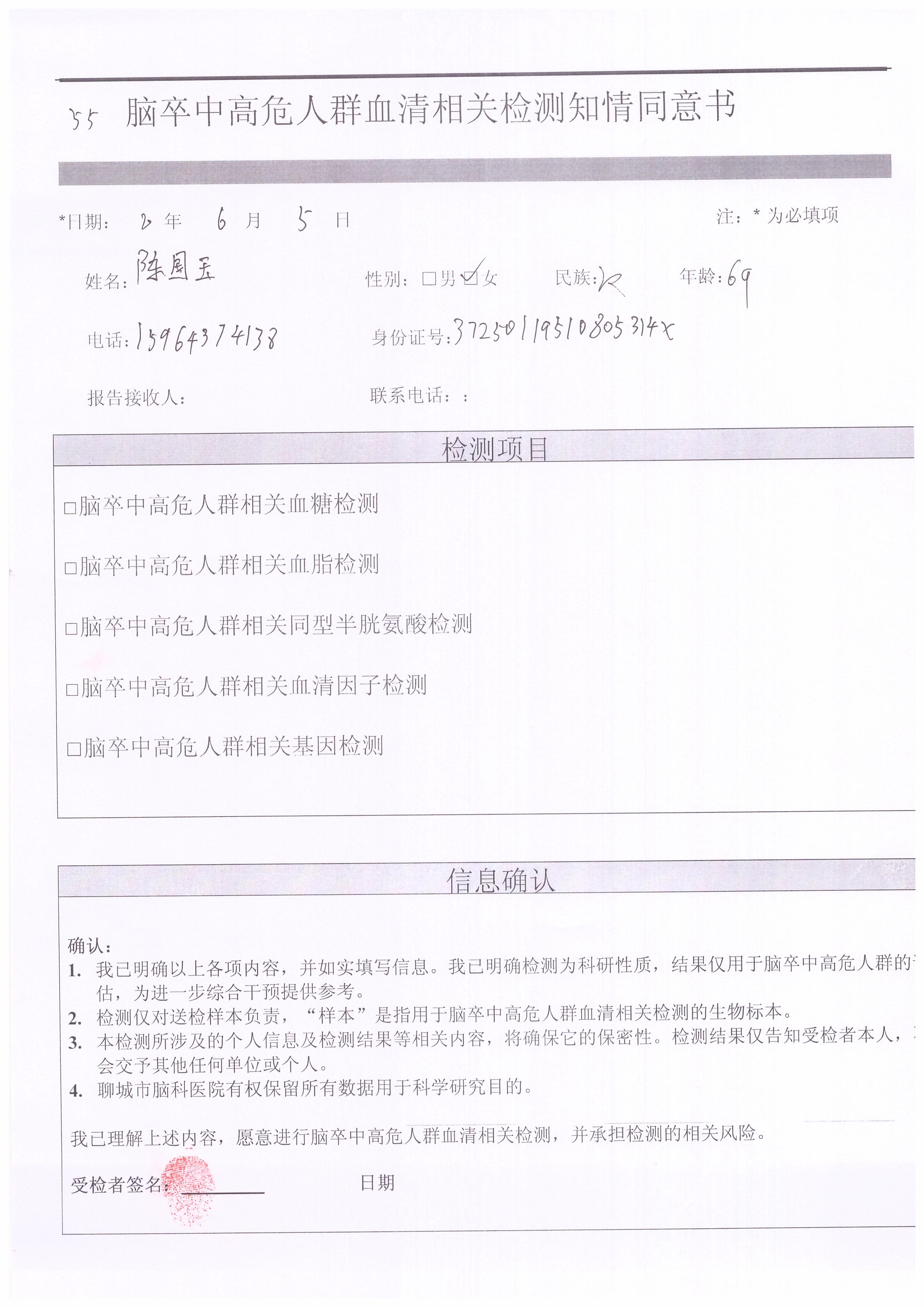

Supplement: Supplementary file 9 — Supplementary file9 (ZIP 24580 KB) [file 10528_2023_10431_MOESM9_ESM.zip › ╓¬╟Θ═1⁄4╥Γ╩Θ7/╡┌2▓┐╖╓/010.jpg]

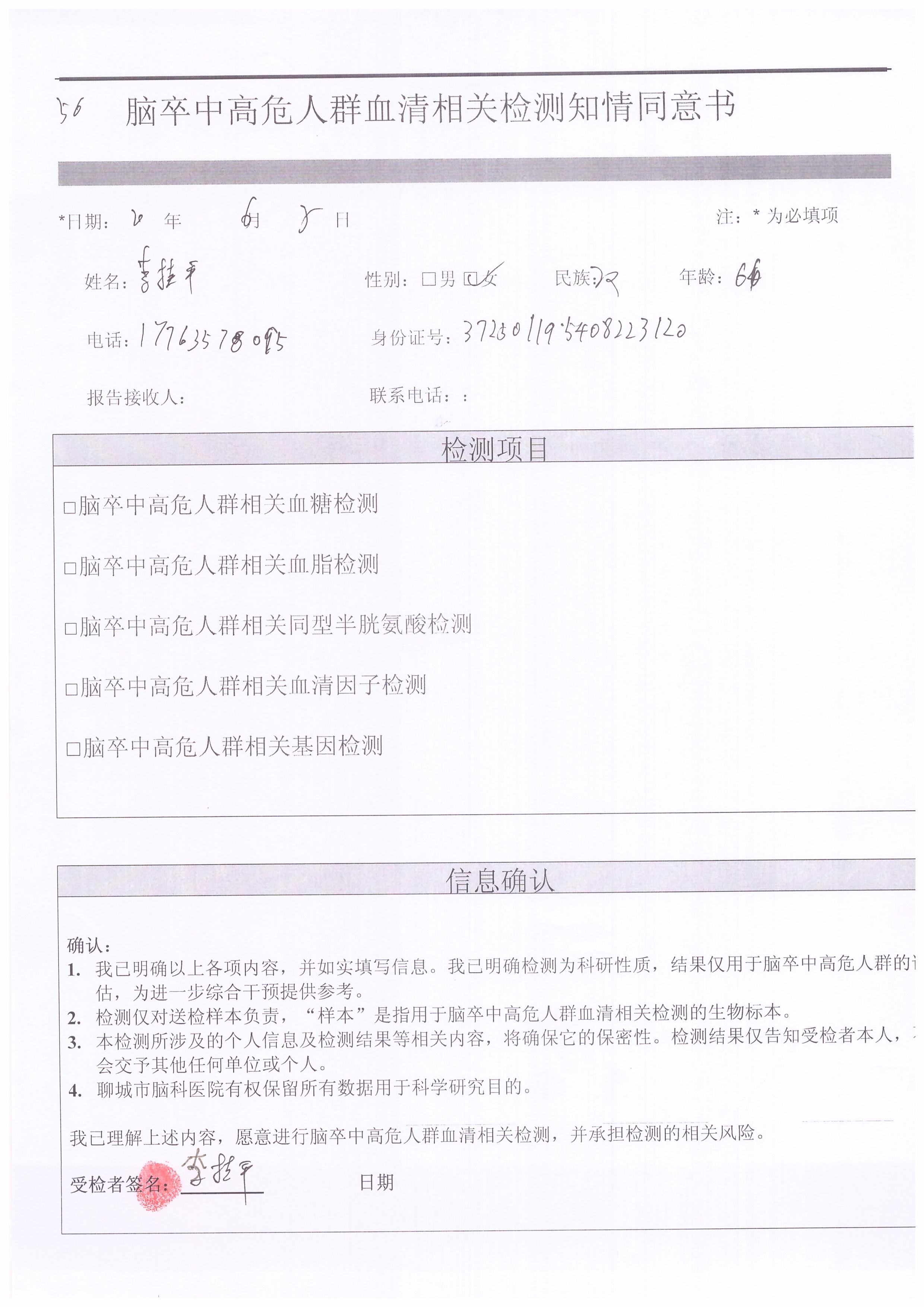

Supplement: Supplementary file 9 — Supplementary file9 (ZIP 24580 KB) [file 10528_2023_10431_MOESM9_ESM.zip › ╓¬╟Θ═1⁄4╥Γ╩Θ7/╡┌2▓┐╖╓/011.jpg]

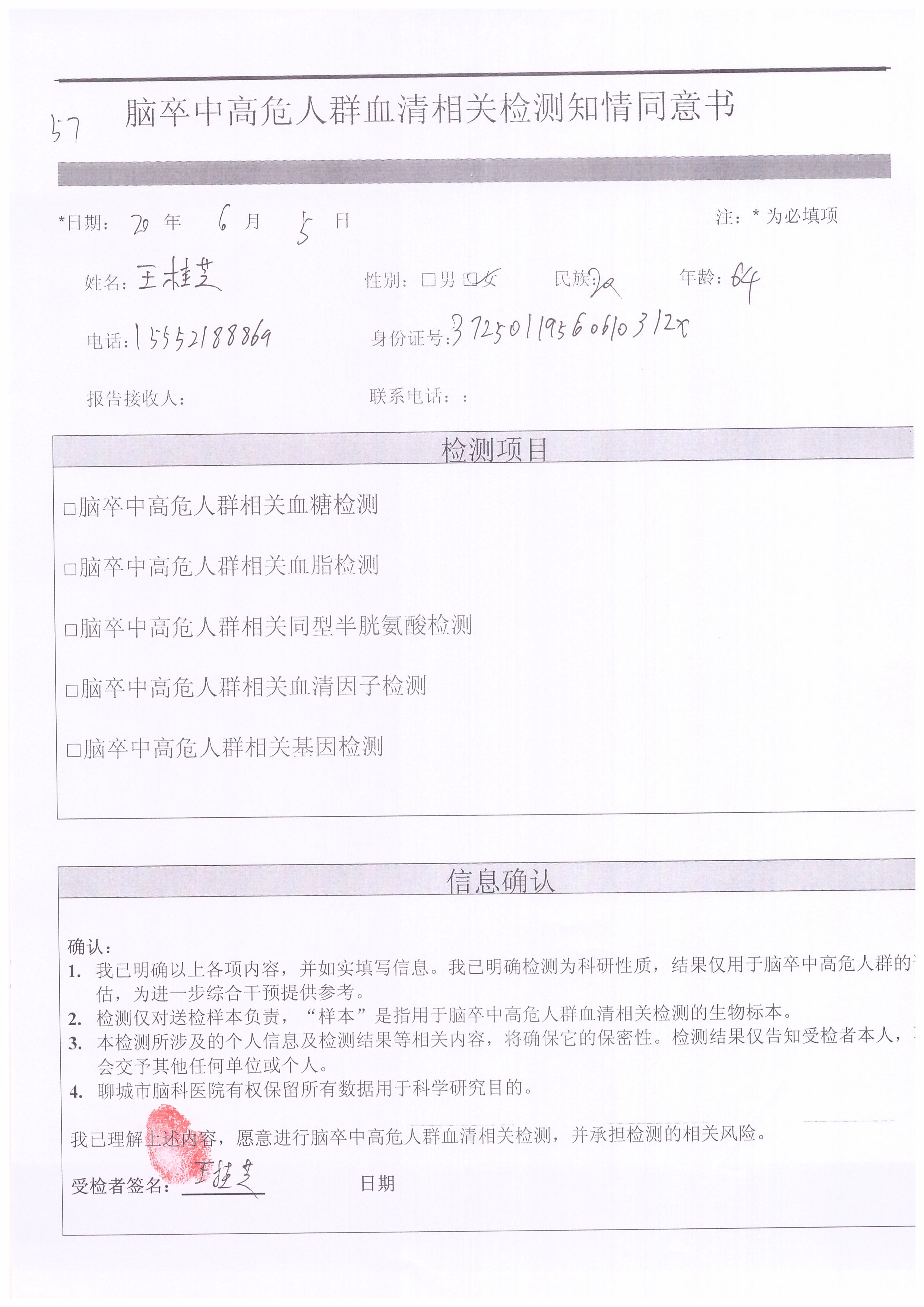

Supplement: Supplementary file 9 — Supplementary file9 (ZIP 24580 KB) [file 10528_2023_10431_MOESM9_ESM.zip › ╓¬╟Θ═1⁄4╥Γ╩Θ7/╡┌2▓┐╖╓/012.jpg]

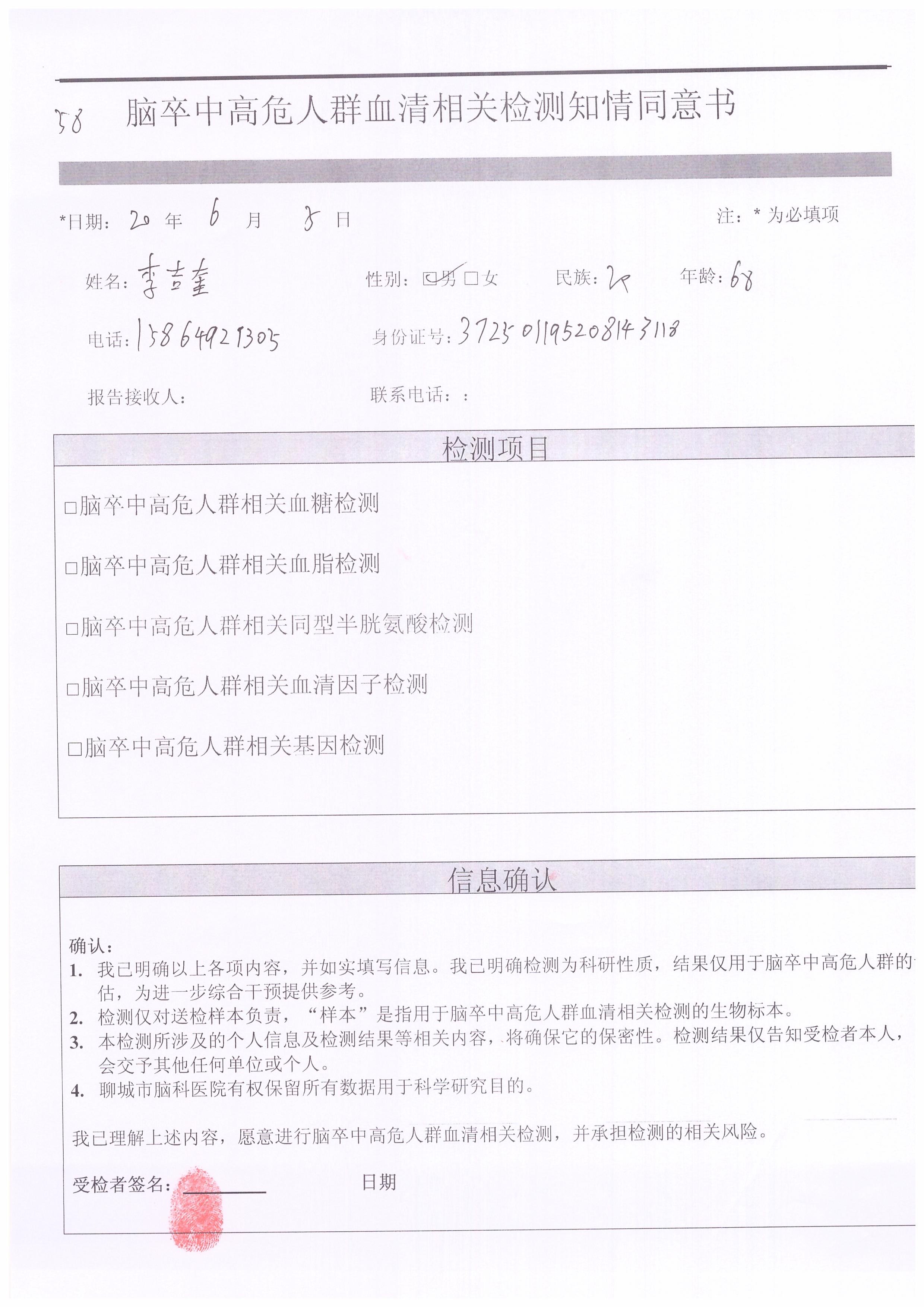

Supplement: Supplementary file 9 — Supplementary file9 (ZIP 24580 KB) [file 10528_2023_10431_MOESM9_ESM.zip › ╓¬╟Θ═1⁄4╥Γ╩Θ7/╡┌2▓┐╖╓/013.jpg]

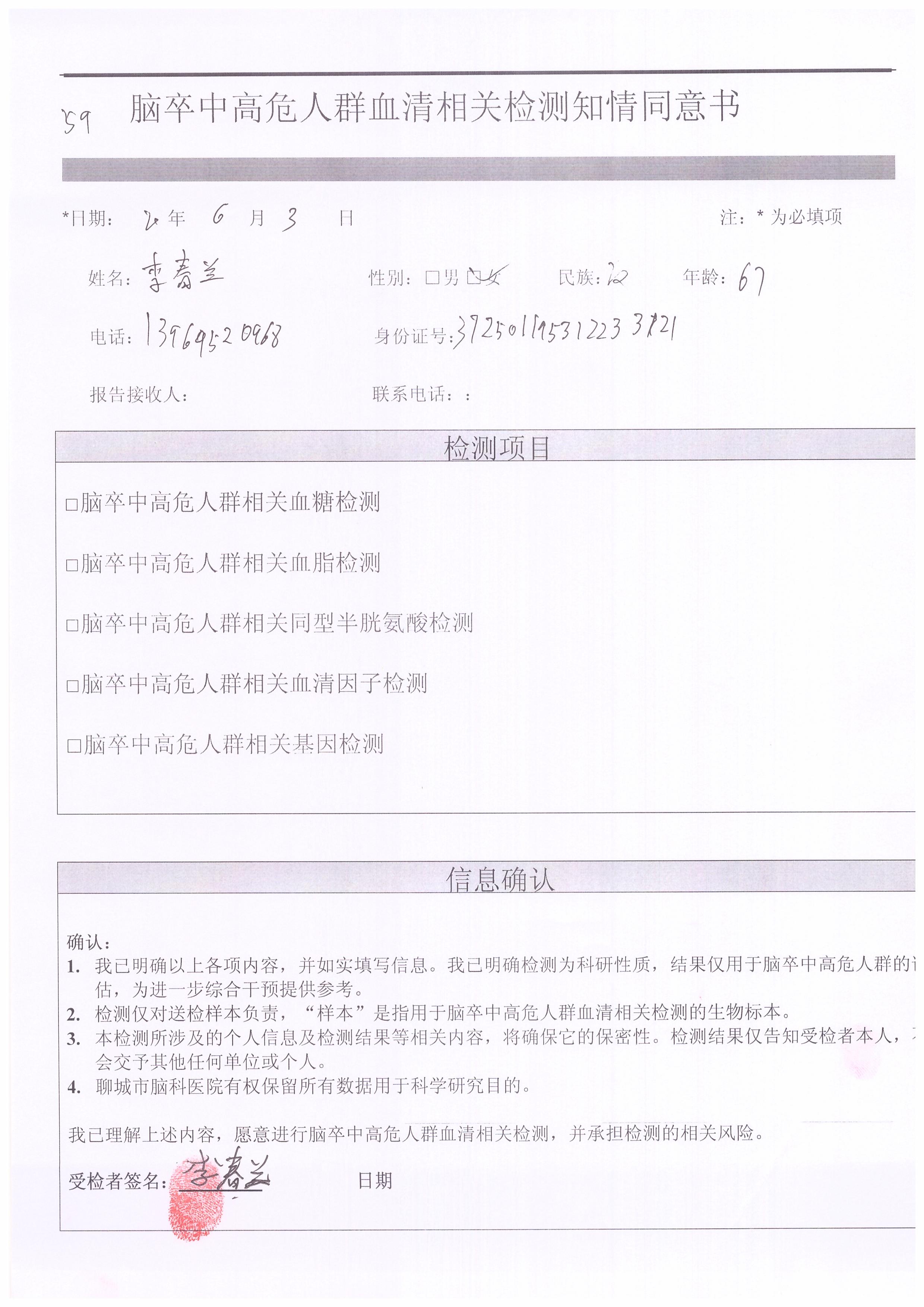

Supplement: Supplementary file 9 — Supplementary file9 (ZIP 24580 KB) [file 10528_2023_10431_MOESM9_ESM.zip › ╓¬╟Θ═1⁄4╥Γ╩Θ7/╡┌2▓┐╖╓/014.jpg]

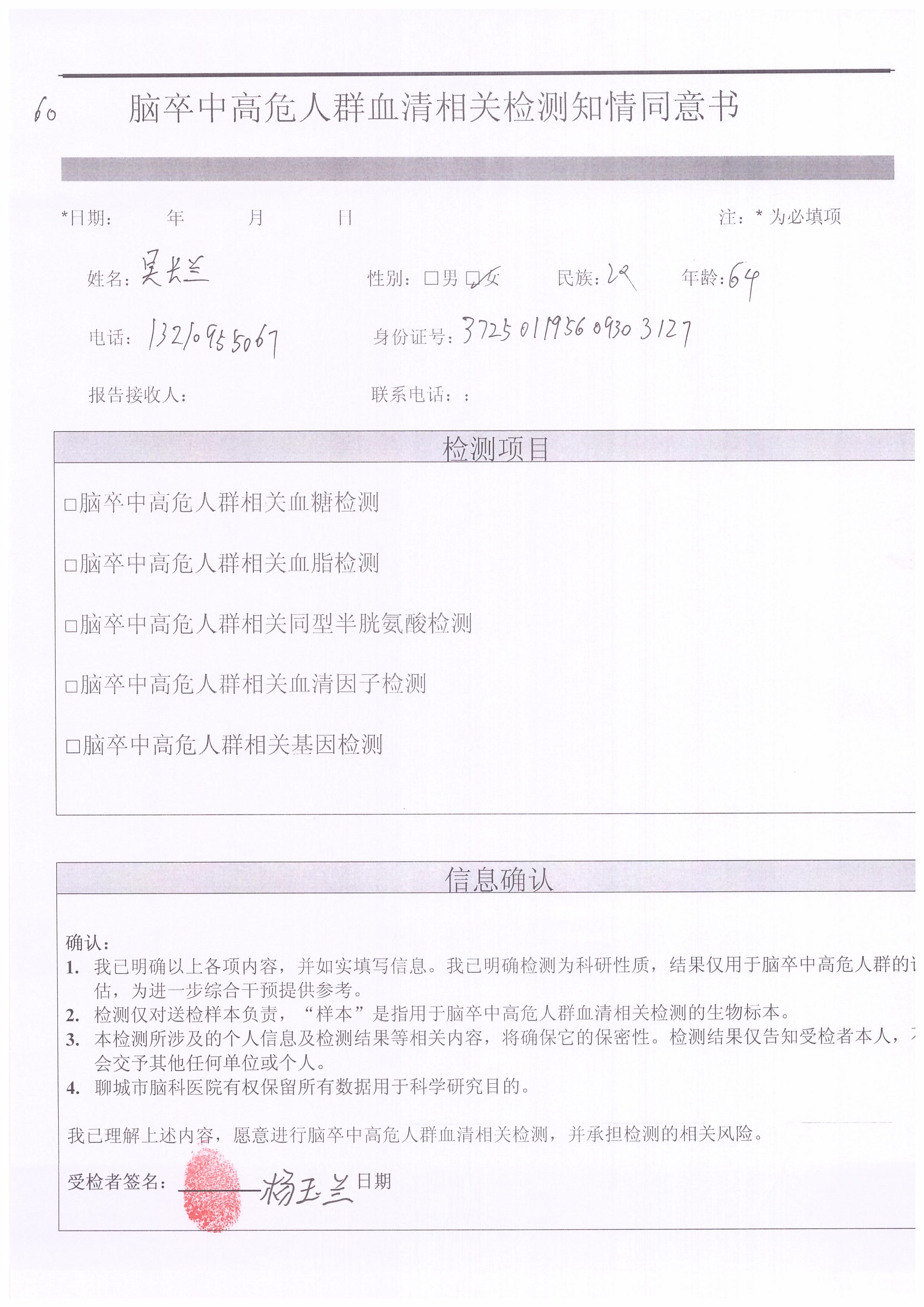

Supplement: Supplementary file 9 — Supplementary file9 (ZIP 24580 KB) [file 10528_2023_10431_MOESM9_ESM.zip › ╓¬╟Θ═1⁄4╥Γ╩Θ7/╡┌2▓┐╖╓/015.jpg]

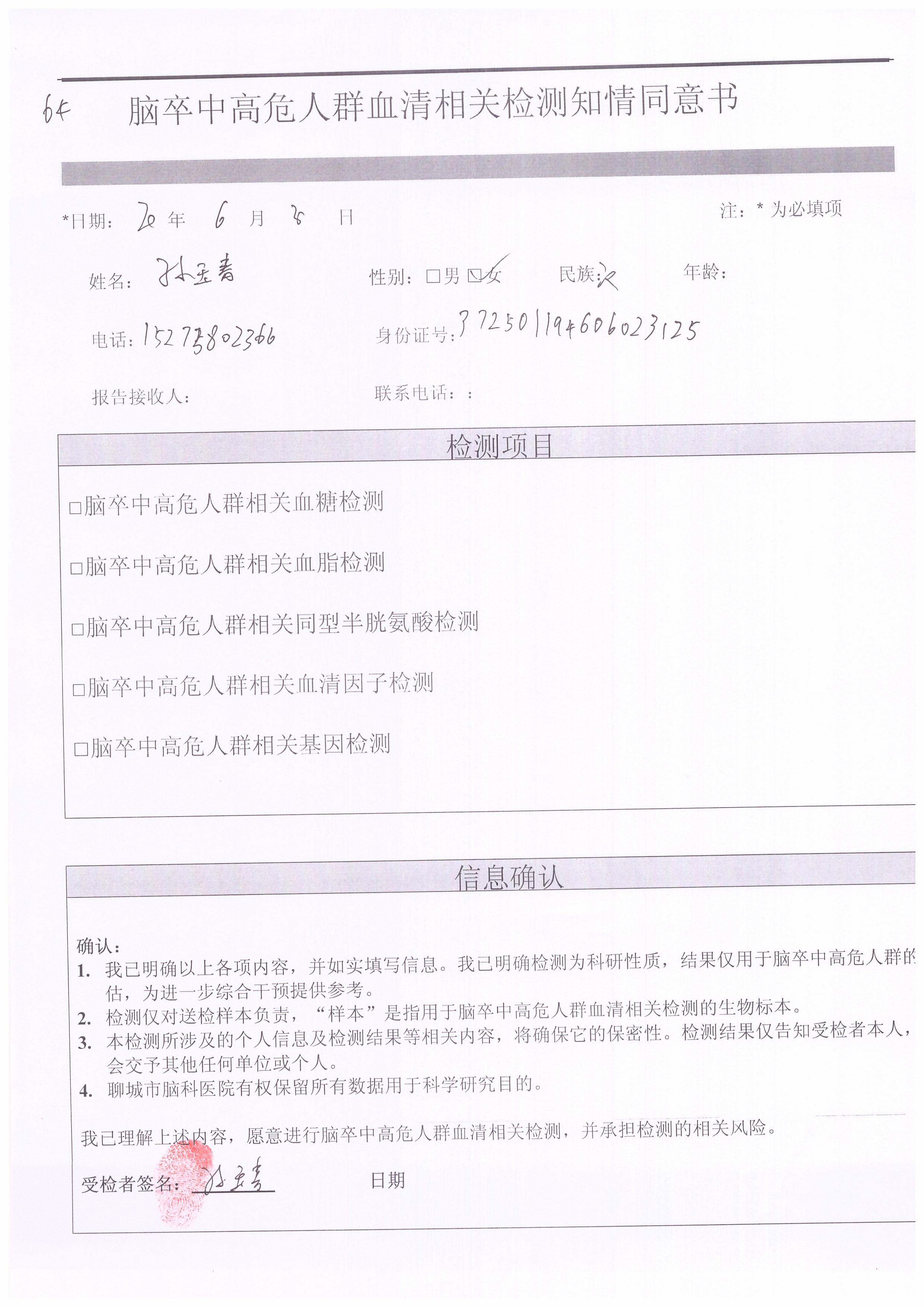

Supplement: Supplementary file 9 — Supplementary file9 (ZIP 24580 KB) [file 10528_2023_10431_MOESM9_ESM.zip › ╓¬╟Θ═1⁄4╥Γ╩Θ7/╡┌2▓┐╖╓/019.jpg]

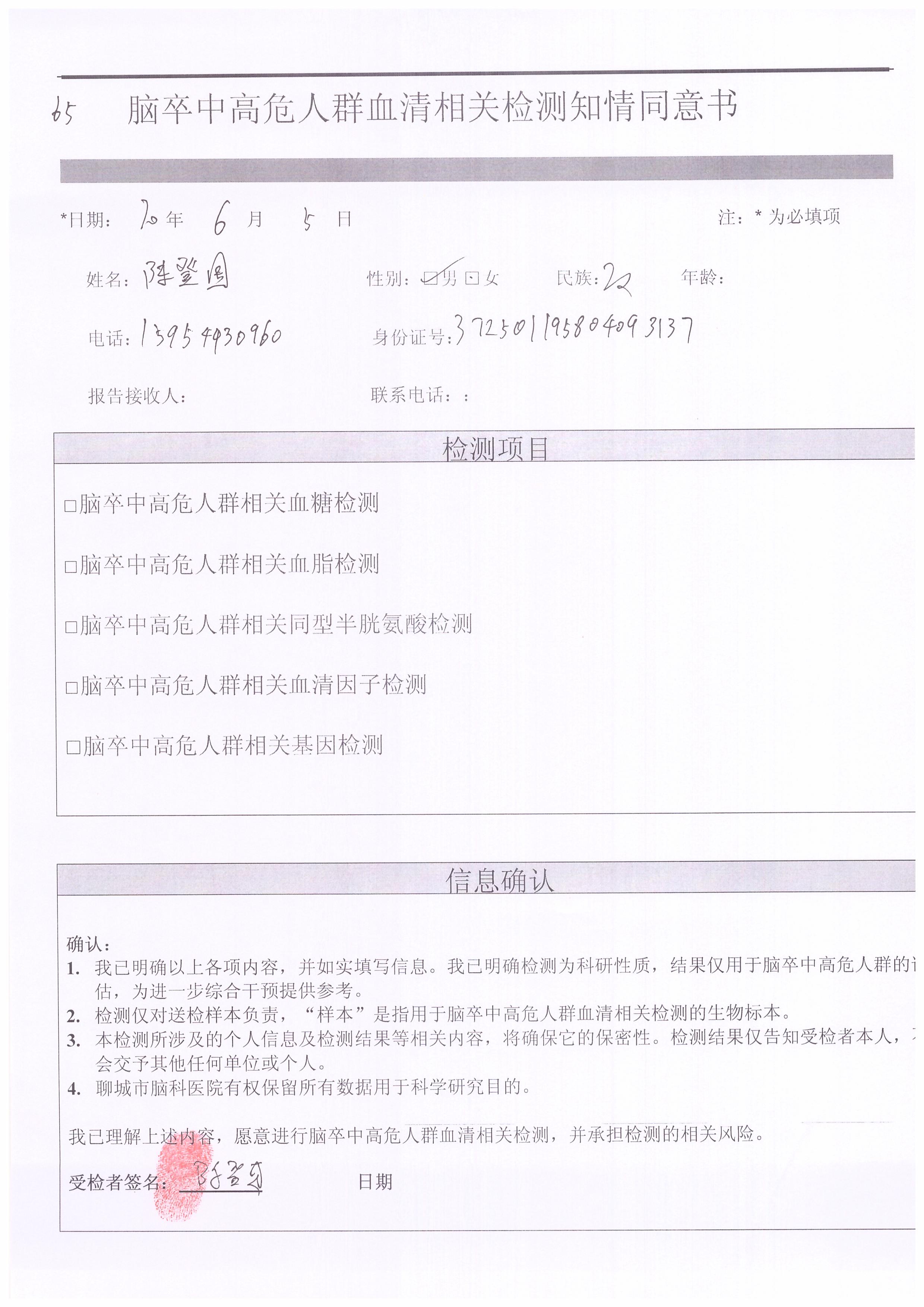

Supplement: Supplementary file 9 — Supplementary file9 (ZIP 24580 KB) [file 10528_2023_10431_MOESM9_ESM.zip › ╓¬╟Θ═1⁄4╥Γ╩Θ7/╡┌2▓┐╖╓/020.jpg]

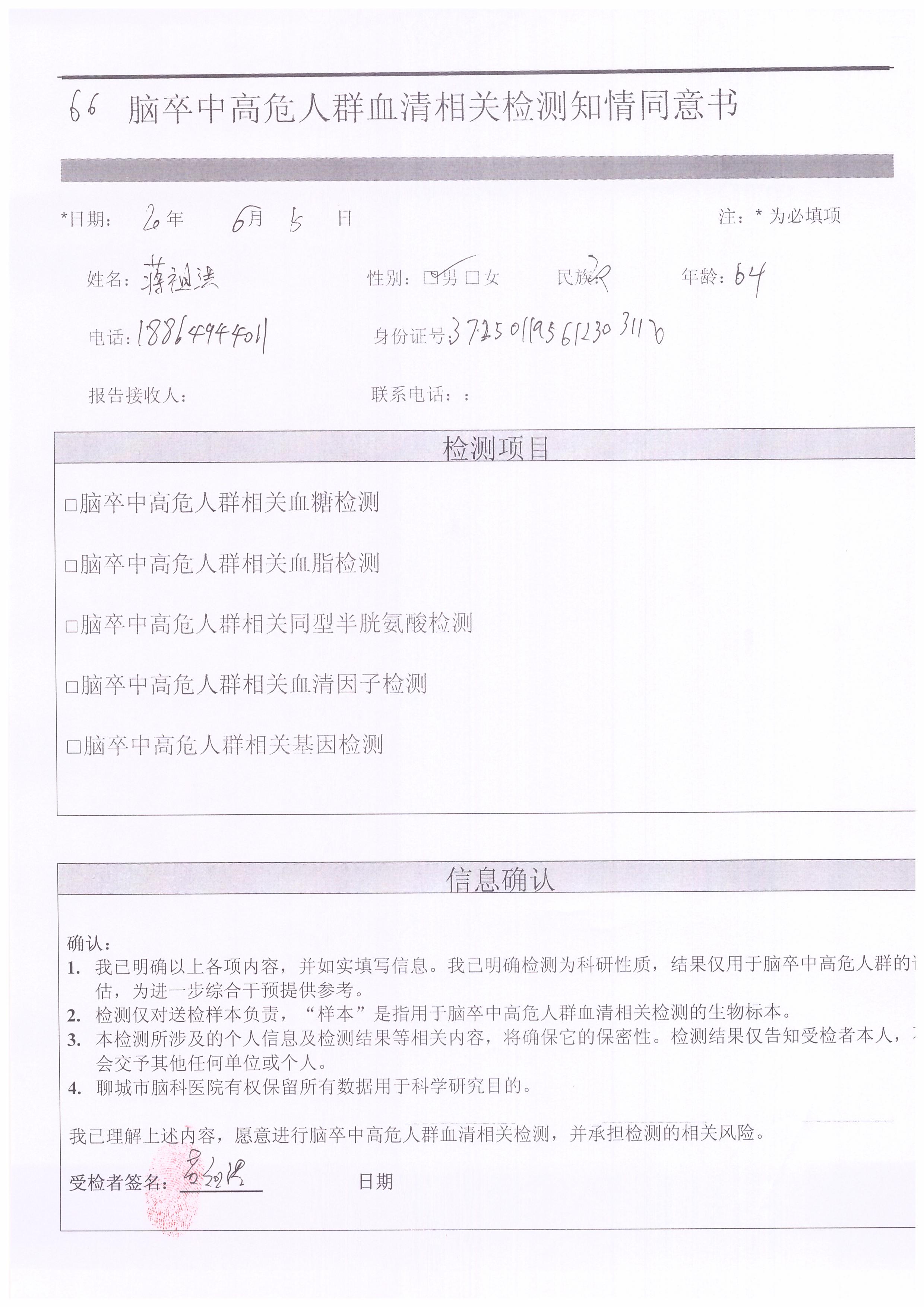

Supplement: Supplementary file 9 — Supplementary file9 (ZIP 24580 KB) [file 10528_2023_10431_MOESM9_ESM.zip › ╓¬╟Θ═1⁄4╥Γ╩Θ7/╡┌2▓┐╖╓/021.jpg]

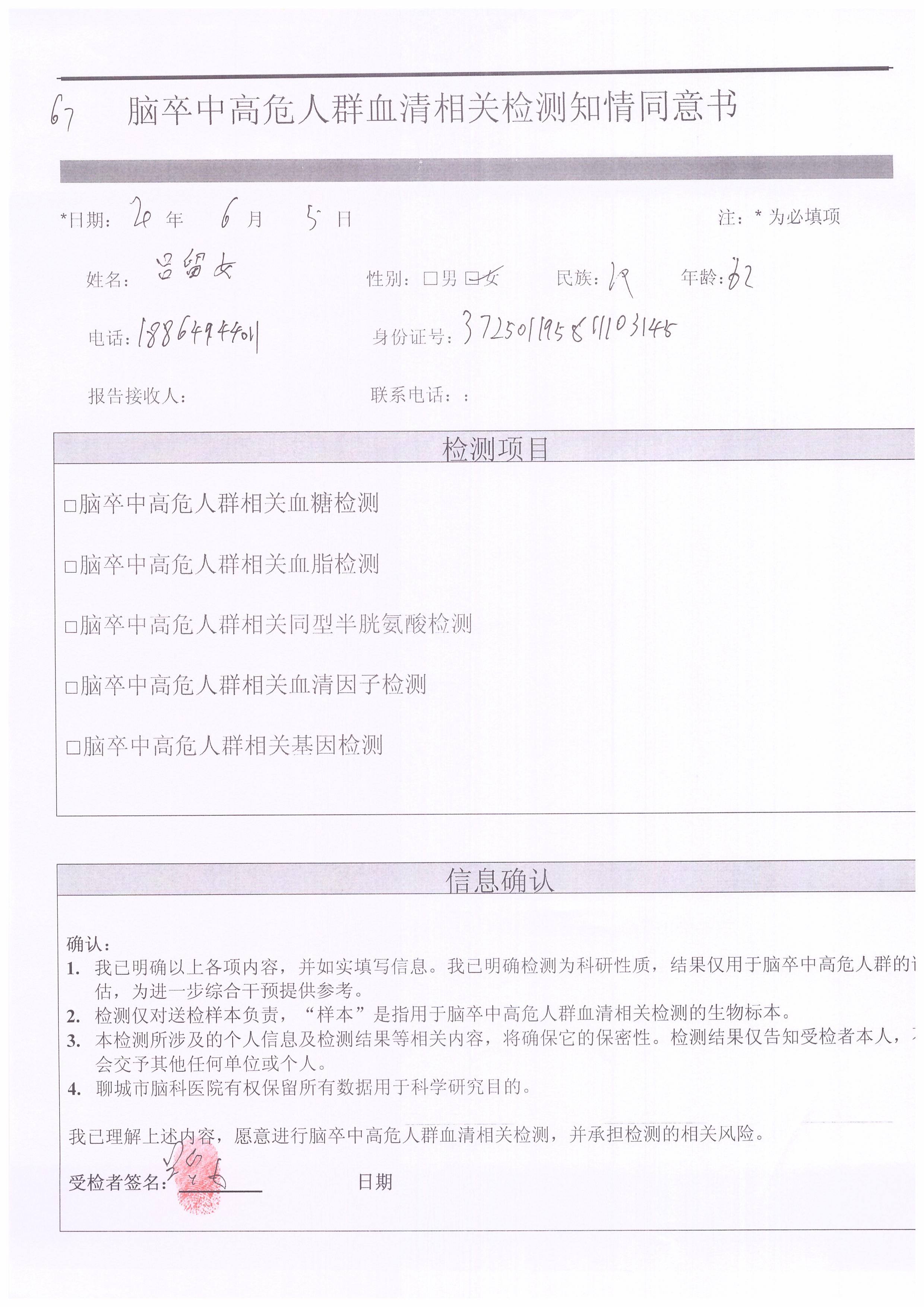

Supplement: Supplementary file 9 — Supplementary file9 (ZIP 24580 KB) [file 10528_2023_10431_MOESM9_ESM.zip › ╓¬╟Θ═1⁄4╥Γ╩Θ7/╡┌2▓┐╖╓/022.jpg]

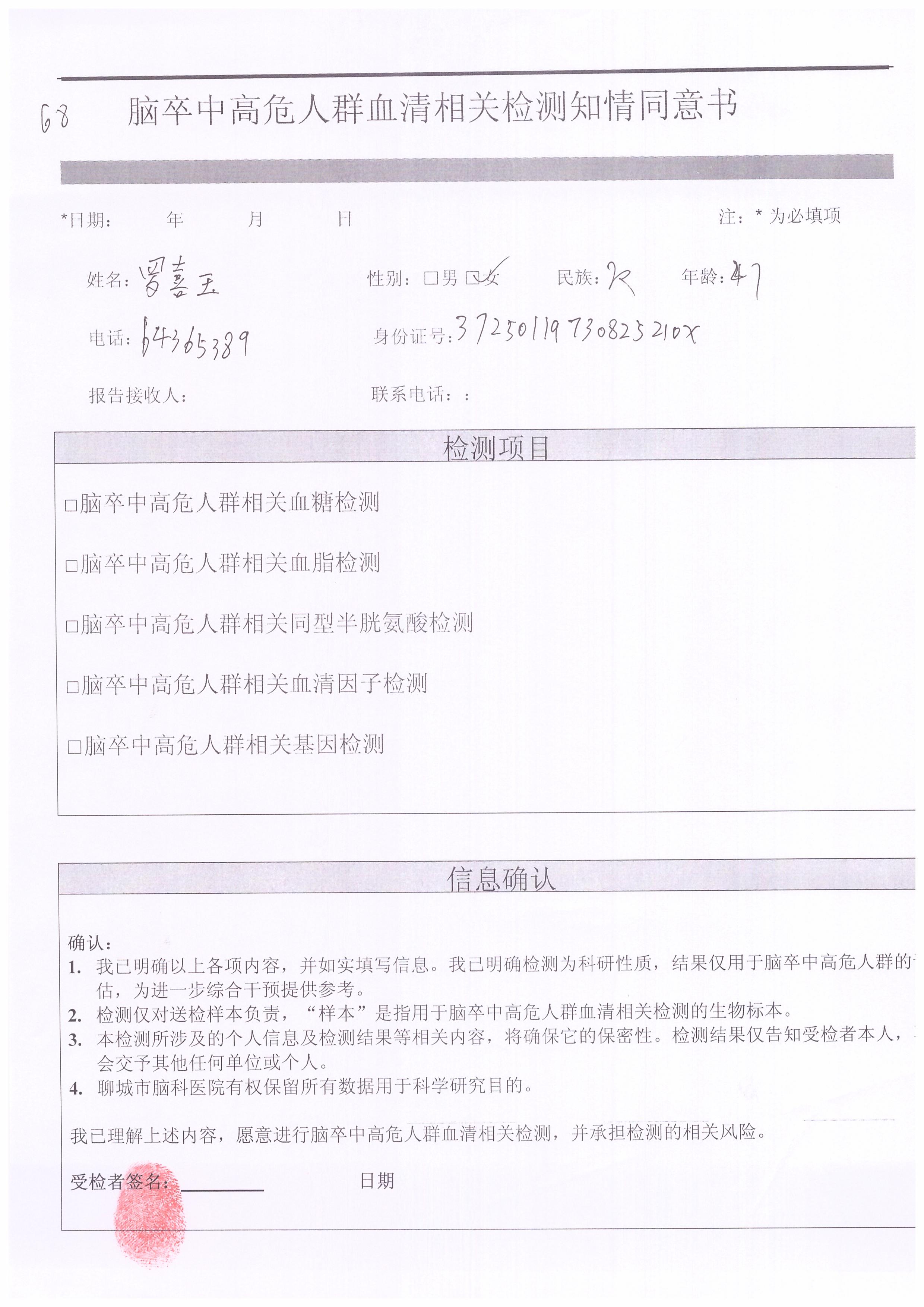

Supplement: Supplementary file 9 — Supplementary file9 (ZIP 24580 KB) [file 10528_2023_10431_MOESM9_ESM.zip › ╓¬╟Θ═1⁄4╥Γ╩Θ7/╡┌2▓┐╖╓/023.jpg]

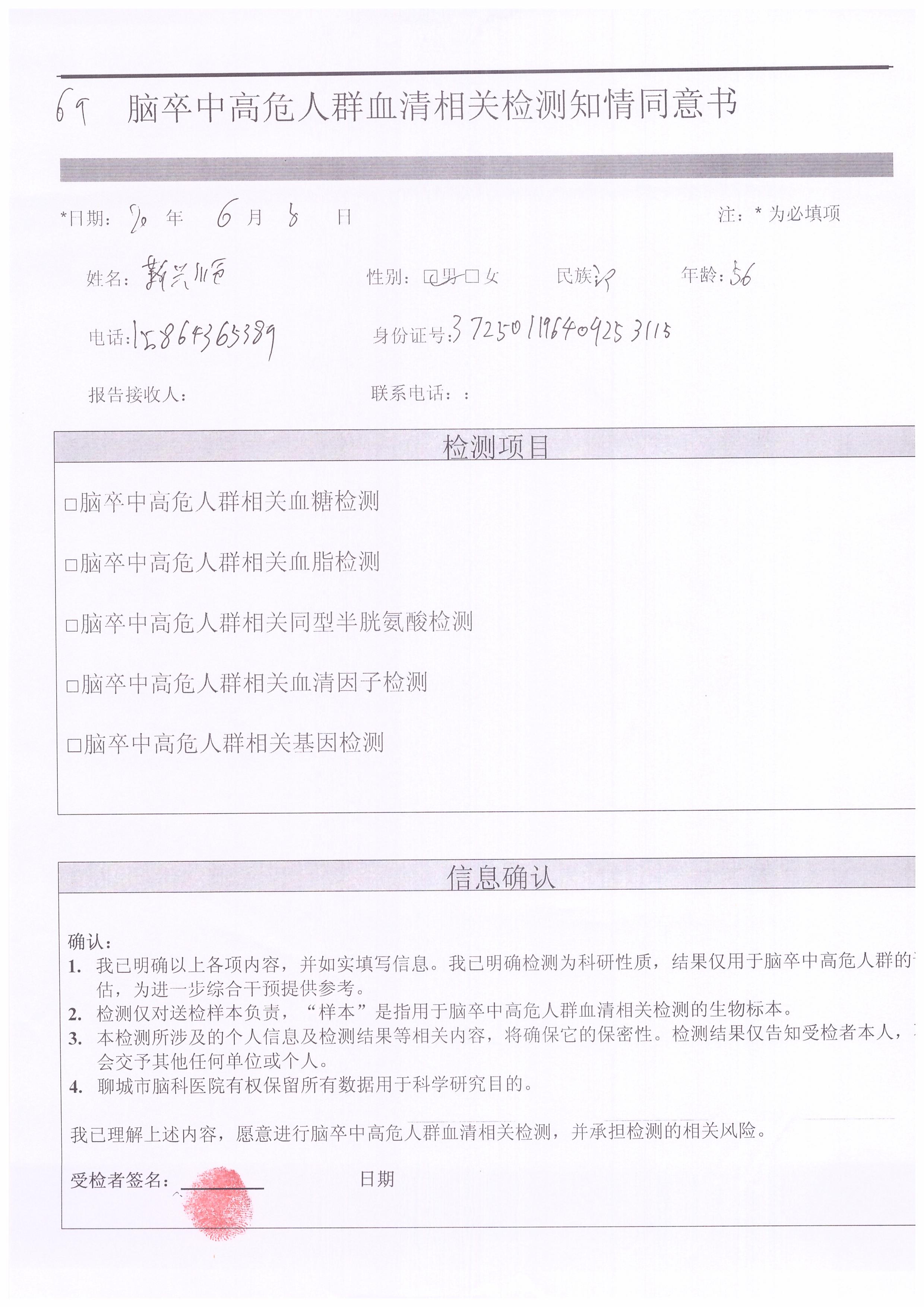

Supplement: Supplementary file 9 — Supplementary file9 (ZIP 24580 KB) [file 10528_2023_10431_MOESM9_ESM.zip › ╓¬╟Θ═1⁄4╥Γ╩Θ7/╡┌2▓┐╖╓/024.jpg]

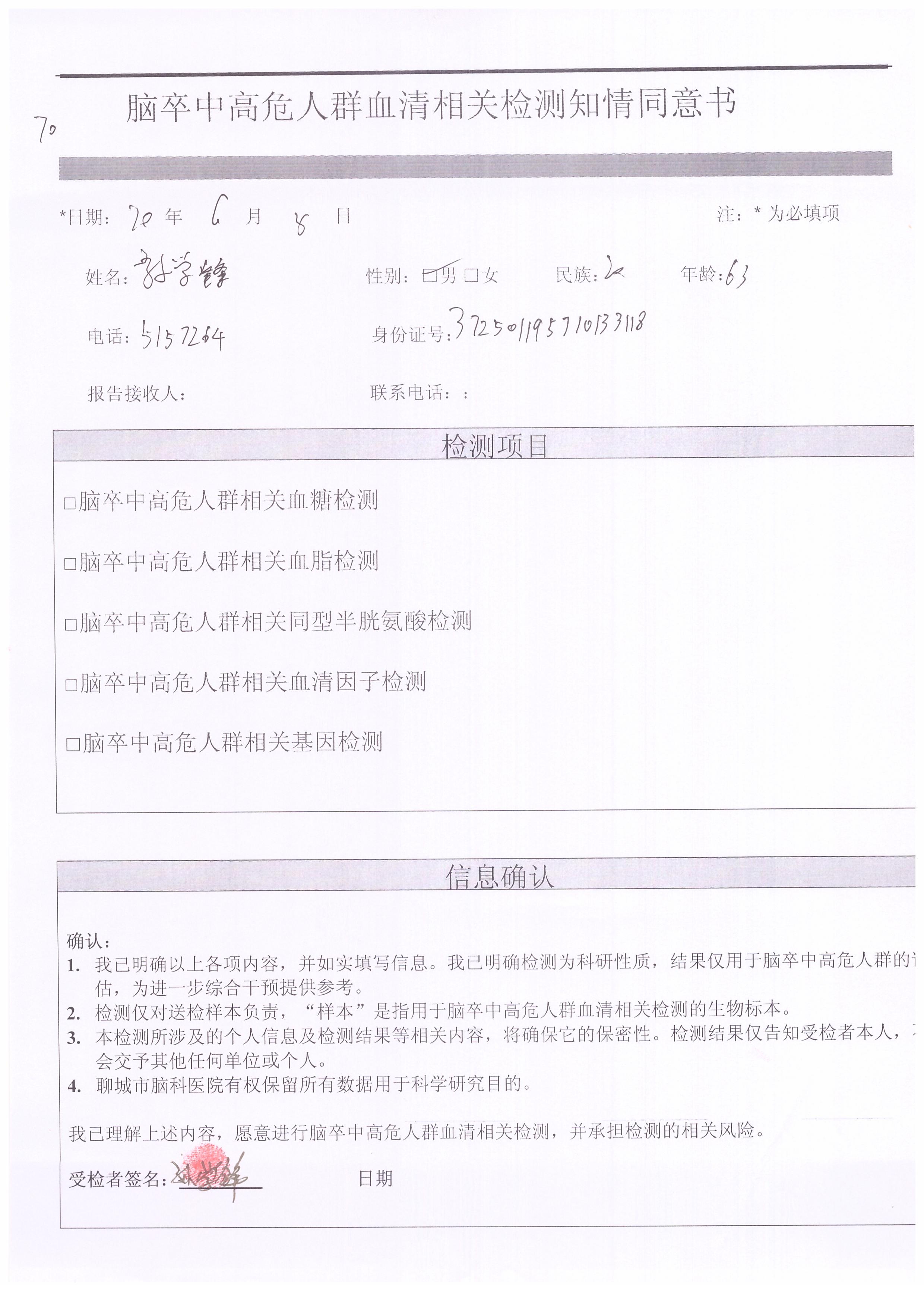

Supplement: Supplementary file 9 — Supplementary file9 (ZIP 24580 KB) [file 10528_2023_10431_MOESM9_ESM.zip › ╓¬╟Θ═1⁄4╥Γ╩Θ7/╡┌2▓┐╖╓/025.jpg]
