# Supplementary figures and images for: The Association Between Thymidylate Synthase Gene Polymorphisms and the Risk of Ischemic Stroke in Chinese Han Population (part 4 of 6)
Source: Biochem Genet. 2023 Jun 28;62(1):468–84. doi: 10.1007/s10528-023-10431-8 (PMC10901929; doi:10.1007/s10528-023-10431-8)

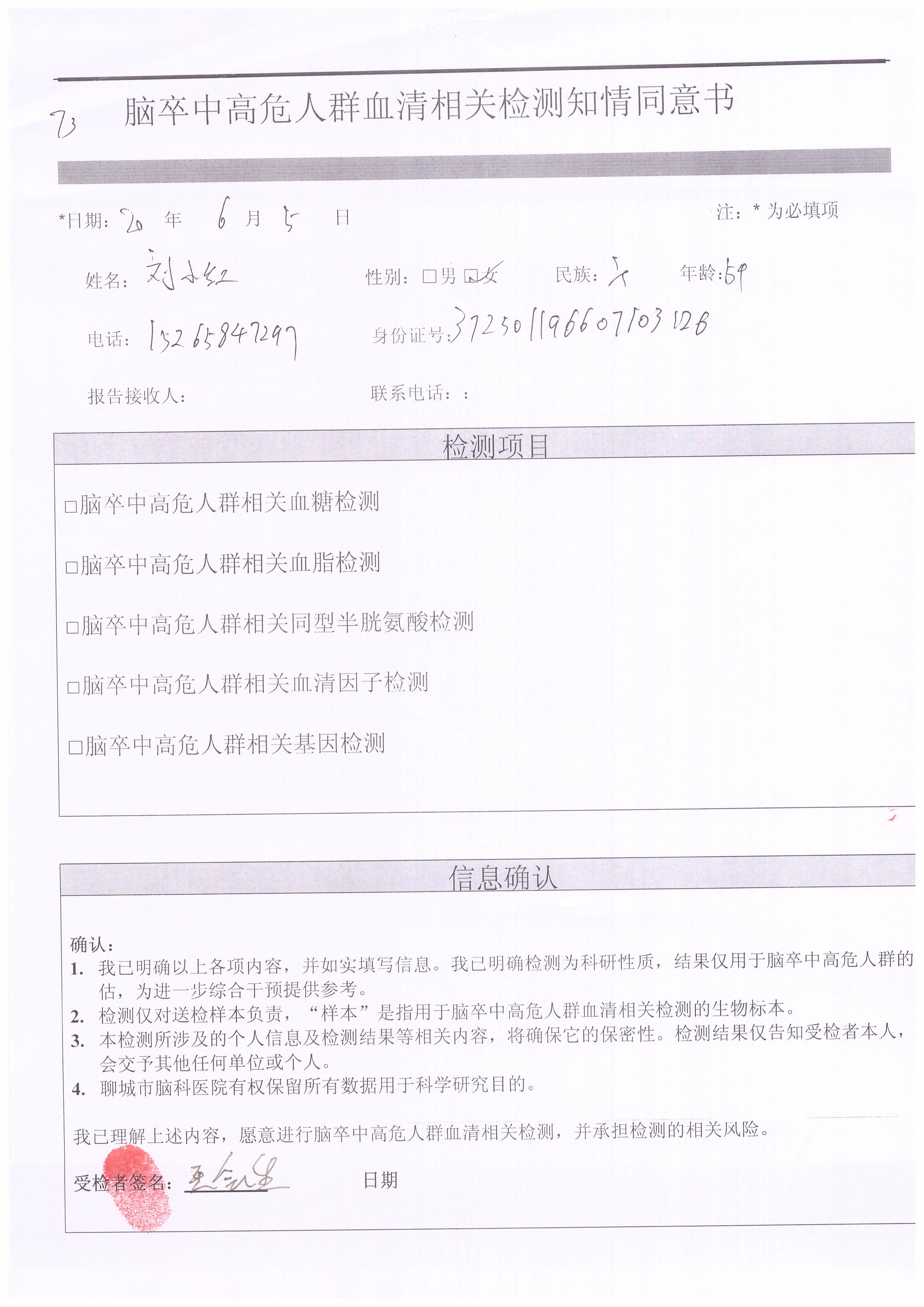

Supplement: Supplementary file 9 — Supplementary file9 (ZIP 24580 KB) [file 10528_2023_10431_MOESM9_ESM.zip › ╓¬╟Θ═1⁄4╥Γ╩Θ7/╡┌2▓┐╖╓/027.jpg]

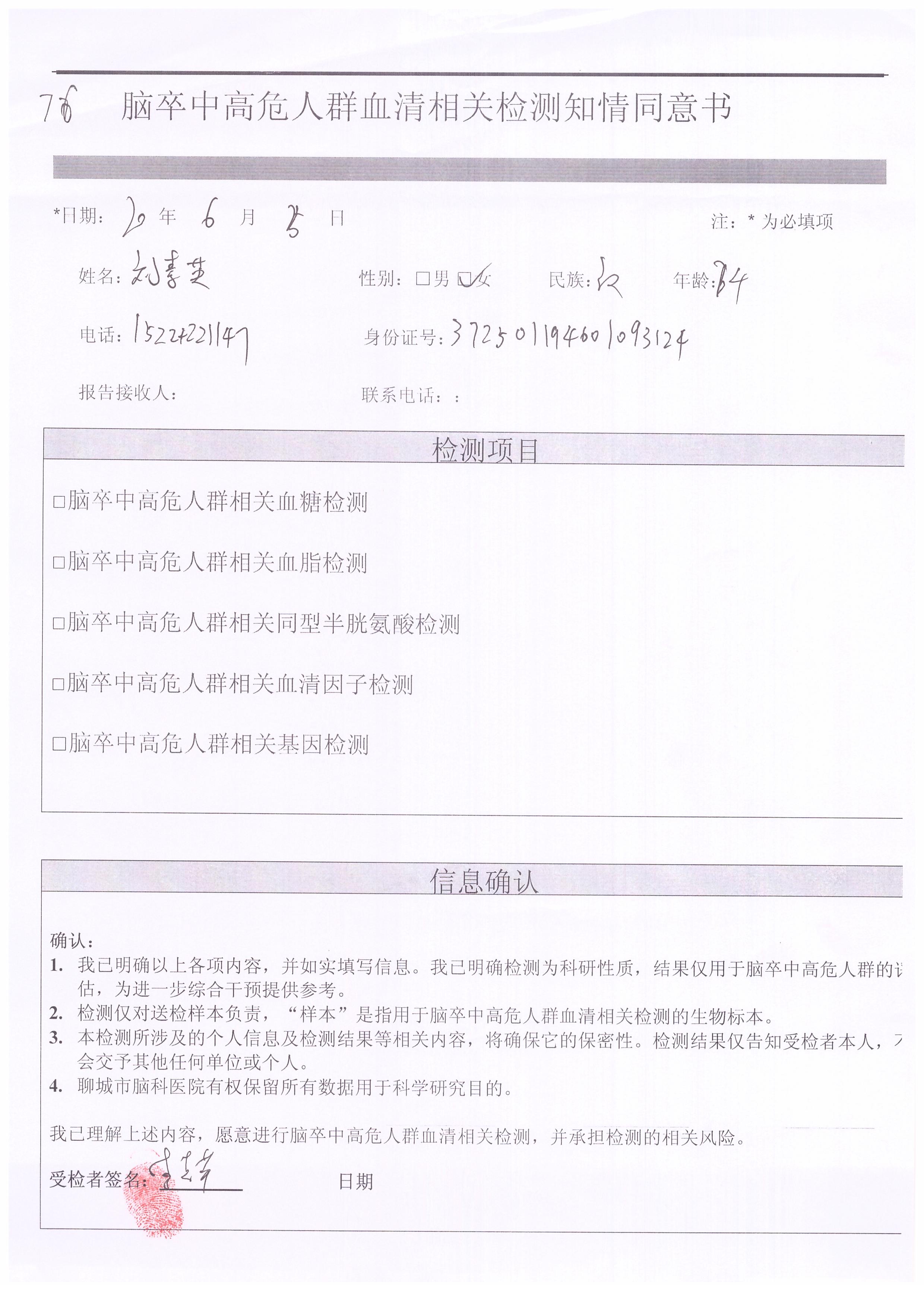

Supplement: Supplementary file 9 — Supplementary file9 (ZIP 24580 KB) [file 10528_2023_10431_MOESM9_ESM.zip › ╓¬╟Θ═1⁄4╥Γ╩Θ7/╡┌2▓┐╖╓/029.jpg]

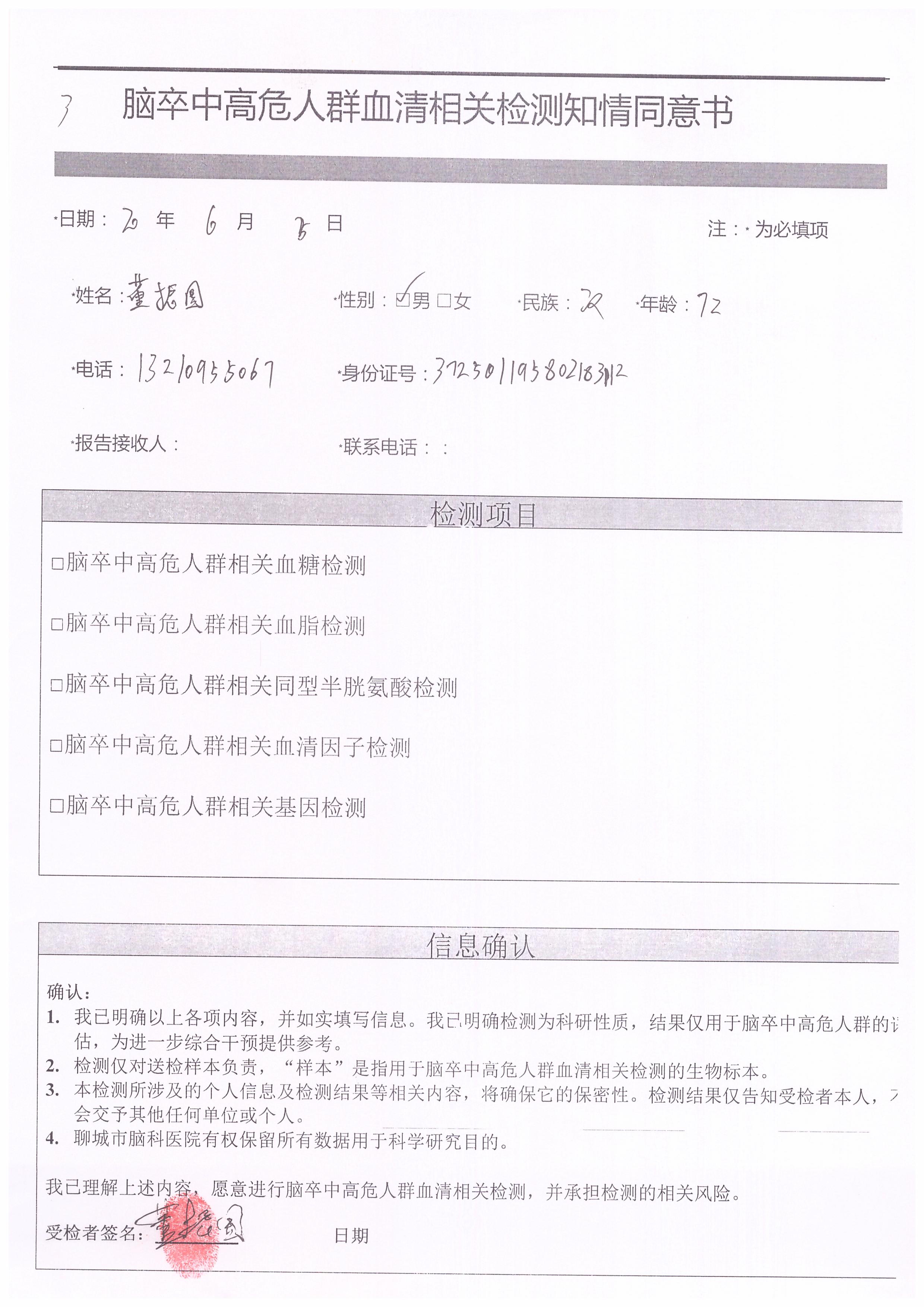

Supplement: Supplementary file 9 — Supplementary file9 (ZIP 24580 KB) [file 10528_2023_10431_MOESM9_ESM.zip › ╓¬╟Θ═1⁄4╥Γ╩Θ7/╡┌╥╗▓┐╖╓í┐/001.jpg]

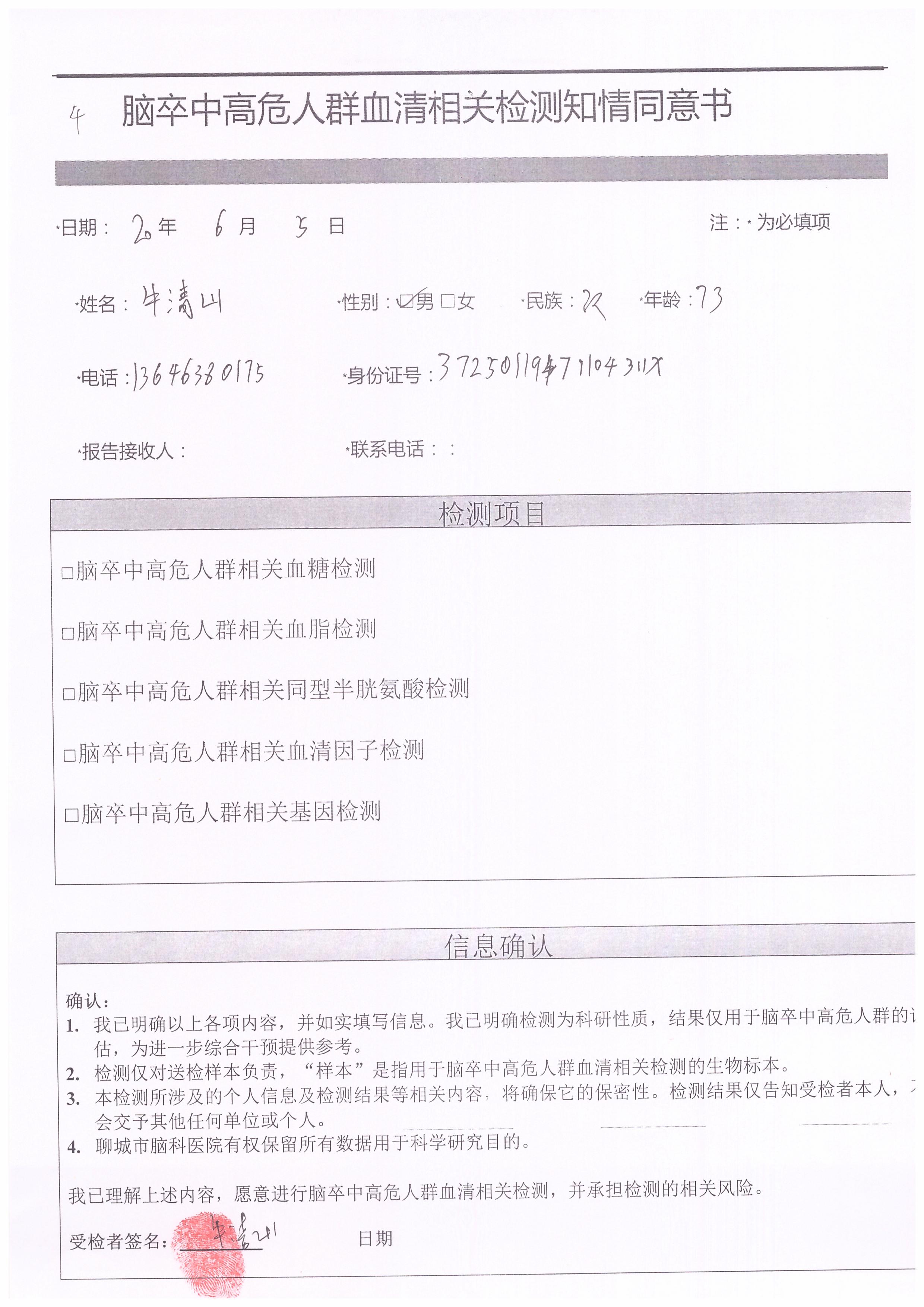

Supplement: Supplementary file 9 — Supplementary file9 (ZIP 24580 KB) [file 10528_2023_10431_MOESM9_ESM.zip › ╓¬╟Θ═1⁄4╥Γ╩Θ7/╡┌╥╗▓┐╖╓í┐/002.jpg]

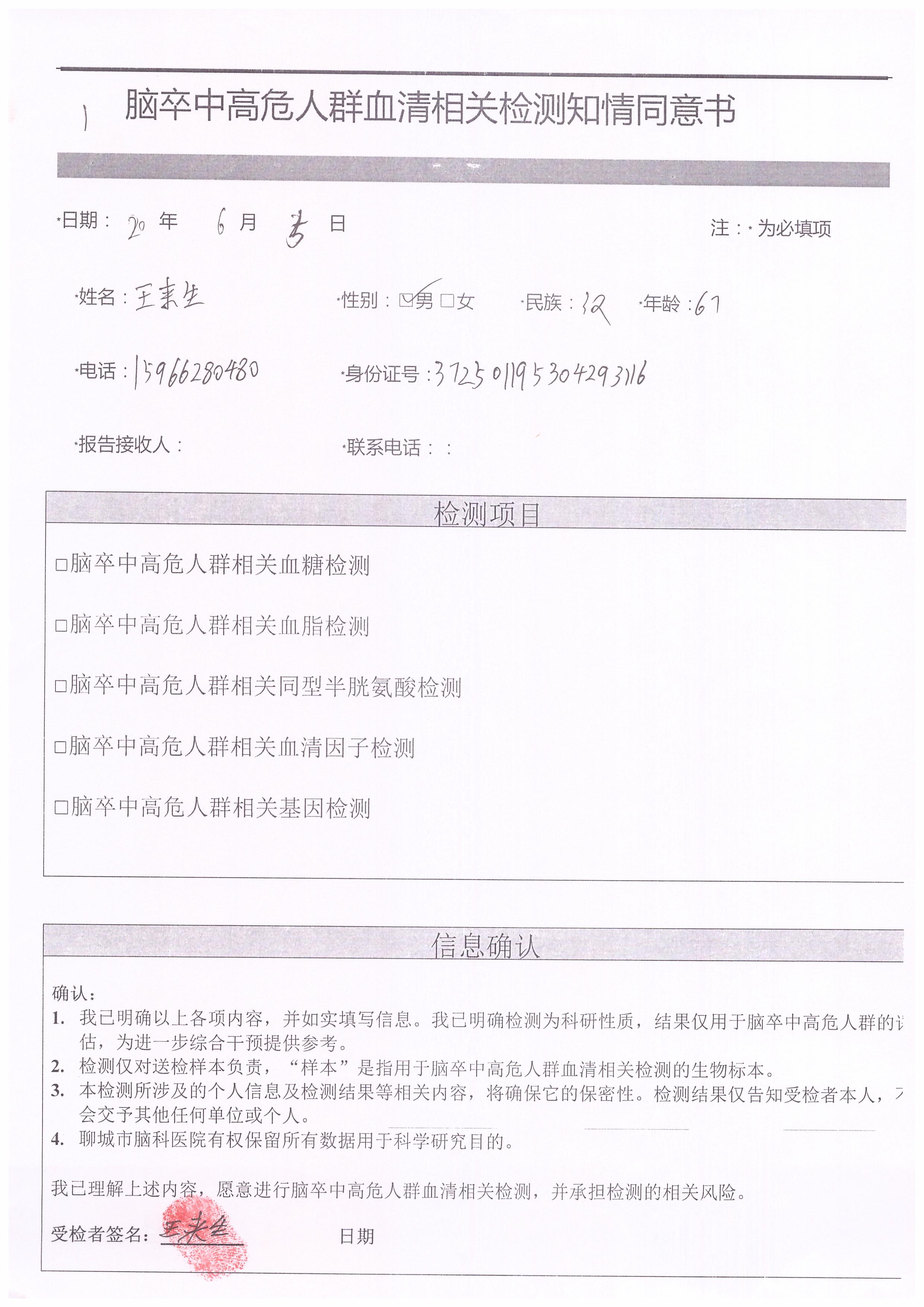

Supplement: Supplementary file 9 — Supplementary file9 (ZIP 24580 KB) [file 10528_2023_10431_MOESM9_ESM.zip › ╓¬╟Θ═1⁄4╥Γ╩Θ7/╡┌╥╗▓┐╖╓í┐/003.jpg]

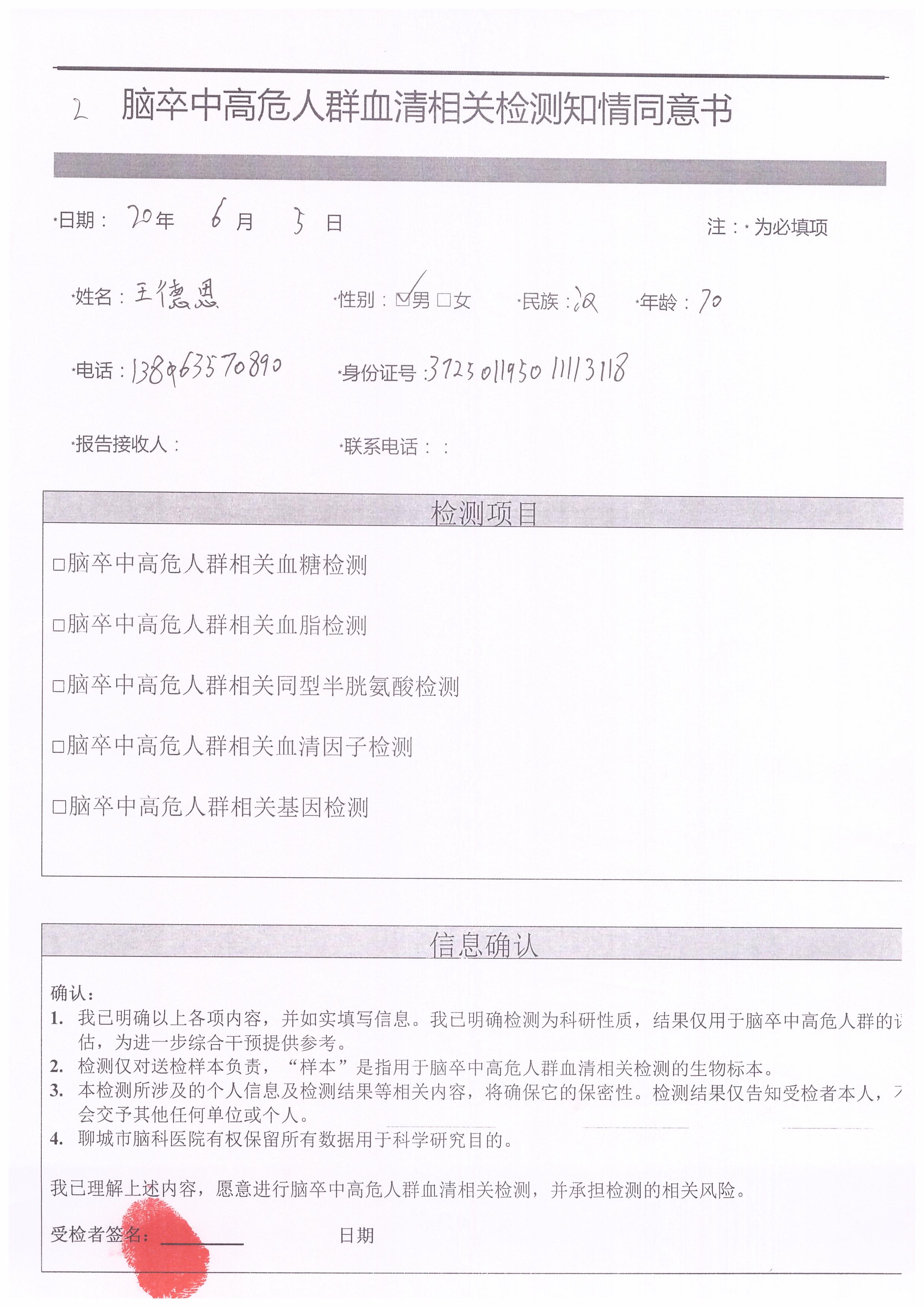

Supplement: Supplementary file 9 — Supplementary file9 (ZIP 24580 KB) [file 10528_2023_10431_MOESM9_ESM.zip › ╓¬╟Θ═1⁄4╥Γ╩Θ7/╡┌╥╗▓┐╖╓í┐/004.jpg]

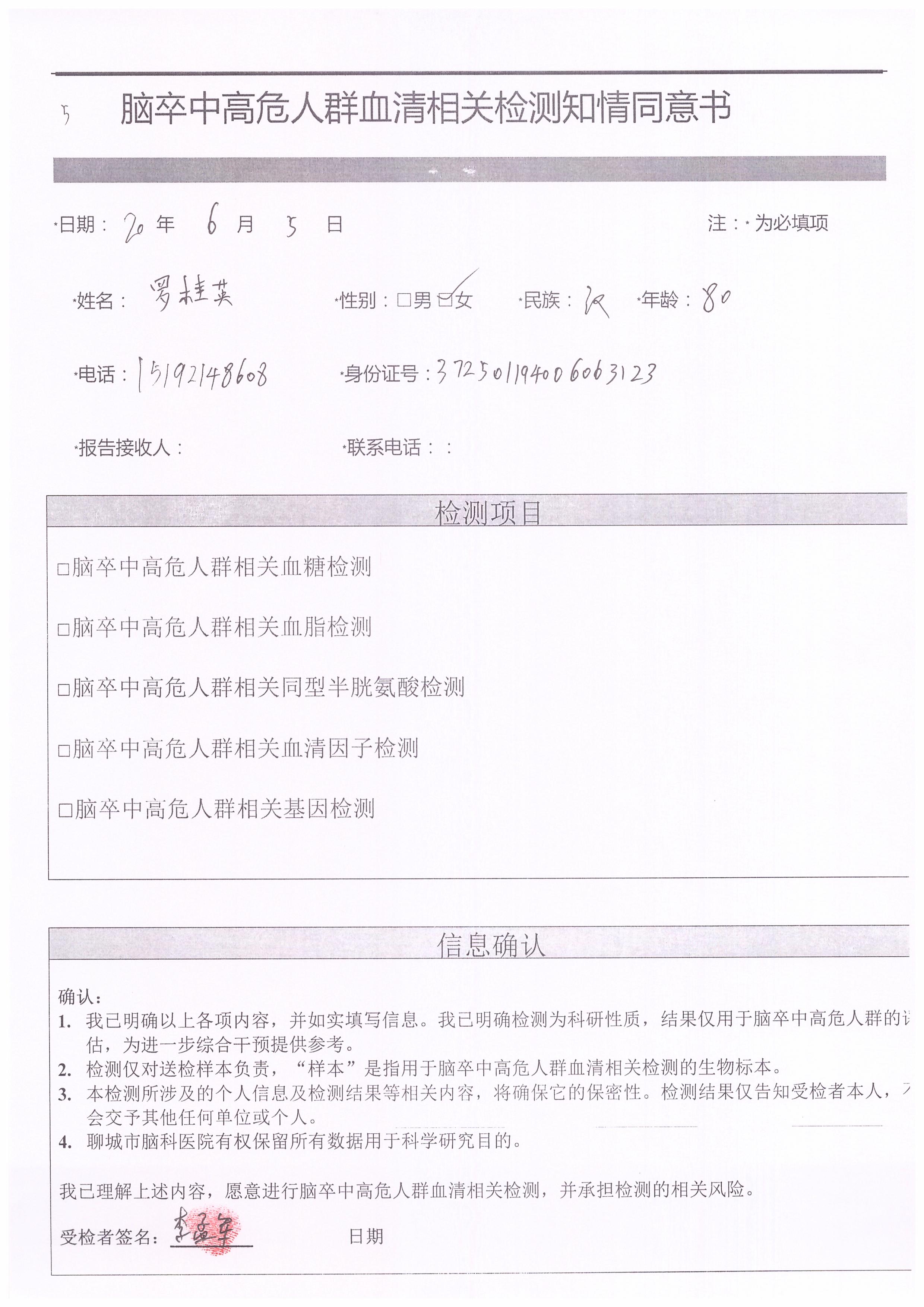

Supplement: Supplementary file 9 — Supplementary file9 (ZIP 24580 KB) [file 10528_2023_10431_MOESM9_ESM.zip › ╓¬╟Θ═1⁄4╥Γ╩Θ7/╡┌╥╗▓┐╖╓í┐/005.jpg]

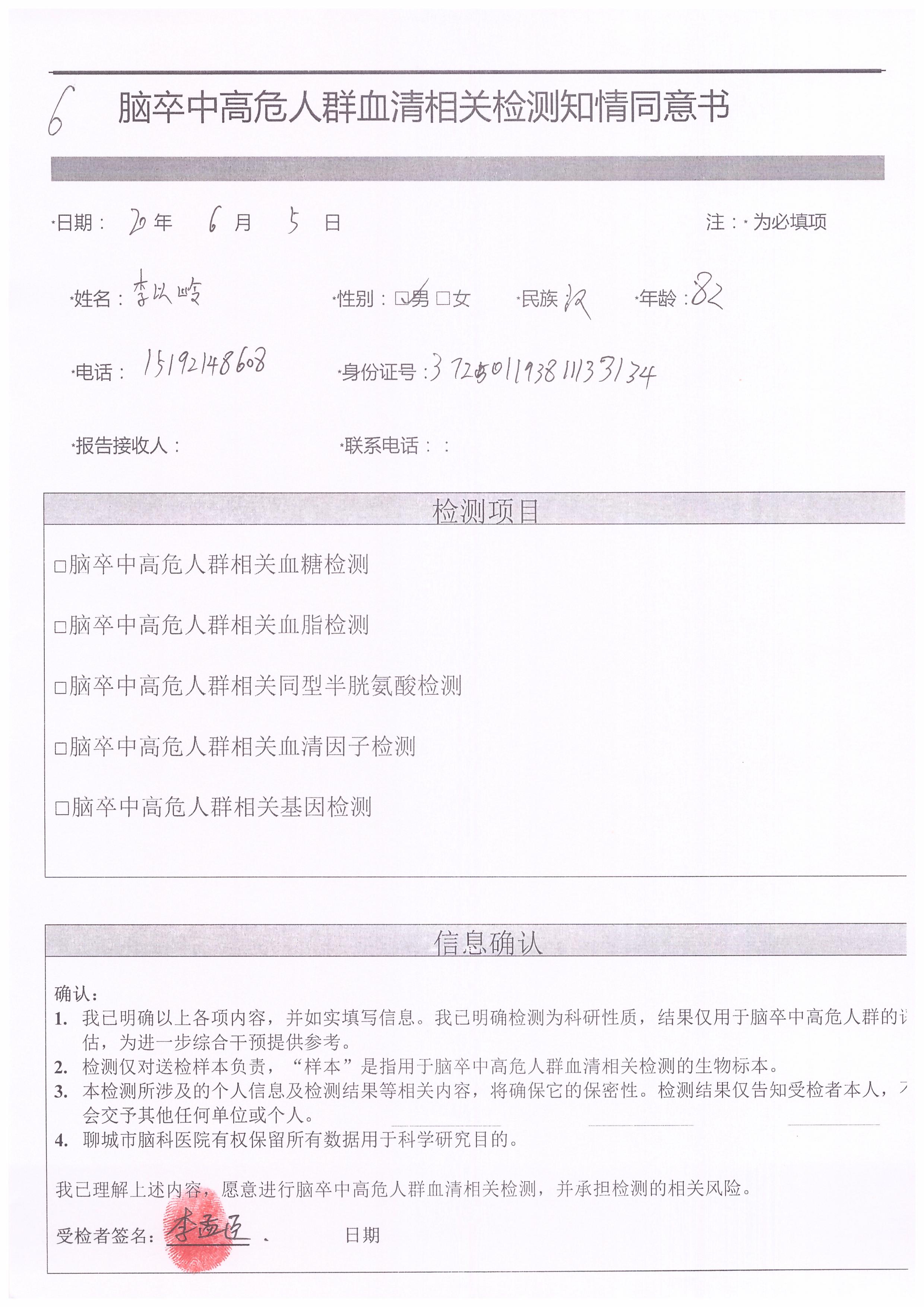

Supplement: Supplementary file 9 — Supplementary file9 (ZIP 24580 KB) [file 10528_2023_10431_MOESM9_ESM.zip › ╓¬╟Θ═1⁄4╥Γ╩Θ7/╡┌╥╗▓┐╖╓í┐/006.jpg]

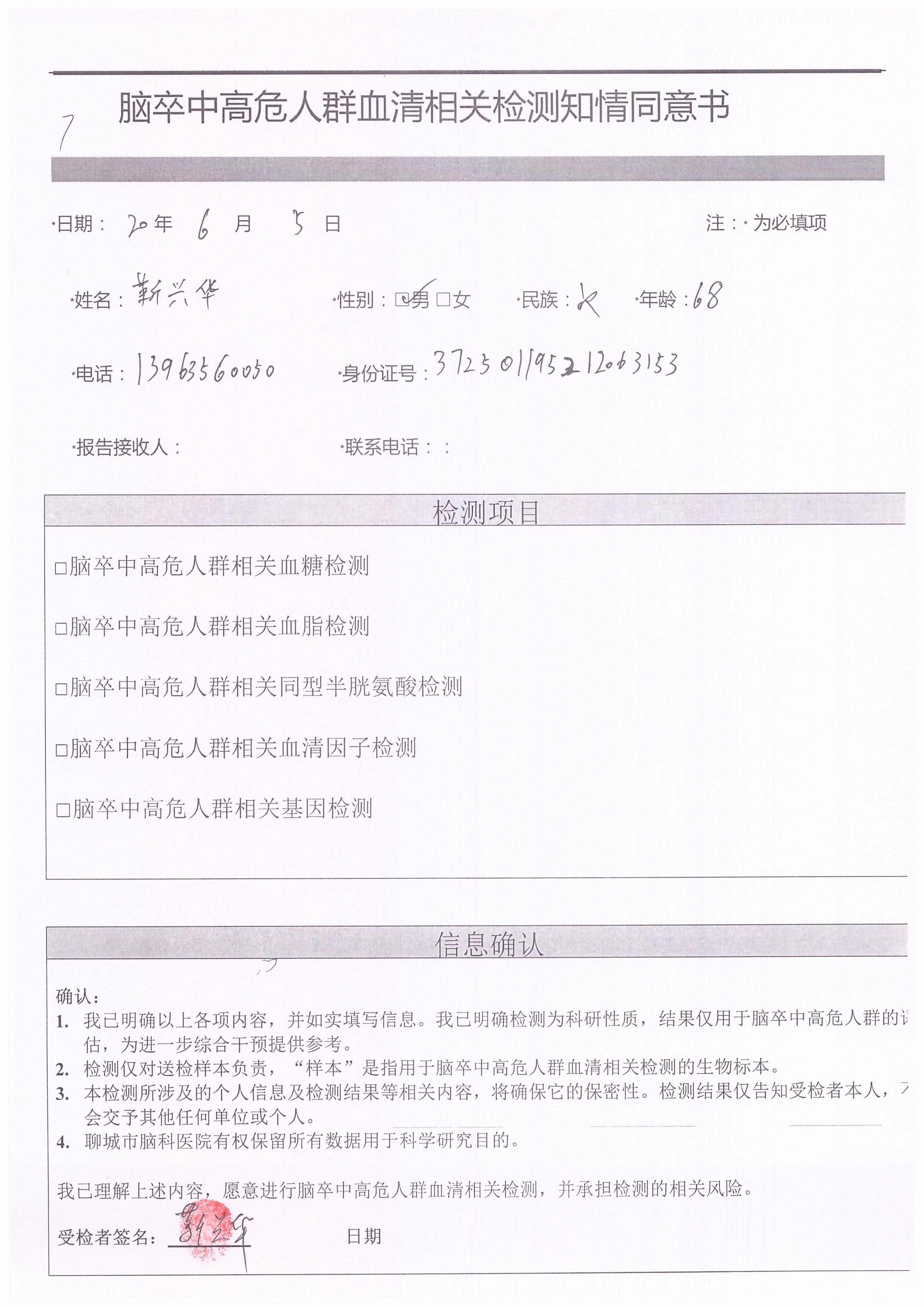

Supplement: Supplementary file 9 — Supplementary file9 (ZIP 24580 KB) [file 10528_2023_10431_MOESM9_ESM.zip › ╓¬╟Θ═1⁄4╥Γ╩Θ7/╡┌╥╗▓┐╖╓í┐/007.jpg]

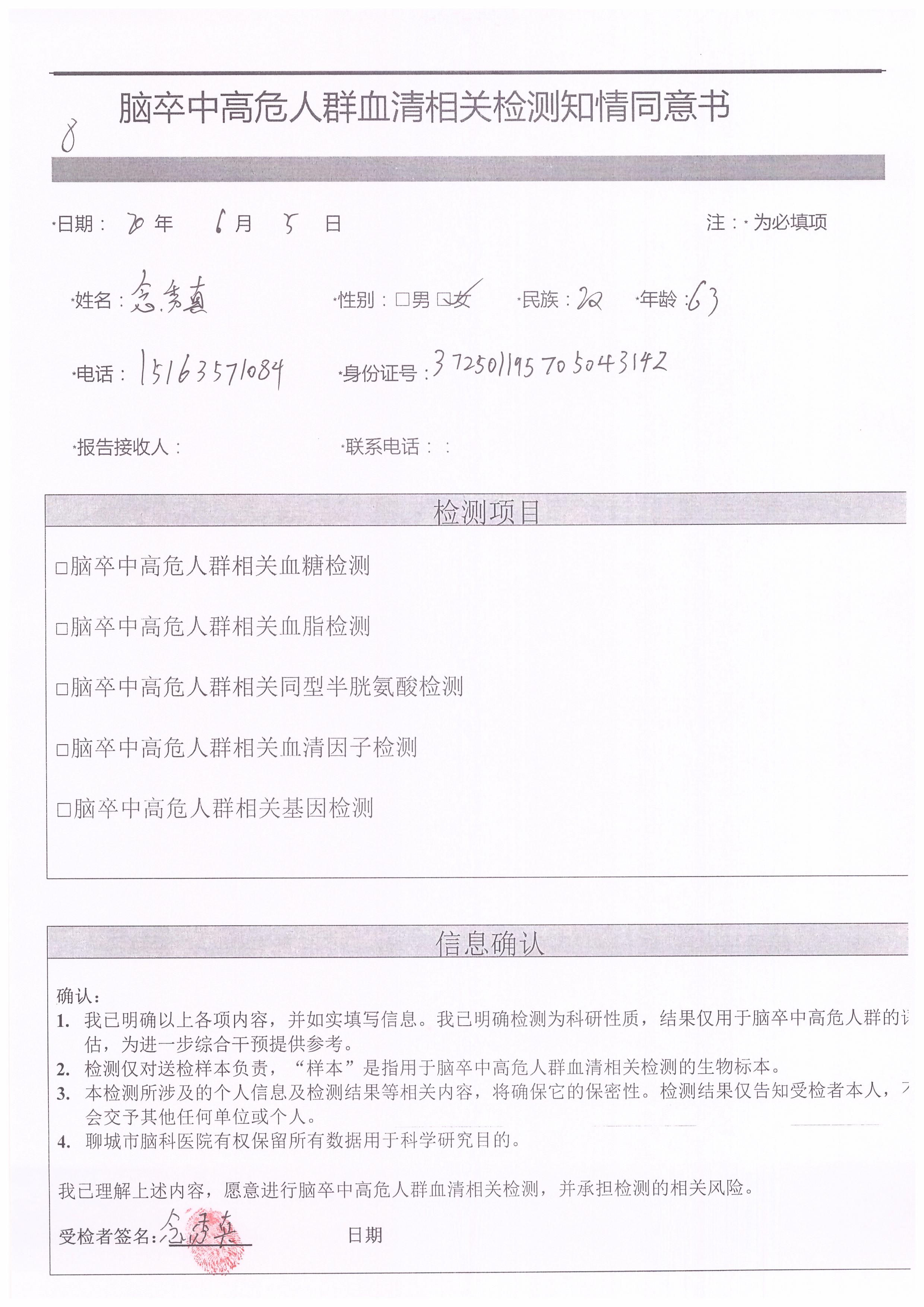

Supplement: Supplementary file 9 — Supplementary file9 (ZIP 24580 KB) [file 10528_2023_10431_MOESM9_ESM.zip › ╓¬╟Θ═1⁄4╥Γ╩Θ7/╡┌╥╗▓┐╖╓í┐/008.jpg]

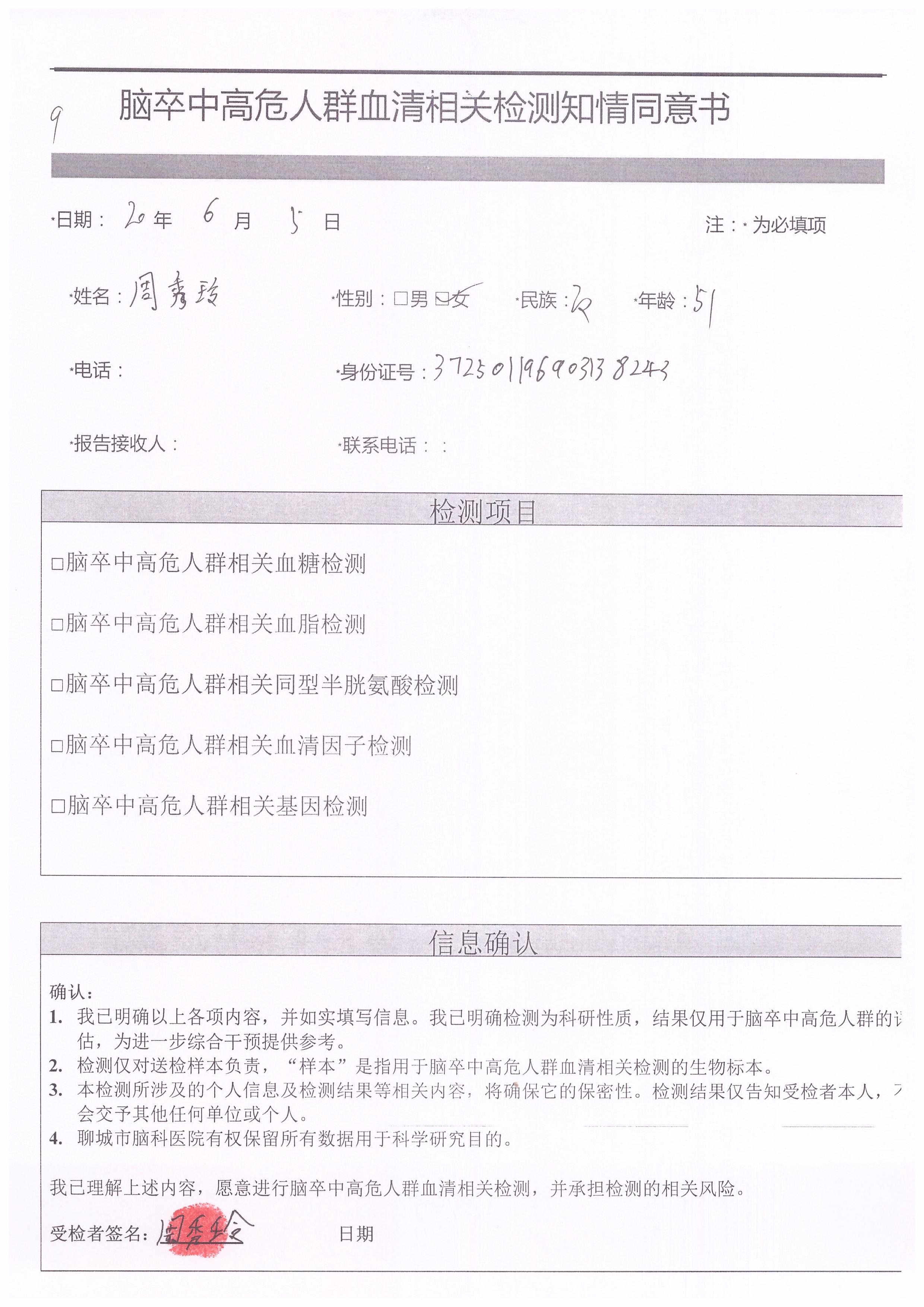

Supplement: Supplementary file 9 — Supplementary file9 (ZIP 24580 KB) [file 10528_2023_10431_MOESM9_ESM.zip › ╓¬╟Θ═1⁄4╥Γ╩Θ7/╡┌╥╗▓┐╖╓í┐/009.jpg]

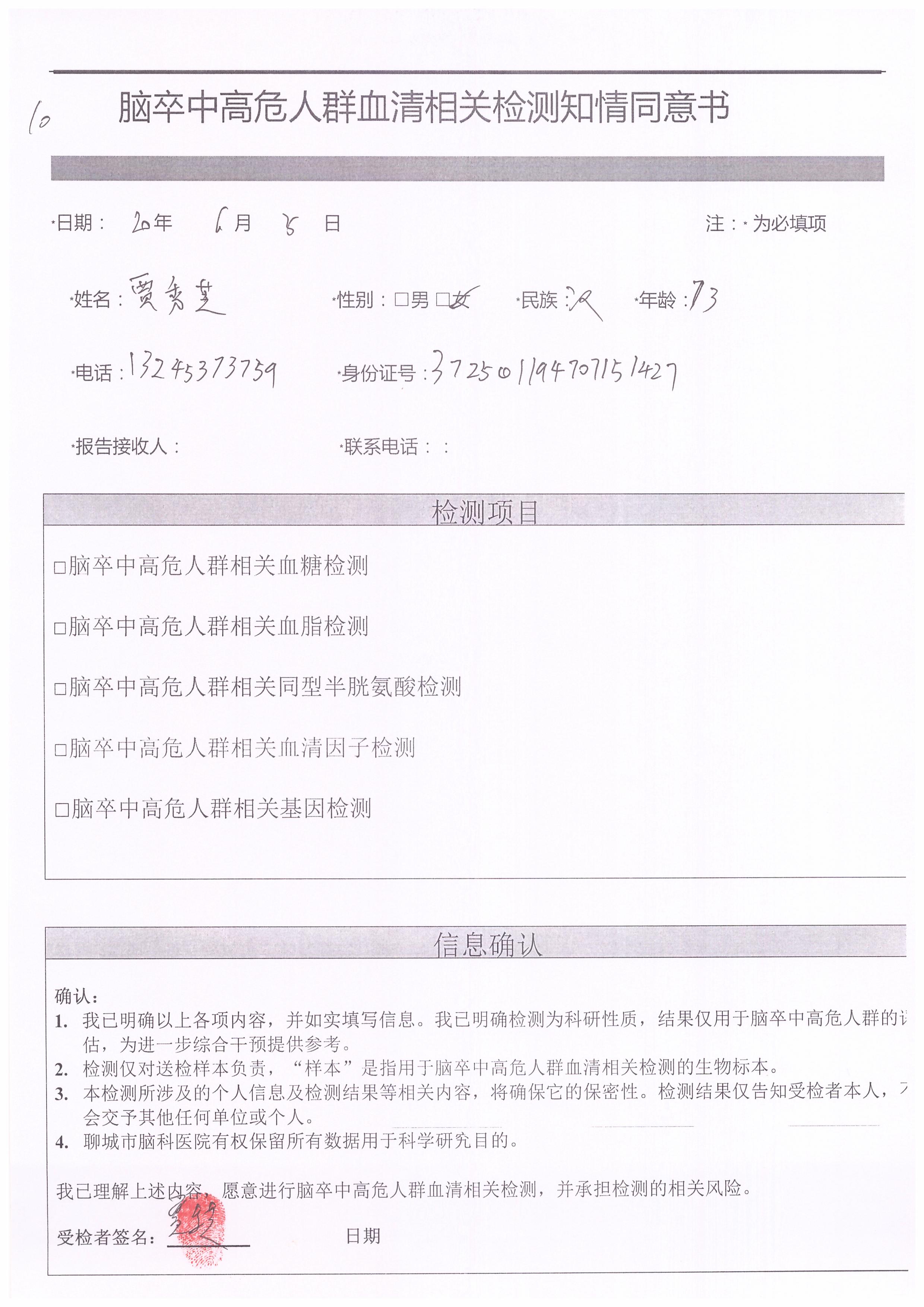

Supplement: Supplementary file 9 — Supplementary file9 (ZIP 24580 KB) [file 10528_2023_10431_MOESM9_ESM.zip › ╓¬╟Θ═1⁄4╥Γ╩Θ7/╡┌╥╗▓┐╖╓í┐/010.jpg]

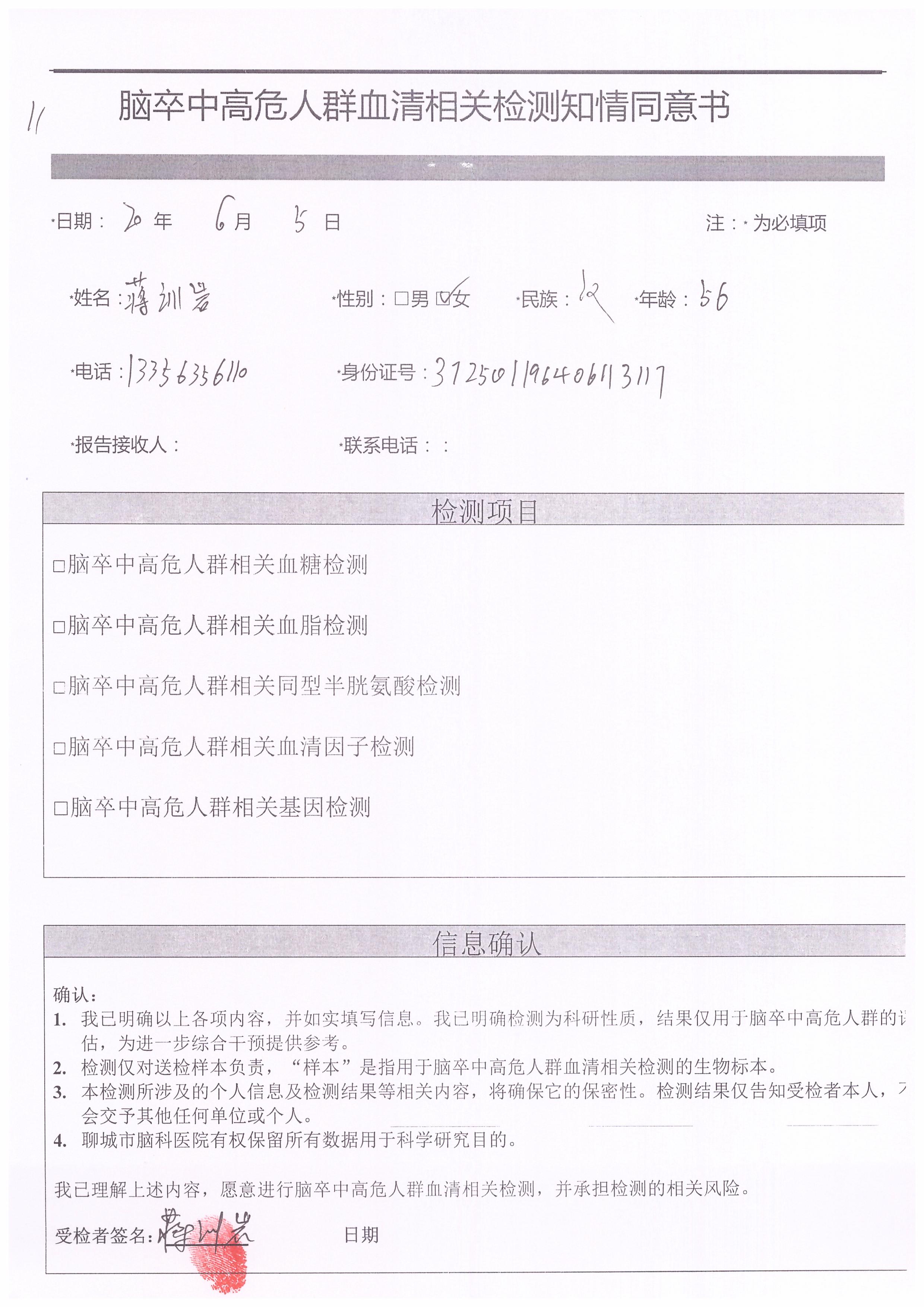

Supplement: Supplementary file 9 — Supplementary file9 (ZIP 24580 KB) [file 10528_2023_10431_MOESM9_ESM.zip › ╓¬╟Θ═1⁄4╥Γ╩Θ7/╡┌╥╗▓┐╖╓í┐/011.jpg]

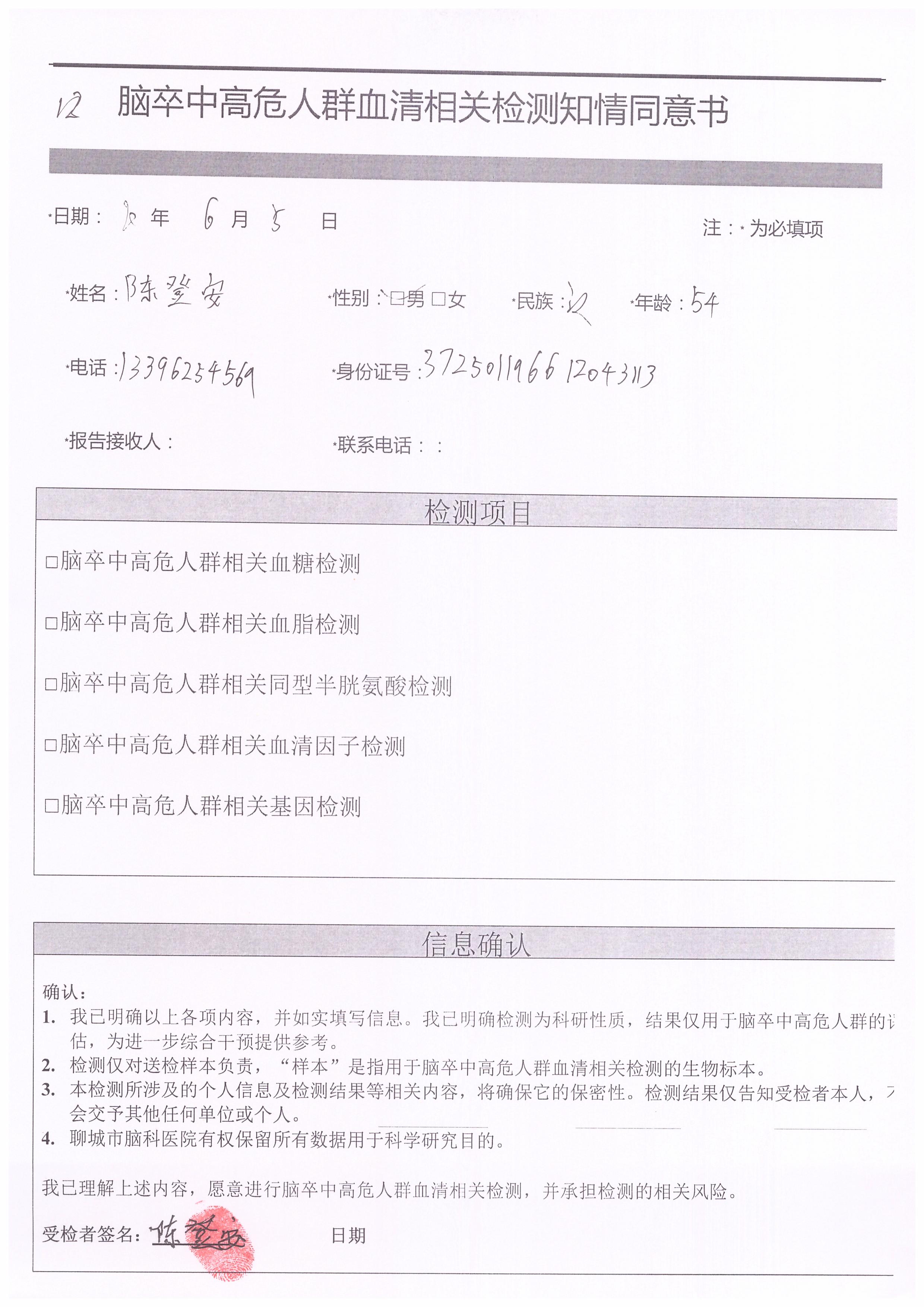

Supplement: Supplementary file 9 — Supplementary file9 (ZIP 24580 KB) [file 10528_2023_10431_MOESM9_ESM.zip › ╓¬╟Θ═1⁄4╥Γ╩Θ7/╡┌╥╗▓┐╖╓í┐/012.jpg]

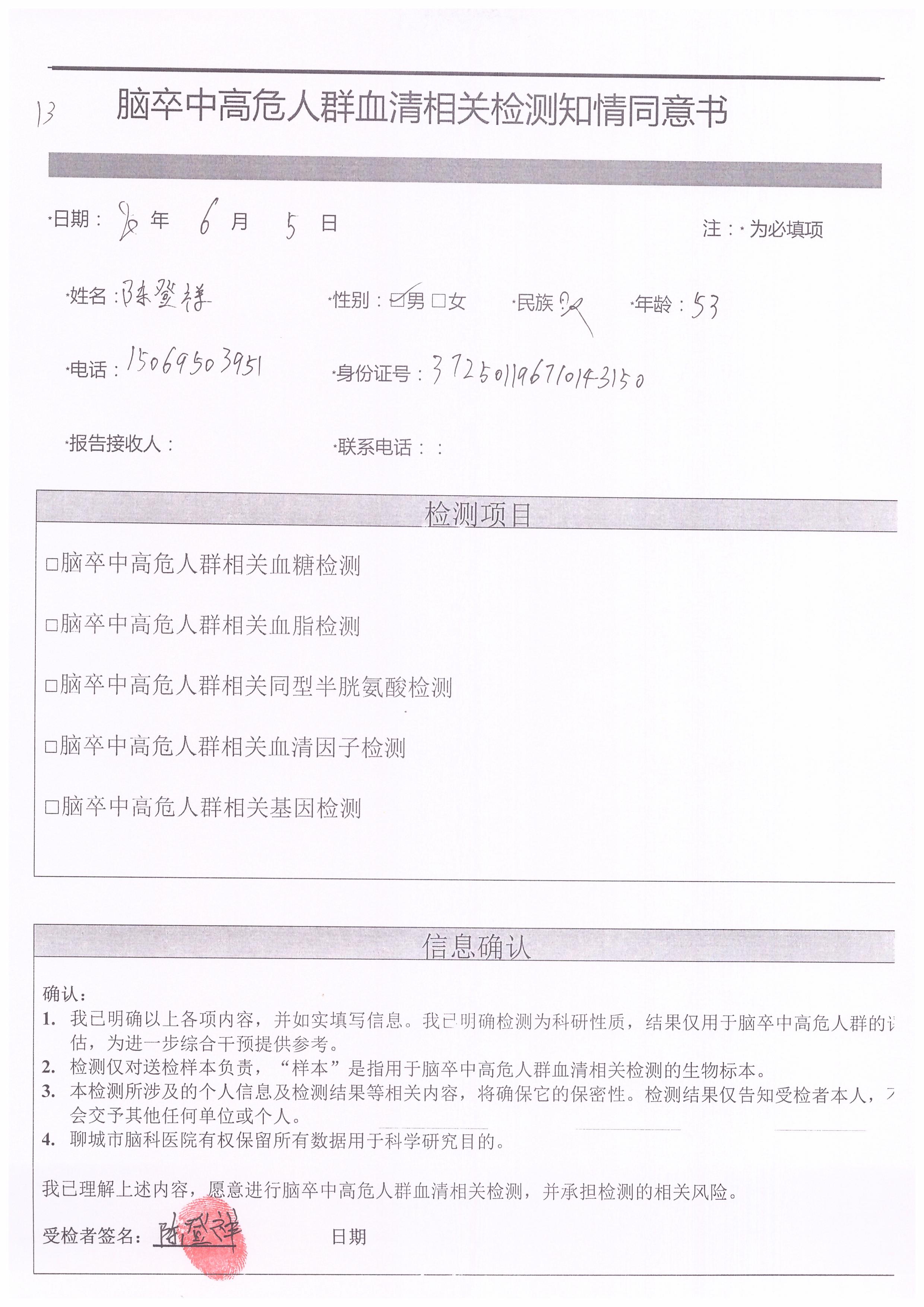

Supplement: Supplementary file 9 — Supplementary file9 (ZIP 24580 KB) [file 10528_2023_10431_MOESM9_ESM.zip › ╓¬╟Θ═1⁄4╥Γ╩Θ7/╡┌╥╗▓┐╖╓í┐/013.jpg]

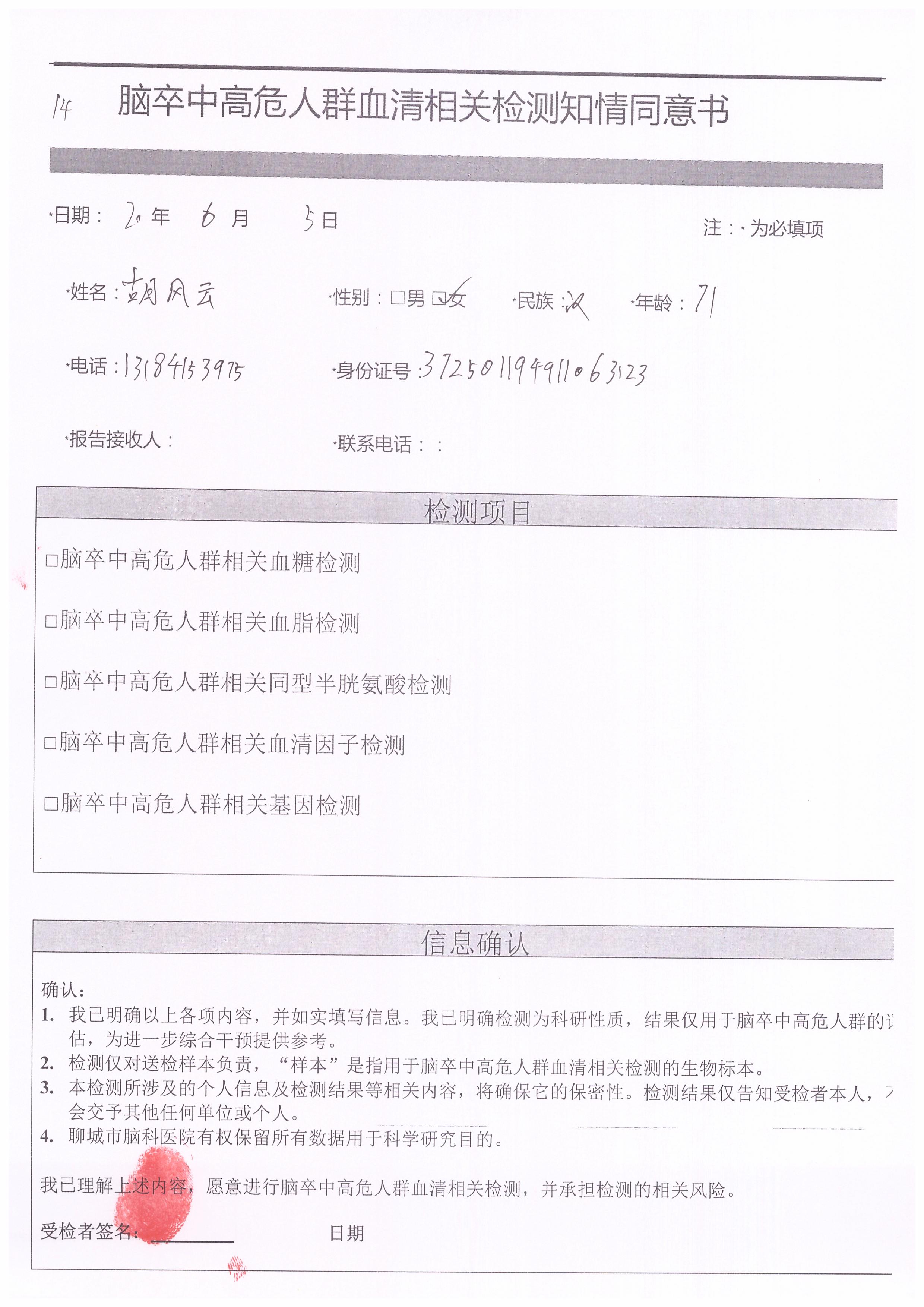

Supplement: Supplementary file 9 — Supplementary file9 (ZIP 24580 KB) [file 10528_2023_10431_MOESM9_ESM.zip › ╓¬╟Θ═1⁄4╥Γ╩Θ7/╡┌╥╗▓┐╖╓í┐/014.jpg]

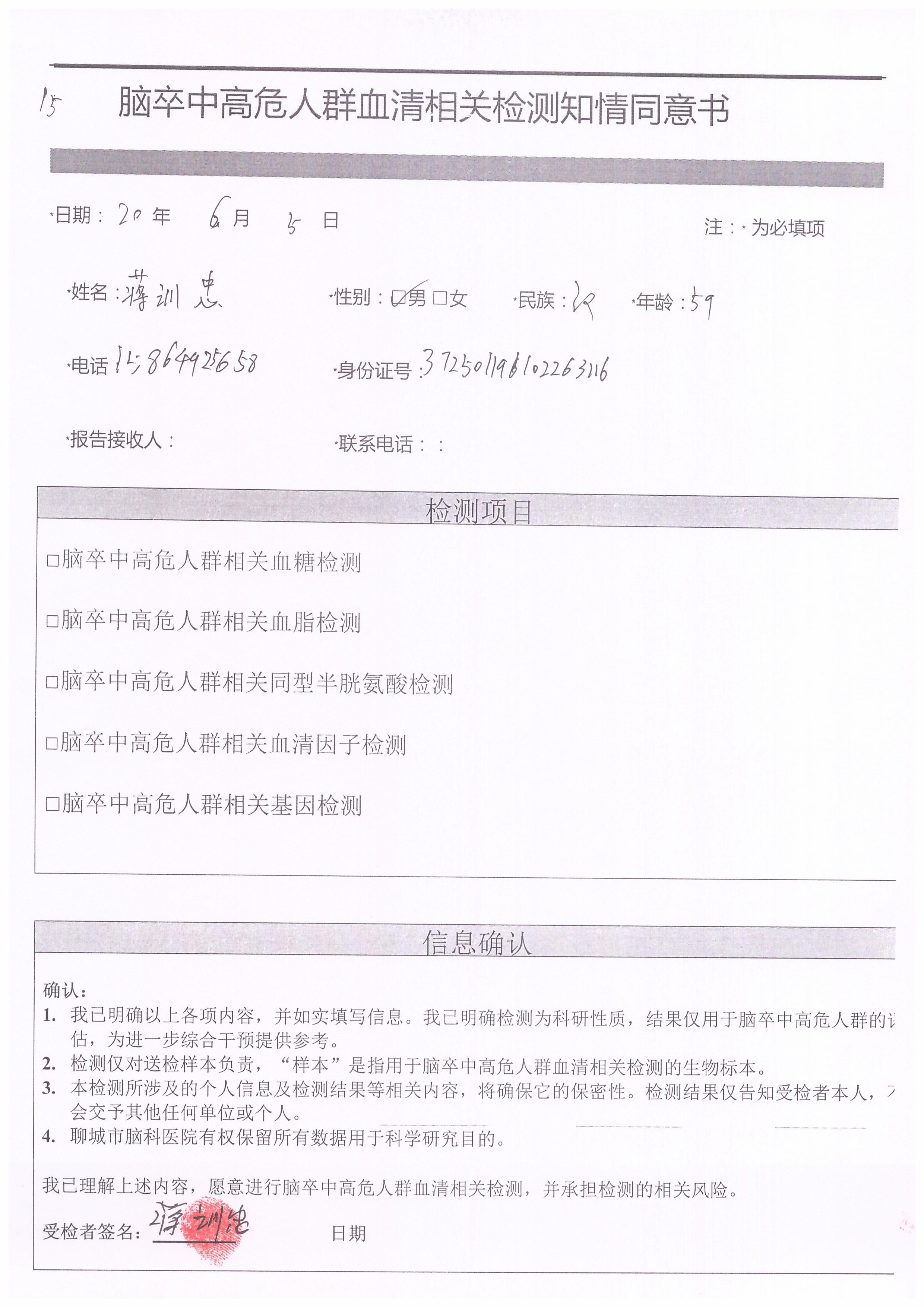

Supplement: Supplementary file 9 — Supplementary file9 (ZIP 24580 KB) [file 10528_2023_10431_MOESM9_ESM.zip › ╓¬╟Θ═1⁄4╥Γ╩Θ7/╡┌╥╗▓┐╖╓í┐/015.jpg]

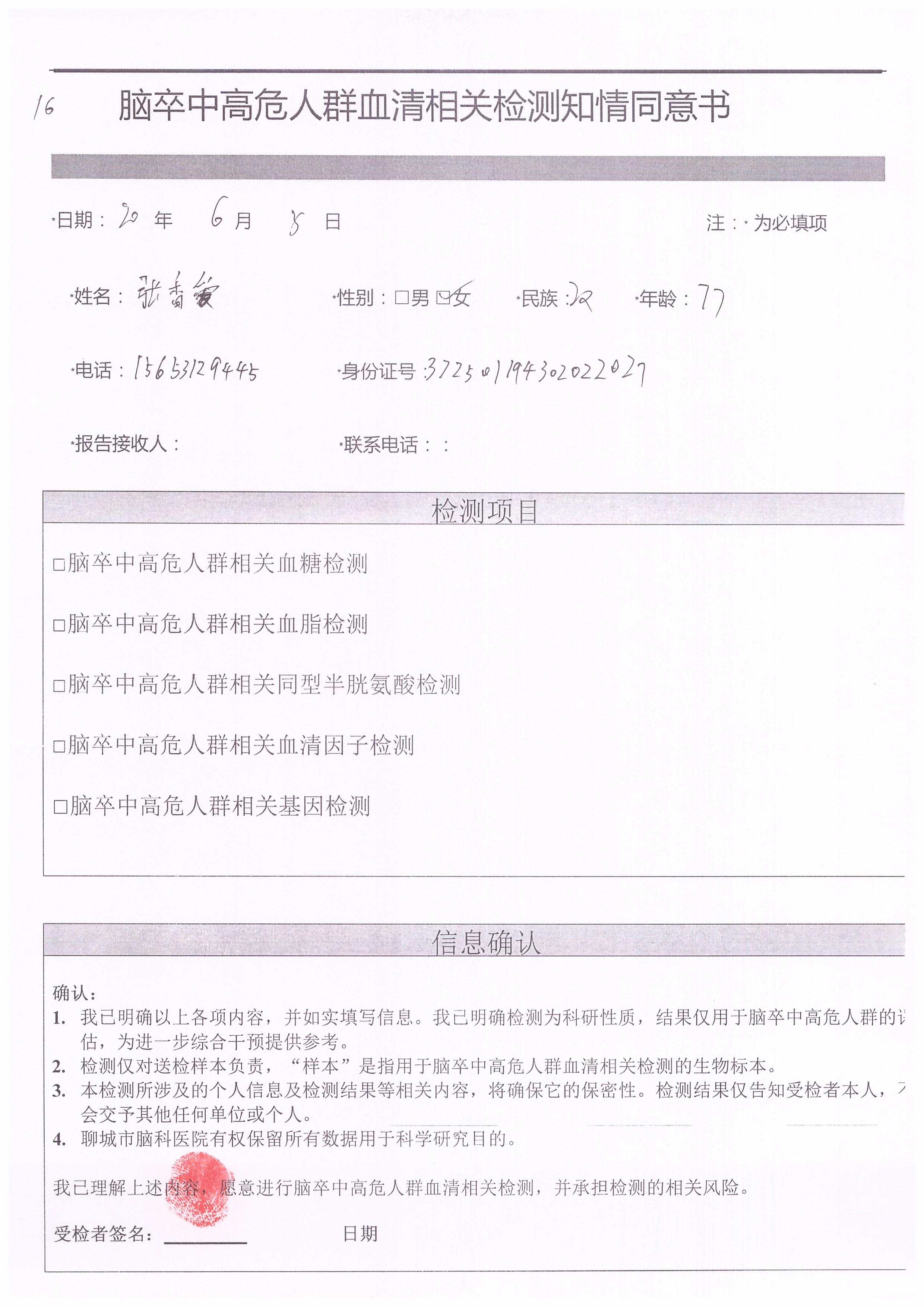

Supplement: Supplementary file 9 — Supplementary file9 (ZIP 24580 KB) [file 10528_2023_10431_MOESM9_ESM.zip › ╓¬╟Θ═1⁄4╥Γ╩Θ7/╡┌╥╗▓┐╖╓í┐/016.jpg]

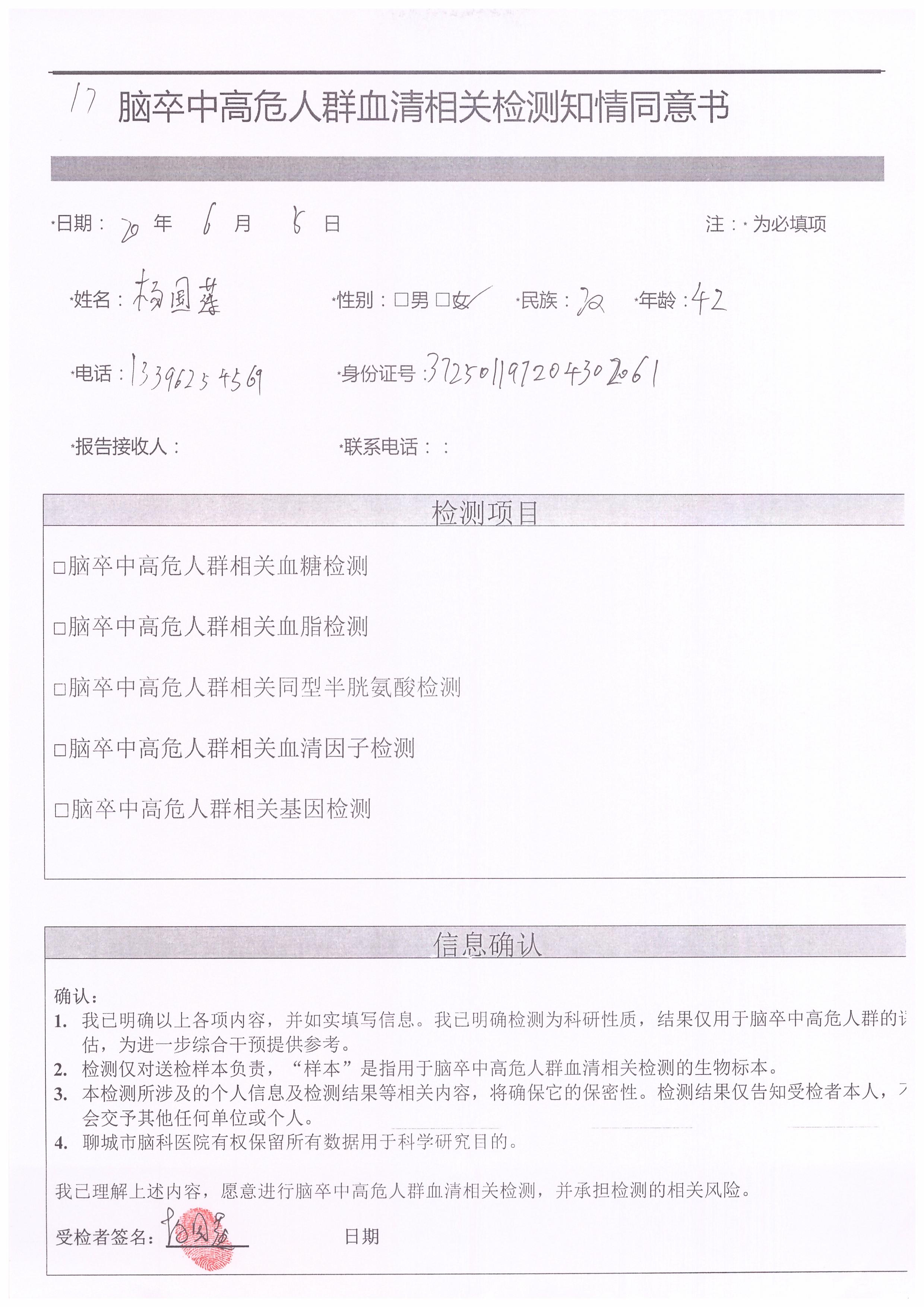

Supplement: Supplementary file 9 — Supplementary file9 (ZIP 24580 KB) [file 10528_2023_10431_MOESM9_ESM.zip › ╓¬╟Θ═1⁄4╥Γ╩Θ7/╡┌╥╗▓┐╖╓í┐/017.jpg]

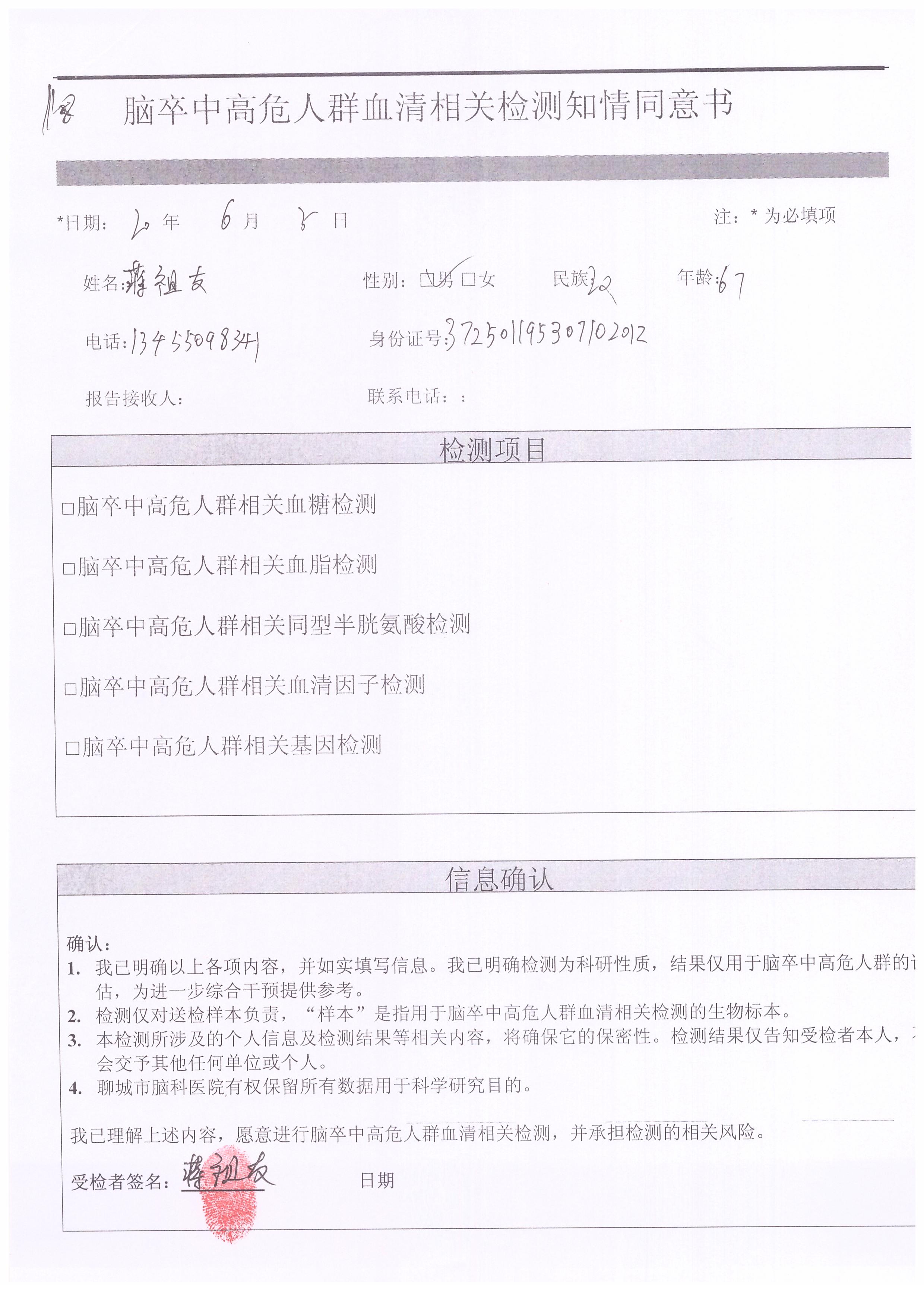

Supplement: Supplementary file 9 — Supplementary file9 (ZIP 24580 KB) [file 10528_2023_10431_MOESM9_ESM.zip › ╓¬╟Θ═1⁄4╥Γ╩Θ7/╡┌╥╗▓┐╖╓í┐/018.jpg]

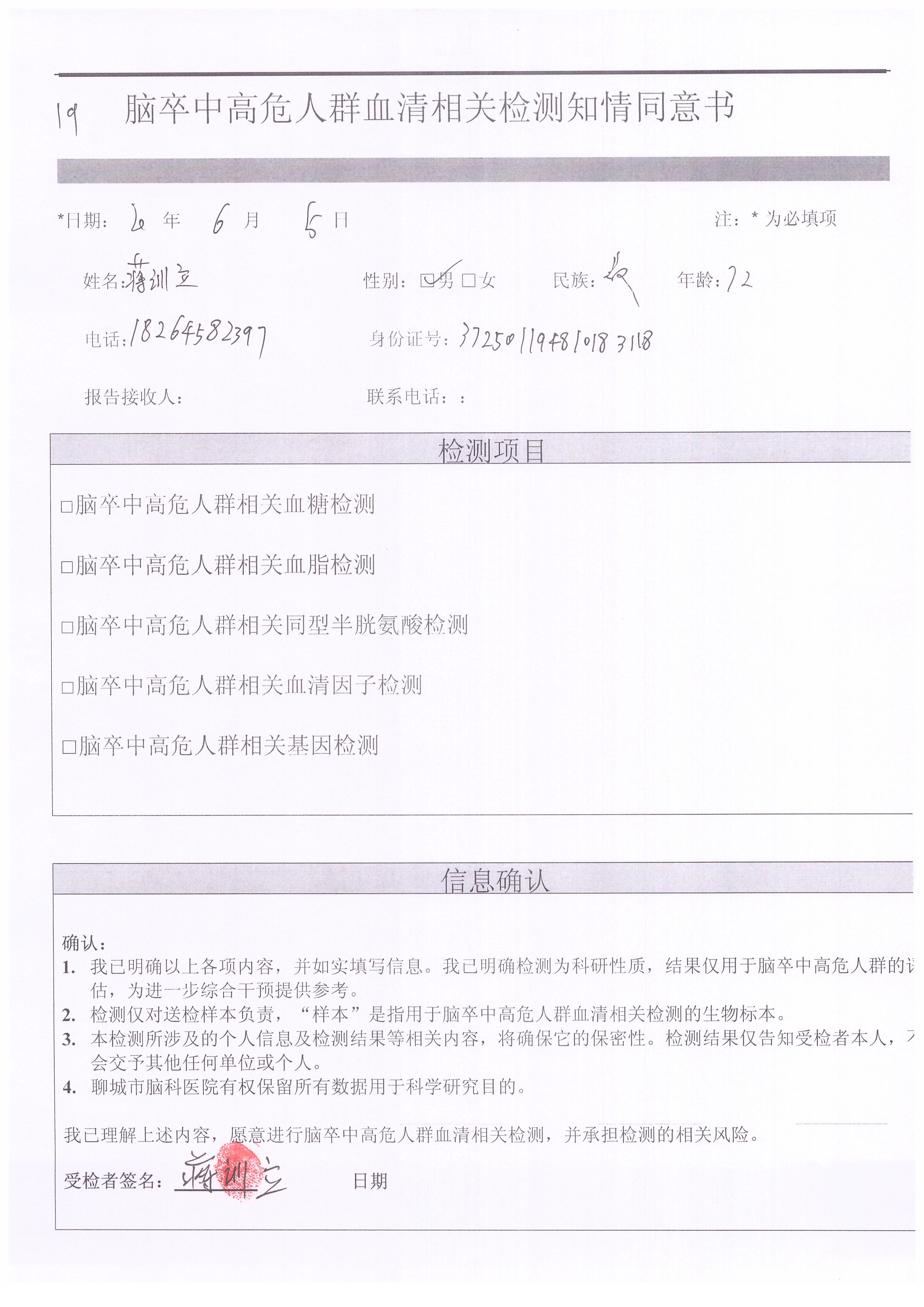

Supplement: Supplementary file 9 — Supplementary file9 (ZIP 24580 KB) [file 10528_2023_10431_MOESM9_ESM.zip › ╓¬╟Θ═1⁄4╥Γ╩Θ7/╡┌╥╗▓┐╖╓í┐/019.jpg]

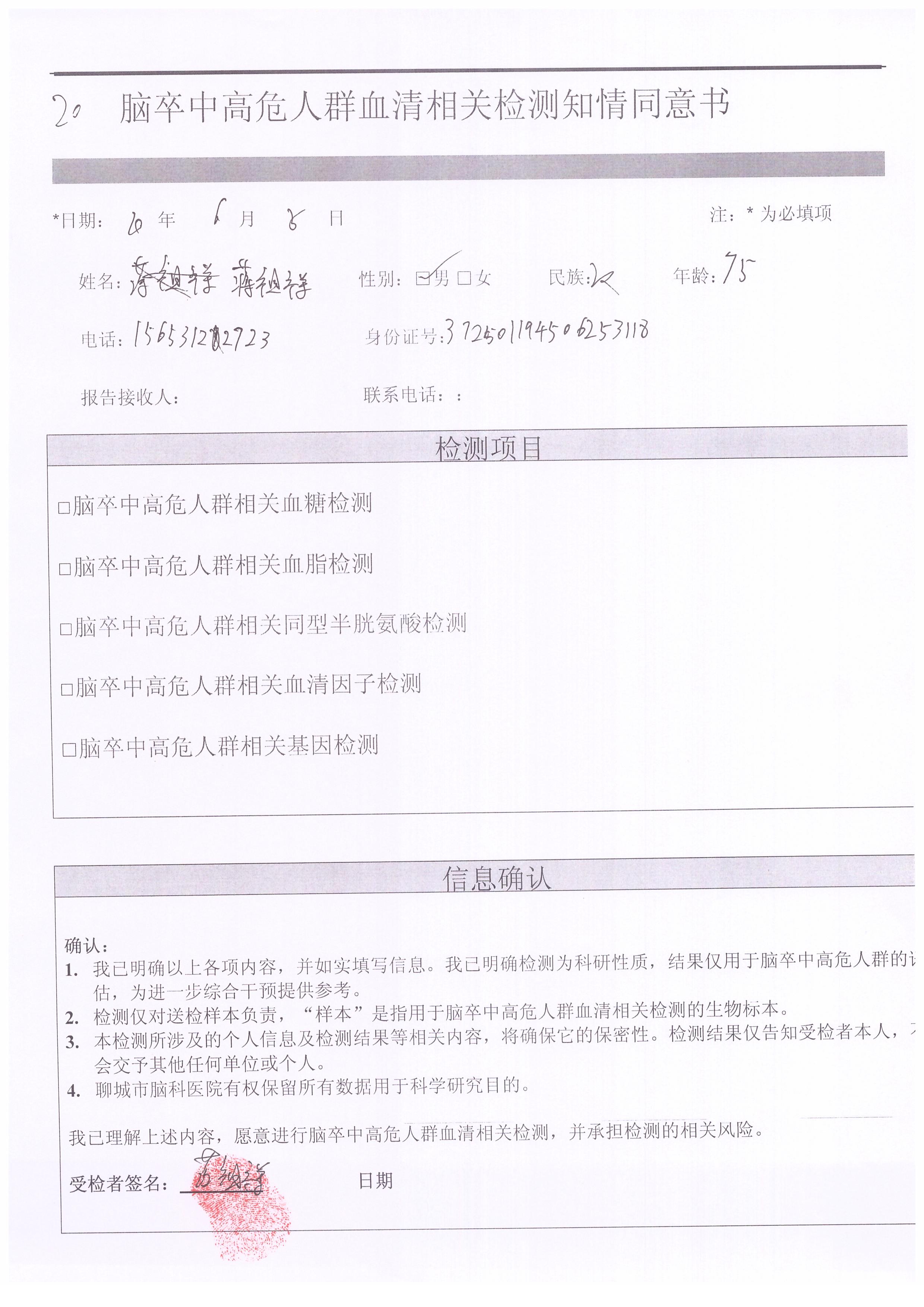

Supplement: Supplementary file 9 — Supplementary file9 (ZIP 24580 KB) [file 10528_2023_10431_MOESM9_ESM.zip › ╓¬╟Θ═1⁄4╥Γ╩Θ7/╡┌╥╗▓┐╖╓í┐/020.jpg]

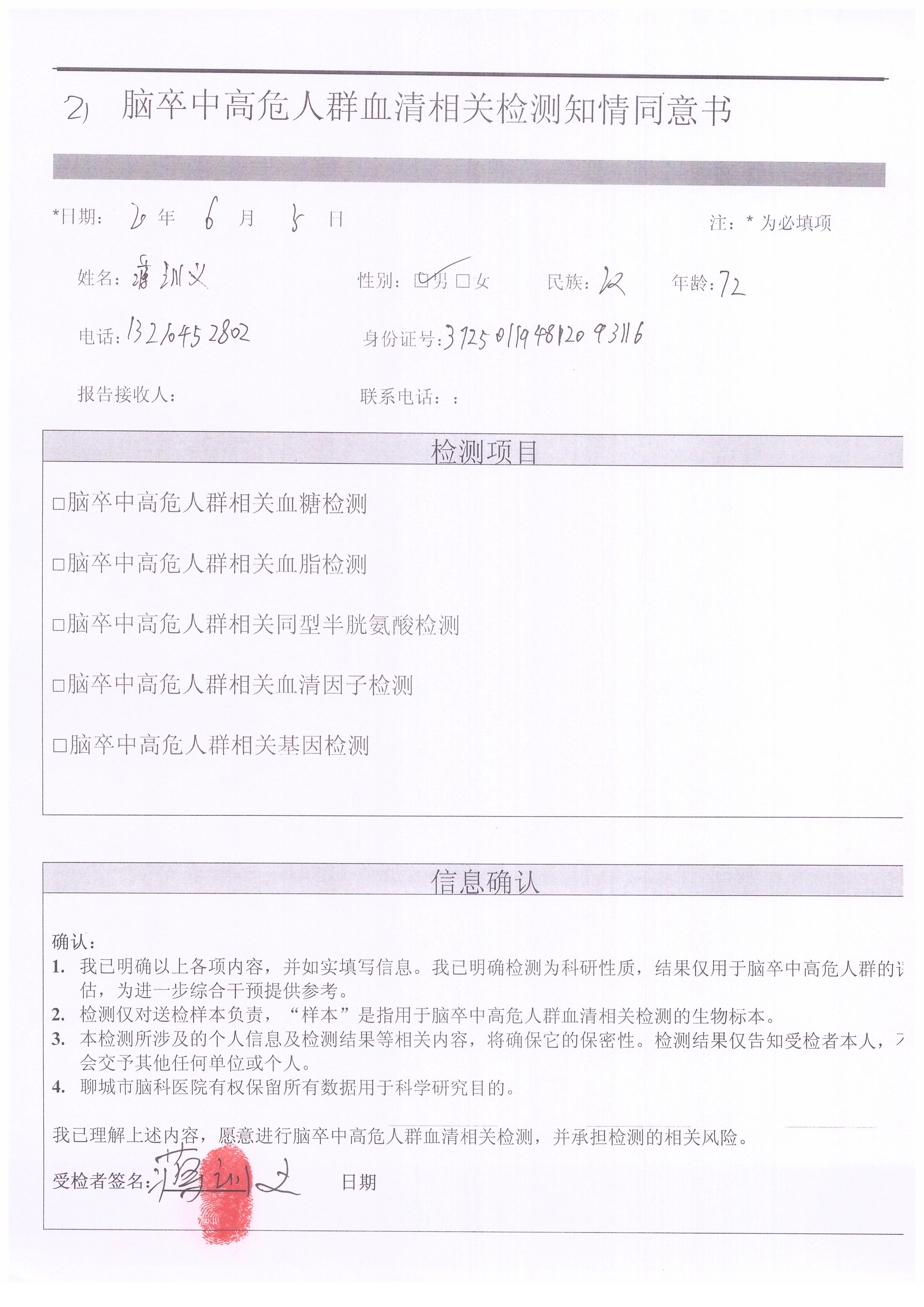

Supplement: Supplementary file 9 — Supplementary file9 (ZIP 24580 KB) [file 10528_2023_10431_MOESM9_ESM.zip › ╓¬╟Θ═1⁄4╥Γ╩Θ7/╡┌╥╗▓┐╖╓í┐/021.jpg]

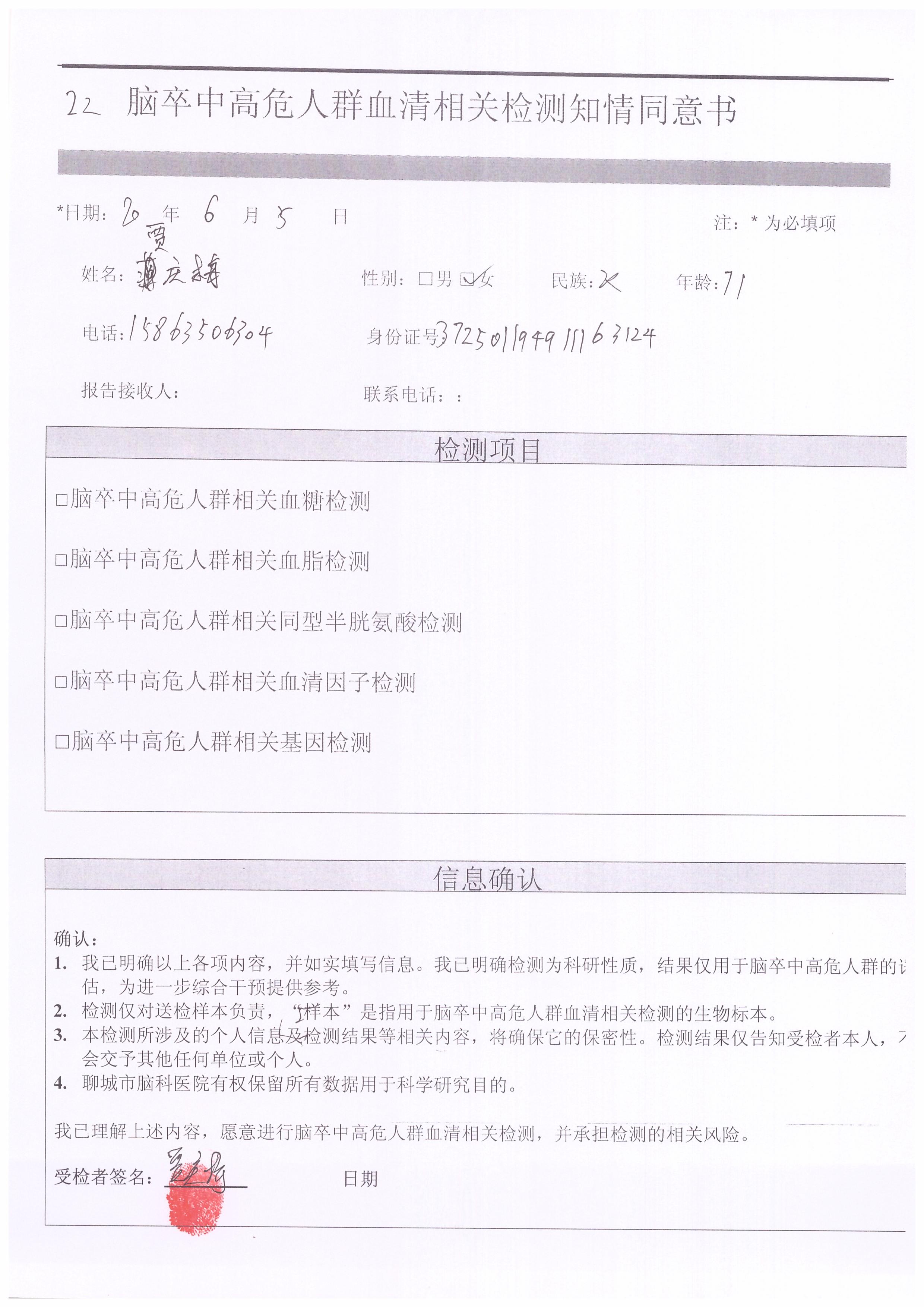

Supplement: Supplementary file 9 — Supplementary file9 (ZIP 24580 KB) [file 10528_2023_10431_MOESM9_ESM.zip › ╓¬╟Θ═1⁄4╥Γ╩Θ7/╡┌╥╗▓┐╖╓í┐/022.jpg]

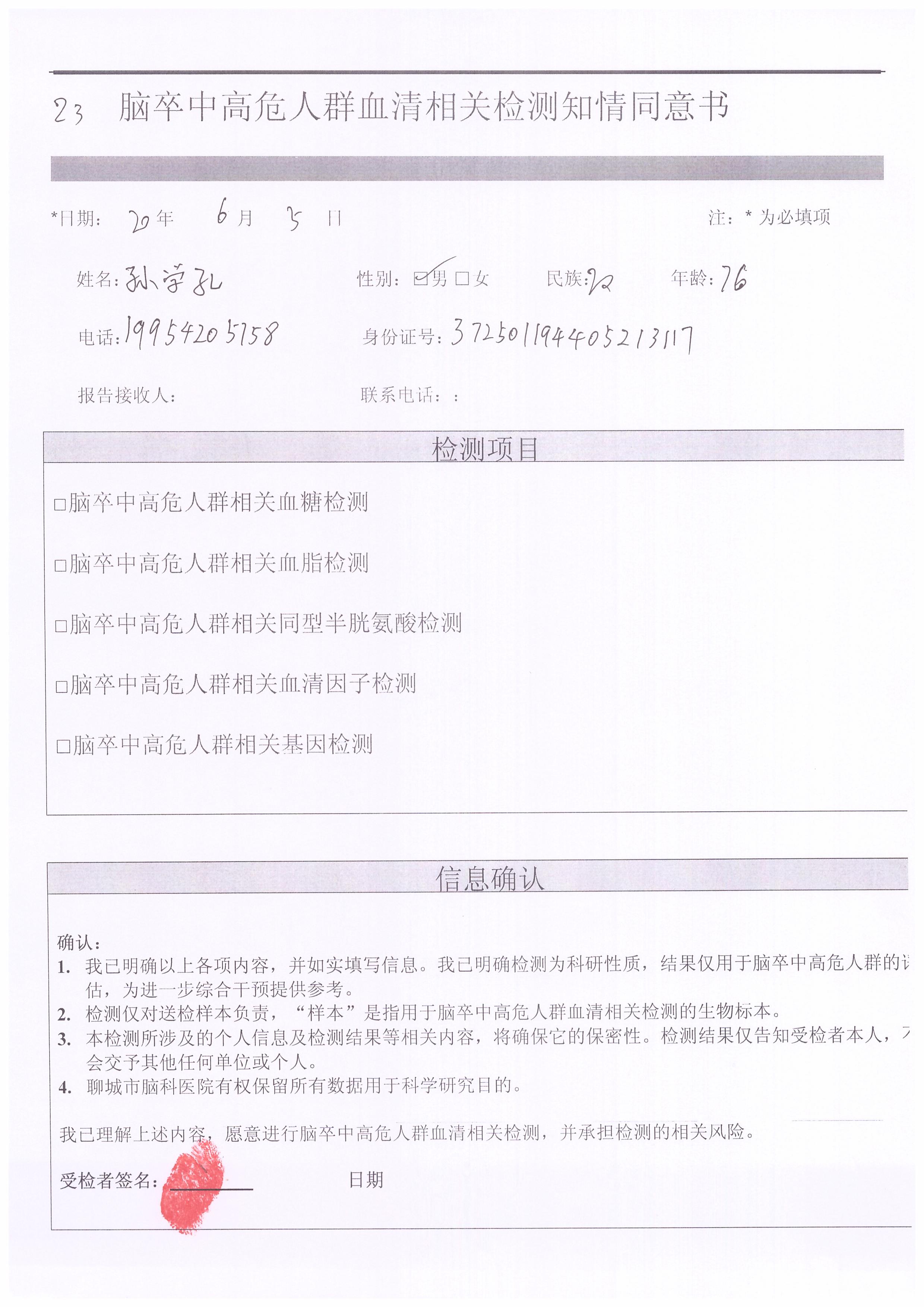

Supplement: Supplementary file 9 — Supplementary file9 (ZIP 24580 KB) [file 10528_2023_10431_MOESM9_ESM.zip › ╓¬╟Θ═1⁄4╥Γ╩Θ7/╡┌╥╗▓┐╖╓í┐/023.jpg]

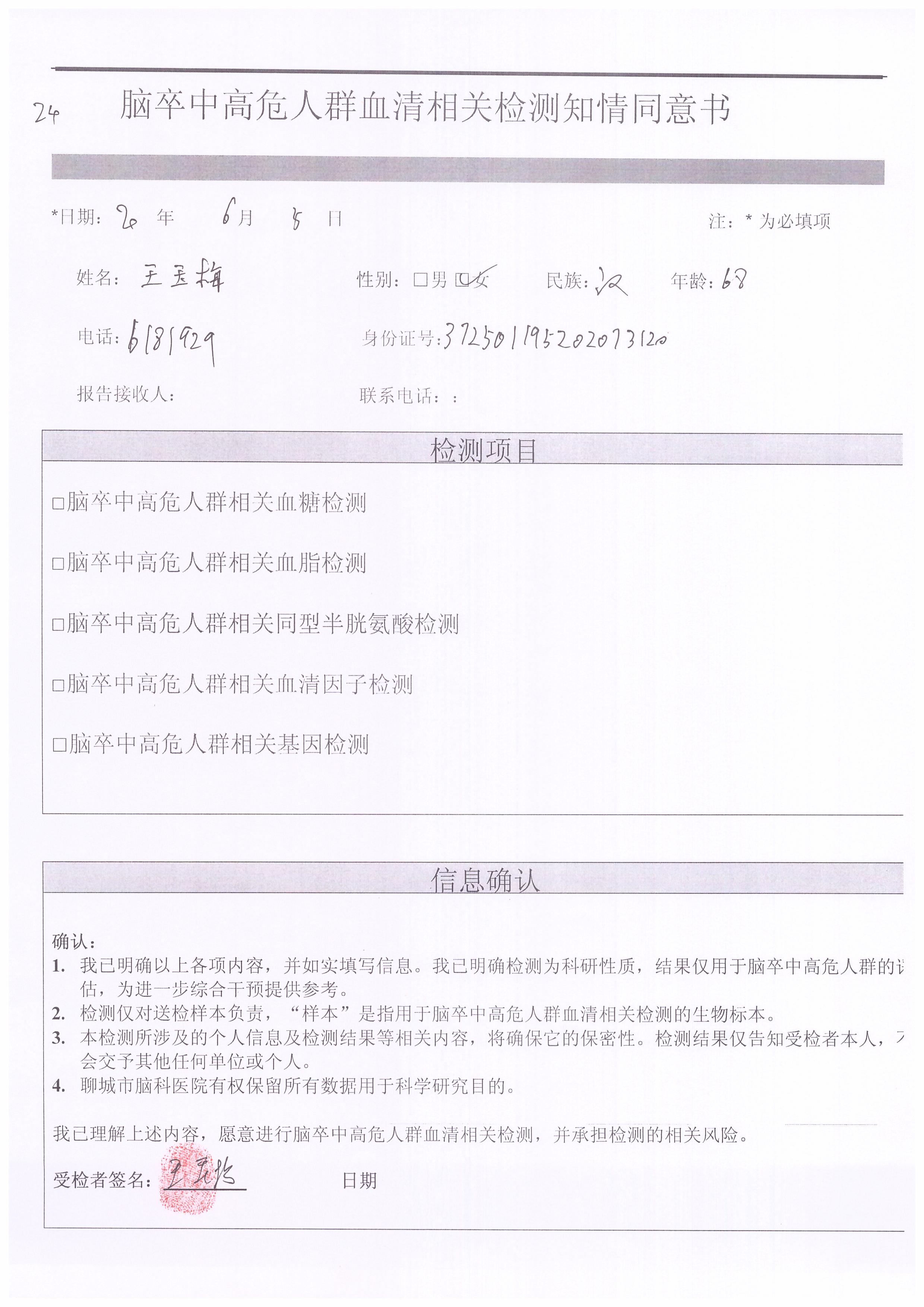

Supplement: Supplementary file 9 — Supplementary file9 (ZIP 24580 KB) [file 10528_2023_10431_MOESM9_ESM.zip › ╓¬╟Θ═1⁄4╥Γ╩Θ7/╡┌╥╗▓┐╖╓í┐/024.jpg]

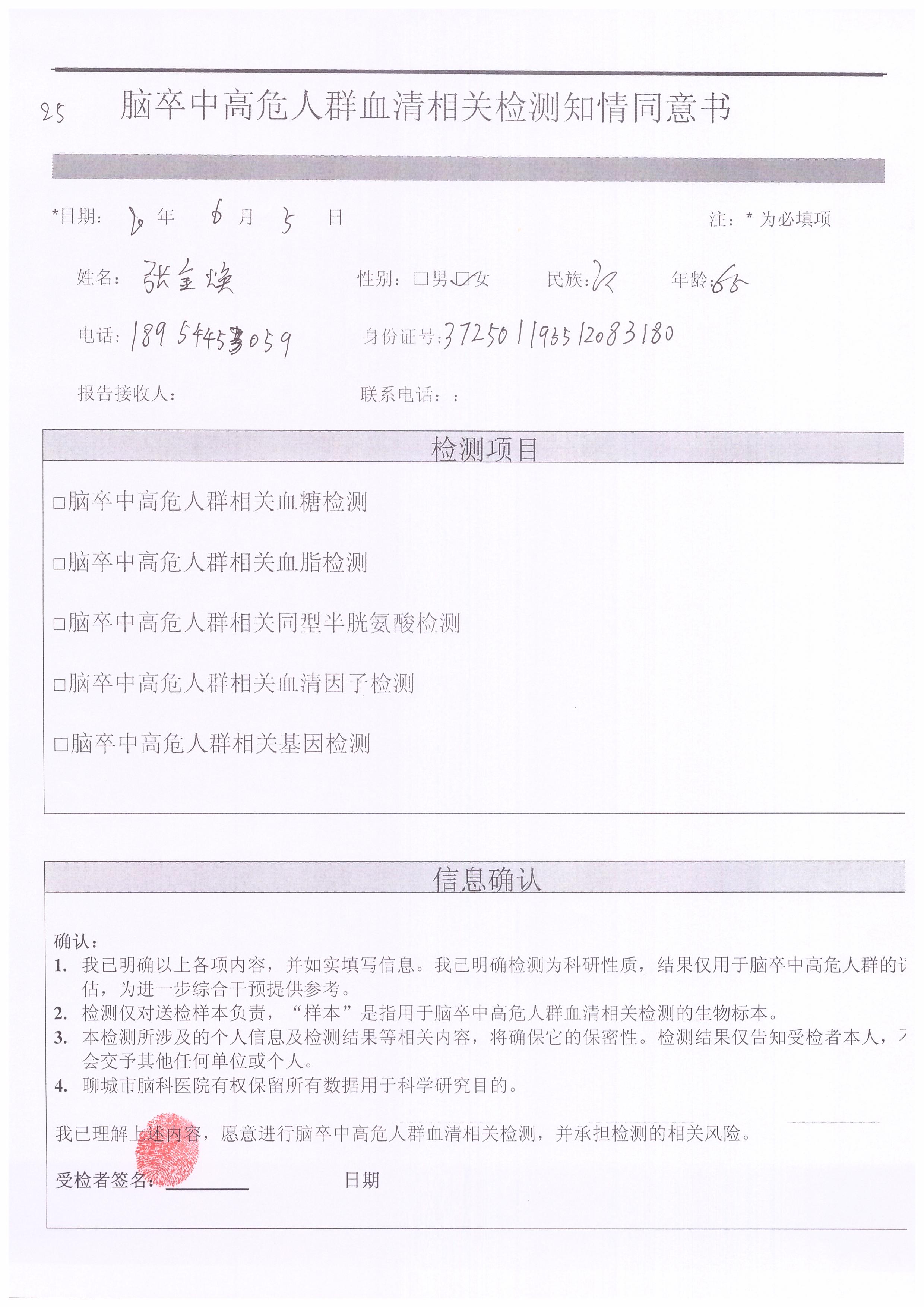

Supplement: Supplementary file 9 — Supplementary file9 (ZIP 24580 KB) [file 10528_2023_10431_MOESM9_ESM.zip › ╓¬╟Θ═1⁄4╥Γ╩Θ7/╡┌╥╗▓┐╖╓í┐/025.jpg]

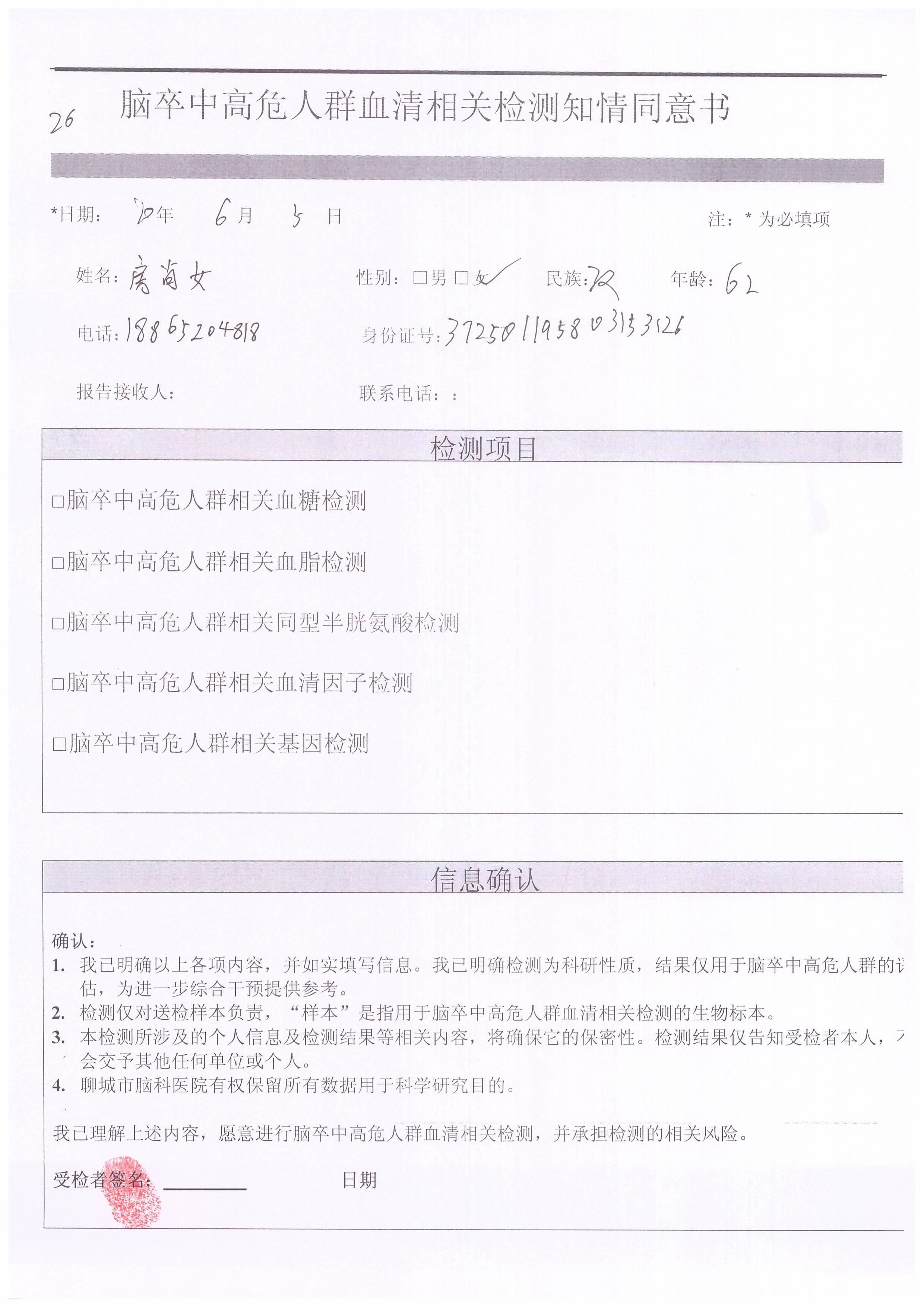

Supplement: Supplementary file 9 — Supplementary file9 (ZIP 24580 KB) [file 10528_2023_10431_MOESM9_ESM.zip › ╓¬╟Θ═1⁄4╥Γ╩Θ7/╡┌╥╗▓┐╖╓í┐/026.jpg]

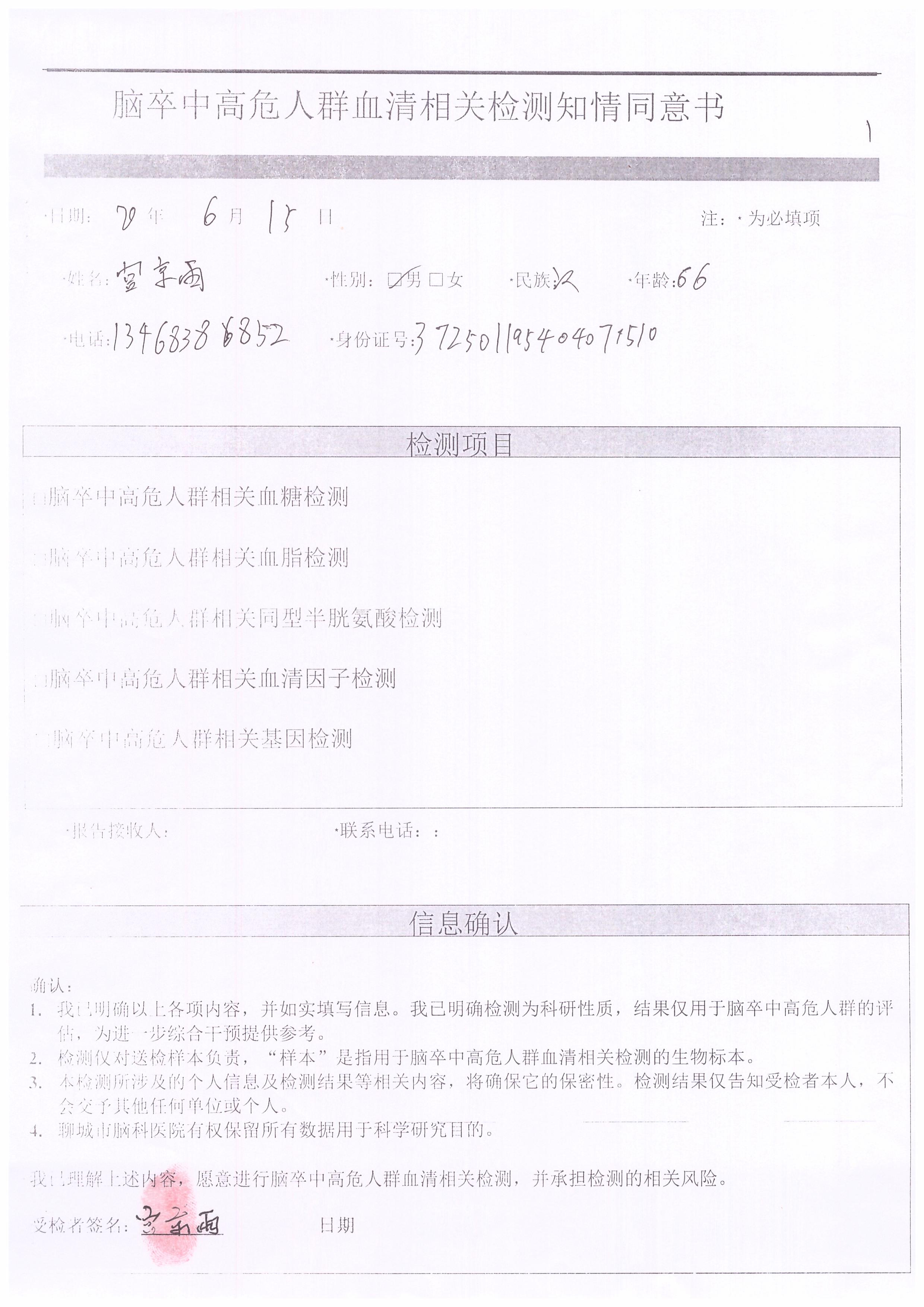

Supplement: Supplementary file 10 — Supplementary file10 (ZIP 21741 KB) [file 10528_2023_10431_MOESM10_ESM.zip › ╓¬╟Θ═1⁄4╥Γ╩Θ8/001.jpg]

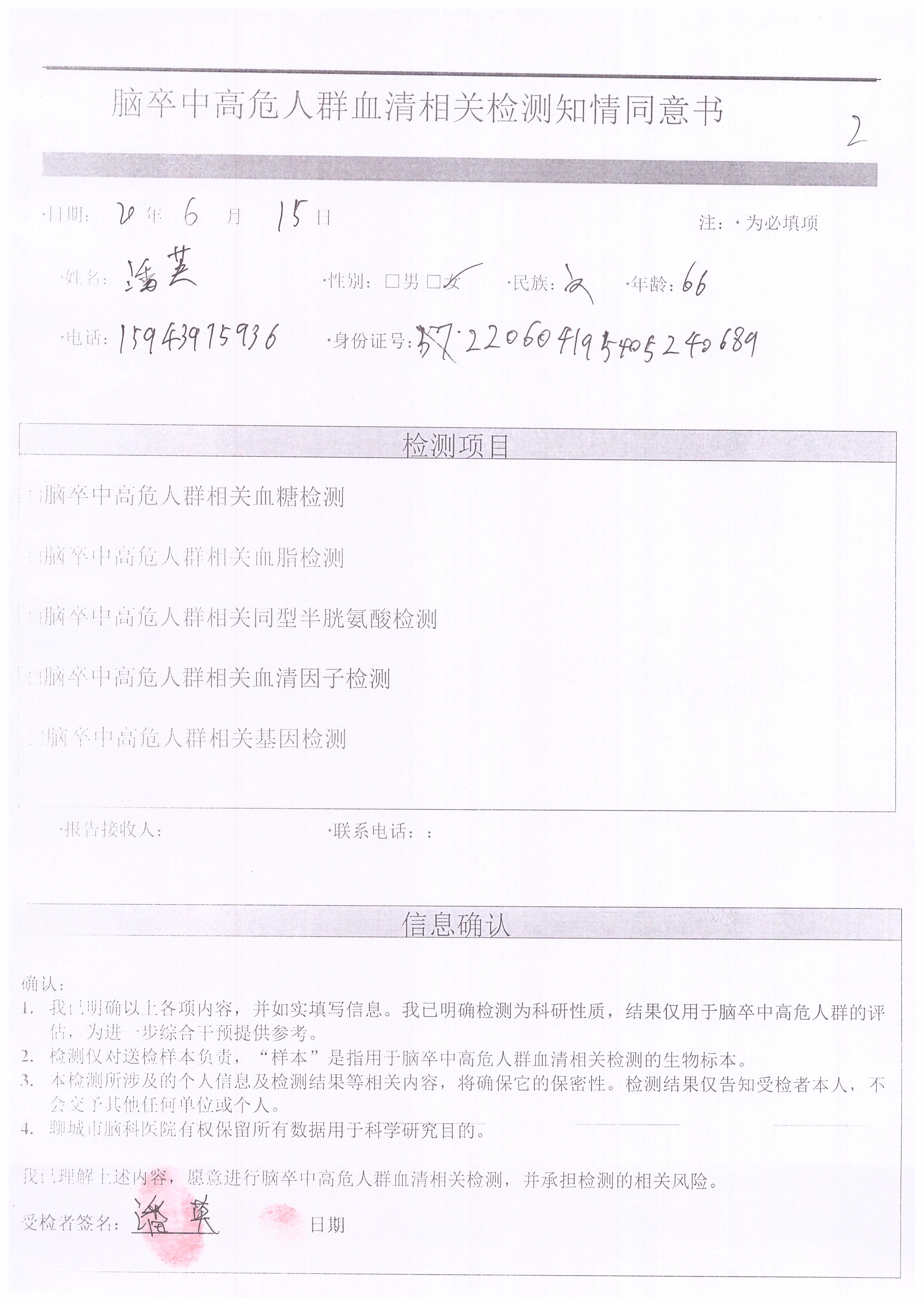

Supplement: Supplementary file 10 — Supplementary file10 (ZIP 21741 KB) [file 10528_2023_10431_MOESM10_ESM.zip › ╓¬╟Θ═1⁄4╥Γ╩Θ8/002.jpg]

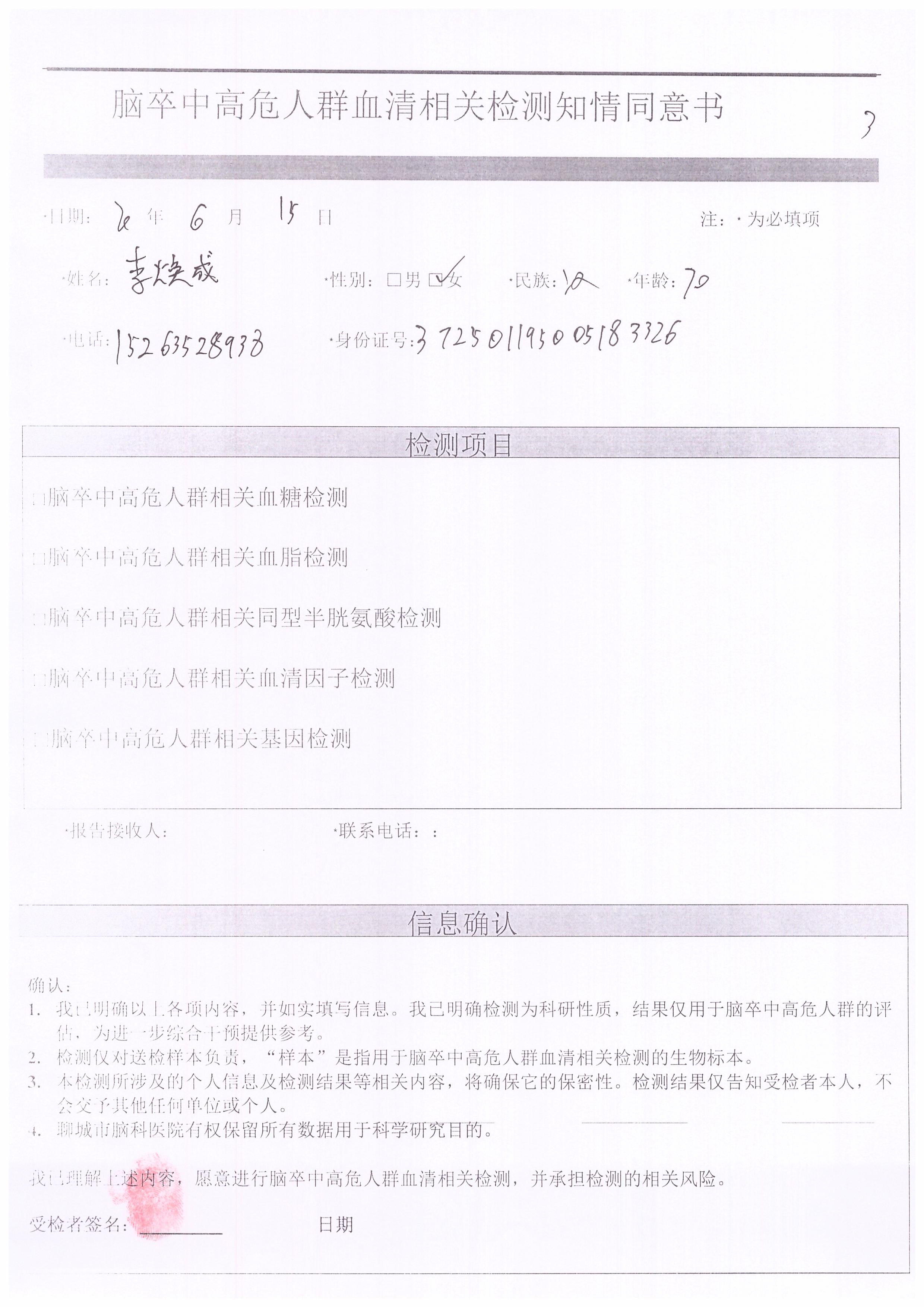

Supplement: Supplementary file 10 — Supplementary file10 (ZIP 21741 KB) [file 10528_2023_10431_MOESM10_ESM.zip › ╓¬╟Θ═1⁄4╥Γ╩Θ8/003.jpg]

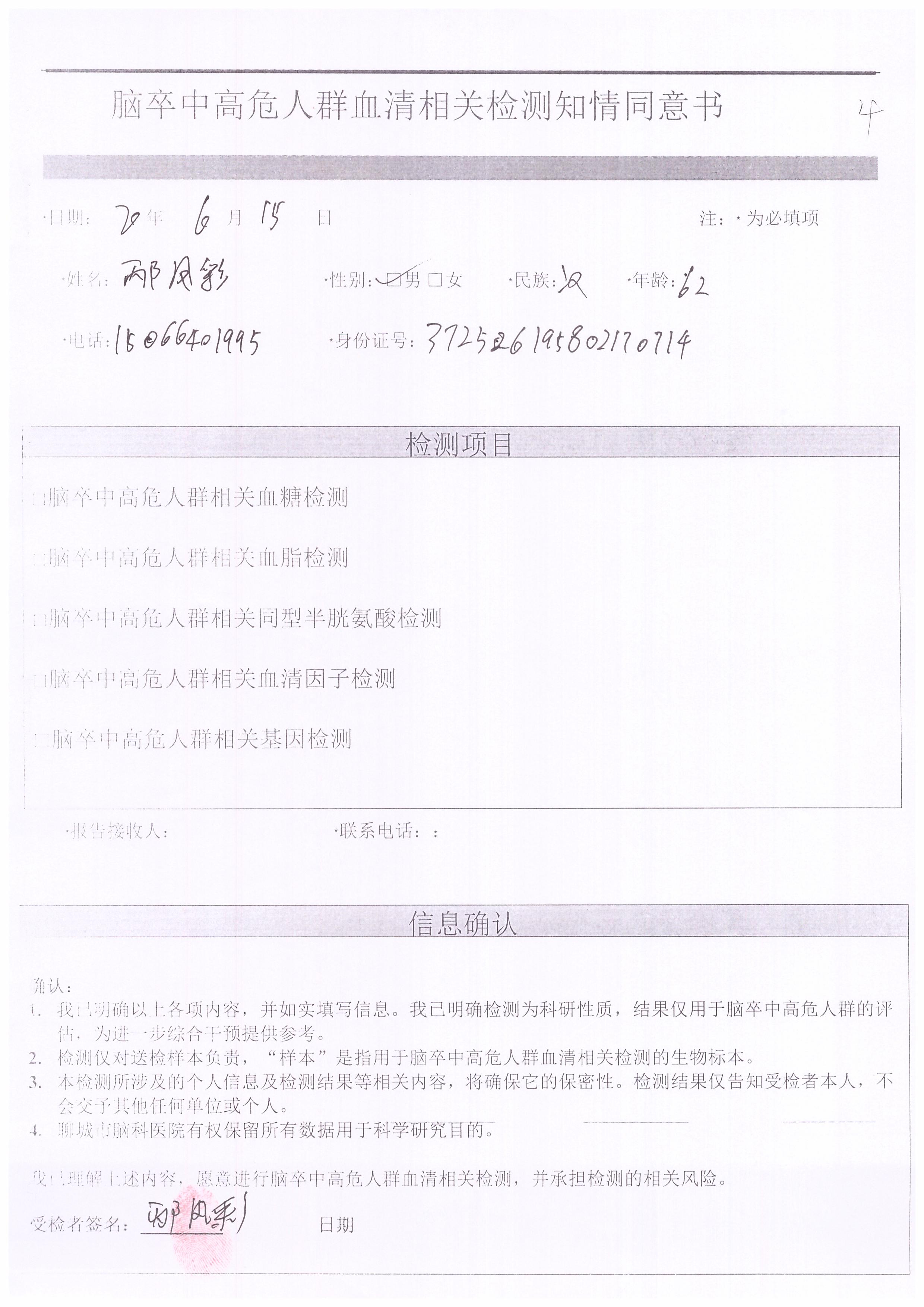

Supplement: Supplementary file 10 — Supplementary file10 (ZIP 21741 KB) [file 10528_2023_10431_MOESM10_ESM.zip › ╓¬╟Θ═1⁄4╥Γ╩Θ8/004.jpg]

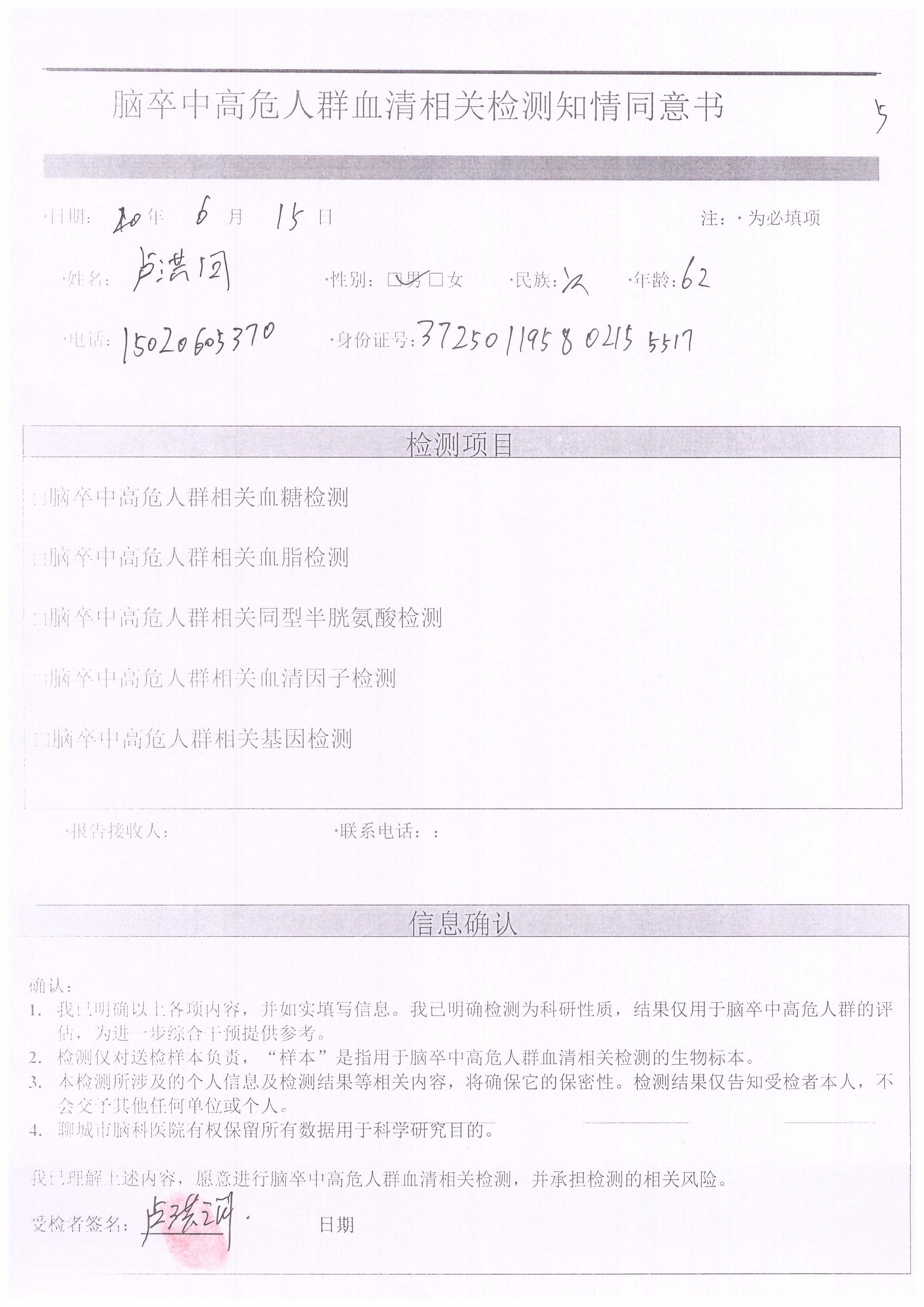

Supplement: Supplementary file 10 — Supplementary file10 (ZIP 21741 KB) [file 10528_2023_10431_MOESM10_ESM.zip › ╓¬╟Θ═1⁄4╥Γ╩Θ8/005.jpg]

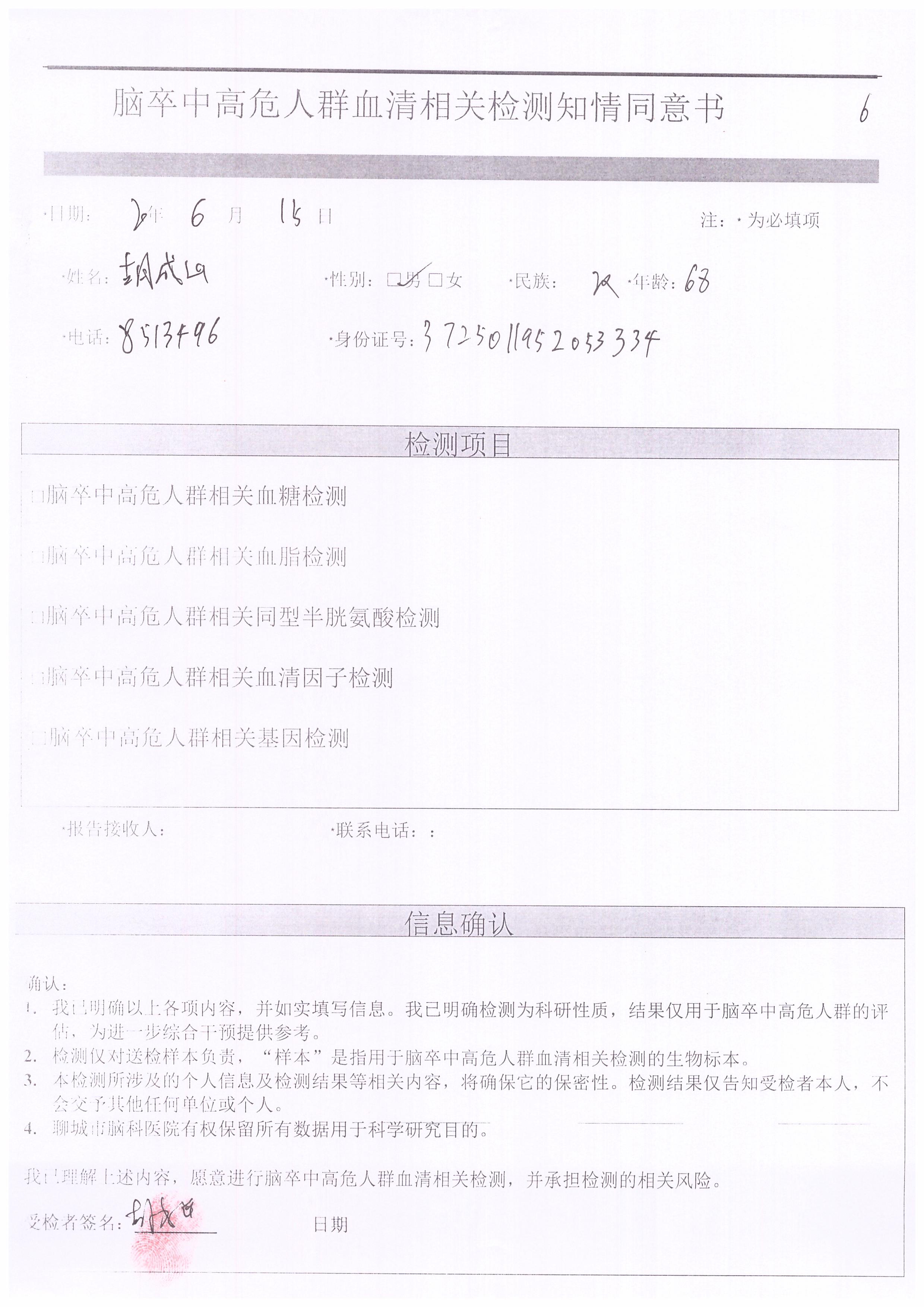

Supplement: Supplementary file 10 — Supplementary file10 (ZIP 21741 KB) [file 10528_2023_10431_MOESM10_ESM.zip › ╓¬╟Θ═1⁄4╥Γ╩Θ8/006.jpg]

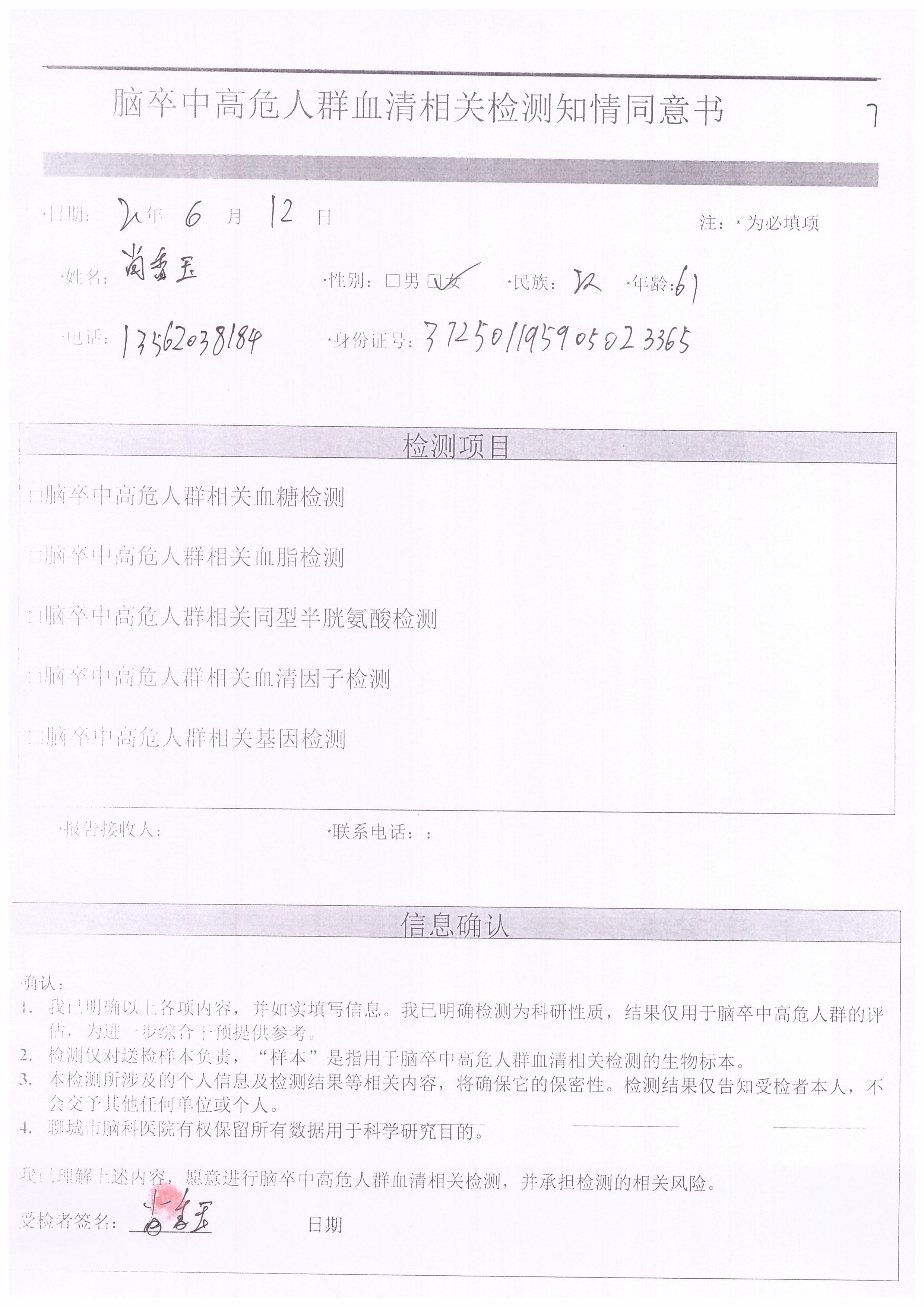

Supplement: Supplementary file 10 — Supplementary file10 (ZIP 21741 KB) [file 10528_2023_10431_MOESM10_ESM.zip › ╓¬╟Θ═1⁄4╥Γ╩Θ8/007.jpg]

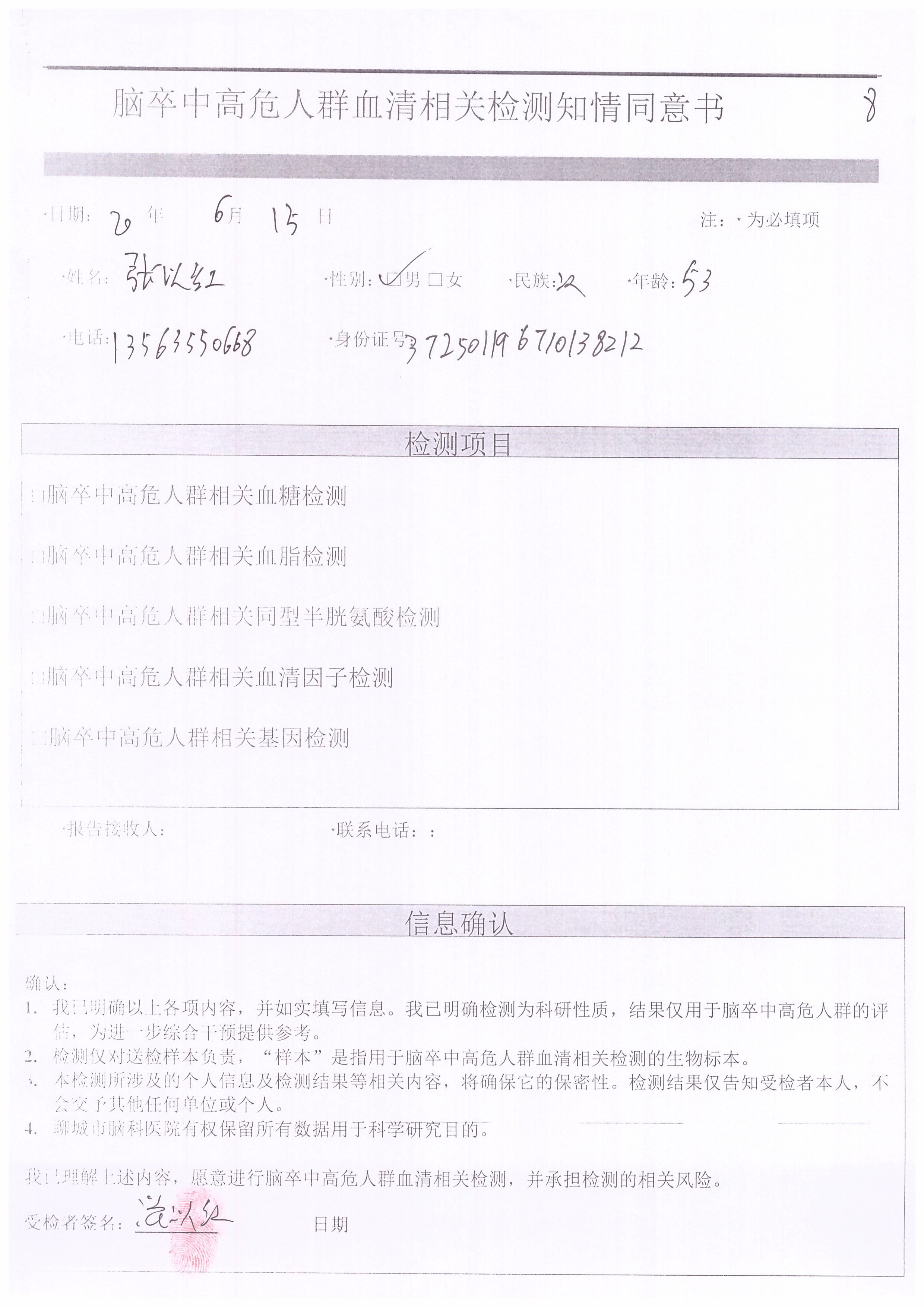

Supplement: Supplementary file 10 — Supplementary file10 (ZIP 21741 KB) [file 10528_2023_10431_MOESM10_ESM.zip › ╓¬╟Θ═1⁄4╥Γ╩Θ8/008.jpg]

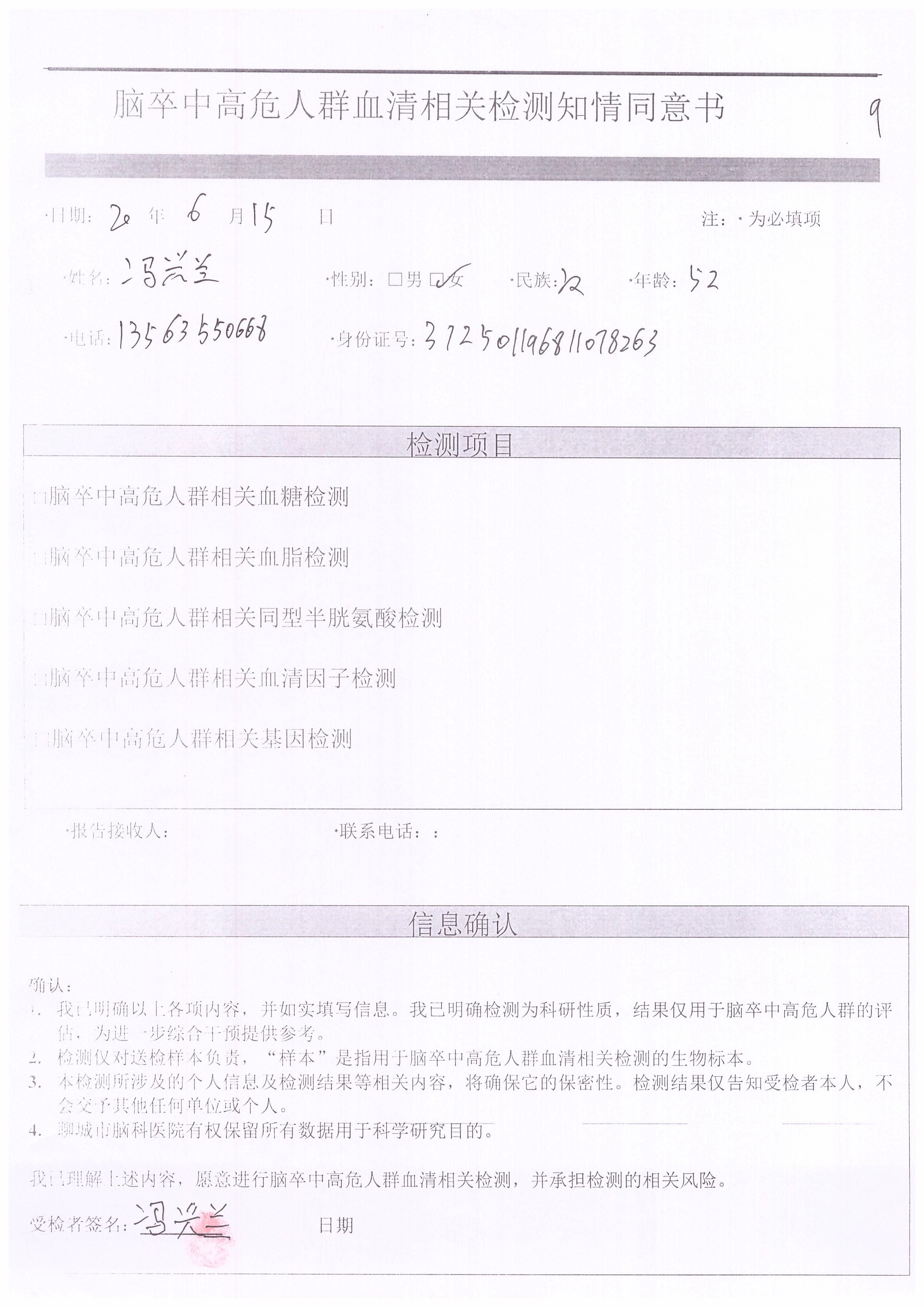

Supplement: Supplementary file 10 — Supplementary file10 (ZIP 21741 KB) [file 10528_2023_10431_MOESM10_ESM.zip › ╓¬╟Θ═1⁄4╥Γ╩Θ8/009.jpg]

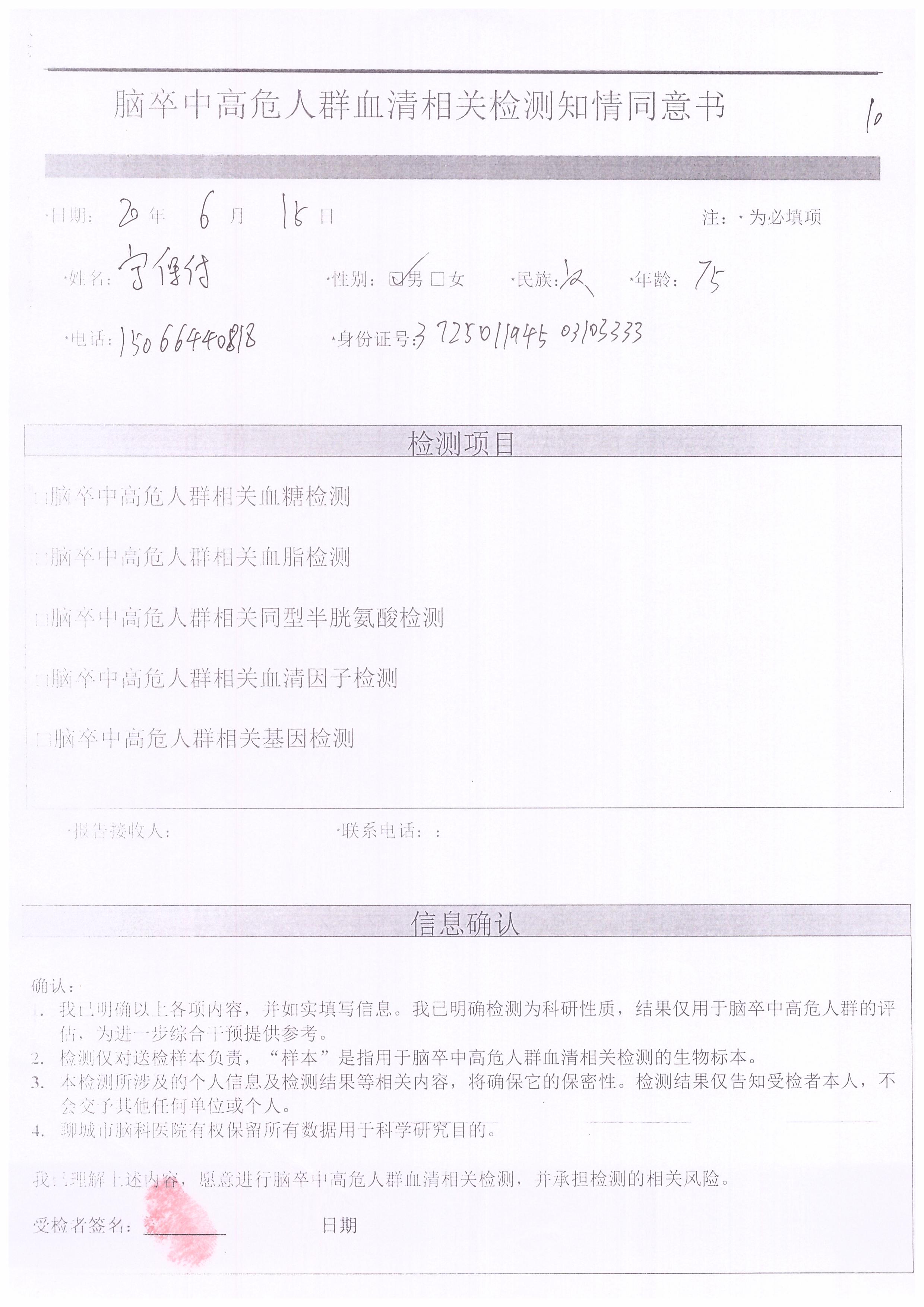

Supplement: Supplementary file 10 — Supplementary file10 (ZIP 21741 KB) [file 10528_2023_10431_MOESM10_ESM.zip › ╓¬╟Θ═1⁄4╥Γ╩Θ8/010.jpg]

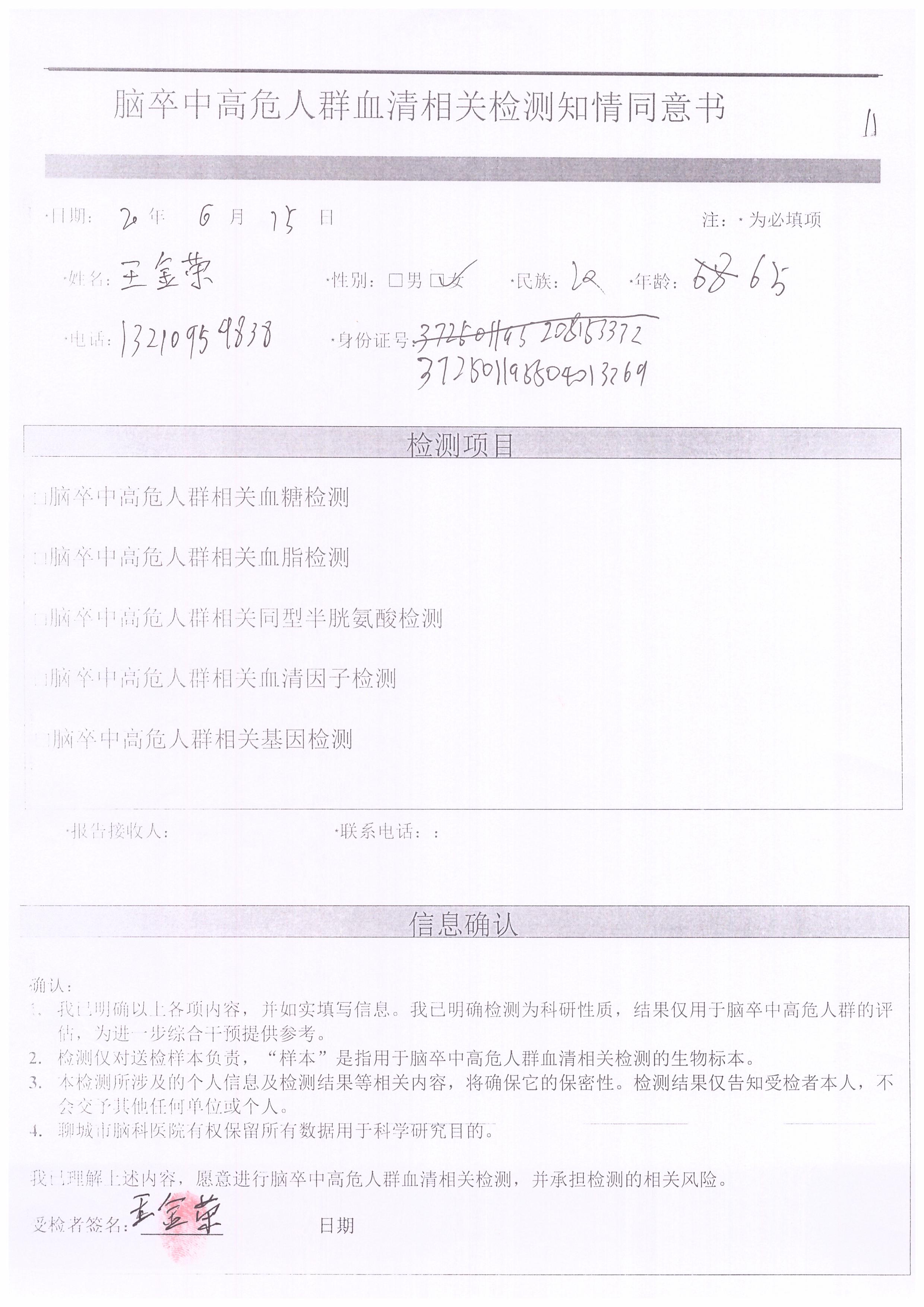

Supplement: Supplementary file 10 — Supplementary file10 (ZIP 21741 KB) [file 10528_2023_10431_MOESM10_ESM.zip › ╓¬╟Θ═1⁄4╥Γ╩Θ8/011.jpg]

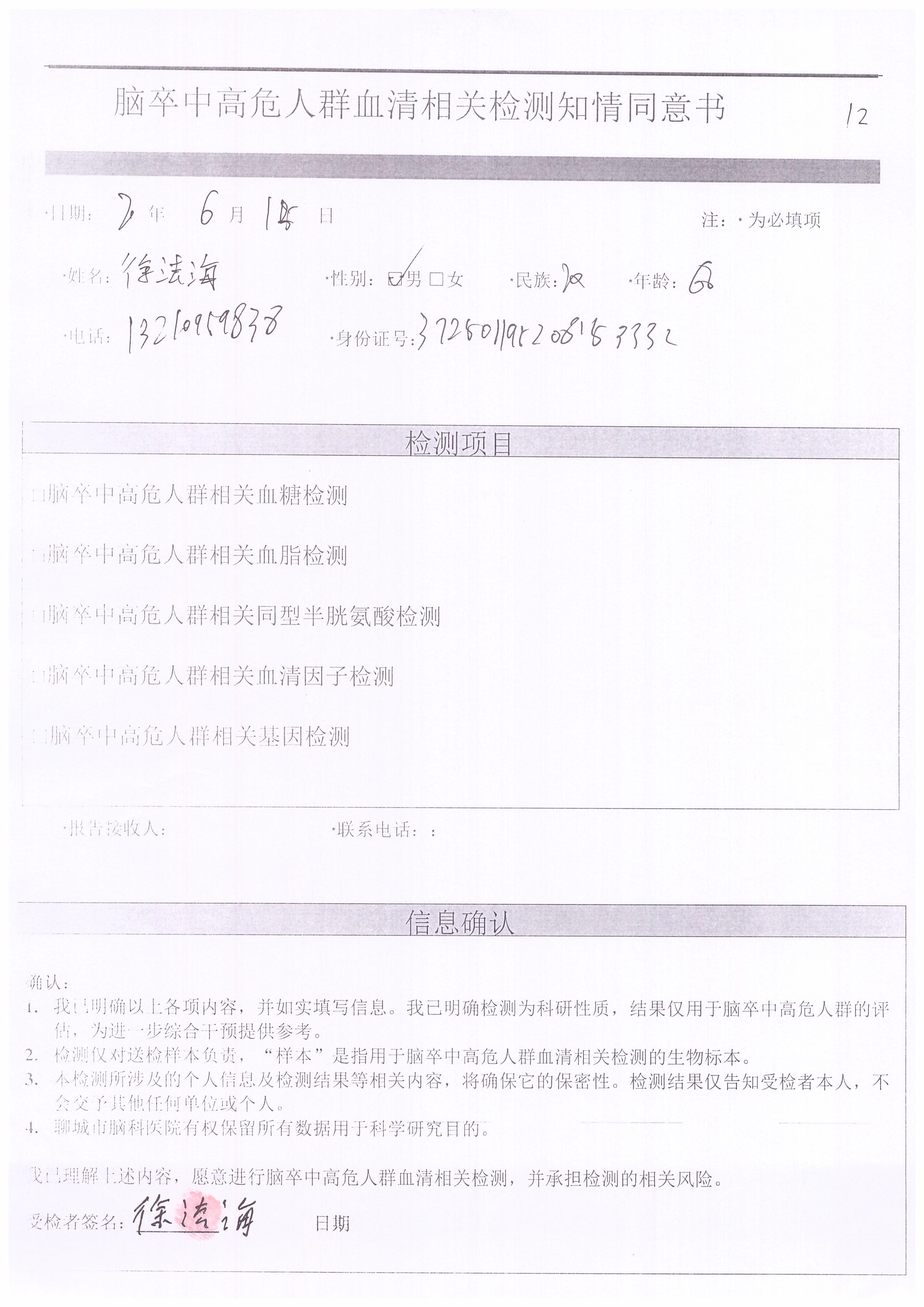

Supplement: Supplementary file 10 — Supplementary file10 (ZIP 21741 KB) [file 10528_2023_10431_MOESM10_ESM.zip › ╓¬╟Θ═1⁄4╥Γ╩Θ8/012.jpg]

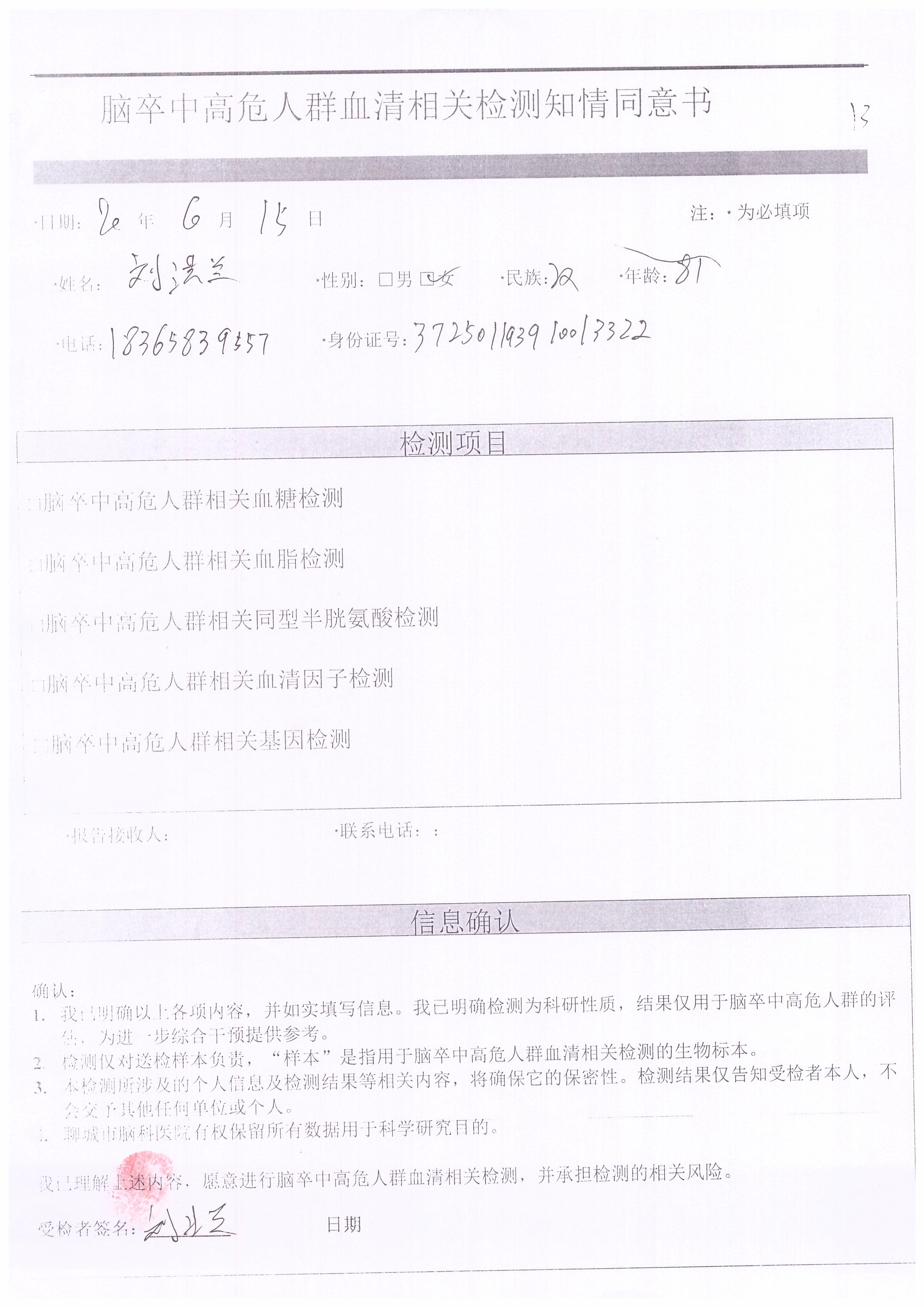

Supplement: Supplementary file 10 — Supplementary file10 (ZIP 21741 KB) [file 10528_2023_10431_MOESM10_ESM.zip › ╓¬╟Θ═1⁄4╥Γ╩Θ8/013.jpg]

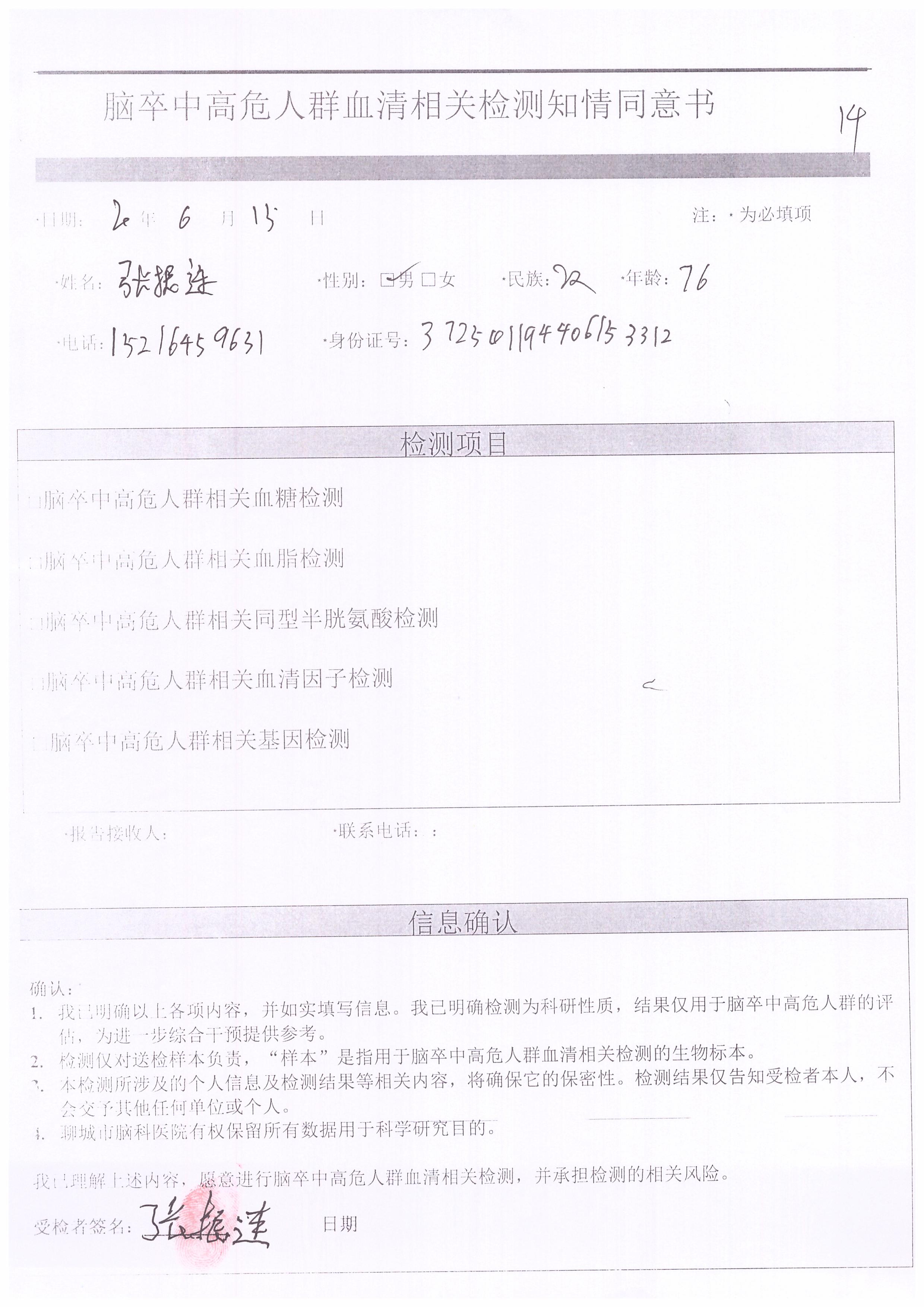

Supplement: Supplementary file 10 — Supplementary file10 (ZIP 21741 KB) [file 10528_2023_10431_MOESM10_ESM.zip › ╓¬╟Θ═1⁄4╥Γ╩Θ8/014.jpg]

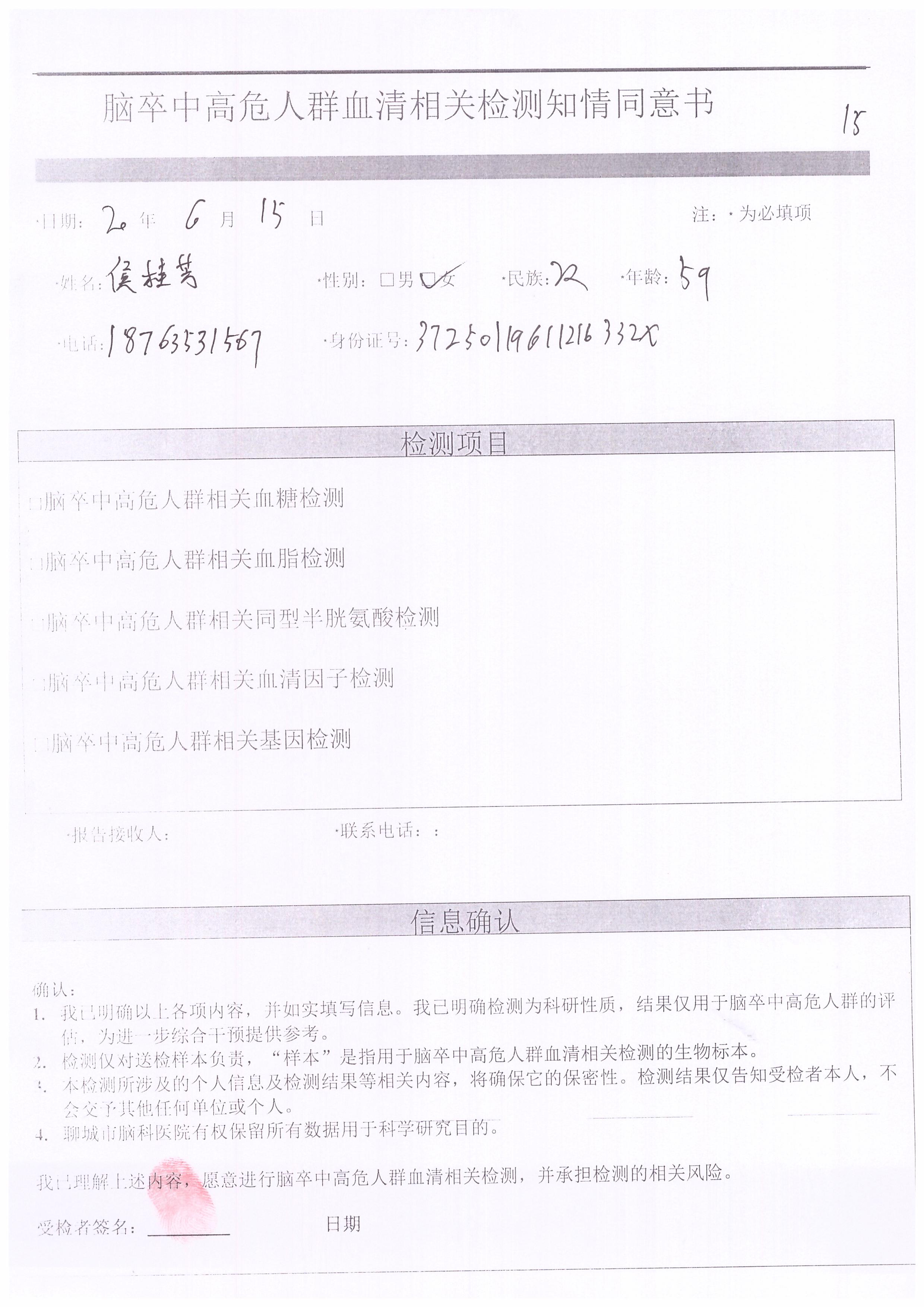

Supplement: Supplementary file 10 — Supplementary file10 (ZIP 21741 KB) [file 10528_2023_10431_MOESM10_ESM.zip › ╓¬╟Θ═1⁄4╥Γ╩Θ8/015.jpg]

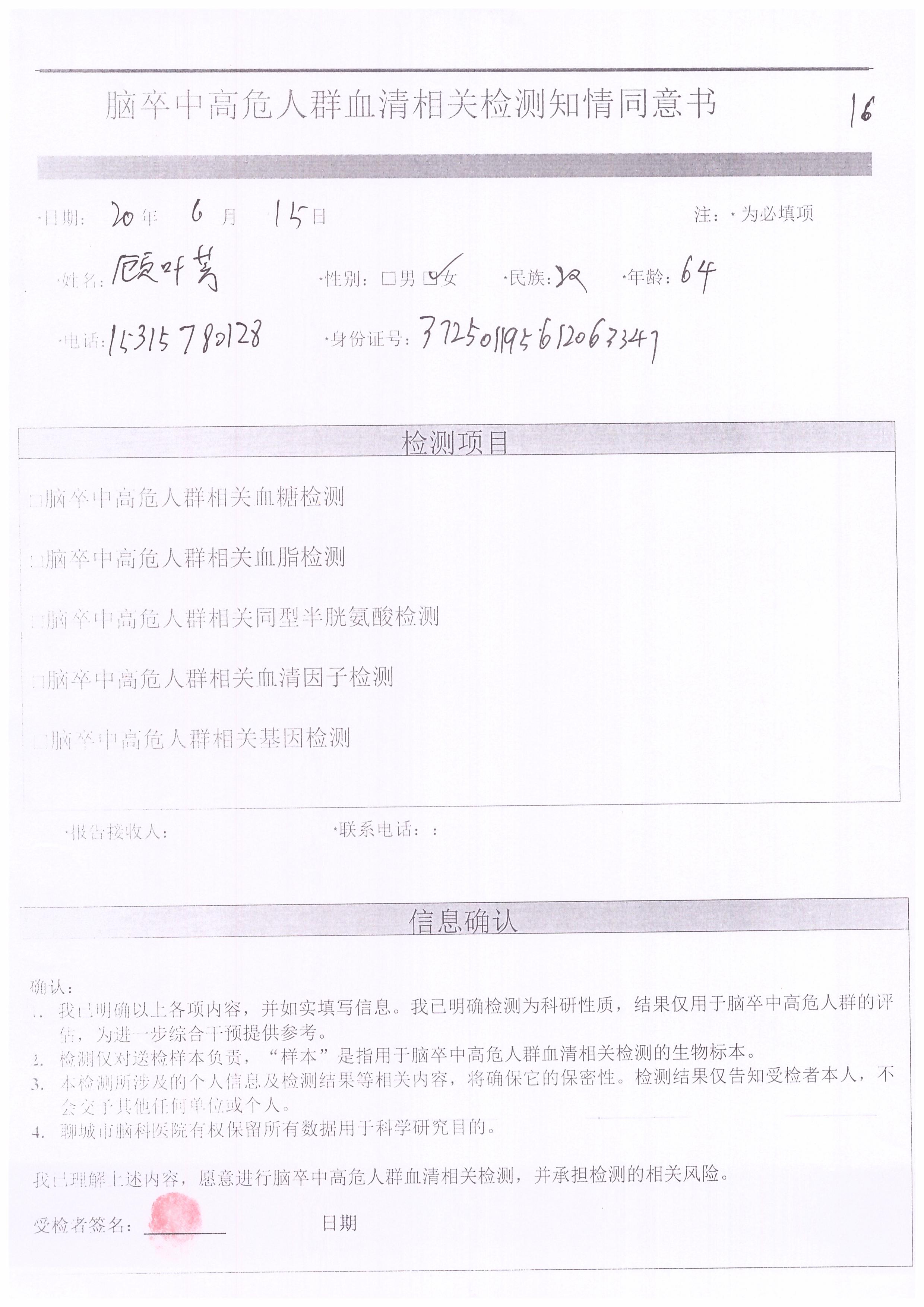

Supplement: Supplementary file 10 — Supplementary file10 (ZIP 21741 KB) [file 10528_2023_10431_MOESM10_ESM.zip › ╓¬╟Θ═1⁄4╥Γ╩Θ8/016.jpg]

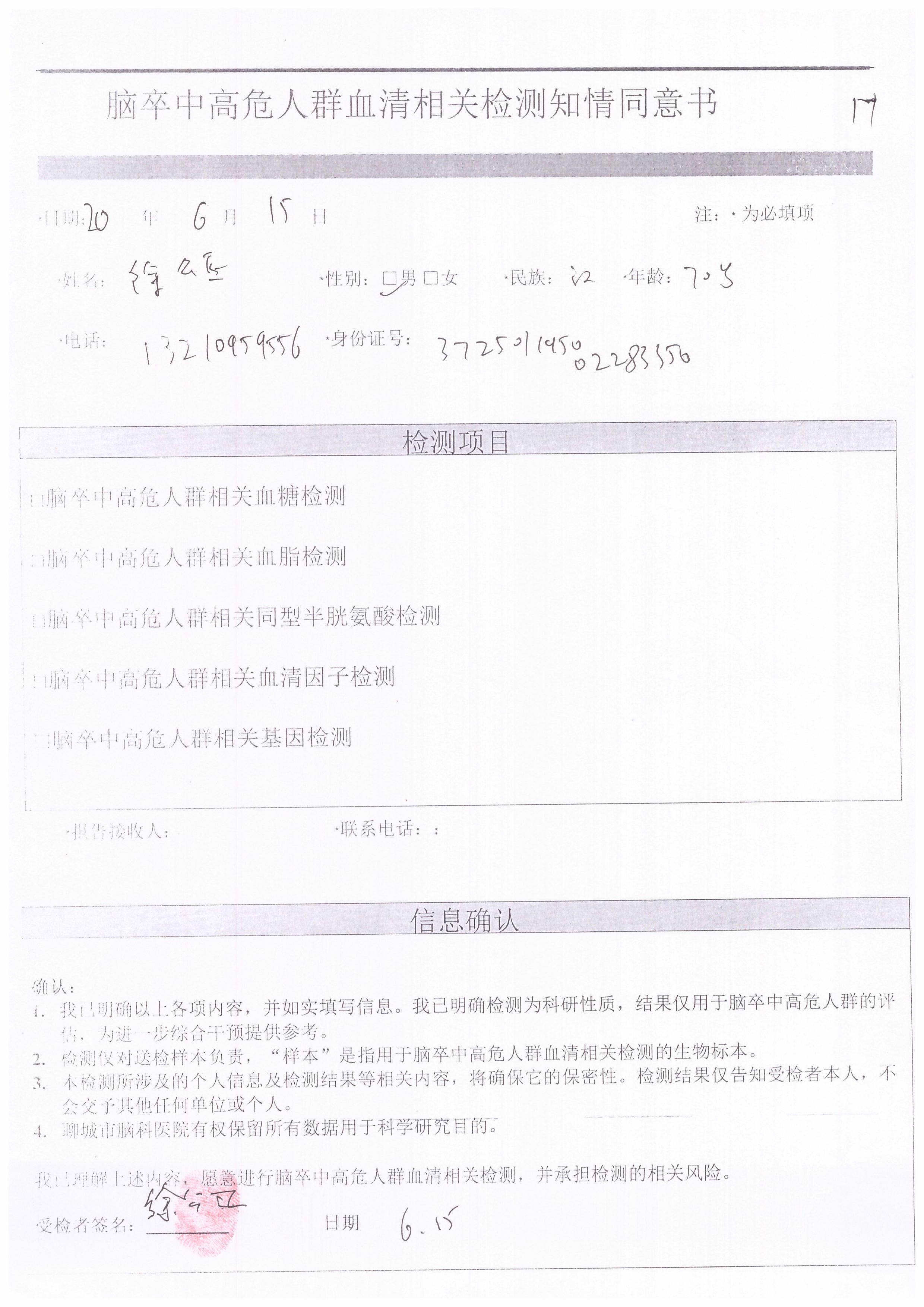

Supplement: Supplementary file 10 — Supplementary file10 (ZIP 21741 KB) [file 10528_2023_10431_MOESM10_ESM.zip › ╓¬╟Θ═1⁄4╥Γ╩Θ8/017.jpg]

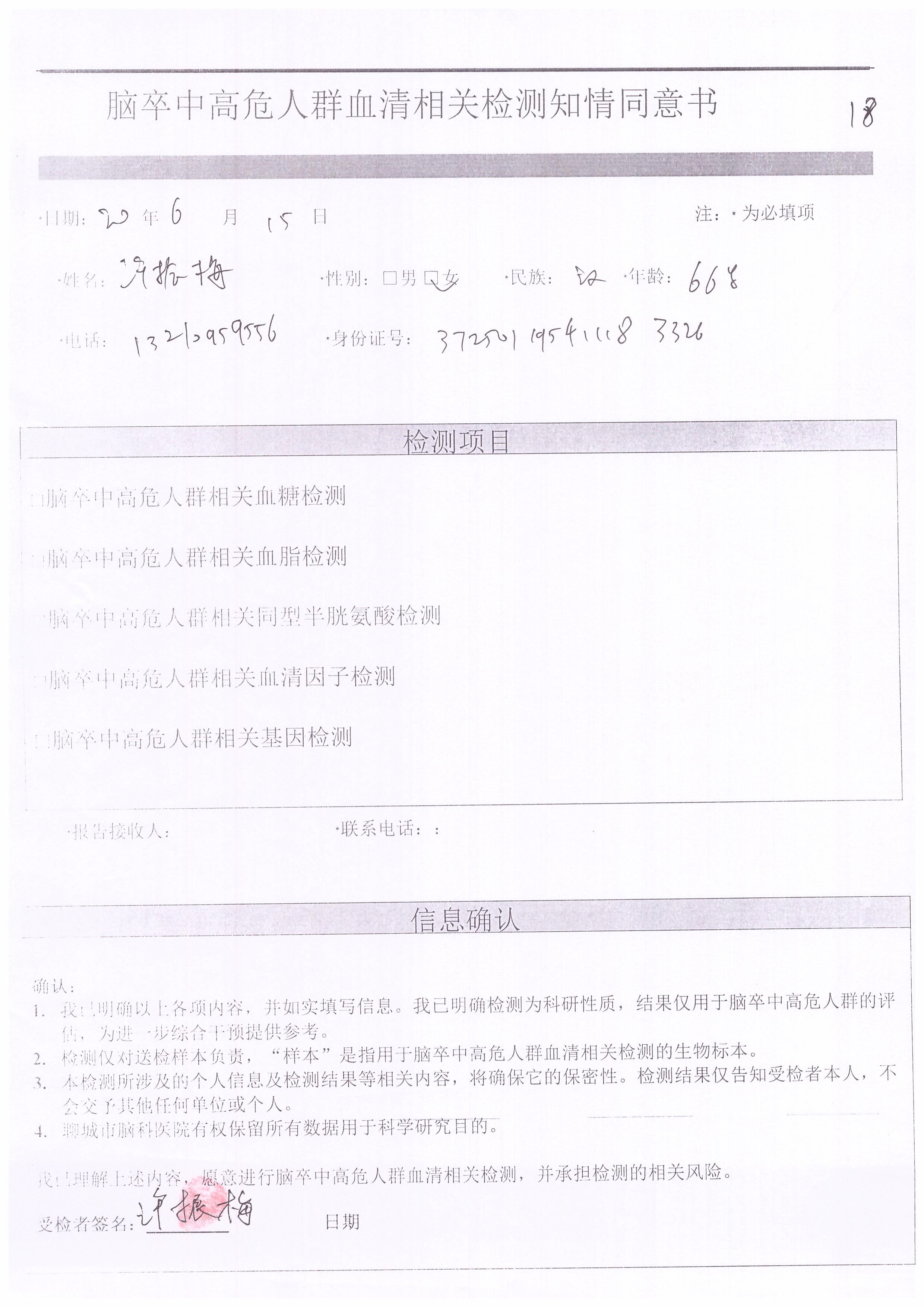

Supplement: Supplementary file 10 — Supplementary file10 (ZIP 21741 KB) [file 10528_2023_10431_MOESM10_ESM.zip › ╓¬╟Θ═1⁄4╥Γ╩Θ8/018.jpg]

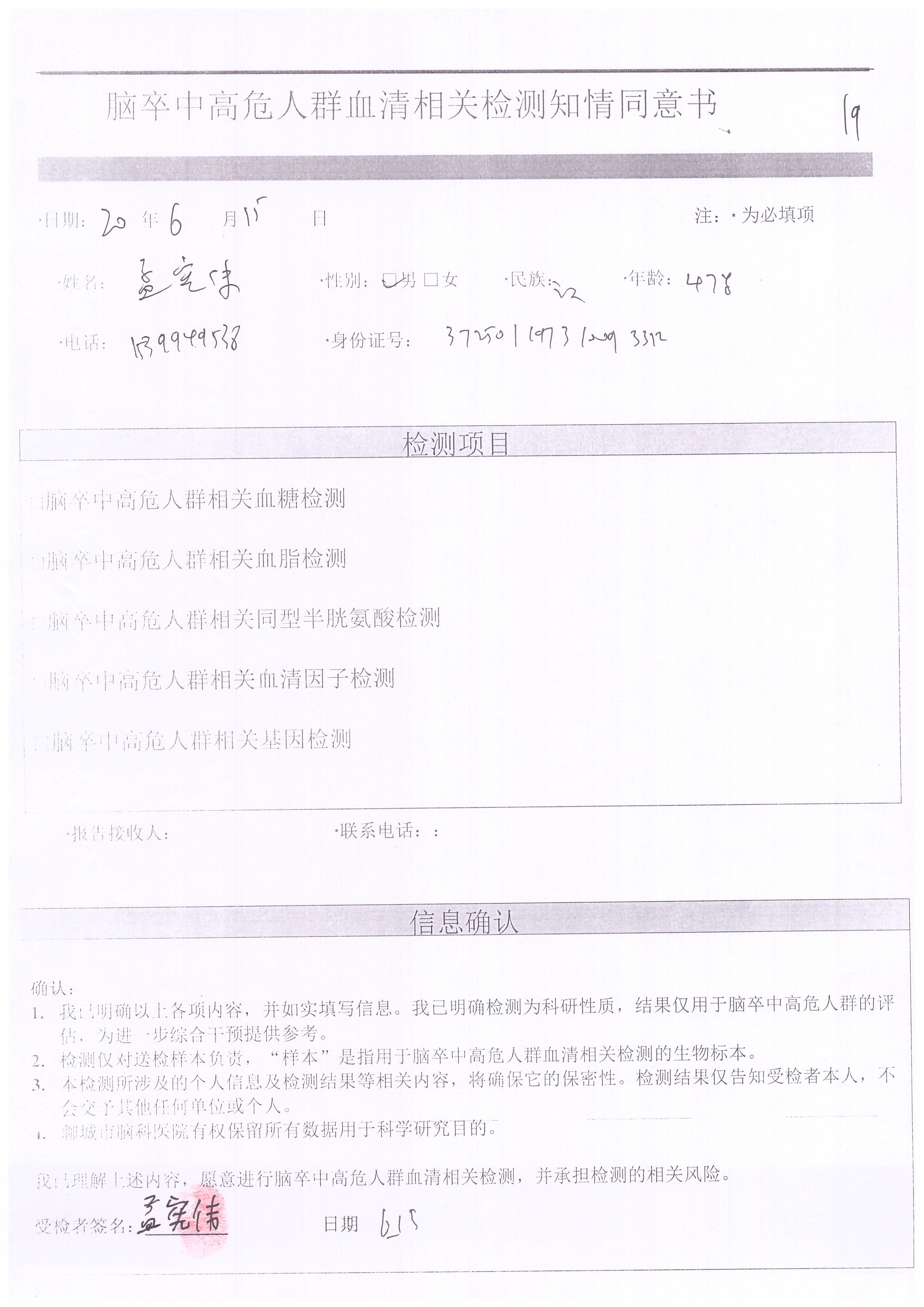

Supplement: Supplementary file 10 — Supplementary file10 (ZIP 21741 KB) [file 10528_2023_10431_MOESM10_ESM.zip › ╓¬╟Θ═1⁄4╥Γ╩Θ8/019.jpg]

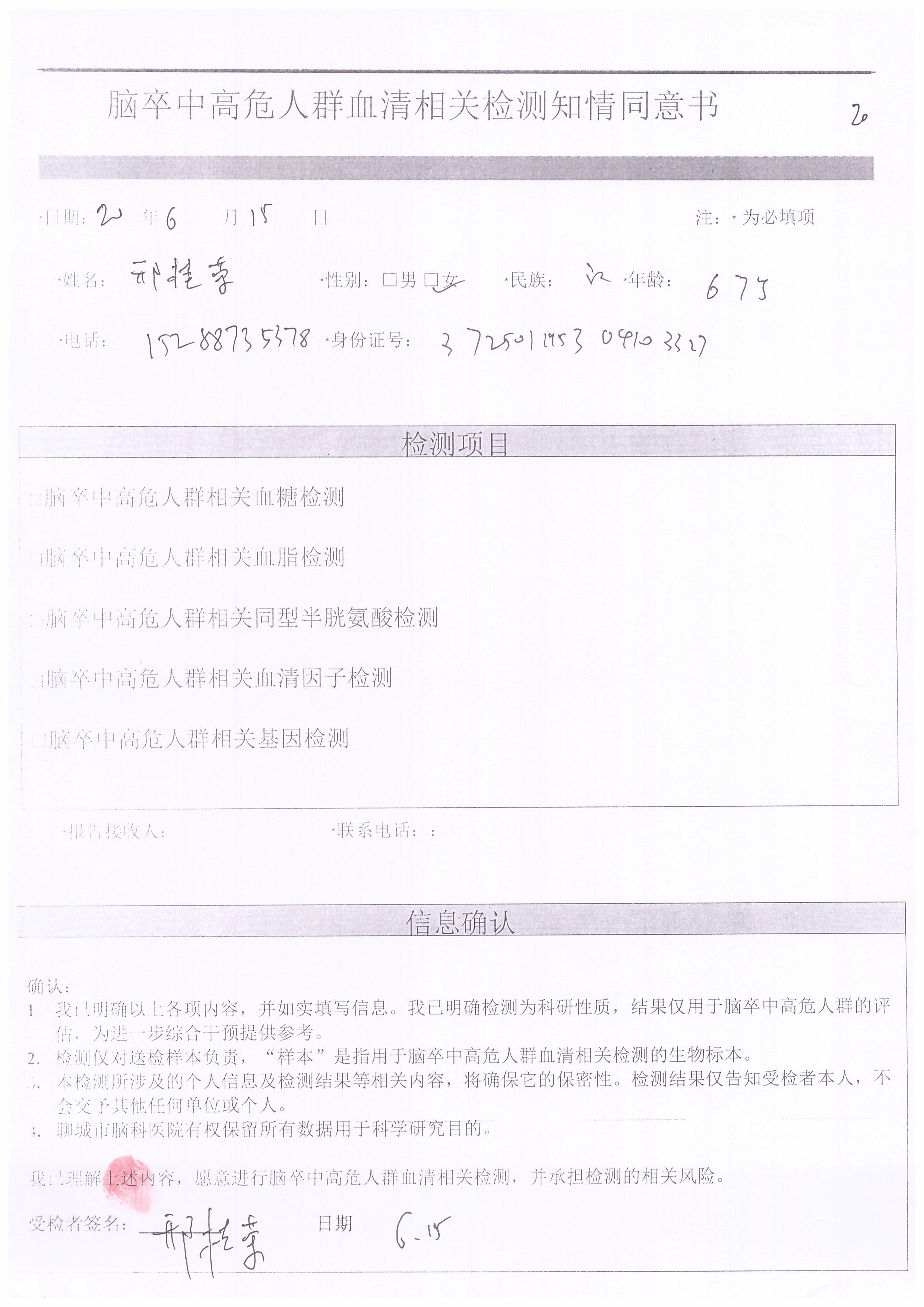

Supplement: Supplementary file 10 — Supplementary file10 (ZIP 21741 KB) [file 10528_2023_10431_MOESM10_ESM.zip › ╓¬╟Θ═1⁄4╥Γ╩Θ8/020.jpg]

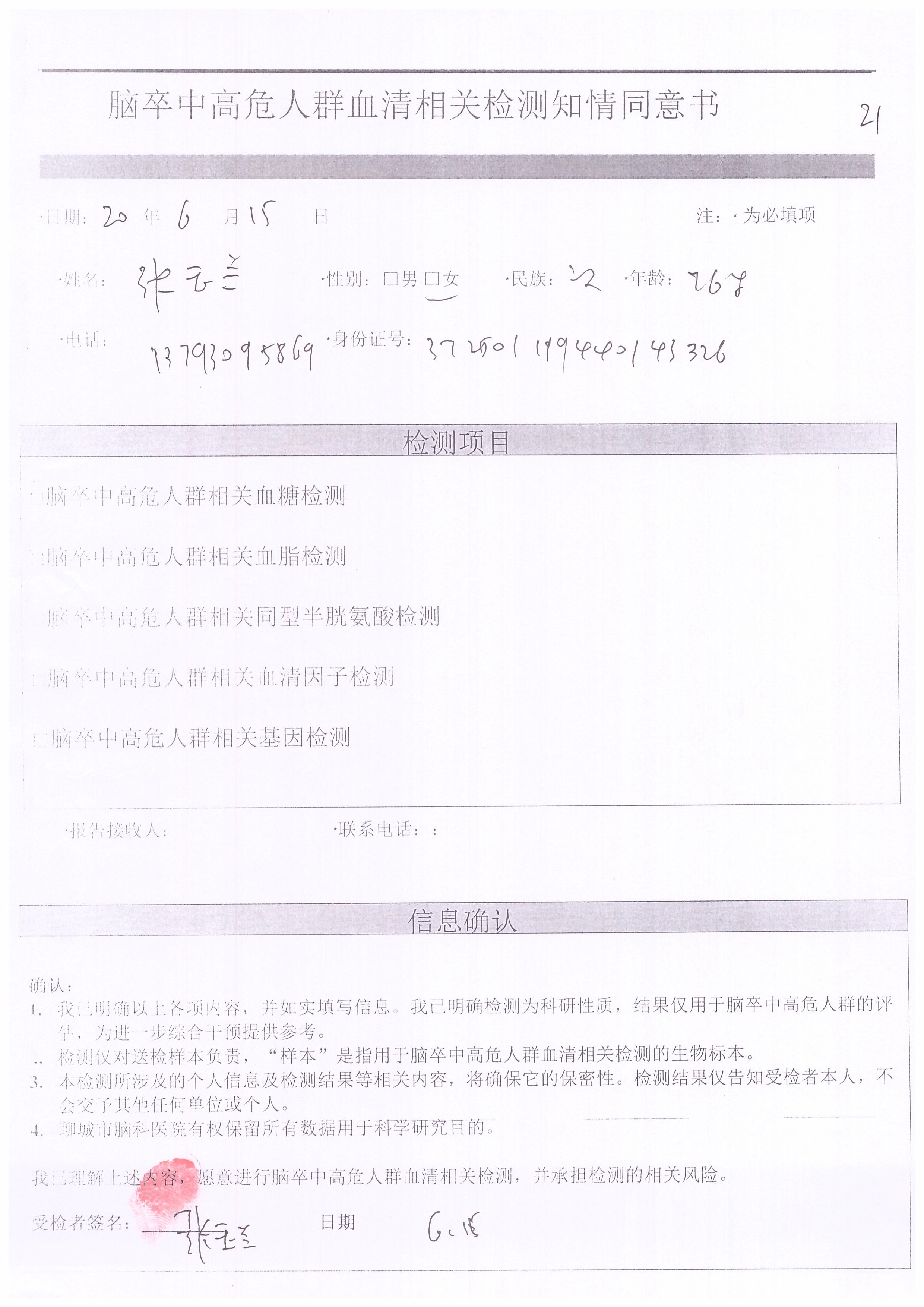

Supplement: Supplementary file 10 — Supplementary file10 (ZIP 21741 KB) [file 10528_2023_10431_MOESM10_ESM.zip › ╓¬╟Θ═1⁄4╥Γ╩Θ8/021.jpg]

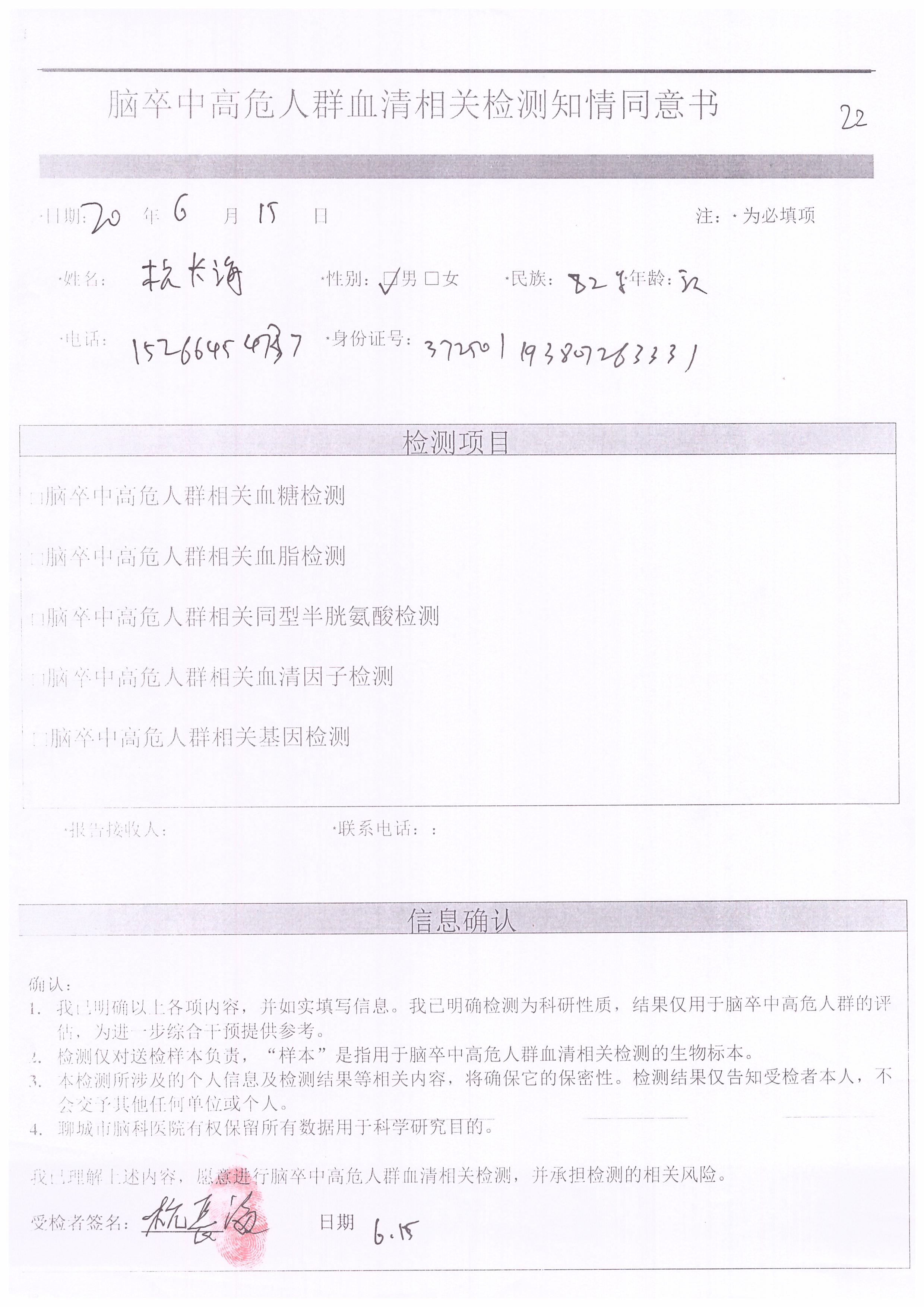

Supplement: Supplementary file 10 — Supplementary file10 (ZIP 21741 KB) [file 10528_2023_10431_MOESM10_ESM.zip › ╓¬╟Θ═1⁄4╥Γ╩Θ8/022.jpg]

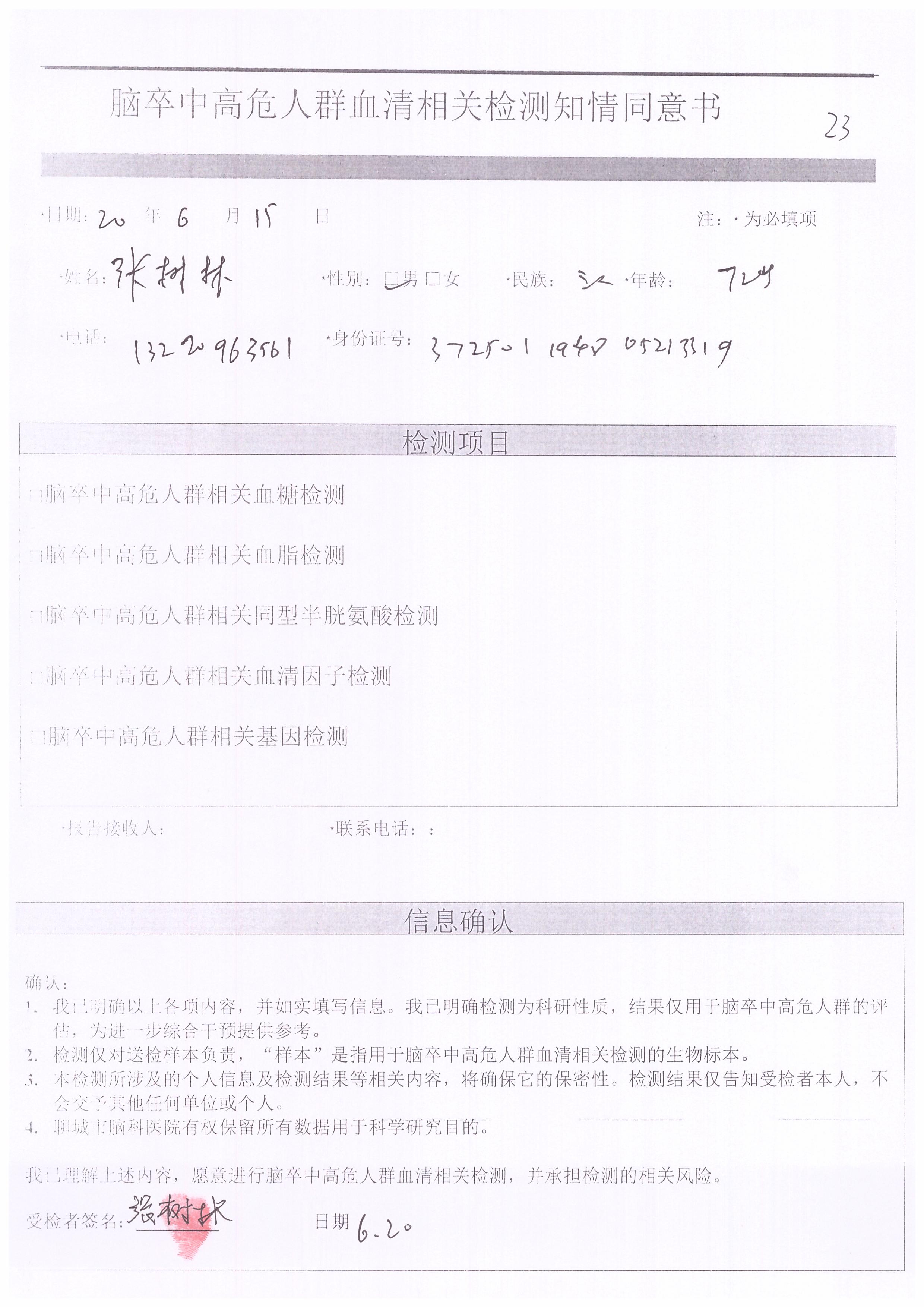

Supplement: Supplementary file 10 — Supplementary file10 (ZIP 21741 KB) [file 10528_2023_10431_MOESM10_ESM.zip › ╓¬╟Θ═1⁄4╥Γ╩Θ8/023.jpg]

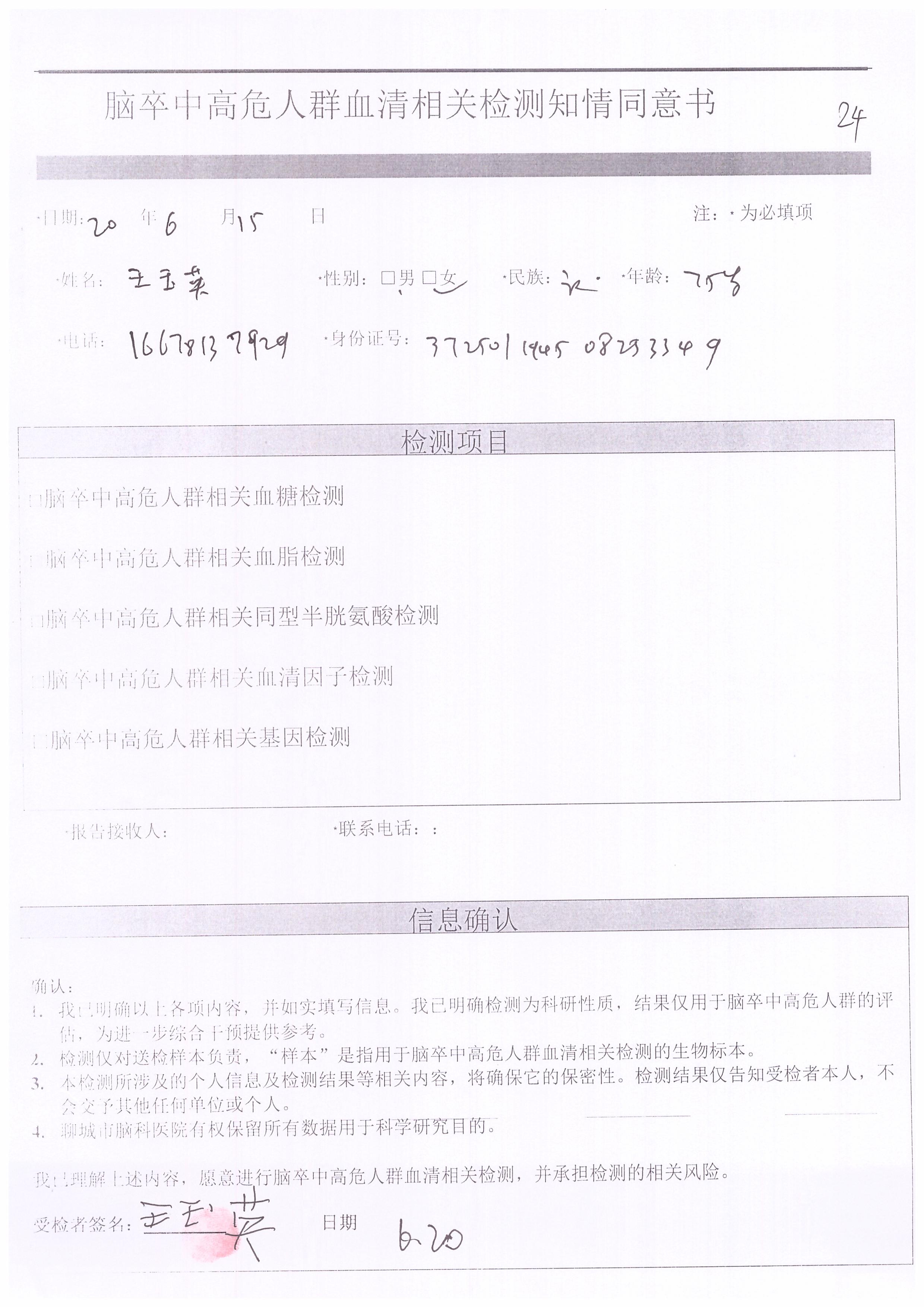

Supplement: Supplementary file 10 — Supplementary file10 (ZIP 21741 KB) [file 10528_2023_10431_MOESM10_ESM.zip › ╓¬╟Θ═1⁄4╥Γ╩Θ8/024.jpg]

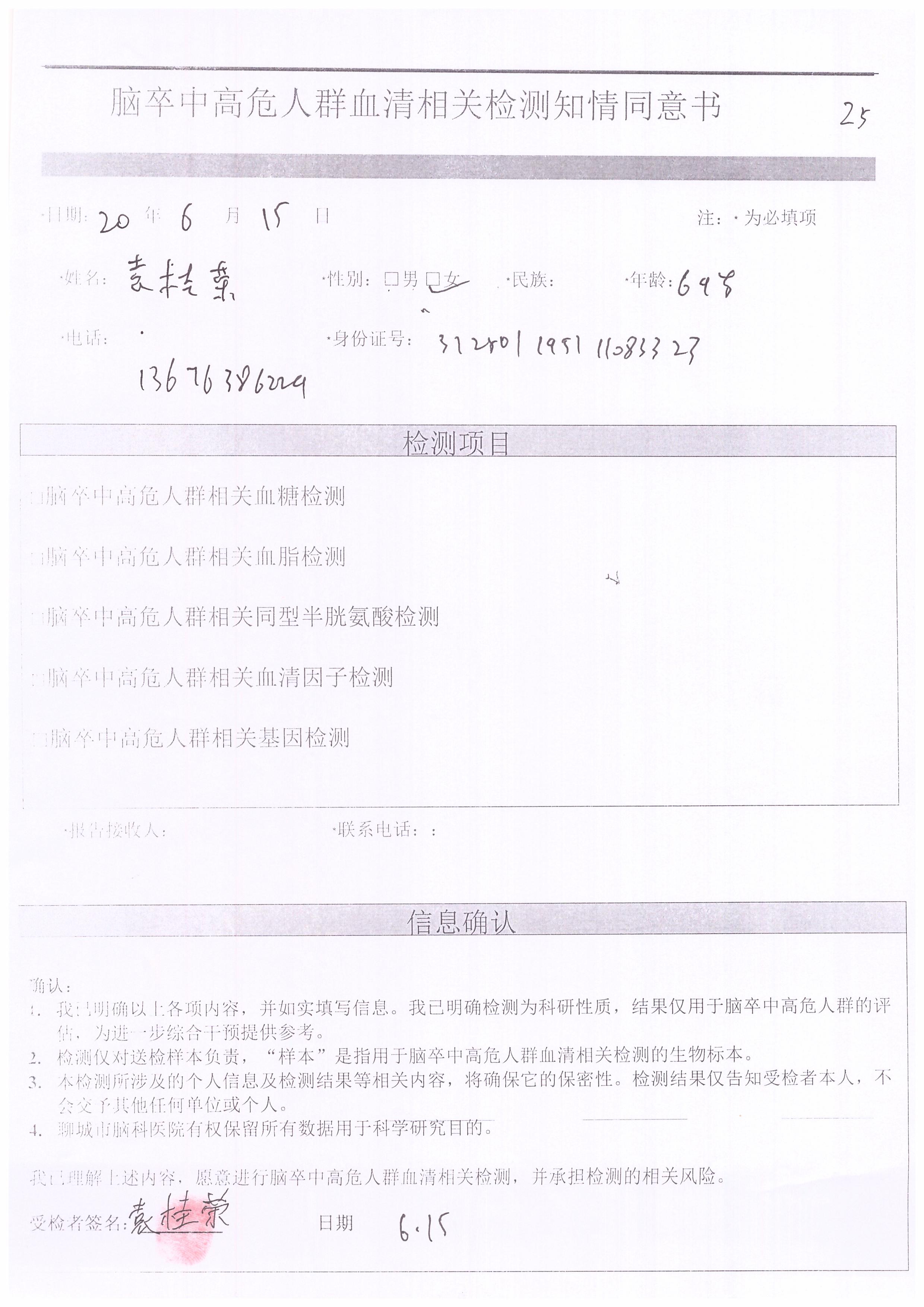

Supplement: Supplementary file 10 — Supplementary file10 (ZIP 21741 KB) [file 10528_2023_10431_MOESM10_ESM.zip › ╓¬╟Θ═1⁄4╥Γ╩Θ8/025.jpg]

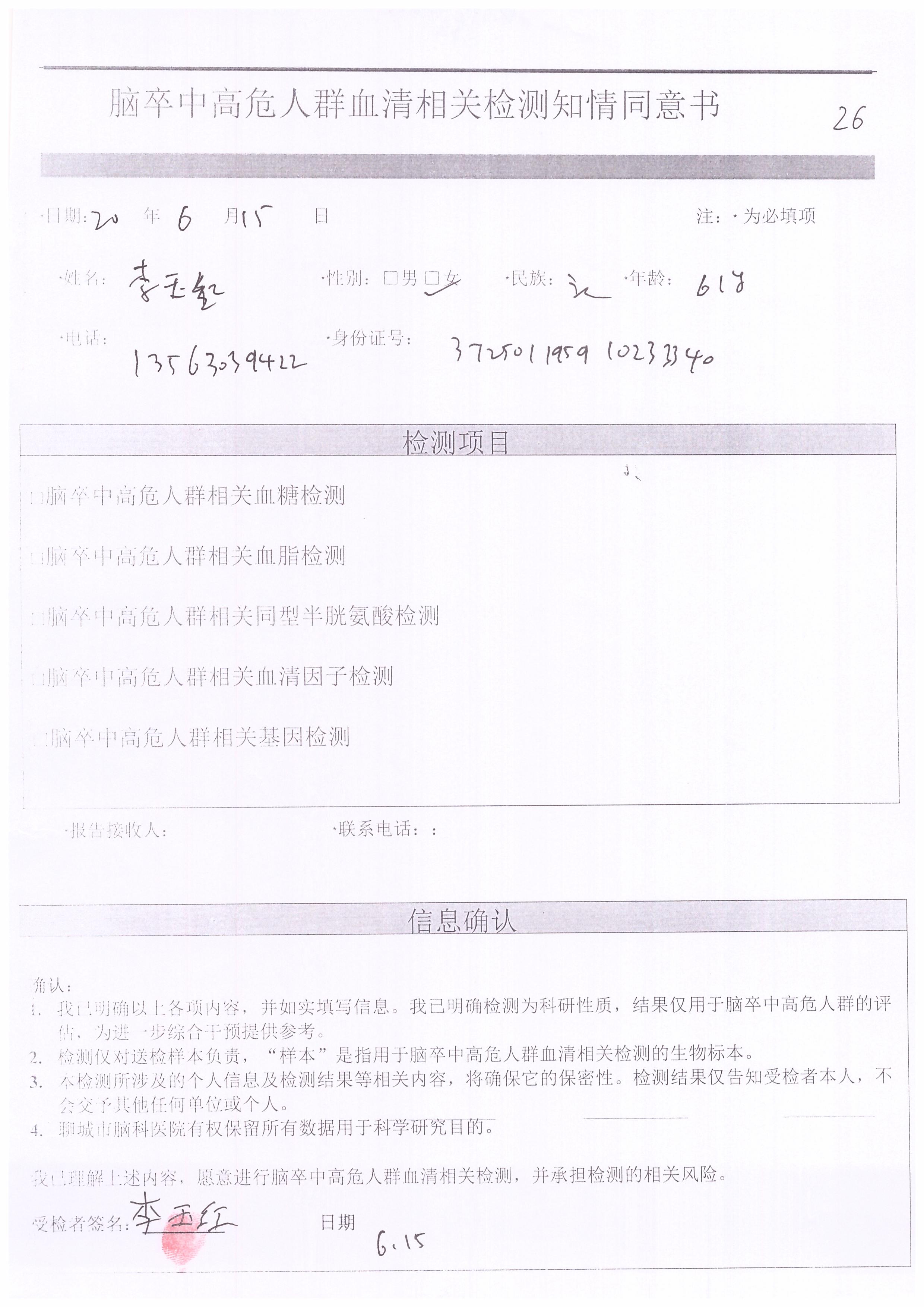

Supplement: Supplementary file 10 — Supplementary file10 (ZIP 21741 KB) [file 10528_2023_10431_MOESM10_ESM.zip › ╓¬╟Θ═1⁄4╥Γ╩Θ8/026.jpg]

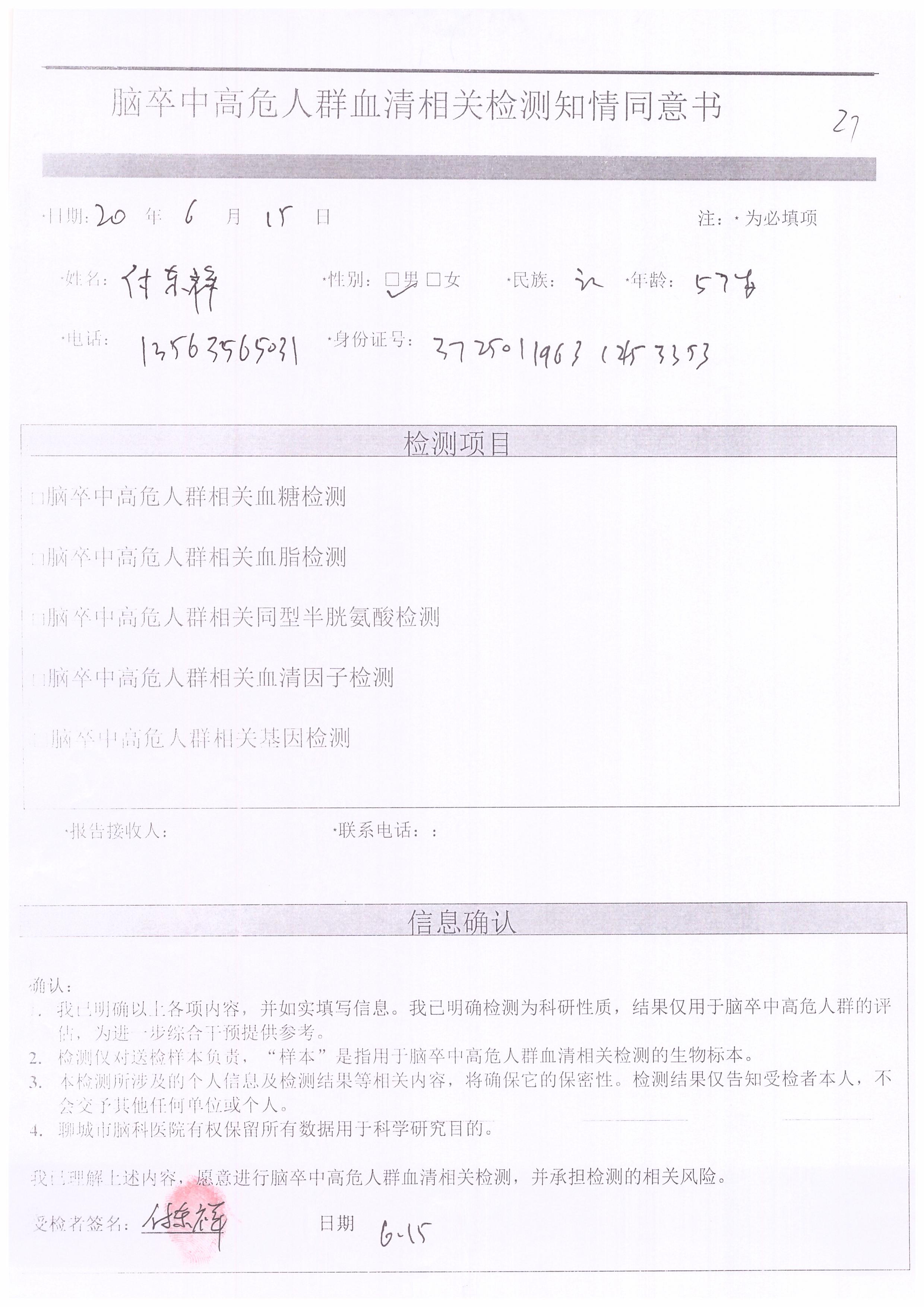

Supplement: Supplementary file 10 — Supplementary file10 (ZIP 21741 KB) [file 10528_2023_10431_MOESM10_ESM.zip › ╓¬╟Θ═1⁄4╥Γ╩Θ8/027.jpg]

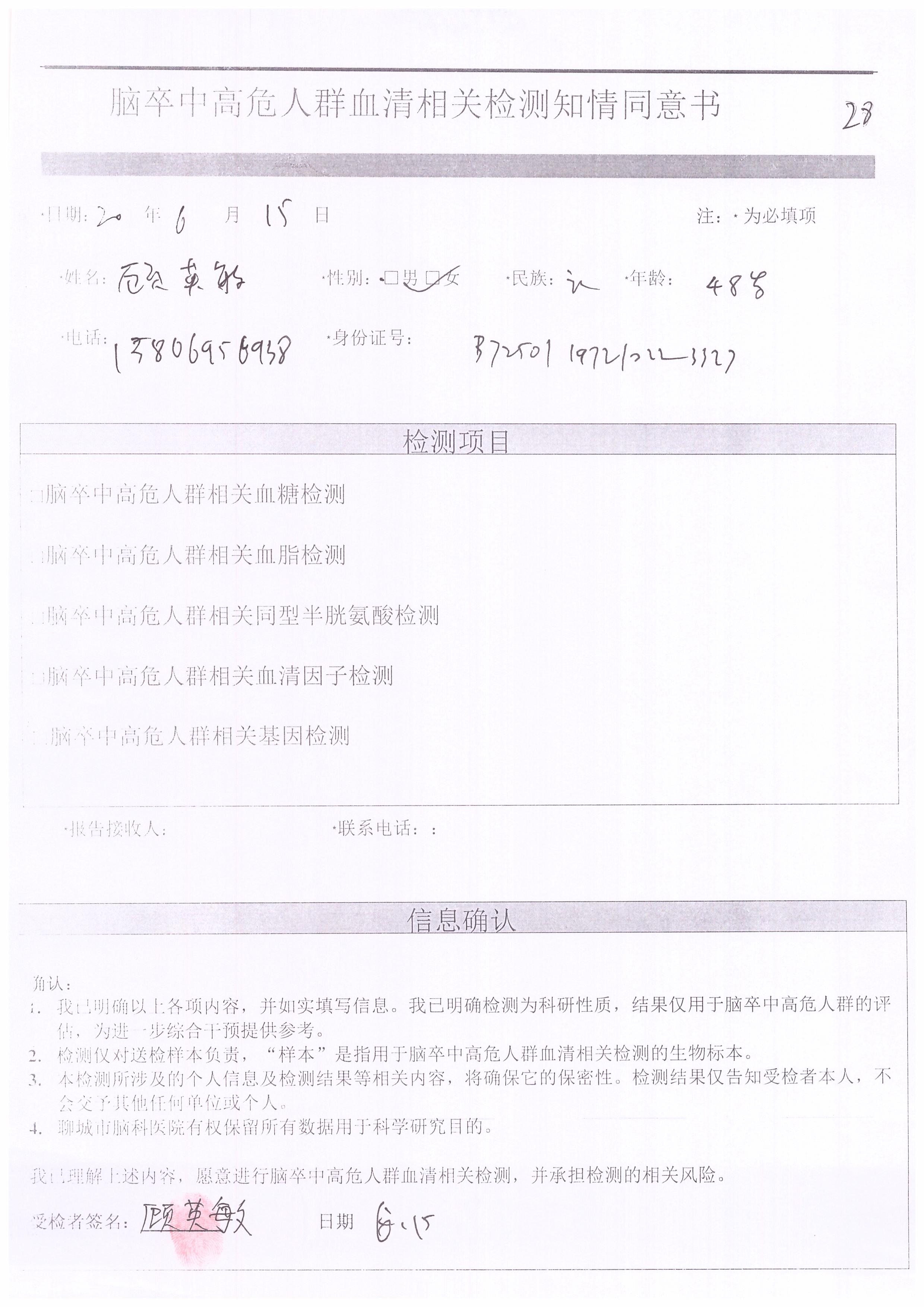

Supplement: Supplementary file 10 — Supplementary file10 (ZIP 21741 KB) [file 10528_2023_10431_MOESM10_ESM.zip › ╓¬╟Θ═1⁄4╥Γ╩Θ8/028.jpg]

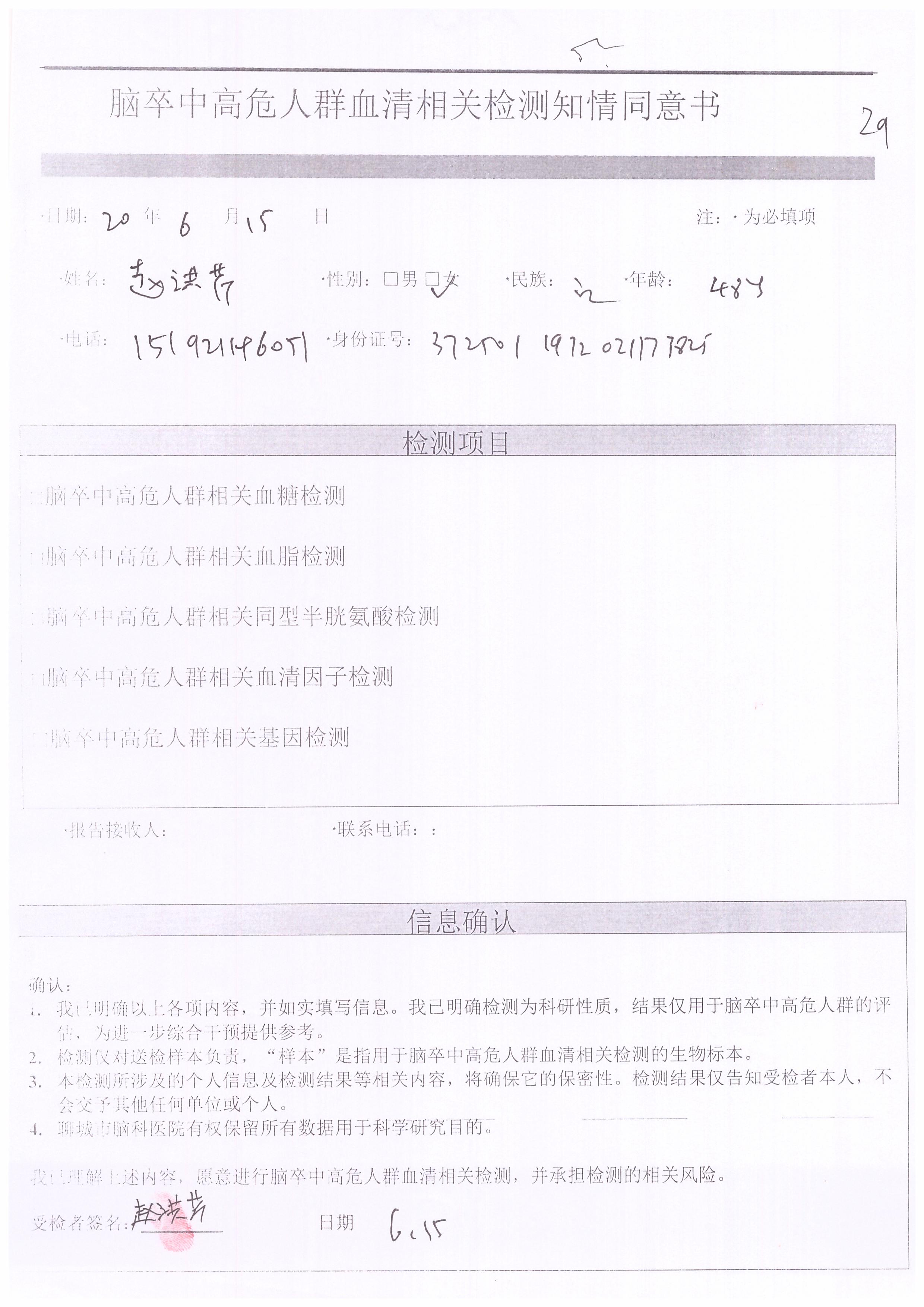

Supplement: Supplementary file 10 — Supplementary file10 (ZIP 21741 KB) [file 10528_2023_10431_MOESM10_ESM.zip › ╓¬╟Θ═1⁄4╥Γ╩Θ8/029.jpg]

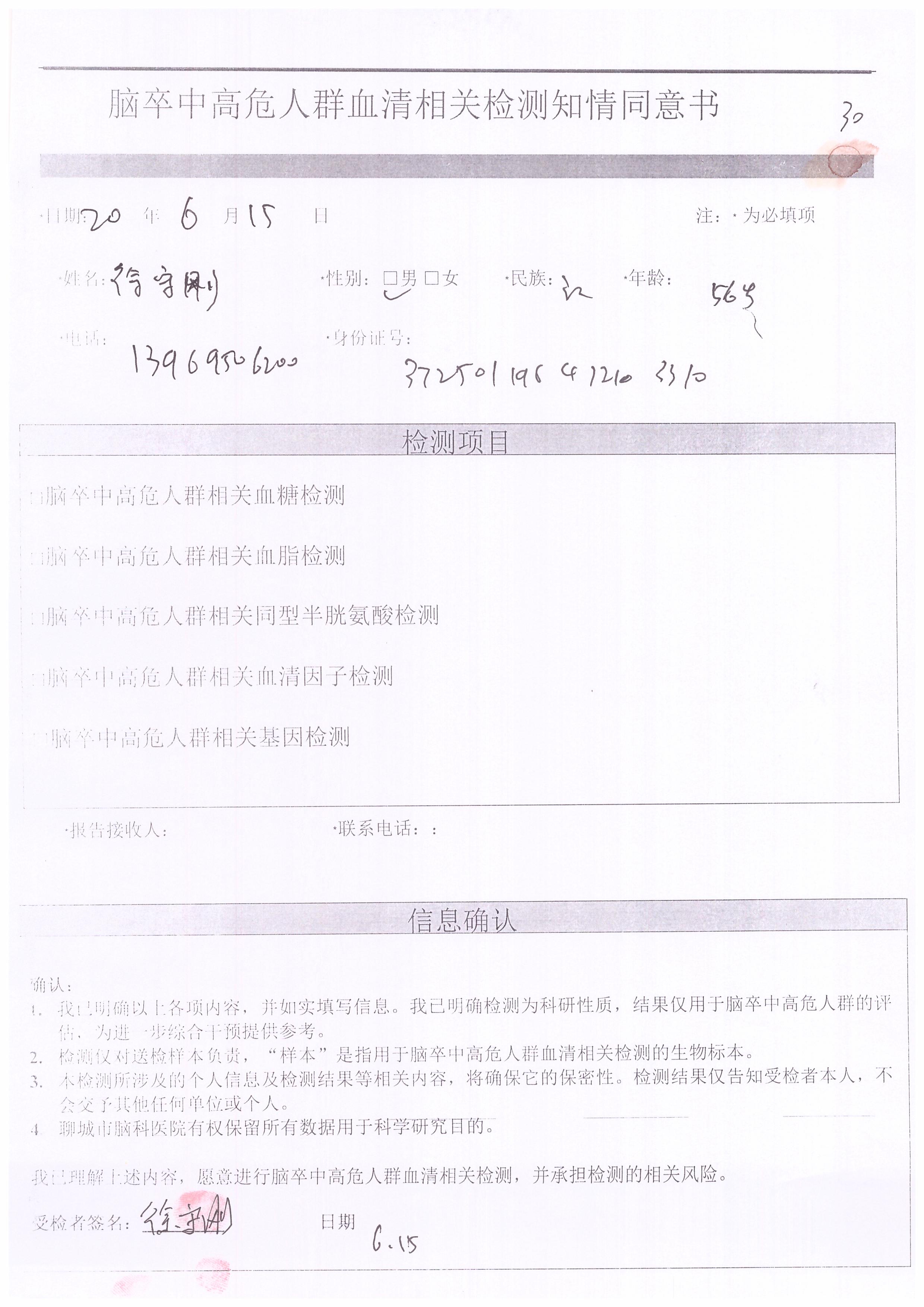

Supplement: Supplementary file 10 — Supplementary file10 (ZIP 21741 KB) [file 10528_2023_10431_MOESM10_ESM.zip › ╓¬╟Θ═1⁄4╥Γ╩Θ8/030.jpg]

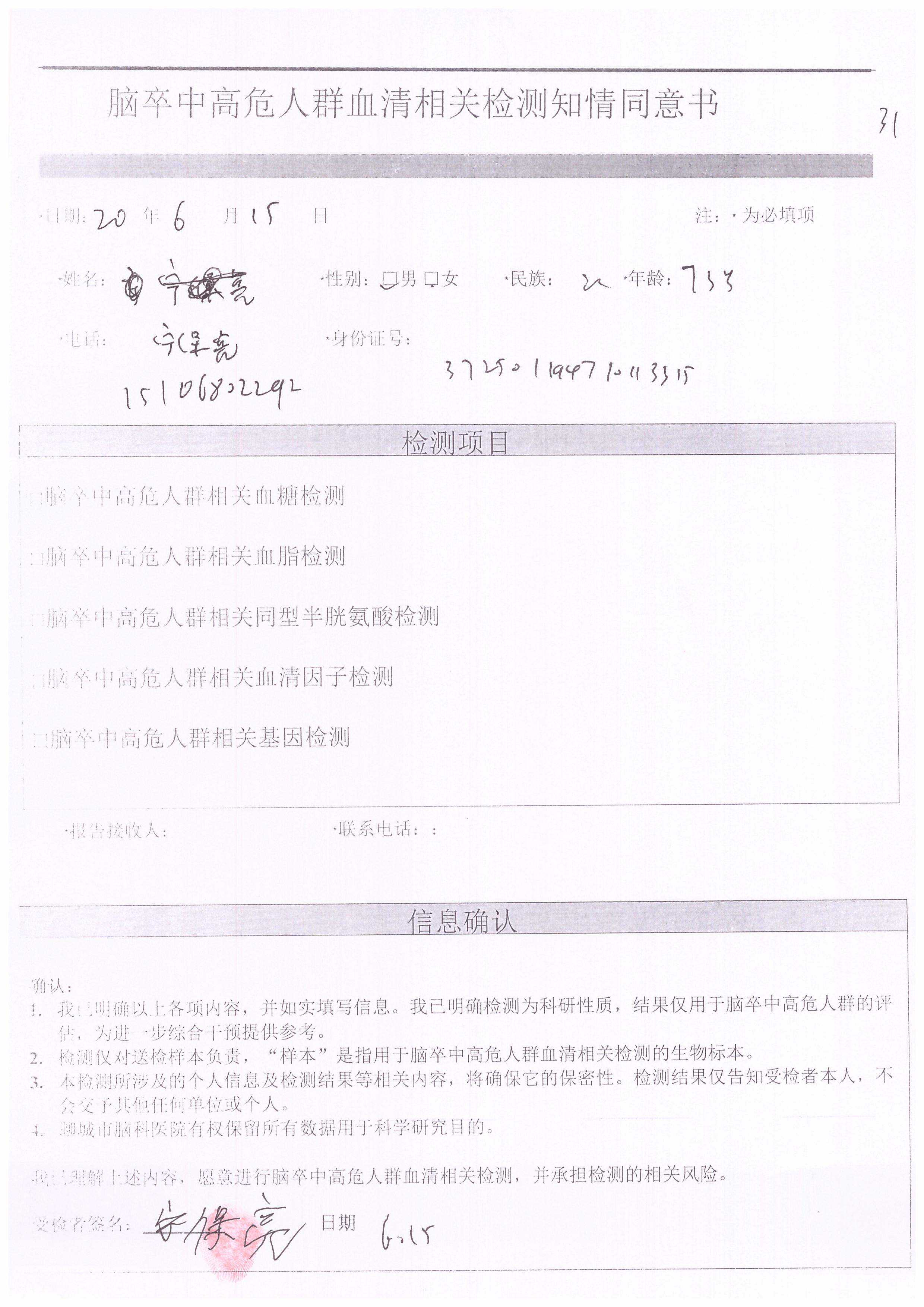

Supplement: Supplementary file 10 — Supplementary file10 (ZIP 21741 KB) [file 10528_2023_10431_MOESM10_ESM.zip › ╓¬╟Θ═1⁄4╥Γ╩Θ8/031.jpg]

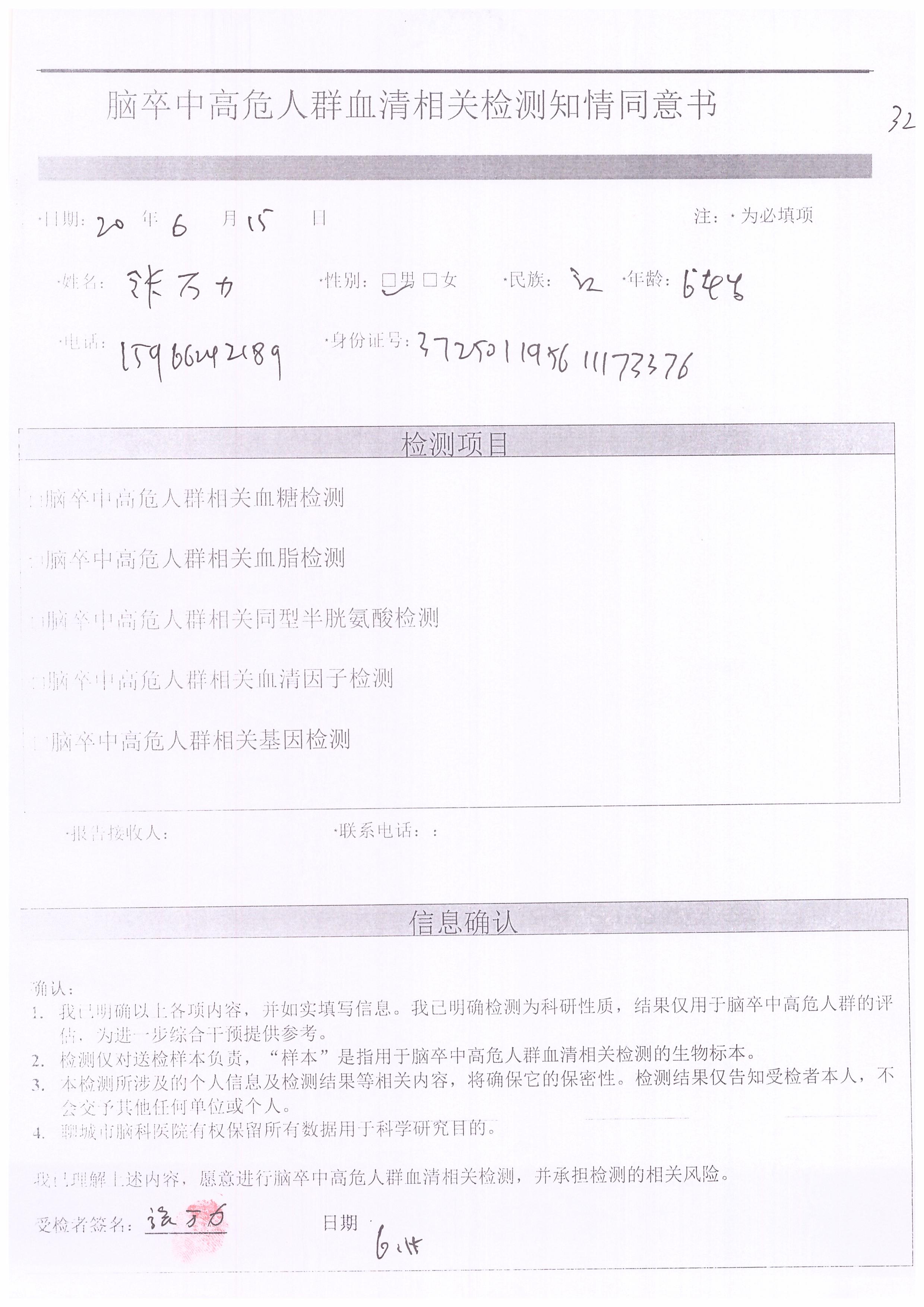

Supplement: Supplementary file 10 — Supplementary file10 (ZIP 21741 KB) [file 10528_2023_10431_MOESM10_ESM.zip › ╓¬╟Θ═1⁄4╥Γ╩Θ8/032.jpg]

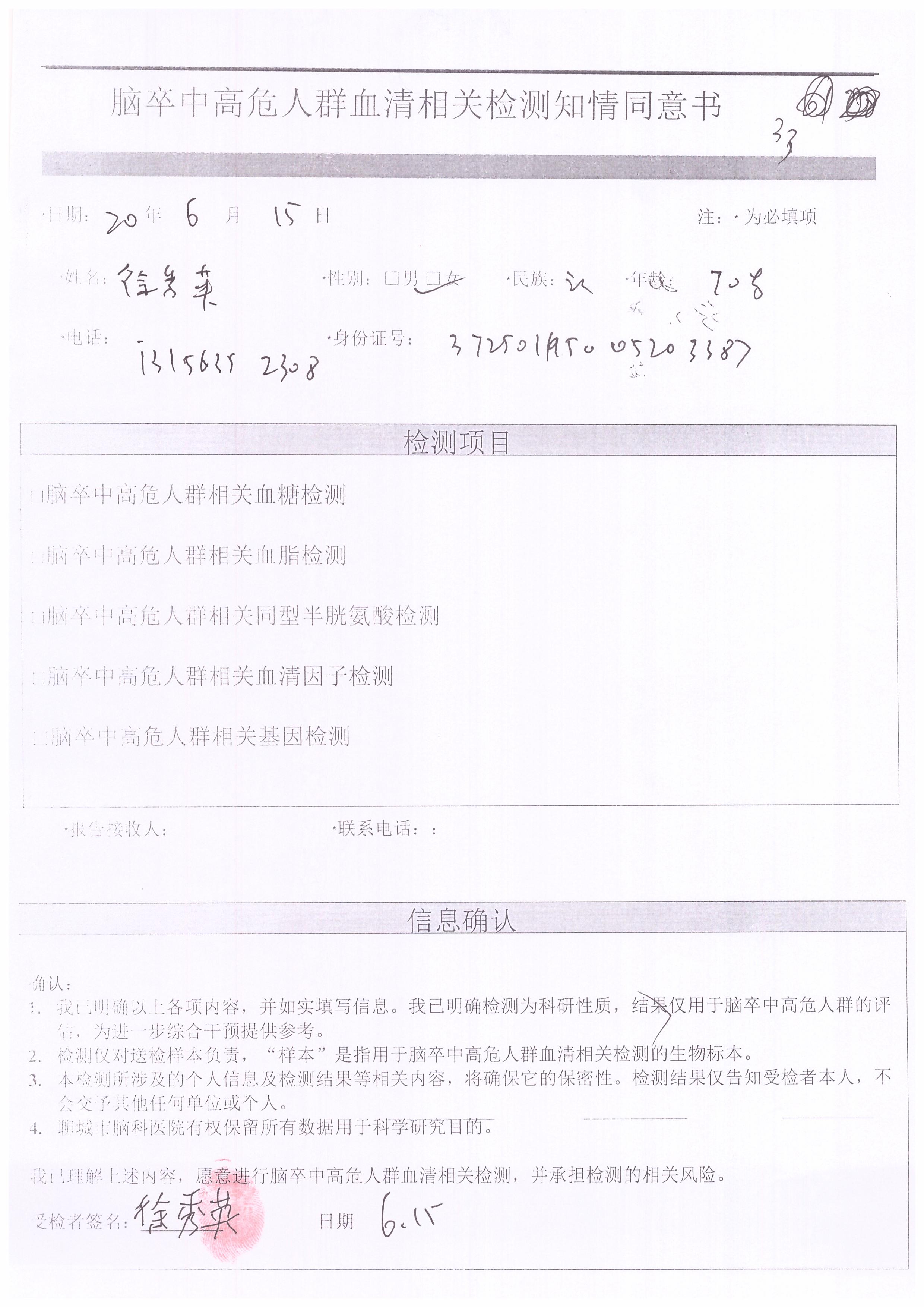

Supplement: Supplementary file 10 — Supplementary file10 (ZIP 21741 KB) [file 10528_2023_10431_MOESM10_ESM.zip › ╓¬╟Θ═1⁄4╥Γ╩Θ8/033.jpg]

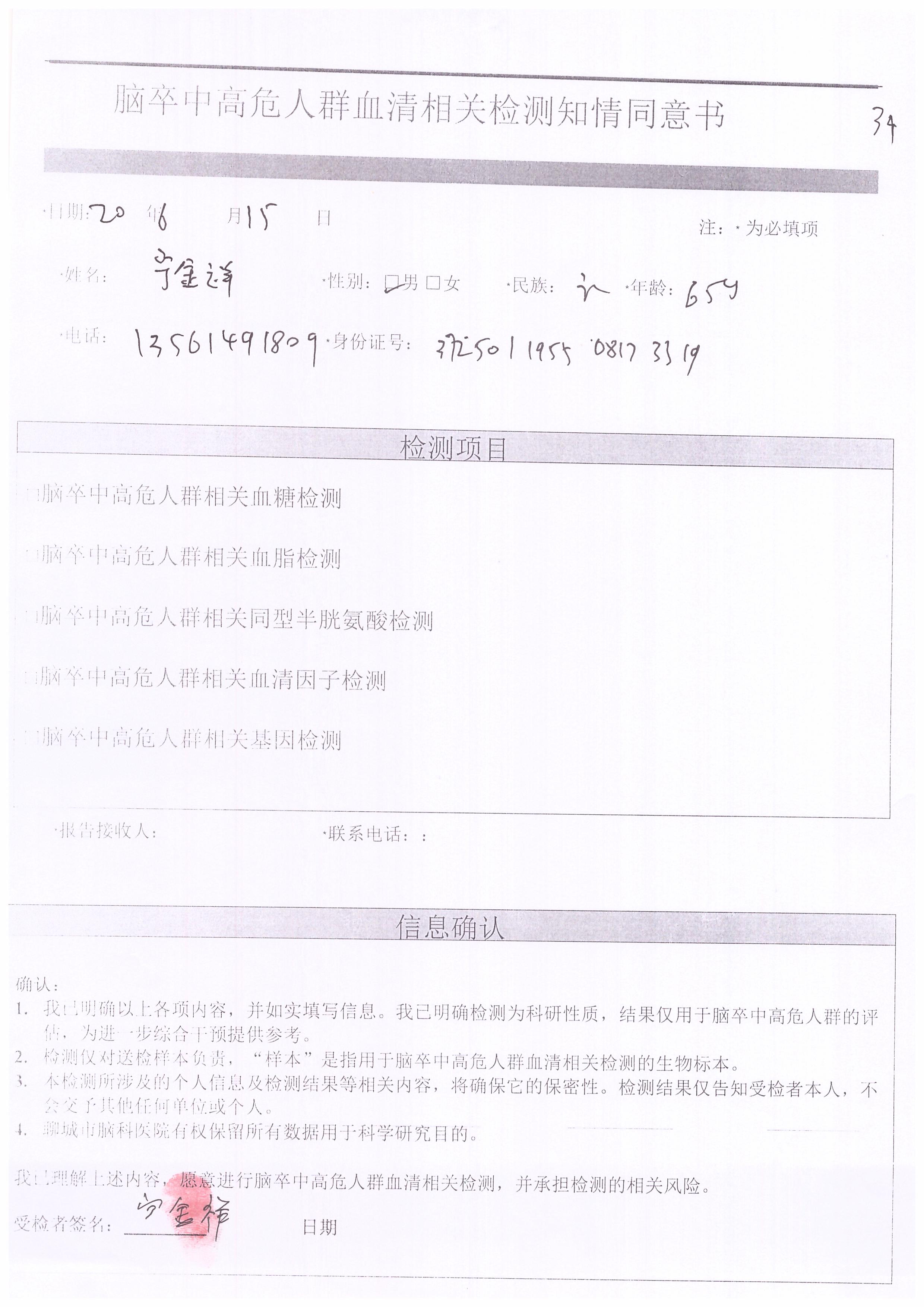

Supplement: Supplementary file 10 — Supplementary file10 (ZIP 21741 KB) [file 10528_2023_10431_MOESM10_ESM.zip › ╓¬╟Θ═1⁄4╥Γ╩Θ8/034.jpg]

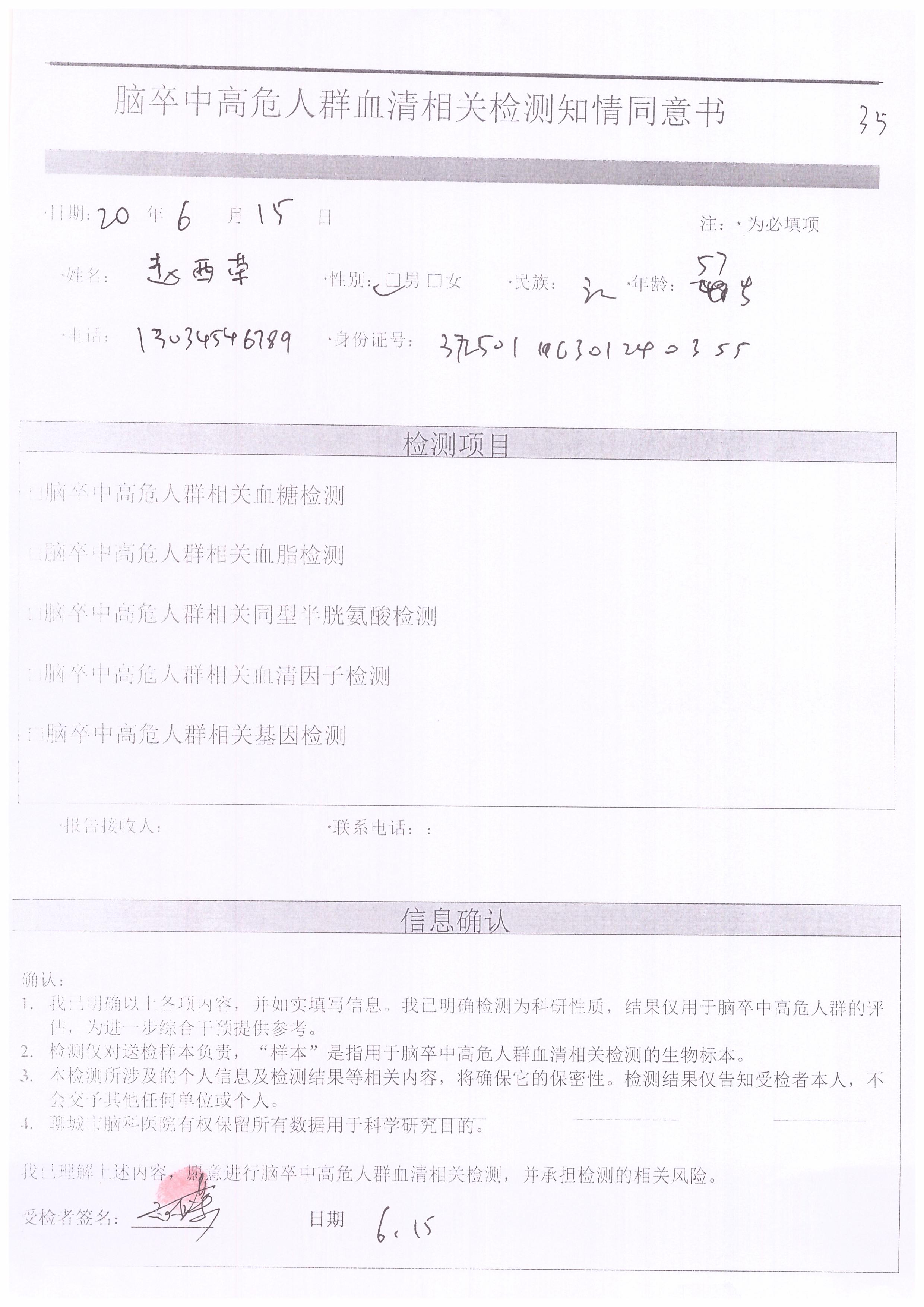

Supplement: Supplementary file 10 — Supplementary file10 (ZIP 21741 KB) [file 10528_2023_10431_MOESM10_ESM.zip › ╓¬╟Θ═1⁄4╥Γ╩Θ8/035.jpg]

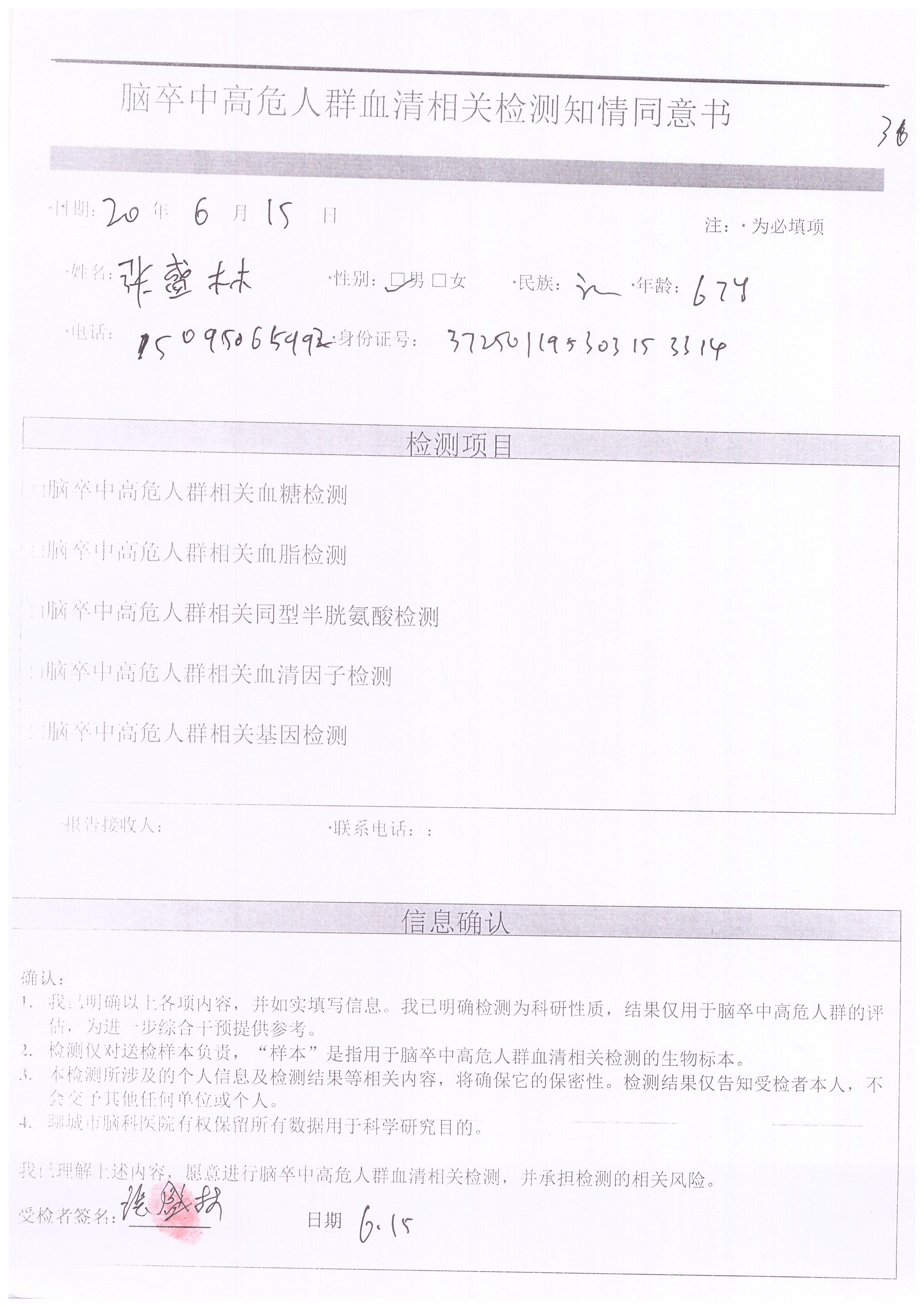

Supplement: Supplementary file 10 — Supplementary file10 (ZIP 21741 KB) [file 10528_2023_10431_MOESM10_ESM.zip › ╓¬╟Θ═1⁄4╥Γ╩Θ8/036.jpg]

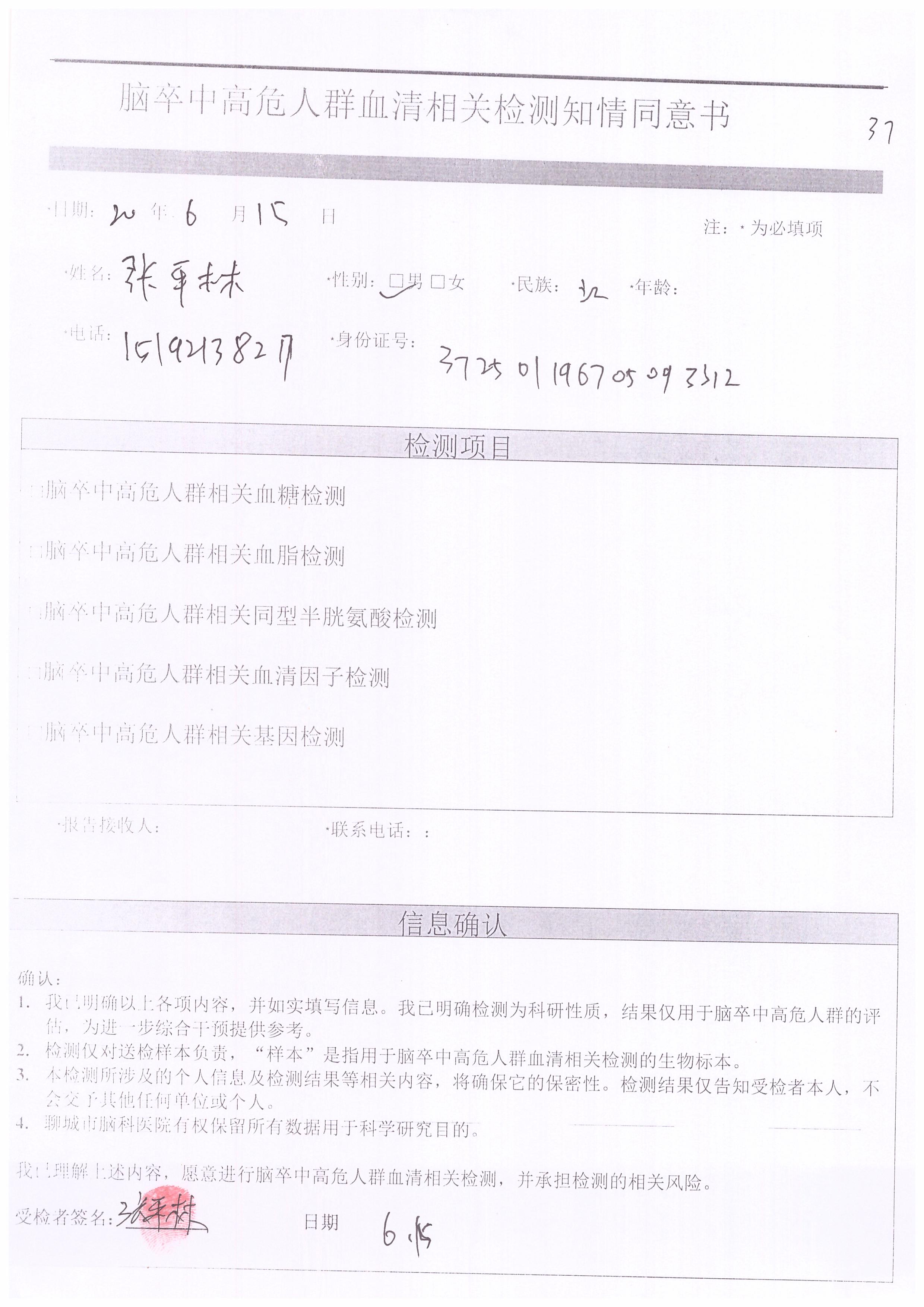

Supplement: Supplementary file 10 — Supplementary file10 (ZIP 21741 KB) [file 10528_2023_10431_MOESM10_ESM.zip › ╓¬╟Θ═1⁄4╥Γ╩Θ8/037.jpg]

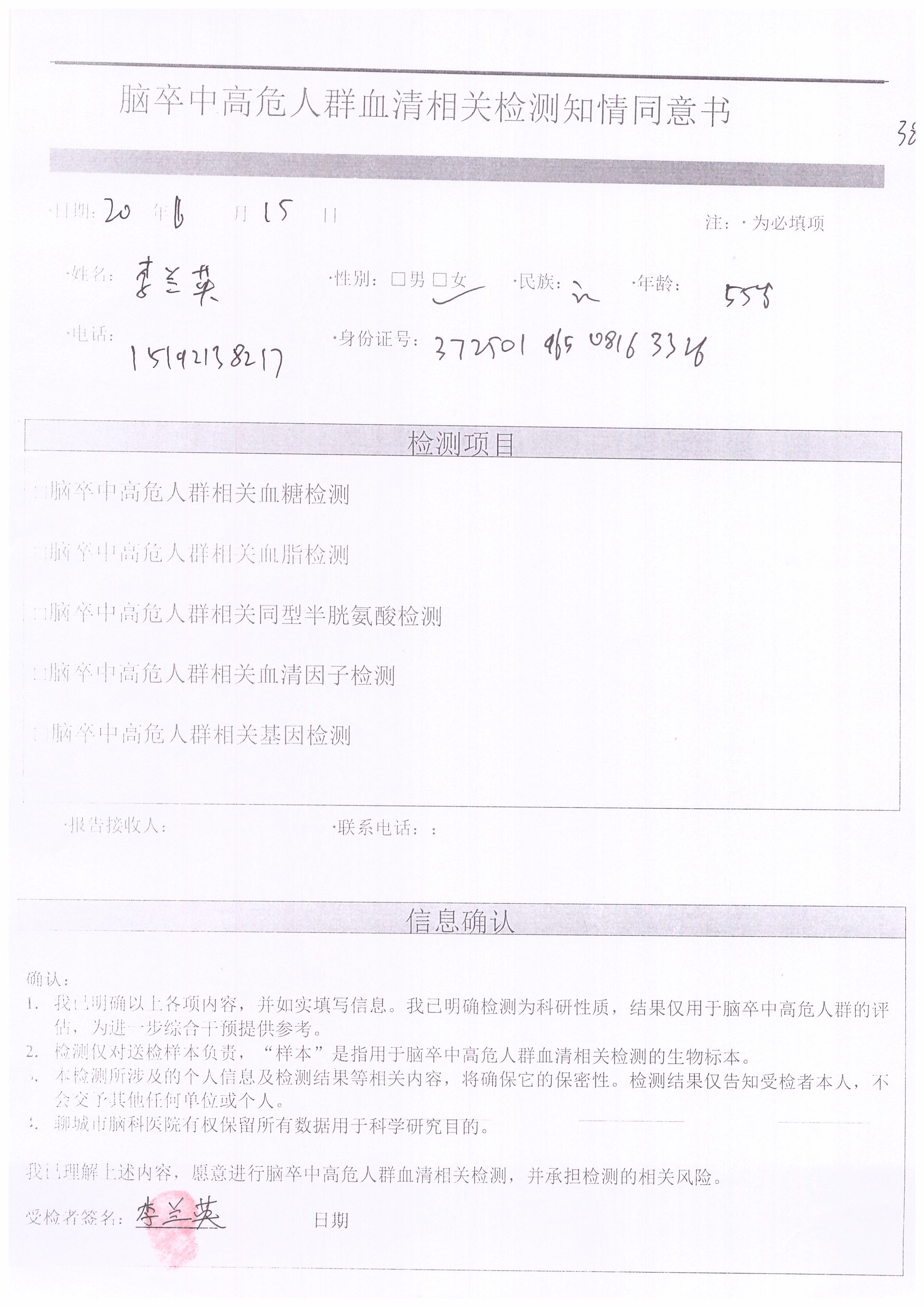

Supplement: Supplementary file 10 — Supplementary file10 (ZIP 21741 KB) [file 10528_2023_10431_MOESM10_ESM.zip › ╓¬╟Θ═1⁄4╥Γ╩Θ8/038.jpg]

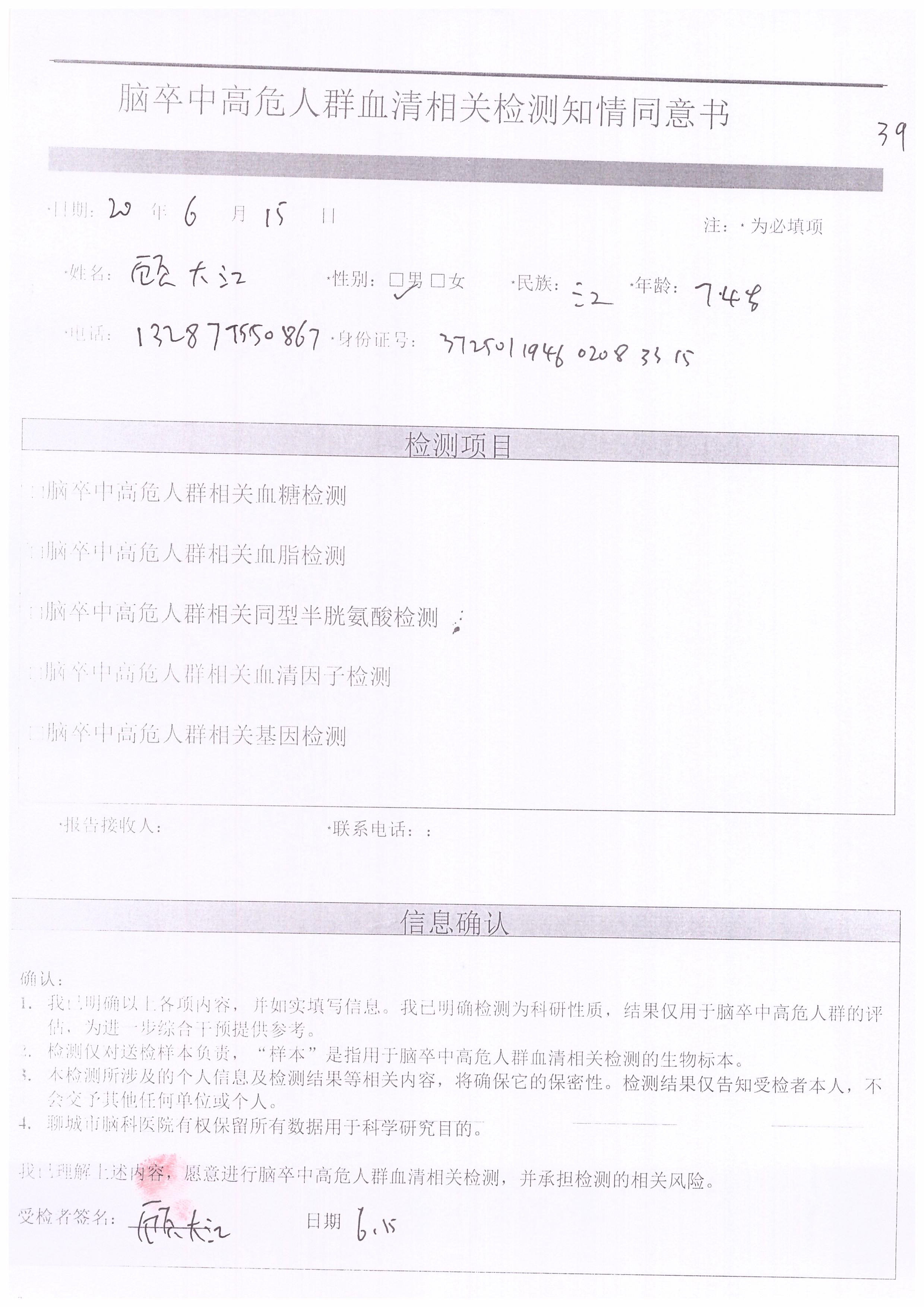

Supplement: Supplementary file 10 — Supplementary file10 (ZIP 21741 KB) [file 10528_2023_10431_MOESM10_ESM.zip › ╓¬╟Θ═1⁄4╥Γ╩Θ8/039.jpg]

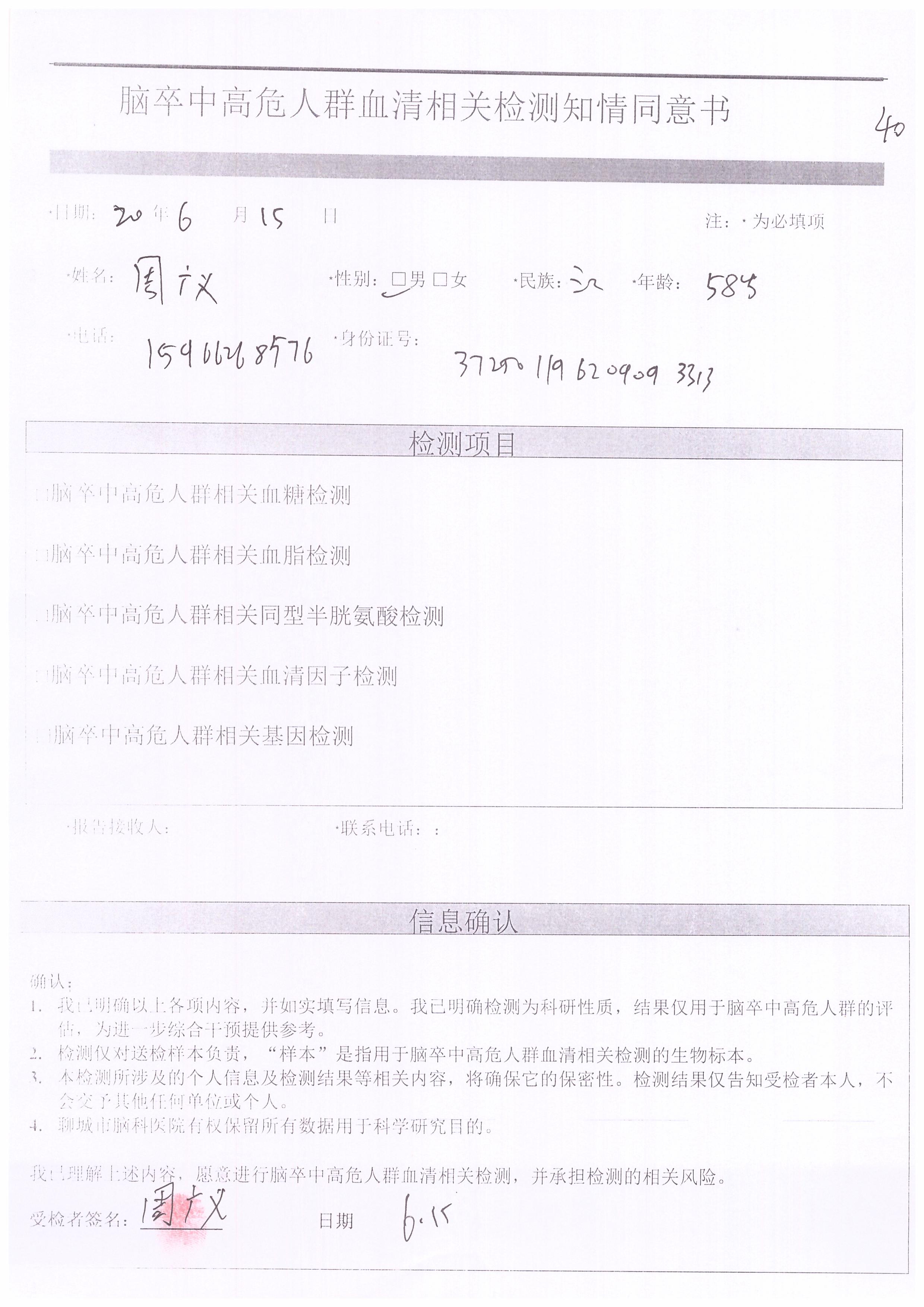

Supplement: Supplementary file 10 — Supplementary file10 (ZIP 21741 KB) [file 10528_2023_10431_MOESM10_ESM.zip › ╓¬╟Θ═1⁄4╥Γ╩Θ8/040.jpg]

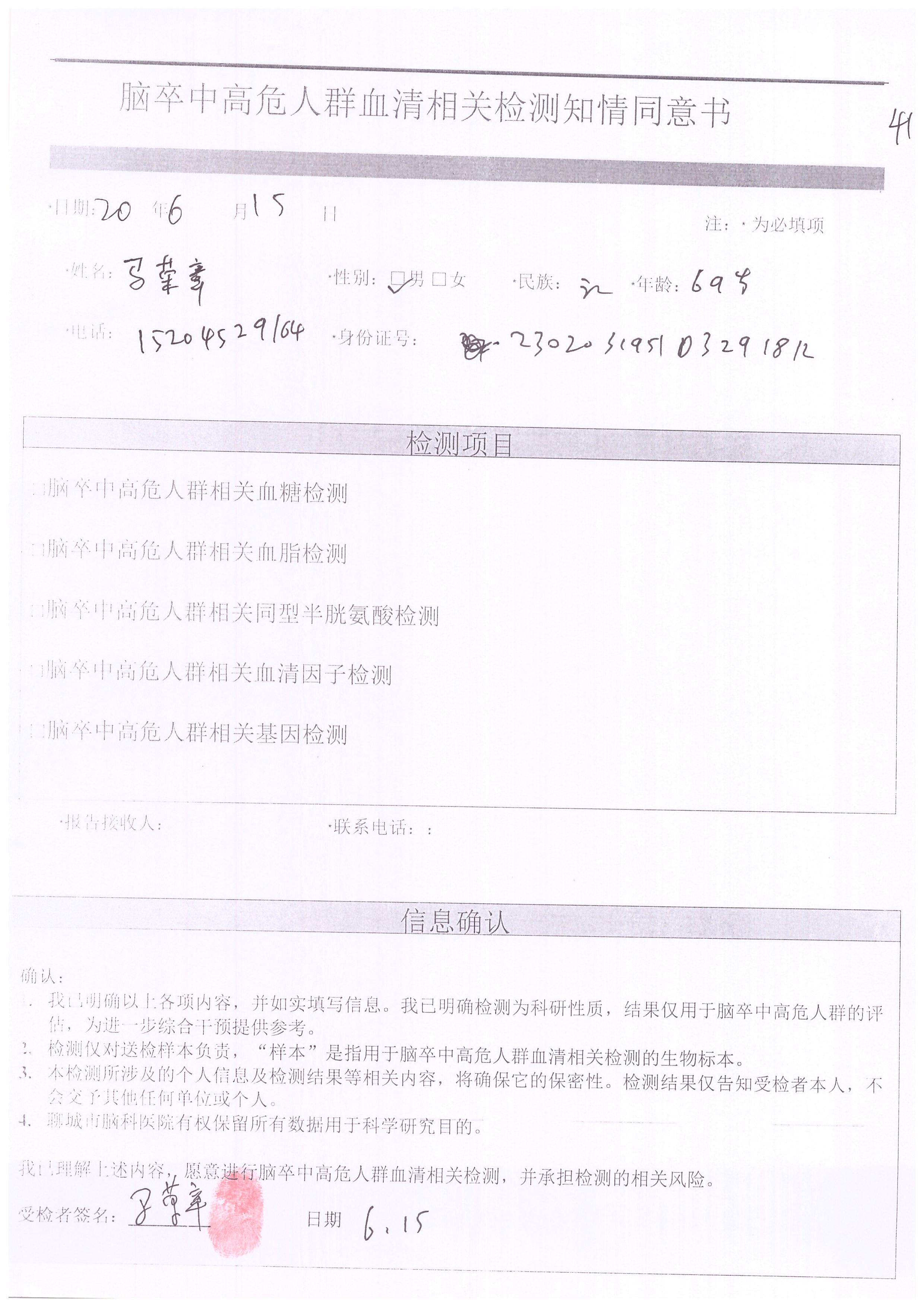

Supplement: Supplementary file 10 — Supplementary file10 (ZIP 21741 KB) [file 10528_2023_10431_MOESM10_ESM.zip › ╓¬╟Θ═1⁄4╥Γ╩Θ8/041.jpg]

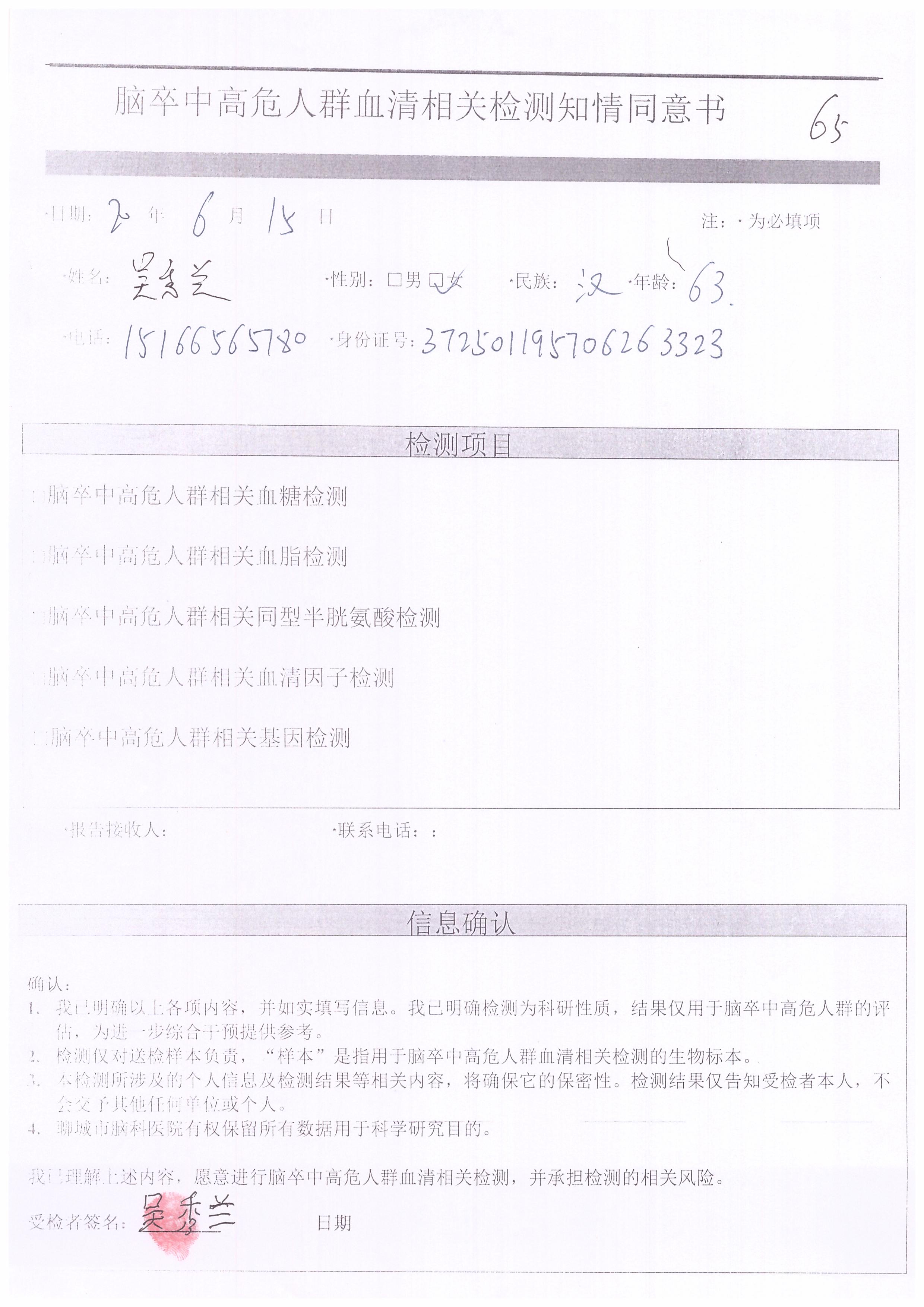

Supplement: Supplementary file 11 — Supplementary file11 (ZIP 25089 KB) [file 10528_2023_10431_MOESM11_ESM.zip › ╓¬╟Θ═1⁄4╥Γ╩Θ9/023.jpg]

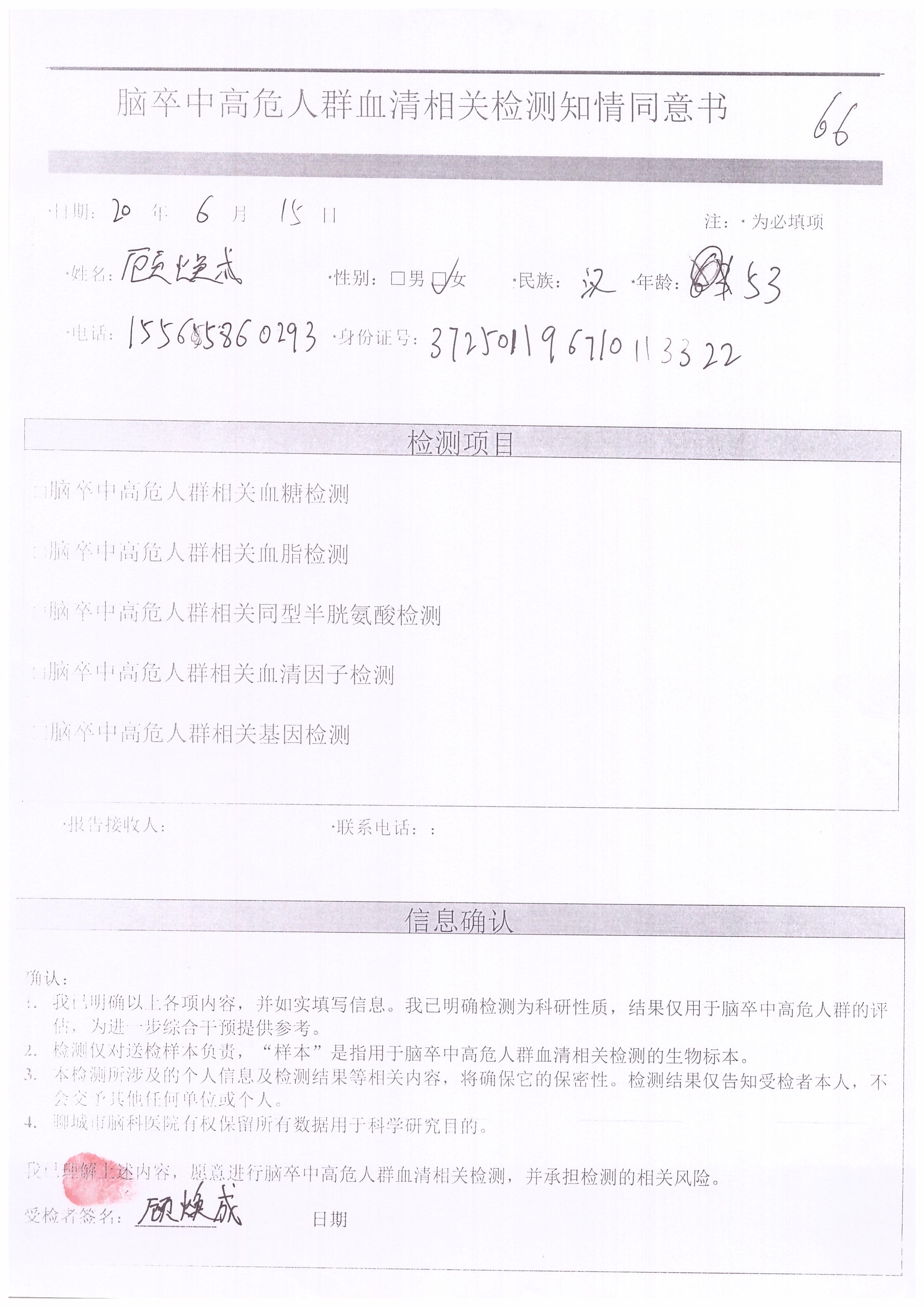

Supplement: Supplementary file 11 — Supplementary file11 (ZIP 25089 KB) [file 10528_2023_10431_MOESM11_ESM.zip › ╓¬╟Θ═1⁄4╥Γ╩Θ9/024.jpg]

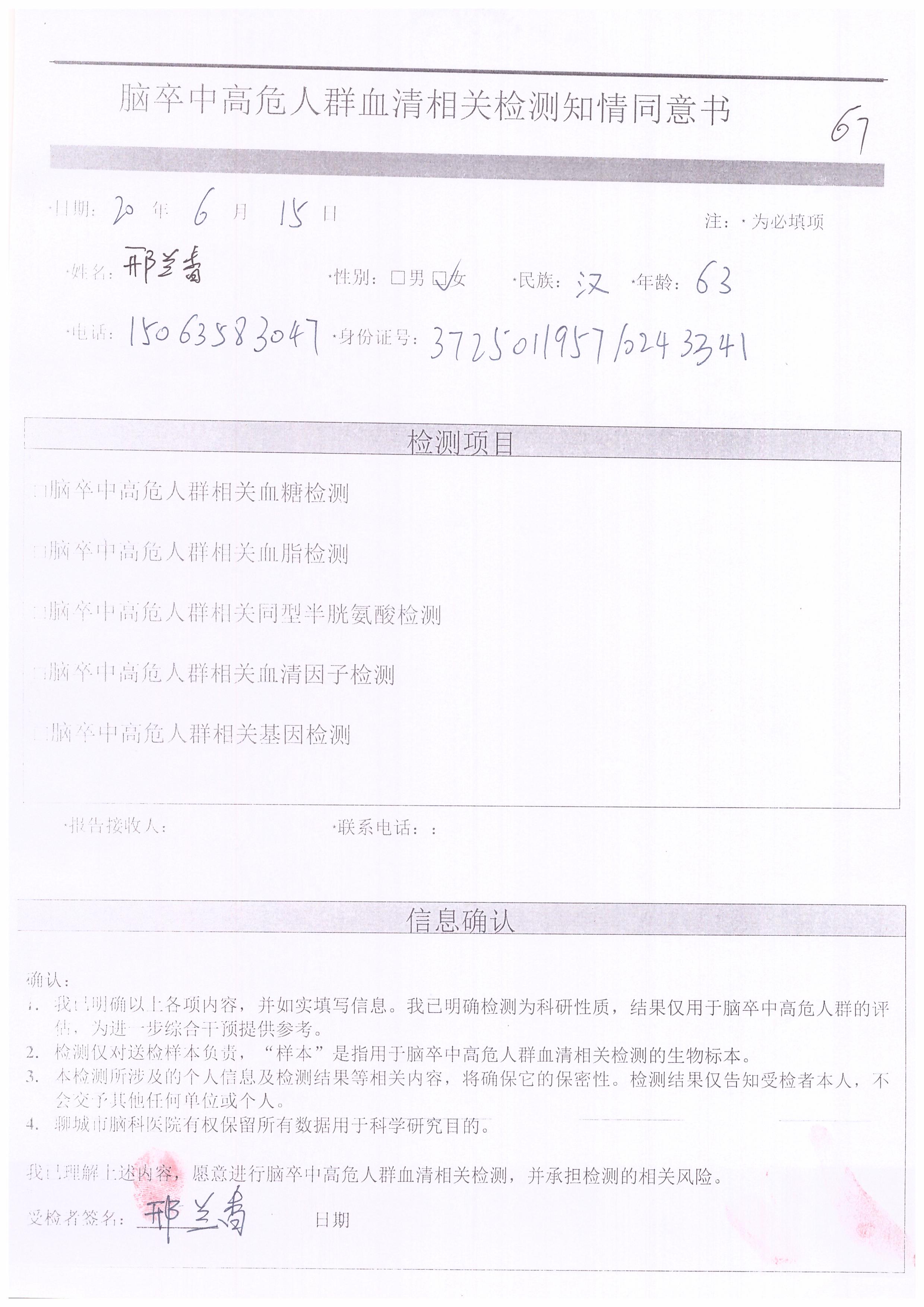

Supplement: Supplementary file 11 — Supplementary file11 (ZIP 25089 KB) [file 10528_2023_10431_MOESM11_ESM.zip › ╓¬╟Θ═1⁄4╥Γ╩Θ9/025.jpg]

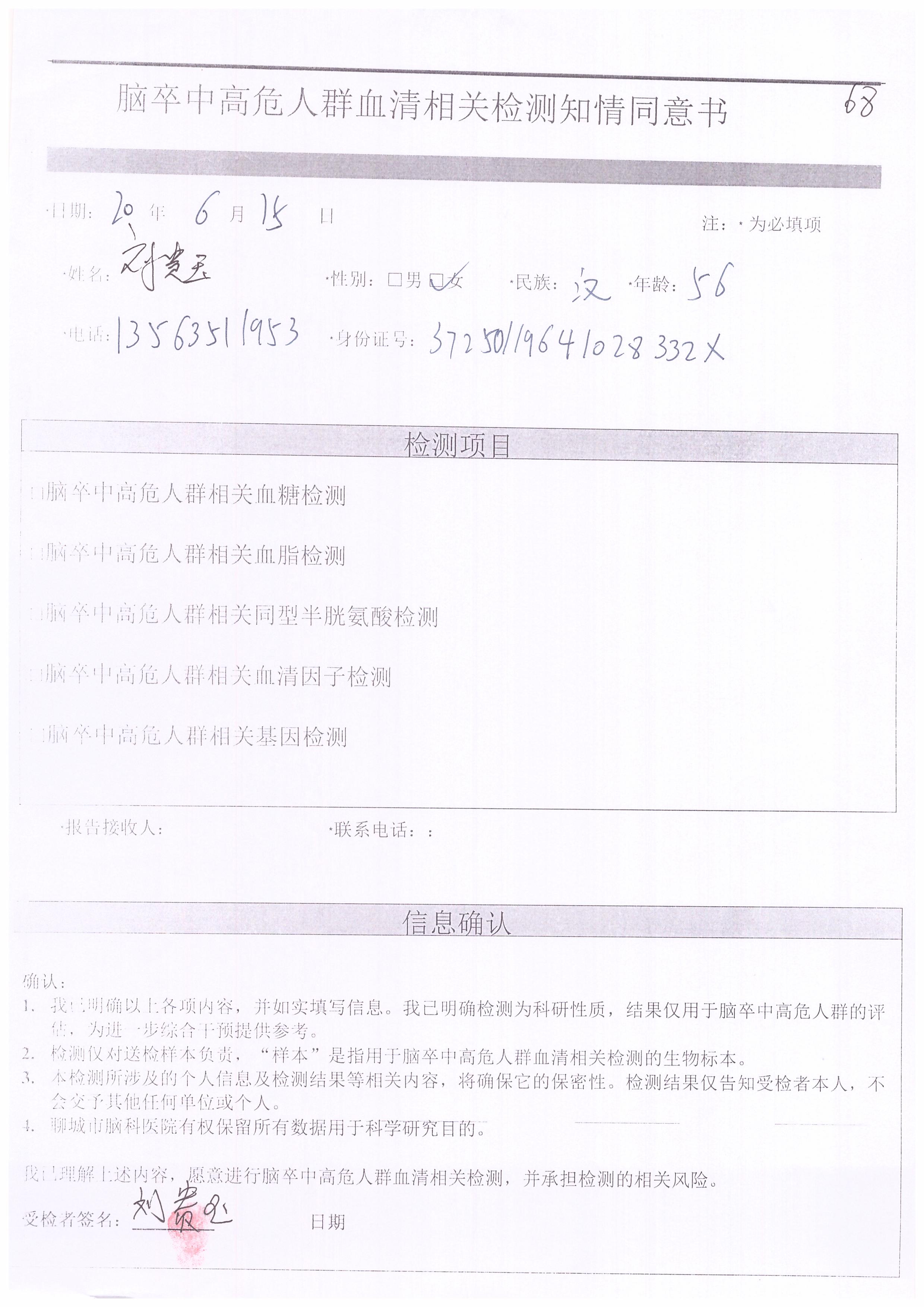

Supplement: Supplementary file 11 — Supplementary file11 (ZIP 25089 KB) [file 10528_2023_10431_MOESM11_ESM.zip › ╓¬╟Θ═1⁄4╥Γ╩Θ9/026.jpg]

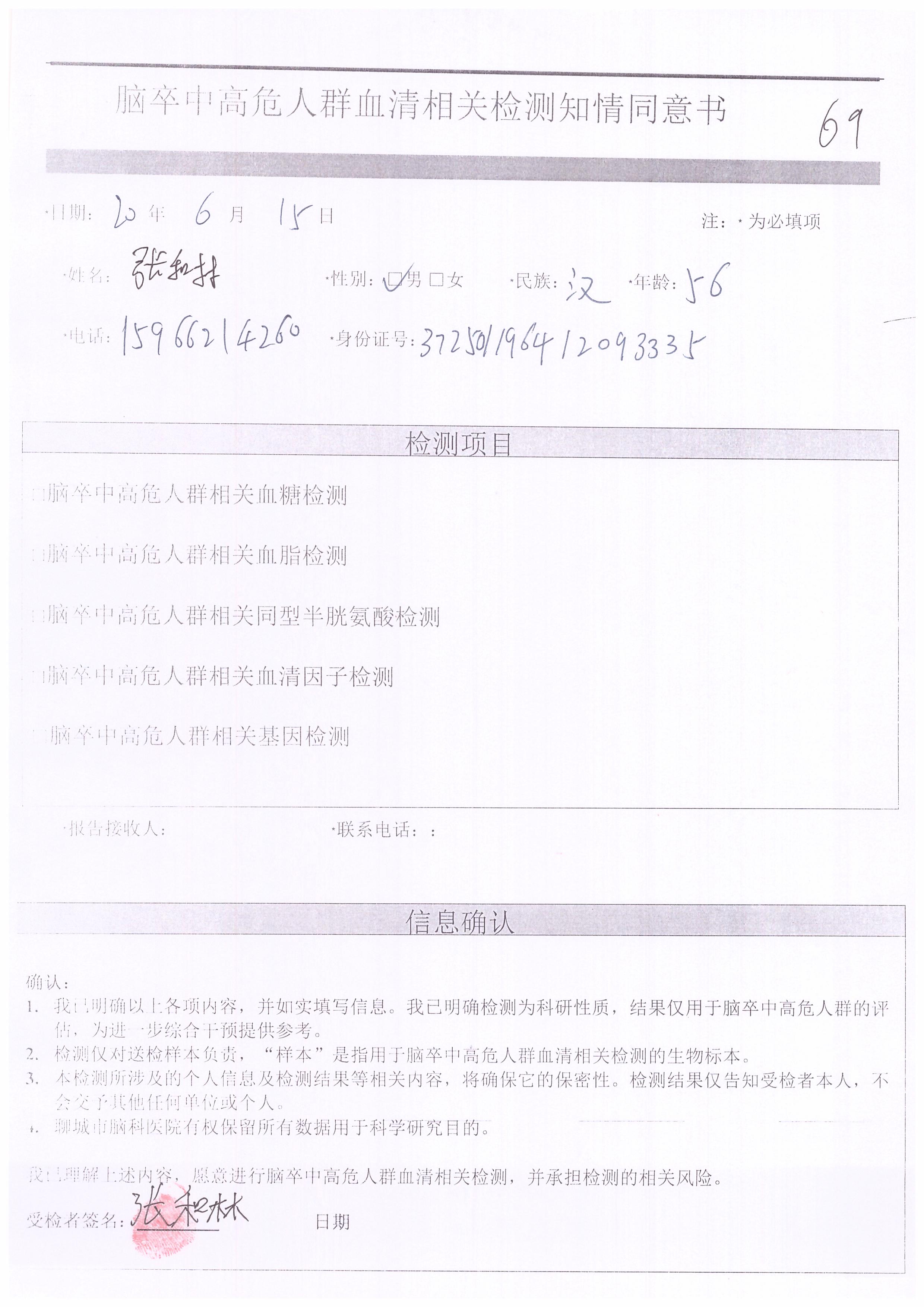

Supplement: Supplementary file 11 — Supplementary file11 (ZIP 25089 KB) [file 10528_2023_10431_MOESM11_ESM.zip › ╓¬╟Θ═1⁄4╥Γ╩Θ9/027.jpg]

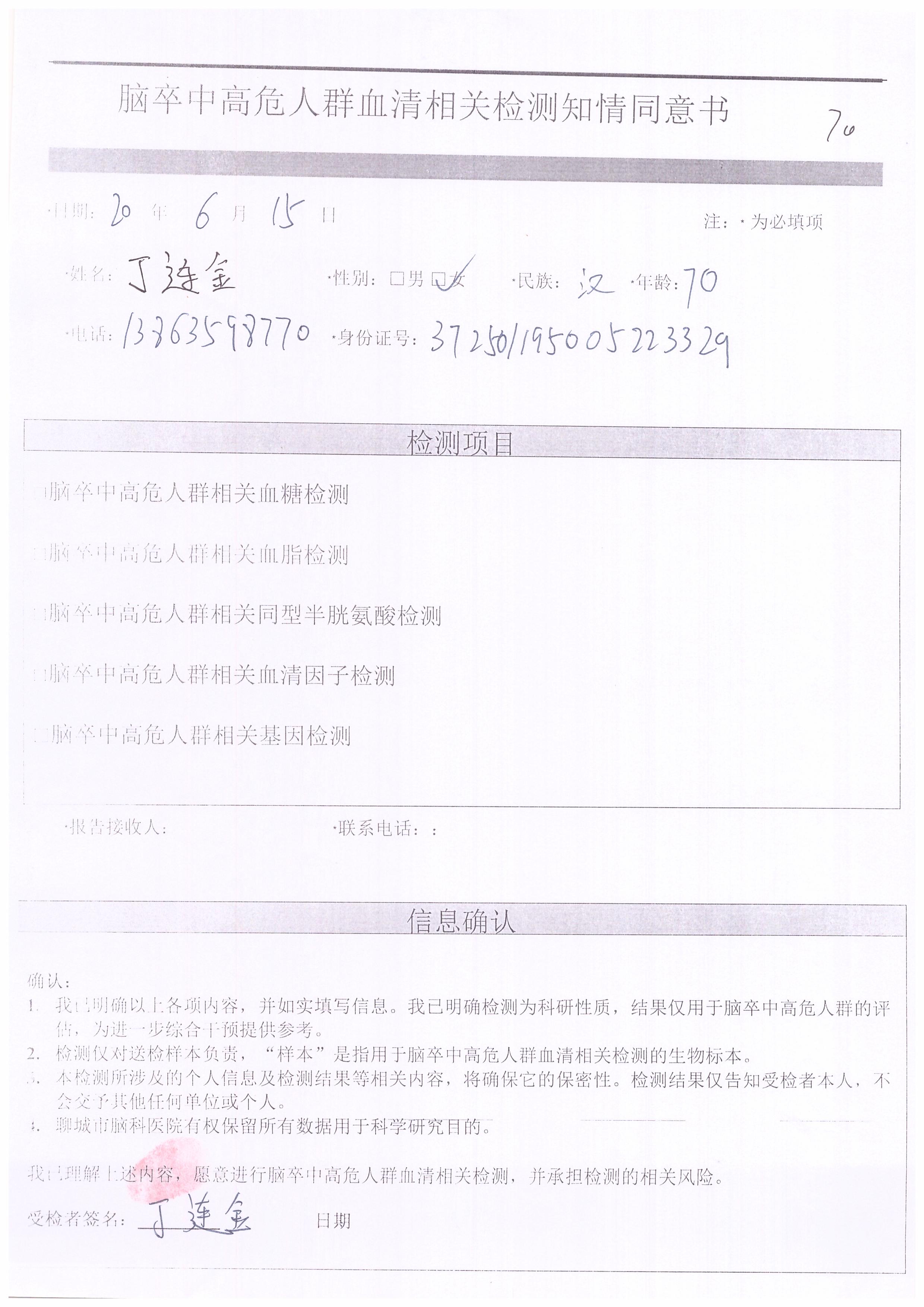

Supplement: Supplementary file 11 — Supplementary file11 (ZIP 25089 KB) [file 10528_2023_10431_MOESM11_ESM.zip › ╓¬╟Θ═1⁄4╥Γ╩Θ9/028.jpg]

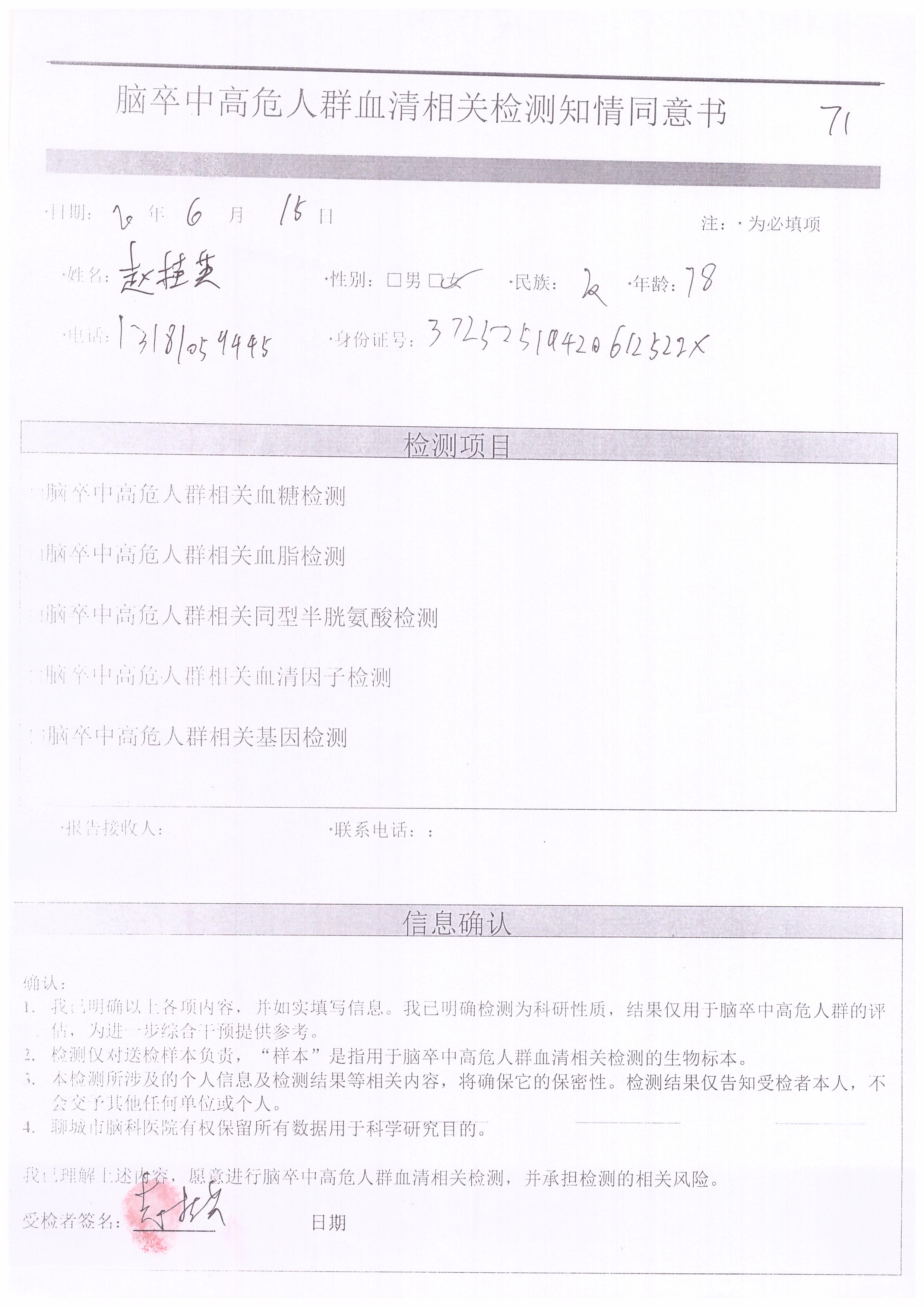

Supplement: Supplementary file 11 — Supplementary file11 (ZIP 25089 KB) [file 10528_2023_10431_MOESM11_ESM.zip › ╓¬╟Θ═1⁄4╥Γ╩Θ9/029.jpg]

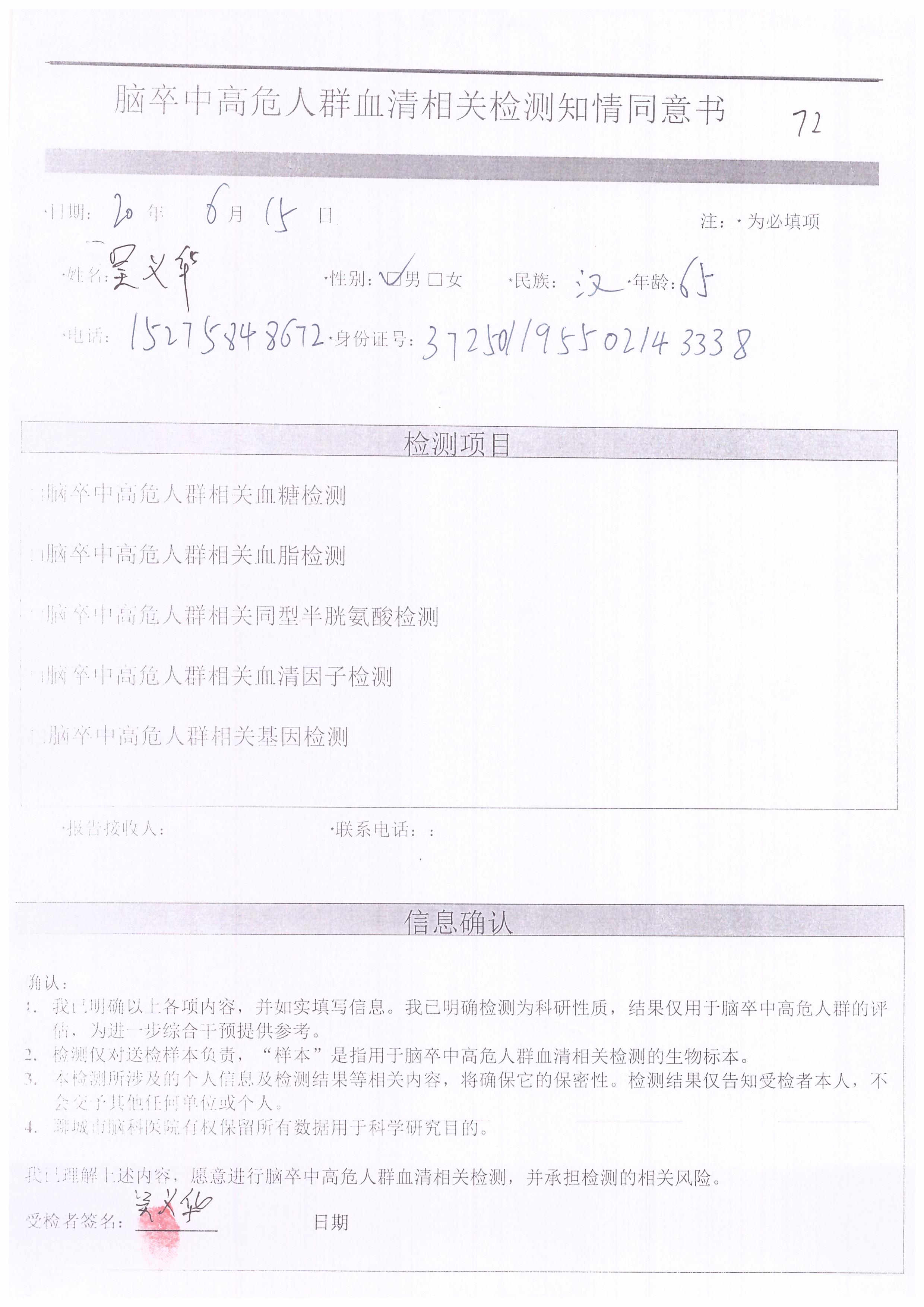

Supplement: Supplementary file 11 — Supplementary file11 (ZIP 25089 KB) [file 10528_2023_10431_MOESM11_ESM.zip › ╓¬╟Θ═1⁄4╥Γ╩Θ9/030.jpg]

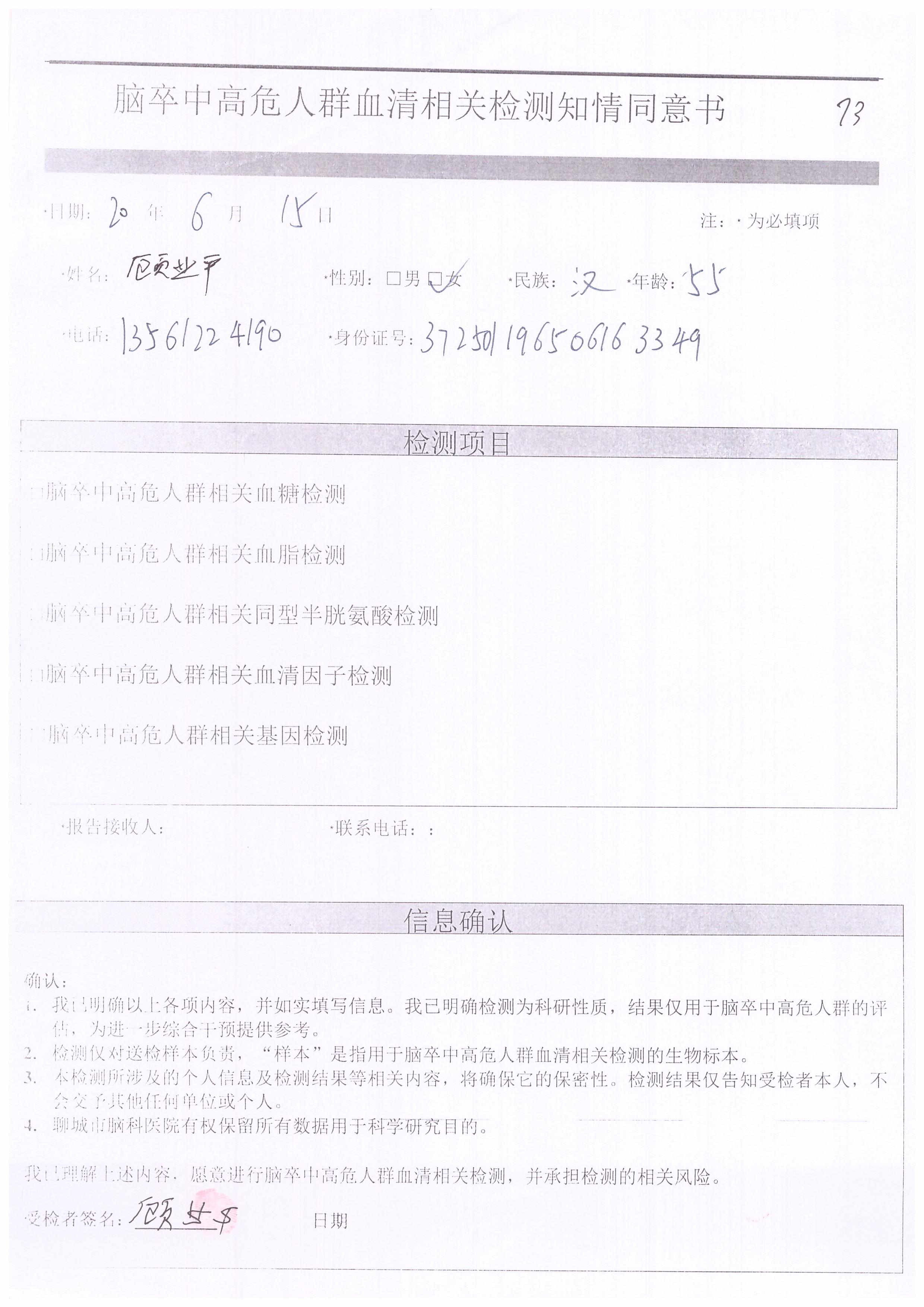

Supplement: Supplementary file 11 — Supplementary file11 (ZIP 25089 KB) [file 10528_2023_10431_MOESM11_ESM.zip › ╓¬╟Θ═1⁄4╥Γ╩Θ9/031.jpg]

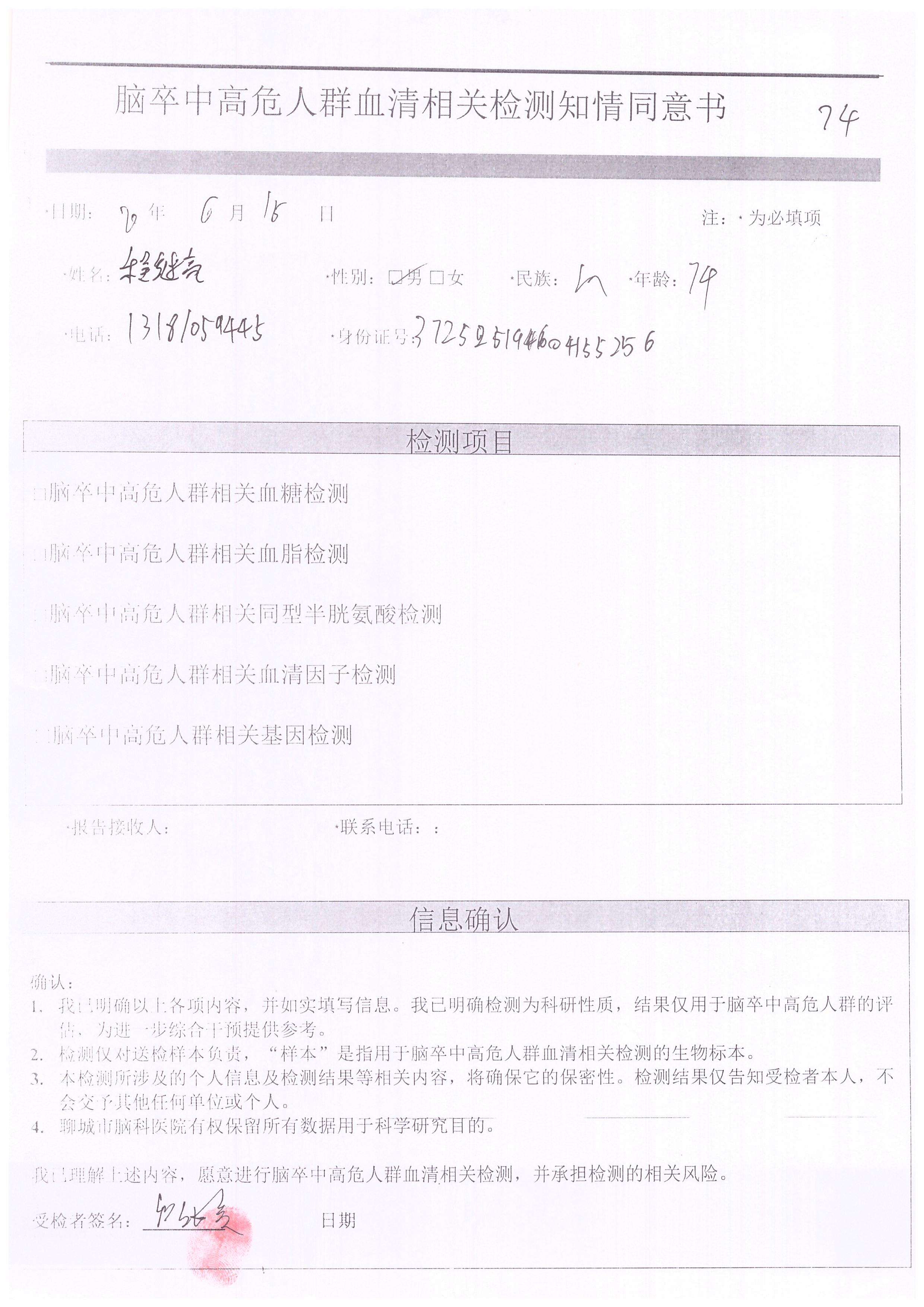

Supplement: Supplementary file 11 — Supplementary file11 (ZIP 25089 KB) [file 10528_2023_10431_MOESM11_ESM.zip › ╓¬╟Θ═1⁄4╥Γ╩Θ9/032.jpg]

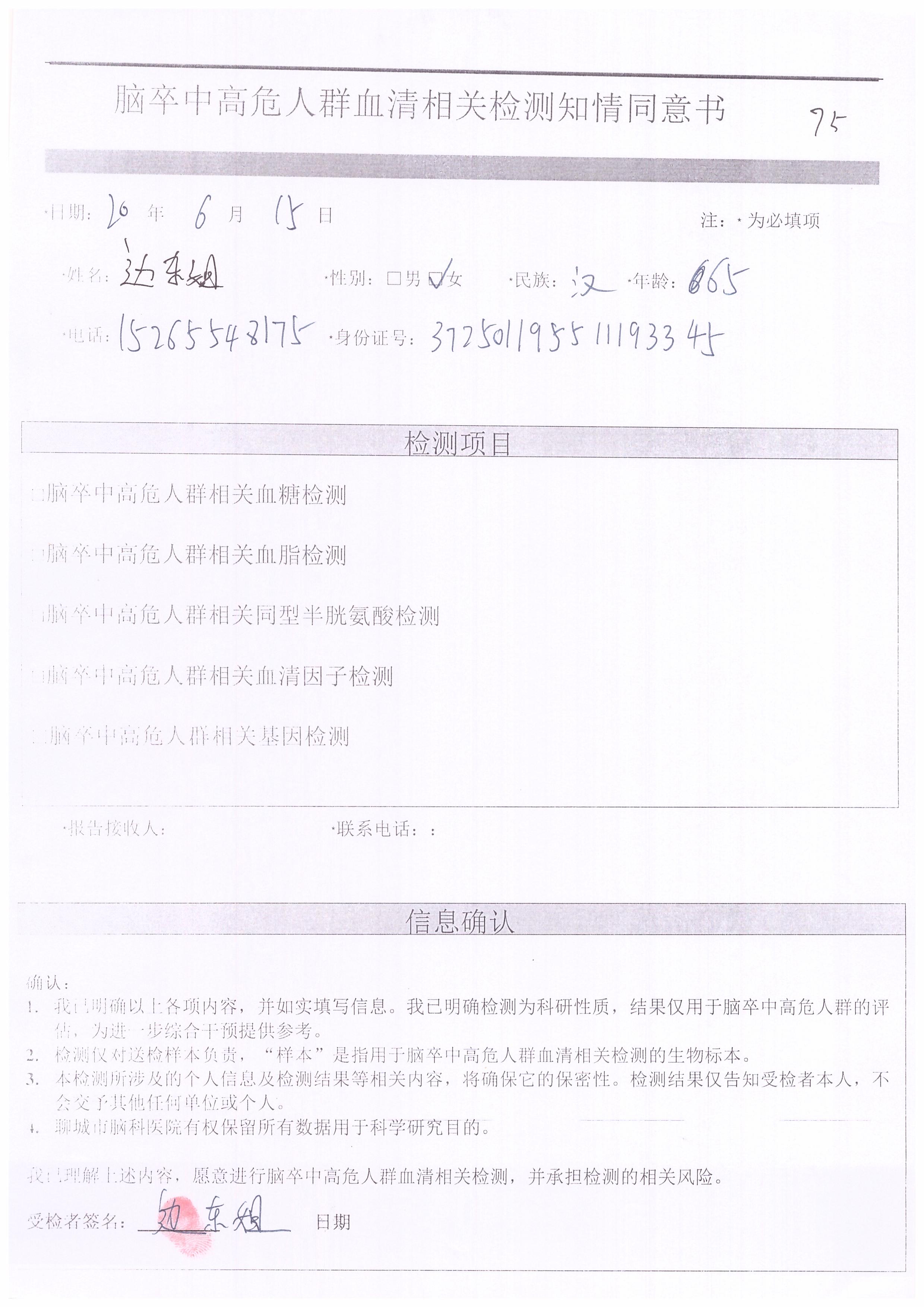

Supplement: Supplementary file 11 — Supplementary file11 (ZIP 25089 KB) [file 10528_2023_10431_MOESM11_ESM.zip › ╓¬╟Θ═1⁄4╥Γ╩Θ9/033.jpg]

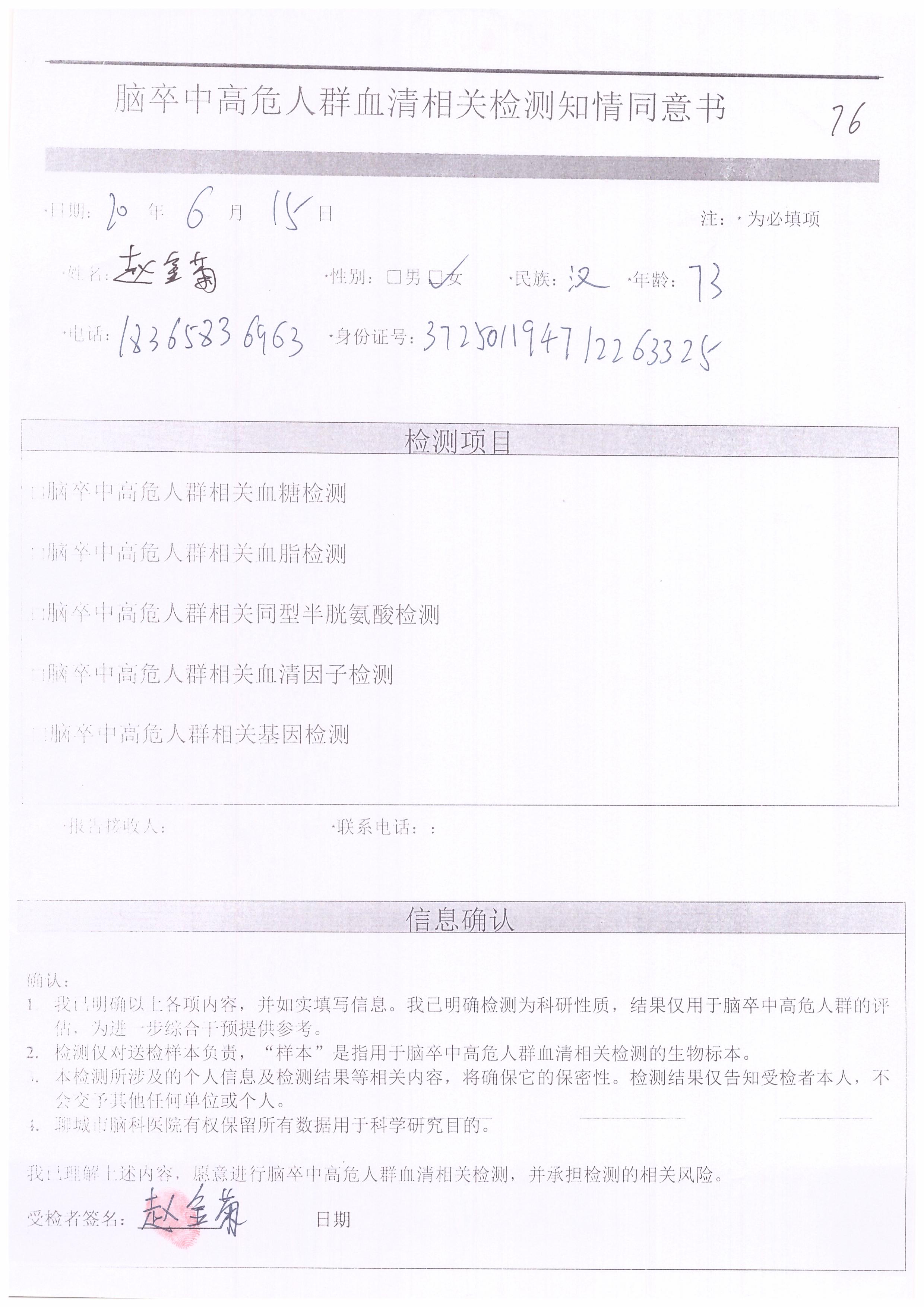

Supplement: Supplementary file 11 — Supplementary file11 (ZIP 25089 KB) [file 10528_2023_10431_MOESM11_ESM.zip › ╓¬╟Θ═1⁄4╥Γ╩Θ9/034.jpg]

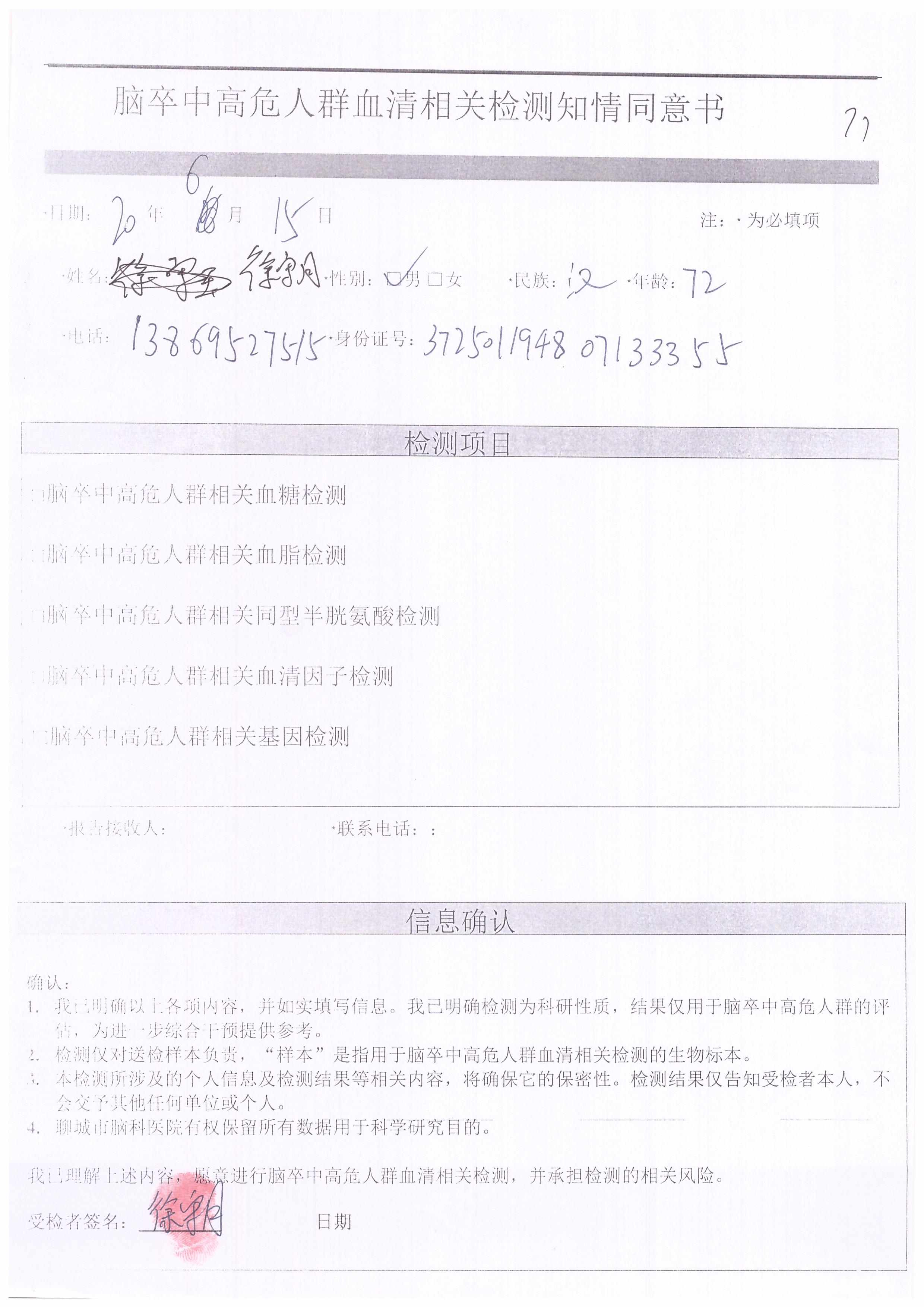

Supplement: Supplementary file 11 — Supplementary file11 (ZIP 25089 KB) [file 10528_2023_10431_MOESM11_ESM.zip › ╓¬╟Θ═1⁄4╥Γ╩Θ9/035.jpg]

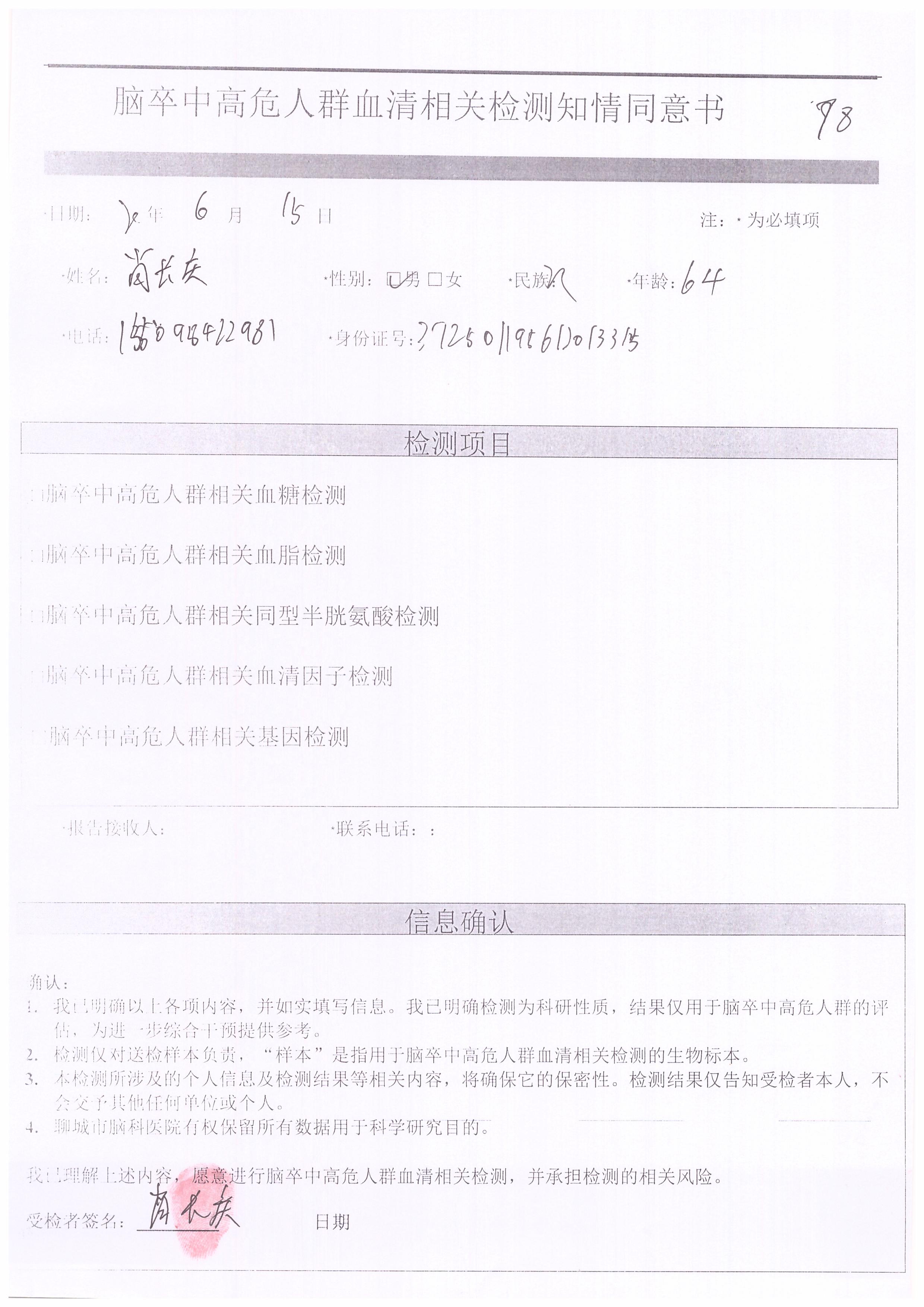

Supplement: Supplementary file 11 — Supplementary file11 (ZIP 25089 KB) [file 10528_2023_10431_MOESM11_ESM.zip › ╓¬╟Θ═1⁄4╥Γ╩Θ9/036.jpg]

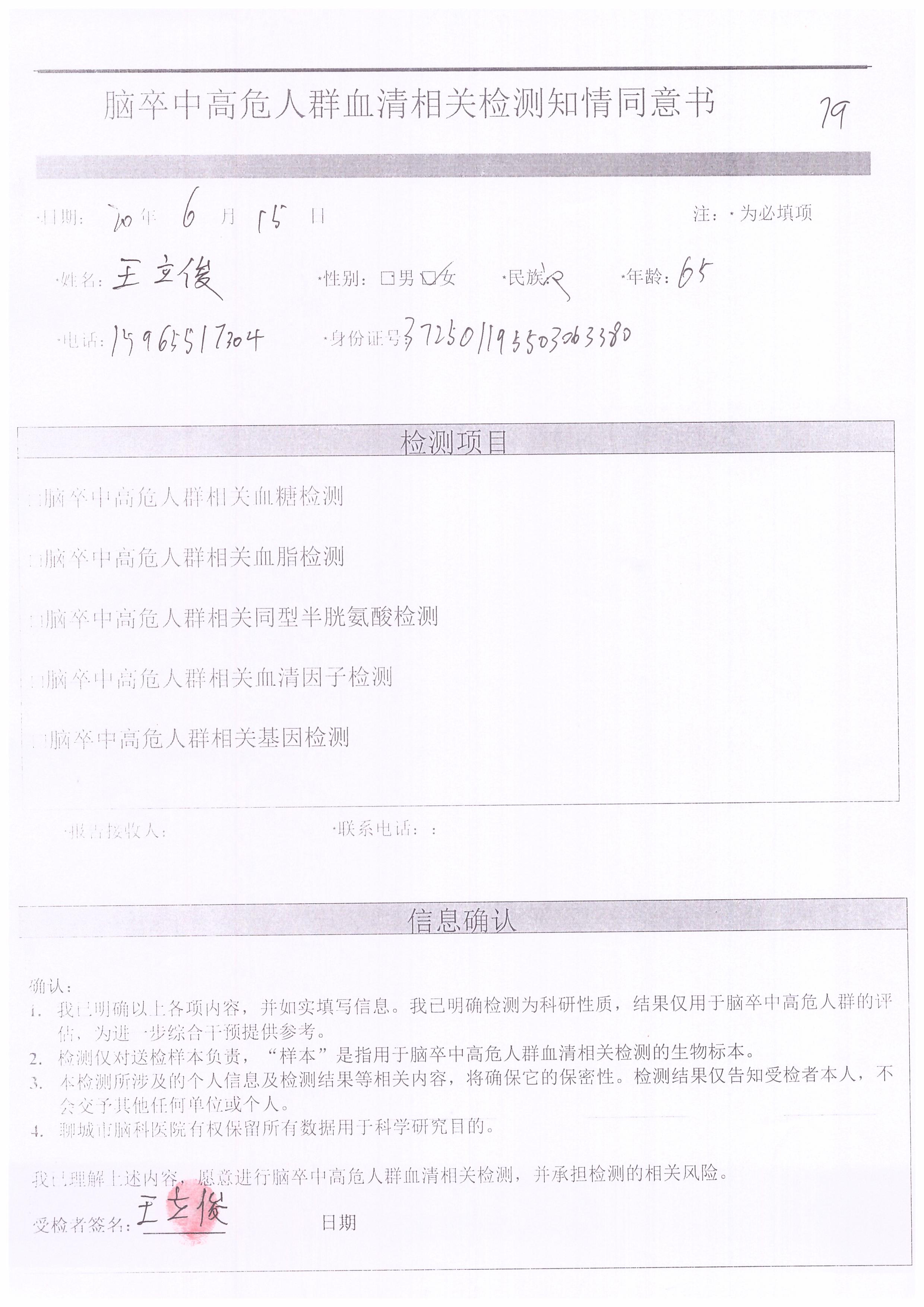

Supplement: Supplementary file 11 — Supplementary file11 (ZIP 25089 KB) [file 10528_2023_10431_MOESM11_ESM.zip › ╓¬╟Θ═1⁄4╥Γ╩Θ9/037.jpg]

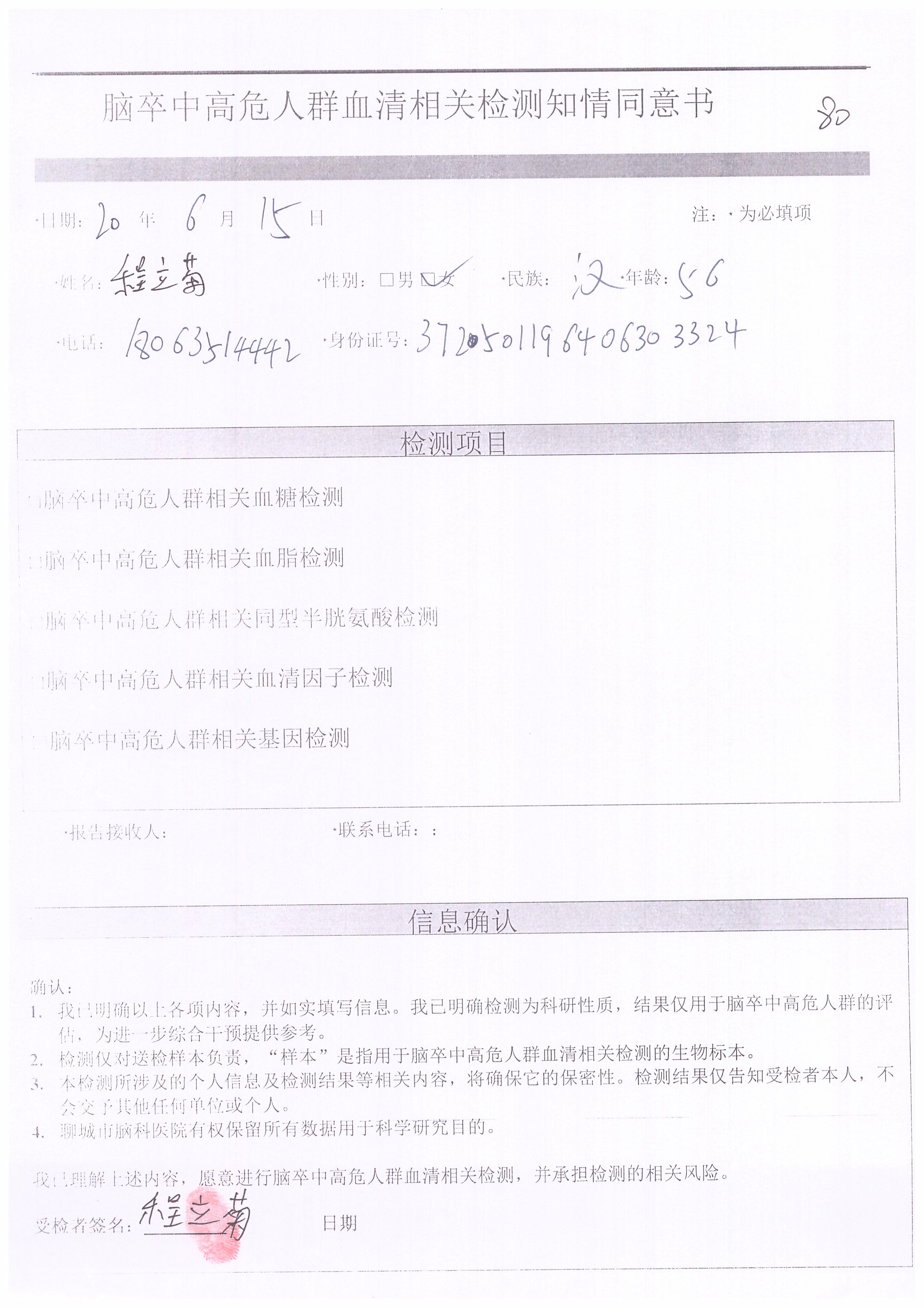

Supplement: Supplementary file 11 — Supplementary file11 (ZIP 25089 KB) [file 10528_2023_10431_MOESM11_ESM.zip › ╓¬╟Θ═1⁄4╥Γ╩Θ9/038.jpg]

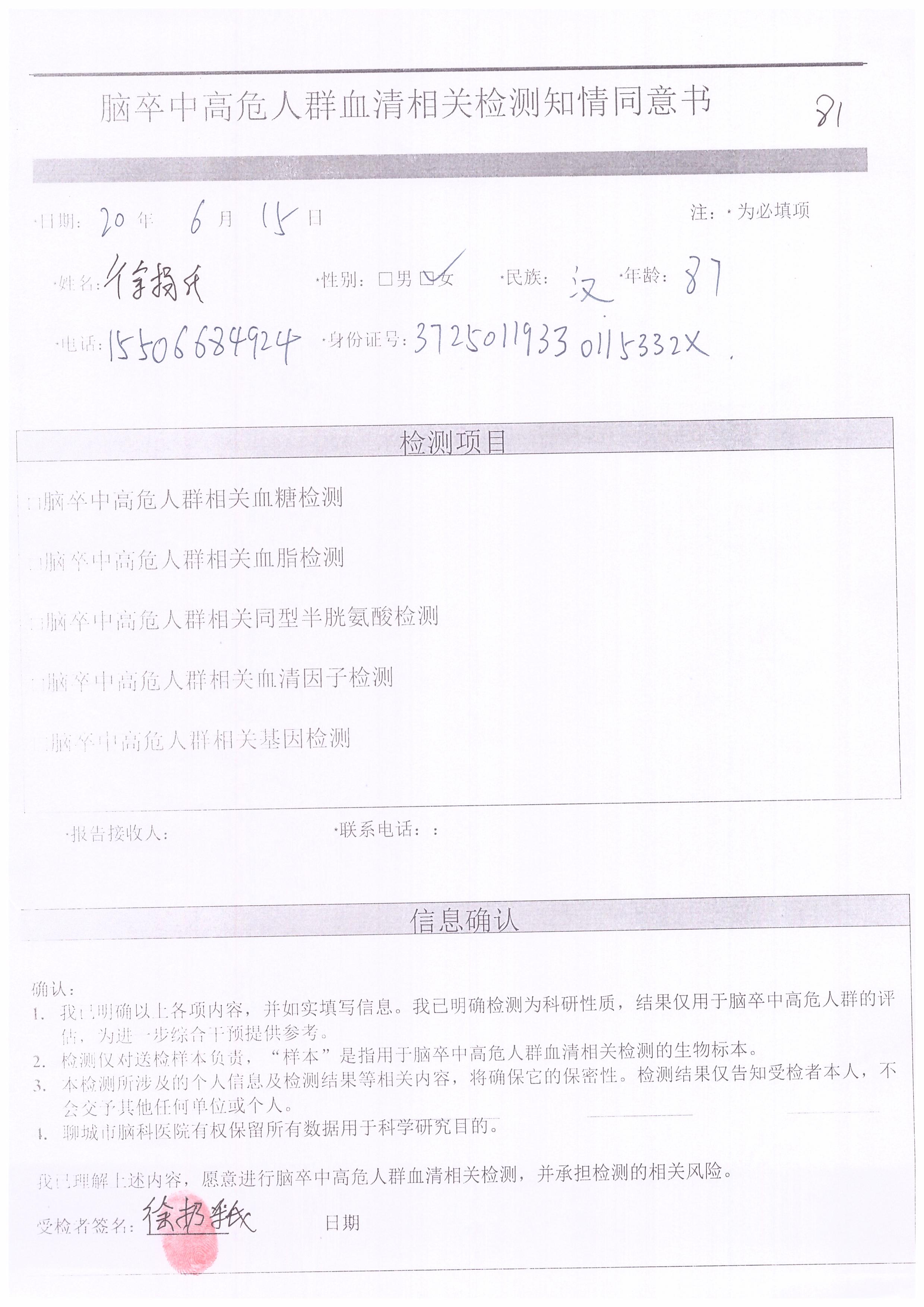

Supplement: Supplementary file 11 — Supplementary file11 (ZIP 25089 KB) [file 10528_2023_10431_MOESM11_ESM.zip › ╓¬╟Θ═1⁄4╥Γ╩Θ9/039.jpg]

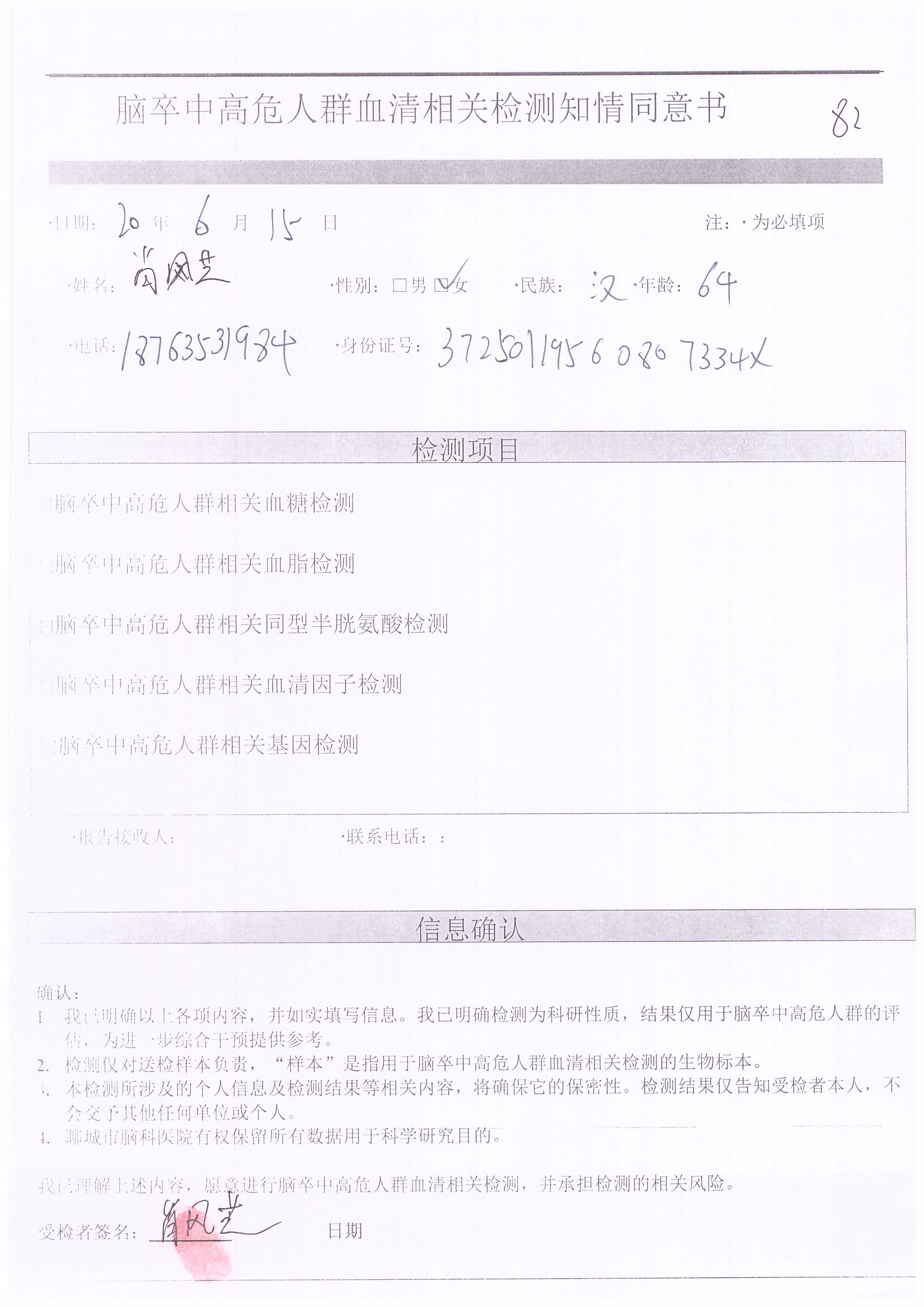

Supplement: Supplementary file 11 — Supplementary file11 (ZIP 25089 KB) [file 10528_2023_10431_MOESM11_ESM.zip › ╓¬╟Θ═1⁄4╥Γ╩Θ9/040.jpg]

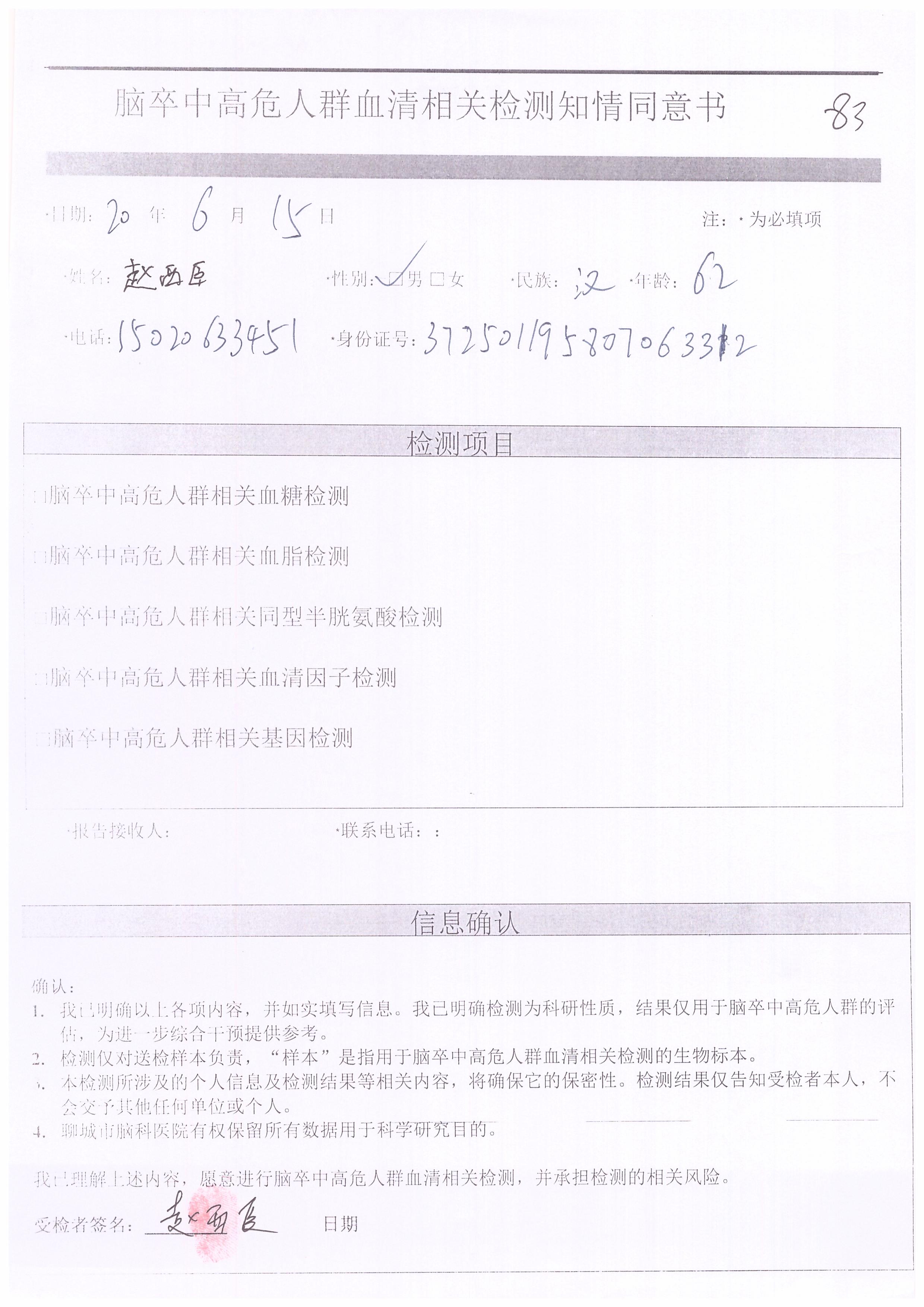

Supplement: Supplementary file 11 — Supplementary file11 (ZIP 25089 KB) [file 10528_2023_10431_MOESM11_ESM.zip › ╓¬╟Θ═1⁄4╥Γ╩Θ9/041.jpg]

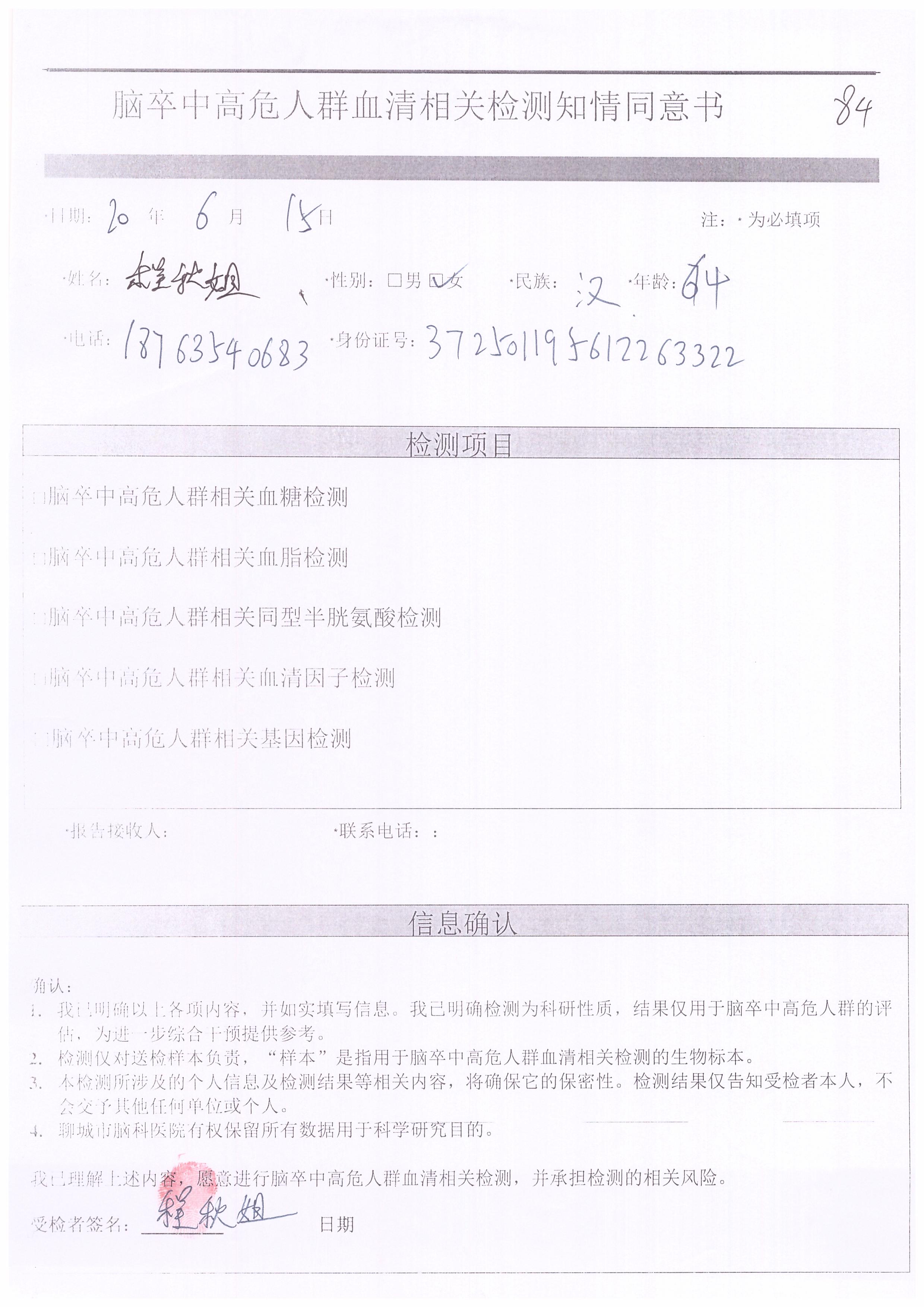

Supplement: Supplementary file 11 — Supplementary file11 (ZIP 25089 KB) [file 10528_2023_10431_MOESM11_ESM.zip › ╓¬╟Θ═1⁄4╥Γ╩Θ9/042.jpg]

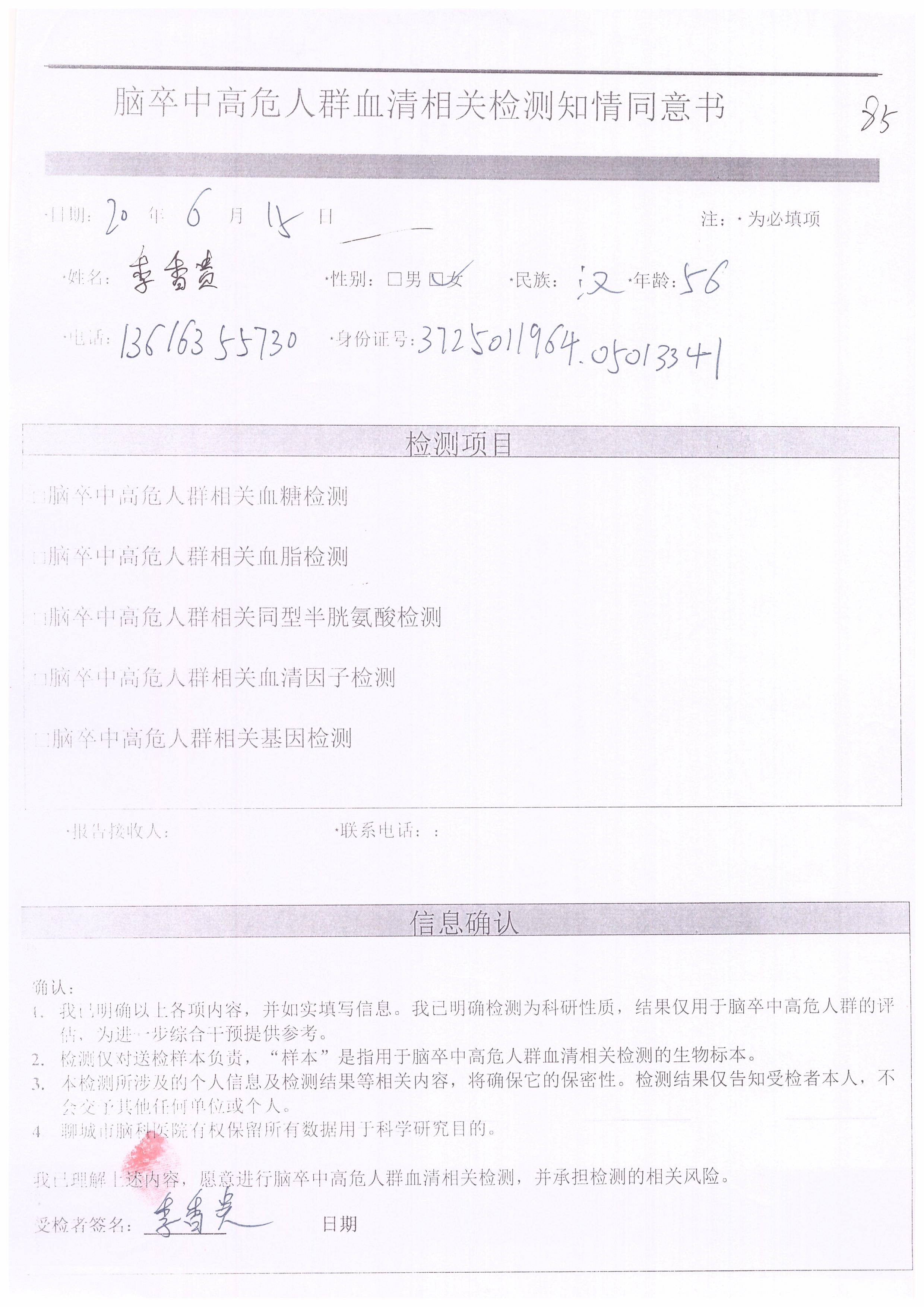

Supplement: Supplementary file 11 — Supplementary file11 (ZIP 25089 KB) [file 10528_2023_10431_MOESM11_ESM.zip › ╓¬╟Θ═1⁄4╥Γ╩Θ9/043.jpg]

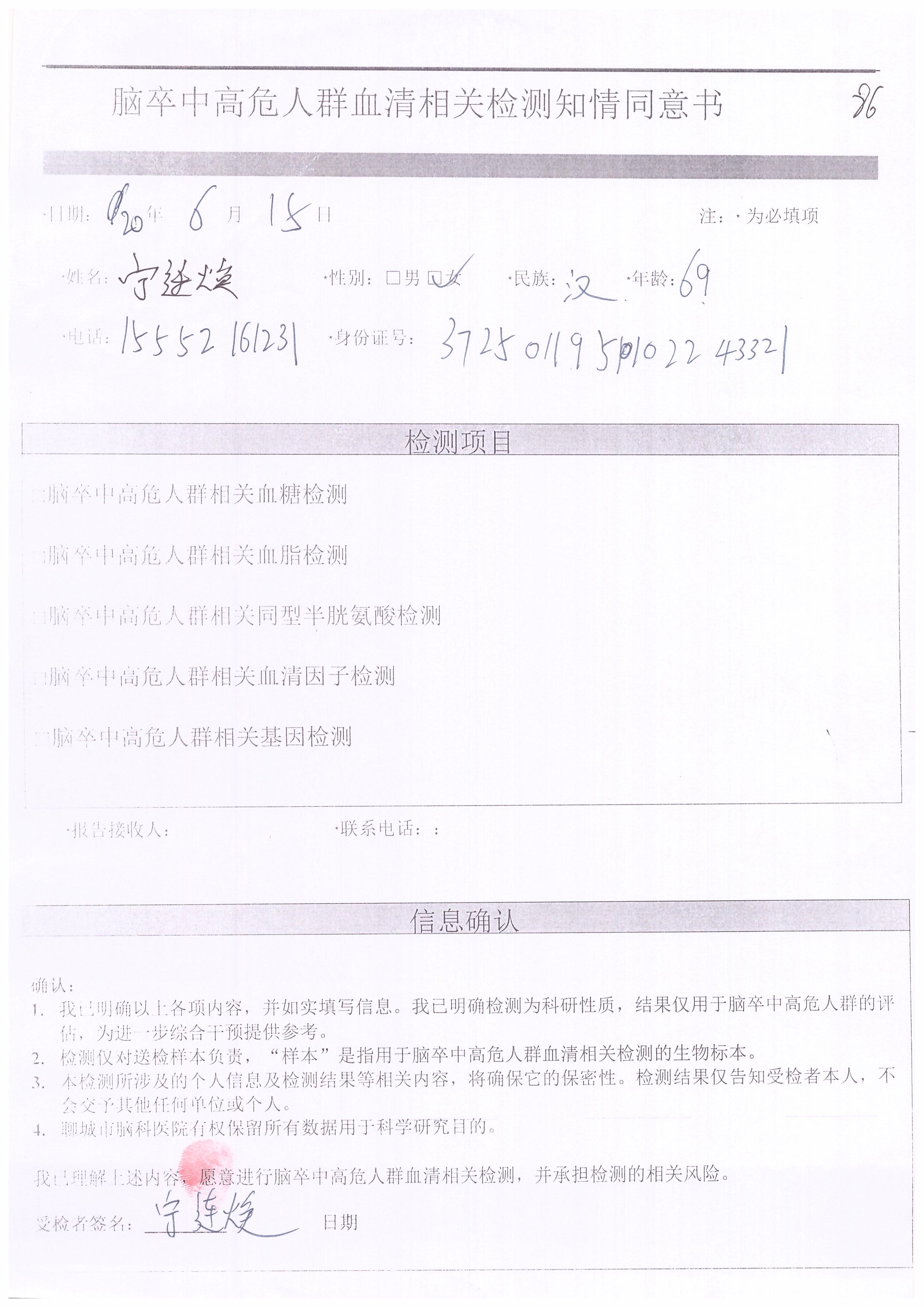

Supplement: Supplementary file 11 — Supplementary file11 (ZIP 25089 KB) [file 10528_2023_10431_MOESM11_ESM.zip › ╓¬╟Θ═1⁄4╥Γ╩Θ9/044.jpg]

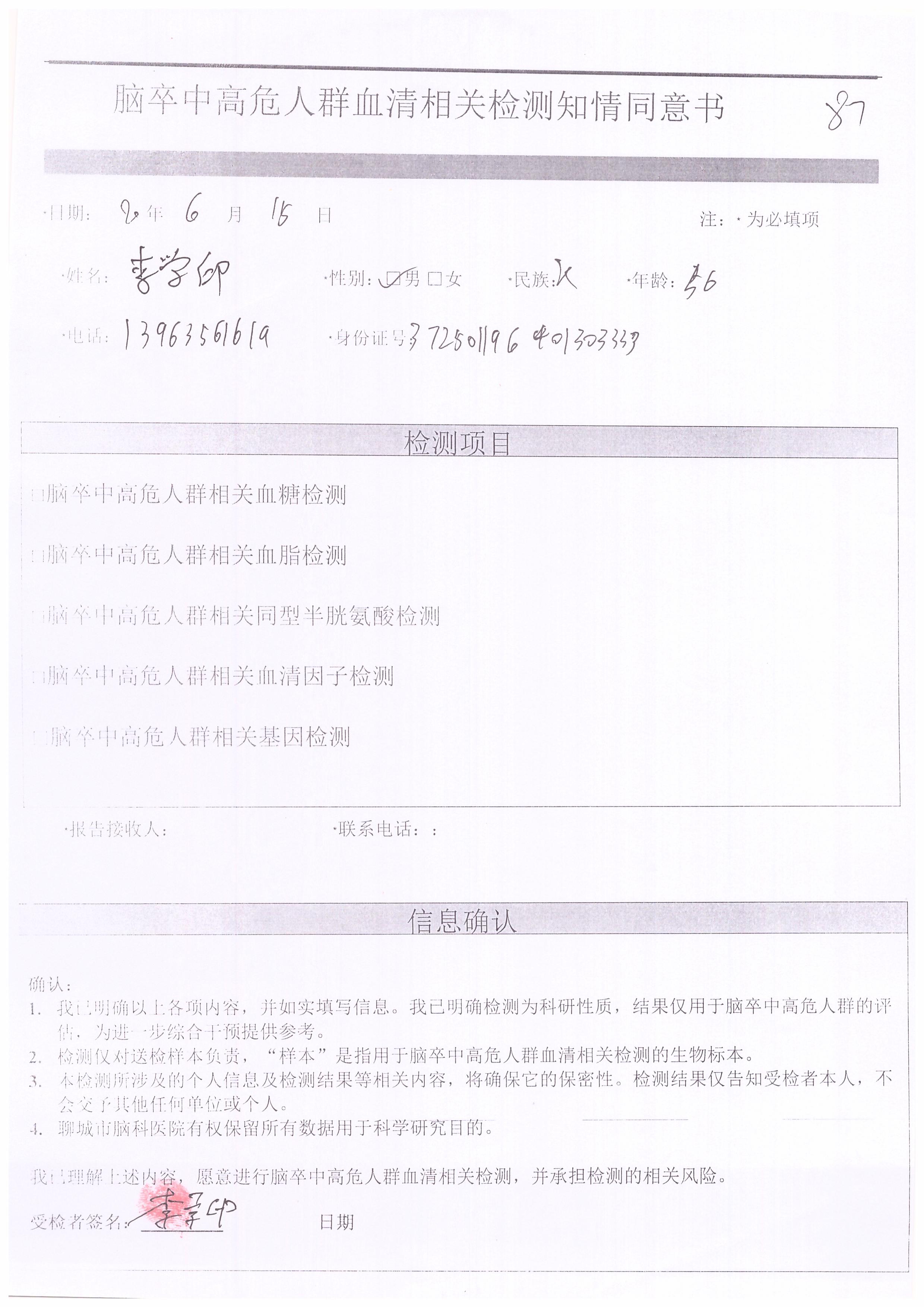

Supplement: Supplementary file 11 — Supplementary file11 (ZIP 25089 KB) [file 10528_2023_10431_MOESM11_ESM.zip › ╓¬╟Θ═1⁄4╥Γ╩Θ9/045.jpg]

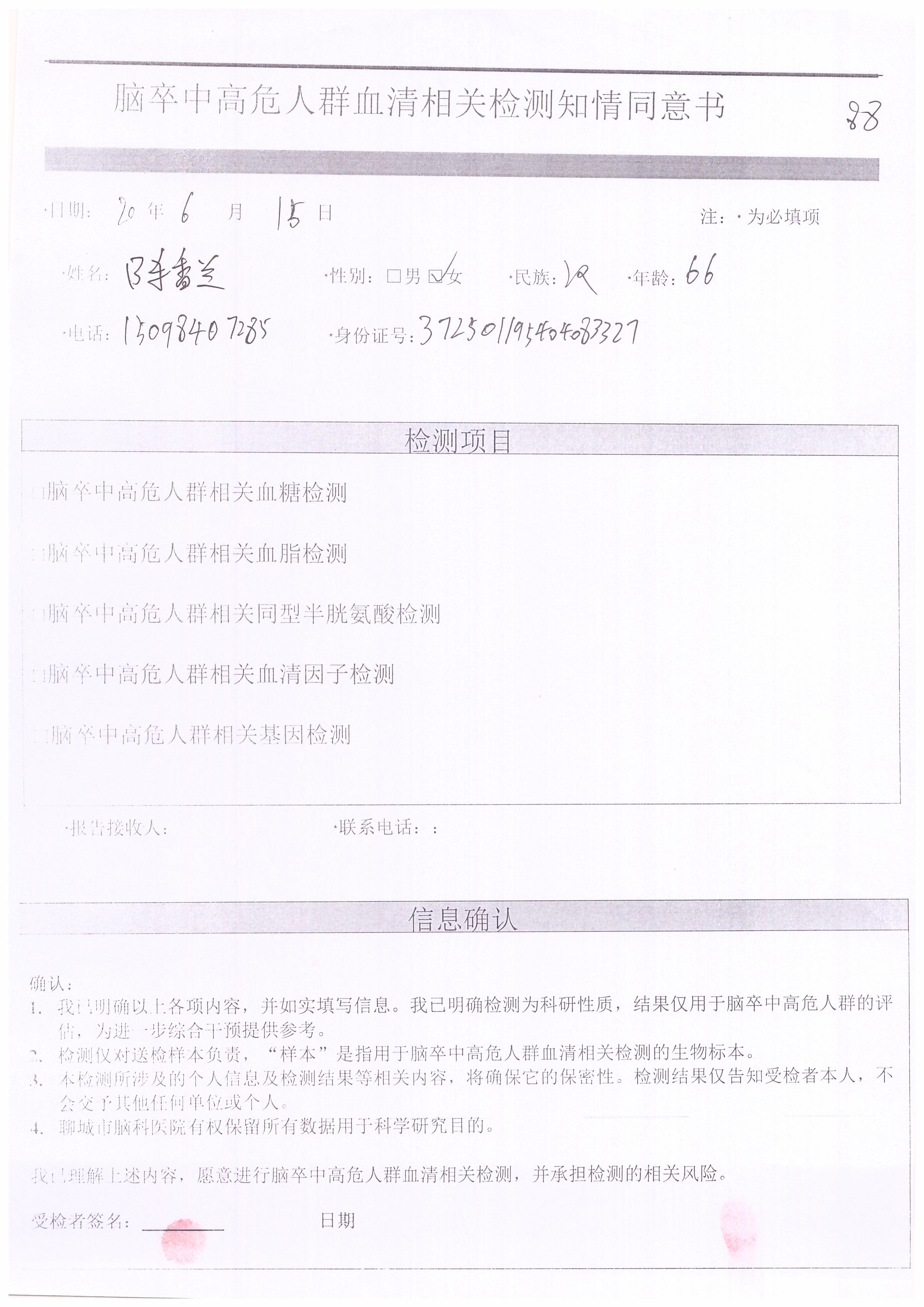

Supplement: Supplementary file 11 — Supplementary file11 (ZIP 25089 KB) [file 10528_2023_10431_MOESM11_ESM.zip › ╓¬╟Θ═1⁄4╥Γ╩Θ9/046.jpg]

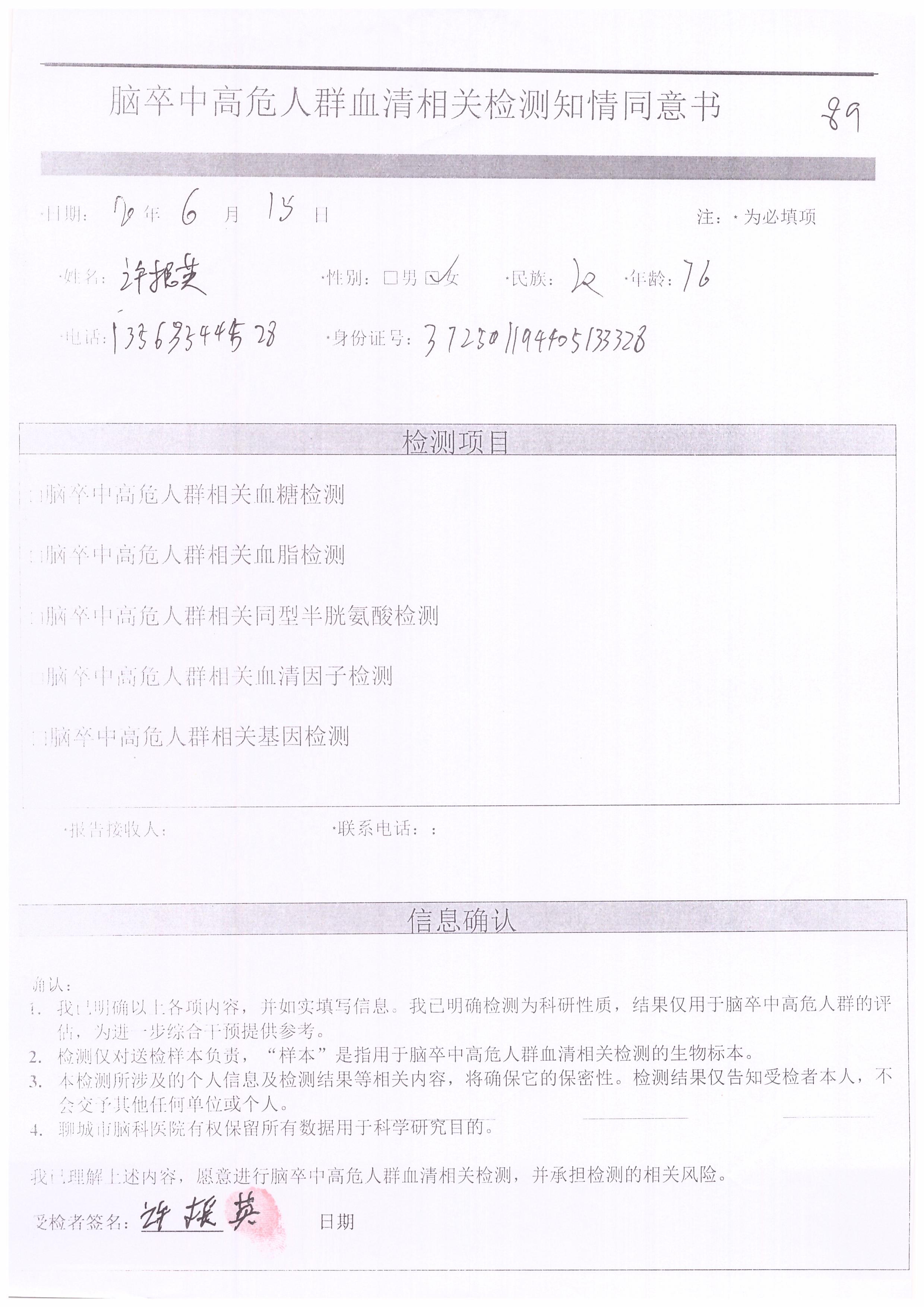

Supplement: Supplementary file 11 — Supplementary file11 (ZIP 25089 KB) [file 10528_2023_10431_MOESM11_ESM.zip › ╓¬╟Θ═1⁄4╥Γ╩Θ9/047.jpg]

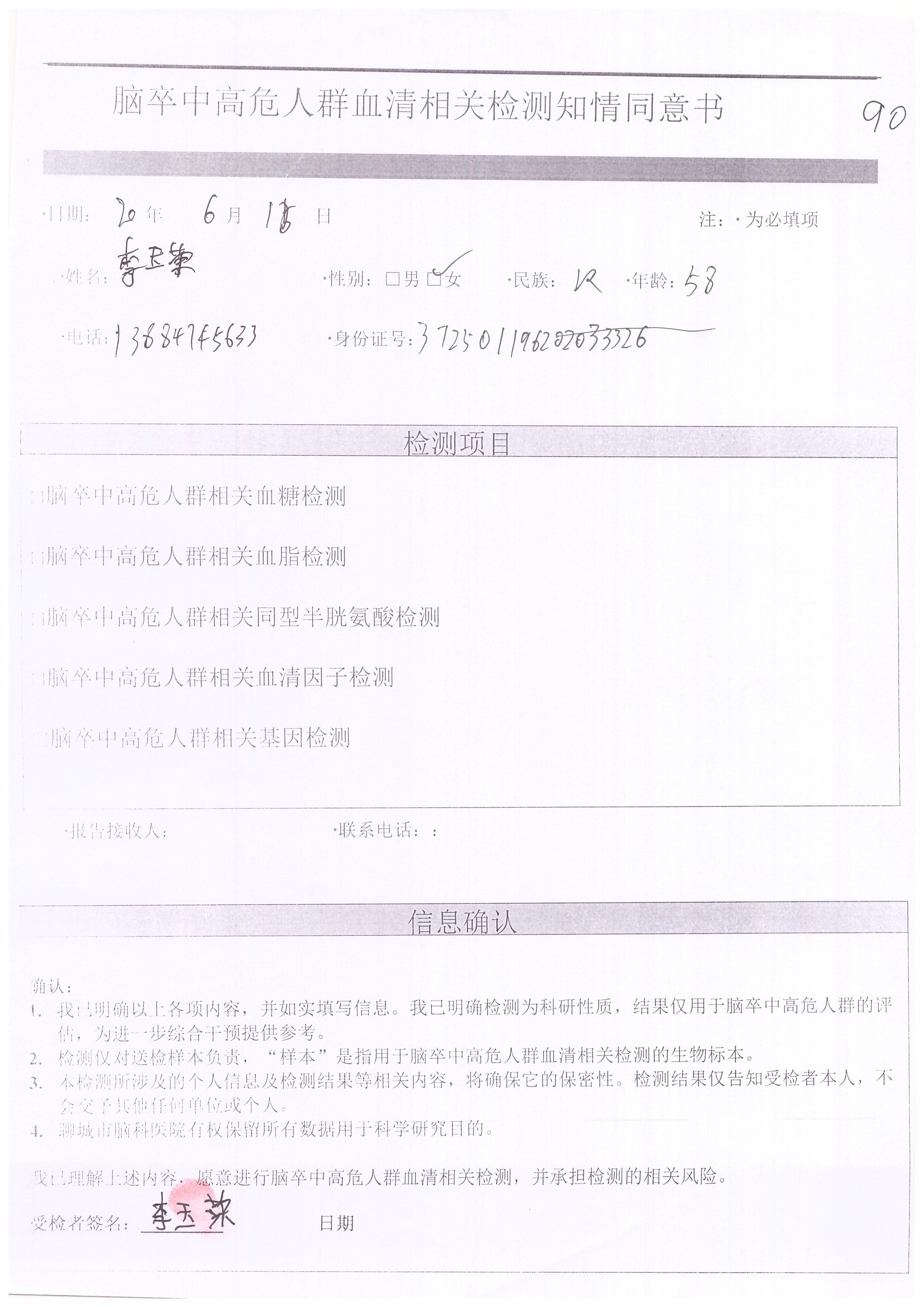

Supplement: Supplementary file 11 — Supplementary file11 (ZIP 25089 KB) [file 10528_2023_10431_MOESM11_ESM.zip › ╓¬╟Θ═1⁄4╥Γ╩Θ9/048.jpg]

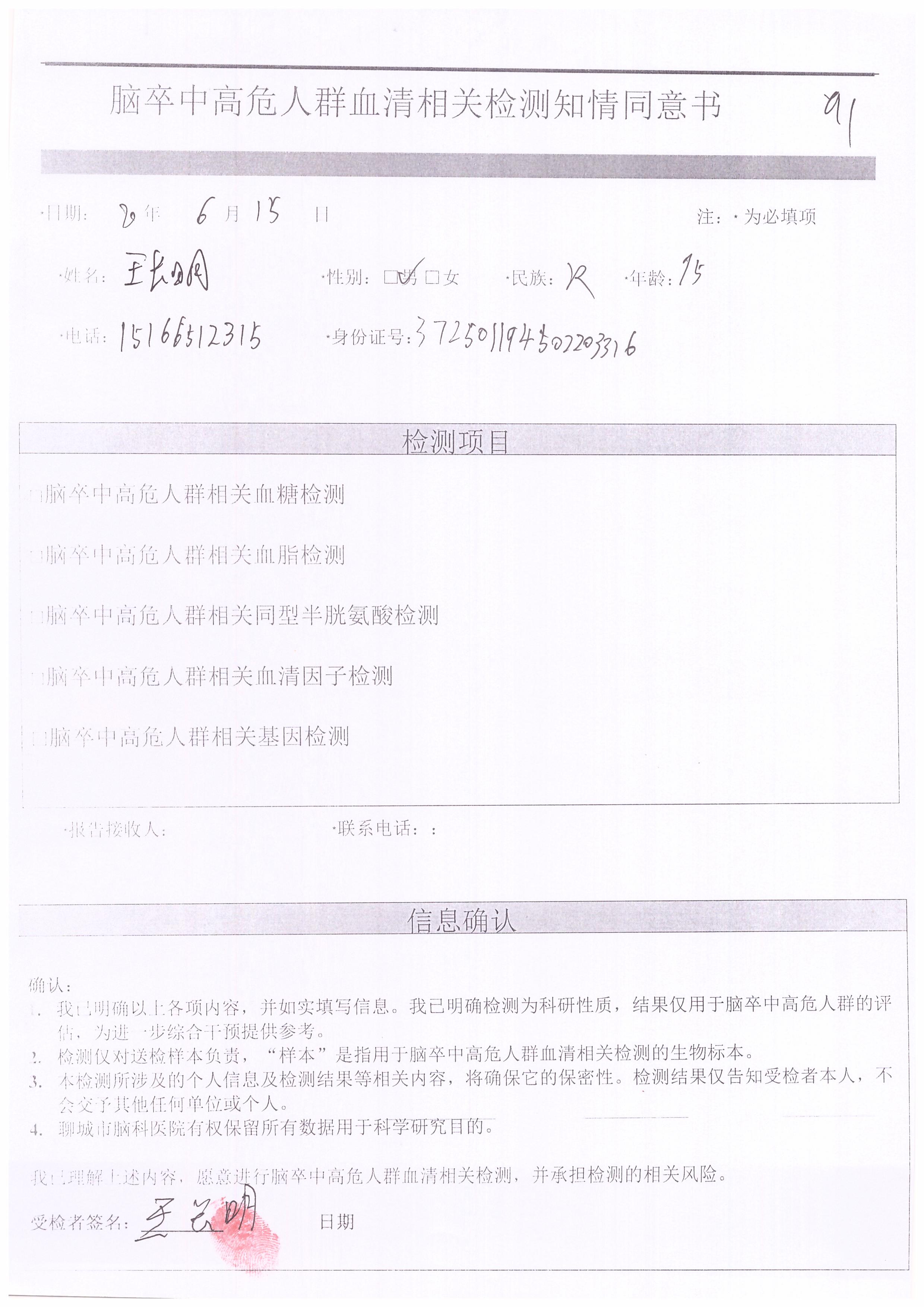

Supplement: Supplementary file 11 — Supplementary file11 (ZIP 25089 KB) [file 10528_2023_10431_MOESM11_ESM.zip › ╓¬╟Θ═1⁄4╥Γ╩Θ9/049.jpg]

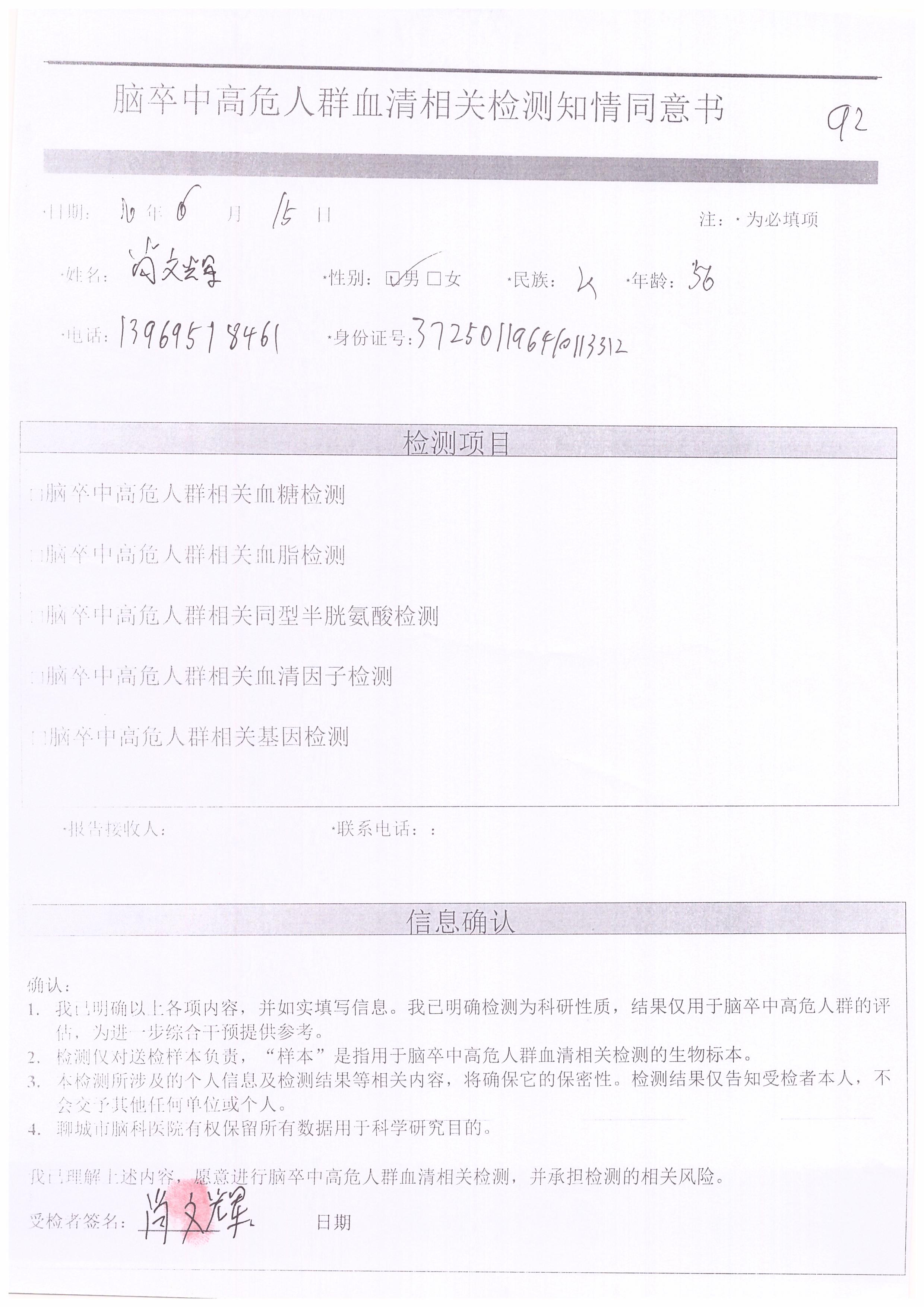

Supplement: Supplementary file 11 — Supplementary file11 (ZIP 25089 KB) [file 10528_2023_10431_MOESM11_ESM.zip › ╓¬╟Θ═1⁄4╥Γ╩Θ9/050.jpg]

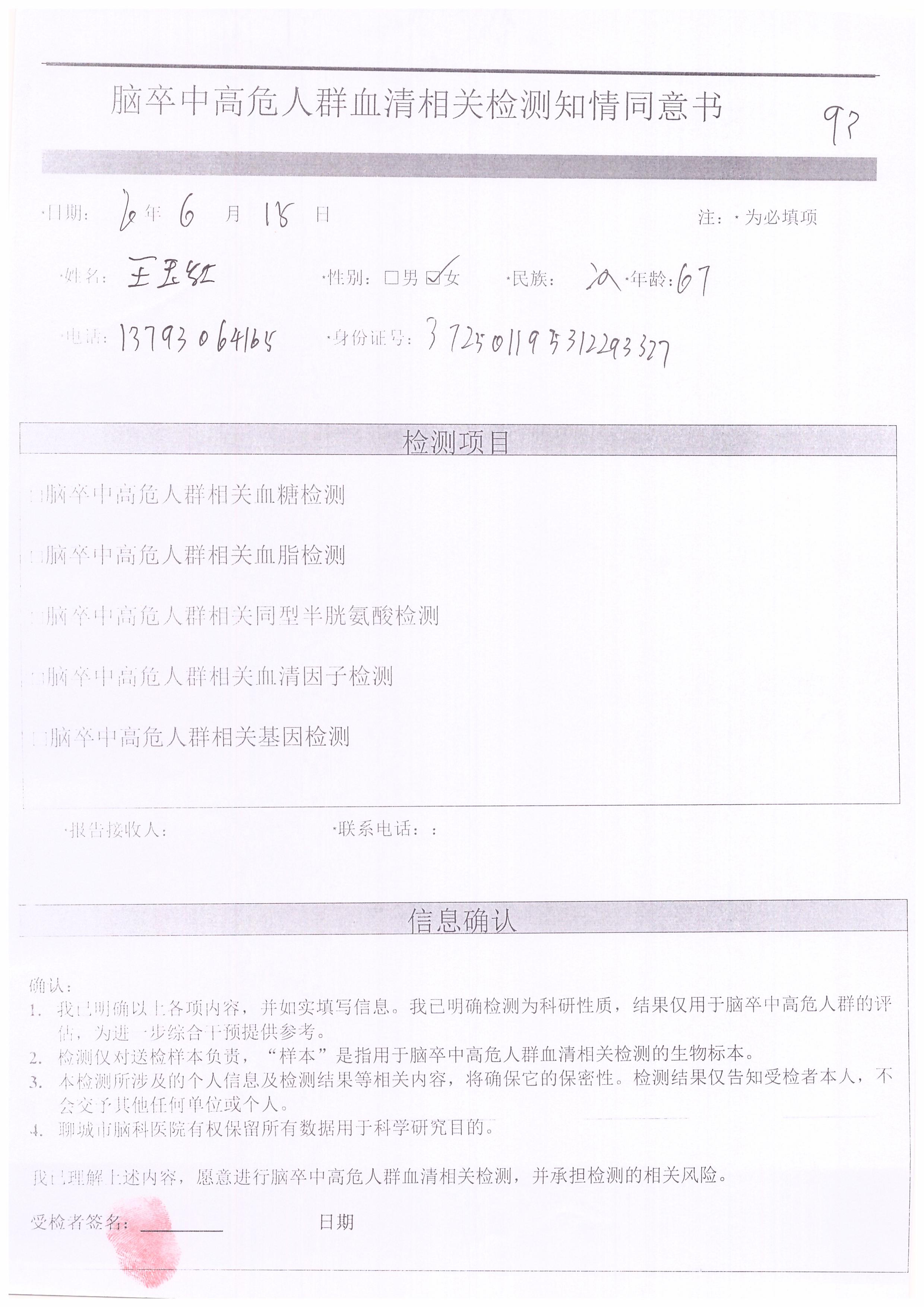

Supplement: Supplementary file 11 — Supplementary file11 (ZIP 25089 KB) [file 10528_2023_10431_MOESM11_ESM.zip › ╓¬╟Θ═1⁄4╥Γ╩Θ9/051.jpg]

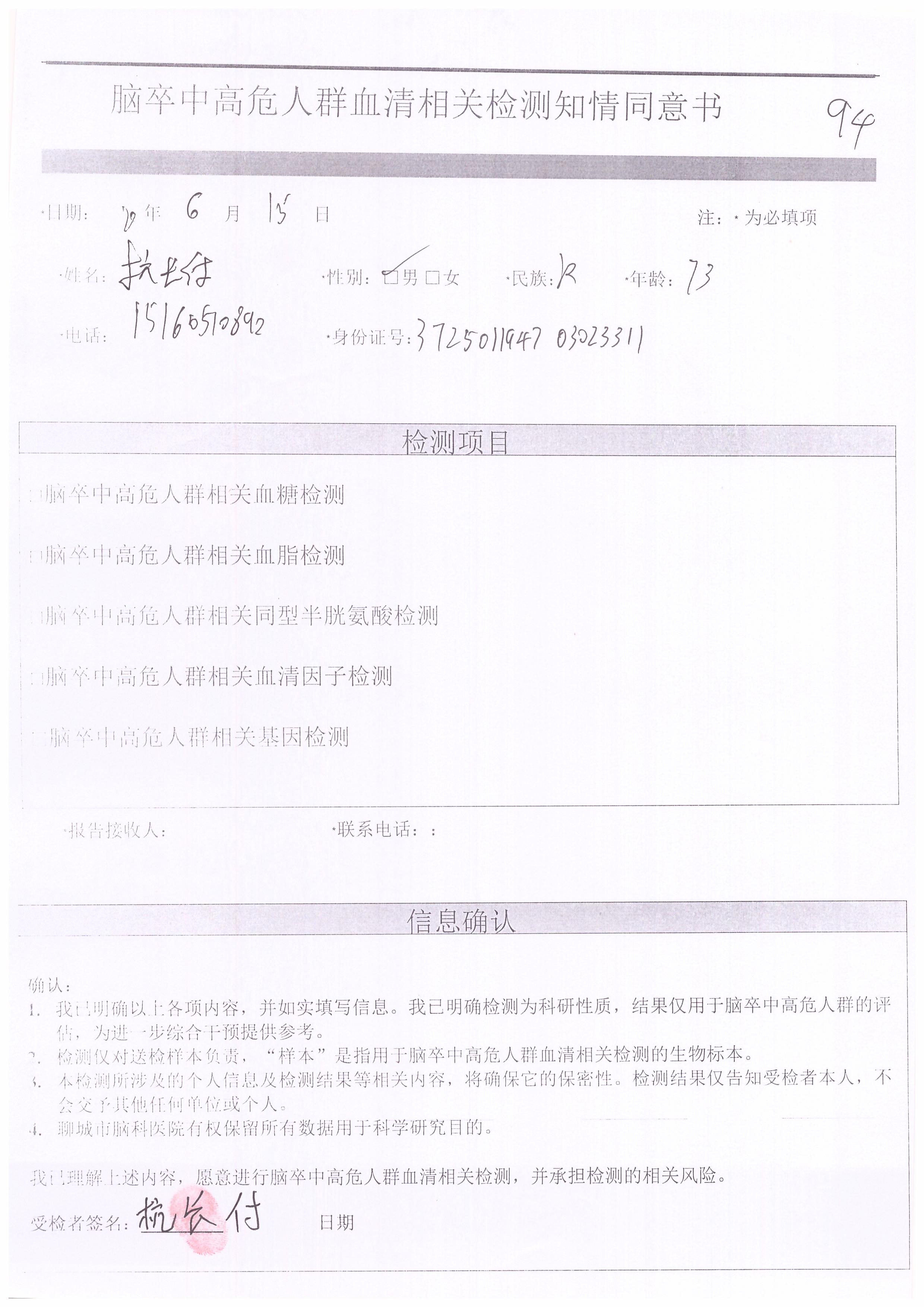

Supplement: Supplementary file 11 — Supplementary file11 (ZIP 25089 KB) [file 10528_2023_10431_MOESM11_ESM.zip › ╓¬╟Θ═1⁄4╥Γ╩Θ9/052.jpg]

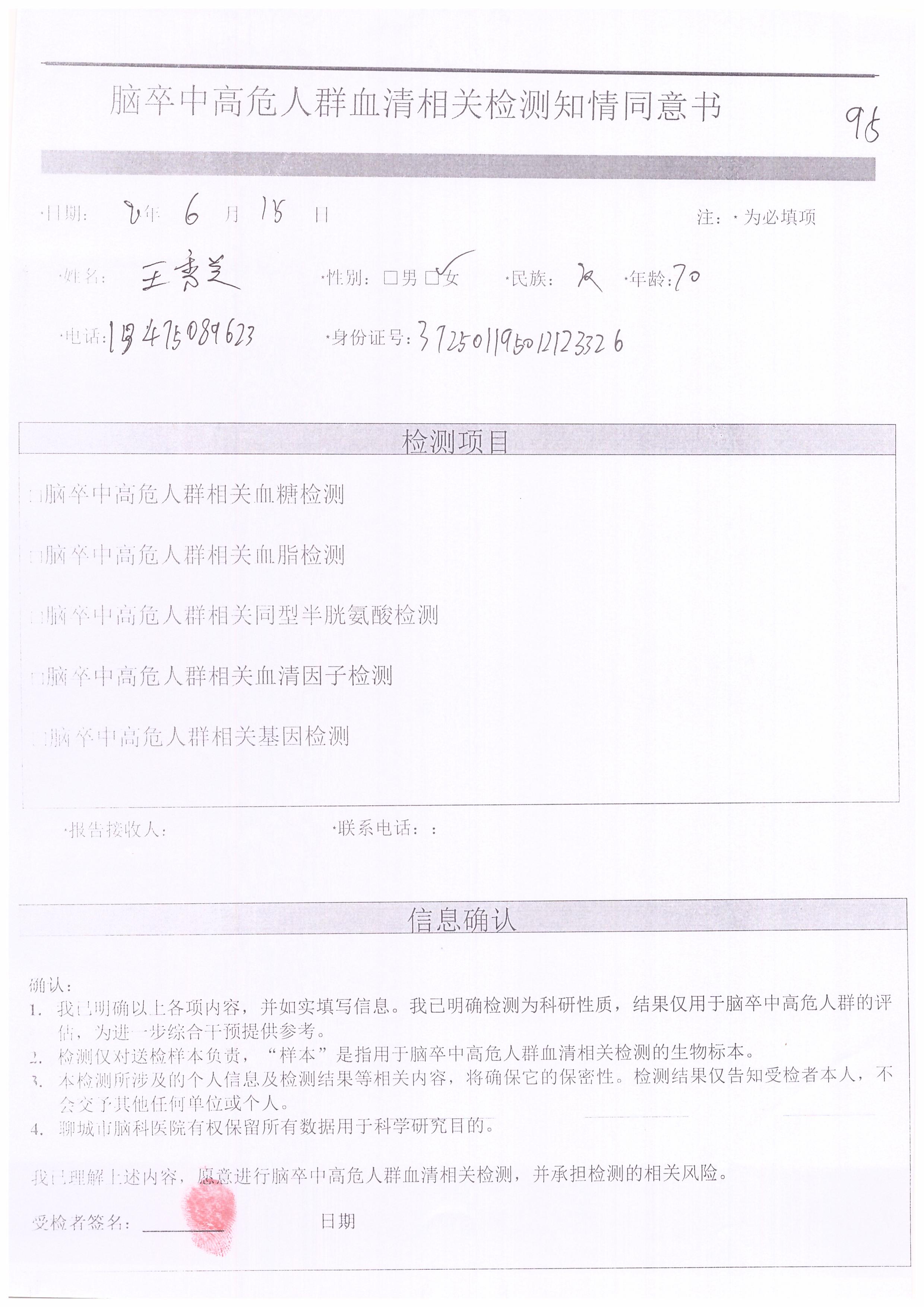

Supplement: Supplementary file 11 — Supplementary file11 (ZIP 25089 KB) [file 10528_2023_10431_MOESM11_ESM.zip › ╓¬╟Θ═1⁄4╥Γ╩Θ9/053.jpg]
