# Supplementary figures and images for: The Association Between Thymidylate Synthase Gene Polymorphisms and the Risk of Ischemic Stroke in Chinese Han Population (part 5 of 6)
Source: Biochem Genet. 2023 Jun 28;62(1):468–84. doi: 10.1007/s10528-023-10431-8 (PMC10901929; doi:10.1007/s10528-023-10431-8)

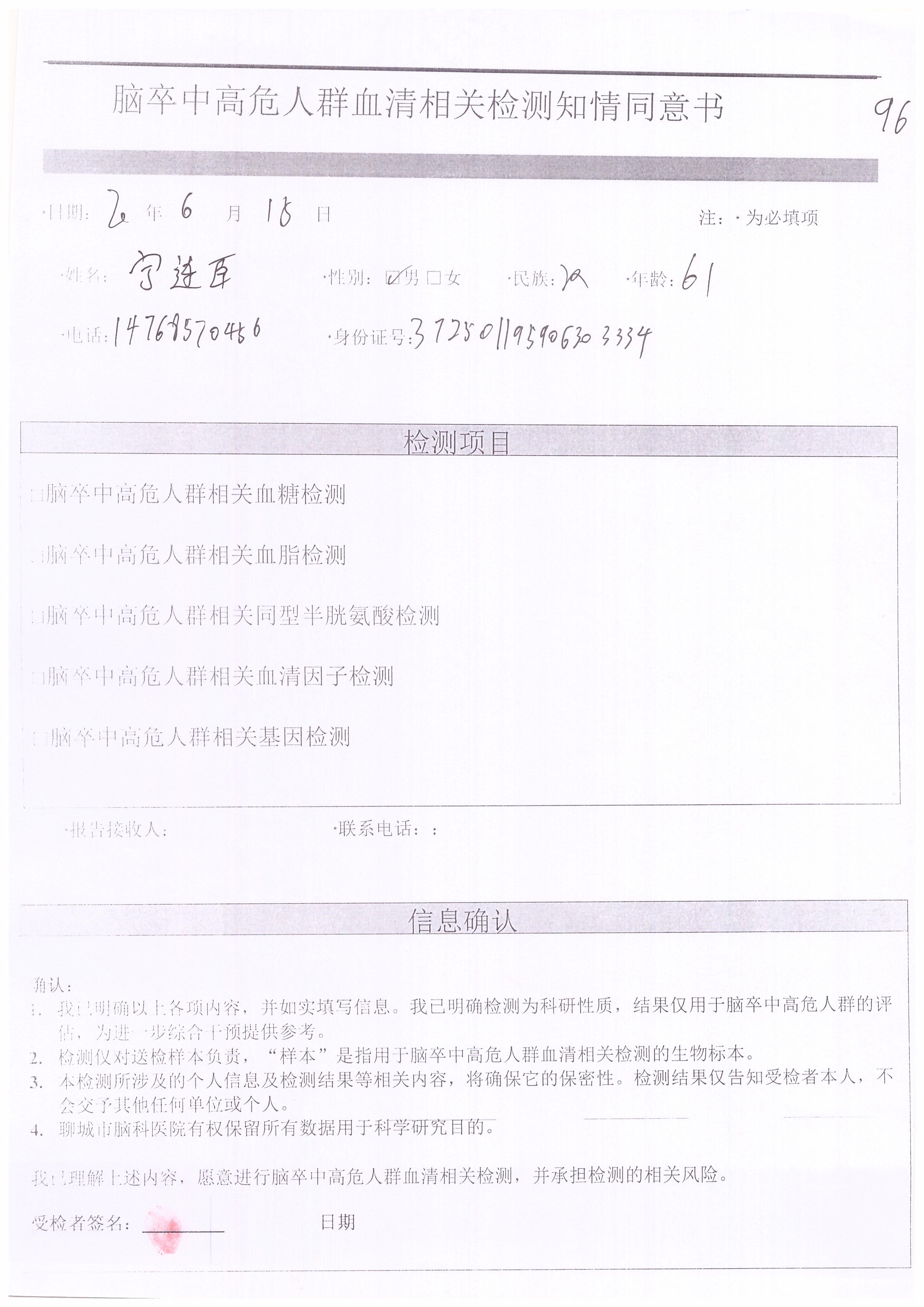

Supplement: Supplementary file 11 — Supplementary file11 (ZIP 25089 KB) [file 10528_2023_10431_MOESM11_ESM.zip › ╓¬╟Θ═1⁄4╥Γ╩Θ9/054.jpg]

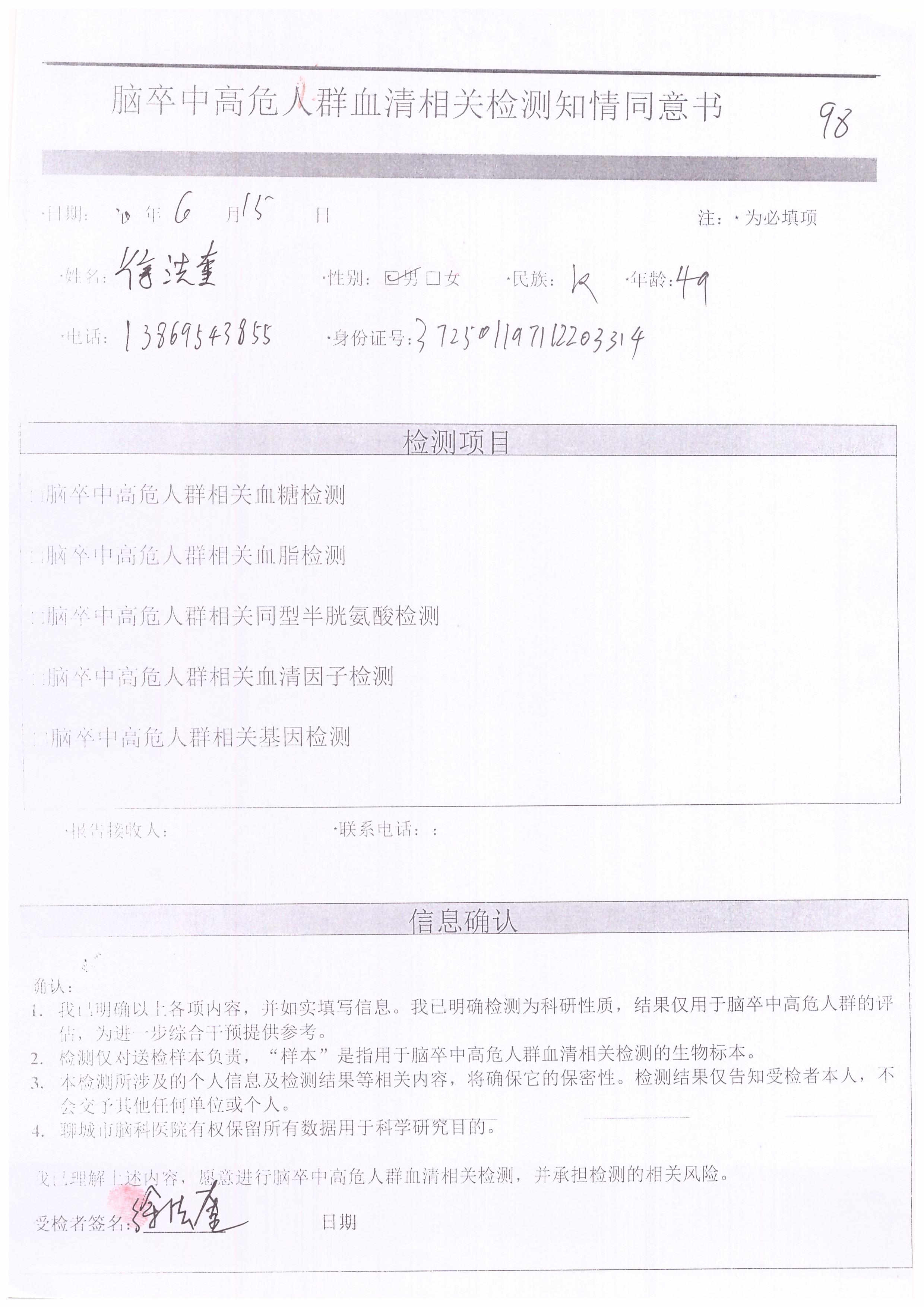

Supplement: Supplementary file 11 — Supplementary file11 (ZIP 25089 KB) [file 10528_2023_10431_MOESM11_ESM.zip › ╓¬╟Θ═1⁄4╥Γ╩Θ9/055.jpg]

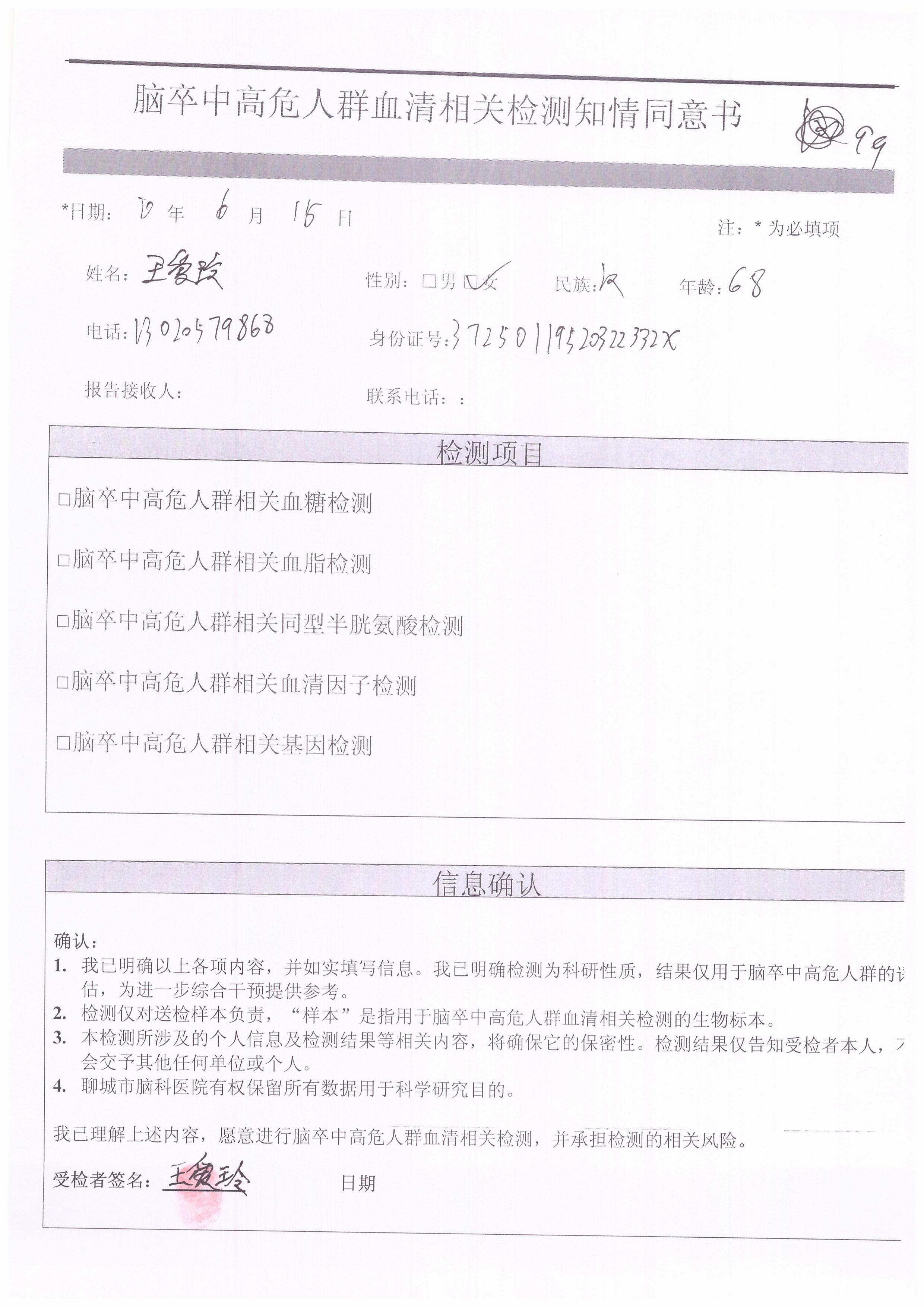

Supplement: Supplementary file 11 — Supplementary file11 (ZIP 25089 KB) [file 10528_2023_10431_MOESM11_ESM.zip › ╓¬╟Θ═1⁄4╥Γ╩Θ9/056.jpg]

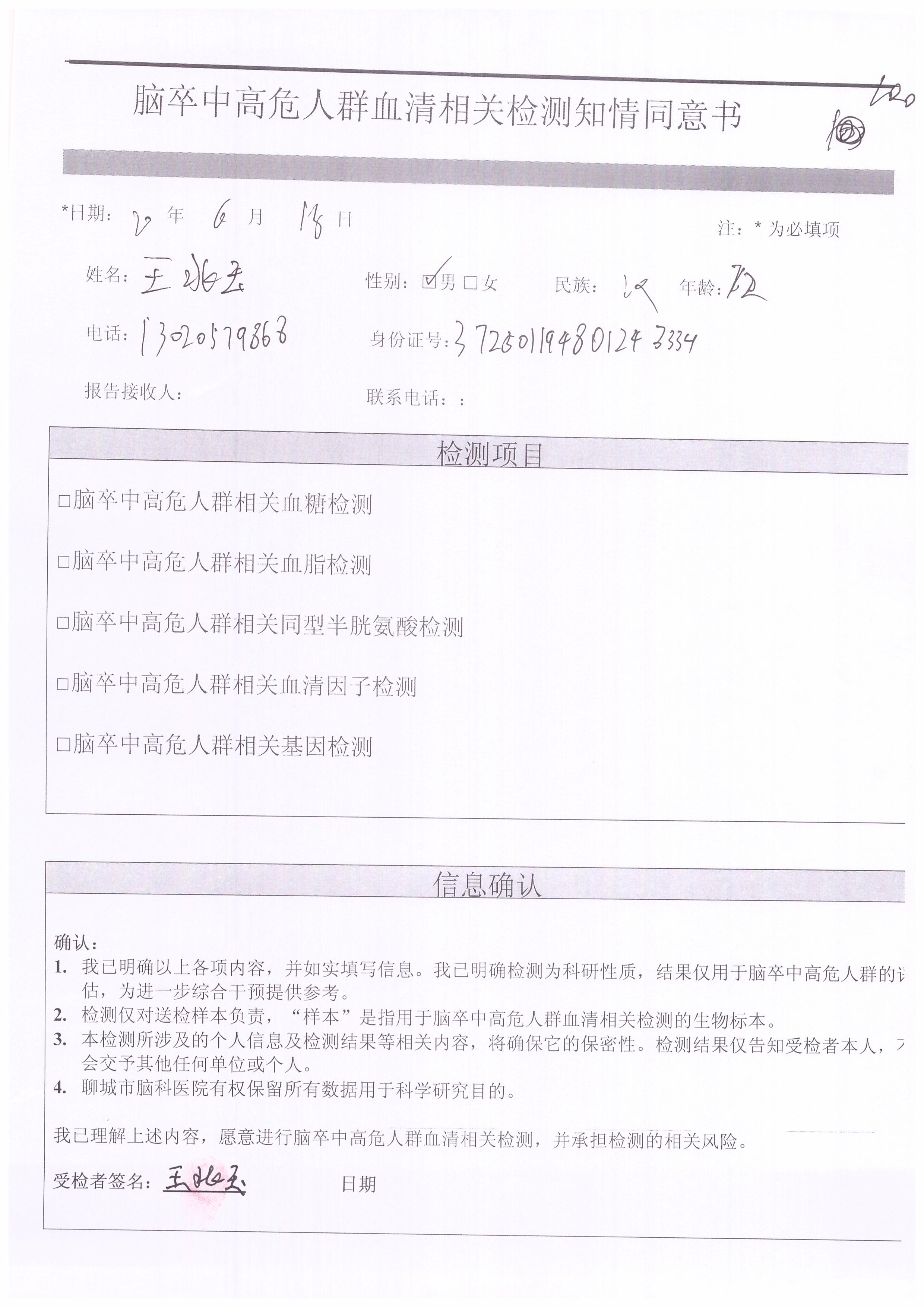

Supplement: Supplementary file 11 — Supplementary file11 (ZIP 25089 KB) [file 10528_2023_10431_MOESM11_ESM.zip › ╓¬╟Θ═1⁄4╥Γ╩Θ9/057.jpg]

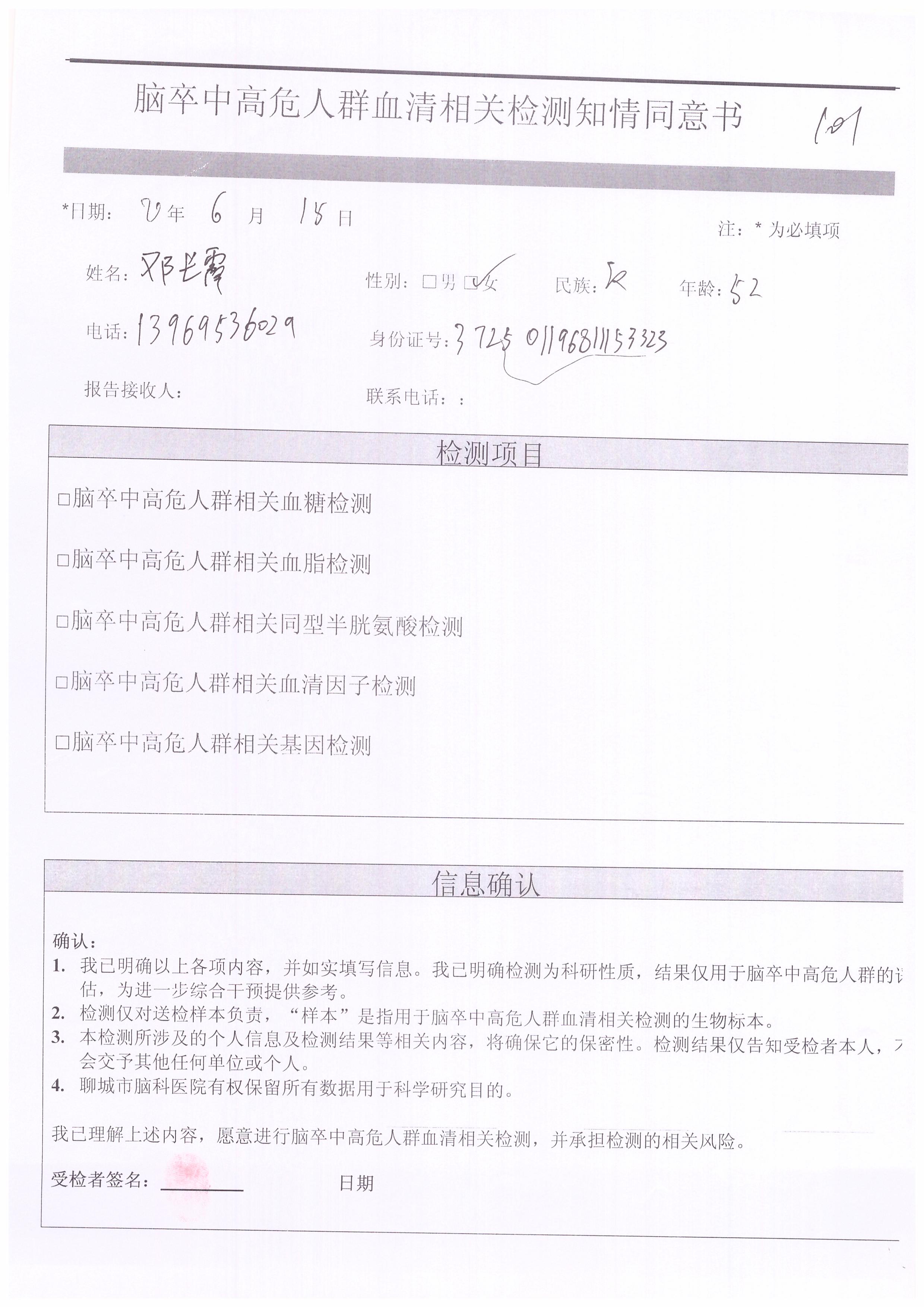

Supplement: Supplementary file 11 — Supplementary file11 (ZIP 25089 KB) [file 10528_2023_10431_MOESM11_ESM.zip › ╓¬╟Θ═1⁄4╥Γ╩Θ9/058.jpg]

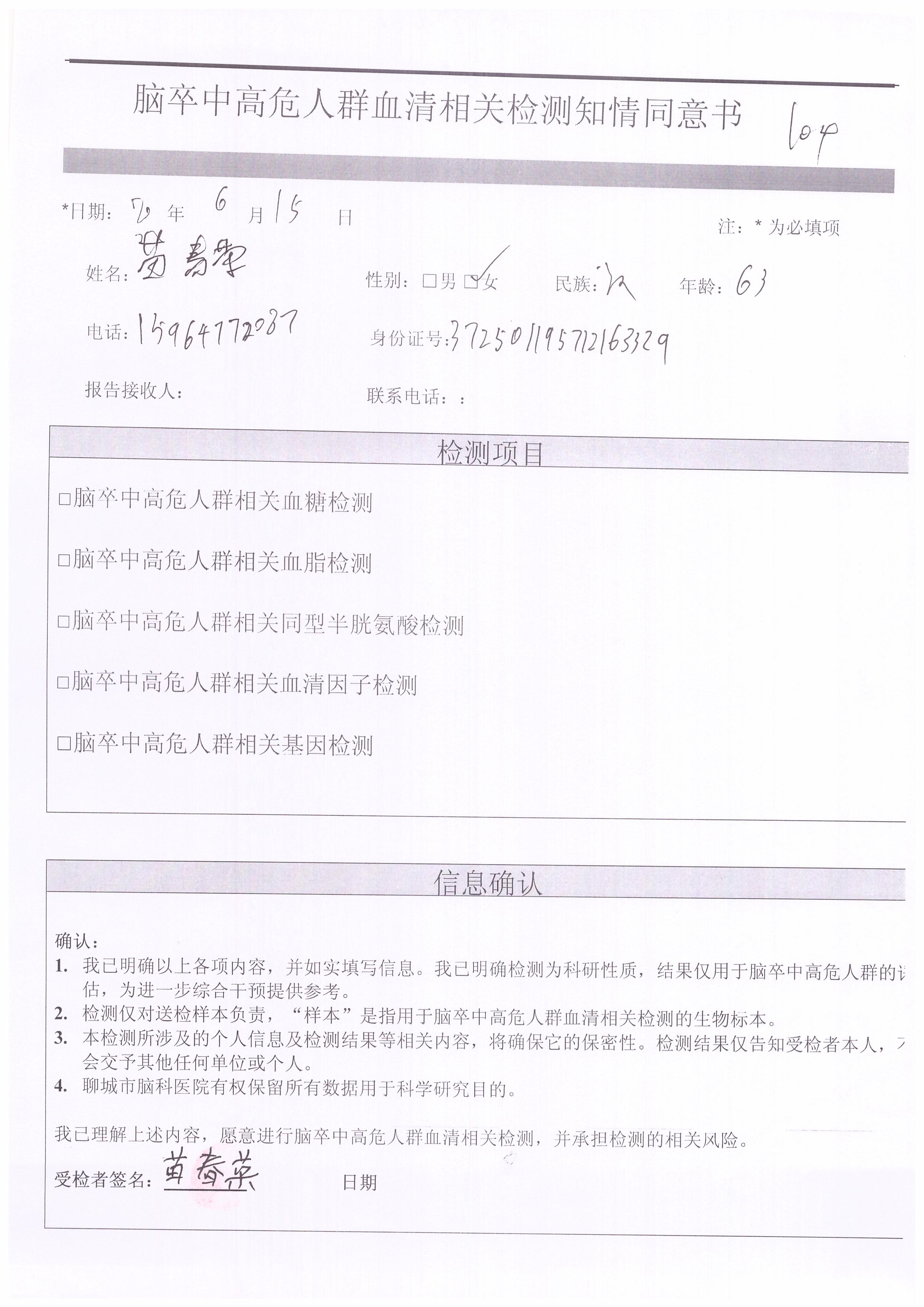

Supplement: Supplementary file 11 — Supplementary file11 (ZIP 25089 KB) [file 10528_2023_10431_MOESM11_ESM.zip › ╓¬╟Θ═1⁄4╥Γ╩Θ9/059.jpg]

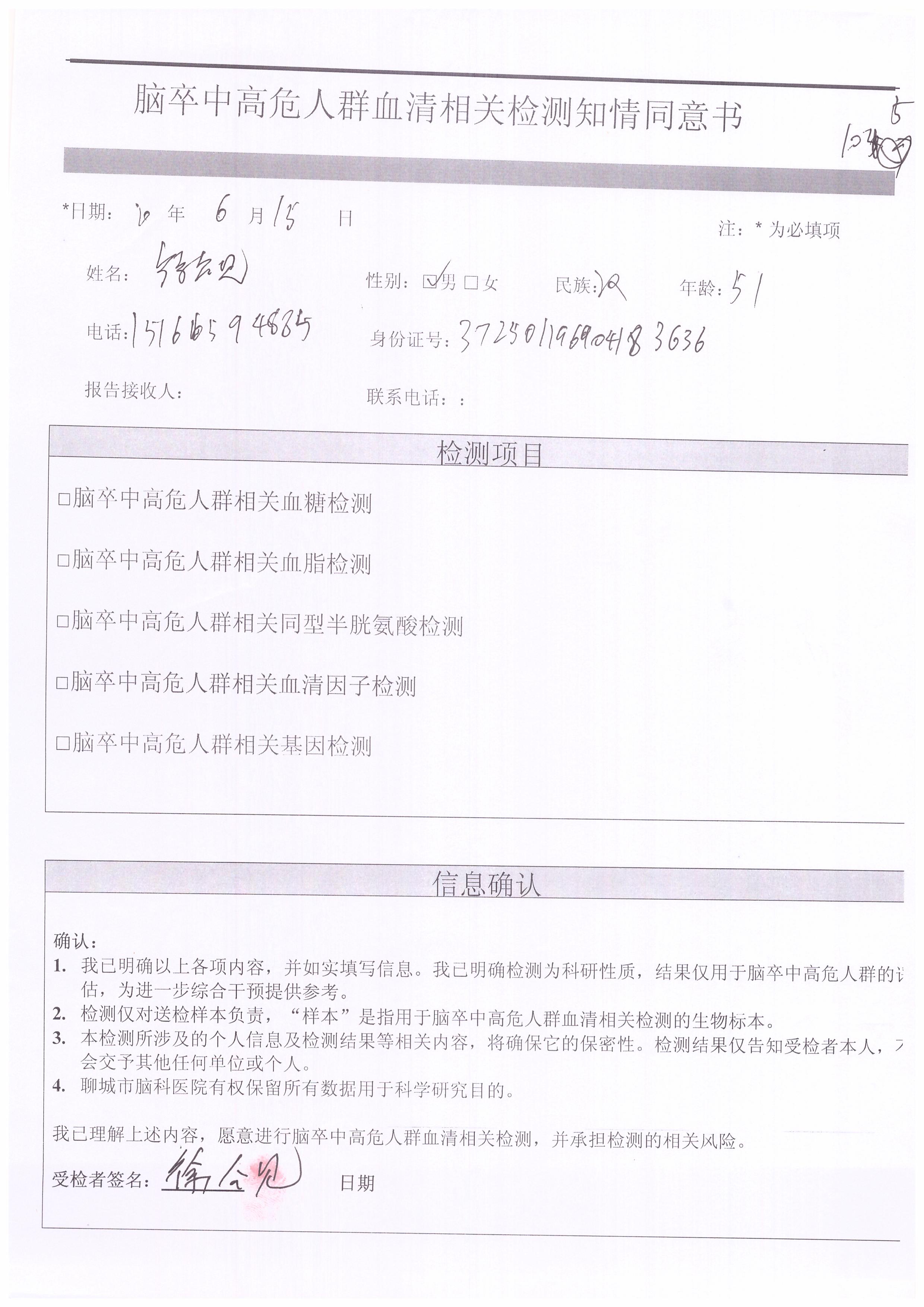

Supplement: Supplementary file 11 — Supplementary file11 (ZIP 25089 KB) [file 10528_2023_10431_MOESM11_ESM.zip › ╓¬╟Θ═1⁄4╥Γ╩Θ9/060.jpg]

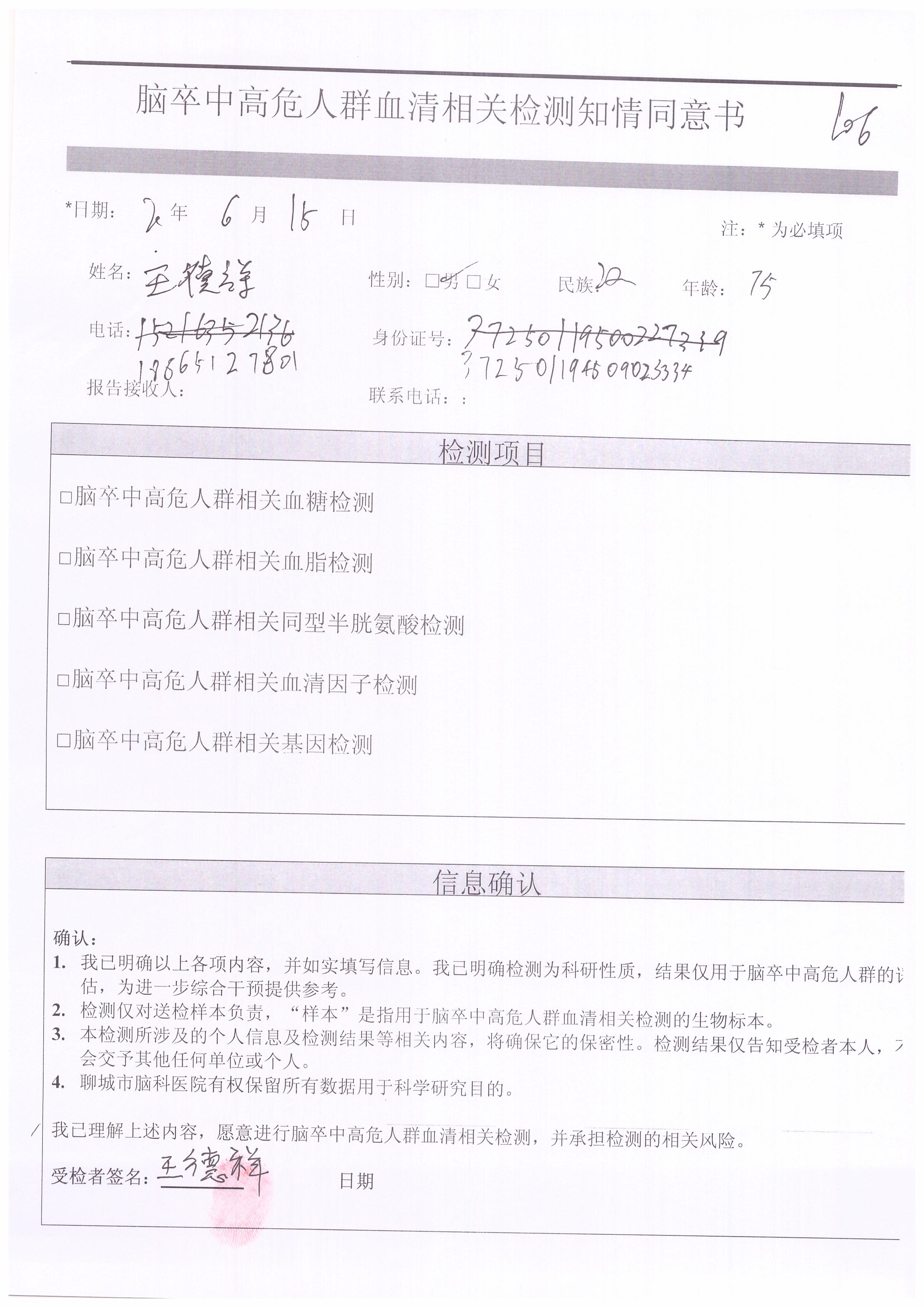

Supplement: Supplementary file 11 — Supplementary file11 (ZIP 25089 KB) [file 10528_2023_10431_MOESM11_ESM.zip › ╓¬╟Θ═1⁄4╥Γ╩Θ9/061.jpg]

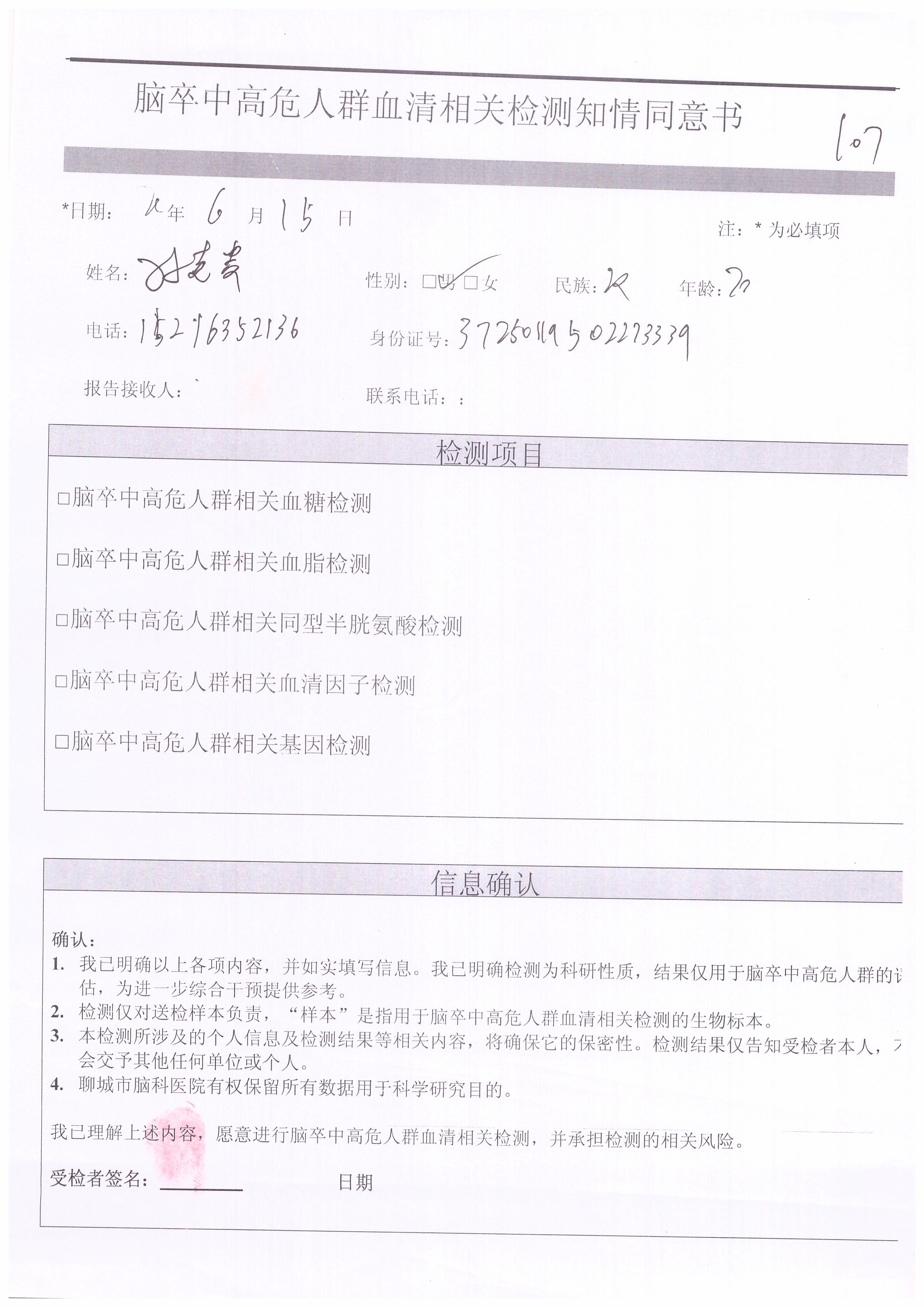

Supplement: Supplementary file 11 — Supplementary file11 (ZIP 25089 KB) [file 10528_2023_10431_MOESM11_ESM.zip › ╓¬╟Θ═1⁄4╥Γ╩Θ9/062.jpg]

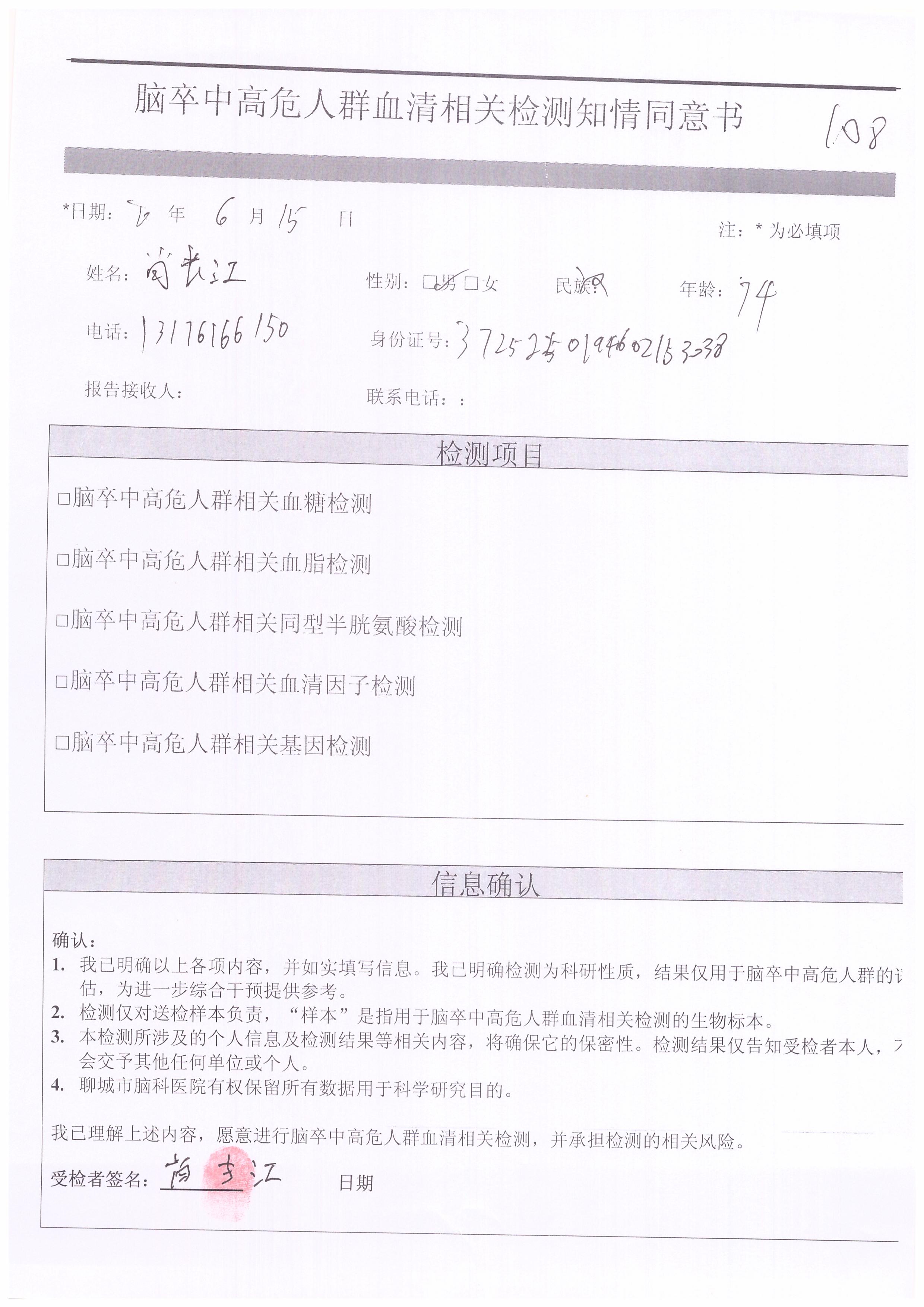

Supplement: Supplementary file 11 — Supplementary file11 (ZIP 25089 KB) [file 10528_2023_10431_MOESM11_ESM.zip › ╓¬╟Θ═1⁄4╥Γ╩Θ9/063.jpg]

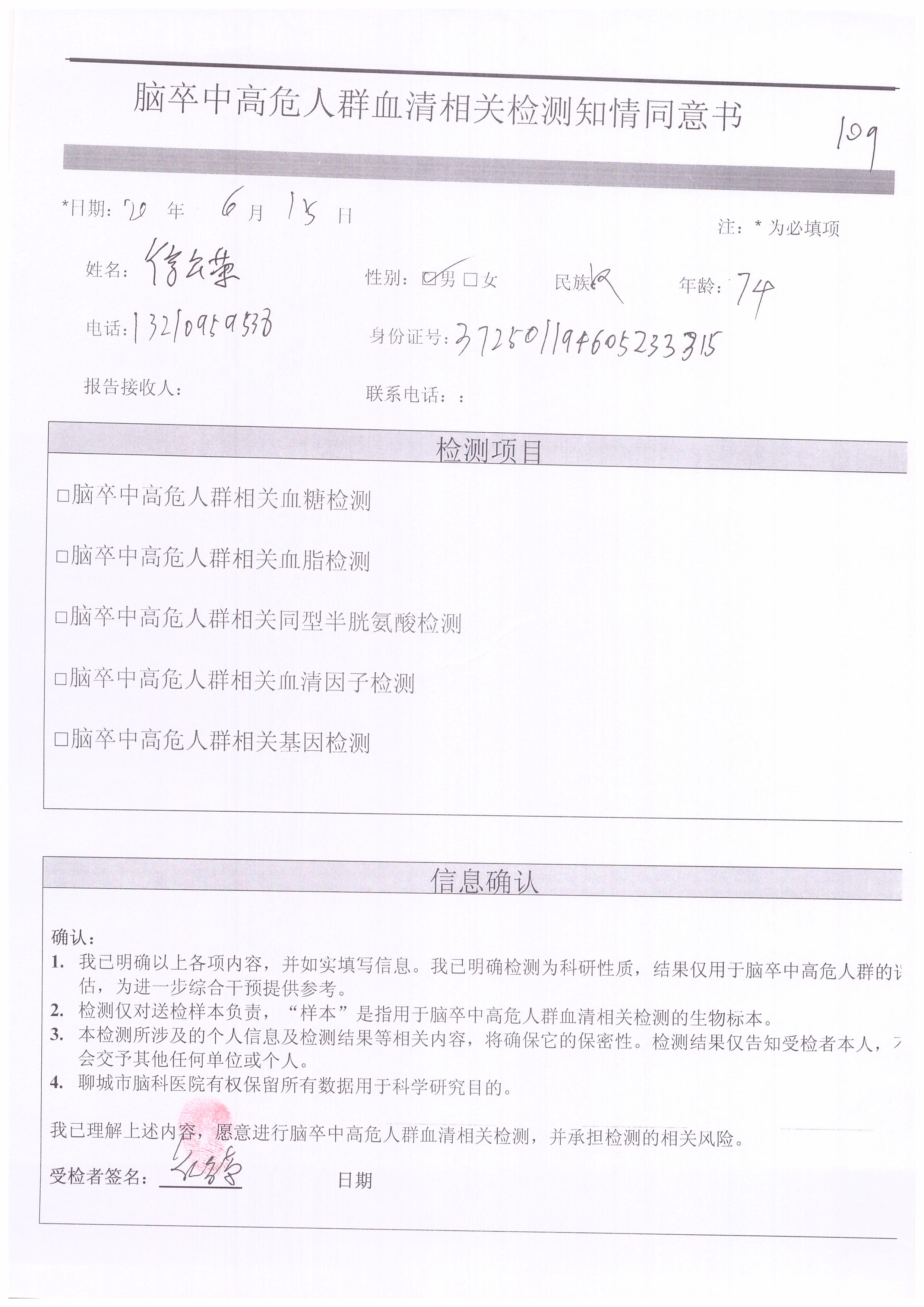

Supplement: Supplementary file 11 — Supplementary file11 (ZIP 25089 KB) [file 10528_2023_10431_MOESM11_ESM.zip › ╓¬╟Θ═1⁄4╥Γ╩Θ9/064.jpg]

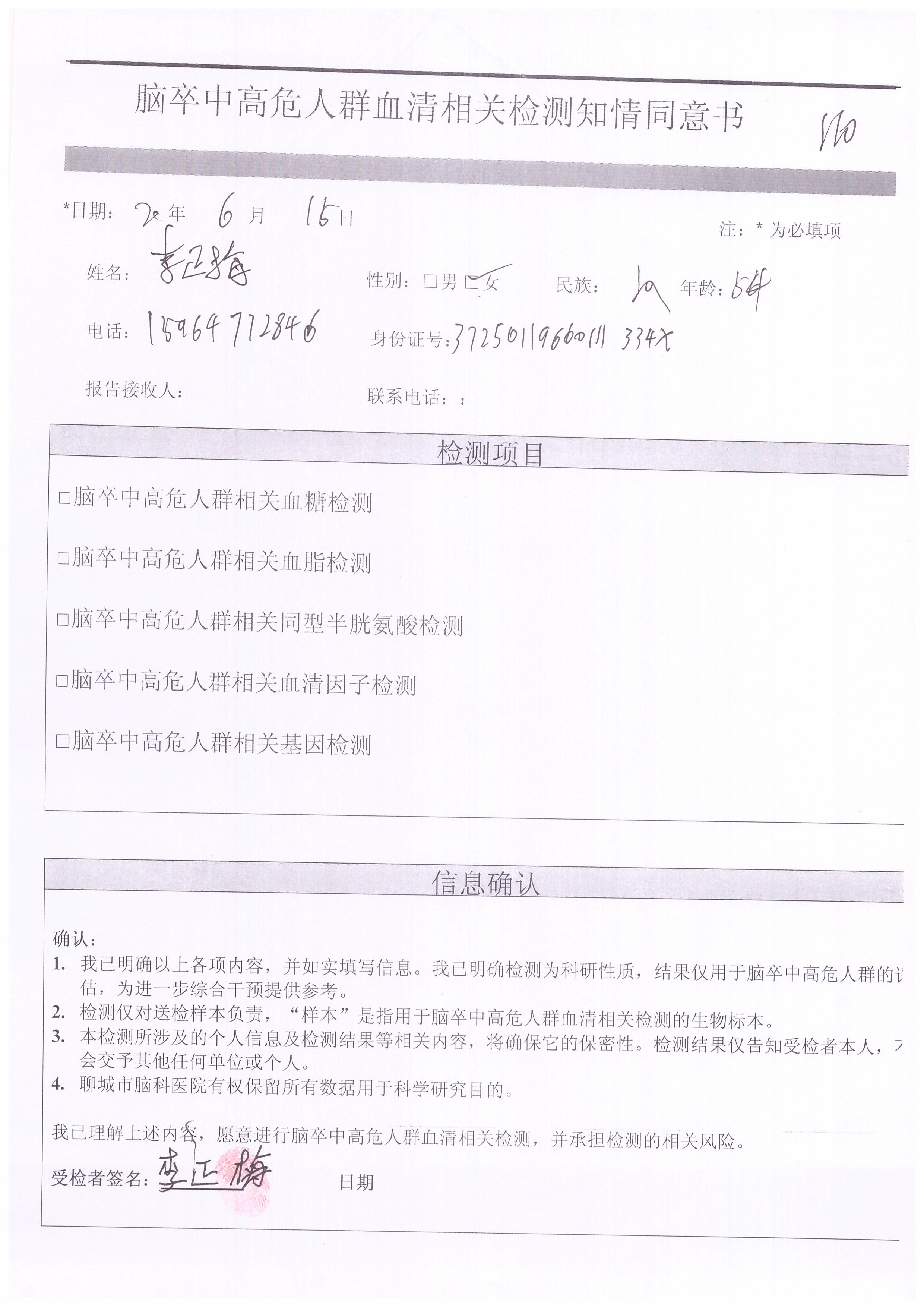

Supplement: Supplementary file 11 — Supplementary file11 (ZIP 25089 KB) [file 10528_2023_10431_MOESM11_ESM.zip › ╓¬╟Θ═1⁄4╥Γ╩Θ9/065.jpg]

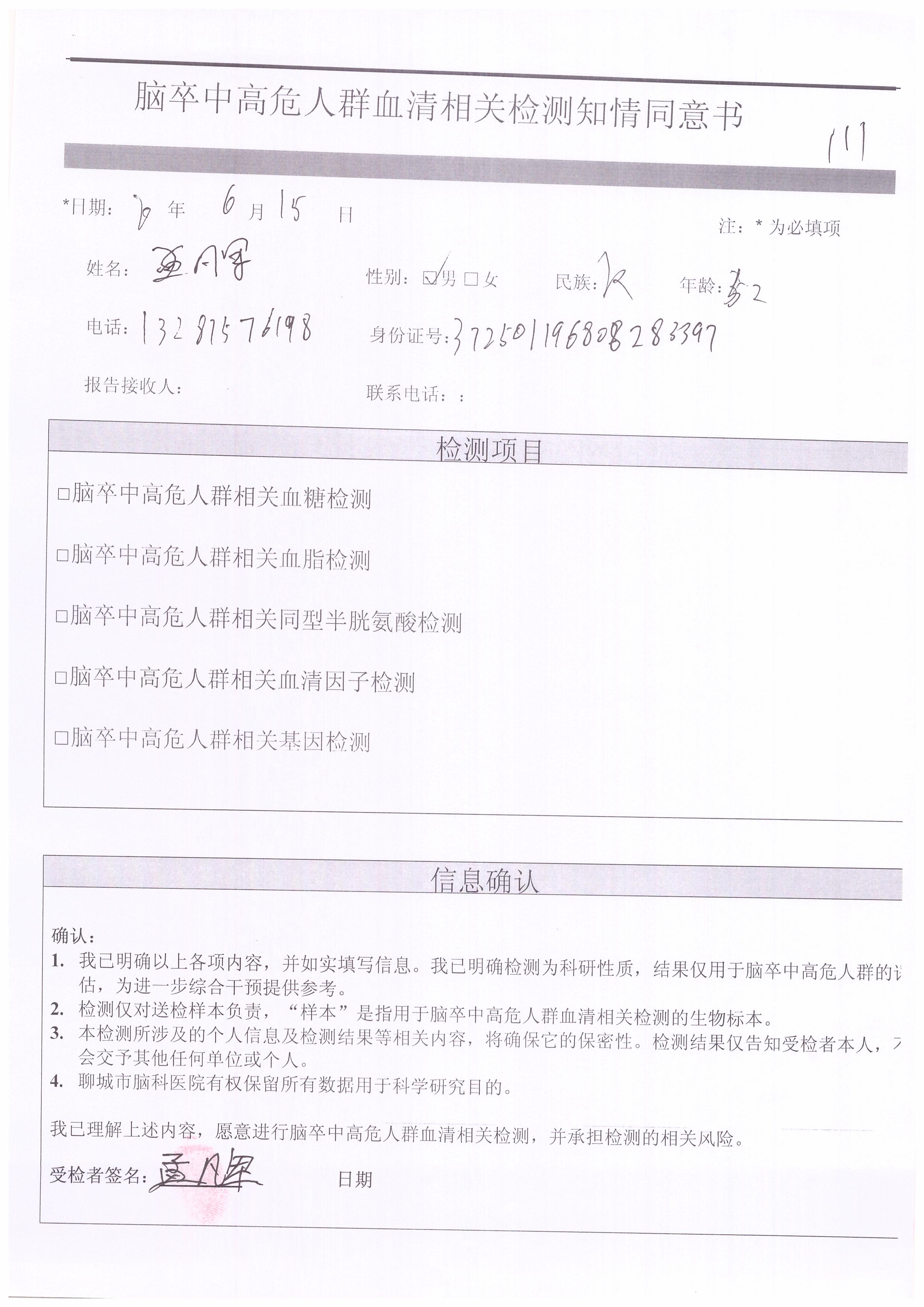

Supplement: Supplementary file 11 — Supplementary file11 (ZIP 25089 KB) [file 10528_2023_10431_MOESM11_ESM.zip › ╓¬╟Θ═1⁄4╥Γ╩Θ9/066.jpg]

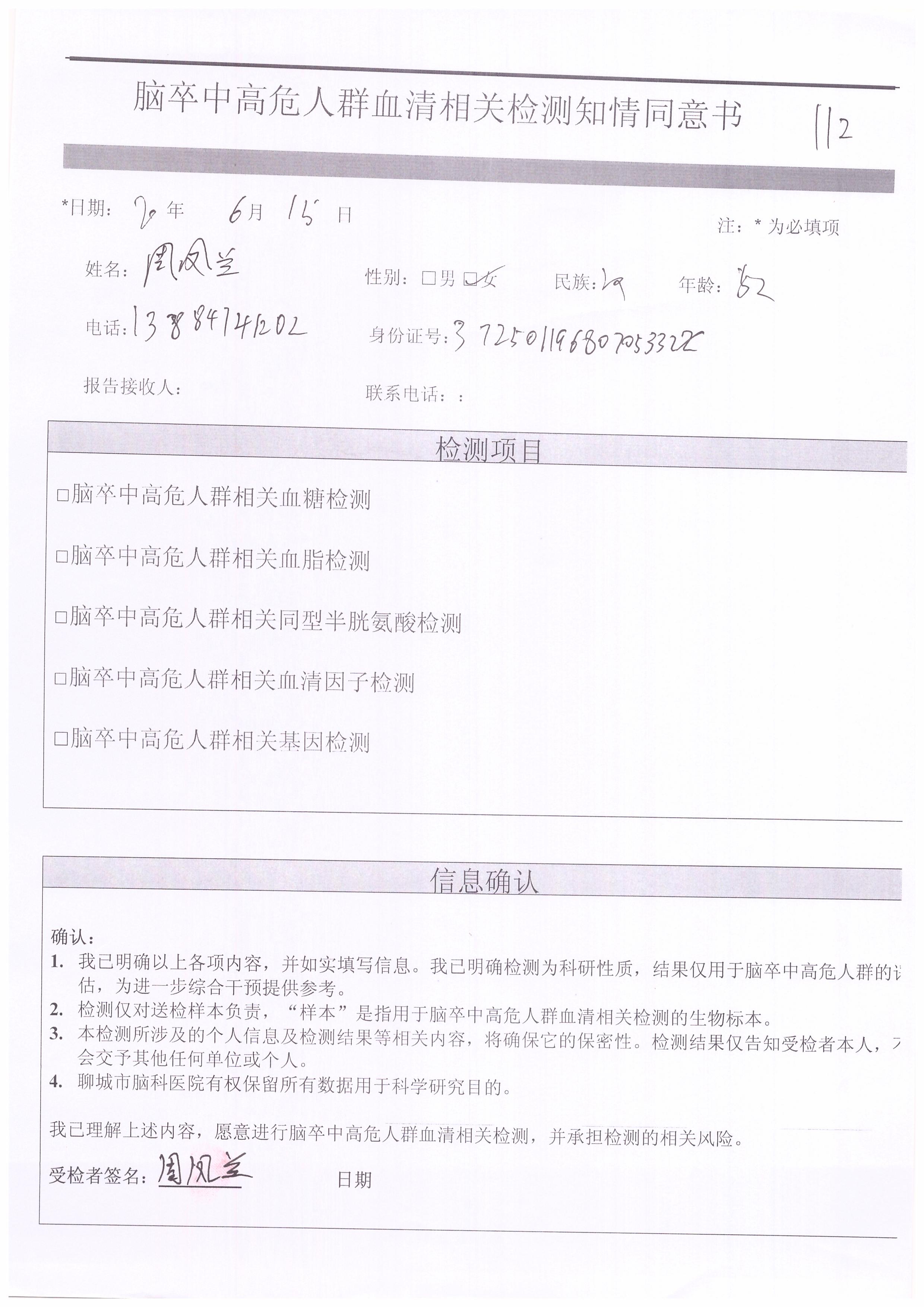

Supplement: Supplementary file 11 — Supplementary file11 (ZIP 25089 KB) [file 10528_2023_10431_MOESM11_ESM.zip › ╓¬╟Θ═1⁄4╥Γ╩Θ9/067.jpg]

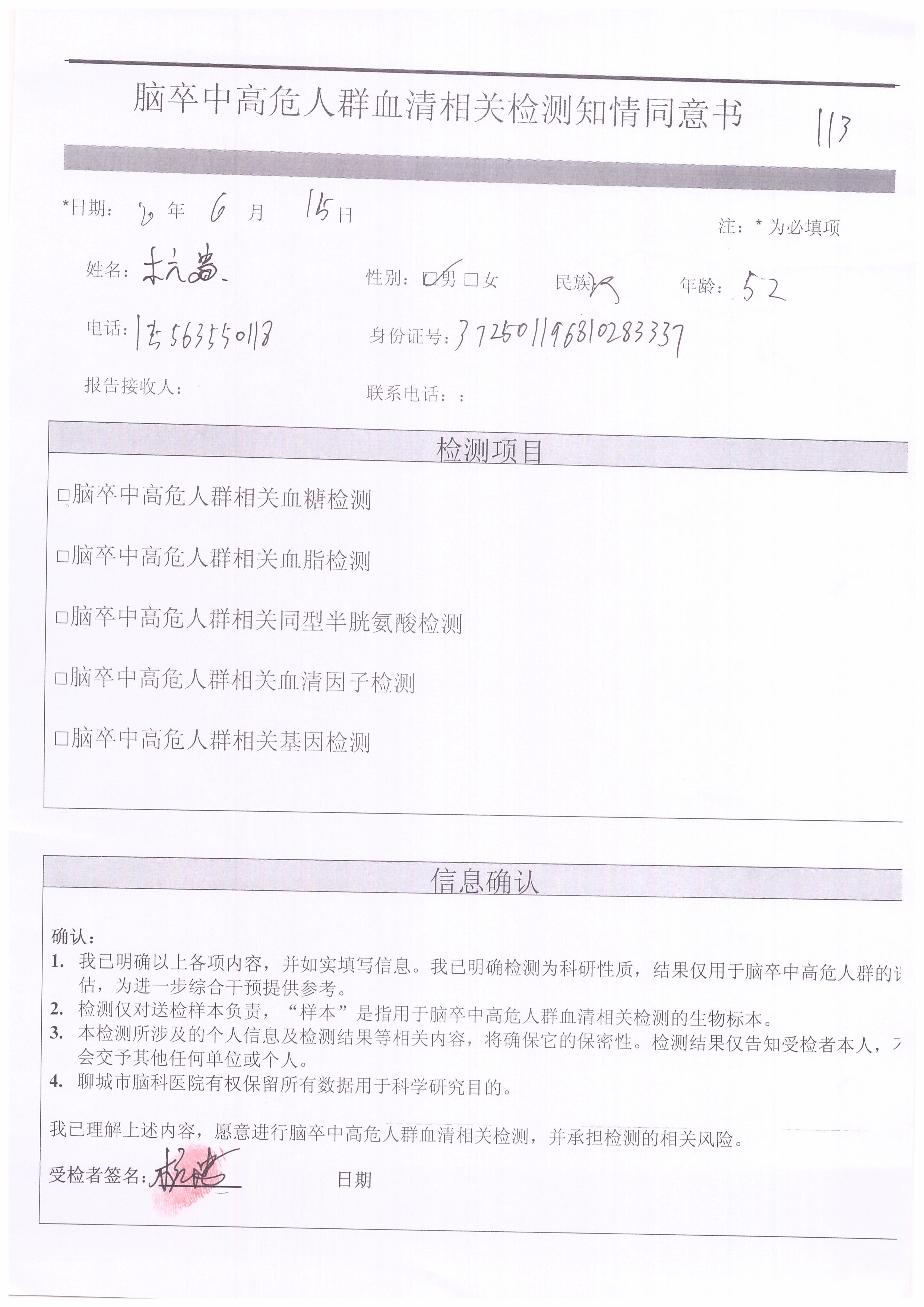

Supplement: Supplementary file 11 — Supplementary file11 (ZIP 25089 KB) [file 10528_2023_10431_MOESM11_ESM.zip › ╓¬╟Θ═1⁄4╥Γ╩Θ9/068.jpg]

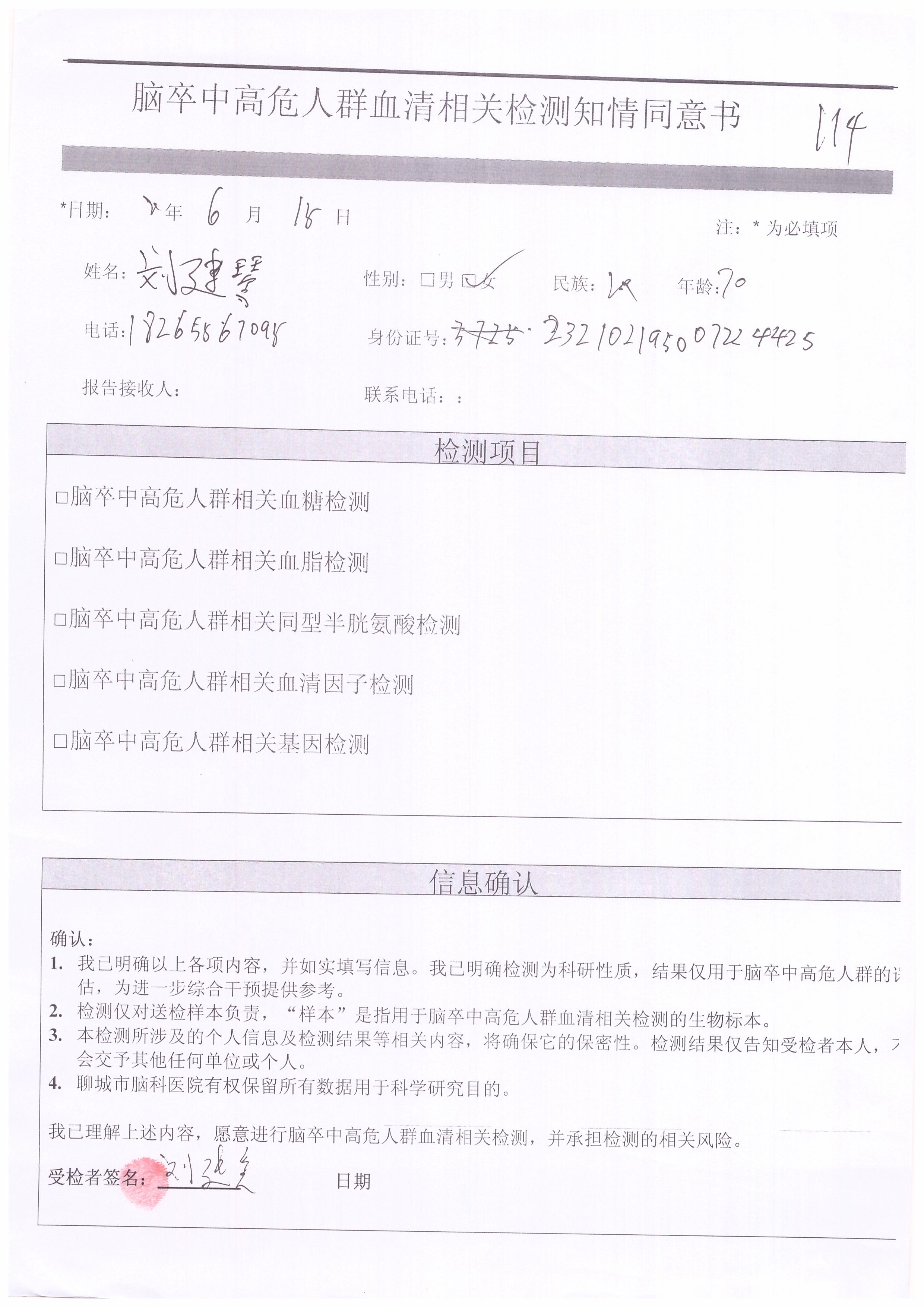

Supplement: Supplementary file 11 — Supplementary file11 (ZIP 25089 KB) [file 10528_2023_10431_MOESM11_ESM.zip › ╓¬╟Θ═1⁄4╥Γ╩Θ9/069.jpg]

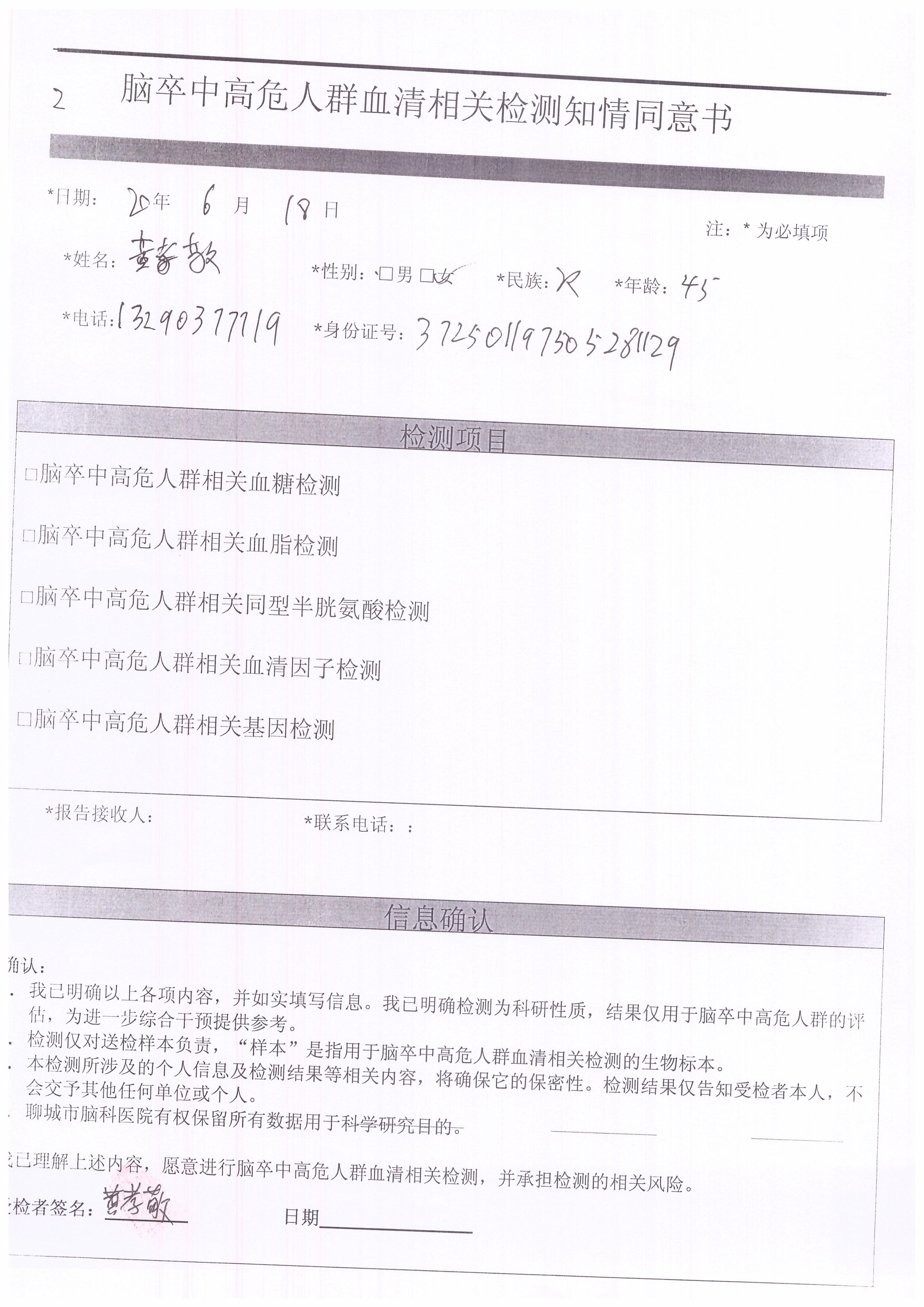

Supplement: Supplementary file 12 — Supplementary file12 (ZIP 2998 KB) [file 10528_2023_10431_MOESM12_ESM.zip › ╓¬╟Θ═1⁄4╥Γ╩Θ10/002.jpg]

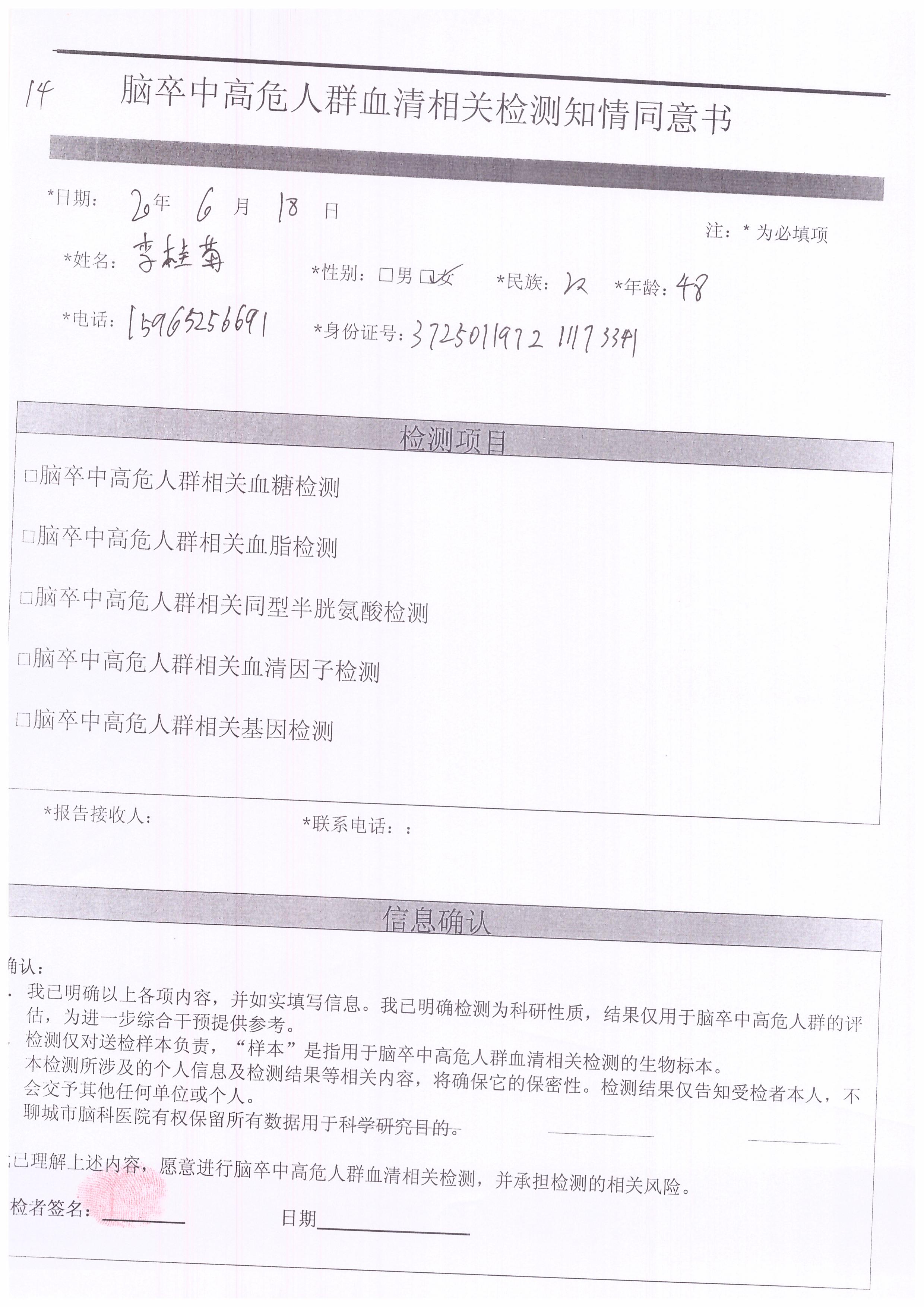

Supplement: Supplementary file 12 — Supplementary file12 (ZIP 2998 KB) [file 10528_2023_10431_MOESM12_ESM.zip › ╓¬╟Θ═1⁄4╥Γ╩Θ10/014.jpg]

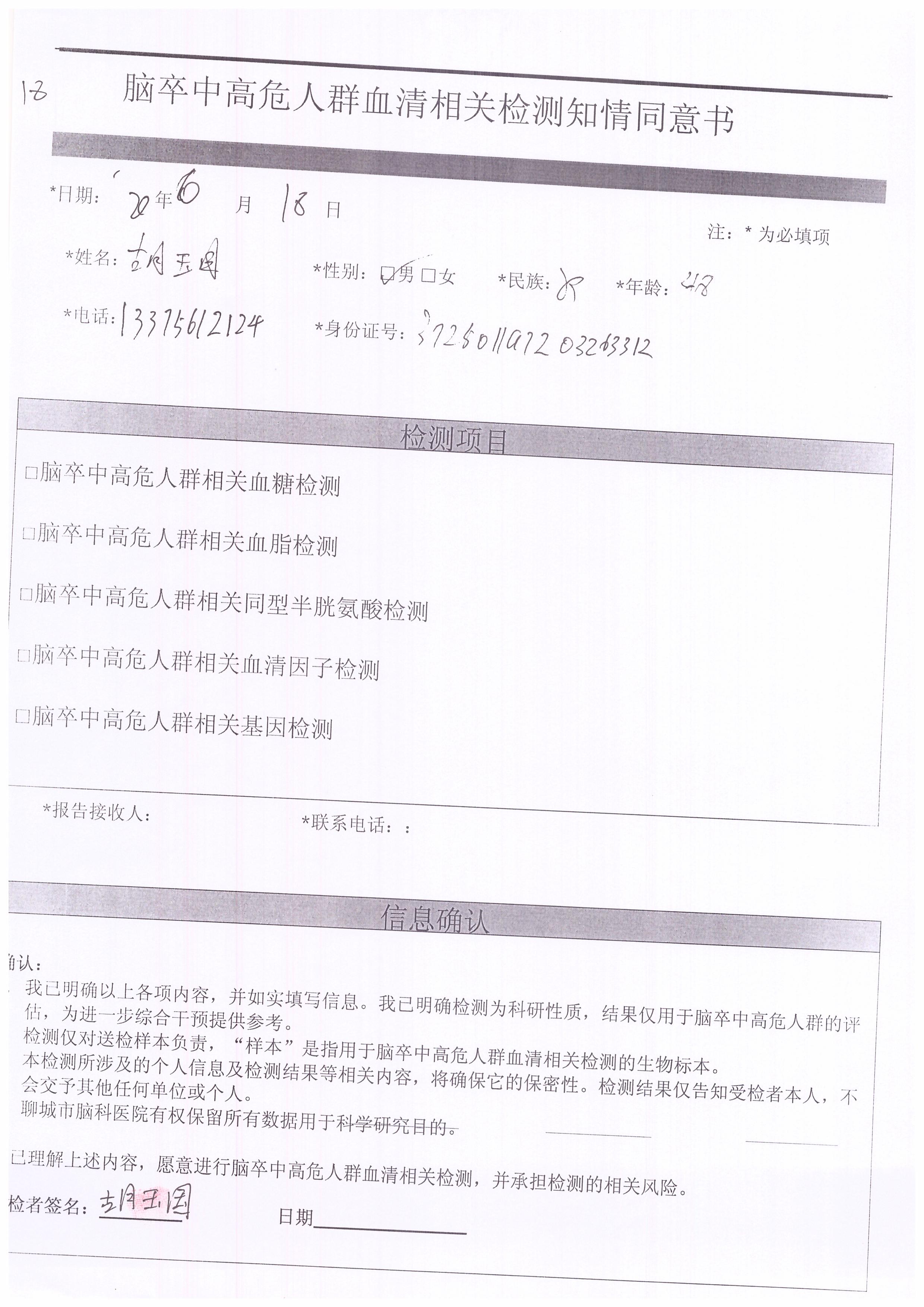

Supplement: Supplementary file 12 — Supplementary file12 (ZIP 2998 KB) [file 10528_2023_10431_MOESM12_ESM.zip › ╓¬╟Θ═1⁄4╥Γ╩Θ10/018.jpg]

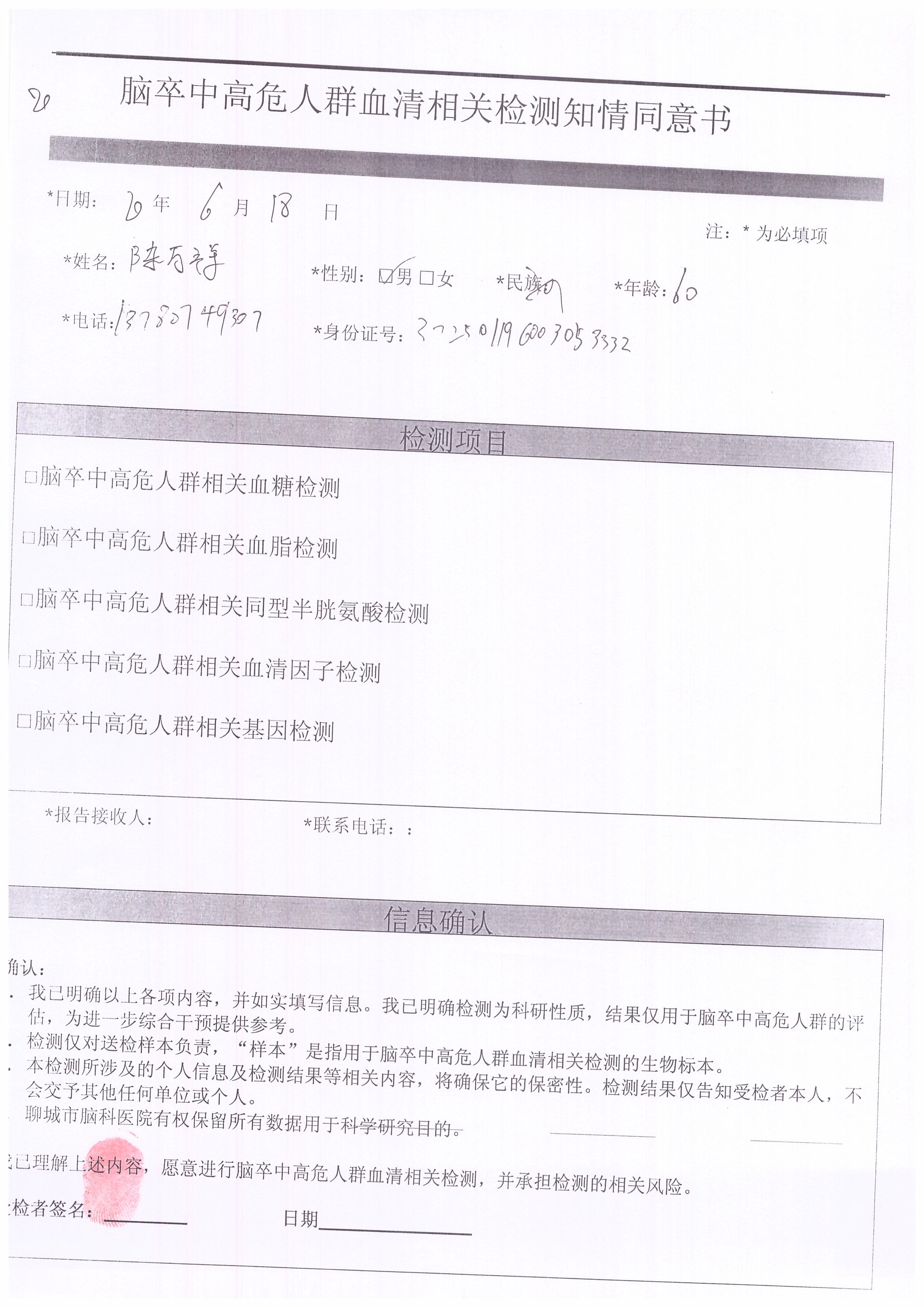

Supplement: Supplementary file 12 — Supplementary file12 (ZIP 2998 KB) [file 10528_2023_10431_MOESM12_ESM.zip › ╓¬╟Θ═1⁄4╥Γ╩Θ10/020.jpg]

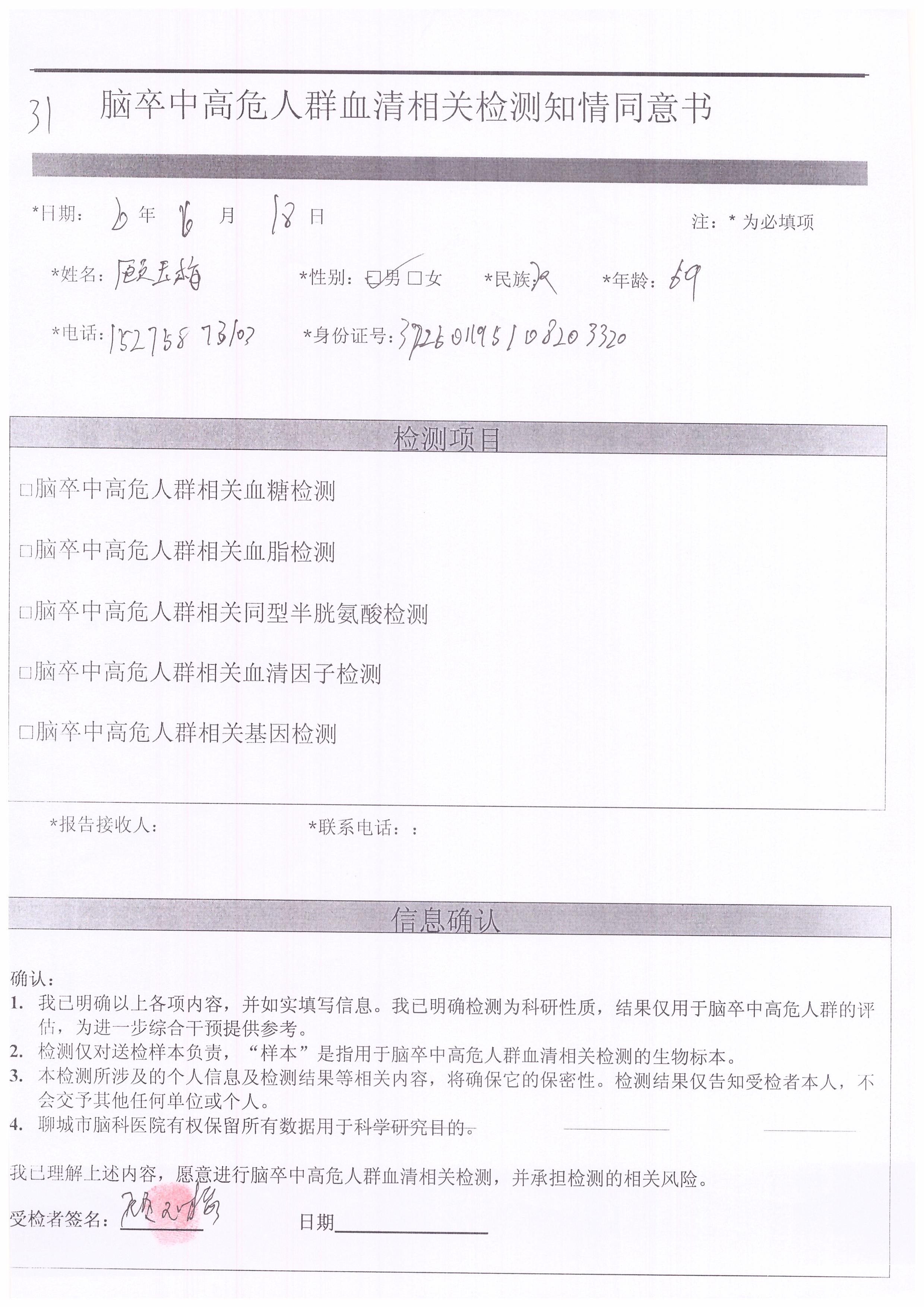

Supplement: Supplementary file 12 — Supplementary file12 (ZIP 2998 KB) [file 10528_2023_10431_MOESM12_ESM.zip › ╓¬╟Θ═1⁄4╥Γ╩Θ10/031.jpg]

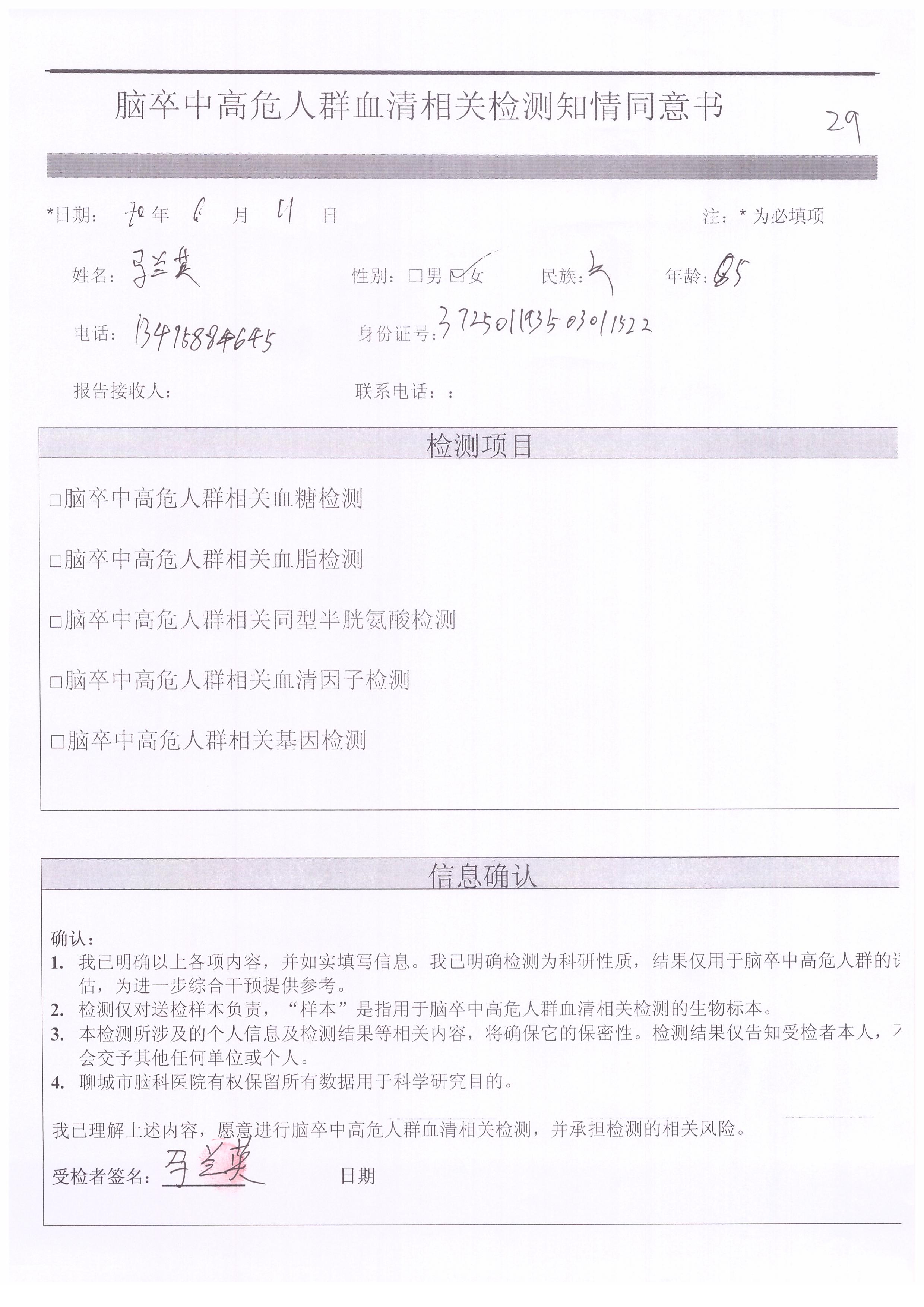

Supplement: Supplementary file 13 — Supplementary file13 (ZIP 28344 KB) [file 10528_2023_10431_MOESM13_ESM.zip › ╓¬╟Θ═1⁄4╥Γ╩Θ11/╡┌2▓┐╖╓/001.jpg]

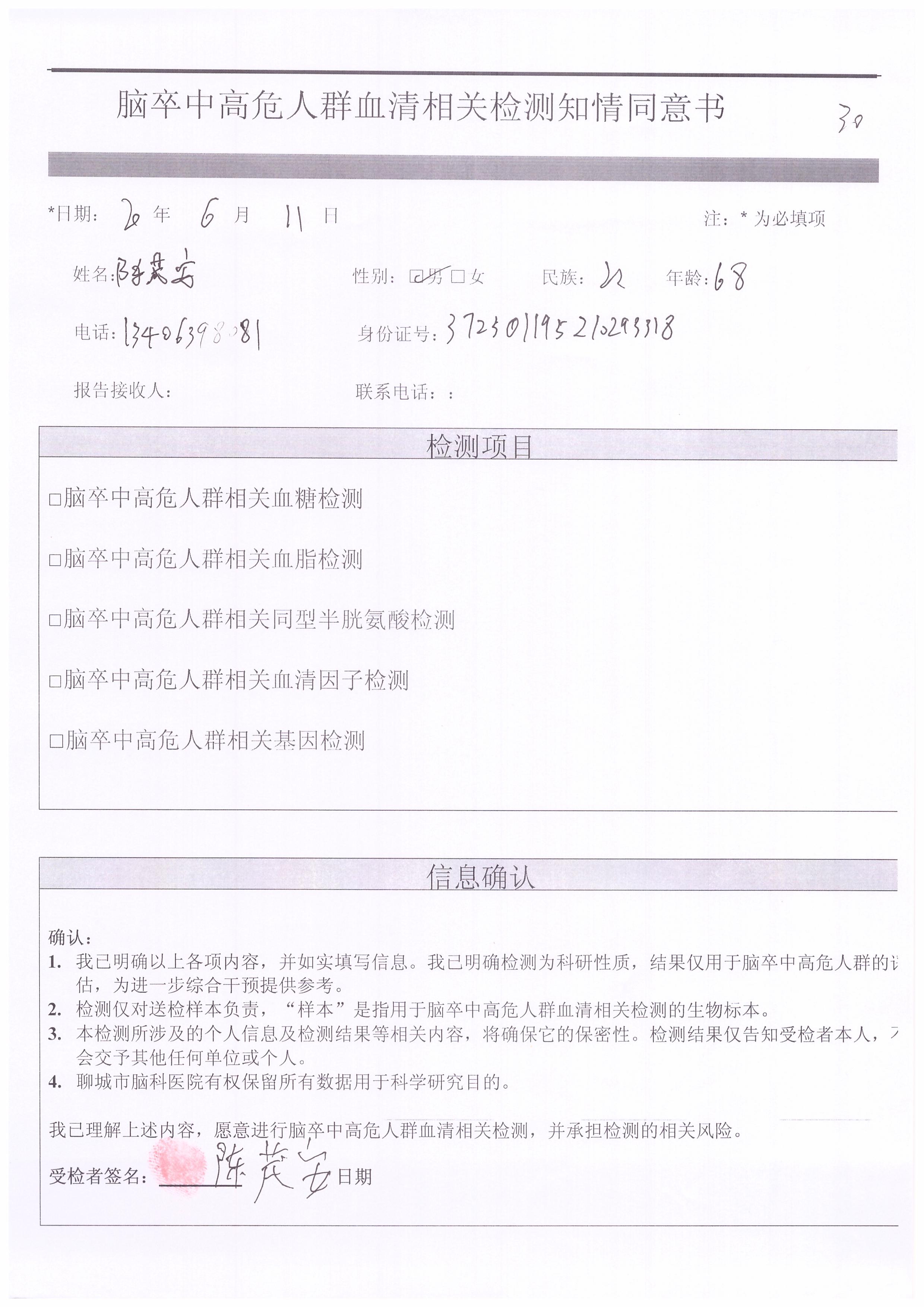

Supplement: Supplementary file 13 — Supplementary file13 (ZIP 28344 KB) [file 10528_2023_10431_MOESM13_ESM.zip › ╓¬╟Θ═1⁄4╥Γ╩Θ11/╡┌2▓┐╖╓/002.jpg]

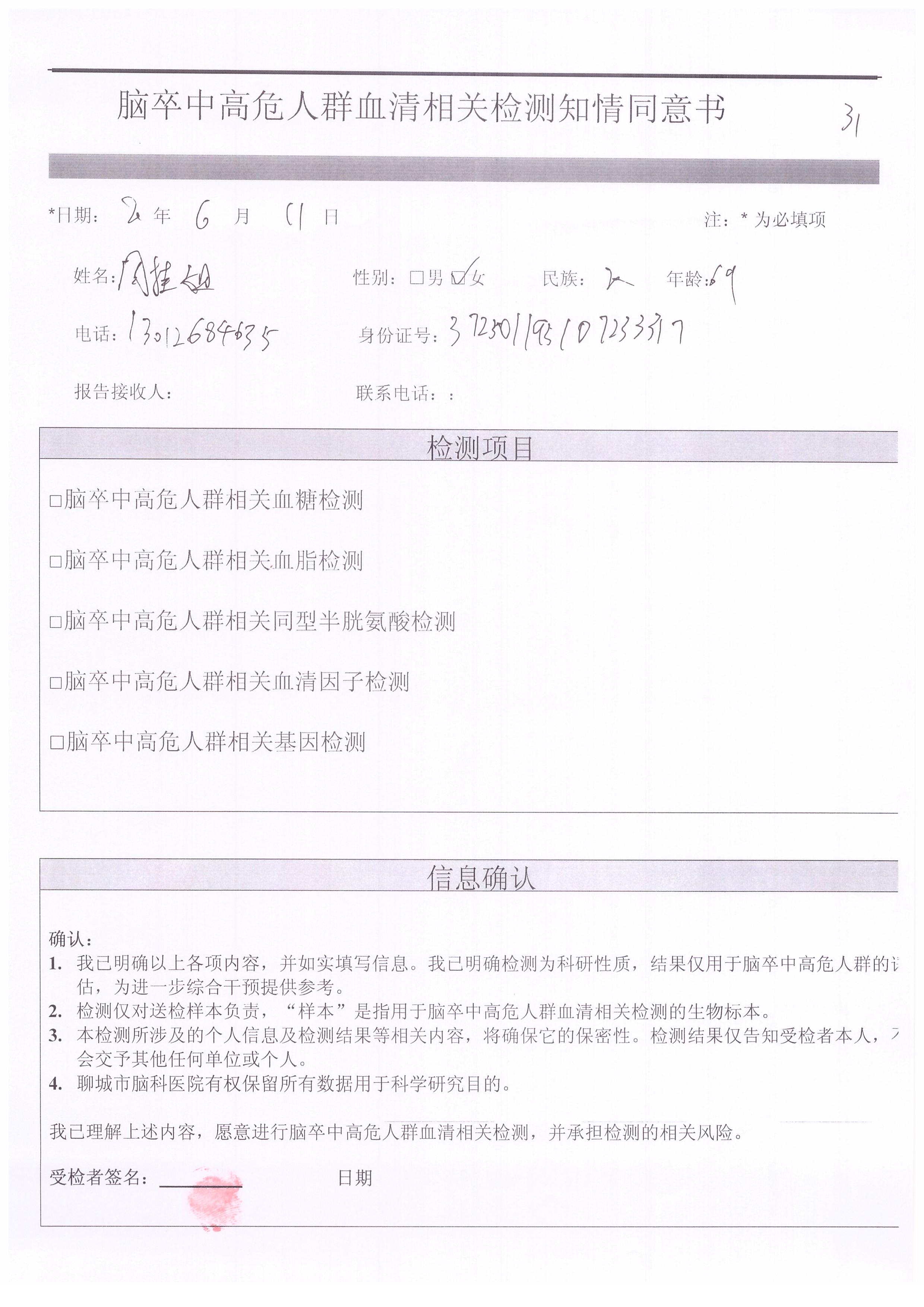

Supplement: Supplementary file 13 — Supplementary file13 (ZIP 28344 KB) [file 10528_2023_10431_MOESM13_ESM.zip › ╓¬╟Θ═1⁄4╥Γ╩Θ11/╡┌2▓┐╖╓/003.jpg]

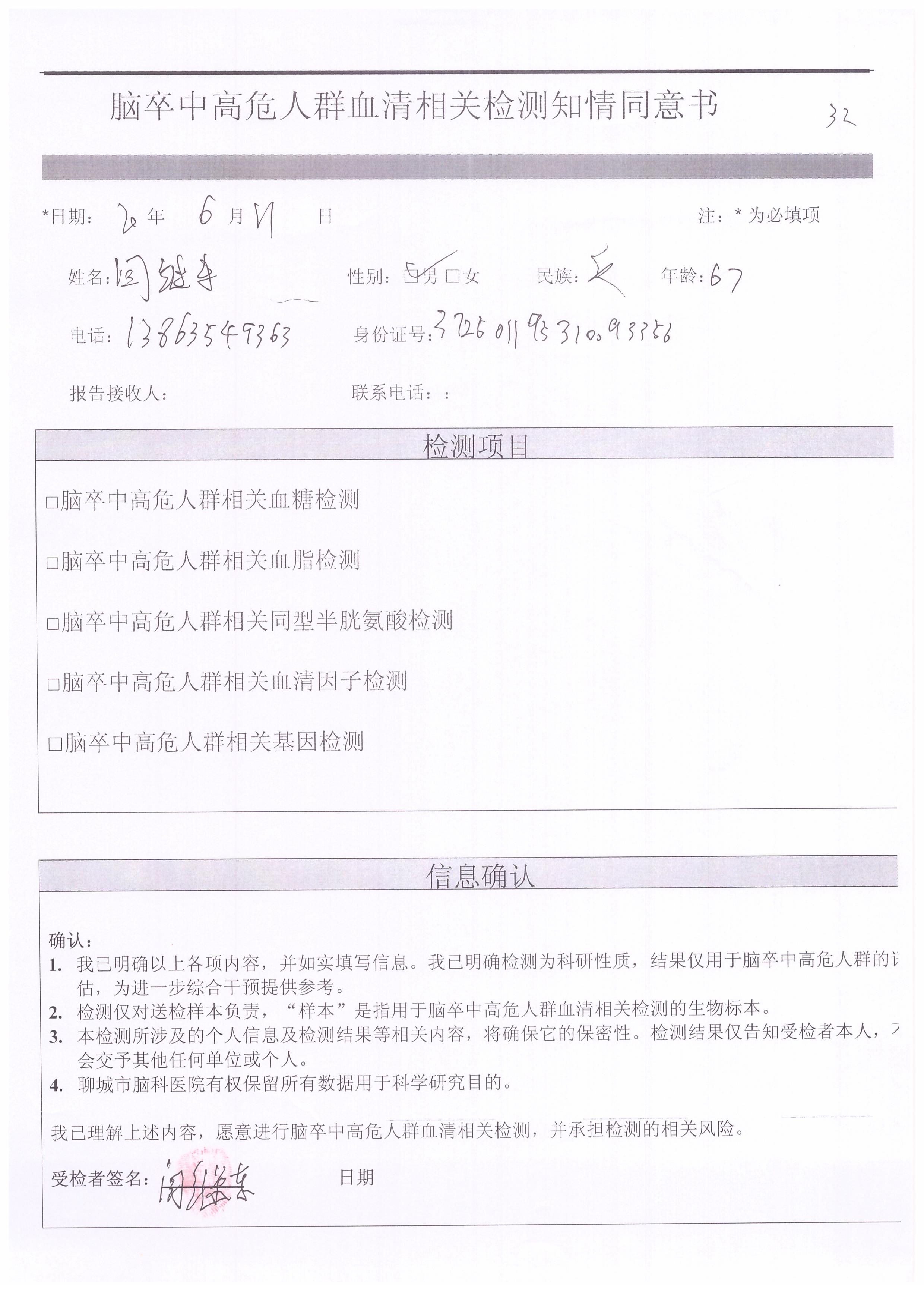

Supplement: Supplementary file 13 — Supplementary file13 (ZIP 28344 KB) [file 10528_2023_10431_MOESM13_ESM.zip › ╓¬╟Θ═1⁄4╥Γ╩Θ11/╡┌2▓┐╖╓/004.jpg]

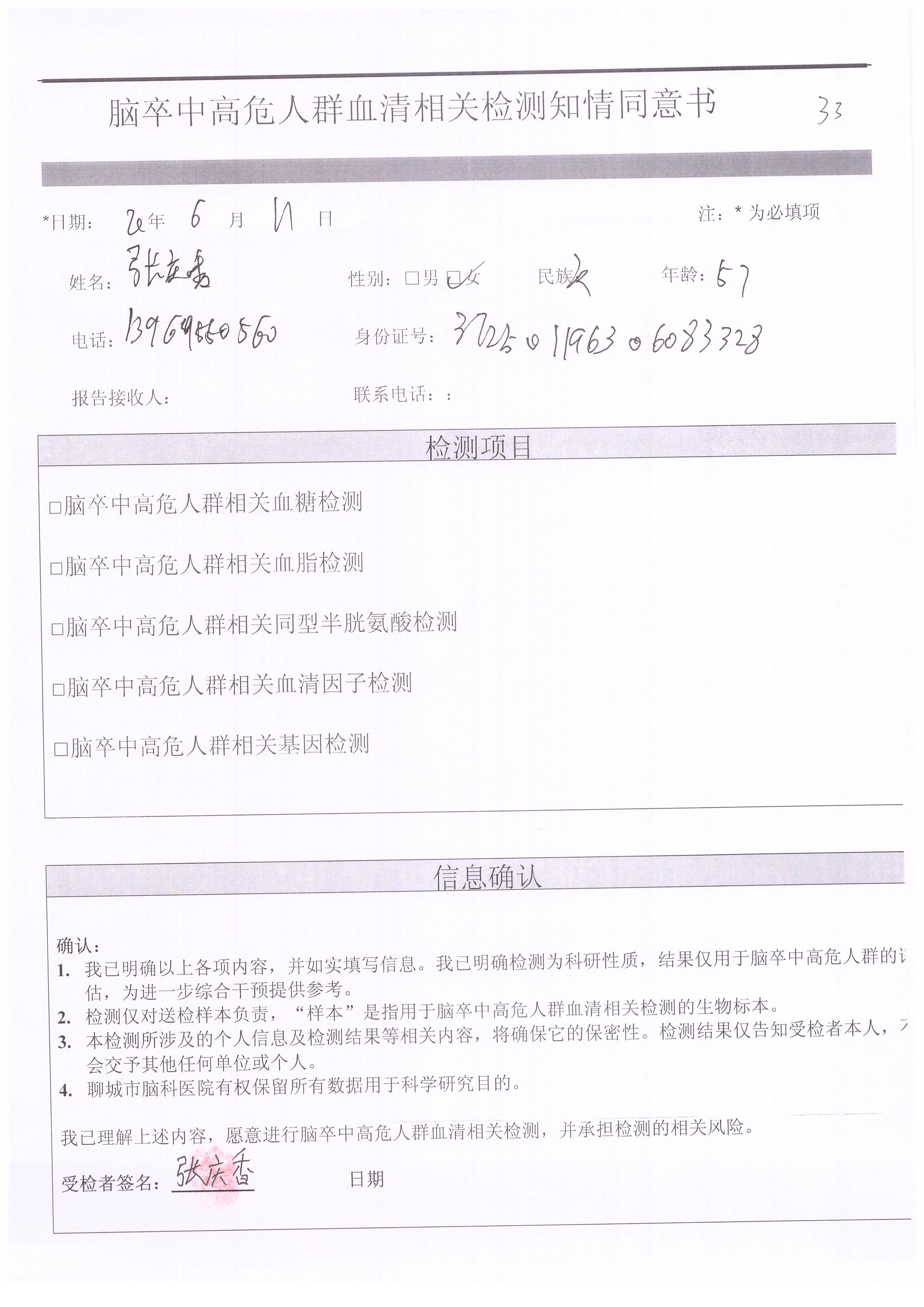

Supplement: Supplementary file 13 — Supplementary file13 (ZIP 28344 KB) [file 10528_2023_10431_MOESM13_ESM.zip › ╓¬╟Θ═1⁄4╥Γ╩Θ11/╡┌2▓┐╖╓/005.jpg]

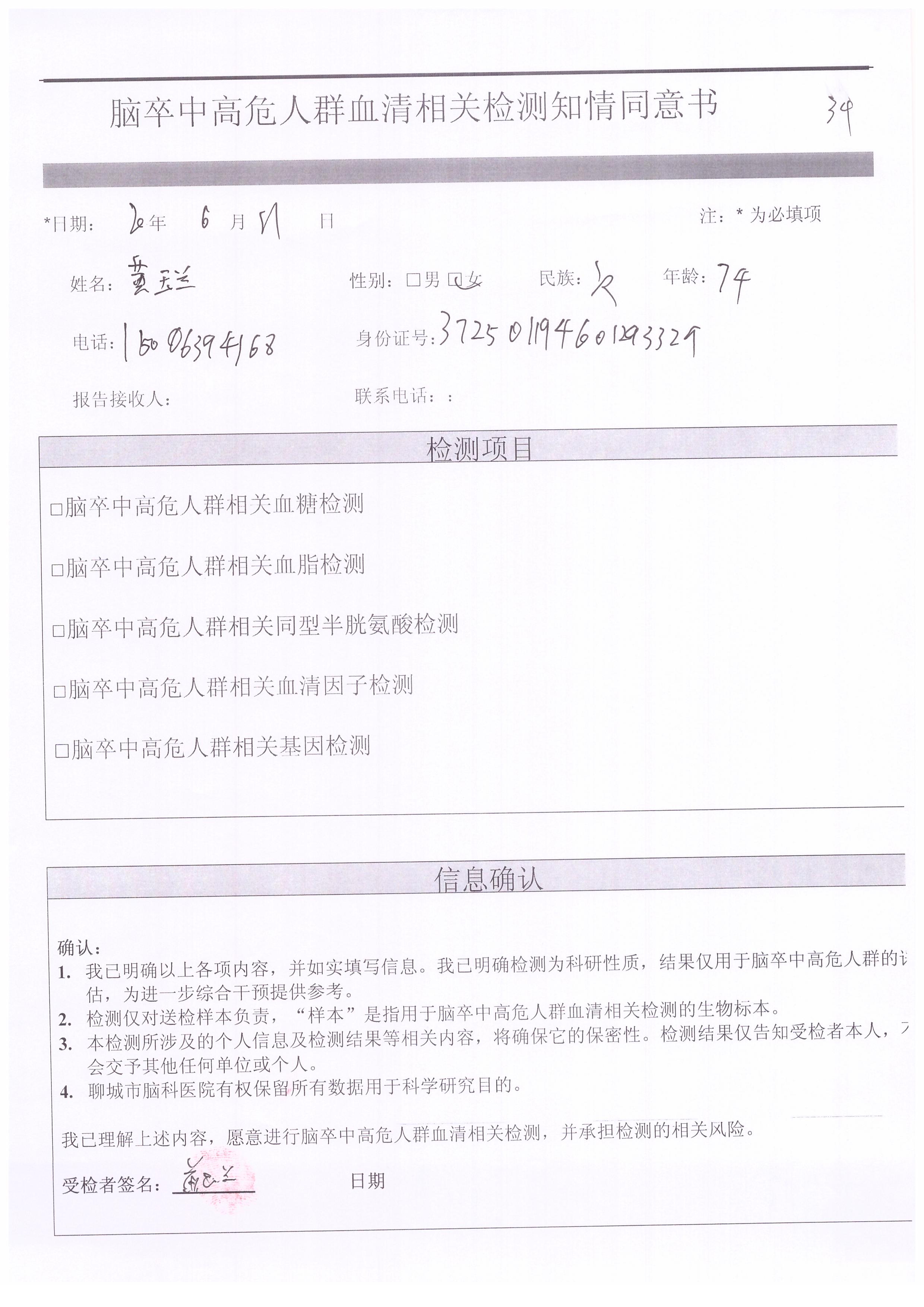

Supplement: Supplementary file 13 — Supplementary file13 (ZIP 28344 KB) [file 10528_2023_10431_MOESM13_ESM.zip › ╓¬╟Θ═1⁄4╥Γ╩Θ11/╡┌2▓┐╖╓/006.jpg]

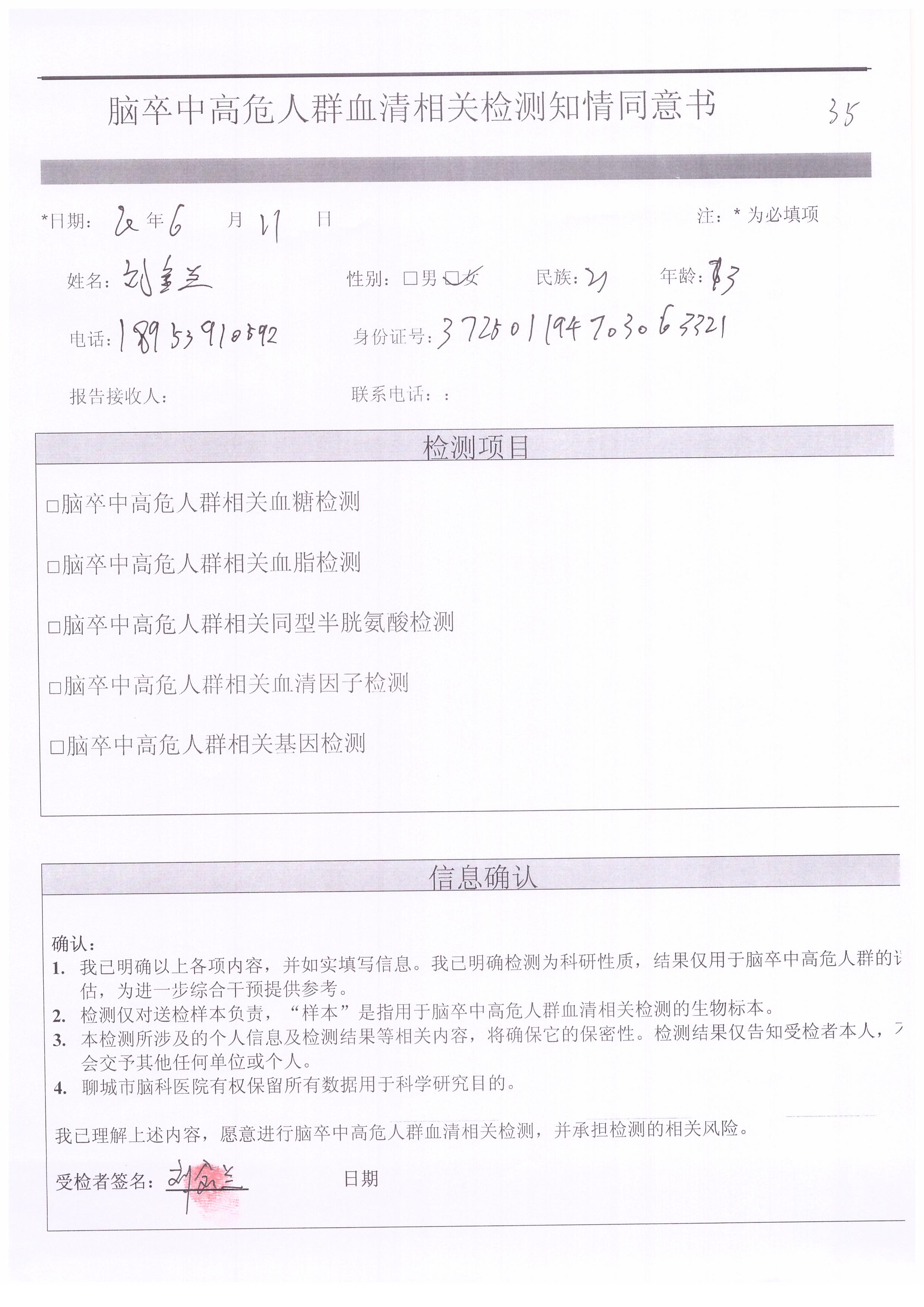

Supplement: Supplementary file 13 — Supplementary file13 (ZIP 28344 KB) [file 10528_2023_10431_MOESM13_ESM.zip › ╓¬╟Θ═1⁄4╥Γ╩Θ11/╡┌2▓┐╖╓/007.jpg]

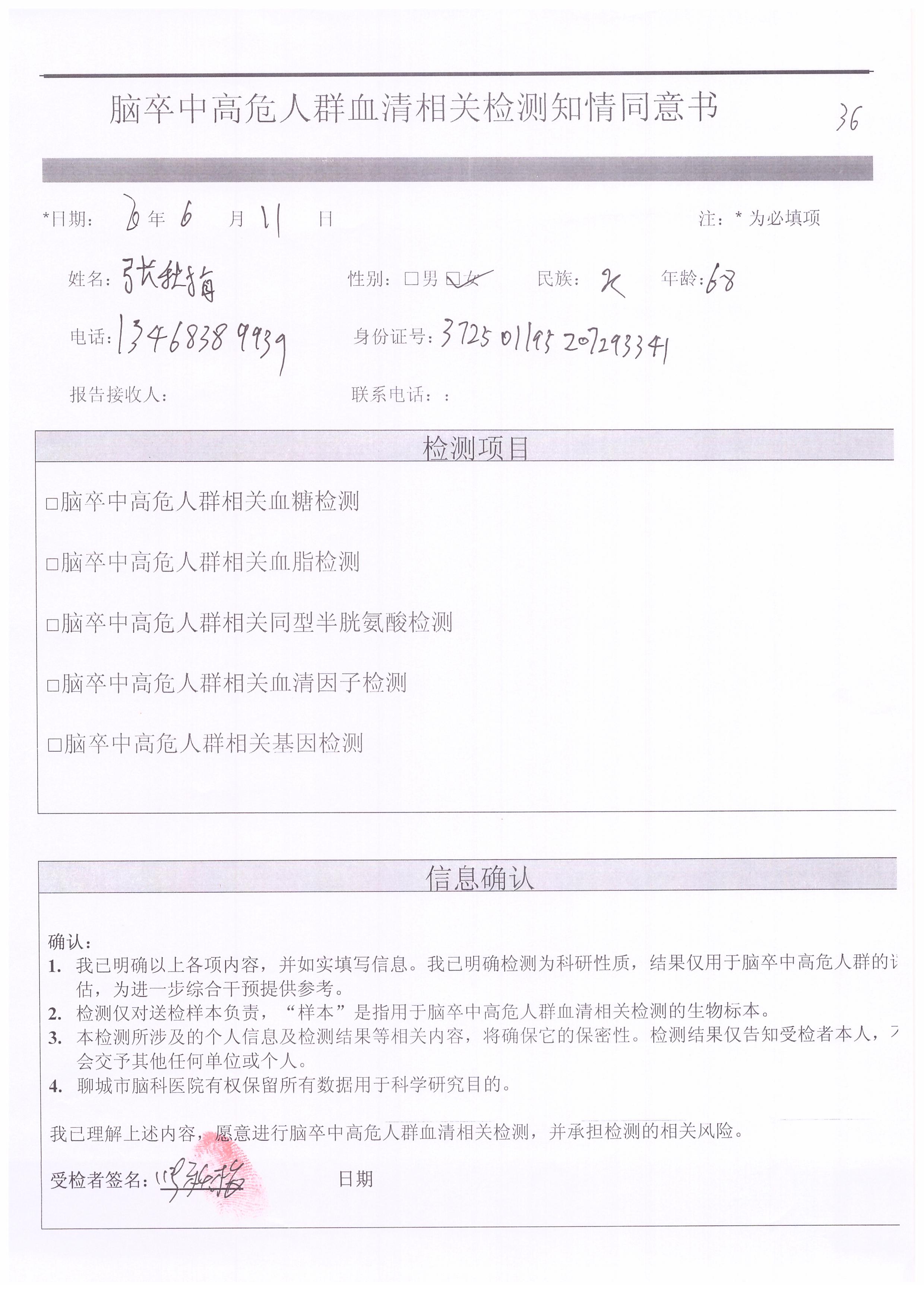

Supplement: Supplementary file 13 — Supplementary file13 (ZIP 28344 KB) [file 10528_2023_10431_MOESM13_ESM.zip › ╓¬╟Θ═1⁄4╥Γ╩Θ11/╡┌2▓┐╖╓/008.jpg]

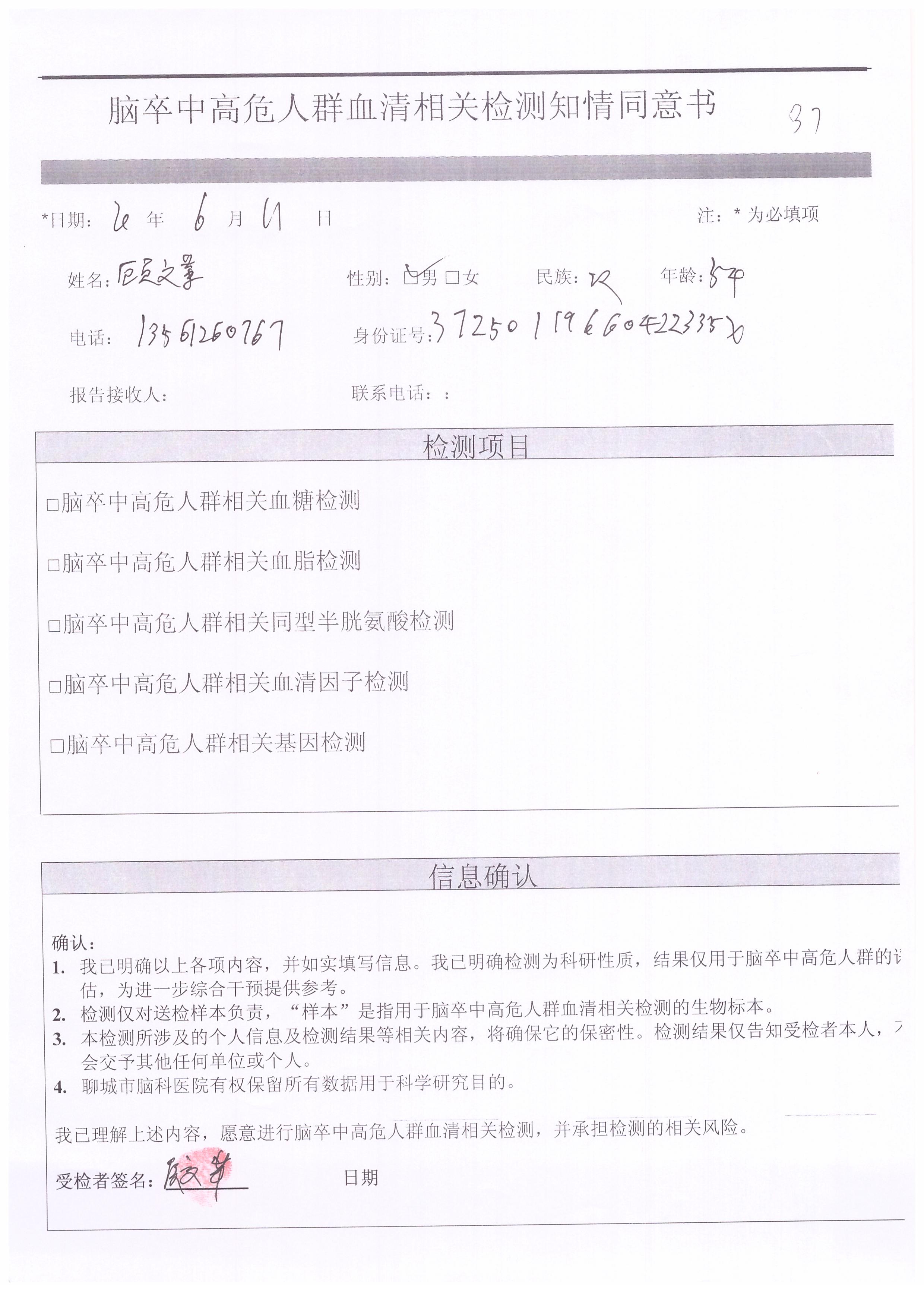

Supplement: Supplementary file 13 — Supplementary file13 (ZIP 28344 KB) [file 10528_2023_10431_MOESM13_ESM.zip › ╓¬╟Θ═1⁄4╥Γ╩Θ11/╡┌2▓┐╖╓/009.jpg]

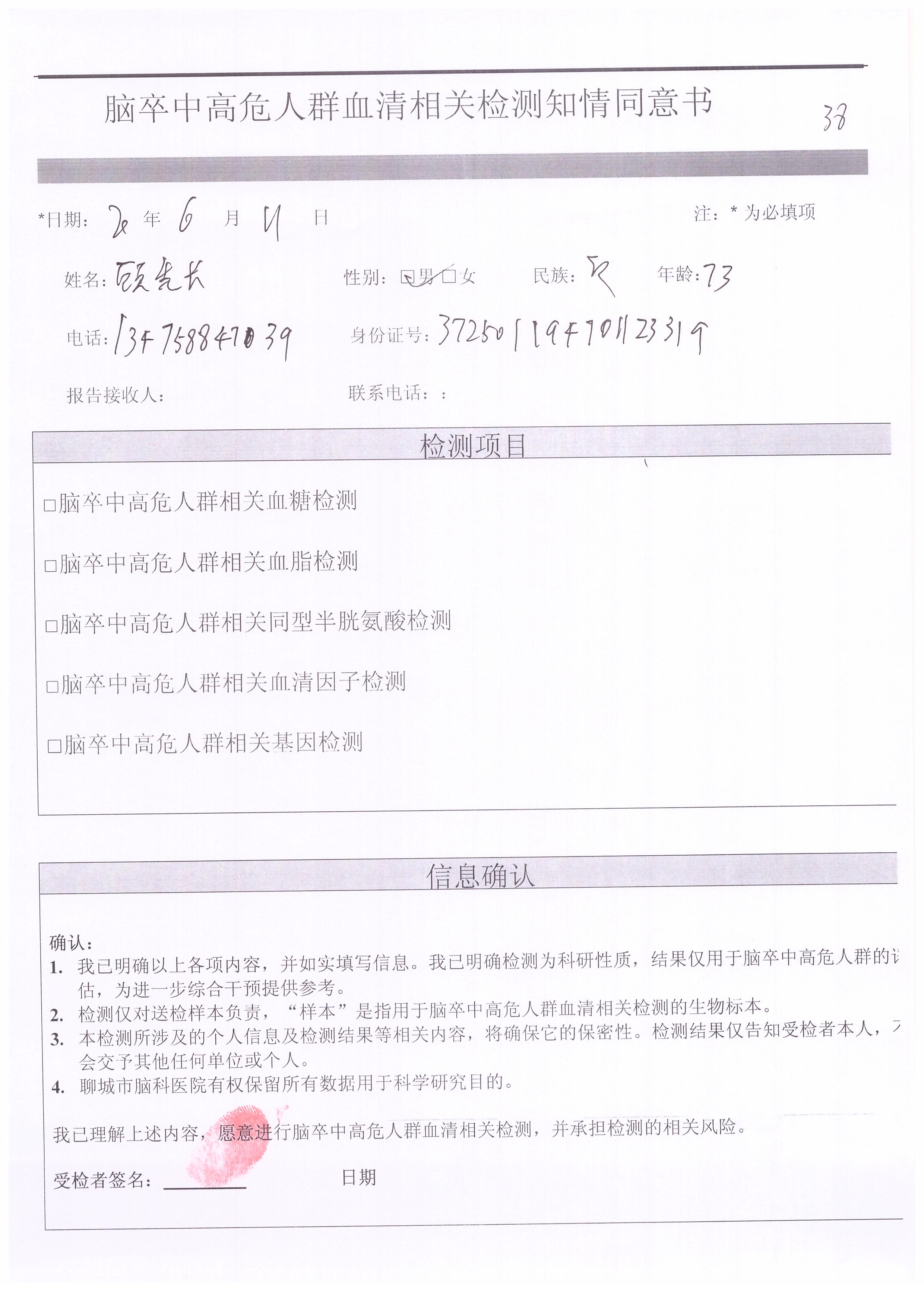

Supplement: Supplementary file 13 — Supplementary file13 (ZIP 28344 KB) [file 10528_2023_10431_MOESM13_ESM.zip › ╓¬╟Θ═1⁄4╥Γ╩Θ11/╡┌2▓┐╖╓/010.jpg]

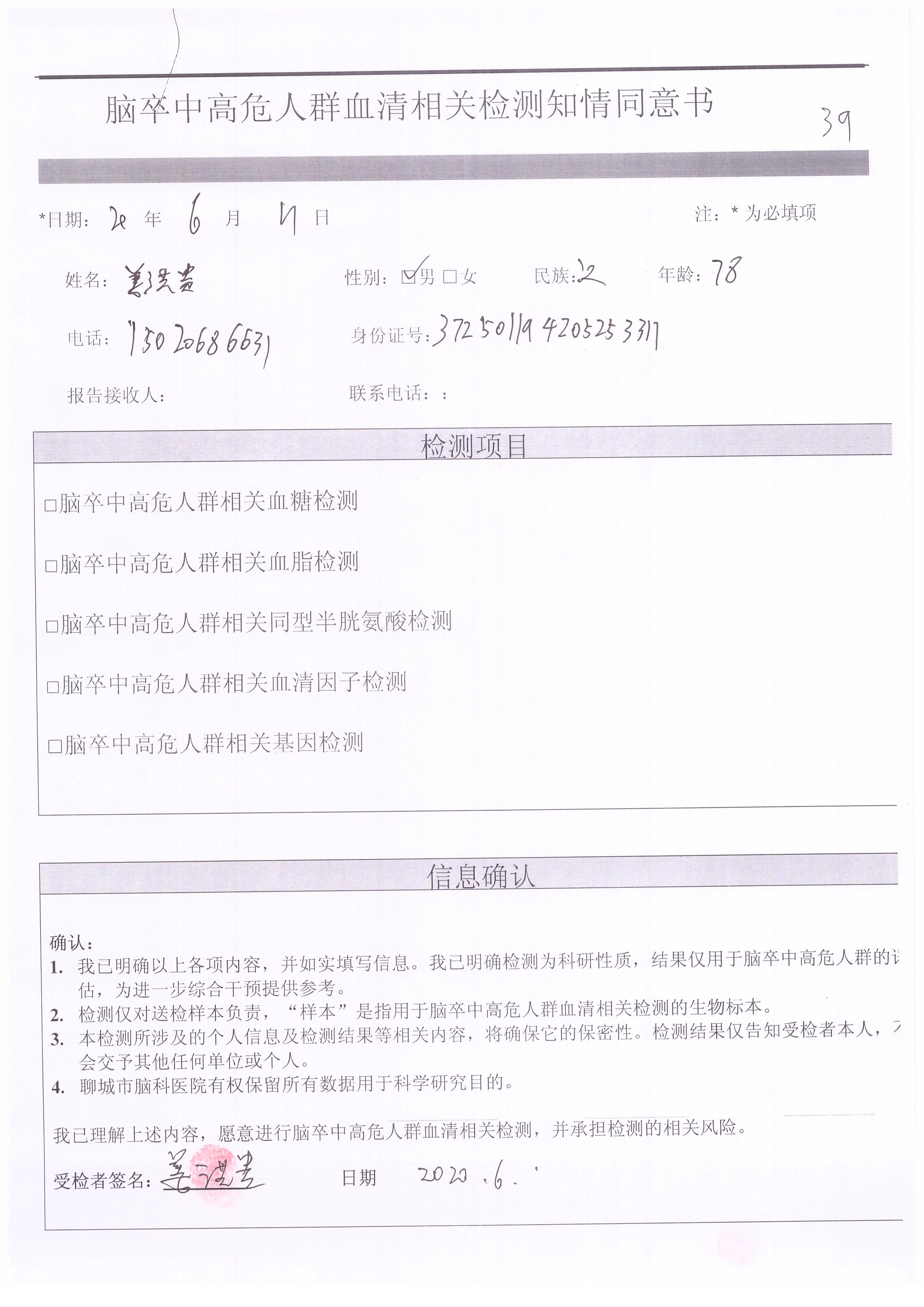

Supplement: Supplementary file 13 — Supplementary file13 (ZIP 28344 KB) [file 10528_2023_10431_MOESM13_ESM.zip › ╓¬╟Θ═1⁄4╥Γ╩Θ11/╡┌2▓┐╖╓/011.jpg]

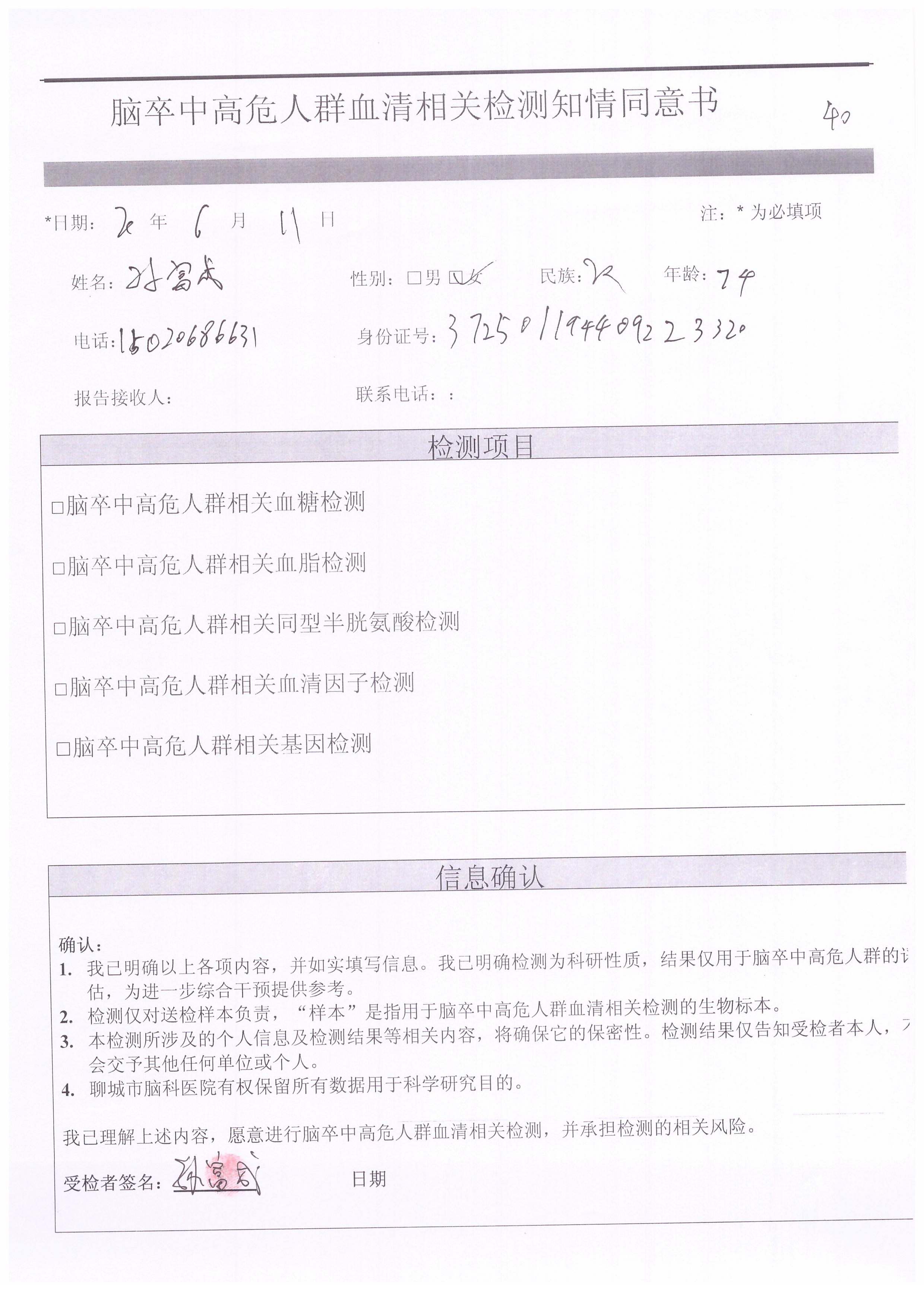

Supplement: Supplementary file 13 — Supplementary file13 (ZIP 28344 KB) [file 10528_2023_10431_MOESM13_ESM.zip › ╓¬╟Θ═1⁄4╥Γ╩Θ11/╡┌2▓┐╖╓/012.jpg]

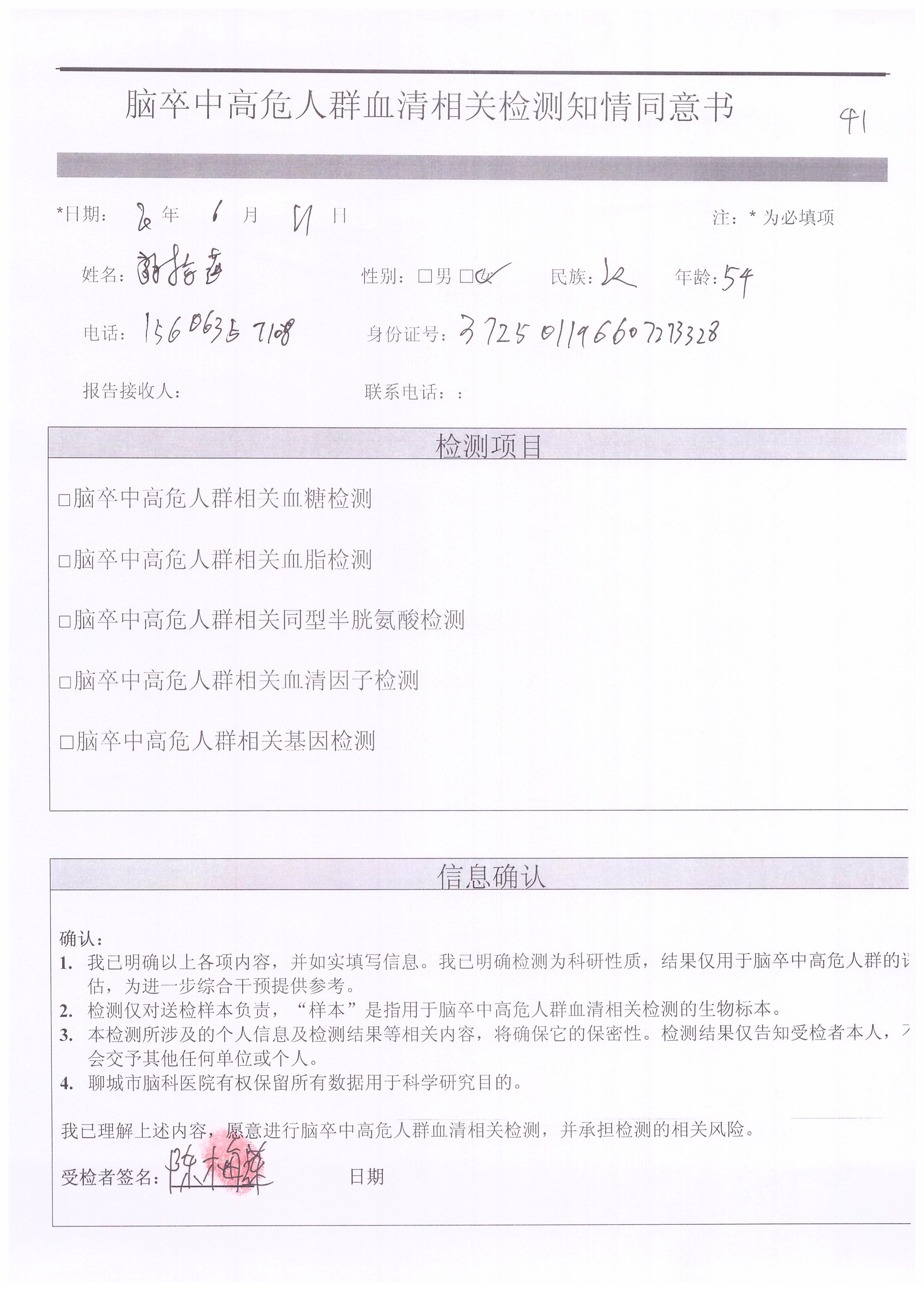

Supplement: Supplementary file 13 — Supplementary file13 (ZIP 28344 KB) [file 10528_2023_10431_MOESM13_ESM.zip › ╓¬╟Θ═1⁄4╥Γ╩Θ11/╡┌2▓┐╖╓/013.jpg]

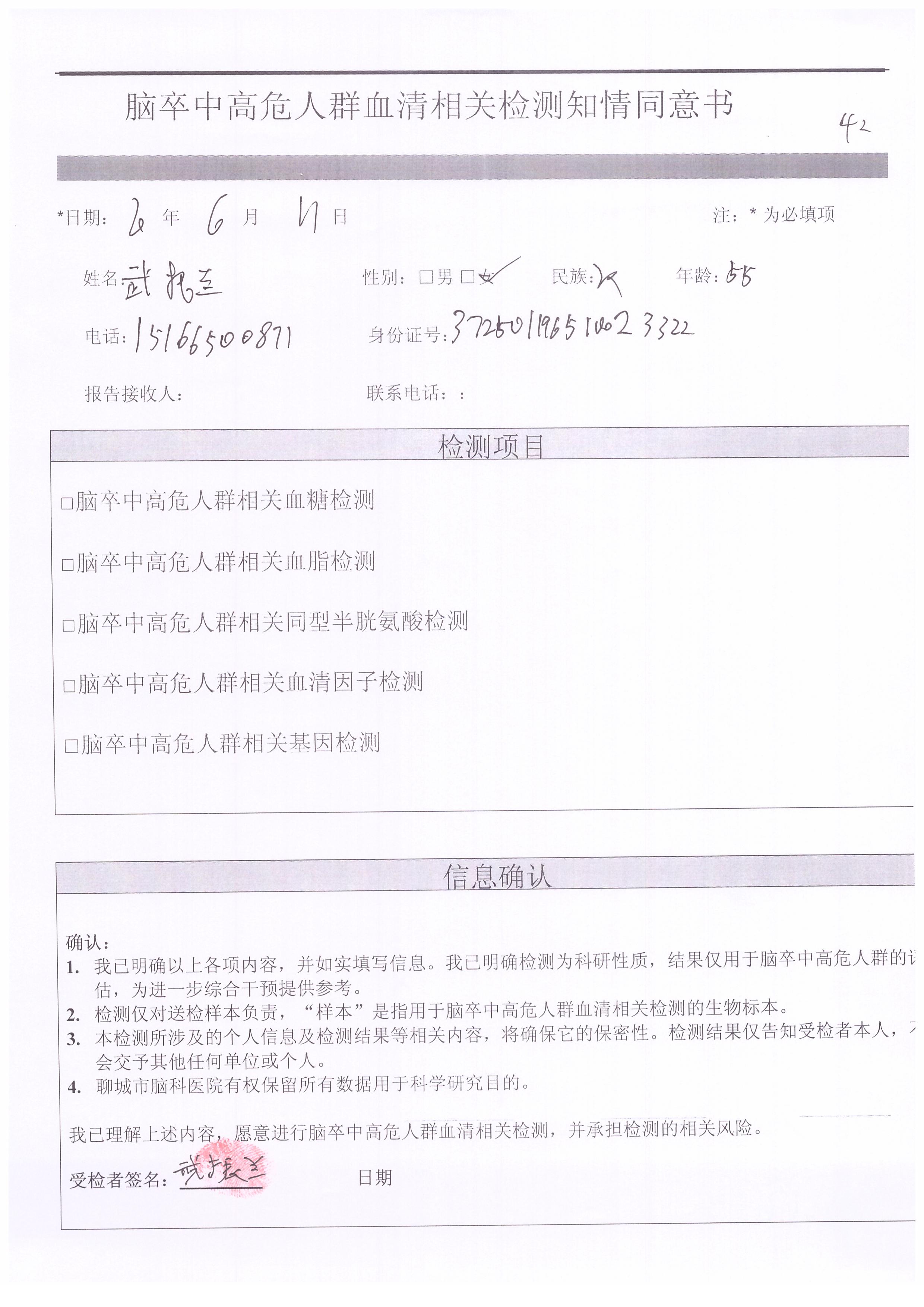

Supplement: Supplementary file 13 — Supplementary file13 (ZIP 28344 KB) [file 10528_2023_10431_MOESM13_ESM.zip › ╓¬╟Θ═1⁄4╥Γ╩Θ11/╡┌2▓┐╖╓/014.jpg]

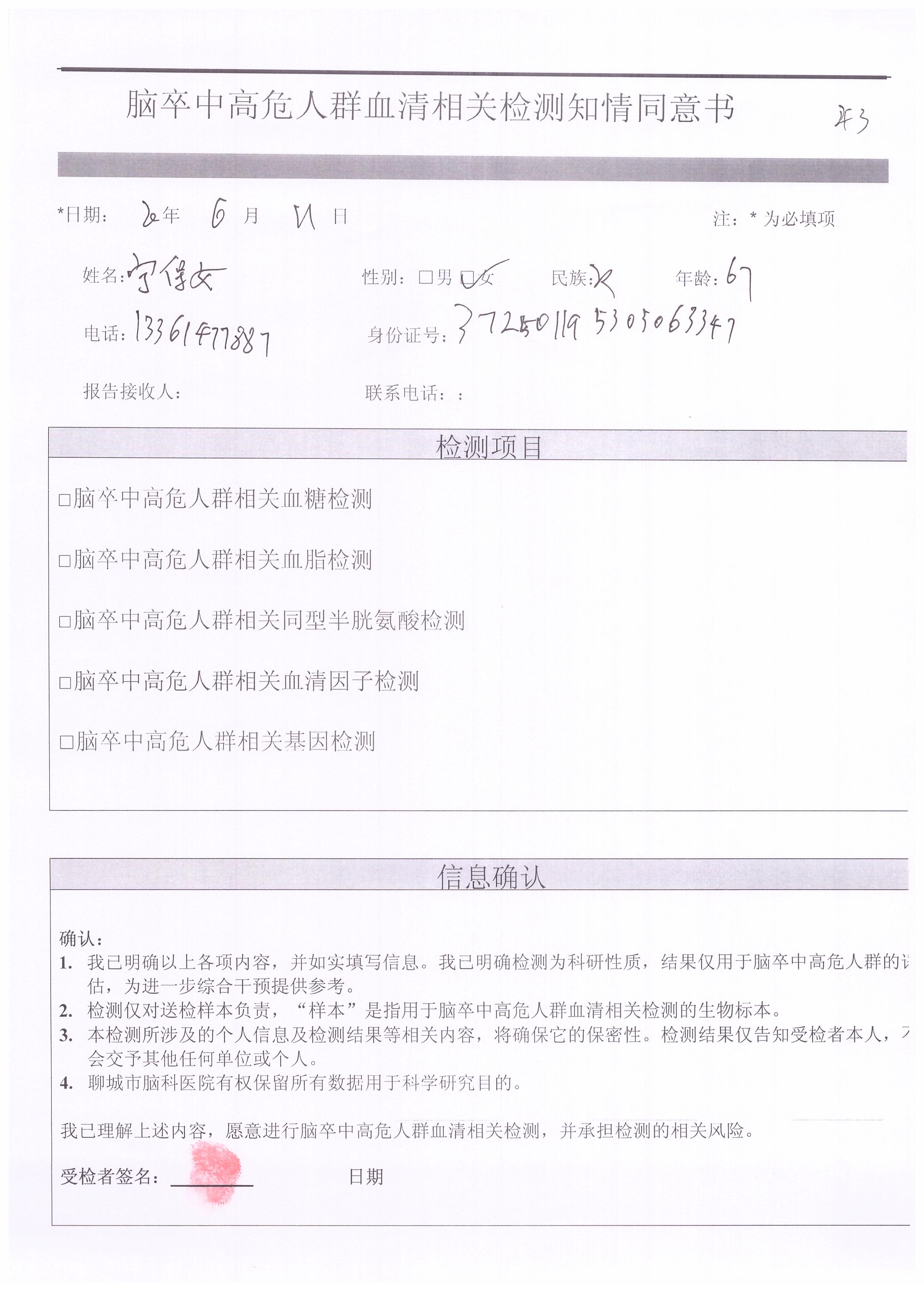

Supplement: Supplementary file 13 — Supplementary file13 (ZIP 28344 KB) [file 10528_2023_10431_MOESM13_ESM.zip › ╓¬╟Θ═1⁄4╥Γ╩Θ11/╡┌2▓┐╖╓/015.jpg]

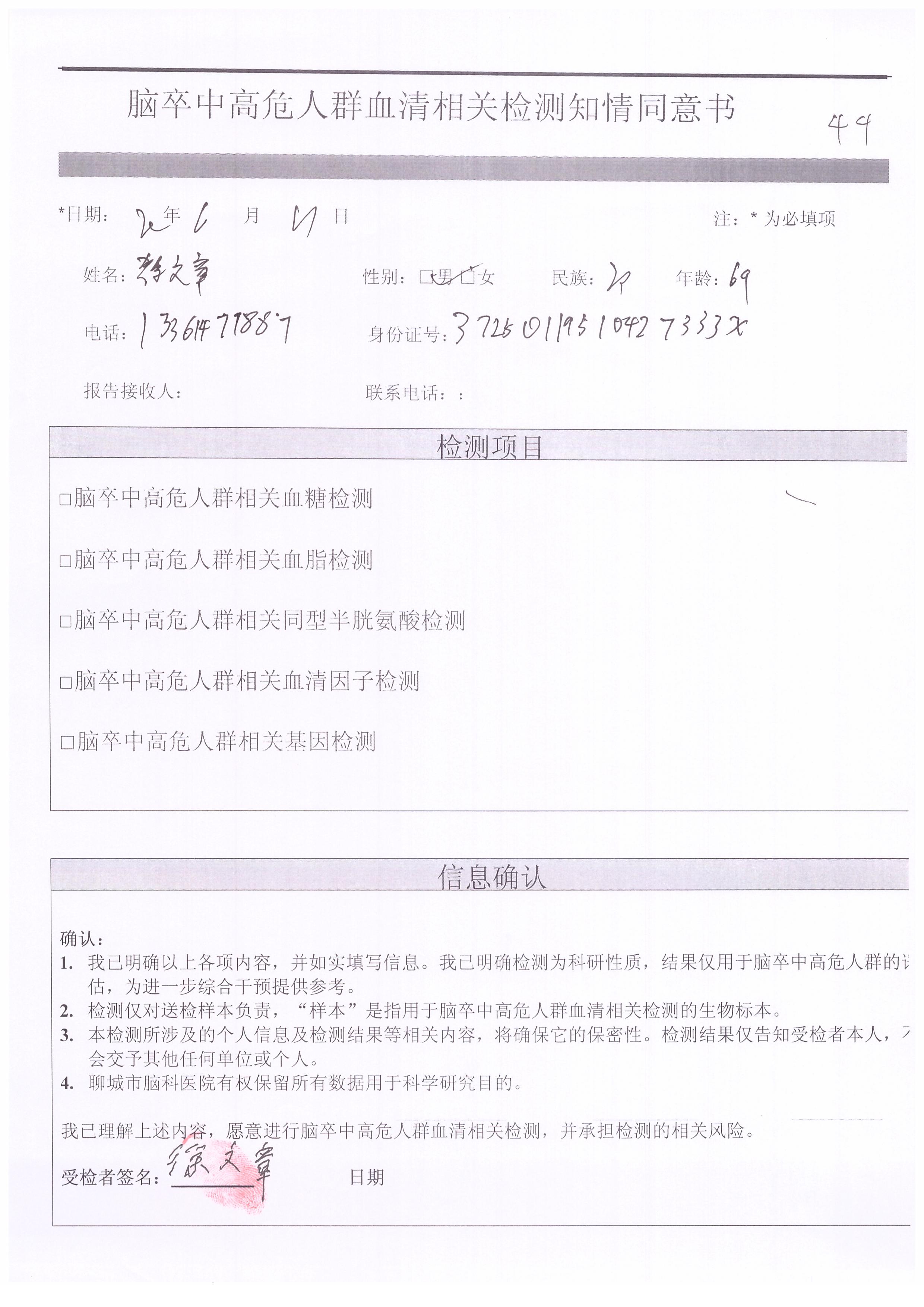

Supplement: Supplementary file 13 — Supplementary file13 (ZIP 28344 KB) [file 10528_2023_10431_MOESM13_ESM.zip › ╓¬╟Θ═1⁄4╥Γ╩Θ11/╡┌2▓┐╖╓/016.jpg]

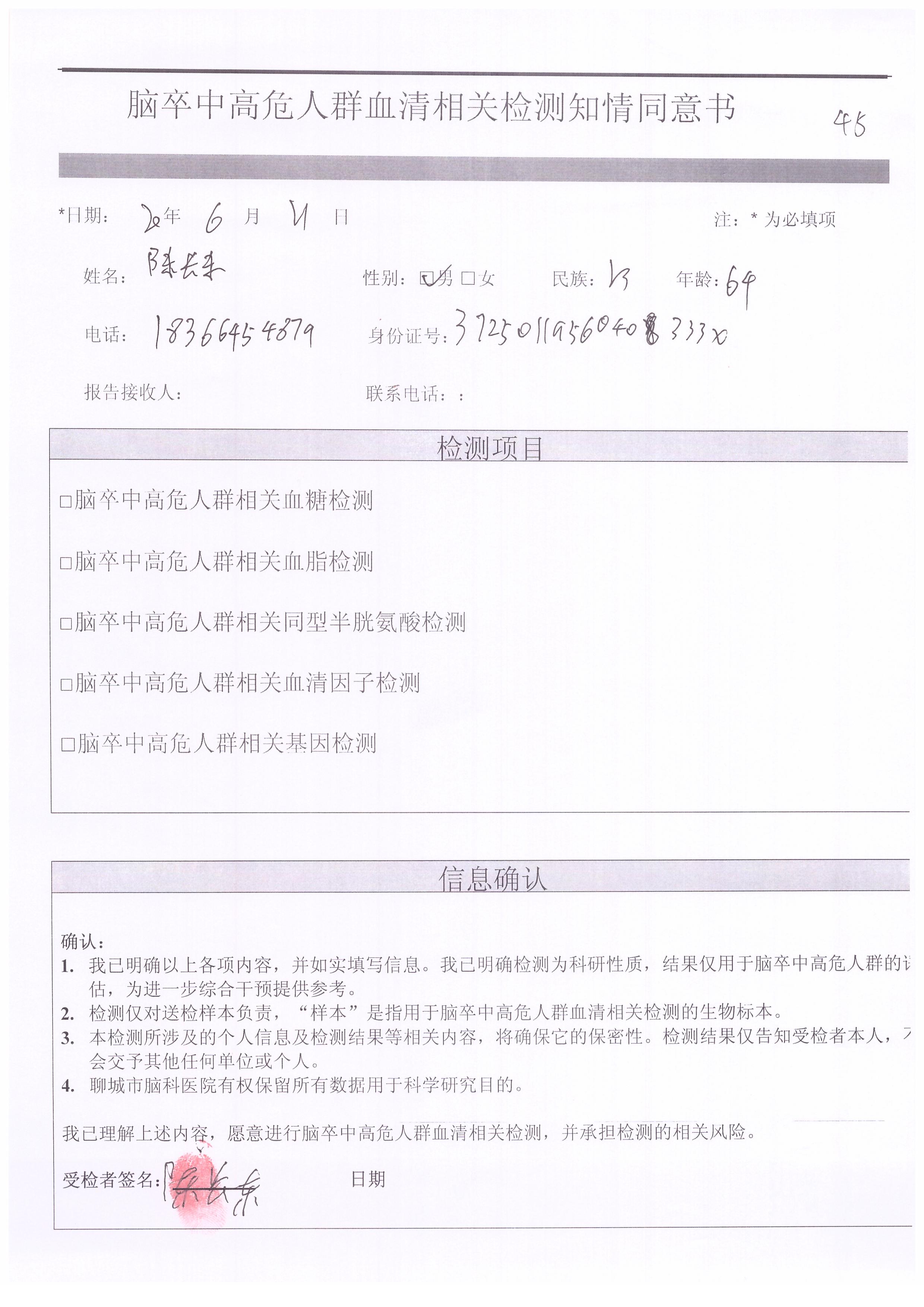

Supplement: Supplementary file 13 — Supplementary file13 (ZIP 28344 KB) [file 10528_2023_10431_MOESM13_ESM.zip › ╓¬╟Θ═1⁄4╥Γ╩Θ11/╡┌2▓┐╖╓/017.jpg]

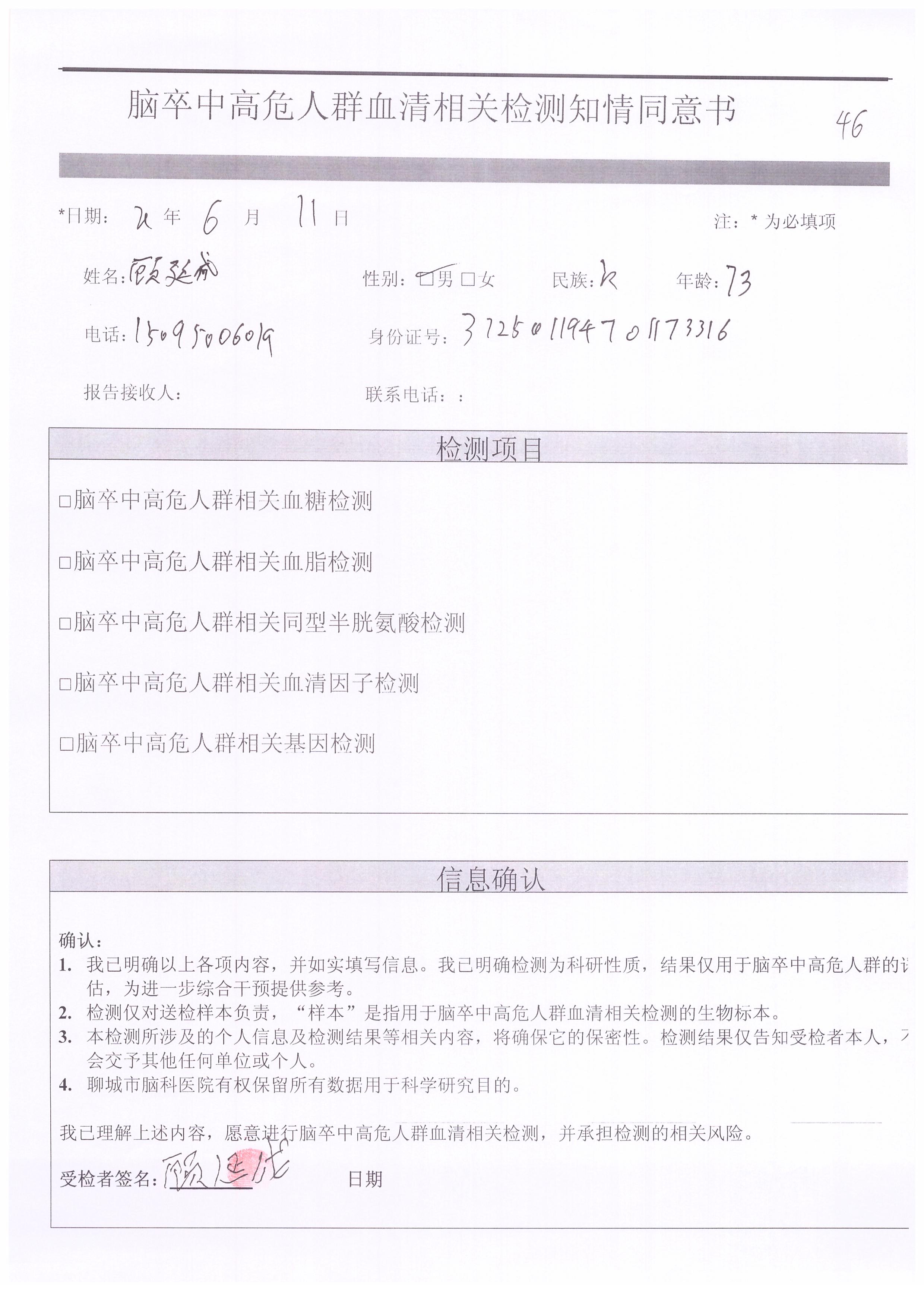

Supplement: Supplementary file 13 — Supplementary file13 (ZIP 28344 KB) [file 10528_2023_10431_MOESM13_ESM.zip › ╓¬╟Θ═1⁄4╥Γ╩Θ11/╡┌2▓┐╖╓/018.jpg]

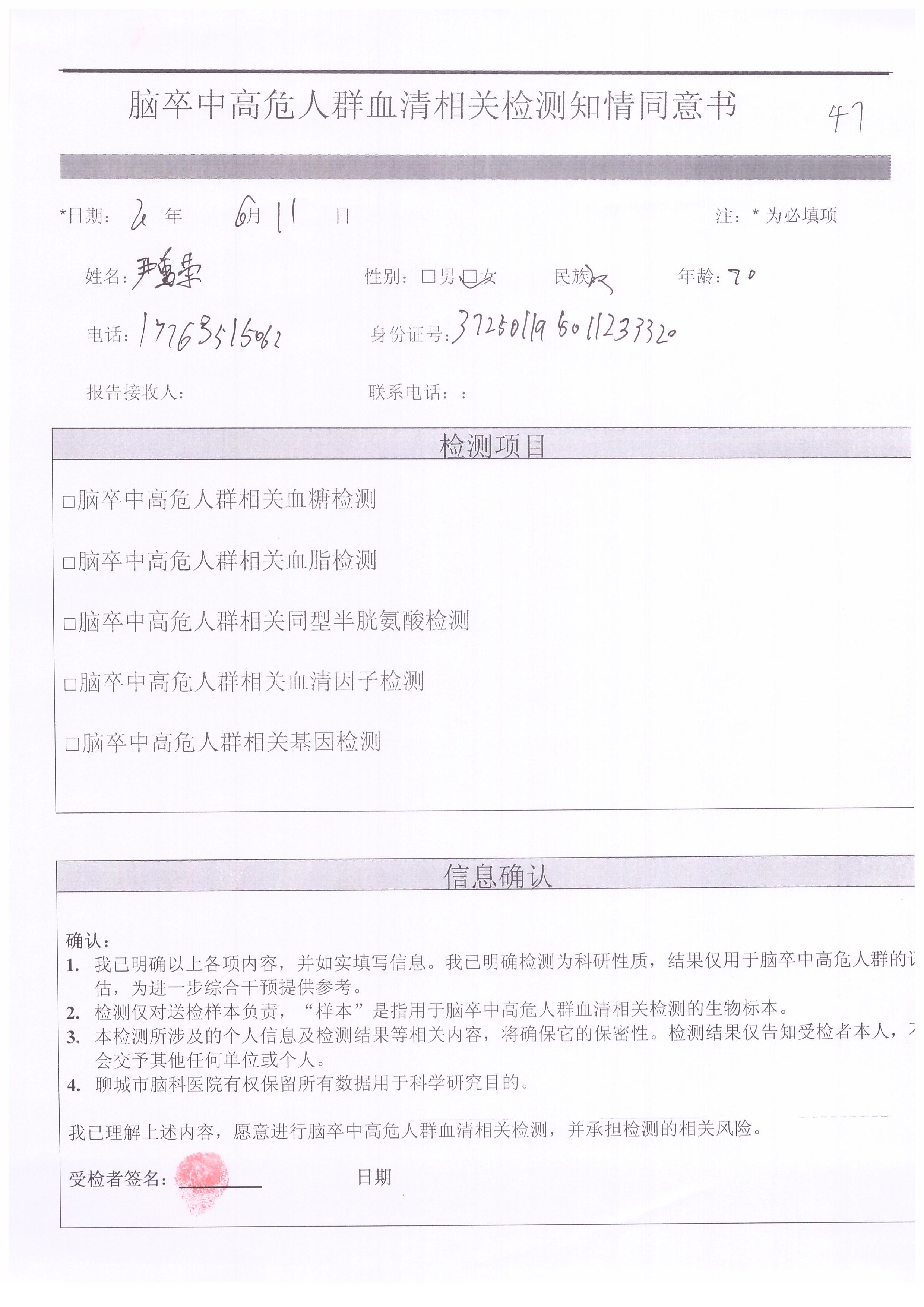

Supplement: Supplementary file 13 — Supplementary file13 (ZIP 28344 KB) [file 10528_2023_10431_MOESM13_ESM.zip › ╓¬╟Θ═1⁄4╥Γ╩Θ11/╡┌2▓┐╖╓/019.jpg]

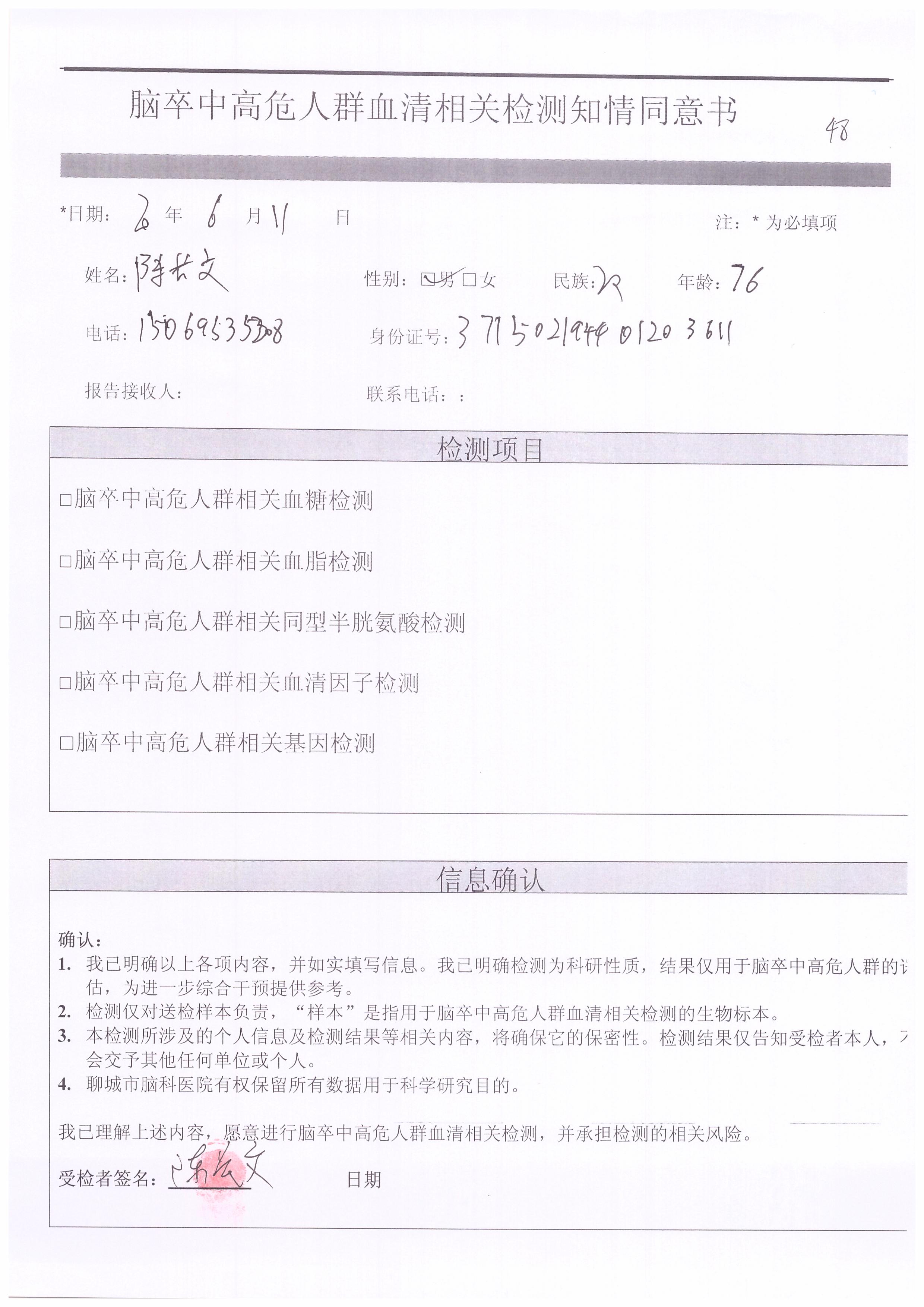

Supplement: Supplementary file 13 — Supplementary file13 (ZIP 28344 KB) [file 10528_2023_10431_MOESM13_ESM.zip › ╓¬╟Θ═1⁄4╥Γ╩Θ11/╡┌2▓┐╖╓/020.jpg]

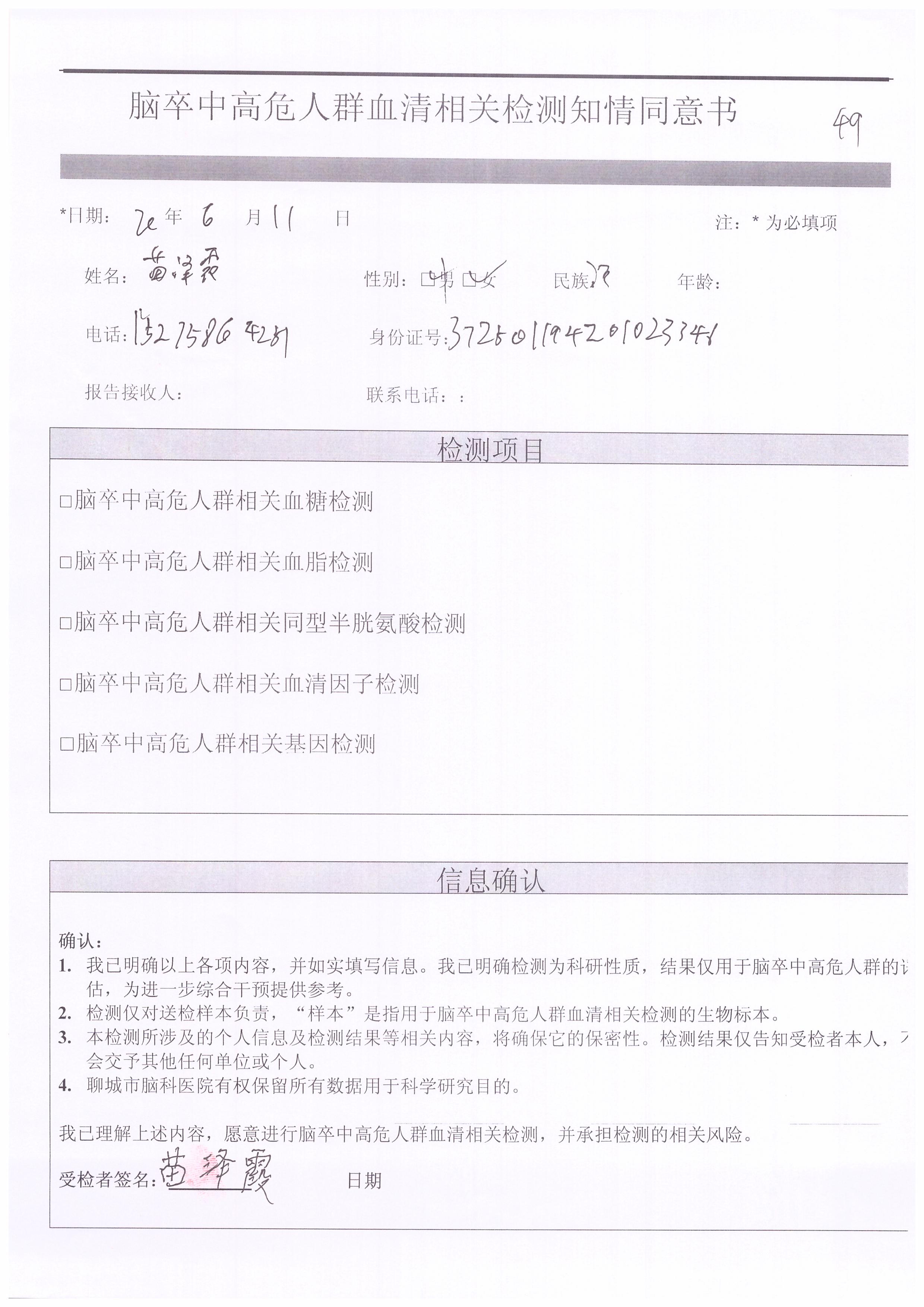

Supplement: Supplementary file 13 — Supplementary file13 (ZIP 28344 KB) [file 10528_2023_10431_MOESM13_ESM.zip › ╓¬╟Θ═1⁄4╥Γ╩Θ11/╡┌2▓┐╖╓/021.jpg]

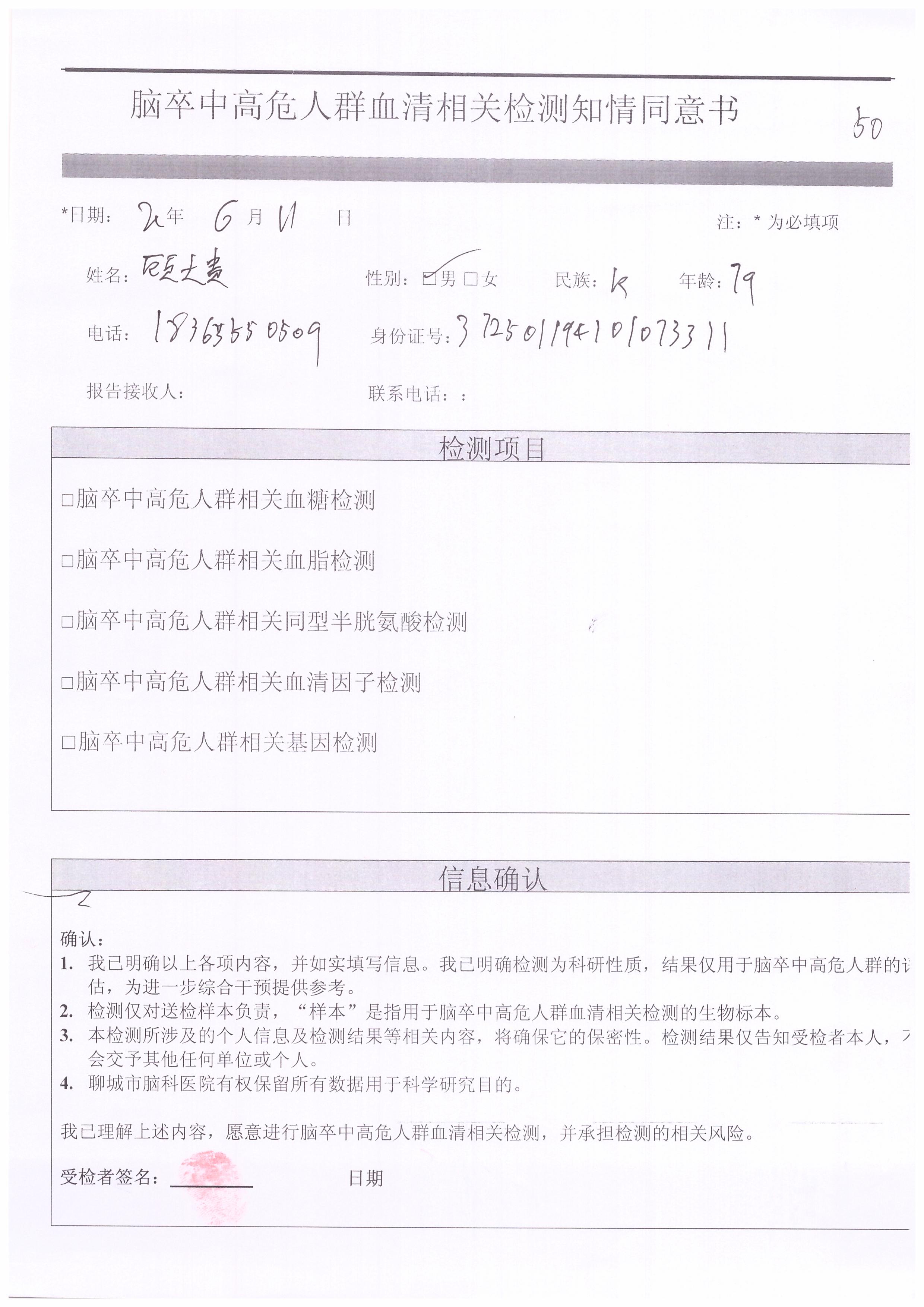

Supplement: Supplementary file 13 — Supplementary file13 (ZIP 28344 KB) [file 10528_2023_10431_MOESM13_ESM.zip › ╓¬╟Θ═1⁄4╥Γ╩Θ11/╡┌2▓┐╖╓/022.jpg]

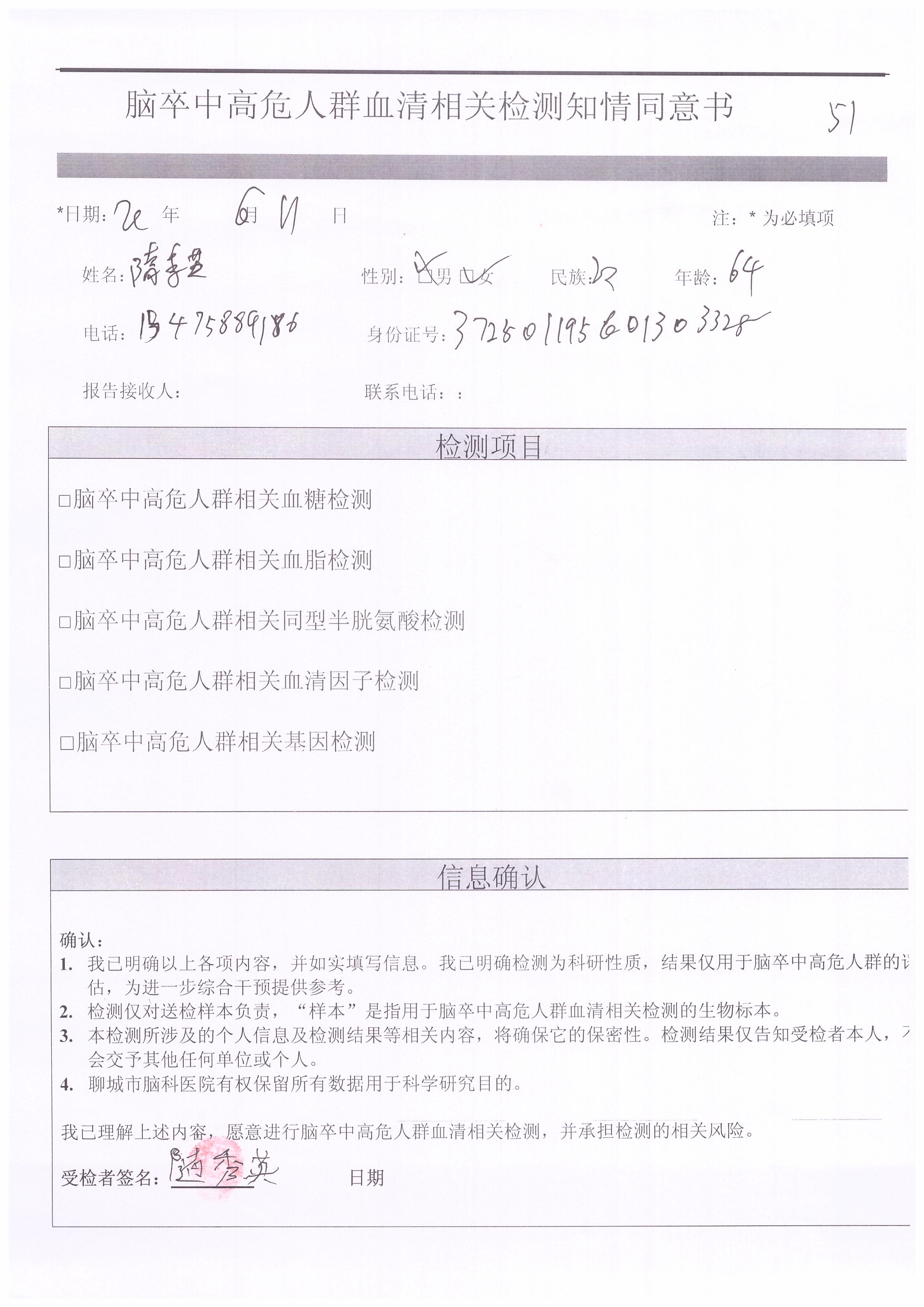

Supplement: Supplementary file 13 — Supplementary file13 (ZIP 28344 KB) [file 10528_2023_10431_MOESM13_ESM.zip › ╓¬╟Θ═1⁄4╥Γ╩Θ11/╡┌2▓┐╖╓/023.jpg]

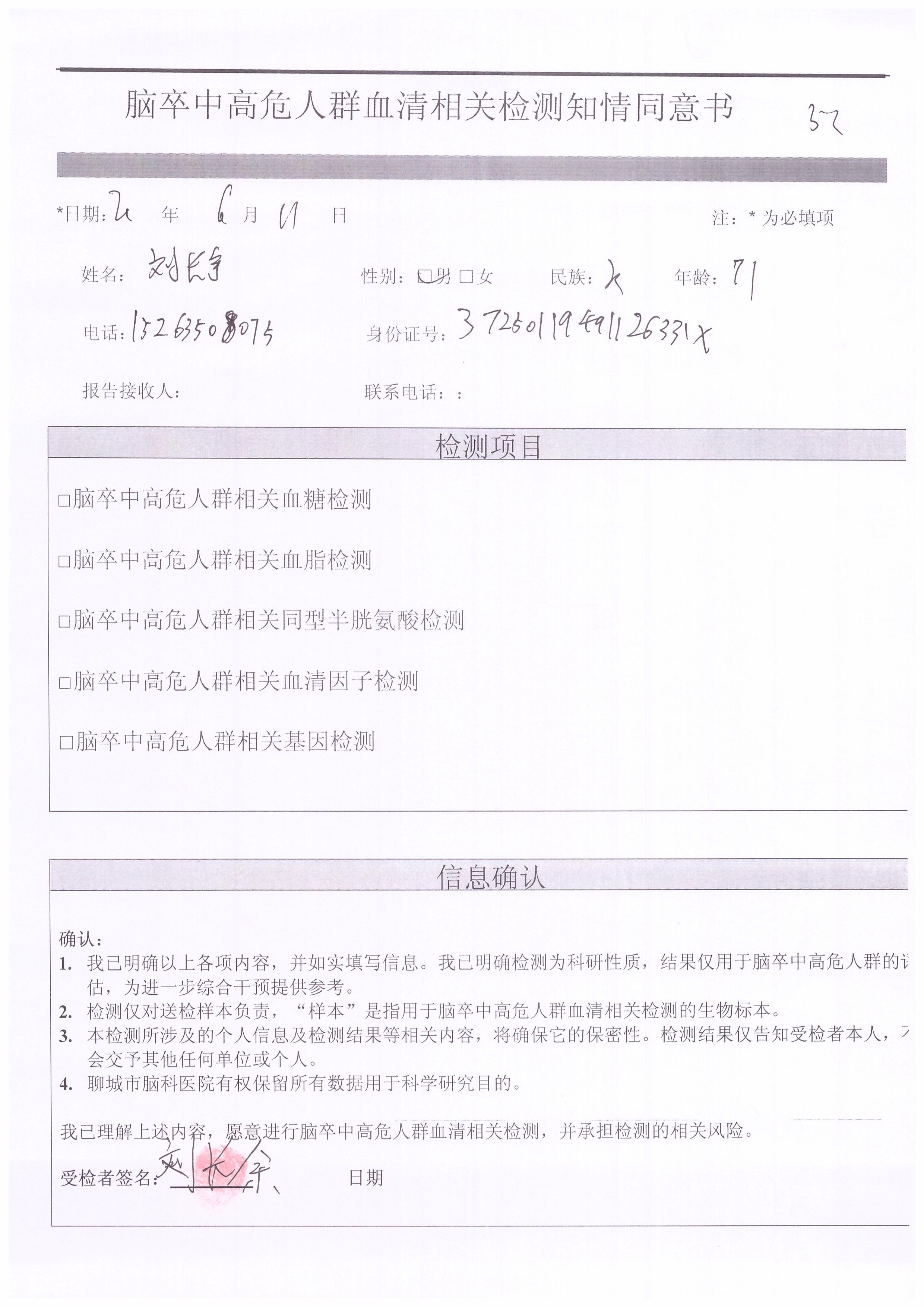

Supplement: Supplementary file 13 — Supplementary file13 (ZIP 28344 KB) [file 10528_2023_10431_MOESM13_ESM.zip › ╓¬╟Θ═1⁄4╥Γ╩Θ11/╡┌2▓┐╖╓/024.jpg]

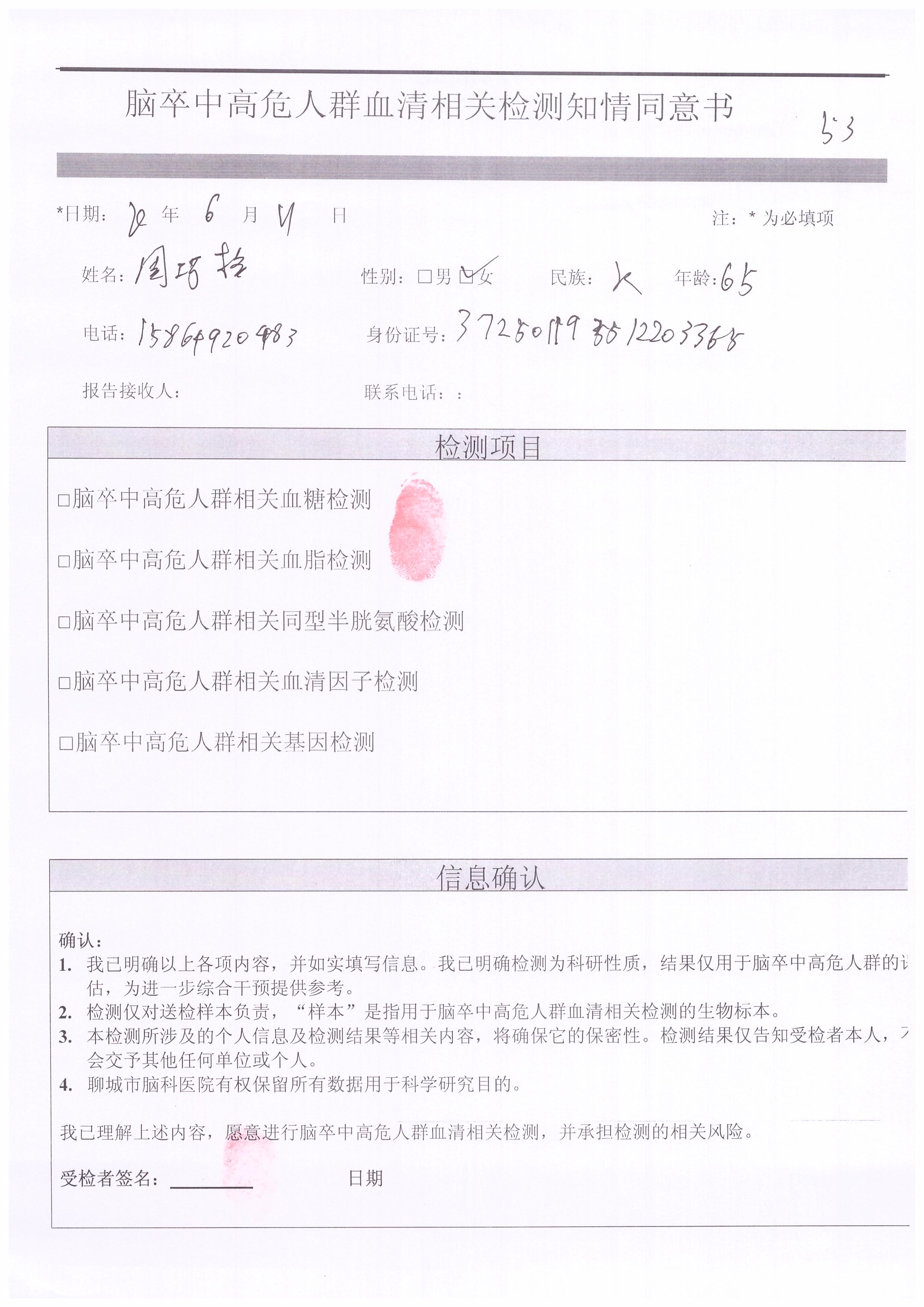

Supplement: Supplementary file 13 — Supplementary file13 (ZIP 28344 KB) [file 10528_2023_10431_MOESM13_ESM.zip › ╓¬╟Θ═1⁄4╥Γ╩Θ11/╡┌2▓┐╖╓/025.jpg]

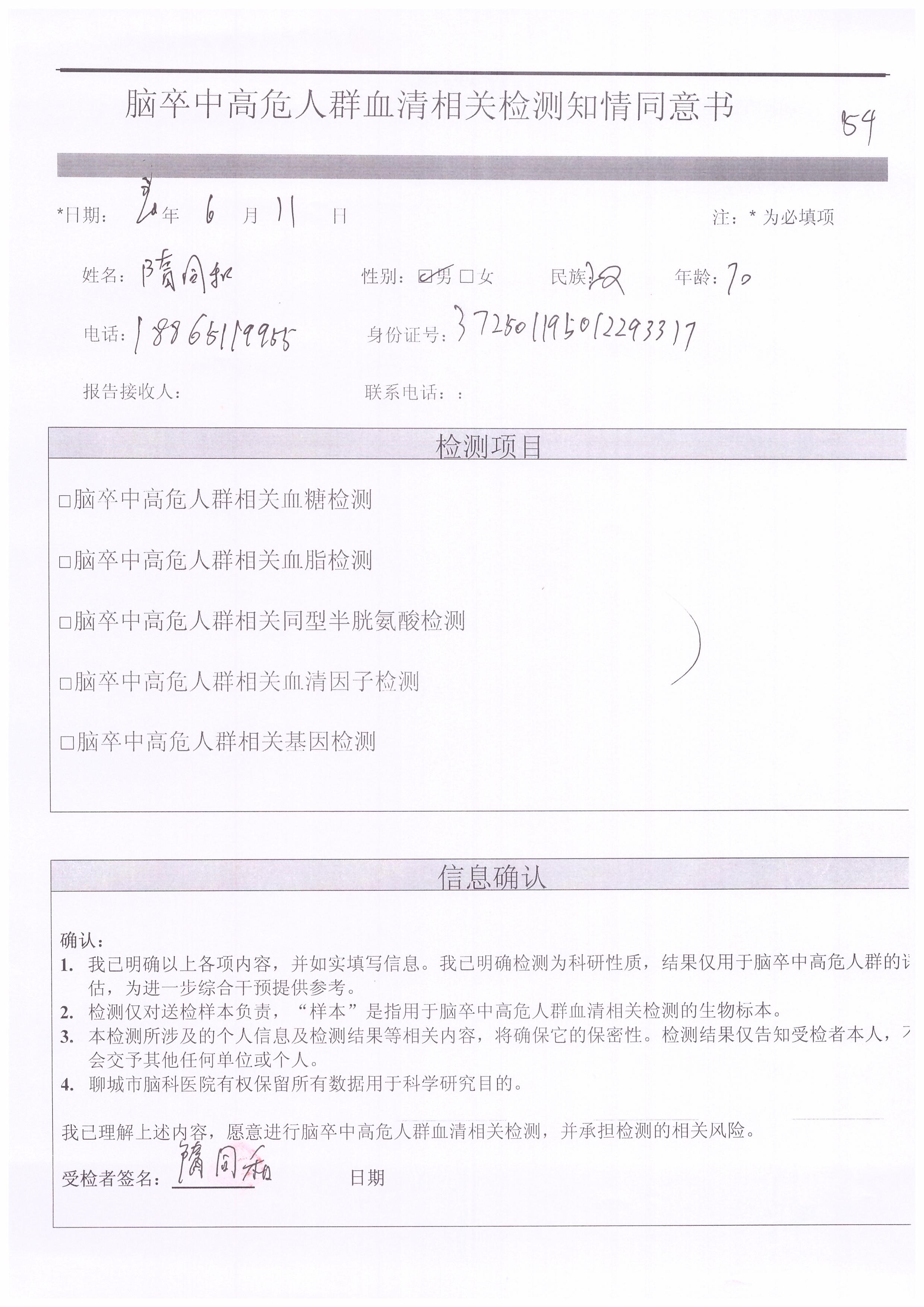

Supplement: Supplementary file 13 — Supplementary file13 (ZIP 28344 KB) [file 10528_2023_10431_MOESM13_ESM.zip › ╓¬╟Θ═1⁄4╥Γ╩Θ11/╡┌2▓┐╖╓/026.jpg]

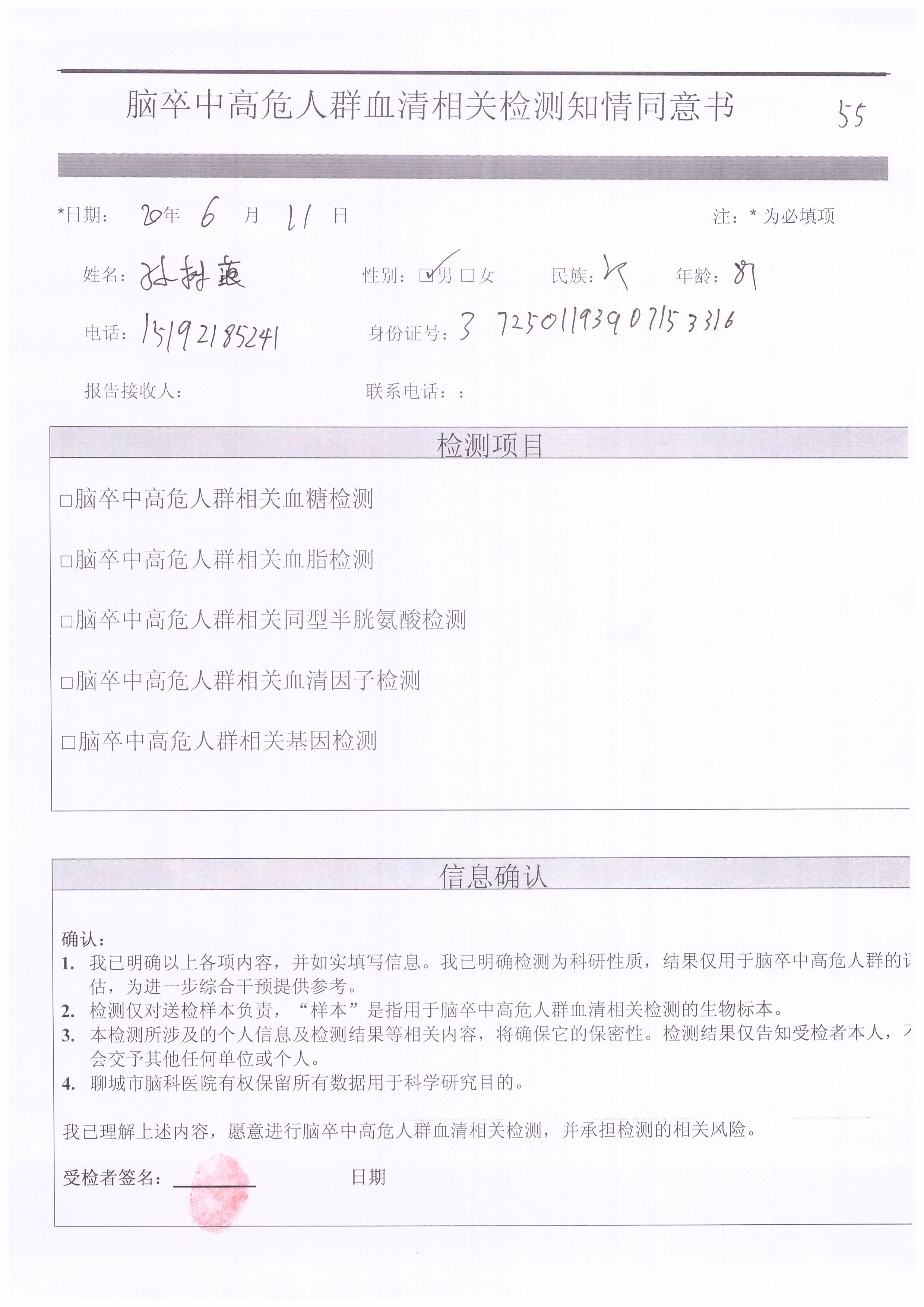

Supplement: Supplementary file 13 — Supplementary file13 (ZIP 28344 KB) [file 10528_2023_10431_MOESM13_ESM.zip › ╓¬╟Θ═1⁄4╥Γ╩Θ11/╡┌2▓┐╖╓/027.jpg]

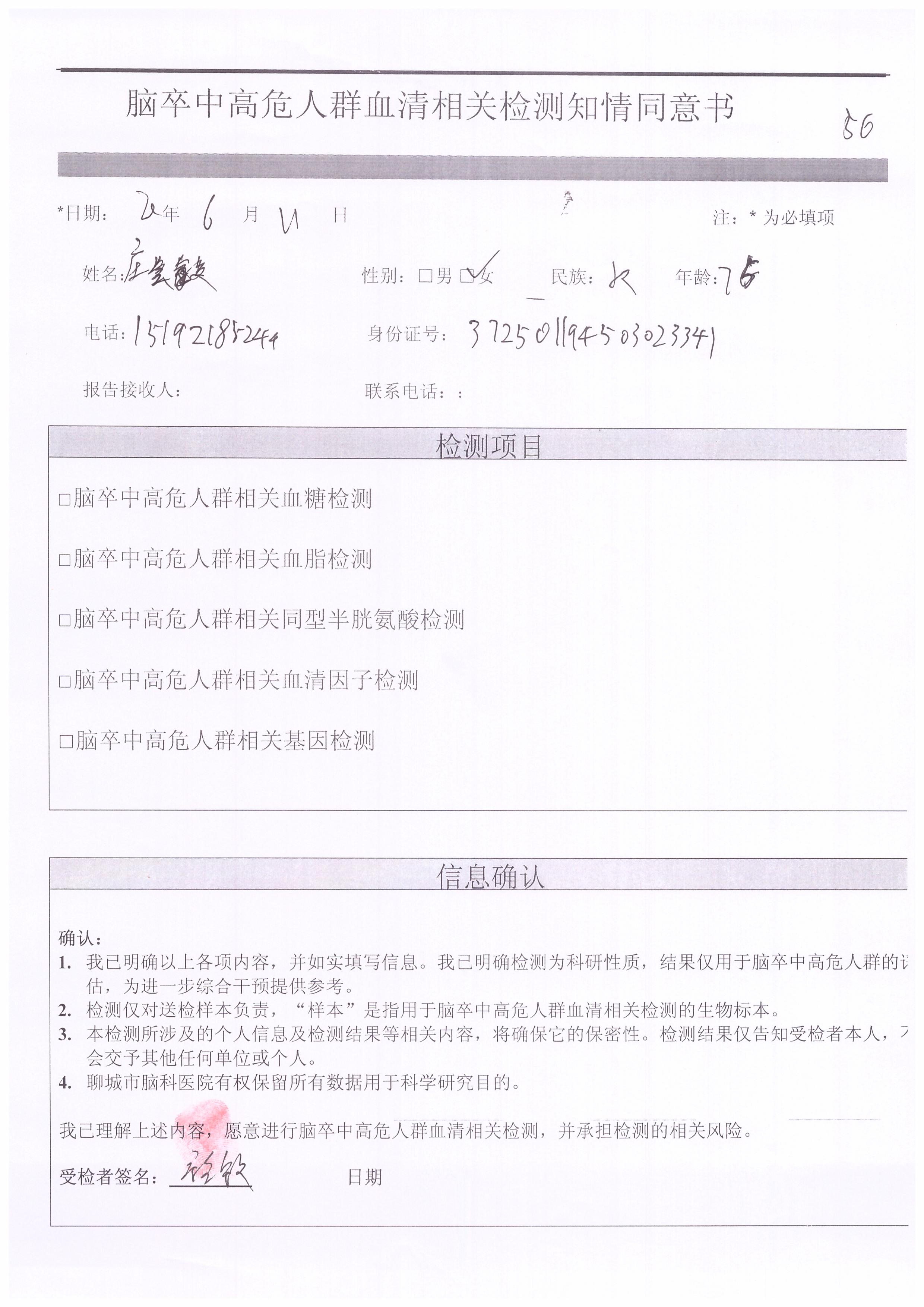

Supplement: Supplementary file 13 — Supplementary file13 (ZIP 28344 KB) [file 10528_2023_10431_MOESM13_ESM.zip › ╓¬╟Θ═1⁄4╥Γ╩Θ11/╡┌2▓┐╖╓/028.jpg]

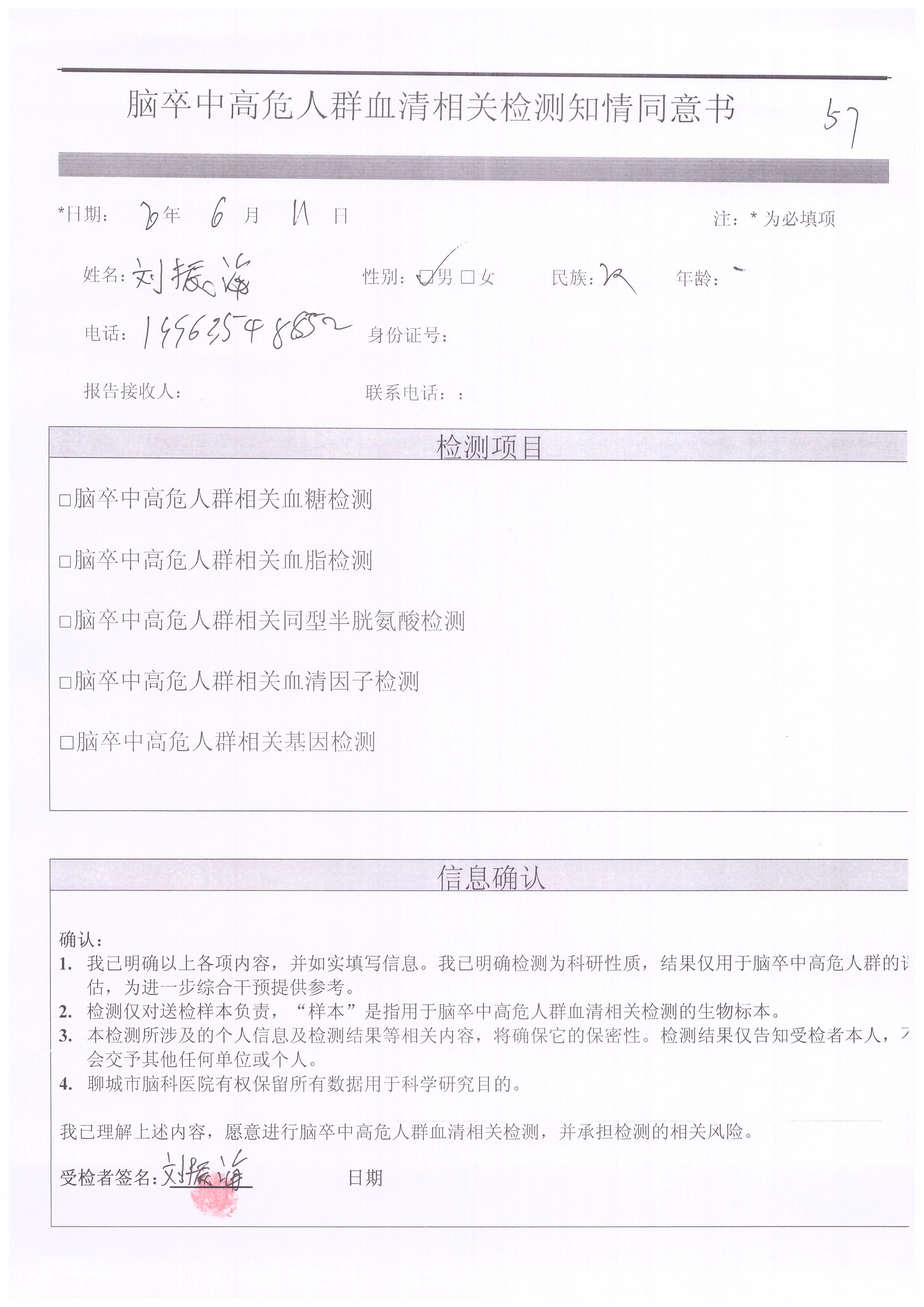

Supplement: Supplementary file 13 — Supplementary file13 (ZIP 28344 KB) [file 10528_2023_10431_MOESM13_ESM.zip › ╓¬╟Θ═1⁄4╥Γ╩Θ11/╡┌2▓┐╖╓/029.jpg]

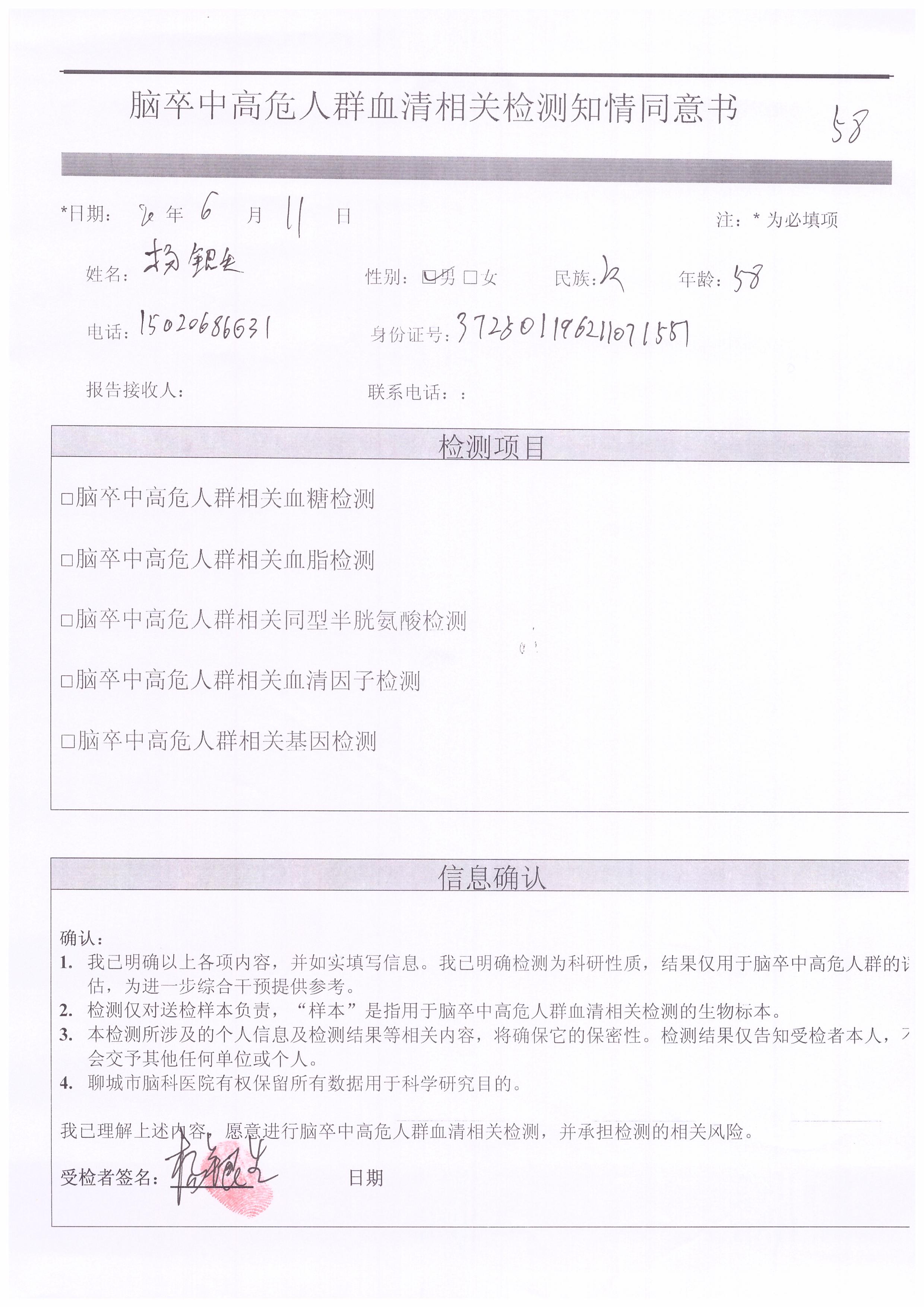

Supplement: Supplementary file 13 — Supplementary file13 (ZIP 28344 KB) [file 10528_2023_10431_MOESM13_ESM.zip › ╓¬╟Θ═1⁄4╥Γ╩Θ11/╡┌2▓┐╖╓/030.jpg]

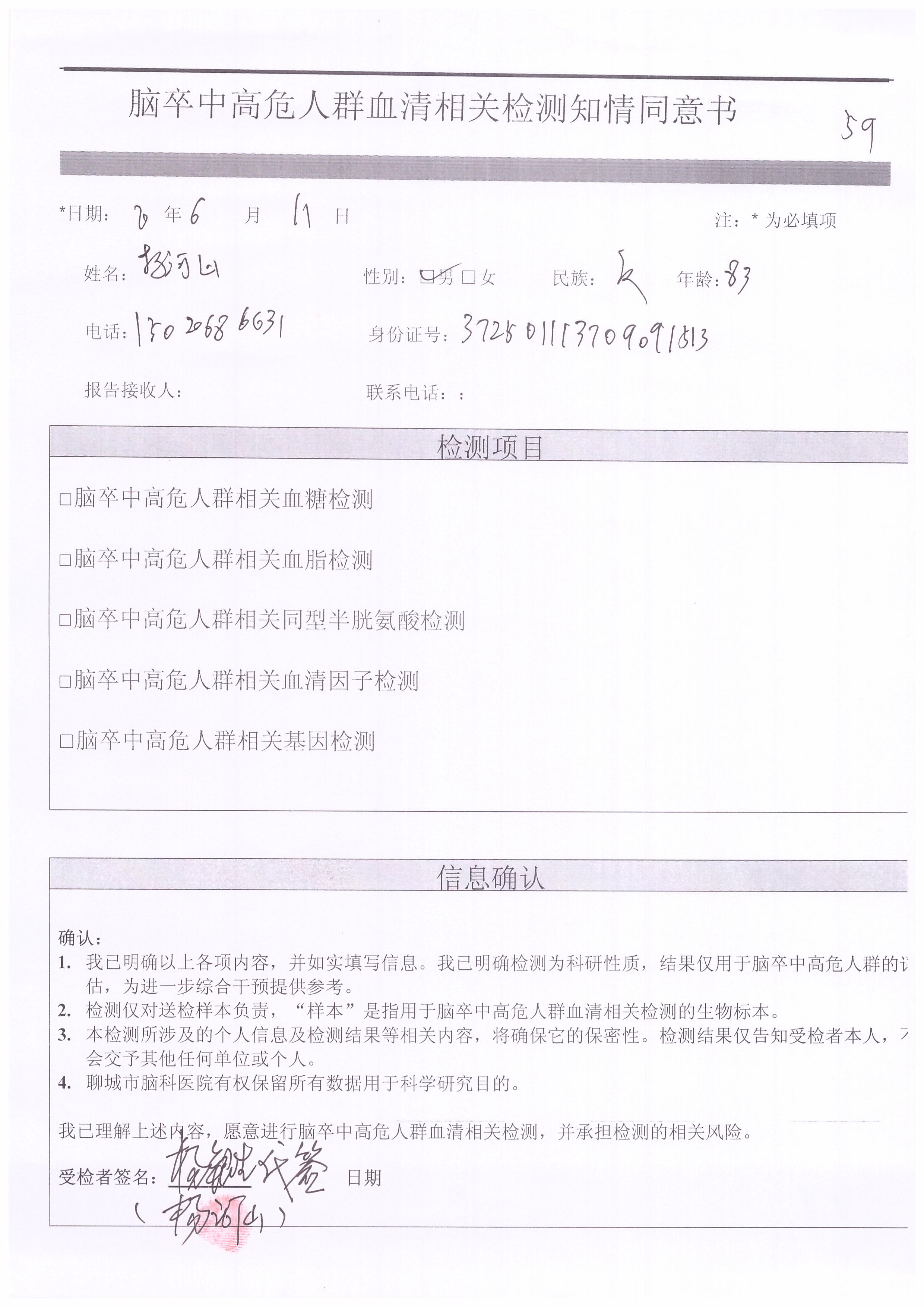

Supplement: Supplementary file 13 — Supplementary file13 (ZIP 28344 KB) [file 10528_2023_10431_MOESM13_ESM.zip › ╓¬╟Θ═1⁄4╥Γ╩Θ11/╡┌2▓┐╖╓/031.jpg]

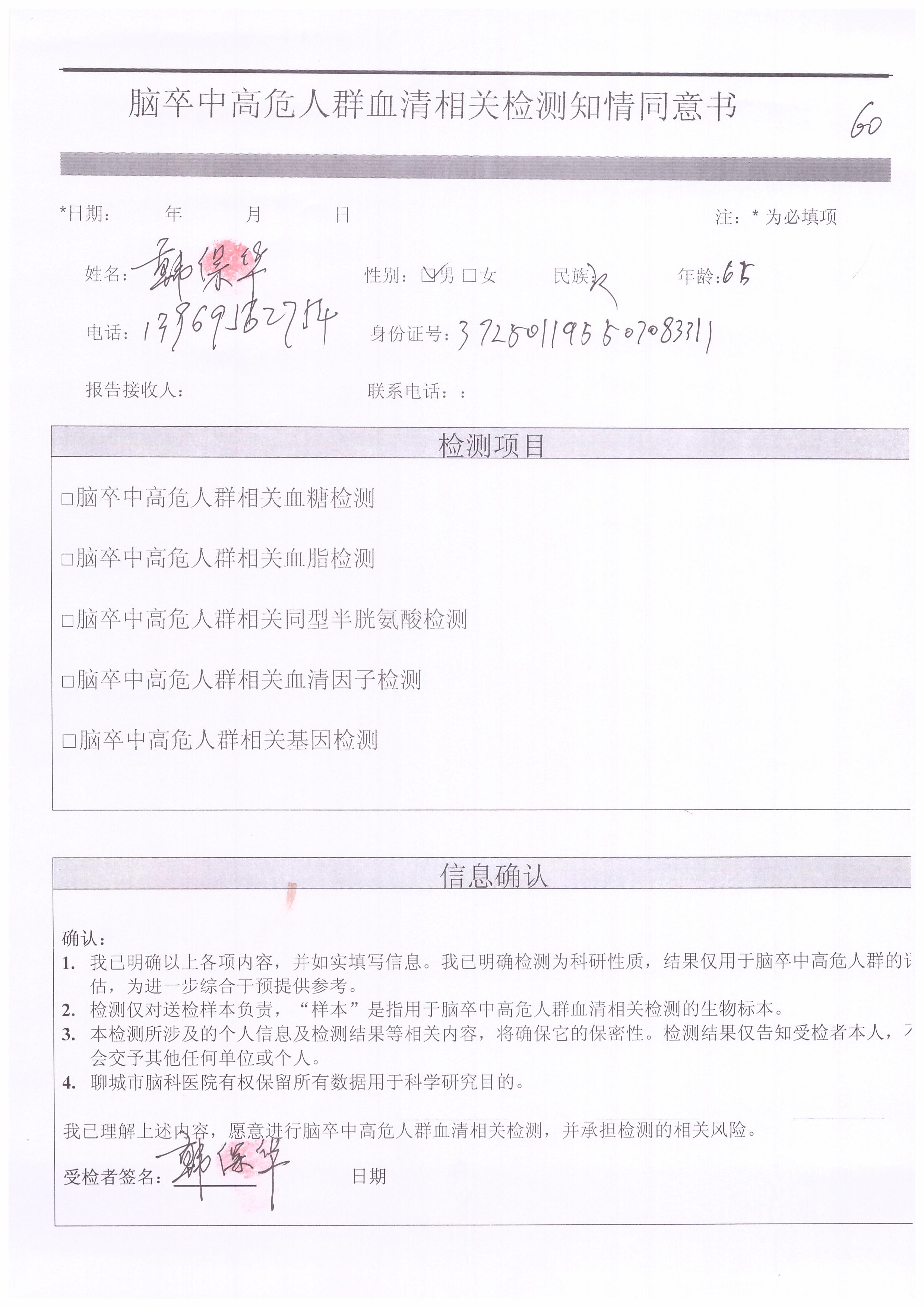

Supplement: Supplementary file 13 — Supplementary file13 (ZIP 28344 KB) [file 10528_2023_10431_MOESM13_ESM.zip › ╓¬╟Θ═1⁄4╥Γ╩Θ11/╡┌2▓┐╖╓/032.jpg]

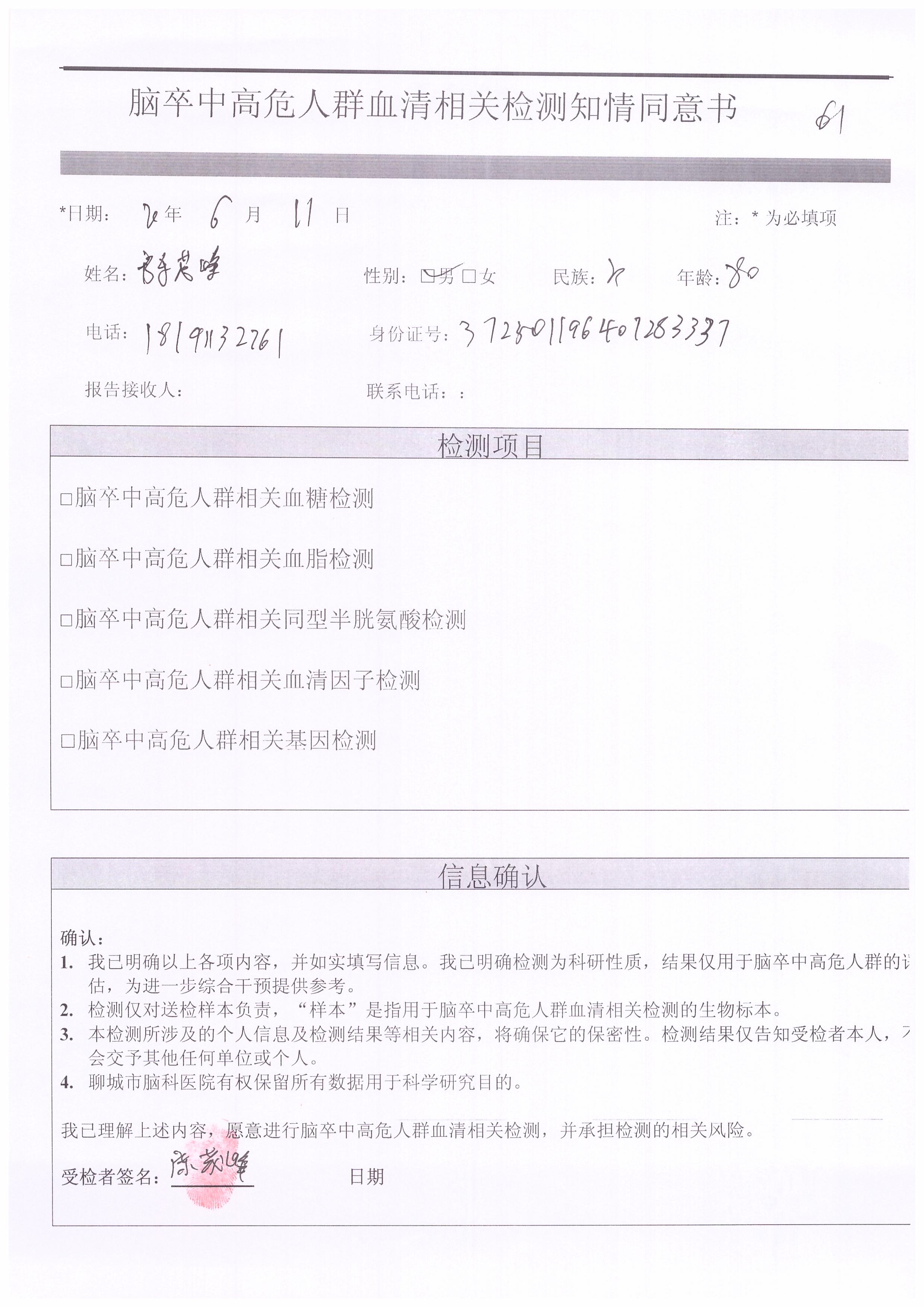

Supplement: Supplementary file 13 — Supplementary file13 (ZIP 28344 KB) [file 10528_2023_10431_MOESM13_ESM.zip › ╓¬╟Θ═1⁄4╥Γ╩Θ11/╡┌2▓┐╖╓/033.jpg]

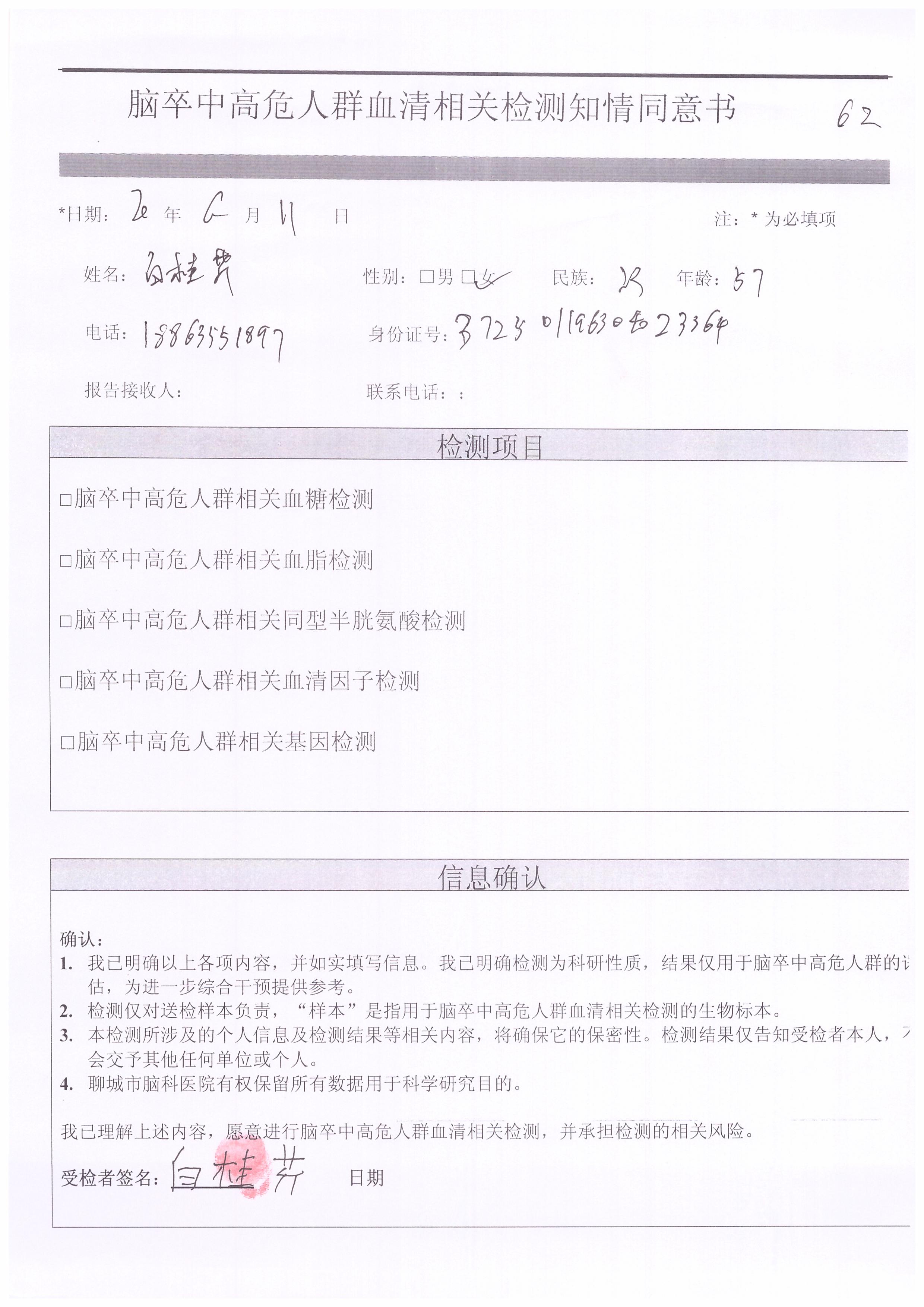

Supplement: Supplementary file 13 — Supplementary file13 (ZIP 28344 KB) [file 10528_2023_10431_MOESM13_ESM.zip › ╓¬╟Θ═1⁄4╥Γ╩Θ11/╡┌2▓┐╖╓/034.jpg]

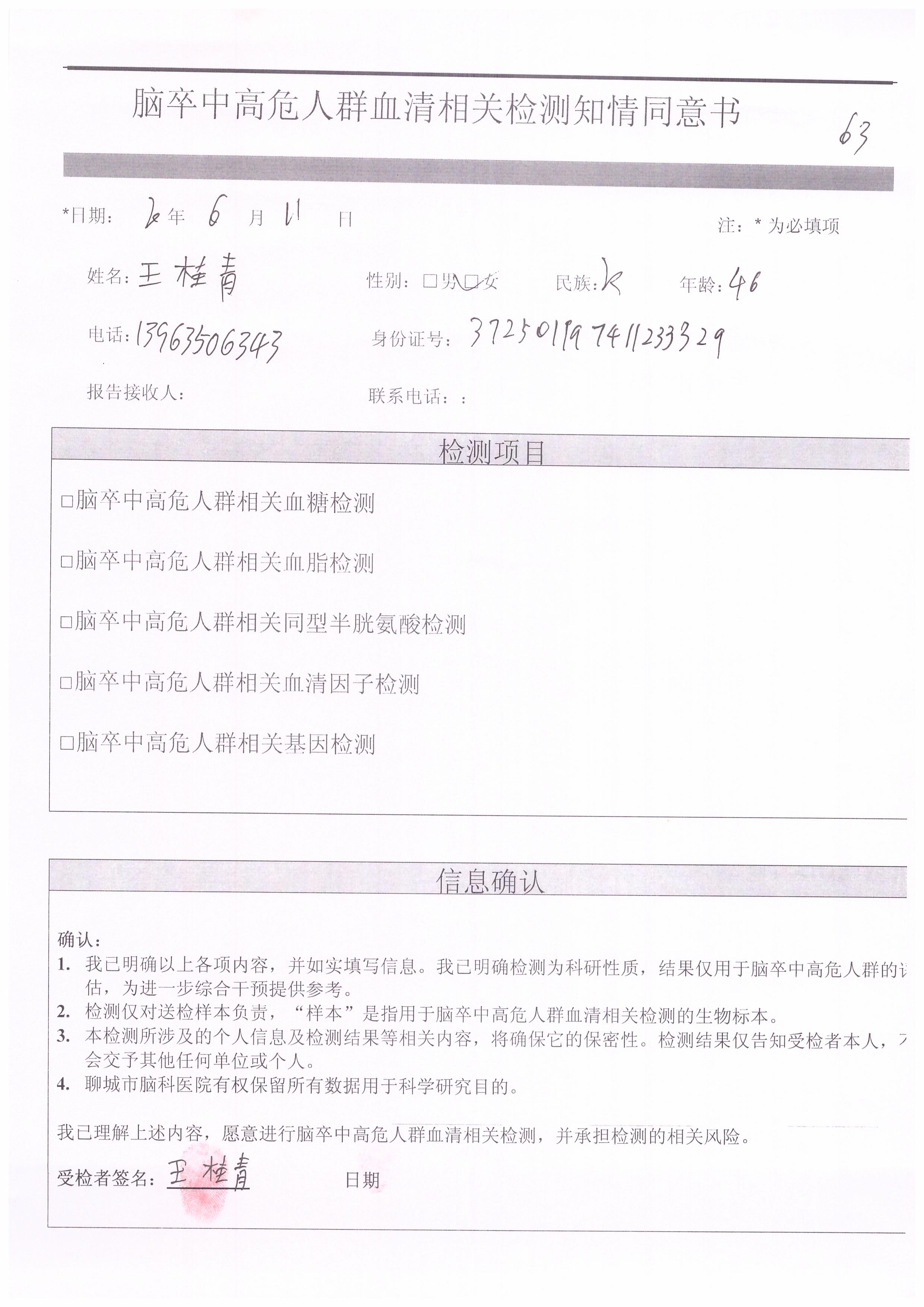

Supplement: Supplementary file 13 — Supplementary file13 (ZIP 28344 KB) [file 10528_2023_10431_MOESM13_ESM.zip › ╓¬╟Θ═1⁄4╥Γ╩Θ11/╡┌2▓┐╖╓/035.jpg]

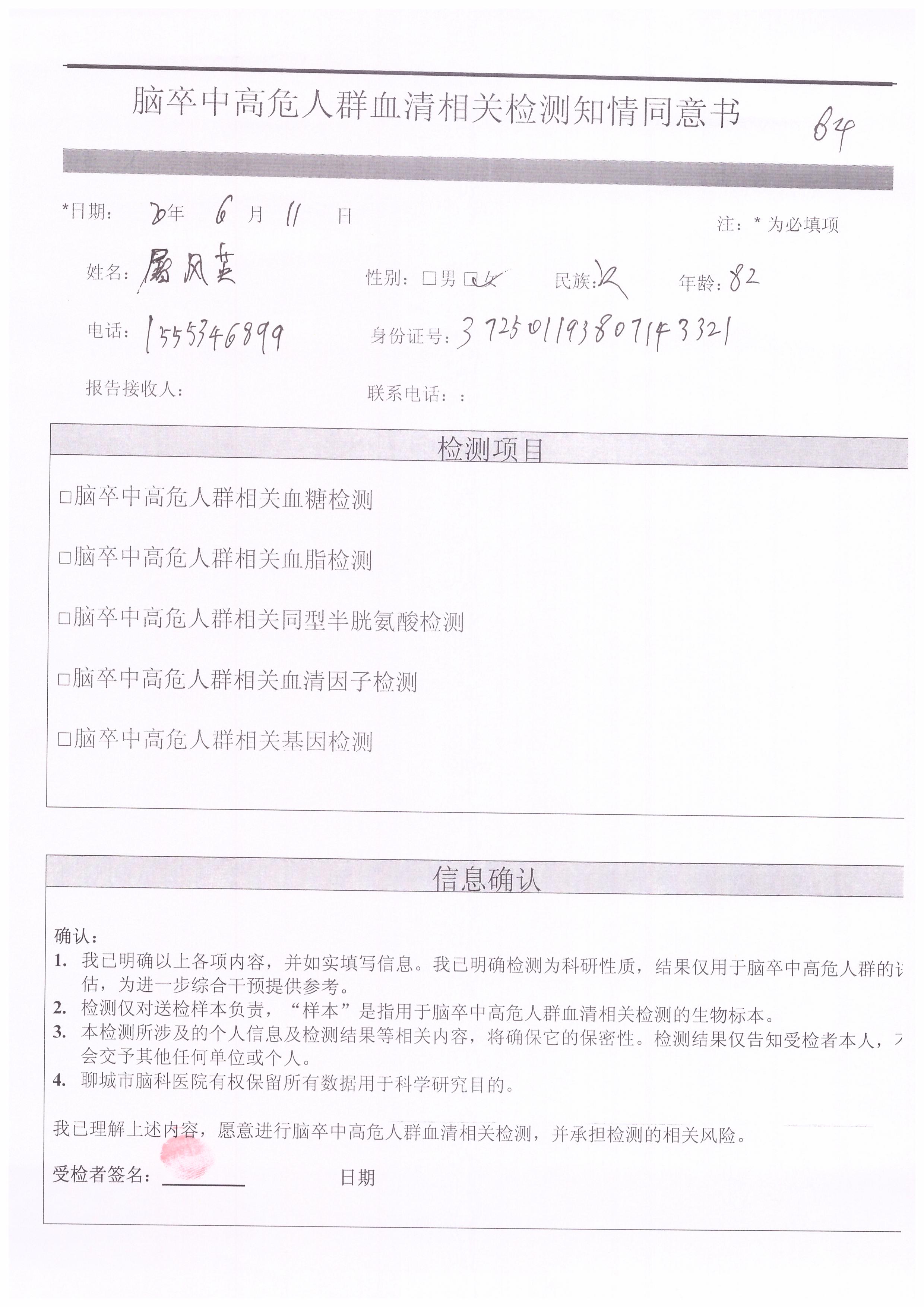

Supplement: Supplementary file 13 — Supplementary file13 (ZIP 28344 KB) [file 10528_2023_10431_MOESM13_ESM.zip › ╓¬╟Θ═1⁄4╥Γ╩Θ11/╡┌2▓┐╖╓/036.jpg]

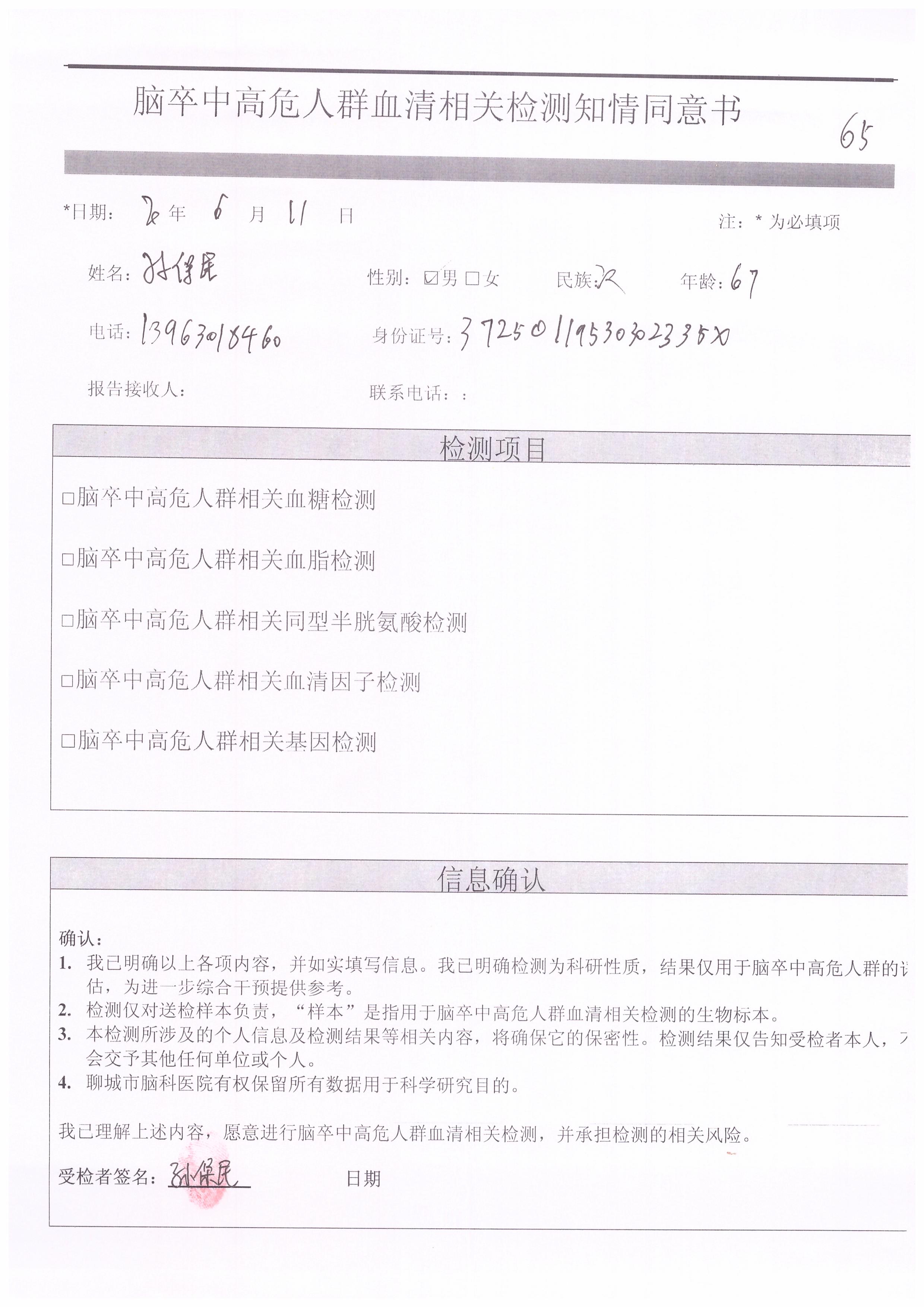

Supplement: Supplementary file 13 — Supplementary file13 (ZIP 28344 KB) [file 10528_2023_10431_MOESM13_ESM.zip › ╓¬╟Θ═1⁄4╥Γ╩Θ11/╡┌2▓┐╖╓/037.jpg]

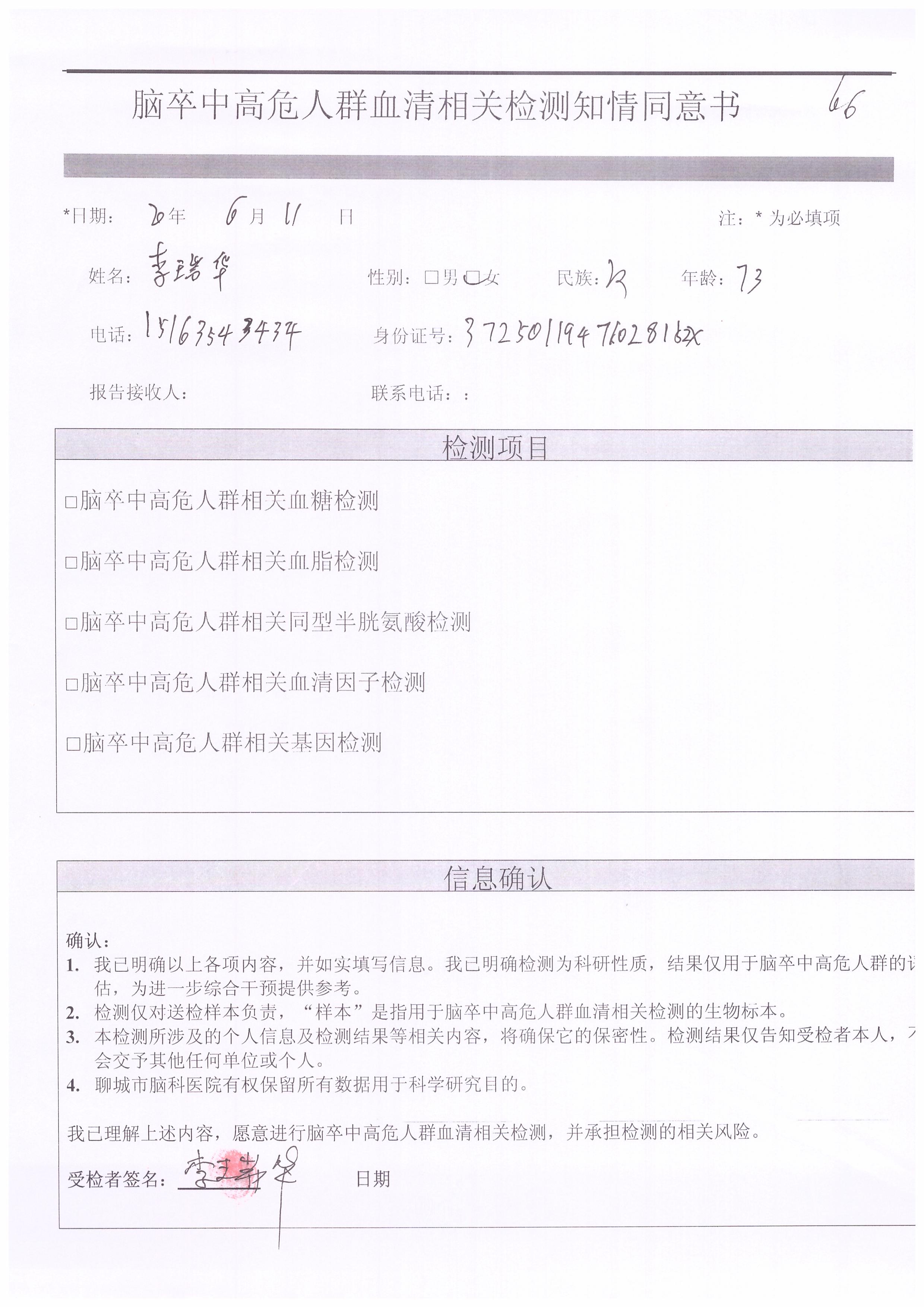

Supplement: Supplementary file 13 — Supplementary file13 (ZIP 28344 KB) [file 10528_2023_10431_MOESM13_ESM.zip › ╓¬╟Θ═1⁄4╥Γ╩Θ11/╡┌2▓┐╖╓/038.jpg]

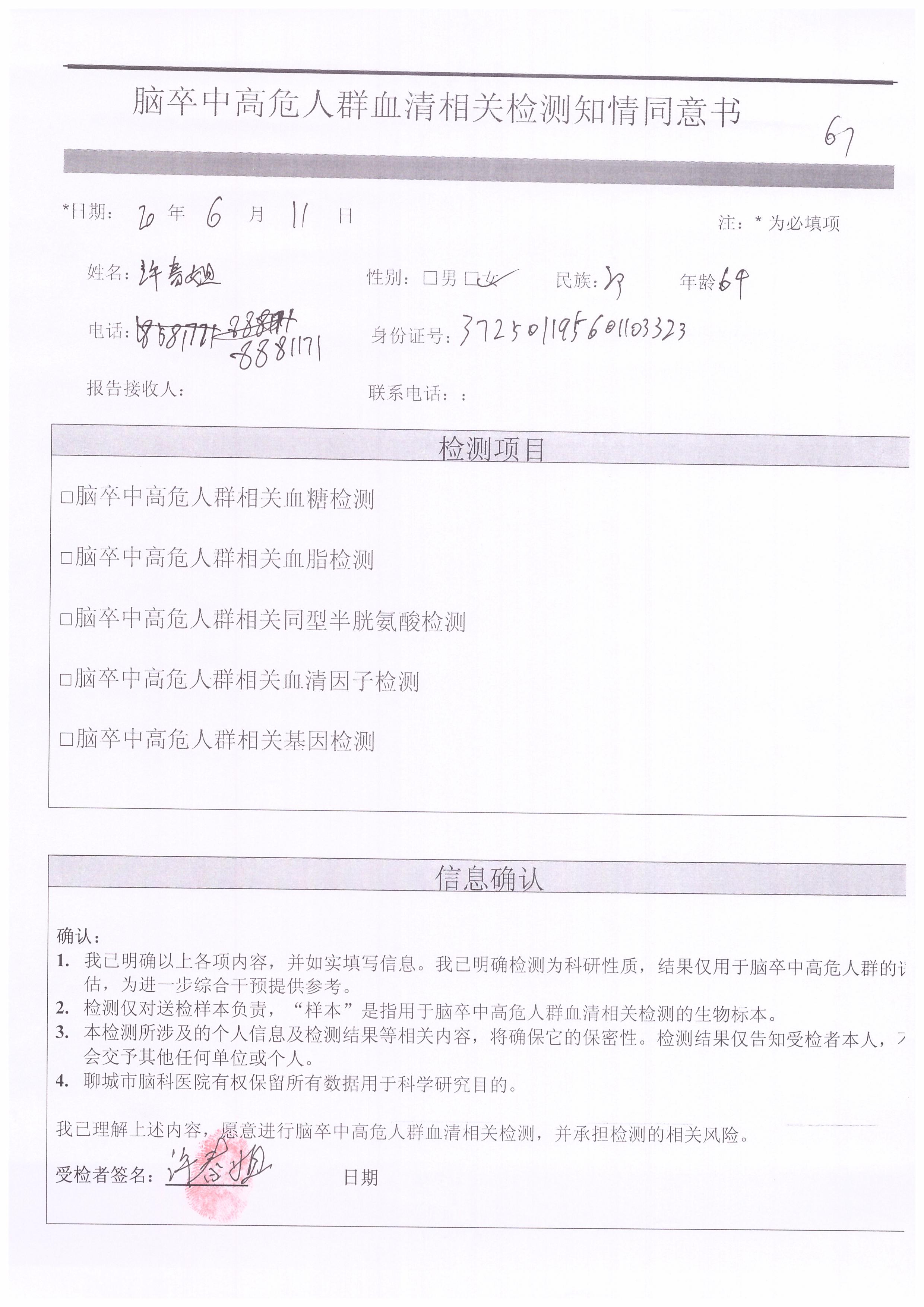

Supplement: Supplementary file 13 — Supplementary file13 (ZIP 28344 KB) [file 10528_2023_10431_MOESM13_ESM.zip › ╓¬╟Θ═1⁄4╥Γ╩Θ11/╡┌2▓┐╖╓/039.jpg]

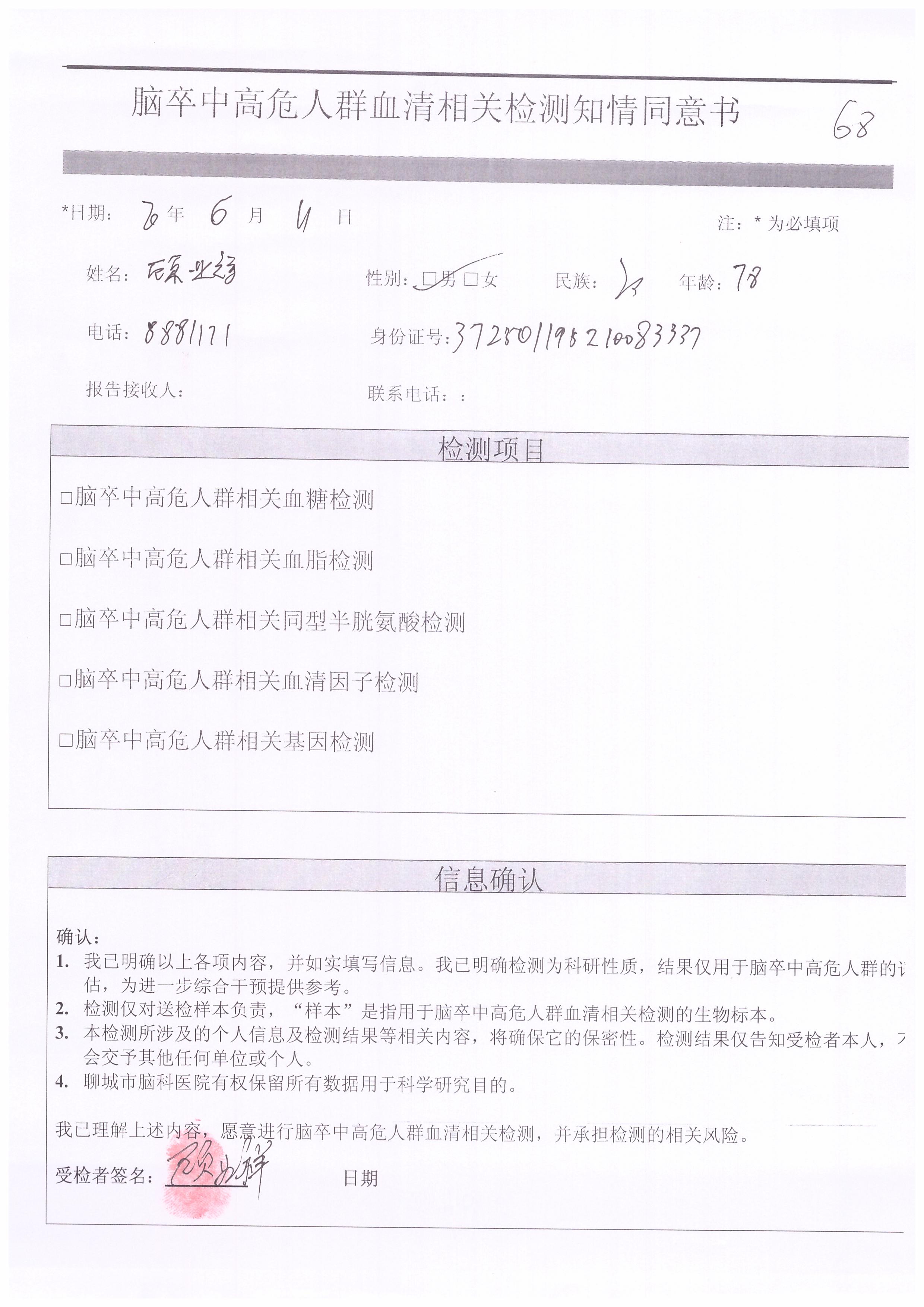

Supplement: Supplementary file 13 — Supplementary file13 (ZIP 28344 KB) [file 10528_2023_10431_MOESM13_ESM.zip › ╓¬╟Θ═1⁄4╥Γ╩Θ11/╡┌2▓┐╖╓/040.jpg]

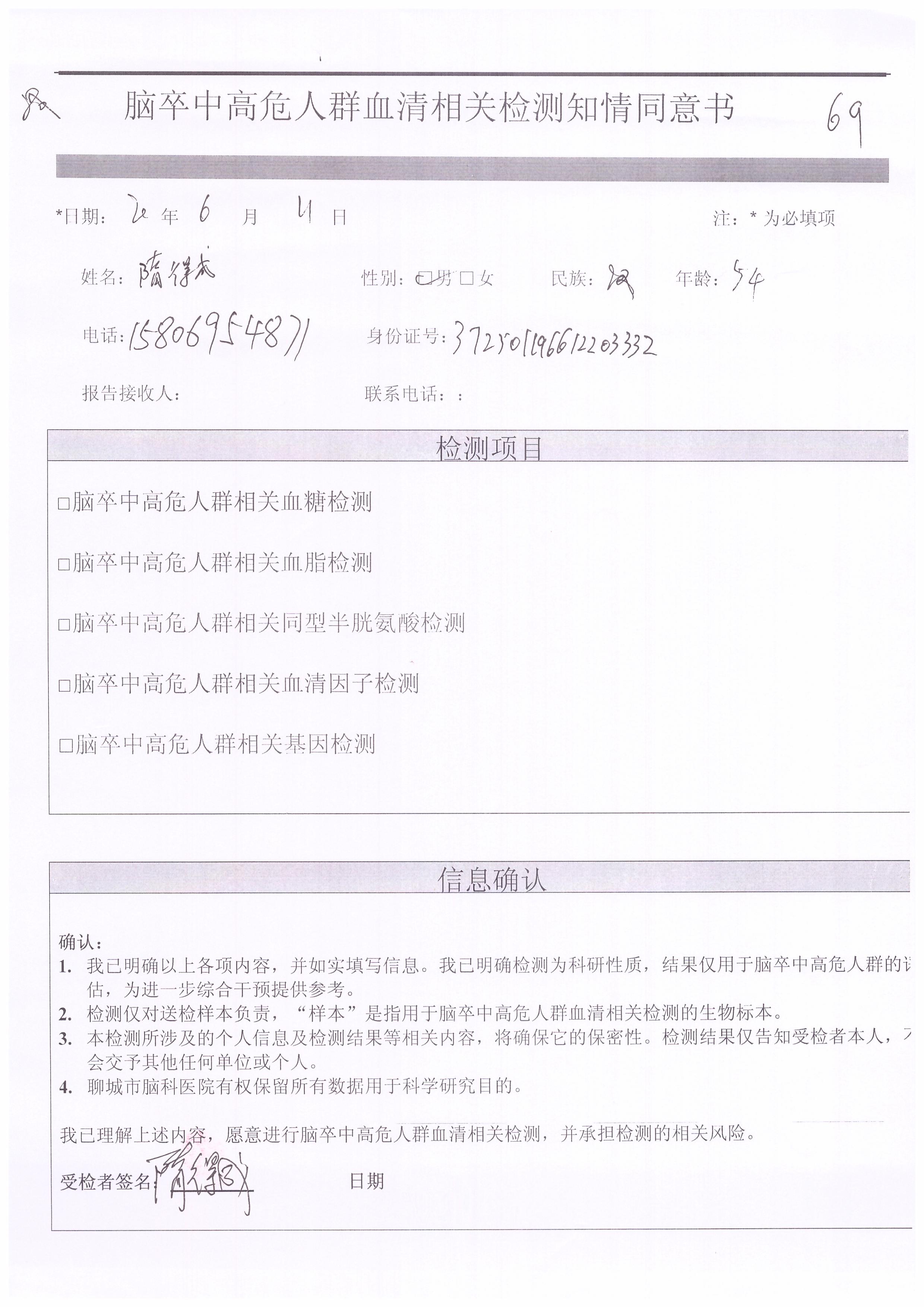

Supplement: Supplementary file 13 — Supplementary file13 (ZIP 28344 KB) [file 10528_2023_10431_MOESM13_ESM.zip › ╓¬╟Θ═1⁄4╥Γ╩Θ11/╡┌2▓┐╖╓/041.jpg]

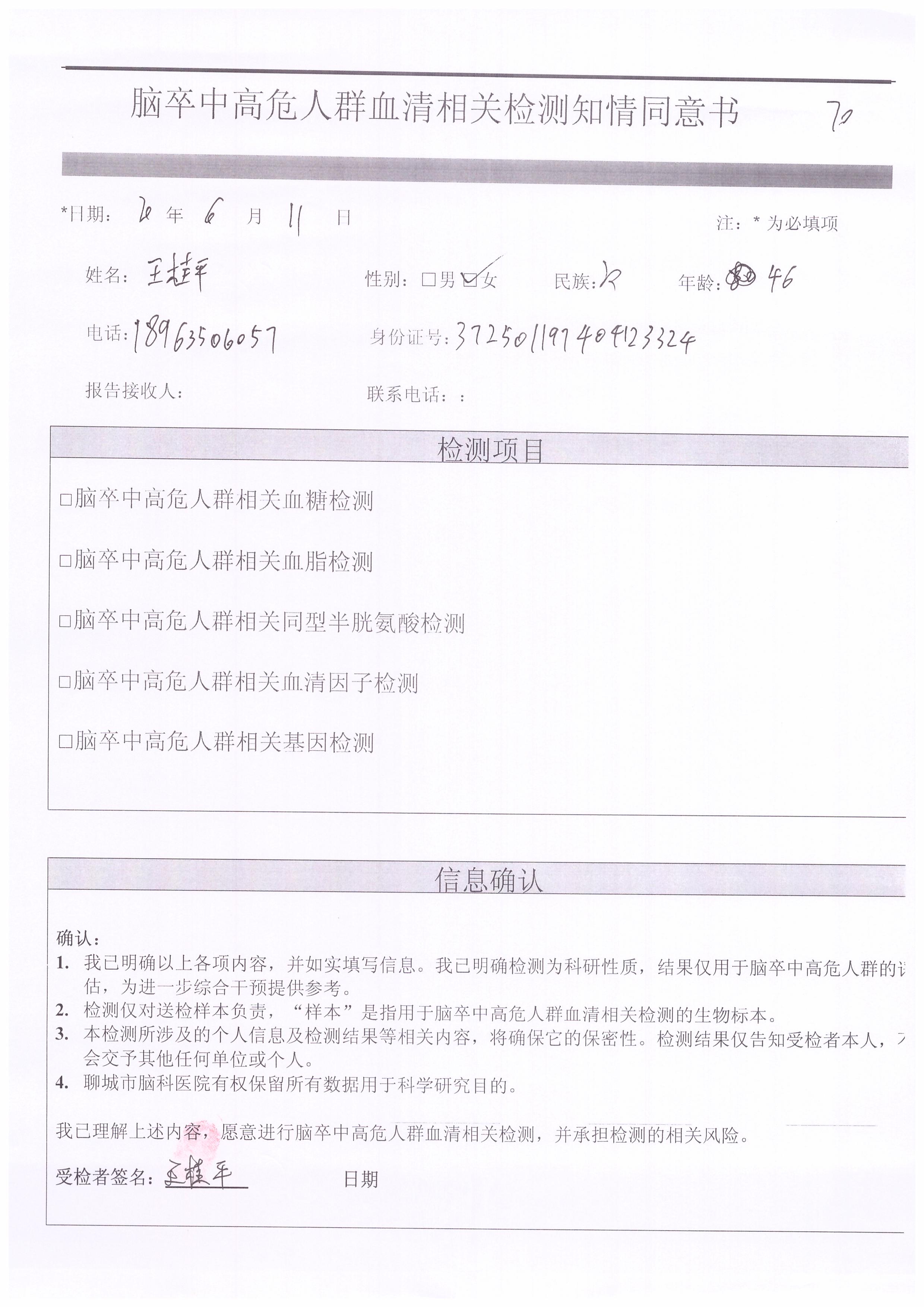

Supplement: Supplementary file 13 — Supplementary file13 (ZIP 28344 KB) [file 10528_2023_10431_MOESM13_ESM.zip › ╓¬╟Θ═1⁄4╥Γ╩Θ11/╡┌2▓┐╖╓/042.jpg]

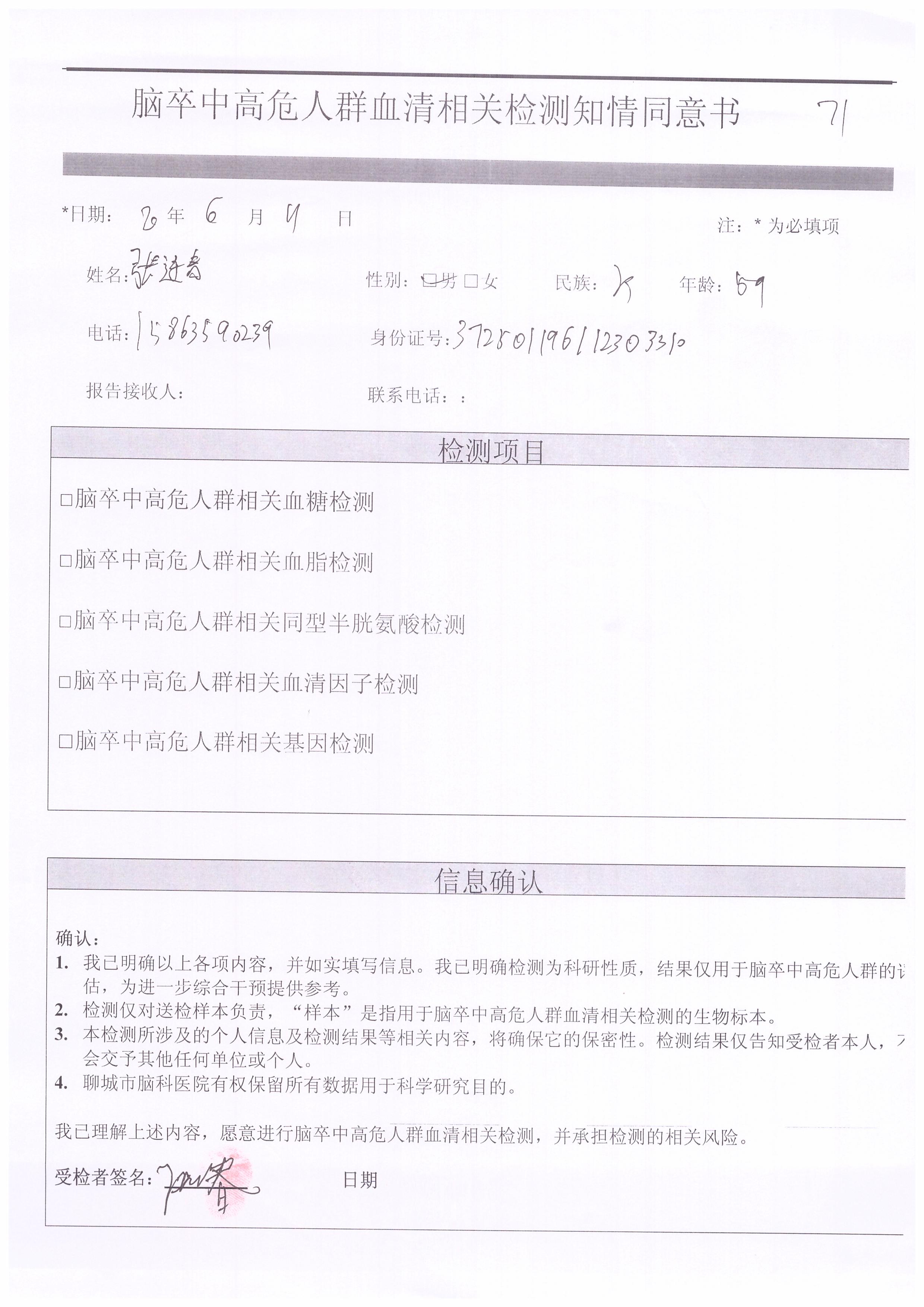

Supplement: Supplementary file 13 — Supplementary file13 (ZIP 28344 KB) [file 10528_2023_10431_MOESM13_ESM.zip › ╓¬╟Θ═1⁄4╥Γ╩Θ11/╡┌2▓┐╖╓/043.jpg]

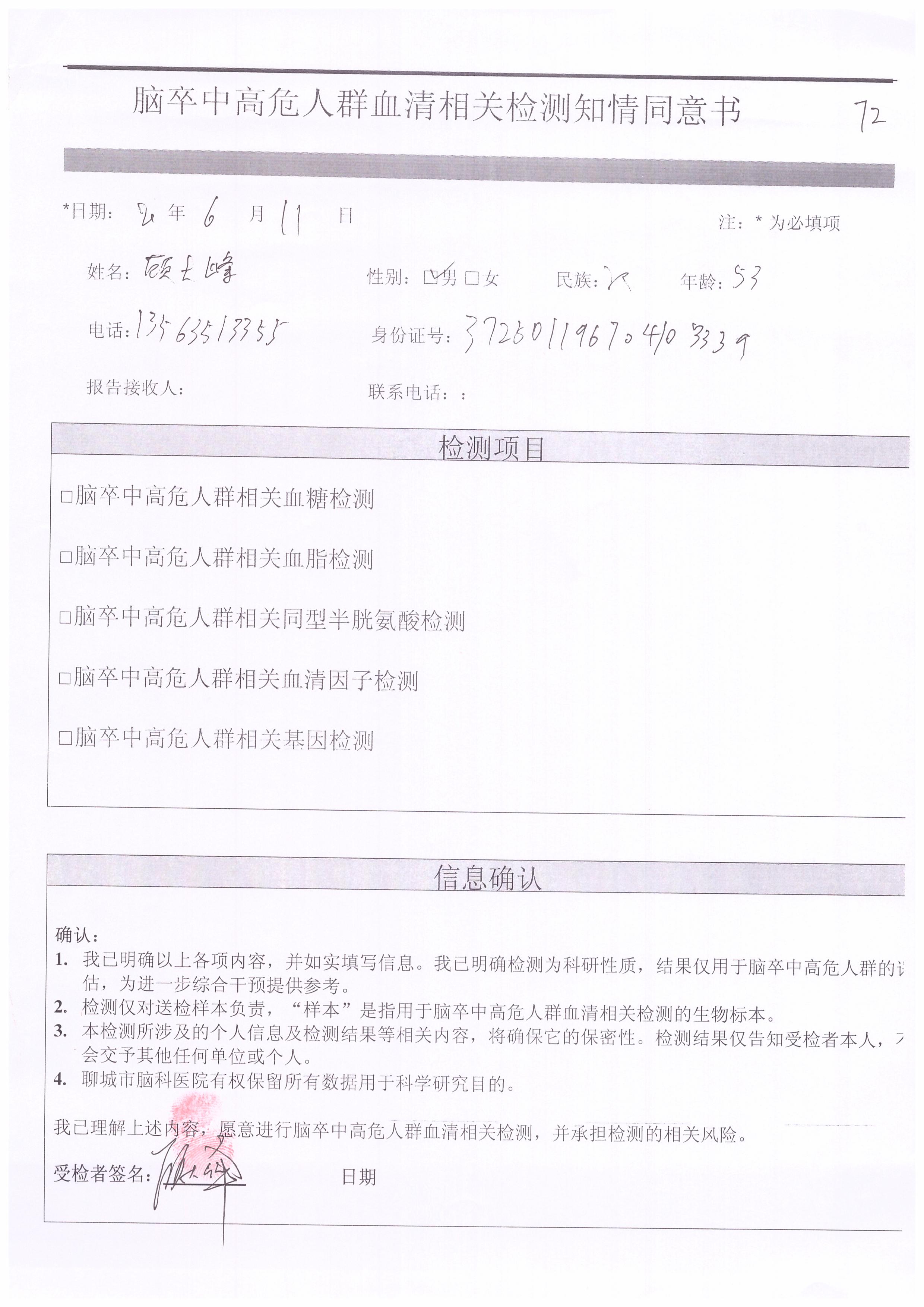

Supplement: Supplementary file 13 — Supplementary file13 (ZIP 28344 KB) [file 10528_2023_10431_MOESM13_ESM.zip › ╓¬╟Θ═1⁄4╥Γ╩Θ11/╡┌2▓┐╖╓/044.jpg]

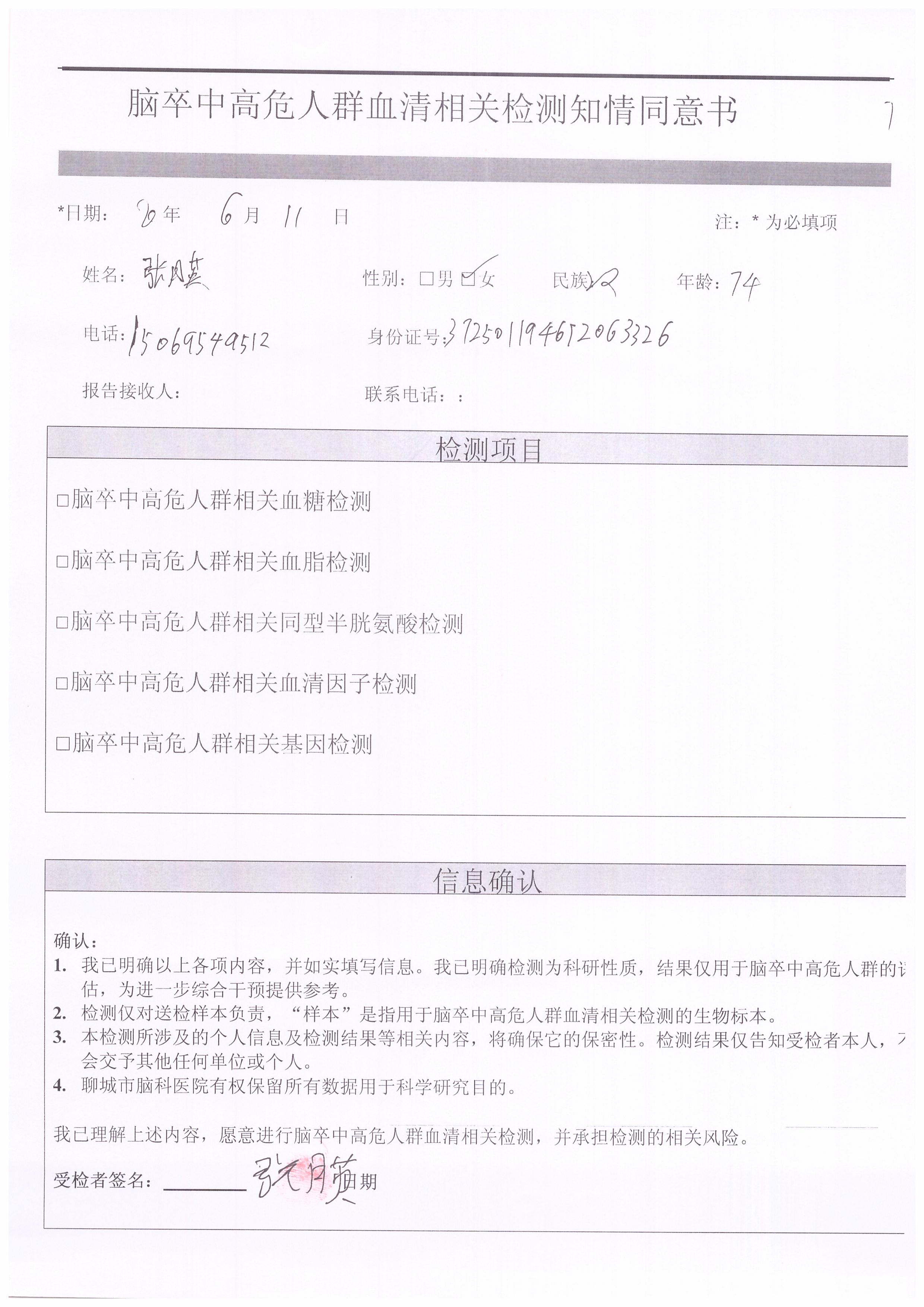

Supplement: Supplementary file 13 — Supplementary file13 (ZIP 28344 KB) [file 10528_2023_10431_MOESM13_ESM.zip › ╓¬╟Θ═1⁄4╥Γ╩Θ11/╡┌╥╗▓┐╖╓/007.jpg]

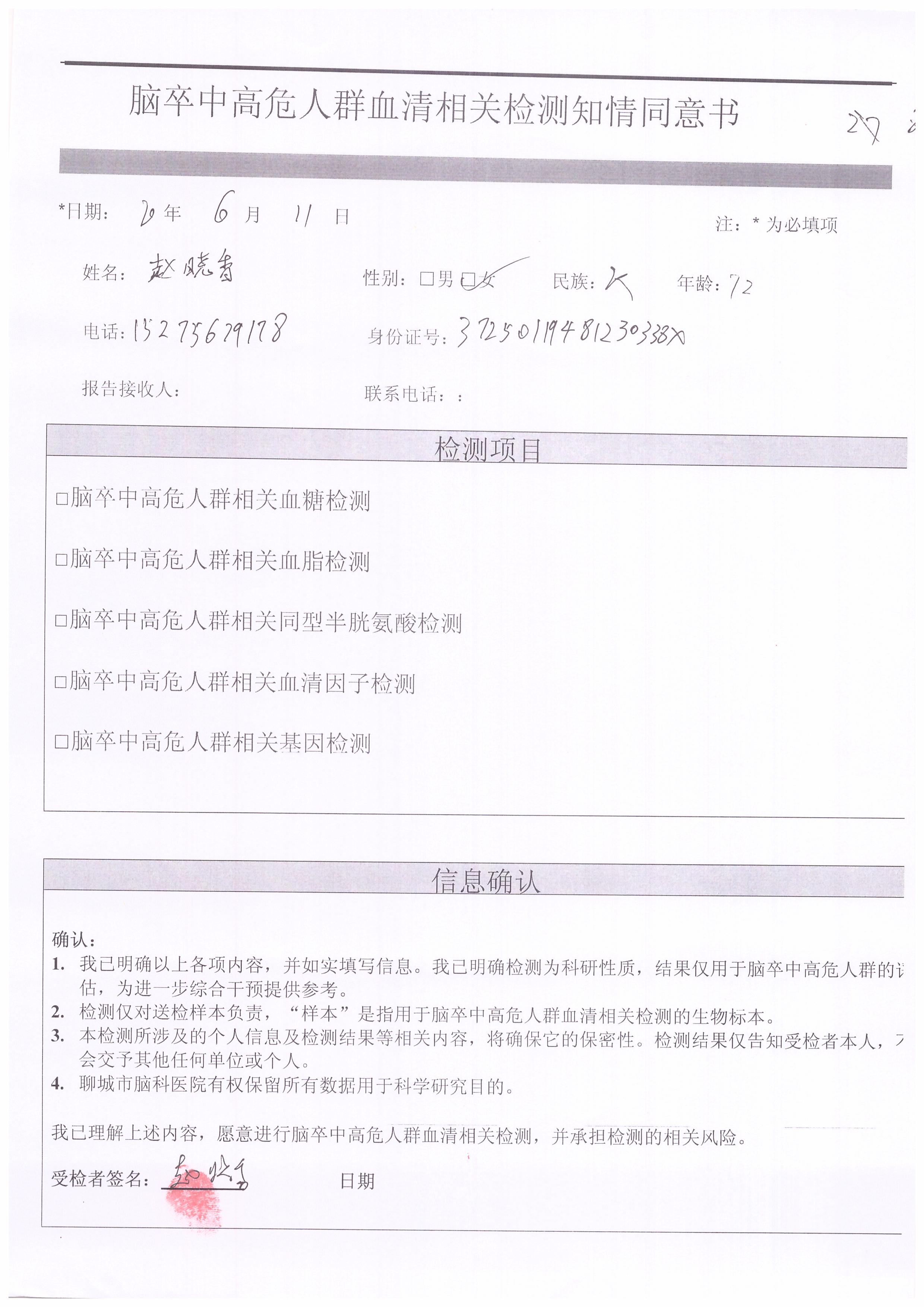

Supplement: Supplementary file 13 — Supplementary file13 (ZIP 28344 KB) [file 10528_2023_10431_MOESM13_ESM.zip › ╓¬╟Θ═1⁄4╥Γ╩Θ11/╡┌╥╗▓┐╖╓/008.jpg]

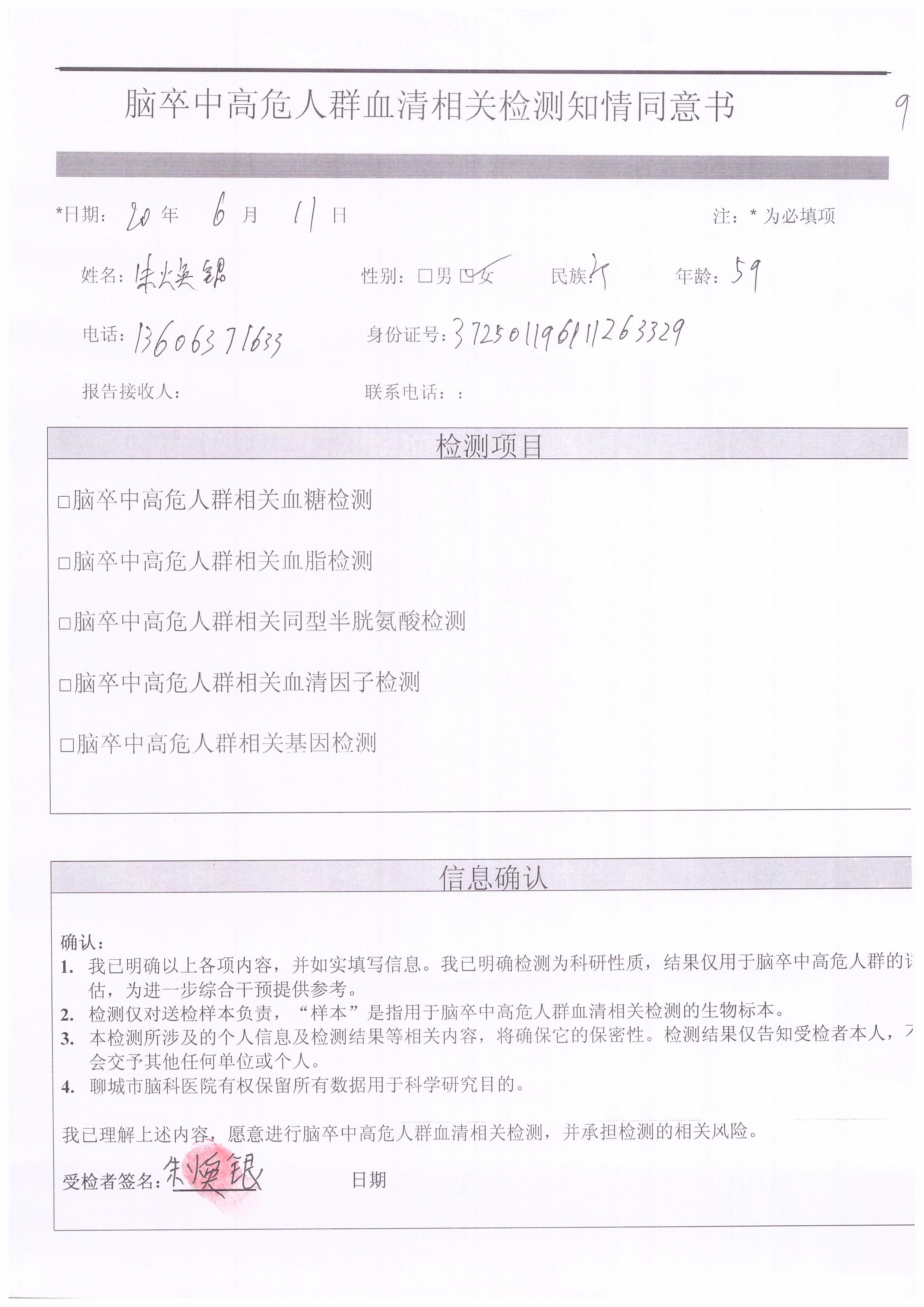

Supplement: Supplementary file 13 — Supplementary file13 (ZIP 28344 KB) [file 10528_2023_10431_MOESM13_ESM.zip › ╓¬╟Θ═1⁄4╥Γ╩Θ11/╡┌╥╗▓┐╖╓/009.jpg]

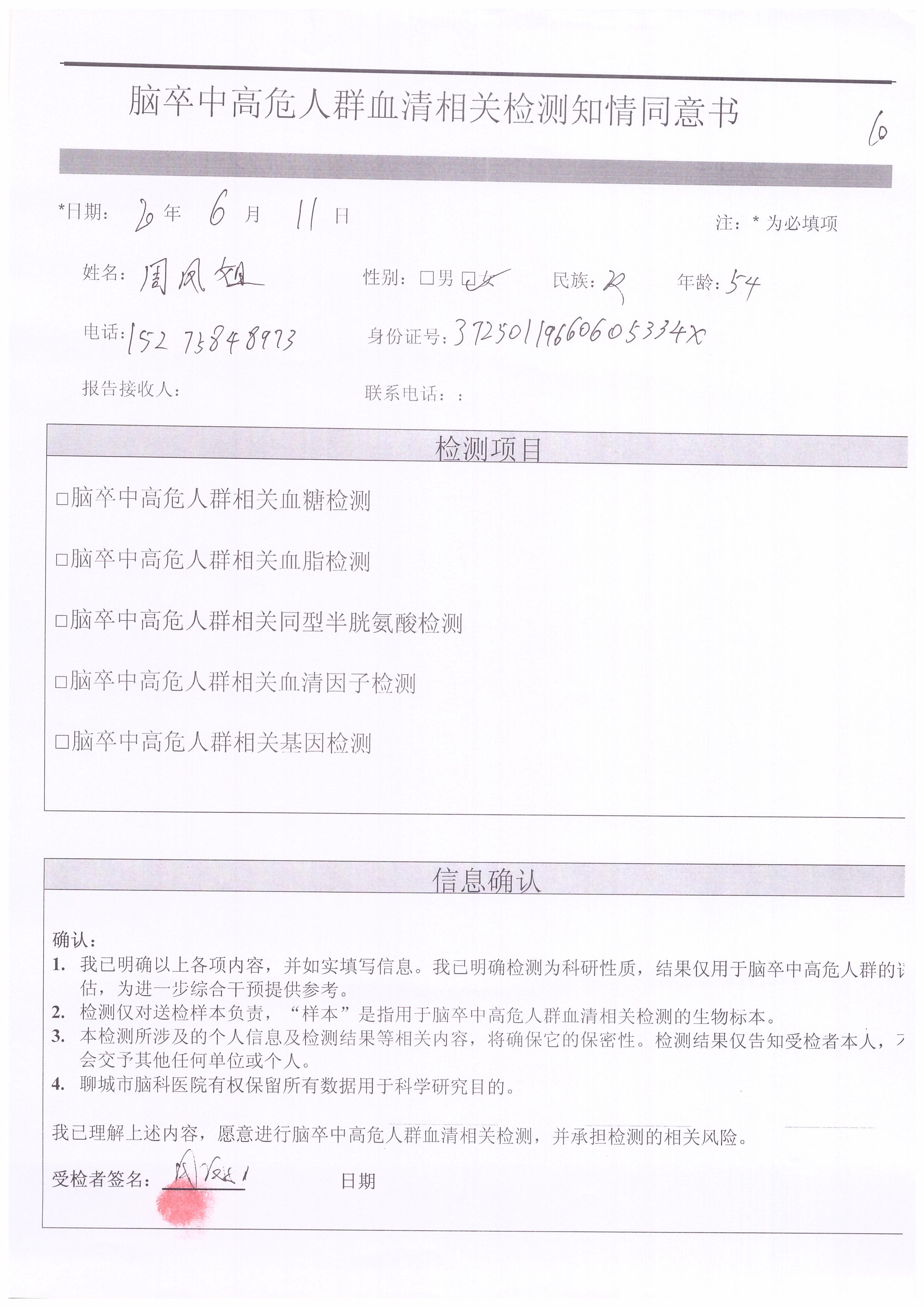

Supplement: Supplementary file 13 — Supplementary file13 (ZIP 28344 KB) [file 10528_2023_10431_MOESM13_ESM.zip › ╓¬╟Θ═1⁄4╥Γ╩Θ11/╡┌╥╗▓┐╖╓/010.jpg]

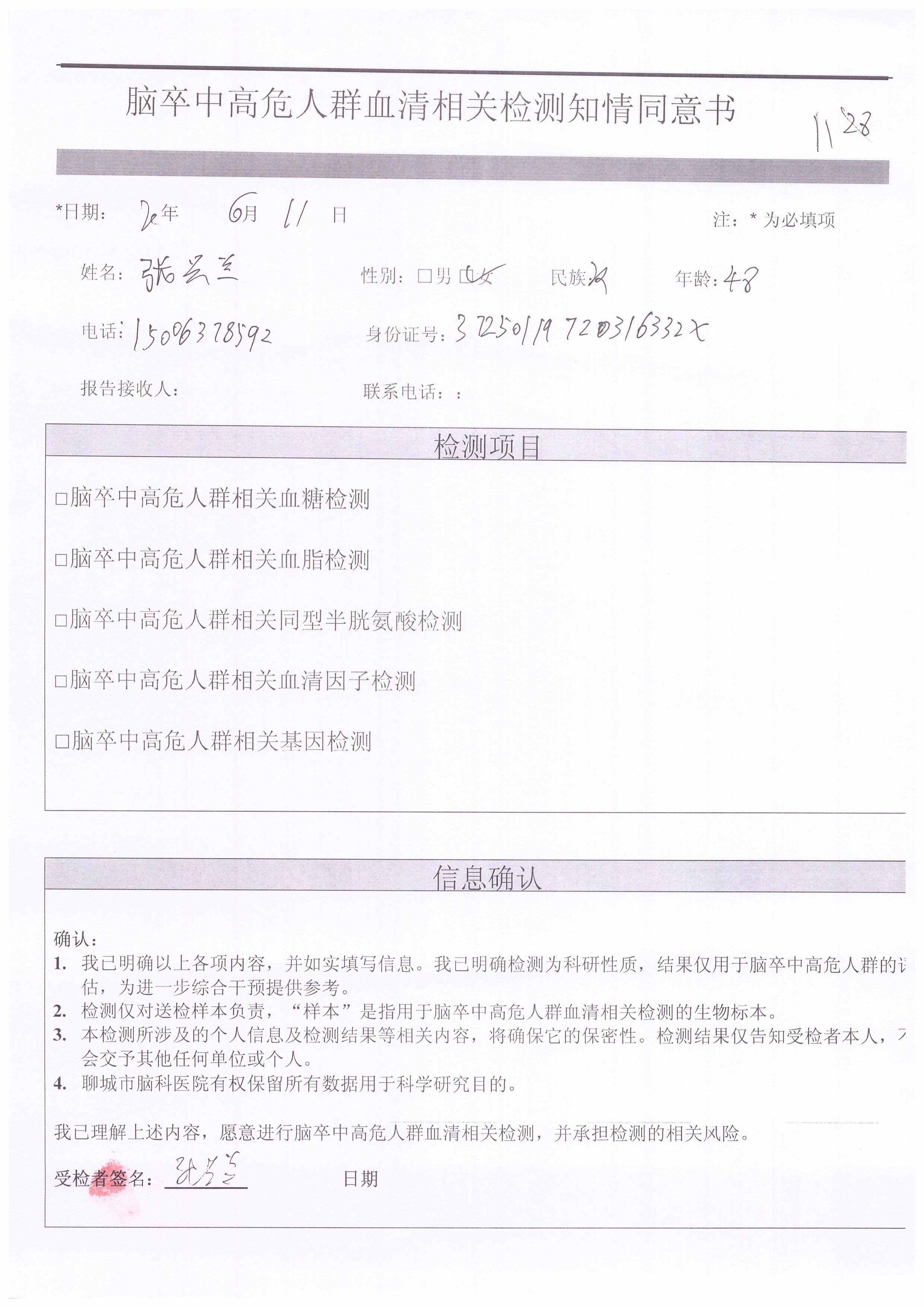

Supplement: Supplementary file 13 — Supplementary file13 (ZIP 28344 KB) [file 10528_2023_10431_MOESM13_ESM.zip › ╓¬╟Θ═1⁄4╥Γ╩Θ11/╡┌╥╗▓┐╖╓/011.jpg]

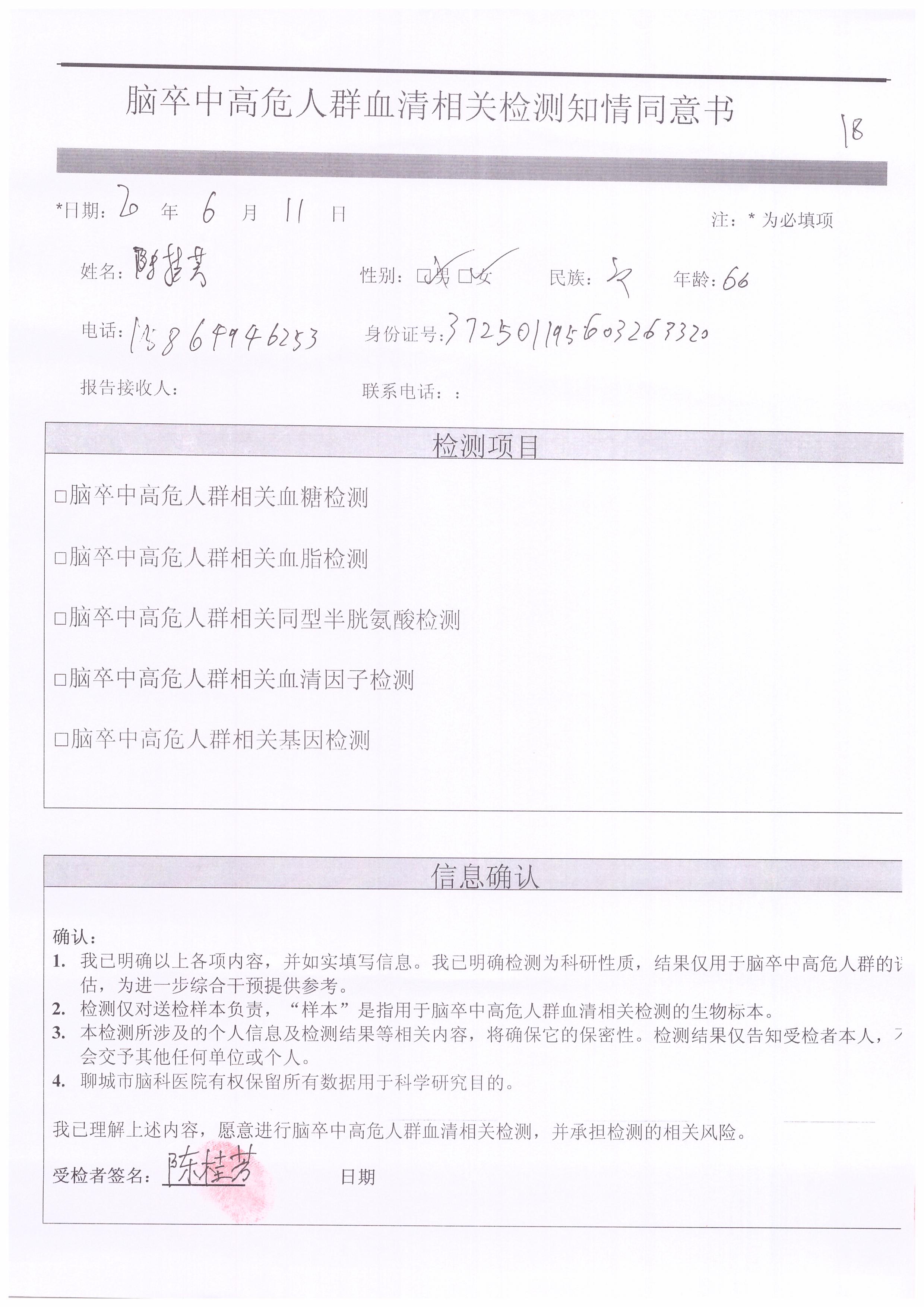

Supplement: Supplementary file 13 — Supplementary file13 (ZIP 28344 KB) [file 10528_2023_10431_MOESM13_ESM.zip › ╓¬╟Θ═1⁄4╥Γ╩Θ11/╡┌╥╗▓┐╖╓/018.jpg]

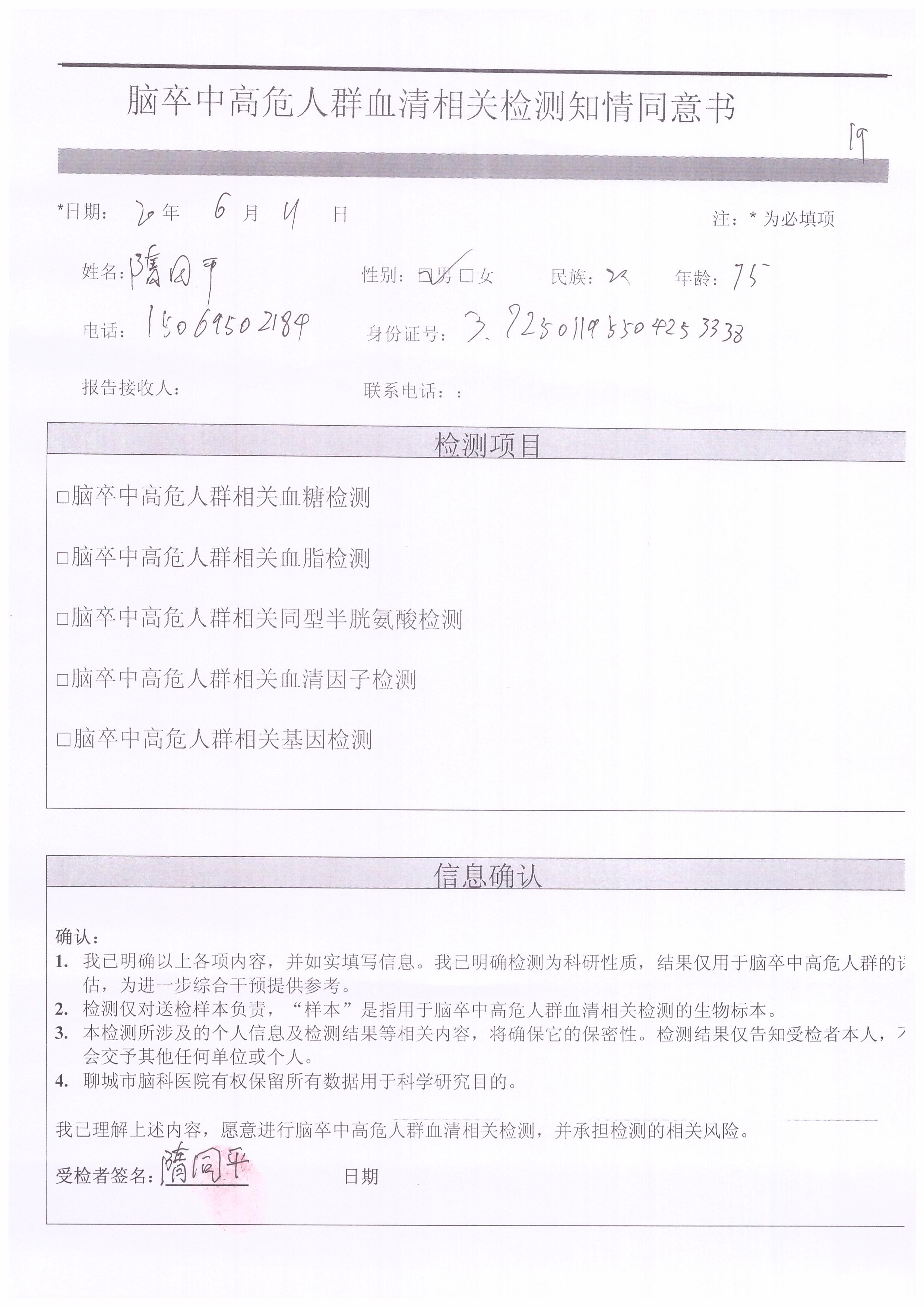

Supplement: Supplementary file 13 — Supplementary file13 (ZIP 28344 KB) [file 10528_2023_10431_MOESM13_ESM.zip › ╓¬╟Θ═1⁄4╥Γ╩Θ11/╡┌╥╗▓┐╖╓/019.jpg]

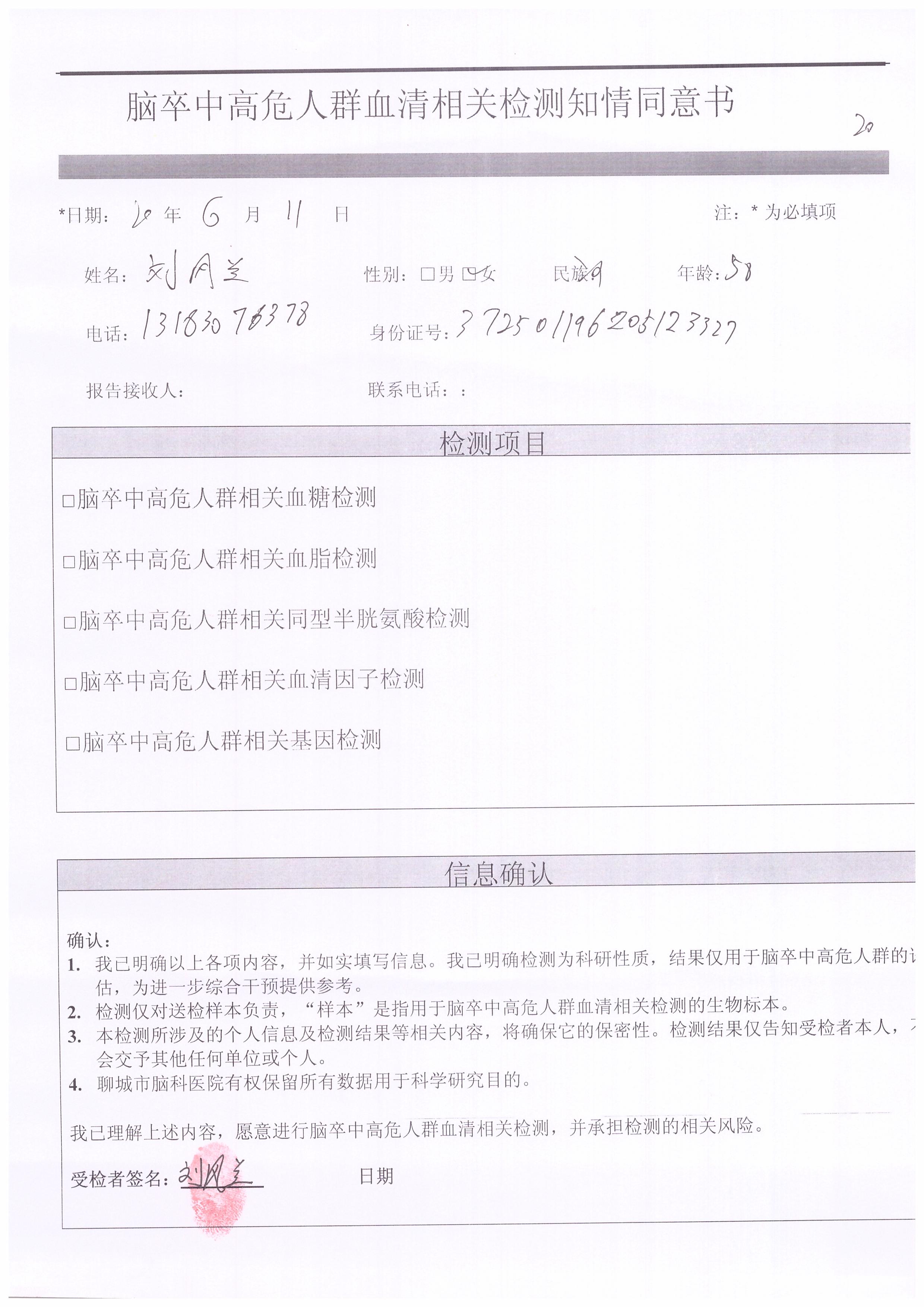

Supplement: Supplementary file 13 — Supplementary file13 (ZIP 28344 KB) [file 10528_2023_10431_MOESM13_ESM.zip › ╓¬╟Θ═1⁄4╥Γ╩Θ11/╡┌╥╗▓┐╖╓/020.jpg]

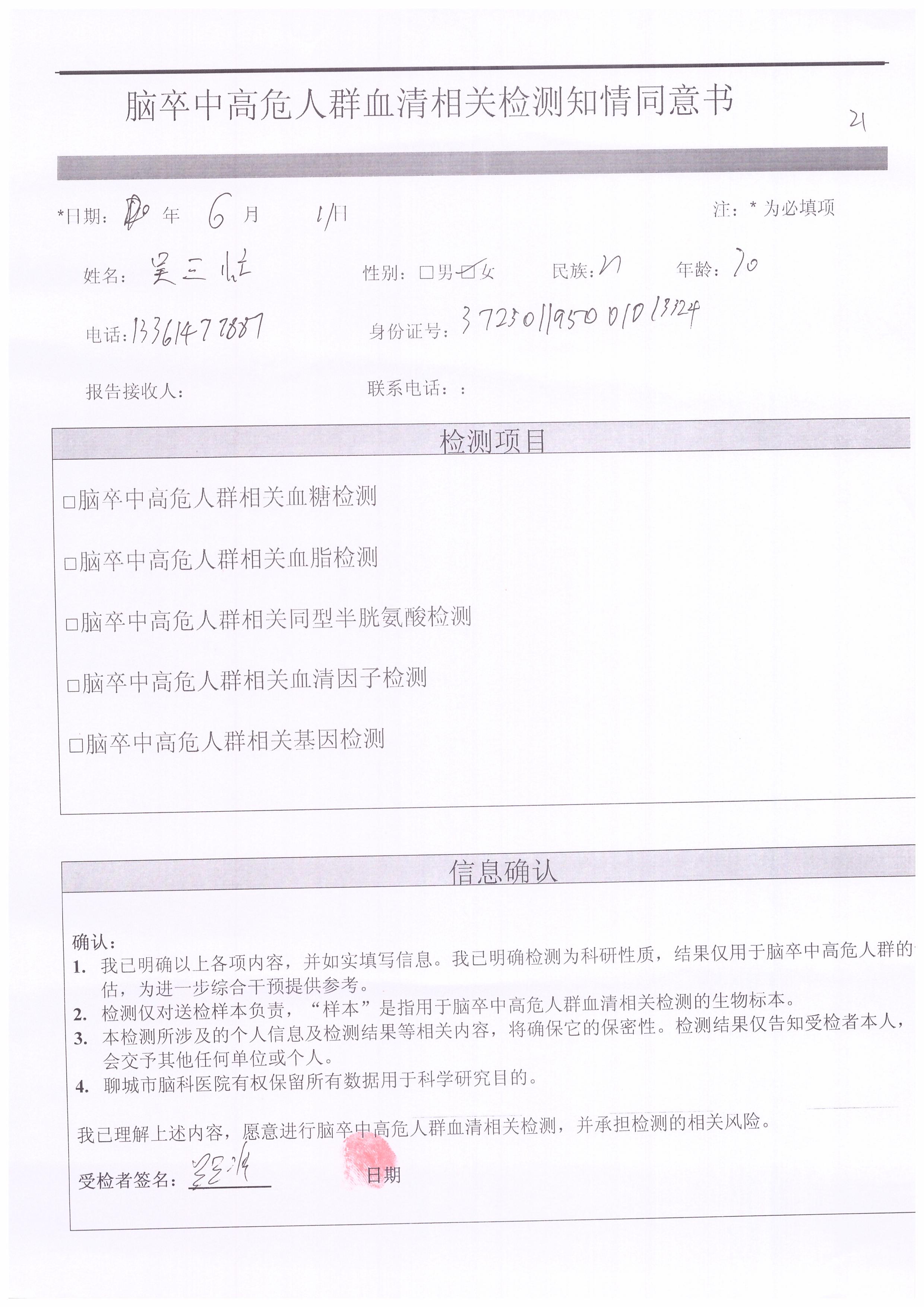

Supplement: Supplementary file 13 — Supplementary file13 (ZIP 28344 KB) [file 10528_2023_10431_MOESM13_ESM.zip › ╓¬╟Θ═1⁄4╥Γ╩Θ11/╡┌╥╗▓┐╖╓/021.jpg]

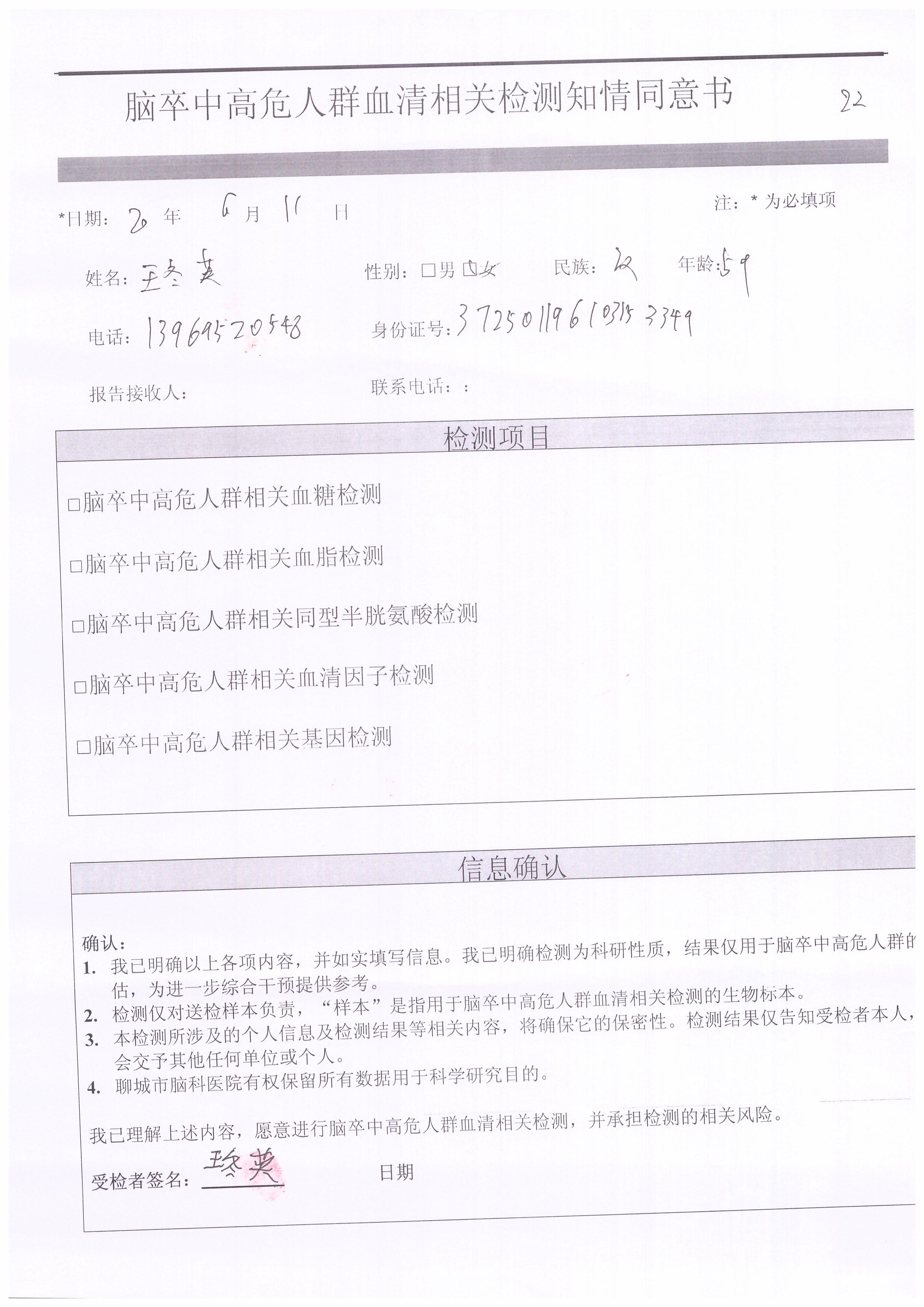

Supplement: Supplementary file 13 — Supplementary file13 (ZIP 28344 KB) [file 10528_2023_10431_MOESM13_ESM.zip › ╓¬╟Θ═1⁄4╥Γ╩Θ11/╡┌╥╗▓┐╖╓/022.jpg]

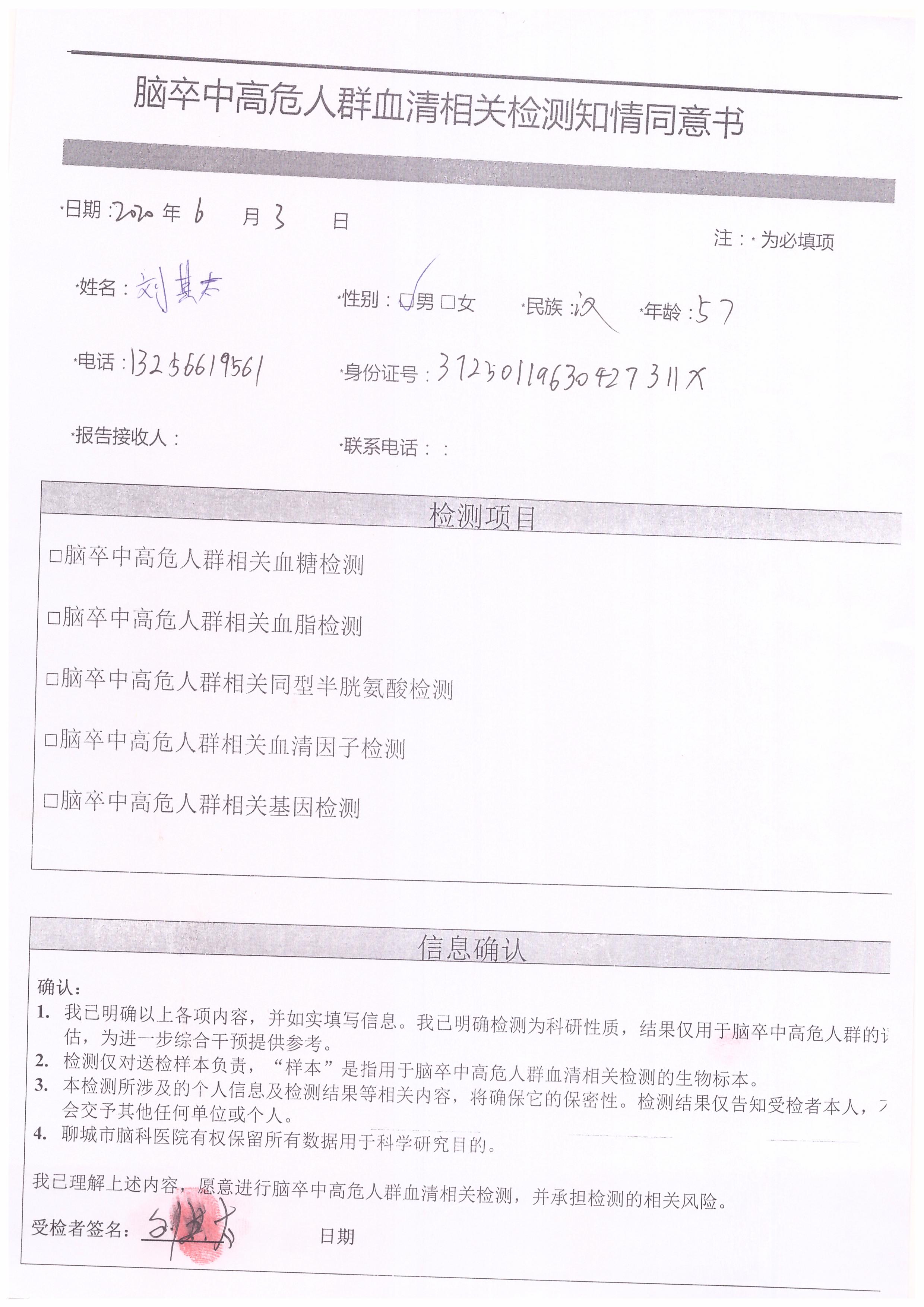

Supplement: Supplementary file 14 — Supplementary file14 (ZIP 27750 KB) [file 10528_2023_10431_MOESM14_ESM.zip › ╓¬╟Θ═1⁄4╥Γ╩Θ12/╡┌╥╗▓┐╖╓í┐/001.jpg]

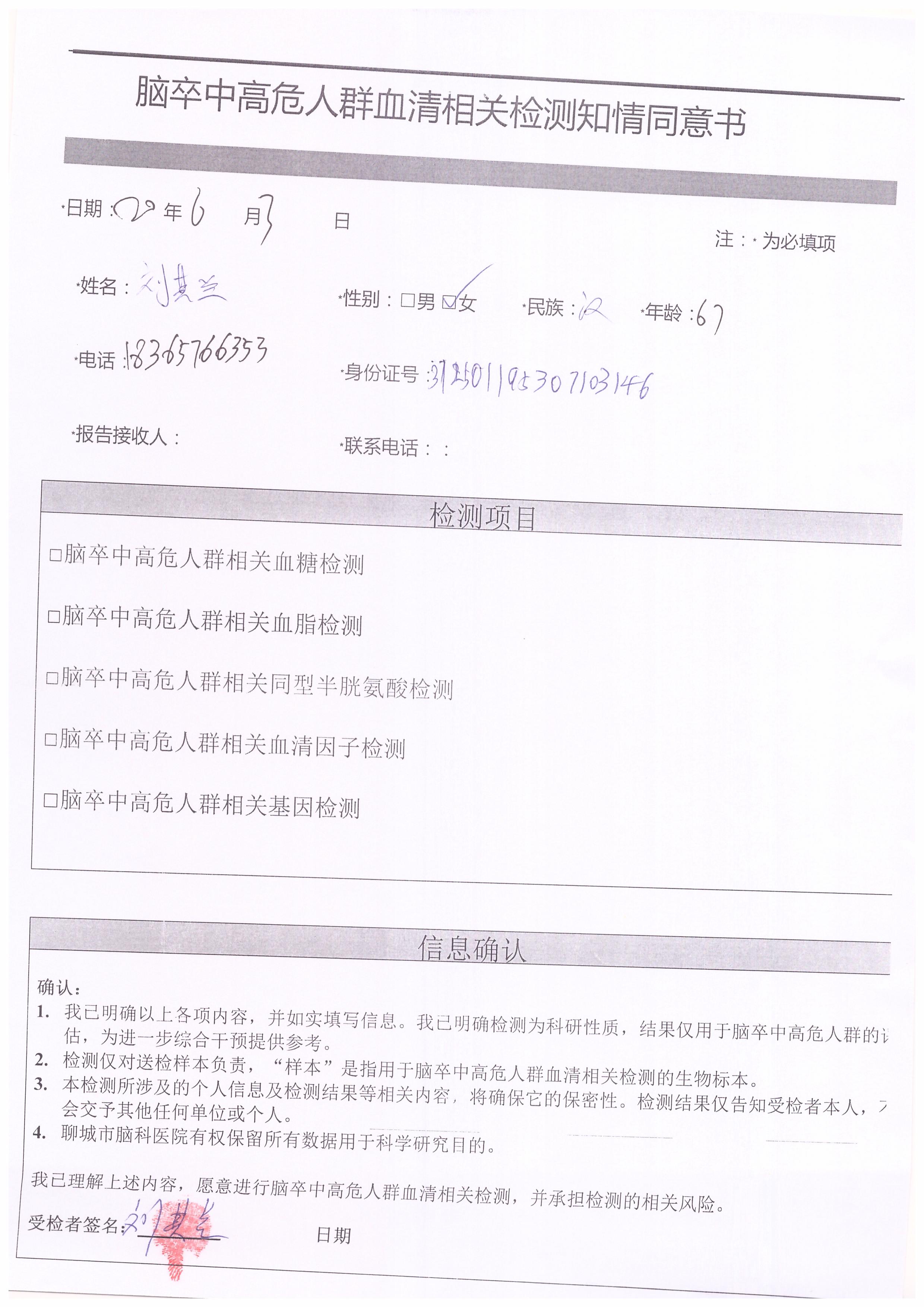

Supplement: Supplementary file 14 — Supplementary file14 (ZIP 27750 KB) [file 10528_2023_10431_MOESM14_ESM.zip › ╓¬╟Θ═1⁄4╥Γ╩Θ12/╡┌╥╗▓┐╖╓í┐/002.jpg]

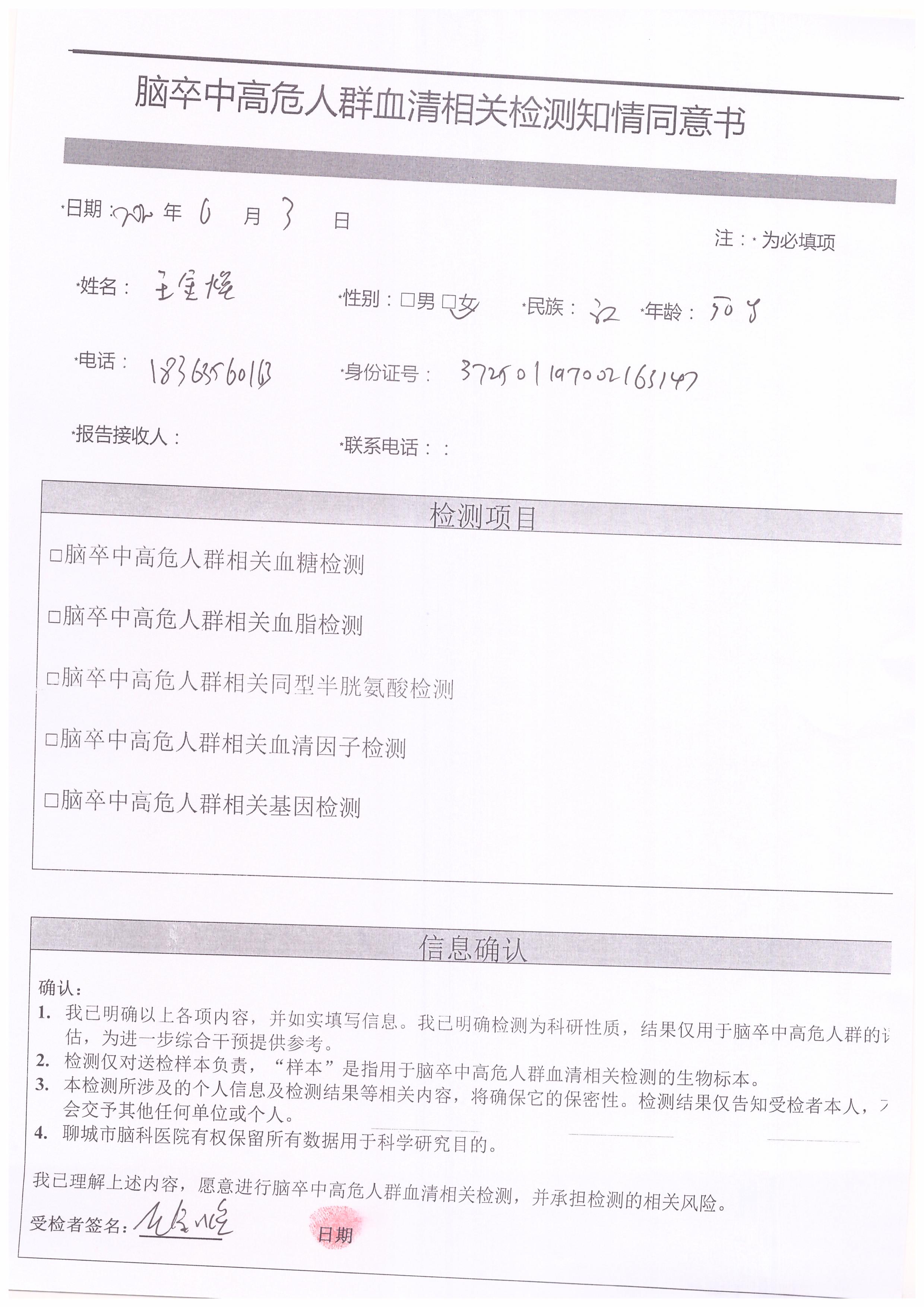

Supplement: Supplementary file 14 — Supplementary file14 (ZIP 27750 KB) [file 10528_2023_10431_MOESM14_ESM.zip › ╓¬╟Θ═1⁄4╥Γ╩Θ12/╡┌╥╗▓┐╖╓í┐/003.jpg]

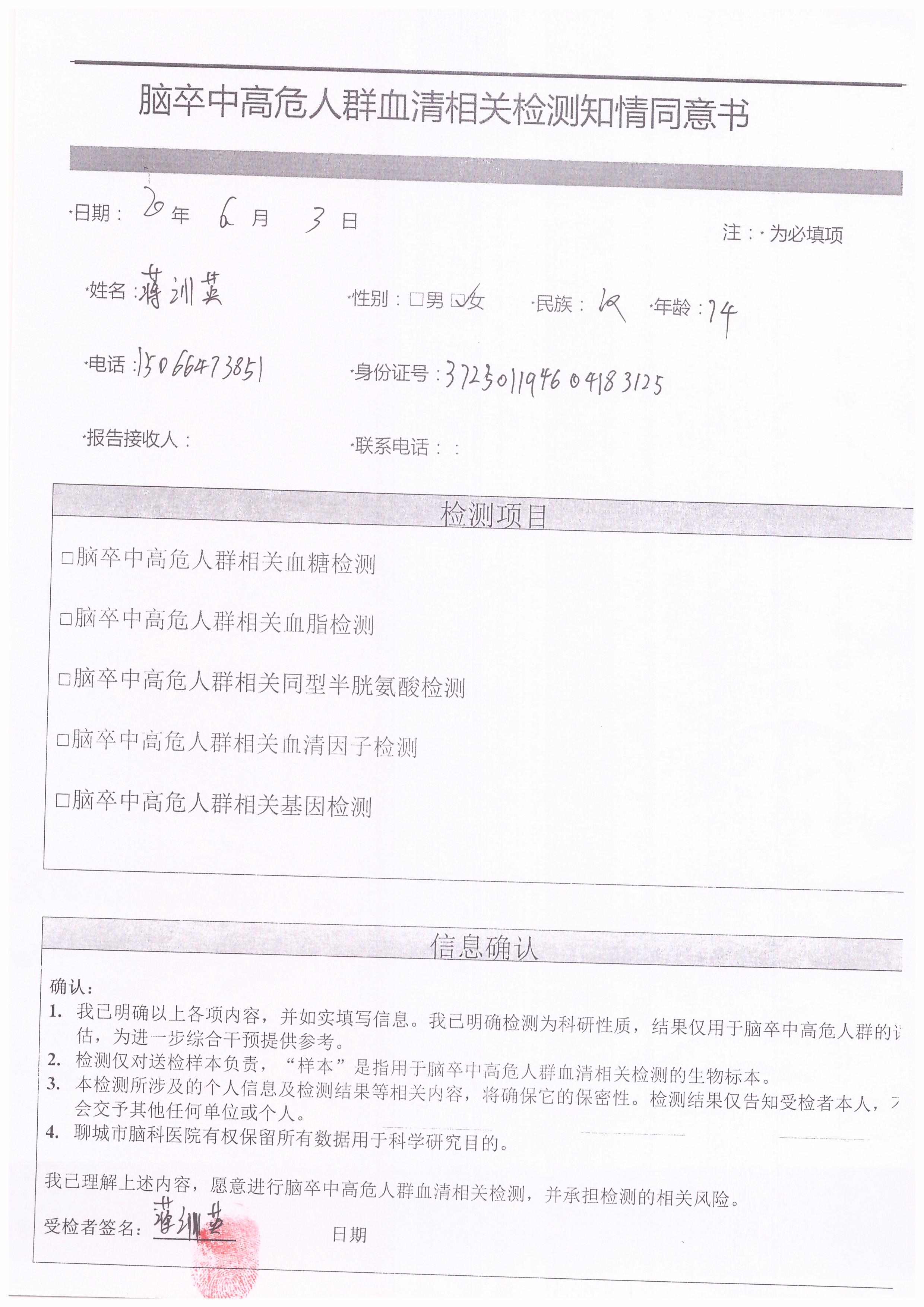

Supplement: Supplementary file 14 — Supplementary file14 (ZIP 27750 KB) [file 10528_2023_10431_MOESM14_ESM.zip › ╓¬╟Θ═1⁄4╥Γ╩Θ12/╡┌╥╗▓┐╖╓í┐/004.jpg]

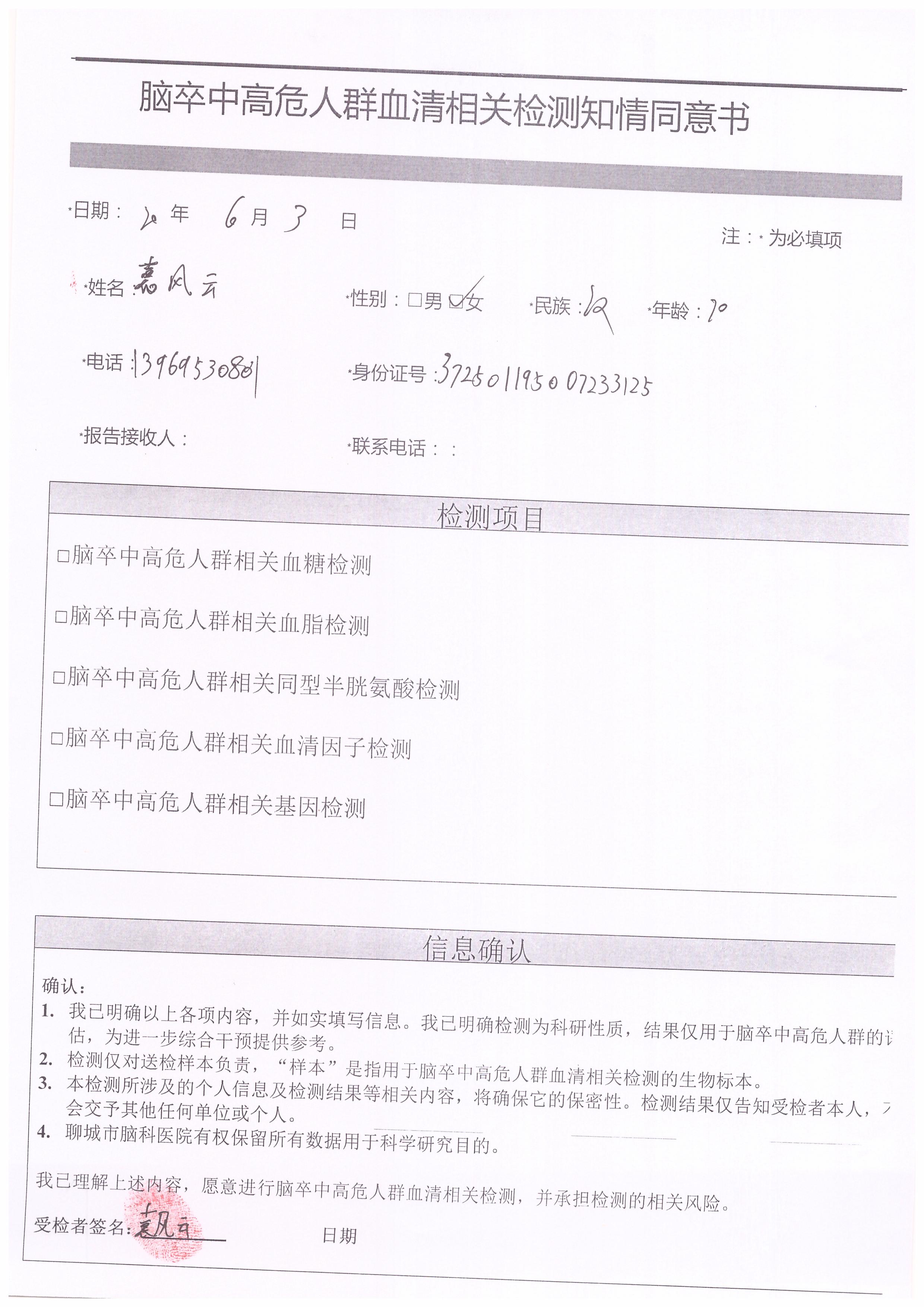

Supplement: Supplementary file 14 — Supplementary file14 (ZIP 27750 KB) [file 10528_2023_10431_MOESM14_ESM.zip › ╓¬╟Θ═1⁄4╥Γ╩Θ12/╡┌╥╗▓┐╖╓í┐/005.jpg]

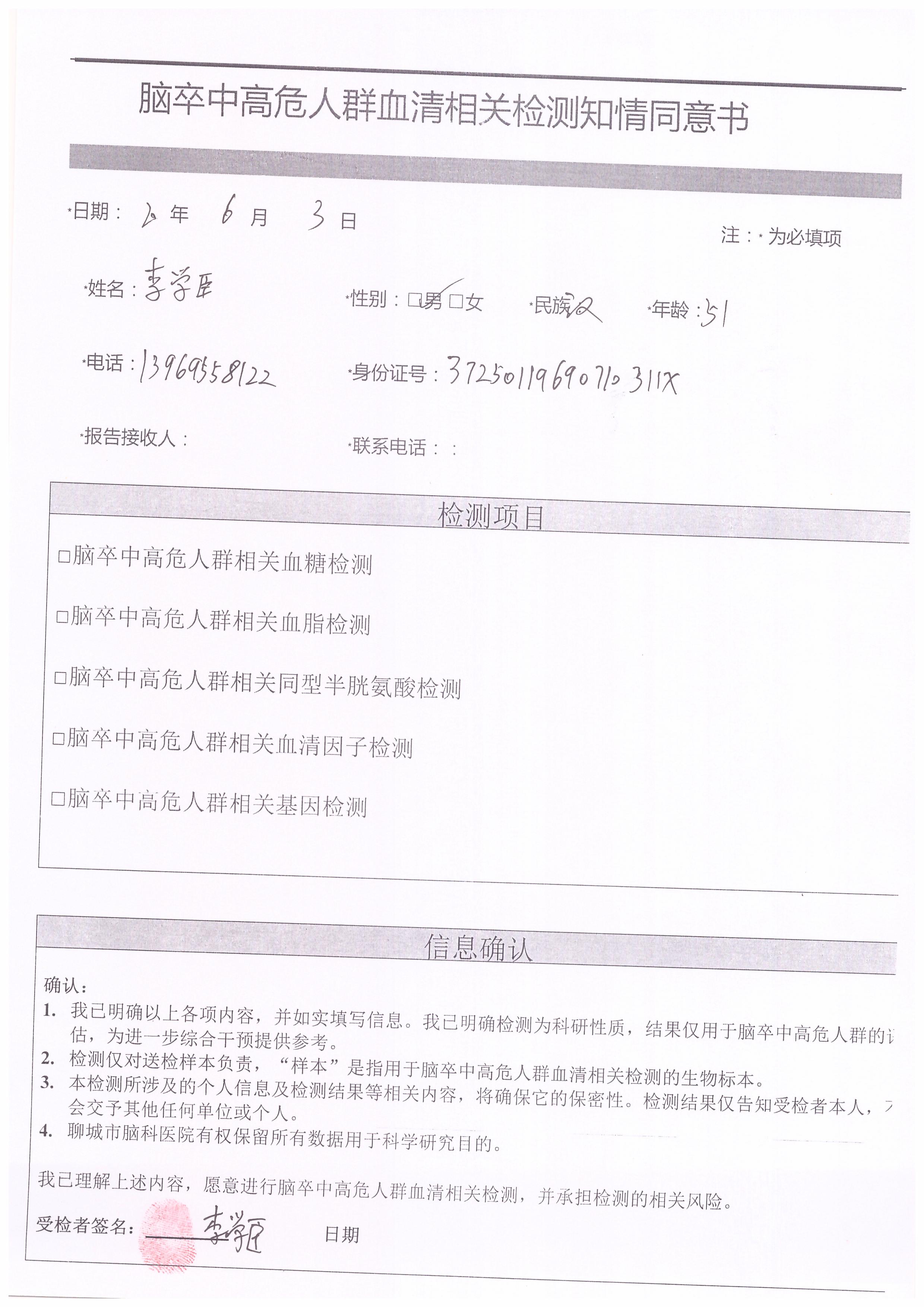

Supplement: Supplementary file 14 — Supplementary file14 (ZIP 27750 KB) [file 10528_2023_10431_MOESM14_ESM.zip › ╓¬╟Θ═1⁄4╥Γ╩Θ12/╡┌╥╗▓┐╖╓í┐/006.jpg]

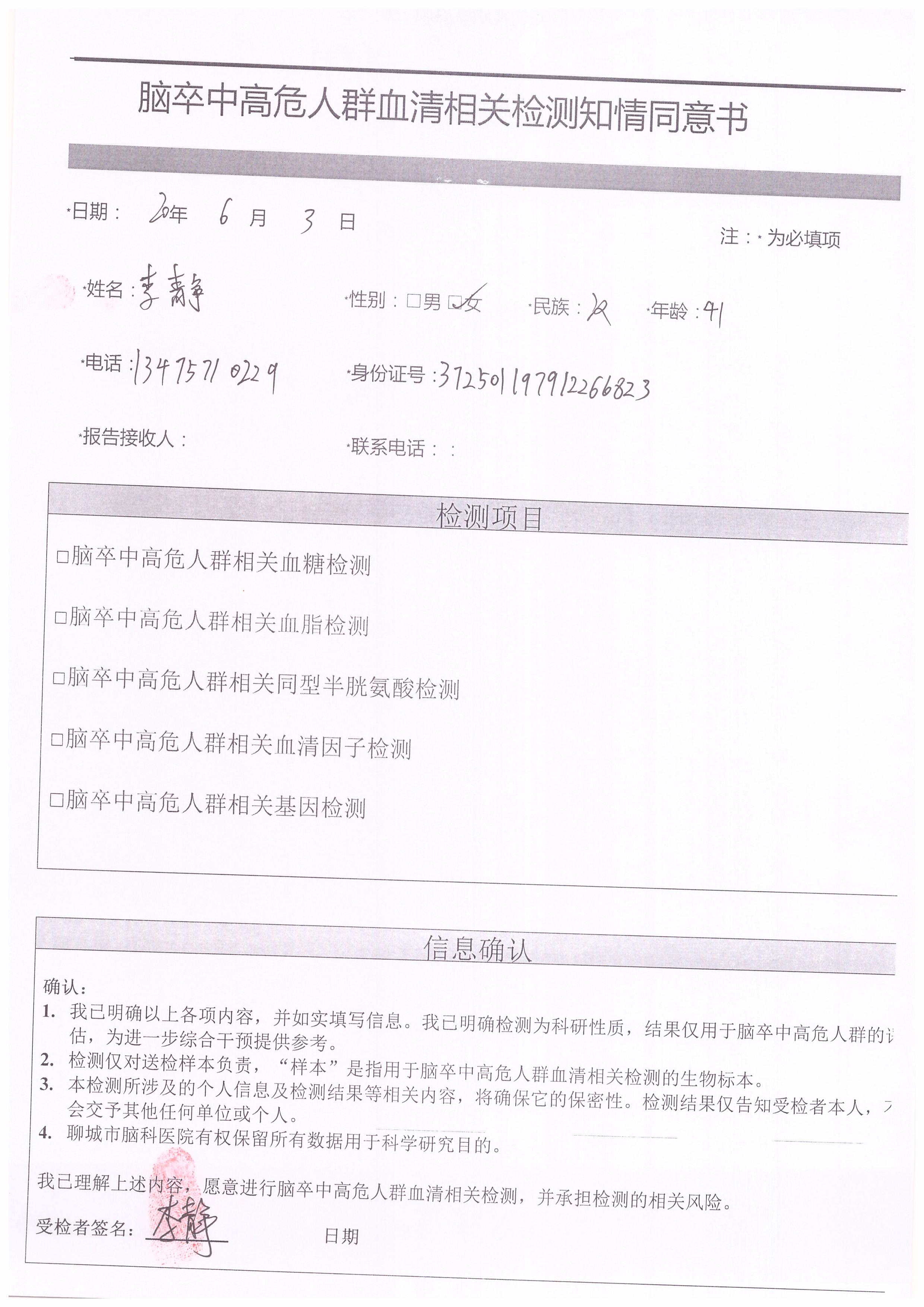

Supplement: Supplementary file 14 — Supplementary file14 (ZIP 27750 KB) [file 10528_2023_10431_MOESM14_ESM.zip › ╓¬╟Θ═1⁄4╥Γ╩Θ12/╡┌╥╗▓┐╖╓í┐/007.jpg]

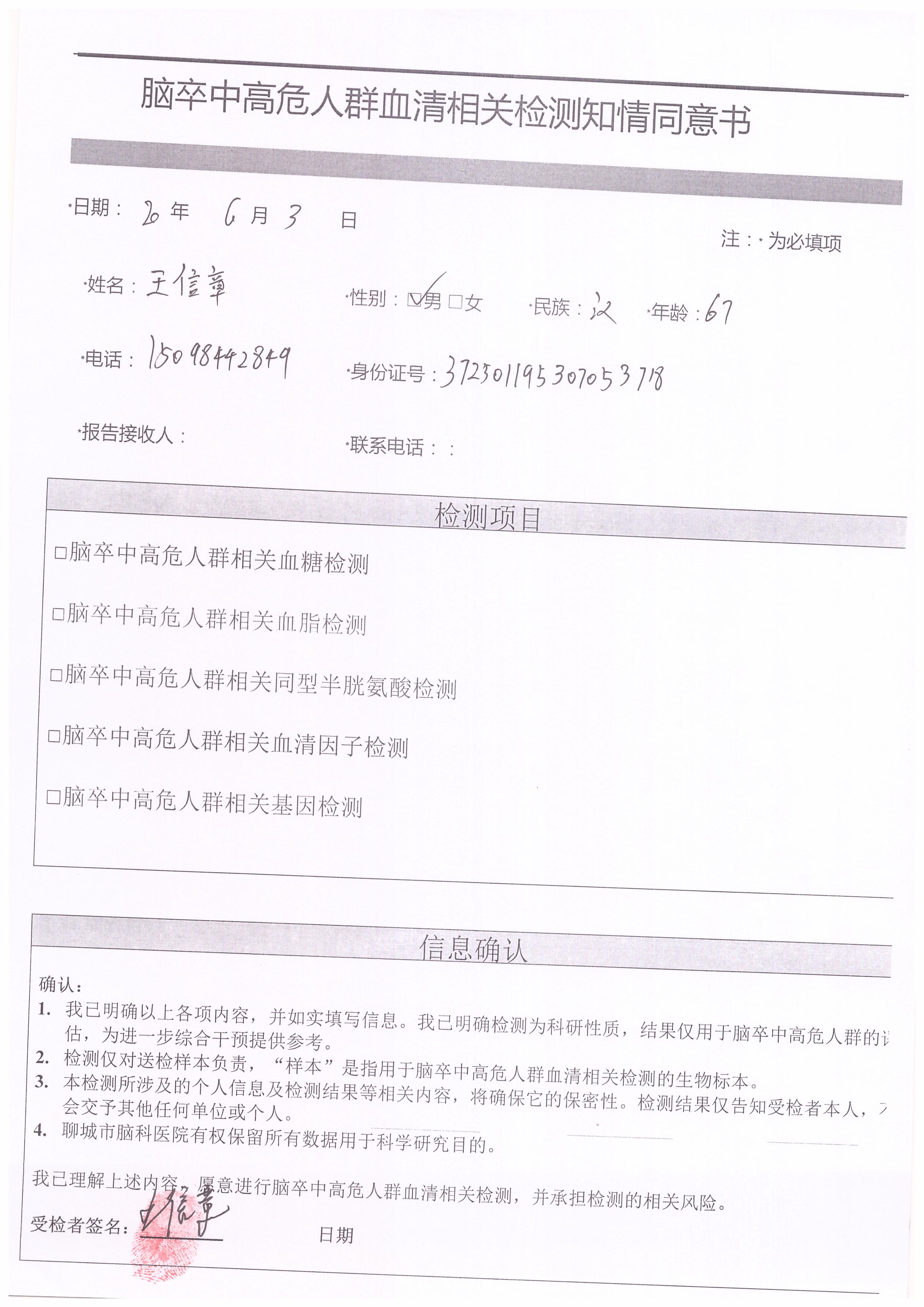

Supplement: Supplementary file 14 — Supplementary file14 (ZIP 27750 KB) [file 10528_2023_10431_MOESM14_ESM.zip › ╓¬╟Θ═1⁄4╥Γ╩Θ12/╡┌╥╗▓┐╖╓í┐/008.jpg]

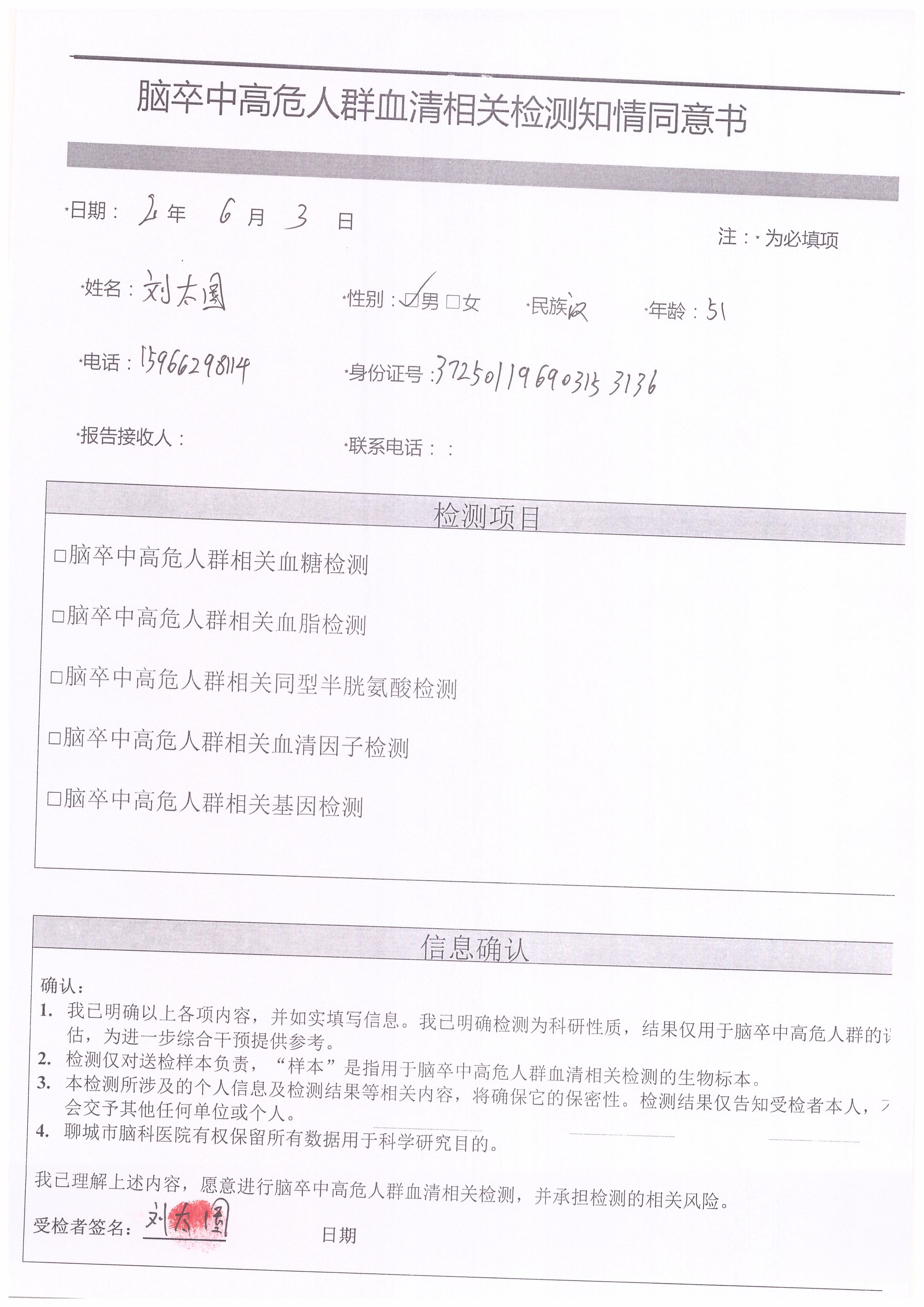

Supplement: Supplementary file 14 — Supplementary file14 (ZIP 27750 KB) [file 10528_2023_10431_MOESM14_ESM.zip › ╓¬╟Θ═1⁄4╥Γ╩Θ12/╡┌╥╗▓┐╖╓í┐/009.jpg]

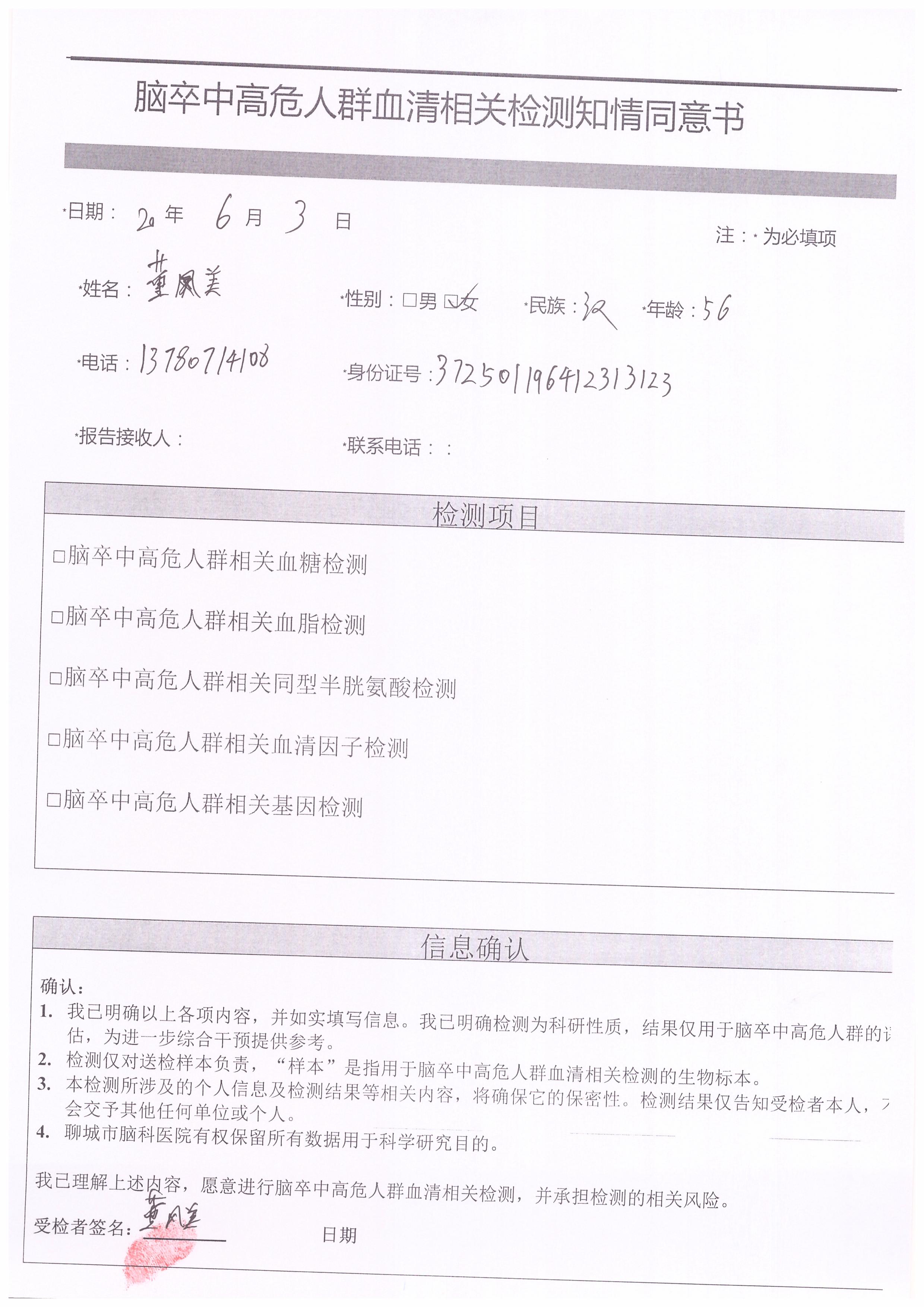

Supplement: Supplementary file 14 — Supplementary file14 (ZIP 27750 KB) [file 10528_2023_10431_MOESM14_ESM.zip › ╓¬╟Θ═1⁄4╥Γ╩Θ12/╡┌╥╗▓┐╖╓í┐/010.jpg]

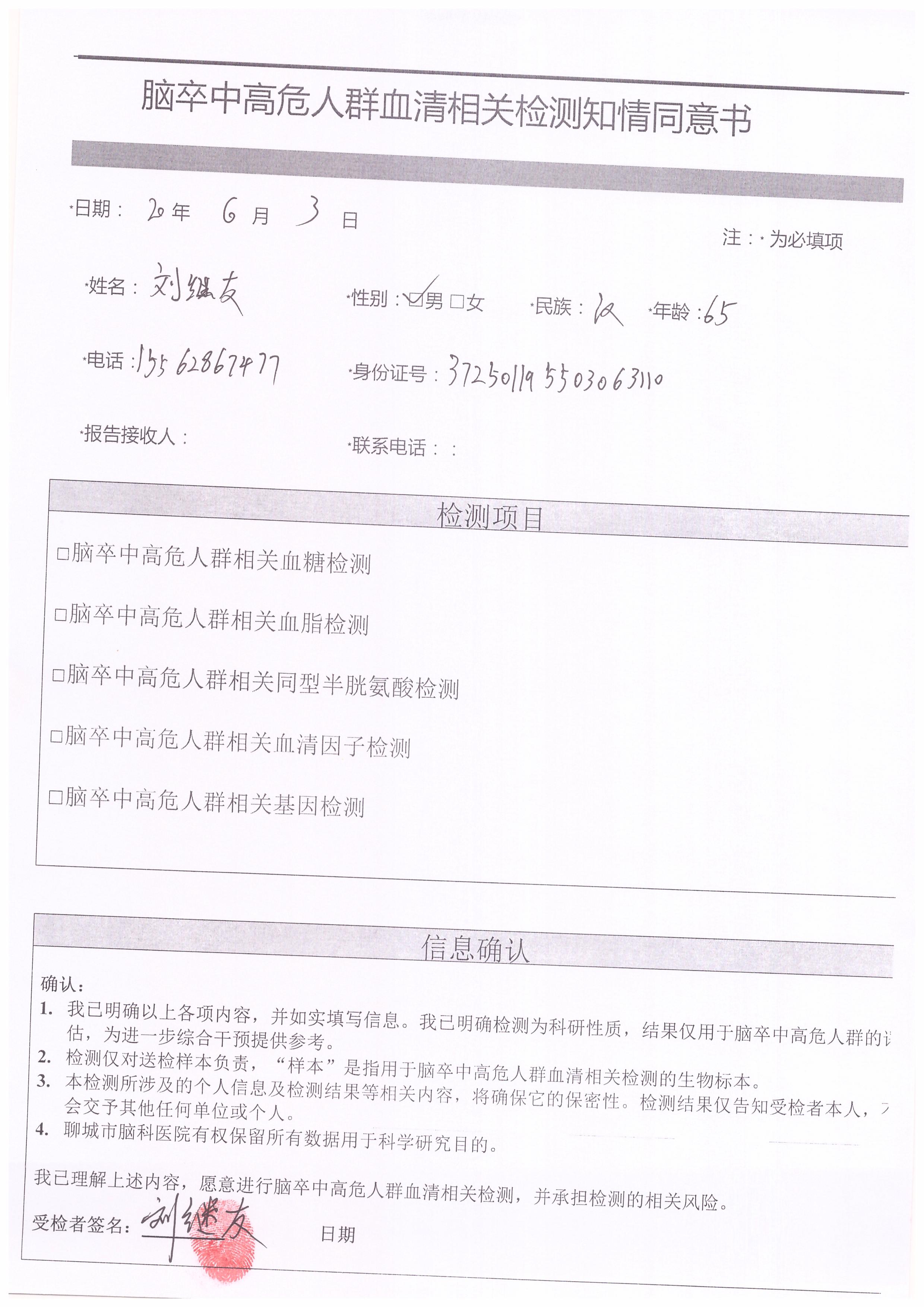

Supplement: Supplementary file 14 — Supplementary file14 (ZIP 27750 KB) [file 10528_2023_10431_MOESM14_ESM.zip › ╓¬╟Θ═1⁄4╥Γ╩Θ12/╡┌╥╗▓┐╖╓í┐/011.jpg]

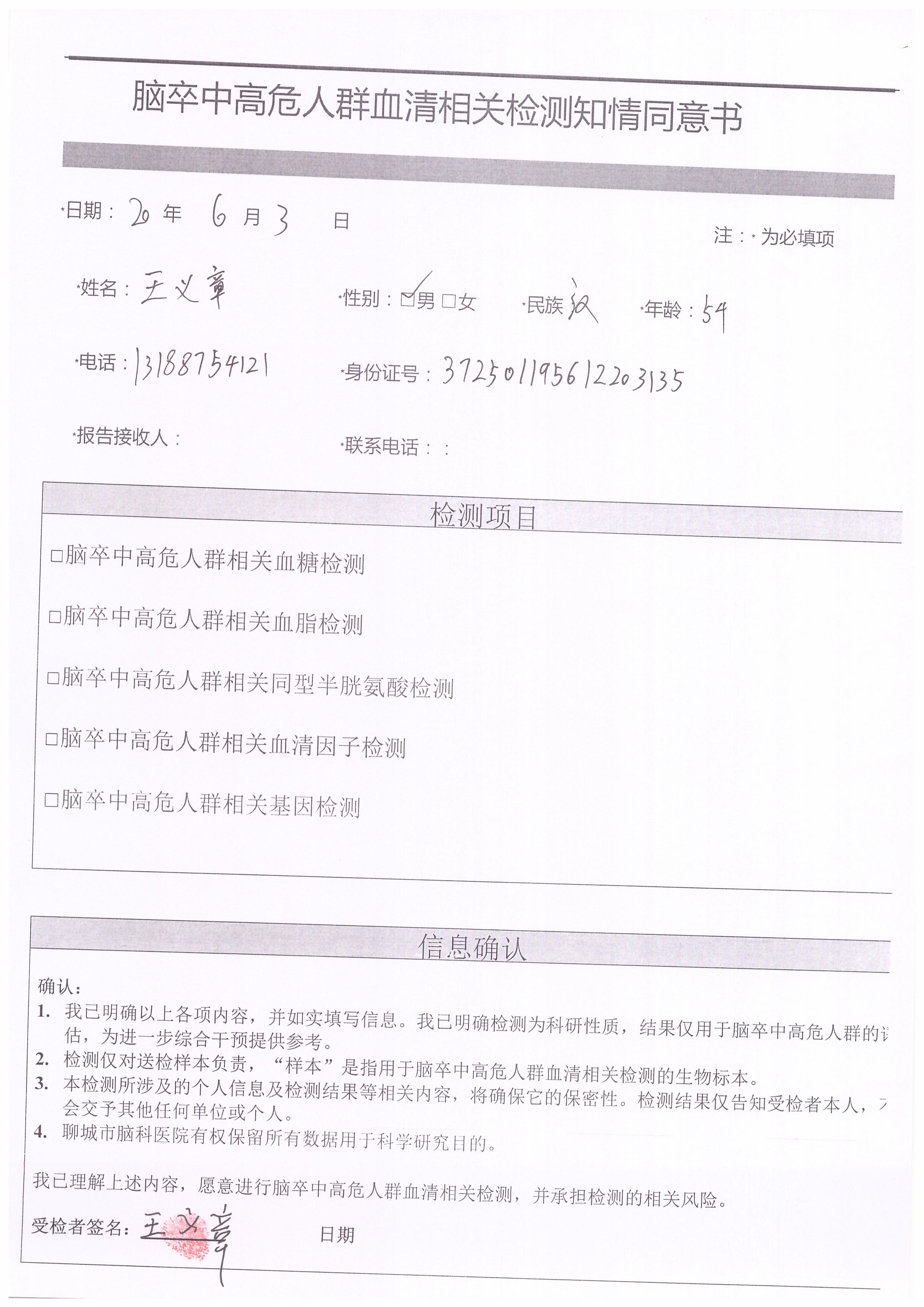

Supplement: Supplementary file 14 — Supplementary file14 (ZIP 27750 KB) [file 10528_2023_10431_MOESM14_ESM.zip › ╓¬╟Θ═1⁄4╥Γ╩Θ12/╡┌╥╗▓┐╖╓í┐/012.jpg]

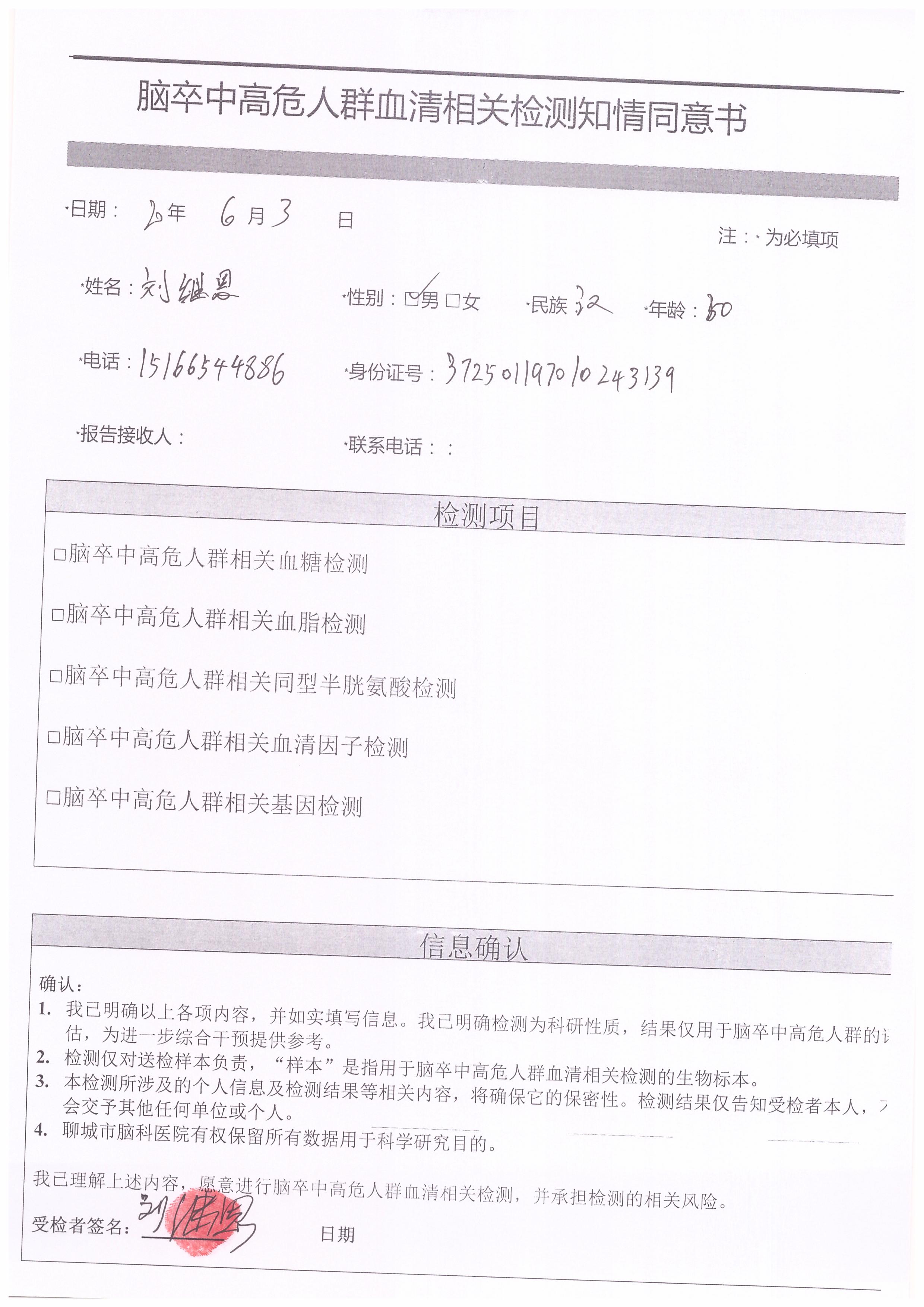

Supplement: Supplementary file 14 — Supplementary file14 (ZIP 27750 KB) [file 10528_2023_10431_MOESM14_ESM.zip › ╓¬╟Θ═1⁄4╥Γ╩Θ12/╡┌╥╗▓┐╖╓í┐/013.jpg]

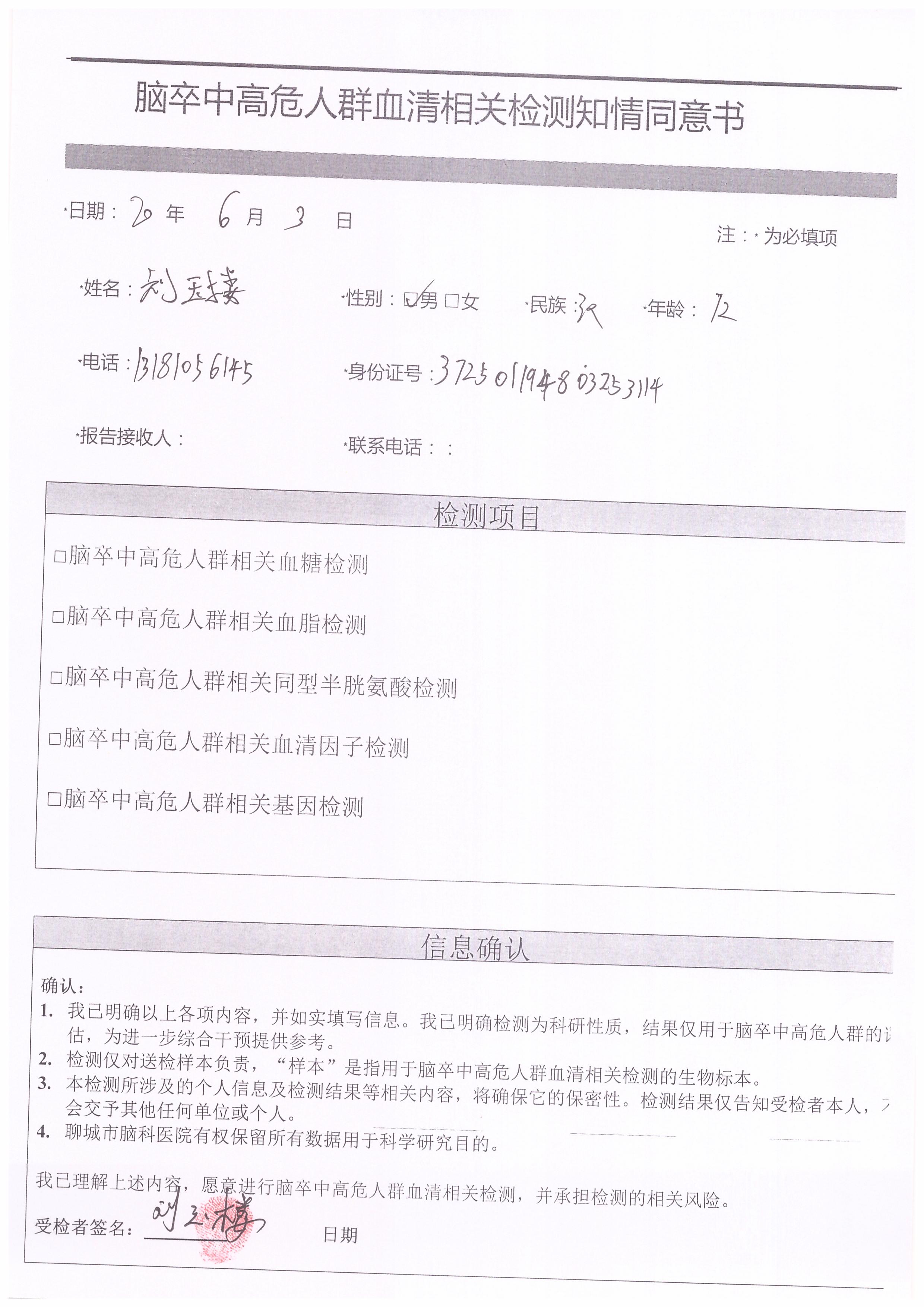

Supplement: Supplementary file 14 — Supplementary file14 (ZIP 27750 KB) [file 10528_2023_10431_MOESM14_ESM.zip › ╓¬╟Θ═1⁄4╥Γ╩Θ12/╡┌╥╗▓┐╖╓í┐/014.jpg]

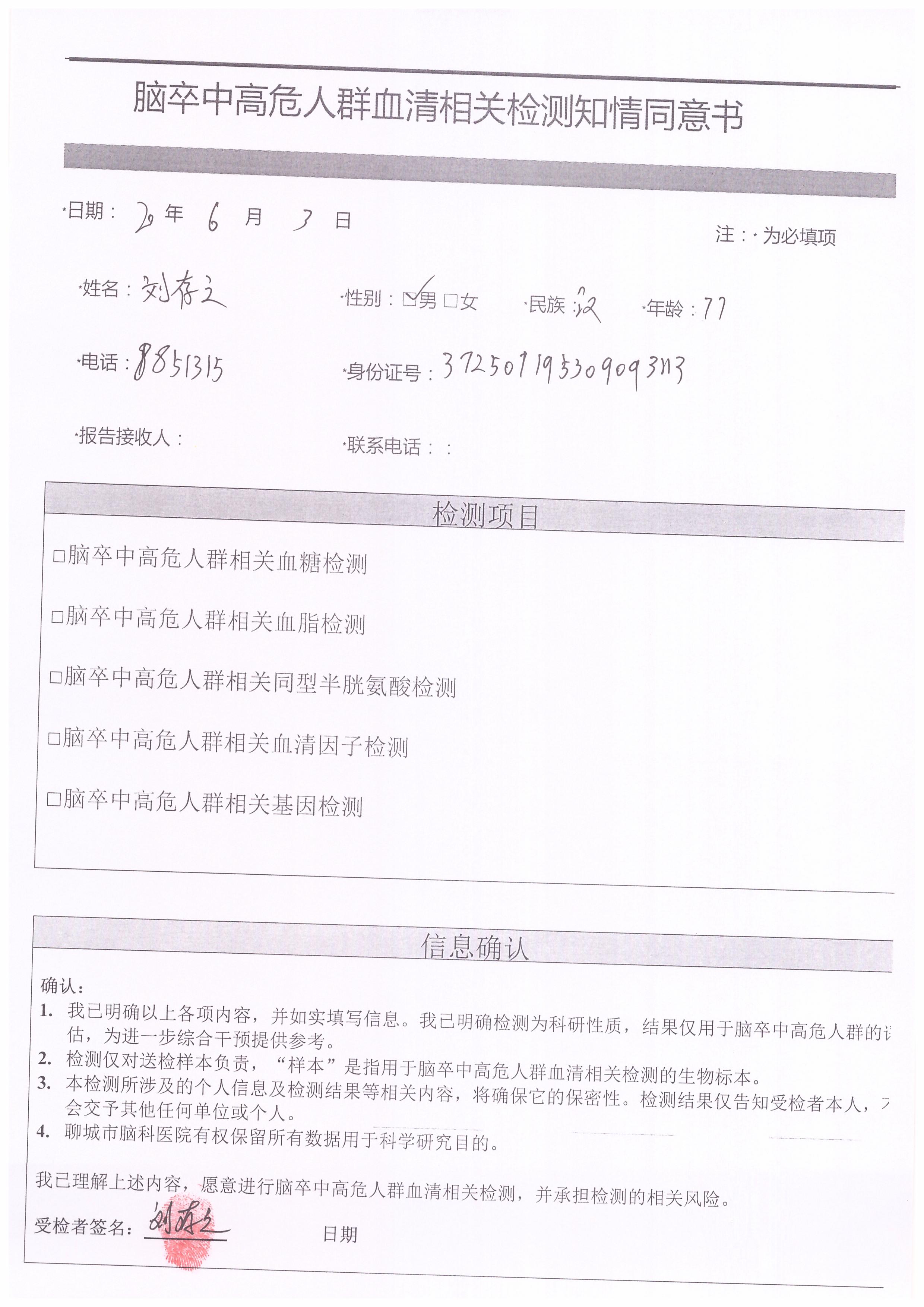

Supplement: Supplementary file 14 — Supplementary file14 (ZIP 27750 KB) [file 10528_2023_10431_MOESM14_ESM.zip › ╓¬╟Θ═1⁄4╥Γ╩Θ12/╡┌╥╗▓┐╖╓í┐/015.jpg]

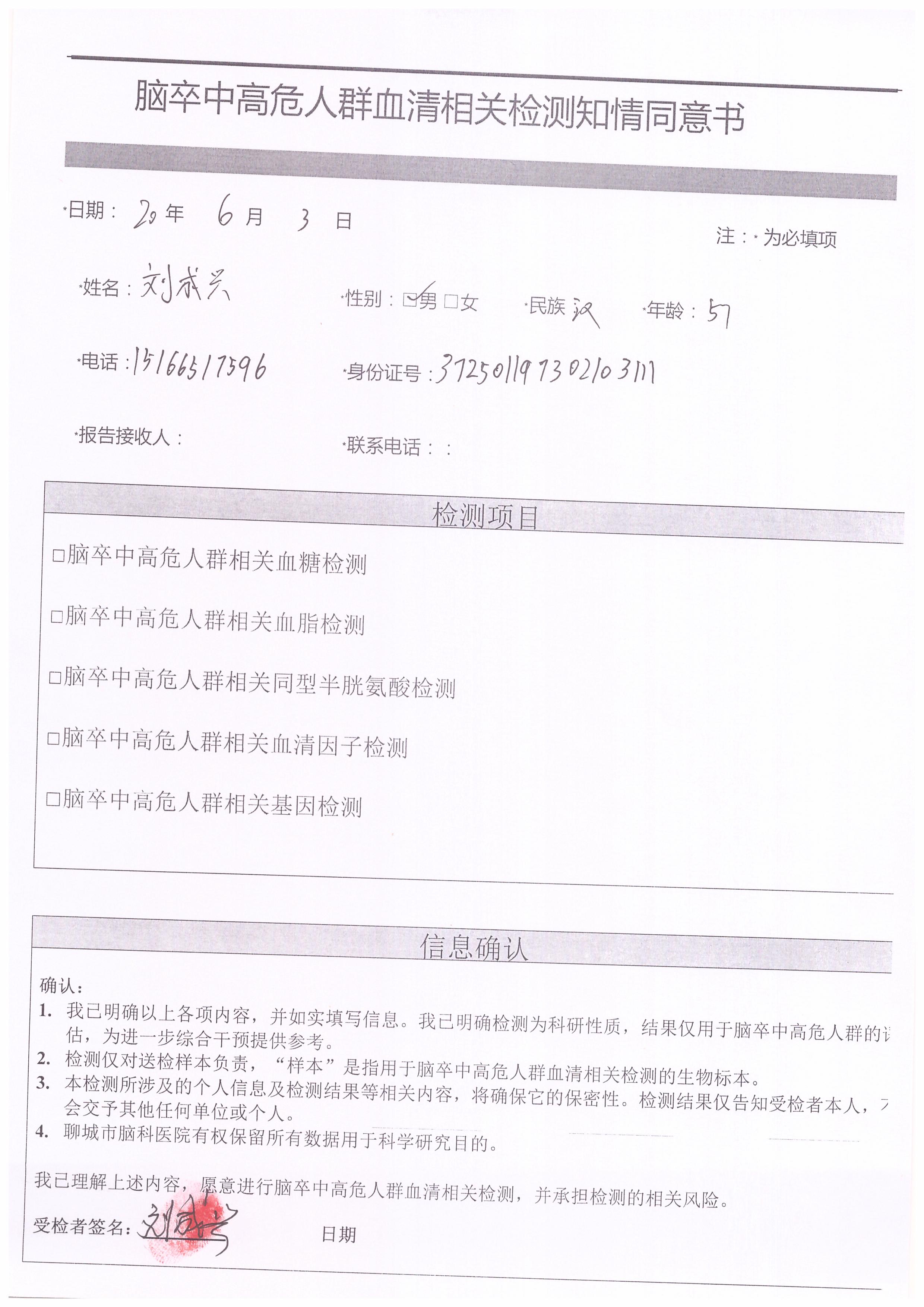

Supplement: Supplementary file 14 — Supplementary file14 (ZIP 27750 KB) [file 10528_2023_10431_MOESM14_ESM.zip › ╓¬╟Θ═1⁄4╥Γ╩Θ12/╡┌╥╗▓┐╖╓í┐/016.jpg]

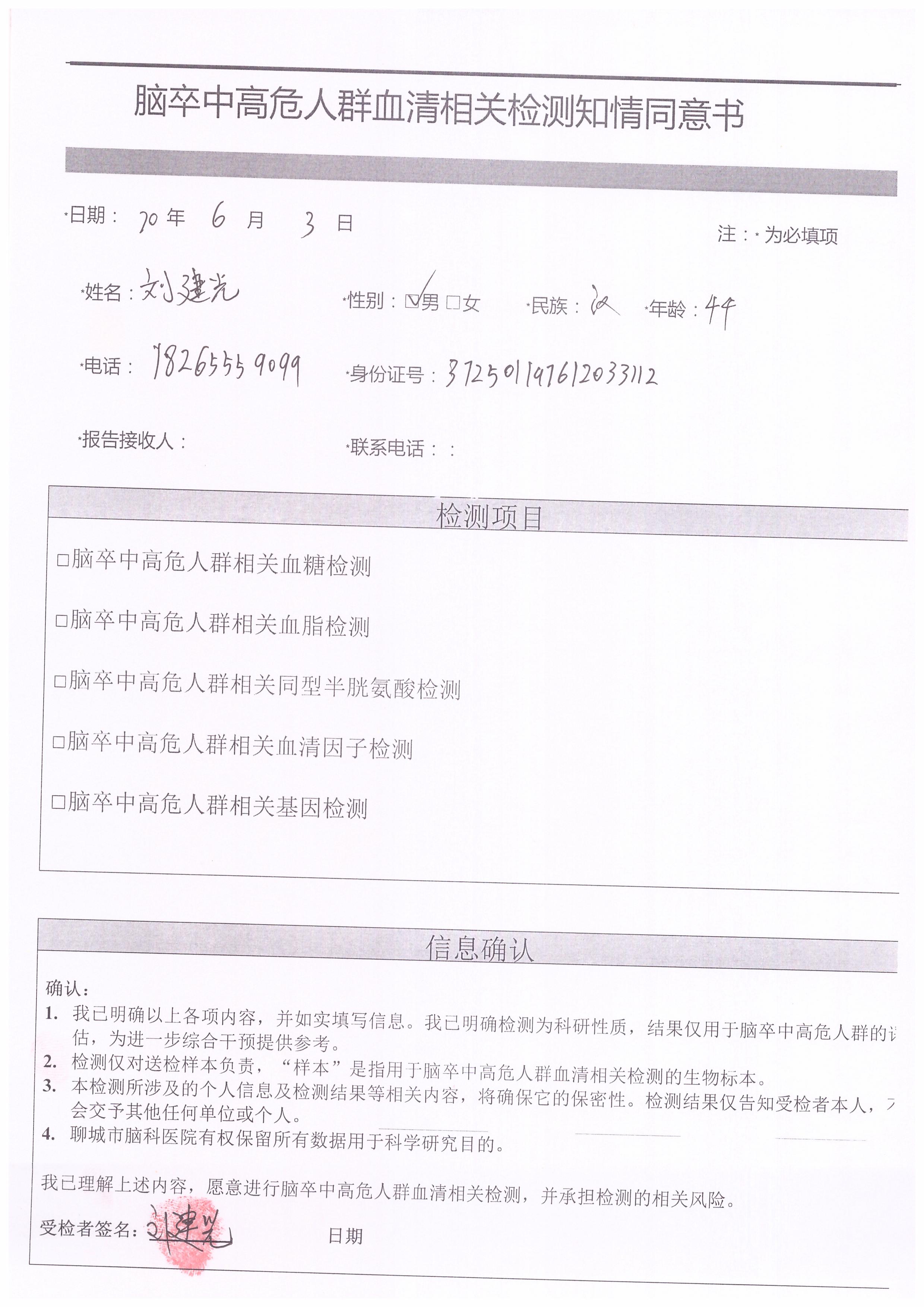

Supplement: Supplementary file 14 — Supplementary file14 (ZIP 27750 KB) [file 10528_2023_10431_MOESM14_ESM.zip › ╓¬╟Θ═1⁄4╥Γ╩Θ12/╡┌╥╗▓┐╖╓í┐/017.jpg]

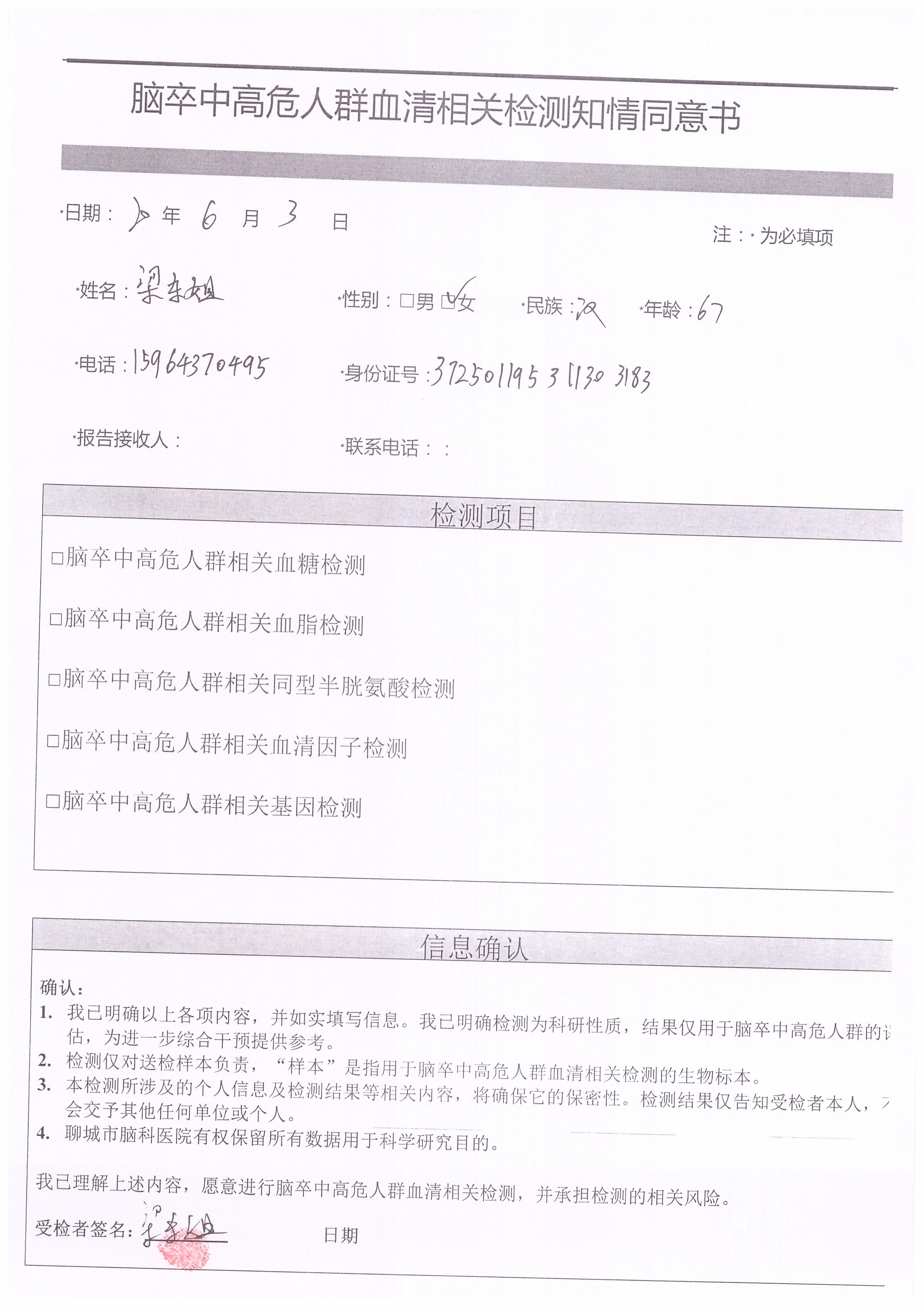

Supplement: Supplementary file 14 — Supplementary file14 (ZIP 27750 KB) [file 10528_2023_10431_MOESM14_ESM.zip › ╓¬╟Θ═1⁄4╥Γ╩Θ12/╡┌╥╗▓┐╖╓í┐/018.jpg]

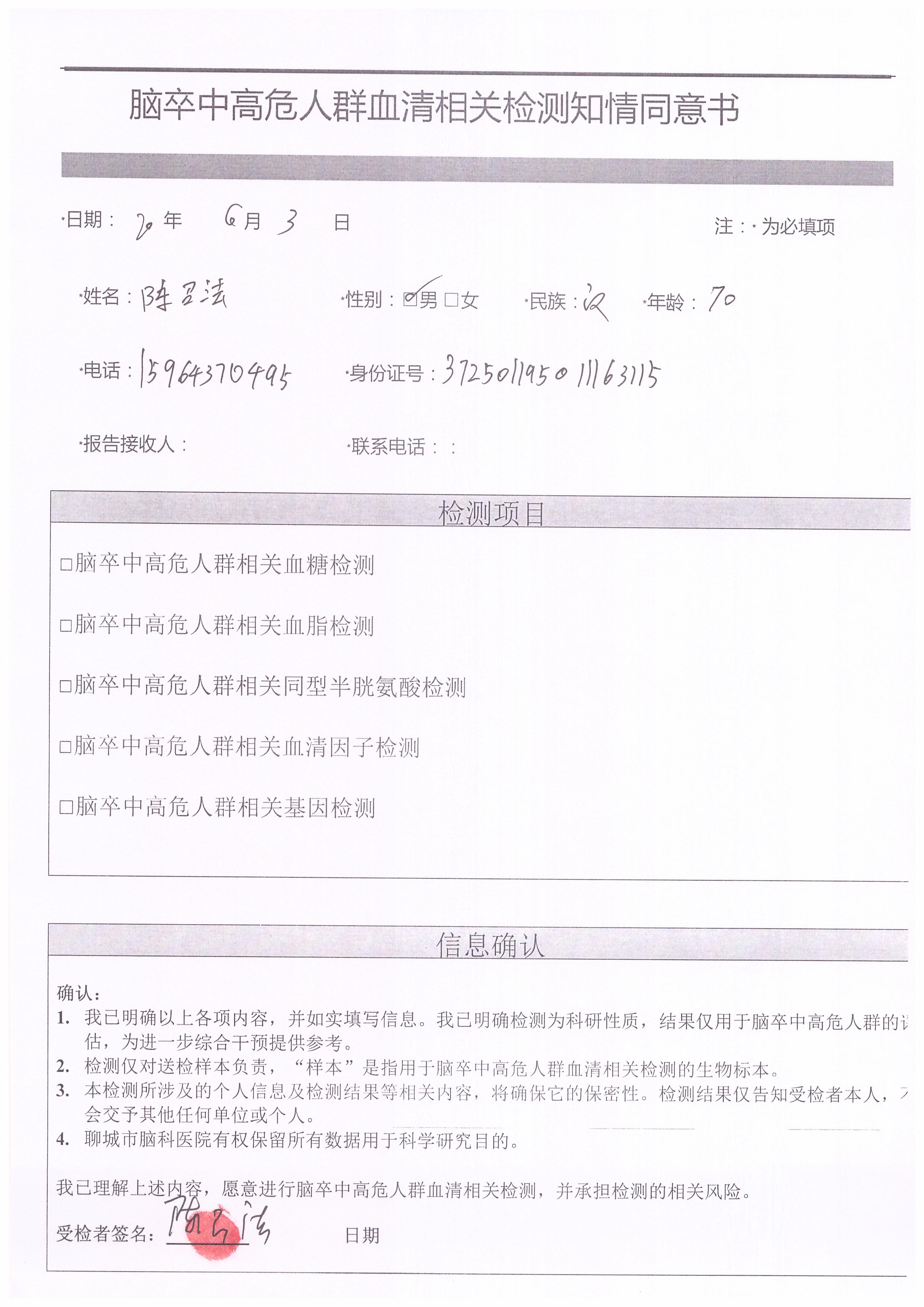

Supplement: Supplementary file 14 — Supplementary file14 (ZIP 27750 KB) [file 10528_2023_10431_MOESM14_ESM.zip › ╓¬╟Θ═1⁄4╥Γ╩Θ12/╡┌╥╗▓┐╖╓í┐/019.jpg]

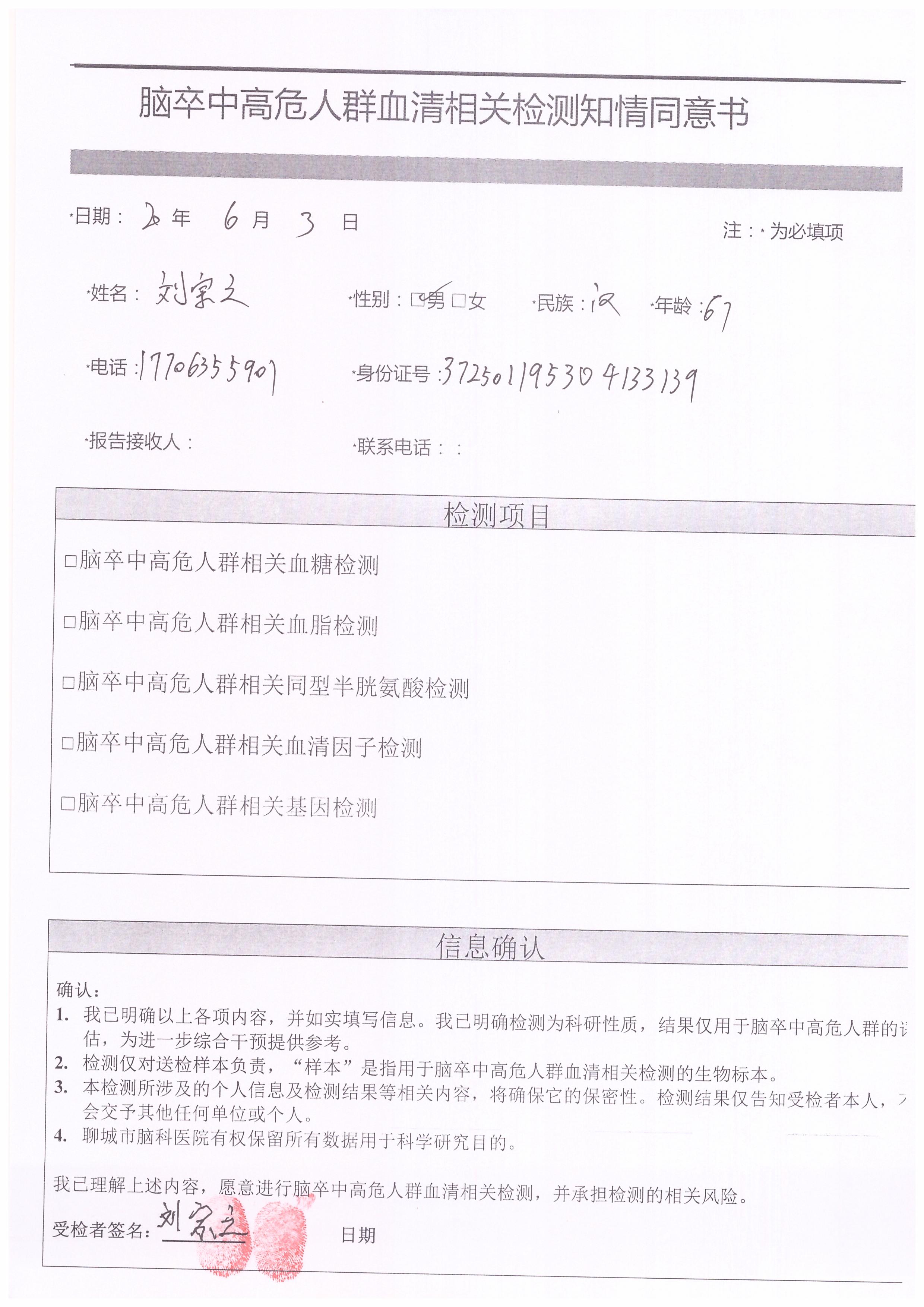

Supplement: Supplementary file 14 — Supplementary file14 (ZIP 27750 KB) [file 10528_2023_10431_MOESM14_ESM.zip › ╓¬╟Θ═1⁄4╥Γ╩Θ12/╡┌╥╗▓┐╖╓í┐/020.jpg]

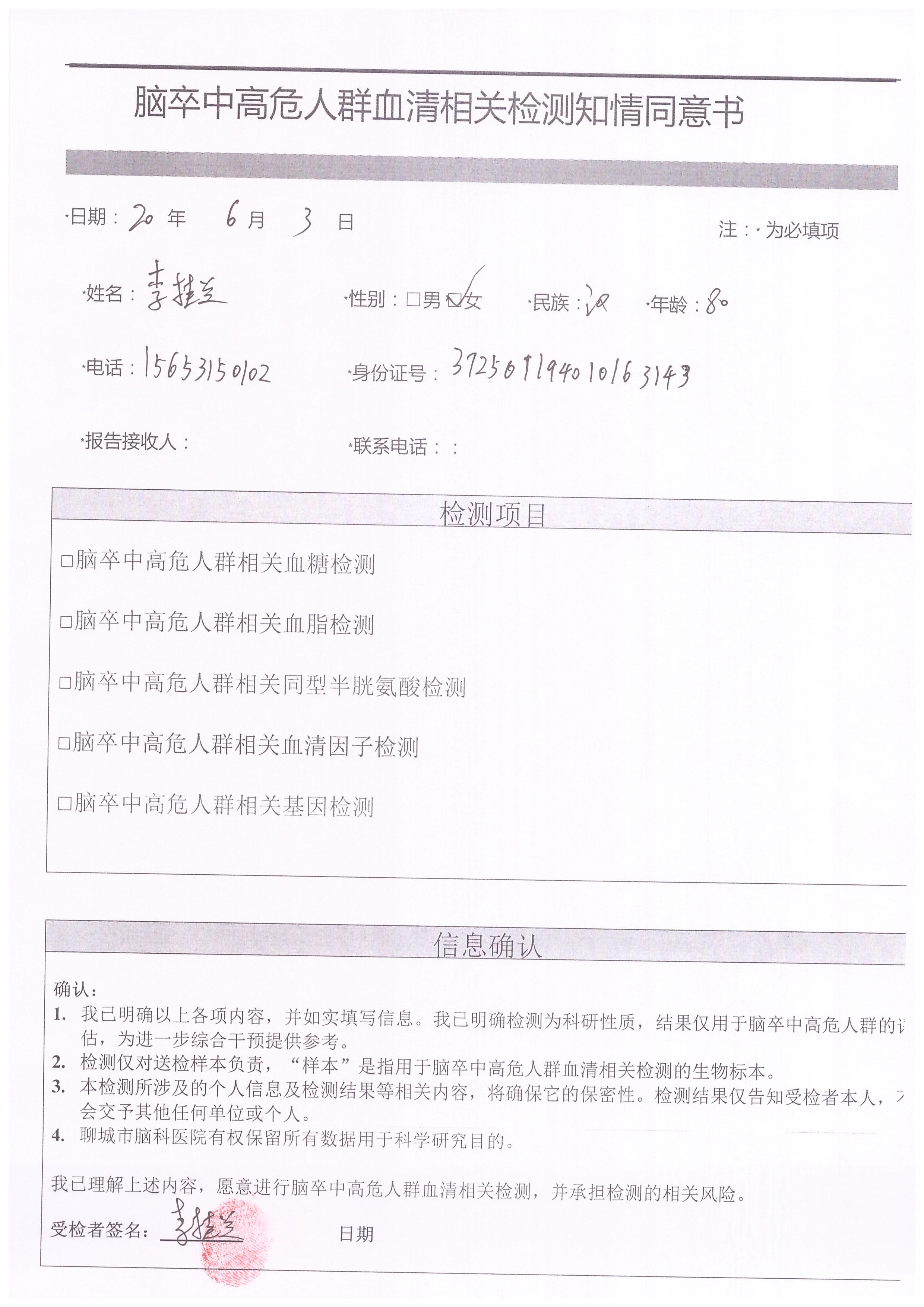

Supplement: Supplementary file 14 — Supplementary file14 (ZIP 27750 KB) [file 10528_2023_10431_MOESM14_ESM.zip › ╓¬╟Θ═1⁄4╥Γ╩Θ12/╡┌╥╗▓┐╖╓í┐/021.jpg]

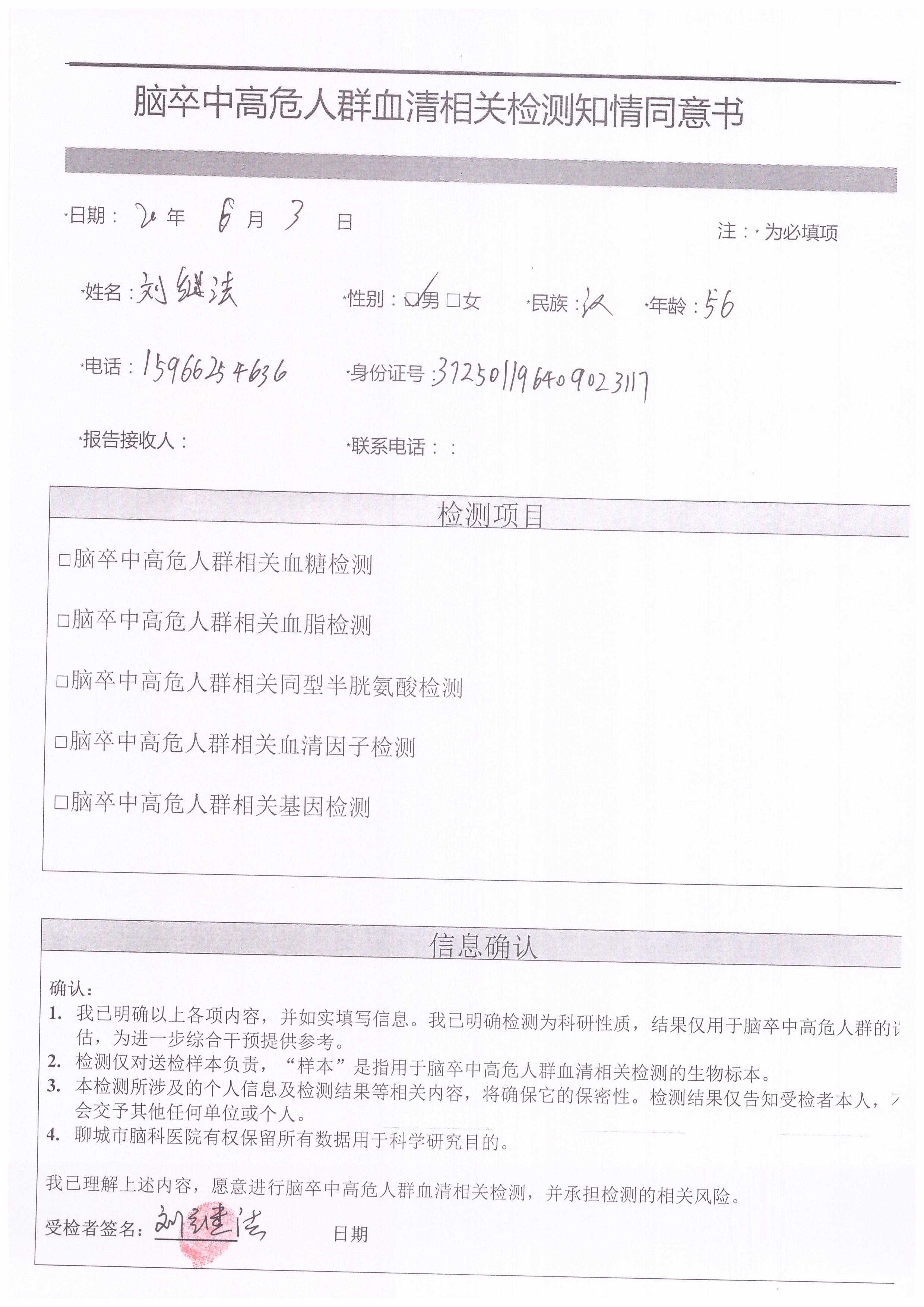

Supplement: Supplementary file 14 — Supplementary file14 (ZIP 27750 KB) [file 10528_2023_10431_MOESM14_ESM.zip › ╓¬╟Θ═1⁄4╥Γ╩Θ12/╡┌╥╗▓┐╖╓í┐/022.jpg]

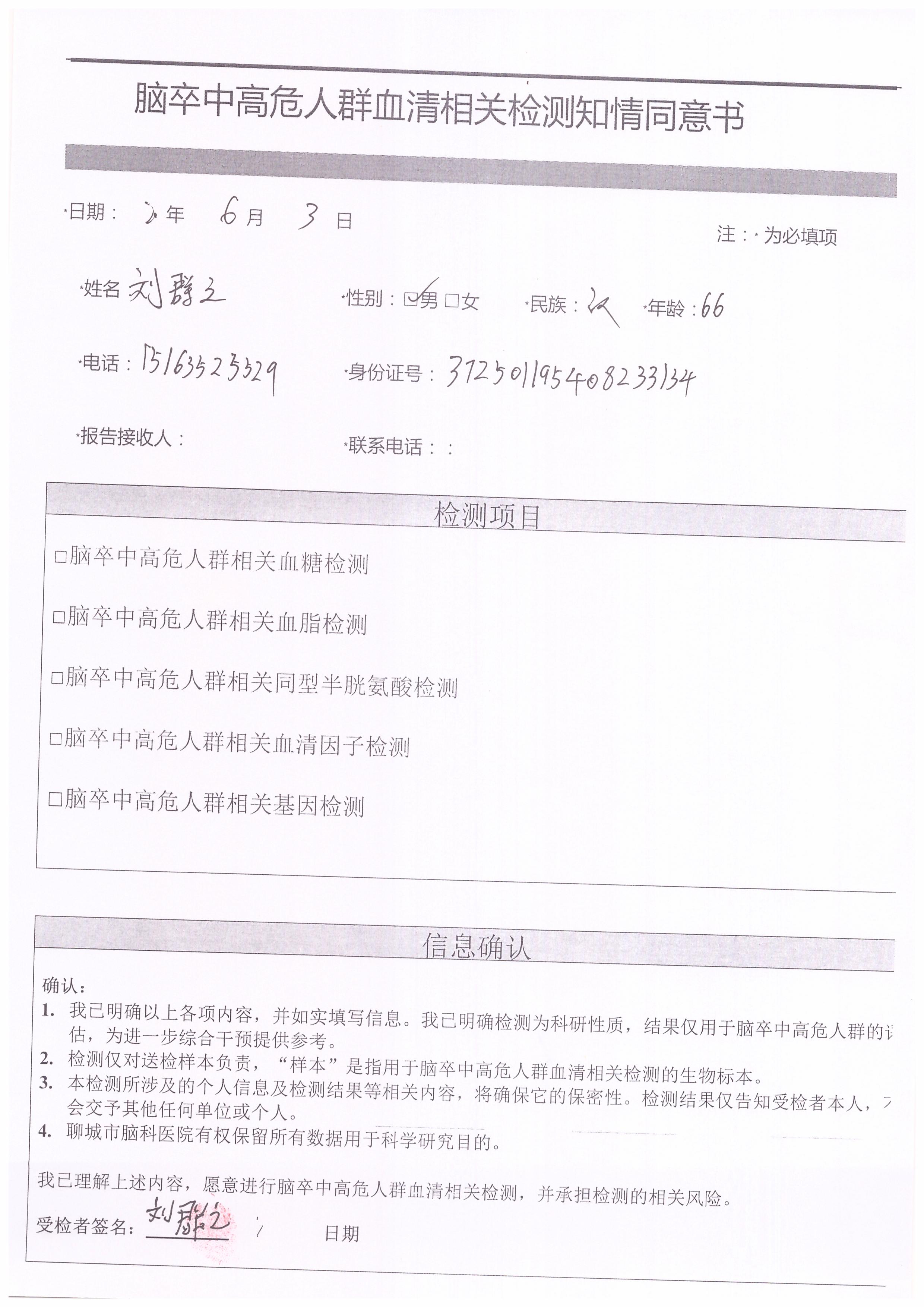

Supplement: Supplementary file 14 — Supplementary file14 (ZIP 27750 KB) [file 10528_2023_10431_MOESM14_ESM.zip › ╓¬╟Θ═1⁄4╥Γ╩Θ12/╡┌╥╗▓┐╖╓í┐/023.jpg]

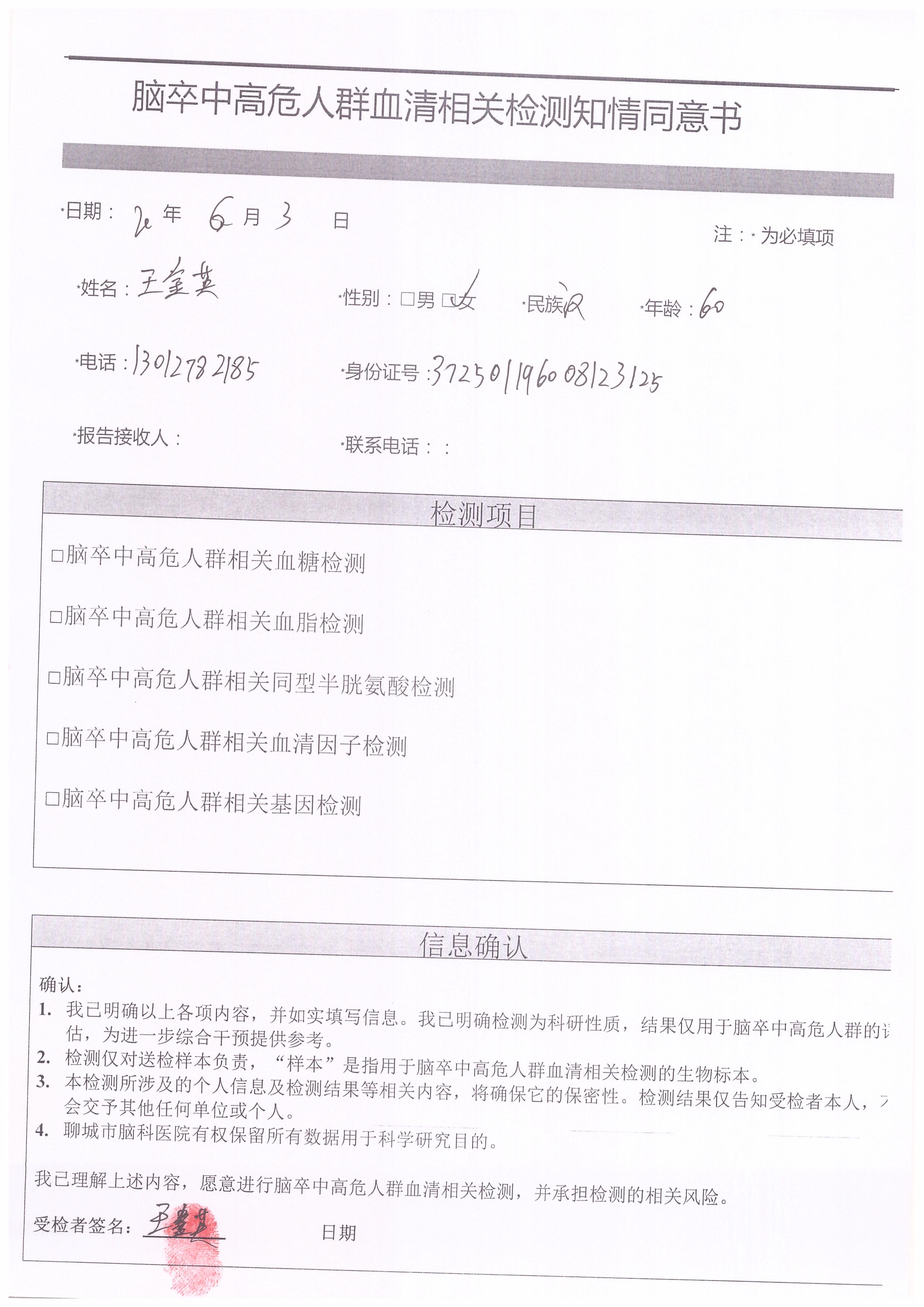

Supplement: Supplementary file 14 — Supplementary file14 (ZIP 27750 KB) [file 10528_2023_10431_MOESM14_ESM.zip › ╓¬╟Θ═1⁄4╥Γ╩Θ12/╡┌╥╗▓┐╖╓í┐/024.jpg]

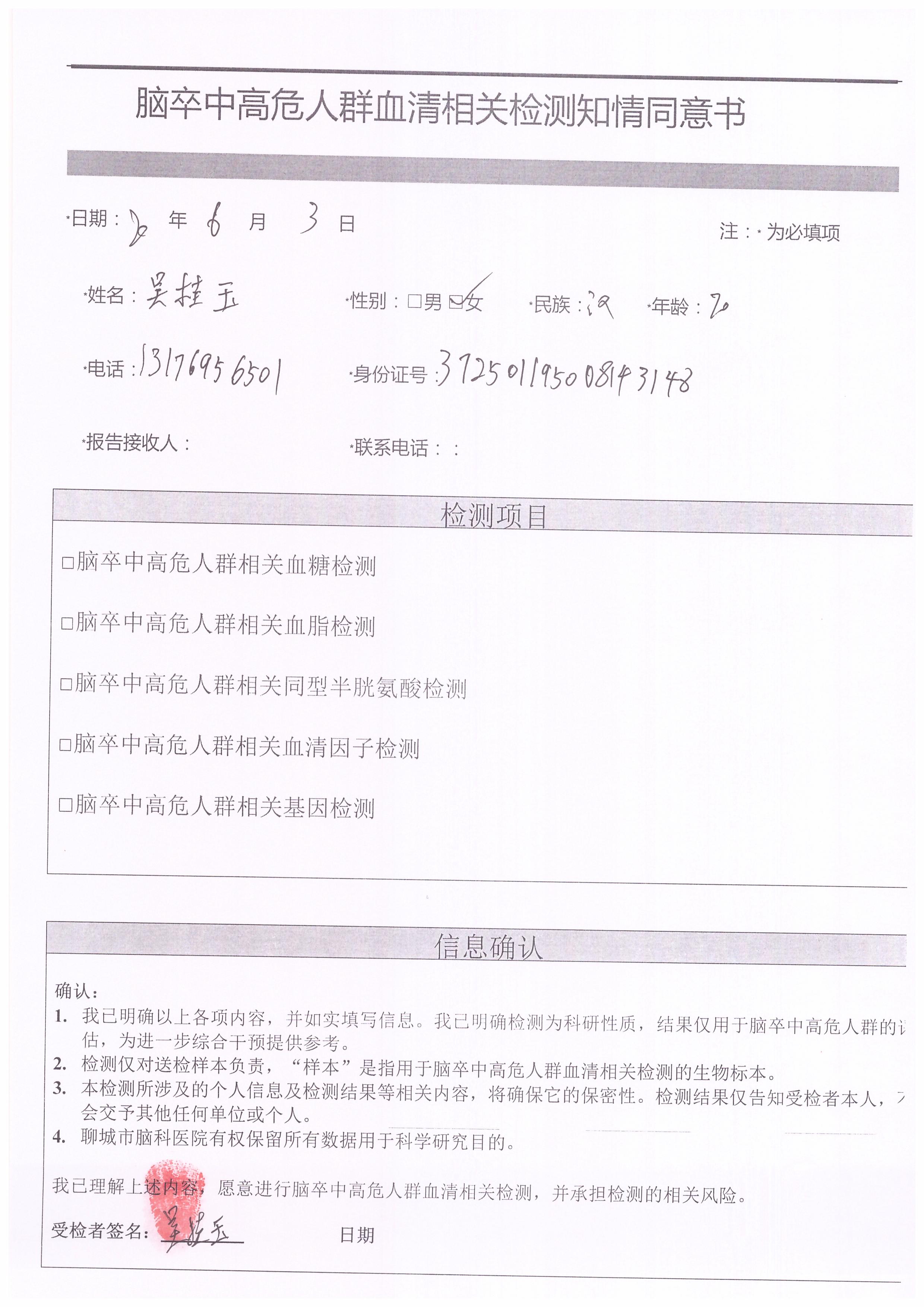

Supplement: Supplementary file 14 — Supplementary file14 (ZIP 27750 KB) [file 10528_2023_10431_MOESM14_ESM.zip › ╓¬╟Θ═1⁄4╥Γ╩Θ12/╡┌╥╗▓┐╖╓í┐/025.jpg]
