# Supplementary figures and images for: The Association Between Thymidylate Synthase Gene Polymorphisms and the Risk of Ischemic Stroke in Chinese Han Population (part 6 of 6)
Source: Biochem Genet. 2023 Jun 28;62(1):468–84. doi: 10.1007/s10528-023-10431-8 (PMC10901929; doi:10.1007/s10528-023-10431-8)

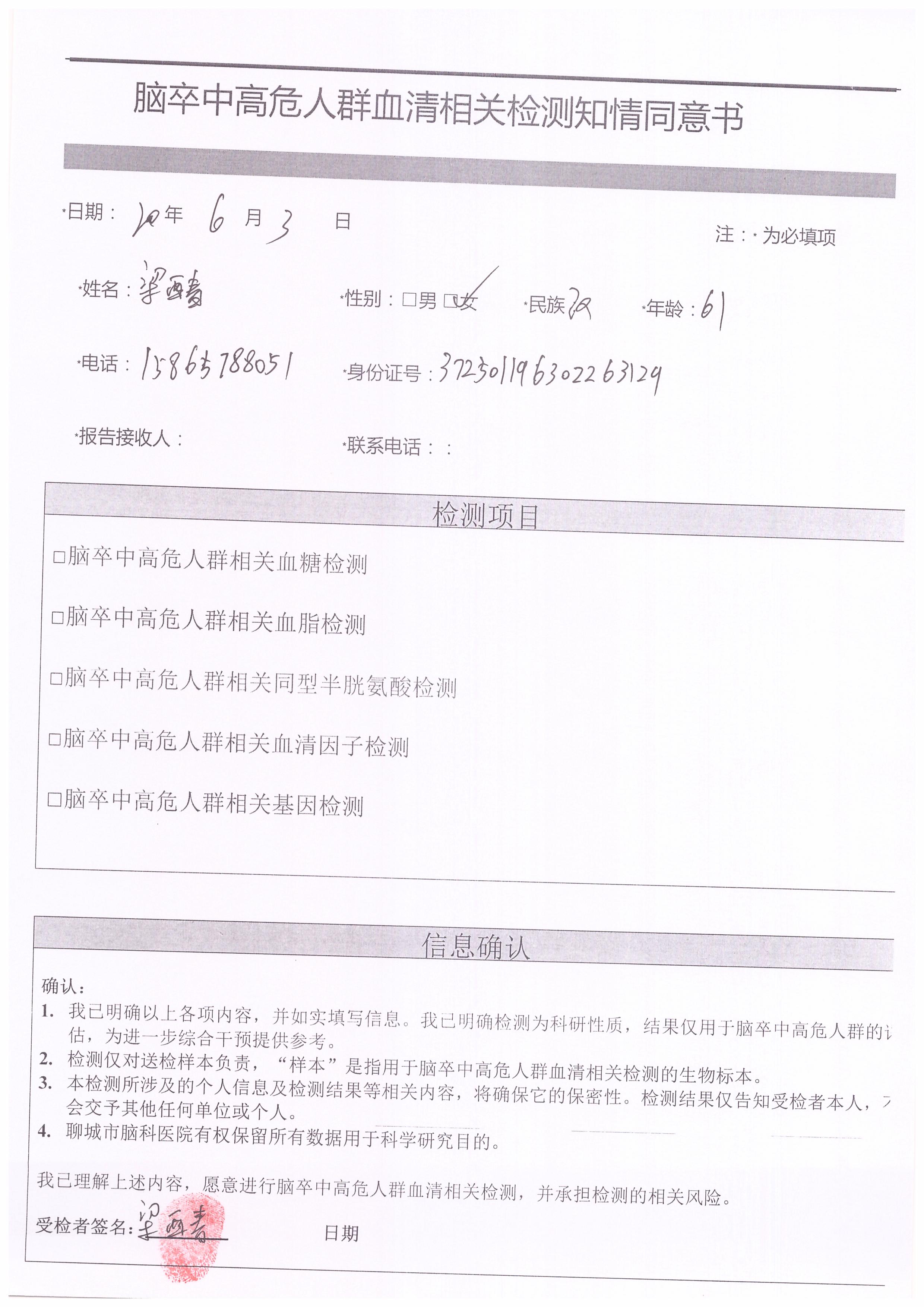

Supplement: Supplementary file 14 — Supplementary file14 (ZIP 27750 KB) [file 10528_2023_10431_MOESM14_ESM.zip › ╓¬╟Θ═1⁄4╥Γ╩Θ12/╡┌╥╗▓┐╖╓í┐/026.jpg]

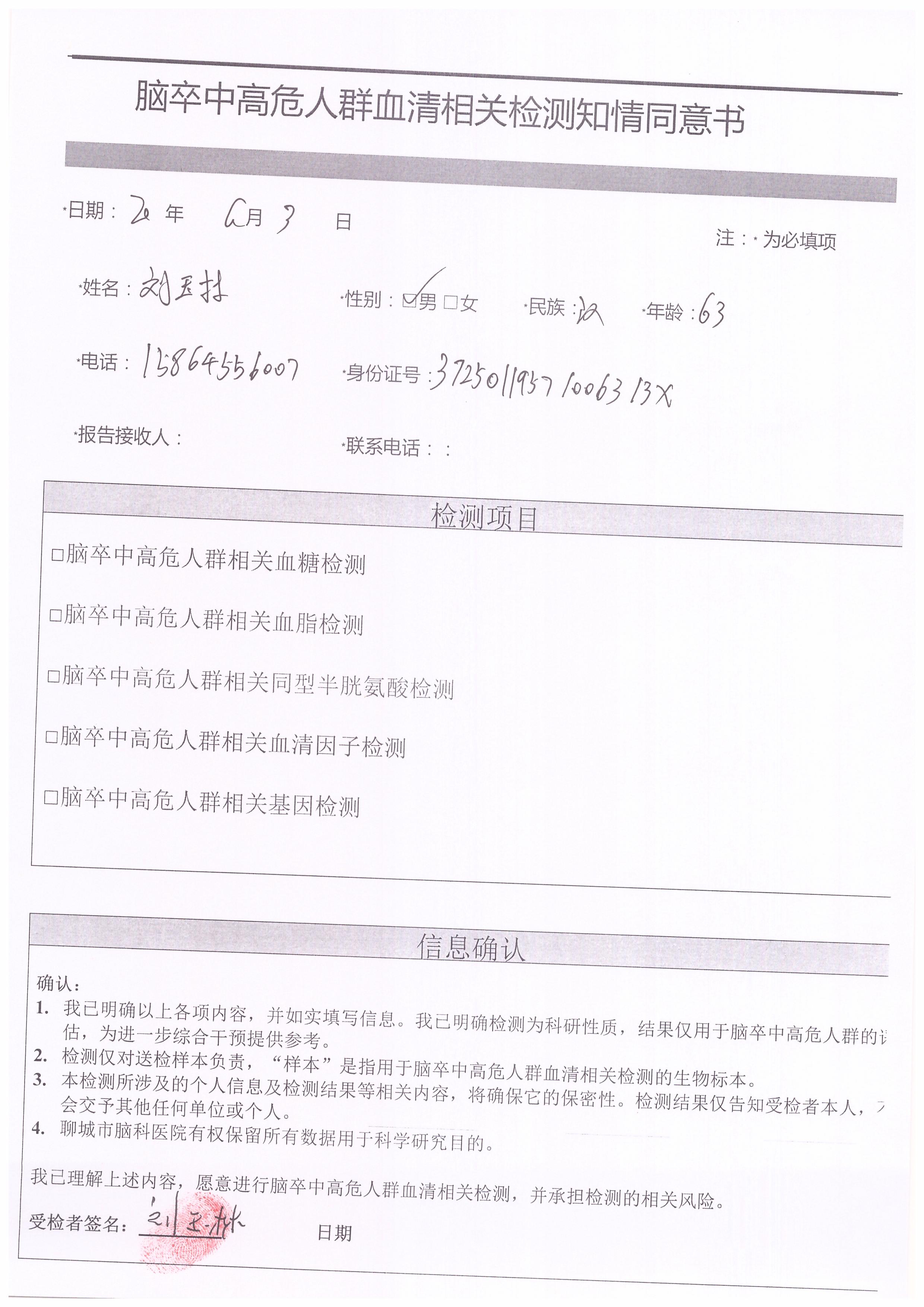

Supplement: Supplementary file 14 — Supplementary file14 (ZIP 27750 KB) [file 10528_2023_10431_MOESM14_ESM.zip › ╓¬╟Θ═1⁄4╥Γ╩Θ12/╡┌╥╗▓┐╖╓í┐/027.jpg]

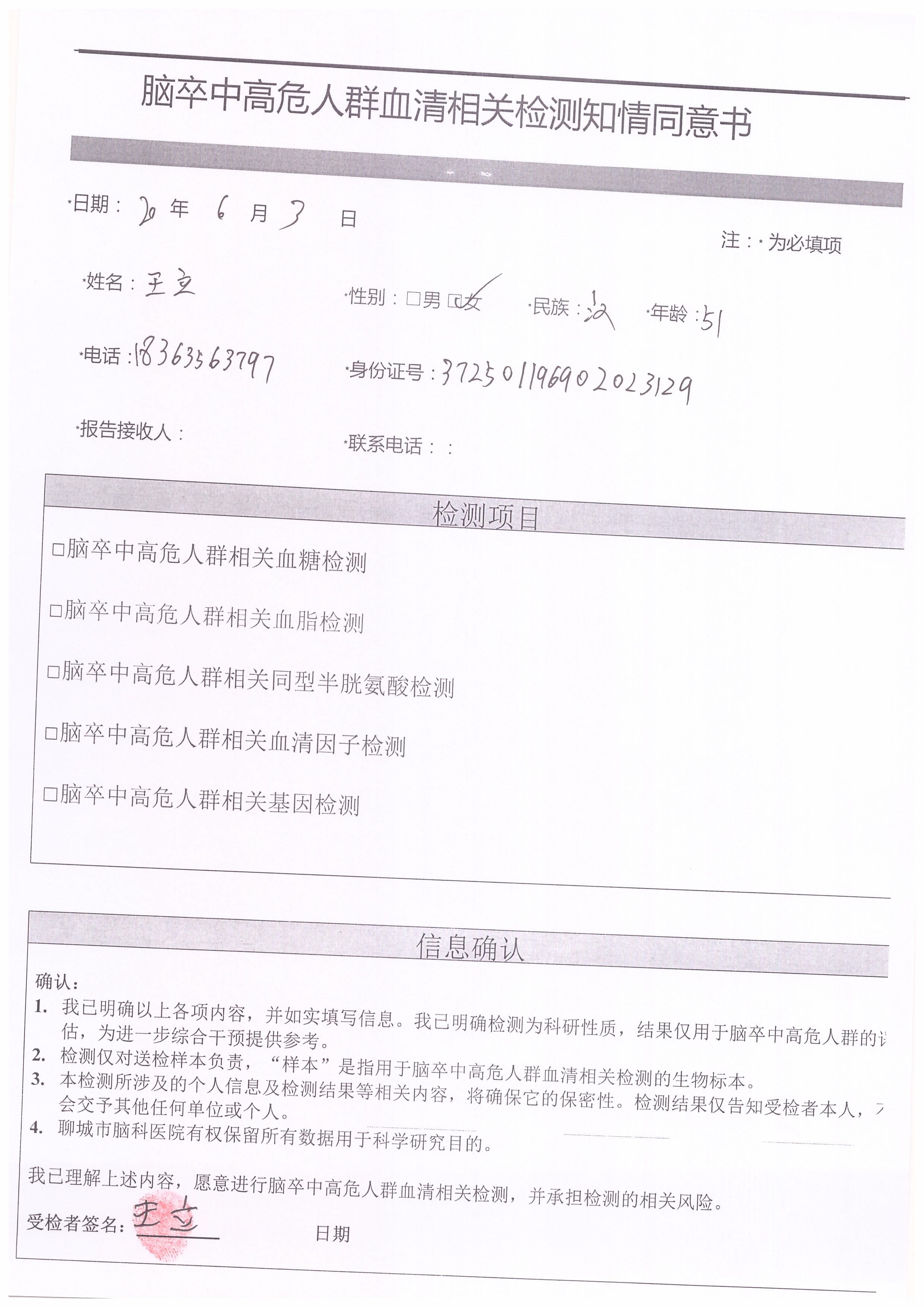

Supplement: Supplementary file 14 — Supplementary file14 (ZIP 27750 KB) [file 10528_2023_10431_MOESM14_ESM.zip › ╓¬╟Θ═1⁄4╥Γ╩Θ12/╡┌╢■▓┐╖╓/001.jpg]

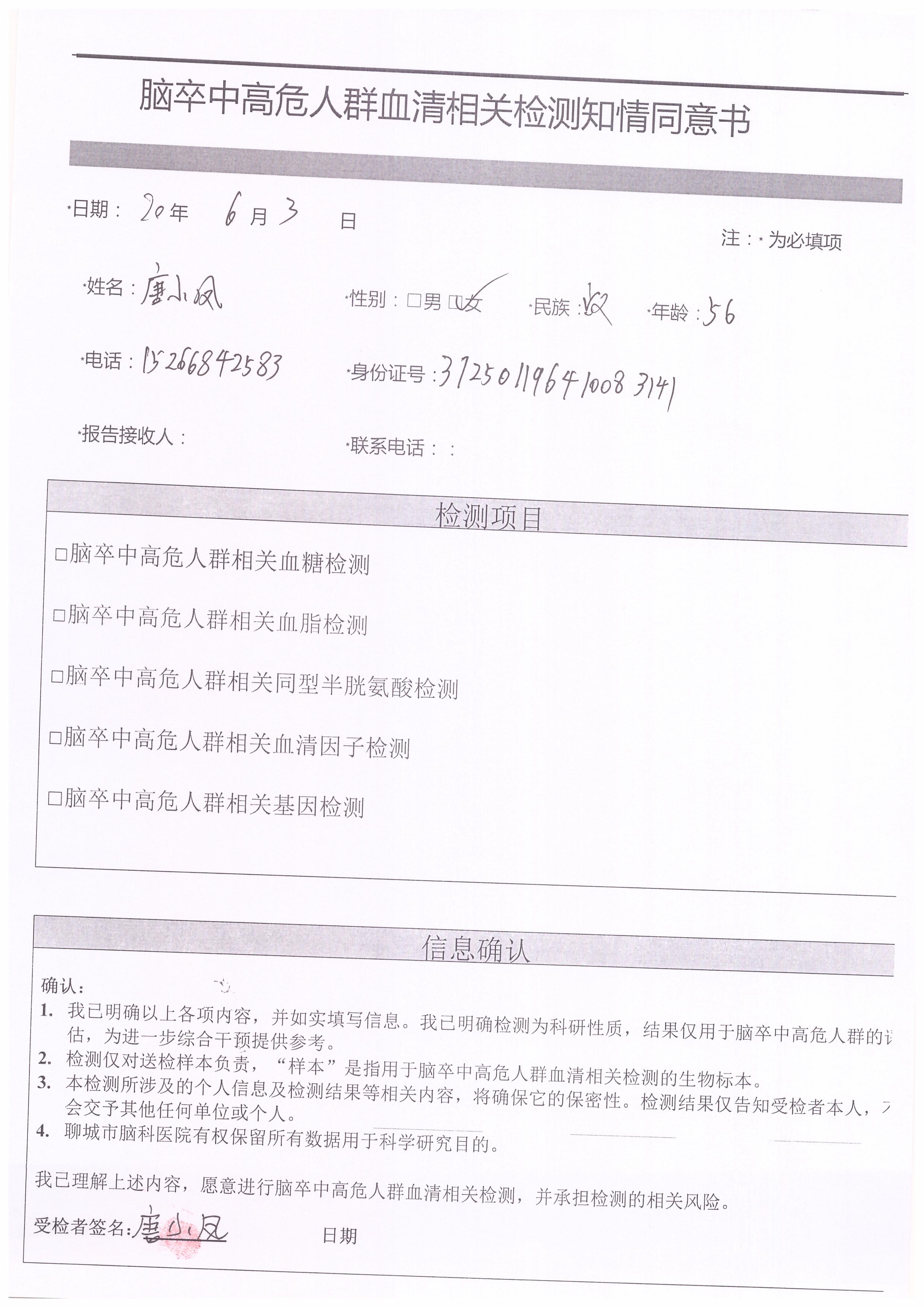

Supplement: Supplementary file 14 — Supplementary file14 (ZIP 27750 KB) [file 10528_2023_10431_MOESM14_ESM.zip › ╓¬╟Θ═1⁄4╥Γ╩Θ12/╡┌╢■▓┐╖╓/002.jpg]

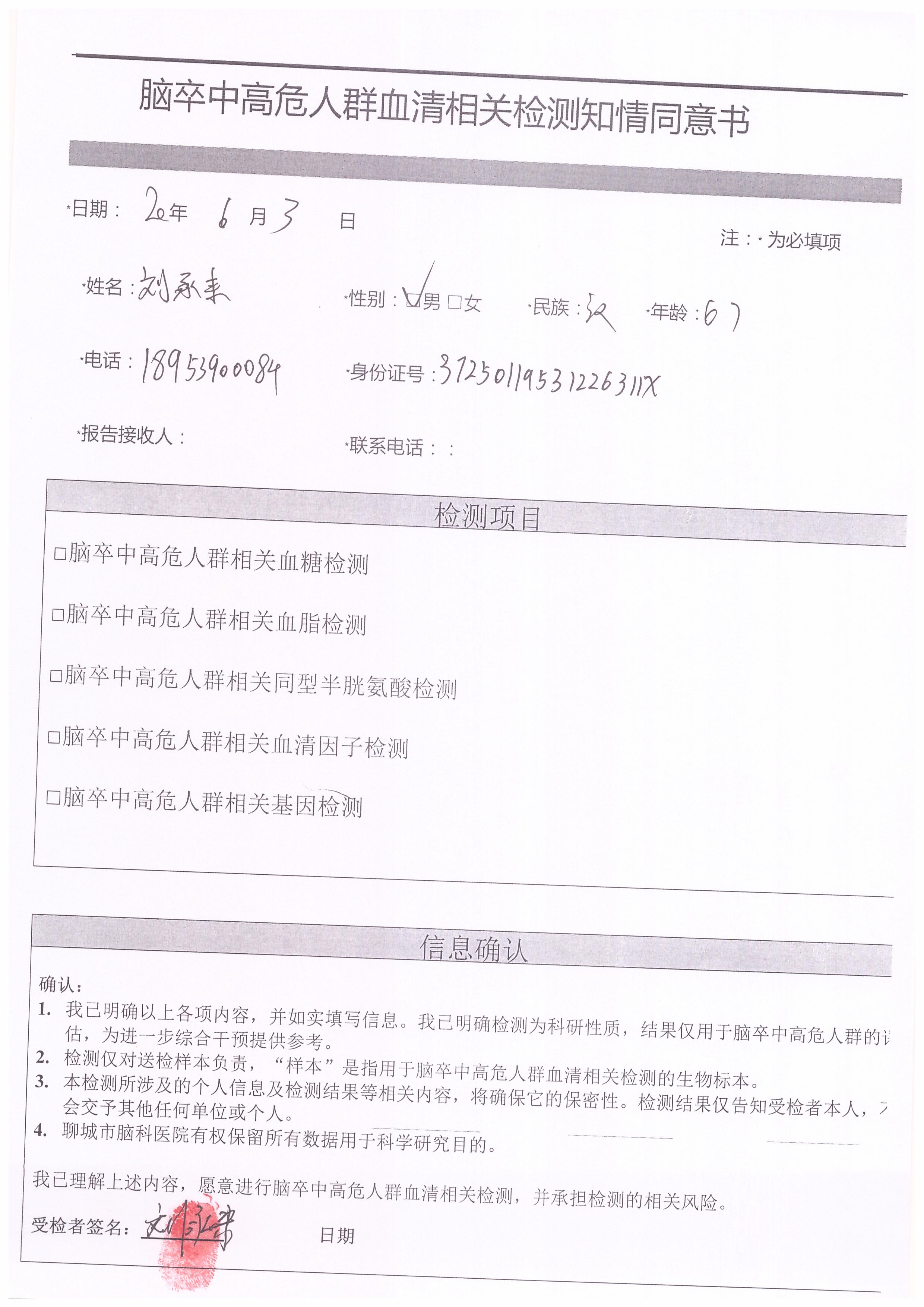

Supplement: Supplementary file 14 — Supplementary file14 (ZIP 27750 KB) [file 10528_2023_10431_MOESM14_ESM.zip › ╓¬╟Θ═1⁄4╥Γ╩Θ12/╡┌╢■▓┐╖╓/003.jpg]

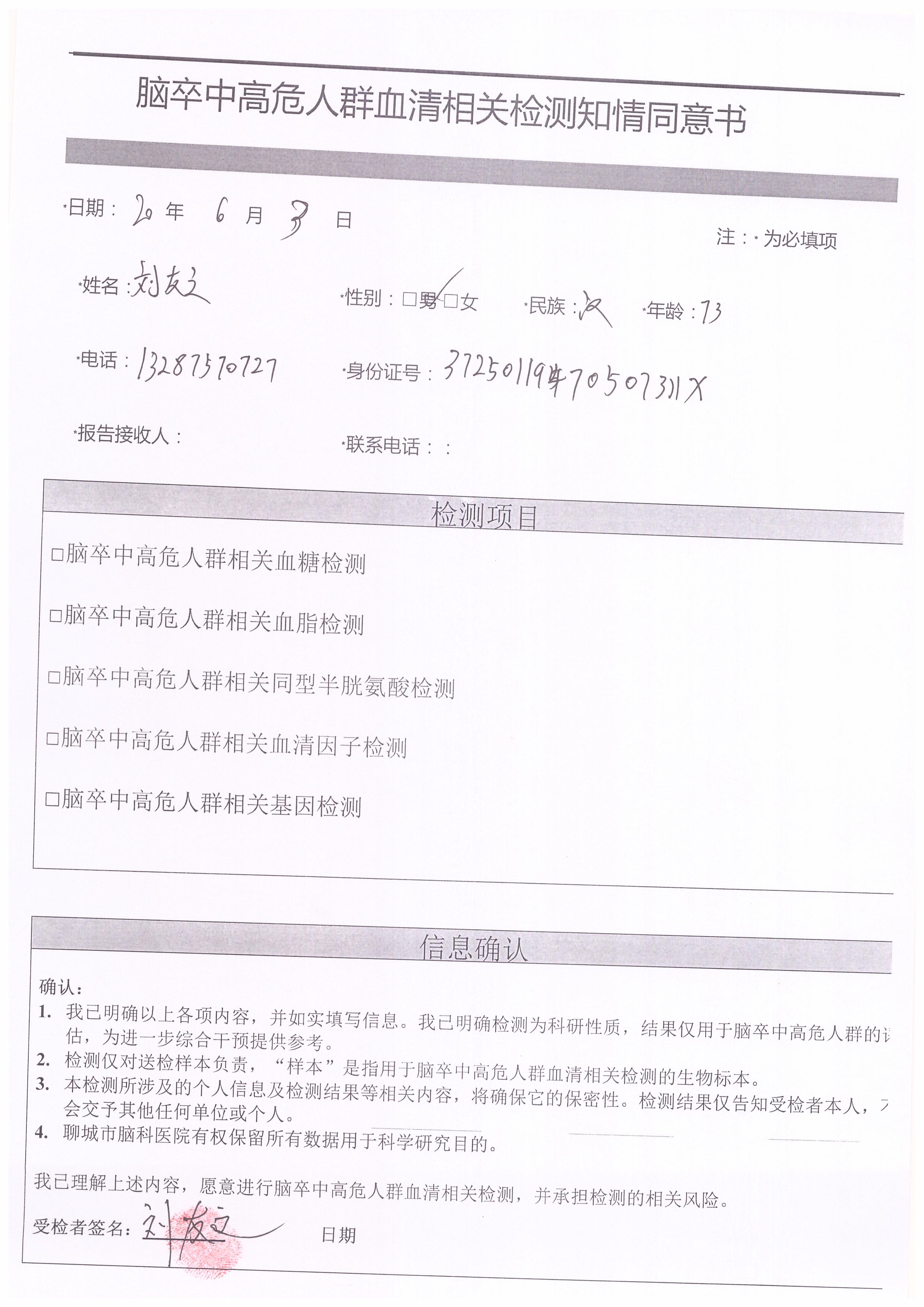

Supplement: Supplementary file 14 — Supplementary file14 (ZIP 27750 KB) [file 10528_2023_10431_MOESM14_ESM.zip › ╓¬╟Θ═1⁄4╥Γ╩Θ12/╡┌╢■▓┐╖╓/004.jpg]

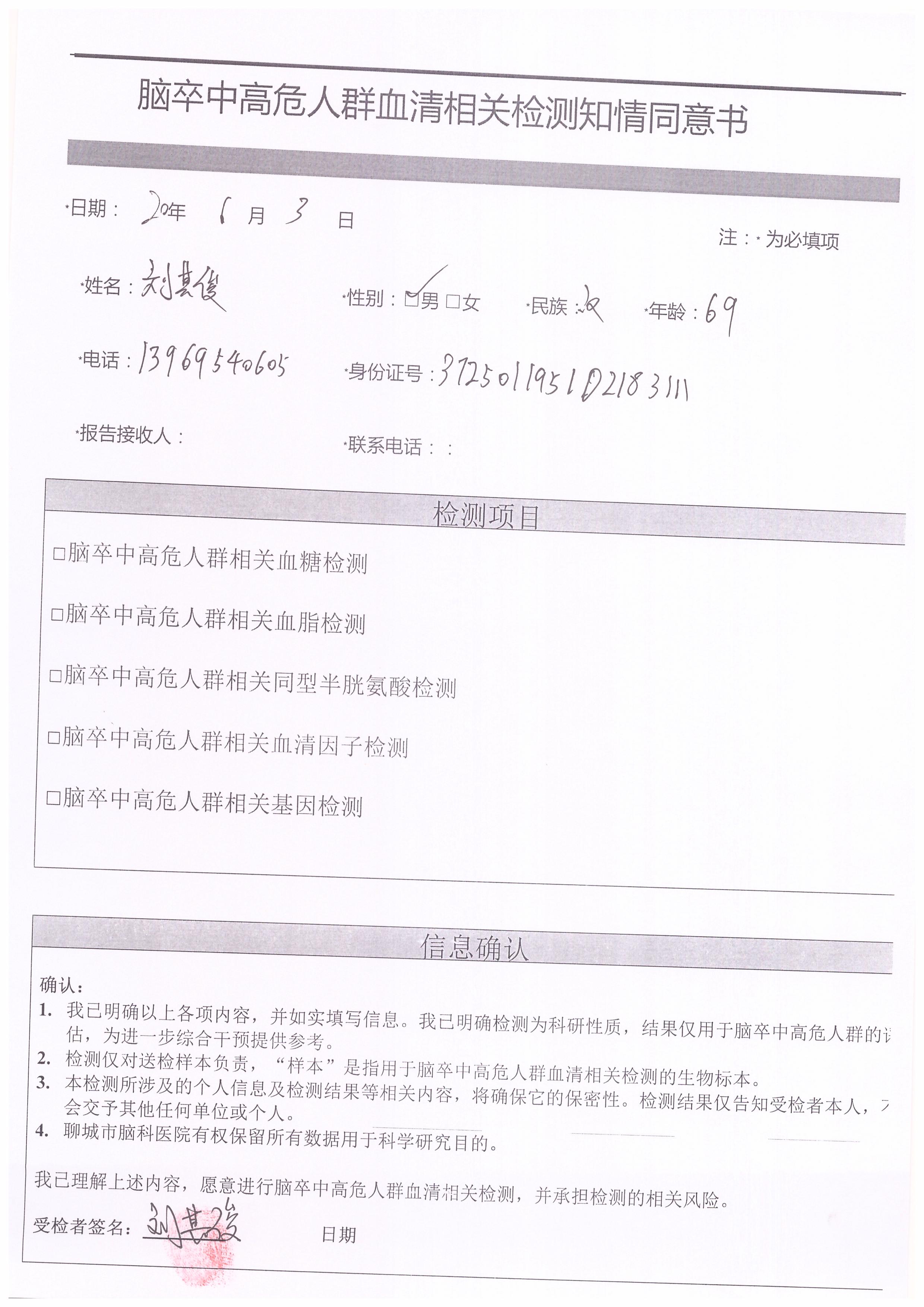

Supplement: Supplementary file 14 — Supplementary file14 (ZIP 27750 KB) [file 10528_2023_10431_MOESM14_ESM.zip › ╓¬╟Θ═1⁄4╥Γ╩Θ12/╡┌╢■▓┐╖╓/005.jpg]

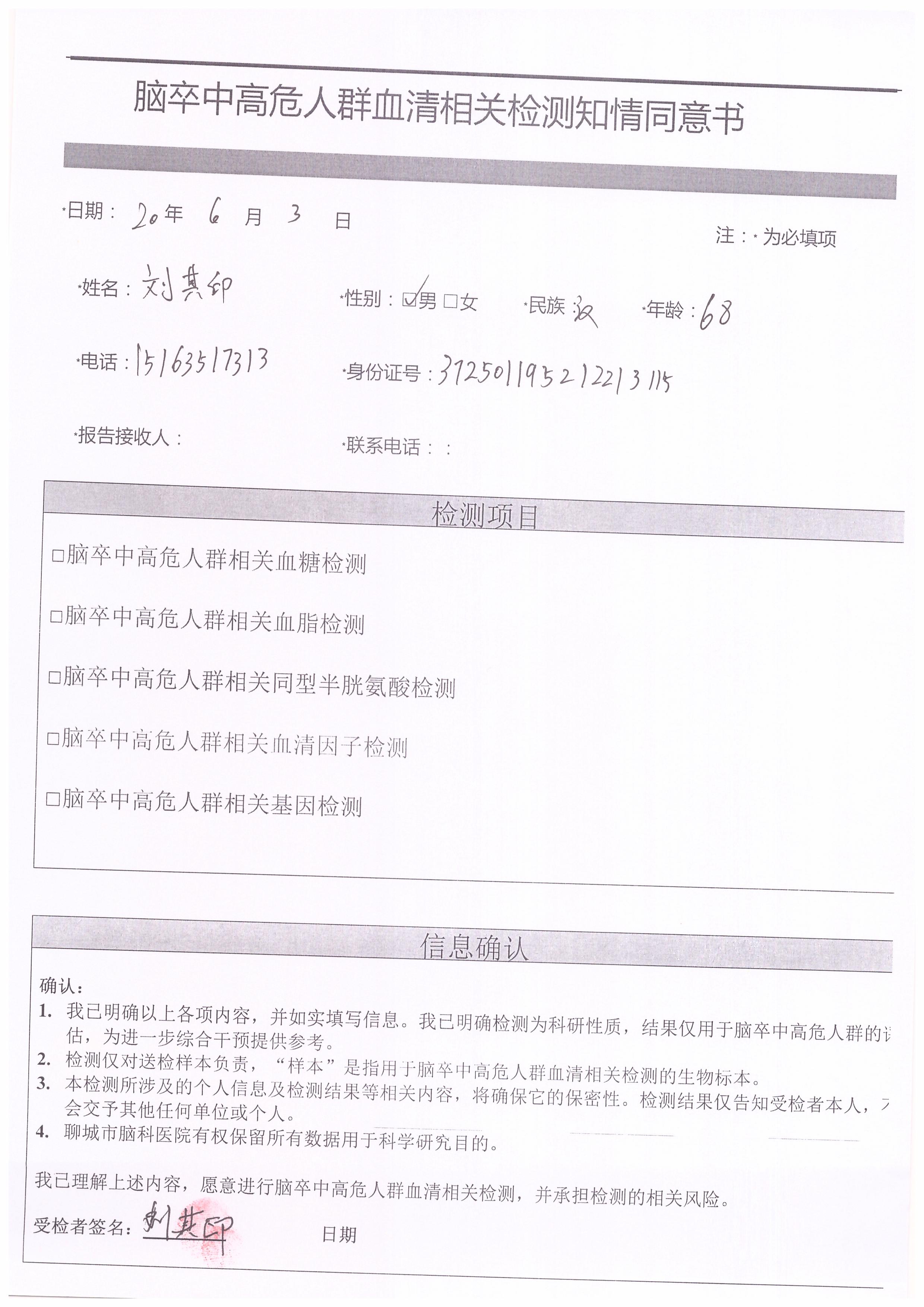

Supplement: Supplementary file 14 — Supplementary file14 (ZIP 27750 KB) [file 10528_2023_10431_MOESM14_ESM.zip › ╓¬╟Θ═1⁄4╥Γ╩Θ12/╡┌╢■▓┐╖╓/006.jpg]

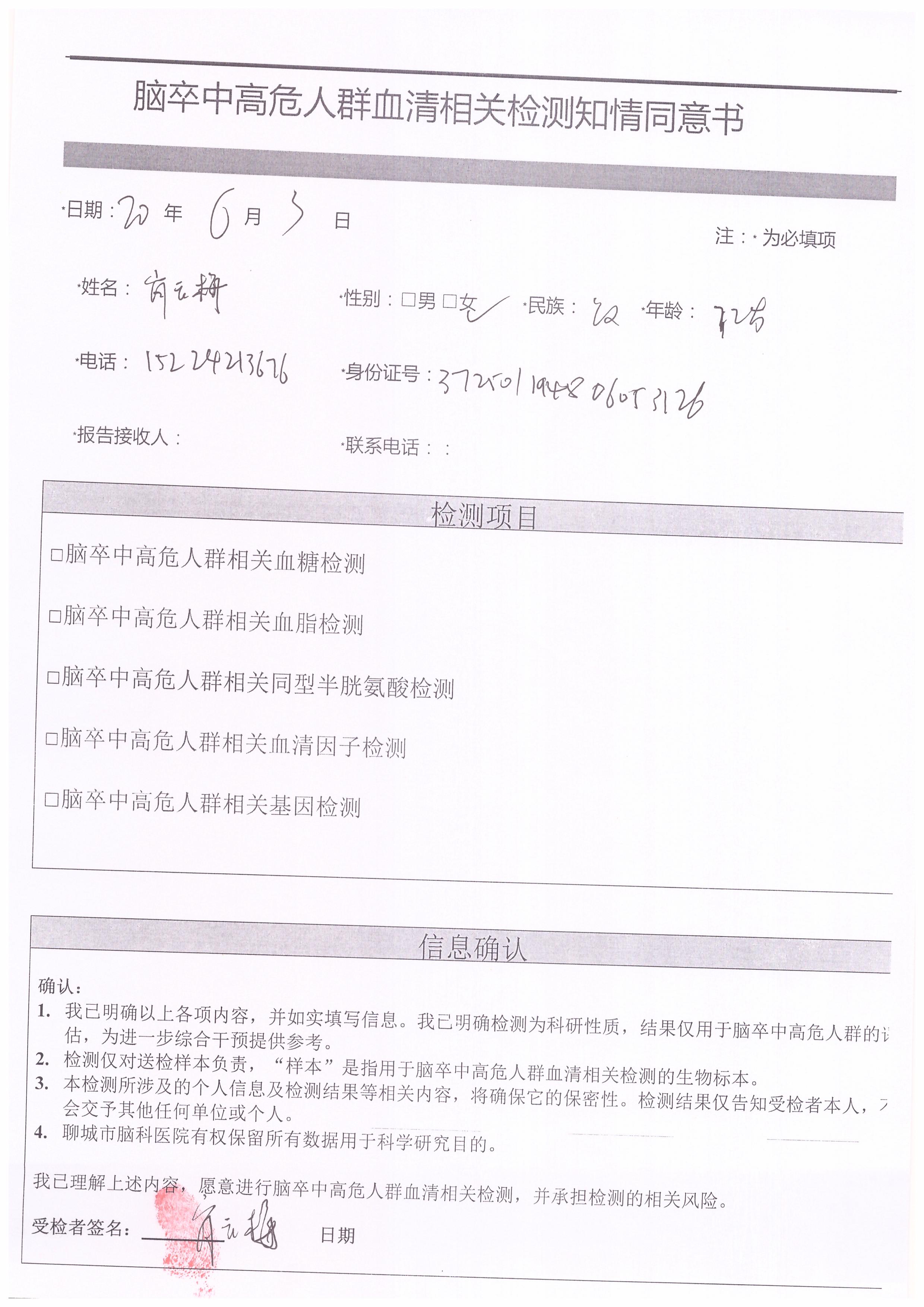

Supplement: Supplementary file 14 — Supplementary file14 (ZIP 27750 KB) [file 10528_2023_10431_MOESM14_ESM.zip › ╓¬╟Θ═1⁄4╥Γ╩Θ12/╡┌╢■▓┐╖╓/007.jpg]

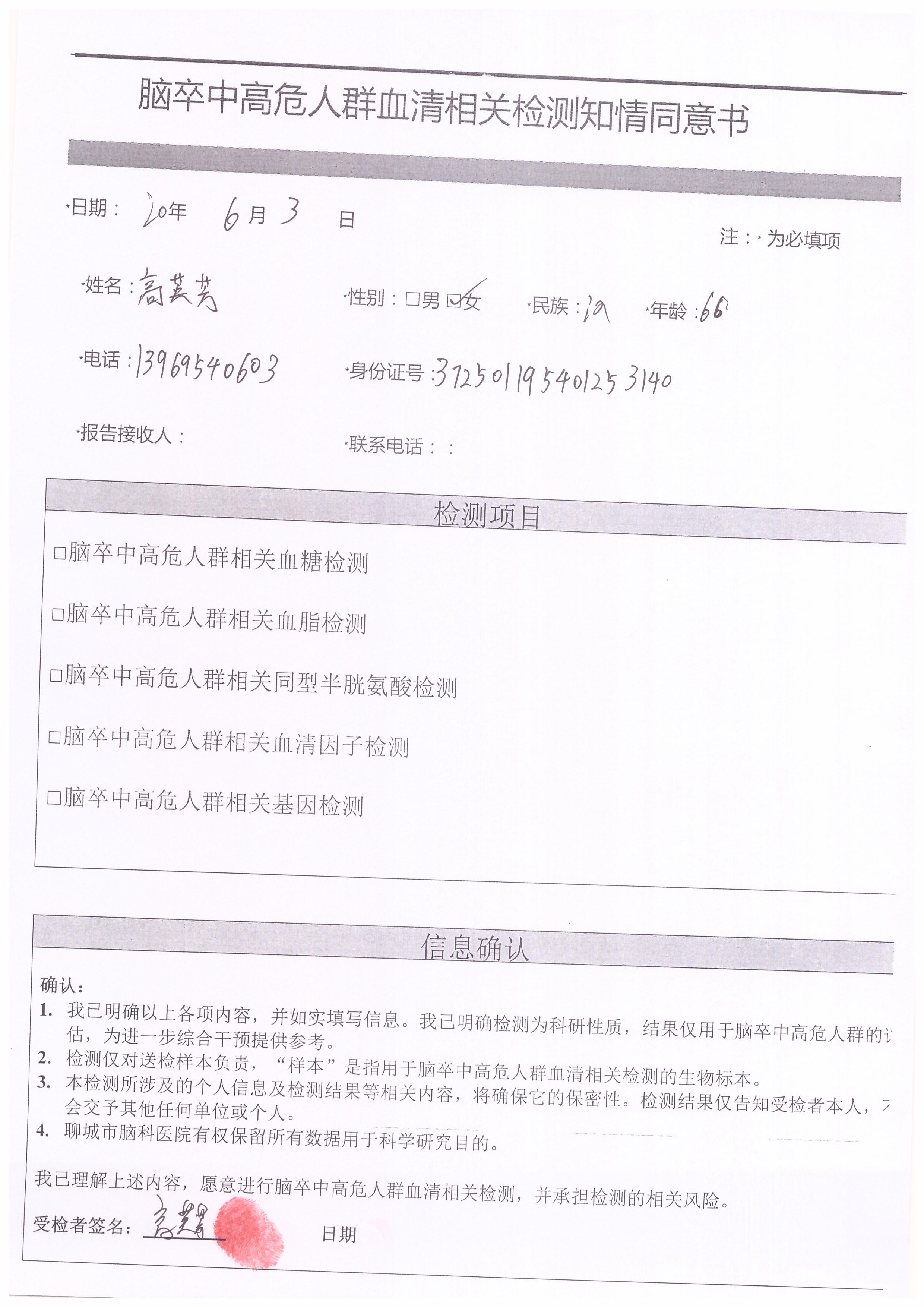

Supplement: Supplementary file 14 — Supplementary file14 (ZIP 27750 KB) [file 10528_2023_10431_MOESM14_ESM.zip › ╓¬╟Θ═1⁄4╥Γ╩Θ12/╡┌╢■▓┐╖╓/008.jpg]

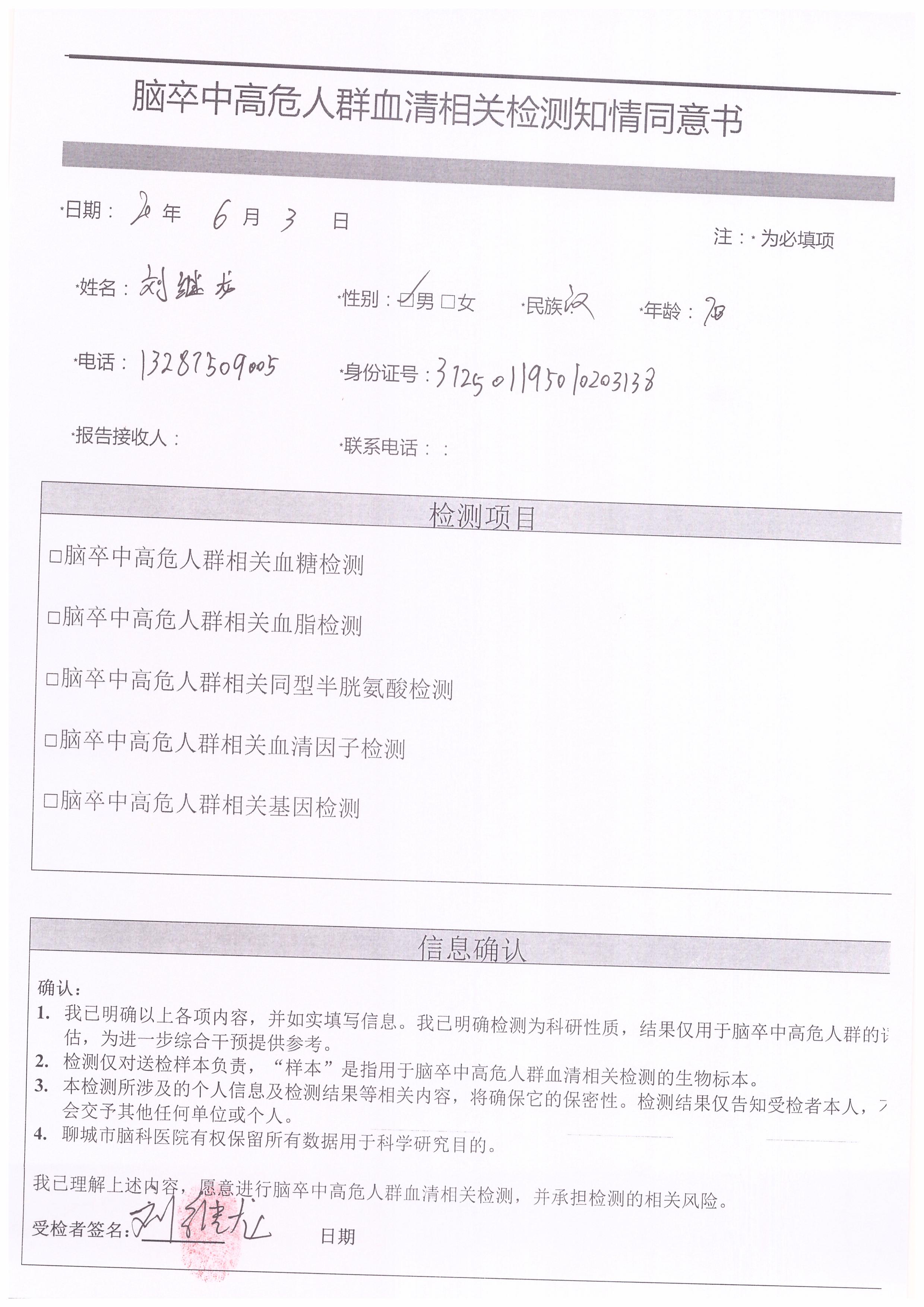

Supplement: Supplementary file 14 — Supplementary file14 (ZIP 27750 KB) [file 10528_2023_10431_MOESM14_ESM.zip › ╓¬╟Θ═1⁄4╥Γ╩Θ12/╡┌╢■▓┐╖╓/009.jpg]

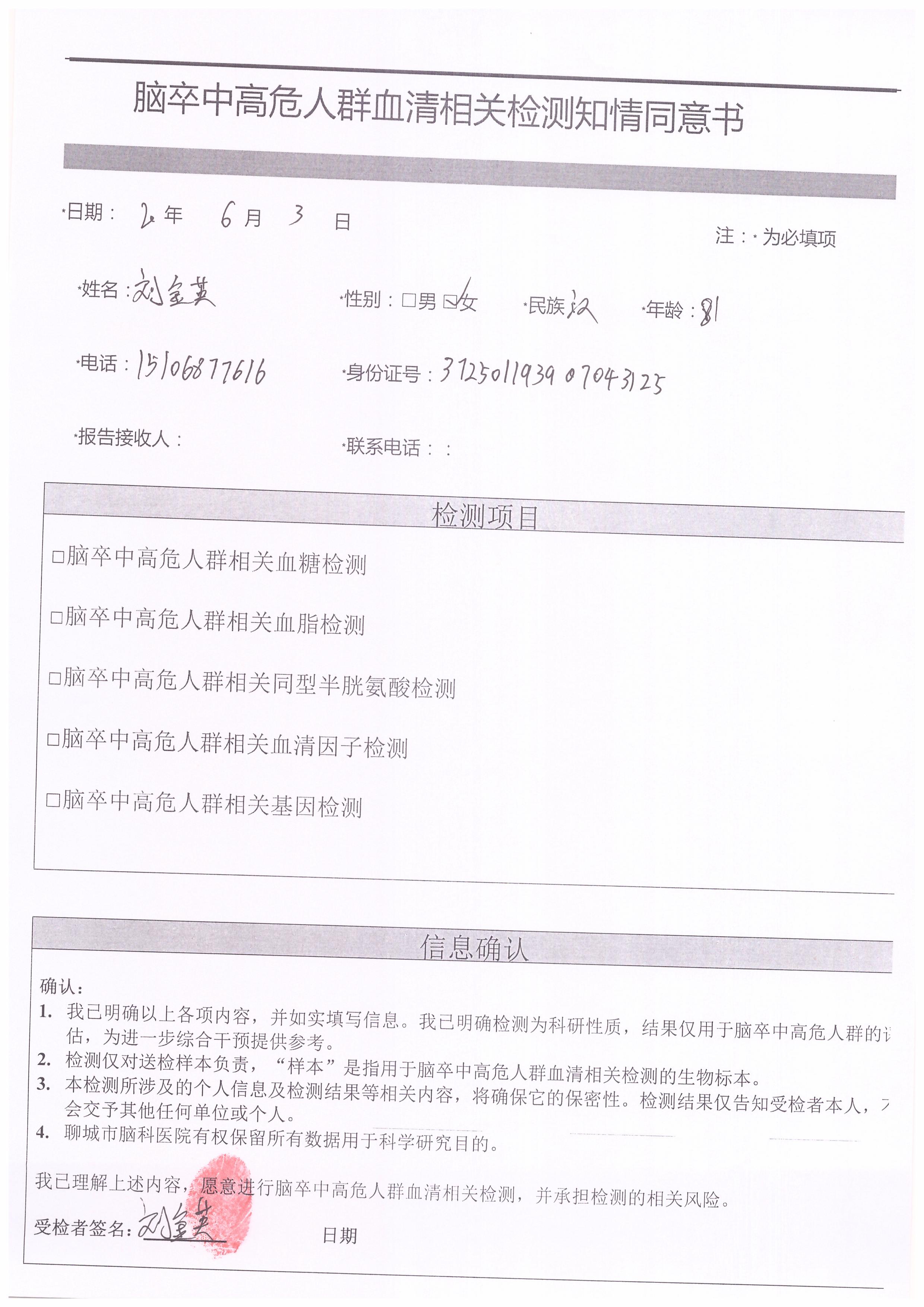

Supplement: Supplementary file 14 — Supplementary file14 (ZIP 27750 KB) [file 10528_2023_10431_MOESM14_ESM.zip › ╓¬╟Θ═1⁄4╥Γ╩Θ12/╡┌╢■▓┐╖╓/010.jpg]

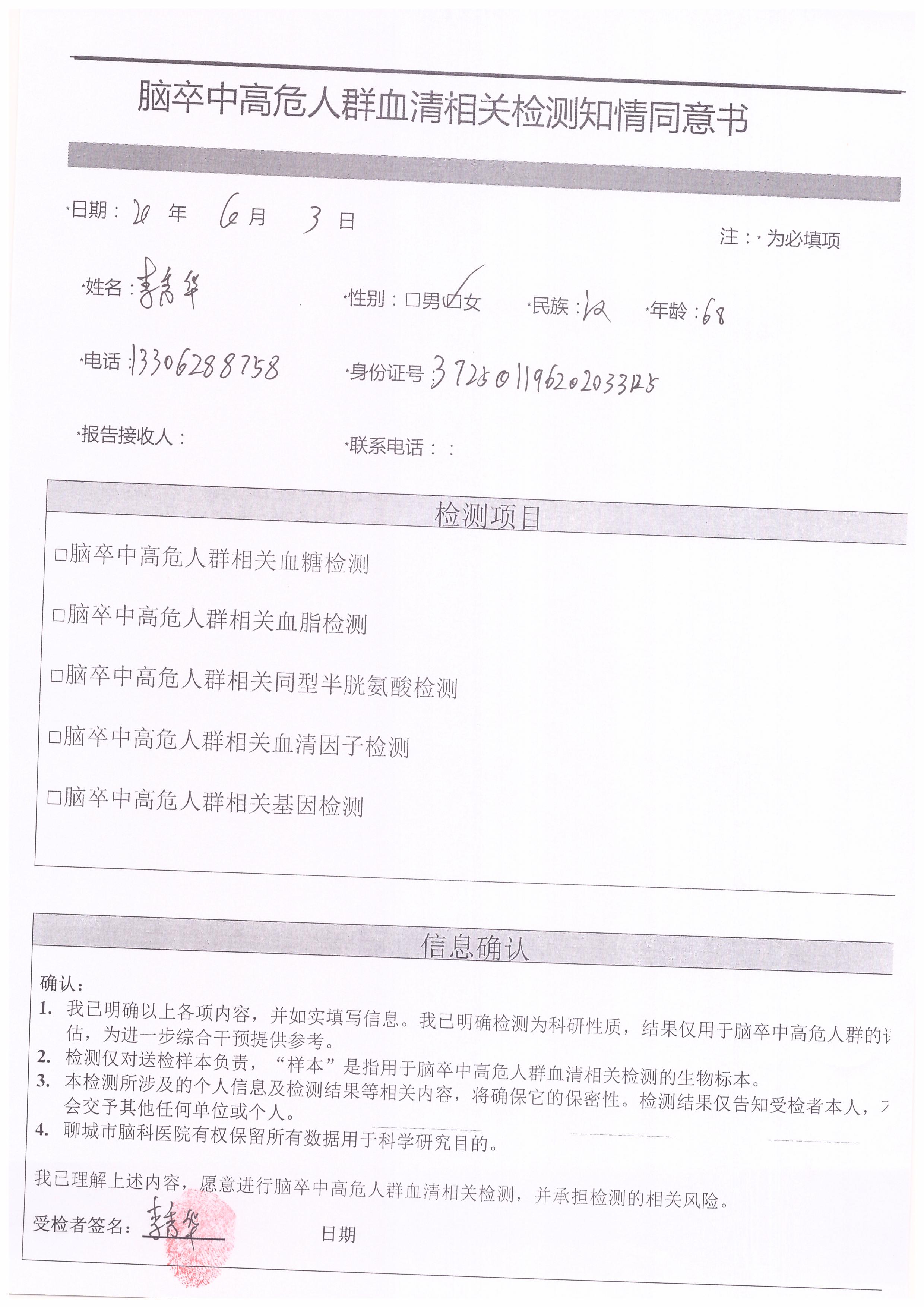

Supplement: Supplementary file 14 — Supplementary file14 (ZIP 27750 KB) [file 10528_2023_10431_MOESM14_ESM.zip › ╓¬╟Θ═1⁄4╥Γ╩Θ12/╡┌╢■▓┐╖╓/011.jpg]

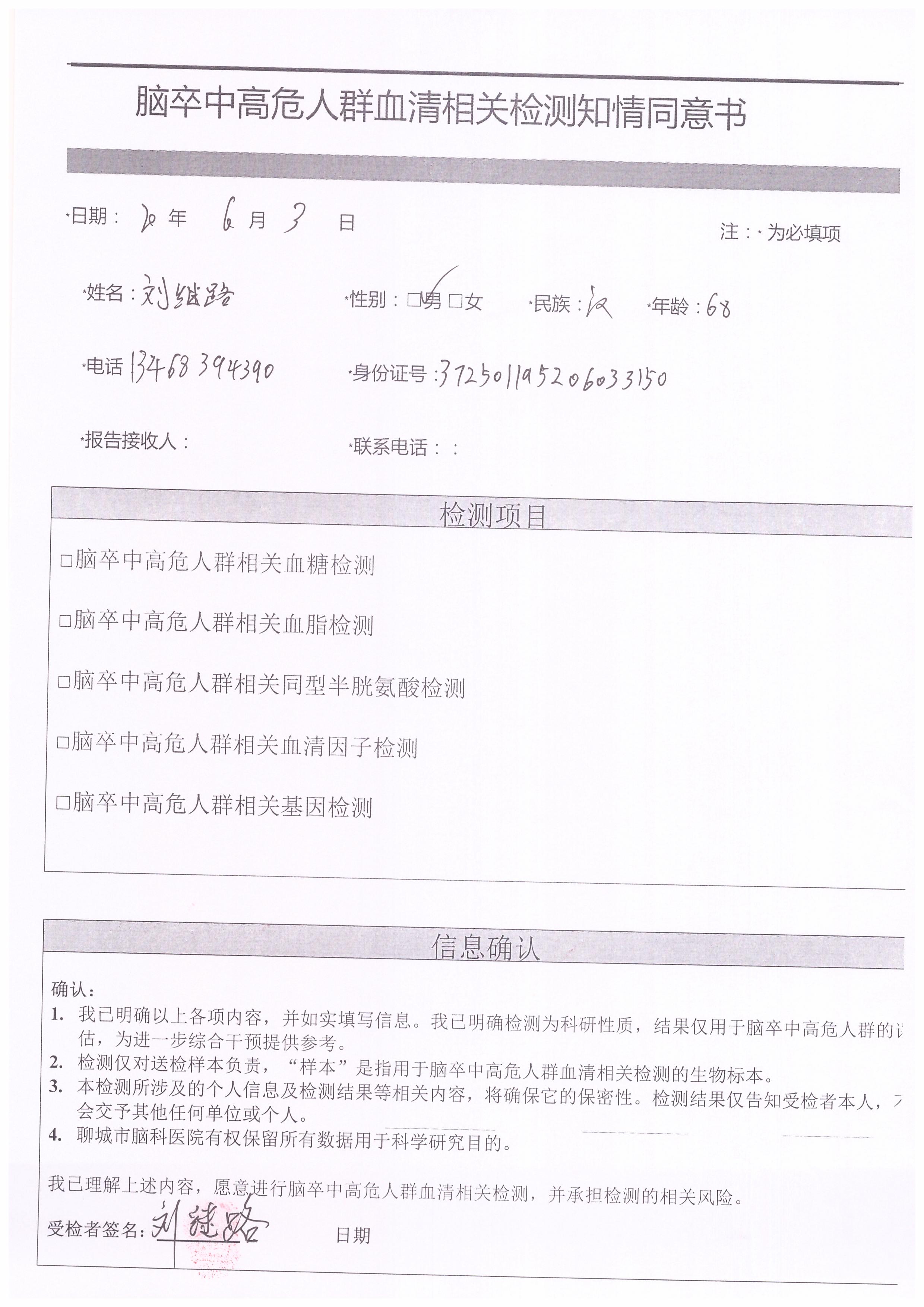

Supplement: Supplementary file 14 — Supplementary file14 (ZIP 27750 KB) [file 10528_2023_10431_MOESM14_ESM.zip › ╓¬╟Θ═1⁄4╥Γ╩Θ12/╡┌╢■▓┐╖╓/012.jpg]

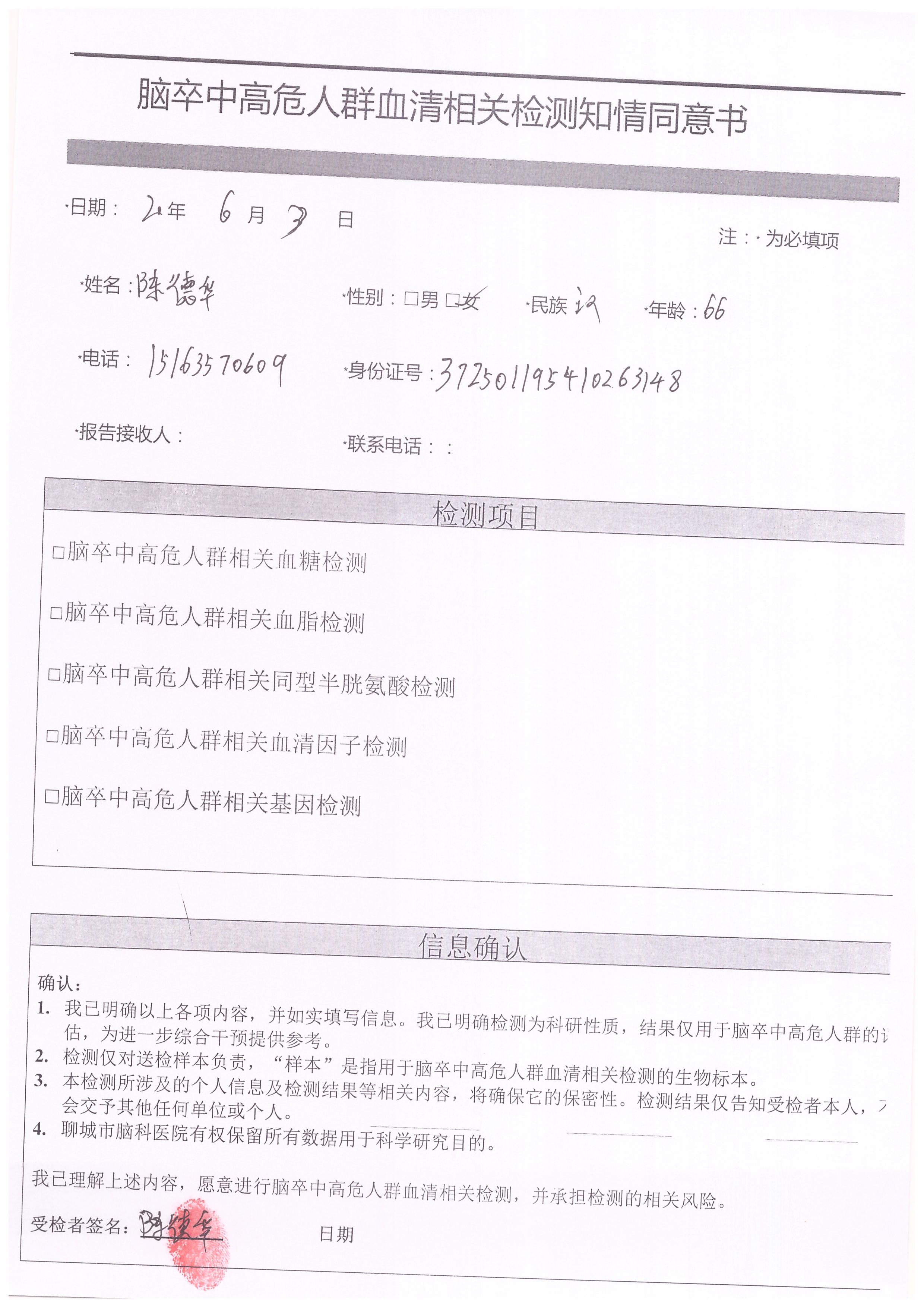

Supplement: Supplementary file 14 — Supplementary file14 (ZIP 27750 KB) [file 10528_2023_10431_MOESM14_ESM.zip › ╓¬╟Θ═1⁄4╥Γ╩Θ12/╡┌╢■▓┐╖╓/013.jpg]

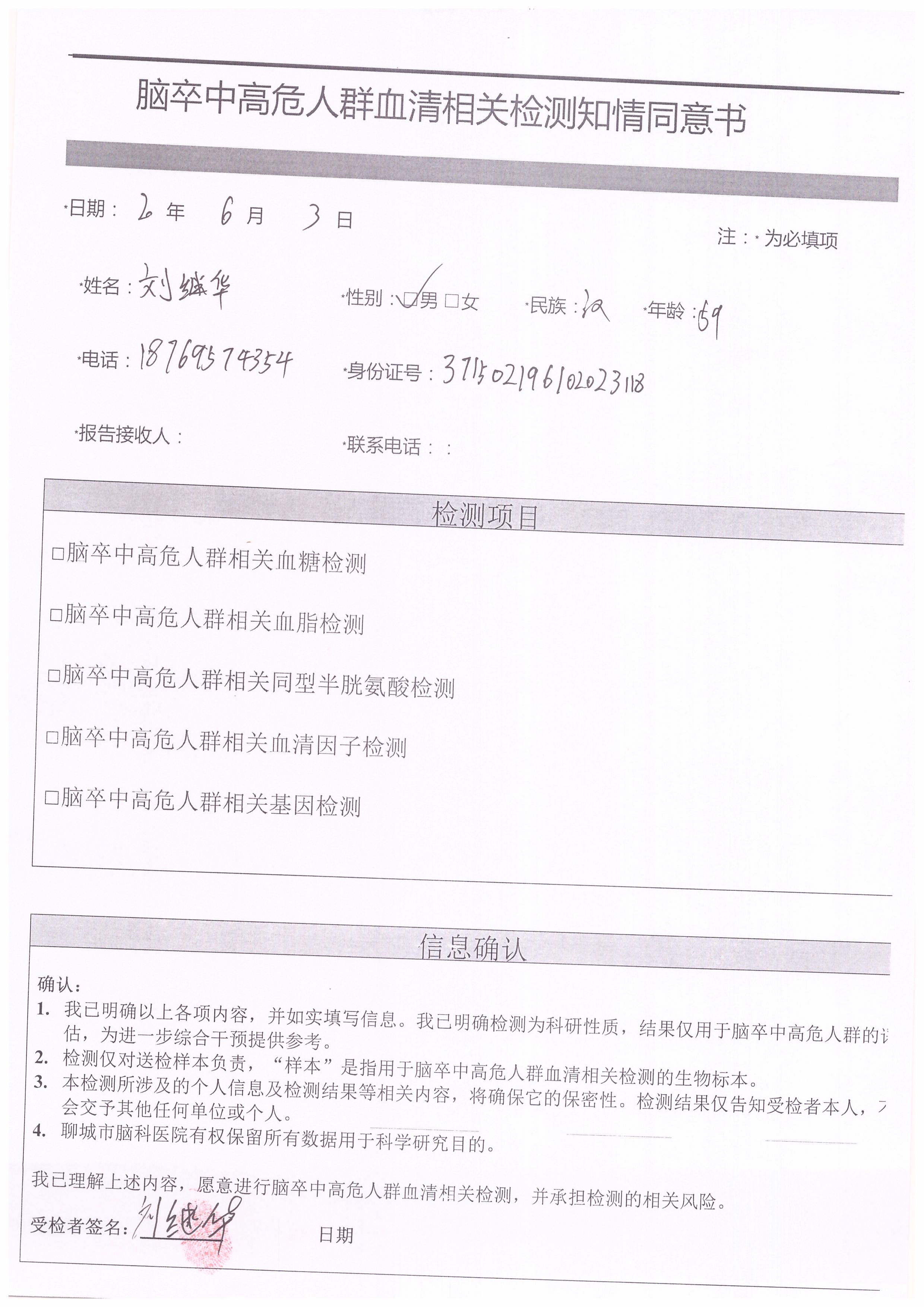

Supplement: Supplementary file 14 — Supplementary file14 (ZIP 27750 KB) [file 10528_2023_10431_MOESM14_ESM.zip › ╓¬╟Θ═1⁄4╥Γ╩Θ12/╡┌╢■▓┐╖╓/014.jpg]

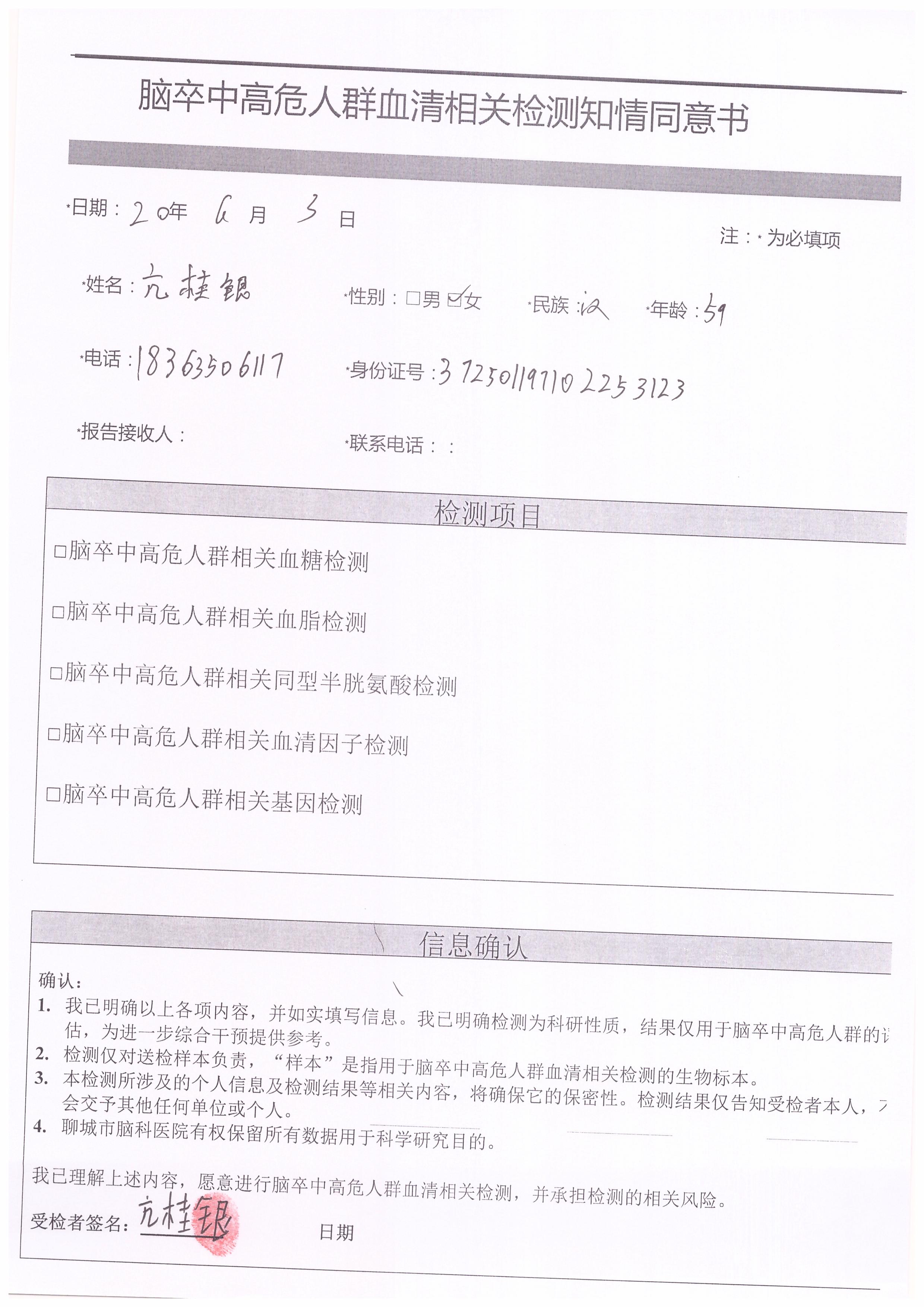

Supplement: Supplementary file 14 — Supplementary file14 (ZIP 27750 KB) [file 10528_2023_10431_MOESM14_ESM.zip › ╓¬╟Θ═1⁄4╥Γ╩Θ12/╡┌╢■▓┐╖╓/016.jpg]

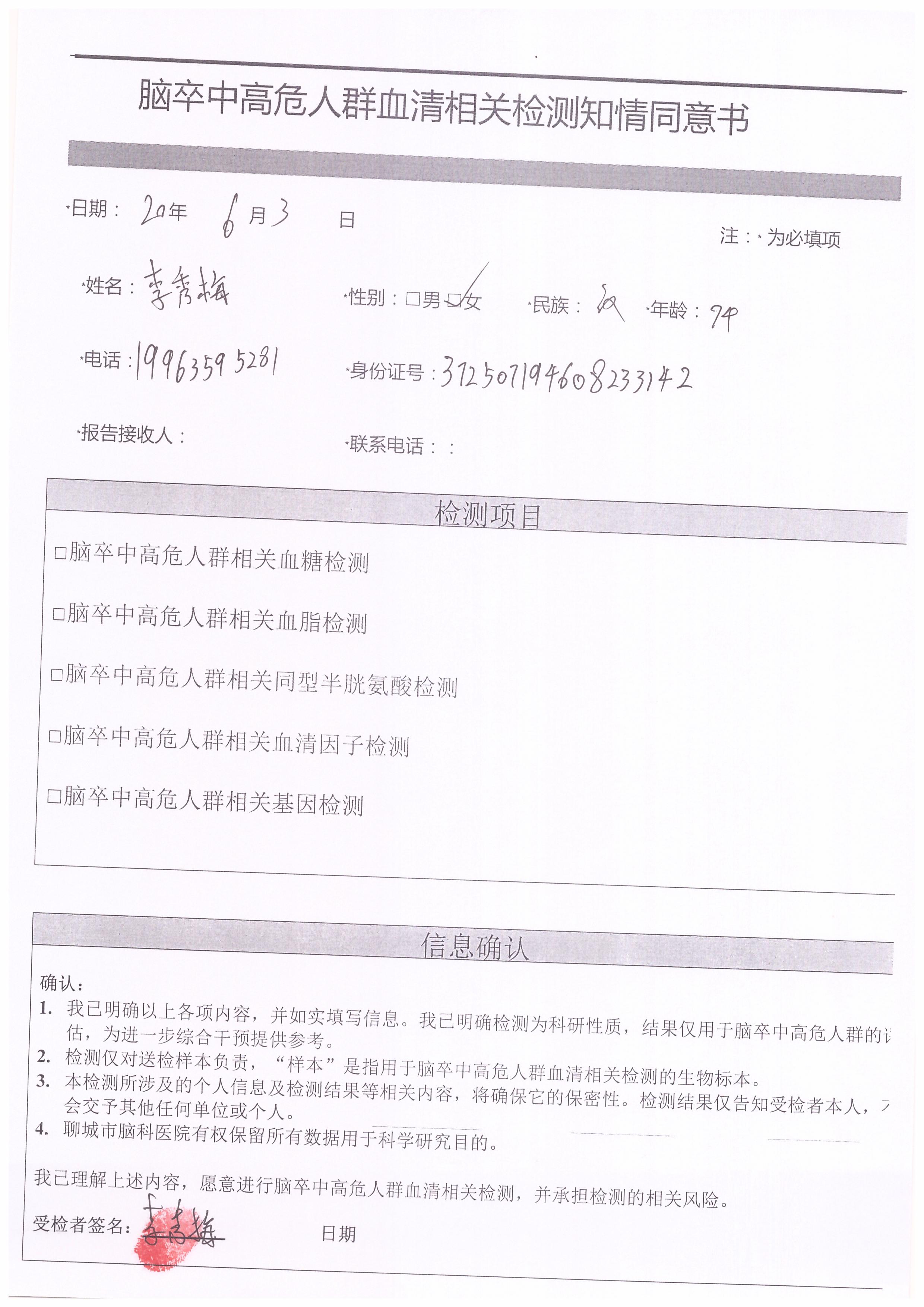

Supplement: Supplementary file 14 — Supplementary file14 (ZIP 27750 KB) [file 10528_2023_10431_MOESM14_ESM.zip › ╓¬╟Θ═1⁄4╥Γ╩Θ12/╡┌╢■▓┐╖╓/017.jpg]

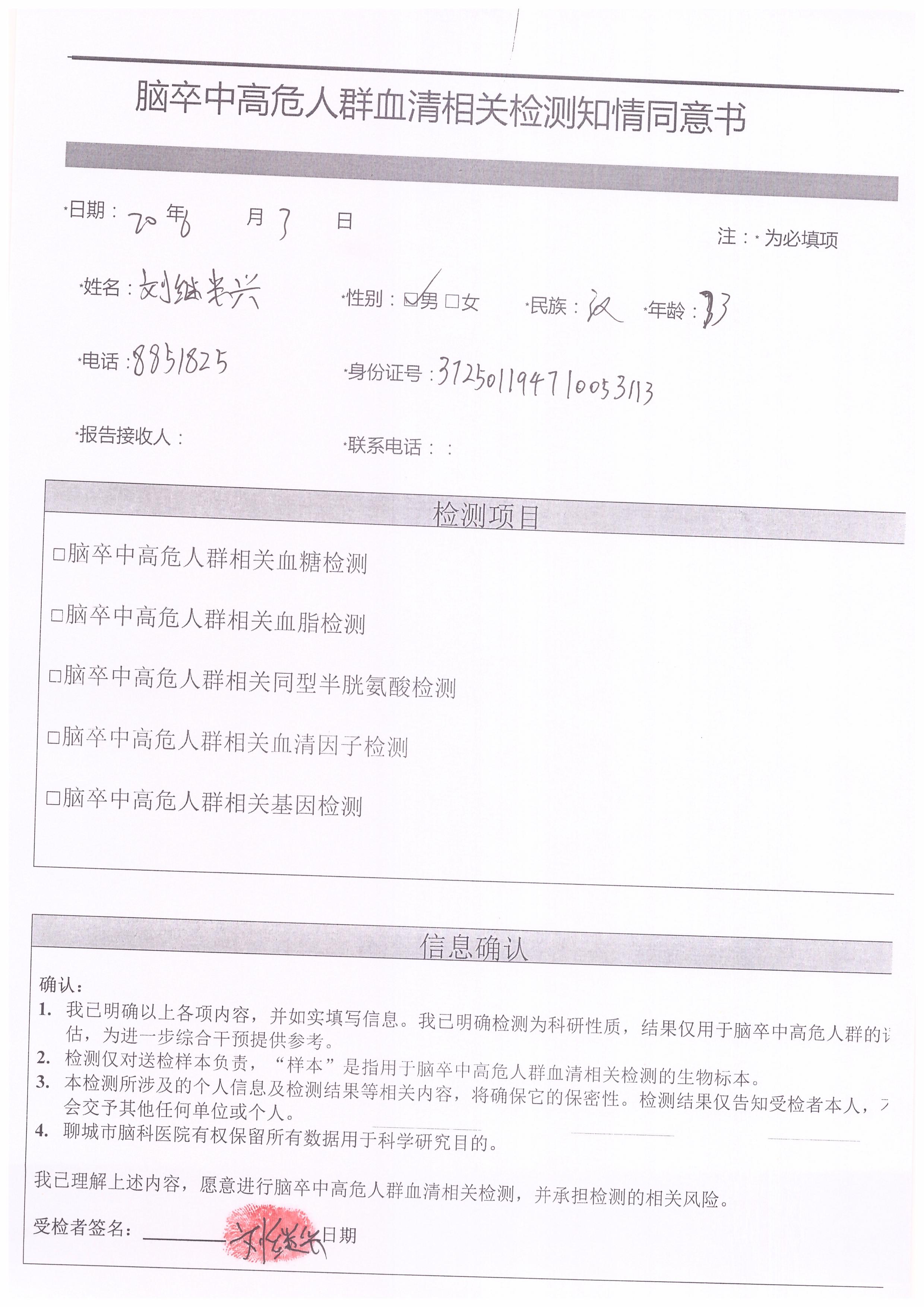

Supplement: Supplementary file 14 — Supplementary file14 (ZIP 27750 KB) [file 10528_2023_10431_MOESM14_ESM.zip › ╓¬╟Θ═1⁄4╥Γ╩Θ12/╡┌╢■▓┐╖╓/018.jpg]

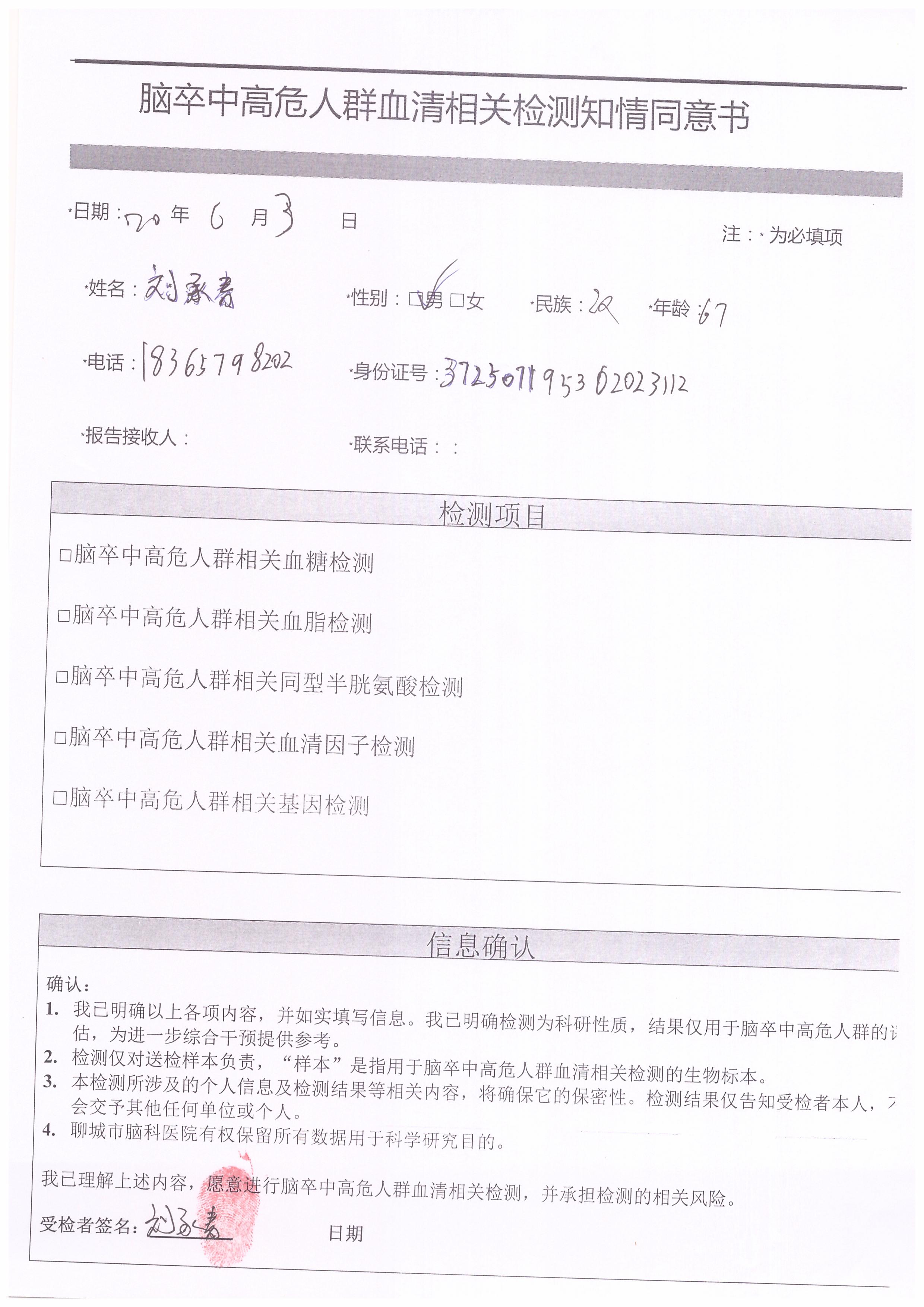

Supplement: Supplementary file 14 — Supplementary file14 (ZIP 27750 KB) [file 10528_2023_10431_MOESM14_ESM.zip › ╓¬╟Θ═1⁄4╥Γ╩Θ12/╡┌╢■▓┐╖╓/019.jpg]

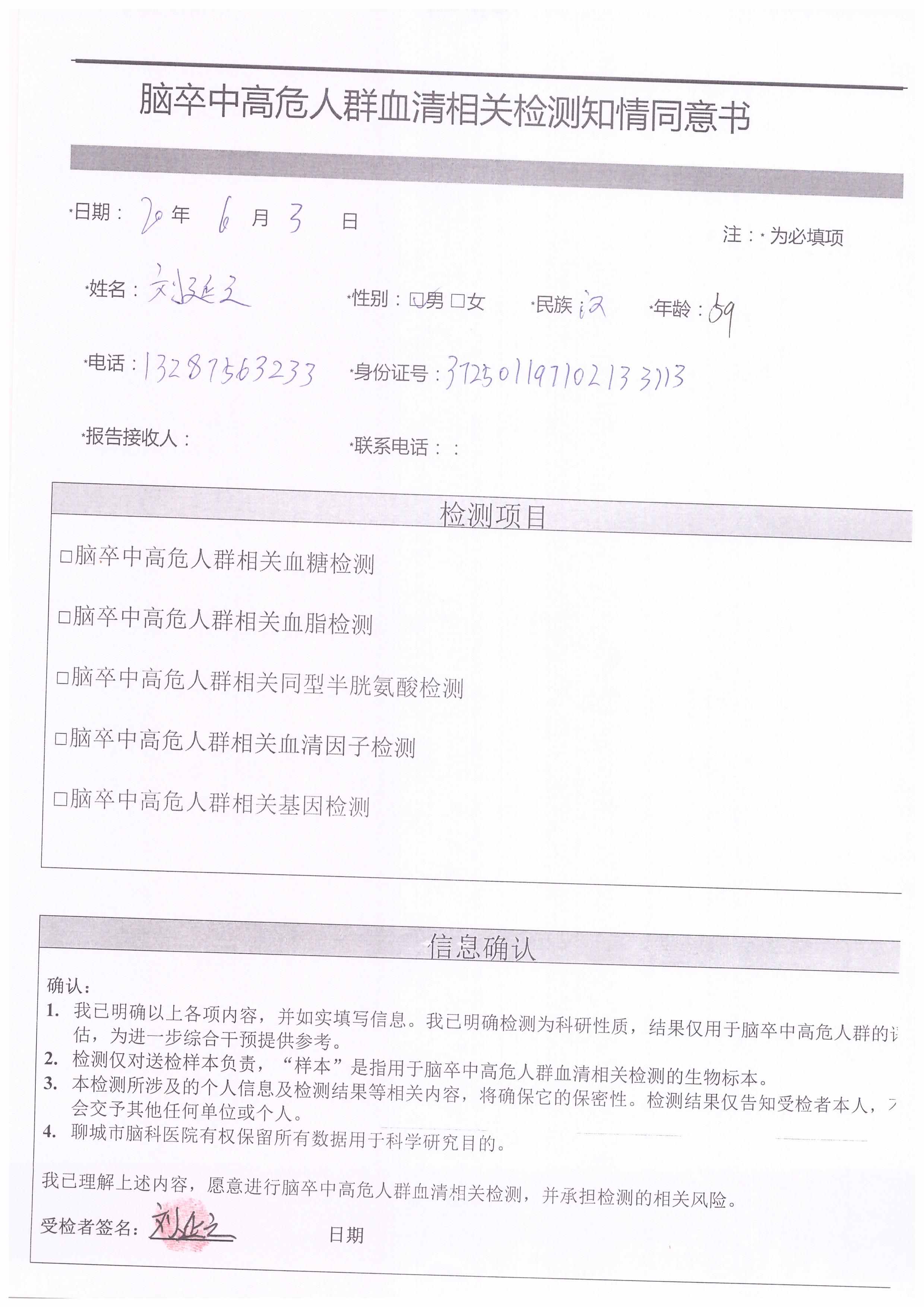

Supplement: Supplementary file 14 — Supplementary file14 (ZIP 27750 KB) [file 10528_2023_10431_MOESM14_ESM.zip › ╓¬╟Θ═1⁄4╥Γ╩Θ12/╡┌╢■▓┐╖╓/020.jpg]

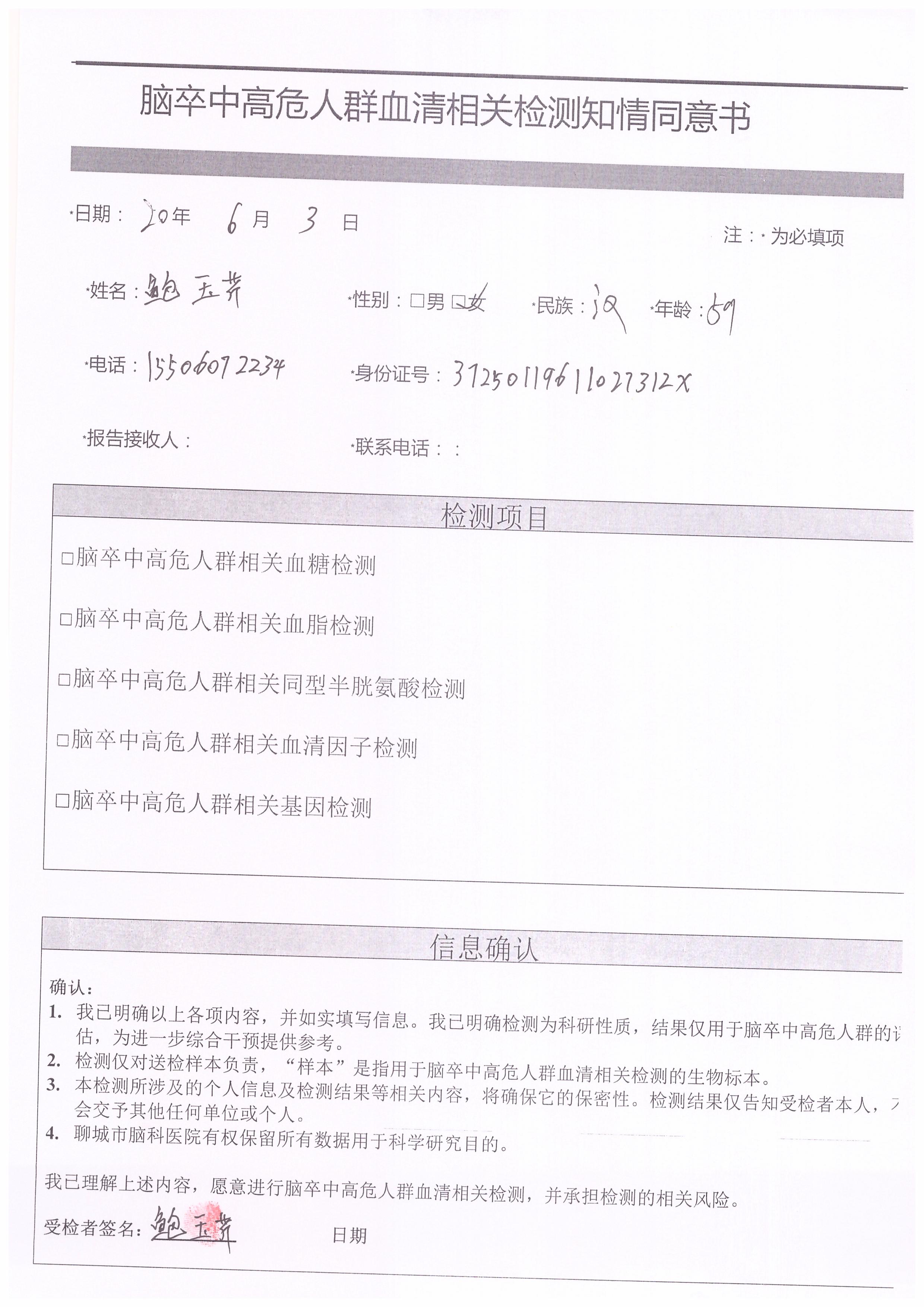

Supplement: Supplementary file 14 — Supplementary file14 (ZIP 27750 KB) [file 10528_2023_10431_MOESM14_ESM.zip › ╓¬╟Θ═1⁄4╥Γ╩Θ12/╡┌╢■▓┐╖╓/021.jpg]

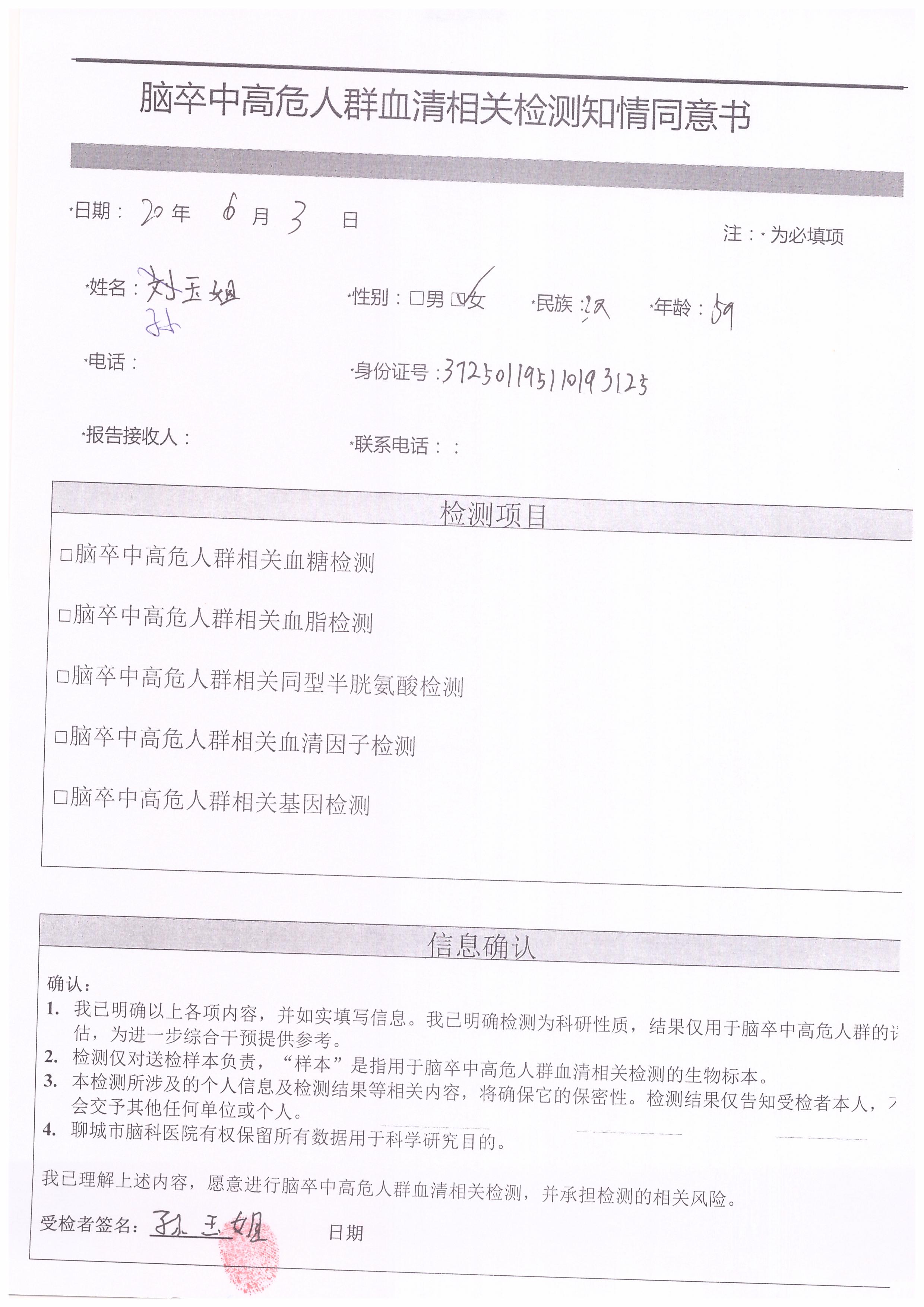

Supplement: Supplementary file 14 — Supplementary file14 (ZIP 27750 KB) [file 10528_2023_10431_MOESM14_ESM.zip › ╓¬╟Θ═1⁄4╥Γ╩Θ12/╡┌╢■▓┐╖╓/022.jpg]

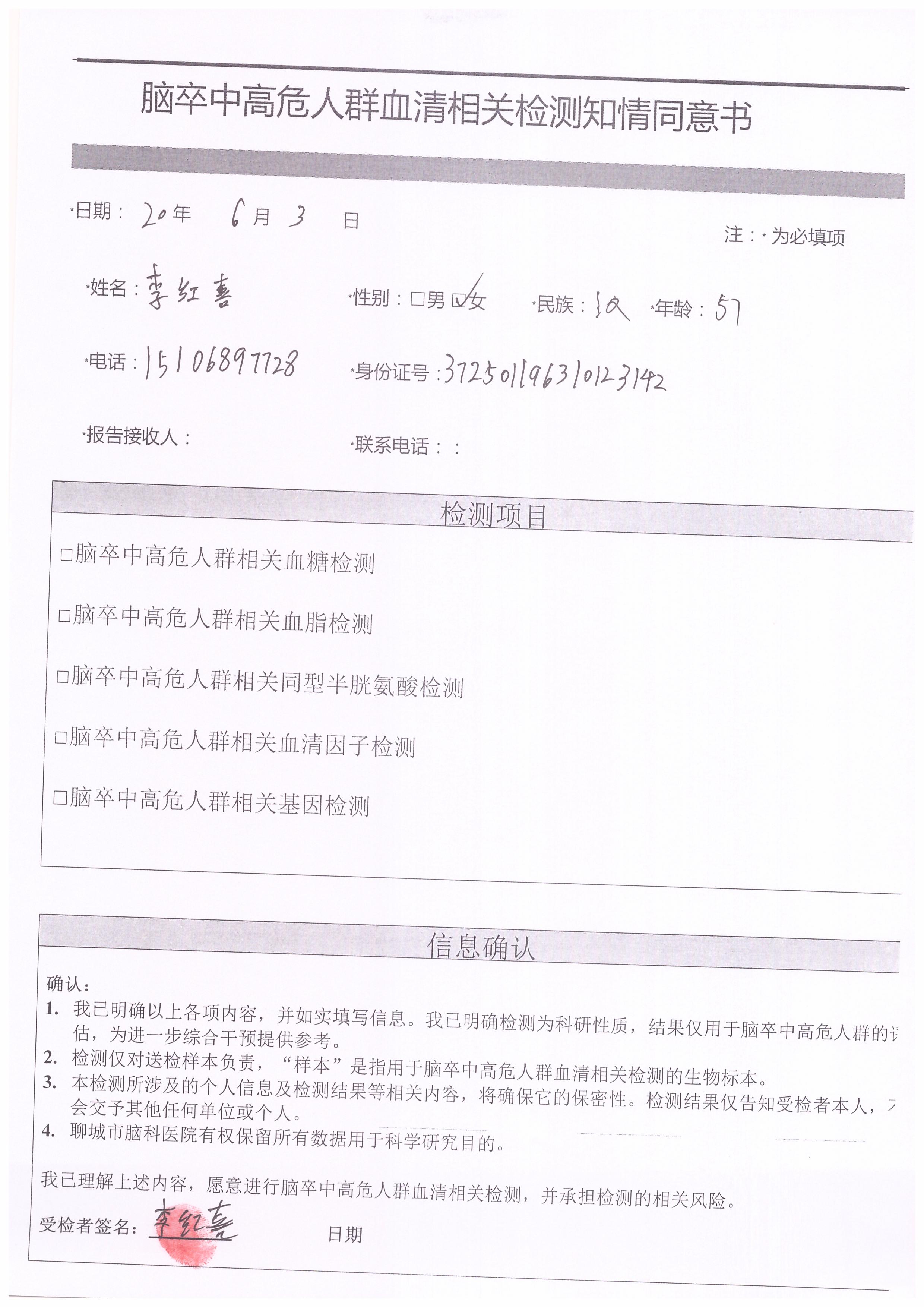

Supplement: Supplementary file 14 — Supplementary file14 (ZIP 27750 KB) [file 10528_2023_10431_MOESM14_ESM.zip › ╓¬╟Θ═1⁄4╥Γ╩Θ12/╡┌╢■▓┐╖╓/023.jpg]

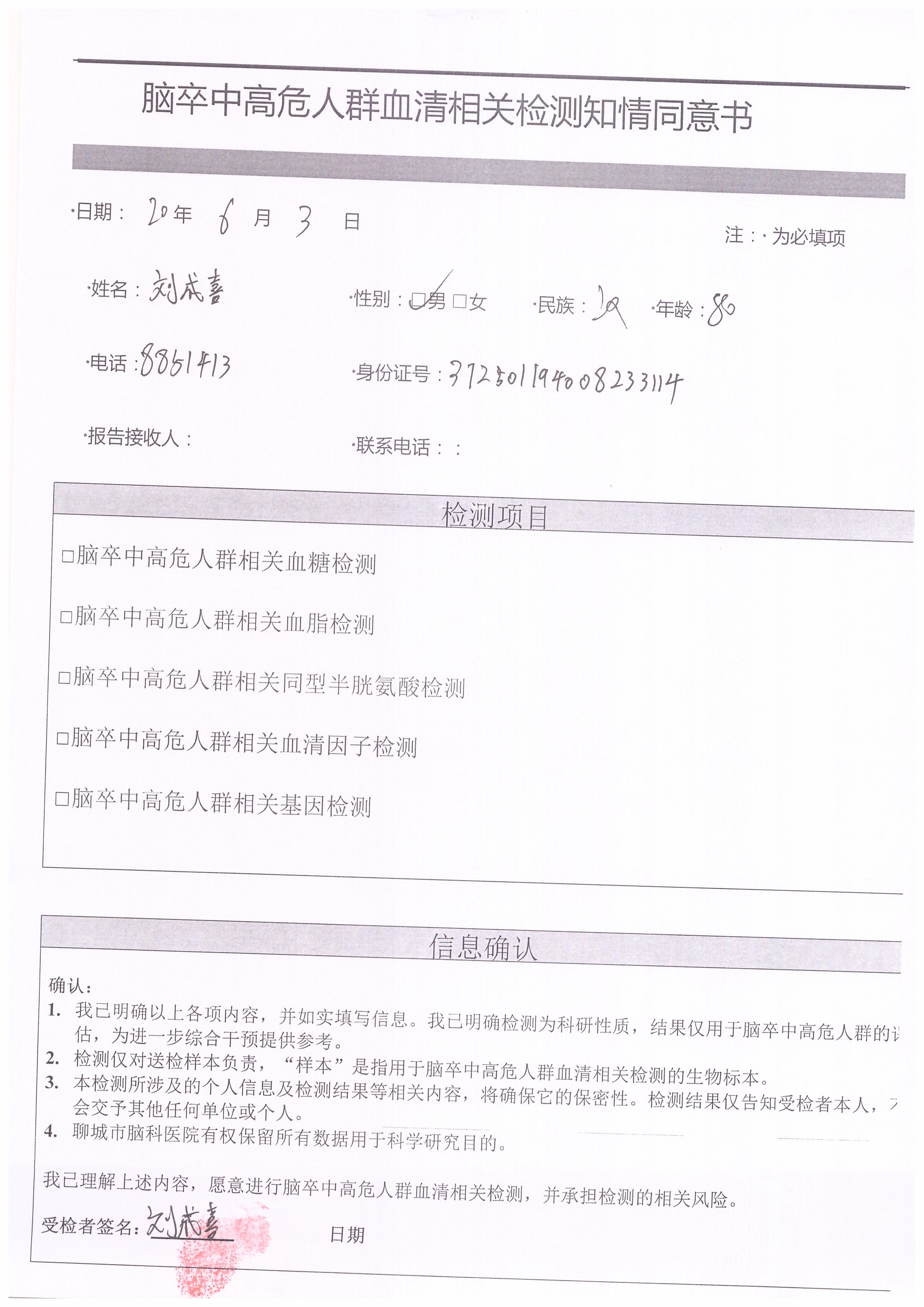

Supplement: Supplementary file 14 — Supplementary file14 (ZIP 27750 KB) [file 10528_2023_10431_MOESM14_ESM.zip › ╓¬╟Θ═1⁄4╥Γ╩Θ12/╡┌╢■▓┐╖╓/024.jpg]

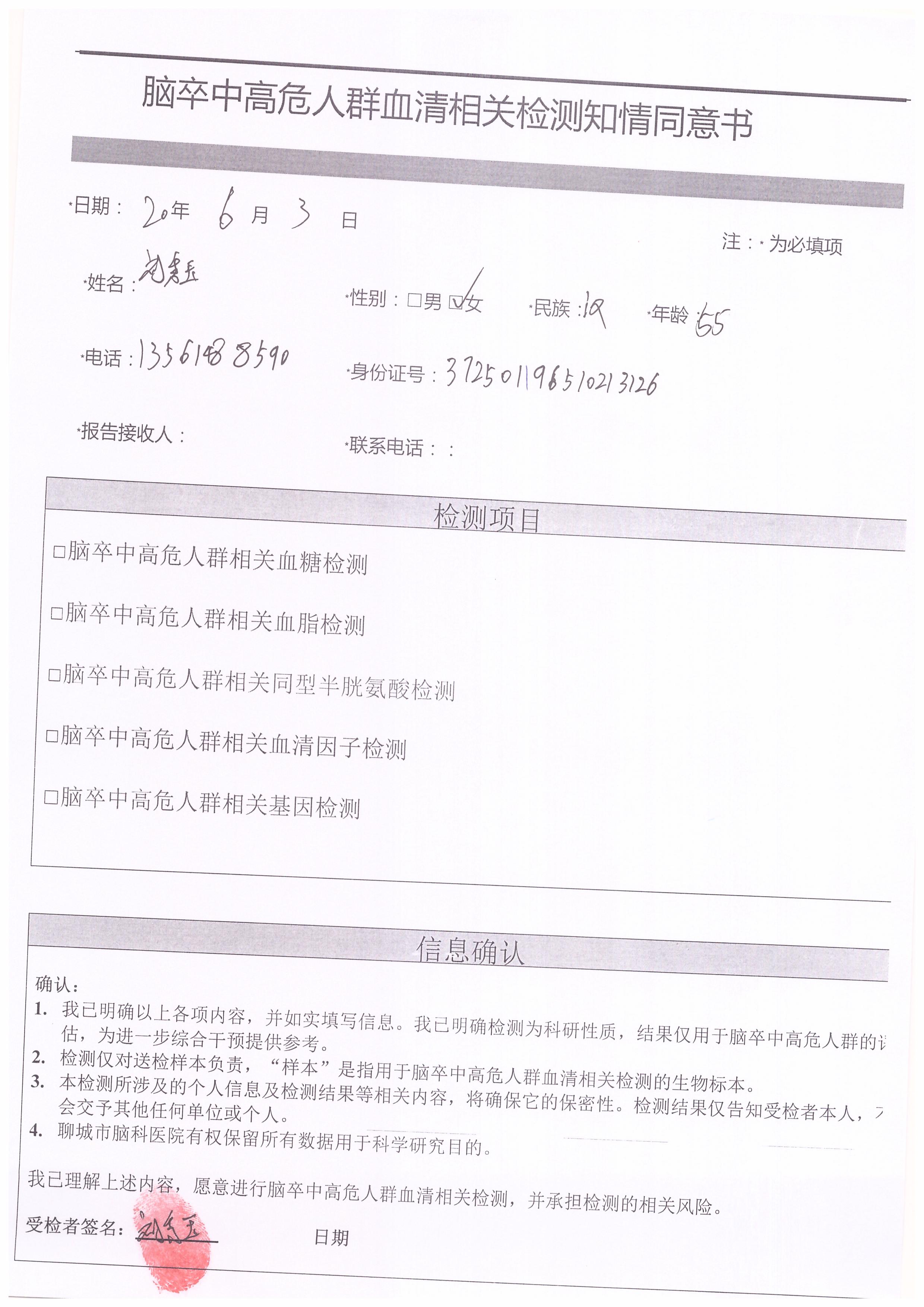

Supplement: Supplementary file 14 — Supplementary file14 (ZIP 27750 KB) [file 10528_2023_10431_MOESM14_ESM.zip › ╓¬╟Θ═1⁄4╥Γ╩Θ12/╡┌╢■▓┐╖╓/025.jpg]

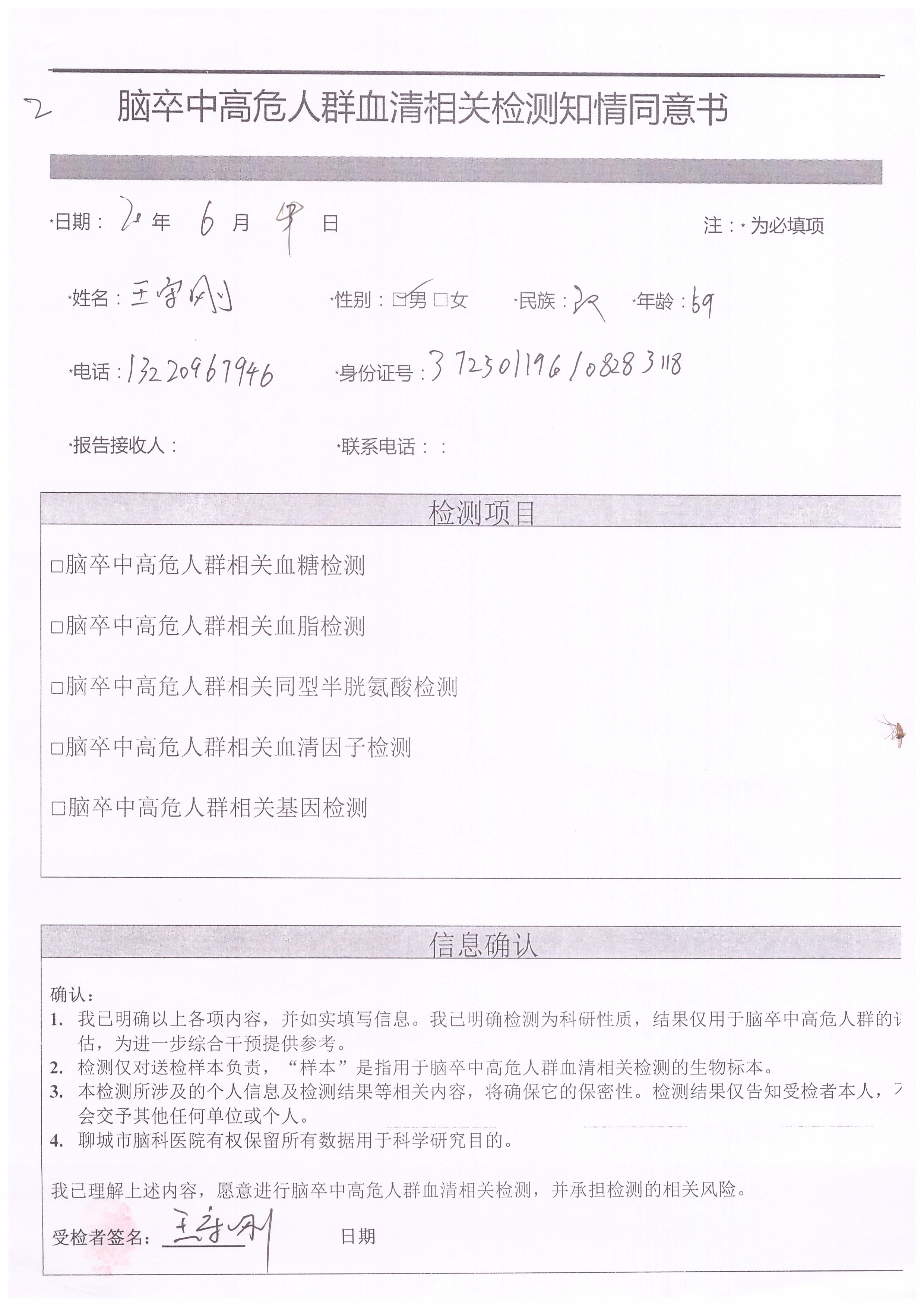

Supplement: Supplementary file 15 — Supplementary file15 (ZIP 22488 KB) [file 10528_2023_10431_MOESM15_ESM.zip › ╓¬╟Θ═1⁄4╥Γ╩Θ13/002.jpg]

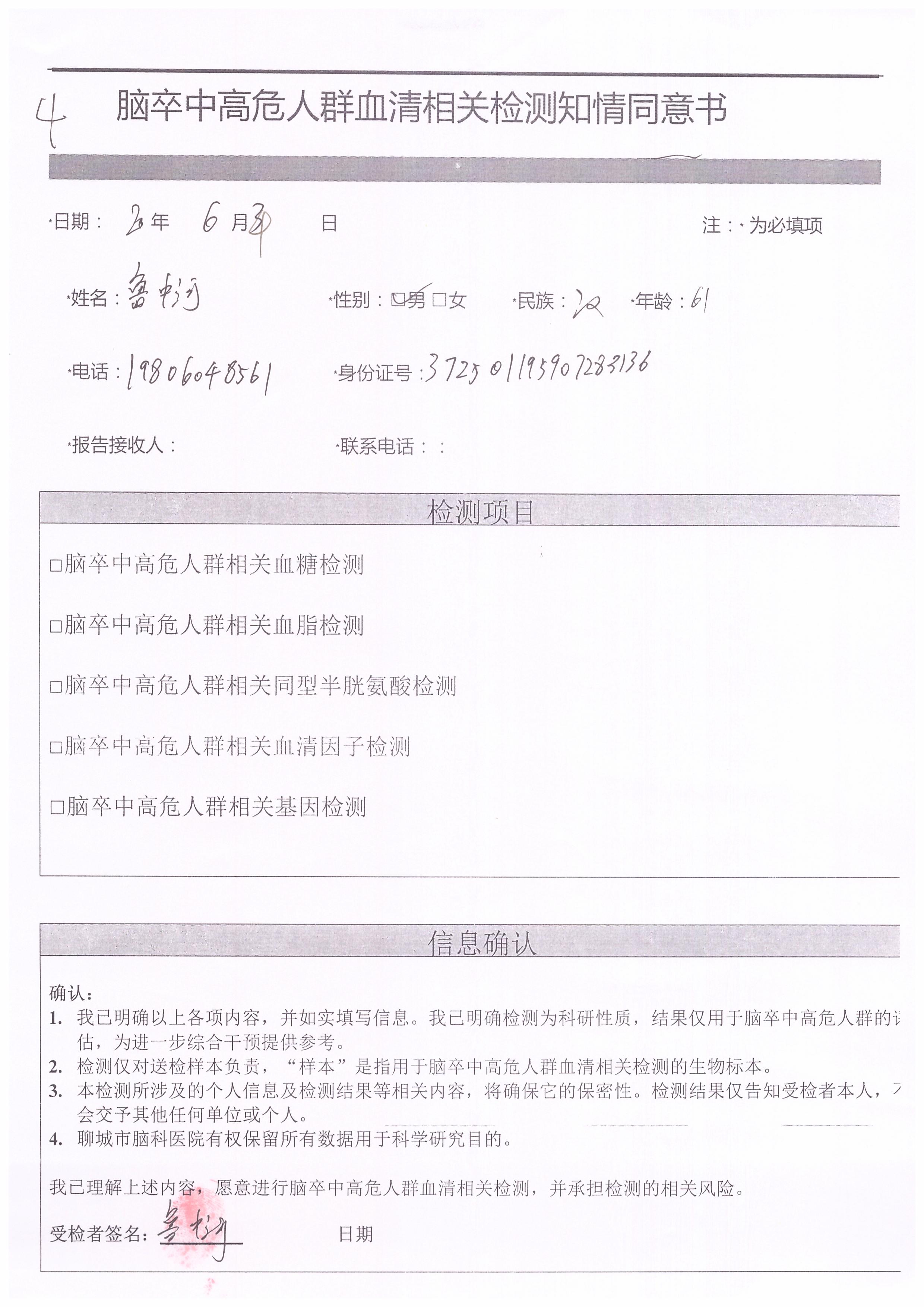

Supplement: Supplementary file 15 — Supplementary file15 (ZIP 22488 KB) [file 10528_2023_10431_MOESM15_ESM.zip › ╓¬╟Θ═1⁄4╥Γ╩Θ13/004.jpg]

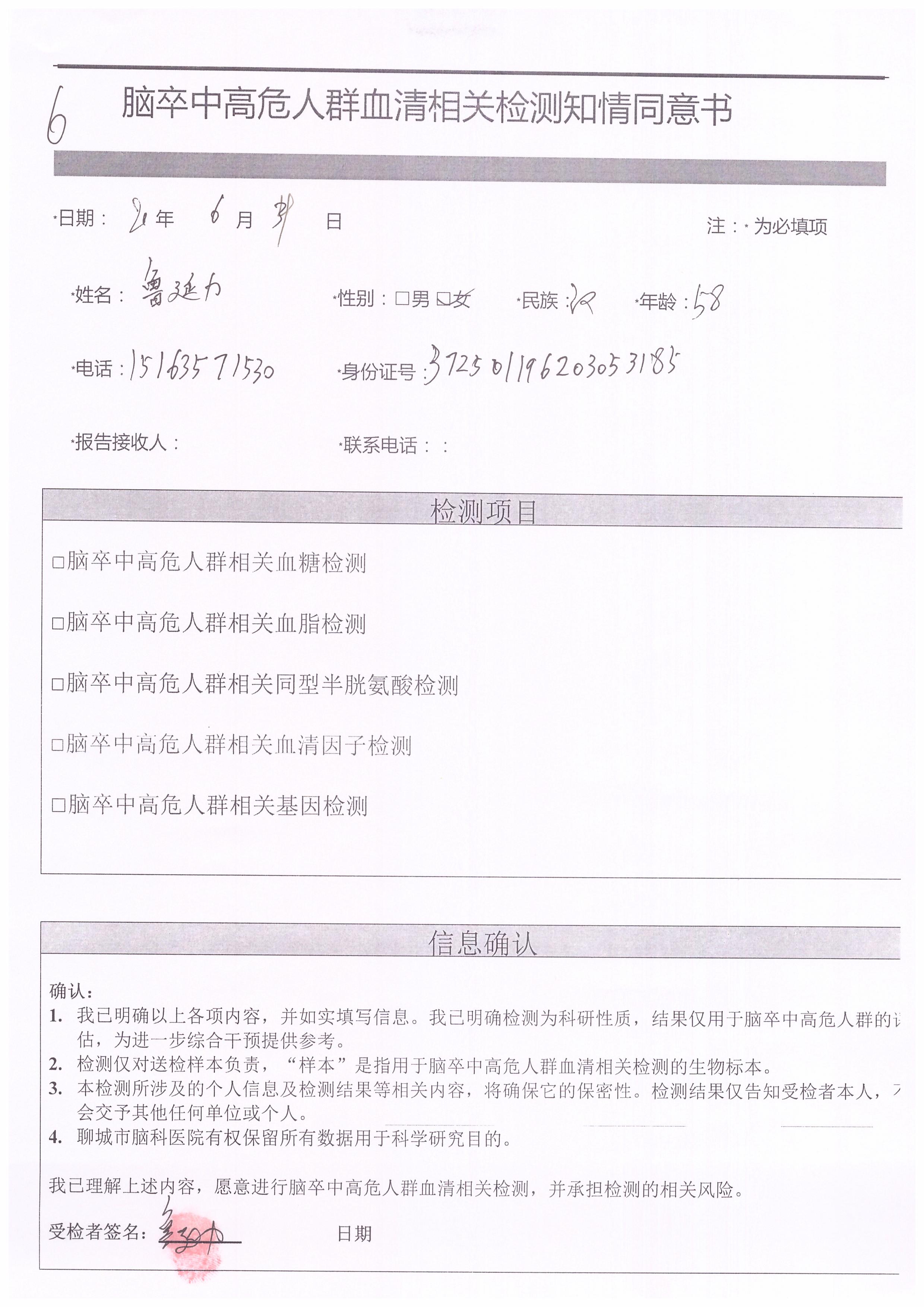

Supplement: Supplementary file 15 — Supplementary file15 (ZIP 22488 KB) [file 10528_2023_10431_MOESM15_ESM.zip › ╓¬╟Θ═1⁄4╥Γ╩Θ13/006.jpg]

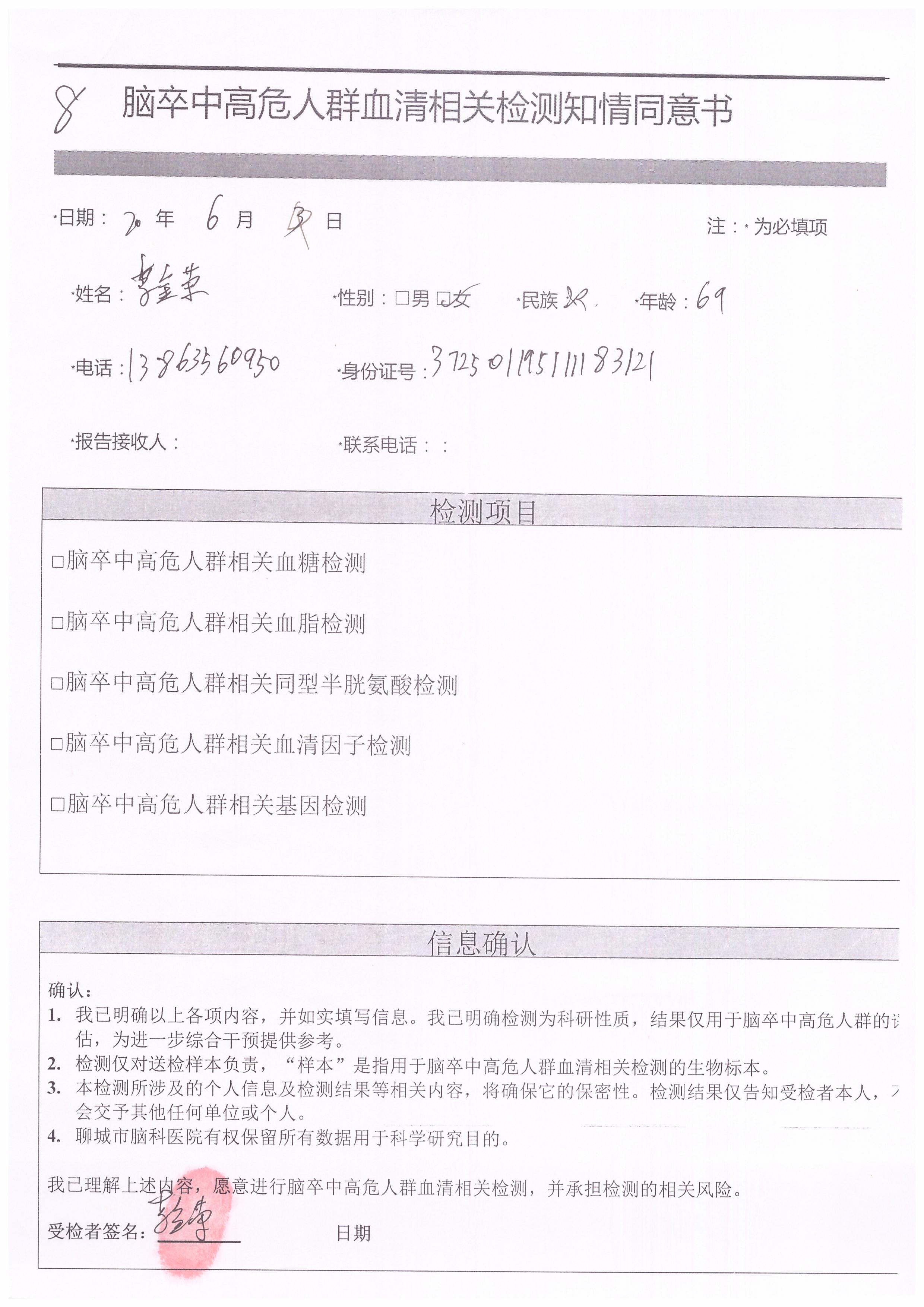

Supplement: Supplementary file 15 — Supplementary file15 (ZIP 22488 KB) [file 10528_2023_10431_MOESM15_ESM.zip › ╓¬╟Θ═1⁄4╥Γ╩Θ13/008.jpg]

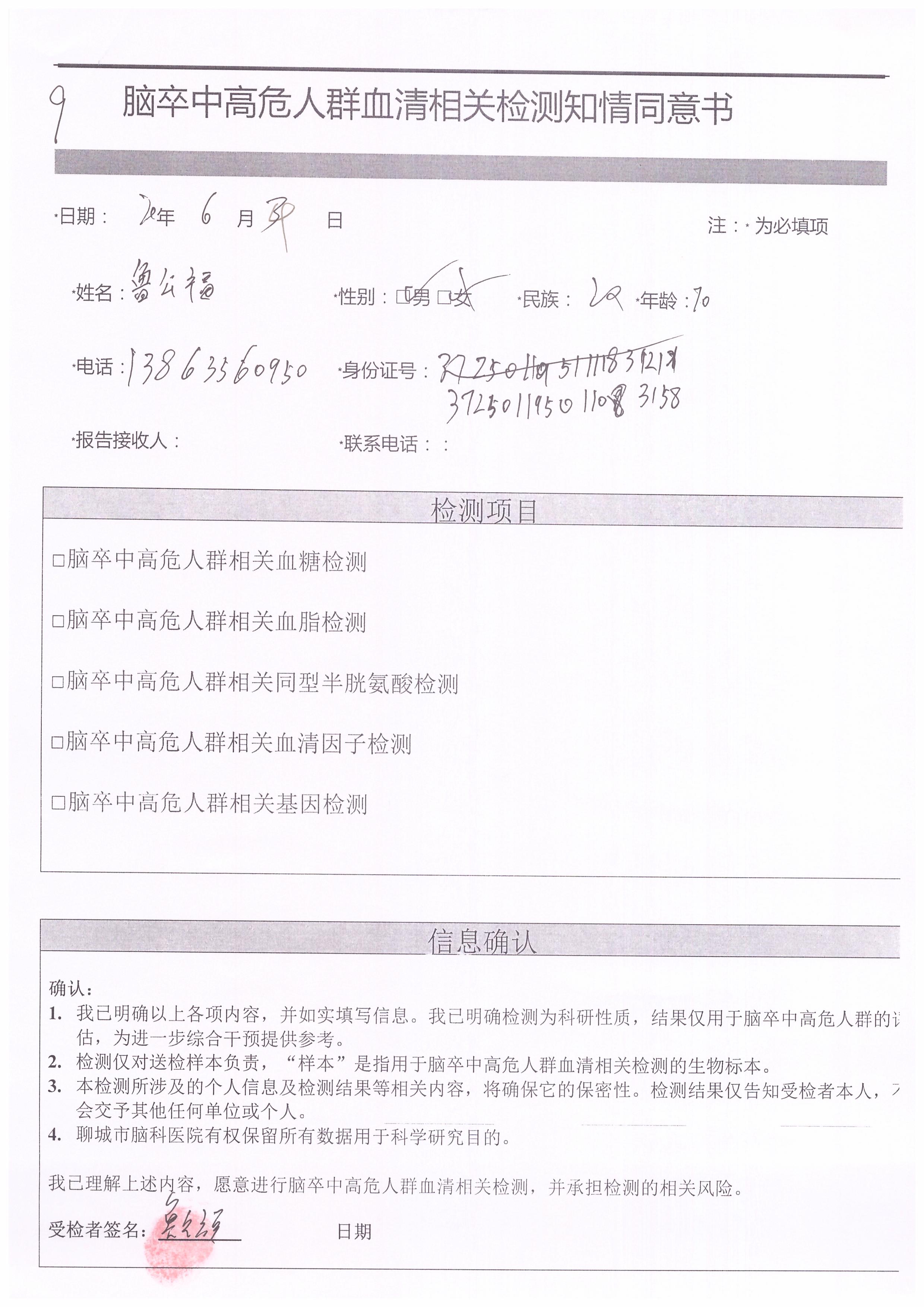

Supplement: Supplementary file 15 — Supplementary file15 (ZIP 22488 KB) [file 10528_2023_10431_MOESM15_ESM.zip › ╓¬╟Θ═1⁄4╥Γ╩Θ13/009.jpg]

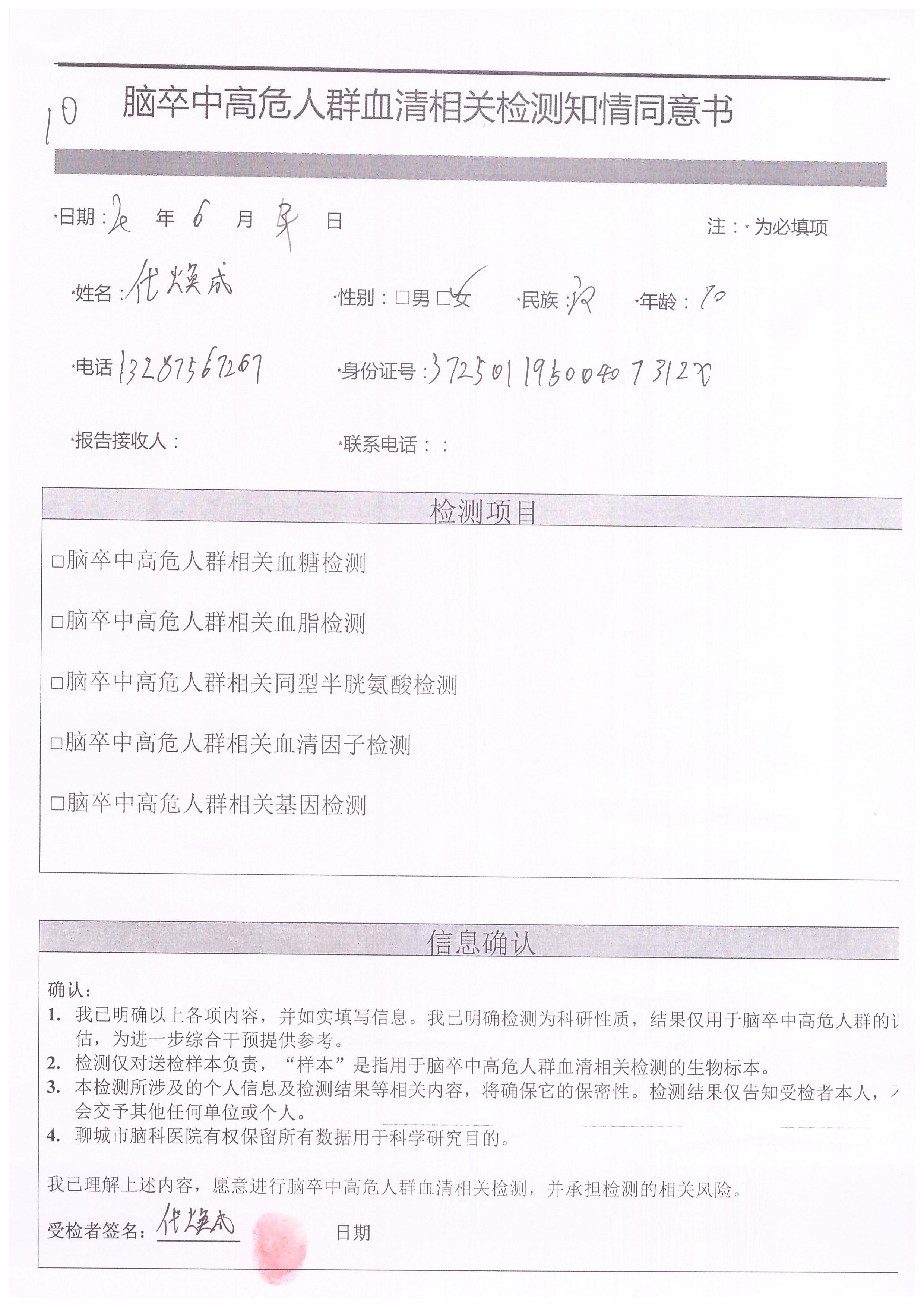

Supplement: Supplementary file 15 — Supplementary file15 (ZIP 22488 KB) [file 10528_2023_10431_MOESM15_ESM.zip › ╓¬╟Θ═1⁄4╥Γ╩Θ13/010.jpg]

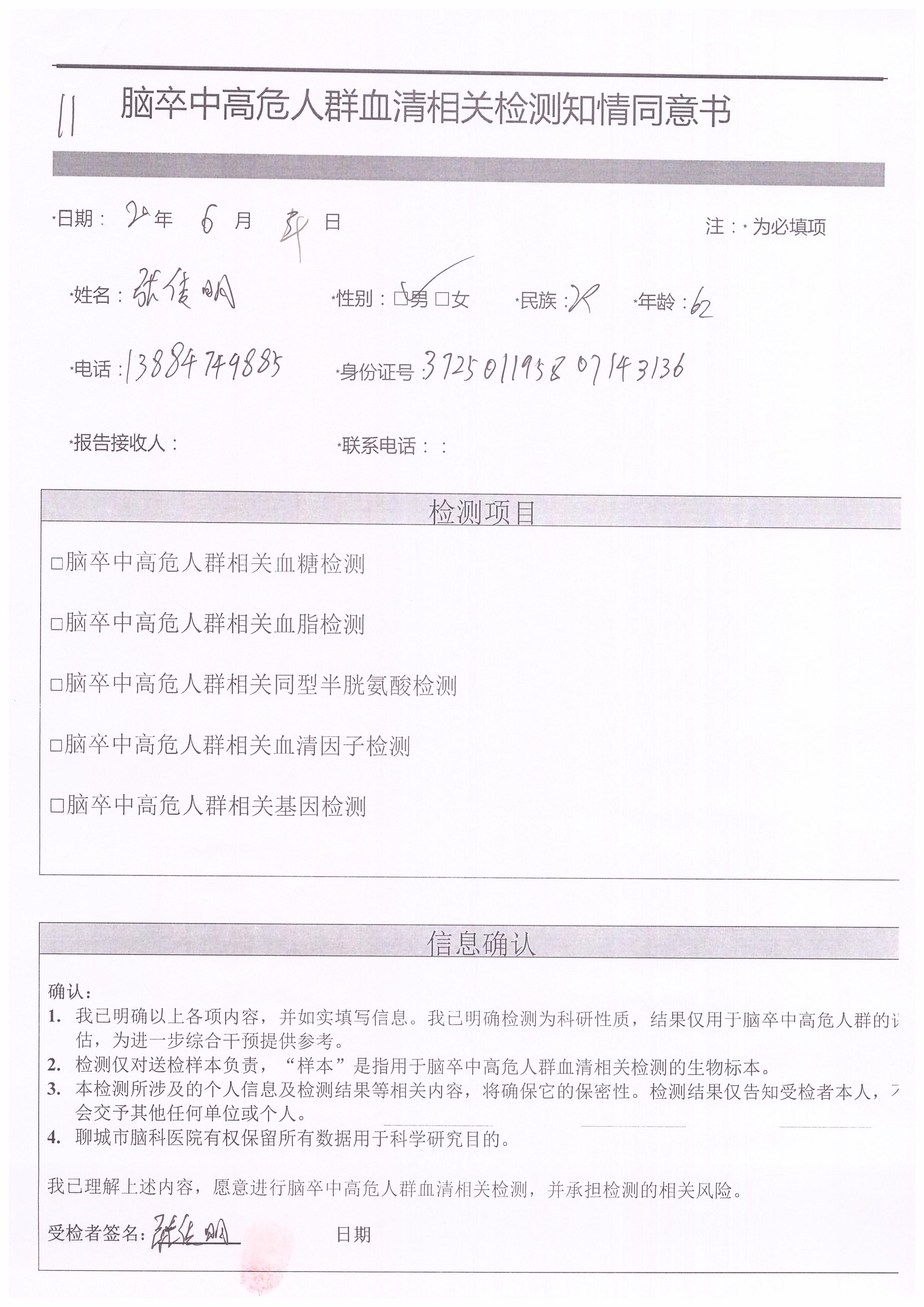

Supplement: Supplementary file 15 — Supplementary file15 (ZIP 22488 KB) [file 10528_2023_10431_MOESM15_ESM.zip › ╓¬╟Θ═1⁄4╥Γ╩Θ13/011.jpg]

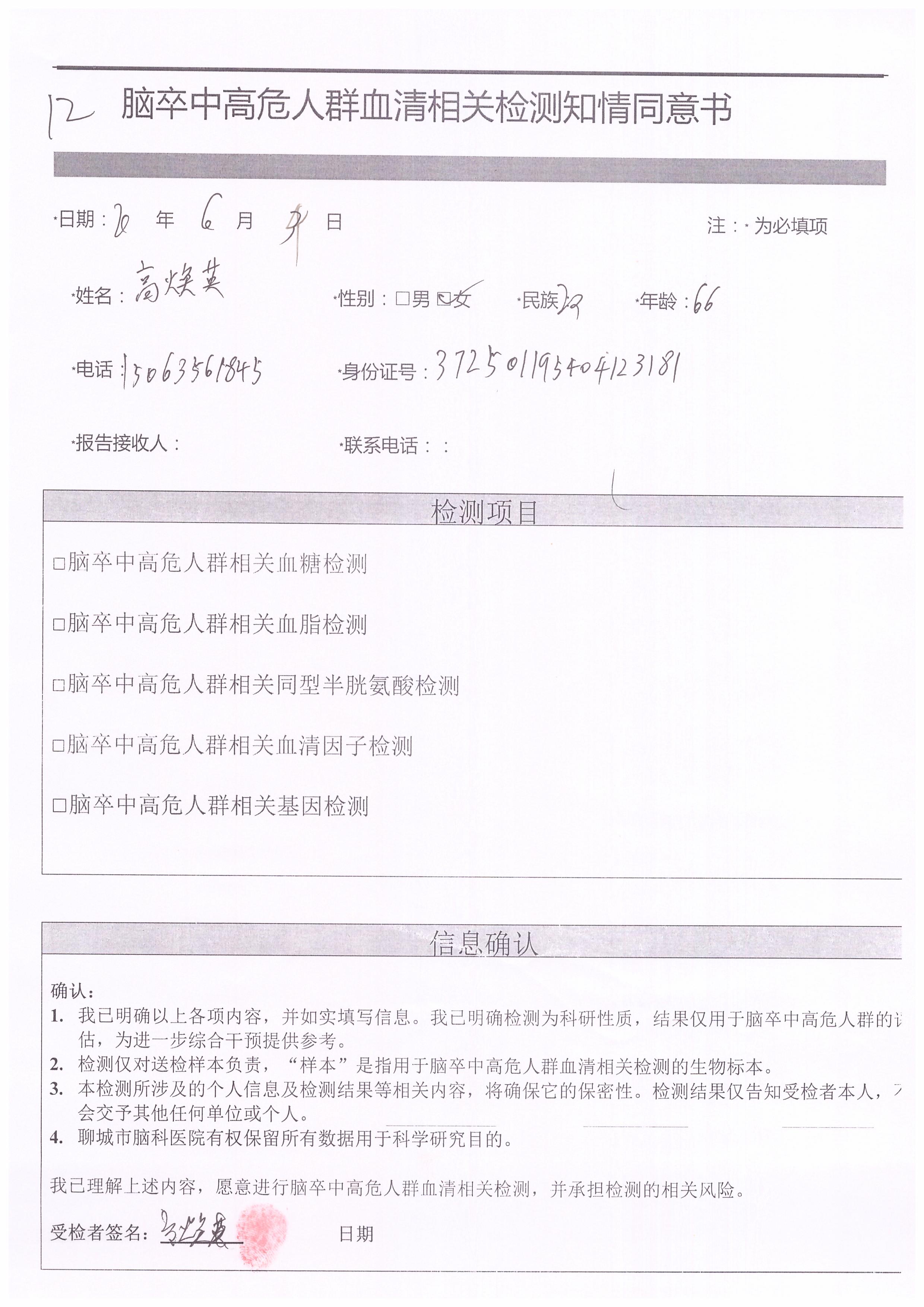

Supplement: Supplementary file 15 — Supplementary file15 (ZIP 22488 KB) [file 10528_2023_10431_MOESM15_ESM.zip › ╓¬╟Θ═1⁄4╥Γ╩Θ13/012.jpg]

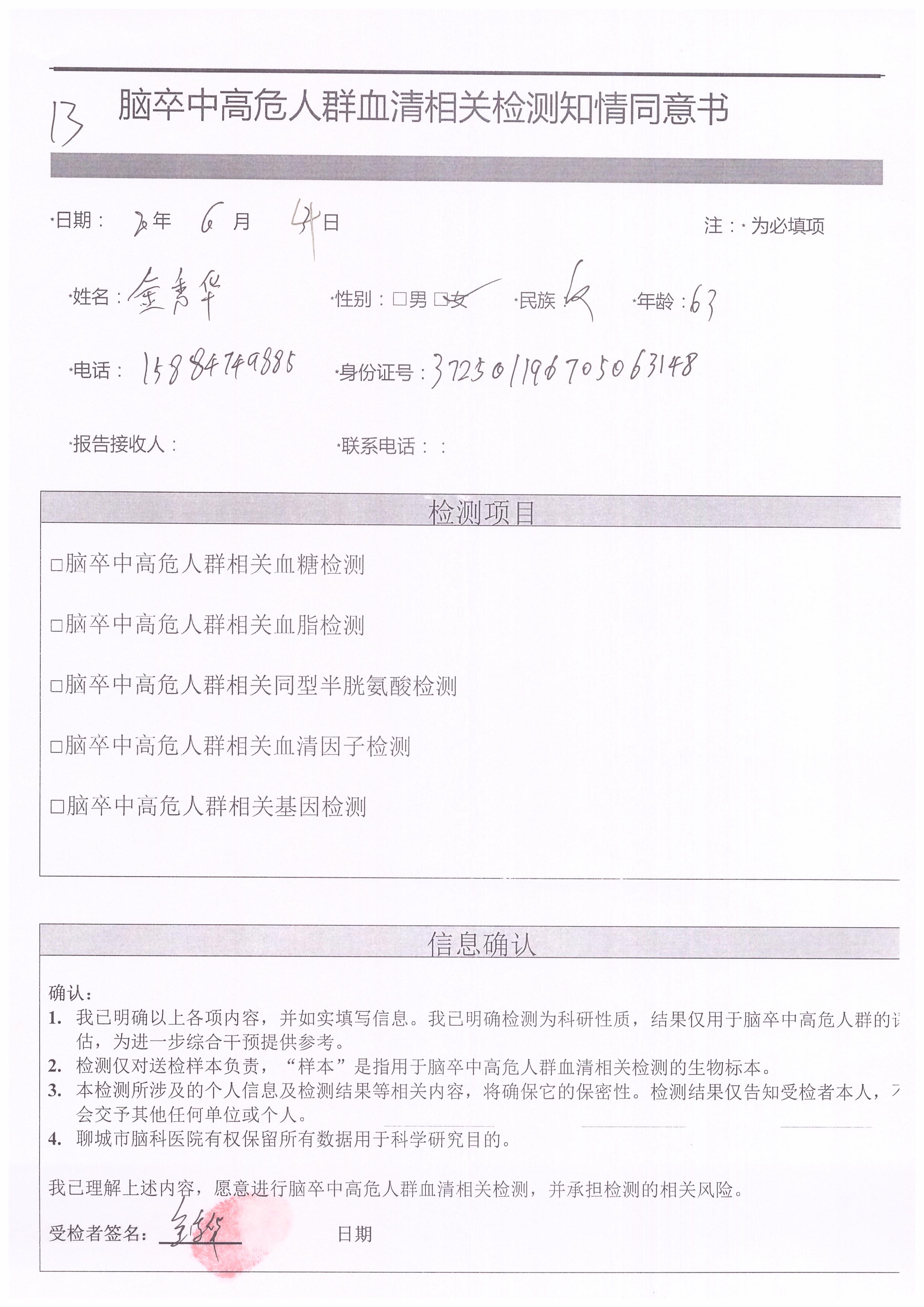

Supplement: Supplementary file 15 — Supplementary file15 (ZIP 22488 KB) [file 10528_2023_10431_MOESM15_ESM.zip › ╓¬╟Θ═1⁄4╥Γ╩Θ13/013.jpg]

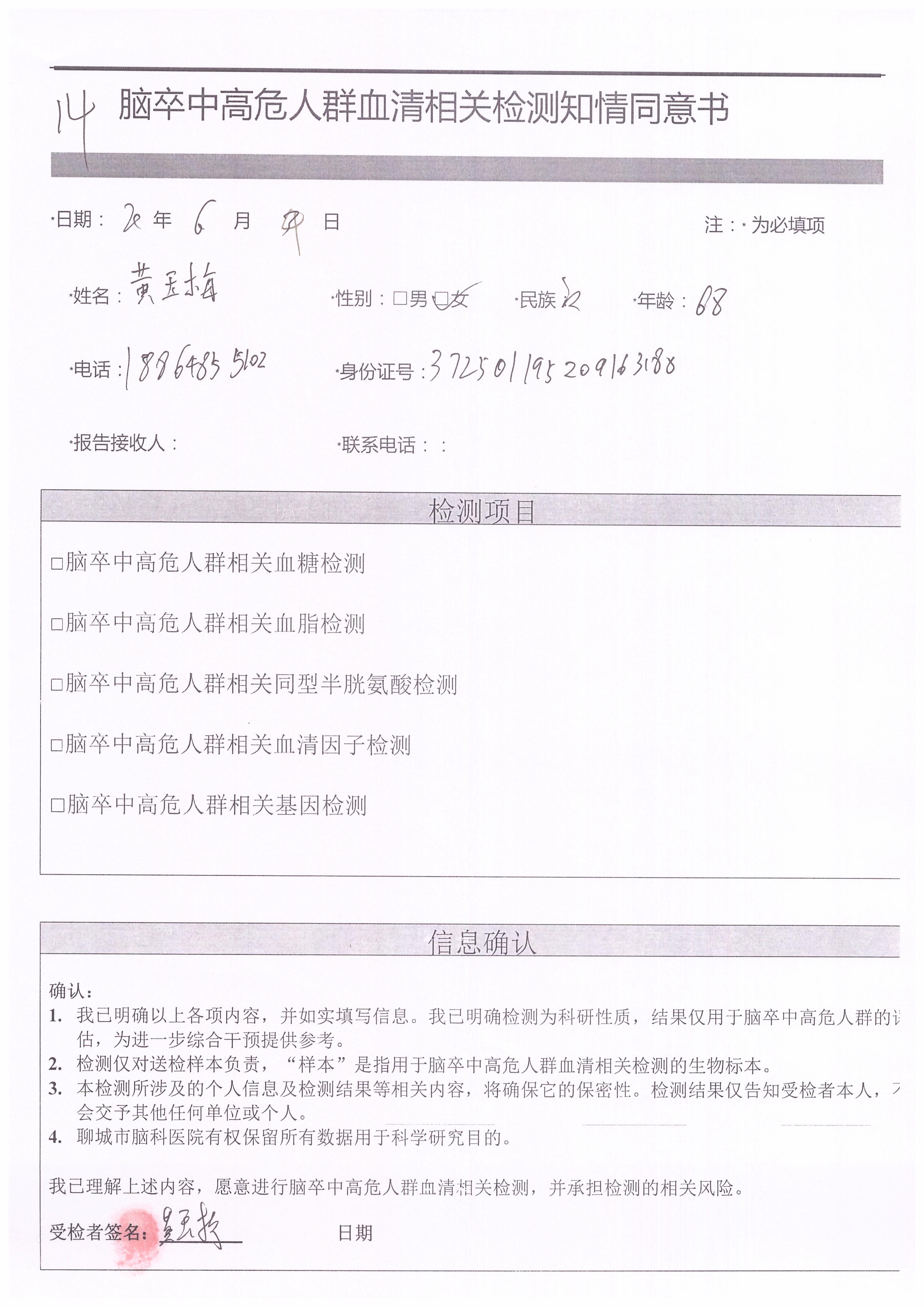

Supplement: Supplementary file 15 — Supplementary file15 (ZIP 22488 KB) [file 10528_2023_10431_MOESM15_ESM.zip › ╓¬╟Θ═1⁄4╥Γ╩Θ13/014.jpg]

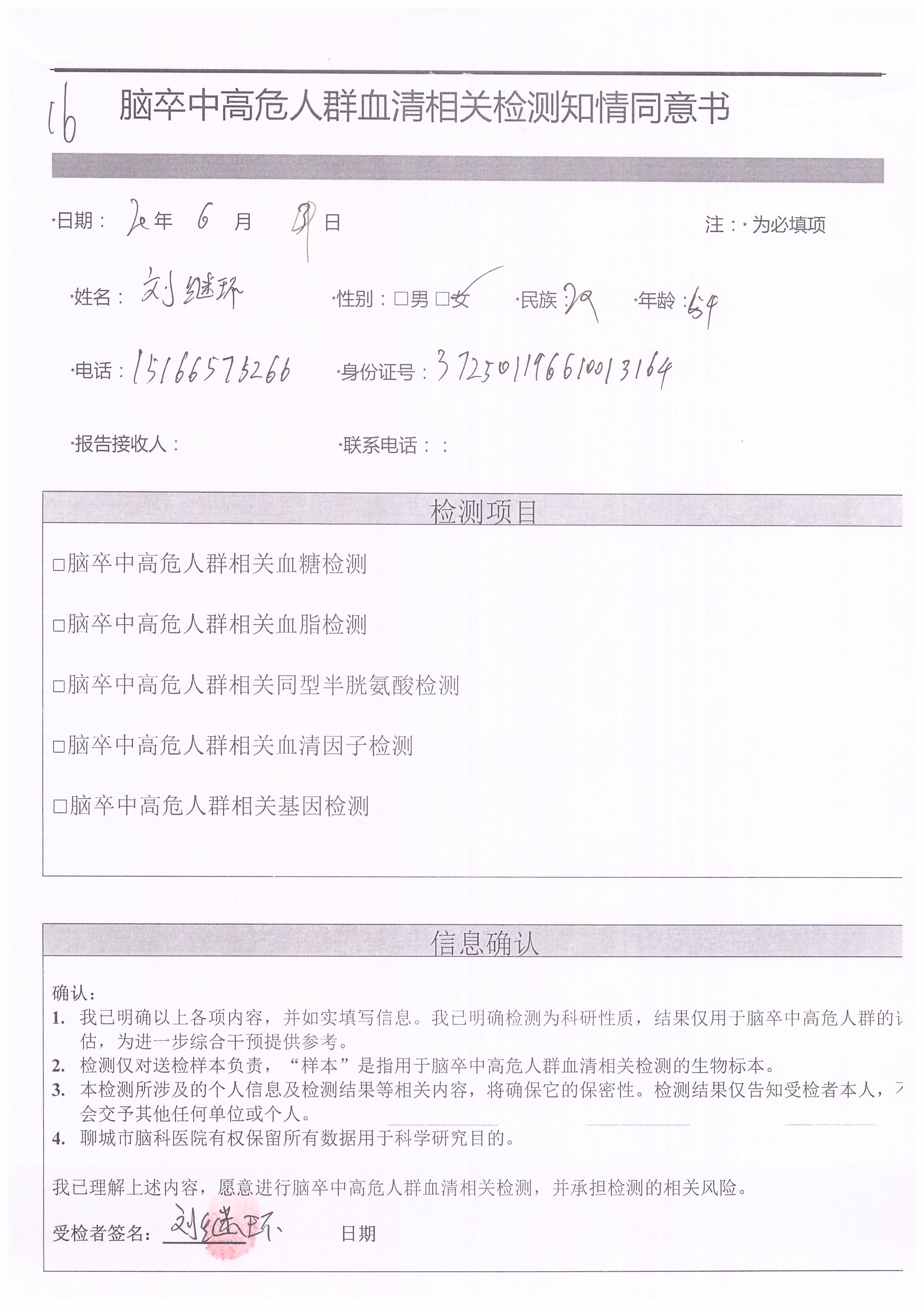

Supplement: Supplementary file 15 — Supplementary file15 (ZIP 22488 KB) [file 10528_2023_10431_MOESM15_ESM.zip › ╓¬╟Θ═1⁄4╥Γ╩Θ13/016.jpg]

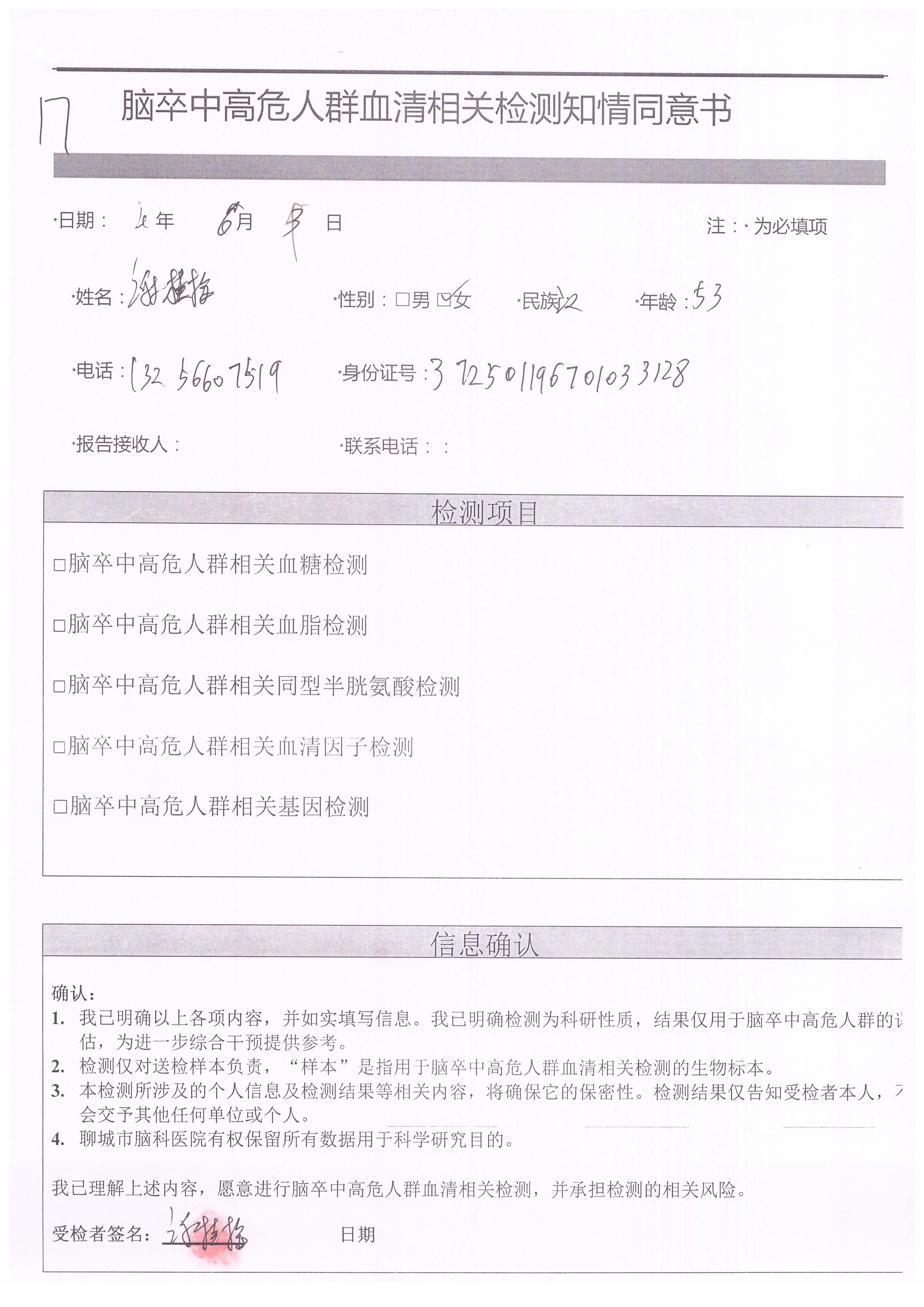

Supplement: Supplementary file 15 — Supplementary file15 (ZIP 22488 KB) [file 10528_2023_10431_MOESM15_ESM.zip › ╓¬╟Θ═1⁄4╥Γ╩Θ13/017.jpg]

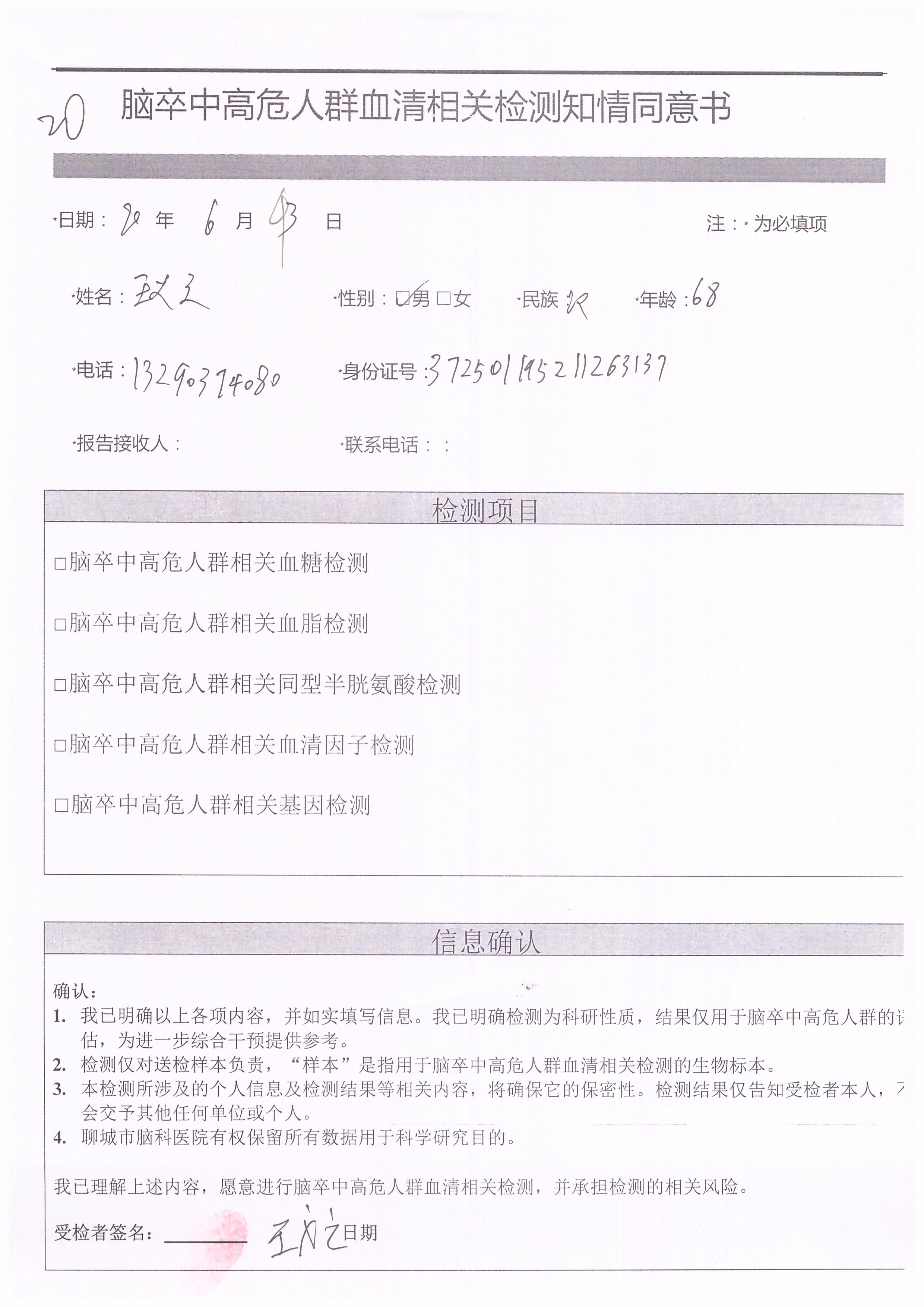

Supplement: Supplementary file 15 — Supplementary file15 (ZIP 22488 KB) [file 10528_2023_10431_MOESM15_ESM.zip › ╓¬╟Θ═1⁄4╥Γ╩Θ13/020.jpg]

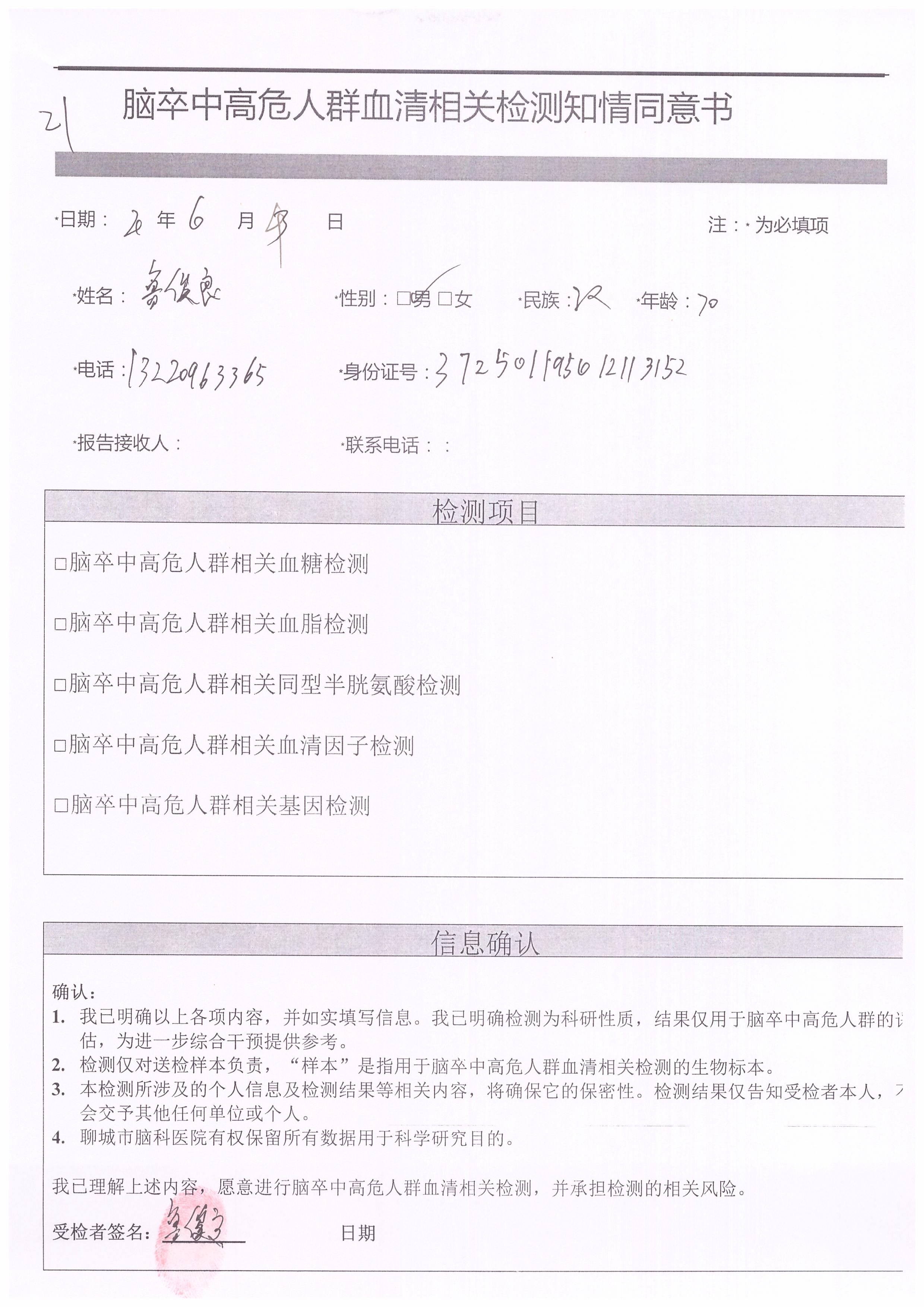

Supplement: Supplementary file 15 — Supplementary file15 (ZIP 22488 KB) [file 10528_2023_10431_MOESM15_ESM.zip › ╓¬╟Θ═1⁄4╥Γ╩Θ13/021.jpg]

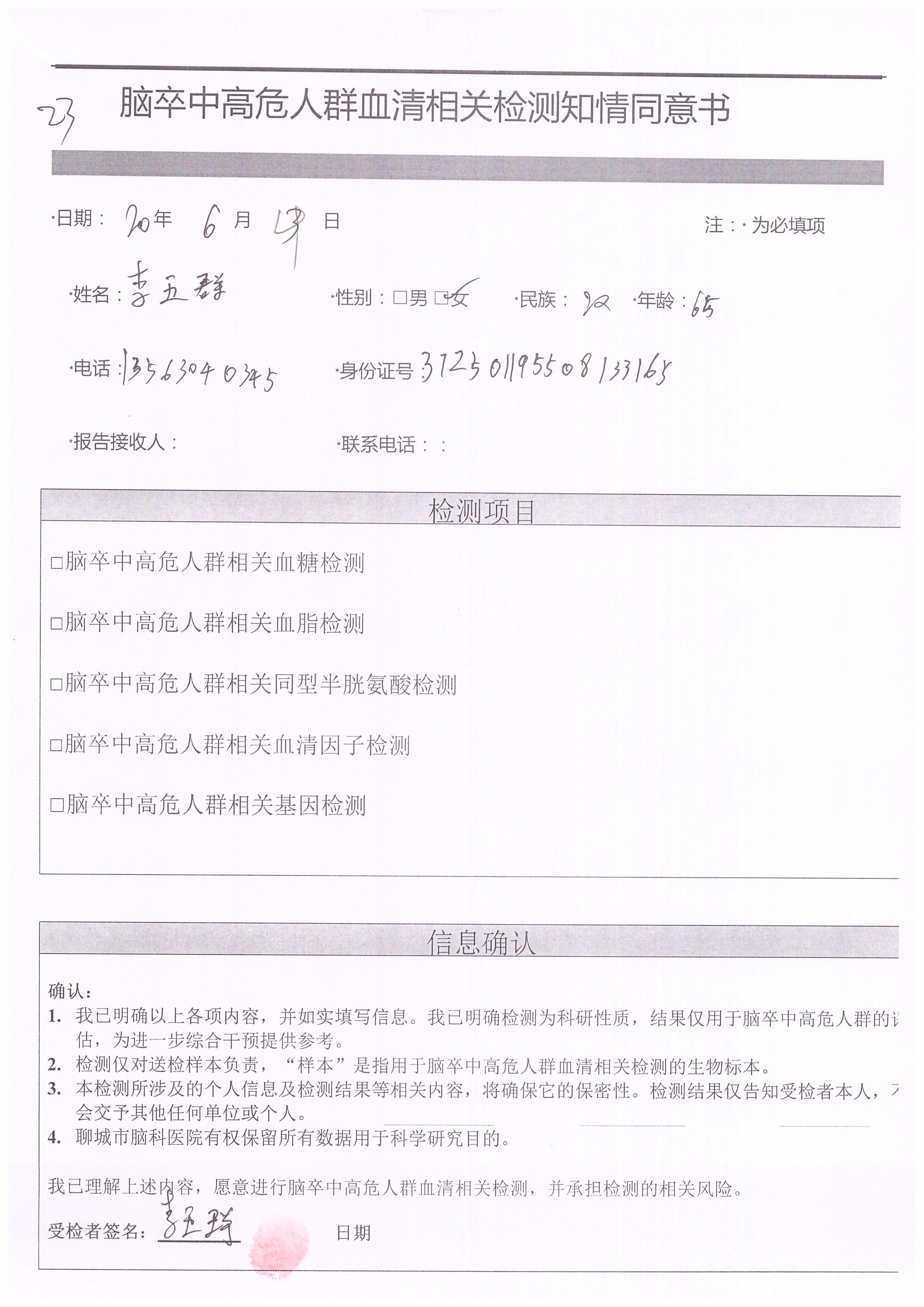

Supplement: Supplementary file 15 — Supplementary file15 (ZIP 22488 KB) [file 10528_2023_10431_MOESM15_ESM.zip › ╓¬╟Θ═1⁄4╥Γ╩Θ13/023.jpg]

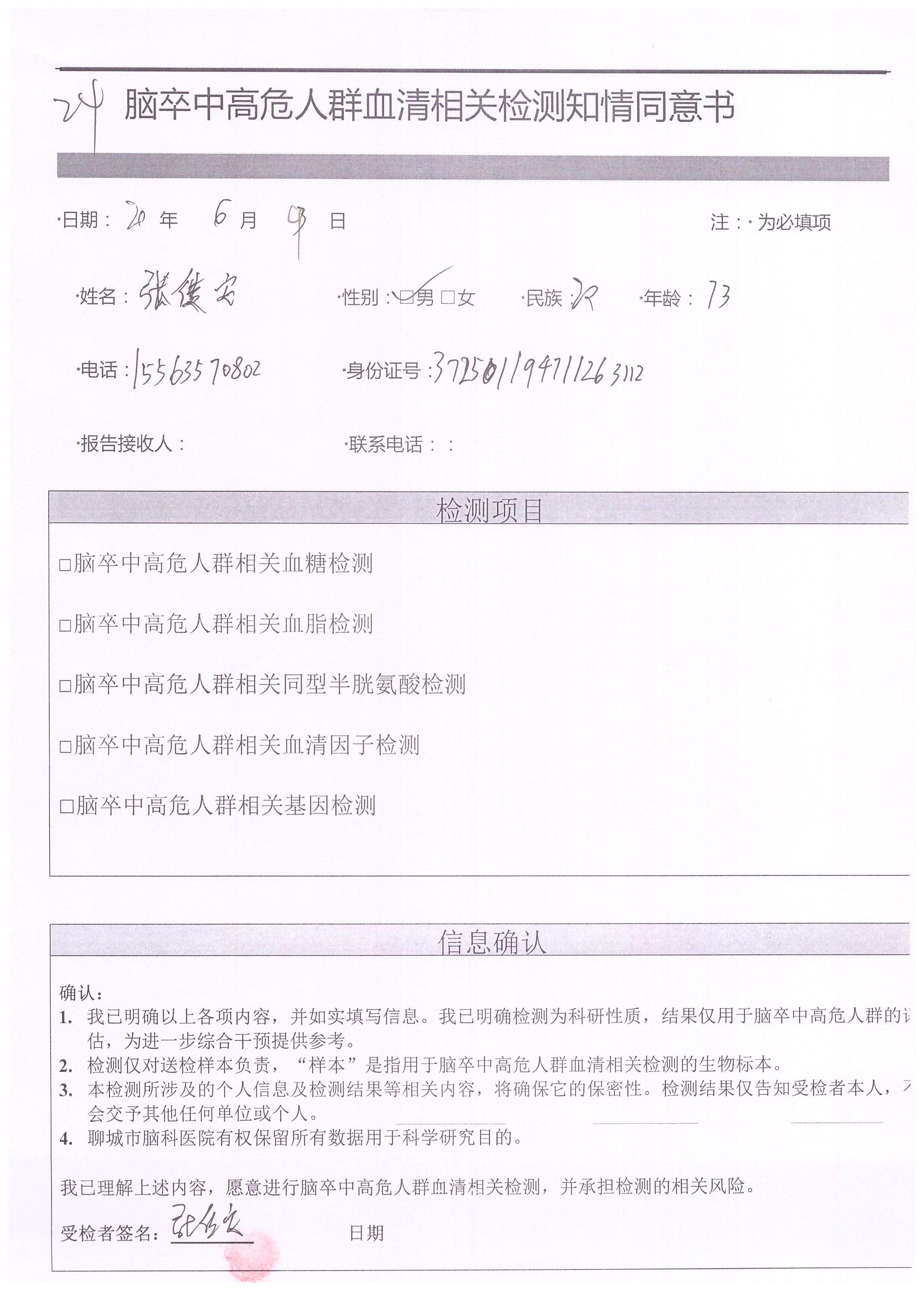

Supplement: Supplementary file 15 — Supplementary file15 (ZIP 22488 KB) [file 10528_2023_10431_MOESM15_ESM.zip › ╓¬╟Θ═1⁄4╥Γ╩Θ13/024.jpg]

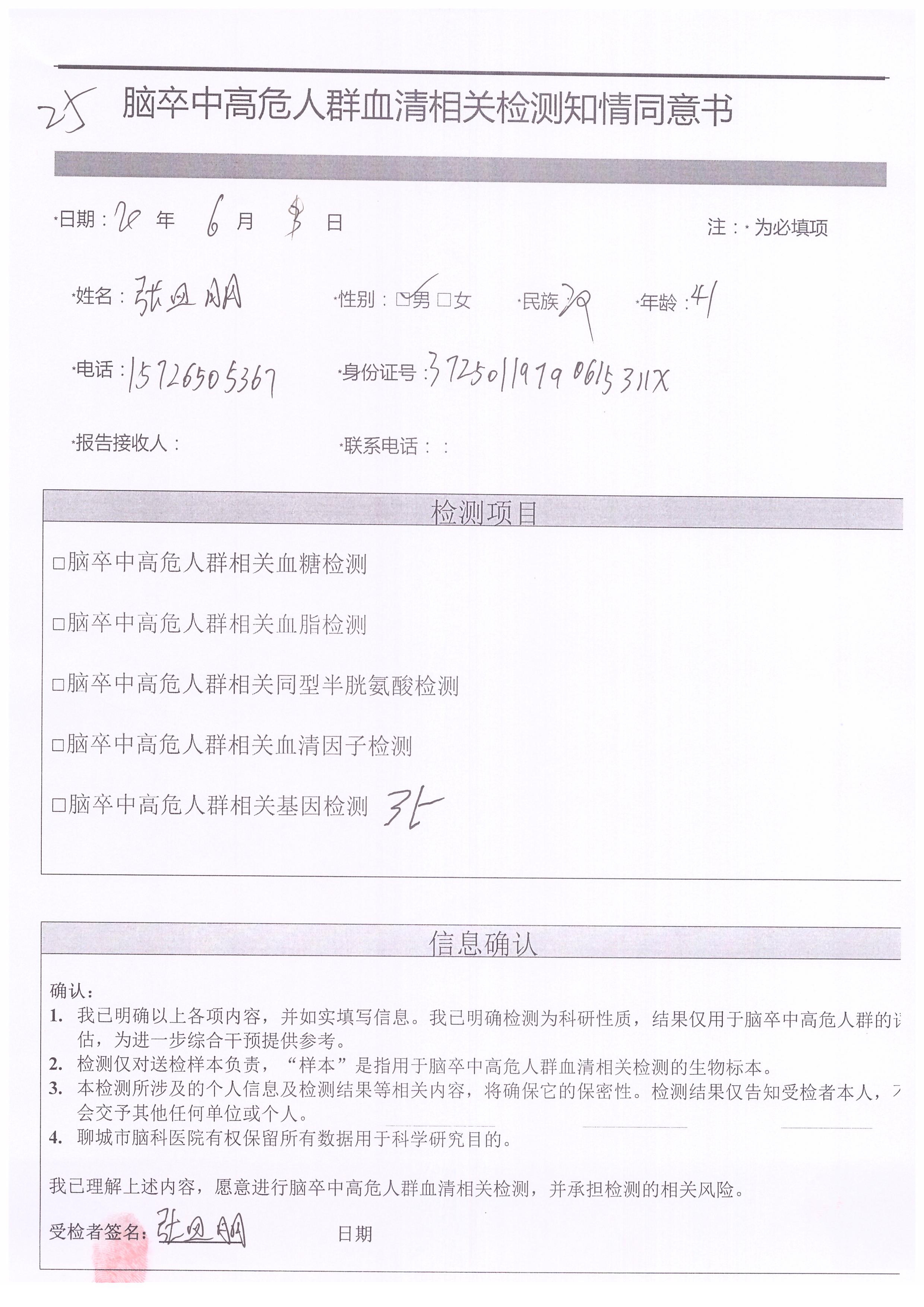

Supplement: Supplementary file 15 — Supplementary file15 (ZIP 22488 KB) [file 10528_2023_10431_MOESM15_ESM.zip › ╓¬╟Θ═1⁄4╥Γ╩Θ13/025.jpg]

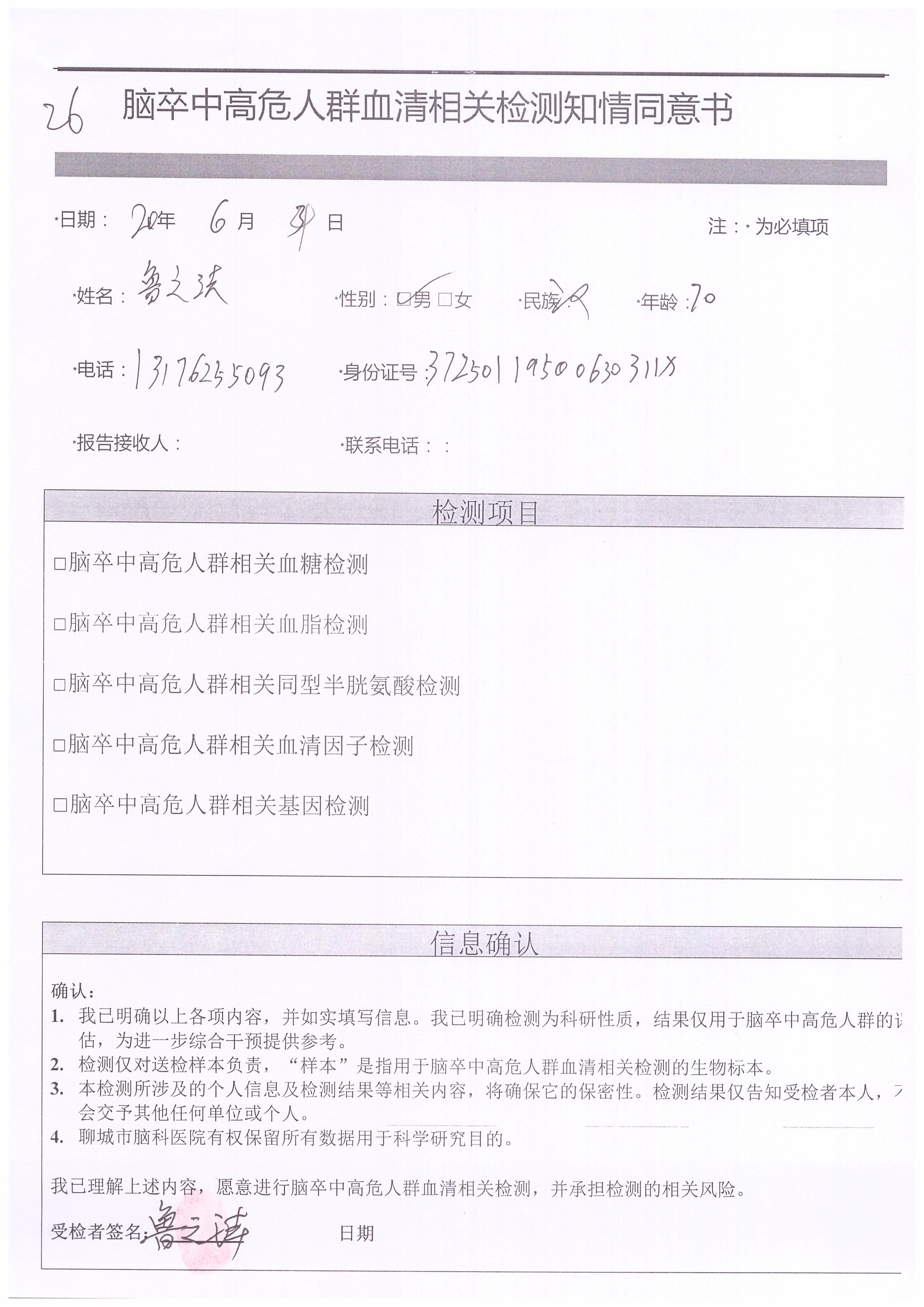

Supplement: Supplementary file 15 — Supplementary file15 (ZIP 22488 KB) [file 10528_2023_10431_MOESM15_ESM.zip › ╓¬╟Θ═1⁄4╥Γ╩Θ13/026.jpg]

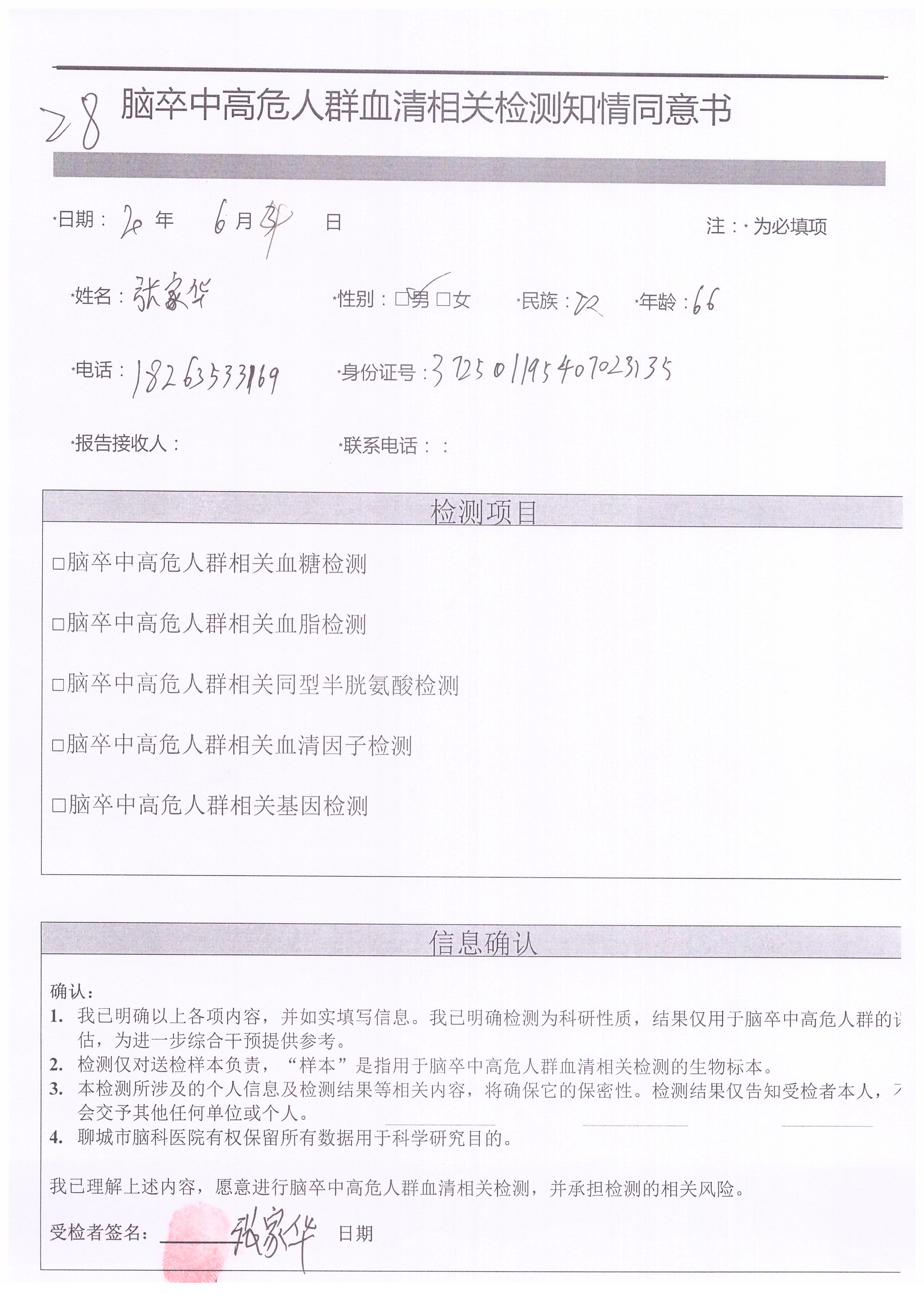

Supplement: Supplementary file 15 — Supplementary file15 (ZIP 22488 KB) [file 10528_2023_10431_MOESM15_ESM.zip › ╓¬╟Θ═1⁄4╥Γ╩Θ13/028.jpg]

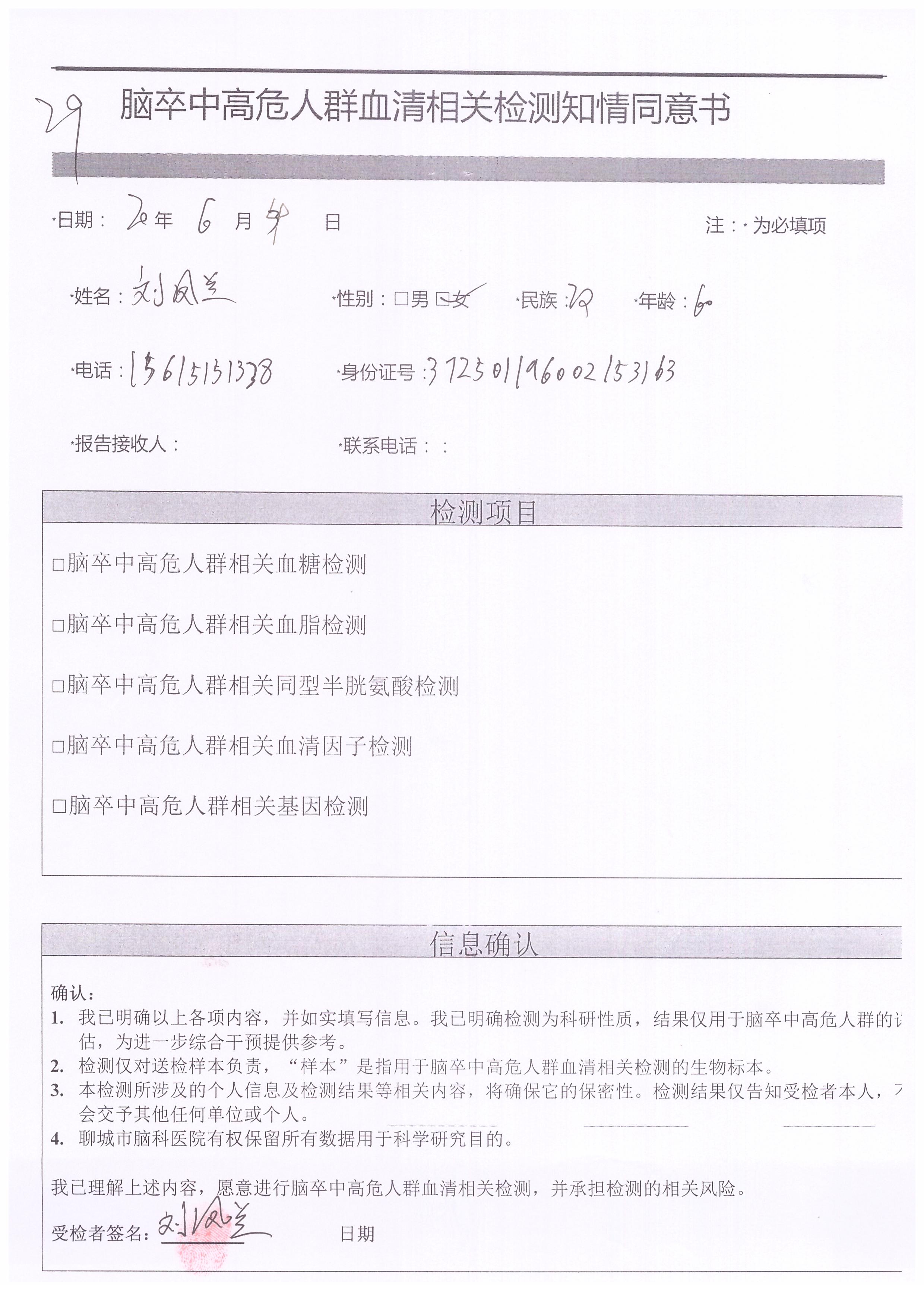

Supplement: Supplementary file 15 — Supplementary file15 (ZIP 22488 KB) [file 10528_2023_10431_MOESM15_ESM.zip › ╓¬╟Θ═1⁄4╥Γ╩Θ13/029.jpg]

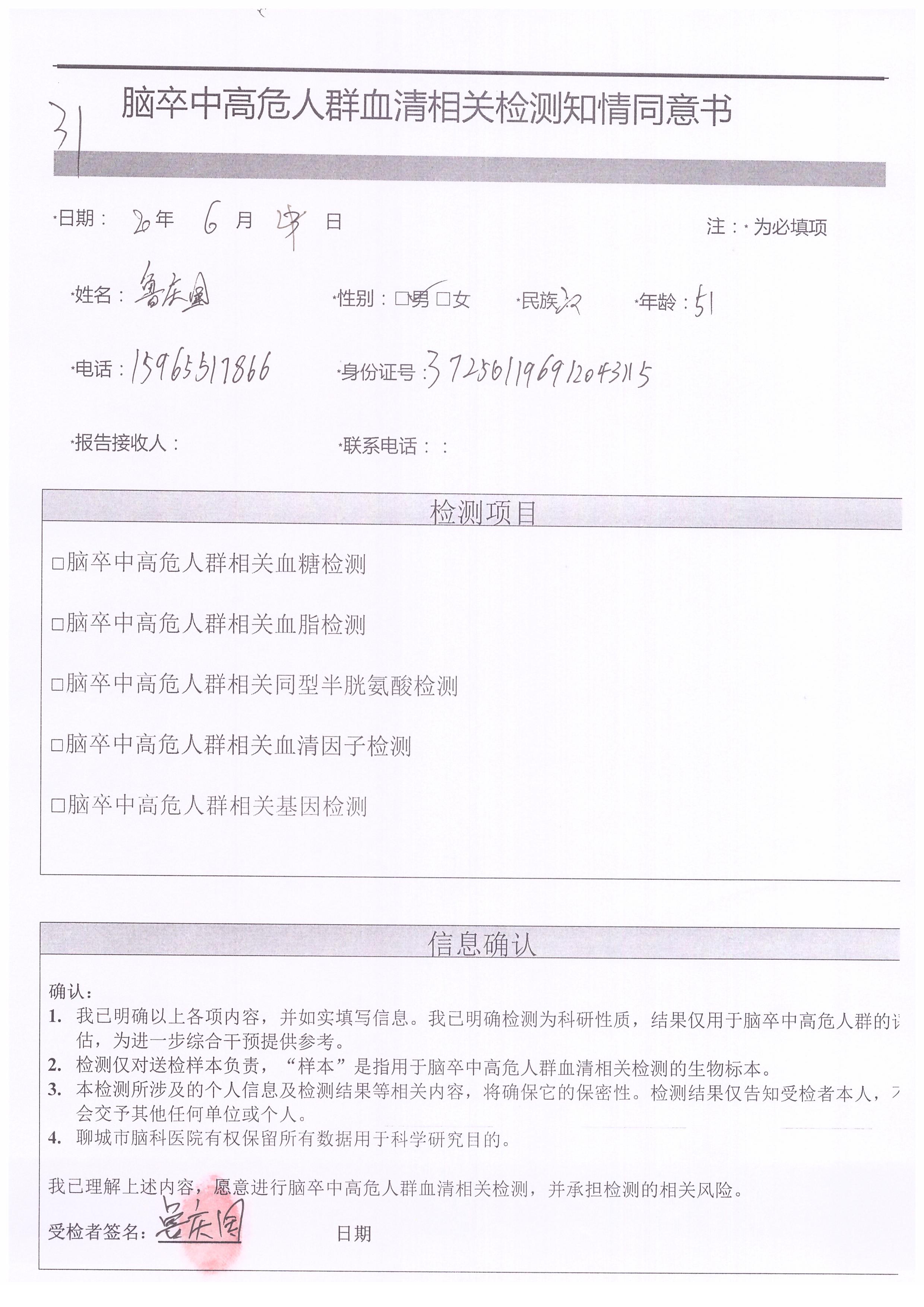

Supplement: Supplementary file 15 — Supplementary file15 (ZIP 22488 KB) [file 10528_2023_10431_MOESM15_ESM.zip › ╓¬╟Θ═1⁄4╥Γ╩Θ13/031.jpg]

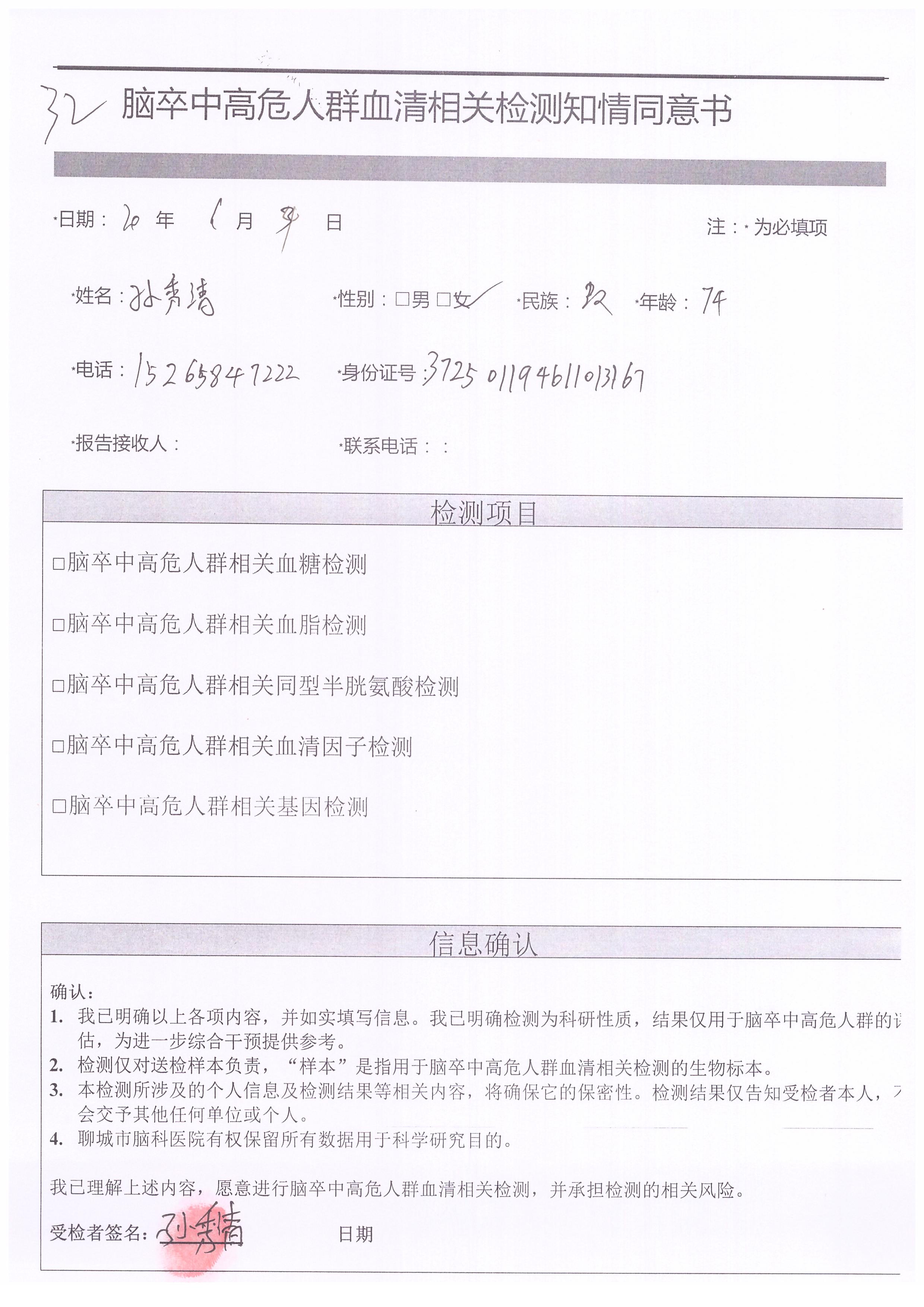

Supplement: Supplementary file 15 — Supplementary file15 (ZIP 22488 KB) [file 10528_2023_10431_MOESM15_ESM.zip › ╓¬╟Θ═1⁄4╥Γ╩Θ13/032.jpg]

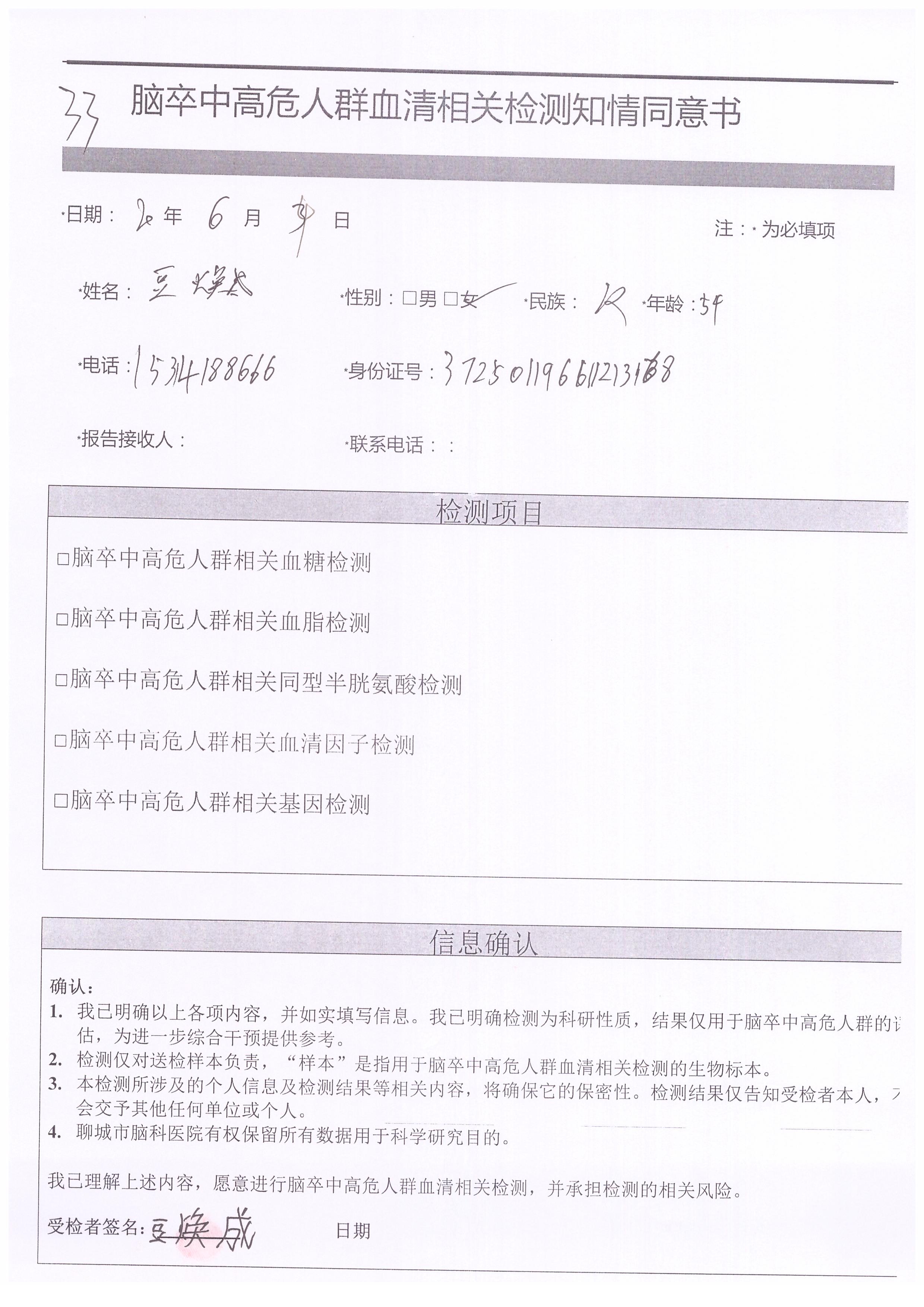

Supplement: Supplementary file 15 — Supplementary file15 (ZIP 22488 KB) [file 10528_2023_10431_MOESM15_ESM.zip › ╓¬╟Θ═1⁄4╥Γ╩Θ13/033.jpg]

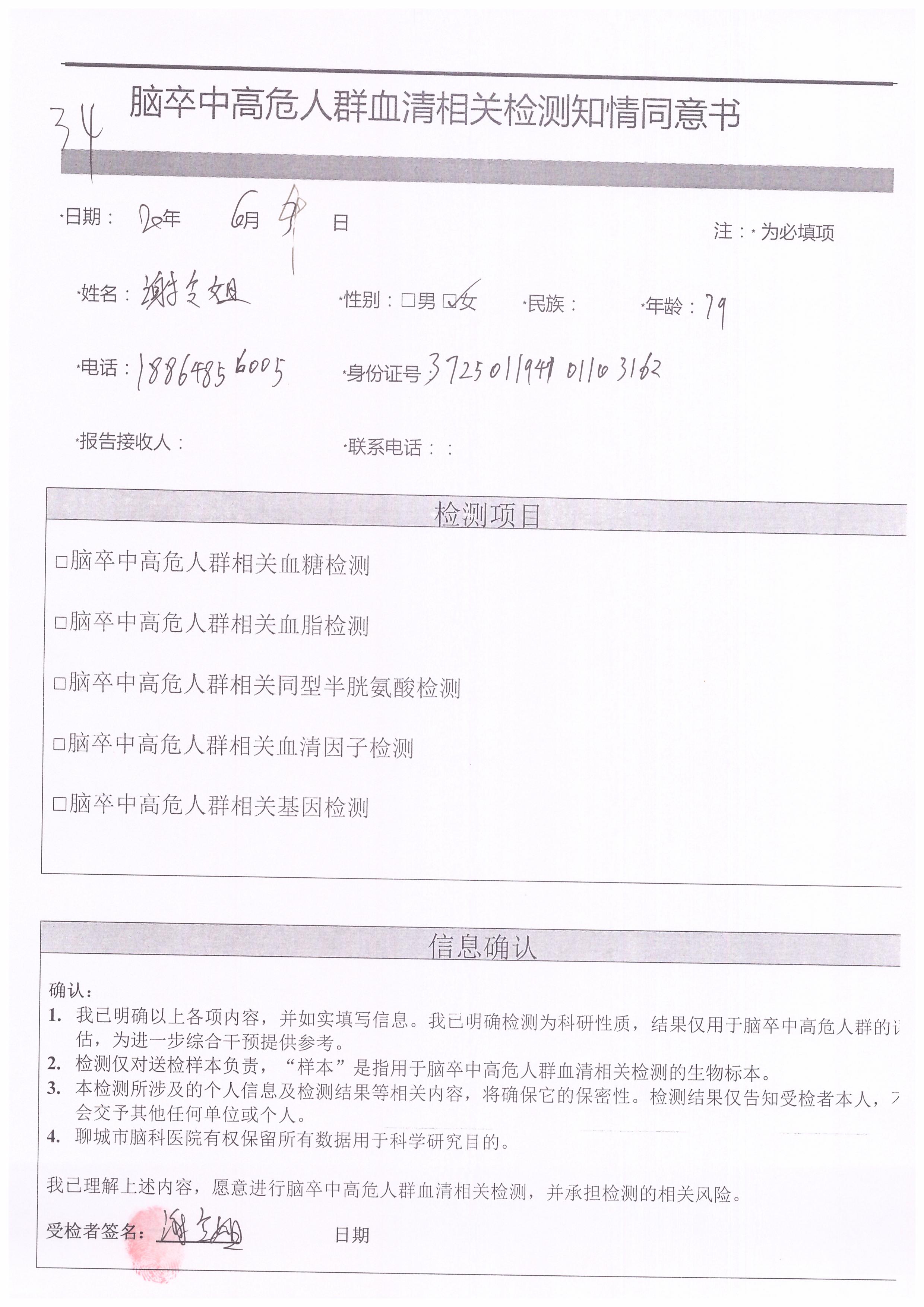

Supplement: Supplementary file 15 — Supplementary file15 (ZIP 22488 KB) [file 10528_2023_10431_MOESM15_ESM.zip › ╓¬╟Θ═1⁄4╥Γ╩Θ13/034.jpg]

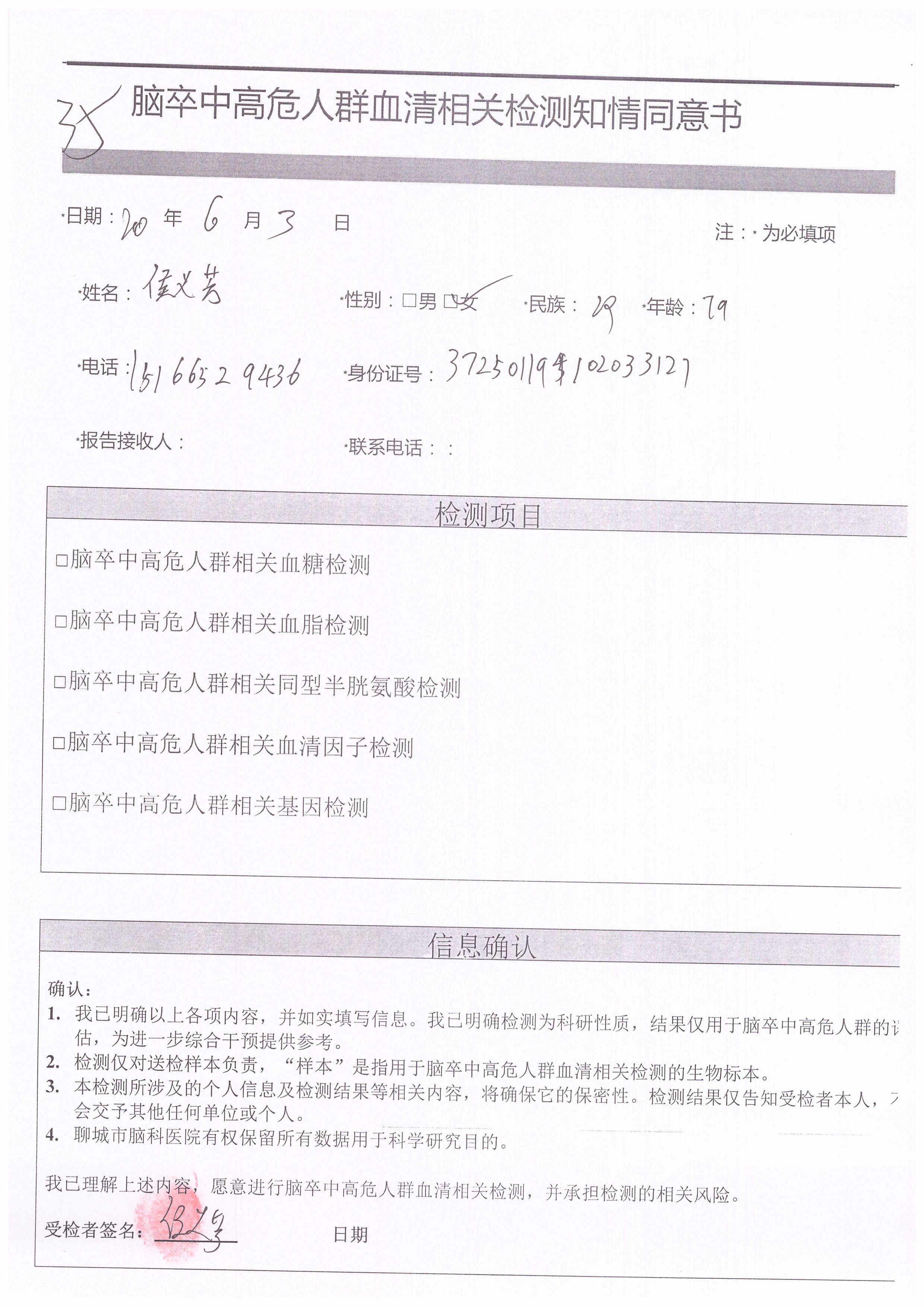

Supplement: Supplementary file 15 — Supplementary file15 (ZIP 22488 KB) [file 10528_2023_10431_MOESM15_ESM.zip › ╓¬╟Θ═1⁄4╥Γ╩Θ13/035.jpg]

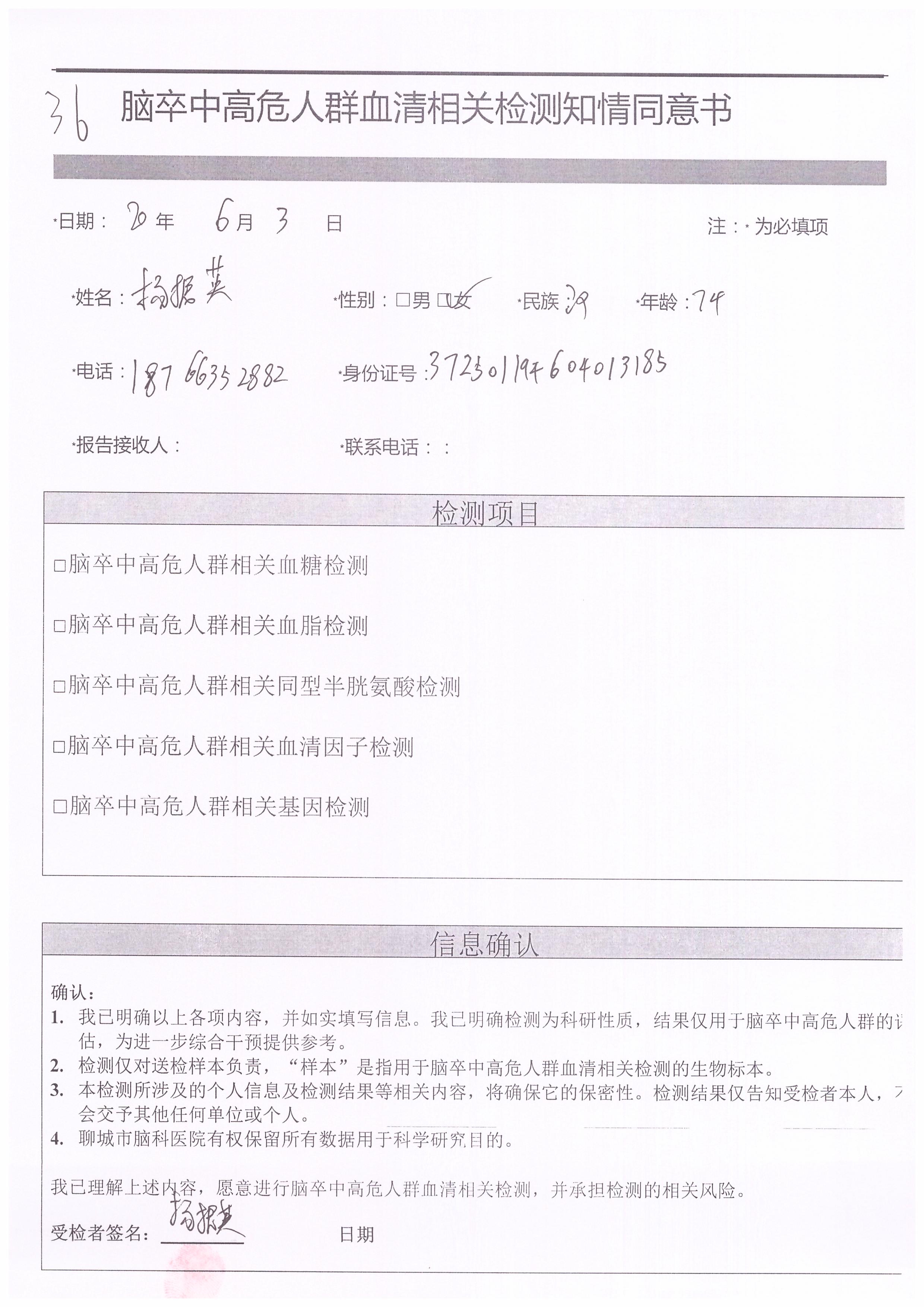

Supplement: Supplementary file 15 — Supplementary file15 (ZIP 22488 KB) [file 10528_2023_10431_MOESM15_ESM.zip › ╓¬╟Θ═1⁄4╥Γ╩Θ13/036.jpg]

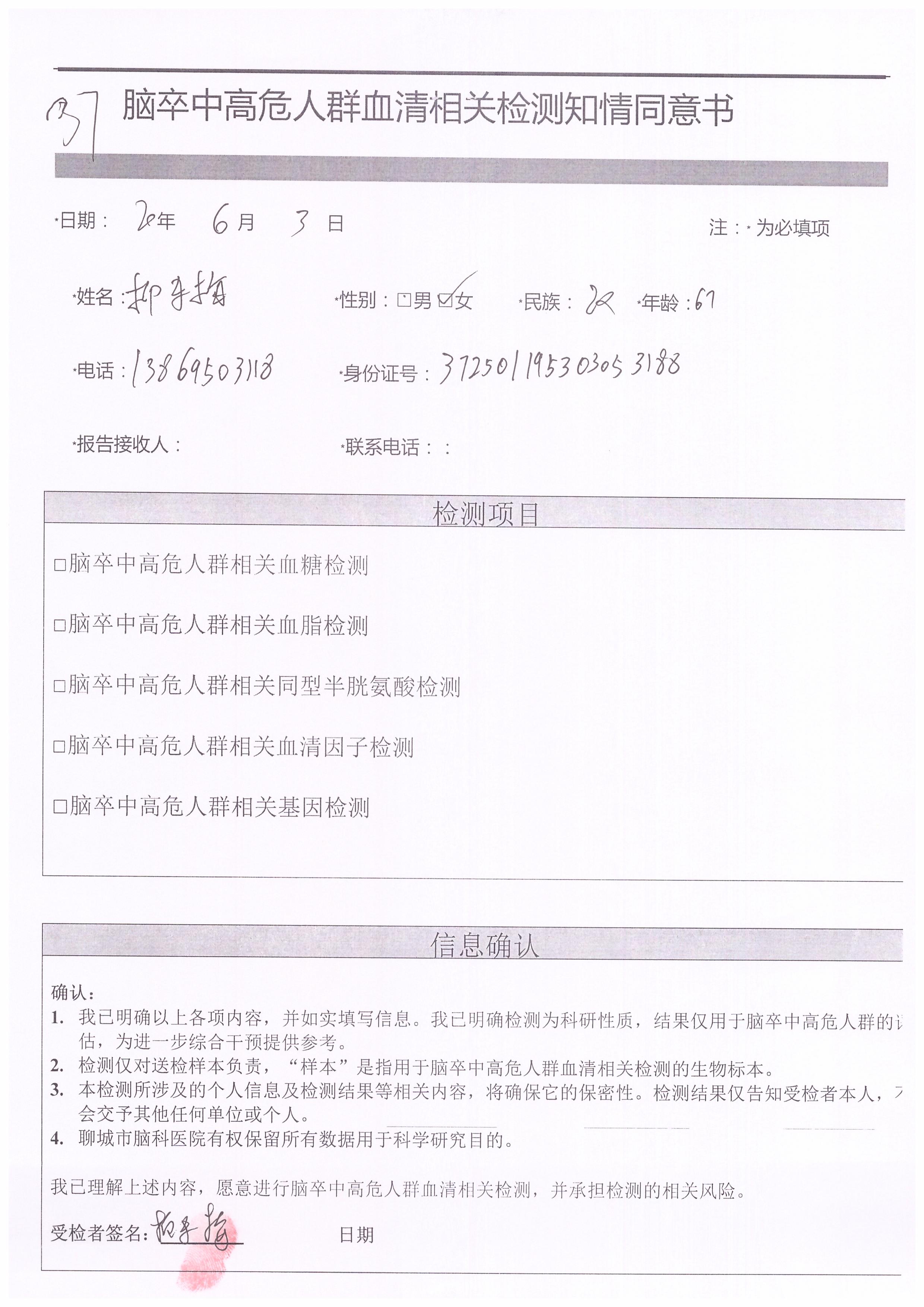

Supplement: Supplementary file 15 — Supplementary file15 (ZIP 22488 KB) [file 10528_2023_10431_MOESM15_ESM.zip › ╓¬╟Θ═1⁄4╥Γ╩Θ13/037.jpg]

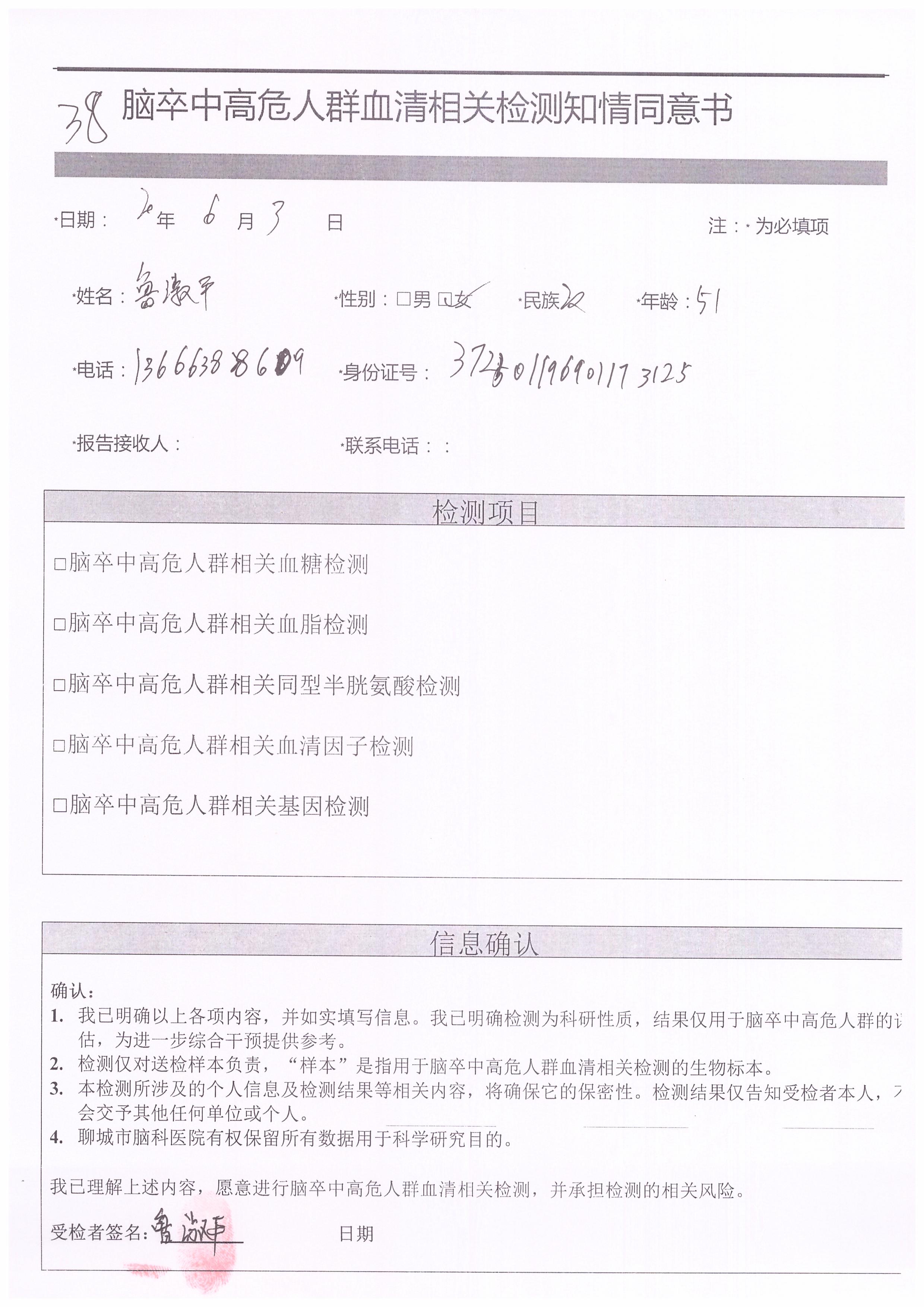

Supplement: Supplementary file 15 — Supplementary file15 (ZIP 22488 KB) [file 10528_2023_10431_MOESM15_ESM.zip › ╓¬╟Θ═1⁄4╥Γ╩Θ13/038.jpg]

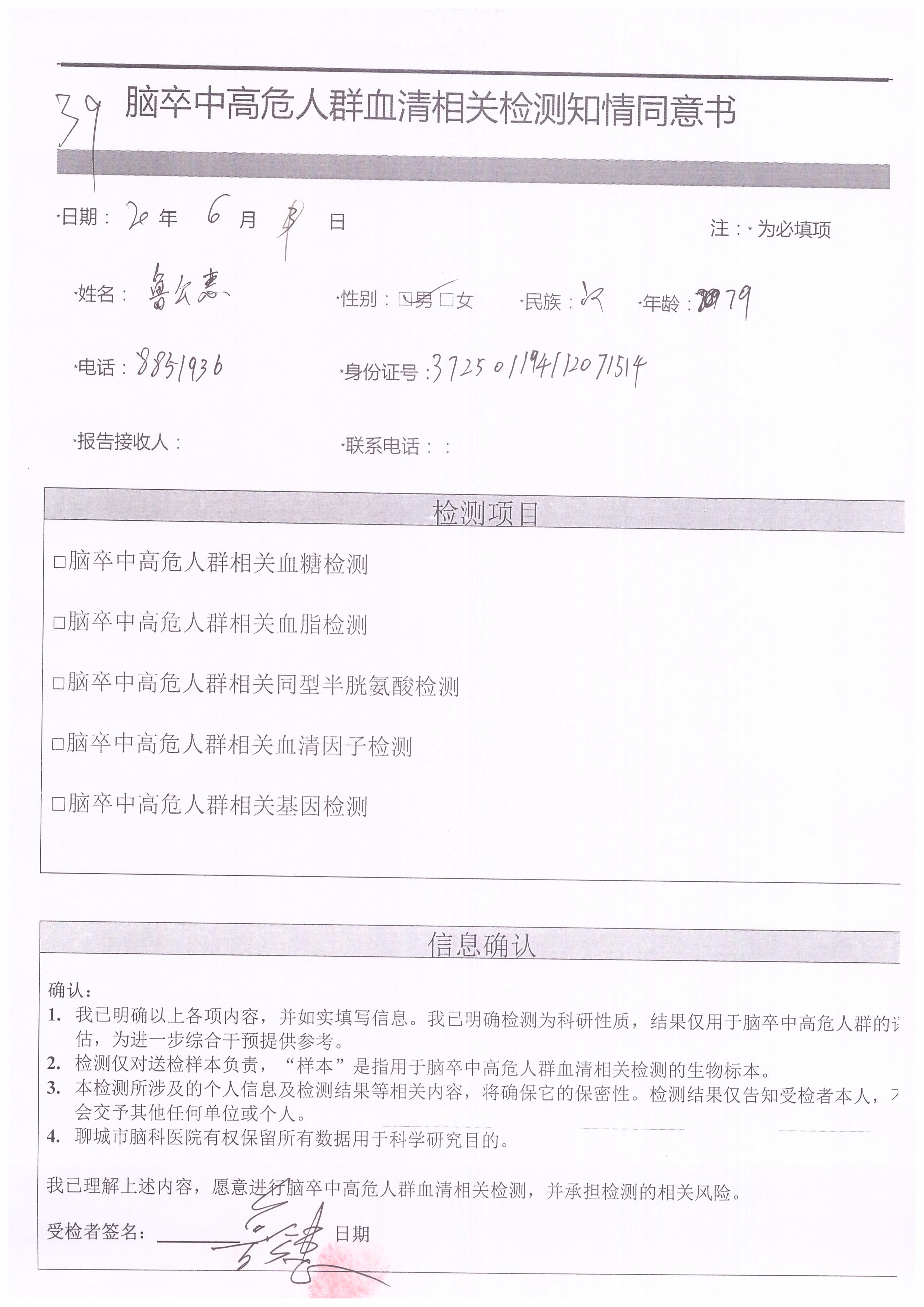

Supplement: Supplementary file 15 — Supplementary file15 (ZIP 22488 KB) [file 10528_2023_10431_MOESM15_ESM.zip › ╓¬╟Θ═1⁄4╥Γ╩Θ13/039.jpg]

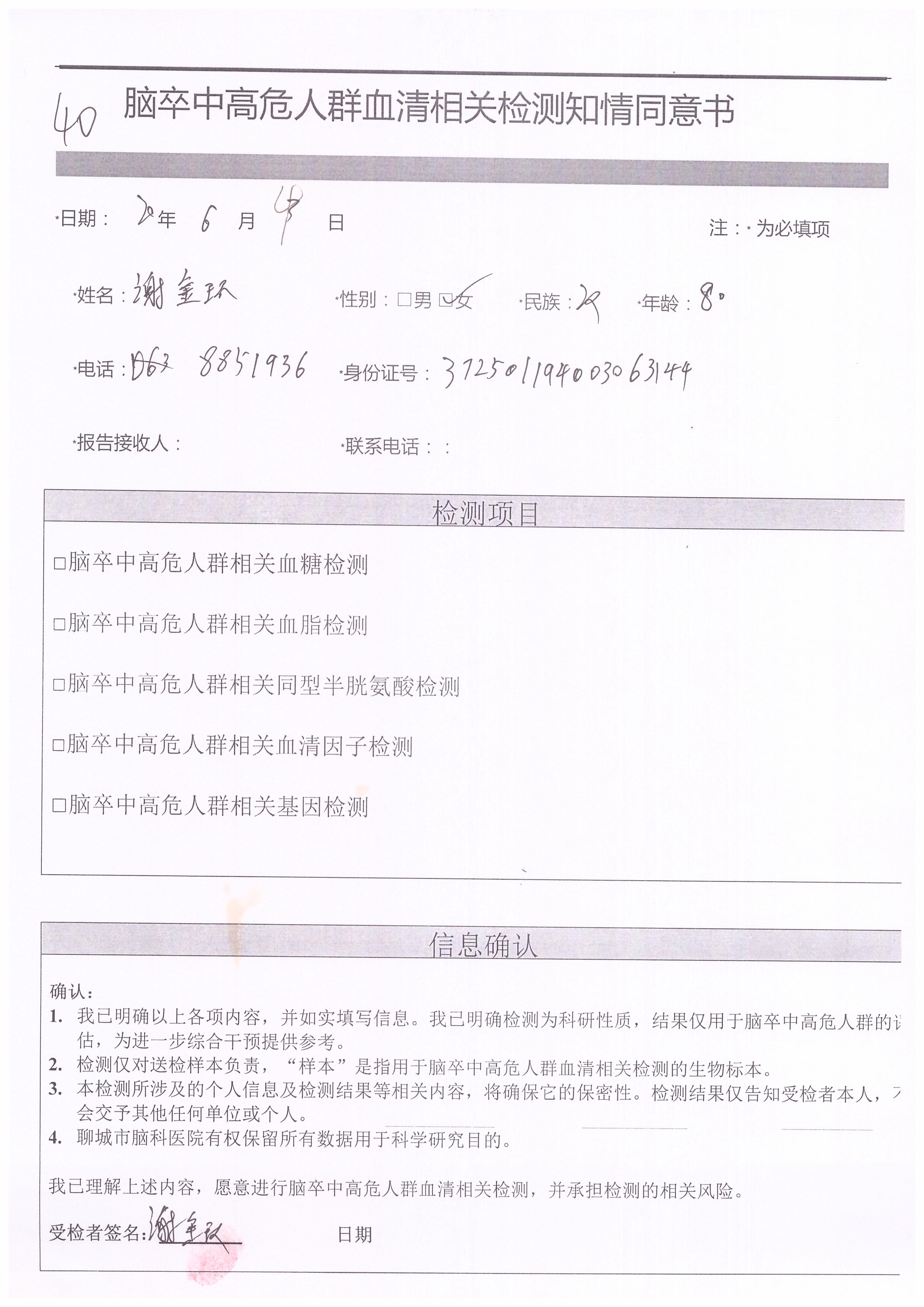

Supplement: Supplementary file 15 — Supplementary file15 (ZIP 22488 KB) [file 10528_2023_10431_MOESM15_ESM.zip › ╓¬╟Θ═1⁄4╥Γ╩Θ13/040.jpg]

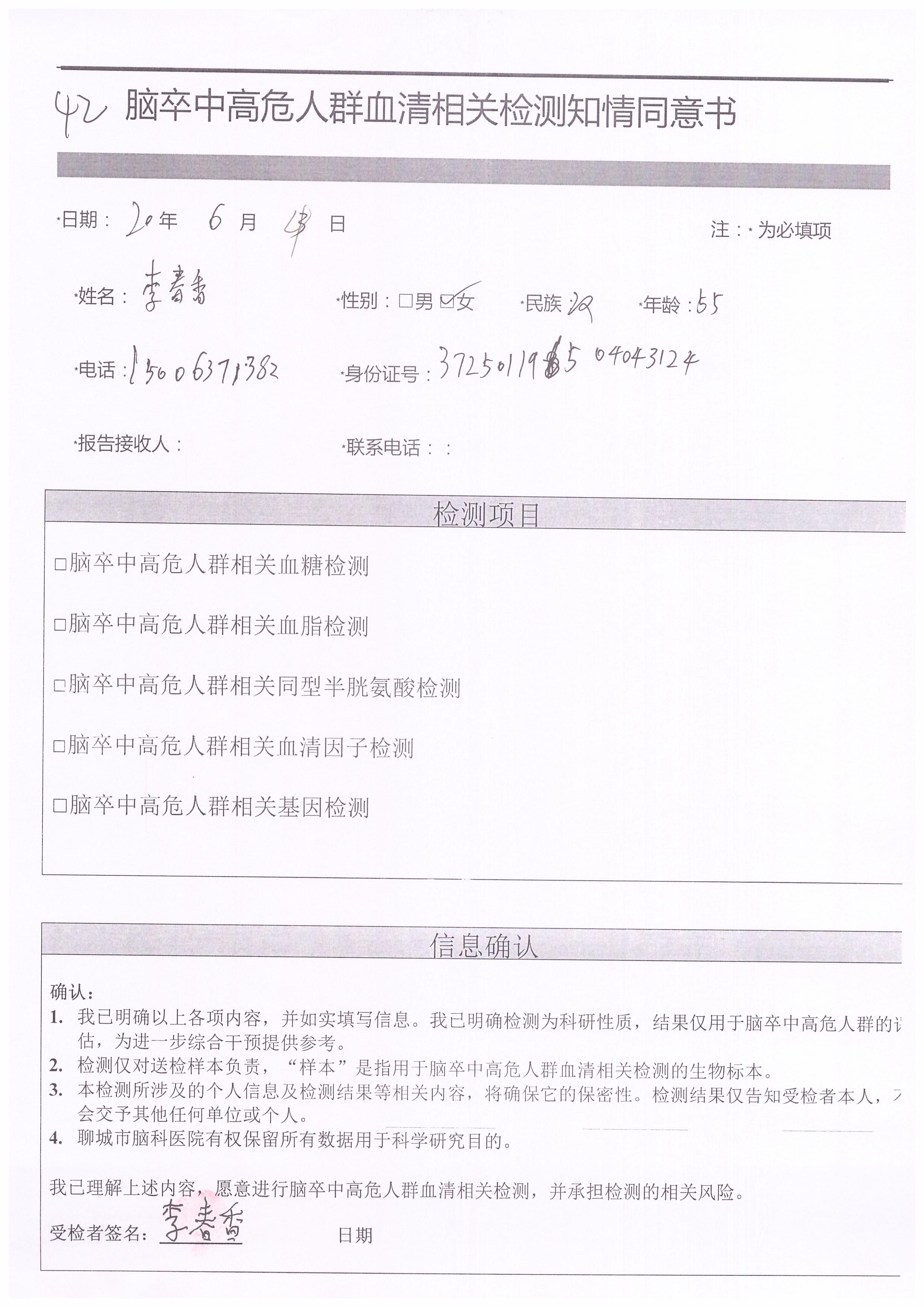

Supplement: Supplementary file 15 — Supplementary file15 (ZIP 22488 KB) [file 10528_2023_10431_MOESM15_ESM.zip › ╓¬╟Θ═1⁄4╥Γ╩Θ13/042.jpg]

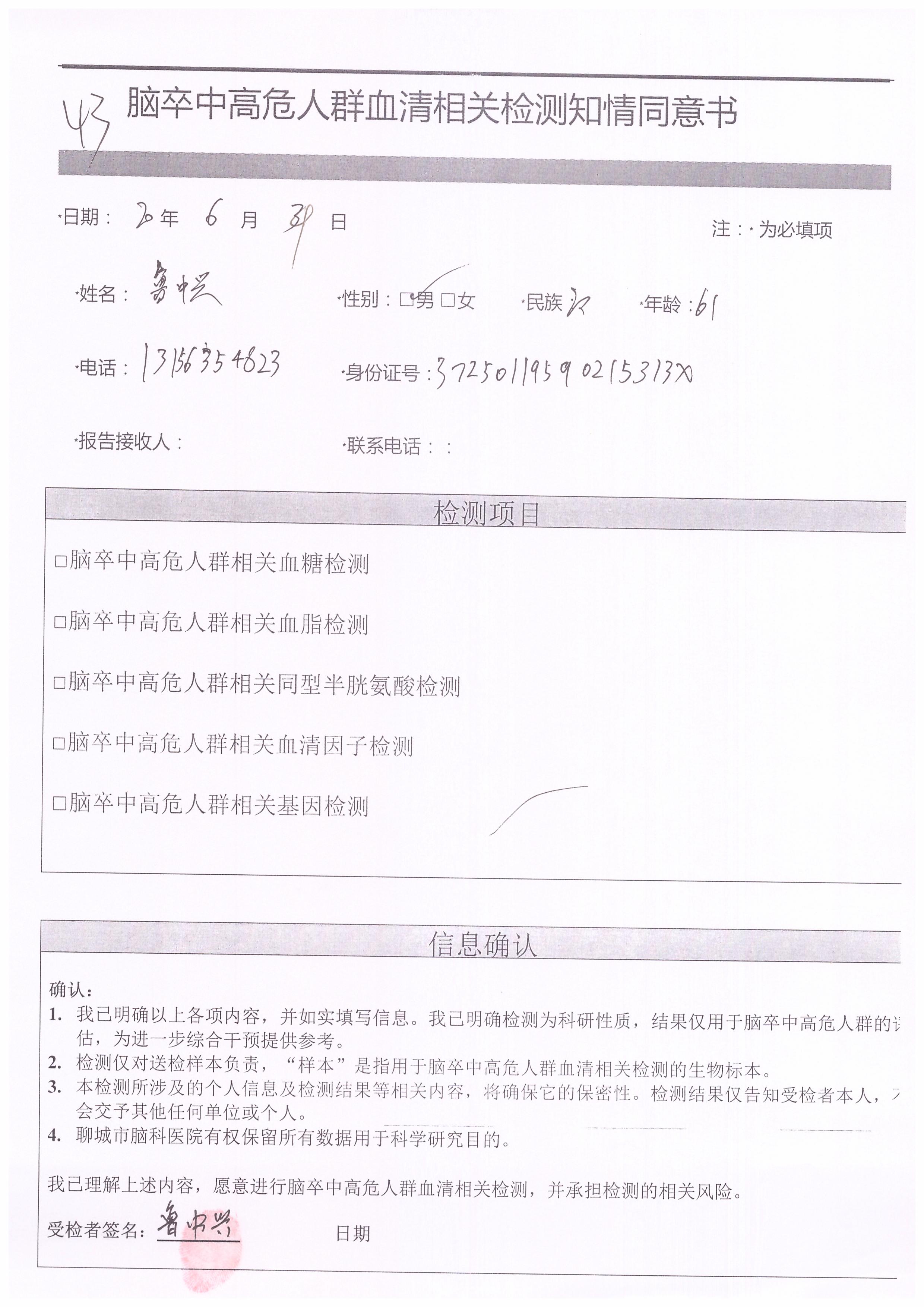

Supplement: Supplementary file 15 — Supplementary file15 (ZIP 22488 KB) [file 10528_2023_10431_MOESM15_ESM.zip › ╓¬╟Θ═1⁄4╥Γ╩Θ13/043.jpg]

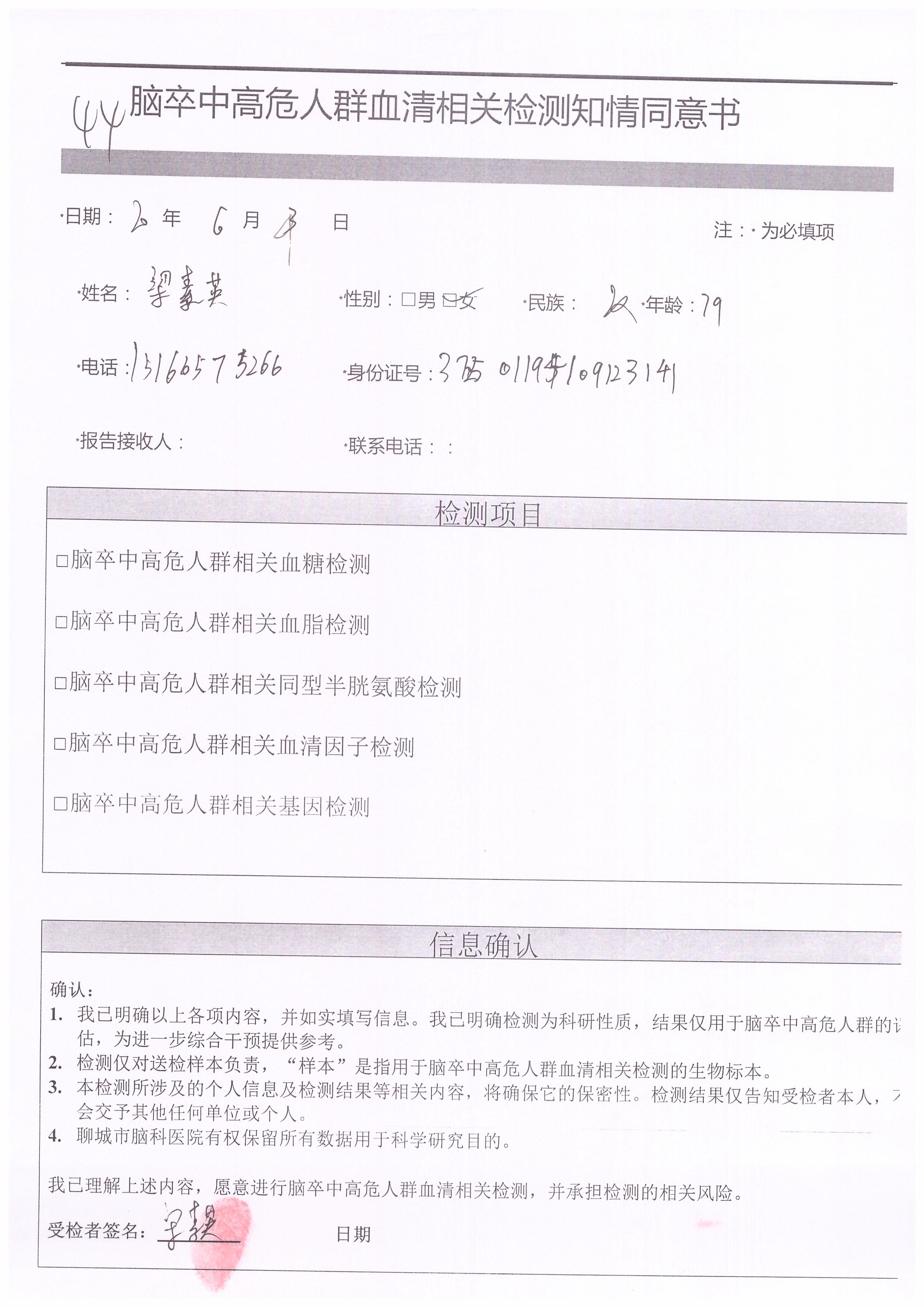

Supplement: Supplementary file 15 — Supplementary file15 (ZIP 22488 KB) [file 10528_2023_10431_MOESM15_ESM.zip › ╓¬╟Θ═1⁄4╥Γ╩Θ13/044.jpg]

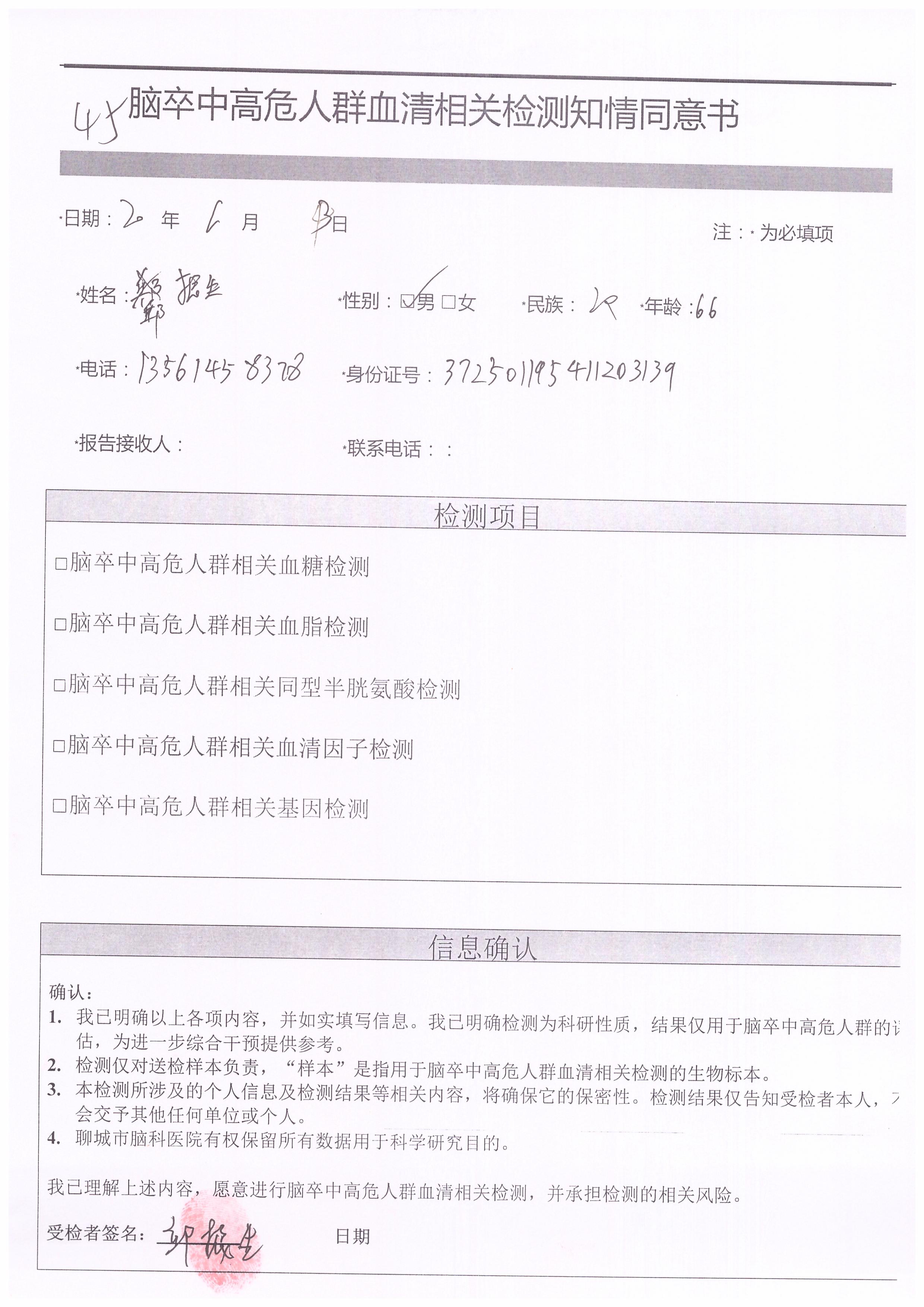

Supplement: Supplementary file 15 — Supplementary file15 (ZIP 22488 KB) [file 10528_2023_10431_MOESM15_ESM.zip › ╓¬╟Θ═1⁄4╥Γ╩Θ13/045.jpg]

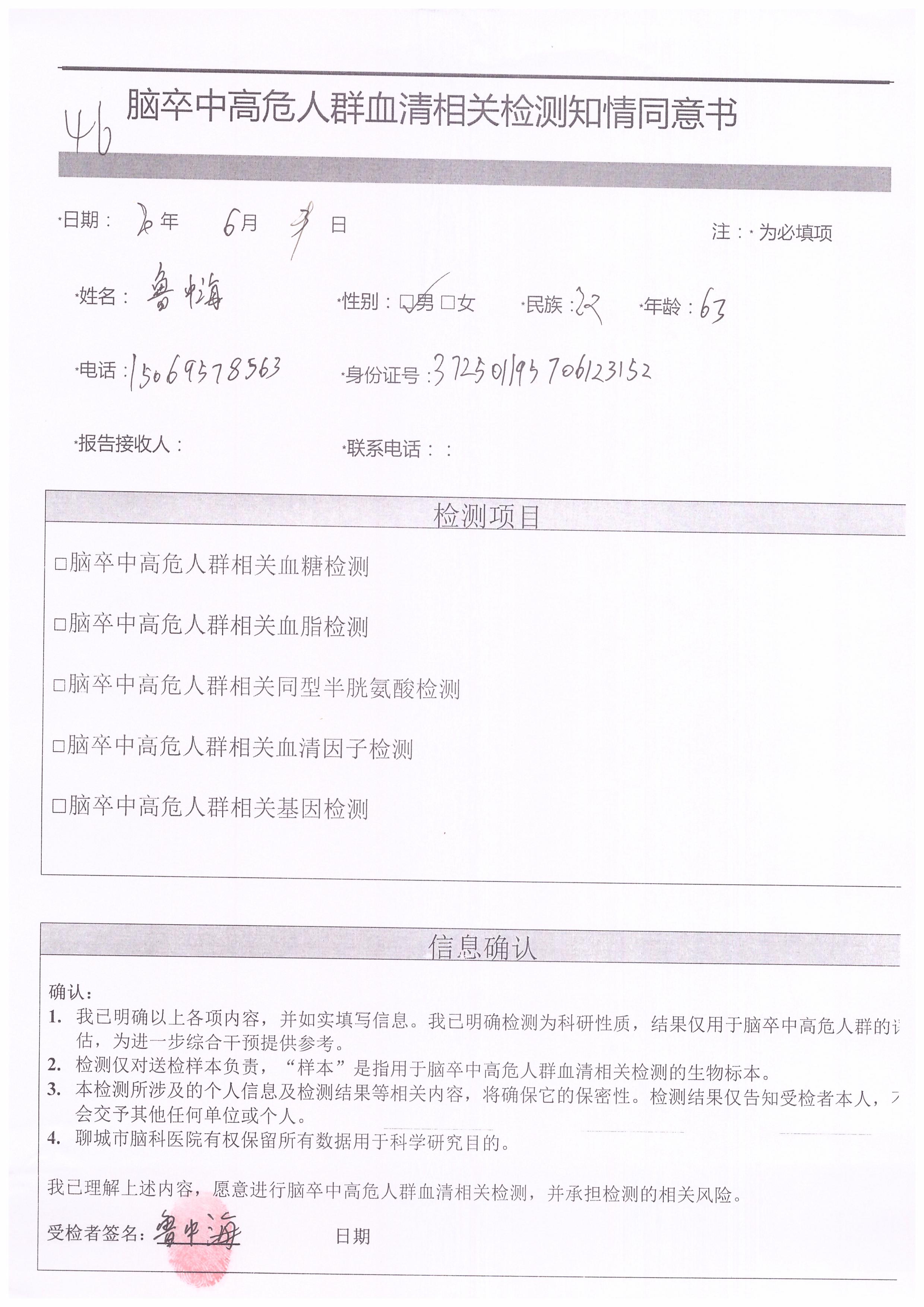

Supplement: Supplementary file 15 — Supplementary file15 (ZIP 22488 KB) [file 10528_2023_10431_MOESM15_ESM.zip › ╓¬╟Θ═1⁄4╥Γ╩Θ13/046.jpg]

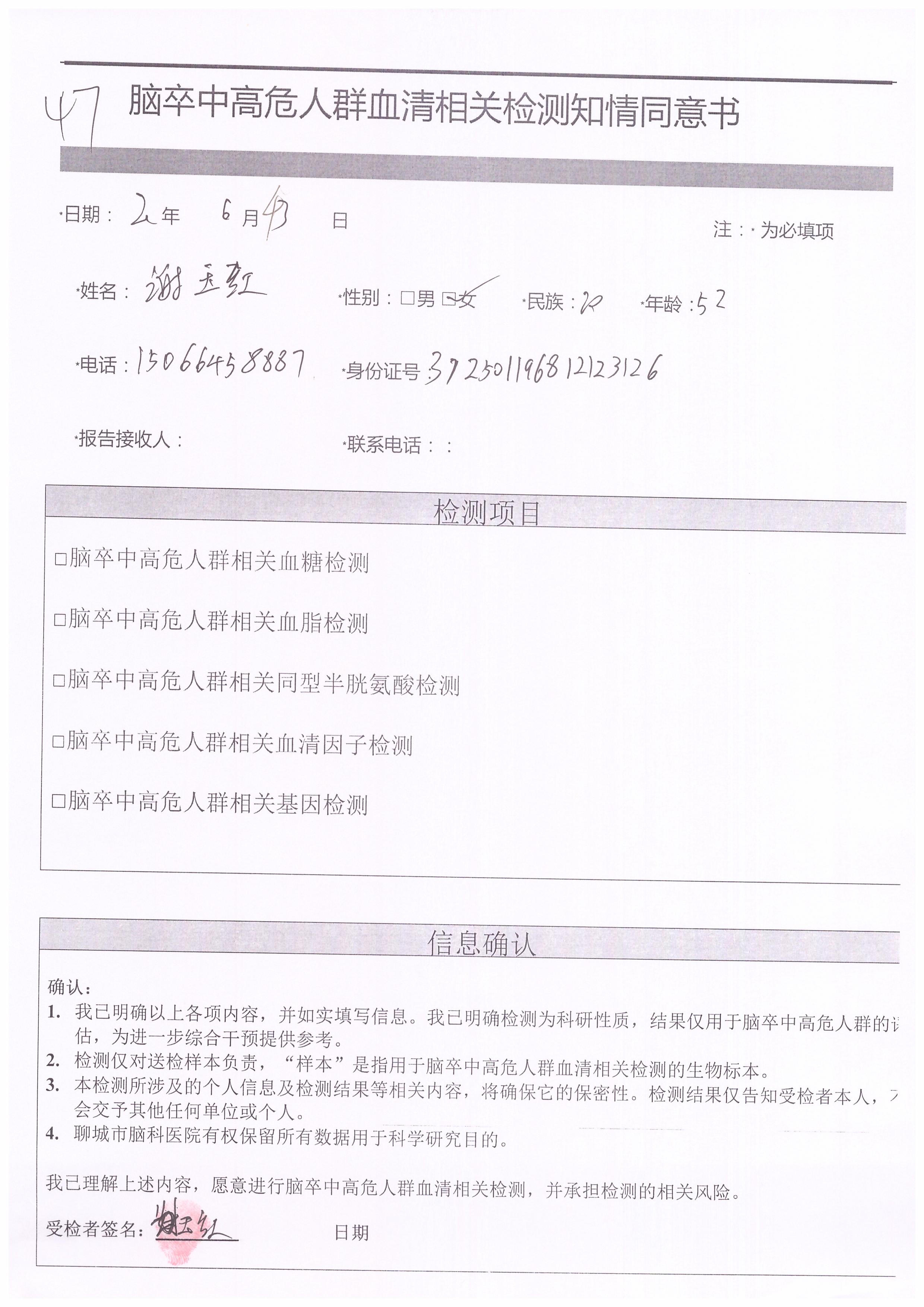

Supplement: Supplementary file 15 — Supplementary file15 (ZIP 22488 KB) [file 10528_2023_10431_MOESM15_ESM.zip › ╓¬╟Θ═1⁄4╥Γ╩Θ13/047.jpg]

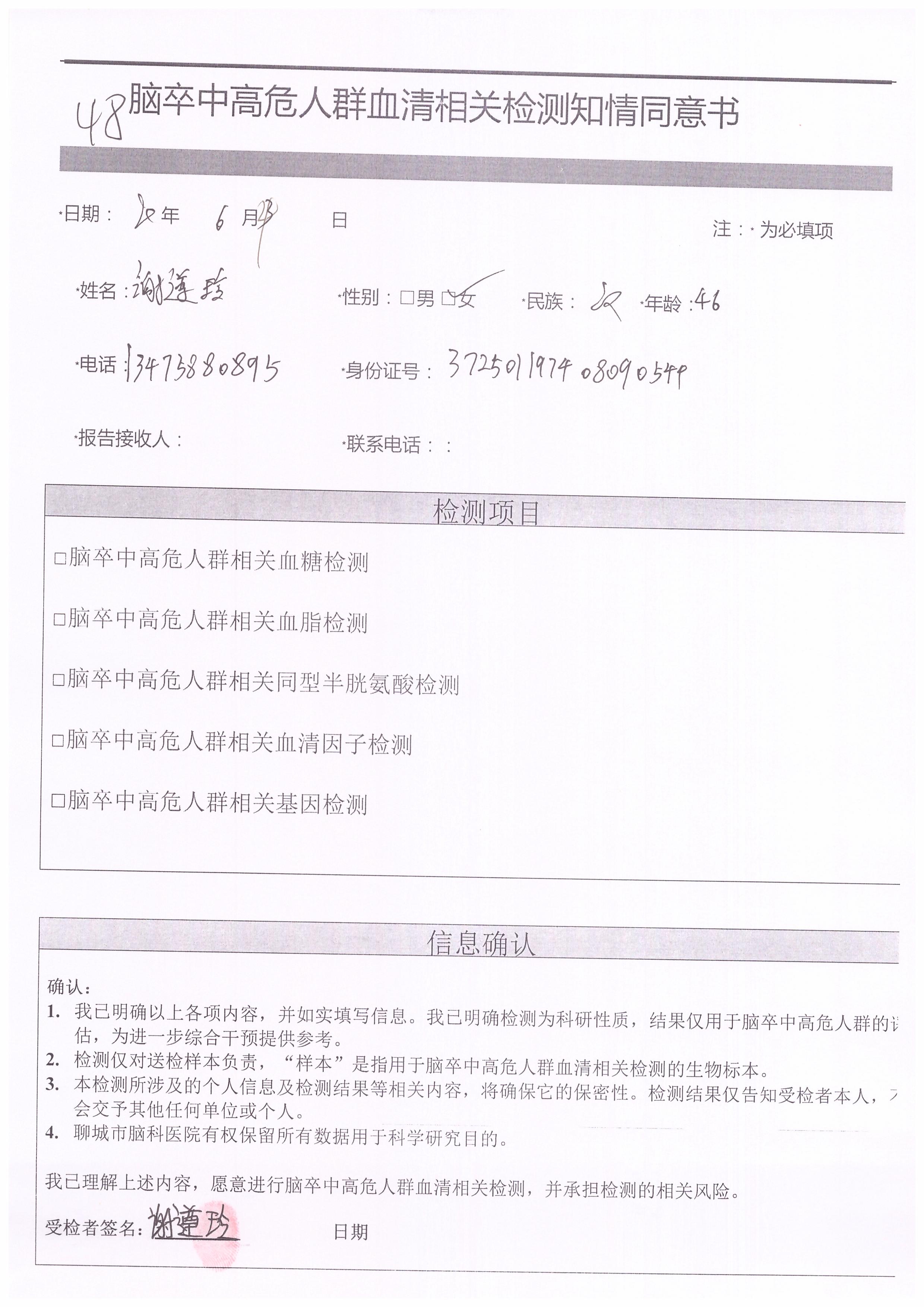

Supplement: Supplementary file 15 — Supplementary file15 (ZIP 22488 KB) [file 10528_2023_10431_MOESM15_ESM.zip › ╓¬╟Θ═1⁄4╥Γ╩Θ13/048.jpg]

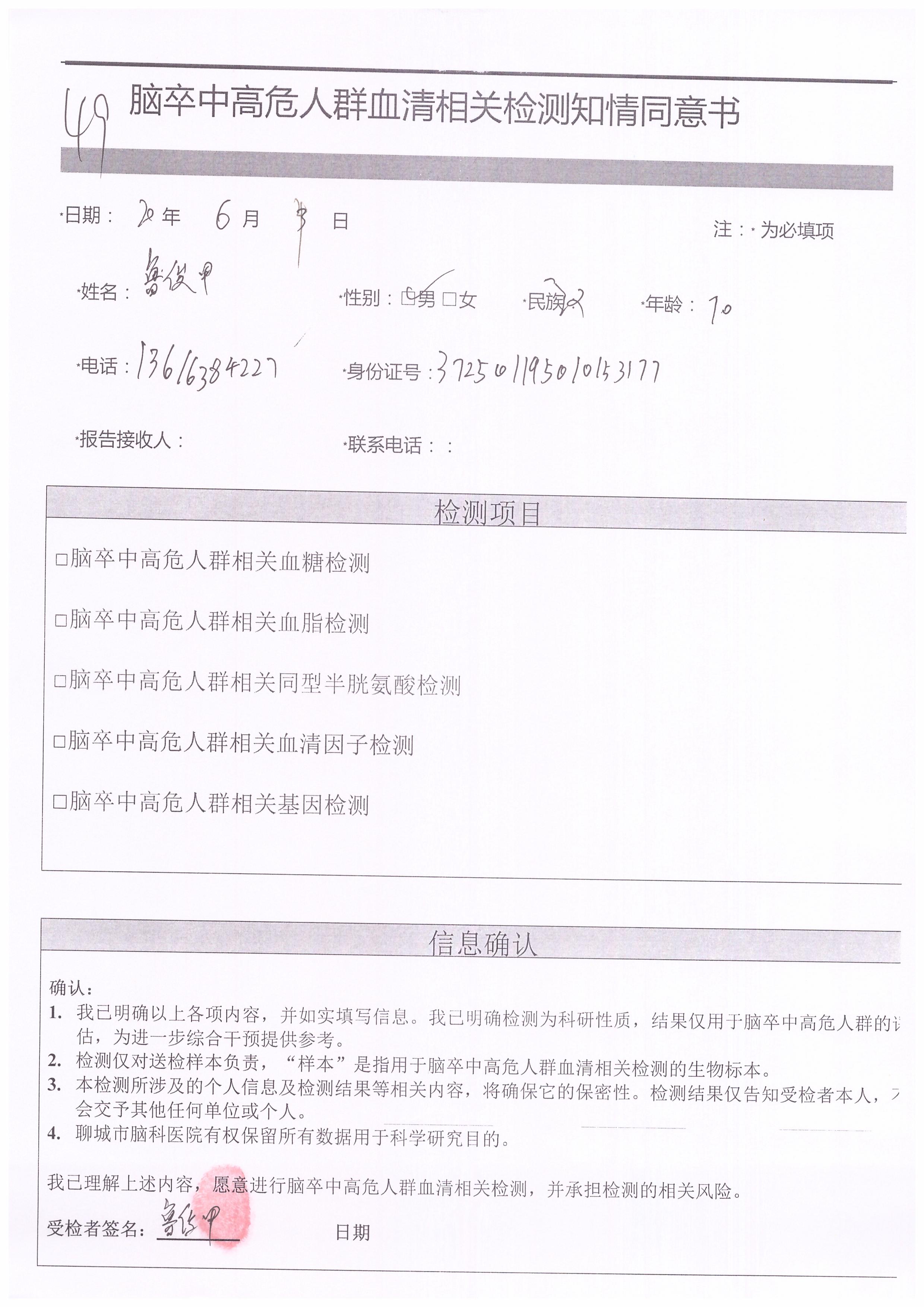

Supplement: Supplementary file 15 — Supplementary file15 (ZIP 22488 KB) [file 10528_2023_10431_MOESM15_ESM.zip › ╓¬╟Θ═1⁄4╥Γ╩Θ13/049.jpg]

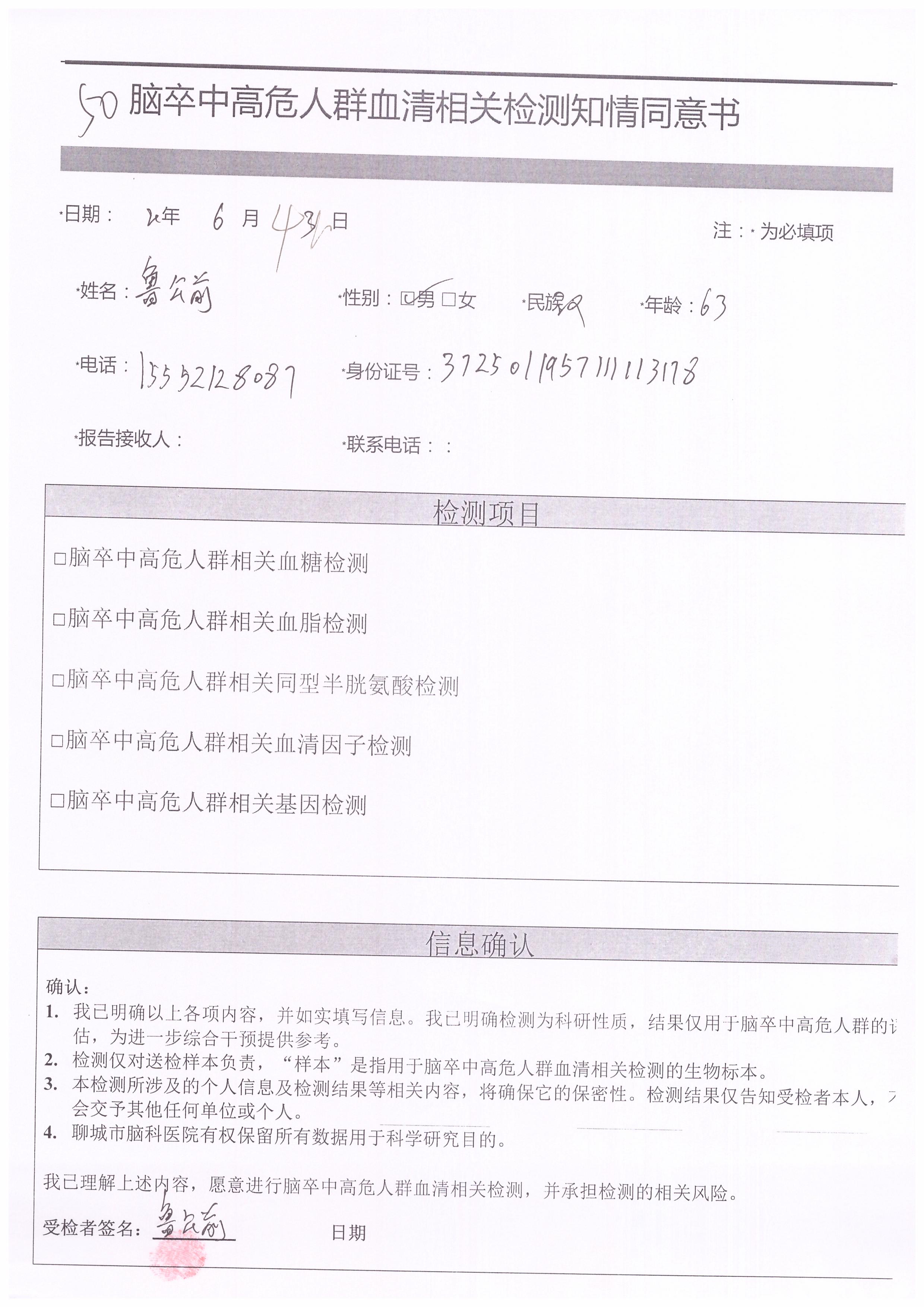

Supplement: Supplementary file 15 — Supplementary file15 (ZIP 22488 KB) [file 10528_2023_10431_MOESM15_ESM.zip › ╓¬╟Θ═1⁄4╥Γ╩Θ13/050.jpg]

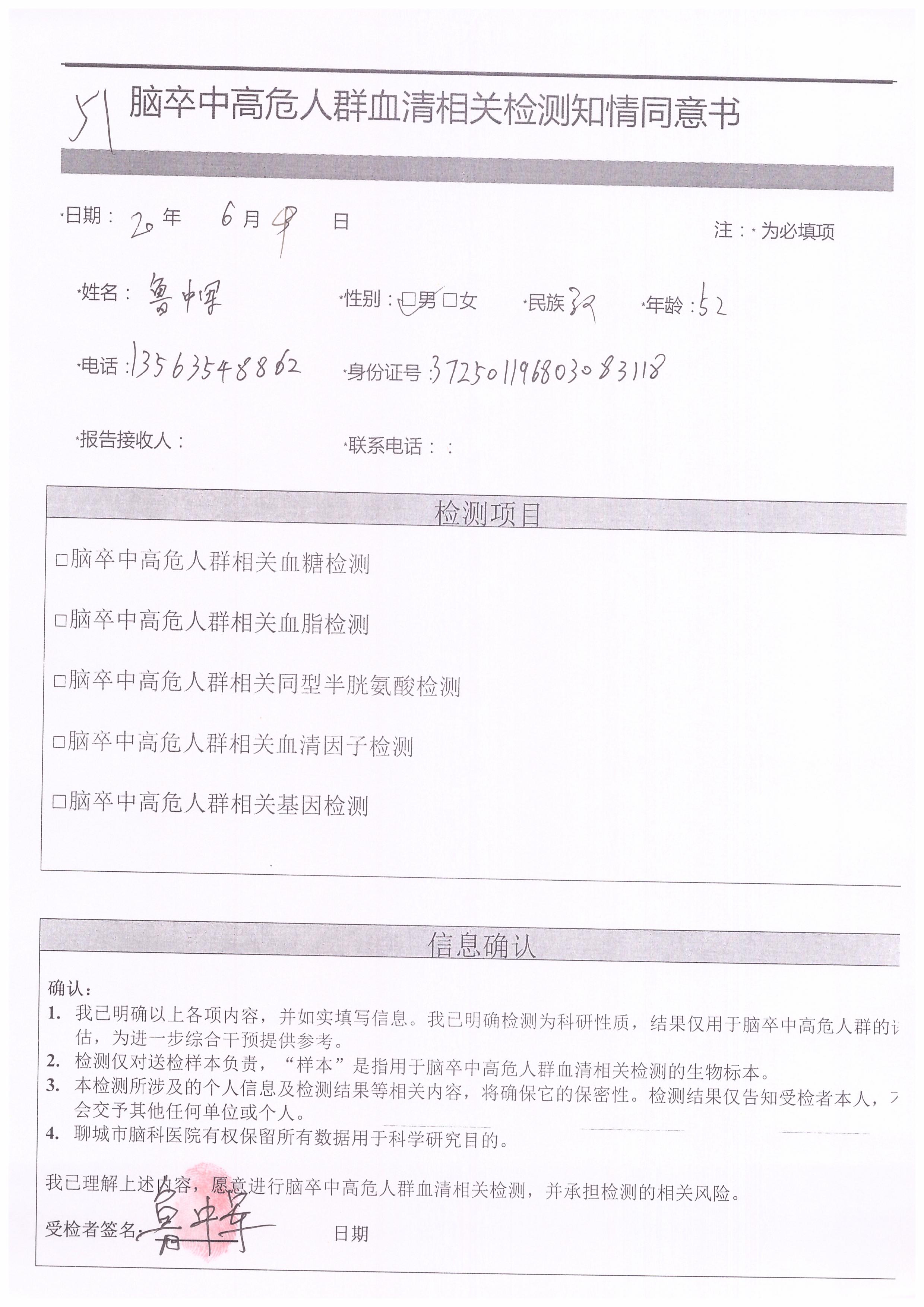

Supplement: Supplementary file 15 — Supplementary file15 (ZIP 22488 KB) [file 10528_2023_10431_MOESM15_ESM.zip › ╓¬╟Θ═1⁄4╥Γ╩Θ13/051.jpg]
